# The burden of diseases, injuries, and risk factors by state in the USA, 1990–2021: a systematic analysis for the Global Burden of Disease Study 2021

**DOI:** 10.1016/S0140-6736(24)01446-6

**Published:** 2024-12-07

**Authors:** GBD 2021 US Burden of Disease Collaborators

## Abstract

**Background:**

The Global Burden of Diseases, Injuries, and Risk Factors Study (GBD) 2021 provides a comprehensive assessment of health and risk factor trends at global, regional, national, and subnational levels. This study aims to examine the burden of diseases, injuries, and risk factors in the USA and highlight the disparities in health outcomes across different states.

**Methods:**

GBD 2021 analysed trends in mortality, morbidity, and disability for 371 diseases and injuries and 88 risk factors in the USA between 1990 and 2021. We used several metrics to report sources of health and health loss related to specific diseases, injuries, and risk factors. GBD 2021 methods accounted for differences in data sources and biases. The analysis of levels and trends for causes and risk factors within the same computational framework enabled comparisons across states, years, age groups, and sex. GBD 2021 estimated years lived with disability (YLDs) and disability-adjusted life-years (DALYs; the sum of years of life lost to premature mortality and YLDs) for 371 diseases and injuries, years of life lost (YLLs) and mortality for 288 causes of death, and life expectancy and healthy life expectancy (HALE). We provided estimates for 88 risk factors in relation to 155 health outcomes for 631 risk–outcome pairs and produced risk-specific estimates of summary exposure value, relative health risk, population attributable fraction, and risk-attributable burden measured in DALYs and deaths. Estimates were produced by sex (male and female), age (25 age groups from birth to ≥95 years), and year (annually between 1990 and 2021). 95% uncertainty intervals (UIs) were generated for all final estimates as the 2·5th and 97·5th percentiles values of 500 draws (ie, 500 random samples from the estimate's distribution). Uncertainty was propagated at each step of the estimation process.

**Findings:**

We found disparities in health outcomes and risk factors across US states. Our analysis of GBD 2021 highlighted the relative decline in life expectancy and HALE compared with other countries, as well as the impact of COVID-19 during the first 2 years of the pandemic. We found a decline in the USA's ranking of life expectancy from 1990 to 2021: in 1990, the USA ranked 35th of 204 countries and territories for males and 19th for females, but dropped to 46th for males and 47th for females in 2021. When comparing life expectancy in the best-performing and worst-performing US states against all 203 other countries and territories (excluding the USA as a whole), Hawaii (the best-ranked state in 1990 and 2021) dropped from sixth-highest life expectancy in the world for males and fourth for females in 1990 to 28th for males and 22nd for females in 2021. The worst-ranked state in 2021 ranked 107th for males (Mississippi) and 99th for females (West Virginia). 14 US states lost life expectancy over the study period, with West Virginia experiencing the greatest loss (2·7 years between 1990 and 2021). HALE ranking declines were even greater; in 1990, the USA was ranked 42nd for males and 32nd for females but dropped to 69th for males and 76th for females in 2021. When comparing HALE in the best-performing and worst-performing US states against all 203 other countries and territories, Hawaii ranked 14th highest HALE for males and fifth for females in 1990, dropping to 39th for males and 34th for females in 2021. In 2021, West Virginia—the lowest-ranked state that year—ranked 141st for males and 137th for females. Nationally, age-standardised mortality rates declined between 1990 and 2021 for many leading causes of death, most notably for ischaemic heart disease (56·1% [95% UI 55·1–57·2] decline), lung cancer (41·9% [39·7–44·6]), and breast cancer (40·9% [38·7–43·7]). Over the same period, age-standardised mortality rates increased for other causes, particularly drug use disorders (878·0% [770·1–1015·5]), chronic kidney disease (158·3% [149·6–167·9]), and falls (89·7% [79·8–95·8]). We found substantial variation in mortality rates between states, with Hawaii having the lowest age-standardised mortality rate (433·2 per 100 000 [380·6–493·4]) in 2021 and Mississippi having the highest (867·5 per 100 000 [772·6–975·7]). Hawaii had the lowest age-standardised mortality rates throughout the study period, whereas Washington, DC, experienced the most improvement (a 40·7% decline [33·2–47·3]). Only six countries had age-standardised rates of YLDs higher than the USA in 2021: Afghanistan, Lesotho, Liberia, Mozambique, South Africa, and the Central African Republic, largely because the impact of musculoskeletal disorders, mental disorders, and substance use disorders on age-standardised disability rates in the USA is so large. At the state level, eight US states had higher age-standardised YLD rates than any country in the world: West Virginia, Kentucky, Oklahoma, Pennsylvania, New Mexico, Ohio, Tennessee, and Arizona. Low back pain was the leading cause of YLDs in the USA in 1990 and 2021, although the age-standardised rate declined by 7·9% (1·8–13·0) from 1990. Depressive disorders (56·0% increase [48·2–64·3]) and drug use disorders (287·6% [247·9–329·8]) were the second-leading and third-leading causes of age-standardised YLDs in 2021. For females, mental health disorders had the highest age-standardised YLD rate, with an increase of 59·8% (50·6–68·5) between 1990 and 2021. Hawaii had the lowest age-standardised rates of YLDs for all sexes combined (12 085·3 per 100 000 [9090·8–15 557·1]), whereas West Virginia had the highest (14 832·9 per 100 000 [11 226·9–18 882·5]). At the national level, the leading GBD Level 2 risk factors for death for all sexes combined in 2021 were high systolic blood pressure, high fasting plasma glucose, and tobacco use. From 1990 to 2021, the age-standardised mortality rates attributable to high systolic blood pressure decreased by 47·8% (43·4–52·5) and for tobacco use by 5·1% (48·3%–54·1%), but rates increased for high fasting plasma glucose by 9·3% (0·4–18·7). The burden attributable to risk factors varied by age and sex. For example, for ages 15–49 years, the leading risk factors for death were drug use, high alcohol use, and dietary risks. By comparison, for ages 50–69 years, tobacco was the leading risk factor for death, followed by dietary risks and high BMI.

**Interpretation:**

GBD 2021 provides valuable information for policy makers, health-care professionals, and researchers in the USA at the national and state levels to prioritise interventions, allocate resources effectively, and assess the effects of health policies and programmes. By addressing socioeconomic determinants, risk behaviours, environmental influences, and health disparities among minority populations, the USA can work towards improving health outcomes so that people can live longer and healthier lives.

**Funding:**

Bill & Melinda Gates Foundation.

## Introduction

The burden of disease in the USA has evolved over the past three decades, influenced by changing demographics, socioeconomic factors, and advancements in health care.^1^, ^2^, ^3^ From 1990 to 2021, the USA has experienced changes to its population structure, with an ageing population and increasing life expectancy.^1^ This demographic shift has contributed to an increase in non-communicable diseases (NCDs), which have been and still are the leading causes of morbidity and mortality in the USA.^4^, ^5^ NCDs, such as cardiovascular diseases, cancers, and respiratory diseases, still account for a large proportion of the overall disease burden, with considerable implications for health-care systems and policy.^5^ The increase in NCDs has been accompanied by a decline in the burden of some communicable, maternal, neonatal, and nutritional (CMNN) diseases, largely due to improvements in public health interventions, vaccination programmes, and access to health care.^5^, ^6^, ^7^ However, certain CMNN diseases, such as sexually transmitted infections and maternal mortality, continue to pose challenges, highlighting the need for targeted strategies and ongoing surveillance.^5^ The COVID-19 pandemic has had a profound impact on the burden of disease in the USA, resulting in many deaths, long-term health consequences, and disruptions to health-care systems.^1^, ^4^, ^5^, ^8^, ^9^ The pandemic has also highlighted existing vulnerabilities and disparities in the US health-care system, underscoring the need for a more resilient, equitable, and accessible health-care infrastructure.^10^, ^11^, ^12^, ^13^

Mental health disorders have also emerged as an important contributor to the burden of disease in the USA, with increasing prevalence rates of depression, anxiety, and substance use disorders.^5^ These conditions not only affect individuals’ quality of life but also have substantial economic and social consequences, necessitating a comprehensive approach to mental health promotion, prevention, and treatment.^14^, ^15^ Another key aspect of the disease burden in the USA is persistent health disparities across different population groups, primarily driven by socioeconomic factors, race, and ethnicity.^16^, ^17^ These disparities manifest in unequal access to health care, variations in health outcomes, and differences in exposure to risk factors, highlighting the need for targeted interventions and policies to address these inequalities.^16^, ^17^ In recent years, the opioid crisis has emerged as a major public health challenge in the USA, contributing to a substantial increase in overdose deaths and exacerbating the burden of mental health and substance use disorders.^4^, ^5^ Addressing this crisis requires a multi-faceted approach, encompassing prevention, treatment, and harm-reduction strategies, as well as policies aimed at curbing the overprescription of opioids and improving access to addiction treatment services.^18^

In this study, we report on the burden of disease across US states using findings from Global Burden of Diseases, Injuries, and Risk Factors Study (GBD) 2021, a comprehensive, systematic effort to quantify the impact of diseases, injuries, and risk factors on population health. This study provides valuable insights into the shifting landscape of disease burden in the USA from 1990 to 2021. The results of this study will guide policy makers, health-care professionals, and researchers in the USA to identify priority areas for interventions, allocate resources more effectively, and assess the impact of health policies and programmes. Additionally, these insights can guide the development of targeted strategies to address specific challenges and reduce health disparities across different population groups. This manuscript was produced as part of the GBD Collaborator Network and in accordance with the GBD Protocol.^19^

**Research in context**

**Evidence before this study**

The Global Burden of Diseases, Injuries, and Risk Factors Study (GBD) 2019 was the last round of GBD estimates before the publication of GBD 2021. GBD 2019 included comprehensive estimates of health and health loss for the United States at the national and state levels. Previously, the US Burden of Disease Collaborators used GBD 2016 estimates to provide a comprehensive assessment of health trends and risk factors at the state and national levels for the USA. The results highlighted the ongoing challenges faced by the country in addressing the burden of non-communicable diseases (NCDs) and the increasing impact of mental health disorders and substance use disorders. However, GBD 2016 and GBD 2019 preceded the COVID-19 pandemic. Previous GBD studies of the USA have also analysed the burden of disease, injuries, and risk factors at the county level by race and ethnicity; this research has also resulted in topic-specific papers on the burden of death due to cardiovascular disease and trends in life expectancy.

**Added value of this study**

GBD 2021 offers an updated analysis of health trends and risk factors at the state level in the USA that accounts for the effects of the first 2 years of the COVID-19 pandemic. This study reveals the disparities in health outcomes and risk factors across states and underscores the need for tailored strategies to address specific health challenges. By presenting comprehensive and timely estimates on mortality, morbidity, and risk factors, GBD 2021 enables stakeholders in the USA to identify priority areas for interventions, allocate resources more effectively, and assess the effects of health policies and programmes. The COVID-19 pandemic underscored the importance of understanding health disparities and addressing the needs of vulnerable populations. GBD 2021 highlights the disease, injuries, and risk factors that are the largest sources of morbidity and mortality; our findings can be used by policy makers, medical professionals, and researchers to inform investing strategies and policy interventions that target the causes of health loss and premature mortality.

**Implications of all the available evidence**

The results of our study reveal that areas of the USA underperform in several key health metrics compared with nearly all high-income and even some middle-income countries and territories. In 2021, eight states (West Virginia, Kentucky, Oklahoma, Pennsylvania, New Mexico, Ohio, Tennessee, and Arizona) had age-standardised rates of years lived with disability greater than any country in the world. The USA's global ranking in healthy life expectancy (HALE) declined compared with other countries; indeed, when comparing HALE in the worst-ranked US state versus the rest of the world, West Virginia ranked 141st among all 203 other countries and territories for males and 137th for females in 2021. This study's comprehensive estimates of disease burden in the USA show that we must shift our focus from a predominantly curative care model to a more comprehensive strategy that places equal emphasis on prevention, social safety nets, and investments in evidence-based solutions. Our study highlights the areas that demand immediate attention and emphasises the importance of addressing social determinants of health, health disparities, and the need for targeted interventions. This study illuminates glaring gaps in our health system and provides policy makers, health-care professionals, researchers, and the public the knowledge needed to put forth a concerted effort to transform the US health-care system and population health landscape.

## Methods

### Overview

GBD 2021 methodology has been published previously.^1^, ^4^, ^5^, ^20^, ^21^, ^22^, ^23^ GBD uses several metrics to report results on health loss related to specific diseases, injuries, and risk factors: deaths, incidence, prevalence, years of life lost due to premature mortality (YLLs), years lived with disability (YLDs), and disability-adjusted life-years (DALYs; the sum of YLLs and YLDs); these metrics are calculated in counts, age-specific and all-age rates, and age-standardised rates. GBD also calculates risk-attributable deaths, YLLs, YLDs, and DALYs for all GBD risk factors. GBD systematically accounts for differences in data sources and biases and analyses levels and trends for causes and risk factors within the same computational framework, which maximises comparability across states, years, and different age groups by sex. GBD 2021 produced YLL and mortality estimates for 288 causes of death,^4^ as well as incidence, prevalence, YLD, and DALY estimates for 371 diseases and injuries,^5^ along with estimates of life expectancy and healthy life expectancy (HALE).^1^, ^5^ For GBD 2021, risk-specific estimates of summary exposure value (SEV), relative health risk, population attributable fraction (PAF), and risk-attributable burden measured in DALYs and deaths were produced for 88 risk factors associated with 155 health outcomes.^21^ Estimates were produced by sex (male and female), age (25 age groups from birth to ≥95 years), and year (annually between 1990 and 2021) for 204 countries and territories, including subnational estimates for 21 countries and territories, including US states. 95% uncertainty intervals (UIs) were generated for all final estimates as the 2·5th and 97·5th percentiles of 500 draws. Uncertainty was propagated at each step of the estimation process.

This research is compliant with the GATHER recommendations.^24^ A completed GATHER checklist is given in appendix 1 (p 278).

### Cause hierarchy

GBD classifies diseases and injuries into a hierarchy with four levels that include both fatal and non-fatal causes.^4^, ^5^ Level 1 consists of three broad aggregate categories: communicable, maternal, neonatal, and nutritional (CMNN) diseases; NCDs; and injuries. Level 2 includes 22 clusters of causes that each fall within a Level 1 category. Level 3 includes 175 causes, of which 132 are specific causes and 43 are clusters of Level 4 causes. Level 4 consists of 302 specific causes, including 170 specific causes that each fall within the 43 Level 3 clusters of causes and the 132 Level 3 specific causes that were not further disaggregated at Level 4. Overall, 365 causes had non-fatal outcomes and 288 causes had fatal outcomes.

For GBD 2021, we separately report on 12 causes of death for the first time: COVID-19, other COVID-19 pandemic-related outcomes—also known as other pandemic-related mortality (OPRM)—pulmonary arterial hypertension, and nine cancer types: hepatoblastoma, Burkitt lymphoma, other non-Hodgkin lymphoma, eye cancer, retinoblastoma, other eye cancers, soft tissue and other extraosseous sarcomas, malignant neoplasm of bone and articular cartilage, and neuroblastoma and other peripheral nervous cell tumours.^4^ OPRM represents excess mortality associated with the pandemic minus mortality directly due to COVID-19, lower respiratory infections, measles, malaria, and pertussis. OPRM accounts for increases in excess mortality in 2020 and 2021 that could not be attributed to a particular cause.

### Risk factor hierarchy

GBD risk factors are categorised into a hierarchy with four levels.^21^ Level 1 includes three broad risk factor categories that encompass all specific risk factors: metabolic risks, behavioural risks, and environmental and occupational risks. The Level 1 risk groups are then disaggregated into 20 Level 2 risks or groups of risks (eg, child and maternal malnutrition, and tobacco). Level 3 risks include those that are further disaggregated from Level 2 (nine groups of risks disaggregated into 52 more detailed risks) as well as those that are not disaggregated beyond Level 2 (11 risks [eg, high systolic blood pressure]), for 53 total Level 3 risk factors. Finally, there are 70 total Level 4 risk factors, including 48 Level 3 risks that are not disaggregated further and 22 additional risks disaggregated from the remaining six Level 3 risks. One new Level 3 risk factor was reported on in GBD for the first time in GBD 2021: nitrogen dioxide, an air pollution risk factor dominated by motor vehicle emissions.^21^

### Data sources and processing

Citations and metadata for all data sources used in this study are available in the GBD 2021 Sources Tool.

We used age-specific mortality rates and standard methods to estimate life expectancy. Data sources for age-specific mortality were extracted from a range of sources, including vital registration systems, surveys, censuses, and more. Details on data sources and processing used as inputs to estimate life expectancy have been previously published.^1^

To estimate the US burden of disease incidence, prevalence, and YLDs, we began with a systematic analysis of published studies and available data sources providing information on prevalence, incidence, remission, and more, as previously detailed.^5^ Non-fatal data for GBD 2021 were extracted from a range of source types including clinical data sources, registries, literature, surveys, and more. Clinical data for the USA included (1) US health insurance claims and (2) US inpatient hospital admission records. Key sources for these clinical data types included Marketscan^25^ and the Healthcare Cost and Utilization Project (HCUP).^26^ Marketscan is a commercial claims dataset that includes both inpatient and outpatient encounters. HCUP covers 48 states plus Washington, DC (all states except Idaho and Alabama), and provides individual-level, statewide inpatient data. HCUP includes the National (Nationwide) Inpatient Sample (NIS), the largest health-care database in the USA for all-payer inpatient data. The sample strategy for the NIS is designed to be nationally representative and is therefore considered complete for all state-years for which data are available. Because HCUP provides individual-level medical record data with near-universal coverage, these data are comparable with data used in GBD from other countries for which we also have complete or nearly complete individual-level medical record data. Of note, the GBD analytical framework for all components of the analysis (including non-fatal causes as well as demographics, causes of death, and risk factor modelling) is designed to ensure comprehensive and comparable estimates across populations. Inpatient hospital admission data were mainly coded with primary diagnosis. We used Marketscan and HCUP data, along with clinical sources from other countries, to adjust aggregate inpatient data sources to account for readmissions, non-primary diagnoses, and outpatient care. We used Marketscan and other sources to adjust sources to include outpatient visits. We treated some of the US claims data as non-reference (non-representative) because of a systematic bias associated with commercial health insurance status. Using standard GBD 2021 methods, non-referent sources were adjusted using crosswalks estimated using meta-regression—Bayesian, regularised, trimmed (MR-BRT).^27^ Further details on these non-fatal data sources and clinical data processing methods, as well as complete methods for processing non-clinical data sources, have been described in detail elsewhere.^5^, ^28^

Data sources for cause-specific mortality and YLLs comprise vital registration systems, surveys, censuses, surveillance, cancer registries, police records, and open-source databases. These data were corrected for standardisation and comparability across ICD-10 death codes, age groups, sexes, locations, and time.^4^

Data sources for estimating risk-attributable burden included those used to estimate relative risk (RR) and those used to estimate exposure. For RR, data were extracted from randomised controlled trials, cohort studies, pooled cohort studies, case-control studies, and meta-analyses. These studies were identified through systematic reviews conducted for GBD 2021 and previous GBD rounds. Exposure data sources included household and health examination surveys and censuses, administrative records, ground-sensing or remote-sensing data, and other sources identified through systematic reviews.^21^

### Statistical analysis

We used GBD 2021 demographic methods to adjust for deaths due to the COVID-19 pandemic.^1^ The analytical approach consists of six main components: (1) estimating age-specific fertility rates, (2) estimating under-5 mortality rates, (3) estimating adult mortality rates, (4) estimating age-specific mortality rates using a relational model life table with HIV adjustments, (5) estimating excess mortality due to the COVID-19 pandemic, and (6) estimating population sizes. Excess mortality due to the COVID-19 pandemic was calculated using similar methods as those of the COVID-19 Excess Mortality Collaborators.^29^ Briefly, the best model is selected on the basis of out-of-sample performance, and mean values and 95% UIs are generated at the national and state levels. To calculate age-sex-specific excess mortality, all-cause mortality is estimated twice, once with data from during the pandemic and once without. Age-sex-specific excess mortality was then calculated and redistributed as needed to ensure consistency with observed high-quality vital registration data. Life expectancy was estimated using age-specific mortality rates and standard demographics methods.^1^

Cause-specific death rates for most causes were estimated using the Cause of Death Ensemble model (CODEm); YLLs were calculated as the number of deaths for each cause-age-sex-location-year multiplied by standard life expectancy (ie, the lowest age-specific mortality rate between locations) for each age group.^4^ Garbage codes (non-specific, implausible, or intermediate cause of death codes) in ICD were redistributed to appropriate target causes using redistribution algorithms.^30^ For estimating COVID-19 deaths, a susceptible-exposed-infectious transmission model was created that accounted for factors such as vaccine uptake, vaccine effectiveness, antiviral administration, new variant emergence, and waning protection from both infection-derived and vaccine-derived immunity. This model was used to estimate past infections, hospitalisations, and deaths by variant, location, and day. To account for increases in excess mortality in 2020 and 2021 that could not be attributed to particular causes, GBD 2021 introduced a new cause of death—OPRM—which represents excess mortality associated with the pandemic minus mortality directly due to COVID-19 and other known causes affected by the pandemic.^4^

Prevalence and incidence for most diseases and injuries were modelled using Disease Modelling Meta-Regression (DisMod-MR; version 2.1).^31^ Prevalence and incidence for the remaining diseases were modelled using spatiotemporal Gaussian process regression (ST-GPR) or, when DisMod-MR and ST-GPR could not adequately model prevalence or incidence, such as for HIV/AIDS, custom models were used.^5^ For non-fatal causes for which disability varies in severity (eg, asymptomatic, mild, moderate, and severe), prevalence and incidence were split by sequelae. To estimate the proportion of cases in each sequela category, the Medical Expenditure Survey^32^ was used for most causes; for a subset of causes, several other sources^33^, ^34^, ^35^ were used. Disability weights—which are used to estimate YLDs—were calculated from general population surveys across nine countries around the world as well as an online survey available in English, Spanish, and Mandarin.^36^, ^37^

YLDs were calculated by multiplying cause-age-sex-location-year-specific prevalence of sequelae by their respective disability weights for each disease and injury. DALYs were calculated by summing YLDs and YLLs by location, age, sex, year, and cause.^5^ HALE was estimated as a complementary metric, representing a population's average number of years of life spent in good health.^5^ HALE values were calculated using age-specific mortality rates and YLDs per capita.

The GBD 2021 analytical framework for risk factors generates estimates of effect size by quantifying the RR of each of the selected GBD health outcomes (155 total outcomes) occurring as a function of exposure to each associated GBD risk factor (88 risk factors).^21^ GBD 2021 produced estimates for 631 total risk–outcome pairs, including 117 pairs that were new for GBD 2021. Risk–outcome pairs are considered potential candidates for inclusion based on several factors, including convincing or probable evidence of an association based on World Cancer Research Fund criteria,^38^ policy importance, data availability, and adequate methods to estimate exposure across locations. We ran a decomposition analysis of changes in all-age, cause-specific DALYs attributable to all risk factors and individual risk factors. Risk-deleted DALY rates are DALY rates after removing the effect of a risk factor or combination of risk factors on overall rates; to obtain risk-deleted DALY rates, we multiplied overall DALY rates by one minus the population attributable fraction for the risk or set of risks.

GBD 2021 included methodological improvements related to RR estimation standardisation as well as the application of new burden of proof risk function (BPRF) methods.^21^, ^39^ These methods produce a conservative assessment of risk–outcome relationships by incorporating between-study heterogeneity into the UIs of the risk function. Further improvements to the methods for GBD 2021 include improving the specification of the mediation matrix and re-evaluating theoretical minimum risk exposure levels (TMRELs) using meta-regression or other methods to incorporate new data. The latter improvement produced updated TMRELs for 19 GBD risk factors, primarily high systolic blood pressure, high LDL cholesterol, high BMI, and various dietary risks.

Using the BPRF methodology, risk–outcome scores (ROSs) were produced for a subset of risk–outcome pairs; the remaining pairs will be assessed using this methodology in future GBD rounds. Higher positive ROSs indicate either a larger average effect size or stronger evidence for an association between risk and outcome, or both.^21^, ^39^ ROSs were then converted to star ratings from one to five, with one and two stars indicating weak evidence of an association between risk and outcome, three stars indicating moderate evidence, four stars indicating strong evidence, and five stars indicating very strong evidence.

Software packages used in GBD 2021 were Python (versions 3.8.17, 3.10, 3.10.4, and 3.10.12), Stata (versions 13.1, 15, and 15.1), and R (versions 3.5, 3.5.1, and 4.2.1). Statistical code used for GBD estimation is publicly available online.

### Role of the funding source

The funder of the study had no role in study design, data collection, data analysis, data interpretation, or writing of the report.

## Results

### Overview

Additional results are available in appendix 1. All estimates are also available via visual exploration through the online tool GBD Compare, and detailed results for HALE and for each disease, injury, and risk factor in the analysis are available in searchable and downloadable form through the GBD Results Tool.

### Life expectancy and HALE

**Figure 1:**
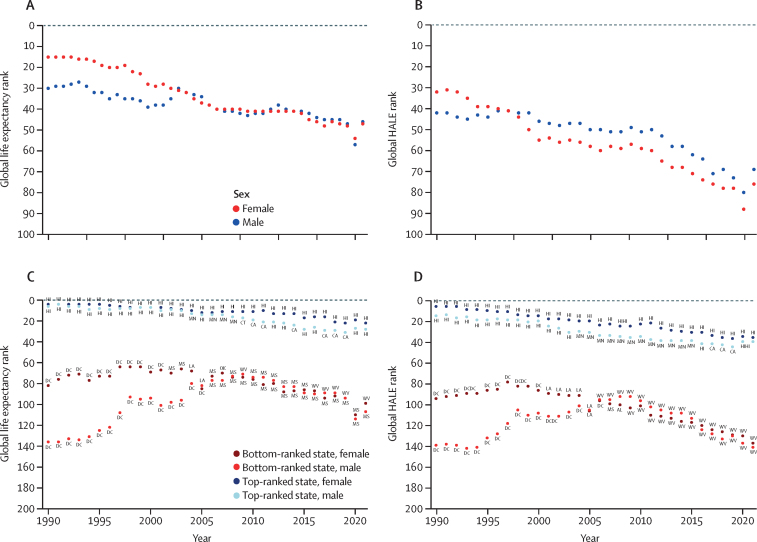
Global life expectancy and HALE ranking for the USA and top and bottom US states (A) Global life expectancy ranking for the USA by sex, 1990–2021. (B) Global HALE ranking for the USA by sex, 1990–2021. (C) Global life expectancy ranking for top and bottom US states and Washington, DC, compared with all other countries and territories, by sex, 1990–2021. (D) Global HALE ranking for top and bottom US states and Washington, DC, compared with all other countries and territories, by sex, 1990–2021. Global life expectancy and HALE rankings for the USA (A and B) depict the USA's ranking among all 204 countries and territories. C and D depict the best-ranked and worst-ranked US state for life expectancy and HALE in each year of the study period, among all 203 other countries and territories in GBD 2021 (excluding the USA as a whole). AL=Alabama. CA=California. GBD=Global Burden of Diseases, Injuries, and Risk Factors Study. DC=Washington, DC. HALE=healthy life expectancy. HI=Hawaii. LA=Louisiana. MN=Minnesota. MS=Mississippi. WV=West Virginia.

In 2021, life expectancy in the USA was 77·1 years (95% UI 77·0–77·2) for all sexes combined: 74·3 years (74·1–74·4) for males and 80·0 years (79·9–80·2) for females (table). HALE was 64·4 years (61·0–67·4) for all sexes combined in 2021: 63·2 (60·2–65·8) for males and 65·7 (61·7–69·1) for females (table; appendix 1 pp 15, 17). The USA's global ranking in life expectancy declined between 1990 and 2021 (figure 1A).^5^ In 1990, the USA had the 35th highest life expectancy for males and 19th highest for females among the 204 countries and territories included in GBD 2021, but that rank dropped to 46th for males and 47th for females in 2021*.* Similar to life expectancy, we found a decline in the USA's global ranking in HALE compared with other countries between 1990 and 2021 (figure 1B).^5^ In 1990, the USA was ranked 32nd of 204 countries and territories for females and 42nd for males. This rank dropped to 69th for males and 76th for females in 2021.

**Table:** Life expectancy and healthy life expectancy in the USA, by US state, and for Washington, DC, for all sexes combined, 1990, 2010, 2019, and 2021

	**Life expectancy, years (95% UI)**				**Healthy life expectancy, years (95% UI)**			
	1990	2010	2019	2021	1990	2010	2019	2021
USA	75·6 (75·5–75·6)	78·8 (78·7–78·8)	79·1 (79·1–79·1)	77·1 (77·0–77·2)	64·8 (61·7–67·4)	66·7 (63·3–69·6)	66·2 (62·7–69·3)	64·4 (60·9–67·4)
Alabama	73·8 (73·6–74·1)	75·4 (75·2–75·6)	75·7 (75·5–75·9)	73·0 (71·5–74·4)	63·4 (60·4–66·0)	63·8 (60·6–66·5)	63·5 (60·0–66·4)	61·1 (57·6–64·3)
Alaska	75·0 (74·6–75·4)	77·8 (77·5–78·2)	78·5 (78·0–79·0)	75·7 (74·5–76·8)	64·3 (61·2–67·0)	66·1 (63·0–69·0)	65·8 (62·2–68·9)	63·4 (60·0–66·5)
Arizona	76·4 (76·2–76·7)	79·3 (79·1–79·5)	79·1 (78·9–79·3)	76·0 (74·6–77·3)	65·2 (61·9–67·9)	66·7 (63·1–69·7)	65·8 (62·2–69·0)	63·1 (59·5–66·4)
Arkansas	74·5 (74·2–74·7)	76·0 (75·7–76·2)	76·0 (75·7–76·2)	73·5 (71·9–75·1)	64·1 (61·1–66·7)	64·9 (61·8–67·5)	64·2 (60·9–67·1)	62·0 (58·8–65·1)
California	76·0 (75·9–76·1)	80·8 (80·7–80·8)	81·4 (81·3–81·5)	79·3 (78·0–80·5)	65·3 (62·3–67·9)	68·6 (65·2–71·5)	68·5 (64·9–71·6)	66·6 (63·1–69·8)
Colorado	77·3 (77·0–77·5)	80·1 (79·9–80·3)	80·5 (80·3–80·7)	78·8 (77·2–80·3)	66·0 (62·6–68·8)	67·7 (64·2–70·7)	67·2 (63·6–70·4)	65·7 (62·3–69·4)
Connecticut	77·3 (77·1–77·6)	80·6 (80·4–80·9)	80·9 (80·6–81·2)	80·3 (78·7–81·8)	66·3 (63·1–69·0)	68·0 (64·7–71·0)	67·7 (64·1–70·9)	67·0 (63·3–70·5)
Delaware	75·0 (74·7–75·4)	78·2 (77·9–78·5)	78·4 (78·0–78·8)	76·7 (75·6–77·9)	64·3 (61·1–66·9)	65·9 (62·4–68·8)	65·2 (61·6–68·5)	63·8 (59·9–66·9)
Florida	76·0 (75·8–76·1)	79·3 (79·2–79·4)	79·5 (79·3–79·6)	77·0 (75·6–78·5)	64·9 (61·7–67·6)	66·8 (63·4–69·8)	66·2 (62·6–69·4)	64·0 (60·1–67·5)
Georgia	73·9 (73·7–74·1)	77·3 (77·2–77·5)	78·0 (77·9–78·2)	75·3 (73·9–76·6)	63·5 (60·5–66·0)	65·7 (62·4–68·4)	65·5 (62·1–68·5)	63·1 (59·6–66·4)
Hawaii	78·4 (78·1–78·8)	81·2 (80·9–81·5)	81·7 (81·4–82·1)	81·2 (79·6–82·8)	67·2 (64·0–70·0)	69·0 (65·5–71·9)	68·9 (65·3–72·1)	68·3 (64·5–71·4)
Idaho	77·0 (76·6–77·4)	79·3 (79·0–79·6)	79·8 (79·4–80·1)	77·5 (76·1–78·9)	65·9 (62·6–68·7)	67·0 (63·6–70·1)	66·8 (63·3–70·0)	64·8 (61·1–68·0)
Illinois	75·1 (75·0–75·2)	78·9 (78·8–79·0)	79·4 (79·3–79·5)	77·9 (76·5–79·4)	64·6 (61·5–67·1)	67·0 (63·7–69·8)	66·8 (63·3–69·8)	65·4 (61·7–68·5)
Indiana	75·6 (75·4–75·8)	77·4 (77·2–77·6)	77·2 (77·0–77·4)	75·3 (73·9–76·8)	64·8 (61·7–67·3)	65·2 (61·8–68·2)	64·4 (61·0–67·4)	62·8 (59·3–66·0)
Iowa	77·8 (77·6–78·1)	79·4 (79·2–79·7)	79·3 (79·0–79·6)	78·3 (76·8–79·7)	66·7 (63·6–69·5)	67·6 (64·2–70·5)	66·8 (63·3–69·9)	65·8 (62·1–69·0)
Kansas	77·1 (76·9–77·4)	78·4 (78·1–78·7)	78·3 (78·0–78·6)	76·7 (75·2–78·3)	66·0 (62·9–68·8)	66·5 (63·1–69·4)	65·8 (62·3–68·9)	64·3 (60·9–67·6)
Kentucky	74·6 (74·4–74·9)	75·8 (75·6–76·0)	75·8 (75·6–76·0)	73·2 (71·7–74·7)	63·9 (60·7–66·6)	63·8 (60·4–66·8)	63·0 (59·5–66·0)	60·6 (57·3–64·0)
Louisiana	73·4 (73·2–73·6)	75·7 (75·5–75·9)	75·8 (75·6–76·0)	73·2 (71·7–74·7)	62·9 (60·0–65·5)	64·0 (60·8–66·8)	63·4 (60·0–66·3)	61·2 (57·9–64·3)
Maine	76·7 (76·4–77·1)	79·0 (78·7–79·4)	78·8 (78·4–79·2)	77·5 (75·9–79·0)	65·7 (62·5–68·4)	66·7 (63·3–69·7)	65·8 (62·2–68·9)	64·6 (60·8–67·8)
Maryland	75·0 (74·8–75·2)	79·2 (79·0–79·4)	79·3 (79·1–79·5)	78·1 (76·5–79·8)	64·6 (61·6–67·2)	67·0 (63·5–69·9)	66·7 (63·2–69·7)	65·5 (61·9–68·7)
Massachusetts	76·9 (76·8–77·1)	80·5 (80·3–80·6)	80·8 (80·6–81·0)	80·6 (79·1–82·1)	65·8 (62·6–68·6)	67·8 (64·4–70·9)	67·5 (63·8–70·7)	67·0 (63·3–70·7)
Michigan	75·3 (75·2–75·5)	77·9 (77·8–78·1)	78·5 (78·3–78·6)	76·6 (75·2–78·1)	64·5 (61·4–67·2)	65·8 (62·5–68·7)	65·3 (61·9–68·5)	63·7 (60·0–66·9)
Minnesota	77·9 (77·7–78·1)	80·7 (80·5–81·0)	80·8 (80·6–81·0)	79·7 (78·3–81·1)	66·8 (63·5–69·5)	68·7 (65·4–71·7)	68·1 (64·7–71·2)	67·0 (63·5–70·3)
Mississippi	73·4 (73·1–73·7)	74·9 (74·6–75·1)	74·6 (74·3–74·8)	71·9 (70·3–73·5)	63·3 (60·4–65·8)	64·1 (61·1–66·7)	63·3 (60·1–66·0)	60·9 (57·7–64·1)
Missouri	75·4 (75·2–75·6)	77·4 (77·2–77·5)	77·3 (77·1–77·5)	75·5 (73·9–77·0)	64·8 (61·7–67·5)	65·4 (62·0–68·3)	64·6 (61·1–67·6)	63·0 (59·6–66·2)
Montana	76·7 (76·3–77·1)	78·5 (78·1–78·9)	79·0 (78·6–79·4)	77·0 (75·5–78·5)	65·6 (62·4–68·4)	66·4 (62·9–69·3)	66·1 (62·6–69·2)	64·4 (60·8–67·5)
Nebraska	77·1 (76·7–77·4)	79·5 (79·2–79·8)	79·6 (79·2–79·9)	78·4 (77·0–79·8)	66·0 (62·8–68·8)	67·5 (64·2–70·5)	67·0 (63·5–70·1)	65·8 (62·1–69·2)
Nevada	74·5 (74·2–74·8)	77·8 (77·6–78·1)	78·5 (78·2–78·8)	75·9 (74·5–77·3)	63·9 (60·9–66·4)	65·9 (62·6–68·9)	65·7 (62·2–68·8)	63·4 (60·0–66·7)
New Hampshire	76·9 (76·6–77·3)	80·2 (79·8–80·5)	79·8 (79·4–80·2)	79·1 (77·6–80·5)	65·9 (62·7–68·7)	67·5 (64·0–70·6)	66·4 (62·8–69·7)	65·6 (61·7–69·1)
New Jersey	75·6 (75·5–75·8)	80·0 (79·8–80·2)	80·8 (80·7–81·0)	79·8 (78·3–81·2)	65·0 (61·9–67·6)	67·8 (64·5–70·8)	67·7 (64·1–70·8)	66·6 (62·9–70·0)
New Mexico	76·0 (75·7–76·4)	78·3 (78·0–78·7)	77·6 (77·3–77·9)	74·9 (73·2–76·5)	64·8 (61·6–67·6)	66·0 (62·6–68·9)	64·5 (61·0–67·7)	62·2 (58·6–65·7)
New York	74·8 (74·7–75·0)	80·3 (80·2–80·4)	81·5 (81·4–81·6)	80·0 (78·6–81·4)	64·0 (60·9–66·7)	67·4 (63·8–70·5)	67·6 (63·8–71·0)	66·3 (62·5–69·8)
North Carolina	74·8 (74·6–74·9)	77·9 (77·7–78·1)	78·0 (77·8–78·1)	75·8 (74·3–77·4)	64·4 (61·3–66·9)	66·2 (63·0–69·0)	65·5 (62·0–68·4)	63·5 (60·0–66·7)
North Dakota	77·8 (77·4–78·1)	79·4 (79·1–79·8)	79·8 (79·3–80·3)	79·7 (78·4–81·0)	66·6 (63·4–69·3)	67·3 (63·8–70·3)	66·9 (63·2–70·0)	66·5 (62·8–70·0)
Ohio	75·5 (75·3–75·6)	77·5 (77·4–77·7)	77·2 (77·1–77·3)	75·3 (73·8–76·7)	64·7 (61·6–67·3)	65·4 (62·1–68·4)	64·3 (60·8–67·4)	62·5 (58·9–65·9)
Oklahoma	75·3 (75·1–75·5)	75·7 (75·4–75·9)	76·0 (75·8–76·2)	73·5 (72·0–75·0)	64·5 (61·4–67·1)	63·9 (60·7–66·8)	63·4 (60·0–66·5)	61·2 (57·6–64·6)
Oregon	76·6 (76·3–76·9)	79·5 (79·3–79·7)	80·1 (79·8–80·3)	78·3 (76·7–79·8)	65·7 (62·6–68·4)	67·5 (64·2–70·5)	67·2 (63·6–70·3)	65·6 (61·8–68·8)
Pennsylvania	75·6 (75·4–75·7)	78·5 (78·3–78·6)	78·7 (78·6–78·8)	77·0 (75·6–78·5)	64·6 (61·4–67·3)	65·9 (62·5–68·9)	65·4 (61·7–68·5)	63·8 (60·1–67·4)
Rhode Island	76·6 (76·3–77·0)	79·5 (79·1–79·8)	80·0 (79·6–80·5)	79·6 (78·2–81·0)	65·6 (62·5–68·4)	67·2 (63·7–70·1)	66·9 (63·2–70·2)	66·3 (62·3–69·7)
South Carolina	73·8 (73·6–74·1)	76·7 (76·5–76·9)	76·9 (76·7–77·1)	74·3 (72·7–75·9)	63·3 (60·3–65·9)	64·8 (61·5–67·7)	64·3 (60·8–67·3)	62·0 (58·3–65·1)
South Dakota	77·0 (76·7–77·4)	79·2 (78·8–79·5)	78·9 (78·4–79·3)	78·1 (76·8–79·4)	66·1 (62·9–68·9)	67·2 (63·8–70·1)	66·3 (62·9–69·4)	65·5 (61·9–68·9)
Tennessee	74·3 (74·1–74·5)	76·2 (76·0–76·4)	76·0 (75·8–76·2)	73·5 (71·9–75·1)	63·8 (60·7–66·4)	64·5 (61·3–67·4)	63·6 (60·1–66·5)	61·3 (57·9–64·4)
Texas	75·3 (75·2–75·4)	78·5 (78·4–78·6)	79·1 (79·0–79·2)	76·3 (75·0–77·6)	64·8 (61·7–67·4)	66·7 (63·4–69·5)	66·6 (63·2–69·6)	64·2 (60·8–67·2)
Utah	77·9 (77·6–78·2)	79·8 (79·5–80·0)	80·1 (79·8–80·3)	78·5 (77·2–79·7)	66·7 (63·4–69·5)	67·4 (64·0–70·5)	67·1 (63·5–70·2)	65·5 (61·9–68·8)
Vermont	76·7 (76·3–77·1)	80·0 (79·6–80·4)	80·2 (79·8–80·7)	79·1 (77·9–80·2)	65·9 (62·7–68·5)	68·0 (64·7–70·9)	67·5 (64·1–70·7)	66·4 (62·6–69·5)
Virginia	75·4 (75·3–75·6)	79·1 (78·9–79·3)	79·5 (79·4–79·7)	77·5 (76·1–79·0)	64·8 (61·7–67·5)	67·0 (63·6–69·9)	66·7 (63·3–69·9)	64·9 (61·4–68·1)
Washington	76·9 (76·7–77·1)	80·0 (79·8–80·2)	80·6 (80·4–80·8)	79·3 (77·9–80·9)	65·8 (62·6–68·6)	67·8 (64·4–70·7)	67·5 (63·8–70·6)	66·2 (62·5–69·8)
Washington, DC	67·3 (66·8–67·7)	77·3 (76·8–77·7)	78·6 (78·1–79·1)	77·0 (75·5–78·7)	58·0 (55·2–60·3)	65·8 (62·6–68·6)	66·3 (62·7–69·3)	64·8 (61·5–68·1)
West Virginia	74·7 (74·3–75·0)	75·4 (75·1–75·7)	74·8 (74·5–75·2)	72·0 (70·4–73·5)	63·9 (60·7–66·5)	63·4 (60·2–66·4)	62·1 (58·6–65·2)	59·5 (56·1–62·9)
Wisconsin	76·9 (76·7–77·1)	79·6 (79·4–79·8)	79·5 (79·3–79·7)	78·4 (76·8–79·9)	65·8 (62·7–68·5)	67·4 (63·9–70·3)	66·7 (63·1–69·8)	65·5 (61·6–68·9)
Wyoming	76·3 (75·9–76·7)	78·2 (77·8–78·5)	78·4 (77·9–78·9)	76·0 (74·9–77·2)	65·3 (62·1–68·0)	66·2 (62·8–69·0)	65·8 (62·2–68·8)	63·6 (60·1–66·9)

**Figure 2:**
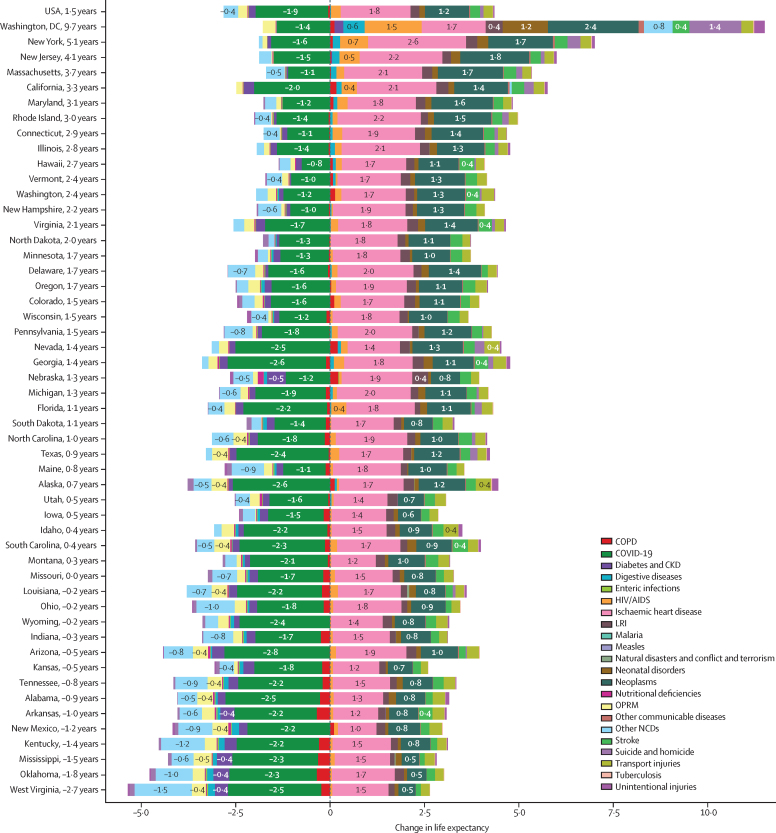
Change in life expectancy attributable to leading causes of death in 1990–2021 for the USA and among US states and Washington, DC CKD=chronic kidney disease. COPD=chronic obstructive pulmonary disease. LRI=lower respiratory infection. OPRM=other pandemic-related mortality.

Figure 2 shows the change in life expectancy attributable to the leading causes of death in 1990–2021, with details for different GBD cause levels and time periods available through GBD Compare. Ischaemic heart disease (IHD), neoplasms, and stroke contributed to improvements in life expectancy in 1990–2000 that increased in 2000–10 but slowed in the later periods. During the 1990–2021 study period, 1·8 years of life expectancy were gained in the USA due to declining rates of IHD mortality.

At the state level, in 2021, life expectancy ranged from 81·2 years (95% UI 79·6–82·8) in Hawaii to 71·9 years (70·3–73·5) in Mississippi (table). Hawaii also had the highest HALE (68·3 years [64·5–71·4]), while West Virginia had the lowest, at 59·5 years (56·1–62·9; table). Over the 1990–2021 study period, the world rankings of life expectancy and HALE at the US state level declined steadily relative to other countries around the world.^1^ When comparing life expectancy in the best-performing and worst-performing US states against all 203 other countries and territories in GBD 2021 (excluding the USA as a whole), Hawaii had the sixth-highest life expectancy in the world for males and fourth-highest for females in 1990, whereas Washington, DC (the worst-ranked US subnational location that year), ranked 136th for males and 82nd for females (figure 1C). In 2021, Hawaii remained the best-performing US state, but ranked just 28th for males and 22nd for females, whereas Mississippi (the lowest-ranked US state for males) ranked 107th for males and West Virginia (the lowest-ranked US state for females) ranked 99th for females.

US states performed even less favourably against world rankings for HALE (figure 1D). Hawaii ranked 14th for males and fifth for females for highest HALE globally compared against all other countries and territories in 1990 but declined to 39th for males and 34th for females in 2021 (figure 1D). Meanwhile, West Virginia—the lowest-ranked state in 2021—ranked 141st for males and 137th for females in 2021 (figure 1D). Over the entire study period, the lowest-ranked US subnational-specific HALE was for males in Washington, DC, in 1993, at 142nd (figure 1D).

14 US states lost life expectancy over the study period, with West Virginia losing 2·7 years between 1990 and 2021 (table; figure 2). For males, life expectancy declined in nine states, with the largest declines in West Virginia (2·0 years; appendix 1 p 15). For females, life expectancy declined over the study period in 17 states, with the largest declines in West Virginia (3·0 years; appendix 1 p 17). The biggest increase in life expectancy over the study period was observed in Washington, DC (9·7 years), followed by New York (5·1 years; table; figure 2).

When considering the leading causes of change in life expectancy over the entire study period, COVID-19 deaths contributed the most to declines in life expectancy across all states (figure 2). The largest COVID-19-related decline in life expectancy occurred in Arizona (2·8-year decline), whereas Hawaii had the smallest COVID-19-related decline (0·8-year decline). Across all states, declines in IHD deaths contributed the most to increases in life expectancy, with the largest increase in New York (2·6-year increase) and the smallest increase in New Mexico (1·0-year increase). Declines in neonatal disorder deaths also contributed to substantial increases in life expectancy across states, including a 2·4-year increase in Washington, DC (figure 2).

In the decade preceding the COVID-19 pandemic (2010–19), life expectancy decreased in New Mexico, West Virginia, New Hampshire, Ohio, South Dakota, Mississippi, Maine, Tennessee, Indiana, Arizona, Iowa, Kansas, Wisconsin, Kentucky, and Missouri for all sexes combined (table). The largest decrease over this pre-pandemic period was observed in New Mexico, with a loss of 0·72 years (table). This trend was more prevalent for males than females, with 16 states experiencing declines in life expectancy for males compared with 11 states for females (table). The largest decrease between 2010 and 2019 in males was in New Mexico (1·2 years) and in females in West Virginia (0·6 years; table).

### Mortality

**Figure 3:**
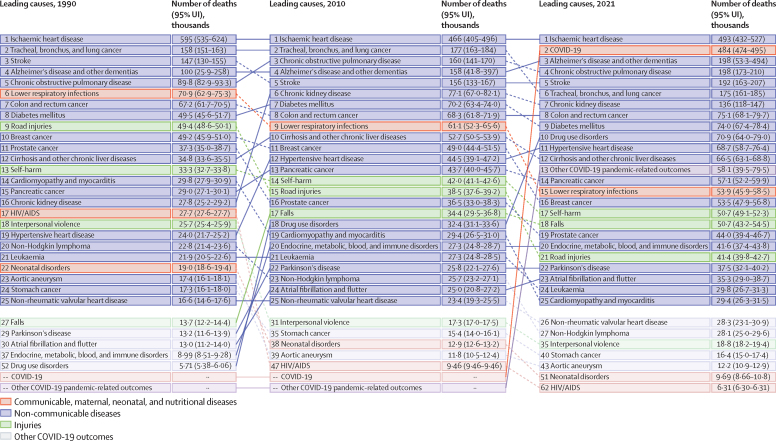
Leading 25 Level 3 causes of death in 1990, 2010, and 2021, in the USA, for all ages and sexes combined Causes are ranked by number of deaths in each year.

IHD was the leading GBD Level 3 cause of death in the USA every year between 1990 and 2021, causing approximately 493 000 deaths (95% UI 432 000–527 000) in 2021 (figure 3). COVID-19 accounted for 426 000 deaths (411 000–442 000) in the USA in 2020 (see online tools) and 484 000 (474 000–495 000) in 2021. The highest rates of COVID-19 deaths were in older age groups, and rates were higher in males than females (see online tools). For example, in 2021, there were 272 000 deaths (267 000–278 000) among males compared with 212 000 deaths (208 000–217 000) in females. For females, three 5-year age groups each experienced more than 25 000 COVID-19 deaths in 2021: 80–84 years, 85–89 years, and 90–94 years, compared with six age groups for males: every 5-year age group between 60–64 years and 85–89 years.

At the national level, age-standardised mortality rates for all sexes combined declined between 1990 and 2021 for IHD (56·1% decline [55·2–57·2]), stroke (31·3% (29·1–34·3]), and diabetes (17·0% [14·2–19·7]; appendix 1 p 4). Other significant decreases in age-standardised deaths were observed in many types of cancers, including lung cancer (41·9% decline [39·7–44·6]), breast cancer (40·9% [38·7–43·7]), colorectal cancer (37·8% [35·7–40·1), prostate cancer (36·3% [33·2–39·6]), lymphoma (34·3% [32·0–37·6]), and leukaemia (25·9% [24·0–28·7]; appendix 1 p 4). However, the age-standardised mortality rate for pancreatic cancer—which had the third-highest age-standardised mortality rate among cancers in 2021—increased by 6·3% (3·5–9·1). Age-standardised mortality rates for road injuries decreased by 41·3% (39·3–43·2) and for interpersonal violence by 39·4% (37·5–41·6). Age**-**standardised mortality rates for other causes increased, including drug use disorders by 878·0% (770·5–1015·5), chronic kidney disease by 158·3% (149·6–167·9), falls by 89·7% (79·8–95·8), hypertensive heart disease by 53·0% (38·6–66·5), and self-harm by 10·5% (6·8–14·3).

In 2021, there was considerable variation in age-standardised death rates for Level 3 causes by age and sex (see online tools). Lung cancer ranked as the third-leading cause of death for males and the sixth for females, with a more substantial decline observed in males (53·5% [95% UI 51·7 to 55·5] decline in the age-standardised rate) compared with in females (23·7% decline [20·5 to 27·9]) between 1990 and 2021. For all sexes combined, road injuries and self-harm were the two leading causes of death in children and adolescents aged 5–14 years, with road injuries decreasing by 69·0% (67·0 to 70·9) and self-harm increasing by 50·3% (38·9 to 62·0). However, self-harm showed a decline of 5·9% (–0·1 to 11·1) for those aged 15–19 years. Among individuals aged 20–24 years, drug use disorders were the leading cause of death for all sexes combined in 2021. For those aged 70 years and older, IHD was the leading cause of death in 2021, although the age-specific rate declined by 54·2% (52·9–56·4) from 1990.

We found significant variation in mortality rates between states, with Hawaii having the lowest age-standardised mortality rate in 2021 (433·2 deaths per 100 000 [95% UI 380·6–493·4]) and Mississippi having the highest (867·5 [772·6–975·7]; see online tools). Hawaii had the lowest age-standardised mortality rate throughout the study period, whereas Washington, DC, had the most improvement (a 40·7% [33·2–47·3] decline). West Virginia, Kentucky, and Alabama had the highest age-standardised mortality rates after Mississippi. All US states had COVID-19 as the leading or second-leading cause of death in 2021, except for Hawaii, where COVID-19 was fourth (appendix 1 p 5).

West Virginia had the highest age-standardised rate of deaths from drug use disorders in 2021 (50·4 deaths per 100 000 [95% UI 40·9–61·4]), followed by Ohio, Kentucky, and Pennsylvania, with North Dakota having the lowest (7·1 deaths per 100 000 [5·6–8·8]). Alaska had the highest age-standardised rate of self-harm deaths in 2021 (23·6 deaths per 100 000 [20·5–27·1]), and Washington, DC, had the highest age-standardised rate of interpersonal violence deaths (18·0 [15·4–20·9]). West Virginia had the highest age-standardised rate of diabetes deaths (21·9 [18·6–25·3]).

The leading causes of deaths varied by age at the state level. The largest differences in causes of deaths in 2021 across states were observed for adolescents and young adults aged 10–24 years (see online tools). In this age group, drug use disorders were the leading cause of death in Connecticut, New Jersey, Massachusetts, New York, Ohio, Rhode Island, and Pennsylvania. Interpersonal violence was the leading cause of death in Louisiana, Illinois, Maryland, and Washington, DC. COVID-19 was the leading cause of death in Georgia, Texas, Arizona, Nevada, and West Virginia. Road injuries were the leading cause of death in Alabama, Arkansas, California, Delaware, Florida, Kentucky, Mississippi, Nebraska, North Carolina, South Carolina, Tennessee, and Wyoming. Self-harm was the leading cause of death in the remaining 23 states. By comparison, the leading cause of death in 2021 for those aged 70 years or older was IHD in all states except for Alabama, Alaska, Arizona, Georgia, Idaho, Kentucky, Mississippi, South Carolina, Texas, West Virginia, and Wyoming, where it was COVID-19. For infants, children, and adolescents aged 0–14 years, COVID-19 varied in ranking from the 12th-leading cause of death in Arizona to the 34th in Minnesota.

### Disability

In 2021, the leading Level 2 causes of age-standardised YLDs for all sexes combined were musculoskeletal disorders, mental health disorders, and substance use disorders (see online tools). For males, musculoskeletal disorders were the leading Level 2 cause, with an increase of 12·0% (95% UI 6·6%–18·6%) from 1990 to 2021. For females, mental disorders were the leading Level 2 cause, with an increase of 30·2% (25·6–34·9) from 1990. Disability varied widely by age, sex, and state. For example, mental health disorders were the leading cause of YLDs for all sexes combined aged 10–39 years, whereas musculoskeletal disorders were the leading cause for ages 40–89 years. At Level 3, low back pain had the highest age-standardised YLD rate in 1990 and 2021, although this rate declined by 7·9% (1·8–13·0) from 1990 (appendix 1 p 6). Depressive disorders and drug use disorders were the second-leading and third-leading Level 3 causes of age-standardised YLDs in 2021. Depressive disorders increased by 56·0% (48·2–64·3) over the study period, and drug use disorders increased by 287·6% (247·9–329·8). Considering age-standardised YLD rates for all sexes combined around the world, only six countries had higher rates in 2021 than the USA: Afghanistan, Lesotho, Liberia, Mozambique, South Africa, and the Central African Republic, largely because the impact of musculoskeletal disorders, mental health disorders, and substance use disorders on age-standardised disability rates in the USA is so large.^5^

**Figure 4:**
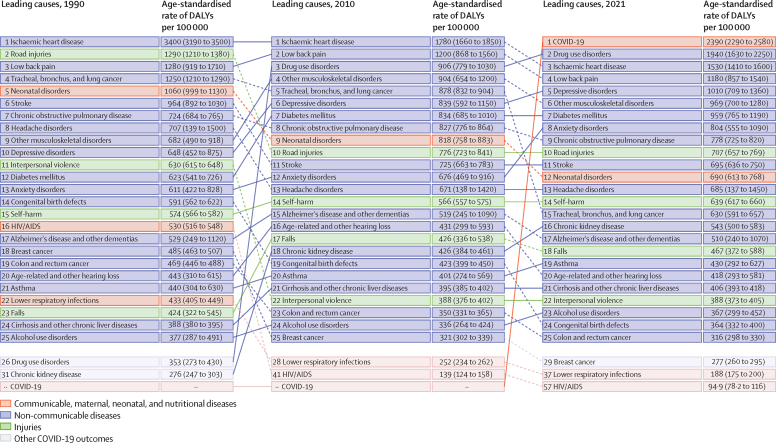
Leading 25 Level 3 causes of age-standardised DALYs in 1990, 2010, and 2021, in the USA, for all sexes combined Causes are ranked by the age-standardised rate of DALYs in each year. DALY=disability-adjusted life-year.

The leading Level 2 cause of age-standardised DALYs for all sexes combined was cardiovascular diseases, followed by neoplasms, musculoskeletal disorders, and mental health disorders, with cardiovascular diseases declining by 41·0% (95% UI 39·5–42·4) and neoplasms by 33·2% (31·9–34·7; appendix 1 p 7). The order of the top four Level 2 causes did not change from 1990. For females, the leading Level 2 causes of age-standardised DALYs in 2021 were mental disorders, followed by musculoskeletal disorders. For males, the leading Level 2 causes were cardiovascular diseases, followed by respiratory infections and tuberculosis (mainly COVID-19). When considering Level 3 causes, COVID-19 was the leading cause of age-standardised DALYs in 2021, followed by drug use disorders, for males, females, and all sexes combined (figure 4).

DALYs varied by age and sex, with males having a greater burden from self-harm and interpersonal violence as well as substance use disorders in all age groups compared with females (see online tools). For example, for males aged 20–24 years, 10·6% (95% UI 8·9–12·8) of DALYs in 2021 were due to self-harm and interpersonal violence and 19·6% (17·0–22·6) from substance use disorders, compared with 4·1% (3·3–5·2) due to self-harm and interpersonal violence and 16·7% (13·9–19·9) from substance use disorders for females in the same age group. Conversely, females had greater burden from mental disorders compared with males. For example, 27·4% (22·6–32·2) of DALYs were from mental disorders for males aged 10–14 years, 20·3% (16·8–24·0) for those aged 15–19 years, and 14·5% (12·0–17·4) for those aged 20–24 years, compared with 32·7% (26·8–39·4) for females aged 10–14 years, 32·4% (27·4–38·5) for those aged 15–19 years, and 25·7% (21·5–31·0) for those aged 20–24 years.

At the state level, Hawaii had the lowest age-standardised rates of YLDs for all sexes combined (12 085·3 per 100 000 [95% UI 9090·8–15 557·1]), whereas West Virginia had the highest (14 832·9 [11 226·9–18 882·5]; appendix 1 p 70). Eight states had the highest age-standardised rates of YLDs for all sexes combined in the world in 2021, when comparing all US states plus Washington, DC, against all 204 countries and territories: West Virginia, Kentucky, Oklahoma, Pennsylvania, New Mexico, Ohio, Tennessee, and Arizona. Afghanistan ranked ninth behind these states.^5^

**Figure 5:**
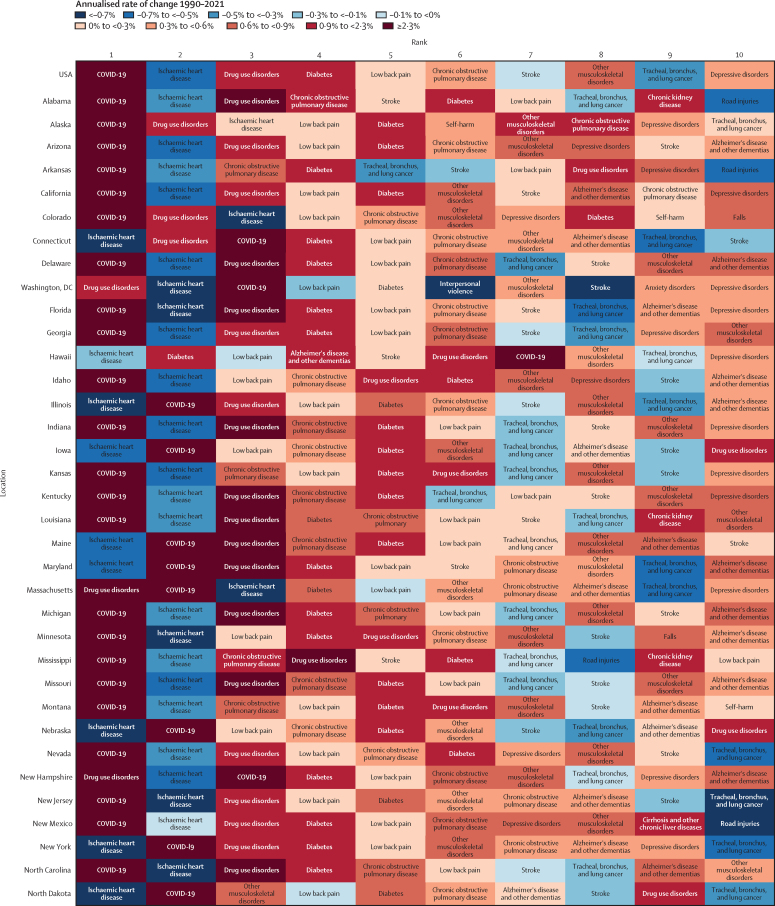

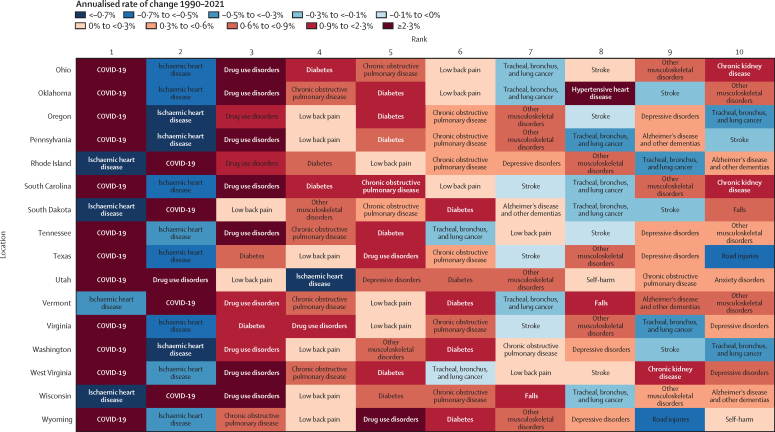
Annualised rate of change in age-standardised DALY rates, 1990–2021, for the ten leading Level 3 causes of DALYs for the USA and by US state and Washington, DC Causes are ranked by number of DALYs. Annualised rate of change reflects the change in the age-standardised rate of DALYs between 1990 and 2021. DALY=disability-adjusted life-year.

At the state level, the leading Level 3 cause of DALYs for all sexes combined was COVID-19 in most states (figure 5). Hawaii was an exception, with COVID-19 being just the seventh-leading cause of DALYs in 2021 (ranked by number of DALYs). For males, IHD was the leading cause in Hawaii, Iowa, Nebraska, New York, and South Dakota, whereas drug use disorders were the leading cause in Connecticut; Delaware; Washington, DC; Illinois; Maine; Maryland; Massachusetts; New Hampshire; New Jersey; North Carolina; Ohio; Pennsylvania; Rhode Island; South Carolina; Vermont; Washington; West Virginia; and Wisconsin (appendix 1 p 8). For females, drug use disorders were the leading cause of DALYs in 22 states, COVID-19 in 18 states, depressive disorders in six states, low back pain in three states, and musculoskeletal disorders in two states (appendix 1 p 9). Findings for all-cause age-standardised DALY rates by state between 1990 and 2021 are given in appendix 1 (p 10).

Figure 5 shows the annualised rate of change in the age-standardised DALY rate for the leading Level 3 causes of DALYs (ranked by number of DALYs in 2021) between 1990 and 2021 for the USA and all US states. Over the study period, there was a general decrease in age-standardised DALY rates for IHD, stroke, and several cancers, alongside rapid increases in drug use disorders and diabetes. The age-standardised rates of drug use disorders increased in all states over the study period, but to different extents. For example, rates increased by 1105·1% (95% UI 935·0–133·8) in West Virginia, 580·3% (477·8–691·9) in Florida, 482·2% (403·9–577·9) in Wyoming, and 463·0% (377·2–566·0) in Colorado. State-specific YLDs, YLLs, and DALYs by cause are available in appendix 1 (pp 19–121).

### Risk factors

**Figure 6:**
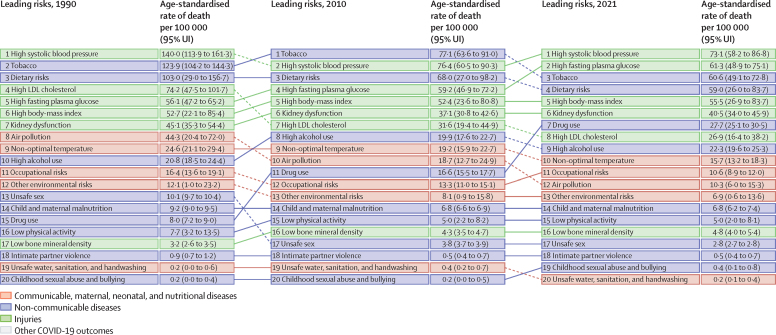
Leading Level 2 risk factors for age-standardised risk-attributable deaths in 1990, 2010, and 2021, in the USA, for all sexes combined Risk factors are ranked by age-standardised rate of deaths attributable to the risk factor in each year.

At the national level, the leading Level 2 risk factors for death in 2021 for all sexes combined (both for number of deaths and age-standardised death rate) were high systolic blood pressure, high fasting plasma glucose, tobacco use, dietary risks, and high BMI (figure 6). From 1990, the age-standardised rates for high systolic blood pressure decreased by 47·8% (95% UI 43·4 to 52·5) and for tobacco by 51·1% (48·3 to 54·1), but rates increased for high fasting plasma glucose by 9·3% (0·4 to 18·7) and high BMI by 5·2% (–4·8 to 25·7; figure 6).

Risk-attributable burden varied by age and sex. For example, for ages 15–49 years, the leading Level 2 risk factors for death were drug use, high alcohol use, and dietary risks (see online tools). By contrast, for ages 50–69 years, tobacco was the leading risk factor for death, followed by dietary risks and high BMI. For all ages combined, tobacco was the second-leading Level 2 risk factor for death for males but the fifth for females. In the USA in 2021, the leading Level 2 risk factor for number of attributable DALYs was high BMI, followed by high fasting plasma glucose and tobacco use. For the age-standardised rate of risk-attributable DALYs, the leading Level 2 risk factors in 2021 were—in descending order—drug use, high BMI, high fasting plasma glucose, tobacco use, and dietary risks (appendix 1 p 11). These five risk factors each contributed over 5% of total DALYs. Tobacco use declined from first in 1990 to fourth in 2021 (ranked by age-standardised rate of attributable DALYs), with a decline in risk-attributable age-standardised DALY rate of 48·8% (95% UI 45·7–52·1). High systolic blood pressure likewise declined from second to sixth, with a decline of 45·2% (41·3–49·3). From 1990 to 2021, age-standardised DALY rates for high BMI increased by 24·9% (11·4–46·3), for high fasting plasma glucose by 34·1% (24·5–43·5), and for drug use by 274·0% (240·6–308·3).

**Figure 7:**
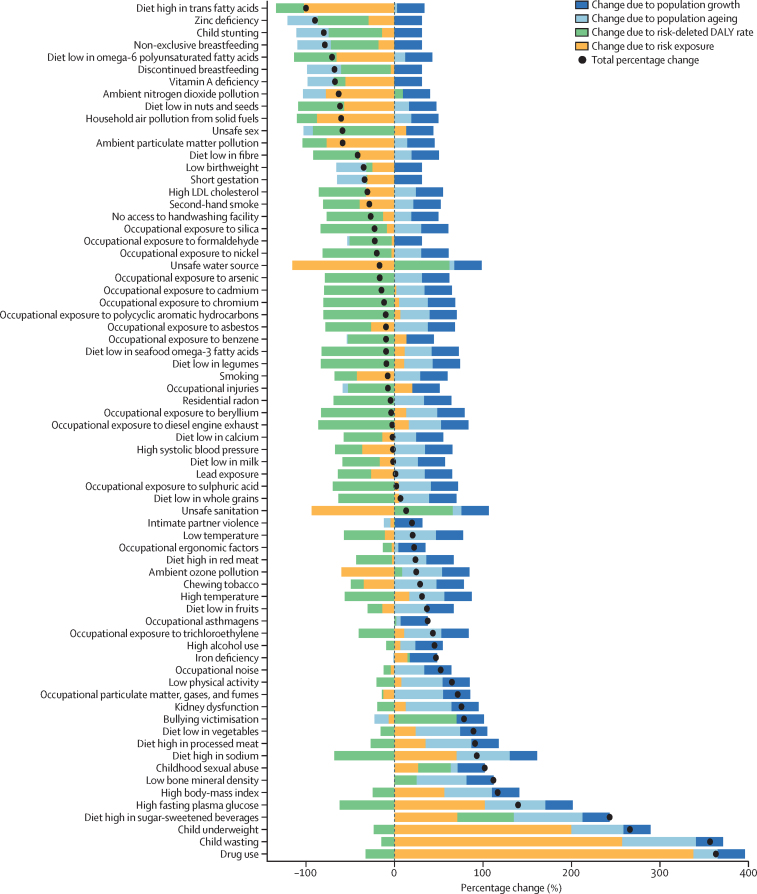
Percentage change in the number of DALYs attributable to Level 4 risk factors in the USA, 1990–2021 This decomposition analysis visualises changes in risk-specific attributable DALYs from 1990 to 2021 due to changes in risk exposure, population growth, population age structure, and risk-deleted DALYs. Risk-deleted DALY rates are DALY rates after removing the effect of a risk factor or combination of risk factors on overall rates. They are calculated as the overall DALY rate multiplied by one minus the population attributable fraction for the risk or set of risks; this calculation isolates the underlying changes in DALY rates unattributable to risk factors. DALY=disability-adjusted life-year.

Between the years 1990, 2000, 2010, and 2021, age-standardised SEVs for environmental and occupational risk factors and behavioural risk factors declined, but increased for metabolic risk factors, from 25·5 (22·4–28·5) in 1990 to 37·5 (33·1–39·9) in 2021 (appendix 1 p 172). The decomposition of changes in number of DALYs and deaths attributable to Level 4 risk factors over the study period due to population growth, population ageing, changes in risk-deleted DALY rates, and changes in risk exposure are shown in figure 7 and appendix 1 (p 12). The number of deaths and DALYs attributable to chewing tobacco, high BMI, high fasting plasma glucose, and diet low in fruits and vegetables increased over the study period. Ageing and population growth drove increases in DALYs for nearly all risk factors, whereas for many risk factors, declines were primarily due to declines in risk-deleted mortality and DALYs. The large changes observed in deaths attributable to unsafe sanitation and water sources and in deaths and DALYs attributable to child underweight and wasting were mainly driven by small numbers.

For nearly all states, behavioural risks were the leading risk factors contributing to age-standardised death rates for males, followed by metabolic and then environmental and occupational factors (with the exception of Utah; appendix 1 p 13 and online tools). For females, metabolic risks were the leading risk factors for age-standardised death rates in most states, with several exceptions (Alaska, Delaware, Kentucky, Missouri, Montana, Nevada, New Mexico, Tennessee, West Virginia, and Wyoming). By contrast, for age-standardised DALY rates, the leading risk factors were behavioural in all US states for both males and females.

For risk-attributable age-standardised DALY rates, the leading Level 2 risk factor for all sexes combined was high BMI in Alabama, California, Georgia, Hawaii, Idaho, Illinois, Iowa, Kansas, Louisiana, Maryland, Minnesota, Mississippi, Nebraska, New York, North Dakota, Oklahoma, South Carolina, South Dakota, Texas, and Virginia (appendix 1 p 14, online tools). Tobacco use was the leading risk factor for age-standardised DALYs in Arkansas, whereas drug use was the leading risk factor in the remaining 30 states plus Washington, DC. For males, tobacco use was the leading Level 2 risk factor for age-standardised DALYs in Alabama, Arkansas, Iowa, Kansas, Louisiana, Mississippi, Montana, South Carolina, South Dakota, and Wyoming, whereas high BMI was the leading risk factor in California, Georgia, Hawaii, Idaho, Minnesota, Nebraska, North Dakota, Oklahoma, Texas, and Virginia. The remaining 31 states plus Washington, DC, were led by drug use. For females, high BMI was the leading risk factor for age-standardised DALYs in most states, except for Alaska, Arizona, Colorado, Connecticut, Florida, Kentucky, Maine, Massachusetts, Montana, Nevada, New Hampshire, New Mexico, Ohio, Pennsylvania, Rhode Island, Tennessee, Washington, West Virginia, and Wyoming.

West Virginia had the highest age-standardised rate of risk-attributable DALYs (16 567·5 per 100 000 [95% UI 14 369·2–18 762·4), whereas Minnesota had the lowest (8592·7 per 100 000 [7267·1–9959·2]; appendix 1 p 141). The age-standardised SEV for all risk factors combined varied between states, with Rhode Island having the highest exposure (30·1 per 100 000 [26·8–33·4]), and Alaska having the lowest (24·0 per 100 000 [21·0–27·1]; appendix 1 p 172).

To examine risk factors’ contributions to disease burden using the BPRF approach, we present age-standardised DALY rates per 100 000 at the US state level for all GBD risk–outcome pairs combined and when excluding those with one-star or two-star associations (appendix 1 p 276). The age-standardised DALY rate varied by state but, when excluding the one-star and two-star associations, the patterns in variation remained similar, indicating a large contribution from the main risk factors.

## Discussion

Our findings show that over the past 30 years, overall population health in the USA has declined compared with that of other countries, including nearly all high-income countries and several middle-income countries; most notably, the global ranks of life expectancy and HALE in the USA declined. Even as the USA increased health spending, undertook large-scale public health campaigns, and used new medical technologies and advancements in treatments, the overall picture of population health in the country and across the states did not look markedly better in 2021 than it did three decades ago. These findings highlight the ongoing challenges that the USA faces in addressing the burden of NCDs and the growing impact of obesity, mental health disorders, and drug use on the quality and length of life in the USA. In addition to informing both state and national health policy, GBD 2021 results can be used by researchers to identify knowledge gaps and areas for further investigation. Importantly, by presenting comprehensive and up-to-date estimates of mortality, morbidity, and risk factors, GBD 2021 enables policy makers and other stakeholders to develop action plans to prioritise for interventions, allocate resources more effectively, and evaluate the effect of health policies and programmes.

The COVID-19 pandemic has had a profound impact on the USA, causing significant setbacks in public health and further highlighting existing disparities within the population.^11^ Systemic racism, socioeconomic vulnerability, access to education, and other structural inequities contributed to the disproportionally high burden of COVID-19 on minority groups, including Native American, Hispanic, and African American individuals, in several ways. First, these disparities contributed to higher proportions of minority groups working in essential worker roles, with less ability to work from home.^11^, ^40^, ^41^ Second, these communities were burdened by higher rates of underlying health conditions that were associated with a higher risk of severe illness and mortality. Third, they were more likely to live in places with insufficient access to health care as compared with White people.^11^, ^40^, ^42^ As a result, although Hispanic populations generally experience lower rates of death for nearly every cause of death than White populations, Hispanics experienced higher mortality rates than White people due to the COVID-19 pandemic. The pandemic exposed the nation's unpreparedness and shortcomings in its response to the crisis, particularly for the most vulnerable populations, further exacerbating the situation.^10^ Fortunately, our findings did not indicate the substantial negative impact on children that many had initially feared,^43^ as the highest death rates were observed among the older populations. Nonetheless, COVID-19 has highlighted the urgent need for better preparedness; more equitable health-care access; greater effort to address social determinants of health and reduce economic, educational, and other structural disparities; and targeted support for vulnerable communities in the face of future public health emergencies.

The USA has experienced a range of health changes and challenges since 1990, with GBD identifying key risk factors contributing to the shifting burden of disease. Apart from the COVID-19 pandemic, several diseases, injuries, and risk factors have substantially impacted the nation. The ongoing opioid crisis has led to increased overdose deaths and addiction cases, straining health-care systems and affecting communities across the country. Chronic diseases such as heart disease, cancer, and diabetes persist as major health concerns. Between 1990 and 2021, obesity and risk factors associated with diabetes increased more than other chronic conditions. Injuries, such as motor vehicle injuries and gun violence were not among the ten leading causes of disability and death in the USA in 2021, but rates were nonetheless higher than in any other high-income country. The burdens of anxiety, depression, and suicide have also increased over the study period. Although some of this increase could be due to better awareness and less stigma surrounding seeking help for mental disorders,^44^ this is unlikely to entirely explain the increased burden, particularly during the COVID-19 pandemic. Despite heterogeneity in the data, there is widespread evidence that rates of this increase accelerated during the COVID-19 pandemic.^45^, ^46^, ^47^, ^48^ For instance, using nationally representative sample data from the US Census Bureau, Twenge and Joiner^49^ found that US adults were more than three times as likely to screen positive for either or both depressive and anxiety disorders in April–May 2020 than in 2019. A 2022 systematic review and meta-analysis^50^ of 255 eligible studies from 50 countries similarly found higher levels of anxiety and depression during the pandemic compared with at baseline, with rates of a probable mental disorder similar to those observed in previous pandemics, major disasters, and armed conflicts. The GBD 2021 Mental Disorders Collaborators^51^ estimated increases of nearly 26% in major depressive disorders and 28% in anxiety disorders globally in 2020 due to the COVID-19 pandemic, with lower human mobility and higher daily infection rates significantly associated with higher mental disorder prevalence. These findings are not unique to the COVID-19 pandemic, with previous studies finding significant increases in mental health disorders during other major shocks, such as previous epidemics^52^, ^53^ and economic crises.^54^, ^55^, ^56^, ^57^

In this Article, we have not specifically examined health disparities by race and ethnicity. However, in our other publications,^16^, ^17^, ^58^, ^59^, ^60^ we have shown significant variations in life expectancy and mortality at the county level by race and ethnicity. For instance, one of our studies^17^ revealed that life expectancy for Native American and Alaska Native populations remained unchanged from 2000 to 2019, whereas it improved for other race and ethnicity groups. Addressing health disparities among racial and ethnic minority groups is crucial for improving overall health outcomes in the USA. Factors such as discrimination, language barriers, and cultural differences might contribute to the unequal distribution of health resources and services among these populations.^61^, ^62^ Disparities in health outcomes based on race and ethnicity, socioeconomic status, access to health care, and variability in exposure to preventable risk factors also highlight the need to address social determinants of health and health inequities. Factors such as income, education, and employment status have a considerable impact on an individual's health outcomes.^63^, ^64^, ^65^, ^66^ For example, low socioeconomic status can contribute substantially to difficulties in accessing health-care services, nutritious food, affordable public transport, and safe housing, which in turn negatively affect overall health among low-income populations, particularly those below the poverty line.^67^, ^68^

The Affordable Care Act (ACA) has played a notable role in expanding health-care coverage and access to medical care in the USA since its implementation in 2010. Despite its success in putting millions more on health insurance who were previously uninsured, improvements in health outcomes as a result of better access to medical care appear to have stalled during the period of expanded access compared with the preceding two decades (1990–2010). This finding suggests that health policies that only increase the proportion of the US population with insurance are only one part of the comprehensive strategy that is needed to reduce health inequities and better population health. We have previously shown that the contribution of the health-care system is not enough to reduce health disparities.^69^ By developing health programmes tailored to local communities that respect cultural differences, and addressing socioeconomic determinants, risk behaviours, environmental influences, and health disparities among minoritised populations, we can work towards creating a more equitable health-care system and a healthier nation for everyone in the USA. The National Institutes of Health (NIH) have recently taken a commendable step towards addressing community health. Although the NIH and Centers for Disease Control and Prevention (CDC) still have a largely disease-focused approach, this strategic shift acknowledges the importance of community health as an essential aspect of the overall wellbeing of the nation.^70^ Through initiatives such as the Community Engagement Alliance (CEAL), the NIH has shown its commitment to improving health outcomes at the grassroots level.^70^ CEAL's efforts have shown promising results, particularly in encouraging COVID-19 vaccine uptake during the pandemic.^71^ By investing in community health, the NIH and CDC are not only strengthening local resilience to public health challenges but also empowering communities to take charge of their health and wellbeing. This innovative approach to health research and funding has the potential to create lasting, positive change, as it addresses the root causes of health disparities and promotes a more equitable health-care landscape for all. Comprehensive and well-functioning community health services are likewise useful for pandemic preparation, early detection, response, and recovery, as well as for other outbreaks and disasters.

Addressing exposure to risk factors such as tobacco use, drug use, poor diet, obesity, physical inactivity, and excessive alcohol consumption is essential because these behaviours are major contributors to chronic diseases and premature mortality.^21^ Public health policies and interventions aimed at reducing these and other risk factors can contribute to substantial improvements in population health. For example, investing in community-based programmes that promote healthy lifestyle choices can help combat obesity, reduce the prevalence of type 2 diabetes, and lower the risk of heart disease.^72^, ^73^, ^74^, ^75^ Another important aspect to consider is the effect of the built environment on health outcomes. Access to safe and well-maintained recreational spaces, public transportation, and affordable housing can influence an individual's ability to maintain a healthy lifestyle.^68^, ^76^, ^77^, ^78^ By working to improve environmental factors within communities, policy makers can create an atmosphere that supports and encourages positive health behaviours. Although the successful implementation of smoking policies has shown that it is possible to reduce harmful health behaviours,^79^ policies to address obesity, diet, drug and alcohol use, and suicide have so far been less effective. National public health agencies in the USA, such as the CDC, can address leading causes of risk-attributable deaths and burden by prioritising the development of guidelines and implementation guidance of evidence-based interventions for the states to implement. The CDC has not always allocated funding to programmes that are regularly evaluated for impact and effectiveness. For example, the State Physical Activity and Nutrition (SPAN) programme was established by the CDC in 2013. SPAN benefitted from a large investment in obesity prevention, but the programme has not been thoroughly evaluated, even amid the ongoing rise in obesity rates.^80^, ^81^, ^82^ Setting evidence-based guidelines, established standards, and expanding investments in evaluation research on interventions that could reduce risk-attributable burden through evidence-based programmes should be a priority moving forward for both federal and state-level agencies.

The implementation of evidence-based programmes will need to coincide with greater attention paid to addressing commercial and political determinants of health, including the tobacco industry, food industries, fossil fuel industries, and the gun lobby and industry. These powerful industries heavily influence risk factor exposure in the USA and are often in opposition to human health; therefore, any comprehensive approach to addressing health risk must consider and work to diminish the role these industries play. The issue of gun control in the USA is a prime example. It is highly contentious and politically charged, fuelled by a combination of a prevailing interpretation of the Second Amendment of the US Constitution that protects an individual's right to bear arms, the influence of a powerful gun lobby, and deeply ingrained cultural attitudes towards firearms.^83^ Although gun violence contributes less than other health conditions to total DALYs in the USA, it remains a greater issue in the USA than in any other high-income country, with the USA ranked first in age-standardised deaths due to physical violence by firearm among all high-income countries and 29th in the world, as well as second in the world for age-standardised deaths due to self-harm by firearm.^4^ Despite these relatively high rates of gun-related violence and suicide, the USA has not taken meaningful steps towards implementing comprehensive gun control policies that could reduce the frequency and scale of such tragic gun-related incidents.^84^, ^85^ The power of the gun lobby in shaping public opinion and influencing political decisions stifles meaningful progress on gun-control legislation.^86^, ^87^ The country's reluctance to address this public health crisis head-on is in stark contrast with the actions taken in other countries that have experienced mass shootings or high rates of gun-related violence, such as Australia and Norway.^88^, ^89^ These nations have implemented robust gun-control measures, often with remarkable success in reducing gun-related deaths. The USA must prioritise the safety and wellbeing of all within its borders by implementing evidence-based policy changes that focus on the public health implications of gun violence.

In addressing the disease burden in the USA, it is crucial to acknowledge the growing issue of drug use and addiction, a problem that has been largely exacerbated by the over-prescription of pain medications.^90^ The failure of pain clinics and other health providers to regulate the distribution of these medications has led to a widespread opioid crisis, resulting not only in increased addiction rates but also a substantial strain on our health-care system.^90^ In recent years, the increasing availability of cheaper, more potent alternatives such as fentanyl has worsened the problem even further.^91^, ^92^ The policies needed to slow and ultimately reverse drug use trends in the USA include tightening regulations on prescription practices, investing in effective prevention and treatment programmes, and fostering a greater understanding of addiction as a public health concern rather than a criminal issue.^93^ Similarly, US authorities must redouble their efforts to control the flow of illegal drugs, which can be aided through international cooperation and intelligence-sharing among law enforcement agencies. By proactively addressing the root causes of drug addiction and implementing evidence-based policies, we can reverse the growing burden of drug use on Americans’ health and avert an even more challenging future.

Mental health remains a stigmatised and under-addressed issue, and in the USA, is often segregated from physical health at both the programmatic level—with the US CDC focusing on physical health and SAMHSA focusing on mental health—and patient level, with limited coordination between primary care physicians and mental health doctors and treatment facilities.^94^ Policy makers and the public should push for a more comprehensive approach to health care that encompasses both mental and physical wellbeing, emphasising prevention, and ensuring that those without mental health issues remain healthy. The current system, where the CDC focuses primarily on physical health while SAMHSA combines mental health with substance abuse, only perpetuates the taboo that mental health issues inevitably lead to drug problems and vice versa. This flawed structure not only reinforces stigma but also hinders our ability to effectively tackle mental health challenges. To build a healthier, more resilient society, we must integrate mental health into all aspects of health care and prioritise preventive measures on national and state levels. However, there is limited knowledge surrounding preventive programmes for mental health. There is a pressing need for more research in this area to identify effective interventions and promote mental wellbeing to improve overall public health and quality of life.

As the US population continues to age,^1^, ^20^ we must recognise the importance of addressing the health-care needs of older people. With an increasing number of retirees and a dwindling workforce due to declining fertility rates,^20^ the financial burden on health-care systems and social safety nets will become even more pronounced.^20^, ^95^ Members of the younger generations face their own challenges as they struggle to save for retirement while grappling with mounting expenses. To mitigate these issues, it is important to explore viable solutions that can ensure the wellbeing of the ageing population while simultaneously supporting economic growth. Innovations to the labour force might include advancements in robotics and artificial intelligence, increased productivity in older ages, shifts in leading US labour sectors, and declines in underemployment, although the potential effects of such changes are complex and difficult to predict.^96^ Policies that provide support to parents to care and pay for their children might also contribute to a small increase in fertility rates as well as greater workforce participation among parents, in addition to myriad health and social benefits.^20^ Another possible but complex solution to the economic and health-care system-related challenges of an ageing population is the ethical and strategic implementation of more open immigration policies.^20^ Our previous work^95^ has found that declining fertility rates and population ageing pose substantial challenges to the economy as a whole—due to unmet labour needs—and to a country's ability to care for its elderly population because taxes from the working-age population fund health care and other social safety nets for older people in the USA (Medicare and Social Security). This study^95^ also found that liberal immigration policies would help the USA to maintain its position as the leading global economy in 2100. As such, welcoming immigrants into the USA, particularly those whose skills and qualifications align with the needs of the US workforce, could help offset the challenges posed by declining fertility rates and an ageing population and bolster the country's economy. However, future immigration policies must be developed with global cooperation and with consideration for the potential harmful effects on the countries and economies these migrants emigrate from.^97^, ^98^

The pursuit of medical advancements is a delicate balance between uncovering innovative medications and devices to alleviate the burden of disease, while also ensuring that existing, effective treatments are efficiently disseminated and implemented. For example, although the medical field has made significant strides in developing an array of safe and effective antihypertensive medications, many of which are available as affordable generics, a large percentage of Americans with high blood pressure still struggle with uncontrolled symptoms. In fact, national rates of blood pressure control have been on a downward trend since 2011, highlighting the need for a more preventive holistic approach to health care.^99^, ^100^ This challenge underscores the importance of not only advancing medical research but also prioritising the accessibility and implementation of established treatments, ultimately striving to improve patient outcomes and public health. At the same time, the USA could greatly benefit from new drugs such as GLP-1 agonists to help reduce obesity and subsequently reduce the risk of diabetes and other obesity-related diseases, if those in need can have affordable access to them.^101^, ^102^

The USA consistently underperforms in key health indicators when compared with other high-income countries and even low-income and middle-income ones. This sobering but unsurprising finding reflects an ongoing pattern of declining health in the USA relative to that of other countries, as identified in previous iterations of GBD.^103^, ^104^, ^105^, ^106^, ^107^, ^108^, ^109^, ^110^ Increases in obesity and diabetes have negatively impacted the public health systems and rankings of the USA compared with the rest of the world. Despite its vast resources, the USA has higher age-standardised rates of YLDs than nearly all countries around the world; in fact, eight US states have higher rates than any country or territory. Life expectancy and HALE in the USA have similarly declined over the past 30 years compared with other countries; the USA ranked 47th for females and 46th for males in life expectancy and 76th for females and 69th for males for HALE out of 204 countries and territories in 2021. The question remains: for how long can we accept and ignore such poor health outcomes without addressing the root causes and taking decisive action to improve the wellbeing of the US population? An alarm needs to keep sounding about the deteriorating state of health in the USA, pointing to the substantial influence of risk factors and social determinants on health outcomes.^28^, ^111^

To address its health challenges, the USA must first consider how much it values and prioritises the health of its population. Doing so will necessitate a more critical look at the country's policy choices at federal, state, and local levels, irrespective of political ideology. Agencies must also collaborate on implementing evidence-based interventions, promoting preventive measures, increasing access to health-care services, and addressing social determinants of health if innovative and more expansive policies are to succeed. These interventions should focus on first strengthening health-care infrastructure, including increasing the capacity and resources of health-care systems to handle the ongoing burden of chronic diseases, the opioid crisis, and mental health issues, while managing the long-term effects of the COVID-19 pandemic. The use of telehealth services can improve access to care, especially in rural and underserved areas.^112^, ^113^ Second, they should focus on implementing evidence-based preventive measures, such as promoting and supporting healthy lifestyle choices, encouraging early detection and screening for chronic diseases, and addressing the root causes of health disparities.^114^, ^115^ Such measures include developing policies and initiatives targeting income, education, employment, housing, and neighbourhood conditions, as well as ensuring equitable distribution of resources and health-care services to vulnerable populations, such as historically minoritised groups such as racial and ethnic minorities struggling with the double burden of economic hardship and lack of access to quality health care, low-income communities, and older adults. Third, they must include expanding access to mental health services, including increasing funding and resources for mental health services, with a particular focus on early intervention and prevention. Implementing mental health awareness and stigma reduction campaigns can encourage help-seeking behaviours. Fourth, they should prioritise strengthening public health preparedness and response, such as improving surveillance systems and data sharing across local, state, and national levels to rapidly detect and respond to emerging health threats. Coordination between public health agencies, health-care providers, and community organisations will effectively manage public health crises, such as the COVID-19 pandemic and the opioid epidemic. And finally, they must emphasise investing in research and innovation to support the development of new treatments, vaccines, and diagnostic tools for chronic diseases and emerging public health threats. Further investigations of how state-level policy interventions and programmes have actually impacted health outcomes will help develop stronger evidence bases for how to improve population health in the USA.

Given the scope of this analysis, this study has several limitations. The overall limitations of the GBD methods as noted in other publications apply to the US analysis.^1^, ^4^, ^5^, ^20^, ^21^ First, the accuracy of the estimates depends on the availability of data by time period and state. This limitation is particularly true for dietary intake data at the state level. GBD methods therefore adjust our dietary intake estimates using commercial sales data. Likewise, some data used in the analyses were of poorer quality and less consistency across certain states and age groups. Second, it is challenging to separate measurement error from variation in disease occurrence. GBD corrects for known biases from non-reference methods or case definitions, but often has to rely on sparse data at the state level to make those adjustments. Third, across US states, there is some variability in how inpatient versus outpatient settings treat different diseases. GBD methods attempt to adjust for these and other potential biases by using a covariate on hospital admission and claims data. Fourth, although this study reported on health disparities between US states, we did not investigate within-state variation. Reporting at the state rather than county or ZIP-code level masks potentially large within-state disparities, especially in large states or between urban and rural areas. Finally, this study largely did not investigate the burden of the many social determinants of health, including housing, racism and discrimination, neighbourhood conditions, and transportation, and focuses solely on behavioural, metabolic, and environmental and occupational risks. Despite these limitations, this study provides valuable insights into US health trends and challenges. Future development and improvement of data sources, methodologies, and computational resources will help address these limitations and produce more accurate and comprehensive GBD estimates.

GBD 2021 delivers an exhaustive evaluation of health trends and risk factors on a global, regional, national, and subnational scale. Specifically focusing on the USA, this study analyses the burden of diseases, injuries, and risk factors, while shedding light on health outcome disparities across states. This study serves as an important resource for policy makers, health-care professionals, and researchers within the USA at both the national and state levels, enabling them to prioritise interventions, allocate resources efficiently, and assess the effectiveness of health policies and programmes. GBD 2021 presents an updated analysis, incorporating the impact of the first 2 years of the COVID-19 pandemic on health trends and risk factors at the state level within the USA. It exposes the disparities in health outcomes and risk factors across states, emphasising the necessity for customised strategies to tackle specific health challenges. By offering comprehensive and up-to-date estimates on mortality, morbidity, and risk factors, GBD 2021 equips stakeholders in the USA with the data needed to identify intervention priorities, optimise resource allocation, and gauge the success of health policies and programmes. This study highlights substantial deficiencies in the country's health system and arms policy makers, health-care professionals, researchers, and the general public with the insights required to initiate a unified effort towards overhauling the US health-care system and improving population health.

### GBD 2021 US Burden of Disease Collaborators

### Affiliations

### Contributors

### Data sharing

To download the data used in these analyses, please visit the Global Health Data Exchange (https://ghdx.healthdata.org/gbd-2021/sources).

Correspondence to: Prof Ali H Mokdad, Institute for Health Metrics and Evaluation, University of Washington Seattle, WA 98195, USA mokdaa@uw.edu

## Declaration of interests

S Ashina reports consulting fees from AbbVie, Allegan, Eli Lilly and Company, Lundbeck, Theranica, Linpharma, Satsuma, and Pfizer; payment or honoraria for lectures, presentations, speakers bureaus, manuscript writing or educational events from Teva, Eli Lilly, and Lundbeck; leadership or fiduciary roles in board, society, committee or advocacy groups, paid or unpaid with HIS as a Member of the Educational Committee and as a Trustee of the IHS Board; outside the submitted work. T Bärnighausen reports grants or contracts from the National Institutes of Health, Alexander von Humboldt Foundation, German National Research Foundation (DFG), the European Union, German Ministry of Education and Research, German Ministry of the Environment, Wellcome, and KfW; payment or honoraria for lectures, presentations, speakers bureaus, manuscript writing or educational events from PLOS; participation on a Data Safety Monitoring Board or Advisory Board for NIH-funded research projects in Africa on Climate Change and Health; Stock or stock options in CHEERS (an SME focusing on approaches to measure climate change and health-related variables in population cohorts); outside the submitted work. M L Bell reports grants or contracts from the US EPA, NIH, High Tide Foundation, Health Effects Institute, Yale Women Faculty Forum, Environmental Defense Fund, Wellcome Trust Foundation, Yale Climate Change and Health Center, Robert Wood Johnson Foundation, and the Hutchinson Postdoctoral Fellowship; Consulting fees from Clinique, ToxiMap, and SciQuest; honoraria for lectures, presentations, speakers bureaus from the Colorado School of Public Health, Duke University, Univ. of Texas, Data4Justice, Korea University, Organization of Teratology Information Specialists, UPenn, Boston University, Honorarium for editorial duties from IOP Publishing, honorarium for grant review from NIH, Health Canada, EHS, PAC-10, UKRI, AXA, Research Fund Fellowship, University of Texas, honorarium for external advisory committees from Harvard University and the University of Montana, Honorarium for online survey/workshop from SciQuest, and payment for teaching/researching for Korea University; Support for attending meetings and/or travel from Colorado School of Public Health, University of Texas, Duke University, Boston University, UPenn, Harvard University, American Journal of Public Health, Columbia Univ., Harvard, CMAS conference, Nature Conference; Leadership or fiduciary role in other board, society, committee or advisory group unpaid with the Fifth National Climate Assessment, Lancet Countdown, Johns Hopkins EHE Advisory Board, Harvard external advisory committee for training grants, WHO Global Air Pollution and Health Technical Advisory Group, National Academies Panels and Committees, and paid with the US EPA Clean Air Scientific Advisory Committee (CASAC); all outside the submitted work. A Beloukas reports grants or contracts from Gilead and GSK; Payment or honoraria for lectures, presentations, speakers bureaus, manuscript writing or educational events from Gilead and GSK; Support for attending meetings and/or travel from Gilead and GSK; and Receipt of equipment, materials, drugs, medical writing, gifts or other services from Cepheid for a research project; outside the submitted work. S Bhaskar reports grants or contracts from the Japan Society for the Promotion of Science (JSPS), Japanese Ministry of Education, Culture, Sports, Science and Technology (MEXT) and from The Australian Academy of Science; leadership or fiduciary roles in board, society, committee or advocacy groups, paid or unpaid as the visiting director in the department of neurology at the National Cerebral and Cardiovascular Center, Suita (Osaka, Japan), district chair of diversity, equity and inclusion at the Rotary District 9675, chair and manager of the Global Health and Migration Hub Community (Berlin, Germany), an editorial member of PLOS One, BMC Neurology, Frontiers in Neurology, Frontiers in Stroke, Frontiers in Aging, Frontiers in Public Health & BMC Medical Research Methodology, a member of the College of Reviewers (Canadian Institutes of Health Research, Government of Canada), a member of the scientific review committee at Cardiff University Biobank (Cardiff, UK), an export advisor and reviewer with the Cariplo Foundation (Milan, Italy), Pandemic Health System Resilience Program (REPROGRAM) Consortium as the global chair; outside the submitted work. E J Boyko reports payment or honoraria for lectures, presentations, speakers bureaus, manuscript writing or educational events from the Korean Diabetes Association, the Diabetes Association of the ROC (Taiwan), the American Diabetes Association, and the International Society for the Diabetic Foot; Support for attending meetings and/or travel from the Korean Diabetes Association, the Diabetes Association of the ROC (Taiwan), and the International Society for the Diabetic Foot; outside the submitted work. S Cortese reports grants or contracts NIHR; payment or honoraria for lectures, presentations, speakers bureaus, manuscript writing or educational events from the ACAMH, BAP, and Medice; Support for attending meetings and/or travel from Medice; Leadership or fiduciary role in other board, society, committee or advocacy group, paid or unpaid with Eunethydis; outside the submitted work. S Das reports leadership or fiduciary role in other board, society, committee or advocacy group, paid or unpaid as the Program Chair of Association for Diagnostic and Laboratory Medicine India Section (Voluntary Role) and as a Member of Women in Global Health; outside the submitted work. L Degenhardt reports grants or contracts from Indivior; outside the submitted work. I Filip reports other financial or non-financial interests with Avicenna Medical and Clinical Institute; outside the submitted work. A Guha reports grants or contracts from the American Heart Association and the Department of Defense; Consulting feeds from Pfizer and Novartis; leadership or fiduciary role in other board, society, committee or advocacy group, paid or unpaid on the health equity task force of ZERO Prostate Cancer; outside the submitted work. V-A Lioutas reports grants or contracts from the NIH and the Alzheimer's Association; Consulting feeds from QMetis and Mindray; Support for attending meetings and/or travel from the World Stroke Organization; outside the submitted work. R Liu reports grants or contracts from National Institute of Mental Health grant #s: R01 MH115905, RF1 MH120830, R01 MH124899, R21 MH130767 (awarded to Massachusetts General Hospital); Consulting fees from Relmada Therapeutics; payment or honoraria for lectures, presentations, speakers bureaus, manuscript writing or educational events from Miami International Child and Adolescent Mental Health Conference, Massachusetts General Hospital, University of California (San Francisco); Support for attending meetings and/or travel from the American Foundation for Suicide Prevention; Participation on a Data Safety Monitoring Board or Advisory Board for the University of Pennsylvania (Chair for DSMB), University of Minnesota, and Massachusetts General Hospital; outside the submitted work. R V Martin reports support for the present manuscript from a NASA Grant; Grants or contracts from the Clean Air Fund; outside the submitted work. S A Meo reports grants or contracts from the Deputyship for Research and Innovation, Ministry of Education in Saudi Arabia (FKSUOR3-4-8); outside the submitted work. S K Panda reports support for the present manuscript from Siksha 'O’ Anusandhan (Deemed to be University); Grants or contracts from DST-GOVT OF ODISHA (Letter number 3444/ST); payment or honoraria for lectures, presentations, speakers bureaus, manuscript writing or educational events from Utkal University; outside the submitted work. M Pigeolet reports grants or contracts from The Belgian Kids’ Fund for Pediatric Research; outside the submitted work. A Radfar reports other financial or non-financial interests in Avicenna Medical and Clinical Research Institute; outside the submitted work. A Rane reports stock or stock options in Agios Pharmaceuticals; outside the submitted work. N Scarmeas reports grants or contracts with Novo Nordisc; Participation on a Data Safety Monitoring Board or Advisory Board with the Multicultural Health Diet to Reduce Cognitive Decline & AD Risk, Primus AD, Albert Einstein College of Medicine (NIH funded study) as the chair of the data safety monitoring board, and on the data safety monitoring board of the Public Private funded Phase II Study in Germany; outside the submitted work. A Sharifan reports leadership or fiduciary role in other board, society, committee or advocacy group, unpaid as a steering member of Cochrane; Reciept of equipment, materials, drugs, medical writing, gifts or other services from Elsevier and Cochrane; outside the submitted work. V Shivarov reports patents planned, issued, or pending with the Bulgarian Patent Office; Stock or stock options in ICON Plc; and financial interests in Icon Plc (salary); outside the submitted work. S J Tromans reports grants or grant contracts from the 2023 Adult Psychiatric Morbidity Survey team, collecting epidemiological data on community-based adults living in England (this is a contracted study from NHS Digital, via the Department of Health and Social Care); leadership or fiduciary role in other board, society, committee or advocacy group, unpaid as the Academic Secretary for the Neurodevelopmental Psychiatry Special Interest Group at the Royal College of Psychiatrists; Editorial Board Member for BMC Psychiatry, Advances in Autism, Advances in Mental Health and Intellectual Disability, and Progress in Neurology and Psychiatry; outside the submitted work. M Zielińska reports other financial interests in AstraZeneca as an employee; outside the submitted work. All other authors declare no competing interests.
