
JOURNAL INFORMATION
==============================
NLM Title Abbreviation: Lancet
Journal ID: Lancet
NLM Journal ID: 2985213R

Lancet (London, England)

ISSN: 0140-6736
EISSN: 1474-547X

ARTICLE INFORMATION
==============================
PMCID: PMC11830965
PMID: 39863369
DOI: 10.1016/S0140-6736(25)00105-9
Article ID: S0140-6736(25)00105-9
Article version: 1
Subjects: Corrections

Department of Error

Print publication date: 2025 Jan 25
Volume: 405
Issue: 10475
First page: 302
Last page: 302
Copyright: © 2025 The Author(s). Published by Elsevier Ltd.
Copyright year: 2025
Copyright holder: Elsevier Ltd
License: This is an open access article under the CC BY license (http://creativecommons.org/licenses/by/4.0/).
License URL: https://creativecommons.org/licenses/by/4.0/

Erratum for: The burden of diseases, injuries, and risk factors by state in the USA, 1990–2021: a systematic analysis for the Global Burden of Disease Study 2021 404 10469 7 12 2024 2314 2340 Lancet (London, England) 10.1016/S0140-6736(24)01446-6 PMC11694014 39645376

==============================
GBD 2021 US Burden of Disease Collaborators. The burden of diseases, injuries, and risk factors by state in the USA, 1990–2021: a systematic analysis for the Global Burden of Disease Study 2021. Lancet 2024; 404: 2314–40—In this Article, the x-axis labels for panels A and B in figure 1 should have run from 1980 to 2021 and S A Meo's declaration of interest should have read “S A Meo reports grants or contracts from the Deputyship for Research and Innovation, Ministry of Education in Saudi Arabia (IFKSUOR3-4-8); outside the submitted work.” Additionally, appendix 2 has been updated. These corrections have been made to the online versions as of January 23, 2025.

