# Supplementary material for: The burden of diseases, injuries, and risk factors by state in the USA, 1990–2021: a systematic analysis for the Global Burden of Disease Study 2021
Source: Lancet. 2024 Dec 7;404(10469):2314–40. doi: 10.1016/S0140-6736(24)01446-6 (PMC11694014; doi:10.1016/S0140-6736(24)01446-6)
Supplement: Supplementary appendix 1 [file mmc1.pdf]

THE LANCET

Supplementary appendix 1

This appendix formed part of the original submission and has been peer reviewed. We post it as supplied by the authors.

Supplement to: GBD 2021 US Burden of Disease Collaborators. The burden of diseases, injuries, and risk factors by state in the USA, 1990–2021: a systematic analysis for the Global Burden of Disease Study 2021. *Lancet* 2024; **404:** 2334–40.

Appendix 1: supplementary results for “The burden of diseases, injuries, and risk factors by state in the USA, 1990–2021: a systematic analysis for the Global Burden of Disease Study 2021”

This appendix provides supplementary tables and figures for “The burden of diseases, injuries, and risk factors by state in the USA, 1990–2021: a systematic analysis for the Global Burden of Disease Study 2021.”

Table of contents

Supplementary figures

Figure S1: Leading 25 Level 3 causes of age-standardised death rates in 1990, 2010, and 2021 in the USA for all ages and sexes combined 4

Figure S2: Annualised rate of change in age-standardised death rate, 1990–2021, for the leading ten Level 3 causes of death for the USA and by US state and Washington, DC 5

Figure S3: Leading 25 Level 3 causes of age-standardised YLD rates in 1990, 2010, and 2021 in the USA for all ages and sexes combined 6

Figure S4: Rank of Level 2 causes of age-standardised DALY rates in 1990, 2010, and 2021 in the USA for all ages and sexes combined 7

Figure S5: Annualised rate of change in age-standardised DALY rate, 1990–2021, for the leading ten Level 3 causes of DALYs for the USA and by US state and Washington, DC, males 8

Figure S6: Annualised rate of change in age-standardised DALY rate, 1990–2021, for the leading ten Level 3 causes of DALYs for the USA and by US state and Washington, DC, females 9

Figure S7: Age-standardised DALY rates by US state and Washington, DC, 1990–2021 10

Figure S8: Rank of Level 2 risk factors for age-standardised risk-attributable DALYs in 1990, 2010, and 2021 in the USA for all sexes combined 11

Figure S9: Percentage change in the number of deaths attributable to Level 4 risk factors in the USA from 1990 to 2021, due to the following drivers: population growth, populating ageing, changes in risk factor exposure, and changes in risk-deleted death rates 12

Figure S10: Sex difference in age-standardised risk-attributable death rates for Level 3 risks by US state and Washington, DC, 2021 13

Figure S11: Sex difference in age-standardised risk-attributable DALY rates for Level 3 risks by US state and Washington, DC, 2021 14

Supplementary tables

Table S1A: Life expectancy (LE) and healthy life expectancy (HALE) in the USA and by US state and Washington, DC for males, 1990, 2010, 2019, and 2021 15

Table S1B: Life expectancy (LE) and healthy life expectancy (HALE) in the USA and by US state and Washington, DC for females, 1990, 2010, 2019, and 2021 17

Table S2: Cause-specific YLLs for 25 leading Level 3 causes by US state and Washington, DC in 2021 and percentage change between 1990 and 2021 19

Table S3: Cause-specific YLDs for the 25 leading Level 3 causes by US state and Washington, DC in 2021 and percentage change between 1990 and 2021 70

Table S4: Cause-specific DALYs for the 25 leading Level 3 causes by US state and Washington, DC in 2021 and percentage change between 1990 and 2021 121

Table S5: Risk-specific age-standardised SEVs for the USA and by US state and Washington, DC in 1990, 2000, 2010, 2021, and annualised rate of change for 1990–2021, 2000–2021, and 2010–2021 172

Table S6: Age-standardised risk-attributable DALY rates per 100 000 in the USA and by US state and Washington, DC for all risk–outcome pairs combined and excluding one- and two-star pairs in 1990, 2010, and 2021 276

Table S7: GATHER checklist 278

Figure S1: Leading 25 Level 3 causes of age-standardised death rates in 1990, 2010, and 2021 in the USA for all ages and sexes combined

1990 Leading causes	Age-standardised rate (per 100,000)	2010 Leading causes	Age-standardised rate (per 100,000)	2021 Leading causes	Age-standardised rate (per 100,000)
1 Ischaemic heart disease	179.8 (162.1 to 188.4)	1 Ischaemic heart disease	94.5 (83.3 to 100.0)	1 COVID-19	86.1 (84.5 to 88.1)
2 Tracheal, bronchus, and lung cancer	49.8 (47.6 to 51.3)	2 Tracheal, bronchus, and lung cancer	38.8 (36.0 to 40.3)	2 Ischaemic heart disease	78.9 (69.9 to 83.9)
3 Stroke	44.1 (39.2 to 46.7)	3 Chronic obstructive pulmonary disease	33.1 (29.4 to 34.9)	3 Chronic obstructive pulmonary disease	31.3 (27.5 to 33.1)
4 Alzheimer's disease and other dementias	29.6 (7.7 to 76.1)	4 Stroke	31.2 (27.0 to 33.3)	4 Stroke	30.3 (26.0 to 32.5)
5 Chronic obstructive pulmonary disease	26.5 (24.5 to 27.5)	5 Alzheimer's disease and other dementias	29.4 (7.8 to 74.7)	5 Alzheimer's disease and other dementias	29.2 (7.7 to 73.5)
6 Lower respiratory infections	21.6 (19.2 to 22.9)	6 Chronic kidney disease	15.8 (13.9 to 16.7)	6 Tracheal, bronchus, and lung cancer	28.9 (26.7 to 30.4)
7 Colon and rectum cancer	20.6 (18.9 to 21.5)	7 Diabetes mellitus	15.0 (13.8 to 15.8)	7 Chronic kidney disease	21.9 (19.3 to 23.5)
8 Road injuries	18.4 (18.2 to 18.7)	8 Colon and rectum cancer	14.6 (13.4 to 15.3)	8 Drug use disorders	19.5 (17.7 to 21.6)
9 Breast cancer	15.9 (14.9 to 16.5)	9 Lower respiratory infections	12.5 (10.9 to 13.3)	9 Self-harm	13.0 (12.6 to 13.4)
10 Diabetes mellitus	15.2 (14.1 to 15.9)	10 Cirrhosis and other chronic liver diseases	12.2 (11.8 to 12.5)	10 Colon and rectum cancer	12.8 (11.7 to 13.5)
11 Self-harm	11.8 (11.6 to 12.0)	11 Self-harm	11.7 (11.5 to 11.9)	11 Cirrhosis and other chronic liver diseases	12.8 (12.2 to 13.2)
12 Cirrhosis and other chronic liver diseases	11.7 (11.4 to 11.9)	12 Road injuries	11.4 (11.1 to 11.5)	12 Diabetes mellitus	12.6 (11.6 to 13.3)
13 Prostate cancer	10.9 (10.2 to 11.2)	13 Breast cancer	10.8 (9.9 to 11.3)	13 Hypertensive heart disease	11.4 (9.9 to 12.6)
14 HIV/AIDS	9.8 (9.7 to 9.8)	14 Pancreatic cancer	9.4 (8.7 to 9.8)	14 Road injuries	10.8 (10.4 to 11.2)
15 Interpersonal violence	9.7 (9.6 to 9.8)	15 Drug use disorders	9.4 (9.1 to 9.8)	15 Other COVID-19 pandemic-related outcomes	10.6 (7.2 to 14.5)
16 Cardiomyopathy and myocarditis	9.5 (9.0 to 9.9)	16 Hypertensive heart disease	9.3 (8.3 to 9.8)	16 Pancreatic cancer	9.5 (8.7 to 9.9)
17 Neonatal disorders	9.5 (9.3 to 9.7)	17 Prostate cancer	7.4 (6.8 to 7.8)	17 Breast cancer	9.4 (8.5 to 9.9)
18 Pancreatic cancer	8.9 (8.4 to 9.3)	18 Falls	6.9 (6.0 to 7.4)	18 Lower respiratory infections	8.9 (7.7 to 9.6)
19 Chronic kidney disease	8.5 (7.7 to 8.9)	19 Neonatal disorders	6.7 (6.6 to 6.9)	19 Falls	8.1 (7.0 to 8.6)
20 Hypertensive heart disease	7.4 (6.7 to 7.8)	20 Cardiomyopathy and myocarditis	6.5 (6.0 to 6.8)	20 Endocrine, metabolic, blood, and immune disorders	7.5 (6.9 to 7.9)
21 Non-Hodgkin lymphoma	7.1 (6.7 to 7.4)	21 Endocrine, metabolic, blood, and immune disorders	6.2 (5.8 to 6.5)	21 Prostate cancer	6.9 (6.2 to 7.3)
22 Leukaemia	7.0 (6.6 to 7.2)	22 Leukaemia	6.1 (5.6 to 6.3)	22 Interpersonal violence	5.9 (5.7 to 6.0)
23 Congenital birth defects	6.1 (6.0 to 6.3)	23 Interpersonal violence	5.7 (5.6 to 5.8)	23 Parkinson's disease	5.8 (5.0 to 6.2)
24 Stomach cancer	5.4 (5.0 to 5.5)	24 Non-Hodgkin lymphoma	5.5 (5.1 to 5.8)	24 Neonatal disorders	5.5 (4.9 to 6.1)
25 Aortic aneurysm	5.2 (4.8 to 5.4)	25 Parkinson's disease	5.1 (4.4 to 5.4)	25 Atrial fibrillation and flutter	5.3 (4.4 to 5.7)
29 Falls	4.3 (3.8 to 4.5)	26 Atrial fibrillation and flutter	4.8 (4.0 to 5.2)	26 Cardiomyopathy and myocarditis	5.2 (4.8 to 5.6)
30 Atrial fibrillation and flutter	3.9 (3.3 to 4.2)	28 Congenital birth defects	4.3 (4.2 to 4.3)	27 Leukaemia	5.2 (4.7 to 5.4)
31 Parkinson's disease	3.8 (3.4 to 4.0)	37 Stomach cancer	3.3 (3.1 to 3.5)	28 Non-Hodgkin lymphoma	4.7 (4.2 to 4.9)
37 Endocrine, metabolic, blood, and immune disorders	3.0 (2.9 to 3.1)	41 HIV/AIDS	2.5 (2.5 to 2.5)	35 Congenital birth defects	3.6 (3.3 to 3.9)
50 Drug use disorders	2.0 (1.9 to 2.1)	42 Aortic aneurysm	2.5 (2.2 to 2.6)	42 Stomach cancer	2.8 (2.6 to 3.0)
-- COVID-19	--	-- COVID-19	--	44 Aortic aneurysm	2.1 (1.9 to 2.2)
-- Other COVID-19 pandemic-related outcomes	--	-- Other COVID-19 pandemic-related outcomes	--	56 HIV/AIDS	1.5 (1.5 to 1.5)

Legend:

Communicable, maternal, neonatal and nutritional

Non-communicable

Injuries

Other COVID Outcomes

Figure S2: Annualised rate of change in age-standardised death rate, 1990–2021, for the leading ten Level 3 causes of death for the USA and by US state and Washington, DC

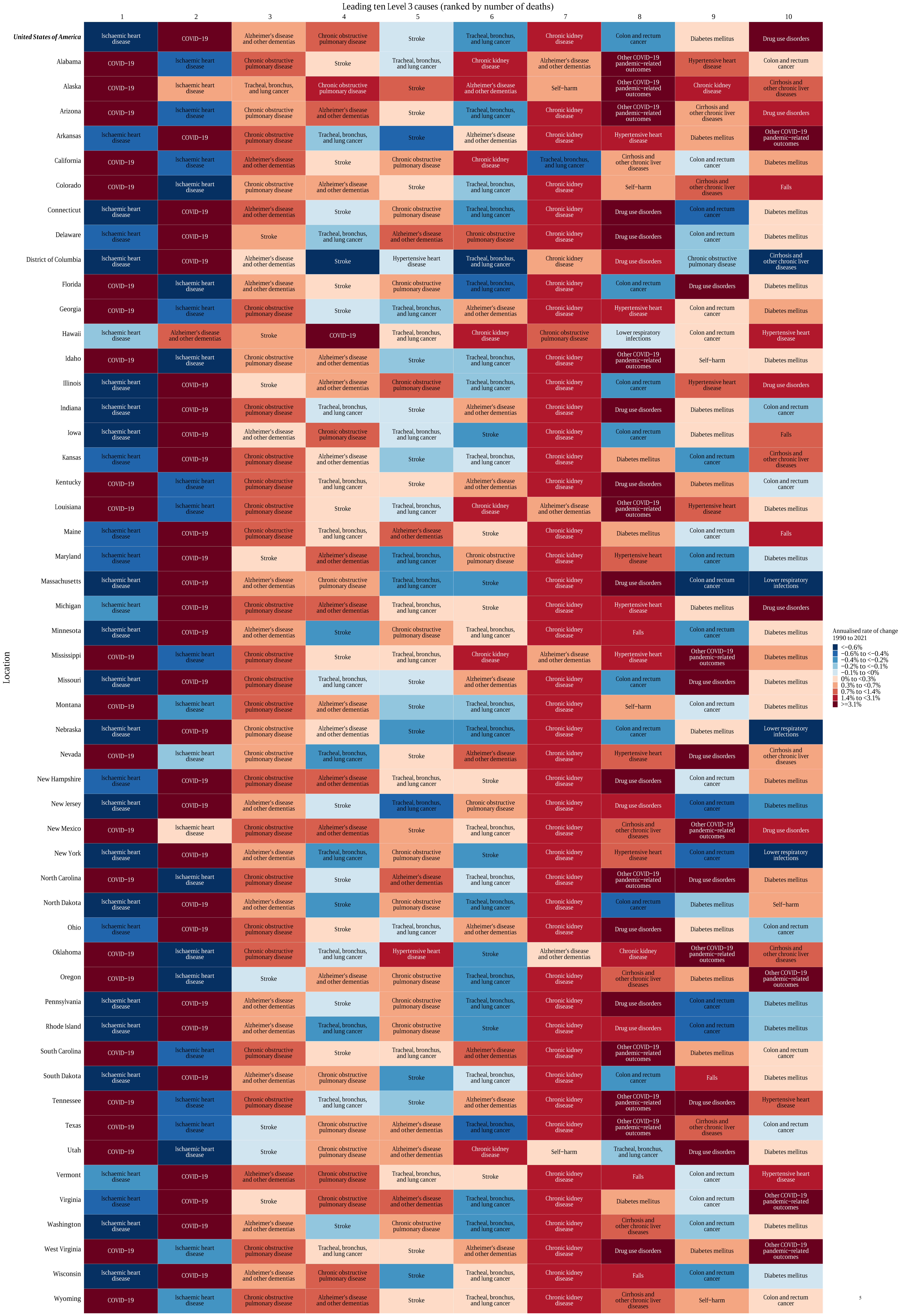

Figure S3: Leading 25 Level 3 causes of age-standardised YLD rates in 1990, 2010, and 2021 in the USA for all ages and sexes combined

1990 Leading causes	Age-standardised rate (per 100,000)	2010 Leading causes	Age-standardised rate (per 100,000)	2021 Leading causes	Age-standardised rate (per 100,000)
1 Low back pain	1279·7 (919·1 to 1708·3)	1 Low back pain	1200·1 (867·8 to 1563·8)	1 Low back pain	1179·1 (857·0 to 1536·4)
2 Headache disorders	707·1 (138·7 to 1500·9)	2 Other musculoskeletal disorders	861·5 (610·7 to 1155·9)	2 Depressive disorders	1010·7 (709·1 to 1360·1)
3 Depressive disorders	647·8 (451·6 to 875·0)	3 Depressive disorders	839·3 (591·9 to 1145·1)	3 Drug use disorders	972·2 (681·2 to 1264·1)
4 Other musculoskeletal disorders	635·8 (443·7 to 872·5)	4 Anxiety disorders	675·6 (469·0 to 915·8)	4 Other musculoskeletal disorders	936·5 (666·6 to 1251·2)
5 Anxiety disorders	611·1 (421·8 to 827·8)	5 Headache disorders	670·9 (138·0 to 1421·3)	5 Anxiety disorders	804·0 (555·3 to 1094·0)
6 Age-related and other hearing loss	443·1 (309·8 to 615·1)	6 Diabetes mellitus	496·0 (348·7 to 680·1)	6 Headache disorders	685·0 (136·7 to 1448·6)
7 Asthma	384·6 (246·7 to 573·9)	7 Drug use disorders	433·5 (310·4 to 553·6)	7 Diabetes mellitus	662·1 (467·0 to 891·9)
8 Falls	323·0 (220·9 to 443·4)	8 Age-related and other hearing loss	430·5 (299·3 to 593·4)	8 Age-related and other hearing loss	418·2 (293·3 to 580·8)
9 Osteoarthritis	295·3 (141·5 to 594·2)	9 Asthma	367·0 (240·6 to 534·6)	9 Asthma	397·6 (259·3 to 594·3)
10 Oral disorders	290·2 (172·0 to 435·8)	10 Falls	294·2 (202·3 to 405·8)	10 Falls	319·1 (221·4 to 441·4)
11 Gynecological diseases	283·2 (198·3 to 396·5)	11 Osteoarthritis	293·1 (142·2 to 586·5)	11 Osteoarthritis	310·8 (149·7 to 627·3)
12 Alcohol use disorders	277·0 (186·7 to 391·8)	12 Chronic obstructive pulmonary disease	243·6 (216·6 to 266·1)	12 Oral disorders	276·2 (162·7 to 421·7)
13 Diabetes mellitus	272·1 (191·0 to 375·6)	13 Schizophrenia	237·4 (177·5 to 301·6)	13 Gynecological diseases	239·9 (164·7 to 339·3)
14 Road injuries	271·8 (195·9 to 368·9)	14 Gynecological diseases	234·5 (162·8 to 327·0)	14 Neck pain	236·5 (157·6 to 342·5)
15 Drug use disorders	250·9 (173·3 to 328·2)	15 Alcohol use disorders	226·8 (154·2 to 314·7)	15 Chronic obstructive pulmonary disease	226·5 (196·0 to 257·8)
16 Neck pain	239·2 (159·4 to 345·6)	16 Oral disorders	212·6 (128·1 to 319·9)	16 Schizophrenia	221·4 (164·6 to 281·5)
17 Schizophrenia	235·0 (173·7 to 299·9)	17 Neonatal disorders	212·2 (152·6 to 278·1)	17 Alcohol use disorders	214·5 (146·6 to 298·1)
18 Chronic obstructive pulmonary disease	216·5 (181·6 to 251·9)	18 Neck pain	210·9 (140·7 to 304·1)	18 Autism spectrum disorders	203·3 (141·0 to 283·7)
19 Neonatal disorders	209·6 (152·9 to 274·5)	19 Autism spectrum disorders	202·5 (141·1 to 284·5)	19 Neonatal disorders	199·4 (142·7 to 257·8)
20 Autism spectrum disorders	199·8 (137·8 to 278·9)	20 Road injuries	189·0 (135·8 to 255·7)	20 Dermatitis	174·9 (101·3 to 279·9)
21 Dermatitis	192·4 (114·4 to 306·4)	21 Stroke	179·1 (130·9 to 225·7)	21 Stroke	166·9 (121·0 to 211·9)
22 Stroke	173·3 (123·2 to 222·4)	22 Dermatitis	178·5 (103·1 to 286·6)	22 Road injuries	165·3 (118·7 to 222·2)
23 Alzheimer's disease and other dementias	166·1 (113·1 to 220·8)	23 Alzheimer's disease and other dementias	159·5 (109·5 to 210·8)	23 Alzheimer's disease and other dementias	154·1 (106·3 to 203·7)
24 Blindness and vision loss	133·4 (84·6 to 202·5)	24 Other mental disorders	131·8 (84·4 to 194·3)	24 Blindness and vision loss	135·0 (84·3 to 208·1)
25 Bipolar disorder	132·3 (87·9 to 187·8)	25 Blindness and vision loss	131·2 (82·3 to 201·1)	25 COVID-19	133·9 (49·6 to 307·8)
27 Other mental disorders	131·5 (84·1 to 193·2)	26 Bipolar disorder	130·5 (86·1 to 185·5)	26 Other mental disorders	130·8 (84·1 to 192·7)
-- COVID-19	--	-- COVID-19	--	27 Bipolar disorder	129·2 (86·1 to 182·9)

Legend:

Communicable, maternal, neonatal and nutritional

Non-communicable

Injuries

Other COVID Outcomes

Figure S4: Rank of Level 2 causes of age-standardised DALY rates in 1990, 2010, and 2021 in the USA for all ages and sexes combined

1990 Leading causes	Age-standardised rate (per 100,000)	2010 Leading causes	Age-standardised rate (per 100,000)	2021 Leading causes	Age-standardised rate (per 100,000)
1 Cardiovascular diseases	5394·4 (5067·1 to 5598·9)	1 Neoplasms	3450·4 (3291·4 to 3567·2)	1 Cardiovascular diseases	3184·6 (2945·5 to 3362·6)
2 Neoplasms	4440·9 (4287·8 to 4550·8)	2 Cardiovascular diseases	3446·0 (3202·8 to 3624·8)	2 Neoplasms	2964·5 (2812·6 to 3077·9)
3 Musculoskeletal disorders	2569·5 (1891·8 to 3447·4)	3 Musculoskeletal disorders	2703·4 (1985·3 to 3536·9)	3 Musculoskeletal disorders	2791·8 (2052·3 to 3646·6)
4 Mental disorders	2170·4 (1634·0 to 2746·5)	4 Mental disorders	2435·6 (1826·8 to 3089·9)	4 Mental disorders	2712·3 (2008·2 to 3467·6)
5 Other non-communicable diseases	1707·5 (1480·4 to 2017·5)	5 Neurological disorders	1554·2 (879·4 to 2476·8)	5 Respiratory infections and tuberculosis	2703·0 (2583·0 to 2894·3)
6 Neurological disorders	1559·1 (857·8 to 2524·0)	6 Chronic respiratory diseases	1349·9 (1204·6 to 1544·9)	6 Substance use disorders	2311·2 (1958·7 to 2672·0)
7 Transport injuries	1371·2 (1288·9 to 1472·6)	7 Other non-communicable diseases	1347·0 (1160·2 to 1608·0)	7 Neurological disorders	1573·1 (896·7 to 2501·8)
8 Chronic respiratory diseases	1255·0 (1099·9 to 1463·5)	8 Diabetes and kidney diseases	1260·2 (1085·8 to 1467·6)	8 Diabetes and kidney diseases	1504·5 (1273·5 to 1768·3)
9 Self-harm and interpersonal violence	1219·0 (1199·0 to 1240·8)	9 Substance use disorders	1242·9 (1052·4 to 1436·4)	9 Other non-communicable diseases	1343·7 (1116·9 to 1626·6)
10 Unintentional injuries	1190·7 (989·9 to 1435·6)	10 Self-harm and interpersonal violence	985·6 (969·3 to 1004·4)	10 Chronic respiratory diseases	1327·3 (1168·2 to 1537·7)
11 Maternal and neonatal disorders	1080·5 (1016·3 to 1151·1)	11 Unintentional injuries	943·1 (789·3 to 1133·3)	11 Self-harm and interpersonal violence	1049·7 (1017·3 to 1081·5)
12 Diabetes and kidney diseases	899·2 (796·3 to 1025·7)	12 Maternal and neonatal disorders	841·7 (780·3 to 907·8)	12 Unintentional injuries	972·1 (814·8 to 1175·8)
13 Digestive diseases	888·5 (811·4 to 988·6)	13 Transport injuries	833·6 (776·1 to 903·3)	13 Digestive diseases	820·3 (755·0 to 904·2)
14 Substance use disorders	729·7 (575·5 to 898·4)	14 Digestive diseases	821·4 (758·3 to 901·6)	14 Transport injuries	762·1 (706·5 to 829·7)
15 Skin and subcutaneous diseases	636·0 (431·2 to 890·9)	15 Skin and subcutaneous diseases	633·5 (431·2 to 882·5)	15 Maternal and neonatal disorders	715·0 (637·4 to 794·8)
16 Sense organ diseases	611·4 (431·5 to 848·3)	16 Sense organ diseases	598·0 (420·5 to 826·2)	16 Skin and subcutaneous diseases	638·7 (436·9 to 885·1)
17 Respiratory infections and tuberculosis	594·3 (537·7 to 663·2)	17 Respiratory infections and tuberculosis	381·5 (331·9 to 441·5)	17 Sense organ diseases	589·5 (415·9 to 810·5)
18 HIV/AIDS and sexually transmitted infections	544·9 (528·8 to 565·7)	18 HIV/AIDS and sexually transmitted infections	152·2 (134·0 to 176·4)	18 Other COVID-19 pandemic-related outcomes	295·3 (200·9 to 404·3)
19 Other infectious diseases	125·9 (121·0 to 132·1)	19 Nutritional deficiencies	132·2 (88·6 to 195·2)	19 Nutritional deficiencies	139·6 (101·0 to 189·2)
20 Nutritional deficiencies	77·5 (49·5 to 119·3)	20 Other infectious diseases	71·0 (66·1 to 77·2)	20 HIV/AIDS and sexually transmitted infections	107·5 (88·5 to 133·2)
21 Enteric infections	29·8 (24·1 to 36·9)	21 Enteric infections	43·0 (39·5 to 45·2)	21 Other infectious diseases	66·9 (61·7 to 72·8)
22 Neglected tropical diseases and malaria	23·8 (15·3 to 35·6)	22 Neglected tropical diseases and malaria	29·0 (18·4 to 43·9)	22 Enteric infections	31·2 (28·9 to 33·0)
-- Other COVID-19 pandemic-related outcomes	--	-- Other COVID-19 pandemic-related outcomes	--	23 Neglected tropical diseases and malaria	26·7 (18·1 to 38·4)

Legend:
Communicable, maternal, neonatal and nutritional
Non-communicable
Injuries
Other COVID Outcomes

Figure S5: Annualised rate of change in age-standardised DALY rate, 1990–2021, for the leading ten Level 3 causes of DALYs for the USA and by US state and Washington, DC, males

Leading ten Level 3 causes (ranked by number of DALYs)

Location	1	2	3	4	5	6	7	8	9	10
United States of America	COVID-19	Ischaemic heart disease	Drug use disorders	Diabetes mellitus	Chronic obstructive pulmonary disease	Low back pain	Tracheal, bronchus, and lung cancer	Stroke	Road injuries	Self-harm
Alabama	COVID-19	Ischaemic heart disease	Drug use disorders	Chronic obstructive pulmonary disease	Tracheal, bronchus, and lung cancer	Diabetes mellitus	Stroke	Road injuries	Low back pain	Chronic kidney disease
Alaska	COVID-19	Ischaemic heart disease	Drug use disorders	Self-harm	Low back pain	Diabetes mellitus	Alcohol use disorders	Tracheal, bronchus, and lung cancer	Chronic obstructive pulmonary disease	Other musculoskeletal disorders
Arizona	COVID-19	Ischaemic heart disease	Drug use disorders	Diabetes mellitus	Low back pain	Chronic obstructive pulmonary disease	Self-harm	Road injuries	Tracheal, bronchus, and lung cancer	Other musculoskeletal disorders
Arkansas	COVID-19	Ischaemic heart disease	Chronic obstructive pulmonary disease	Diabetes mellitus	Tracheal, bronchus, and lung cancer	Drug use disorders	Road injuries	Stroke	Self-harm	Low back pain
California	COVID-19	Ischaemic heart disease	Drug use disorders	Diabetes mellitus	Low back pain	Stroke	Road injuries	Chronic obstructive pulmonary disease	Other musculoskeletal disorders	Chronic kidney disease
Colorado	COVID-19	Ischaemic heart disease	Drug use disorders	Self-harm	Chronic obstructive pulmonary disease	Low back pain	Diabetes mellitus	Road injuries	Other musculoskeletal disorders	Falls
Connecticut	Drug use disorders	Ischaemic heart disease	COVID-19	Diabetes mellitus	Low back pain	Chronic obstructive pulmonary disease	Tracheal, bronchus, and lung cancer	Other musculoskeletal disorders	Stroke	Chronic kidney disease
Delaware	COVID-19	Ischaemic heart disease	Drug use disorders	Diabetes mellitus	Tracheal, bronchus, and lung cancer	Chronic obstructive pulmonary disease	Low back pain	Stroke	Road injuries	Chronic kidney disease
District of Columbia	Drug use disorders	Ischaemic heart disease	COVID-19	Interpersonal violence	Diabetes mellitus	Low back pain	Stroke	Alcohol use disorders	Tracheal, bronchus, and lung cancer	Hypertensive heart disease
Florida	COVID-19	Ischaemic heart disease	Drug use disorders	Diabetes mellitus	Chronic obstructive pulmonary disease	Low back pain	Tracheal, bronchus, and lung cancer	Road injuries	Stroke	Self-harm
Georgia	COVID-19	Ischaemic heart disease	Drug use disorders	Diabetes mellitus	Chronic obstructive pulmonary disease	Tracheal, bronchus, and lung cancer	Low back pain	Stroke	Road injuries	Self-harm
Hawaii	Ischaemic heart disease	Drug use disorders	Diabetes mellitus	COVID-19	Stroke	Low back pain	Tracheal, bronchus, and lung cancer	Self-harm	Chronic obstructive pulmonary disease	Chronic kidney disease
Idaho	COVID-19	Ischaemic heart disease	Chronic obstructive pulmonary disease	Diabetes mellitus	Drug use disorders	Self-harm	Low back pain	Road injuries	Other musculoskeletal disorders	Stroke
Illinois	Ischaemic heart disease	COVID-19	Drug use disorders	Diabetes mellitus	Low back pain	Tracheal, bronchus, and lung cancer	Chronic obstructive pulmonary disease	Stroke	Chronic kidney disease	Other musculoskeletal disorders
Indiana	COVID-19	Ischaemic heart disease	Drug use disorders	Diabetes mellitus	Chronic obstructive pulmonary disease	Tracheal, bronchus, and lung cancer	Low back pain	Stroke	Self-harm	Road injuries
Iowa	Ischaemic heart disease	COVID-19	Diabetes mellitus	Chronic obstructive pulmonary disease	Low back pain	Tracheal, bronchus, and lung cancer	Drug use disorders	Self-harm	Other musculoskeletal disorders	Stroke
Kansas	COVID-19	Ischaemic heart disease	Diabetes mellitus	Chronic obstructive pulmonary disease	Drug use disorders	Low back pain	Tracheal, bronchus, and lung cancer	Self-harm	Road injuries	Stroke
Kentucky	COVID-19	Ischaemic heart disease	Drug use disorders	Chronic obstructive pulmonary disease	Diabetes mellitus	Tracheal, bronchus, and lung cancer	Low back pain	Stroke	Road injuries	Self-harm
Louisiana	COVID-19	Ischaemic heart disease	Drug use disorders	Diabetes mellitus	Tracheal, bronchus, and lung cancer	Chronic obstructive pulmonary disease	Road injuries	Stroke	Low back pain	Interpersonal violence
Maine	Ischaemic heart disease	COVID-19	Drug use disorders	Diabetes mellitus	Chronic obstructive pulmonary disease	Tracheal, bronchus, and lung cancer	Low back pain	Self-harm	Other musculoskeletal disorders	Stroke
Maryland	Ischaemic heart disease	COVID-19	Drug use disorders	Diabetes mellitus	Self-harm	Low back pain	Stroke	Interpersonal violence	Tracheal, bronchus, and lung cancer	Chronic obstructive pulmonary disease
Massachusetts	Drug use disorders	Ischaemic heart disease	COVID-19	Diabetes mellitus	Low back pain	Chronic obstructive pulmonary disease	Tracheal, bronchus, and lung cancer	Other musculoskeletal disorders	Stroke	Chronic kidney disease
Michigan	COVID-19	Ischaemic heart disease	Drug use disorders	Diabetes mellitus	Chronic obstructive pulmonary disease	Tracheal, bronchus, and lung cancer	Low back pain	Stroke	Self-harm	Other musculoskeletal disorders
Minnesota	COVID-19	Ischaemic heart disease	Diabetes mellitus	Drug use disorders	Low back pain	Chronic obstructive pulmonary disease	Tracheal, bronchus, and lung cancer	Self-harm	Stroke	Falls
Mississippi	COVID-19	Ischaemic heart disease	Drug use disorders	Tracheal, bronchus, and lung cancer	Road injuries	Diabetes mellitus	Chronic obstructive pulmonary disease	Stroke	Chronic kidney disease	Other COVID-19 pandemic-related outcomes
Missouri	Ischaemic heart disease	COVID-19	Drug use disorders	Diabetes mellitus	Chronic obstructive pulmonary disease	Tracheal, bronchus, and lung cancer	Low back pain	Self-harm	Road injuries	Stroke
Montana	COVID-19	Ischaemic heart disease	Chronic obstructive pulmonary disease	Self-harm	Diabetes mellitus	Drug use disorders	Low back pain	Road injuries	Tracheal, bronchus, and lung cancer	Stroke
Nebraska	Ischaemic heart disease	COVID-19	Chronic obstructive pulmonary disease	Diabetes mellitus	Low back pain	Tracheal, bronchus, and lung cancer	Road injuries	Self-harm	Stroke	Drug use disorders
Nevada	COVID-19	Ischaemic heart disease	Drug use disorders	Diabetes mellitus	Chronic obstructive pulmonary disease	Self-harm	Low back pain	Tracheal, bronchus, and lung cancer	Stroke	Road injuries
New Hampshire	Drug use disorders	Ischaemic heart disease	COVID-19	Diabetes mellitus	Chronic obstructive pulmonary disease	Low back pain	Tracheal, bronchus, and lung cancer	Self-harm	Other musculoskeletal disorders	Stroke
New Jersey	COVID-19	Ischaemic heart disease	Drug use disorders	Diabetes mellitus	Low back pain	Chronic obstructive pulmonary disease	Tracheal, bronchus, and lung cancer	Other musculoskeletal disorders	Stroke	Chronic kidney disease
New Mexico	COVID-19	Ischaemic heart disease	Drug use disorders	Diabetes mellitus	Self-harm	Road injuries	Chronic obstructive pulmonary disease	Cirrhosis and other chronic liver diseases	Alcohol use disorders	Low back pain
New York	Ischaemic heart disease	COVID-19	Drug use disorders	Diabetes mellitus	Low back pain	Chronic obstructive pulmonary disease	Other musculoskeletal disorders	Tracheal, bronchus, and lung cancer	Stroke	Depressive disorders
North Carolina	COVID-19	Ischaemic heart disease	Drug use disorders	Diabetes mellitus	Tracheal, bronchus, and lung cancer	Chronic obstructive pulmonary disease	Stroke	Road injuries	Low back pain	Chronic kidney disease
North Dakota	Ischaemic heart disease	COVID-19	Diabetes mellitus	Other musculoskeletal disorders	Low back pain	Self-harm	Chronic obstructive pulmonary disease	Road injuries	Drug use disorders	Tracheal, bronchus, and lung cancer
Ohio	COVID-19	Ischaemic heart disease	Drug use disorders	Diabetes mellitus	Chronic obstructive pulmonary disease	Tracheal, bronchus, and lung cancer	Low back pain	Stroke	Self-harm	Chronic kidney disease
Oklahoma	COVID-19	Ischaemic heart disease	Drug use disorders	Chronic obstructive pulmonary disease	Diabetes mellitus	Tracheal, bronchus, and lung cancer	Hypertensive heart disease	Self-harm	Low back pain	Road injuries
Oregon	COVID-19	Ischaemic heart disease	Drug use disorders	Diabetes mellitus	Chronic obstructive pulmonary disease	Low back pain	Self-harm	Stroke	Tracheal, bronchus, and lung cancer	Other musculoskeletal disorders
Pennsylvania	COVID-19	Drug use disorders	Ischaemic heart disease	Diabetes mellitus	Low back pain	Chronic obstructive pulmonary disease	Tracheal, bronchus, and lung cancer	Stroke	Other musculoskeletal disorders	Self-harm
Rhode Island	Ischaemic heart disease	COVID-19	Drug use disorders	Diabetes mellitus	Low back pain	Chronic obstructive pulmonary disease	Tracheal, bronchus, and lung cancer	Stroke	Other musculoskeletal disorders	Falls
South Carolina	COVID-19	Ischaemic heart disease	Drug use disorders	Diabetes mellitus	Chronic obstructive pulmonary disease	Tracheal, bronchus, and lung cancer	Road injuries	Stroke	Low back pain	Chronic kidney disease
South Dakota	Ischaemic heart disease	COVID-19	Diabetes mellitus	Chronic obstructive pulmonary disease	Self-harm	Low back pain	Road injuries	Tracheal, bronchus, and lung cancer	Other musculoskeletal disorders	Stroke
Tennessee	COVID-19	Ischaemic heart disease	Drug use disorders	Diabetes mellitus	Chronic obstructive pulmonary disease	Tracheal, bronchus, and lung cancer	Stroke	Road injuries	Low back pain	Self-harm
Texas	COVID-19	Ischaemic heart disease	Diabetes mellitus	Drug use disorders	Road injuries	Low back pain	Chronic obstructive pulmonary disease	Stroke	Self-harm	Tracheal, bronchus, and lung cancer
Utah	COVID-19	Drug use disorders	Ischaemic heart disease	Self-harm	Diabetes mellitus	Low back pain	Depressive disorders	Other musculoskeletal disorders	Chronic obstructive pulmonary disease	Stroke
Vermont	Ischaemic heart disease	COVID-19	Drug use disorders	Diabetes mellitus	Chronic obstructive pulmonary disease	Tracheal, bronchus, and lung cancer	Low back pain	Self-harm	Falls	Stroke
Virginia	COVID-19	Ischaemic heart disease	Drug use disorders	Diabetes mellitus	Low back pain	Tracheal, bronchus, and lung cancer	Chronic obstructive pulmonary disease	Stroke	Self-harm	Chronic kidney disease
Washington	Ischaemic heart disease	COVID-19	Drug use disorders	Diabetes mellitus	Low back pain	Self-harm	Other musculoskeletal disorders	Chronic obstructive pulmonary disease	Tracheal, bronchus, and lung cancer	Stroke
West Virginia	COVID-19	Drug use disorders	Ischaemic heart disease	Diabetes mellitus	Chronic obstructive pulmonary disease	Tracheal, bronchus, and lung cancer	Low back pain	Stroke	Self-harm	Chronic kidney disease
Wisconsin	Ischaemic heart disease	COVID-19	Drug use disorders	Diabetes mellitus	Low back pain	Chronic obstructive pulmonary disease	Tracheal, bronchus, and lung cancer	Self-harm	Falls	Stroke
Wyoming	COVID-19	Ischaemic heart disease	Chronic obstructive pulmonary disease	Self-harm	Drug use disorders	Low back pain	Road injuries	Diabetes mellitus	Tracheal, bronchus, and lung cancer	Other musculoskeletal disorders

Annualised rate of change 1990 to 2021
- <−0.7%
- −0.7% to <−0.6%
- −0.6% to <−0.5%
- −0.5% to <−0.3%
- −0.3% to <0%
- 0% to <0.1%
- 0.1% to <0.5%
- 0.5% to <1.2%
- 1.2% to <2.4%
- >=2.4%

Figure S6: Annualised rate of change in age-standardised DALY rate, 1990–2021, for the leading ten Level 3 causes of DALYs for the USA and by US state and Washington, DC, females

Leading ten Level 3 causes (ranked by number of DALYs)

Location	1	2	3	4	5	6	7	8	9	10
United States of America	COVID-19	Ischaemic heart disease	Low back pain	Drug use disorders	Chronic obstructive pulmonary disease	Other musculoskeletal disorders	Diabetes mellitus	Depressive disorders	Alzheimer's disease and other dementias	Stroke
Alabama	COVID-19	Ischaemic heart disease	Chronic obstructive pulmonary disease	Stroke	Low back pain	Drug use disorders	Diabetes mellitus	Other musculoskeletal disorders	Depressive disorders	Tracheal, bronchus, and lung cancer
Alaska	COVID-19	Drug use disorders	Low back pain	Other musculoskeletal disorders	Depressive disorders	Diabetes mellitus	Chronic obstructive pulmonary disease	Ischaemic heart disease	Anxiety disorders	Headache disorders
Arizona	COVID-19	Low back pain	Ischaemic heart disease	Drug use disorders	Depressive disorders	Other musculoskeletal disorders	Chronic obstructive pulmonary disease	Diabetes mellitus	Alzheimer's disease and other dementias	Anxiety disorders
Arkansas	COVID-19	Ischaemic heart disease	Chronic obstructive pulmonary disease	Depressive disorders	Low back pain	Stroke	Diabetes mellitus	Tracheal, bronchus, and lung cancer	Drug use disorders	Alzheimer's disease and other dementias
California	COVID-19	Low back pain	Ischaemic heart disease	Other musculoskeletal disorders	Alzheimer's disease and other dementias	Depressive disorders	Anxiety disorders	Diabetes mellitus	Drug use disorders	Stroke
Colorado	COVID-19	Low back pain	Drug use disorders	Other musculoskeletal disorders	Depressive disorders	Chronic obstructive pulmonary disease	Ischaemic heart disease	Alzheimer's disease and other dementias	Anxiety disorders	Falls
Connecticut	Ischaemic heart disease	Low back pain	Alzheimer's disease and other dementias	COVID-19	Other musculoskeletal disorders	Drug use disorders	Chronic obstructive pulmonary disease	Diabetes mellitus	Stroke	Anxiety disorders
Delaware	COVID-19	Ischaemic heart disease	Drug use disorders	Low back pain	Chronic obstructive pulmonary disease	Other musculoskeletal disorders	Diabetes mellitus	Depressive disorders	Stroke	Alzheimer's disease and other dementias
District of Columbia	Drug use disorders	Ischaemic heart disease	COVID-19	Low back pain	Other musculoskeletal disorders	Anxiety disorders	Depressive disorders	Headache disorders	Alzheimer's disease and other dementias	Diabetes mellitus
Florida	COVID-19	Ischaemic heart disease	Low back pain	Drug use disorders	Chronic obstructive pulmonary disease	Alzheimer's disease and other dementias	Diabetes mellitus	Depressive disorders	Stroke	Other musculoskeletal disorders
Georgia	COVID-19	Ischaemic heart disease	Low back pain	Chronic obstructive pulmonary disease	Depressive disorders	Diabetes mellitus	Drug use disorders	Stroke	Other musculoskeletal disorders	Anxiety disorders
Hawaii	Alzheimer's disease and other dementias	Low back pain	Ischaemic heart disease	Stroke	Diabetes mellitus	Other musculoskeletal disorders	Depressive disorders	Anxiety disorders	Headache disorders	COVID-19
Idaho	COVID-19	Low back pain	Other musculoskeletal disorders	Depressive disorders	Chronic obstructive pulmonary disease	Drug use disorders	Ischaemic heart disease	Diabetes mellitus	Alzheimer's disease and other dementias	Anxiety disorders
Illinois	Ischaemic heart disease	COVID-19	Low back pain	Other musculoskeletal disorders	Chronic obstructive pulmonary disease	Drug use disorders	Alzheimer's disease and other dementias	Stroke	Diabetes mellitus	Depressive disorders
Indiana	COVID-19	Ischaemic heart disease	Drug use disorders	Chronic obstructive pulmonary disease	Low back pain	Diabetes mellitus	Other musculoskeletal disorders	Depressive disorders	Stroke	Alzheimer's disease and other dementias
Iowa	COVID-19	Ischaemic heart disease	Low back pain	Other musculoskeletal disorders	Chronic obstructive pulmonary disease	Alzheimer's disease and other dementias	Diabetes mellitus	Stroke	Depressive disorders	Tracheal, bronchus, and lung cancer
Kansas	COVID-19	Ischaemic heart disease	Low back pain	Chronic obstructive pulmonary disease	Other musculoskeletal disorders	Depressive disorders	Diabetes mellitus	Alzheimer's disease and other dementias	Drug use disorders	Stroke
Kentucky	COVID-19	Drug use disorders	Ischaemic heart disease	Chronic obstructive pulmonary disease	Low back pain	Diabetes mellitus	Tracheal, bronchus, and lung cancer	Stroke	Depressive disorders	Other musculoskeletal disorders
Louisiana	COVID-19	Ischaemic heart disease	Drug use disorders	Low back pain	Chronic obstructive pulmonary disease	Diabetes mellitus	Stroke	Other musculoskeletal disorders	Depressive disorders	Tracheal, bronchus, and lung cancer
Maine	Chronic obstructive pulmonary disease	Ischaemic heart disease	Low back pain	Other musculoskeletal disorders	COVID-19	Alzheimer's disease and other dementias	Diabetes mellitus	Tracheal, bronchus, and lung cancer	Drug use disorders	Stroke
Maryland	Ischaemic heart disease	COVID-19	Low back pain	Other musculoskeletal disorders	Diabetes mellitus	Stroke	Alzheimer's disease and other dementias	Chronic obstructive pulmonary disease	Depressive disorders	Anxiety disorders
Massachusetts	Drug use disorders	COVID-19	Low back pain	Other musculoskeletal disorders	Ischaemic heart disease	Alzheimer's disease and other dementias	Chronic obstructive pulmonary disease	Depressive disorders	Diabetes mellitus	Anxiety disorders
Michigan	COVID-19	Ischaemic heart disease	Drug use disorders	Low back pain	Chronic obstructive pulmonary disease	Other musculoskeletal disorders	Diabetes mellitus	Alzheimer's disease and other dementias	Stroke	Depressive disorders
Minnesota	COVID-19	Low back pain	Other musculoskeletal disorders	Alzheimer's disease and other dementias	Ischaemic heart disease	Depressive disorders	Chronic obstructive pulmonary disease	Diabetes mellitus	Falls	Stroke
Mississippi	COVID-19	Ischaemic heart disease	Chronic obstructive pulmonary disease	Stroke	Diabetes mellitus	Drug use disorders	Low back pain	Depressive disorders	Chronic kidney disease	Tracheal, bronchus, and lung cancer
Missouri	COVID-19	Ischaemic heart disease	Chronic obstructive pulmonary disease	Drug use disorders	Low back pain	Other musculoskeletal disorders	Diabetes mellitus	Stroke	Alzheimer's disease and other dementias	Tracheal, bronchus, and lung cancer
Montana	COVID-19	Chronic obstructive pulmonary disease	Ischaemic heart disease	Low back pain	Other musculoskeletal disorders	Alzheimer's disease and other dementias	Drug use disorders	Depressive disorders	Diabetes mellitus	Stroke
Nebraska	Low back pain	COVID-19	Ischaemic heart disease	Chronic obstructive pulmonary disease	Alzheimer's disease and other dementias	Other musculoskeletal disorders	Diabetes mellitus	Stroke	Depressive disorders	Anxiety disorders
Nevada	COVID-19	Drug use disorders	Ischaemic heart disease	Low back pain	Chronic obstructive pulmonary disease	Depressive disorders	Other musculoskeletal disorders	Diabetes mellitus	Stroke	Anxiety disorders
New Hampshire	Drug use disorders	Low back pain	Ischaemic heart disease	Chronic obstructive pulmonary disease	Other musculoskeletal disorders	COVID-19	Alzheimer's disease and other dementias	Depressive disorders	Diabetes mellitus	Tracheal, bronchus, and lung cancer
New Jersey	COVID-19	Ischaemic heart disease	Low back pain	Other musculoskeletal disorders	Alzheimer's disease and other dementias	Drug use disorders	Diabetes mellitus	Chronic obstructive pulmonary disease	Anxiety disorders	Depressive disorders
New Mexico	COVID-19	Drug use disorders	Ischaemic heart disease	Low back pain	Diabetes mellitus	Depressive disorders	Chronic obstructive pulmonary disease	Other musculoskeletal disorders	Alzheimer's disease and other dementias	Falls
New York	Ischaemic heart disease	COVID-19	Low back pain	Other musculoskeletal disorders	Alzheimer's disease and other dementias	Depressive disorders	Diabetes mellitus	Chronic obstructive pulmonary disease	Drug use disorders	Anxiety disorders
North Carolina	COVID-19	Ischaemic heart disease	Drug use disorders	Chronic obstructive pulmonary disease	Low back pain	Diabetes mellitus	Stroke	Alzheimer's disease and other dementias	Other musculoskeletal disorders	Depressive disorders
North Dakota	Other musculoskeletal disorders	COVID-19	Low back pain	Ischaemic heart disease	Alzheimer's disease and other dementias	Diabetes mellitus	Chronic obstructive pulmonary disease	Stroke	Depressive disorders	Anxiety disorders
Ohio	COVID-19	Ischaemic heart disease	Drug use disorders	Chronic obstructive pulmonary disease	Low back pain	Diabetes mellitus	Stroke	Other musculoskeletal disorders	Alzheimer's disease and other dementias	Depressive disorders
Oklahoma	COVID-19	Ischaemic heart disease	Drug use disorders	Chronic obstructive pulmonary disease	Low back pain	Depressive disorders	Diabetes mellitus	Other musculoskeletal disorders	Stroke	Tracheal, bronchus, and lung cancer
Oregon	COVID-19	Low back pain	Other musculoskeletal disorders	Depressive disorders	Chronic obstructive pulmonary disease	Alzheimer's disease and other dementias	Drug use disorders	Stroke	Ischaemic heart disease	Diabetes mellitus
Pennsylvania	COVID-19	Ischaemic heart disease	Drug use disorders	Low back pain	Alzheimer's disease and other dementias	Other musculoskeletal disorders	Chronic obstructive pulmonary disease	Diabetes mellitus	Stroke	Depressive disorders
Rhode Island	Ischaemic heart disease	COVID-19	Low back pain	Drug use disorders	Depressive disorders	Alzheimer's disease and other dementias	Other musculoskeletal disorders	Chronic obstructive pulmonary disease	Diabetes mellitus	Tracheal, bronchus, and lung cancer
South Carolina	COVID-19	Ischaemic heart disease	Drug use disorders	Chronic obstructive pulmonary disease	Low back pain	Diabetes mellitus	Stroke	Depressive disorders	Other musculoskeletal disorders	Alzheimer's disease and other dementias
South Dakota	COVID-19	Ischaemic heart disease	Low back pain	Other musculoskeletal disorders	Alzheimer's disease and other dementias	Chronic obstructive pulmonary disease	Diabetes mellitus	Stroke	Falls	Tracheal, bronchus, and lung cancer
Tennessee	COVID-19	Ischaemic heart disease	Drug use disorders	Chronic obstructive pulmonary disease	Low back pain	Diabetes mellitus	Stroke	Depressive disorders	Other musculoskeletal disorders	Tracheal, bronchus, and lung cancer
Texas	COVID-19	Ischaemic heart disease	Low back pain	Depressive disorders	Other musculoskeletal disorders	Diabetes mellitus	Chronic obstructive pulmonary disease	Stroke	Anxiety disorders	Drug use disorders
Utah	Depressive disorders	COVID-19	Drug use disorders	Low back pain	Other musculoskeletal disorders	Anxiety disorders	Diabetes mellitus	Ischaemic heart disease	Headache disorders	Stroke
Vermont	Ischaemic heart disease	Low back pain	Chronic obstructive pulmonary disease	Alzheimer's disease and other dementias	Depressive disorders	COVID-19	Other musculoskeletal disorders	Falls	Diabetes mellitus	Drug use disorders
Virginia	COVID-19	Ischaemic heart disease	Low back pain	Diabetes mellitus	Depressive disorders	Chronic obstructive pulmonary disease	Other musculoskeletal disorders	Stroke	Drug use disorders	Alzheimer's disease and other dementias
Washington	Other musculoskeletal disorders	Low back pain	COVID-19	Drug use disorders	Depressive disorders	Ischaemic heart disease	Alzheimer's disease and other dementias	Chronic obstructive pulmonary disease	Diabetes mellitus	Stroke
West Virginia	COVID-19	Ischaemic heart disease	Drug use disorders	Chronic obstructive pulmonary disease	Diabetes mellitus	Low back pain	Stroke	Tracheal, bronchus, and lung cancer	Depressive disorders	Alzheimer's disease and other dementias
Wisconsin	COVID-19	Ischaemic heart disease	Low back pain	Drug use disorders	Alzheimer's disease and other dementias	Chronic obstructive pulmonary disease	Falls	Other musculoskeletal disorders	Depressive disorders	Diabetes mellitus
Wyoming	COVID-19	Chronic obstructive pulmonary disease	Low back pain	Ischaemic heart disease	Drug use disorders	Other musculoskeletal disorders	Depressive disorders	Alzheimer's disease and other dementias	Stroke	Diabetes mellitus

Annualised rate of change 1990 to 2021
- <−0.8%
- −0.8% to <−0.6%
- −0.6% to <−0.3%
- −0.3% to <−0.1%
- −0.1% to <0%
- 0% to <0.3%
- 0.3% to <0.6%
- 0.6% to <0.8%
- 0.8% to <2.2%
- >=2.2%

Figure S7: Age-standardised DALY rates by US state and Washington, DC, 1990–2021

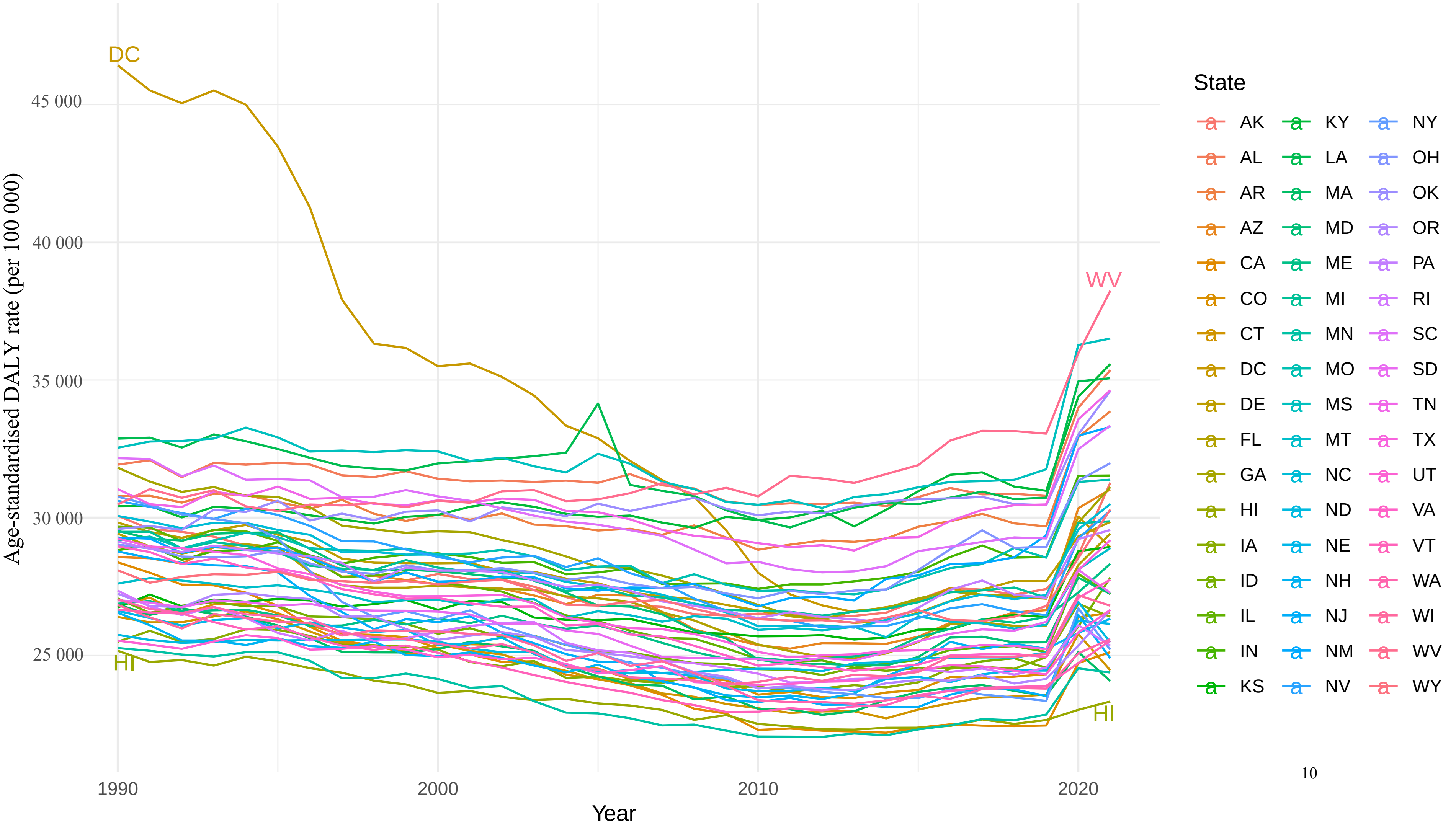

Figure S8. Rank of Level 2 risk factors for age-standardised risk-attributable DALY rates in 1990, 2010, and 2021 in the USA for all sexes combined

1990 Leading risks	Age-standardised rate (per 100,000)	2010 Leading risks	Age-standardised rate (per 100,000)	2021 Leading risks	Age-standardised rate (per 100,000)
1 Tobacco	3631·1 (2991·0 to 4267·7)	1 Tobacco	2298·9 (1828·2 to 2766·6)	1 Drug use	2245·0 (1920·8 to 2561·7)
2 High systolic blood pressure	2622·1 (2180·6 to 3023·2)	2 High body-mass index	2022·4 (888·0 to 3074·2)	2 High body-mass index	2231·9 (1014·5 to 3323·5)
3 Dietary risks	2325·6 (683·6 to 3428·5)	3 Dietary risks	1745·3 (724·0 to 2435·4)	3 High fasting plasma glucose	1930·1 (1584·6 to 2267·0)
4 High body-mass index	1787·3 (727·7 to 2908·2)	4 High fasting plasma glucose	1714·3 (1415·8 to 2031·2)	4 Tobacco	1858·9 (1447·7 to 2270·4)
5 High LDL cholesterol	1618·5 (1110·7 to 2127·1)	5 High systolic blood pressure	1425·7 (1141·7 to 1679·8)	5 Dietary risks	1631·3 (722·3 to 2269·1)
6 High fasting plasma glucose	1439·6 (1221·7 to 1671·3)	6 Drug use	1162·4 (1021·6 to 1285·8)	6 High systolic blood pressure	1437·4 (1171·1 to 1685·0)
7 High alcohol use	1125·8 (976·0 to 1298·0)	7 High alcohol use	1007·2 (878·1 to 1152·6)	7 High alcohol use	1077·7 (942·5 to 1223·3)
8 Air pollution	1030·8 (500·7 to 1604·7)	8 Child and maternal malnutrition	821·4 (747·8 to 905·0)	8 Kidney dysfunction	858·9 (766·9 to 943·8)
9 Child and maternal malnutrition	1007·5 (946·2 to 1080·7)	9 Kidney dysfunction	777·2 (682·7 to 866·6)	9 Child and maternal malnutrition	708·8 (633·9 to 791·2)
10 Kidney dysfunction	869·6 (717·5 to 1004·0)	10 High LDL cholesterol	741·3 (491·6 to 996·1)	10 High LDL cholesterol	642·0 (418·8 to 869·4)
11 Occupational risks	774·1 (655·7 to 896·6)	11 Occupational risks	623·3 (526·9 to 719·8)	11 Occupational risks	559·6 (466·2 to 659·3)
12 Drug use	600·2 (510·9 to 690·0)	12 Air pollution	461·6 (328·3 to 614·3)	12 Air pollution	272·5 (156·5 to 402·3)
13 Unsafe sex	507·7 (479·2 to 532·6)	13 Non-optimal temperature	316·7 (264·9 to 387·2)	13 Non-optimal temperature	258·5 (219·5 to 311·3)
14 Non-optimal temperature	404·8 (338·0 to 506·8)	14 Childhood sexual abuse and bullying	227·0 (124·5 to 374·5)	14 Childhood sexual abuse and bullying	226·5 (125·6 to 370·4)
15 Other environmental risks	265·0 (38·8 to 499·9)	15 Low bone mineral density	203·6 (161·3 to 253·2)	15 Low bone mineral density	221·9 (176·4 to 275·5)
16 Low bone mineral density	196·6 (154·0 to 246·1)	16 Unsafe sex	185·6 (168·4 to 207·0)	16 Other environmental risks	143·5 (22·1 to 270·2)
17 Low physical activity	149·0 (65·0 to 241·2)	17 Other environmental risks	177·1 (28·8 to 328·3)	17 Unsafe sex	142·8 (124·9 to 166·4)
18 Childhood sexual abuse and bullying	142·9 (74·3 to 244·5)	18 Low physical activity	117·0 (52·5 to 184·9)	18 Low physical activity	136·9 (57·7 to 218·3)
19 Intimate partner violence	93·0 (49·2 to 144·8)	19 Intimate partner violence	87·4 (30·5 to 154·8)	19 Intimate partner violence	90·0 (28·7 to 162·3)
20 Unsafe water, sanitation, and handwashing	10·3 (4·1 to 19·9)	20 Unsafe water, sanitation, and handwashing	7·7 (3·6 to 13·4)	20 Unsafe water, sanitation, and handwashing	4·6 (2·1 to 8·1)

Legend:

Environmental/occupational risks
Behavioural risks
Metabolic risks

Figure S9: Percentage change in the number of deaths attributable to Level 4 risk factors in the USA from 1990 to 2021, due to the following drivers: population growth, populating ageing, changes in risk factor exposure, and changes in risk-deleted death rates

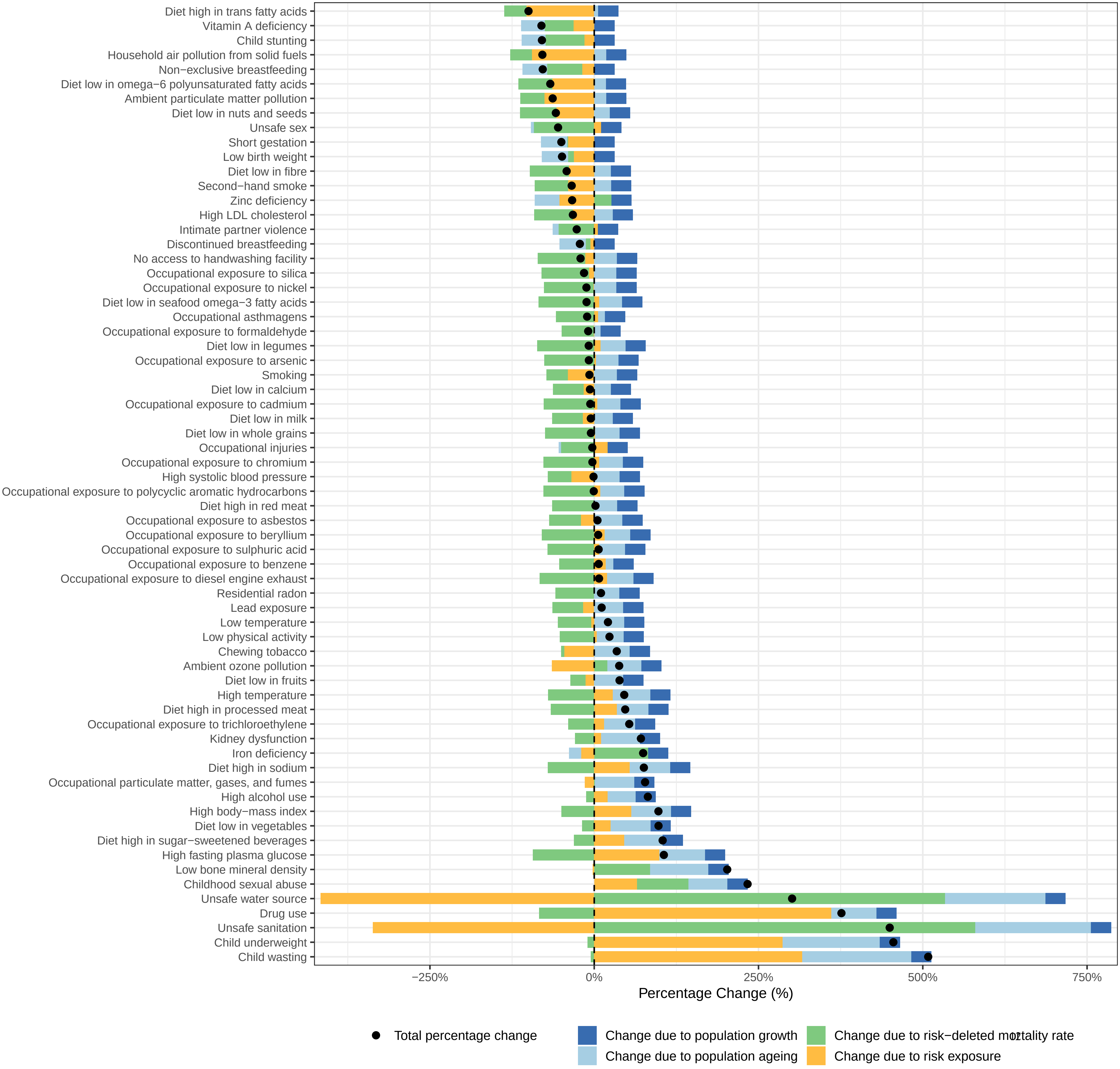

Figure S10: Sex difference in age-standardised risk-attributable death rates for Level 3 risks by US state and Washington, DC, 2021

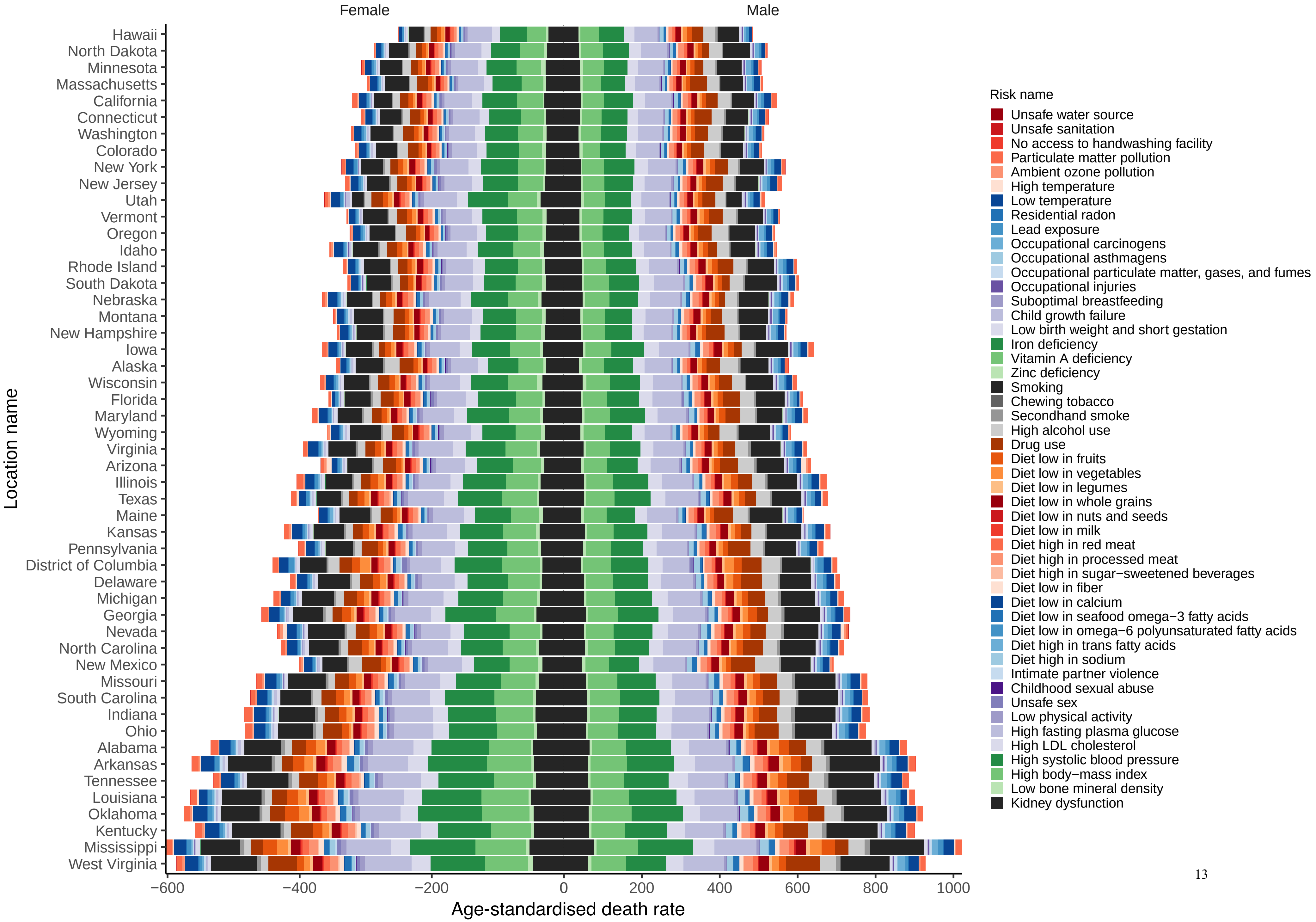

Figure S11: Sex difference in age-standardised risk-attributable DALY rates for Level 3 risks by US state and Washington, DC, 2021

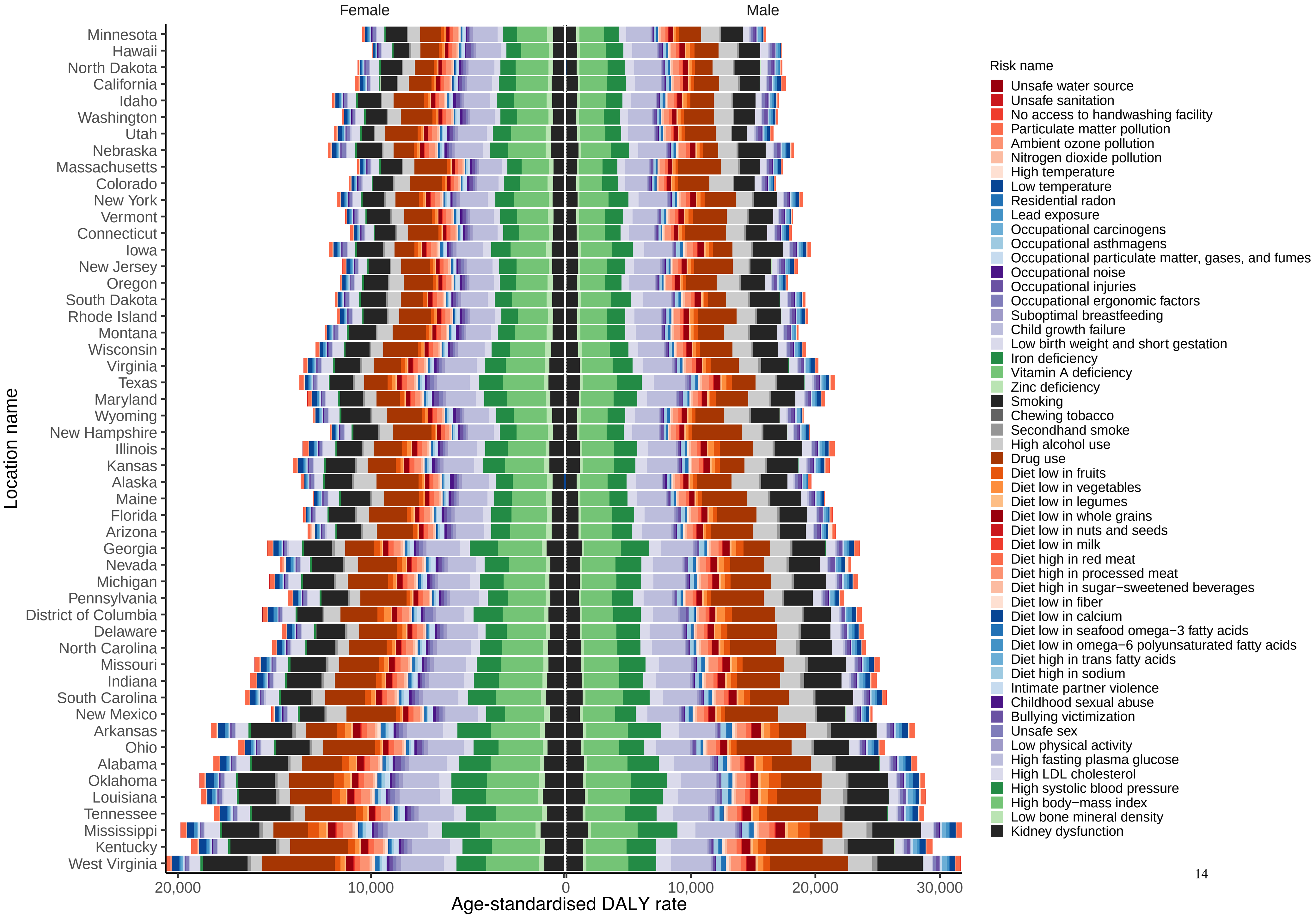

Table S1A: Life expectancy and healthy life expectancy in the USA and by US State and Washington DC for males, 1990, 2010, 2019, and 2021

	Life expectancy				Healthy life expectancy			
Location name	1990	2010	2019	2021	1990	2010	2019	2021
USA	72·1 (72·0–72·1)	76·3 (76·3–76·4)	76·6 (76·5–76·6)	74·3 (74·1–74·4)	62·8 (60·1–65·1)	65·5 (62·5–68·1)	65·1 (62·1–67·9)	63·2 (60·2–65·8)
Alabama	69·9 (69·6–70·1)	72·6 (72·4–72·9)	72·8 (72·5–73·1)	69·7 (67·6–71·8)	61·0 (58·5–63·3)	62·4 (59·6–64·8)	62·1 (59·2–64·7)	59·5 (56·4–62·6)
Alaska	72·0 (71·4–72·5)	75·7 (75·2–76·2)	76·5 (75·8–77·2)	72·9 (71·2–74·7)	62·6 (59·9–65·0)	65·3 (62·5–67·9)	65·2 (62·0–68·1)	62·3 (59·2–65·2)
Arizona	73·0 (72·7–73·3)	76·8 (76·6–77·1)	76·3 (76·0–76·6)	72·7 (70·7–74·7)	63·2 (60·3–65·6)	65·5 (62·4–68·3)	64·5 (61·3–67·3)	61·6 (58·4–64·7)
Arkansas	70·8 (70·4–71·2)	73·3 (72·9–73·6)	73·3 (72·9–73·7)	70·6 (68·5–72·8)	61·9 (59·3–64·2)	63·6 (61·0–66·0)	63·0 (60·3–65·5)	60·7 (57·7–63·7)
California	72·7 (72·6–72·8)	78·6 (78·4–78·7)	78·9 (78·8–79·0)	76·5 (74·6–78·4)	63·4 (60·8–65·7)	67·6 (64·6–70·3)	67·4 (64·2–70·2)	65·4 (62·1–68·6)
Colorado	74·2 (73·8–74·5)	78·0 (77·7–78·3)	78·2 (77·9–78·5)	76·2 (73·9–78·3)	64·4 (61·5–66·8)	67·0 (63·9–69·7)	66·4 (63·2–69·2)	64·7 (61·3–68·2)
Connecticut	74·2 (73·9–74·5)	78·1 (77·8–78·5)	78·3 (78·0–78·7)	77·7 (75·5–79·9)	64·6 (61·8–67·0)	66·8 (63·8–69·5)	66·5 (63·3–69·2)	65·8 (62·1–69·2)
Delaware	72·0 (71·5–72·5)	75·7 (75·2–76·2)	75·4 (74·8–76·0)	73·7 (71·9–75·5)	62·7 (60·0–64·9)	64·7 (61·7–67·4)	63·9 (60·7–66·6)	62·3 (58·9–65·5)
Washington, DC	61·4 (60·8–62·0)	74·6 (73·9–75·2)	75·7 (75·0–76·5)	73·9 (71·4–76·2)	53·7 (51·5–55·7)	64·4 (61·6–67·0)	64·9 (61·7–67·4)	63·3 (60·1–66·6)
Florida	72·2 (72·0–72·4)	76·6 (76·4–76·8)	76·7 (76·5–76·8)	74·0 (72·0–76·0)	62·7 (59·9–65·0)	65·4 (62·4–68·1)	64·9 (61·8–67·7)	62·6 (59·2–65·8)
Georgia	70·0 (69·8–70·2)	74·8 (74·6–75·0)	75·5 (75·3–75·7)	72·3 (70·4–74·4)	61·2 (58·7–63·4)	64·5 (61·6–66·9)	64·5 (61·5–67·1)	61·9 (58·7–64·7)
Hawaii	75·5 (75·0–76·0)	78·3 (77·8–78·8)	78·8 (78·2–79·4)	78·3 (75·9–80·8)	65·5 (62·7–67·9)	67·3 (64·2–70·0)	67·3 (64·3–70·2)	66·8 (63·4–70·0)
Idaho	73·9 (73·4–74·5)	77·3 (76·8–77·7)	77·8 (77·3–78·3)	75·0 (73·0–77·0)	64·2 (61·4–66·7)	66·4 (63·4–69·1)	66·3 (63·2–69·2)	64·0 (60·6–67·1)
Illinois	71·6 (71·4–71·8)	76·4 (76·2–76·6)	76·8 (76·6–77·0)	75·2 (73·0–77·3)	62·5 (59·9–64·7)	65·8 (62·8–68·3)	65·6 (62·5–68·3)	64·1 (60·6–67·4)
Indiana	72·2 (72·0–72·5)	74·9 (74·7–75·2)	74·7 (74·4–75·0)	72·6 (70·4–74·7)	63·0 (60·4–65·3)	64·1 (61·2–66·7)	63·5 (60·6–66·1)	61·7 (58·3–64·8)
Iowa	74·4 (74·1–74·8)	77·0 (76·7–77·4)	76·8 (76·4–77·1)	75·7 (73·7–77·8)	64·8 (62·1–67·3)	66·5 (63·5–69·1)	65·6 (62·6–68·4)	64·6 (61·1–67·7)
Kansas	73·7 (73·3–74·0)	75·9 (75·5–76·2)	76·1 (75·7–76·5)	74·1 (72·0–76·4)	64·1 (61·4–66·5)	65·3 (62·4–67·9)	65·0 (61·8–67·7)	63·3 (60·2–66·5)
Kentucky	71·0 (70·7–71·3)	73·3 (73·1–73·6)	73·3 (73·0–73·6)	70·4 (68·3–72·6)	61·8 (59·2–64·2)	62·7 (59·8–65·3)	62·0 (59·0–64·7)	59·5 (56·4–62·8)
Louisiana	69·5 (69·2–69·8)	72·9 (72·6–73·2)	73·0 (72·8–73·3)	70·1 (67·8–72·1)	60·6 (58·1–62·8)	62·5 (59·7–64·9)	62·1 (59·1–64·6)	59·6 (56·8–62·9)
Maine	73·6 (73·1–74·1)	76·7 (76·2–77·2)	76·3 (75·7–76·8)	74·7 (72·3–77·2)	64·0 (61·3–66·5)	65·7 (62·7–68·3)	64·7 (61·6–67·5)	63·4 (59·9–66·7)
Maryland	71·6 (71·3–71·9)	76·8 (76·5–77·1)	76·6 (76·3–76·9)	75·2 (72·9–77·6)	62·6 (60·0–64·9)	65·9 (62·8–68·5)	65·4 (62·4–68·0)	64·2 (60·7–67·4)
Massachusetts	73·6 (73·3–73·8)	78·0 (77·7–78·2)	78·3 (78·0–78·5)	78·1 (75·8–80·2)	63·9 (61·1–66·3)	66·5 (63·4–69·2)	66·2 (63·0–69·1)	65·8 (62·4–69·7)
Michigan	72·0 (71·8–72·2)	75·6 (75·3–75·8)	76·0 (75·8–76·2)	73·8 (71·7–76·0)	62·7 (60·1–65·0)	64·7 (61·8–67·4)	64·3 (61·3–67·1)	62·5 (59·0–65·6)
Minnesota	74·7 (74·4–75·0)	78·5 (78·2–78·7)	78·6 (78·3–78·9)	77·4 (75·3–79·5)	65·0 (62·3–67·4)	67·7 (64·8–70·4)	67·3 (64·2–70·0)	66·2 (62·9–69·6)
Mississippi	69·4 (69·0–69·7)	72·0 (71·7–72·3)	71·4 (71·1–71·8)	68·5 (66·4–70·7)	60·8 (58·3–63·0)	62·6 (59·9–64·8)	61·6 (59·0–64·1)	59·1 (56·0–62·2)
Missouri	71·9 (71·6–72·1)	74·8 (74·5–75·0)	74·5 (74·2–74·8)	72·5 (70·2–74·6)	62·7 (60·1–65·0)	64·2 (61·2–66·8)	63·4 (60·3–66·0)	61·6 (58·4–64·9)
Montana	73·7 (73·1–74·3)	76·3 (75·7–76·9)	76·8 (76·2–77·4)	74·4 (72·2–76·7)	63·9 (61·2–66·4)	65·5 (62·3–68·1)	65·4 (62·3–68·2)	63·4 (60·0–66·5)
Nebraska	73·7 (73·3–74·1)	77·3 (76·8–77·7)	77·4 (76·9–77·9)	76·1 (74·0–78·3)	64·1 (61·3–66·5)	66·6 (63·5–69·2)	66·2 (63·2–69·0)	65·0 (61·6–68·2)
Nevada	71·3 (70·9–71·7)	75·7 (75·3–76·0)	75·9 (75·6–76·3)	73·0 (71·0–75·2)	62·1 (59·4–64·3)	65·0 (62·1–67·6)	64·7 (61·7–67·4)	62·3 (59·2–65·5)
New Hampshire	73·7 (73·2–74·3)	78·0 (77·5–78·5)	77·5 (76·9–78·0)	76·6 (74·3–78·9)	64·1 (61·4–66·5)	66·6 (63·4–69·3)	65·3 (62·1–68·2)	64·5 (60·9–68·2)
New Jersey	72·4 (72·2–72·6)	77·6 (77·3–77·8)	78·4 (78·1–78·6)	77·1 (75·0–79·4)	63·1 (60·5–65·4)	66·6 (63·7–69·3)	66·5 (63·3–69·2)	65·4 (61·9–68·5)
New Mexico	72·6 (72·1–73·0)	75·7 (75·2–76·1)	74·5 (74·0–74·9)	71·5 (69·1–73·8)	62·7 (60·0–65·2)	64·7 (61·8–67·2)	63·1 (59·9–65·8)	60·6 (57·3–64·0)
New York	71·1 (70·9–71·2)	77·8 (77·7–78·0)	79·0 (78·8–79·1)	77·2 (75·2–79·3)	61·8 (59·1–64·1)	66·2 (63·0–69·0)	66·5 (63·2–69·5)	65·1 (61·7–68·4)
North Carolina	70·9 (70·7–71·1)	75·3 (75·1–75·5)	75·3 (75·0–75·5)	72·8 (70·6–75·0)	62·0 (59·5–64·2)	64·9 (62·1–67·4)	64·1 (61·2–66·8)	62·0 (58·7–65·0)
North Dakota	74·3 (73·8–74·9)	76·9 (76·4–77·4)	77·3 (76·7–77·9)	77·0 (75·2–78·9)	64·6 (61·8–67·0)	66·1 (63·1–68·8)	65·8 (62·7–68·6)	65·4 (62·0–68·7)
Ohio	72·2 (72·0–72·4)	75·2 (75·0–75·3)	74·6 (74·4–74·8)	72·4 (70·4–74·5)	62·9 (60·3–65·2)	64·4 (61·6–66·9)	63·2 (60·1–66·0)	61·3 (57·9–64·4)

Oklahoma	72·0 (71·6–72·3)	73·1 (72·8–73·4)	73·5 (73·2–73·8)	70·7 (68·6–72·8)	62·7 (60·0–64·9)	62·7 (59·9–65·3)	62·4 (59·5–65·1)	60·1 (56·7–63·0)
Oregon	73·4 (73·0–73·7)	77·4 (77·1–77·7)	77·7 (77·4–78·1)	75·6 (73·4–77·8)	64·0 (61·3–66·2)	66·7 (63·7–69·3)	66·4 (63·3–69·2)	64·6 (61·4–67·7)
Pennsylvania	72·2 (72·0–72·4)	75·9 (75·7–76·1)	76·1 (75·9–76·3)	74·2 (72·1–76·3)	62·7 (60·0–65·0)	64·6 (61·6–67·2)	64·2 (61·1–67·0)	62·5 (59·0–65·7)
Rhode Island	73·1 (72·6–73·7)	76·9 (76·3–77·4)	77·5 (76·9–78·1)	76·9 (74·7–79·0)	63·6 (61·0–66·1)	65·9 (62·9–68·5)	65·7 (62·5–68·5)	65·1 (61·5–68·4)
South Carolina	70·1 (69·8–70·4)	73·9 (73·7–74·2)	74·0 (73·7–74·3)	71·1 (68·8–73·3)	61·0 (58·5–63·3)	63·4 (60·5–65·9)	62·9 (59·9–65·5)	60·5 (57·1–63·6)
South Dakota	73·5 (72·9–74·0)	76·7 (76·1–77·2)	76·4 (75·7–77·0)	75·3 (73·4–77·0)	64·0 (61·2–66·4)	66·0 (63·1–68·7)	65·3 (62·2–67·9)	64·3 (61·1–67·3)
Tennessee	70·4 (70·2–70·7)	73·5 (73·3–73·8)	73·1 (72·9–73·4)	70·3 (68·0–72·4)	61·4 (58·9–63·6)	63·2 (60·4–65·6)	62·2 (59·3–64·8)	59·8 (56·7–62·9)
Texas	71·6 (71·4–71·7)	76·2 (76·1–76·3)	76·6 (76·5–76·7)	73·4 (71·6–75·3)	62·5 (59·9–64·7)	65·7 (62·8–68·2)	65·5 (62·6–68·2)	62·9 (59·9–65·9)
Utah	75·1 (74·7–75·6)	77·9 (77·4–78·2)	78·3 (77·9–78·7)	76·4 (74·4–78·4)	65·3 (62·4–67·7)	66·8 (63·6–69·5)	66·6 (63·5–69·4)	65·0 (61·6–68·2)
Vermont	73·5 (72·9–74·0)	77·8 (77·3–78·4)	77·9 (77·2–78·6)	76·6 (74·7–78·5)	64·1 (61·4–66·3)	67·1 (64·0–69·7)	66·5 (63·5–69·4)	65·3 (62·2–68·5)
Virginia	72·0 (71·8–72·2)	76·8 (76·6–77·0)	77·3 (77·0–77·5)	75·0 (73·0–77·2)	62·9 (60·3–65·1)	66·1 (63·2–68·6)	65·9 (62·8–68·7)	64·0 (60·8–67·2)
Washington	73·8 (73·5–74·1)	77·9 (77·6–78·1)	78·5 (78·2–78·8)	77·0 (74·9–79·1)	64·1 (61·4–66·5)	67·0 (64·0–69·6)	66·8 (63·6–69·6)	65·5 (62·0–68·9)
West Virginia	71·0 (70·6–71·4)	72·7 (72·3–73·1)	72·3 (71·8–72·8)	69·0 (66·9–71·3)	61·8 (59·3–64·2)	62·2 (59·4–64·8)	61·0 (58·0–63·6)	58·1 (54·8–61·3)
Wisconsin	73·7 (73·5–74·0)	77·3 (77·0–77·5)	77·3 (77·0–77·6)	76·0 (73·8–78·3)	64·1 (61·3–66·5)	66·4 (63·4–69·0)	65·8 (62·7–68·7)	64·6 (61·3–67·9)
Wyoming	73·2 (72·6–73·8)	76·2 (75·6–76·7)	76·2 (75·5–77·0)	73·5 (71·7–75·1)	63·7 (60·9–66·1)	65·5 (62·4–68·1)	65·0 (61·8–67·8)	62·6 (59·6–65·9)

Table S1B: Life expectancy and healthy life expectancy in the USA and by US State and Washington, DC for females, 1990, 2010, 2019, and 2021

	Life expectancy				Healthy life expectancy			
**Location name**	**1990**	**2010**	**2019**	**2021**	**1990**	**2010**	**2019**	**2021**
USA	79·0 (78·9–79·0)	81·1 (81·1–81·2)	81·6 (81·6–81·7)	80·0 (79·9–80·2)	66·8 (63·2–69·8)	67·7 (63·9–71·0)	67·3 (63·4–70·7)	65·7 (61·7–69·1)
Alabama	77·7 (77·4–78·0)	78·0 (77·8–78·3)	78·7 (78·4–79·0)	76·4 (74·6–78·3)	65·7 (62·3–68·7)	65·1 (61·5–68·2)	64·8 (61·0–68·2)	62·7 (58·8–66·4)
Alaska	78·6 (78·1–79·0)	80·2 (79·8–80·7)	80·8 (80·2–81·4)	79·0 (77·7–80·3)	66·3 (62·8–69·4)	67·0 (63·3–70·2)	66·4 (62·3–69·9)	64·7 (60·7–68·2)
Arizona	79·9 (79·6–80·2)	81·8 (81·6–82·1)	82·1 (81·8–82·3)	79·4 (77·8–81·1)	67·2 (63·5–70·4)	67·9 (63·9–71·2)	67·2 (63·1–70·7)	64·8 (60·5–68·5)
Arkansas	78·1 (77·7–78·6)	78·8 (78·4–79·1)	78·7 (78·4–79·1)	76·6 (74·7–78·5)	66·2 (62·9–69·1)	66·2 (62·5–69·2)	65·4 (61·6–68·6)	63·3 (59·5–67·1)
California	79·3 (79·2–79·4)	82·9 (82·8–83·0)	83·9 (83·8–84·0)	82·2 (80·7–83·7)	67·2 (63·7–70·1)	69·5 (65·7–72·7)	69·6 (65·5–73·0)	67·9 (63·9–71·4)
Colorado	80·2 (79·9–80·6)	82·1 (81·8–82·4)	82·8 (82·5–83·1)	81·5 (79·8–83·1)	67·5 (63·7–70·7)	68·4 (64·4–71·7)	68·0 (64·0–71·6)	66·7 (62·6–70·8)
Connecticut	80·2 (79·9–80·5)	82·9 (82·6–83·2)	83·4 (83·0–83·8)	82·8 (81·1–84·6)	67·8 (64·2–70·9)	69·1 (65·4–72·4)	68·9 (64·8–72·6)	68·2 (63·9–72·2)
Delaware	77·9 (77·5–78·4)	80·6 (80·1–80·9)	81·3 (80·7–81·8)	79·9 (78·7–81·1)	65·8 (62·4–68·8)	66·9 (63·1–70·2)	66·6 (62·5–70·3)	65·2 (61·1–68·9)
Washington, DC	73·4 (72·8–73·9)	79·7 (79·2–80·3)	81·3 (80·6–82·0)	80·2 (78·4–81·8)	62·4 (59·2–65·1)	67·0 (63·4–70·1)	67·6 (63·7–71·0)	66·4 (62·6–70·2)
Florida	79·7 (79·6–79·9)	81·9 (81·8–82·1)	82·2 (82·1–82·4)	80·3 (78·7–81·9)	67·1 (63·4–70·3)	68·1 (64·2–71·5)	67·5 (63·4–71·0)	65·5 (61·2–69·4)
Georgia	77·6 (77·3–77·8)	79·7 (79·5–79·9)	80·4 (80·2–80·6)	78·2 (76·6–79·9)	65·7 (62·3–68·6)	66·7 (63·0–69·9)	66·5 (62·6–69·8)	64·5 (60·5–68·2)
Hawaii	81·7 (81·3–82·1)	84·2 (83·8–84·6)	84·8 (84·2–85·2)	84·2 (82·7–85·6)	69·1 (65·3–72·2)	70·7 (66·9–74·0)	70·5 (66·4–74·1)	69·8 (65·7–73·4)
Idaho	80·2 (79·7–80·7)	81·3 (80·9–81·7)	81·8 (81·3–82·2)	80·1 (78·8–81·3)	67·6 (63·8–70·7)	67·7 (63·8–71·1)	67·3 (63·3–70·9)	65·7 (61·5–69·2)
Illinois	78·5 (78·3–78·7)	81·3 (81·1–81·5)	81·9 (81·7–82·1)	80·7 (79·0–82·6)	66·6 (63·1–69·5)	68·2 (64·4–71·4)	67·9 (64·1–71·2)	66·6 (62·8–70·2)
Indiana	78·8 (78·5–79·1)	79·8 (79·6–80·1)	79·7 (79·5–79·9)	78·2 (76·4–80·0)	66·5 (62·9–69·4)	66·2 (62·5–69·6)	65·4 (61·5–68·8)	63·9 (60·2–67·7)
Iowa	81·1 (80·6–81·4)	81·7 (81·4–82·1)	81·9 (81·5–82·3)	81·1 (79·4–82·8)	68·5 (64·9–71·6)	68·6 (64·8–71·9)	67·9 (63·9–71·3)	66·9 (63·2–70·7)
Kansas	80·5 (80·1–80·9)	81·0 (80·6–81·3)	80·6 (80·2–81·0)	79·4 (77·6–81·2)	67·9 (64·3–71·1)	67·6 (63·8–70·8)	66·7 (62·7–70·0)	65·3 (61·3–69·2)
Kentucky	78·2 (77·9–78·5)	78·3 (78·0–78·6)	78·3 (78·0–78·6)	76·3 (74·3–78·1)	65·9 (62·3–68·9)	64·8 (61·0–68·1)	63·9 (60·0–67·3)	61·9 (58·1–65·8)
Louisiana	77·2 (76·9–77·5)	78·6 (78·3–78·9)	78·6 (78·3–78·9)	76·5 (74·8–78·3)	65·2 (61·8–68·2)	65·5 (61·7–68·6)	64·8 (60·9–68·1)	62·8 (59·0–66·6)
Maine	79·7 (79·2–80·1)	81·3 (80·8–81·7)	81·4 (80·8–81·9)	80·4 (78·8–82·0)	67·3 (63·6–70·3)	67·6 (63·9–71·0)	66·8 (62·8–70·5)	65·9 (61·8–69·8)
Maryland	78·3 (78·0–78·6)	81·4 (81·1–81·6)	82·0 (81·7–82·3)	80·9 (79·0–82·8)	66·5 (63·0–69·4)	68·0 (64·2–71·3)	67·9 (64·0–71·4)	66·7 (62·7–70·6)
Massachusetts	80·0 (79·7–80·2)	82·8 (82·5–83·0)	83·2 (83·0–83·5)	83·0 (81·4–84·6)	67·5 (63·9–70·6)	69·0 (65·3–72·4)	68·7 (64·5–72·1)	68·2 (63·9–72·1)
Michigan	78·5 (78·3–78·7)	80·2 (80·0–80·4)	80·9 (80·7–81·2)	79·5 (77·7–81·2)	66·3 (62·7–69·3)	66·8 (63·0–70·0)	66·3 (62·4–69·8)	64·8 (60·7–68·6)
Minnesota	81·0 (80·6–81·3)	82·9 (82·7–83·2)	83·0 (82·7–83·3)	82·0 (80·4–83·6)	68·5 (64·8–71·6)	69·7 (65·8–73·0)	69·0 (65·1–72·4)	67·9 (63·8–71·6)
Mississippi	77·3 (77·0–77·7)	77·7 (77·4–78·1)	77·8 (77·4–78·2)	75·5 (73·6–77·4)	65·8 (62·4–68·7)	65·6 (62·2–68·6)	65·0 (61·4–68·1)	62·8 (59·1–66·4)
Missouri	78·8 (78·5–79·1)	79·9 (79·6–80·1)	80·2 (79·9–80·4)	78·6 (76·8–80·6)	66·7 (63·2–69·8)	66·5 (62·8–69·8)	65·9 (61·9–69·4)	64·4 (60·3–68·2)
Montana	79·7 (79·3–80·2)	80·8 (80·3–81·2)	81·3 (80·8–81·8)	79·9 (78·6–81·2)	67·3 (63·6–70·4)	67·3 (63·5–70·6)	66·9 (63·0–70·4)	65·6 (61·5–69·1)
Nebraska	80·4 (80·0–80·8)	81·8 (81·4–82·1)	81·7 (81·3–82·1)	80·8 (79·3–82·2)	67·9 (64·2–71·1)	68·5 (64·7–71·8)	67·7 (63·9–71·1)	66·6 (62·7–70·4)
Nevada	78·0 (77·6–78·4)	80·1 (79·8–80·4)	81·2 (80·8–81·6)	79·0 (77·7–80·3)	65·9 (62·4–68·8)	66·9 (63·2–70·2)	66·8 (62·8–70·4)	64·8 (61·0–68·5)
New Hampshire	80·0 (79·6–80·4)	82·2 (81·8–82·6)	82·2 (81·7–82·7)	81·7 (80·2–83·1)	67·5 (63·9–70·7)	68·4 (64·4–71·7)	67·5 (63·5–71·1)	66·8 (62·6–70·4)
New Jersey	78·7 (78·4–78·9)	82·3 (82·0–82·5)	83·2 (82·9–83·4)	82·3 (80·6–84·1)	66·7 (63·2–69·7)	68·9 (65·2–72·2)	68·7 (64·8–72·3)	67·8 (63·5–71·6)
New Mexico	79·6 (79·1–80·1)	81·1 (80·6–81·5)	80·9 (80·5–81·4)	78·5 (76·5–80·4)	66·9 (63·3–70·0)	67·2 (63·3–70·6)	66·1 (62·1–69·7)	63·9 (59·8–68·0)
New York	78·4 (78·3–78·6)	82·5 (82·4–82·7)	83·9 (83·7–84·1)	82·7 (81·0–84·3)	66·1 (62·5–69·1)	68·4 (64·4–71·9)	68·7 (64·3–72·3)	67·4 (63·1–71·3)
North Carolina	78·5 (78·3–78·7)	80·4 (80·1–80·6)	80·6 (80·4–80·8)	78·9 (77·2–80·7)	66·7 (63·2–69·6)	67·4 (63·9–70·6)	66·8 (62·9–70·0)	65·1 (61·3–68·9)
North Dakota	81·4 (81·0–81·8)	82·0 (81·6–82·5)	82·5 (81·9–83·1)	82·7 (81·3–83·9)	68·7 (64·9–71·8)	68·4 (64·5–71·7)	68·0 (63·7–71·6)	67·8 (63·4–71·6)
Ohio	78·5 (78·4–78·7)	79·8 (79·7–80·0)	79·8 (79·6–80·0)	78·2 (76·5–79·9)	66·3 (62·7–69·4)	66·4 (62·6–69·7)	65·3 (61·4–68·8)	63·8 (59·7–67·5)

Oklahoma	78·5 (78·1–78·8)	78·2 (77·9–78·6)	78·6 (78·3–79·0)	76·6 (74·7–78·4)	66·2 (62·7–69·2)	65·0 (61·4–68·3)	64·5 (60·6–67·9)	62·5 (58·5–66·3)
Oregon	79·8 (79·4–80·1)	81·6 (81·2–81·9)	82·5 (82·1–82·8)	81·1 (79·2–82·9)	67·5 (63·8–70·6)	68·3 (64·6–71·6)	68·1 (64·0–71·5)	66·7 (62·2–70·5)
Pennsylvania	78·7 (78·5–78·8)	81·0 (80·8–81·2)	81·3 (81·1–81·5)	79·9 (78·2–81·7)	66·4 (62·8–69·4)	67·1 (63·3–70·5)	66·5 (62·4–70·0)	65·1 (61·1–69·1)
Rhode Island	79·8 (79·3–80·2)	81·9 (81·4–82·4)	82·5 (81·9–83·0)	82·3 (80·8–83·6)	67·4 (63·7–70·6)	68·3 (64·4–71·7)	68·0 (64·0–71·5)	67·4 (63·1–71·1)
South Carolina	77·5 (77·1–77·8)	79·4 (79·2–79·7)	79·7 (79·4–80·0)	77·6 (75·8–79·4)	65·4 (62·1–68·4)	66·2 (62·4–69·4)	65·6 (61·8–69·0)	63·6 (59·4–67·5)
South Dakota	80·7 (80·2–81·2)	81·8 (81·3–82·2)	81·5 (80·9–82·1)	81·2 (79·8–82·5)	68·2 (64·6–71·5)	68·3 (64·5–71·6)	67·4 (63·5–70·8)	66·8 (62·7–70·4)
Tennessee	78·1 (77·9–78·4)	78·8 (78·6–79·1)	78·9 (78·7–79·2)	76·9 (75·0–78·9)	66·1 (62·5–69·1)	65·8 (62·1–69·0)	64·9 (61·0–68·3)	63·0 (58·9–66·6)
Texas	79·1 (78·9–79·2)	80·7 (80·6–80·9)	81·6 (81·4–81·7)	79·3 (77·9–80·9)	67·0 (63·6–70·1)	67·7 (64·0–70·8)	67·6 (63·7–70·9)	65·5 (61·6–69·1)
Utah	80·7 (80·3–81·1)	81·7 (81·4–82·0)	81·9 (81·6–82·2)	80·6 (79·4–81·8)	68·0 (64·2–71·2)	68·1 (64·2–71·5)	67·5 (63·4–71·1)	66·1 (62·1–69·8)
Vermont	79·8 (79·4–80·2)	82·1 (81·7–82·6)	82·5 (82·0–83·2)	81·7 (80·5–82·9)	67·7 (64·0–70·7)	68·9 (65·3–72·1)	68·5 (64·7–71·8)	67·5 (63·3–71·1)
Virginia	78·8 (78·5–79·0)	81·3 (81·0–81·5)	81·8 (81·5–82·0)	80·2 (78·4–81·9)	66·6 (63·1–69·7)	67·8 (64·0–71·1)	67·5 (63·5–71·0)	65·9 (61·9–69·5)
Washington	80·0 (79·7–80·4)	82·1 (81·8–82·4)	82·7 (82·4–82·9)	81·7 (79·9–83·4)	67·5 (63·9–70·7)	68·6 (64·7–71·9)	68·1 (64·0–71·6)	67·0 (62·9–71·1)
West Virginia	78·3 (77·8–78·7)	78·1 (77·7–78·5)	77·5 (77·0–78·0)	75·3 (73·3–77·3)	66·0 (62·2–69·0)	64·7 (60·9–68·0)	63·2 (59·2–66·8)	61·1 (57·0–64·7)
Wisconsin	80·0 (79·7–80·3)	81·8 (81·6–82·1)	81·8 (81·5–82·0)	80·8 (79·2–82·6)	67·5 (64·0–70·7)	68·4 (64·4–71·6)	67·5 (63·5–71·0)	66·4 (61·8–70·4)
Wyoming	79·4 (78·9–79·9)	80·3 (79·9–80·8)	80·9 (80·3–81·5)	79·1 (77·9–80·3)	67·0 (63·3–70·1)	67·0 (63·3–70·2)	66·6 (62·7–70·0)	64·8 (60·7–68·4)

Table S2: Cause-specific YLLs for the 25 leading Level 3 causes by US state in 2021 and percentage change between 1990 and 2021

Table S2: Cause-specific YLLs by US state in 2021 and percentage change between 1990 and 2021, Alabama						
**Rank**	**Cause Name**	**2021 Percentage of all cause YLLs**	**2021 YLLs (millions)**	**2021 Age Standardised Rate (per 100 000)**	**Percentage change YLL count 1990 to 2021**	**Percentage change age-standardised YLL rate 1990 to 2021**
	All causes	100.0<br>(100.0–100.0)	1.53<br>(1.35–1.71)	21560.0<br>(19220.5–23996.8)	59.0<br>(41.0–78.3)	6.1<br>(-5.3–18.3)
1	COVID-19	17.3<br>(14.9–20.4)	0.263<br>(0.252–0.300)	3656.3<br>(3504.2–4178.2)	--	--
2	Ischaemic heart disease	11.0<br>(10.1–11.8)	0.168<br>(0.143–0.194)	2000.9<br>(1709.8–2322.5)	-11.0<br>(-23.1–3.1)	-44.2<br>(-51.9—35.1)
3	Chronic obstructive pulmonary disease	5.6<br>(5.1–6.1)	0.0861<br>(0.0738–0.0992)	943.4<br>(807.9–1086.5)	152.6<br>(120.8–189.6)	51.4<br>(32.7–74.3)
4	Tracheal, bronchus, and lung cancer	5.2<br>(4.8–5.7)	0.0795<br>(0.0681–0.0935)	907.3<br>(776.7–1071.2)	6.0<br>(-10.1–25.0)	-38.5<br>(-48.1—27.4)
5	Stroke	4.8<br>(4.4–5.2)	0.0733<br>(0.0625–0.0852)	868.7<br>(742.6–1011.3)	28.3<br>(9.8–48.3)	-20.1<br>(-31.5—7.9)
6	Drug use disorders	3.6<br>(3.0–4.3)	0.0547<br>(0.0439–0.0676)	1078.3<br>(869.9–1332.7)	2132.3<br>(1646.5–2742.4)	1857.4<br>(1430.3–2385.7)
7	Chronic kidney disease	3.4<br>(3.1–3.6)	0.0513<br>(0.0442–0.0594)	624.1<br>(539.9–719.9)	282.4<br>(230.2–341.5)	134.0<br>(102.5–169.9)
8	Road injuries	3.0<br>(2.8–3.2)	0.0463<br>(0.0397–0.0535)	934.8<br>(808.8–1076.3)	-29.6<br>(-40.4—18.2)	-40.7<br>(-49.5—31.4)
9	Cirrhosis and other chronic liver diseases	2.4<br>(2.2–2.6)	0.0374<br>(0.0316–0.0438)	523.5<br>(441.7–611.7)	99.4<br>(69.8–133.2)	31.0<br>(11.7–53.4)
10	Self-harm	2.4<br>(2.1–2.6)	0.0361<br>(0.0305–0.0419)	694.0<br>(588.9–803.2)	47.1<br>(23.8–72.6)	24.7<br>(5.4–45.6)
11	Alzheimer's disease and other dementias	2.2<br>(0.6–5.8)	0.0332<br>(0.00843–0.0882)	345.9<br>(87.7–919.0)	67.9<br>(47.2–90.1)	-3.2<br>(-15.2–10.2)
12	Colon and rectum cancer	2.0<br>(1.9–2.3)	0.0311<br>(0.0268–0.0365)	386.6<br>(333.3–452.8)	29.3<br>(8.9–52.1)	-17.1<br>(-30.3—2.1)
13	Diabetes mellitus	1.9<br>(1.8–2.1)	0.0296<br>(0.0254–0.0350)	379.7<br>(324.7–447.8)	36.6<br>(16.5–62.8)	-10.4<br>(-23.2–6.1)
14	Interpersonal violence	1.9<br>(1.7–2.0)	0.0287<br>(0.0246–0.0332)	637.6<br>(549.3–733.4)	-10.3<br>(-23.3–4.8)	-16.9<br>(-28.4—3.5)
15	Hypertensive heart disease	1.8<br>(1.6–2.0)	0.0278<br>(0.0234–0.0324)	352.7<br>(296.6–410.3)	158.2<br>(118.3–202.7)	68.1<br>(41.0–97.6)
16	Breast cancer	1.5<br>(1.2–1.8)	0.0231<br>(0.0184–0.0276)	298.2<br>(236.9–360.9)	1.8<br>(-18.9–24.4)	-36.3<br>(-49.5—21.8)
17	Lower respiratory infections	1.5<br>(1.3–1.6)	0.0224<br>(0.0184–0.0266)	295.0<br>(247.1–346.8)	-9.2<br>(-24.3–8.5)	-41.2<br>(-50.6—30.1)
18	Pancreatic cancer	1.5<br>(1.4–1.6)	0.0222<br>(0.0190–0.0259)	258.1<br>(221.5–302.4)	92.6<br>(62.9–124.8)	16.5<br>(-1.2–35.9)
19	Neonatal disorders	1.1<br>(0.9–1.3)	0.0169<br>(0.0149–0.0193)	597.0<br>(523.3–679.7)	-48.5<br>(-55.6—40.5)	-44.5<br>(-52.1—35.8)
20	Endocrine, metabolic, blood, and immune disorders	1.1<br>(1.0–1.2)	0.0166<br>(0.0144–0.0193)	250.0<br>(218.5–287.6)	224.7<br>(179.8–281.6)	114.0<br>(86.4–147.8)
21	Prostate cancer	0.9<br>(0.7–1.0)	0.0134<br>(0.0105–0.0167)	143.0<br>(111.8–177.6)	9.3<br>(-15.4–39.0)	-34.3<br>(-49.3—16.4)
22	Cardiomyopathy and myocarditis	0.8<br>(0.7–0.9)	0.0122<br>(0.0103–0.0143)	180.5<br>(154.0–210.0)	-16.1<br>(-28.8—0.5)	-40.9<br>(-49.4—30.4)
23	Congenital birth defects	0.8<br>(0.7–0.9)	0.0122<br>(0.0109–0.0135)	377.9<br>(335.1–422.8)	-31.3<br>(-39.8—22.3)	-32.1<br>(-40.4—21.8)
24	Brain and central nervous system cancer	0.8<br>(0.7–0.8)	0.0119<br>(0.0103–0.0138)	177.4<br>(154.7–202.8)	45.5<br>(25.4–68.7)	-2.4<br>(-14.8–12.4)
25	Leukaemia	0.8<br>(0.7–0.8)	0.0115<br>(0.00986–0.0133)	159.1<br>(138.0–182.4)	7.3<br>(-7.3–24.1)	-29.9<br>(-38.9—19.4)

Table S2: Cause-specific YLLs by US state in 2021 and percentage change between 1990 and 2021, Alaska

Rank	Cause Name	2021 Percentage of all cause YLLs	2021 YLLs (millions)	2021 Age Standardised Rate (per 100 000)	Percentage change YLL count 1990 to 2021	Percentage change age-standardised YLL rate 1990 to 2021
	All causes	100.0<br>(100.0–100.0)	0.158<br>(0.143–0.173)	18059.9<br>(16510.9–19734.2)	99.1<br>(81.1–118.9)	-2.2<br>(-10.9–7.3)
1	COVID-19	17.9<br>(14.4–21.3)	0.0282<br>(0.0223–0.0331)	3033.5<br>(2397.4–3562.5)	--	--
2	Ischaemic heart disease	8.0<br>(7.3–8.6)	0.0126<br>(0.0109–0.0146)	1223.3<br>(1059.0–1414.0)	40.3<br>(23.5–61.2)	-57.4<br>(-62.4—51.4)
3	Self-harm	5.7<br>(5.2–6.1)	0.00891<br>(0.00773–0.0102)	1230.8<br>(1067.7–1408.6)	74.4<br>(50.0–101.9)	42.4<br>(22.6–64.4)
4	Drug use disorders	4.7<br>(4.0–5.5)	0.00743<br>(0.00612–0.00889)	988.0<br>(815.0–1182.2)	1078.2<br>(830.1–1387.4)	958.9<br>(744.6–1238.2)
5	Tracheal, bronchus, and lung cancer	4.3<br>(3.9–4.7)	0.00683<br>(0.00593–0.00776)	627.5<br>(545.3–711.0)	58.3<br>(37.6–82.3)	-50.2<br>(-56.6—42.9)
6	Chronic obstructive pulmonary disease	3.4<br>(3.1–3.7)	0.00542<br>(0.00474–0.00625)	506.2<br>(441.6–584.6)	221.3<br>(179.1–268.5)	-16.9<br>(-27.5—4.8)
7	Stroke	3.2<br>(2.9–3.5)	0.00510<br>(0.00441–0.00586)	527.1<br>(453.3–603.2)	100.8<br>(77.3–129.7)	-36.2<br>(-43.0—27.8)
8	Cirrhosis and other chronic liver diseases	3.0<br>(2.8–3.2)	0.00474<br>(0.00420–0.00542)	504.6<br>(446.6–578.9)	135.5<br>(107.1–169.4)	18.3<br>(4.0–34.7)
9	Alcohol use disorders	2.6<br>(2.5–2.8)	0.00416<br>(0.00368–0.00474)	484.0<br>(426.6–548.5)	167.5<br>(132.7–208.2)	77.6<br>(54.6–103.7)
10	Road injuries	2.4<br>(2.3–2.6)	0.00383<br>(0.00338–0.00433)	553.3<br>(486.0–631.1)	-36.6<br>(-43.9—26.9)	-47.1<br>(-53.2—38.4)
11	Alzheimer's disease and other dementias	2.2<br>(0.5–5.6)	0.00341<br>(0.000885–0.00905)	356.7<br>(93.3–932.1)	380.9<br>(332.7–456.9)	-2.3<br>(-11.3–10.2)
12	Chronic kidney disease	2.0<br>(1.9–2.2)	0.00323<br>(0.00281–0.00371)	334.5<br>(292.0–383.7)	539.9<br>(458.8–640.3)	131.9<br>(105.9–166.1)
13	Colon and rectum cancer	1.9<br>(1.7–2.1)	0.00296<br>(0.00253–0.00342)	290.0<br>(249.4–334.3)	91.4<br>(63.0–123.7)	-35.8<br>(-44.8—25.3)
14	Diabetes mellitus	1.8<br>(1.6–1.9)	0.00276<br>(0.00236–0.00317)	278.1<br>(238.9–317.1)	118.0<br>(87.1–150.3)	-19.3<br>(-30.3—7.2)
15	Interpersonal violence	1.7<br>(1.5–1.8)	0.00260<br>(0.00229–0.00293)	390.3<br>(342.9–442.6)	-8.0<br>(-19.9–4.2)	-16.2<br>(-27.1—5.0)
16	Pancreatic cancer	1.4<br>(1.3–1.5)	0.00216<br>(0.00189–0.00248)	201.4<br>(176.0–231.1)	221.4<br>(179.1–269.4)	1.6<br>(-10.8–16.1)
17	Breast cancer	1.4<br>(1.2–1.6)	0.00214<br>(0.00184–0.00252)	218.6<br>(187.9–257.7)	48.8<br>(26.1–77.9)	-36.4<br>(-45.8—23.6)
18	Other transport injuries	1.3<br>(1.2–1.5)	0.00212<br>(0.00180–0.00249)	293.8<br>(249.2–351.4)	-38.3<br>(-48.0—27.4)	-47.1<br>(-55.5—36.7)
19	Hypertensive heart disease	1.2<br>(1.1–1.3)	0.00191<br>(0.00166–0.00221)	198.0<br>(172.6–228.0)	406.2<br>(341.4–485.1)	87.8<br>(63.9–116.6)
20	Endocrine, metabolic, blood, and immune disorders	1.2<br>(1.1–1.3)	0.00186<br>(0.00164–0.00212)	222.4<br>(195.6–255.7)	269.1<br>(223.3–322.2)	117.1<br>(89.7–149.7)
21	Neonatal disorders	1.1<br>(0.9–1.4)	0.00172<br>(0.00142–0.00208)	423.0<br>(348.8–511.0)	-55.3<br>(-64.1—45.1)	-39.2<br>(-51.1—25.3)
22	Congenital birth defects	1.1<br>(0.9–1.3)	0.00169<br>(0.00141–0.00206)	360.5<br>(295.3–441.2)	-39.6<br>(-51.0—25.0)	-27.6<br>(-42.2—10.2)
23	Falls	1.1<br>(1.0–1.1)	0.00168<br>(0.00145–0.00193)	186.0<br>(160.8–213.6)	192.5<br>(150.6–236.1)	40.0<br>(22.1–60.2)
24	Liver cancer	0.9<br>(0.9–1.0)	0.00148<br>(0.00128–0.00174)	140.9<br>(122.4–164.7)	457.7<br>(374.1–558.2)	104.7<br>(76.2–138.9)
25	Lower respiratory infections	0.9<br>(0.8–1.0)	0.00136<br>(0.00116–0.00160)	159.5<br>(137.6–186.2)	-8.6<br>(-21.5–6.6)	-60.6<br>(-65.7—54.6)

Table S2: Cause-specific YLLs by US state in 2021 and percentage change between 1990 and 2021, Arizona						
**Rank**	**Cause Name**	**2021 Percentage of all cause YLLs**	**2021 YLLs (millions)**	**2021 Age Standardised Rate (per 100 000)**	**Percentage change YLL count 1990 to 2021**	**Percentage change age-standardised YLL rate 1990 to 2021**
	All causes	100.0 (100.0–100.0)	1.74 (1.56–1.94)	17190.6 (15460.6–19088.8)	147.4 (121.4–176.3)	3.0 (-7.6–14.5)
1	COVID-19	19.9 (17.3–23.0)	0.345 (0.332–0.393)	3327.1 (3199.8–3786.5)	--	--
2	Ischaemic heart disease	9.9 (8.9–10.6)	0.172 (0.146–0.201)	1367.3 (1158.1–1599.4)	28.5 (10.3–49.5)	-52.9 (-59.8–-45.2)
3	Drug use disorders	4.7 (4.0–5.6)	0.0822 (0.0661–0.102)	1127.8 (911.8–1389.4)	1484.9 (1098.4–1948.7)	784.6 (573.4–1036.8)
4	Chronic obstructive pulmonary disease	4.3 (3.9–4.7)	0.0750 (0.0643–0.0873)	556.9 (478.5–647.7)	154.0 (118.1–193.8)	-7.6 (-20.7–7.0)
5	Tracheal, bronchus, and lung cancer	3.8 (3.5–4.2)	0.0668 (0.0568–0.0778)	523.2 (445.7–611.6)	36.7 (16.0–60.0)	-51.3 (-58.8–-42.8)
6	Self-harm	3.7 (3.4–4.0)	0.0645 (0.0547–0.0758)	850.6 (724.8–996.3)	104.7 (72.8–141.1)	6.3 (-10.3–24.5)
7	Stroke	3.3 (2.9–3.6)	0.0572 (0.0488–0.0664)	456.4 (389.8–530.5)	95.1 (66.4–124.8)	-27.9 (-38.6–-17.0)
8	Cirrhosis and other chronic liver diseases	3.0 (2.8–3.3)	0.0525 (0.0440–0.0623)	534.6 (450.6–633.6)	187.0 (141.0–239.5)	19.1 (0.3–40.8)
9	Alzheimer's disease and other dementias	3.0 (0.8–7.5)	0.0519 (0.0136–0.133)	358.9 (93.4–922.5)	205.4 (168.6–245.4)	-0.6 (-12.3–12.2)
10	Road injuries	2.7 (2.5–2.9)	0.0466 (0.0393–0.0537)	641.7 (545.8–736.8)	1.0 (-14.9–16.9)	-48.2 (-56.2–-40.3)
11	Chronic kidney disease	2.6 (2.4–2.8)	0.0458 (0.0391–0.0529)	379.7 (325.0–436.5)	664.4 (554.4–782.3)	181.4 (141.4–225.0)
12	Diabetes mellitus	2.1 (2.0–2.3)	0.0373 (0.0319–0.0435)	330.9 (281.3–386.4)	176.6 (136.3–224.3)	9.1 (-6.7–28.0)
13	Colon and rectum cancer	1.9 (1.7–2.1)	0.0332 (0.0279–0.0390)	286.2 (241.0–336.7)	94.7 (61.2–135.4)	-23.0 (-36.4–-6.9)
14	Hypertensive heart disease	1.6 (1.3–1.8)	0.0275 (0.0223–0.0337)	236.8 (193.6–290.8)	551.0 (425.9–704.1)	146.3 (100.1–205.0)
15	Pancreatic cancer	1.5 (1.4–1.6)	0.0261 (0.0223–0.0301)	210.9 (179.7–243.6)	198.0 (154.4–245.4)	10.3 (-6.0–28.0)
16	Breast cancer	1.5 (1.2–1.7)	0.0255 (0.0204–0.0308)	232.2 (184.7–283.0)	57.3 (26.8–90.9)	-39.4 (-51.7–-26.2)
17	Interpersonal violence	1.3 (1.2–1.4)	0.0232 (0.0197–0.0267)	355.2 (305.7–406.3)	31.7 (11.9–53.0)	-24.2 (-35.2–-12.5)
18	Falls	1.3 (1.1–1.4)	0.0223 (0.0191–0.0259)	190.0 (164.7–219.5)	362.0 (297.9–436.2)	66.9 (44.0–93.3)
19	Endocrine, metabolic, blood, and immune disorders	1.3 (1.2–1.4)	0.0223 (0.0193–0.0256)	229.8 (200.3–260.9)	471.4 (392.9–555.2)	127.2 (96.4–159.6)
20	Alcohol use disorders	1.2 (1.1–1.3)	0.0213 (0.0177–0.0251)	250.6 (209.9–294.1)	251.5 (192.9–316.1)	63.9 (36.7–93.1)
21	Lower respiratory infections	1.1 (1.0–1.2)	0.0191 (0.0159–0.0227)	178.3 (150.8–209.4)	3.6 (-14.9–21.3)	-58.3 (-65.6–-51.1)
22	Neonatal disorders	1.0 (0.8–1.1)	0.0167 (0.0146–0.0189)	452.7 (394.8–511.8)	-27.8 (-37.4–-17.0)	-37.7 (-45.9–-28.4)
23	Prostate cancer	0.9 (0.8–1.1)	0.0160 (0.0126–0.0200)	115.8 (91.7–145.7)	72.3 (35.3–118.6)	-36.8 (-50.4–-19.7)
24	Congenital birth defects	0.8 (0.7–0.9)	0.0142 (0.0127–0.0158)	332.7 (294.4–369.8)	-23.8 (-33.3–-13.5)	-42.3 (-49.6–-34.3)
25	Leukaemia	0.8 (0.7–0.8)	0.0137 (0.0120–0.0158)	132.4 (115.8–151.0)	67.1 (45.3–93.4)	-32.6 (-41.2–-22.9)

Table S2: Cause-specific YLLs by US state in 2021 and percentage change between 1990 and 2021, Arkansas

Rank	Cause Name	2021 Percentage of all cause YLLs	2021 YLLs (millions)	2021 Age Standardised Rate (per 100 000)	Percentage change YLL count 1990 to 2021	Percentage change age-standardised YLL rate 1990 to 2021
	All causes	100.0 (100.0–100.0)	0.871 (0.780–0.976)	20651.8 (18518.7–23022.2)	53.7 (35.9–71.9)	6.9 (-5.2–19.6)
1	COVID-19	15.3 (13.3–18.5)	0.133 (0.125–0.157)	3107.6 (2924.3–3683.8)	--	--
2	Ischaemic heart disease	13.9 (12.7–14.9)	0.121 (0.103–0.141)	2435.6 (2076.1–2845.0)	-2.8 (-16.5–13.5)	-33.9 (-43.3—22.6)
3	Chronic obstructive pulmonary disease	5.7 (5.2–6.0)	0.0493 (0.0426–0.0565)	911.0 (784.4–1043.6)	140.2 (107.4–174.1)	57.7 (36.0–80.9)
4	Tracheal, bronchus, and lung cancer	5.4 (4.9–5.9)	0.0471 (0.0400–0.0552)	913.3 (774.9–1073.1)	-1.9 (-17.7–15.7)	-40.3 (-50.0—29.3)
5	Stroke	4.0 (3.6–4.3)	0.0349 (0.0295–0.0402)	701.1 (596.0–809.5)	-3.9 (-17.2–11.2)	-33.7 (-43.0—23.1)
6	Chronic kidney disease	3.1 (2.9–3.4)	0.0273 (0.0235–0.0312)	554.6 (475.2–631.4)	316.2 (258.5–379.2)	170.4 (132.7–210.7)
7	Self-harm	3.1 (2.8–3.4)	0.0271 (0.0227–0.0321)	893.6 (751.7–1059.4)	79.0 (50.8–112.8)	46.2 (23.5–73.7)
8	Road injuries	3.0 (2.8–3.2)	0.0262 (0.0225–0.0302)	887.7 (764.3–1018.0)	-25.9 (-36.9—14.6)	-41.0 (-49.5—32.1)
9	Diabetes mellitus	2.4 (2.2–2.6)	0.0212 (0.0181–0.0248)	455.6 (387.3–534.3)	75.4 (49.8–103.7)	20.9 (3.2–41.0)
10	Hypertensive heart disease	2.4 (2.2–2.7)	0.0211 (0.0176–0.0248)	443.4 (369.5–522.4)	373.2 (295.8–462.2)	223.3 (168.2–286.1)
11	Alzheimer's disease and other dementias	2.4 (0.6–6.1)	0.0208 (0.00531–0.0526)	349.1 (89.0–887.0)	51.4 (33.1–72.6)	-2.1 (-14.0–11.6)
12	Cirrhosis and other chronic liver diseases	2.3 (2.1–2.4)	0.0197 (0.0166–0.0232)	481.6 (407.4–567.2)	112.2 (77.6–151.1)	46.2 (22.4–71.8)
13	Drug use disorders	2.1 (1.6–2.7)	0.0187 (0.0134–0.0250)	628.5 (451.1–837.4)	1316.9 (853.5–1904.7)	1082.5 (697.2–1575.6)
14	Colon and rectum cancer	2.1 (1.9–2.3)	0.0182 (0.0152–0.0214)	383.3 (319.8–449.6)	22.2 (2.1–44.7)	-16.2 (-30.2—0.1)
15	Interpersonal violence	1.6 (1.5–1.7)	0.0139 (0.0119–0.0162)	516.8 (441.2–601.2)	-10.5 (-24.9–4.4)	-22.8 (-35.3—10.5)
16	Pancreatic cancer	1.4 (1.3–1.5)	0.0122 (0.0104–0.0141)	242.9 (207.0–281.8)	72.9 (47.6–100.6)	13.9 (-3.0–31.9)
17	Lower respiratory infections	1.4 (1.2–1.5)	0.0121 (0.00999–0.0144)	270.0 (224.1–319.1)	-25.0 (-38.4—11.1)	-47.7 (-56.5—38.3)
18	Breast cancer	1.4 (1.2–1.7)	0.0121 (0.00959–0.0147)	268.2 (212.2–327.1)	-0.2 (-20.9–21.9)	-34.5 (-48.2—19.1)
19	Neonatal disorders	1.2 (0.9–1.4)	0.0101 (0.00827–0.0122)	588.4 (481.6–710.5)	-27.9 (-41.2—12.4)	-27.0 (-40.5—11.3)
20	Endocrine, metabolic, blood, and immune disorders	1.1 (1.0–1.2)	0.00960 (0.00827–0.0111)	242.9 (210.9–277.2)	247.0 (197.2–303.6)	129.0 (96.8–164.1)
21	Congenital birth defects	0.9 (0.7–1.1)	0.00775 (0.00650–0.00893)	396.8 (331.0–461.9)	-22.2 (-35.5—9.3)	-27.3 (-40.6—14.3)
22	Prostate cancer	0.9 (0.7–1.0)	0.00759 (0.00595–0.00947)	135.2 (105.7–169.0)	1.2 (-21.9–26.3)	-32.4 (-47.8—15.6)
23	Leukaemia	0.8 (0.7–0.8)	0.00679 (0.00583–0.00787)	160.5 (139.3–183.2)	5.5 (-9.6–22.2)	-28.4 (-38.5—17.8)
24	Brain and central nervous system cancer	0.8 (0.7–0.8)	0.00675 (0.00581–0.00778)	173.4 (150.6–198.5)	37.0 (17.7–59.1)	-5.6 (-18.8–8.9)
25	Falls	0.8 (0.7–0.8)	0.00674 (0.00578–0.00769)	143.0 (123.2–163.4)	145.1 (111.1–180.1)	57.6 (35.7–81.4)

Table S2: Cause-specific YLLs by US state in 2021 and percentage change between 1990 and 2021, California

Rank	Cause Name	2021 Percentage of all cause YLLs	2021 YLLs (millions)	2021 Age Standardised Rate (per 100 000)	Percentage change YLL count 1990 to 2021	Percentage change age-standardised YLL rate 1990 to 2021
	All causes	100.0 (100.0–100.0)	7.00 (6.31–7.76)	12670.9 (11468.0–14013.4)	28.5 (15.5–42.4)	-25.6 (-33.0–-17.8)
1	COVID-19	16.4 (14.6–18.5)	1.14 (1.14–1.24)	2073.3 (2059.6–2253.3)	--	--
2	Ischaemic heart disease	11.6 (10.3–12.5)	0.813 (0.689–0.935)	1240.0 (1055.6–1424.3)	-17.5 (-28.8–-6.0)	-58.9 (-64.7–-53.1)
3	Stroke	4.5 (3.9–4.9)	0.315 (0.263–0.362)	481.2 (407.6–552.8)	22.1 (5.9–38.8)	-38.7 (-46.7–-30.2)
4	Drug use disorders	3.9 (3.3–4.5)	0.273 (0.215–0.332)	625.4 (496.5–760.6)	307.5 (213.4–427.1)	232.1 (157.8–328.0)
5	Alzheimer's disease and other dementias	3.8 (1.0–9.3)	0.264 (0.0681–0.668)	353.7 (89.6–900.8)	119.9 (97.6–149.8)	-2.2 (-12.7–10.4)
6	Tracheal, bronchus, and lung cancer	3.7 (3.3–4.0)	0.258 (0.215–0.300)	394.0 (330.6–457.7)	-25.6 (-37.2–-12.2)	-64.0 (-69.7–-57.5)
7	Chronic obstructive pulmonary disease	3.6 (3.1–3.8)	0.249 (0.211–0.283)	363.0 (307.6–412.6)	45.3 (25.7–63.2)	-29.2 (-38.8–-20.2)
8	Chronic kidney disease	3.5 (3.1–3.8)	0.242 (0.203–0.279)	379.8 (321.7–435.6)	372.8 (305.5–437.9)	138.9 (106.8–171.4)
9	Cirrhosis and other chronic liver diseases	3.2 (3.0–3.4)	0.224 (0.192–0.256)	409.1 (349.9–467.0)	43.5 (23.3–65.3)	-16.9 (-28.5–-4.5)
10	Self-harm	2.9 (2.7–3.2)	0.206 (0.175–0.238)	480.4 (411.5–554.0)	16.7 (-0.8–34.1)	-9.8 (-22.8–3.5)
11	Road injuries	2.8 (2.6–3.0)	0.198 (0.173–0.224)	484.8 (422.1–547.2)	-35.8 (-43.9–-26.5)	-50.6 (-56.7–-43.6)
12	Colon and rectum cancer	2.2 (2.0–2.4)	0.156 (0.131–0.181)	257.7 (216.4–299.3)	25.7 (6.9–50.2)	-32.7 (-43.0–-19.6)
13	Diabetes mellitus	2.1 (2.0–2.3)	0.151 (0.128–0.174)	248.6 (211.9–286.2)	88.1 (59.4–118.4)	0.9 (-14.3–17.2)
14	Breast cancer	1.8 (1.5–2.1)	0.126 (0.102–0.153)	214.3 (172.2–261.1)	-2.0 (-20.7–19.1)	-47.2 (-57.5–-35.4)
15	Hypertensive heart disease	1.7 (1.5–2.0)	0.121 (0.0998–0.146)	194.3 (160.0–234.0)	129.6 (92.9–177.3)	18.9 (-0.9–45.1)
16	Pancreatic cancer	1.6 (1.5–1.8)	0.115 (0.0977–0.132)	179.0 (152.4–206.3)	84.0 (57.8–113.7)	-7.7 (-20.6–6.9)
17	Interpersonal violence	1.5 (1.4–1.6)	0.106 (0.0914–0.121)	283.6 (247.5–322.2)	-52.1 (-58.6–-45.1)	-58.6 (-64.0–-52.8)
18	Lower respiratory infections	1.4 (1.2–1.5)	0.0981 (0.0811–0.115)	166.5 (139.4–193.3)	-33.7 (-44.6–-22.6)	-63.6 (-69.4–-57.8)
19	Endocrine, metabolic, blood, and immune disorders	1.1 (1.0–1.2)	0.0793 (0.0689–0.0893)	150.4 (131.8–169.0)	178.7 (144.2–214.6)	62.5 (43.3–82.5)
20	Prostate cancer	1.1 (0.9–1.2)	0.0742 (0.0582–0.0911)	107.7 (84.2–132.1)	26.5 (-2.2–54.6)	-37.8 (-52.2–-23.6)
21	Neonatal disorders	1.1 (0.9–1.3)	0.0741 (0.0658–0.0834)	371.4 (329.7–418.3)	-63.5 (-68.0–-58.8)	-49.1 (-55.3–-42.6)
22	Cardiomyopathy and myocarditis	1.0 (0.9–1.1)	0.0720 (0.0607–0.0830)	134.6 (114.8–154.3)	-26.4 (-37.8–-15.2)	-56.2 (-62.6–-49.8)
23	Liver cancer	1.0 (0.9–1.1)	0.0676 (0.0571–0.0789)	109.3 (92.7–127.2)	234.7 (185.1–289.1)	71.1 (46.6–98.3)
24	Alcohol use disorders	0.9 (0.8–0.9)	0.0613 (0.0518–0.0716)	124.6 (106.2–145.1)	98.4 (66.1–132.8)	31.6 (10.7–54.3)
25	Brain and central nervous system cancer	0.9 (0.8–0.9)	0.0601 (0.0524–0.0681)	116.7 (103.3–131.4)	29.6 (12.0–47.1)	-22.5 (-31.8–-12.4)

Table S2: Cause-specific YLLs by US state in 2021 and percentage change between 1990 and 2021, Colorado

Rank	Cause Name	2021 Percentage of all cause YLLs	2021 YLLs (millions)	2021 Age Standardised Rate (per 100 000)	Percentage change YLL count 1990 to 2021	Percentage change age-standardised YLL rate 1990 to 2021
	All causes	100.0 (100.0–100.0)	1.06 (0.920–1.21)	13411.4 (11708.2–15212.5)	102.3 (73.2–131.8)	-11.3 (-23.6–1.3)
1	COVID-19	13.2 (11.1–16.0)	0.140 (0.133–0.169)	1731.6 (1649.5–2091.0)	--	--
2	Ischaemic heart disease	8.8 (7.8–9.6)	0.0937 (0.0763–0.111)	990.1 (806.5–1177.9)	3.2 (-13.8–21.3)	-61.2 (-67.6—54.2)
3	Self-harm	5.5 (5.1–6.0)	0.0586 (0.0493–0.0691)	972.5 (822.5–1140.9)	107.7 (73.5–144.2)	25.5 (5.5–47.2)
4	Chronic obstructive pulmonary disease	5.2 (4.6–5.6)	0.0549 (0.0447–0.0642)	553.7 (451.1–648.8)	130.6 (93.4–170.9)	-16.2 (-29.6—1.5)
5	Drug use disorders	4.6 (3.9–5.3)	0.0485 (0.0382–0.0598)	801.8 (635.5–984.1)	1607.1 (1194.9–2103.7)	1011.1 (745.9–1330.0)
6	Stroke	3.8 (3.3–4.2)	0.0405 (0.0326–0.0472)	433.4 (350.2–506.0)	76.9 (50.6–105.6)	-31.8 (-41.8—20.8)
7	Tracheal, bronchus, and lung cancer	3.7 (3.4–4.1)	0.0397 (0.0327–0.0468)	408.5 (336.3–480.6)	31.5 (7.5–56.0)	-52.8 (-61.4—44.2)
8	Cirrhosis and other chronic liver diseases	3.5 (3.2–3.8)	0.0372 (0.0307–0.0446)	461.2 (383.7–551.3)	201.0 (148.1–264.9)	34.2 (10.6–61.7)
9	Alzheimer's disease and other dementias	3.4 (0.9–9.0)	0.0353 (0.00891–0.0912)	355.1 (90.3–917.9)	169.5 (134.6–210.4)	-2.0 (-14.7–12.7)
10	Road injuries	2.8 (2.6–3.0)	0.0301 (0.0253–0.0358)	510.2 (432.8–602.1)	-5.0 (-20.1–13.1)	-45.3 (-53.6—35.0)
11	Chronic kidney disease	2.8 (2.5–3.0)	0.0298 (0.0243–0.0348)	324.6 (266.9–377.6)	573.3 (469.0–687.2)	158.0 (117.6–200.9)
12	Colon and rectum cancer	2.0 (1.8–2.2)	0.0213 (0.0176–0.0254)	236.4 (194.5–281.3)	59.6 (29.8–93.9)	-37.2 (-48.9—23.9)
13	Falls	1.8 (1.6–1.9)	0.0189 (0.0155–0.0219)	215.1 (178.7–249.9)	305.8 (241.0–369.5)	65.0 (39.9–90.0)
14	Diabetes mellitus	1.7 (1.5–1.8)	0.0181 (0.0150–0.0212)	203.4 (170.4–239.0)	93.8 (62.8–130.7)	-21.8 (-34.5—7.0)
15	Alcohol use disorders	1.7 (1.6–1.8)	0.0179 (0.0150–0.0212)	250.6 (210.0–296.4)	283.6 (217.5–356.1)	97.4 (64.5–134.9)
16	Breast cancer	1.7 (1.4–1.9)	0.0176 (0.0140–0.0217)	200.5 (159.6–247.9)	35.1 (7.9–66.8)	-45.2 (-56.3—32.0)
17	Pancreatic cancer	1.6 (1.4–1.7)	0.0168 (0.0140–0.0198)	175.6 (147.3–207.0)	170.8 (127.9–218.7)	-0.1 (-15.9–17.9)
18	Hypertensive heart disease	1.5 (1.3–1.7)	0.0160 (0.0128–0.0195)	180.2 (145.2–220.0)	385.6 (296.1–495.8)	95.9 (59.7–141.2)
19	Neonatal disorders	1.2 (1.0–1.5)	0.0128 (0.0112–0.0146)	394.5 (345.4–450.1)	-27.5 (-38.3—15.6)	-41.3 (-50.0—31.6)
20	Endocrine, metabolic, blood, and immune disorders	1.2 (1.1–1.3)	0.0126 (0.0106–0.0146)	165.2 (140.5–190.8)	350.7 (279.4–425.6)	98.2 (69.5–129.1)
21	Interpersonal violence	1.2 (1.1–1.2)	0.0123 (0.0104–0.0145)	226.9 (192.5–266.0)	20.8 (2.9–44.1)	-23.4 (-34.7—9.0)
22	Prostate cancer	1.1 (0.9–1.2)	0.0114 (0.00897–0.0142)	113.6 (89.4–141.5)	69.5 (32.5–117.5)	-38.7 (-52.1—21.4)
23	Lower respiratory infections	1.1 (0.9–1.2)	0.0112 (0.00897–0.0135)	136.9 (111.2–164.0)	-20.9 (-34.8—3.6)	-66.4 (-72.2—59.5)
24	Brain and central nervous system cancer	0.9 (0.9–1.0)	0.00970 (0.00811–0.0113)	126.1 (107.2–145.5)	86.9 (55.6–117.9)	-16.5 (-29.8—3.1)
25	Leukaemia	0.9 (0.8–0.9)	0.00938 (0.00784–0.0111)	115.0 (97.5–134.7)	54.1 (30.1–82.6)	-34.8 (-44.7—23.3)

Table S2: Cause-specific YLLs by US state in 2021 and percentage change between 1990 and 2021, Connecticut

Rank	Cause Name	2021 Percentage of all cause YLLs	2021 YLLs (millions)	2021 Age Standardised Rate (per 100 000)	Percentage change YLL count 1990 to 2021	Percentage change age-standardised YLL rate 1990 to 2021
	All causes	100.0 (100.0–100.0)	0.654 (0.564–0.741)	11600.6 (10017.7–13137.2)	7.5 (-7.2–22.5)	-22.4 (-33.0—11.8)
1	Ischaemic heart disease	12.2 (10.8–13.2)	0.0799 (0.0649–0.0941)	1111.7 (916.9–1305.7)	-37.8 (-47.6—28.1)	-60.4 (-67.1—53.9)
2	COVID-19	10.1 (8.8–11.6)	0.0655 (0.0655–0.0655)	1171.0 (1170.1–1170.8)	--	--
3	Drug use disorders	6.4 (5.7–7.3)	0.0420 (0.0334–0.0512)	1144.7 (912.0–1382.4)	1116.6 (841.9–1450.4)	1167.6 (893.1–1501.2)
4	Tracheal, bronchus, and lung cancer	5.3 (4.8–5.8)	0.0349 (0.0289–0.0411)	507.4 (419.1–598.0)	-25.1 (-38.4—10.4)	-53.4 (-61.9—43.9)
5	Alzheimer's disease and other dementias	4.7 (1.2–11.4)	0.0304 (0.00802–0.0802)	357.3 (94.0–949.7)	67.9 (46.0–93.9)	-1.6 (-15.0–14.0)
6	Chronic obstructive pulmonary disease	4.4 (3.8–4.7)	0.0285 (0.0238–0.0333)	383.0 (319.2–445.9)	51.6 (27.1–76.2)	-3.9 (-19.6–12.2)
7	Stroke	4.0 (3.4–4.4)	0.0263 (0.0216–0.0308)	359.2 (297.3–419.5)	-8.7 (-22.5–5.9)	-42.7 (-51.7—33.2)
8	Chronic kidney disease	3.3 (2.9–3.6)	0.0217 (0.0179–0.0255)	315.1 (262.9–367.6)	248.0 (191.8–307.1)	119.0 (84.4–155.5)
9	Cirrhosis and other chronic liver diseases	2.6 (2.4–2.8)	0.0169 (0.0138–0.0201)	305.4 (247.9–361.5)	21.4 (-1.9–44.4)	-12.8 (-29.8–3.5)
10	Self-harm	2.6 (2.3–2.8)	0.0167 (0.0136–0.0202)	434.4 (355.8–521.0)	0.4 (-18.9–20.5)	-4.4 (-22.7–14.9)
11	Colon and rectum cancer	2.3 (2.0–2.5)	0.0149 (0.0123–0.0175)	230.9 (191.1–271.8)	-24.2 (-37.5—9.1)	-47.7 (-57.3—37.1)
12	Diabetes mellitus	2.0 (1.8–2.2)	0.0133 (0.0111–0.0156)	211.0 (174.1–247.2)	14.3 (-5.4–34.0)	-20.5 (-34.5—6.7)
13	Pancreatic cancer	2.0 (1.8–2.1)	0.0128 (0.0107–0.0150)	189.9 (158.4–221.2)	47.9 (23.5–74.0)	-3.9 (-19.9–13.3)
14	Breast cancer	1.9 (1.6–2.2)	0.0123 (0.00975–0.0151)	205.6 (161.9–253.0)	-30.5 (-44.1—15.5)	-51.7 (-61.5—40.7)
15	Road injuries	1.9 (1.7–2.0)	0.0123 (0.0102–0.0145)	344.3 (287.0–405.5)	-53.0 (-60.9—44.2)	-55.9 (-63.1—47.9)
16	Lower respiratory infections	1.5 (1.3–1.6)	0.00951 (0.00768–0.0115)	147.9 (120.7–177.4)	-44.3 (-54.8—33.9)	-62.2 (-69.1—55.3)
17	Endocrine, metabolic, blood, and immune disorders	1.4 (1.3–1.5)	0.00932 (0.00778–0.0109)	173.2 (145.9–201.0)	191.9 (145.8–241.4)	98.2 (67.5–130.7)
18	Hypertensive heart disease	1.4 (1.2–1.6)	0.00894 (0.00718–0.0109)	139.7 (112.3–172.7)	87.2 (51.5–130.5)	27.6 (2.5–57.7)
19	Falls	1.3 (1.2–1.4)	0.00876 (0.00721–0.0102)	131.7 (109.5–153.3)	127.6 (92.2–162.0)	44.2 (20.5–66.2)
20	Prostate cancer	1.2 (1.0–1.4)	0.00772 (0.00598–0.00971)	102.4 (79.2–129.8)	-8.5 (-30.1–19.3)	-41.6 (-55.6—23.5)
21	Neonatal disorders	1.1 (0.8–1.3)	0.00704 (0.00571–0.00856)	420.1 (340.9–511.0)	-65.6 (-72.1—57.4)	-49.5 (-59.2—37.6)
22	Leukaemia	1.0 (0.9–1.1)	0.00680 (0.00568–0.00797)	118.4 (99.5–137.9)	-10.8 (-25.3–5.1)	-39.0 (-48.8—28.3)
23	Alcohol use disorders	1.0 (0.9–1.1)	0.00670 (0.00544–0.00805)	145.5 (118.2–173.9)	116.2 (75.2–162.3)	81.2 (46.3–119.1)
24	Liver cancer	1.0 (0.9–1.1)	0.00663 (0.00542–0.00805)	103.7 (85.1–125.6)	208.9 (150.6–276.7)	102.6 (64.5–146.7)
25	Cardiomyopathy and myocarditis	1.0 (0.9–1.0)	0.00623 (0.00507–0.00739)	108.4 (89.9–127.9)	-34.8 (-46.3—22.4)	-53.6 (-61.6—44.9)

Table S2: Cause-specific YLLs by US state in 2021 and percentage change between 1990 and 2021, Delaware

Rank	Cause Name	2021 Percentage of all cause YLLs	2021 YLLs (millions)	2021 Age Standardised Rate (per 100 000)	Percentage change YLL count 1990 to 2021	Percentage change age-standardised YLL rate 1990 to 2021
	All causes	100.0 (100.0–100.0)	0.238 (0.217–0.262)	16134.2 (14719.4–17731.4)	71.2 (55.2–88.8)	-10.9 (-19.2–-2.3)
1	COVID-19	12.2 (10.6–14.1)	0.0290 (0.0272–0.0335)	1926.0 (1805.2–2220.6)	--	--
2	Ischaemic heart disease	11.1 (10.0–11.9)	0.0264 (0.0229–0.0300)	1391.5 (1214.6–1583.8)	-8.3 (-18.5–3.4)	-60.1 (-64.8–-54.8)
3	Drug use disorders	6.0 (5.1–7.0)	0.0143 (0.0118–0.0174)	1452.6 (1209.4–1753.8)	1497.2 (1130.8–1956.9)	1145.7 (870.0–1489.8)
4	Tracheal, bronchus, and lung cancer	5.6 (5.1–6.0)	0.0133 (0.0115–0.0152)	710.8 (615.8–814.2)	15.9 (1.2–31.8)	-49.1 (-55.8–-41.8)
5	Chronic obstructive pulmonary disease	4.5 (4.1–4.8)	0.0107 (0.00943–0.0120)	536.1 (475.1–601.6)	144.7 (116.7–172.8)	5.9 (-6.4–18.1)
6	Stroke	4.4 (3.9–4.8)	0.0105 (0.00909–0.0119)	554.0 (484.5–627.3)	72.1 (52.8–93.4)	-25.6 (-33.7–-16.4)
7	Chronic kidney disease	3.2 (3.0–3.5)	0.00771 (0.00684–0.00878)	436.4 (390.7–494.9)	369.0 (317.9–424.7)	110.2 (86.5–136.0)
8	Alzheimer's disease and other dementias	3.2 (0.8–8.3)	0.00764 (0.00199–0.0193)	352.3 (91.8–889.4)	154.4 (134.0–182.4)	-2.8 (-10.9–7.6)
9	Self-harm	2.5 (2.3–2.7)	0.00593 (0.00510–0.00687)	581.1 (502.3–676.0)	52.3 (30.2–78.1)	11.2 (-5.1–30.1)
10	Road injuries	2.4 (2.3–2.6)	0.00582 (0.00514–0.00657)	608.2 (536.8–686.6)	-18.9 (-28.6–-8.0)	-40.8 (-47.7–-33.2)
11	Cirrhosis and other chronic liver diseases	2.4 (2.2–2.6)	0.00571 (0.00502–0.00653)	393.5 (344.1–451.5)	94.9 (70.0–125.1)	3.1 (-10.4–18.9)
12	Diabetes mellitus	2.2 (2.0–2.4)	0.00522 (0.00460–0.00597)	313.8 (274.9–359.3)	43.9 (27.1–64.7)	-29.0 (-37.5–-18.7)
13	Colon and rectum cancer	2.2 (1.9–2.4)	0.00514 (0.00439–0.00595)	296.6 (255.0–342.8)	22.6 (3.7–43.7)	-41.7 (-51.0–-31.8)
14	Hypertensive heart disease	2.0 (1.9–2.2)	0.00483 (0.00420–0.00548)	292.6 (255.6–333.0)	206.5 (168.3–247.8)	48.7 (30.3–69.2)
15	Breast cancer	1.7 (1.5–2.0)	0.00414 (0.00359–0.00477)	260.4 (225.1–300.9)	10.2 (-5.7–27.4)	-45.3 (-53.4–-36.7)
16	Pancreatic cancer	1.7 (1.6–1.8)	0.00410 (0.00357–0.00470)	223.0 (194.4–256.3)	145.4 (115.6–181.0)	9.8 (-3.5–25.9)
17	Endocrine, metabolic, blood, and immune disorders	1.6 (1.5–1.7)	0.00386 (0.00345–0.00434)	280.1 (250.6–311.9)	311.6 (264.7–366.1)	113.6 (89.6–139.9)
18	Neonatal disorders	1.4 (1.1–1.7)	0.00334 (0.00272–0.00407)	658.6 (537.1–802.9)	-43.5 (-55.3–-29.9)	-41.6 (-53.8–-27.5)
19	Cardiomyopathy and myocarditis	1.2 (1.1–1.3)	0.00282 (0.00248–0.00325)	187.2 (164.8–215.1)	18.0 (1.7–37.1)	-39.5 (-47.8–-30.3)
20	Interpersonal violence	1.2 (1.1–1.3)	0.00282 (0.00249–0.00322)	325.3 (287.6–369.4)	2.1 (-10.7–16.9)	-16.7 (-26.6–-5.0)
21	Lower respiratory infections	1.1 (1.0–1.2)	0.00262 (0.00222–0.00307)	162.9 (139.7–189.0)	-15.7 (-27.8–-1.7)	-59.5 (-65.0–-53.2)
22	Prostate cancer	1.0 (0.8–1.1)	0.00238 (0.00195–0.00284)	116.0 (95.2–139.0)	34.8 (10.0–64.5)	-42.9 (-53.7–-30.5)
23	Alcohol use disorders	1.0 (0.9–1.0)	0.00230 (0.00198–0.00266)	189.3 (163.3–219.2)	197.1 (155.9–246.3)	85.2 (59.7–113.7)
24	Falls	0.9 (0.8–1.0)	0.00224 (0.00196–0.00254)	132.0 (117.6–149.4)	231.4 (194.1–272.7)	52.1 (35.0–72.1)
25	Leukaemia	0.9 (0.9–1.0)	0.00218 (0.00193–0.00246)	142.0 (126.0–159.5)	37.9 (21.4–56.0)	-31.6 (-39.6–-23.0)

Table S2: Cause-specific YLLs by US state in 2021 and percentage change between 1990 and 2021, District of Columbia						
Rank	Cause Name	2021 Percentage of all cause YLLs	2021 YLLs (millions)	2021 Age Standardised Rate (per 100 000)	Percentage change YLL count 1990 to 2021	Percentage change age-standardised YLL rate 1990 to 2021
	All causes	100.0 (100.0–100.0)	0.139 (0.123–0.157)	16137.3 (14290.8–18217.2)	-38.9 (-46.1—30.8)	-53.2 (-58.7—46.9)
1	Ischaemic heart disease	11.5 (10.1–12.8)	0.0159 (0.0133–0.0189)	1655.4 (1375.9–1964.1)	-39.1 (-49.0—28.1)	-52.0 (-60.0—43.3)
2	COVID-19	9.8 (7.0–13.4)	0.0137 (0.0101–0.0182)	1471.2 (1085.0–1963.5)	--	--
3	Drug use disorders	6.9 (6.1–7.8)	0.00964 (0.00796–0.0115)	1142.4 (944.9–1366.4)	432.5 (322.8–582.8)	373.5 (275.0–506.6)
4	Interpersonal violence	4.9 (4.5–5.3)	0.00688 (0.00590–0.00791)	1094.2 (936.1–1265.3)	-72.2 (-76.3—67.4)	-71.0 (-75.4—66.0)
5	Tracheal, bronchus, and lung cancer	3.9 (3.6–4.3)	0.00547 (0.00465–0.00648)	582.7 (494.8–690.5)	-52.5 (-59.8—43.0)	-63.3 (-69.1—56.1)
6	Hypertensive heart disease	3.9 (3.3–4.5)	0.00538 (0.00437–0.00665)	567.7 (460.6–707.2)	-1.5 (-20.2–22.8)	-23.7 (-38.6—4.0)
7	Stroke	3.7 (3.2–4.1)	0.00511 (0.00422–0.00607)	518.5 (430.5–615.3)	-41.1 (-50.0—30.9)	-55.0 (-61.8—47.4)
8	Chronic kidney disease	3.0 (1.6–3.7)	0.00412 (0.00213–0.00543)	435.1 (224.5–572.1)	33.6 (-31.7–77.5)	-0.3 (-48.2–32.4)
9	Cirrhosis and other chronic liver diseases	2.9 (2.7–3.1)	0.00399 (0.00338–0.00462)	446.8 (378.9–517.8)	-47.7 (-56.1—38.9)	-59.5 (-66.1—52.8)
10	Alzheimer's disease and other dementias	2.8 (0.8–7.0)	0.00394 (0.00106–0.00981)	341.6 (88.7–865.8)	28.1 (13.6–49.0)	-5.9 (-17.7–8.0)
11	Chronic obstructive pulmonary disease	2.4 (2.2–2.6)	0.00332 (0.00281–0.00382)	338.9 (288.9–389.7)	0.6 (-14.0–16.1)	-18.1 (-29.6—5.4)
12	Diabetes mellitus	2.2 (2.0–2.4)	0.00302 (0.00253–0.00350)	328.0 (276.2–381.0)	-36.1 (-46.2—25.0)	-48.2 (-56.4—39.0)
13	Colon and rectum cancer	2.1 (1.9–2.4)	0.00298 (0.00252–0.00352)	319.9 (270.2–377.5)	-44.8 (-53.3—33.8)	-55.9 (-62.9—47.1)
14	Breast cancer	2.0 (1.6–2.3)	0.00271 (0.00226–0.00323)	293.9 (244.9–351.0)	-46.6 (-56.1—35.3)	-58.7 (-66.2—49.9)
15	Neonatal disorders	1.8 (1.4–2.3)	0.00255 (0.00205–0.00315)	604.1 (484.7–746.1)	-83.1 (-86.6—79.0)	-83.7 (-87.1—79.7)
16	Pancreatic cancer	1.8 (1.6–2.0)	0.00251 (0.00211–0.00294)	267.4 (225.3–313.0)	3.8 (-10.9–21.4)	-17.7 (-29.4—3.3)
17	HIV/AIDS	1.8 (1.6–2.0)	0.00248 (0.00248–0.00249)	286.1 (285.4–286.8)	-89.7 (-90.0—89.5)	-91.3 (-91.5—91.0)
18	Self-harm	1.6 (1.5–1.8)	0.00225 (0.00190–0.00267)	295.2 (248.3–350.9)	-43.0 (-52.4—31.6)	-46.9 (-56.3—36.1)
19	Alcohol use disorders	1.5 (1.3–1.6)	0.00204 (0.00170–0.00242)	233.6 (194.2–277.1)	5.8 (-12.0–25.8)	-14.5 (-28.9–1.6)
20	Endocrine, metabolic, blood, and immune disorders	1.4 (1.3–1.5)	0.00195 (0.00168–0.00222)	238.2 (208.1–271.6)	38.4 (18.4–61.6)	0.7 (-13.3–17.5)
21	Road injuries	1.2 (1.1–1.4)	0.00173 (0.00146–0.00202)	259.2 (219.1–301.6)	-68.6 (-73.6—62.9)	-69.9 (-74.6—64.5)
22	Lower respiratory infections	1.2 (1.0–1.3)	0.00161 (0.00130–0.00190)	180.9 (149.2–212.9)	-70.8 (-76.3—65.1)	-78.6 (-82.4—74.7)
23	Prostate cancer	1.1 (0.9–1.3)	0.00153 (0.00118–0.00193)	155.0 (119.4–194.9)	-43.2 (-56.7—28.3)	-53.4 (-64.8—41.0)
24	Falls	1.1 (1.0–1.2)	0.00151 (0.00127–0.00177)	162.5 (136.8–189.1)	8.0 (-9.1–26.3)	-19.0 (-31.2—5.9)
25	Liver cancer	1.1 (1.0–1.2)	0.00148 (0.00124–0.00175)	164.0 (137.8–194.4)	86.0 (52.7–121.7)	45.0 (19.3–72.3)

Table S2: Cause-specific YLLs by US state in 2021 and percentage change between 1990 and 2021, Florida						
**Rank**	**Cause Name**	**2021 Percentage of all cause YLLs**	**2021 YLLs (millions)**	**2021 Age Standardised Rate (per 100 000)**	**Percentage change YLL count 1990 to 2021**	**Percentage change age-standardised YLL rate 1990 to 2021**
	All causes	100.0 (100.0–100.0)	5.31 (4.72–5.93)	15859.4 (14169.6–17632.7)	73.8 (54.9–94.0)	-10.5 (-19.9—0.5)
1	COVID-19	16.4 (14.4–18.7)	0.869 (0.849–0.991)	2531.6 (2472.4–2887.8)	--	--
2	Ischaemic heart disease	11.4 (10.3–12.3)	0.608 (0.505–0.710)	1409.5 (1177.0–1646.8)	-7.6 (-20.5–6.2)	-53.7 (-60.3—46.6)
3	Tracheal, bronchus, and lung cancer	5.0 (4.6–5.5)	0.268 (0.227–0.313)	622.8 (528.4–726.6)	5.4 (-9.5–23.3)	-51.8 (-58.7—43.3)
4	Drug use disorders	4.7 (4.1–5.5)	0.252 (0.206–0.306)	1154.7 (952.5–1395.4)	2284.8 (1800.6–2914.4)	1428.9 (1127.2–1818.5)
5	Chronic obstructive pulmonary disease	4.5 (4.0–4.9)	0.238 (0.197–0.276)	515.3 (429.5–596.1)	128.7 (94.3–162.7)	14.2 (-2.7–31.9)
6	Stroke	4.4 (3.8–4.7)	0.231 (0.188–0.270)	527.7 (437.6–611.8)	64.2 (40.6–87.7)	-21.5 (-33.3—9.6)
7	Alzheimer's disease and other dementias	3.5 (0.9–8.9)	0.187 (0.0486–0.463)	354.7 (92.6–892.8)	113.6 (89.8–146.9)	-1.4 (-11.8–13.2)
8	Road injuries	2.8 (2.6–3.0)	0.147 (0.126–0.170)	687.5 (593.5–791.0)	-2.4 (-16.4–13.0)	-42.7 (-50.8—34.0)
9	Chronic kidney disease	2.7 (2.5–2.9)	0.145 (0.122–0.169)	355.1 (302.3–412.5)	437.0 (360.6–521.0)	152.6 (116.9–191.6)
10	Self-harm	2.7 (2.5–2.9)	0.144 (0.122–0.169)	613.7 (523.1–715.3)	60.0 (34.7–87.6)	-4.5 (-19.2–11.9)
11	Cirrhosis and other chronic liver diseases	2.5 (2.3–2.7)	0.135 (0.112–0.159)	409.5 (341.0–482.4)	112.6 (76.4–150.6)	6.7 (-11.6–25.5)
12	Colon and rectum cancer	2.1 (1.9–2.3)	0.113 (0.0950–0.134)	294.9 (247.0–352.3)	35.3 (14.6–62.9)	-27.0 (-38.4—11.8)
13	Diabetes mellitus	2.1 (1.9–2.3)	0.112 (0.0952–0.133)	297.1 (250.5–351.1)	83.0 (56.6–117.3)	-5.0 (-18.9–12.8)
14	Breast cancer	1.6 (1.4–2.0)	0.0871 (0.0710–0.105)	242.0 (196.3–294.3)	19.2 (-3.1–44.4)	-41.5 (-52.6—28.2)
15	Pancreatic cancer	1.6 (1.5–1.8)	0.0861 (0.0724–0.101)	205.2 (172.4–239.8)	121.1 (88.7–158.9)	8.3 (-8.0–26.2)
16	Hypertensive heart disease	1.6 (1.4–1.8)	0.0851 (0.0706–0.101)	231.8 (192.7–275.8)	160.6 (118.2–212.9)	38.5 (15.4–65.4)
17	Interpersonal violence	1.4 (1.3–1.5)	0.0724 (0.0622–0.0838)	386.4 (333.4–445.2)	-18.8 (-30.1—5.5)	-45.7 (-52.8—37.4)
18	Endocrine, metabolic, blood, and immune disorders	1.3 (1.1–1.3)	0.0666 (0.0567–0.0778)	211.1 (182.9–243.1)	299.8 (243.7–358.8)	83.8 (59.9–109.3)
19	Falls	1.1 (1.0–1.3)	0.0607 (0.0500–0.0707)	144.6 (120.6–167.0)	299.1 (241.9–359.8)	63.8 (40.1–88.9)
20	Neonatal disorders	1.1 (0.9–1.3)	0.0568 (0.0504–0.0648)	548.0 (485.6–624.5)	-36.5 (-44.5—26.5)	-44.4 (-51.3—35.6)
21	Cardiomyopathy and myocarditis	1.0 (0.9–1.1)	0.0535 (0.0444–0.0627)	160.0 (135.0–184.9)	3.1 (-14.8–21.0)	-47.3 (-56.0—38.5)
22	Prostate cancer	1.0 (0.8–1.2)	0.0531 (0.0418–0.0646)	110.5 (86.5–134.8)	19.3 (-4.4–45.9)	-39.1 (-51.6—25.0)
23	Lower respiratory infections	1.0 (0.8–1.1)	0.0509 (0.0414–0.0613)	140.4 (117.3–167.8)	-7.0 (-22.2–11.0)	-53.1 (-60.5—44.3)
24	Alcohol use disorders	0.8 (0.8–0.9)	0.0447 (0.0375–0.0533)	163.5 (137.7–192.6)	152.9 (112.7–200.1)	36.3 (14.0–61.5)
25	Leukaemia	0.8 (0.8–0.9)	0.0445 (0.0376–0.0516)	126.9 (109.2–145.2)	37.2 (17.8–57.7)	-34.2 (-42.9—24.6)

Table S2: Cause-specific YLLs by US state in 2021 and percentage change between 1990 and 2021, Georgia						
Rank	Cause Name	2021 Percentage of all cause YLLs	2021 YLLs (millions)	2021 Age Standardised Rate (per 100 000)	Percentage change YLL count 1990 to 2021	Percentage change age-standardised YLL rate 1990 to 2021
	All causes	100.0 (100.0–100.0)	2.55 (2.28–2.85)	17911.0 (16059.1–19854.6)	85.0 (64.8–107.6)	-11.6 (-20.9—1.5)
1	COVID-19	19.1 (17.0–21.3)	0.486 (0.484–0.515)	3363.1 (3348.2–3563.0)	--	--
2	Ischaemic heart disease	10.3 (9.3–11.0)	0.262 (0.222–0.306)	1595.4 (1352.6–1865.3)	4.6 (-10.9–21.9)	-55.6 (-62.3—48.3)
3	Tracheal, bronchus, and lung cancer	4.7 (4.3–5.1)	0.121 (0.103–0.139)	697.3 (591.6–803.2)	22.4 (3.4–43.3)	-51.5 (-59.0—43.2)
4	Chronic obstructive pulmonary disease	4.6 (4.2–4.9)	0.119 (0.101–0.137)	680.2 (580.1–791.8)	182.6 (143.2–226.5)	14.9 (-0.9–33.1)
5	Stroke	4.4 (3.9–4.7)	0.111 (0.0941–0.129)	680.8 (577.7–787.6)	46.2 (24.7–69.2)	-37.4 (-46.5—27.8)
6	Chronic kidney disease	3.5 (3.2–3.8)	0.0904 (0.0775–0.105)	562.0 (483.1–647.0)	401.8 (329.3–481.6)	114.0 (84.6–148.3)
7	Drug use disorders	3.1 (2.6–3.7)	0.0788 (0.0638–0.0973)	695.4 (564.7–854.5)	1378.9 (1063.8–1827.9)	888.7 (683.6–1176.0)
8	Self-harm	2.8 (2.6–3.1)	0.0722 (0.0607–0.0841)	635.7 (536.1–739.3)	66.2 (39.8–94.1)	5.9 (-10.6–23.5)
9	Road injuries	2.8 (2.6–3.0)	0.0717 (0.0612–0.0831)	655.6 (565.2–754.4)	-19.0 (-31.1—5.2)	-49.2 (-56.5—40.9)
10	Hypertensive heart disease	2.6 (2.3–2.9)	0.0660 (0.0547–0.0795)	421.0 (348.6–505.2)	346.8 (273.1–429.6)	98.2 (65.5–135.2)
11	Alzheimer's disease and other dementias	2.4 (0.6–6.1)	0.0600 (0.0153–0.157)	348.9 (89.1–906.3)	146.4 (116.6–180.0)	-2.6 (-13.9–10.3)
12	Cirrhosis and other chronic liver diseases	2.2 (2.1–2.4)	0.0573 (0.0486–0.0673)	378.5 (321.7–442.2)	104.6 (72.9–141.9)	-5.6 (-20.3–11.2)
13	Colon and rectum cancer	2.1 (1.9–2.4)	0.0539 (0.0453–0.0626)	333.7 (279.9–387.4)	78.5 (48.2–112.6)	-22.7 (-35.9—8.2)
14	Diabetes mellitus	2.0 (1.8–2.2)	0.0511 (0.0433–0.0600)	324.1 (275.6–380.4)	94.4 (65.7–129.8)	-13.5 (-26.1–1.6)
15	Interpersonal violence	1.8 (1.7–1.9)	0.0462 (0.0396–0.0530)	464.6 (401.2–529.2)	-9.7 (-23.4–4.3)	-35.8 (-45.1—26.5)
16	Breast cancer	1.6 (1.4–1.9)	0.0419 (0.0339–0.0508)	269.3 (218.3–327.7)	36.3 (10.6–65.9)	-39.0 (-50.5—25.8)
17	Pancreatic cancer	1.5 (1.3–1.6)	0.0372 (0.0321–0.0432)	218.8 (188.6–254.2)	151.8 (113.8–190.8)	3.1 (-12.6–19.2)
18	Neonatal disorders	1.4 (1.2–1.7)	0.0366 (0.0321–0.0414)	619.9 (542.6–701.6)	-44.3 (-51.2—36.6)	-48.7 (-55.1—41.7)
19	Lower respiratory infections	1.2 (1.1–1.4)	0.0315 (0.0262–0.0376)	213.8 (179.5–252.9)	-15.0 (-29.3–0.8)	-60.8 (-67.1—53.8)
20	Endocrine, metabolic, blood, and immune disorders	1.1 (1.1–1.2)	0.0293 (0.0257–0.0335)	216.2 (191.9–244.6)	275.7 (230.1–334.1)	83.6 (62.0–109.5)
21	Prostate cancer	0.9 (0.8–1.1)	0.0239 (0.0189–0.0292)	135.4 (107.6–165.6)	48.5 (18.1–83.8)	-39.6 (-51.8—25.3)
22	Cardiomyopathy and myocarditis	0.9 (0.8–1.0)	0.0234 (0.0199–0.0274)	166.3 (143.9–193.3)	0.3 (-14.7–17.0)	-50.9 (-58.0—43.1)
23	Falls	0.8 (0.7–0.9)	0.0211 (0.0180–0.0244)	136.2 (116.4–157.3)	167.9 (129.2–209.0)	20.5 (3.9–38.9)
24	Alcohol use disorders	0.8 (0.7–0.9)	0.0201 (0.0170–0.0236)	151.8 (128.8–177.6)	65.5 (38.4–94.7)	-11.5 (-25.5–3.5)
25	Congenital birth defects	0.8 (0.7–0.9)	0.0200 (0.0181–0.0224)	293.6 (262.1–332.7)	-33.4 (-40.8—23.8)	-45.6 (-52.2—37.1)

Table S2: Cause-specific YLLs by US state in 2021 and percentage change between 1990 and 2021, Hawaii						
Rank	Cause Name	2021 Percentage of all cause YLLs	2021 YLLs (millions)	2021 Age Standardised Rate (per 100 000)	Percentage change YLL count 1990 to 2021	Percentage change age-standardised YLL rate 1990 to 2021
	All causes	100.0 (100.0–100.0)	0.251 (0.220–0.287)	11237.9 (9859.1–12767.7)	48.3 (29.2–70.7)	-19.4 (-29.7–-7.6)
1	Ischaemic heart disease	11.9 (10.5–12.9)	0.0299 (0.0245–0.0353)	1075.1 (882.0–1273.3)	4.2 (-12.6–22.4)	-52.5 (-60.8–-43.9)
2	COVID-19	5.9 (5.1–7.2)	0.0149 (0.0144–0.0190)	651.4 (631.4–831.0)	--	--
3	Stroke	5.7 (4.8–6.3)	0.0143 (0.0117–0.0167)	493.2 (409.0–577.1)	43.7 (22.5–67.6)	-37.3 (-46.5–-27.3)
4	Tracheal, bronchus, and lung cancer	5.4 (4.8–5.8)	0.0135 (0.0113–0.0158)	493.8 (416.1–581.9)	21.2 (1.4–43.9)	-42.7 (-51.8–-32.2)
5	Alzheimer's disease and other dementias	5.3 (1.4–12.5)	0.0132 (0.00366–0.0325)	357.4 (96.8–902.5)	197.7 (159.0–249.8)	-0.8 (-11.3–14.6)
6	Chronic kidney disease	3.7 (3.3–4.0)	0.00936 (0.00781–0.0109)	345.1 (294.4–400.3)	440.4 (366.4–529.4)	146.9 (113.3–185.7)
7	Drug use disorders	3.7 (3.3–4.2)	0.00935 (0.00757–0.0113)	618.1 (503.6–740.6)	1057.9 (806.1–1355.7)	895.9 (685.0–1154.1)
8	Self-harm	3.6 (3.3–3.9)	0.00904 (0.00750–0.0107)	599.1 (495.1–707.9)	27.8 (4.8–52.5)	5.7 (-13.5–25.8)
9	Chronic obstructive pulmonary disease	3.1 (2.7–3.4)	0.00786 (0.00648–0.00930)	261.8 (217.7–310.9)	111.2 (80.3–146.9)	-6.6 (-20.6–9.5)
10	Colon and rectum cancer	2.7 (2.3–2.9)	0.00666 (0.00556–0.00793)	262.6 (218.7–313.0)	31.8 (6.7–57.3)	-33.5 (-46.2–-20.2)
11	Cirrhosis and other chronic liver diseases	2.5 (2.3–2.7)	0.00632 (0.00531–0.00747)	302.1 (255.1–357.5)	82.4 (50.9–118.4)	9.6 (-9.5–30.8)
12	Pancreatic cancer	2.2 (2.0–2.4)	0.00551 (0.00460–0.00646)	209.2 (175.5–245.7)	115.7 (82.1–152.3)	6.0 (-10.3–24.3)
13	Road injuries	2.2 (2.1–2.3)	0.00549 (0.00470–0.00642)	392.8 (339.5–454.2)	-39.4 (-48.6–-28.5)	-50.0 (-57.6–-41.6)
14	Lower respiratory infections	2.1 (1.7–2.3)	0.00523 (0.00419–0.00638)	199.4 (163.5–238.3)	-3.9 (-22.0–14.7)	-56.2 (-63.7–-48.2)
15	Hypertensive heart disease	2.0 (1.8–2.2)	0.00512 (0.00427–0.00606)	203.9 (170.7–244.2)	292.8 (233.8–367.7)	95.8 (62.9–136.9)
16	Diabetes mellitus	2.0 (1.8–2.2)	0.00508 (0.00424–0.00594)	204.2 (171.8–238.5)	39.6 (17.6–63.6)	-28.3 (-39.8–-15.7)
17	Breast cancer	1.9 (1.6–2.3)	0.00479 (0.00395–0.00565)	205.5 (169.3–243.2)	25.5 (3.8–50.8)	-32.9 (-44.8–-18.8)
18	Cardiomyopathy and myocarditis	1.6 (1.5–1.8)	0.00414 (0.00345–0.00493)	189.0 (159.6–223.8)	-7.8 (-22.8–11.4)	-47.6 (-56.3–-37.2)
19	Liver cancer	1.5 (1.4–1.7)	0.00383 (0.00315–0.00457)	156.0 (128.3–186.4)	261.1 (197.2–329.6)	84.8 (52.4–119.3)
20	Neonatal disorders	1.5 (1.2–1.9)	0.00380 (0.00314–0.00461)	522.1 (431.5–633.6)	-46.7 (-56.6–-33.9)	-32.6 (-45.1–-16.3)
21	Falls	1.4 (1.2–1.5)	0.00351 (0.00290–0.00410)	131.7 (110.5–154.8)	205.5 (155.2–255.8)	41.1 (18.1–65.5)
22	Endocrine, metabolic, blood, and immune disorders	1.4 (1.3–1.5)	0.00347 (0.00296–0.00399)	169.1 (147.4–192.8)	243.8 (197.0–295.4)	94.3 (69.6–122.2)
23	Prostate cancer	1.1 (0.9–1.3)	0.00271 (0.00208–0.00342)	88.0 (66.9–111.4)	33.6 (4.3–67.9)	-42.4 (-55.4–-27.3)
24	Stomach cancer	1.0 (0.9–1.1)	0.00256 (0.00216–0.00303)	103.5 (87.3–123.4)	-5.6 (-21.7–11.6)	-50.8 (-59.2–-41.4)
25	Parkinson's disease	1.0 (0.9–1.1)	0.00256 (0.00208–0.00302)	78.8 (64.4–93.2)	255.8 (203.3–314.7)	41.2 (19.9–65.3)

Table S2: Cause-specific YLLs by US state in 2021 and percentage change between 1990 and 2021, Idaho						
**Rank**	**Cause Name**	**2021 Percentage of all cause YLLs**	**2021 YLLs (millions)**	**2021 Age Standardised Rate (per 100 000)**	**Percentage change YLL count 1990 to 2021**	**Percentage change age-standardised YLL rate 1990 to 2021**
	All causes	100.0 (100.0–100.0)	0.375 (0.330–0.417)	14668.2 (12930.7–16273.6)	114.6 (87.7–139.4)	-5.1 (-16.7–5.5)
1	COVID-19	18.0 (15.9–21.0)	0.0671 (0.0649–0.0769)	2668.8 (2580.4–3058.6)	--	--
2	Ischaemic heart disease	9.7 (8.7–10.5)	0.0363 (0.0302–0.0421)	1206.0 (1008.7–1403.2)	10.3 (-6.5–27.0)	-54.9 (-61.9–48.0)
3	Chronic obstructive pulmonary disease	5.4 (4.8–5.8)	0.0202 (0.0170–0.0232)	625.4 (529.2–719.4)	172.6 (131.5–209.5)	8.6 (-7.9–23.6)
4	Self-harm	4.3 (3.9–4.7)	0.0161 (0.0134–0.0190)	849.5 (708.8–993.6)	101.2 (67.3–138.7)	9.9 (-8.3–30.3)
5	Tracheal, bronchus, and lung cancer	4.1 (3.6–4.5)	0.0152 (0.0128–0.0176)	495.8 (417.8–575.1)	41.7 (18.0–67.4)	-45.8 (-55.1–35.9)
6	Stroke	3.7 (3.3–4.0)	0.0139 (0.0117–0.0160)	460.8 (390.3–526.7)	53.8 (32.0–76.0)	-36.4 (-45.3–26.9)
7	Alzheimer's disease and other dementias	3.1 (0.8–8.1)	0.0117 (0.00306–0.0309)	356.0 (92.8–938.5)	151.6 (122.8–179.2)	-0.5 (-11.9–10.2)
8	Road injuries	2.9 (2.7–3.1)	0.0109 (0.00933–0.0126)	588.4 (504.3–680.0)	-11.7 (-24.7–2.9)	-51.9 (-58.8–44.0)
9	Drug use disorders	2.8 (2.3–3.4)	0.0106 (0.00828–0.0133)	578.0 (454.4–723.1)	1681.1 (1237.0–2293.4)	927.3 (670.3–1276.5)
10	Chronic kidney disease	2.7 (2.4–2.9)	0.0100 (0.00848–0.0116)	339.6 (288.4–390.9)	523.7 (439.0–614.4)	151.8 (117.5–188.6)
11	Cirrhosis and other chronic liver diseases	2.6 (2.4–2.8)	0.00972 (0.00814–0.0113)	391.9 (328.3–453.6)	234.0 (179.8–288.4)	47.4 (23.4–72.0)
12	Colon and rectum cancer	1.9 (1.7–2.1)	0.00713 (0.00596–0.00842)	250.6 (209.0–295.5)	66.8 (36.2–97.6)	-29.3 (-42.4–16.1)
13	Diabetes mellitus	1.9 (1.7–2.1)	0.00708 (0.00594–0.00819)	250.9 (210.8–289.8)	95.1 (62.8–127.4)	-17.5 (-31.6–3.9)
14	Pancreatic cancer	1.6 (1.4–1.7)	0.00582 (0.00484–0.00679)	192.7 (161.0–223.8)	165.2 (121.5–210.1)	6.0 (-11.8–23.7)
15	Breast cancer	1.6 (1.3–1.8)	0.00582 (0.00489–0.00696)	217.1 (182.2–261.0)	37.4 (15.0–66.7)	-42.7 (-52.0–30.5)
16	Endocrine, metabolic, blood, and immune disorders	1.3 (1.2–1.4)	0.00501 (0.00428–0.00575)	204.2 (175.3–233.6)	371.4 (302.8–445.6)	104.2 (75.7–136.2)
17	Falls	1.3 (1.2–1.4)	0.00482 (0.00407–0.00551)	168.3 (143.3–192.4)	301.0 (245.6–356.2)	62.2 (40.0–84.9)
18	Lower respiratory infections	1.1 (1.0–1.2)	0.00418 (0.00336–0.00494)	151.7 (123.7–178.1)	-10.3 (-26.7–5.4)	-61.4 (-68.3–55.0)
19	Hypertensive heart disease	1.1 (1.0–1.2)	0.00417 (0.00348–0.00489)	147.6 (123.5–173.3)	315.2 (257.1–387.4)	75.7 (50.3–105.9)
20	Neonatal disorders	1.1 (0.9–1.4)	0.00413 (0.00343–0.00502)	352.1 (292.1–427.9)	-22.3 (-36.6–3.5)	-44.5 (-54.7–31.0)
21	Prostate cancer	1.1 (0.9–1.2)	0.00404 (0.00325–0.00495)	122.6 (98.8–150.5)	47.9 (18.8–84.9)	-40.9 (-52.7–26.2)
22	Congenital birth defects	0.9 (0.8–1.1)	0.00337 (0.00284–0.00394)	256.6 (215.2–301.9)	-23.9 (-36.6–10.3)	-49.9 (-58.6–40.6)
23	Brain and central nervous system cancer	0.9 (0.8–1.0)	0.00337 (0.00283–0.00394)	139.2 (118.2–161.1)	96.1 (65.2–129.9)	-12.2 (-25.6–2.5)
24	Leukaemia	0.9 (0.8–0.9)	0.00330 (0.00278–0.00382)	124.7 (106.7–143.6)	53.9 (30.1–76.4)	-34.1 (-44.1–24.6)
25	Parkinson's disease	0.9 (0.8–0.9)	0.00321 (0.00267–0.00370)	96.7 (80.4–111.5)	305.9 (247.2–363.3)	63.8 (40.2–86.7)

Table S2: Cause-specific YLLs by US state in 2021 and percentage change between 1990 and 2021, Illinois						
Rank	Cause Name	2021 Percentage of all cause YLLs	2021 YLLs (millions)	2021 Age Standardised Rate (per 100 000)	Percentage change YLL count 1990 to 2021	Percentage change age-standardised YLL rate 1990 to 2021
	All causes	100.0 (100.0–100.0)	2.61 (2.29–2.95)	14309.3 (12665.2–16060.3)	7.4 (-5.4–21.9)	-21.6 (-30.7–-11.6)
1	Ischaemic heart disease	12.2 (11.2–13.1)	0.320 (0.271–0.372)	1447.8 (1233.1–1693.1)	-38.5 (-46.8–-29.4)	-59.1 (-64.8–-52.7)
2	COVID-19	11.1 (9.8–12.7)	0.288 (0.285–0.326)	1572.2 (1554.8–1780.3)	--	--
3	Tracheal, bronchus, and lung cancer	5.5 (5.0–6.0)	0.143 (0.120–0.168)	648.9 (544.3–759.6)	-18.2 (-31.8–-3.4)	-48.8 (-57.4–-39.3)
4	Drug use disorders	4.7 (4.1–5.4)	0.123 (0.102–0.149)	921.5 (766.5–1108.0)	664.2 (512.2–850.4)	648.5 (501.2–828.1)
5	Stroke	4.6 (4.1–5.0)	0.121 (0.102–0.141)	541.6 (460.8–628.5)	1.6 (-12.2–17.0)	-33.1 (-42.3–-22.9)
6	Chronic obstructive pulmonary disease	4.5 (4.0–4.9)	0.118 (0.0998–0.138)	508.3 (430.4–593.4)	62.3 (38.7–90.4)	5.7 (-9.8–24.3)
7	Chronic kidney disease	3.7 (3.3–4.2)	0.0973 (0.0820–0.115)	452.1 (383.4–530.2)	234.8 (185.5–291.7)	118.1 (86.7–152.9)
8	Alzheimer's disease and other dementias	3.5 (0.9–8.6)	0.0907 (0.0237–0.233)	352.9 (90.7–904.3)	58.1 (40.3–81.5)	-2.6 (-13.6–11.8)
9	Self-harm	2.5 (2.3–2.8)	0.0663 (0.0557–0.0782)	495.2 (416.8–580.5)	10.7 (-7.3–30.9)	3.4 (-13.5–21.6)
10	Colon and rectum cancer	2.4 (2.2–2.6)	0.0632 (0.0527–0.0747)	304.7 (253.4–360.5)	-12.0 (-26.6–4.3)	-39.2 (-49.6–-27.9)
11	Cirrhosis and other chronic liver diseases	2.3 (2.1–2.4)	0.0600 (0.0513–0.0706)	326.1 (278.2–383.5)	6.4 (-10.0–25.4)	-24.4 (-36.2–-10.8)
12	Interpersonal violence	2.2 (2.0–2.3)	0.0568 (0.0479–0.0653)	504.1 (429.1–577.3)	-31.7 (-42.0–-20.7)	-28.9 (-39.3–-18.0)
13	Diabetes mellitus	2.1 (1.9–2.2)	0.0548 (0.0465–0.0645)	269.9 (229.3–316.8)	7.2 (-7.7–25.7)	-25.3 (-35.7–-12.2)
14	Hypertensive heart disease	2.1 (1.8–2.3)	0.0545 (0.0451–0.0648)	270.7 (224.4–321.2)	113.3 (77.4–153.1)	48.2 (23.3–76.8)
15	Road injuries	2.0 (1.9–2.1)	0.0527 (0.0458–0.0609)	409.0 (356.7–470.0)	-48.7 (-56.3–-40.6)	-53.4 (-59.9–-46.3)
16	Breast cancer	1.9 (1.5–2.2)	0.0492 (0.0392–0.0604)	248.2 (198.2–305.7)	-22.9 (-38.0–-5.9)	-47.8 (-58.4–-36.0)
17	Pancreatic cancer	1.8 (1.7–1.9)	0.0468 (0.0401–0.0547)	216.3 (185.3–253.4)	51.8 (29.9–75.5)	-0.9 (-15.5–14.5)
18	Lower respiratory infections	1.5 (1.3–1.7)	0.0394 (0.0321–0.0474)	198.3 (163.1–236.3)	-39.7 (-49.8–-27.2)	-57.7 (-64.3–-49.5)
19	Neonatal disorders	1.4 (1.1–1.6)	0.0355 (0.0312–0.0402)	565.8 (496.8–641.6)	-63.7 (-68.2–-58.2)	-44.8 (-51.7–-36.5)
20	Endocrine, metabolic, blood, and immune disorders	1.2 (1.1–1.3)	0.0309 (0.0267–0.0356)	179.3 (156.8–204.2)	135.9 (102.5–171.6)	67.6 (44.8–91.1)
21	Cardiomyopathy and myocarditis	1.1 (1.0–1.2)	0.0294 (0.0249–0.0349)	163.7 (140.8–192.1)	-28.5 (-39.0–-15.1)	-47.5 (-55.0–-38.3)
22	Prostate cancer	1.0 (0.9–1.2)	0.0271 (0.0214–0.0336)	115.5 (90.6–143.5)	-10.4 (-29.6–10.9)	-41.1 (-53.7–-26.7)
23	Falls	1.0 (0.9–1.1)	0.0270 (0.0230–0.0314)	128.3 (109.8–148.9)	85.1 (58.1–113.0)	18.4 (1.4–36.2)
24	Leukaemia	0.9 (0.8–1.0)	0.0239 (0.0202–0.0276)	128.1 (110.4–146.9)	-17.3 (-28.7–-5.2)	-42.0 (-49.6–-33.7)
25	Non-Hodgkin lymphoma	0.8 (0.8–0.9)	0.0217 (0.0184–0.0258)	104.3 (87.8–123.1)	-18.6 (-30.7–-4.9)	-46.1 (-54.2–-37.2)

Table S2: Cause-specific YLLs by US state in 2021 and percentage change between 1990 and 2021, Indiana						
Rank	Cause Name	2021 Percentage of all cause YLLs	2021 YLLs (millions)	2021 Age Standardised Rate (per 100 000)	Percentage change YLL count 1990 to 2021	Percentage change age-standardised YLL rate 1990 to 2021
	All causes	100.0 (100.0–100.0)	1.68 (1.49–1.88)	17811.8 (15914.7–19904.0)	49.6 (33.2–67.4)	3.5 (-7.3–15.4)
1	COVID-19	13.2 (11.5–15.7)	0.221 (0.213–0.257)	2325.1 (2242.4–2708.2)	--	--
2	Ischaemic heart disease	11.5 (10.4–12.2)	0.193 (0.163–0.223)	1713.0 (1447.4–1979.3)	-22.8 (-33.3—11.5)	-50.9 (-57.7—43.6)
3	Chronic obstructive pulmonary disease	5.9 (5.4–6.3)	0.0986 (0.0842–0.113)	821.3 (702.3–939.4)	136.6 (103.4–171.4)	45.0 (24.3–66.9)
4	Drug use disorders	5.6 (5.1–6.4)	0.0948 (0.0793–0.113)	1400.4 (1172.3–1662.3)	3114.5 (2437.7–3889.0)	2843.0 (2226.9–3546.1)
5	Tracheal, bronchus, and lung cancer	5.6 (5.1–6.0)	0.0940 (0.0789–0.110)	812.5 (682.5–950.4)	3.3 (-12.0–19.9)	-39.0 (-48.2—28.9)
6	Stroke	4.3 (3.8–4.6)	0.0714 (0.0610–0.0816)	630.8 (536.9–719.9)	15.2 (0.5–31.5)	-26.3 (-35.8—15.3)
7	Chronic kidney disease	3.5 (3.2–3.7)	0.0590 (0.0505–0.0671)	531.4 (455.7–604.5)	357.4 (295.4–419.8)	181.5 (143.7–220.2)
8	Self-harm	2.8 (2.6–3.1)	0.0472 (0.0401–0.0554)	676.6 (576.3–791.2)	30.3 (10.2–52.6)	13.0 (-4.5–32.4)
9	Alzheimer's disease and other dementias	2.7 (0.7–6.9)	0.0445 (0.0114–0.114)	351.1 (89.7–903.9)	62.4 (42.4–83.2)	-1.3 (-13.4–11.3)
10	Cirrhosis and other chronic liver diseases	2.5 (2.3–2.6)	0.0413 (0.0352–0.0478)	435.1 (369.0–502.9)	122.3 (90.5–160.7)	50.6 (29.0–76.2)
11	Road injuries	2.4 (2.2–2.5)	0.0397 (0.0344–0.0457)	588.8 (513.1–674.1)	-33.0 (-41.9—22.9)	-43.3 (-50.5—35.1)
12	Diabetes mellitus	2.2 (2.0–2.4)	0.0375 (0.0321–0.0433)	355.3 (303.0–409.8)	38.8 (18.4–59.7)	-8.5 (-22.1–5.2)
13	Colon and rectum cancer	2.2 (1.9–2.4)	0.0364 (0.0304–0.0432)	338.3 (283.0–401.1)	12.3 (-6.6–33.9)	-27.0 (-39.2—13.2)
14	Pancreatic cancer	1.6 (1.4–1.7)	0.0262 (0.0221–0.0302)	230.3 (194.9–265.8)	90.0 (63.7–122.0)	15.8 (-0.2–35.0)
15	Breast cancer	1.5 (1.3–1.8)	0.0254 (0.0207–0.0310)	247.9 (200.4–305.1)	-9.6 (-27.6–10.5)	-41.9 (-53.6—28.4)
16	Interpersonal violence	1.5 (1.3–1.6)	0.0244 (0.0212–0.0279)	402.5 (351.2–459.7)	11.2 (-3.8–29.1)	4.9 (-9.0–21.2)
17	Endocrine, metabolic, blood, and immune disorders	1.4 (1.3–1.5)	0.0237 (0.0205–0.0270)	259.5 (226.6–294.5)	241.4 (196.3–292.4)	123.0 (95.7–154.4)
18	Hypertensive heart disease	1.3 (1.2–1.5)	0.0220 (0.0184–0.0259)	209.4 (175.8–247.5)	193.7 (148.9–244.7)	97.1 (66.5–133.0)
19	Neonatal disorders	1.2 (1.0–1.4)	0.0204 (0.0180–0.0232)	524.5 (461.4–596.2)	-44.1 (-51.3—35.8)	-37.9 (-45.9—28.6)
20	Lower respiratory infections	1.2 (1.1–1.3)	0.0201 (0.0168–0.0235)	201.2 (170.2–234.0)	-26.4 (-38.4—13.5)	-50.1 (-57.7—41.6)
21	Congenital birth defects	1.0 (0.8–1.1)	0.0161 (0.0142–0.0177)	363.1 (319.4–400.5)	-28.0 (-36.8—19.0)	-27.2 (-36.3—17.7)
22	Cardiomyopathy and myocarditis	0.9 (0.8–1.0)	0.0157 (0.0133–0.0182)	169.1 (144.7–195.6)	-8.4 (-22.4–8.4)	-35.9 (-45.5—24.5)
23	Falls	0.9 (0.8–1.0)	0.0154 (0.0131–0.0178)	144.7 (123.7–166.7)	163.2 (130.2–202.1)	67.0 (46.1–91.6)
24	Prostate cancer	0.9 (0.7–1.0)	0.0149 (0.0119–0.0184)	121.7 (96.8–150.4)	9.9 (-13.2–36.8)	-32.2 (-46.5—15.7)
25	Leukaemia	0.9 (0.8–0.9)	0.0144 (0.0123–0.0164)	148.4 (128.4–168.1)	12.5 (-2.4–28.9)	-25.9 (-35.0—15.7)

Table S2: Cause-specific YLLs by US state in 2021 and percentage change between 1990 and 2021, Iowa

Rank	Cause Name	2021 Percentage of all cause YLLs	2021 YLLs (millions)	2021 Age Standardised Rate (per 100 000)	Percentage change YLL count 1990 to 2021	Percentage change age-standardised YLL rate 1990 to 2021
	All causes	100.0 (100.0–100.0)	0.662 (0.585–0.746)	13590.9 (11940.9–15307.4)	25.9 (10.4–42.3)	-4.1 (-16.3–8.1)
1	Ischaemic heart disease	14.0 (12.8–14.9)	0.0928 (0.0782–0.109)	1592.5 (1332.6–1864.8)	-28.1 (-38.6—16.6)	-46.3 (-54.6—37.4)
2	COVID-19	12.3 (10.9–13.9)	0.0813 (0.0813–0.0813)	1727.0 (1726.5–1727.6)	--	--
3	Tracheal, bronchus, and lung cancer	5.7 (5.2–6.2)	0.0378 (0.0314–0.0443)	657.9 (545.8–771.0)	0.3 (-16.8–16.8)	-33.3 (-44.9—22.0)
4	Chronic obstructive pulmonary disease	5.6 (5.0–6.0)	0.0369 (0.0315–0.0425)	593.5 (504.7–684.1)	79.8 (51.9–108.9)	26.6 (6.5–47.4)
5	Stroke	4.1 (3.6–4.4)	0.0273 (0.0229–0.0316)	458.2 (384.4–529.4)	-7.5 (-20.4–6.7)	-29.4 (-39.9—17.8)
6	Alzheimer's disease and other dementias	3.9 (1.0–10.0)	0.0259 (0.00672–0.0670)	361.4 (91.7–948.6)	35.1 (16.9–55.1)	0.3 (-13.5–14.9)
7	Self-harm	3.4 (3.0–3.7)	0.0223 (0.0184–0.0267)	699.1 (577.6–833.2)	48.2 (21.4–77.0)	36.6 (12.1–62.9)
8	Chronic kidney disease	3.1 (2.8–3.3)	0.0205 (0.0171–0.0240)	349.9 (293.9–407.6)	330.6 (267.9–394.3)	205.0 (158.7–252.2)
9	Road injuries	2.4 (2.3–2.6)	0.0162 (0.0136–0.0188)	517.4 (435.1–598.5)	-38.5 (-49.3—28.6)	-46.2 (-55.2—37.5)
10	Colon and rectum cancer	2.3 (2.1–2.6)	0.0154 (0.0125–0.0182)	284.0 (232.8–336.7)	-8.8 (-25.9–8.7)	-32.2 (-44.9—19.1)
11	Cirrhosis and other chronic liver diseases	2.2 (2.0–2.4)	0.0146 (0.0121–0.0172)	326.5 (269.3–385.8)	107.1 (71.1–147.3)	56.1 (28.9–85.4)
12	Drug use disorders	2.1 (1.9–2.4)	0.0141 (0.0114–0.0174)	451.2 (361.7–556.1)	1262.9 (967.9–1655.5)	1186.8 (907.0–1552.6)
13	Diabetes mellitus	2.0 (1.8–2.1)	0.0132 (0.0111–0.0154)	247.8 (207.7–289.5)	26.5 (6.4–49.3)	-5.6 (-20.9–11.6)
14	Pancreatic cancer	1.7 (1.6–1.8)	0.0114 (0.00968–0.0132)	201.9 (170.6–233.3)	77.1 (46.3–107.9)	25.9 (3.8–48.6)
15	Breast cancer	1.6 (1.3–1.9)	0.0107 (0.00845–0.0132)	213.4 (167.1–263.3)	-18.1 (-34.9–1.7)	-41.4 (-54.3—26.7)
16	Endocrine, metabolic, blood, and immune disorders	1.5 (1.4–1.6)	0.0102 (0.00867–0.0118)	222.5 (188.8–253.9)	256.9 (204.9–316.3)	146.0 (110.1–185.6)
17	Hypertensive heart disease	1.5 (1.4–1.7)	0.0101 (0.00844–0.0118)	184.7 (152.9–216.1)	145.2 (105.6–187.5)	86.3 (54.2–120.6)
18	Falls	1.5 (1.3–1.6)	0.0100 (0.00838–0.0117)	176.5 (149.3–205.8)	149.5 (112.6–193.7)	74.1 (47.0–106.5)
19	Lower respiratory infections	1.4 (1.2–1.5)	0.00916 (0.00751–0.0110)	168.0 (138.0–199.2)	-40.2 (-50.8—29.0)	-53.7 (-61.8—45.1)
20	Prostate cancer	1.1 (0.9–1.3)	0.00732 (0.00569–0.00908)	113.4 (88.2–141.3)	-4.0 (-26.2–21.5)	-31.0 (-47.1—12.4)
21	Congenital birth defects	1.0 (0.8–1.2)	0.00645 (0.00558–0.00751)	307.1 (262.4–362.9)	-35.0 (-44.8—22.6)	-34.8 (-45.2—21.1)
22	Leukaemia	1.0 (0.9–1.0)	0.00644 (0.00547–0.00741)	131.4 (112.0–150.3)	5.2 (-9.9–21.9)	-24.2 (-35.0—12.7)
23	Neonatal disorders	1.0 (0.8–1.2)	0.00636 (0.00528–0.00772)	351.1 (291.3–426.1)	-48.8 (-58.4—37.7)	-43.2 (-53.9—31.0)
24	Brain and central nervous system cancer	1.0 (0.9–1.0)	0.00635 (0.00537–0.00736)	148.9 (127.4–170.8)	29.2 (8.5–50.5)	-3.2 (-18.1–12.2)
25	Non-Hodgkin lymphoma	0.9 (0.8–1.0)	0.00591 (0.00500–0.00691)	107.5 (91.1–125.8)	-7.7 (-22.3–8.7)	-36.0 (-46.4—24.4)

Table S2: Cause-specific YLLs by US state in 2021 and percentage change between 1990 and 2021, Kansas

Rank	Cause Name	2021 Percentage of all cause YLLs	2021 YLLs (millions)	2021 Age Standardised Rate (per 100 000)	Percentage change YLL count 1990 to 2021	Percentage change age-standardised YLL rate 1990 to 2021
	All causes	100.0 (100.0–100.0)	0.653 (0.567–0.738)	15811.9 (13749.0–17727.5)	40.1 (22.0–58.9)	3.7 (-9.2–16.8)
1	COVID-19	14.2 (12.5–16.3)	0.0925 (0.0924–0.0924)	2256.0 (2254.0–2255.4)	--	--
2	Ischaemic heart disease	12.2 (11.2–13.0)	0.0799 (0.0674–0.0928)	1612.8 (1351.6–1890.8)	-21.0 (-33.6–-7.7)	-44.4 (-53.6–-34.6)
3	Chronic obstructive pulmonary disease	5.6 (5.1–5.9)	0.0364 (0.0307–0.0424)	697.9 (590.3–813.4)	96.9 (67.1–126.7)	33.5 (12.9–54.2)
4	Tracheal, bronchus, and lung cancer	5.3 (4.8–5.8)	0.0347 (0.0291–0.0409)	705.0 (589.1–831.3)	1.2 (-15.4–19.7)	-36.2 (-46.9–-24.4)
5	Stroke	4.0 (3.6–4.3)	0.0262 (0.0223–0.0306)	525.1 (447.0–612.7)	1.7 (-13.7–17.5)	-26.6 (-37.8–-14.6)
6	Self-harm	3.6 (3.4–4.0)	0.0238 (0.0195–0.0281)	810.7 (665.1–955.4)	61.5 (31.5–93.4)	44.3 (18.1–72.6)
7	Chronic kidney disease	3.3 (3.0–3.5)	0.0213 (0.0181–0.0249)	439.9 (374.2–511.6)	342.5 (278.7–413.2)	206.7 (161.9–256.5)
8	Alzheimer's disease and other dementias	3.1 (0.8–8.0)	0.0206 (0.00525–0.0535)	352.7 (89.8–928.7)	37.8 (18.4–55.8)	-1.4 (-15.2–11.0)
9	Road injuries	2.8 (2.6–3.0)	0.0184 (0.0153–0.0216)	636.2 (532.8–742.0)	-28.5 (-40.5–-15.5)	-39.3 (-49.4–-28.5)
10	Drug use disorders	2.6 (2.2–3.2)	0.0173 (0.0134–0.0220)	591.3 (458.8–751.4)	1302.4 (984.4–1748.9)	1191.8 (898.0–1598.8)
11	Cirrhosis and other chronic liver diseases	2.5 (2.3–2.7)	0.0161 (0.0132–0.0191)	403.5 (331.2–480.3)	122.7 (80.3–165.1)	58.1 (28.6–88.5)
12	Diabetes mellitus	2.3 (2.1–2.4)	0.0147 (0.0123–0.0172)	322.8 (268.1–378.4)	49.4 (22.9–75.6)	5.4 (-13.7–24.9)
13	Colon and rectum cancer	2.1 (1.9–2.3)	0.0138 (0.0114–0.0163)	298.6 (247.1–356.1)	2.2 (-15.2–23.2)	-27.0 (-39.7–-11.8)
14	Breast cancer	1.7 (1.4–2.0)	0.0109 (0.00869–0.0135)	246.9 (197.3–307.8)	-2.8 (-22.3–20.4)	-33.7 (-47.3–-17.3)
15	Pancreatic cancer	1.6 (1.5–1.7)	0.0106 (0.00876–0.0124)	217.6 (180.0–255.9)	82.9 (50.8–116.9)	21.9 (0.0–44.8)
16	Lower respiratory infections	1.4 (1.2–1.5)	0.00912 (0.00739–0.0110)	201.5 (166.4–240.7)	-31.8 (-44.5–-18.8)	-49.1 (-58.5–-39.3)
17	Endocrine, metabolic, blood, and immune disorders	1.4 (1.3–1.4)	0.00887 (0.00743–0.0103)	226.4 (190.6–260.2)	237.3 (181.8–293.3)	135.1 (98.3–173.0)
18	Neonatal disorders	1.3 (1.0–1.6)	0.00857 (0.00711–0.0103)	518.1 (429.7–621.7)	-33.2 (-46.2–-18.6)	-21.3 (-36.7–-4.1)
19	Falls	1.3 (1.2–1.4)	0.00850 (0.00717–0.0100)	178.1 (150.0–209.3)	190.6 (145.9–237.4)	95.9 (64.6–128.5)
20	Hypertensive heart disease	1.3 (1.1–1.4)	0.00823 (0.00692–0.00960)	174.4 (145.5–203.9)	178.1 (131.8–224.1)	101.4 (66.3–136.5)
21	Interpersonal violence	1.1 (1.0–1.2)	0.00717 (0.00594–0.00843)	271.0 (226.8–317.4)	-9.0 (-24.1–7.4)	-15.3 (-28.9–-0.4)
22	Congenital birth defects	1.0 (0.9–1.2)	0.00668 (0.00577–0.00768)	355.4 (302.6–413.4)	-31.1 (-41.8–-19.4)	-26.2 (-38.3–-12.2)
23	Leukaemia	0.9 (0.9–1.0)	0.00611 (0.00514–0.00704)	144.8 (123.2–164.8)	7.2 (-9.9–24.1)	-24.6 (-36.6–-13.4)
24	Prostate cancer	0.9 (0.8–1.1)	0.00598 (0.00474–0.00743)	111.1 (87.5–138.4)	-4.9 (-25.8–20.2)	-33.9 (-48.6–-16.4)
25	Brain and central nervous system cancer	0.9 (0.9–1.0)	0.00595 (0.00499–0.00693)	156.1 (131.8–181.0)	37.1 (15.0–59.8)	-1.8 (-16.8–14.2)

Table S2: Cause-specific YLLs by US state in 2021 and percentage change between 1990 and 2021, Kentucky						
Rank	Cause Name	2021 Percentage of all cause YLLs	2021 YLLs (millions)	2021 Age Standardised Rate (per 100 000)	Percentage change YLL count 1990 to 2021	Percentage change age-standardised YLL rate 1990 to 2021
	All causes	100.0 (100.0–100.0)	1.34 (1.18–1.50)	21030.1 (18638.1–23499.4)	65.5 (46.1–87.0)	13.1 (0.0–27.2)
1	COVID-19	15.4 (13.4–17.9)	0.205 (0.199–0.234)	3198.2 (3103.2–3648.6)	--	--
2	Ischaemic heart disease	11.5 (10.6–12.3)	0.154 (0.129–0.180)	2058.3 (1720.6–2407.7)	-15.6 (-27.7–-2.3)	-46.4 (-54.2–-37.6)
3	Tracheal, bronchus, and lung cancer	6.1 (5.5–6.7)	0.0813 (0.0687–0.0960)	1035.6 (874.0–1223.1)	8.0 (-9.8–27.2)	-37.6 (-47.9–-26.5)
4	Drug use disorders	6.1 (5.2–7.1)	0.0811 (0.0655–0.100)	1792.4 (1453.9–2194.1)	2483.0 (1922.5–3310.2)	2250.4 (1760.7–2985.0)
5	Chronic obstructive pulmonary disease	6.0 (5.5–6.5)	0.0809 (0.0689–0.0938)	1000.3 (850.6–1161.5)	143.0 (108.8–181.3)	46.0 (25.4–69.0)
6	Stroke	3.9 (3.5–4.3)	0.0526 (0.0442–0.0615)	696.6 (587.0–815.9)	23.4 (6.2–42.6)	-21.1 (-32.2–-8.8)
7	Chronic kidney disease	3.2 (2.9–3.5)	0.0434 (0.0365–0.0500)	580.6 (493.2–668.3)	373.5 (303.7–450.2)	188.8 (147.8–233.5)
8	Self-harm	2.7 (2.4–2.9)	0.0357 (0.0302–0.0419)	772.6 (656.9–905.1)	45.7 (21.8–71.6)	28.0 (7.6–50.4)
9	Cirrhosis and other chronic liver diseases	2.6 (2.4–2.8)	0.0346 (0.0291–0.0406)	537.1 (451.5–630.7)	146.4 (107.2–189.9)	64.4 (37.9–94.2)
10	Road injuries	2.5 (2.3–2.7)	0.0336 (0.0282–0.0390)	752.9 (630.8–868.4)	-29.0 (-40.3–-17.3)	-39.6 (-49.1–-29.8)
11	Diabetes mellitus	2.2 (2.1–2.4)	0.0298 (0.0252–0.0350)	423.6 (360.3–498.3)	59.3 (36.2–85.2)	5.3 (-10.1–23.1)
12	Alzheimer's disease and other dementias	2.2 (0.6–5.7)	0.0294 (0.00773–0.0764)	347.1 (91.2–895.6)	62.7 (45.3–85.8)	-2.5 (-13.1–11.3)
13	Colon and rectum cancer	2.1 (1.9–2.4)	0.0283 (0.0234–0.0337)	388.5 (323.6–463.8)	22.1 (-0.2–44.2)	-21.7 (-35.7–-6.8)
14	Hypertensive heart disease	1.6 (1.4–1.8)	0.0211 (0.0178–0.0253)	304.5 (256.8–365.4)	252.1 (196.3–321.9)	137.4 (100.2–185.1)
15	Breast cancer	1.5 (1.2–1.8)	0.0196 (0.0160–0.0241)	283.4 (228.2–351.2)	4.7 (-14.6–29.6)	-33.5 (-46.1–-16.2)
16	Pancreatic cancer	1.4 (1.3–1.5)	0.0188 (0.0158–0.0218)	244.6 (205.3–284.2)	101.0 (70.6–134.9)	22.0 (3.5–42.6)
17	Lower respiratory infections	1.4 (1.2–1.5)	0.0185 (0.0151–0.0221)	271.1 (224.4–321.3)	-20.0 (-33.0–-5.2)	-46.9 (-55.4–-37.4)
18	Endocrine, metabolic, blood, and immune disorders	1.2 (1.1–1.3)	0.0165 (0.0140–0.0191)	271.6 (233.7–311.7)	270.3 (216.9–327.0)	142.8 (109.2–178.5)
19	Interpersonal violence	1.0 (0.9–1.1)	0.0138 (0.0117–0.0162)	343.5 (292.9–402.2)	-4.4 (-19.6–12.9)	-9.0 (-23.2–7.0)
20	Falls	0.9 (0.8–1.0)	0.0122 (0.0103–0.0141)	170.9 (145.7–198.6)	170.7 (133.6–215.9)	69.8 (46.6–98.8)
21	Neonatal disorders	0.9 (0.7–1.1)	0.0121 (0.00973–0.0150)	479.5 (385.5–593.1)	-32.7 (-46.5–-15.6)	-28.2 (-42.9–-9.9)
22	Leukaemia	0.8 (0.7–0.8)	0.0106 (0.00909–0.0123)	161.5 (139.2–185.0)	21.0 (3.6–40.6)	-21.3 (-32.3–-8.5)
23	Congenital birth defects	0.8 (0.7–0.9)	0.0104 (0.00885–0.0122)	354.5 (296.5–418.1)	-30.8 (-42.6–-18.4)	-33.4 (-45.1–-19.9)
24	Alcohol use disorders	0.8 (0.7–0.8)	0.0102 (0.00854–0.0120)	187.8 (157.9–220.9)	170.7 (127.8–222.2)	107.8 (75.3–146.6)
25	Prostate cancer	0.7 (0.6–0.8)	0.00975 (0.00775–0.0120)	117.2 (93.1–144.4)	4.0 (-18.9–31.5)	-36.6 (-50.6–-19.7)

Table S2: Cause-specific YLLs by US state in 2021 and percentage change between 1990 and 2021, Louisiana						
Rank	Cause Name	2021 Percentage of all cause YLLs	2021 YLLs (millions)	2021 Age Standardised Rate (per 100 000)	Percentage change YLL count 1990 to 2021	Percentage change age-standardised YLL rate 1990 to 2021
	All causes	100.0 (100.0–100.0)	1.32 (1.17–1.49)	21285.9 (18985.7–23961.6)	36.7 (21.2–54.7)	0.9 (-10.1–13.6)
1	COVID-19	15.6 (12.3–19.5)	0.205 (0.173–0.255)	3243.4 (2734.8–4022.1)	--	--
2	Ischaemic heart disease	11.5 (10.6–12.3)	0.151 (0.130–0.174)	2069.1 (1776.7–2389.4)	-20.8 (-31.7—8.6)	-47.6 (-54.9—39.4)
3	Tracheal, bronchus, and lung cancer	5.1 (4.6–5.5)	0.0668 (0.0572–0.0784)	874.9 (747.8–1028.7)	-8.4 (-22.3–9.7)	-43.3 (-52.0—32.0)
4	Drug use disorders	4.5 (3.7–5.4)	0.0591 (0.0466–0.0734)	1242.4 (981.8–1538.7)	1499.8 (1134.4–1971.4)	1455.8 (1105.3–1900.7)
5	Chronic obstructive pulmonary disease	4.3 (3.9–4.6)	0.0563 (0.0483–0.0650)	716.7 (614.7–829.5)	120.1 (88.0–154.2)	40.2 (19.8–62.3)
6	Stroke	4.1 (3.7–4.5)	0.0545 (0.0470–0.0632)	748.5 (644.7–868.6)	12.8 (-1.1–30.2)	-25.3 (-34.8—13.4)
7	Chronic kidney disease	3.7 (3.4–4.0)	0.0486 (0.0421–0.0563)	683.5 (593.3–790.8)	253.1 (207.2–308.3)	134.1 (103.0–170.5)
8	Road injuries	2.8 (2.6–3.0)	0.0366 (0.0315–0.0427)	802.7 (696.0–931.0)	-33.7 (-42.9—22.4)	-36.6 (-45.0—26.3)
9	Self-harm	2.6 (2.4–2.9)	0.0345 (0.0293–0.0409)	727.0 (618.7–856.6)	24.9 (5.2–47.5)	18.7 (0.2–40.1)
10	Interpersonal violence	2.6 (2.3–2.8)	0.0340 (0.0290–0.0394)	813.0 (700.2–940.5)	-24.0 (-35.3—11.5)	-20.0 (-31.3—7.0)
11	Hypertensive heart disease	2.3 (2.0–2.6)	0.0304 (0.0254–0.0364)	451.5 (380.7–540.0)	114.7 (77.6–157.4)	51.2 (25.1–81.0)
12	Diabetes mellitus	2.3 (2.1–2.5)	0.0304 (0.0260–0.0351)	440.6 (377.6–508.5)	9.7 (-7.6–27.1)	-23.6 (-35.2—11.4)
13	Colon and rectum cancer	2.2 (2.0–2.4)	0.0287 (0.0239–0.0339)	403.6 (336.5–477.8)	22.2 (2.3–45.1)	-17.2 (-30.7—1.4)
14	Cirrhosis and other chronic liver diseases	2.2 (2.0–2.3)	0.0285 (0.0243–0.0335)	443.7 (377.1–522.3)	54.9 (31.8–83.1)	9.6 (-7.0–30.1)
15	Alzheimer's disease and other dementias	2.1 (0.5–5.6)	0.0278 (0.00713–0.0726)	348.9 (89.4–912.4)	57.8 (40.5–81.6)	-1.8 (-12.9–12.4)
16	Pancreatic cancer	1.6 (1.5–1.7)	0.0208 (0.0179–0.0243)	279.1 (239.5–325.8)	77.5 (52.9–106.6)	14.5 (-1.4–33.5)
17	Breast cancer	1.6 (1.3–1.8)	0.0208 (0.0169–0.0252)	305.6 (246.4–371.7)	-6.0 (-25.1–16.2)	-36.1 (-49.2—20.6)
18	Neonatal disorders	1.3 (1.1–1.5)	0.0167 (0.0146–0.0186)	593.0 (517.2–660.5)	-53.7 (-59.8—47.5)	-42.6 (-50.2—34.9)
19	Endocrine, metabolic, blood, and immune disorders	1.2 (1.1–1.3)	0.0161 (0.0139–0.0188)	274.3 (238.1–317.4)	160.8 (125.3–205.6)	94.2 (69.9–126.9)
20	Lower respiratory infections	1.2 (1.1–1.3)	0.0156 (0.0129–0.0183)	240.0 (202.4–279.5)	-26.2 (-38.2—12.4)	-47.5 (-55.5—38.0)
21	Congenital birth defects	0.9 (0.8–1.0)	0.0121 (0.0106–0.0134)	381.7 (335.2–429.1)	-42.9 (-50.5—35.1)	-34.8 (-43.3—25.5)
22	Prostate cancer	0.8 (0.7–1.0)	0.0111 (0.00873–0.0138)	139.3 (109.5–173.4)	-2.7 (-23.7–20.0)	-37.7 (-51.1—23.2)
23	Cardiomyopathy and myocarditis	0.8 (0.8–0.9)	0.0110 (0.00944–0.0128)	181.3 (155.7–210.8)	-27.5 (-37.9—15.0)	-44.8 (-52.6—35.6)
24	Leukaemia	0.8 (0.7–0.8)	0.0102 (0.00880–0.0116)	159.9 (138.8–182.9)	-2.2 (-16.4–12.8)	-29.5 (-39.1—19.3)
25	Falls	0.7 (0.7–0.8)	0.00954 (0.00828–0.0110)	140.4 (122.1–162.0)	112.9 (82.8–145.1)	46.6 (26.1–68.8)

Table S2: Cause-specific YLLs by US state in 2021 and percentage change between 1990 and 2021, Maine

Rank	Cause Name	2021 Percentage of all cause YLLs	2021 YLLs (millions)	2021 Age Standardised Rate (per 100 000)	Percentage change YLL count 1990 to 2021	Percentage change age-standardised YLL rate 1990 to 2021
	All causes	100.0 (100.0–100.0)	0.336 (0.295–0.382)	14973.6 (13145.6–17048.6)	44.1 (25.8–63.6)	-2.5 (-14.7–10.3)
1	Ischaemic heart disease	11.0 (9.9–11.9)	0.0371 (0.0314–0.0436)	1266.4 (1063.8–1483.6)	-29.5 (-40.1—18.6)	-59.3 (-65.5—52.6)
2	COVID-19	9.2 (6.7–12.3)	0.0310 (0.0237–0.0417)	1372.6 (1049.3–1843.5)	--	--
3	Tracheal, bronchus, and lung cancer	6.7 (6.0–7.4)	0.0225 (0.0189–0.0265)	774.4 (650.3–912.0)	11.7 (-8.0–32.0)	-40.0 (-50.6—28.6)
4	Chronic obstructive pulmonary disease	6.0 (5.5–6.5)	0.0203 (0.0174–0.0234)	646.7 (557.9–746.0)	108.4 (81.0–138.4)	15.5 (0.0–33.0)
5	Drug use disorders	4.9 (4.3–5.6)	0.0164 (0.0136–0.0201)	1282.0 (1063.1–1551.9)	1820.6 (1439.2–2307.6)	1959.3 (1560.9–2467.9)
6	Stroke	3.8 (3.4–4.2)	0.0129 (0.0110–0.0149)	445.0 (376.7–513.0)	11.7 (-2.6–27.9)	-33.4 (-42.3—22.9)
7	Alzheimer's disease and other dementias	3.6 (0.9–9.3)	0.0122 (0.00303–0.0310)	353.9 (86.9–899.7)	79.4 (59.0–102.4)	-1.4 (-12.8–11.8)
8	Self-harm	3.3 (3.0–3.6)	0.0110 (0.00912–0.0131)	805.6 (673.3–958.2)	41.7 (16.1–70.6)	38.7 (14.8–66.1)
9	Chronic kidney disease	3.1 (2.8–3.4)	0.0104 (0.00888–0.0120)	370.5 (319.8–424.5)	345.0 (284.9–407.3)	160.1 (123.9–197.9)
10	Cirrhosis and other chronic liver diseases	2.6 (2.4–2.8)	0.00868 (0.00723–0.0101)	398.3 (330.9–462.7)	104.5 (72.4–138.1)	38.3 (16.1–61.3)
11	Diabetes mellitus	2.4 (2.2–2.6)	0.00810 (0.00683–0.00953)	311.3 (264.1–367.5)	51.1 (27.8–78.1)	-6.1 (-20.6–10.5)
12	Colon and rectum cancer	2.3 (2.0–2.5)	0.00759 (0.00636–0.00901)	285.9 (238.7–338.8)	1.8 (-16.1–21.1)	-37.1 (-48.5—25.1)
13	Road injuries	2.0 (1.9–2.2)	0.00680 (0.00580–0.00793)	527.3 (449.7–610.5)	-39.5 (-48.8—29.5)	-42.2 (-50.8—33.3)
14	Pancreatic cancer	1.8 (1.6–1.9)	0.00606 (0.00515–0.00711)	214.0 (182.1–250.3)	90.8 (61.4–123.0)	8.9 (-8.0–27.9)
15	Endocrine, metabolic, blood, and immune disorders	1.7 (1.6–1.9)	0.00584 (0.00503–0.00674)	275.2 (237.0–317.0)	274.7 (223.4–337.3)	141.3 (108.1–179.6)
16	Breast cancer	1.6 (1.4–2.0)	0.00553 (0.00454–0.00665)	225.4 (183.9–272.7)	-7.0 (-23.7–13.3)	-42.2 (-53.0—29.2)
17	Falls	1.6 (1.4–1.7)	0.00523 (0.00445–0.00601)	187.3 (160.9–215.3)	272.3 (222.7–323.9)	107.7 (79.7–137.0)
18	Hypertensive heart disease	1.4 (1.2–1.5)	0.00469 (0.00395–0.00547)	181.0 (153.0–213.1)	216.2 (170.8–267.8)	101.8 (73.0–136.9)
19	Lower respiratory infections	1.4 (1.2–1.5)	0.00455 (0.00367–0.00543)	173.9 (143.5–204.8)	-21.5 (-34.5—8.2)	-51.1 (-58.7—42.5)
20	Prostate cancer	1.1 (0.9–1.3)	0.00366 (0.00287–0.00465)	112.7 (88.5–143.0)	9.7 (-14.1–40.6)	-39.8 (-53.0—23.0)
21	Alcohol use disorders	1.0 (0.9–1.1)	0.00347 (0.00287–0.00416)	201.1 (167.7–239.7)	146.7 (103.7–198.8)	101.4 (67.5–140.6)
22	Oesophageal cancer	1.0 (0.8–1.1)	0.00320 (0.00258–0.00391)	117.5 (94.8–144.4)	101.4 (64.5–149.2)	15.9 (-5.8–43.8)
23	Leukaemia	0.9 (0.8–1.0)	0.00312 (0.00267–0.00363)	131.1 (113.4–151.3)	17.4 (-0.6–36.9)	-27.1 (-37.7—15.6)
24	Brain and central nervous system cancer	0.9 (0.8–1.0)	0.00302 (0.00259–0.00352)	146.2 (126.1–169.8)	45.5 (22.3–69.4)	-2.2 (-17.6–13.8)
25	Parkinson's disease	0.9 (0.8–1.0)	0.00301 (0.00257–0.00351)	90.6 (77.2–105.7)	168.9 (134.2–207.4)	50.3 (30.6–72.2)

Table S2: Cause-specific YLLs by US state in 2021 and percentage change between 1990 and 2021, Maryland						
Rank	Cause Name	2021 Percentage of all cause YLLs	2021 YLLs (millions)	2021 Age Standardised Rate (per 100 000)	Percentage change YLL count 1990 to 2021	Percentage change age-standardised YLL rate 1990 to 2021
	All causes	100.0 (100.0–100.0)	1.26 (1.09–1.43)	14461.1 (12620.3–16412.4)	31.0 (13.3–49.5)	-20.6 (-30.8—9.7)
1	Ischaemic heart disease	11.7 (10.6–12.5)	0.147 (0.121–0.173)	1371.6 (1132.3–1611.6)	-14.5 (-27.5—1.2)	-56.3 (-63.2—49.3)
2	COVID-19	9.8 (8.6–11.6)	0.123 (0.122–0.134)	1381.5 (1368.9–1509.8)	--	--
3	Tracheal, bronchus, and lung cancer	4.9 (4.4–5.3)	0.0611 (0.0512–0.0720)	572.5 (478.5–675.3)	-20.3 (-33.0—6.0)	-59.3 (-65.8—51.9)
4	Drug use disorders	4.8 (4.1–5.6)	0.0602 (0.0472–0.0743)	937.3 (738.6–1154.4)	1795.2 (1349.8–2334.8)	1577.0 (1184.0–2036.8)
5	Stroke	4.6 (4.0–5.0)	0.0580 (0.0479–0.0677)	540.3 (451.3–630.0)	34.8 (13.8–57.6)	-31.3 (-42.2—19.9)
6	Self-harm	4.3 (3.9–4.6)	0.0537 (0.0440–0.0636)	805.7 (663.3–949.9)	66.5 (36.5–100.3)	35.7 (12.0–62.3)
7	Chronic obstructive pulmonary disease	3.7 (3.3–3.9)	0.0459 (0.0382–0.0539)	411.8 (342.9–482.9)	66.0 (41.1–92.0)	-16.7 (-29.2—3.4)
8	Alzheimer's disease and other dementias	3.4 (0.9–8.6)	0.0427 (0.0111–0.107)	348.0 (91.5–879.0)	120.8 (94.0–157.8)	-3.7 (-15.7–10.8)
9	Chronic kidney disease	3.3 (3.0–3.6)	0.0411 (0.0341–0.0483)	405.9 (338.0–473.7)	237.3 (186.6–293.8)	78.8 (52.7–107.4)
10	Interpersonal violence	2.7 (2.5–2.9)	0.0340 (0.0279–0.0393)	605.7 (500.7–700.0)	-15.0 (-30.4—0.2)	-23.3 (-36.8—10.0)
11	Hypertensive heart disease	2.6 (2.4–2.9)	0.0328 (0.0267–0.0390)	339.6 (278.5–404.5)	144.4 (100.1–191.4)	37.3 (12.4–63.5)
12	Colon and rectum cancer	2.4 (2.1–2.6)	0.0296 (0.0244–0.0351)	299.1 (247.1–355.1)	3.0 (-15.0–23.4)	-42.7 (-52.7—31.2)
13	Diabetes mellitus	2.2 (2.1–2.4)	0.0281 (0.0234–0.0330)	289.7 (243.3–341.6)	18.2 (-0.8–39.2)	-32.7 (-43.7—20.3)
14	Cirrhosis and other chronic liver diseases	2.2 (2.0–2.3)	0.0274 (0.0229–0.0325)	306.3 (256.5–364.1)	37.0 (14.1–63.5)	-16.0 (-29.7–0.4)
15	Road injuries	2.1 (2.0–2.2)	0.0264 (0.0223–0.0306)	431.7 (364.4–495.1)	-38.7 (-48.6—28.8)	-50.4 (-58.0—42.8)
16	Breast cancer	1.9 (1.5–2.2)	0.0233 (0.0186–0.0285)	245.2 (195.7–301.8)	-9.4 (-28.1–12.3)	-47.8 (-58.7—34.8)
17	Pancreatic cancer	1.8 (1.6–1.9)	0.0221 (0.0186–0.0259)	211.1 (177.4–247.9)	85.5 (55.1–118.0)	-2.6 (-18.4–14.6)
18	Neonatal disorders	1.5 (1.3–1.8)	0.0191 (0.0168–0.0214)	579.0 (508.9–647.5)	-49.9 (-56.4—42.2)	-40.2 (-47.9—30.9)
19	Endocrine, metabolic, blood, and immune disorders	1.5 (1.4–1.6)	0.0189 (0.0159–0.0219)	226.5 (192.7–259.3)	203.8 (158.0–252.0)	84.3 (58.0–113.1)
20	Cardiomyopathy and myocarditis	1.3 (1.2–1.5)	0.0166 (0.0137–0.0198)	187.9 (157.3–221.5)	-21.3 (-35.6—6.9)	-52.2 (-60.4—43.2)
21	Lower respiratory infections	1.3 (1.1–1.4)	0.0162 (0.0131–0.0195)	173.4 (142.4–206.0)	-30.2 (-43.0—16.3)	-60.6 (-67.4—53.4)
22	Falls	1.2 (1.1–1.3)	0.0150 (0.0123–0.0174)	149.6 (124.7–173.7)	166.6 (122.5–207.6)	42.0 (20.1–65.3)
23	Prostate cancer	1.1 (0.9–1.2)	0.0133 (0.0105–0.0165)	118.1 (93.1–146.7)	6.8 (-15.4–34.4)	-46.5 (-57.8—32.8)
24	Leukaemia	0.9 (0.9–1.0)	0.0115 (0.00982–0.0134)	129.1 (111.1–149.5)	6.6 (-9.2–23.3)	-37.4 (-46.3—27.8)
25	Liver cancer	0.8 (0.8–0.9)	0.0106 (0.00886–0.0125)	105.6 (88.8–125.0)	247.7 (184.1–312.6)	87.9 (54.1–123.1)

Table S2: Cause-specific YLLs by US state in 2021 and percentage change between 1990 and 2021, Massachusetts

Rank	Cause Name	2021 Percentage of all cause YLLs	2021 YLLs (millions)	2021 Age Standardised Rate (per 100 000)	Percentage change YLL count 1990 to 2021	Percentage change age-standardised YLL rate 1990 to 2021
	All causes	100.0 (100.0–100.0)	1.19 (1.03–1.36)	10930.5 (9427.6–12465.5)	4.5 (-10.1–19.5)	-28.1 (-38.0—18.0)
1	COVID-19	11.1 (9.6–12.8)	0.131 (0.131–0.131)	1233.2 (1232.9–1233.6)	--	--
2	Ischaemic heart disease	11.0 (9.8–11.7)	0.131 (0.108–0.153)	988.7 (821.5–1169.1)	-47.6 (-55.9—38.8)	-67.1 (-72.5—61.3)
3	Drug use disorders	7.3 (6.5–8.1)	0.0870 (0.0708–0.105)	1166.9 (942.0–1411.4)	2219.7 (1675.5–2857.8)	2086.3 (1575.5–2670.6)
4	Tracheal, bronchus, and lung cancer	5.9 (5.2–6.4)	0.0697 (0.0572–0.0816)	532.2 (438.3–623.9)	-21.7 (-36.2—7.4)	-54.6 (-63.1—46.2)
5	Chronic obstructive pulmonary disease	4.8 (4.2–5.1)	0.0566 (0.0469–0.0658)	412.2 (342.3–480.6)	52.5 (30.6–78.4)	-4.9 (-18.9–11.9)
6	Alzheimer's disease and other dementias	4.4 (1.1–10.8)	0.0528 (0.0137–0.132)	354.9 (91.9–894.5)	53.7 (35.0–76.8)	-2.2 (-14.3–12.6)
7	Stroke	3.9 (3.4–4.3)	0.0469 (0.0383–0.0545)	350.2 (289.3–407.5)	-14.6 (-27.7—1.1)	-45.9 (-54.3—37.0)
8	Chronic kidney disease	3.4 (3.0–3.7)	0.0401 (0.0328–0.0472)	311.7 (257.3–366.1)	217.5 (166.1–269.9)	99.1 (67.3–132.6)
9	Cirrhosis and other chronic liver diseases	2.5 (2.3–2.7)	0.0298 (0.0244–0.0358)	277.5 (226.7–334.0)	10.0 (-10.3–31.6)	-26.8 (-40.6—12.7)
10	Colon and rectum cancer	2.3 (2.0–2.6)	0.0277 (0.0228–0.0327)	229.8 (188.9–271.3)	-30.8 (-43.7—16.4)	-53.9 (-62.8—43.9)
11	Self-harm	2.3 (2.1–2.5)	0.0277 (0.0226–0.0336)	356.5 (292.4–432.2)	-21.4 (-36.3—4.2)	-30.6 (-43.7—15.5)
12	Diabetes mellitus	2.0 (1.8–2.2)	0.0243 (0.0200–0.0287)	201.1 (165.4–239.6)	-4.8 (-21.5–12.6)	-36.9 (-48.1—25.0)
13	Pancreatic cancer	2.0 (1.8–2.1)	0.0237 (0.0196–0.0279)	184.2 (152.1–217.4)	46.2 (21.5–71.4)	-10.4 (-25.8–5.5)
14	Breast cancer	1.7 (1.4–2.1)	0.0204 (0.0158–0.0253)	175.2 (134.1–217.3)	-40.7 (-54.2—26.8)	-62.4 (-71.3—53.2)
15	Lower respiratory infections	1.6 (1.4–1.8)	0.0196 (0.0156–0.0239)	159.8 (127.4–193.6)	-43.7 (-54.9—31.3)	-62.9 (-70.3—54.8)
16	Road injuries	1.4 (1.3–1.5)	0.0169 (0.0138–0.0205)	229.9 (189.1–277.4)	-58.0 (-65.5—49.3)	-64.2 (-70.3—57.0)
17	Falls	1.4 (1.2–1.5)	0.0164 (0.0135–0.0194)	128.2 (106.3–151.8)	139.3 (102.1–182.5)	44.9 (21.9–70.5)
18	Endocrine, metabolic, blood, and immune disorders	1.3 (1.2–1.4)	0.0154 (0.0129–0.0182)	147.1 (123.4–172.9)	153.3 (110.4–197.6)	62.5 (35.4–90.4)
19	Hypertensive heart disease	1.2 (1.0–1.3)	0.0141 (0.0116–0.0166)	115.5 (95.6–137.1)	74.1 (46.7–105.9)	12.9 (-6.3–34.3)
20	Alcohol use disorders	1.1 (1.0–1.2)	0.0136 (0.0110–0.0166)	150.8 (123.0–184.0)	133.3 (89.4–184.8)	78.2 (44.3–118.3)
21	Prostate cancer	1.1 (0.9–1.3)	0.0135 (0.0105–0.0168)	97.3 (75.4–121.0)	-14.4 (-34.1–9.9)	-45.8 (-58.4—30.5)
22	Liver cancer	1.1 (1.0–1.2)	0.0130 (0.0106–0.0161)	106.7 (86.7–131.5)	236.9 (170.5–316.7)	107.6 (67.7–155.9)
23	Brain and central nervous system cancer	1.0 (1.0–1.1)	0.0121 (0.0101–0.0144)	118.6 (99.6–140.3)	15.3 (-5.1–37.9)	-23.9 (-37.0—9.5)
24	Neonatal disorders	1.0 (0.8–1.3)	0.0120 (0.0101–0.0142)	338.7 (284.1–400.5)	-61.0 (-67.9—52.8)	-50.7 (-59.4—40.3)
25	Leukaemia	1.0 (0.9–1.0)	0.0116 (0.00963–0.0137)	102.7 (85.7–119.5)	-12.7 (-27.1–3.8)	-44.4 (-53.2—34.5)

Table S2: Cause-specific YLLs by US state in 2021 and percentage change between 1990 and 2021, Michigan						
Rank	Cause Name	2021 Percentage of all cause YLLs	2021 YLLs (millions)	2021 Age Standardised Rate (per 100 000)	Percentage change YLL count 1990 to 2021	Percentage change age-standardised YLL rate 1990 to 2021
	All causes	100.0 (100.0–100.0)	2.43 (2.15–2.73)	16147.3 (14323.2–18035.7)	29.1 (14.0–45.6)	-9.3 (-19.7–1.5)
1	COVID-19	14.3 (12.7–16.2)	0.347 (0.344–0.383)	2295.3 (2274.3–2534.2)	--	--
2	Ischaemic heart disease	13.2 (12.0–14.1)	0.322 (0.274–0.374)	1718.8 (1469.0–2000.8)	-24.5 (-34.8–-13.3)	-54.0 (-60.3–-46.6)
3	Tracheal, bronchus, and lung cancer	5.4 (4.9–5.9)	0.132 (0.111–0.155)	695.8 (585.5–815.9)	-2.0 (-17.9–16.6)	-43.1 (-52.4–-32.1)
4	Chronic obstructive pulmonary disease	4.9 (4.4–5.2)	0.118 (0.100–0.136)	589.5 (498.2–678.9)	105.4 (76.1–136.2)	20.2 (3.0–38.2)
5	Drug use disorders	4.8 (4.0–5.5)	0.116 (0.0935–0.142)	1166.1 (949.6–1413.7)	1011.2 (767.4–1322.5)	1082.0 (828.2–1396.8)
6	Stroke	4.0 (3.5–4.3)	0.0962 (0.0818–0.110)	510.1 (437.0–582.3)	7.5 (-6.9–22.8)	-35.0 (-43.9–-25.2)
7	Alzheimer's disease and other dementias	3.1 (0.8–7.9)	0.0760 (0.0191–0.198)	349.9 (88.0–913.8)	80.7 (58.8–105.8)	-1.8 (-13.1–11.2)
8	Chronic kidney disease	3.1 (2.8–3.3)	0.0754 (0.0638–0.0870)	416.0 (353.9–477.2)	296.9 (242.3–357.6)	140.0 (108.3–175.8)
9	Self-harm	2.9 (2.7–3.2)	0.0711 (0.0603–0.0838)	696.5 (592.6–823.2)	27.0 (7.1–50.2)	25.9 (6.7–49.0)
10	Cirrhosis and other chronic liver diseases	2.3 (2.2–2.5)	0.0567 (0.0479–0.0659)	382.6 (323.9–446.4)	30.6 (10.4–53.4)	-6.8 (-21.9–9.4)
11	Diabetes mellitus	2.2 (2.0–2.3)	0.0529 (0.0452–0.0614)	308.9 (263.2–357.3)	19.6 (1.3–39.0)	-21.5 (-33.6–-8.4)
12	Colon and rectum cancer	2.0 (1.8–2.2)	0.0495 (0.0416–0.0584)	286.2 (239.9–338.0)	-2.1 (-19.4–17.1)	-36.0 (-47.3–-23.6)
13	Hypertensive heart disease	2.0 (1.8–2.3)	0.0494 (0.0417–0.0580)	299.2 (252.8–351.1)	206.8 (159.9–260.3)	106.1 (73.0–142.0)
14	Road injuries	1.9 (1.8–2.1)	0.0472 (0.0405–0.0551)	476.2 (410.2–549.2)	-47.7 (-55.0–-38.5)	-49.6 (-56.6–-41.1)
15	Pancreatic cancer	1.7 (1.6–1.8)	0.0412 (0.0356–0.0474)	221.4 (191.6–255.1)	78.1 (51.8–107.4)	7.4 (-8.2–25.6)
16	Breast cancer	1.6 (1.3–1.9)	0.0385 (0.0312–0.0469)	237.2 (190.1–289.6)	-19.2 (-35.1–-0.6)	-46.5 (-57.6–-34.1)
17	Interpersonal violence	1.4 (1.3–1.5)	0.0341 (0.0296–0.0391)	388.3 (337.1–442.4)	-47.2 (-54.7–-39.5)	-42.1 (-50.1–-33.9)
18	Neonatal disorders	1.3 (1.0–1.5)	0.0306 (0.0273–0.0345)	568.3 (505.5–640.2)	-60.8 (-65.5–-55.4)	-44.0 (-50.8–-36.4)
19	Lower respiratory infections	1.3 (1.1–1.4)	0.0305 (0.0250–0.0366)	188.1 (157.2–222.9)	-31.2 (-43.3–-18.0)	-53.9 (-61.7–-45.4)
20	Endocrine, metabolic, blood, and immune disorders	1.2 (1.1–1.3)	0.0293 (0.0252–0.0339)	207.3 (179.5–237.0)	157.5 (123.5–196.3)	81.1 (56.9–107.2)
21	Falls	1.1 (0.9–1.1)	0.0256 (0.0218–0.0293)	143.3 (123.1–164.4)	145.3 (112.0–179.4)	48.1 (28.3–67.9)
22	Cardiomyopathy and myocarditis	0.9 (0.8–1.0)	0.0221 (0.0187–0.0257)	152.0 (130.6–176.8)	-28.9 (-39.9–-18.0)	-48.3 (-56.0–-40.6)
23	Prostate cancer	0.9 (0.8–1.0)	0.0216 (0.0174–0.0262)	105.4 (84.7–128.3)	-6.1 (-25.8–14.6)	-45.0 (-56.6–-32.5)
24	Leukaemia	0.8 (0.8–0.9)	0.0206 (0.0177–0.0237)	131.4 (113.9–150.6)	-6.4 (-19.8–7.2)	-37.6 (-46.1–-28.9)
25	Brain and central nervous system cancer	0.8 (0.8–0.9)	0.0196 (0.0169–0.0226)	141.9 (123.4–161.7)	15.8 (-0.6–34.5)	-15.1 (-26.6–-2.3)

Table S2: Cause-specific YLLs by US state in 2021 and percentage change between 1990 and 2021, Minnesota						
Rank	Cause Name	2021 Percentage of all cause YLLs	2021 YLLs (millions)	2021 Age Standardised Rate (per 100 000)	Percentage change YLL count 1990 to 2021	Percentage change age-standardised YLL rate 1990 to 2021
	All causes	100.0<br>(100.0–100.0)	1.01<br>(0.885–1.14)	11978.4<br>(10508.7–13492.1)	39.3<br>(21.9–57.5)	-13.9<br>(-24.4—2.7)
1	COVID-19	11.6<br>(10.2–13.2)	0.117<br>(0.116–0.118)	1411.9<br>(1409.5–1427.1)	--	--
2	Ischaemic heart disease	9.8<br>(8.8–10.6)	0.0995<br>(0.0813–0.115)	983.6<br>(799.4–1146.8)	-35.3<br>(-45.5—24.5)	-63.4<br>(-69.3—56.9)
3	Tracheal, bronchus, and lung cancer	5.5<br>(5.0–6.0)	0.0560<br>(0.0465–0.0666)	548.0<br>(454.6–654.7)	13.9<br>(-4.2–36.3)	-42.8<br>(-51.9—31.2)
4	Chronic obstructive pulmonary disease	4.7<br>(4.1–5.1)	0.0480<br>(0.0389–0.0558)	446.1<br>(363.0–522.2)	92.0<br>(63.8–123.5)	4.7<br>(-11.1–22.2)
5	Stroke	4.6<br>(4.1–5.0)	0.0464<br>(0.0386–0.0534)	449.9<br>(375.4–519.8)	8.1<br>(-7.3–23.5)	-36.4<br>(-45.6—27.0)
6	Alzheimer's disease and other dementias	4.1<br>(1.0–10.5)	0.0413<br>(0.0103–0.109)	357.8<br>(88.4–941.8)	70.0<br>(48.9–94.6)	-0.8<br>(-12.7–13.6)
7	Self-harm	3.6<br>(3.3–4.0)	0.0369<br>(0.0301–0.0437)	637.6<br>(520.1–751.8)	39.8<br>(13.2–66.8)	13.2<br>(-7.6–34.9)
8	Chronic kidney disease	3.3<br>(3.0–3.6)	0.0338<br>(0.0281–0.0392)	338.6<br>(283.8–391.7)	377.3<br>(308.4–453.7)	168.0<br>(128.3–209.7)
9	Drug use disorders	3.1<br>(2.7–3.5)	0.0309<br>(0.0248–0.0384)	545.1<br>(438.2–676.2)	1466.0<br>(1106.5–1883.9)	1263.3<br>(949.8–1624.5)
10	Cirrhosis and other chronic liver diseases	2.7<br>(2.5–2.9)	0.0271<br>(0.0227–0.0323)	330.5<br>(276.3–393.4)	144.7<br>(101.4–190.7)	46.6<br>(20.8–73.6)
11	Colon and rectum cancer	2.3<br>(2.0–2.5)	0.0231<br>(0.0191–0.0279)	243.7<br>(201.1–294.3)	5.0<br>(-13.4–28.0)	-39.8<br>(-50.5—26.3)
12	Diabetes mellitus	2.2<br>(2.0–2.4)	0.0224<br>(0.0185–0.0264)	239.0<br>(199.0–281.8)	52.6<br>(28.8–79.3)	-10.9<br>(-25.0–4.9)
13	Falls	2.1<br>(1.9–2.3)	0.0214<br>(0.0177–0.0247)	216.6<br>(181.1–249.8)	215.2<br>(168.3–261.8)	76.9<br>(50.4–103.7)
14	Road injuries	2.1<br>(2.0–2.2)	0.0210<br>(0.0178–0.0244)	368.0<br>(313.9–424.4)	-39.9<br>(-49.7—29.2)	-53.6<br>(-60.8—45.9)
15	Pancreatic cancer	1.9<br>(1.8–2.0)	0.0194<br>(0.0162–0.0228)	194.0<br>(162.1–228.6)	96.1<br>(65.3–131.4)	5.3<br>(-11.4–24.7)
16	Breast cancer	1.8<br>(1.5–2.2)	0.0184<br>(0.0146–0.0226)	204.6<br>(163.4–251.6)	-8.7<br>(-26.1–12.7)	-48.7<br>(-58.7—35.9)
17	Endocrine, metabolic, blood, and immune disorders	1.3<br>(1.2–1.4)	0.0133<br>(0.0111–0.0156)	167.0<br>(142.1–194.4)	256.2<br>(203.9–316.6)	112.1<br>(81.3–146.0)
18	Prostate cancer	1.2<br>(1.0–1.5)	0.0126<br>(0.00979–0.0158)	115.4<br>(89.2–144.7)	11.0<br>(-14.6–39.7)	-38.8<br>(-53.1—22.7)
19	Neonatal disorders	1.2<br>(0.9–1.4)	0.0117<br>(0.00971–0.0139)	366.4<br>(303.1–434.3)	-34.5<br>(-46.0—20.9)	-29.1<br>(-41.5—14.3)
20	Alcohol use disorders	1.1<br>(1.0–1.2)	0.0113<br>(0.00919–0.0137)	158.4<br>(129.2–189.9)	151.1<br>(104.5–203.8)	69.0<br>(37.0–105.2)
21	Leukaemia	1.1<br>(1.0–1.2)	0.0112<br>(0.00932–0.0132)	130.6<br>(110.1–152.3)	12.6<br>(-4.6–31.9)	-34.0<br>(-43.7—23.3)
22	Lower respiratory infections	1.1<br>(1.0–1.2)	0.0112<br>(0.00891–0.0133)	126.1<br>(101.8–147.7)	-44.2<br>(-54.5—33.5)	-63.3<br>(-69.6—56.3)
23	Congenital birth defects	1.1<br>(0.9–1.2)	0.0109<br>(0.00930–0.0125)	290.9<br>(246.1–336.0)	-32.1<br>(-42.0—21.4)	-34.5<br>(-45.3—23.5)
24	Hypertensive heart disease	1.0<br>(0.9–1.1)	0.0104<br>(0.00856–0.0123)	107.0<br>(87.7–126.9)	169.6<br>(126.9–216.0)	57.7<br>(30.9–86.9)
25	Parkinson's disease	1.0<br>(0.9–1.1)	0.0103<br>(0.00835–0.0119)	93.2<br>(75.8–108.2)	180.7<br>(138.4–224.3)	63.5<br>(38.5–89.6)

Table S2: Cause-specific YLLs by US state in 2021 and percentage change between 1990 and 2021, Mississippi

Rank	Cause Name	2021 Percentage of all cause YLLs	2021 YLLs (millions)	2021 Age Standardised Rate (per 100 000)	Percentage change YLL count 1990 to 2021	Percentage change age-standardised YLL rate 1990 to 2021
	All causes	100.0 (100.0–100.0)	0.934 (0.826–1.06)	23439.8 (20819.2–26364.6)	51.0 (34.3–70.9)	10.3 (-1.7–24.1)
1	COVID-19	15.4 (12.5–19.3)	0.143 (0.128–0.177)	3501.4 (3145.2–4337.6)	--	--
2	Ischaemic heart disease	11.8 (10.8–12.7)	0.110 (0.0944–0.129)	2366.2 (2024.8–2766.5)	-18.1 (-28.8–-4.5)	-43.6 (-51.3–-34.3)
3	Tracheal, bronchus, and lung cancer	5.2 (4.7–5.8)	0.0487 (0.0411–0.0581)	985.9 (833.7–1173.6)	10.7 (-7.3–32.0)	-31.6 (-42.6–-18.4)
4	Chronic obstructive pulmonary disease	5.0 (4.6–5.4)	0.0470 (0.0403–0.0541)	916.1 (784.2–1057.8)	154.3 (119.4–195.8)	64.9 (42.0–92.8)
5	Stroke	4.6 (4.2–4.9)	0.0426 (0.0367–0.0496)	906.7 (781.5–1055.3)	23.8 (6.5–44.6)	-15.2 (-27.2–-1.0)
6	Chronic kidney disease	3.8 (3.5–4.1)	0.0355 (0.0307–0.0412)	777.8 (676.6–899.8)	315.2 (260.7–381.0)	180.5 (143.6–223.9)
7	Road injuries	3.6 (3.4–3.9)	0.0337 (0.0292–0.0391)	1167.4 (1016.1–1345.4)	-26.1 (-35.6–-14.5)	-32.0 (-40.3–-21.5)
8	Hypertensive heart disease	2.8 (2.5–3.1)	0.0263 (0.0221–0.0312)	592.1 (496.8–705.5)	223.9 (172.4–285.1)	126.6 (89.1–171.0)
9	Drug use disorders	2.5 (2.0–3.1)	0.0237 (0.0182–0.0309)	802.2 (619.2–1049.4)	1395.7 (1007.7–1924.1)	1285.2 (931.6–1774.8)
10	Interpersonal violence	2.3 (2.1–2.5)	0.0214 (0.0184–0.0248)	809.3 (700.3–931.0)	-0.6 (-15.5–15.3)	-0.6 (-15.0–14.5)
11	Cirrhosis and other chronic liver diseases	2.3 (2.1–2.4)	0.0211 (0.0178–0.0247)	521.0 (439.8–609.8)	106.2 (73.7–142.5)	43.6 (20.5–68.5)
12	Self-harm	2.2 (2.0–2.5)	0.0210 (0.0177–0.0248)	701.4 (594.6–826.3)	48.5 (25.0–74.8)	35.7 (14.3–59.1)
13	Diabetes mellitus	2.2 (2.0–2.4)	0.0206 (0.0177–0.0242)	463.5 (396.7–543.5)	76.9 (51.7–106.4)	22.0 (5.0–43.1)
14	Colon and rectum cancer	2.2 (1.9–2.5)	0.0206 (0.0170–0.0248)	450.9 (374.7–544.8)	45.1 (19.4–77.3)	-0.4 (-17.8–21.0)
15	Alzheimer's disease and other dementias	2.0 (0.5–5.2)	0.0185 (0.00476–0.0489)	350.3 (90.2–927.6)	46.8 (30.6–68.5)	-1.4 (-12.1–13.0)
16	Lower respiratory infections	1.5 (1.4–1.7)	0.0145 (0.0120–0.0171)	334.1 (281.1–393.8)	-10.3 (-24.5–6.7)	-37.0 (-46.2–-25.6)
17	Breast cancer	1.5 (1.2–1.7)	0.0136 (0.0110–0.0169)	315.2 (253.1–392.5)	7.9 (-12.9–34.4)	-27.7 (-42.3–-9.9)
18	Pancreatic cancer	1.4 (1.3–1.5)	0.0132 (0.0113–0.0154)	275.2 (235.7–323.4)	83.5 (58.4–115.2)	19.6 (2.9–40.7)
19	Neonatal disorders	1.3 (1.1–1.6)	0.0123 (0.0103–0.0147)	742.2 (619.9–885.2)	-48.1 (-57.3–-37.3)	-35.3 (-46.7–-21.8)
20	Endocrine, metabolic, blood, and immune disorders	1.0 (1.0–1.1)	0.00956 (0.00835–0.0111)	257.6 (224.1–296.8)	198.0 (158.4–245.2)	117.0 (89.7–148.4)
21	Congenital birth defects	1.0 (0.8–1.1)	0.00897 (0.00773–0.0104)	479.9 (406.8–562.8)	-30.3 (-41.2–-18.1)	-20.2 (-33.4–-5.5)
22	Prostate cancer	0.9 (0.8–1.1)	0.00865 (0.00679–0.0108)	165.4 (129.7–206.0)	6.3 (-16.1–33.2)	-29.4 (-44.5–-11.4)
23	Cardiomyopathy and myocarditis	0.9 (0.8–0.9)	0.00794 (0.00671–0.00922)	206.1 (176.5–237.1)	-21.5 (-33.0–-7.1)	-40.3 (-48.8–-29.7)
24	Falls	0.8 (0.8–0.9)	0.00768 (0.00656–0.00891)	172.9 (148.4–199.6)	171.5 (133.4–211.4)	83.7 (58.0–109.4)
25	Leukaemia	0.8 (0.7–0.8)	0.00719 (0.00621–0.00836)	176.8 (154.4–203.8)	11.8 (-3.5–30.9)	-20.4 (-31.0–-7.6)

Table S2: Cause-specific YLLs by US state in 2021 and percentage change between 1990 and 2021, Missouri						
Rank	Cause Name	2021 Percentage of all cause YLLs	2021 YLLs (millions)	2021 Age Standardised Rate (per 100 000)	Percentage change YLL count 1990 to 2021	Percentage change age-standardised YLL rate 1990 to 2021
	All causes	100.0 (100.0–100.0)	1.57 (1.40–1.78)	17803.0 (15864.7–20002.7)	40.4 (24.0–58.7)	0.6 (-10.7–13.1)
1	Ischaemic heart disease	12.8 (11.8–13.5)	0.201 (0.172–0.233)	1865.7 (1589.0–2169.0)	-20.9 (-32.6–-8.3)	-46.8 (-54.9–-37.8)
2	COVID-19	12.7 (11.2–14.6)	0.199 (0.196–0.225)	2217.3 (2175.0–2503.6)	--	--
3	Tracheal, bronchus, and lung cancer	5.7 (5.2–6.2)	0.0901 (0.0760–0.105)	818.4 (687.3–957.8)	-1.2 (-17.4–16.2)	-41.0 (-50.8–-30.7)
4	Chronic obstructive pulmonary disease	5.4 (4.9–5.8)	0.0853 (0.0735–0.0990)	736.7 (634.0–856.8)	105.6 (76.3–139.5)	31.2 (12.5–53.0)
5	Drug use disorders	4.8 (4.2–5.4)	0.0749 (0.0606–0.0910)	1228.5 (999.1–1483.5)	1867.3 (1454.7–2380.5)	1718.0 (1346.5–2198.3)
6	Stroke	4.1 (3.7–4.5)	0.0646 (0.0554–0.0748)	587.7 (507.0–680.8)	11.4 (-4.9–27.9)	-25.7 (-36.7–-14.2)
7	Self-harm	3.2 (3.0–3.5)	0.0510 (0.0425–0.0603)	816.8 (681.1–963.4)	53.1 (26.9–81.9)	35.1 (11.9–60.2)
8	Chronic kidney disease	3.2 (2.9–3.4)	0.0500 (0.0428–0.0577)	469.7 (405.5–542.9)	326.3 (265.3–388.3)	175.2 (135.2–215.2)
9	Alzheimer's disease and other dementias	2.9 (0.7–7.3)	0.0456 (0.0114–0.114)	350.2 (87.4–888.9)	52.4 (32.3–72.7)	-2.4 (-15.3–10.8)
10	Road injuries	2.7 (2.5–2.8)	0.0420 (0.0359–0.0488)	698.7 (601.5–808.9)	-29.2 (-39.7–-16.9)	-39.9 (-48.3–-30.0)
11	Cirrhosis and other chronic liver diseases	2.2 (2.0–2.3)	0.0345 (0.0288–0.0405)	394.3 (330.4–463.5)	90.2 (57.8–125.9)	31.7 (10.2–56.4)
12	Colon and rectum cancer	2.1 (1.9–2.3)	0.0334 (0.0284–0.0392)	329.4 (278.8–386.2)	4.9 (-13.0–26.0)	-28.2 (-40.2–-13.7)
13	Diabetes mellitus	2.0 (1.9–2.1)	0.0316 (0.0266–0.0370)	317.6 (267.4–370.7)	35.8 (12.6–59.6)	-6.4 (-22.4–9.4)
14	Interpersonal violence	1.9 (1.8–2.1)	0.0307 (0.0263–0.0357)	566.3 (488.2–657.5)	3.6 (-11.7–20.2)	-1.8 (-15.9–13.6)
15	Hypertensive heart disease	1.6 (1.5–1.8)	0.0258 (0.0217–0.0302)	260.5 (218.7–305.9)	167.1 (123.2–215.5)	87.5 (55.9–122.7)
16	Pancreatic cancer	1.6 (1.5–1.7)	0.0253 (0.0216–0.0294)	236.6 (200.7–275.1)	94.4 (64.3–127.0)	23.8 (4.4–44.8)
17	Breast cancer	1.5 (1.3–1.8)	0.0242 (0.0195–0.0294)	252.5 (202.6–306.7)	-14.5 (-31.5–2.8)	-43.7 (-55.1–-31.8)
18	Lower respiratory infections	1.4 (1.2–1.5)	0.0217 (0.0182–0.0256)	224.2 (191.0–261.7)	-28.5 (-40.2–-15.5)	-48.8 (-56.8–-40.3)
19	Endocrine, metabolic, blood, and immune disorders	1.2 (1.1–1.2)	0.0183 (0.0157–0.0211)	218.7 (189.2–249.4)	203.7 (158.8–252.6)	106.8 (77.5–138.5)
20	Falls	1.1 (1.0–1.2)	0.0180 (0.0155–0.0208)	174.3 (151.2–200.8)	153.1 (118.1–194.0)	62.8 (39.1–89.7)
21	Neonatal disorders	1.1 (0.9–1.3)	0.0171 (0.0150–0.0192)	518.8 (456.0–582.7)	-45.4 (-52.8–-37.1)	-34.3 (-43.2–-24.2)
22	Prostate cancer	0.9 (0.7–1.0)	0.0142 (0.0112–0.0175)	118.8 (93.3–146.8)	4.2 (-19.2–29.2)	-32.4 (-47.6–-15.9)
23	Cardiomyopathy and myocarditis	0.9 (0.8–0.9)	0.0135 (0.0114–0.0159)	153.0 (129.3–179.7)	-14.4 (-28.0–-2.1)	-39.7 (-48.8–-29.1)
24	Leukaemia	0.9 (0.8–0.9)	0.0134 (0.0116–0.0155)	147.0 (127.0–167.4)	3.2 (-11.0–19.6)	-30.8 (-39.7–-20.2)
25	Congenital birth defects	0.8 (0.7–0.9)	0.0124 (0.0110–0.0137)	319.9 (281.5–355.7)	-40.2 (-46.9–-31.9)	-36.6 (-44.4–-28.1)

Table S2: Cause-specific YLLs by US state in 2021 and percentage change between 1990 and 2021, Montana						
Rank	Cause Name	2021 Percentage of all cause YLLs	2021 YLLs (millions)	2021 Age Standardised Rate (per 100 000)	Percentage change YLL count 1990 to 2021	Percentage change age-standardised YLL rate 1990 to 2021
	All causes	100.0<br>(100.0–100.0)	0.258<br>(0.229–0.291)	15698.3<br>(13991.5–17673.0)	70.1<br>(51.2–92.7)	-1.9<br>(-12.4–10.6)
1	COVID-19	16.0<br>(14.2–17.9)	0.0412<br>(0.0411–0.0420)	2456.8<br>(2452.2–2504.3)	--	--
2	Ischaemic heart disease	11.4<br>(10.2–12.2)	0.0294<br>(0.0246–0.0344)	1397.4<br>(1165.6–1653.8)	6.2<br>(-9.8–24.7)	-45.5<br>(-54.0—35.7)
3	Chronic obstructive pulmonary disease	6.0<br>(5.3–6.4)	0.0154<br>(0.0133–0.0178)	679.5<br>(587.7–784.5)	113.4<br>(83.7–147.5)	6.3<br>(-8.7–22.9)
4	Self-harm	4.6<br>(4.2–5.0)	0.0118<br>(0.00988–0.0141)	1120.0<br>(941.6–1331.2)	64.1<br>(36.0–96.8)	27.4<br>(6.3–52.6)
5	Tracheal, bronchus, and lung cancer	4.4<br>(4.0–4.9)	0.0115<br>(0.00978–0.0135)	546.9<br>(465.0–641.9)	8.2<br>(-8.8–29.6)	-46.8<br>(-55.4—36.1)
6	Stroke	3.5<br>(3.1–3.9)	0.00910<br>(0.00778–0.0105)	439.3<br>(373.7–505.8)	13.7<br>(-0.5–31.8)	-39.7<br>(-47.5—30.2)
7	Alzheimer's disease and other dementias	3.4<br>(0.9–8.4)	0.00865<br>(0.00231–0.0227)	350.1<br>(92.4–923.8)	105.5<br>(84.9–131.5)	-2.0<br>(-11.7–10.4)
8	Road injuries	3.1<br>(2.9–3.2)	0.00794<br>(0.00683–0.00924)	786.0<br>(682.9–911.5)	-23.5<br>(-33.7—10.4)	-41.7<br>(-49.1—32.0)
9	Cirrhosis and other chronic liver diseases	2.7<br>(2.5–3.0)	0.00709<br>(0.00603–0.00827)	465.7<br>(396.0–542.5)	129.7<br>(94.6–173.7)	41.4<br>(19.8–68.9)
10	Drug use disorders	2.6<br>(2.1–3.1)	0.00668<br>(0.00517–0.00841)	646.2<br>(499.0–812.0)	1479.1<br>(1070.2–2044.0)	1186.6<br>(848.8–1646.7)
11	Chronic kidney disease	2.6<br>(2.3–2.7)	0.00662<br>(0.00562–0.00773)	326.3<br>(277.4–378.0)	393.3<br>(322.8–467.6)	152.5<br>(114.9–192.8)
12	Colon and rectum cancer	2.0<br>(1.8–2.2)	0.00518<br>(0.00433–0.00621)	265.8<br>(222.4–318.2)	24.7<br>(1.7–51.8)	-32.9<br>(-44.9—18.2)
13	Diabetes mellitus	2.0<br>(1.8–2.1)	0.00514<br>(0.00432–0.00604)	276.8<br>(233.9–324.6)	55.6<br>(32.2–83.1)	-12.9<br>(-26.1–2.5)
14	Pancreatic cancer	1.6<br>(1.5–1.7)	0.00411<br>(0.00352–0.00477)	200.3<br>(170.6–232.3)	114.8<br>(82.7–151.0)	9.5<br>(-6.6–29.1)
15	Breast cancer	1.5<br>(1.3–1.8)	0.00399<br>(0.00339–0.00466)	223.7<br>(189.6–261.3)	8.7<br>(-8.6–29.0)	-40.6<br>(-50.5—29.5)
16	Falls	1.5<br>(1.3–1.6)	0.00388<br>(0.00329–0.00451)	202.7<br>(172.3–234.2)	223.2<br>(177.3–276.7)	65.2<br>(42.2–93.3)
17	Alcohol use disorders	1.3<br>(1.2–1.4)	0.00333<br>(0.00278–0.00394)	254.9<br>(214.2–299.2)	192.7<br>(141.7–255.8)	100.1<br>(66.1–142.1)
18	Endocrine, metabolic, blood, and immune disorders	1.2<br>(1.1–1.3)	0.00314<br>(0.00269–0.00362)	209.1<br>(182.0–239.4)	291.9<br>(238.0–355.1)	125.9<br>(96.5–160.2)
19	Prostate cancer	1.2<br>(1.0–1.4)	0.00302<br>(0.00232–0.00388)	127.7<br>(98.2–164.6)	25.7<br>(-2.8–63.2)	-38.4<br>(-52.5—20.0)
20	Lower respiratory infections	1.1<br>(1.0–1.2)	0.00291<br>(0.00240–0.00346)	162.0<br>(136.3–190.5)	-27.4<br>(-38.3—14.1)	-58.9<br>(-64.8—51.5)
21	Hypertensive heart disease	1.1<br>(1.0–1.2)	0.00283<br>(0.00235–0.00334)	149.7<br>(124.5–178.2)	206.4<br>(153.8–270.9)	70.0<br>(41.1–108.5)
22	Leukaemia	0.9<br>(0.8–0.9)	0.00228<br>(0.00195–0.00266)	135.3<br>(116.6–155.3)	28.2<br>(9.1–50.5)	-28.3<br>(-37.6—17.2)
23	Brain and central nervous system cancer	0.8<br>(0.8–0.9)	0.00218<br>(0.00188–0.00253)	146.6<br>(128.5–169.0)	64.2<br>(40.9–90.9)	-1.5<br>(-14.9–14.4)
24	Neonatal disorders	0.8<br>(0.6–1.0)	0.00209<br>(0.00171–0.00257)	367.2<br>(300.6–451.1)	-39.9<br>(-51.6—26.4)	-37.8<br>(-49.9—23.9)
25	Congenital birth defects	0.8<br>(0.6–0.9)	0.00199<br>(0.00167–0.00231)	295.9<br>(245.7–351.2)	-32.1<br>(-42.5—19.7)	-37.1<br>(-47.4—24.7)

Table S2: Cause-specific YLLs by US state in 2021 and percentage change between 1990 and 2021, Nebraska						
Rank	Cause Name	2021 Percentage of all cause YLLs	2021 YLLs (millions)	2021 Age Standardised Rate (per 100 000)	Percentage change YLL count 1990 to 2021	Percentage change age-standardised YLL rate 1990 to 2021
	All causes	100.0 (100.0–100.0)	0.381 (0.335–0.427)	13502.2 (11811.2–15106.3)	25.9 (9.8–41.7)	-11.1 (-22.5–0.2)
1	Ischaemic heart disease	11.2 (10.1–12.0)	0.0428 (0.0359–0.0500)	1267.2 (1053.9–1488.5)	-35.3 (-44.6—25.4)	-55.9 (-62.6—48.7)
2	COVID-19	9.8 (8.7–11.1)	0.0373 (0.0370–0.0407)	1351.6 (1342.3–1476.4)	--	--
3	Chronic obstructive pulmonary disease	6.2 (5.5–6.6)	0.0236 (0.0200–0.0271)	667.2 (565.1–768.0)	85.0 (58.5–112.4)	22.4 (4.5–41.0)
4	Tracheal, bronchus, and lung cancer	5.4 (4.9–5.8)	0.0205 (0.0172–0.0239)	624.0 (521.2–729.5)	-6.4 (-20.6–10.9)	-43.0 (-51.8—32.3)
5	Stroke	4.3 (3.8–4.7)	0.0166 (0.0139–0.0190)	485.8 (411.3–556.1)	-3.8 (-16.7–8.7)	-32.7 (-41.9—23.4)
6	Alzheimer's disease and other dementias	3.8 (1.0–9.6)	0.0144 (0.00369–0.0360)	360.1 (91.9–906.5)	42.4 (27.3–60.0)	0.1 (-10.7–12.4)
7	Chronic kidney disease	3.6 (3.2–3.9)	0.0138 (0.0117–0.0160)	416.7 (356.3–482.5)	308.8 (252.7–370.5)	174.0 (136.3–215.5)
8	Self-harm	3.4 (3.1–3.7)	0.0131 (0.0106–0.0153)	653.1 (535.1–764.0)	36.8 (11.3–61.8)	13.0 (-7.6–33.0)
9	Road injuries	3.1 (2.9–3.2)	0.0117 (0.00996–0.0133)	617.1 (526.0–700.9)	-26.1 (-37.6—15.4)	-39.3 (-48.6—30.7)
10	Cirrhosis and other chronic liver diseases	2.6 (2.4–2.8)	0.00989 (0.00829–0.0115)	375.3 (314.6–437.1)	108.4 (74.2–143.2)	42.4 (18.8–66.2)
11	Colon and rectum cancer	2.5 (2.2–2.7)	0.00944 (0.00782–0.0110)	301.4 (248.4–353.9)	3.4 (-13.5–23.9)	-29.5 (-41.4—15.1)
12	Diabetes mellitus	2.3 (2.2–2.5)	0.00897 (0.00756–0.0104)	292.8 (246.8–343.0)	52.1 (28.6–77.6)	4.4 (-12.2–22.9)
13	Drug use disorders	1.9 (1.6–2.3)	0.00738 (0.00583–0.00927)	378.0 (298.3–476.8)	1134.5 (843.0–1539.4)	967.6 (715.8–1324.7)
14	Breast cancer	1.9 (1.6–2.2)	0.00733 (0.00620–0.00865)	249.6 (212.0–296.2)	-6.3 (-20.5–10.8)	-38.1 (-47.8—26.2)
15	Pancreatic cancer	1.8 (1.6–1.9)	0.00672 (0.00570–0.00781)	206.9 (175.2–240.9)	70.3 (44.5–97.7)	10.7 (-6.4–28.7)
16	Endocrine, metabolic, blood, and immune disorders	1.6 (1.5–1.7)	0.00622 (0.00535–0.00709)	234.0 (202.6–264.9)	250.9 (199.9–299.8)	132.9 (100.2–163.5)
17	Lower respiratory infections	1.5 (1.3–1.7)	0.00584 (0.00485–0.00696)	186.3 (156.9–221.2)	-38.1 (-48.3—26.9)	-55.2 (-62.6—47.4)
18	Falls	1.4 (1.2–1.5)	0.00533 (0.00451–0.00616)	165.9 (139.5–191.1)	157.3 (121.3–195.8)	70.6 (46.1–96.5)
19	Neonatal disorders	1.3 (1.0–1.7)	0.00504 (0.00406–0.00613)	410.9 (330.9–499.8)	-30.5 (-44.8—13.4)	-30.1 (-44.5—12.8)
20	Hypertensive heart disease	1.3 (1.1–1.4)	0.00477 (0.00396–0.00556)	149.5 (124.7–174.0)	139.4 (104.2–178.4)	64.7 (38.4–94.0)
21	Congenital birth defects	1.1 (0.9–1.4)	0.00429 (0.00362–0.00503)	314.0 (261.7–374.9)	-30.2 (-41.7—16.6)	-34.5 (-46.3—21.0)
22	Prostate cancer	1.0 (0.9–1.2)	0.00394 (0.00311–0.00489)	108.6 (85.6–134.7)	-7.8 (-28.1–16.0)	-38.5 (-52.4—22.2)
23	Leukaemia	1.0 (0.9–1.1)	0.00393 (0.00335–0.00451)	138.1 (118.4–157.7)	8.1 (-8.5–27.0)	-27.6 (-38.6—16.0)
24	Brain and central nervous system cancer	1.0 (1.0–1.1)	0.00393 (0.00336–0.00452)	154.7 (133.5–177.8)	34.4 (13.9–56.3)	-7.3 (-21.2–7.8)
25	Parkinson's disease	0.9 (0.8–1.0)	0.00350 (0.00294–0.00403)	94.4 (79.6–108.7)	137.3 (106.6–168.9)	69.0 (46.9–92.0)

Table S2: Cause-specific YLLs by US state in 2021 and percentage change between 1990 and 2021, Nevada						
Rank	Cause Name	2021 Percentage of all cause YLLs	2021 YLLs (millions)	2021 Age Standardised Rate (per 100 000)	Percentage change YLL count 1990 to 2021	Percentage change age-standardised YLL rate 1990 to 2021
	All causes	100.0 (100.0–100.0)	0.727 (0.643–0.812)	16843.9 (14966.8–18743.4)	191.3 (156.5–226.6)	-11.0 (-21.2–-0.8)
1	COVID-19	18.5 (16.5–21.0)	0.134 (0.133–0.141)	3096.3 (3080.5–3266.8)	--	--
2	Ischaemic heart disease	11.8 (10.7–12.7)	0.0857 (0.0718–0.0996)	1688.6 (1414.4–1965.6)	93.1 (63.1–126.3)	-49.4 (-57.5–-40.8)
3	Chronic obstructive pulmonary disease	4.9 (4.4–5.2)	0.0354 (0.0304–0.0406)	673.0 (577.4–772.3)	201.2 (160.6–242.0)	-20.2 (-30.8–-9.4)
4	Drug use disorders	4.6 (3.9–5.3)	0.0336 (0.0270–0.0412)	976.8 (791.2–1183.9)	2028.4 (1552.1–2700.0)	784.1 (586.8–1057.2)
5	Tracheal, bronchus, and lung cancer	4.6 (4.1–5.1)	0.0334 (0.0281–0.0392)	650.3 (548.3–763.4)	65.1 (38.3–94.9)	-54.1 (-61.5–-46.0)
6	Self-harm	3.9 (3.6–4.2)	0.0283 (0.0236–0.0331)	830.1 (698.0–968.9)	104.3 (69.2–140.3)	-18.9 (-32.3–-5.2)
7	Stroke	3.6 (3.3–4.0)	0.0265 (0.0226–0.0304)	531.4 (453.4–610.2)	144.1 (109.2–178.9)	-36.6 (-45.7–-27.6)
8	Cirrhosis and other chronic liver diseases	2.9 (2.7–3.1)	0.0213 (0.0177–0.0248)	483.2 (403.1–562.5)	202.4 (148.7–253.5)	-3.2 (-20.1–12.7)
9	Chronic kidney disease	2.7 (2.5–2.9)	0.0198 (0.0167–0.0229)	405.8 (344.1–467.9)	739.3 (614.5–870.2)	124.0 (92.1–159.3)
10	Alzheimer's disease and other dementias	2.6 (0.7–6.7)	0.0187 (0.00482–0.0464)	346.3 (89.2–861.3)	380.9 (325.3–454.5)	-0.8 (-10.5–11.7)
11	Hypertensive heart disease	2.5 (2.1–2.9)	0.0182 (0.0149–0.0223)	379.8 (311.9–466.4)	257.4 (189.2–354.4)	2.3 (-16.9–29.6)
12	Road injuries	2.1 (1.9–2.2)	0.0150 (0.0127–0.0173)	466.8 (399.1–536.5)	-2.8 (-17.6–12.1)	-62.2 (-67.6–-56.5)
13	Colon and rectum cancer	2.0 (1.8–2.3)	0.0148 (0.0122–0.0175)	303.3 (250.1–358.9)	126.5 (85.9–171.5)	-35.5 (-47.0–-22.7)
14	Diabetes mellitus	1.8 (1.6–1.9)	0.0129 (0.0108–0.0151)	268.6 (225.6–312.2)	221.7 (169.4–275.1)	-7.4 (-22.7–7.8)
15	Breast cancer	1.7 (1.4–2.0)	0.0123 (0.0104–0.0143)	265.1 (223.8–308.4)	117.1 (81.2–158.9)	-34.7 (-45.3–-22.0)
16	Pancreatic cancer	1.5 (1.3–1.6)	0.0107 (0.00910–0.0124)	213.0 (180.1–245.2)	241.7 (187.6–295.7)	-4.5 (-19.7–10.5)
17	Interpersonal violence	1.3 (1.2–1.4)	0.00977 (0.00843–0.0112)	335.1 (290.7–383.5)	28.1 (10.1–48.1)	-44.4 (-51.7–-36.0)
18	Lower respiratory infections	1.3 (1.2–1.4)	0.00947 (0.00783–0.0113)	212.0 (177.3–251.5)	48.2 (21.9–74.9)	-58.9 (-65.8–-51.6)
19	Alcohol use disorders	1.2 (1.1–1.3)	0.00861 (0.00709–0.0103)	215.4 (178.0–255.0)	234.6 (171.8–302.0)	17.9 (-3.5–41.0)
20	Endocrine, metabolic, blood, and immune disorders	1.1 (1.0–1.1)	0.00771 (0.00656–0.00878)	185.0 (160.1–209.0)	551.7 (455.4–650.4)	102.0 (73.5–130.2)
21	Prostate cancer	1.0 (0.8–1.1)	0.00716 (0.00556–0.00882)	133.8 (104.0–164.9)	153.0 (97.3–214.3)	-35.7 (-49.7–-20.3)
22	Neonatal disorders	1.0 (0.8–1.2)	0.00713 (0.00590–0.00852)	434.1 (358.9–518.6)	15.3 (-5.3–39.5)	-26.2 (-39.3–-10.7)
23	Falls	0.9 (0.8–1.0)	0.00652 (0.00551–0.00744)	137.2 (116.2–156.6)	434.4 (359.8–520.4)	45.3 (25.0–67.8)
24	Leukaemia	0.8 (0.7–0.8)	0.00563 (0.00481–0.00647)	128.2 (110.5–146.2)	113.9 (81.7–147.7)	-35.8 (-44.8–-26.0)
25	Cardiomyopathy and myocarditis	0.8 (0.7–0.8)	0.00556 (0.00466–0.00650)	131.6 (111.1–152.5)	24.9 (4.5–49.2)	-59.8 (-66.0–-52.2)

Table S2: Cause-specific YLLs by US state in 2021 and percentage change between 1990 and 2021, New Hampshire						
Rank	Cause Name	2021 Percentage of all cause YLLs	2021 YLLs (millions)	2021 Age Standardised Rate (per 100 000)	Percentage change YLL count 1990 to 2021	Percentage change age-standardised YLL rate 1990 to 2021
	All causes	100.0 (100.0–100.0)	0.278 (0.243–0.316)	12700.1 (11169.4–14359.7)	48.9 (30.5–69.0)	-15.2 (-25.2—4.0)
1	Ischaemic heart disease	11.7 (10.5–12.6)	0.0327 (0.0271–0.0386)	1181.3 (986.6–1396.3)	-21.3 (-32.9—7.7)	-62.4 (-68.1—55.7)
2	COVID-19	9.4 (8.2–11.2)	0.0262 (0.0258–0.0303)	1199.9 (1184.7–1390.1)	--	--
3	Drug use disorders	6.1 (5.4–6.8)	0.0169 (0.0137–0.0208)	1265.0 (1033.3–1539.3)	1929.6 (1462.5–2515.7)	1850.6 (1407.8–2387.1)
4	Tracheal, bronchus, and lung cancer	6.0 (5.4–6.5)	0.0168 (0.0140–0.0200)	603.2 (505.7–717.1)	13.9 (-4.3–34.8)	-48.4 (-56.8—39.0)
5	Chronic obstructive pulmonary disease	5.5 (4.9–5.9)	0.0153 (0.0129–0.0177)	527.3 (446.2–611.4)	115.9 (84.2–147.8)	0.7 (-14.0–15.7)
6	Stroke	3.8 (3.3–4.2)	0.0107 (0.00899–0.0124)	393.3 (332.6–452.4)	15.7 (0.7–32.2)	-42.5 (-50.1—34.0)
7	Alzheimer's disease and other dementias	3.9 (1.0–9.8)	0.0107 (0.00279–0.0278)	353.0 (91.9–916.9)	110.5 (88.0–137.6)	-1.7 (-12.4–10.8)
8	Self-harm	3.5 (3.2–3.8)	0.00965 (0.00787–0.0116)	679.2 (559.6–808.4)	30.0 (5.9–57.1)	12.3 (-8.0–35.1)
9	Chronic kidney disease	3.0 (2.7–3.4)	0.00836 (0.00700–0.0100)	315.5 (264.9–377.9)	381.2 (303.0–475.8)	136.3 (99.0–181.8)
10	Cirrhosis and other chronic liver diseases	2.5 (2.3–2.7)	0.00707 (0.00587–0.00839)	325.2 (271.3–385.0)	84.9 (51.5–118.3)	5.8 (-13.2–25.3)
11	Colon and rectum cancer	2.3 (2.1–2.5)	0.00636 (0.00532–0.00763)	246.9 (207.1–294.7)	11.1 (-7.3–32.9)	-43.8 (-52.9—32.7)
12	Diabetes mellitus	2.3 (2.1–2.4)	0.00628 (0.00526–0.00746)	250.0 (209.5–296.9)	50.0 (26.8–77.6)	-22.0 (-34.0—7.6)
13	Pancreatic cancer	2.0 (1.8–2.1)	0.00561 (0.00469–0.00658)	204.1 (170.6–239.1)	122.3 (91.2–159.1)	4.1 (-10.2–21.4)
14	Breast cancer	1.9 (1.6–2.3)	0.00536 (0.00441–0.00637)	221.2 (182.8–262.6)	-2.1 (-18.2–19.2)	-49.3 (-58.1—38.1)
15	Road injuries	1.8 (1.7–1.9)	0.00504 (0.00424–0.00593)	374.3 (317.8–436.4)	-43.1 (-52.1—32.8)	-52.0 (-59.4—43.9)
16	Endocrine, metabolic, blood, and immune disorders	1.6 (1.5–1.7)	0.00451 (0.00380–0.00524)	216.7 (184.2–249.0)	275.0 (218.9–336.9)	112.5 (81.8–145.4)
17	Hypertensive heart disease	1.6 (1.4–1.7)	0.00437 (0.00366–0.00515)	175.0 (147.5–206.3)	216.1 (168.6–273.4)	65.5 (40.3–95.8)
18	Falls	1.6 (1.4–1.7)	0.00432 (0.00362–0.00500)	169.1 (142.5–196.2)	242.5 (192.6–292.6)	73.9 (48.7–100.4)
19	Lower respiratory infections	1.2 (1.1–1.4)	0.00343 (0.00276–0.00411)	139.6 (113.5–165.5)	-23.2 (-36.6—9.2)	-59.2 (-65.9—52.1)
20	Prostate cancer	1.2 (1.0–1.4)	0.00342 (0.00258–0.00433)	115.8 (87.2–146.2)	32.9 (-0.3–70.0)	-38.0 (-53.5—20.7)
21	Alcohol use disorders	1.1 (1.0–1.2)	0.00304 (0.00248–0.00369)	170.4 (139.8–204.1)	151.0 (107.0–205.5)	74.7 (43.9–110.4)
22	Parkinson's disease	1.0 (0.8–1.0)	0.00268 (0.00222–0.00316)	91.3 (75.5–107.4)	215.9 (171.1–267.2)	52.0 (30.5–76.9)
23	Brain and central nervous system cancer	1.0 (0.9–1.0)	0.00267 (0.00227–0.00312)	128.3 (109.7–149.2)	53.5 (29.8–81.8)	-11.6 (-25.1–3.8)
24	Leukaemia	0.9 (0.8–1.0)	0.00259 (0.00217–0.00302)	113.6 (96.6–131.7)	24.7 (5.0–46.6)	-32.3 (-42.8—21.1)
25	Oesophageal cancer	0.9 (0.8–1.0)	0.00259 (0.00211–0.00321)	96.1 (78.6–119.4)	100.3 (60.6–150.6)	-5.8 (-24.6–17.6)

Table S2: Cause-specific YLLs by US state in 2021 and percentage change between 1990 and 2021, New Jersey						
Rank	Cause Name	2021 Percentage of all cause YLLs	2021 YLLs (millions)	2021 Age Standardised Rate (per 100 000)	Percentage change YLL count 1990 to 2021	Percentage change age-standardised YLL rate 1990 to 2021
	All causes	100.0<br>(100.0–100.0)	1.67<br>(1.46–1.89)	11969.2<br>(10464.0–13499.9)	2.6<br>(-10.4–16.8)	-30.8<br>(-39.3–-21.3)
1	COVID-19	12.9<br>(11.4–14.8)	0.216<br>(0.216–0.216)	1574.8<br>(1574.0–1575.4)	--	--
2	Ischaemic heart disease	12.8<br>(11.3–13.7)	0.214<br>(0.178–0.251)	1249.9<br>(1048.0–1465.2)	-38.3<br>(-47.2–-28.0)	-62.3<br>(-68.0–-55.9)
3	Drug use disorders	6.4<br>(5.6–7.3)	0.107<br>(0.0859–0.129)	1126.8<br>(906.4–1343.8)	859.7<br>(656.0–1099.3)	807.9<br>(619.5–1028.4)
4	Tracheal, bronchus, and lung cancer	5.0<br>(4.5–5.5)	0.0842<br>(0.0691–0.100)	503.6<br>(414.2–600.1)	-31.9<br>(-44.0–-19.6)	-58.9<br>(-66.1–-51.4)
5	Stroke	4.2<br>(3.6–4.5)	0.0696<br>(0.0573–0.0820)	410.6<br>(341.3–484.6)	-4.4<br>(-19.8–10.5)	-41.7<br>(-50.5–-32.1)
6	Alzheimer's disease and other dementias	4.1<br>(1.1–10.4)	0.0692<br>(0.0179–0.180)	353.5<br>(89.8–936.4)	76.9<br>(55.7–105.0)	-2.4<br>(-14.4–12.5)
7	Chronic obstructive pulmonary disease	3.9<br>(3.4–4.2)	0.0656<br>(0.0539–0.0766)	371.0<br>(307.3–433.0)	49.8<br>(27.0–73.7)	-7.9<br>(-21.5–7.2)
8	Chronic kidney disease	3.3<br>(2.9–3.7)	0.0557<br>(0.0458–0.0661)	339.1<br>(277.9–402.3)	214.3<br>(162.4–269.0)	89.7<br>(57.9–122.4)
9	Colon and rectum cancer	2.5<br>(2.2–2.8)	0.0417<br>(0.0339–0.0499)	267.1<br>(218.5–319.4)	-21.3<br>(-35.7–-6.1)	-47.8<br>(-57.4–-37.6)
10	Cirrhosis and other chronic liver diseases	2.3<br>(2.2–2.5)	0.0389<br>(0.0320–0.0463)	280.3<br>(230.2–333.9)	-3.9<br>(-20.8–15.1)	-34.8<br>(-46.8–-21.9)
11	Diabetes mellitus	2.2<br>(2.0–2.4)	0.0371<br>(0.0306–0.0442)	240.4<br>(198.8–284.9)	-16.8<br>(-30.7–-1.4)	-45.0<br>(-54.2–-34.7)
12	Breast cancer	2.1<br>(1.7–2.5)	0.0351<br>(0.0277–0.0434)	235.0<br>(184.9–291.9)	-27.9<br>(-43.1–-9.2)	-52.9<br>(-63.2–-40.4)
13	Self-harm	1.9<br>(1.8–2.1)	0.0324<br>(0.0264–0.0390)	327.2<br>(267.6–392.8)	6.0<br>(-13.9–26.8)	-7.6<br>(-24.7–10.4)
14	Pancreatic cancer	1.9<br>(1.8–2.0)	0.0322<br>(0.0272–0.0379)	195.3<br>(164.6–230.2)	45.7<br>(22.4–71.4)	-9.0<br>(-23.7–6.9)
15	Road injuries	1.6<br>(1.5–1.7)	0.0270<br>(0.0227–0.0320)	288.1<br>(243.0–339.3)	-52.7<br>(-60.8–-43.9)	-60.4<br>(-67.0–-53.5)
16	Endocrine, metabolic, blood, and immune disorders	1.5<br>(1.4–1.6)	0.0248<br>(0.0211–0.0291)	185.5<br>(158.3–216.0)	133.6<br>(97.0–171.6)	49.3<br>(25.9–72.6)
17	Hypertensive heart disease	1.5<br>(1.3–1.7)	0.0248<br>(0.0201–0.0302)	157.3<br>(127.0–191.8)	64.0<br>(33.4–101.4)	6.4<br>(-14.0–31.3)
18	Lower respiratory infections	1.4<br>(1.2–1.5)	0.0230<br>(0.0185–0.0275)	147.4<br>(120.4–176.7)	-41.2<br>(-51.7–-30.2)	-63.4<br>(-69.8–-56.6)
19	Cardiomyopathy and myocarditis	1.1<br>(1.0–1.2)	0.0185<br>(0.0150–0.0225)	130.2<br>(107.1–157.5)	-26.2<br>(-40.2–-11.1)	-51.1<br>(-60.2–-41.3)
20	Neonatal disorders	1.1<br>(0.9–1.3)	0.0179<br>(0.0158–0.0201)	348.8<br>(307.3–392.5)	-67.3<br>(-71.7–-62.7)	-62.4<br>(-67.4–-57.0)
21	Interpersonal violence	1.0<br>(0.9–1.1)	0.0170<br>(0.0141–0.0199)	206.3<br>(172.0–240.3)	-44.6<br>(-54.3–-35.7)	-46.5<br>(-55.6–-38.0)
22	Prostate cancer	1.0<br>(0.8–1.2)	0.0168<br>(0.0130–0.0209)	94.5<br>(72.8–118.0)	-23.7<br>(-41.7–-4.4)	-52.3<br>(-63.6–-40.1)
23	Leukaemia	1.0<br>(0.9–1.0)	0.0160<br>(0.0134–0.0188)	112.3<br>(94.8–130.6)	-14.7<br>(-27.5–-0.7)	-45.0<br>(-53.0–-36.2)
24	Falls	0.9<br>(0.8–1.0)	0.0154<br>(0.0127–0.0182)	96.9<br>(80.6–113.3)	89.2<br>(57.7–122.9)	13.2<br>(-5.2–33.5)
25	Alcohol use disorders	0.9<br>(0.8–1.0)	0.0149<br>(0.0120–0.0179)	127.6<br>(103.6–153.8)	107.0<br>(66.9–153.7)	62.8<br>(31.5–99.3)

Table S2: Cause-specific YLLs by US state in 2021 and percentage change between 1990 and 2021, New Mexico						
Rank	Cause Name	2021 Percentage of all cause YLLs	2021 YLLs (millions)	2021 Age Standardised Rate (per 100 000)	Percentage change YLL count 1990 to 2021	Percentage change age-standardised YLL rate 1990 to 2021
	All causes	100.0 (100.0–100.0)	0.565 (0.498–0.632)	19313.9 (17079.7–21561.0)	101.3 (76.1–126.1)	11.3 (-2.5–25.2)
1	COVID-19	14.7 (11.8–18.1)	0.0827 (0.0711–0.102)	2656.1 (2282.0–3267.6)	--	--
2	Ischaemic heart disease	10.8 (9.7–11.8)	0.0613 (0.0516–0.0718)	1603.0 (1347.0–1876.8)	34.9 (14.9–55.7)	-40.3 (-49.4–-30.6)
3	Drug use disorders	5.5 (4.8–6.3)	0.0311 (0.0252–0.0370)	1502.4 (1227.0–1782.2)	868.5 (642.9–1133.5)	685.0 (505.2–896.1)
4	Cirrhosis and other chronic liver diseases	4.5 (4.1–4.8)	0.0252 (0.0213–0.0296)	919.4 (777.9–1078.1)	173.1 (129.2–223.4)	62.6 (36.9–92.4)
5	Chronic obstructive pulmonary disease	4.2 (3.7–4.5)	0.0236 (0.0201–0.0275)	581.0 (493.9–677.1)	131.3 (98.8–170.6)	-0.2 (-13.7–16.9)
6	Self-harm	4.1 (3.8–4.5)	0.0233 (0.0197–0.0271)	1107.0 (935.9–1284.6)	62.3 (35.5–90.6)	23.2 (2.9–44.3)
7	Road injuries	3.3 (3.0–3.5)	0.0184 (0.0156–0.0212)	900.4 (769.0–1033.4)	-24.9 (-36.5–-13.0)	-43.4 (-51.7–-34.8)
8	Tracheal, bronchus, and lung cancer	3.2 (2.9–3.5)	0.0181 (0.0151–0.0213)	485.4 (406.1–572.9)	22.4 (2.6–43.3)	-44.7 (-53.6–-35.2)
9	Stroke	3.1 (2.7–3.4)	0.0173 (0.0144–0.0201)	458.6 (388.0–530.5)	56.1 (33.8–80.3)	-30.3 (-40.5–-19.0)
10	Alzheimer's disease and other dementias	2.9 (0.8–7.0)	0.0164 (0.00430–0.0399)	355.0 (91.8–868.8)	168.3 (137.0–213.8)	-2.1 (-13.3–13.6)
11	Chronic kidney disease	2.7 (2.5–3.0)	0.0154 (0.0131–0.0180)	433.1 (370.7–501.7)	494.8 (410.0–581.0)	177.6 (140.3–216.5)
12	Alcohol use disorders	2.5 (2.2–2.7)	0.0139 (0.0116–0.0164)	579.4 (484.3–682.3)	214.8 (159.3–275.1)	114.1 (75.6–154.1)
13	Diabetes mellitus	2.3 (2.1–2.5)	0.0130 (0.0111–0.0152)	395.5 (335.7–458.7)	77.7 (50.4–106.7)	-8.5 (-22.7–6.4)
14	Colon and rectum cancer	1.8 (1.6–2.0)	0.0104 (0.00859–0.0123)	306.4 (253.6–362.6)	63.2 (32.5–95.1)	-19.0 (-34.2–3.0)
15	Interpersonal violence	1.8 (1.6–1.9)	0.00994 (0.00848–0.0114)	528.5 (451.7–604.7)	9.0 (-7.3–26.8)	-10.2 (-23.4–3.9)
16	Breast cancer	1.5 (1.3–1.8)	0.00864 (0.00689–0.0106)	272.6 (215.4–334.6)	46.1 (15.3–84.6)	-23.8 (-40.2–-2.9)
17	Falls	1.5 (1.3–1.6)	0.00857 (0.00722–0.00996)	250.1 (213.3–288.8)	259.5 (207.5–312.9)	71.3 (45.5–96.3)
18	Pancreatic cancer	1.4 (1.3–1.5)	0.00786 (0.00668–0.00920)	216.3 (184.1–253.0)	149.0 (111.5–191.2)	16.1 (-1.4–35.8)
19	Endocrine, metabolic, blood, and immune disorders	1.3 (1.2–1.4)	0.00749 (0.00645–0.00865)	272.3 (237.1–309.8)	310.3 (249.5–372.2)	133.5 (100.5–165.5)
20	Lower respiratory infections	1.1 (1.0–1.3)	0.00649 (0.00530–0.00762)	205.1 (171.1–237.0)	-4.7 (-20.7–12.8)	-51.1 (-59.0–-42.2)
21	Hypertensive heart disease	1.1 (0.9–1.2)	0.00615 (0.00502–0.00742)	186.0 (152.1–225.8)	196.5 (142.3–262.5)	49.4 (22.9–84.4)
22	Liver cancer	0.9 (0.8–1.0)	0.00518 (0.00435–0.00618)	151.3 (126.4–180.9)	488.8 (381.0–602.4)	184.2 (131.9–237.7)
23	Neonatal disorders	0.9 (0.7–1.1)	0.00506 (0.00416–0.00612)	498.1 (409.2–602.6)	-45.1 (-55.9–-32.7)	-28.4 (-42.5–-12.3)
24	Prostate cancer	0.9 (0.7–1.1)	0.00503 (0.00394–0.00630)	119.5 (93.3–149.6)	54.8 (23.7–96.5)	-35.2 (-48.4–-17.3)
25	Congenital birth defects	0.8 (0.6–0.9)	0.00427 (0.00371–0.00492)	340.5 (290.3–401.2)	-44.4 (-52.5–-34.6)	-39.7 (-49.9–-28.3)

Table S2: Cause-specific YLLs by US state in 2021 and percentage change between 1990 and 2021, New York						
Rank	Cause Name	2021 Percentage of all cause YLLs	2021 YLLs (millions)	2021 Age Standardised Rate (per 100 000)	Percentage change YLL count 1990 to 2021	Percentage change age-standardised YLL rate 1990 to 2021
	All causes	100.0 (100.0–100.0)	3.61 (3.20–4.05)	11783.1 (10437.6–13168.1)	-11.1 (-21.2—0.3)	-37.6 (-44.7—30.2)
1	Ischaemic heart disease	15.7 (13.9–16.9)	0.568 (0.467–0.659)	1522.2 (1250.6–1768.3)	-39.2 (-48.1—30.0)	-60.6 (-66.4—54.6)
2	COVID-19	12.9 (11.4–14.8)	0.465 (0.463–0.498)	1551.8 (1543.4–1661.7)	--	--
3	Tracheal, bronchus, and lung cancer	5.2 (4.6–5.6)	0.187 (0.155–0.220)	523.3 (433.7–615.8)	-28.9 (-41.4—15.7)	-54.7 (-62.5—46.2)
4	Drug use disorders	4.9 (4.4–5.4)	0.177 (0.145–0.209)	823.2 (679.6–969.6)	389.9 (287.4–505.7)	375.1 (279.7–486.8)
5	Alzheimer's disease and other dementias	4.4 (1.2–10.6)	0.157 (0.0432–0.385)	353.1 (96.6–881.7)	65.8 (44.3–93.5)	-2.6 (-13.5–13.2)
6	Chronic obstructive pulmonary disease	3.8 (3.3–4.1)	0.136 (0.113–0.159)	356.5 (295.1–416.5)	33.4 (13.7–55.5)	-13.6 (-26.2–0.6)
7	Stroke	3.3 (2.9–3.6)	0.118 (0.0973–0.137)	327.7 (274.5–382.3)	-25.2 (-36.3—13.6)	-50.9 (-58.2—43.0)
8	Chronic kidney disease	2.9 (2.6–3.2)	0.106 (0.0875–0.122)	296.2 (248.6–342.2)	150.9 (114.9–188.0)	58.3 (36.5–81.4)
9	Colon and rectum cancer	2.3 (2.0–2.6)	0.0834 (0.0694–0.0990)	250.2 (207.3–297.0)	-26.9 (-39.0—13.3)	-48.4 (-57.3—38.6)
10	Self-harm	2.2 (2.1–2.5)	0.0812 (0.0687–0.0960)	381.4 (322.7–449.0)	-1.5 (-17.9–16.6)	-7.4 (-22.6–9.2)
11	Hypertensive heart disease	2.1 (1.8–2.6)	0.0769 (0.0604–0.0967)	222.4 (173.7–280.1)	73.5 (37.9–119.9)	14.8 (-8.7–45.6)
12	Diabetes mellitus	2.1 (1.9–2.3)	0.0766 (0.0639–0.0902)	229.7 (193.6–269.6)	12.8 (-4.8–31.3)	-22.3 (-33.9—9.3)
13	Cirrhosis and other chronic liver diseases	2.1 (1.9–2.2)	0.0755 (0.0629–0.0884)	253.6 (212.1–297.3)	-18.8 (-32.1—5.2)	-42.2 (-51.6—32.0)
14	Breast cancer	1.9 (1.6–2.3)	0.0694 (0.0561–0.0853)	217.8 (175.3–268.1)	-35.9 (-48.4—20.9)	-55.7 (-64.7—45.2)
15	Pancreatic cancer	1.9 (1.7–2.0)	0.0676 (0.0570–0.0783)	192.0 (163.3–222.7)	36.2 (17.1–57.8)	-10.0 (-22.8–4.7)
16	Lower respiratory infections	1.8 (1.6–2.0)	0.0655 (0.0534–0.0785)	195.6 (161.2–232.0)	-44.2 (-53.7—33.2)	-63.0 (-69.3—56.3)
17	Road injuries	1.4 (1.3–1.5)	0.0522 (0.0443–0.0607)	254.6 (217.8–293.8)	-61.4 (-67.3—55.3)	-65.2 (-70.5—60.2)
18	Endocrine, metabolic, blood, and immune disorders	1.2 (1.1–1.3)	0.0437 (0.0370–0.0505)	150.6 (129.7–172.5)	116.2 (84.8–151.1)	43.7 (24.7–66.5)
19	Interpersonal violence	1.0 (1.0–1.1)	0.0378 (0.0324–0.0439)	209.8 (181.8–242.4)	-76.1 (-79.7—72.1)	-75.0 (-78.5—71.0)
20	Neonatal disorders	1.0 (0.9–1.2)	0.0377 (0.0333–0.0423)	355.9 (314.5–399.9)	-74.3 (-77.5—71.1)	-66.0 (-70.2—61.8)
21	Prostate cancer	1.0 (0.8–1.2)	0.0365 (0.0288–0.0453)	94.2 (74.4–117.3)	-17.0 (-33.5–4.2)	-45.6 (-56.4—31.5)
22	Falls	1.0 (0.9–1.1)	0.0363 (0.0299–0.0425)	104.0 (87.1–121.0)	58.2 (33.4–85.3)	-2.2 (-17.4–14.8)
23	Leukaemia	0.9 (0.8–1.0)	0.0333 (0.0281–0.0386)	109.4 (93.4–125.4)	-22.9 (-34.4—10.6)	-47.3 (-54.6—39.5)
24	Alcohol use disorders	0.9 (0.8–0.9)	0.0311 (0.0257–0.0368)	122.9 (102.1–145.1)	26.4 (5.9–50.5)	3.0 (-13.5–22.7)
25	Non-Hodgkin lymphoma	0.8 (0.7–0.9)	0.0295 (0.0246–0.0339)	86.2 (72.3–99.0)	-38.0 (-47.9—28.2)	-59.8 (-66.2—53.4)

Table S2: Cause-specific YLLs by US state in 2021 and percentage change between 1990 and 2021, North Carolina						
**Rank**	**Cause Name**	**2021 Percentage of all cause YLLs**	**2021 YLLs (millions)**	**2021 Age Standardised Rate (per 100 000)**	**Percentage change YLL count 1990 to 2021**	**Percentage change age-standardised YLL rate 1990 to 2021**
	All causes	100.0 (100.0–100.0)	2.55 (2.24–2.87)	17341.7 (15341.4–19413.3)	79.1 (57.4–100.9)	-7.5 (-18.1–3.4)
1	COVID-19	13.2 (10.4–16.7)	0.336 (0.284–0.429)	2233.4 (1888.3–2849.8)	--	--
2	Ischaemic heart disease	10.6 (9.6–11.4)	0.271 (0.229–0.316)	1513.1 (1276.3–1760.4)	-8.9 (-22.1–5.5)	-58.1 (-64.3—51.3)
3	Drug use disorders	5.6 (4.9–6.2)	0.142 (0.117–0.168)	1346.6 (1109.5–1585.3)	2742.2 (2143.1–3408.4)	1967.6 (1534.8–2431.3)
4	Tracheal, bronchus, and lung cancer	5.3 (4.8–5.8)	0.135 (0.113–0.158)	735.3 (615.9–863.2)	26.6 (6.8–49.9)	-44.3 (-53.1—34.0)
5	Chronic obstructive pulmonary disease	4.8 (4.2–5.2)	0.122 (0.105–0.140)	637.5 (544.6–732.0)	189.5 (147.9–234.8)	29.2 (10.8–49.1)
6	Stroke	4.4 (3.9–4.8)	0.113 (0.0953–0.131)	630.0 (531.3–730.8)	39.0 (18.4–60.0)	-36.7 (-46.2—27.4)
7	Chronic kidney disease	3.4 (3.1–3.7)	0.0870 (0.0739–0.100)	501.0 (425.4–575.2)	423.1 (350.3–501.6)	138.7 (106.2–174.6)
8	Alzheimer's disease and other dementias	3.1 (0.8–7.9)	0.0798 (0.0201–0.201)	391.1 (98.3–987.2)	144.6 (115.6–175.6)	-0.8 (-12.9–12.4)
9	Road injuries	2.8 (2.6–3.0)	0.0721 (0.0614–0.0834)	686.8 (592.0–785.5)	-13.4 (-26.7–0.1)	-42.5 (-50.9—33.9)
10	Cirrhosis and other chronic liver diseases	2.5 (2.3–2.7)	0.0636 (0.0535–0.0742)	417.2 (350.8–487.8)	113.9 (80.3–149.3)	7.7 (-9.2–26.0)
11	Self-harm	2.5 (2.3–2.7)	0.0634 (0.0531–0.0739)	577.5 (484.2–672.2)	50.3 (25.6–76.2)	2.0 (-14.4–19.3)
12	Diabetes mellitus	2.4 (2.2–2.6)	0.0607 (0.0519–0.0700)	363.8 (311.0–421.0)	88.3 (61.5–119.2)	-8.5 (-21.2–6.4)
13	Colon and rectum cancer	2.0 (1.8–2.2)	0.0513 (0.0436–0.0600)	303.1 (258.6–355.1)	47.2 (23.3–77.3)	-28.9 (-40.4—13.9)
14	Breast cancer	1.6 (1.3–1.9)	0.0412 (0.0323–0.0502)	255.5 (199.7–312.8)	21.9 (-3.6–50.4)	-40.8 (-53.6—26.7)
15	Neonatal disorders	1.6 (1.3–1.8)	0.0394 (0.0346–0.0446)	715.6 (628.5–809.0)	-26.6 (-35.1—16.9)	-32.1 (-40.0—23.2)
16	Endocrine, metabolic, blood, and immune disorders	1.5 (1.4–1.6)	0.0384 (0.0324–0.0442)	270.6 (232.1–308.1)	307.1 (247.0–369.0)	104.5 (75.2–132.7)
17	Pancreatic cancer	1.5 (1.4–1.6)	0.0383 (0.0322–0.0446)	212.3 (178.5–247.7)	131.3 (95.4–170.2)	5.6 (-10.6–23.3)
18	Interpersonal violence	1.5 (1.4–1.6)	0.0380 (0.0322–0.0440)	404.7 (347.2–465.5)	-14.3 (-27.3–0.1)	-34.7 (-44.1—24.2)
19	Lower respiratory infections	1.4 (1.2–1.5)	0.0358 (0.0294–0.0425)	225.3 (188.3–265.7)	1.9 (-15.3–20.5)	-50.9 (-58.8—42.7)
20	Hypertensive heart disease	1.3 (1.2–1.5)	0.0336 (0.0276–0.0390)	204.6 (170.2–238.1)	154.2 (110.3–198.1)	25.2 (3.0–46.9)
21	Falls	1.2 (1.0–1.3)	0.0299 (0.0253–0.0343)	173.7 (148.5–199.0)	257.3 (206.3–310.0)	60.6 (38.6–84.1)
22	Prostate cancer	0.9 (0.8–1.1)	0.0240 (0.0186–0.0296)	122.4 (95.2–150.6)	27.1 (0.4–56.8)	-43.5 (-55.4—30.2)
23	Cardiomyopathy and myocarditis	0.9 (0.8–1.0)	0.0233 (0.0195–0.0273)	159.9 (136.5–185.4)	5.8 (-11.2–23.1)	-44.4 (-53.0—35.3)
24	Alcohol use disorders	0.9 (0.8–1.0)	0.0229 (0.0191–0.0271)	177.1 (148.2–208.7)	96.7 (64.0–133.7)	14.6 (-3.9–35.7)
25	Congenital birth defects	0.8 (0.7–1.0)	0.0216 (0.0194–0.0238)	339.3 (301.7–377.8)	-18.4 (-27.7–-7.4)	-32.2 (-40.4—23.0)

Table S2: Cause-specific YLLs by US state in 2021 and percentage change between 1990 and 2021, North Dakota						
Rank	Cause Name	2021 Percentage of all cause YLLs	2021 YLLs (millions)	2021 Age Standardised Rate (per 100 000)	Percentage change YLL count 1990 to 2021	Percentage change age-standardised YLL rate 1990 to 2021
	All causes	100.0 (100.0–100.0)	0.139 (0.124–0.155)	12619.5 (11290.7–14093.5)	21.6 (8.4–35.7)	-12.4 (-21.9—2.0)
1	Ischaemic heart disease	12.1 (10.7–13.1)	0.0169 (0.0142–0.0194)	1233.4 (1037.3–1428.9)	-35.1 (-44.1—25.6)	-56.5 (-62.9—49.5)
2	COVID-19	11.6 (10.3–12.9)	0.0160 (0.0160–0.0161)	1413.8 (1413.1–1414.3)	--	--
3	Tracheal, bronchus, and lung cancer	5.0 (4.5–5.4)	0.00693 (0.00582–0.00801)	520.6 (436.8–604.3)	-5.9 (-20.7–9.8)	-42.4 (-51.6—32.6)
4	Self-harm	4.4 (4.1–4.8)	0.00615 (0.00514–0.00724)	797.6 (667.2–938.8)	68.1 (41.3–100.3)	45.6 (22.7–73.6)
5	Alzheimer's disease and other dementias	4.4 (1.2–10.7)	0.00614 (0.00168–0.0148)	356.1 (96.8–883.6)	53.2 (36.6–74.6)	-0.9 (-11.6–13.0)
6	Chronic obstructive pulmonary disease	4.4 (3.8–4.7)	0.00608 (0.00518–0.00700)	415.8 (355.2–476.8)	62.6 (40.4–87.5)	6.1 (-8.5–22.6)
7	Stroke	4.3 (3.7–4.7)	0.00603 (0.00513–0.00691)	437.5 (376.2–501.9)	-7.8 (-19.6–6.3)	-35.9 (-44.6—25.9)
8	Road injuries	3.4 (3.2–3.6)	0.00475 (0.00412–0.00540)	639.0 (556.3–725.8)	-24.3 (-34.5—13.7)	-35.3 (-44.1—26.2)
9	Chronic kidney disease	2.9 (2.1–3.5)	0.00408 (0.00281–0.00506)	306.2 (215.8–374.2)	233.3 (129.8–311.2)	122.4 (56.8–174.1)
10	Cirrhosis and other chronic liver diseases	2.7 (2.5–2.8)	0.00370 (0.00312–0.00428)	365.5 (308.2–422.5)	83.1 (55.8–113.6)	29.0 (9.1–51.1)
11	Colon and rectum cancer	2.4 (2.1–2.6)	0.00328 (0.00273–0.00383)	260.8 (218.2–304.3)	-6.7 (-21.8–9.6)	-36.6 (-47.0—25.2)
12	Diabetes mellitus	2.3 (2.1–2.5)	0.00327 (0.00276–0.00378)	270.9 (228.4–312.4)	16.7 (-0.4–34.6)	-16.9 (-28.9—3.6)
13	Drug use disorders	2.0 (1.7–2.4)	0.00282 (0.00223–0.00353)	365.7 (289.5–457.9)	1461.3 (1084.0–1921.1)	1262.6 (932.7–1661.8)
14	Endocrine, metabolic, blood, and immune disorders	1.9 (1.7–2.0)	0.00260 (0.00222–0.00296)	253.7 (219.3–288.9)	247.1 (199.8–297.2)	136.1 (106.2–167.9)
15	Pancreatic cancer	1.7 (1.5–1.8)	0.00231 (0.00196–0.00268)	177.3 (151.1–204.8)	55.5 (32.6–79.1)	1.4 (-13.6–17.1)
16	Breast cancer	1.6 (1.4–1.8)	0.00223 (0.00189–0.00263)	197.8 (167.7–235.4)	-17.4 (-30.2—2.6)	-43.6 (-52.7—33.0)
17	Neonatal disorders	1.6 (1.3–1.9)	0.00216 (0.00181–0.00261)	443.4 (370.8–534.4)	-27.3 (-42.0—8.8)	-31.1 (-45.0—13.5)
18	Congenital birth defects	1.4 (1.2–1.6)	0.00194 (0.00163–0.00225)	360.4 (301.8–421.7)	-21.5 (-35.5—5.2)	-28.7 (-41.7—13.0)
19	Falls	1.4 (1.2–1.5)	0.00191 (0.00163–0.00220)	147.1 (127.4–168.0)	146.8 (117.4–184.8)	61.4 (42.0–86.6)
20	Lower respiratory infections	1.4 (1.2–1.5)	0.00190 (0.00157–0.00224)	152.4 (128.3–178.9)	-37.9 (-47.2—27.4)	-55.8 (-62.5—48.1)
21	Alcohol use disorders	1.3 (1.2–1.4)	0.00177 (0.00146–0.00209)	196.6 (163.0–231.8)	152.8 (109.7–201.5)	90.0 (58.1–127.8)
22	Hypertensive heart disease	1.1 (1.0–1.3)	0.00156 (0.00129–0.00187)	123.4 (101.7–149.5)	128.2 (91.3–176.2)	58.3 (30.2–95.7)
23	Prostate cancer	1.1 (1.0–1.3)	0.00155 (0.00124–0.00187)	102.6 (82.2–123.6)	-29.7 (-43.8—12.7)	-52.9 (-62.5—41.4)
24	Leukaemia	1.1 (1.0–1.1)	0.00148 (0.00128–0.00169)	134.2 (117.0–152.3)	1.3 (-12.4–16.6)	-28.7 (-37.9—18.5)
25	Brain and central nervous system cancer	1.0 (1.0–1.1)	0.00142 (0.00123–0.00162)	146.4 (127.3–166.2)	26.0 (7.9–45.5)	-8.6 (-20.7–5.5)

Table S2: Cause-specific YLLs by US state in 2021 and percentage change between 1990 and 2021, Ohio						
Rank	Cause Name	2021 Percentage of all cause YLLs	2021 YLLs (millions)	2021 Age Standardised Rate (per 100 000)	Percentage change YLL count 1990 to 2021	Percentage change age-standardised YLL rate 1990 to 2021
	All causes	100.0 (100.0–100.0)	3.10 (2.74–3.46)	18000.5 (16006.2–20086.4)	37.5 (22.0–54.6)	3.7 (-7.6–15.8)
1	COVID-19	13.2 (11.4–15.5)	0.408 (0.394–0.472)	2341.1 (2265.1–2711.3)	--	--
2	Ischaemic heart disease	11.9 (10.8–12.8)	0.370 (0.316–0.425)	1732.5 (1478.5–1996.7)	-29.6 (-38.9–-19.4)	-52.8 (-59.2–-45.6)
3	Drug use disorders	6.8 (6.0–7.7)	0.211 (0.175–0.253)	1819.7 (1523.2–2164.3)	2413.5 (1930.6–2979.7)	2543.1 (2045.0–3122.4)
4	Tracheal, bronchus, and lung cancer	5.5 (4.9–6.0)	0.169 (0.142–0.197)	786.0 (659.8–913.0)	-8.0 (-23.1–7.8)	-41.4 (-51.2–-31.2)
5	Chronic obstructive pulmonary disease	5.2 (4.7–5.5)	0.160 (0.137–0.183)	706.5 (605.2–807.0)	92.4 (66.5–119.8)	25.6 (9.0–44.3)
6	Stroke	4.2 (3.8–4.6)	0.131 (0.111–0.150)	606.2 (515.3–692.6)	18.9 (4.2–36.4)	-21.1 (-31.1–-9.0)
7	Chronic kidney disease	3.3 (3.0–3.5)	0.102 (0.0873–0.115)	489.9 (420.7–555.4)	306.3 (251.4–366.8)	167.2 (132.4–205.0)
8	Alzheimer's disease and other dementias	2.8 (0.7–7.2)	0.0868 (0.0227–0.224)	349.5 (90.4–907.0)	60.1 (43.1–78.9)	-2.1 (-12.6–9.7)
9	Self-harm	2.6 (2.4–2.8)	0.0801 (0.0683–0.0936)	671.6 (578.1–781.1)	30.0 (10.5–52.9)	28.3 (9.2–50.2)
10	Diabetes mellitus	2.3 (2.1–2.5)	0.0707 (0.0604–0.0812)	363.4 (310.9–418.4)	18.2 (0.9–36.1)	-14.8 (-27.3–-1.2)
11	Cirrhosis and other chronic liver diseases	2.2 (2.0–2.4)	0.0681 (0.0577–0.0794)	388.8 (330.3–453.4)	76.8 (49.0–105.9)	30.2 (9.7–51.4)
12	Colon and rectum cancer	2.1 (1.9–2.3)	0.0648 (0.0540–0.0758)	325.0 (271.6–378.9)	-5.4 (-22.7–14.3)	-33.4 (-45.4–-19.6)
13	Road injuries	1.8 (1.7–2.0)	0.0564 (0.0481–0.0652)	483.8 (415.4–554.4)	-45.0 (-52.9–-35.9)	-48.2 (-55.4–-40.3)
14	Hypertensive heart disease	1.6 (1.5–1.8)	0.0506 (0.0421–0.0588)	263.0 (220.4–306.1)	120.5 (86.4–155.1)	59.4 (34.5–86.0)
15	Pancreatic cancer	1.6 (1.5–1.7)	0.0506 (0.0430–0.0586)	238.1 (202.8–275.3)	85.1 (59.1–114.8)	21.7 (4.3–41.5)
16	Breast cancer	1.6 (1.3–1.9)	0.0492 (0.0396–0.0589)	261.6 (209.9–315.4)	-19.9 (-34.7–-3.5)	-43.9 (-54.6–-31.6)
17	Endocrine, metabolic, blood, and immune disorders	1.5 (1.4–1.6)	0.0475 (0.0415–0.0543)	282.3 (248.9–319.9)	235.8 (192.9–286.0)	132.8 (105.1–166.6)
18	Lower respiratory infections	1.3 (1.1–1.4)	0.0399 (0.0330–0.0468)	211.1 (177.0–245.7)	-24.3 (-37.6–-11.1)	-46.2 (-55.0–-37.2)
19	Neonatal disorders	1.3 (1.1–1.5)	0.0393 (0.0350–0.0442)	608.2 (541.1–683.5)	-45.6 (-52.2–-38.1)	-29.5 (-38.0–-19.8)
20	Interpersonal violence	1.2 (1.1–1.3)	0.0383 (0.0329–0.0439)	374.0 (323.4–427.6)	-9.6 (-22.7–3.7)	-2.4 (-16.1–11.2)
21	Falls	1.1 (1.0–1.2)	0.0340 (0.0288–0.0387)	166.8 (142.7–190.1)	151.7 (117.9–188.2)	64.2 (42.4–87.8)
22	Cardiomyopathy and myocarditis	0.9 (0.9–1.0)	0.0293 (0.0245–0.0344)	171.1 (143.6–198.9)	-17.7 (-31.1–-4.2)	-37.7 (-47.2–-27.9)
23	Prostate cancer	0.9 (0.7–1.1)	0.0281 (0.0224–0.0345)	120.4 (96.0–148.3)	-1.3 (-22.5–25.4)	-35.5 (-49.5–-17.9)
24	Leukaemia	0.8 (0.8–0.9)	0.0260 (0.0224–0.0296)	143.7 (126.0–163.5)	-3.1 (-15.8–12.1)	-31.9 (-40.6–-21.6)
25	Congenital birth defects	0.8 (0.7–0.9)	0.0245 (0.0220–0.0270)	326.9 (294.0–362.0)	-44.2 (-50.7–-37.9)	-35.4 (-43.3–-27.6)

Table S2: Cause-specific YLLs by US state in 2021 and percentage change between 1990 and 2021, Oklahoma						
Rank	Cause Name	2021 Percentage of all cause YLLs	2021 YLLs (millions)	2021 Age Standardised Rate (per 100 000)	Percentage change YLL count 1990 to 2021	Percentage change age-standardised YLL rate 1990 to 2021
	All causes	100.0 (100.0–100.0)	1.12 (1.00–1.25)	20458.5 (18406.6–22819.8)	64.4 (47.6–83.8)	14.4 (2.7–27.8)
1	COVID-19	16.3 (14.1–19.2)	0.182 (0.176–0.211)	3326.5 (3221.8–3872.0)	--	--
2	Ischaemic heart disease	11.4 (10.4–12.2)	0.127 (0.109–0.148)	1989.6 (1717.9–2330.3)	-21.9 (-32.0–-10.1)	-47.8 (-54.8–-39.6)
3	Chronic obstructive pulmonary disease	5.5 (5.1–5.9)	0.0615 (0.0532–0.0706)	899.4 (778.7–1035.2)	129.4 (101.0–163.1)	46.9 (28.6–68.4)
4	Tracheal, bronchus, and lung cancer	5.1 (4.6–5.5)	0.0566 (0.0484–0.0667)	869.1 (741.4–1025.0)	5.1 (-11.7–22.3)	-35.8 (-46.2–-25.2)
5	Hypertensive heart disease	4.6 (4.2–4.9)	0.0510 (0.0435–0.0594)	826.8 (706.3–961.1)	853.5 (716.7–999.9)	533.3 (441.4–631.6)
6	Stroke	3.6 (3.2–3.9)	0.0401 (0.0346–0.0465)	631.2 (545.2–729.2)	6.2 (-8.0–22.0)	-27.4 (-37.1–-16.3)
7	Drug use disorders	3.5 (2.8–4.3)	0.0391 (0.0299–0.0503)	960.3 (737.3–1226.3)	1984.8 (1455.7–2687.2)	1649.0 (1211.2–2226.4)
8	Chronic kidney disease	3.4 (3.1–3.7)	0.0381 (0.0331–0.0441)	602.7 (523.4–700.8)	419.5 (347.9–499.7)	237.3 (191.5–289.0)
9	Self-harm	3.3 (3.0–3.6)	0.0364 (0.0307–0.0423)	897.3 (761.4–1039.3)	79.1 (50.3–111.1)	47.6 (24.1–73.6)
10	Cirrhosis and other chronic liver diseases	2.7 (2.5–2.9)	0.0306 (0.0261–0.0355)	573.4 (490.5–665.1)	152.1 (116.0–193.7)	74.1 (49.3–103.2)
11	Road injuries	2.7 (2.5–2.9)	0.0303 (0.0264–0.0348)	759.4 (666.9–866.5)	-23.3 (-33.1–-10.7)	-39.4 (-47.0–-29.9)
12	Alzheimer's disease and other dementias	2.3 (0.6–6.0)	0.0257 (0.00663–0.0676)	349.6 (90.0–922.8)	48.4 (31.9–67.0)	-2.6 (-13.7–9.3)
13	Diabetes mellitus	2.0 (1.8–2.1)	0.0219 (0.0187–0.0255)	372.7 (319.1–434.1)	67.2 (42.6–95.5)	14.2 (-2.1–33.0)
14	Colon and rectum cancer	1.9 (1.7–2.1)	0.0215 (0.0182–0.0251)	354.3 (300.0–413.3)	26.1 (4.3–48.0)	-14.1 (-28.8–1.2)
15	Breast cancer	1.4 (1.2–1.7)	0.0159 (0.0128–0.0193)	274.9 (219.9–335.0)	2.0 (-19.1–24.5)	-33.5 (-47.4–-18.4)
16	Interpersonal violence	1.4 (1.3–1.5)	0.0152 (0.0131–0.0175)	416.4 (362.2–478.3)	3.9 (-10.9–20.3)	-10.2 (-22.6–3.8)
17	Pancreatic cancer	1.3 (1.2–1.4)	0.0145 (0.0125–0.0169)	226.6 (195.9–264.0)	90.1 (64.4–121.9)	23.1 (6.3–44.1)
18	Falls	1.2 (1.1–1.3)	0.0134 (0.0116–0.0155)	215.2 (186.8–248.4)	247.0 (202.2–296.5)	116.3 (88.7–147.4)
19	Lower respiratory infections	1.2 (1.0–1.3)	0.0130 (0.0109–0.0157)	228.0 (194.2–273.0)	-33.5 (-43.6–-21.0)	-52.4 (-59.3–-44.1)
20	Neonatal disorders	1.1 (0.9–1.3)	0.0124 (0.0106–0.0140)	520.5 (445.6–591.0)	-27.0 (-38.3–-15.4)	-27.6 (-38.8–-16.0)
21	Endocrine, metabolic, blood, and immune disorders	1.0 (1.0–1.1)	0.0115 (0.0100–0.0134)	223.6 (196.6–259.7)	241.3 (196.1–295.7)	126.6 (97.7–161.3)
22	Congenital birth defects	0.8 (0.7–0.9)	0.00928 (0.00826–0.0104)	341.9 (301.2–386.9)	-25.6 (-35.1–-15.9)	-32.2 (-41.8–-22.6)
23	Prostate cancer	0.8 (0.7–1.0)	0.00923 (0.00755–0.0113)	130.9 (106.8–160.6)	10.0 (-13.0–35.7)	-28.3 (-43.3–-11.2)
24	Alcohol use disorders	0.8 (0.7–0.9)	0.00883 (0.00752–0.0105)	187.3 (159.2–221.9)	191.5 (144.9–247.6)	117.1 (83.0–159.3)
25	Leukaemia	0.8 (0.7–0.9)	0.00883 (0.00768–0.0103)	158.2 (139.2–182.1)	15.7 (-0.4–34.2)	-22.6 (-32.9–-11.3)

Table S2: Cause-specific YLLs by US state in 2021 and percentage change between 1990 and 2021, Oregon						
Rank	Cause Name	2021 Percentage of all cause YLLs	2021 YLLs (millions)	2021 Age Standardised Rate (per 100 000)	Percentage change YLL count 1990 to 2021	Percentage change age-standardised YLL rate 1990 to 2021
	All causes	100.0 (100.0–100.0)	0.906 (0.792–1.04)	13813.7 (12095.6–15710.1)	64.1 (44.2–87.3)	-12.9 (-23.4—0.7)
1	COVID-19	12.6 (9.5–16.8)	0.114 (0.0917–0.152)	1757.1 (1414.3–2344.2)	--	--
2	Ischaemic heart disease	9.0 (8.1–9.8)	0.0819 (0.0685–0.0977)	1005.2 (844.3–1206.4)	-26.7 (-37.2—13.3)	-64.8 (-70.0—58.1)
3	Tracheal, bronchus, and lung cancer	5.1 (4.6–5.7)	0.0465 (0.0386–0.0555)	575.2 (477.7–687.3)	3.8 (-14.8–24.7)	-52.9 (-61.2—43.5)
4	Chronic obstructive pulmonary disease	5.0 (4.5–5.4)	0.0454 (0.0386–0.0535)	528.9 (450.8–624.0)	97.2 (71.2–130.1)	-6.6 (-19.1–9.0)
5	Stroke	4.6 (4.0–5.0)	0.0416 (0.0350–0.0487)	503.9 (429.8–590.3)	33.0 (15.1–54.4)	-35.4 (-44.4—24.9)
6	Self-harm	4.1 (3.8–4.4)	0.0370 (0.0313–0.0443)	834.2 (706.7–995.4)	69.7 (42.0–102.4)	17.4 (-1.7–39.6)
7	Drug use disorders	3.7 (3.1–4.5)	0.0335 (0.0263–0.0430)	761.0 (597.5–969.9)	1008.3 (744.9–1341.4)	707.7 (522.1–931.9)
8	Alzheimer's disease and other dementias	3.7 (0.9–9.4)	0.0331 (0.00841–0.0844)	351.5 (89.5–898.3)	116.5 (91.4–153.1)	-1.6 (-13.1–14.8)
9	Cirrhosis and other chronic liver diseases	3.2 (2.9–3.4)	0.0290 (0.0245–0.0348)	456.6 (387.5–547.2)	173.5 (129.0–229.3)	47.1 (22.9–76.0)
10	Chronic kidney disease	3.0 (2.7–3.2)	0.0271 (0.0230–0.0320)	340.2 (289.8–401.9)	458.8 (381.6–553.9)	165.6 (129.3–210.3)
11	Road injuries	2.4 (2.2–2.6)	0.0217 (0.0184–0.0252)	506.9 (431.0–585.1)	-26.3 (-37.5—14.3)	-51.6 (-58.5—43.8)
12	Diabetes mellitus	2.3 (2.1–2.5)	0.0211 (0.0176–0.0256)	288.2 (242.1–350.2)	91.0 (60.2–128.1)	-2.7 (-18.2–15.9)
13	Colon and rectum cancer	2.1 (1.9–2.4)	0.0192 (0.0159–0.0231)	257.0 (211.1–309.3)	31.9 (7.3–58.9)	-33.4 (-45.8—19.3)
14	Pancreatic cancer	1.8 (1.6–1.9)	0.0159 (0.0134–0.0191)	201.0 (169.1–240.1)	123.5 (87.4–164.4)	6.3 (-10.5–25.1)
15	Breast cancer	1.7 (1.4–2.0)	0.0153 (0.0122–0.0192)	217.2 (172.8–274.8)	17.9 (-5.9–45.6)	-41.0 (-53.3—26.6)
16	Endocrine, metabolic, blood, and immune disorders	1.6 (1.4–1.7)	0.0141 (0.0120–0.0166)	225.5 (195.0–263.7)	306.2 (249.1–378.8)	105.1 (78.0–138.5)
17	Falls	1.5 (1.3–1.7)	0.0138 (0.0115–0.0161)	176.5 (149.0–205.7)	259.3 (211.5–315.9)	62.5 (40.5–88.5)
18	Alcohol use disorders	1.4 (1.3–1.5)	0.0126 (0.0105–0.0154)	228.8 (191.8–275.9)	189.5 (137.9–253.6)	70.6 (41.4–106.9)
19	Hypertensive heart disease	1.1 (1.0–1.3)	0.0104 (0.00859–0.0125)	138.7 (116.0–167.6)	183.8 (142.2–242.9)	46.0 (23.5–77.6)
20	Prostate cancer	1.1 (0.9–1.3)	0.00997 (0.00780–0.0125)	113.0 (88.2–142.8)	27.4 (0.7–65.5)	-39.3 (-52.2—20.8)
21	Non-rheumatic valvular heart disease	1.0 (0.9–1.1)	0.00926 (0.00749–0.0110)	104.9 (85.9–124.5)	99.7 (71.8–134.8)	-9.4 (-22.1–6.3)
22	Lower respiratory infections	1.0 (0.9–1.1)	0.00908 (0.00737–0.0111)	127.5 (104.8–154.6)	-33.0 (-44.3—19.2)	-64.5 (-70.3—57.6)
23	Liver cancer	1.0 (0.9–1.1)	0.00886 (0.00739–0.0108)	116.8 (97.1–142.7)	408.0 (318.8–511.0)	140.9 (99.2–189.1)
24	Brain and central nervous system cancer	0.9 (0.8–1.0)	0.00820 (0.00707–0.00969)	136.4 (118.5–159.7)	48.6 (27.7–76.1)	-20.3 (-31.5—6.3)
25	Leukaemia	0.9 (0.8–1.0)	0.00808 (0.00687–0.00944)	119.3 (102.6–139.0)	24.2 (6.5–45.9)	-37.2 (-45.7—26.4)

Table S2: Cause-specific YLLs by US state in 2021 and percentage change between 1990 and 2021, Pennsylvania						
Rank	Cause Name	2021 Percentage of all cause YLLs	2021 YLLs (millions)	2021 Age Standardised Rate (per 100 000)	Percentage change YLL count 1990 to 2021	Percentage change age-standardised YLL rate 1990 to 2021
	All causes	100.0 (100.0–100.0)	3.09 (2.76–3.45)	15548.7 (13933.5–17271.1)	15.3 (2.7–29.0)	-10.1 (-19.8–0.2)
1	COVID-19	14.1 (12.6–15.8)	0.435 (0.434–0.449)	2180.7 (2175.1–2251.6)	--	--
2	Ischaemic heart disease	12.2 (10.8–13.0)	0.377 (0.317–0.436)	1493.4 (1260.0–1726.6)	-40.0 (-48.3—31.4)	-57.6 (-63.7—51.5)
3	Drug use disorders	7.1 (6.4–7.7)	0.218 (0.183–0.261)	1690.3 (1424.4–2013.4)	1146.0 (900.1–1432.1)	1167.7 (925.8–1457.7)
4	Tracheal, bronchus, and lung cancer	5.3 (4.7–5.8)	0.164 (0.136–0.195)	657.5 (546.5–779.5)	-21.1 (-34.1—6.5)	-46.8 (-55.7—36.9)
5	Chronic obstructive pulmonary disease	4.3 (3.9–4.7)	0.134 (0.113–0.155)	502.4 (426.8–579.4)	49.1 (28.7–70.8)	5.2 (-9.5–21.0)
6	Stroke	4.0 (3.5–4.3)	0.124 (0.102–0.143)	478.9 (404.4–552.9)	-7.1 (-19.5–7.4)	-35.8 (-44.4—25.8)
7	Alzheimer's disease and other dementias	3.9 (1.0–9.8)	0.120 (0.0312–0.302)	396.5 (100.8–1004.3)	55.0 (36.6–77.3)	-1.5 (-12.7–11.9)
8	Chronic kidney disease	3.2 (2.8–3.4)	0.0978 (0.0820–0.113)	397.6 (337.3–457.8)	221.9 (177.2–270.0)	117.9 (88.0–149.9)
9	Self-harm	2.6 (2.3–2.8)	0.0797 (0.0667–0.0926)	589.1 (495.5–684.0)	10.0 (-8.5–27.1)	4.5 (-12.6–20.3)
10	Colon and rectum cancer	2.2 (1.9–2.4)	0.0682 (0.0559–0.0822)	297.6 (244.7–358.0)	-22.3 (-36.6—5.4)	-40.7 (-51.8—27.9)
11	Cirrhosis and other chronic liver diseases	2.1 (2.0–2.2)	0.0651 (0.0554–0.0762)	324.2 (276.7–378.6)	25.9 (7.3–47.4)	-6.2 (-20.2–10.1)
12	Diabetes mellitus	2.1 (1.9–2.2)	0.0640 (0.0540–0.0738)	283.2 (239.8–327.8)	-6.2 (-20.1–8.8)	-29.1 (-40.0—17.8)
13	Pancreatic cancer	1.7 (1.5–1.8)	0.0522 (0.0442–0.0605)	213.6 (180.9–248.0)	45.9 (24.0–67.3)	3.0 (-12.7–18.9)
14	Breast cancer	1.7 (1.3–2.0)	0.0512 (0.0407–0.0622)	236.2 (188.2–289.0)	-33.5 (-46.1—19.2)	-51.8 (-61.5—40.6)
15	Road injuries	1.6 (1.5–1.8)	0.0503 (0.0431–0.0582)	389.6 (335.6–447.5)	-50.3 (-57.9—42.5)	-54.5 (-61.2—47.8)
16	Lower respiratory infections	1.4 (1.2–1.5)	0.0428 (0.0347–0.0509)	188.8 (156.7–222.3)	-30.8 (-42.3—18.9)	-49.9 (-58.1—41.8)
17	Hypertensive heart disease	1.3 (1.2–1.5)	0.0410 (0.0347–0.0481)	180.7 (152.3–212.3)	99.1 (70.1–135.7)	49.4 (27.5–78.2)
18	Endocrine, metabolic, blood, and immune disorders	1.3 (1.2–1.3)	0.0390 (0.0333–0.0447)	204.4 (177.4–232.6)	157.1 (120.8–192.7)	83.9 (58.9–108.3)
19	Interpersonal violence	1.2 (1.1–1.3)	0.0380 (0.0324–0.0438)	342.3 (293.2–394.7)	-23.3 (-34.7—10.7)	-18.4 (-30.0—5.5)
20	Falls	1.2 (1.1–1.3)	0.0377 (0.0319–0.0436)	154.5 (131.9–179.1)	145.8 (111.3–183.8)	57.1 (35.3–81.1)
21	Neonatal disorders	1.2 (1.0–1.4)	0.0359 (0.0316–0.0407)	562.6 (495.9–637.8)	-54.3 (-60.1—48.1)	-38.8 (-46.5—30.4)
22	Cardiomyopathy and myocarditis	1.0 (0.9–1.1)	0.0312 (0.0262–0.0364)	153.0 (129.8–177.2)	-27.0 (-38.1—14.3)	-44.8 (-52.8—35.2)
23	Prostate cancer	1.0 (0.8–1.1)	0.0300 (0.0241–0.0368)	110.2 (88.1–135.3)	-19.3 (-35.8—2.0)	-42.2 (-54.5—29.7)
24	Leukaemia	0.9 (0.8–1.0)	0.0278 (0.0236–0.0317)	132.6 (114.4–151.0)	-12.7 (-25.1–0.1)	-37.8 (-46.0—29.5)
25	Non-Hodgkin lymphoma	0.8 (0.7–0.9)	0.0252 (0.0213–0.0289)	107.7 (91.3–123.6)	-22.8 (-34.1—11.7)	-46.4 (-54.0—38.6)

Table S2: Cause-specific YLLs by US state in 2021 and percentage change between 1990 and 2021, Rhode Island						
Rank	Cause Name	2021 Percentage of all cause YLLs	2021 YLLs (millions)	2021 Age Standardised Rate (per 100 000)	Percentage change YLL count 1990 to 2021	Percentage change age-standardised YLL rate 1990 to 2021
	All causes	100.0 (100.0–100.0)	0.209 (0.184–0.235)	12065.4 (10626.3–13618.9)	2.5 (-10.1–16.2)	-23.4 (-32.7—13.2)
1	Ischaemic heart disease	13.8 (12.1–14.7)	0.0287 (0.0241–0.0339)	1327.7 (1111.2–1571.6)	-42.6 (-50.6—33.4)	-60.9 (-66.6—54.2)
2	COVID-19	12.3 (10.9–13.8)	0.0255 (0.0255–0.0255)	1510.2 (1508.1–1509.1)	--	--
3	Tracheal, bronchus, and lung cancer	5.9 (5.3–6.4)	0.0123 (0.0104–0.0145)	593.3 (501.7–700.2)	-24.6 (-35.6—12.1)	-51.1 (-58.3—42.7)
4	Drug use disorders	5.6 (5.0–6.4)	0.0118 (0.00943–0.0143)	1008.6 (814.5–1220.3)	765.2 (587.7–972.7)	746.5 (576.4–937.5)
5	Chronic obstructive pulmonary disease	4.5 (4.0–4.8)	0.00936 (0.00785–0.0110)	424.1 (353.7–496.7)	45.7 (24.5–69.5)	1.4 (-13.3–18.7)
6	Alzheimer's disease and other dementias	4.3 (1.1–10.4)	0.00887 (0.00239–0.0233)	357.3 (96.3–950.7)	45.4 (29.4–66.9)	-1.7 (-12.7–13.0)
7	Stroke	3.7 (3.2–4.0)	0.00766 (0.00635–0.00901)	357.1 (298.1–421.1)	-17.3 (-28.7—5.0)	-42.6 (-50.5—33.8)
8	Chronic kidney disease	2.9 (2.6–3.1)	0.00604 (0.00508–0.00706)	293.5 (249.2–343.1)	194.7 (153.3–238.9)	99.4 (71.1–129.9)
9	Cirrhosis and other chronic liver diseases	2.6 (2.4–2.8)	0.00550 (0.00457–0.00656)	328.5 (273.9–390.7)	29.5 (6.8–56.2)	-6.7 (-23.5–12.8)
10	Self-harm	2.4 (2.2–2.6)	0.00493 (0.00407–0.00597)	404.3 (335.1–489.5)	-19.2 (-33.7—1.4)	-25.7 (-38.9—9.6)
11	Colon and rectum cancer	2.3 (2.0–2.5)	0.00479 (0.00400–0.00566)	242.9 (201.4–287.4)	-30.0 (-40.6—17.9)	-49.6 (-57.3—40.5)
12	Diabetes mellitus	2.1 (1.9–2.2)	0.00431 (0.00361–0.00516)	221.9 (186.4–265.6)	-5.5 (-20.2–12.1)	-31.8 (-42.5—18.7)
13	Pancreatic cancer	1.9 (1.7–2.0)	0.00387 (0.00328–0.00458)	188.4 (159.8–223.1)	38.7 (18.6–62.0)	-5.8 (-19.7–10.3)
14	Breast cancer	1.8 (1.5–2.1)	0.00369 (0.00301–0.00447)	200.1 (161.8–243.3)	-37.3 (-48.6—23.7)	-56.9 (-64.9—47.4)
15	Falls	1.6 (1.4–1.7)	0.00325 (0.00267–0.00381)	159.0 (132.7–186.0)	130.4 (94.9–165.7)	51.4 (28.5–74.9)
16	Hypertensive heart disease	1.6 (1.4–1.7)	0.00325 (0.00266–0.00388)	166.1 (136.9–199.8)	123.4 (84.5–165.3)	59.1 (30.3–90.5)
17	Road injuries	1.4 (1.3–1.5)	0.00293 (0.00243–0.00348)	262.4 (218.1–310.7)	-56.3 (-64.2—47.7)	-58.9 (-66.1—51.2)
18	Endocrine, metabolic, blood, and immune disorders	1.4 (1.3–1.5)	0.00293 (0.00248–0.00343)	180.6 (155.5–210.7)	144.1 (108.8–183.7)	71.5 (46.4–100.4)
19	Neonatal disorders	1.2 (1.0–1.6)	0.00257 (0.00209–0.00318)	483.6 (392.9–597.7)	-58.1 (-66.8—47.1)	-43.4 (-55.1—28.6)
20	Lower respiratory infections	1.2 (1.0–1.3)	0.00253 (0.00202–0.00304)	130.7 (106.2–155.9)	-45.5 (-54.7—36.3)	-61.3 (-68.2—54.9)
21	Liver cancer	1.1 (1.0–1.2)	0.00229 (0.00188–0.00276)	117.5 (97.4–141.4)	196.1 (140.8–261.7)	100.7 (63.9–144.5)
22	Prostate cancer	1.1 (0.9–1.2)	0.00221 (0.00172–0.00282)	99.1 (77.2–126.8)	-16.2 (-35.1–5.2)	-40.3 (-53.9—24.4)
23	Alcohol use disorders	1.0 (0.9–1.1)	0.00207 (0.00169–0.00251)	145.4 (119.6–175.9)	66.8 (33.7–107.3)	34.0 (7.7–66.2)
24	Brain and central nervous system cancer	0.9 (0.8–1.0)	0.00190 (0.00161–0.00224)	121.6 (102.6–142.5)	1.0 (-15.9–20.5)	-25.0 (-38.0—11.1)
25	Leukaemia	0.9 (0.8–1.0)	0.00189 (0.00160–0.00221)	108.8 (92.8–126.6)	-16.7 (-29.5—2.9)	-39.7 (-49.2—29.9)

Table S2: Cause-specific YLLs by US state in 2021 and percentage change between 1990 and 2021, South Carolina						
Rank	Cause Name	2021 Percentage of all cause YLLs	2021 YLLs (millions)	2021 Age Standardised Rate (per 100 000)	Percentage change YLL count 1990 to 2021	Percentage change age-standardised YLL rate 1990 to 2021
	All causes	100.0 (100.0–100.0)	1.44 (1.26–1.61)	19610.8 (17290.7–21915.7)	84.6 (61.9–107.9)	-3.7 (-15.0–7.8)
1	COVID-19	16.1 (13.1–20.3)	0.231 (0.201–0.284)	3081.6 (2689.4–3796.8)	--	--
2	Ischaemic heart disease	10.3 (9.4–11.1)	0.148 (0.125–0.170)	1688.5 (1433.3–1948.7)	-2.3 (-16.1–12.0)	-54.9 (-61.6—48.1)
3	Tracheal, bronchus, and lung cancer	5.1 (4.5–5.6)	0.0731 (0.0608–0.0848)	785.1 (653.3–913.7)	33.4 (11.8–57.4)	-42.0 (-51.5—31.7)
4	Chronic obstructive pulmonary disease	4.8 (4.3–5.2)	0.0687 (0.0580–0.0794)	710.1 (600.2–821.4)	204.8 (156.0–254.6)	32.2 (11.0–54.0)
5	Stroke	4.5 (4.0–4.8)	0.0642 (0.0542–0.0739)	728.6 (614.5–838.0)	32.6 (13.2–53.7)	-39.6 (-48.6—29.9)
6	Drug use disorders	4.0 (3.3–4.8)	0.0573 (0.0458–0.0723)	1104.7 (884.7–1381.8)	1744.5 (1319.9–2266.8)	1308.5 (990.7–1694.9)
7	Chronic kidney disease	3.5 (3.2–3.8)	0.0502 (0.0424–0.0575)	582.5 (493.1–665.7)	389.0 (315.5–462.0)	123.0 (90.5–154.8)
8	Road injuries	3.2 (2.9–3.4)	0.0456 (0.0388–0.0523)	894.1 (770.5–1023.2)	-13.5 (-26.3—0.1)	-37.4 (-46.2—28.0)
9	Self-harm	2.6 (2.4–2.9)	0.0377 (0.0316–0.0444)	718.6 (604.6–840.8)	77.2 (46.7–108.4)	30.7 (9.0–53.7)
10	Cirrhosis and other chronic liver diseases	2.5 (2.3–2.7)	0.0362 (0.0302–0.0425)	488.5 (406.4–574.2)	121.5 (86.4–160.6)	16.3 (-2.0–36.7)
11	Alzheimer's disease and other dementias	2.4 (0.6–6.4)	0.0349 (0.00890–0.0940)	352.3 (90.0–952.6)	153.6 (122.4–187.3)	-1.1 (-12.6–11.4)
12	Diabetes mellitus	2.2 (2.0–2.4)	0.0319 (0.0268–0.0371)	385.8 (324.3–448.2)	77.0 (49.2–108.5)	-13.4 (-27.1–1.4)
13	Colon and rectum cancer	2.0 (1.8–2.2)	0.0287 (0.0238–0.0340)	340.9 (284.4–403.8)	61.7 (35.0–92.2)	-22.3 (-35.2—7.4)
14	Interpersonal violence	1.8 (1.6–1.9)	0.0257 (0.0218–0.0298)	564.6 (479.7–653.0)	8.7 (-8.8–27.5)	-10.9 (-25.0–4.8)
15	Hypertensive heart disease	1.6 (1.4–1.8)	0.0229 (0.0193–0.0271)	283.3 (238.7–334.6)	219.9 (172.0–283.3)	58.0 (33.0–88.3)
16	Breast cancer	1.6 (1.3–1.9)	0.0228 (0.0186–0.0279)	284.1 (228.9–347.8)	28.1 (3.2–56.6)	-37.3 (-49.6—23.0)
17	Pancreatic cancer	1.5 (1.4–1.6)	0.0215 (0.0180–0.0248)	236.0 (198.1–273.1)	138.4 (101.7–175.9)	7.7 (-8.7–24.6)
18	Endocrine, metabolic, blood, and immune disorders	1.4 (1.3–1.5)	0.0205 (0.0175–0.0233)	290.8 (252.0–326.1)	298.8 (239.8–359.0)	106.7 (78.5–135.3)
19	Neonatal disorders	1.2 (1.0–1.4)	0.0169 (0.0149–0.0189)	608.0 (535.0–676.8)	-49.2 (-55.9—42.3)	-48.9 (-55.7—42.0)
20	Lower respiratory infections	1.1 (1.0–1.2)	0.0162 (0.0134–0.0189)	207.9 (173.4–241.1)	-5.8 (-22.4–10.1)	-54.1 (-61.6—46.7)
21	Falls	1.0 (0.9–1.1)	0.0141 (0.0119–0.0161)	168.9 (144.0–193.0)	266.6 (208.2–318.1)	69.5 (43.3–93.7)
22	Prostate cancer	1.0 (0.8–1.1)	0.0139 (0.0108–0.0174)	138.9 (108.3–174.7)	39.2 (9.4–76.1)	-40.3 (-52.9—24.9)
23	Cardiomyopathy and myocarditis	0.9 (0.8–1.0)	0.0134 (0.0113–0.0157)	187.5 (159.0–218.7)	6.5 (-10.7–25.3)	-42.2 (-51.0—32.0)
24	Alcohol use disorders	0.8 (0.7–0.9)	0.0113 (0.00930–0.0133)	179.3 (148.7–210.7)	78.6 (46.8–112.6)	9.4 (-9.9–29.6)
25	Leukaemia	0.8 (0.7–0.8)	0.0111 (0.00946–0.0128)	145.8 (126.0–166.3)	44.3 (22.8–66.9)	-27.5 (-37.8—16.7)

Table S2: Cause-specific YLLs by US state in 2021 and percentage change between 1990 and 2021, South Dakota						
Rank	Cause Name	2021 Percentage of all cause YLLs	2021 YLLs (millions)	2021 Age Standardised Rate (per 100 000)	Percentage change YLL count 1990 to 2021	Percentage change age-standardised YLL rate 1990 to 2021
	All causes	100.0 (100.0–100.0)	0.183 (0.165–0.202)	14497.5 (13113.7–15998.0)	33.8 (21.1–48.4)	-7.8 (-16.6–2.4)
1	Ischaemic heart disease	13.1 (11.8–14.0)	0.0240 (0.0208–0.0275)	1525.1 (1319.9–1752.7)	-24.4 (-33.9–-12.8)	-50.5 (-57.1–-42.5)
2	COVID-19	11.3 (10.2–12.5)	0.0206 (0.0206–0.0206)	1593.5 (1593.0–1594.1)	--	--
3	Tracheal, bronchus, and lung cancer	5.2 (4.7–5.7)	0.00956 (0.00818–0.0110)	616.3 (523.3–709.4)	13.0 (-3.2–31.5)	-34.1 (-43.4–-23.3)
4	Chronic obstructive pulmonary disease	4.9 (4.4–5.3)	0.00905 (0.00779–0.0103)	531.7 (460.8–608.0)	86.9 (61.6–115.0)	16.1 (0.3–33.6)
5	Self-harm	4.2 (3.9–4.5)	0.00770 (0.00661–0.00891)	907.2 (780.1–1052.2)	64.6 (39.7–92.1)	36.2 (15.4–59.1)
6	Alzheimer's disease and other dementias	3.9 (1.0–9.6)	0.00721 (0.00199–0.0181)	357.8 (95.6–912.4)	58.0 (43.0–78.5)	0.3 (-9.6–13.1)
7	Stroke	3.9 (3.4–4.2)	0.00711 (0.00608–0.00808)	447.6 (390.7–508.3)	-0.8 (-12.7–12.2)	-33.5 (-41.6–-24.2)
8	Road injuries	3.4 (3.2–3.6)	0.00623 (0.00544–0.00708)	751.6 (659.8–849.7)	-26.2 (-35.5–-15.9)	-39.4 (-47.0–-31.5)
9	Cirrhosis and other chronic liver diseases	3.0 (2.8–3.2)	0.00551 (0.00479–0.00635)	488.2 (423.3–561.8)	126.0 (95.6–160.8)	54.9 (33.8–79.7)
10	Chronic kidney disease	2.9 (2.6–3.2)	0.00537 (0.00463–0.00617)	348.5 (303.7–396.7)	330.0 (279.6–385.0)	170.8 (139.6–205.5)
11	Colon and rectum cancer	2.2 (2.0–2.4)	0.00410 (0.00348–0.00474)	283.4 (240.5–326.4)	2.7 (-11.4–19.0)	-32.6 (-42.0–-22.1)
12	Diabetes mellitus	2.2 (2.0–2.3)	0.00400 (0.00345–0.00458)	286.9 (247.8–328.5)	48.5 (29.1–70.4)	0.4 (-13.6–15.3)
13	Drug use disorders	1.7 (1.5–2.0)	0.00318 (0.00262–0.00386)	381.6 (313.9–462.8)	1348.8 (1049.9–1706.4)	1141.2 (887.4–1441.8)
14	Pancreatic cancer	1.7 (1.6–1.8)	0.00316 (0.00272–0.00362)	206.1 (177.9–236.2)	86.4 (62.3–115.8)	14.3 (-0.9–32.2)
15	Falls	1.7 (1.5–1.8)	0.00303 (0.00259–0.00345)	201.2 (175.2–227.8)	187.8 (153.4–224.1)	77.0 (56.0–99.8)
16	Breast cancer	1.6 (1.4–1.9)	0.00296 (0.00249–0.00351)	226.8 (190.8–268.8)	-1.7 (-18.7–17.6)	-36.6 (-47.6–-23.6)
17	Neonatal disorders	1.5 (1.2–1.8)	0.00267 (0.00219–0.00327)	496.9 (406.0–608.3)	-27.5 (-40.9–-10.3)	-25.4 (-39.2–-7.6)
18	Lower respiratory infections	1.5 (1.3–1.6)	0.00267 (0.00224–0.00314)	186.4 (157.8–216.1)	-30.2 (-39.9–-17.6)	-52.8 (-59.2–-45.0)
19	Congenital birth defects	1.4 (1.1–1.6)	0.00257 (0.00215–0.00302)	438.7 (364.1–518.9)	-20.8 (-34.3–-5.5)	-22.7 (-36.2–-6.8)
20	Endocrine, metabolic, blood, and immune disorders	1.3 (1.2–1.3)	0.00231 (0.00202–0.00262)	198.5 (176.3–222.2)	240.7 (199.0–287.2)	124.3 (97.7–153.7)
21	Hypertensive heart disease	1.2 (1.1–1.4)	0.00227 (0.00192–0.00264)	152.3 (130.9–177.2)	211.5 (168.1–261.4)	102.7 (72.8–137.2)
22	Alcohol use disorders	1.1 (1.0–1.2)	0.00203 (0.00175–0.00236)	202.6 (175.1–234.8)	148.8 (108.6–194.4)	82.2 (52.7–115.5)
23	Brain and central nervous system cancer	1.0 (1.0–1.1)	0.00185 (0.00163–0.00211)	163.7 (145.1–185.2)	40.7 (21.7–61.7)	-4.2 (-16.5–9.4)
24	Prostate cancer	1.0 (0.8–1.2)	0.00184 (0.00150–0.00233)	104.5 (84.5–132.7)	-8.0 (-26.2–15.5)	-42.0 (-53.6–-27.0)
25	Leukaemia	1.0 (0.9–1.0)	0.00180 (0.00157–0.00205)	141.6 (124.7–159.7)	10.3 (-3.9–26.2)	-26.6 (-35.8–-16.2)

Table S2: Cause-specific YLLs by US state in 2021 and percentage change between 1990 and 2021, Tennessee						
Rank	Cause Name	2021 Percentage of all cause YLLs	2021 YLLs (millions)	2021 Age Standardised Rate (per 100 000)	Percentage change YLL count 1990 to 2021	Percentage change age-standardised YLL rate 1990 to 2021
	All causes	100.0 (100.0–100.0)	2.02 (1.79–2.26)	20686.0 (18403.7–23111.2)	81.9 (60.8–103.8)	6.8 (-5.2–19.0)
1	COVID-19	15.7 (13.3–18.7)	0.316 (0.293–0.378)	3190.0 (2954.6–3806.1)	--	--
2	Ischaemic heart disease	12.4 (11.3–13.3)	0.250 (0.215–0.292)	2149.6 (1835.8–2511.6)	0.2 (-14.0–16.0)	-45.6 (-53.5–-36.6)
3	Tracheal, bronchus, and lung cancer	5.4 (4.9–5.9)	0.108 (0.0928–0.127)	894.6 (770.2–1054.5)	19.1 (0.8–40.3)	-40.4 (-49.3–-29.5)
4	Drug use disorders	5.2 (4.3–6.3)	0.105 (0.0813–0.130)	1491.9 (1165.5–1831.3)	2414.9 (1783.9–3141.8)	1871.6 (1380.6–2421.1)
5	Chronic obstructive pulmonary disease	5.2 (4.7–5.5)	0.104 (0.0899–0.119)	831.0 (716.5–952.0)	174.2 (137.1–211.9)	41.5 (22.1–61.4)
6	Stroke	4.0 (3.6–4.3)	0.0799 (0.0684–0.0919)	690.3 (593.0–797.0)	24.8 (9.2–43.1)	-31.3 (-39.9–-21.2)
7	Chronic kidney disease	2.9 (2.6–3.1)	0.0579 (0.0486–0.0673)	505.6 (425.5–588.4)	386.0 (314.4–461.8)	156.4 (117.1–198.8)
8	Self-harm	2.7 (2.5–3.0)	0.0552 (0.0472–0.0646)	762.3 (654.6–892.7)	71.0 (45.4–103.0)	27.4 (8.2–50.7)
9	Road injuries	2.6 (2.4–2.8)	0.0531 (0.0457–0.0614)	765.8 (663.5–877.9)	-20.1 (-31.5–-7.8)	-42.0 (-50.1–-33.3)
10	Cirrhosis and other chronic liver diseases	2.6 (2.4–2.8)	0.0522 (0.0442–0.0610)	531.0 (448.1–620.7)	160.9 (120.7–204.2)	52.7 (29.0–78.3)
11	Alzheimer's disease and other dementias	2.3 (0.6–5.9)	0.0457 (0.0116–0.123)	350.1 (88.8–938.1)	93.5 (71.5–118.7)	-1.8 (-13.3–10.5)
12	Diabetes mellitus	2.1 (1.9–2.3)	0.0420 (0.0360–0.0486)	388.5 (333.6–449.7)	91.4 (62.5–122.6)	9.2 (-7.2–27.5)
13	Hypertensive heart disease	2.0 (1.8–2.3)	0.0407 (0.0334–0.0478)	382.0 (316.5–448.8)	237.4 (178.5–300.7)	95.2 (60.6–131.5)
14	Colon and rectum cancer	2.0 (1.7–2.3)	0.0404 (0.0338–0.0482)	363.2 (305.7–432.8)	45.8 (19.7–77.4)	-17.8 (-32.4–-0.2)
15	Interpersonal violence	1.6 (1.5–1.8)	0.0325 (0.0278–0.0381)	523.2 (451.6–609.5)	0.5 (-15.0–18.1)	-17.3 (-29.6–-3.2)
16	Breast cancer	1.5 (1.2–1.7)	0.0294 (0.0238–0.0356)	273.7 (220.8–332.5)	16.0 (-6.4–43.4)	-36.7 (-49.2–-21.0)
17	Lower respiratory infections	1.4 (1.3–1.6)	0.0287 (0.0238–0.0341)	274.7 (231.8–323.3)	-3.1 (-19.3–14.2)	-44.9 (-53.7–-35.9)
18	Pancreatic cancer	1.3 (1.2–1.4)	0.0269 (0.0230–0.0314)	228.2 (194.9–266.5)	105.5 (76.8–141.3)	8.6 (-6.8–27.9)
19	Endocrine, metabolic, blood, and immune disorders	1.3 (1.2–1.4)	0.0261 (0.0227–0.0301)	278.7 (243.3–319.0)	317.8 (260.2–379.9)	134.7 (103.2–166.0)
20	Neonatal disorders	1.1 (0.9–1.3)	0.0223 (0.0197–0.0253)	566.0 (500.5–641.1)	-36.3 (-45.1–-27.3)	-40.8 (-49.0–-32.5)
21	Falls	1.0 (0.9–1.1)	0.0203 (0.0175–0.0235)	184.8 (159.5–212.4)	217.5 (176.3–261.2)	69.2 (47.9–92.6)
22	Prostate cancer	0.8 (0.7–0.9)	0.0164 (0.0129–0.0203)	126.8 (99.7–157.4)	28.4 (1.0–59.0)	-33.0 (-47.3–-17.0)
23	Congenital birth defects	0.8 (0.7–0.9)	0.0153 (0.0136–0.0170)	338.9 (300.9–376.5)	-21.1 (-30.8–-10.9)	-33.3 (-41.6–-24.5)
24	Alcohol use disorders	0.8 (0.7–0.8)	0.0152 (0.0129–0.0180)	179.1 (152.3–211.1)	168.1 (126.1–219.5)	76.9 (50.0–110.8)
25	Brain and central nervous system cancer	0.7 (0.7–0.8)	0.0150 (0.0129–0.0174)	162.6 (142.0–187.0)	54.2 (32.5–78.2)	-8.3 (-20.7–5.7)

Table S2: Cause-specific YLLs by US state in 2021 and percentage change between 1990 and 2021, Texas						
Rank	Cause Name	2021 Percentage of all cause YLLs	2021 YLLs (millions)	2021 Age Standardised Rate (per 100 000)	Percentage change YLL count 1990 to 2021	Percentage change age-standardised YLL rate 1990 to 2021
	All causes	100.0 (100.0–100.0)	6.08 (5.45–6.73)	16351.8 (14706.3–18031.5)	90.1 (69.7–110.2)	-8.6 (-18.1–0.9)
1	COVID-19	18.5 (16.2–21.6)	1.12 (1.07–1.31)	2999.2 (2861.6–3492.0)	--	--
2	Ischaemic heart disease	11.2 (10.2–12.1)	0.683 (0.591–0.787)	1629.5 (1409.1–1876.3)	11.8 (-2.7–28.0)	-51.6 (-57.9–-44.6)
3	Stroke	4.1 (3.7–4.4)	0.248 (0.216–0.284)	598.5 (521.9–684.3)	59.7 (39.3–82.0)	-29.3 (-38.2–-19.2)
4	Chronic obstructive pulmonary disease	3.9 (3.5–4.2)	0.239 (0.206–0.269)	544.2 (470.2–614.9)	146.2 (113.5–181.2)	4.1 (-9.6–19.1)
5	Tracheal, bronchus, and lung cancer	3.9 (3.5–4.2)	0.236 (0.203–0.272)	543.0 (467.9–625.2)	3.2 (-12.0–19.4)	-58.0 (-64.2–-51.3)
6	Chronic kidney disease	3.4 (3.1–3.6)	0.205 (0.179–0.235)	498.6 (437.4–568.6)	512.6 (437.2–593.7)	168.7 (135.6–205.0)
7	Road injuries	3.2 (3.0–3.4)	0.192 (0.168–0.218)	643.3 (567.2–728.1)	-0.4 (-13.1–13.4)	-40.5 (-47.9–-32.7)
8	Self-harm	3.1 (2.9–3.4)	0.191 (0.165–0.221)	621.7 (539.0–716.2)	74.5 (50.3–102.0)	4.7 (-9.6–21.0)
9	Cirrhosis and other chronic liver diseases	3.0 (2.8–3.3)	0.184 (0.157–0.215)	471.4 (403.8–550.1)	159.5 (120.3–203.6)	17.9 (0.1–37.8)
10	Drug use disorders	2.7 (2.3–3.2)	0.165 (0.133–0.205)	527.6 (428.2–654.8)	858.4 (660.8–1111.3)	500.0 (377.8–655.8)
11	Alzheimer's disease and other dementias	2.5 (0.6–6.3)	0.153 (0.0394–0.391)	349.6 (90.3–897.4)	130.4 (105.6–160.0)	-1.9 (-12.5–10.4)
12	Colon and rectum cancer	2.2 (1.9–2.4)	0.131 (0.112–0.152)	320.3 (274.1–370.3)	78.0 (51.2–110.4)	-21.4 (-33.2–-7.2)
13	Diabetes mellitus	2.1 (1.9–2.2)	0.126 (0.109–0.146)	311.3 (269.8–360.1)	61.1 (39.5–85.1)	-28.1 (-37.5–-17.6)
14	Hypertensive heart disease	1.8 (1.6–2.1)	0.111 (0.0918–0.134)	278.4 (229.8–334.3)	238.7 (176.9–310.4)	52.4 (24.9–84.4)
15	Breast cancer	1.6 (1.3–1.9)	0.0966 (0.0777–0.117)	242.7 (194.4–294.8)	38.2 (10.4–68.2)	-38.4 (-50.9–-24.8)
16	Interpersonal violence	1.6 (1.4–1.7)	0.0944 (0.0829–0.107)	340.2 (299.9–382.4)	-31.4 (-40.4–-21.4)	-54.6 (-60.2–-48.3)
17	Neonatal disorders	1.5 (1.3–1.7)	0.0887 (0.0789–0.0990)	498.6 (443.8–556.8)	-14.3 (-24.6–-3.6)	-26.5 (-35.4–-17.3)
18	Pancreatic cancer	1.4 (1.3–1.5)	0.0860 (0.0744–0.0990)	201.0 (174.1–231.2)	135.9 (104.8–172.8)	-1.0 (-13.9–14.5)
19	Endocrine, metabolic, blood, and immune disorders	1.3 (1.2–1.4)	0.0800 (0.0705–0.0910)	218.7 (194.0–247.7)	292.9 (243.2–348.5)	86.1 (63.1–111.8)
20	Lower respiratory infections	1.2 (1.1–1.3)	0.0721 (0.0607–0.0850)	189.6 (160.9–222.0)	-5.5 (-19.6–10.7)	-55.2 (-61.7–-47.7)
21	Congenital birth defects	1.0 (0.9–1.1)	0.0596 (0.0537–0.0657)	299.5 (268.8–331.2)	-27.4 (-35.0–-18.4)	-43.6 (-49.8–-36.5)
22	Falls	0.9 (0.8–1.0)	0.0539 (0.0469–0.0620)	133.9 (116.6–153.7)	203.5 (164.9–244.9)	37.8 (20.4–56.1)
23	Liver cancer	0.9 (0.8–0.9)	0.0520 (0.0442–0.0600)	124.3 (106.1–143.3)	293.5 (232.8–353.4)	68.1 (42.8–93.7)
24	Prostate cancer	0.8 (0.7–1.0)	0.0493 (0.0402–0.0608)	111.9 (91.1–137.8)	43.4 (15.1–75.9)	-38.8 (-50.8–-24.8)
25	Cardiomyopathy and myocarditis	0.8 (0.7–0.8)	0.0462 (0.0397–0.0536)	129.4 (112.4–149.3)	2.9 (-12.5–19.4)	-48.6 (-56.0–-40.6)

Table S2: Cause-specific YLLs by US state in 2021 and percentage change between 1990 and 2021, Utah						
Rank	Cause Name	2021 Percentage of all cause YLLs	2021 YLLs (millions)	2021 Age Standardised Rate (per 100 000)	Percentage change YLL count 1990 to 2021	Percentage change age-standardised YLL rate 1990 to 2021
	All causes	100.0 (100.0–100.0)	0.504 (0.446–0.564)	13471.5 (11927.0–15057.3)	117.4 (91.4–143.3)	-4.3 (-15.6–6.7)
1	COVID-19	13.7 (11.6–16.7)	0.0690 (0.0638–0.0827)	1820.9 (1681.9–2181.1)	--	--
2	Ischaemic heart disease	8.5 (7.6–9.2)	0.0428 (0.0360–0.0495)	1050.4 (881.7–1216.1)	11.1 (-5.0–27.8)	-57.0 (-63.4–-50.6)
3	Self-harm	6.1 (5.6–6.6)	0.0309 (0.0261–0.0361)	893.5 (757.0–1043.7)	115.7 (81.9–153.5)	9.3 (-8.0–28.3)
4	Drug use disorders	5.7 (5.1–6.5)	0.0290 (0.0240–0.0347)	851.2 (705.0–1017.0)	2772.0 (2240.6–3386.7)	1374.7 (1098.0–1683.4)
5	Chronic obstructive pulmonary disease	4.0 (3.6–4.3)	0.0201 (0.0172–0.0230)	478.4 (410.8–546.9)	178.4 (141.2–218.1)	7.4 (-6.8–22.7)
6	Stroke	3.9 (3.5–4.2)	0.0195 (0.0168–0.0223)	480.1 (415.5–549.6)	67.2 (47.0–90.8)	-34.1 (-42.1–-24.7)
7	Chronic kidney disease	3.1 (2.8–3.3)	0.0156 (0.0134–0.0178)	388.1 (336.6–442.3)	573.2 (484.7–674.9)	171.7 (136.1–212.6)
8	Alzheimer's disease and other dementias	2.9 (0.7–7.4)	0.0144 (0.00366–0.0379)	345.8 (87.6–902.7)	165.5 (137.3–197.8)	-0.3 (-10.8–11.9)
9	Tracheal, bronchus, and lung cancer	2.6 (2.4–2.9)	0.0133 (0.0113–0.0155)	320.5 (272.8–373.9)	49.7 (26.3–78.0)	-44.0 (-53.0–-33.3)
10	Road injuries	2.5 (2.4–2.7)	0.0128 (0.0110–0.0148)	375.1 (325.5–433.4)	-19.2 (-30.4–-6.7)	-57.3 (-63.1–-50.9)
11	Diabetes mellitus	2.5 (2.2–2.7)	0.0125 (0.0106–0.0145)	313.6 (267.1–365.1)	93.5 (65.0–126.7)	-22.3 (-33.7–-8.9)
12	Cirrhosis and other chronic liver diseases	2.4 (2.2–2.6)	0.0121 (0.0103–0.0140)	320.5 (272.9–372.0)	165.6 (123.1–211.2)	9.8 (-7.9–28.8)
13	Neonatal disorders	2.1 (1.7–2.6)	0.0106 (0.00874–0.0128)	456.2 (376.9–551.2)	36.0 (8.5–66.9)	5.2 (-16.1–29.1)
14	Colon and rectum cancer	1.8 (1.6–2.1)	0.00921 (0.00768–0.0109)	230.3 (192.8–273.7)	78.4 (49.3–112.4)	-30.0 (-41.6–-16.6)
15	Breast cancer	1.7 (1.5–2.0)	0.00877 (0.00754–0.0103)	225.3 (193.2–265.9)	65.3 (39.4–96.1)	-34.8 (-45.3–-22.7)
16	Congenital birth defects	1.6 (1.4–2.0)	0.00818 (0.00700–0.00956)	327.5 (277.2–387.7)	-11.3 (-26.1–4.5)	-36.0 (-47.2–-24.0)
17	Endocrine, metabolic, blood, and immune disorders	1.5 (1.4–1.6)	0.00739 (0.00644–0.00840)	200.9 (175.7–227.5)	316.9 (263.2–373.8)	90.5 (66.6–115.5)
18	Pancreatic cancer	1.4 (1.3–1.6)	0.00726 (0.00622–0.00833)	177.1 (151.8–203.2)	202.2 (160.5–252.9)	15.7 (-0.2–34.8)
19	Falls	1.4 (1.3–1.5)	0.00717 (0.00614–0.00822)	181.1 (155.0–207.5)	288.6 (238.8–346.0)	61.7 (41.6–85.2)
20	Hypertensive heart disease	1.4 (1.2–1.5)	0.00697 (0.00600–0.00814)	177.3 (152.7–207.1)	267.3 (212.4–331.4)	43.8 (22.4–69.5)
21	Lower respiratory infections	1.3 (1.1–1.4)	0.00643 (0.00546–0.00751)	168.2 (143.2–195.8)	-9.3 (-22.3–5.2)	-61.4 (-66.8–-55.2)
22	Alcohol use disorders	1.2 (1.1–1.3)	0.00590 (0.00493–0.00699)	165.5 (138.8–196.0)	231.0 (176.7–294.3)	47.5 (22.7–75.2)
23	Prostate cancer	1.1 (0.9–1.3)	0.00551 (0.00430–0.00689)	130.4 (101.8–162.9)	63.5 (27.7–107.1)	-36.6 (-50.5–-19.6)
24	Brain and central nervous system cancer	0.9 (0.9–1.0)	0.00465 (0.00405–0.00534)	125.5 (109.6–143.6)	93.3 (67.1–122.0)	-13.8 (-25.2–-1.2)
25	Leukaemia	0.9 (0.8–1.0)	0.00456 (0.00395–0.00526)	118.6 (102.9–135.8)	53.3 (33.4–76.4)	-33.0 (-41.6–-23.3)

Table S2: Cause-specific YLLs by US state in 2021 and percentage change between 1990 and 2021, Vermont						
Rank	Cause Name	2021 Percentage of all cause YLLs	2021 YLLs (millions)	2021 Age Standardised Rate (per 100 000)	Percentage change YLL count 1990 to 2021	Percentage change age-standardised YLL rate 1990 to 2021
	All causes	100.0 (100.0–100.0)	0.133 (0.121–0.149)	12802.6 (11617.1–14241.9)	33.8 (20.6–50.0)	-16.8 (-25.1–-7.4)
1	Ischaemic heart disease	13.0 (11.6–13.9)	0.0174 (0.0148–0.0198)	1310.0 (1120.6–1500.9)	-16.4 (-26.1–-4.9)	-55.8 (-61.3–-49.4)
2	COVID-19	8.9 (5.7–11.9)	0.0119 (0.00730–0.0160)	1145.1 (705.1–1542.7)	--	--
3	Tracheal, bronchus, and lung cancer	5.8 (5.2–6.4)	0.00778 (0.00669–0.00908)	593.3 (511.0–695.3)	2.6 (-12.5–19.9)	-48.6 (-56.2–-39.9)
4	Chronic obstructive pulmonary disease	5.3 (4.7–5.7)	0.00702 (0.00602–0.00789)	493.1 (426.2–554.2)	75.7 (55.0–96.9)	-11.9 (-22.2–-1.1)
5	Drug use disorders	4.1 (3.6–4.7)	0.00547 (0.00454–0.00657)	892.4 (743.1–1077.5)	1108.3 (846.5–1439.0)	1163.2 (886.5–1500.0)
6	Alzheimer's disease and other dementias	4.1 (1.0–9.9)	0.00543 (0.00137–0.0138)	353.8 (88.9–900.4)	94.2 (76.6–116.6)	-1.9 (-11.1–9.4)
7	Stroke	3.8 (3.3–4.1)	0.00500 (0.00426–0.00570)	377.4 (325.3–429.2)	5.3 (-6.2–19.8)	-43.0 (-49.4–-35.0)
8	Self-harm	3.7 (3.4–3.9)	0.00488 (0.00426–0.00566)	750.5 (656.1–865.9)	21.1 (4.2–40.7)	15.0 (-0.7–32.5)
9	Chronic kidney disease	2.6 (2.3–2.9)	0.00350 (0.00304–0.00411)	272.6 (237.8–315.8)	289.1 (242.6–350.3)	109.4 (86.0–140.5)
10	Colon and rectum cancer	2.4 (2.1–2.6)	0.00318 (0.00274–0.00373)	259.4 (224.2–305.2)	5.7 (-10.0–24.9)	-41.6 (-50.2–-31.2)
11	Cirrhosis and other chronic liver diseases	2.4 (2.2–2.5)	0.00318 (0.00279–0.00367)	325.3 (286.3–375.7)	62.0 (41.2–90.4)	4.4 (-9.3–22.6)
12	Road injuries	2.2 (2.1–2.4)	0.00296 (0.00262–0.00338)	492.4 (436.9–558.9)	-43.4 (-50.5–-35.0)	-45.5 (-52.1–-37.8)
13	Diabetes mellitus	2.0 (1.8–2.2)	0.00271 (0.00236–0.00313)	233.4 (205.4–267.9)	19.8 (3.8–37.7)	-30.2 (-39.5–-19.7)
14	Falls	2.0 (1.7–2.1)	0.00261 (0.00224–0.00296)	211.1 (184.9–238.4)	209.3 (175.0–248.8)	69.4 (50.7–91.6)
15	Pancreatic cancer	1.8 (1.7–2.0)	0.00243 (0.00208–0.00279)	186.1 (161.1–212.2)	85.8 (61.5–114.2)	-5.0 (-17.4–9.6)
16	Hypertensive heart disease	1.8 (1.6–1.9)	0.00239 (0.00206–0.00272)	200.5 (175.3–229.4)	180.7 (148.0–218.1)	61.1 (41.8–83.9)
17	Endocrine, metabolic, blood, and immune disorders	1.8 (1.6–1.9)	0.00237 (0.00211–0.00269)	248.0 (222.0–278.3)	228.9 (191.3–275.5)	107.0 (83.9–135.6)
18	Breast cancer	1.7 (1.5–2.0)	0.00232 (0.00198–0.00272)	209.3 (178.2–245.6)	-9.5 (-23.2–5.9)	-46.8 (-55.0–-37.3)
19	Prostate cancer	1.2 (1.0–1.4)	0.00158 (0.00127–0.00192)	107.6 (86.0–130.6)	4.2 (-18.1–27.8)	-48.2 (-59.4–-36.5)
20	Alcohol use disorders	1.1 (1.0–1.2)	0.00145 (0.00126–0.00168)	179.2 (157.2–206.4)	125.8 (91.5–163.6)	76.1 (50.5–104.0)
21	Lower respiratory infections	1.0 (0.9–1.1)	0.00137 (0.00115–0.00160)	121.2 (105.5–140.6)	-46.0 (-53.1–-36.8)	-66.8 (-71.2–-61.6)
22	Parkinson's disease	1.0 (0.9–1.1)	0.00136 (0.00113–0.00156)	92.3 (77.0–105.4)	174.3 (145.7–208.5)	40.7 (25.9–58.4)
23	Leukaemia	0.9 (0.9–1.0)	0.00126 (0.00111–0.00143)	118.8 (106.4–133.2)	6.7 (-6.4–20.4)	-36.1 (-43.6–-28.4)
24	Brain and central nervous system cancer	0.9 (0.9–1.0)	0.00122 (0.00108–0.00140)	131.7 (118.1–148.9)	35.9 (19.2–54.1)	-10.8 (-20.9–1.1)
25	Non-rheumatic valvular heart disease	0.9 (0.8–1.0)	0.00122 (0.00101–0.00140)	86.6 (72.2–98.5)	39.5 (24.5–56.6)	-28.0 (-35.7–-19.1)

Table S2: Cause-specific YLLs by US state in 2021 and percentage change between 1990 and 2021, Virginia

Rank	Cause Name	2021 Percentage of all cause YLLs	2021 YLLs (millions)	2021 Age Standardised Rate (per 100 000)	Percentage change YLL count 1990 to 2021	Percentage change age-standardised YLL rate 1990 to 2021
	All causes	100.0 (100.0–100.0)	1.80 (1.58–2.04)	14731.3 (13015.8–16583.7)	52.9 (33.9–73.3)	-15.7 (-25.8—5.0)
1	COVID-19	13.8 (11.6–17.4)	0.248 (0.229–0.306)	2017.8 (1869.3–2493.3)	--	--
2	Ischaemic heart disease	11.1 (10.1–12.0)	0.199 (0.168–0.231)	1379.7 (1156.2–1601.8)	-13.5 (-27.8–0.5)	-57.9 (-65.0—51.0)
3	Tracheal, bronchus, and lung cancer	5.3 (4.8–5.7)	0.0947 (0.0795–0.112)	641.0 (537.6–756.7)	0.0 (-16.3–18.2)	-52.8 (-60.6—44.2)
4	Chronic obstructive pulmonary disease	4.5 (4.0–4.9)	0.0812 (0.0684–0.0943)	529.1 (444.9–613.8)	114.2 (82.2–149.7)	0.2 (-14.9–17.3)
5	Stroke	4.5 (4.0–4.8)	0.0808 (0.0682–0.0934)	554.1 (468.6–641.9)	26.7 (7.5–47.1)	-38.8 (-48.1—28.9)
6	Drug use disorders	3.8 (3.3–4.4)	0.0687 (0.0557–0.0858)	763.8 (619.9–950.5)	1283.0 (1000.3–1650.3)	1038.1 (806.4–1331.3)
7	Chronic kidney disease	3.6 (3.2–3.9)	0.0651 (0.0548–0.0758)	458.4 (390.1–533.8)	350.5 (283.5–422.7)	118.0 (86.1–152.2)
8	Alzheimer's disease and other dementias	3.2 (0.8–8.1)	0.0569 (0.0139–0.144)	352.2 (86.5–893.0)	128.4 (101.4–163.7)	-1.9 (-14.0–12.6)
9	Self-harm	3.0 (2.7–3.3)	0.0538 (0.0447–0.0634)	596.3 (497.7–700.9)	38.1 (14.5–64.8)	7.2 (-10.5–27.4)
10	Cirrhosis and other chronic liver diseases	2.5 (2.3–2.7)	0.0448 (0.0374–0.0526)	355.3 (297.6–416.3)	97.4 (63.1–132.8)	8.9 (-10.2–28.9)
11	Diabetes mellitus	2.4 (2.2–2.6)	0.0441 (0.0372–0.0517)	323.1 (272.4–377.7)	97.2 (65.2–133.0)	1.8 (-14.9–20.6)
12	Colon and rectum cancer	2.3 (2.0–2.5)	0.0409 (0.0342–0.0484)	295.9 (247.2–351.1)	26.5 (4.2–50.4)	-35.5 (-47.3—23.1)
13	Road injuries	2.2 (2.1–2.4)	0.0405 (0.0340–0.0471)	460.5 (390.5–530.6)	-31.2 (-41.7—19.8)	-49.2 (-56.6—41.3)
14	Breast cancer	1.8 (1.5–2.1)	0.0329 (0.0266–0.0401)	248.1 (200.2–303.7)	1.9 (-18.1–25.7)	-46.4 (-57.3—34.1)
15	Pancreatic cancer	1.7 (1.5–1.8)	0.0300 (0.0254–0.0355)	205.9 (173.2–242.8)	102.2 (70.8–138.3)	-2.4 (-17.6–14.9)
16	Endocrine, metabolic, blood, and immune disorders	1.4 (1.3–1.5)	0.0250 (0.0211–0.0286)	212.3 (181.4–241.6)	276.0 (217.5–331.7)	106.1 (75.8–134.4)
17	Interpersonal violence	1.4 (1.3–1.5)	0.0250 (0.0212–0.0289)	322.0 (276.5–370.9)	-25.4 (-37.1—13.1)	-35.3 (-44.9—25.1)
18	Neonatal disorders	1.4 (1.1–1.6)	0.0245 (0.0217–0.0277)	542.6 (479.4–612.2)	-49.8 (-55.7—42.5)	-45.6 (-52.0—37.7)
19	Lower respiratory infections	1.3 (1.2–1.4)	0.0235 (0.0196–0.0277)	182.2 (154.1–212.6)	-25.9 (-38.4—12.3)	-61.3 (-67.6—54.6)
20	Hypertensive heart disease	1.3 (1.1–1.4)	0.0228 (0.0191–0.0272)	168.0 (142.3–200.7)	153.3 (111.7–205.4)	31.0 (9.0–57.4)
21	Falls	1.2 (1.0–1.3)	0.0209 (0.0176–0.0242)	150.3 (128.0–173.9)	201.2 (157.1–248.9)	49.2 (27.2–72.2)
22	Cardiomyopathy and myocarditis	1.0 (0.9–1.1)	0.0185 (0.0155–0.0219)	149.0 (126.2–174.9)	-0.9 (-18.6–18.2)	-45.2 (-54.5—35.0)
23	Prostate cancer	1.0 (0.8–1.2)	0.0181 (0.0142–0.0222)	116.2 (90.6–142.8)	14.5 (-11.8–41.1)	-46.6 (-59.0—34.2)
24	Leukaemia	0.9 (0.8–0.9)	0.0159 (0.0135–0.0183)	128.4 (109.8–146.2)	16.3 (-0.8–34.3)	-37.2 (-46.0—27.8)
25	Alcohol use disorders	0.8 (0.8–0.9)	0.0150 (0.0123–0.0179)	138.4 (113.3–164.5)	115.4 (74.8–158.5)	39.0 (13.1–65.8)

Table S2: Cause-specific YLLs by US state in 2021 and percentage change between 1990 and 2021, Washington

Rank	Cause Name	2021 Percentage of all cause YLLs	2021 YLLs (millions)	2021 Age Standardised Rate (per 100 000)	Percentage change YLL count 1990 to 2021	Percentage change age-standardised YLL rate 1990 to 2021
	All causes	100.0 (100.0–100.0)	1.40 (1.21–1.58)	12523.6 (10943.3–14065.7)	64.6 (42.7–85.8)	-18.7 (-29.3—8.7)
1	COVID-19	10.8 (8.8–14.2)	0.151 (0.137–0.201)	1358.3 (1232.6–1804.7)	--	--
2	Ischaemic heart disease	10.5 (9.4–11.4)	0.148 (0.124–0.172)	1095.7 (919.7–1272.6)	-9.1 (-23.3–5.7)	-60.1 (-66.4—53.4)
3	Tracheal, bronchus, and lung cancer	5.1 (4.6–5.6)	0.0717 (0.0599–0.0846)	524.8 (437.4–618.8)	9.6 (-8.6–29.7)	-54.7 (-62.6—46.4)
4	Drug use disorders	4.6 (3.9–5.5)	0.0650 (0.0513–0.0817)	801.6 (632.3–1001.8)	1100.8 (833.0–1448.3)	744.4 (557.9–987.1)
5	Chronic obstructive pulmonary disease	4.6 (4.1–4.9)	0.0638 (0.0534–0.0745)	450.2 (377.0–526.2)	81.8 (52.1–111.9)	-22.3 (-35.1—9.2)
6	Stroke	4.1 (3.6–4.5)	0.0581 (0.0479–0.0671)	433.6 (361.4–501.3)	31.5 (11.9–50.6)	-41.1 (-49.8—32.6)
7	Self-harm	4.0 (3.6–4.4)	0.0562 (0.0472–0.0657)	695.0 (585.2–810.6)	58.9 (31.6–87.0)	5.3 (-12.4–24.1)
8	Alzheimer's disease and other dementias	3.7 (1.0–9.2)	0.0518 (0.0135–0.133)	352.8 (92.6–904.9)	133.3 (105.0–166.1)	-0.9 (-13.2–13.2)
9	Cirrhosis and other chronic liver diseases	3.1 (2.9–3.4)	0.0440 (0.0362–0.0516)	394.9 (325.5–461.6)	166.0 (119.6–214.4)	31.5 (8.8–55.6)
10	Chronic kidney disease	2.8 (2.5–3.0)	0.0387 (0.0325–0.0450)	295.8 (249.2–342.5)	452.5 (368.3–536.4)	145.0 (107.7–181.2)
11	Colon and rectum cancer	2.2 (1.9–2.4)	0.0304 (0.0252–0.0365)	239.9 (198.3–287.3)	43.0 (16.6–75.0)	-34.3 (-46.5—19.4)
12	Road injuries	2.1 (2.0–2.3)	0.0301 (0.0253–0.0346)	386.5 (328.5–441.0)	-36.8 (-46.7—27.4)	-59.9 (-65.9—53.9)
13	Diabetes mellitus	2.1 (1.9–2.3)	0.0297 (0.0250–0.0349)	237.9 (200.6–278.2)	71.8 (43.7–101.8)	-20.7 (-33.7—7.0)
14	Breast cancer	1.8 (1.5–2.2)	0.0254 (0.0204–0.0316)	210.0 (167.4–262.7)	22.6 (-1.8–52.0)	-43.4 (-54.6—29.6)
15	Pancreatic cancer	1.8 (1.6–1.9)	0.0248 (0.0208–0.0287)	184.8 (155.2–213.6)	127.0 (89.6–162.2)	-1.9 (-18.4–13.7)
16	Hypertensive heart disease	1.6 (1.4–1.7)	0.0221 (0.0182–0.0261)	173.8 (144.6–205.5)	261.5 (204.7–321.9)	66.2 (39.8–93.8)
17	Endocrine, metabolic, blood, and immune disorders	1.6 (1.4–1.7)	0.0219 (0.0186–0.0251)	202.1 (173.4–229.3)	314.1 (253.0–378.5)	98.7 (70.8–127.3)
18	Falls	1.5 (1.3–1.6)	0.0208 (0.0174–0.0242)	164.1 (138.0–189.8)	238.9 (186.6–291.9)	50.7 (28.0–73.0)
19	Lower respiratory infections	1.2 (1.0–1.3)	0.0162 (0.0133–0.0191)	135.1 (112.0–158.9)	-21.2 (-36.2—5.9)	-61.9 (-68.6—55.0)
20	Prostate cancer	1.1 (0.9–1.3)	0.0154 (0.0123–0.0192)	106.7 (85.0–133.5)	40.0 (8.3–77.4)	-39.7 (-53.3—23.6)
21	Liver cancer	1.1 (1.0–1.2)	0.0152 (0.0125–0.0177)	117.7 (96.5–137.0)	348.9 (266.3–431.3)	96.0 (60.4–132.5)
22	Neonatal disorders	1.0 (0.9–1.2)	0.0144 (0.0125–0.0162)	338.3 (295.6–381.2)	-32.3 (-41.5—23.0)	-35.8 (-44.6—27.0)
23	Brain and central nervous system cancer	1.0 (0.9–1.1)	0.0143 (0.0120–0.0166)	136.7 (115.3–156.6)	58.2 (32.4–84.6)	-19.9 (-32.7—7.5)
24	Cardiomyopathy and myocarditis	1.0 (0.9–1.1)	0.0139 (0.0115–0.0163)	125.0 (103.4–146.9)	14.5 (-6.2–35.9)	-42.9 (-52.9—32.3)
25	Alcohol use disorders	1.0 (0.9–1.0)	0.0136 (0.0111–0.0158)	137.4 (112.8–159.5)	193.0 (138.3–247.3)	63.2 (32.8–93.6)

Table S2: Cause-specific YLLs by US state in 2021 and percentage change between 1990 and 2021, West Virginia						
Rank	Cause Name	2021 Percentage of all cause YLLs	2021 YLLs (millions)	2021 Age Standardised Rate (per 100 000)	Percentage change YLL count 1990 to 2021	Percentage change age-standardised YLL rate 1990 to 2021
	All causes	100.0 (100.0–100.0)	0.633 (0.567–0.705)	23409.4 (21006.9–26025.8)	47.8 (31.0–65.4)	25.3 (11.3–40.2)
1	COVID-19	16.6 (12.7–20.7)	0.105 (0.0841–0.129)	3733.0 (2998.1–4611.5)	--	--
2	Ischaemic heart disease	11.8 (10.8–12.7)	0.0748 (0.0640–0.0864)	2158.0 (1843.3–2501.4)	-29.2 (-38.7–-18.0)	-45.9 (-53.3–-36.8)
3	Drug use disorders	6.9 (5.9–7.9)	0.0435 (0.0352–0.0530)	2524.7 (2070.4–3036.2)	2421.0 (1875.5–3078.3)	2736.8 (2149.9–3442.2)
4	Chronic obstructive pulmonary disease	5.8 (5.3–6.2)	0.0364 (0.0316–0.0418)	980.2 (851.7–1128.2)	93.4 (67.3–124.5)	44.4 (25.0–68.0)
5	Tracheal, bronchus, and lung cancer	5.5 (5.0–6.0)	0.0349 (0.0295–0.0412)	1011.8 (854.7–1201.9)	-7.6 (-22.1–9.7)	-32.0 (-43.0–-18.9)
6	Stroke	3.6 (3.2–3.9)	0.0226 (0.0195–0.0259)	659.0 (569.7–761.1)	6.9 (-7.8–22.3)	-17.2 (-29.2–-4.3)
7	Chronic kidney disease	3.2 (2.9–3.4)	0.0201 (0.0174–0.0230)	602.3 (524.8–693.1)	302.7 (250.8–364.8)	200.7 (162.8–250.1)
8	Diabetes mellitus	2.7 (2.5–2.9)	0.0169 (0.0143–0.0197)	538.2 (456.0–628.0)	51.1 (29.3–76.0)	21.7 (3.7–42.2)
9	Cirrhosis and other chronic liver diseases	2.6 (2.4–2.8)	0.0166 (0.0142–0.0194)	622.6 (532.1–728.8)	110.9 (78.5–146.7)	78.5 (50.8–110.5)
10	Self-harm	2.5 (2.2–2.7)	0.0156 (0.0130–0.0185)	884.4 (739.8–1043.4)	39.7 (16.5–66.7)	52.9 (27.6–82.2)
11	Alzheimer's disease and other dementias	2.3 (0.6–6.0)	0.0148 (0.00376–0.0388)	348.7 (87.5–918.8)	44.2 (27.9–63.5)	-2.4 (-13.4–10.4)
12	Road injuries	2.1 (1.9–2.3)	0.0134 (0.0114–0.0155)	779.1 (665.9–901.0)	-42.8 (-50.9–-33.5)	-40.7 (-49.1–-31.2)
13	Colon and rectum cancer	2.1 (1.9–2.3)	0.0131 (0.0111–0.0155)	411.6 (345.3–487.8)	6.9 (-11.3–27.3)	-12.4 (-26.8–5.1)
14	Endocrine, metabolic, blood, and immune disorders	1.5 (1.4–1.6)	0.00965 (0.00831–0.0110)	380.7 (331.5–435.2)	247.8 (201.3–300.4)	175.7 (141.2–218.2)
15	Breast cancer	1.4 (1.2–1.7)	0.00886 (0.00722–0.0109)	301.5 (240.4–374.3)	-7.4 (-26.6–14.9)	-25.2 (-41.3–-6.0)
16	Hypertensive heart disease	1.4 (1.2–1.5)	0.00859 (0.00732–0.0101)	290.6 (247.1–341.4)	136.9 (101.2–180.2)	102.8 (71.1–141.3)
17	Pancreatic cancer	1.3 (1.2–1.4)	0.00833 (0.00710–0.00967)	245.6 (209.8–284.9)	75.7 (49.2–107.0)	35.6 (15.2–60.3)
18	Lower respiratory infections	1.2 (1.1–1.4)	0.00789 (0.00652–0.00939)	264.5 (223.4–311.5)	-30.3 (-42.2–-16.1)	-43.1 (-52.4–-31.9)
19	Falls	1.1 (1.0–1.2)	0.00722 (0.00619–0.00817)	219.7 (189.2–250.7)	199.1 (156.8–240.5)	114.0 (83.0–144.2)
20	Interpersonal violence	0.8 (0.8–0.9)	0.00517 (0.00445–0.00600)	342.4 (295.9–394.7)	-25.7 (-36.5–-13.2)	-13.5 (-25.2–1.0)
21	Leukaemia	0.8 (0.7–0.8)	0.00494 (0.00428–0.00571)	175.7 (152.2–202.6)	7.2 (-7.9–26.0)	-13.7 (-25.5–1.3)
22	Prostate cancer	0.8 (0.6–0.9)	0.00477 (0.00368–0.00588)	121.6 (93.5–149.6)	-7.8 (-29.2–15.8)	-31.9 (-47.7–-14.3)
23	Alcohol use disorders	0.7 (0.7–0.8)	0.00469 (0.00389–0.00561)	218.5 (181.9–261.9)	148.2 (104.0–196.6)	143.3 (100.4–192.1)
24	Neonatal disorders	0.7 (0.6–0.9)	0.00457 (0.00372–0.00561)	568.2 (462.0–696.8)	-48.2 (-59.0–-36.3)	-31.1 (-45.5–-15.3)
25	Non-Hodgkin lymphoma	0.7 (0.6–0.8)	0.00448 (0.00383–0.00517)	140.1 (120.0–162.0)	3.7 (-10.5–20.1)	-20.3 (-31.4–-7.5)

Table S2: Cause-specific YLLs by US state in 2021 and percentage change between 1990 and 2021, Wisconsin						
Rank	Cause Name	2021 Percentage of all cause YLLs	2021 YLLs (millions)	2021 Age Standardised Rate (per 100 000)	Percentage change YLL count 1990 to 2021	Percentage change age-standardised YLL rate 1990 to 2021
	All causes	100.0<br>(100.0–100.0)	1.20<br>(1.05–1.37)	13669.6<br>(12064.3–15485.9)	32.1<br>(15.1–50.5)	-10.5<br>(-21.5–1.6)
1	Ischaemic heart disease	12.1<br>(10.8–13.0)	0.146<br>(0.121–0.173)	1345.5<br>(1113.6–1605.2)	-29.7<br>(-40.0–-17.5)	-56.6<br>(-63.2–-48.7)
2	COVID-19	10.7<br>(9.3–12.3)	0.128<br>(0.128–0.140)	1465.7<br>(1459.1–1596.7)	--	--
3	Tracheal, bronchus, and lung cancer	5.5<br>(5.0–5.9)	0.0660<br>(0.0551–0.0791)	606.0<br>(507.0–725.9)	4.8<br>(-12.6–25.8)	-41.8<br>(-51.6–-30.0)
4	Chronic obstructive pulmonary disease	4.7<br>(4.2–5.0)	0.0567<br>(0.0471–0.0664)	490.2<br>(408.2–575.7)	92.6<br>(63.6–125.3)	14.8<br>(-2.8–34.5)
5	Drug use disorders	4.1<br>(3.6–4.7)	0.0495<br>(0.0398–0.0613)	852.9<br>(689.3–1053.0)	1540.3<br>(1185.1–1972.5)	1430.9<br>(1104.0–1828.8)
6	Stroke	4.0<br>(3.6–4.4)	0.0485<br>(0.0403–0.0565)	442.2<br>(367.4–514.5)	-2.9<br>(-16.6–12.1)	-39.1<br>(-47.8–-29.4)
7	Alzheimer's disease and other dementias	3.8<br>(1.0–9.8)	0.0454<br>(0.0120–0.112)	361.0<br>(95.3–896.2)	64.8<br>(44.7–86.2)	-0.3<br>(-13.1–13.4)
8	Chronic kidney disease	3.3<br>(3.0–3.6)	0.0399<br>(0.0335–0.0471)	375.6<br>(317.8–440.4)	337.1<br>(273.4–411.6)	164.3<br>(125.8–209.0)
9	Self-harm	3.2<br>(3.0–3.5)	0.0389<br>(0.0321–0.0465)	648.4<br>(535.3–772.4)	29.5<br>(7.0–54.6)	12.3<br>(-7.0–33.2)
10	Cirrhosis and other chronic liver diseases	2.3<br>(2.2–2.5)	0.0283<br>(0.0236–0.0337)	325.1<br>(270.0–387.9)	90.3<br>(59.3–127.4)	23.3<br>(3.0–47.9)
11	Falls	2.3<br>(2.0–2.4)	0.0274<br>(0.0224–0.0320)	254.2<br>(212.4–297.6)	280.4<br>(223.0–344.4)	114.9<br>(82.7–150.1)
12	Road injuries	2.3<br>(2.1–2.4)	0.0273<br>(0.0230–0.0319)	471.4<br>(398.9–548.5)	-38.4<br>(-48.4–-28.0)	-47.5<br>(-55.9–-38.7)
13	Colon and rectum cancer	2.1<br>(1.9–2.4)	0.0256<br>(0.0210–0.0309)	254.1<br>(208.8–305.4)	-10.1<br>(-27.7–9.9)	-42.9<br>(-54.0–-29.9)
14	Diabetes mellitus	2.0<br>(1.9–2.2)	0.0247<br>(0.0208–0.0292)	249.4<br>(210.5–294.0)	16.5<br>(-2.7–38.4)	-25.3<br>(-37.8–-11.4)
15	Pancreatic cancer	1.8<br>(1.7–1.9)	0.0220<br>(0.0184–0.0259)	205.3<br>(172.0–242.4)	73.7<br>(45.9–104.3)	2.2<br>(-14.3–20.7)
16	Breast cancer	1.7<br>(1.4–2.0)	0.0201<br>(0.0161–0.0243)	213.6<br>(170.3–261.6)	-18.3<br>(-35.0–-0.4)	-49.0<br>(-59.7–-36.9)
17	Endocrine, metabolic, blood, and immune disorders	1.6<br>(1.4–1.7)	0.0189<br>(0.0158–0.0222)	220.5<br>(186.0–256.5)	279.2<br>(217.3–348.3)	137.4<br>(99.3–178.4)
18	Hypertensive heart disease	1.4<br>(1.3–1.6)	0.0171<br>(0.0139–0.0206)	171.2<br>(141.4–205.9)	165.3<br>(121.8–216.2)	72.2<br>(42.9–106.5)
19	Neonatal disorders	1.3<br>(1.1–1.6)	0.0159<br>(0.0142–0.0178)	533.4<br>(475.0–596.7)	-30.4<br>(-39.5–-20.3)	-13.7<br>(-24.9–-1.1)
20	Lower respiratory infections	1.2<br>(1.1–1.3)	0.0145<br>(0.0117–0.0174)	153.0<br>(126.0–182.1)	-40.2<br>(-50.8–-28.4)	-58.8<br>(-65.8–-51.1)
21	Prostate cancer	1.1<br>(0.9–1.3)	0.0137<br>(0.0109–0.0172)	116.6<br>(92.4–146.6)	-2.8<br>(-24.0–21.2)	-41.3<br>(-54.2–-26.6)
22	Alcohol use disorders	1.1<br>(1.0–1.2)	0.0134<br>(0.0110–0.0162)	180.7<br>(148.1–217.3)	180.4<br>(131.2–238.6)	105.1<br>(68.3–149.0)
23	Cardiomyopathy and myocarditis	1.0<br>(0.9–1.1)	0.0123<br>(0.0102–0.0145)	137.6<br>(115.4–161.2)	-9.5<br>(-24.1–7.2)	-41.5<br>(-50.8–-31.2)
24	Leukaemia	1.0<br>(0.9–1.1)	0.0121<br>(0.0102–0.0142)	133.6<br>(114.6–155.2)	4.1<br>(-11.4–22.1)	-33.1<br>(-42.9–-22.4)
25	Brain and central nervous system cancer	0.9<br>(0.9–1.0)	0.0113<br>(0.00964–0.0133)	140.8<br>(121.1–162.6)	31.1<br>(10.9–55.5)	-11.8<br>(-24.8–3.0)

Table S2: Cause-specific YLLs by US state in 2021 and percentage change between 1990 and 2021, Wyoming

Rank	Cause Name	2021 Percentage of all cause YLLs	2021 YLLs (millions)	2021 Age Standardised Rate (per 100 000)	Percentage change YLL count 1990 to 2021	Percentage change age-standardised YLL rate 1990 to 2021
	All causes	100.0 (100.0–100.0)	0.137 (0.125–0.150)	16900.3 (15562.7–18498.3)	74.8 (59.3–91.7)	2.2 (-6.6–11.5)
1	COVID-19	17.7 (16.1–19.5)	0.0242 (0.0241–0.0256)	2946.5 (2931.2–3116.1)	--	--
2	Ischaemic heart disease	9.8 (8.9–10.6)	0.0134 (0.0118–0.0154)	1350.5 (1182.1–1547.0)	-4.3 (-16.2–9.0)	-51.9 (-58.1—45.0)
3	Chronic obstructive pulmonary disease	6.0 (5.4–6.5)	0.00822 (0.00716–0.00923)	775.0 (674.0–869.0)	113.6 (89.6–140.0)	3.5 (-8.3–16.1)
4	Self-harm	4.5 (4.1–4.8)	0.00608 (0.00532–0.00704)	1048.3 (916.6–1209.4)	53.9 (31.4–78.8)	24.1 (6.4–43.7)
5	Tracheal, bronchus, and lung cancer	4.3 (3.8–4.7)	0.00586 (0.00500–0.00668)	583.6 (500.0–665.0)	19.9 (2.1–38.5)	-41.0 (-49.7—31.8)
6	Stroke	3.4 (3.0–3.7)	0.00462 (0.00395–0.00524)	465.5 (402.3–526.5)	29.9 (14.3–45.6)	-34.5 (-42.4—26.6)
7	Road injuries	3.4 (3.2–3.6)	0.00461 (0.00408–0.00522)	845.3 (751.2–950.7)	-26.9 (-35.9—16.6)	-40.1 (-47.2—31.9)
8	Cirrhosis and other chronic liver diseases	3.2 (2.9–3.4)	0.00430 (0.00376–0.00496)	554.4 (486.9–637.5)	140.4 (108.5–179.3)	50.9 (31.1–75.8)
9	Alzheimer's disease and other dementias	2.9 (0.8–7.5)	0.00402 (0.00103–0.0102)	354.6 (90.2–909.2)	118.5 (97.8–141.8)	-2.3 (-11.1–8.2)
10	Drug use disorders	2.8 (2.3–3.4)	0.00388 (0.00309–0.00483)	684.8 (548.0–848.3)	1187.4 (883.0–1517.3)	1014.2 (755.3–1288.1)
11	Chronic kidney disease	2.5 (2.2–2.7)	0.00337 (0.00289–0.00391)	348.6 (302.9–399.2)	436.1 (371.1–517.3)	170.3 (138.0–208.9)
12	Colon and rectum cancer	1.9 (1.7–2.1)	0.00261 (0.00224–0.00298)	278.0 (238.7–318.1)	32.5 (12.7–52.3)	-29.8 (-40.4—19.1)
13	Diabetes mellitus	1.8 (1.7–2.0)	0.00247 (0.00213–0.00283)	271.5 (235.0–310.2)	59.2 (37.1–81.5)	-12.6 (-24.6—0.1)
14	Pancreatic cancer	1.5 (1.4–1.6)	0.00203 (0.00178–0.00231)	208.1 (182.3–236.3)	123.4 (95.9–153.8)	13.6 (-0.1–29.5)
15	Alcohol use disorders	1.4 (1.3–1.6)	0.00198 (0.00171–0.00228)	288.4 (250.7–333.0)	171.2 (130.6–212.6)	93.6 (65.4–122.9)
16	Breast cancer	1.4 (1.2–1.7)	0.00197 (0.00169–0.00228)	228.5 (196.7–264.3)	9.3 (-7.1–27.4)	-37.2 (-46.9—26.9)
17	Falls	1.4 (1.2–1.5)	0.00186 (0.00160–0.00211)	201.2 (176.3–230.3)	220.9 (182.7–262.7)	68.2 (48.5–90.0)
18	Lower respiratory infections	1.2 (1.1–1.4)	0.00170 (0.00146–0.00196)	192.8 (168.5–221.1)	-22.9 (-32.5—11.4)	-58.1 (-63.3—52.2)
19	Endocrine, metabolic, blood, and immune disorders	1.2 (1.1–1.3)	0.00165 (0.00146–0.00187)	214.7 (191.1–240.3)	259.9 (221.3–303.8)	114.1 (91.5–139.1)
20	Hypertensive heart disease	1.1 (1.0–1.2)	0.00154 (0.00132–0.00177)	170.0 (146.2–194.5)	250.5 (200.3–305.4)	91.2 (63.7–121.5)
21	Neonatal disorders	1.1 (0.9–1.3)	0.00144 (0.00117–0.00174)	499.5 (406.3–602.4)	-36.8 (-49.2—20.7)	-27.3 (-41.5—8.8)
22	Prostate cancer	1.0 (0.8–1.1)	0.00131 (0.00105–0.00160)	120.1 (96.3–146.6)	29.8 (2.7–61.8)	-38.3 (-51.3—22.9)
23	Leukaemia	0.9 (0.8–0.9)	0.00118 (0.00104–0.00133)	139.8 (124.4–156.4)	25.0 (9.8–41.3)	-29.1 (-37.3—20.2)
24	Brain and central nervous system cancer	0.8 (0.7–0.8)	0.00107 (0.000955–0.00121)	139.5 (124.7–156.5)	49.7 (32.4–70.5)	-7.9 (-18.1–4.3)
25	Liver cancer	0.7 (0.7–0.8)	0.00100 (0.000867–0.00117)	106.9 (93.0–124.5)	324.3 (266.9–398.8)	121.3 (91.3–159.5)

Table S3: Cause-specific YLDs for the 25 leading Level 3 causes by US state in 2021 and percentage change between 1990 and 2021

Table S3: Cause-specific YLDs by US state in 2021 and percentage change between 1990 and 2021, Alabama						
Rank	Cause Name	2021 Percentage of all cause YLDs	2021 YLDs (millions)	2021 Age Standardised Rate (per 100 000)	Percentage change YLD count 1990 to 2021	Percentage change age-standardised YLD rate 1990 to 2021
	All causes	100.0 (100.0–100.0)	0.842 (0.638–1.08)	13799.1 (10434.8–17703.3)	58.5 (53.2–64.7)	18.9 (14.2–24.4)
1	Low back pain	9.8 (8.7–11.2)	0.0828 (0.0598–0.108)	1332.0 (954.4–1728.7)	31.6 (23.6–40.3)	-2.5 (-8.0–3.6)
2	Diabetes mellitus	6.9 (5.9–7.8)	0.0581 (0.0408–0.0793)	730.7 (511.8–987.7)	274.7 (246.4–304.5)	135.8 (118.5–153.5)
3	Other musculoskeletal disorders	6.8 (5.3–8.4)	0.0569 (0.0403–0.0766)	951.4 (680.1–1291.3)	108.1 (82.8–135.5)	59.1 (39.8–80.2)
4	Drug use disorders	6.5 (4.8–8.2)	0.0545 (0.0390–0.0707)	1132.9 (807.6–1479.7)	459.0 (387.3–536.9)	402.5 (337.2–472.4)
5	Depressive disorders	6.2 (4.7–8.1)	0.0522 (0.0337–0.0742)	1031.0 (668.8–1475.9)	85.8 (52.6–123.2)	61.0 (31.7–93.7)
6	Anxiety disorders	5.2 (3.9–7.1)	0.0440 (0.0285–0.0636)	839.7 (549.7–1239.7)	63.1 (26.9–108.0)	36.9 (6.5–74.1)
7	Headache disorders	4.2 (1.0–8.2)	0.0358 (0.00795–0.0748)	684.3 (137.6–1447.3)	15.6 (9.4–28.5)	-3.9 (-8.4–1.5)
8	Age-related and other hearing loss	4.0 (3.2–5.0)	0.0341 (0.0240–0.0476)	419.0 (294.5–583.9)	49.1 (43.7–55.0)	-5.3 (-8.4–-1.8)
9	Osteoarthritis	3.1 (1.8–5.8)	0.0264 (0.0128–0.0535)	313.8 (151.0–633.2)	74.0 (68.8–79.0)	6.1 (2.8–9.0)
10	Falls	2.8 (2.3–3.4)	0.0237 (0.0165–0.0331)	297.7 (204.9–412.9)	52.4 (41.2–63.4)	-6.7 (-12.7–-0.7)
11	Chronic obstructive pulmonary disease	2.6 (2.1–3.2)	0.0212 (0.0183–0.0241)	244.5 (211.7–278.9)	76.1 (63.7–92.7)	7.3 (0.1–17.4)
12	Oral disorders	2.5 (1.8–3.4)	0.0209 (0.0127–0.0308)	291.7 (173.4–437.3)	45.2 (39.3–51.3)	-1.3 (-5.5–3.2)
13	Asthma	2.3 (1.8–2.9)	0.0196 (0.0128–0.0284)	405.1 (265.2–603.4)	34.0 (23.6–45.6)	10.3 (1.7–19.2)
14	Stroke	2.1 (1.8–2.5)	0.0177 (0.0129–0.0225)	221.5 (160.1–282.8)	64.8 (51.7–77.1)	5.7 (-2.2–13.1)
15	Road injuries	1.7 (1.4–1.9)	0.0140 (0.00999–0.0189)	213.3 (153.2–288.5)	-6.2 (-9.2–-3.2)	-34.0 (-35.7–-32.2)
16	Neck pain	1.6 (1.3–2.1)	0.0139 (0.00931–0.0197)	236.8 (157.8–343.3)	27.9 (21.1–35.5)	-1.5 (-4.7–1.5)
17	Alzheimer's disease and other dementias	1.6 (1.3–2.1)	0.0138 (0.00958–0.0184)	145.6 (100.6–193.0)	59.1 (51.2–66.6)	-6.1 (-10.7–-1.8)
18	Gynecological diseases	1.6 (1.3–1.9)	0.0132 (0.00920–0.0184)	254.0 (175.1–359.1)	-1.1 (-7.8–7.0)	-14.8 (-21.2–-7.6)
19	Schizophrenia	1.5 (1.1–1.9)	0.0121 (0.00893–0.0153)	217.8 (160.4–278.1)	15.9 (7.8–25.2)	-6.6 (-13.4–0.6)
20	Alcohol use disorders	1.2 (1.0–1.5)	0.0100 (0.00689–0.0138)	189.5 (129.7–261.6)	-8.0 (-16.7–1.5)	-23.2 (-30.2–-15.2)
21	Neonatal disorders	1.2 (1.0–1.4)	0.00994 (0.00715–0.0131)	228.8 (163.2–301.4)	19.0 (-2.7–42.1)	6.1 (-13.0–26.2)
22	Chronic kidney disease	1.2 (1.0–1.4)	0.00988 (0.00695–0.0126)	130.4 (92.6–167.6)	95.8 (77.2–116.6)	28.8 (17.3–41.0)
23	Blindness and vision loss	1.1 (0.8–1.6)	0.00923 (0.00588–0.0142)	131.7 (82.3–205.0)	49.1 (40.4–60.0)	1.5 (-4.5–8.9)
24	Autism spectrum disorders	1.1 (0.7–1.6)	0.00920 (0.00639–0.0130)	194.6 (134.5–273.6)	19.3 (8.2–30.5)	1.3 (-8.3–10.6)
25	COVID-19	1.1 (0.4–2.4)	0.00903 (0.00338–0.0207)	165.6 (59.9–385.7)	0.0 (0.0–0.0)	0.0 (0.0–0.0)

Table S3: Cause-specific YLDs by US state in 2021 and percentage change between 1990 and 2021, Alaska

Rank	Cause Name	2021 Percentage of all cause YLDs	2021 YLDs (millions)	2021 Age Standardised Rate (per 100 000)	Percentage change YLD count 1990 to 2021	Percentage change age-standardised YLD rate 1990 to 2021
	All causes	100.0 (100.0–100.0)	0.111 (0.0842–0.142)	13210.4 (9996.8–16947.5)	82.5 (75.0–90.5)	13.8 (10.0–18.5)
1	Low back pain	9.9 (8.7–11.2)	0.0110 (0.00790–0.0144)	1261.5 (913.2–1645.0)	50.5 (38.1–64.6)	-5.7 (-11.3–0.4)
2	Other musculoskeletal disorders	7.6 (6.0–9.4)	0.00839 (0.00597–0.0112)	1009.4 (712.5–1350.1)	155.2 (123.9–192.2)	66.3 (47.0–88.1)
3	Drug use disorders	7.2 (5.4–9.0)	0.00791 (0.00555–0.0103)	1098.1 (769.5–1421.6)	357.3 (296.5–423.0)	305.3 (249.3–367.8)
4	Depressive disorders	6.5 (4.9–8.6)	0.00720 (0.00483–0.0103)	959.9 (639.9–1376.5)	80.4 (46.1–118.7)	42.4 (16.0–74.1)
5	Diabetes mellitus	5.7 (4.9–6.5)	0.00636 (0.00445–0.00868)	612.5 (430.6–833.2)	490.6 (439.4–552.8)	120.5 (104.1–139.7)
6	Anxiety disorders	5.2 (3.7–7.1)	0.00570 (0.00373–0.00853)	737.4 (483.2–1110.6)	61.5 (26.7–105.8)	23.6 (-2.6–57.1)
7	Headache disorders	4.6 (1.0–9.0)	0.00515 (0.00111–0.0109)	670.0 (132.5–1415.2)	23.9 (15.9–43.8)	-2.9 (-7.5–2.6)
8	Age-related and other hearing loss	3.8 (3.0–4.8)	0.00425 (0.00296–0.00585)	425.4 (299.2–582.4)	151.5 (135.5–171.0)	-5.9 (-9.4–-2.2)
9	Falls	3.4 (2.8–4.0)	0.00374 (0.00260–0.00518)	382.6 (263.9–527.1)	99.0 (79.7–118.1)	-7.4 (-12.7–-2.2)
10	Osteoarthritis	2.9 (1.7–5.4)	0.00322 (0.00156–0.00654)	302.1 (145.4–613.1)	216.5 (201.3–232.1)	6.9 (3.8–10.1)
11	Asthma	2.6 (2.0–3.3)	0.00284 (0.00185–0.00417)	393.5 (254.1–586.2)	39.4 (24.4–52.5)	2.1 (-5.7–9.1)
12	Oral disorders	2.4 (1.7–3.3)	0.00265 (0.00158–0.00396)	281.0 (165.1–421.2)	105.1 (90.0–122.5)	-3.6 (-7.8–1.1)
13	Alcohol use disorders	2.3 (1.9–2.8)	0.00251 (0.00175–0.00347)	324.7 (224.4–452.8)	0.1 (-9.2–10.2)	-17.9 (-25.0–-10.4)
14	Chronic obstructive pulmonary disease	1.8 (1.5–2.3)	0.00200 (0.00168–0.00233)	190.1 (160.5–220.1)	202.2 (178.7–230.4)	-3.4 (-9.7–3.8)
15	Neck pain	1.8 (1.3–2.2)	0.00196 (0.00131–0.00279)	235.4 (157.8–341.8)	44.9 (30.1–62.3)	-0.8 (-3.8–2.3)
16	Schizophrenia	1.6 (1.2–2.2)	0.00181 (0.00134–0.00234)	220.3 (163.9–284.9)	21.9 (11.7–33.8)	-6.0 (-13.1–1.8)
17	Gynecological diseases	1.5 (1.2–1.8)	0.00162 (0.00113–0.00226)	212.3 (146.6–300.6)	-1.0 (-10.3–7.7)	-16.8 (-24.2–-10.0)
18	Stroke	1.4 (1.2–1.6)	0.00153 (0.00111–0.00196)	156.8 (113.4–200.8)	146.3 (125.6–171.0)	-8.6 (-15.4–-1.1)
19	Alzheimer's disease and other dementias	1.3 (1.0–1.6)	0.00145 (0.00100–0.00195)	147.0 (101.2–195.5)	320.4 (299.0–343.0)	-7.2 (-11.3–-3.0)
20	Autism spectrum disorders	1.3 (0.8–1.9)	0.00142 (0.000983–0.00198)	201.4 (139.7–279.8)	25.5 (14.9–36.5)	2.5 (-6.0–11.4)
21	Road injuries	1.2 (1.0–1.3)	0.00128 (0.000916–0.00172)	142.7 (102.1–191.8)	-12.2 (-15.6–-8.4)	-47.2 (-48.6–-45.7)
22	Blindness and vision loss	1.1 (0.7–1.6)	0.00119 (0.000752–0.00182)	132.3 (83.9–203.0)	106.2 (86.9–126.6)	2.0 (-3.9–8.7)
23	Other mental disorders	1.0 (0.7–1.4)	0.00113 (0.000736–0.00164)	133.7 (86.5–192.4)	44.3 (36.9–53.6)	-0.8 (-5.3–4.0)
24	COVID-19	1.0 (0.4–2.3)	0.00113 (0.000405–0.00255)	144.2 (49.8–335.0)	0.0 (0.0–0.0)	0.0 (0.0–0.0)
25	Neonatal disorders	0.9 (0.8–1.2)	0.00104 (0.000731–0.00138)	154.6 (108.8–206.0)	25.1 (1.0–56.3)	10.1 (-11.2–37.3)

Table S3: Cause-specific YLDs by US state in 2021 and percentage change between 1990 and 2021, Arizona						
Rank	Cause Name	2021 Percentage of all cause YLDs	2021 YLDs (millions)	2021 Age Standardised Rate (per 100 000)	Percentage change YLD count 1990 to 2021	Percentage change age-standardised YLD rate 1990 to 2021
	All causes	100.0 (100.0–100.0)	1.22 (0.922–1.56)	13837.6 (10383.4–17795.6)	151.5 (143.6–160.5)	16.5 (12.2–21.5)
1	Low back pain	9.7 (8.5–10.9)	0.118 (0.0852–0.152)	1292.1 (938.8–1681.6)	114.0 (100.3–129.9)	-3.8 (-9.8–3.3)
2	Other musculoskeletal disorders	7.2 (5.7–8.8)	0.0874 (0.0616–0.118)	1006.6 (713.2–1346.2)	225.6 (191.8–265.5)	51.1 (36.2–69.7)
3	Depressive disorders	7.2 (5.4–9.3)	0.0872 (0.0565–0.122)	1188.8 (771.8–1659.1)	204.6 (145.9–271.0)	62.7 (31.1–97.2)
4	Drug use disorders	6.4 (4.9–7.9)	0.0768 (0.0553–0.0990)	1091.8 (784.4–1405.1)	520.4 (445.5–596.6)	247.5 (203.2–290.2)
5	Diabetes mellitus	6.1 (5.2–6.9)	0.0747 (0.0526–0.101)	652.4 (460.7–886.4)	554.1 (500.8–609.7)	151.4 (133.0–172.5)
6	Anxiety disorders	5.2 (3.7–7.1)	0.0631 (0.0408–0.0908)	833.8 (534.8–1204.9)	164.1 (103.4–233.7)	37.3 (6.1–74.3)
7	Headache disorders	4.2 (1.0–8.2)	0.0516 (0.0113–0.108)	682.6 (136.9–1437.3)	88.8 (78.7–107.1)	-3.1 (-7.8–2.4)
8	Age-related and other hearing loss	4.0 (3.2–4.9)	0.0492 (0.0345–0.0677)	418.0 (295.4–575.3)	141.2 (132.2–151.0)	-6.3 (-9.5–-3.0)
9	Falls	3.5 (2.8–4.1)	0.0424 (0.0298–0.0593)	360.3 (250.6–499.9)	193.3 (171.3–213.2)	7.1 (-0.1–13.7)
10	Osteoarthritis	3.1 (1.8–5.8)	0.0374 (0.0181–0.0759)	312.0 (150.4–628.4)	182.1 (173.8–192.3)	5.8 (2.5–9.6)
11	Asthma	2.6 (2.0–3.3)	0.0315 (0.0203–0.0460)	443.5 (286.9–662.2)	94.1 (79.1–109.4)	0.8 (-6.2–8.8)
12	Chronic obstructive pulmonary disease	2.4 (1.9–3.0)	0.0290 (0.0247–0.0336)	226.4 (192.8–261.4)	181.8 (162.8–203.1)	3.1 (-3.6–10.9)
13	Oral disorders	2.3 (1.6–3.1)	0.0274 (0.0166–0.0409)	270.4 (159.1–410.1)	121.8 (113.9–130.3)	-5.9 (-9.7–-1.7)
14	Alcohol use disorders	1.8 (1.5–2.2)	0.0221 (0.0152–0.0306)	288.6 (199.2–399.7)	50.4 (37.8–65.0)	-21.7 (-27.8–-13.8)
15	Alzheimer's disease and other dementias	1.7 (1.3–2.1)	0.0206 (0.0143–0.0273)	146.5 (101.2–192.9)	168.2 (154.4–181.8)	-8.2 (-12.6–-3.8)
16	Neck pain	1.6 (1.3–2.1)	0.0199 (0.0134–0.0279)	236.6 (156.8–342.0)	107.6 (95.9–120.2)	-1.1 (-3.8–2.0)
17	Gynecological diseases	1.6 (1.3–1.9)	0.0190 (0.0131–0.0263)	253.9 (173.4–358.2)	61.8 (50.4–75.3)	-13.4 (-19.8–-5.8)
18	Road injuries	1.5 (1.3–1.8)	0.0186 (0.0132–0.0249)	196.0 (140.0–262.9)	37.6 (33.4–42.3)	-40.3 (-42.0–-38.7)
19	Schizophrenia	1.5 (1.1–1.9)	0.0175 (0.0129–0.0221)	219.5 (160.9–279.8)	85.1 (70.7–99.8)	-6.3 (-13.4–0.8)
20	Stroke	1.4 (1.2–1.7)	0.0173 (0.0126–0.0222)	148.8 (108.3–188.5)	146.6 (128.7–169.2)	-3.8 (-10.5–4.7)
21	Autism spectrum disorders	1.1 (0.7–1.7)	0.0136 (0.00932–0.0191)	199.5 (137.5–280.4)	88.5 (73.1–107.8)	1.8 (-6.9–12.1)
22	Blindness and vision loss	1.1 (0.8–1.5)	0.0132 (0.00854–0.0201)	131.3 (82.6–202.4)	143.4 (128.1–159.8)	1.5 (-4.9–8.5)
23	Chronic kidney disease	1.1 (0.9–1.3)	0.0128 (0.00909–0.0166)	114.5 (81.2–146.9)	210.2 (184.3–238.4)	22.0 (11.9–32.2)
24	Neonatal disorders	1.0 (0.8–1.2)	0.0122 (0.00896–0.0162)	191.1 (140.9–257.2)	83.6 (49.2–127.6)	3.3 (-16.3–28.6)
25	Dermatitis	1.0 (0.7–1.4)	0.0120 (0.00708–0.0192)	180.0 (102.9–289.9)	66.5 (58.5–73.7)	-8.8 (-13.7–-4.3)

Table S3: Cause-specific YLDs by US state in 2021 and percentage change between 1990 and 2021, Arkansas						
Rank	Cause Name	2021 Percentage of all cause YLDs	2021 YLDs (millions)	2021 Age Standardised Rate (per 100 000)	Percentage change YLD count 1990 to 2021	Percentage change age-standardised YLD rate 1990 to 2021
	All causes	100.0 (100.0–100.0)	0.478 (0.364–0.614)	13214.7 (9929.6–16932.4)	53.5 (48.8–59.0)	15.3 (11.1–20.6)
1	Low back pain	9.2 (8.1–10.3)	0.0440 (0.0321–0.0567)	1197.0 (870.7–1547.3)	26.5 (18.4–36.2)	-6.4 (-12.4–0.9)
2	Depressive disorders	7.9 (6.0–10.3)	0.0380 (0.0256–0.0542)	1258.0 (842.9–1791.4)	98.1 (63.6–138.9)	64.3 (36.0–98.2)
3	Diabetes mellitus	6.3 (5.5–7.1)	0.0303 (0.0214–0.0407)	647.6 (456.7–873.8)	261.8 (234.0–289.2)	140.1 (122.4–157.0)
4	Other musculoskeletal disorders	5.6 (4.5–7.0)	0.0267 (0.0189–0.0359)	760.3 (535.7–1028.6)	92.7 (68.1–123.5)	46.4 (27.2–68.2)
5	Anxiety disorders	5.4 (3.8–7.3)	0.0258 (0.0157–0.0375)	827.5 (502.3–1204.0)	66.0 (28.6–113.1)	34.7 (4.2–72.7)
6	Drug use disorders	5.3 (4.0–6.7)	0.0251 (0.0178–0.0324)	884.3 (624.4–1148.1)	347.0 (286.0–422.2)	277.2 (224.4–342.3)
7	Headache disorders	4.4 (1.0–8.6)	0.0213 (0.00465–0.0451)	685.5 (136.5–1453.2)	20.8 (15.3–30.5)	-3.5 (-8.1–1.7)
8	Age-related and other hearing loss	4.3 (3.4–5.2)	0.0205 (0.0143–0.0284)	422.5 (295.7–589.1)	39.1 (34.0–44.8)	-5.5 (-8.7–-2.1)
9	Falls	3.1 (2.5–3.6)	0.0147 (0.0102–0.0205)	311.8 (215.0–431.3)	51.2 (41.1–60.8)	-4.1 (-10.2–1.9)
10	Osteoarthritis	3.0 (1.7–5.7)	0.0146 (0.00710–0.0294)	293.1 (141.9–588.8)	64.2 (58.7–69.9)	7.1 (3.4–11.0)
11	Chronic obstructive pulmonary disease	2.6 (2.1–3.3)	0.0124 (0.0107–0.0141)	239.3 (206.3–273.2)	63.3 (51.9–77.8)	7.2 (-0.2–16.8)
12	Oral disorders	2.5 (1.8–3.4)	0.0122 (0.00743–0.0178)	288.1 (170.5–432.3)	38.2 (33.4–43.5)	-1.9 (-5.6–2.4)
13	Asthma	2.3 (1.8–2.9)	0.0111 (0.00722–0.0161)	374.8 (243.4–557.9)	27.3 (17.7–38.3)	1.5 (-6.6–9.9)
14	Stroke	2.1 (1.8–2.5)	0.0101 (0.00733–0.0128)	213.6 (154.4–268.3)	50.4 (37.0–65.2)	4.1 (-4.4–13.3)
15	Alzheimer's disease and other dementias	1.8 (1.5–2.3)	0.00878 (0.00603–0.0117)	151.3 (103.4–201.4)	42.8 (35.8–50.8)	-5.5 (-9.9–-0.6)
16	Road injuries	1.8 (1.5–2.1)	0.00852 (0.00607–0.0114)	220.1 (157.8–296.4)	-1.7 (-4.4–1.2)	-30.7 (-32.6–-28.9)
17	Neck pain	1.7 (1.3–2.2)	0.00822 (0.00553–0.0116)	237.4 (158.2–344.0)	30.3 (24.8–36.4)	-1.3 (-4.0–1.5)
18	Schizophrenia	1.5 (1.1–2.0)	0.00719 (0.00539–0.00907)	219.2 (162.9–277.8)	20.5 (12.3–30.1)	-6.0 (-12.5–1.8)
19	Gynecological diseases	1.4 (1.2–1.7)	0.00693 (0.00477–0.00965)	228.7 (157.6–321.8)	-2.6 (-9.7–5.0)	-18.4 (-24.9–-11.2)
20	Alcohol use disorders	1.3 (1.1–1.6)	0.00629 (0.00443–0.00860)	201.8 (141.2–279.4)	-2.4 (-10.8–7.4)	-22.5 (-28.8–-15.3)
21	Chronic kidney disease	1.2 (1.0–1.5)	0.00592 (0.00418–0.00759)	129.4 (91.8–166.8)	90.5 (75.0–107.6)	31.4 (21.4–42.2)
22	Neonatal disorders	1.2 (1.0–1.4)	0.00558 (0.00402–0.00728)	212.2 (152.0–277.5)	13.6 (-8.8–38.3)	-4.3 (-23.3–16.4)
23	Autism spectrum disorders	1.2 (0.8–1.7)	0.00557 (0.00382–0.00772)	196.3 (133.9–273.3)	24.6 (14.0–37.0)	1.6 (-7.2–11.6)
24	Blindness and vision loss	1.2 (0.8–1.7)	0.00556 (0.00358–0.00840)	133.2 (83.9–205.8)	44.4 (35.6–56.0)	2.0 (-3.9–9.8)
25	COVID-19	1.0 (0.4–2.3)	0.00481 (0.00175–0.0106)	148.1 (51.0–339.3)	0.0 (0.0–0.0)	0.0 (0.0–0.0)

Table S3: Cause-specific YLDs by US state in 2021 and percentage change between 1990 and 2021, California						
**Rank**	**Cause Name**	**2021 Percentage of all cause YLDs**	**2021 YLDs (millions)**	**2021 Age Standardised Rate (per 100 000)**	**Percentage change YLD count 1990 to 2021**	**Percentage change age-standardised YLD rate 1990 to 2021**
	All causes	100.0 (100.0–100.0)	5.96 (4.50–7.67)	12446.0 (9333.6–16078.1)	63.4 (58.2–69.6)	9.7 (5.8–14.1)
1	Low back pain	9.2 (8.1–10.4)	0.550 (0.402–0.713)	1104.0 (808.0–1447.2)	35.3 (25.5–45.8)	-11.6 (-17.5—5.1)
2	Other musculoskeletal disorders	7.1 (5.6–8.8)	0.423 (0.299–0.569)	877.1 (621.6–1179.9)	112.4 (89.2–140.8)	42.5 (26.3–61.1)
3	Depressive disorders	6.1 (4.5–8.1)	0.365 (0.234–0.515)	892.0 (564.1–1263.8)	107.7 (66.4–152.8)	65.3 (34.1–103.2)
4	Anxiety disorders	5.9 (4.2–8.2)	0.350 (0.218–0.516)	827.2 (513.0–1232.3)	76.6 (36.3–126.9)	35.8 (4.6–73.9)
5	Diabetes mellitus	5.8 (5.0–6.5)	0.345 (0.243–0.466)	571.8 (404.2–775.2)	372.6 (335.3–418.8)	149.6 (130.2–173.1)
6	Headache disorders	4.8 (1.1–9.4)	0.291 (0.0640–0.616)	687.5 (139.0–1463.3)	27.7 (20.5–41.2)	-2.3 (-7.0–3.2)
7	Drug use disorders	4.7 (3.5–6.0)	0.278 (0.193–0.362)	679.4 (469.6–884.3)	199.3 (166.6–239.4)	152.1 (124.6–185.9)
8	Age-related and other hearing loss	4.3 (3.4–5.2)	0.254 (0.179–0.351)	420.5 (294.6–581.9)	73.4 (67.2–80.4)	-5.8 (-8.9—2.5)
9	Osteoarthritis	2.9 (1.7–5.4)	0.172 (0.0828–0.346)	276.0 (132.5–557.4)	112.7 (105.4–122.0)	9.2 (5.5–14.0)
10	Falls	2.7 (2.2–3.2)	0.161 (0.111–0.223)	271.2 (186.5–373.2)	68.3 (55.5–80.2)	-8.0 (-13.8—2.6)
11	Oral disorders	2.4 (1.7–3.4)	0.146 (0.0861–0.219)	271.1 (158.1–408.2)	57.5 (51.5–63.9)	-6.3 (-10.2—1.8)
12	Asthma	2.4 (1.9–3.1)	0.145 (0.0953–0.211)	379.0 (245.9–556.0)	29.3 (19.7–38.7)	-2.2 (-8.9–4.7)
13	Alzheimer's disease and other dementias	2.3 (1.8–2.8)	0.134 (0.0930–0.177)	187.6 (129.9–248.7)	98.9 (89.9–110.0)	-6.9 (-11.0—2.3)
14	Chronic obstructive pulmonary disease	2.1 (1.7–2.7)	0.126 (0.107–0.146)	193.4 (164.3–223.6)	88.2 (75.3–102.5)	-5.2 (-11.7–1.7)
15	Neck pain	1.9 (1.4–2.4)	0.111 (0.0746–0.158)	238.2 (160.0–345.7)	41.7 (32.3–51.1)	-0.6 (-3.7–2.1)
16	Schizophrenia	1.7 (1.3–2.3)	0.102 (0.0746–0.128)	224.6 (165.4–284.7)	26.1 (16.0–37.4)	-5.3 (-12.8–3.0)
17	Alcohol use disorders	1.7 (1.4–2.1)	0.0999 (0.0684–0.139)	231.5 (158.3–325.6)	-4.4 (-13.3–5.6)	-24.6 (-30.7—17.3)
18	Gynecological diseases	1.7 (1.3–2.0)	0.0987 (0.0676–0.138)	232.5 (159.1–327.7)	8.2 (-0.7–16.2)	-14.0 (-21.0—7.1)
19	Stroke	1.5 (1.3–1.8)	0.0894 (0.0648–0.114)	148.8 (107.4–190.6)	69.0 (55.6–85.2)	-7.8 (-14.7–0.7)
20	Blindness and vision loss	1.4 (1.0–2.1)	0.0856 (0.0539–0.131)	162.3 (100.4–252.5)	58.5 (44.6–72.0)	-4.9 (-12.3–2.8)
21	Road injuries	1.4 (1.2–1.6)	0.0815 (0.0583–0.110)	158.9 (114.2–214.8)	-8.7 (-11.6—5.5)	-42.0 (-43.6—40.3)
22	Autism spectrum disorders	1.3 (0.9–1.9)	0.0771 (0.0530–0.108)	204.7 (140.4–285.6)	28.6 (18.0–39.1)	2.9 (-5.2–11.6)
23	Dermatitis	1.2 (0.8–1.7)	0.0715 (0.0419–0.114)	196.7 (112.6–316.5)	13.9 (8.7–18.3)	-8.0 (-13.0—3.7)
24	Chronic kidney disease	1.2 (1.0–1.5)	0.0711 (0.0498–0.0932)	120.9 (85.7–157.8)	110.0 (90.9–129.7)	17.4 (7.8–26.7)
25	Other mental disorders	1.0 (0.7–1.4)	0.0625 (0.0405–0.0912)	131.9 (85.1–193.4)	40.8 (33.3–47.8)	-0.6 (-5.7–3.9)

Table S3: Cause-specific YLDs by US state in 2021 and percentage change between 1990 and 2021, Colorado						
Rank	Cause Name	2021 Percentage of all cause YLDs	2021 YLDs (millions)	2021 Age Standardised Rate (per 100 000)	Percentage change YLD count 1990 to 2021	Percentage change age-standardised YLD rate 1990 to 2021
	All causes	100.0 (100.0–100.0)	0.921 (0.705–1.19)	13132.1 (9961.8–16988.8)	120.3 (112.6–128.6)	12.2 (8.2–17.2)
1	Low back pain	9.2 (8.0–10.3)	0.0845 (0.0612–0.110)	1146.7 (829.9–1501.0)	80.5 (67.2–95.3)	-10.4 (-16.3—4.0)
2	Other musculoskeletal disorders	7.3 (5.8–9.1)	0.0673 (0.0472–0.0905)	965.4 (679.8–1296.1)	178.7 (145.9–215.7)	44.2 (28.1–63.0)
3	Depressive disorders	7.2 (5.5–9.2)	0.0660 (0.0440–0.0925)	1098.0 (732.5–1542.9)	143.5 (99.7–194.9)	46.6 (19.9–76.8)
4	Drug use disorders	6.1 (4.7–7.7)	0.0558 (0.0399–0.0726)	964.4 (691.2–1256.9)	529.0 (438.9–635.4)	299.2 (238.1–368.5)
5	Anxiety disorders	5.3 (3.7–7.0)	0.0485 (0.0303–0.0697)	778.5 (484.1–1139.6)	120.1 (70.6–173.0)	28.2 (-0.7–56.8)
6	Diabetes mellitus	4.9 (4.2–5.5)	0.0448 (0.0316–0.0602)	495.8 (352.0–665.0)	476.9 (427.3–532.2)	125.0 (106.5–145.0)
7	Headache disorders	4.6 (1.0–8.9)	0.0424 (0.00936–0.0891)	683.6 (137.2–1442.2)	66.2 (56.8–85.3)	-3.0 (-7.5–3.0)
8	Falls	4.0 (3.4–4.8)	0.0373 (0.0263–0.0518)	422.6 (295.3–580.3)	158.1 (139.6–175.9)	5.5 (-0.5–11.4)
9	Age-related and other hearing loss	4.0 (3.2–5.0)	0.0372 (0.0261–0.0514)	417.6 (293.3–574.6)	134.0 (125.4–144.4)	-5.7 (-8.7—2.2)
10	Osteoarthritis	3.2 (1.9–6.1)	0.0300 (0.0146–0.0598)	322.1 (155.7–644.0)	180.7 (172.0–189.2)	5.8 (2.7–8.8)
11	Asthma	2.5 (1.9–3.2)	0.0229 (0.0150–0.0340)	402.8 (261.7–603.5)	74.0 (60.0–88.1)	-1.5 (-8.6–6.4)
12	Oral disorders	2.4 (1.6–3.3)	0.0220 (0.0132–0.0329)	273.6 (160.6–414.0)	114.2 (106.1–124.2)	-5.1 (-9.1—1.3)
13	Chronic obstructive pulmonary disease	2.2 (1.7–2.8)	0.0201 (0.0170–0.0232)	207.2 (176.1–237.7)	166.5 (148.3–187.2)	-1.4 (-8.2–6.1)
14	Alcohol use disorders	1.9 (1.5–2.3)	0.0171 (0.0119–0.0239)	273.1 (190.6–379.9)	39.6 (25.1–55.6)	-16.7 (-24.0—8.2)
15	Neck pain	1.8 (1.4–2.2)	0.0162 (0.0109–0.0231)	237.6 (158.3–341.7)	85.9 (71.6–100.1)	-0.7 (-3.8–2.1)
16	Schizophrenia	1.6 (1.2–2.1)	0.0147 (0.0108–0.0188)	222.7 (163.2–286.6)	63.6 (49.7–75.8)	-5.3 (-12.9–1.7)
17	Alzheimer's disease and other dementias	1.6 (1.3–2.0)	0.0146 (0.0101–0.0194)	147.8 (102.2–195.6)	149.2 (137.1–161.8)	-8.9 (-12.9—4.4)
18	Gynecological diseases	1.6 (1.3–1.9)	0.0143 (0.0100–0.0200)	232.8 (161.5–327.7)	36.0 (26.0–47.0)	-15.6 (-21.8—8.9)
19	Road injuries	1.4 (1.2–1.7)	0.0133 (0.00950–0.0178)	174.9 (125.6–235.8)	25.2 (20.9–29.8)	-39.7 (-41.4—38.0)
20	Stroke	1.3 (1.1–1.5)	0.0117 (0.00838–0.0149)	132.8 (96.1–170.9)	129.4 (112.2–147.6)	-6.1 (-13.2–1.4)
21	Autism spectrum disorders	1.2 (0.8–1.8)	0.0113 (0.00792–0.0158)	203.2 (141.5–283.7)	70.4 (54.9–85.5)	2.1 (-7.4–11.1)
22	Blindness and vision loss	1.1 (0.8–1.6)	0.0104 (0.00655–0.0158)	132.4 (82.6–204.8)	129.0 (114.1–145.9)	2.9 (-3.1–10.2)
23	Other cardiovascular and circulatory diseases	1.0 (0.8–1.4)	0.00968 (0.00619–0.0149)	116.0 (74.0–176.2)	203.6 (146.1–274.0)	29.6 (8.4–55.6)
24	Other mental disorders	1.0 (0.7–1.3)	0.00914 (0.00593–0.0133)	131.3 (85.0–191.3)	84.8 (75.4–95.5)	-0.6 (-5.7–4.7)
25	Neonatal disorders	1.0 (0.8–1.2)	0.00907 (0.00639–0.0119)	172.6 (120.7–227.2)	70.6 (34.8–109.7)	6.7 (-15.3–32.9)

Table S3: Cause-specific YLDs by US state in 2021 and percentage change between 1990 and 2021, Connecticut						
Rank	Cause Name	2021 Percentage of all cause YLDs	2021 YLDs (millions)	2021 Age Standardised Rate (per 100 000)	Percentage change YLD count 1990 to 2021	Percentage change age-standardised YLD rate 1990 to 2021
	All causes	100.0 (100.0–100.0)	0.586 (0.444–0.748)	12857.2 (9655.3–16417.1)	34.7 (31.0–39.3)	12.3 (8.6–16.7)
1	Low back pain	8.7 (7.6–9.8)	0.0508 (0.0371–0.0651)	1075.4 (787.9–1386.3)	10.4 (2.2–19.3)	-9.7 (-15.9–-3.0)
2	Other musculoskeletal disorders	7.2 (5.6–8.9)	0.0420 (0.0295–0.0565)	943.4 (667.5–1265.7)	67.8 (48.4–90.6)	43.4 (27.7–62.0)
3	Diabetes mellitus	6.8 (5.8–7.6)	0.0397 (0.0279–0.0540)	638.4 (446.9–868.0)	282.4 (252.2–313.2)	157.8 (138.5–178.5)
4	Drug use disorders	6.2 (4.8–7.8)	0.0363 (0.0255–0.0467)	1055.1 (741.6–1365.8)	232.2 (190.5–275.1)	250.9 (205.3–300.4)
5	Anxiety disorders	5.0 (3.6–6.8)	0.0293 (0.0190–0.0444)	765.2 (489.3–1150.3)	30.8 (3.8–69.8)	24.7 (-1.5–61.9)
6	Depressive disorders	4.8 (3.7–6.1)	0.0281 (0.0186–0.0405)	758.6 (499.2–1102.0)	55.6 (28.3–90.0)	52.5 (25.8–89.3)
7	Age-related and other hearing loss	4.5 (3.6–5.4)	0.0262 (0.0182–0.0362)	416.6 (290.0–576.7)	36.3 (31.5–41.9)	-5.3 (-8.8–-2.0)
8	Headache disorders	4.4 (1.0–8.7)	0.0261 (0.00585–0.0548)	687.9 (137.6–1463.5)	2.1 (-3.5–14.0)	-3.1 (-8.1–2.8)
9	Osteoarthritis	3.5 (2.0–6.5)	0.0205 (0.00991–0.0413)	316.7 (151.8–634.0)	59.1 (55.0–63.9)	5.7 (2.7–8.7)
10	Falls	3.3 (2.8–3.9)	0.0196 (0.0138–0.0273)	312.4 (217.3–431.3)	56.1 (45.2–66.5)	2.3 (-4.3–8.3)
11	Chronic obstructive pulmonary disease	2.7 (2.1–3.3)	0.0154 (0.0131–0.0178)	224.9 (191.2–257.1)	62.4 (51.3–76.7)	6.0 (-1.2–14.7)
12	Oral disorders	2.5 (1.7–3.4)	0.0146 (0.00875–0.0219)	272.2 (159.0–414.1)	23.6 (19.2–27.9)	-6.1 (-9.9–-2.0)
13	Asthma	2.4 (1.9–3.1)	0.0144 (0.00944–0.0214)	425.2 (278.8–643.9)	16.1 (7.9–25.9)	8.3 (0.3–17.7)
14	Alzheimer's disease and other dementias	2.0 (1.6–2.5)	0.0118 (0.00808–0.0155)	148.8 (101.5–195.8)	46.2 (38.9–54.2)	-9.2 (-13.5–-4.1)
15	Neck pain	1.8 (1.4–2.2)	0.0103 (0.00695–0.0146)	238.2 (157.8–344.5)	13.5 (6.3–22.1)	-0.9 (-3.5–1.9)
16	Schizophrenia	1.6 (1.2–2.1)	0.00917 (0.00673–0.0116)	223.9 (163.6–283.8)	1.1 (-7.6–9.9)	-5.2 (-13.1–2.9)
17	Gynecological diseases	1.5 (1.3–1.9)	0.00897 (0.00623–0.0124)	241.3 (166.4–341.4)	-18.1 (-23.9–-12.0)	-16.3 (-22.8–-9.6)
18	Stroke	1.5 (1.2–1.7)	0.00868 (0.00629–0.0111)	138.0 (99.5–176.5)	25.4 (15.4–36.4)	-12.8 (-19.7–-6.0)
19	Alcohol use disorders	1.4 (1.1–1.7)	0.00803 (0.00565–0.0112)	208.5 (146.2–292.5)	-20.3 (-27.7–-12.3)	-22.9 (-29.0–-15.3)
20	Blindness and vision loss	1.2 (0.8–1.7)	0.00705 (0.00448–0.0108)	131.0 (81.1–204.0)	38.4 (29.0–48.4)	2.9 (-3.6–9.2)
21	Autism spectrum disorders	1.2 (0.8–1.8)	0.00694 (0.00485–0.00962)	206.7 (143.9–286.5)	6.0 (-2.0–14.3)	2.3 (-5.5–10.1)
22	Road injuries	1.2 (1.0–1.3)	0.00684 (0.00486–0.00921)	138.1 (98.5–185.8)	-31.0 (-33.4–-28.2)	-45.7 (-47.3–-43.8)
23	Dermatitis	1.1 (0.8–1.6)	0.00672 (0.00397–0.0106)	204.7 (118.2–325.0)	-5.0 (-8.8–-0.9)	-8.1 (-12.4–-3.3)
24	Chronic kidney disease	1.2 (0.9–1.4)	0.00672 (0.00479–0.00875)	109.2 (78.0–142.2)	74.6 (60.0–92.6)	23.4 (13.6–34.7)
25	Atrial fibrillation and flutter	1.1 (0.9–1.3)	0.00630 (0.00437–0.00839)	86.4 (60.1–115.0)	90.1 (51.5–142.6)	23.4 (-1.1–56.7)

Table S3: Cause-specific YLDs by US state in 2021 and percentage change between 1990 and 2021, Delaware						
**Rank**	**Cause Name**	**2021 Percentage of all cause YLDs**	**2021 YLDs (millions)**	**2021 Age Standardised Rate (per 100 000)**	**Percentage change YLD count 1990 to 2021**	**Percentage change age-standardised YLD rate 1990 to 2021**
	All causes	100.0 (100.0–100.0)	0.171 (0.131–0.220)	13712.9 (10388.6–17697.9)	95.4 (88.8–102.2)	17.2 (13.0–22.2)
1	Low back pain	9.1 (8.0–10.2)	0.0155 (0.0112–0.0201)	1193.0 (873.5–1552.9)	58.7 (46.8–70.9)	-7.1 (-13.9–0.3)
2	Diabetes mellitus	7.3 (6.3–8.2)	0.0125 (0.00886–0.0168)	730.1 (516.3–981.9)	395.7 (357.0–437.6)	132.7 (114.9–152.3)
3	Other musculoskeletal disorders	7.3 (5.8–9.1)	0.0125 (0.00883–0.0167)	1005.2 (714.9–1349.3)	154.2 (128.7–183.5)	55.0 (38.0–73.0)
4	Drug use disorders	6.4 (4.8–8.1)	0.0109 (0.00767–0.0141)	1154.9 (815.2–1503.0)	401.8 (343.4–470.8)	295.2 (246.8–350.5)
5	Depressive disorders	6.3 (4.7–8.2)	0.0107 (0.00727–0.0150)	1071.2 (723.4–1503.6)	117.2 (75.8–160.0)	58.6 (27.9–90.9)
6	Anxiety disorders	4.9 (3.5–6.9)	0.00846 (0.00536–0.0126)	811.8 (514.8–1229.3)	88.5 (47.1–140.0)	32.8 (4.0–67.7)
7	Age-related and other hearing loss	4.2 (3.4–5.3)	0.00720 (0.00506–0.00990)	415.4 (291.0–574.0)	100.7 (92.8–109.1)	-5.9 (-9.0–-2.5)
8	Headache disorders	4.1 (1.0–8.0)	0.00712 (0.00160–0.0148)	686.3 (136.9–1447.0)	37.9 (30.2–53.4)	-3.4 (-8.2–2.3)
9	Osteoarthritis	3.5 (2.1–6.5)	0.00607 (0.00296–0.0121)	341.2 (164.9–679.8)	131.0 (123.7–138.9)	5.4 (2.1–8.7)
10	Falls	2.8 (2.3–3.4)	0.00481 (0.00334–0.00672)	287.3 (198.3–397.0)	95.5 (82.1–108.0)	-8.9 (-14.2–-4.0)
11	Chronic obstructive pulmonary disease	2.6 (2.1–3.3)	0.00447 (0.00384–0.00511)	237.5 (205.5–269.0)	145.0 (126.8–167.2)	7.9 (0.2–17.4)
12	Asthma	2.4 (1.8–3.0)	0.00409 (0.00265–0.00606)	432.0 (280.2–649.2)	65.0 (51.9–78.5)	13.0 (5.0–22.7)
13	Oral disorders	2.3 (1.6–3.2)	0.00395 (0.00237–0.00605)	267.9 (156.3–411.5)	76.3 (61.0–91.6)	-6.7 (-15.0–2.3)
14	Alzheimer's disease and other dementias	1.8 (1.4–2.3)	0.00312 (0.00217–0.00412)	148.1 (102.8–195.4)	131.7 (121.1–142.9)	-8.3 (-12.5–-4.0)
15	Stroke	1.7 (1.4–2.0)	0.00284 (0.00204–0.00359)	164.3 (118.0–208.7)	104.0 (89.5–123.0)	-4.8 (-11.1–3.1)
16	Neck pain	1.6 (1.3–2.1)	0.00280 (0.00188–0.00395)	237.3 (156.9–341.2)	55.8 (45.5–66.7)	-1.1 (-4.0–1.9)
17	Gynecological diseases	1.4 (1.2–1.7)	0.00245 (0.00171–0.00343)	243.4 (168.3–346.5)	14.4 (5.3–23.9)	-14.2 (-21.0–-7.1)
18	Schizophrenia	1.4 (1.0–1.9)	0.00244 (0.00183–0.00310)	219.7 (162.7–279.9)	35.8 (24.9–47.2)	-6.5 (-13.8–1.0)
19	Road injuries	1.3 (1.1–1.5)	0.00222 (0.00159–0.00298)	165.6 (118.6–221.7)	3.9 (0.3–7.8)	-41.0 (-42.6–-39.1)
20	Alcohol use disorders	1.2 (1.0–1.5)	0.00207 (0.00144–0.00287)	196.7 (135.2–275.2)	8.4 (-2.9–21.4)	-22.1 (-29.6–-13.5)
21	Chronic kidney disease	1.2 (1.0–1.5)	0.00205 (0.00145–0.00261)	125.5 (89.3–159.1)	153.8 (129.4–181.5)	23.8 (13.6–36.6)
22	Blindness and vision loss	1.1 (0.8–1.6)	0.00190 (0.00122–0.00285)	130.7 (82.3–203.1)	95.0 (83.0–109.1)	2.0 (-3.9–9.5)
23	Autism spectrum disorders	1.1 (0.7–1.6)	0.00185 (0.00127–0.00262)	199.3 (136.4–282.8)	41.9 (30.6–55.2)	2.0 (-6.3–11.2)
24	Bipolar disorder	1.1 (0.7–1.4)	0.00180 (0.00117–0.00252)	170.6 (111.3–239.8)	43.2 (34.3–53.8)	-1.0 (-7.8–6.5)
25	Atrial fibrillation and flutter	1.0 (0.8–1.2)	0.00173 (0.00119–0.00232)	85.9 (59.1–114.7)	191.6 (132.3–265.8)	22.4 (-2.2–53.1)

Table S3: Cause-specific YLDs by US state in 2021 and percentage change between 1990 and 2021, District of Columbia						
Rank	Cause Name	2021 Percentage of all cause YLDs	2021 YLDs (millions)	2021 Age Standardised Rate (per 100 000)	Percentage change YLD count 1990 to 2021	Percentage change age-standardised YLD rate 1990 to 2021
	All causes	100.0 (100.0–100.0)	0.106 (0.0802–0.136)	12794.8 (9637.5–16443.2)	25.7 (21.7–29.6)	6.9 (3.2–10.5)
1	Drug use disorders	9.5 (7.1–12.0)	0.00999 (0.00692–0.0130)	1191.1 (820.4–1553.7)	189.4 (155.7–228.9)	158.2 (129.5–192.8)
2	Low back pain	7.3 (6.4–8.3)	0.00777 (0.00567–0.0102)	889.0 (650.1–1164.1)	-3.9 (-11.5–5.5)	-20.8 (-27.1–-13.5)
3	Other musculoskeletal disorders	6.3 (5.0–7.8)	0.00664 (0.00470–0.00894)	801.5 (566.3–1078.8)	52.8 (36.5–70.2)	33.0 (18.0–49.2)
4	Anxiety disorders	5.9 (4.2–7.8)	0.00627 (0.00395–0.00894)	791.8 (493.9–1117.9)	45.2 (13.2–80.8)	28.2 (0.1–59.2)
5	Depressive disorders	5.9 (4.4–7.7)	0.00626 (0.00403–0.00896)	821.6 (538.3–1182.4)	51.0 (23.2–82.3)	39.9 (13.8–70.2)
6	Headache disorders	5.1 (1.1–10.1)	0.00552 (0.00119–0.0116)	699.0 (138.9–1471.2)	11.1 (4.6–22.3)	-2.5 (-7.5–2.9)
7	Diabetes mellitus	4.5 (3.8–5.1)	0.00473 (0.00333–0.00641)	506.7 (356.2–688.5)	127.6 (108.9–149.0)	79.7 (64.8–97.5)
8	Age-related and other hearing loss	3.7 (3.0–4.6)	0.00395 (0.00275–0.00545)	419.4 (291.0–581.8)	16.7 (12.7–21.1)	-4.6 (-7.6–-1.0)
9	Falls	3.0 (2.5–3.6)	0.00316 (0.00220–0.00437)	336.9 (234.4–465.1)	22.2 (14.4–30.1)	-3.8 (-9.7–2.2)
10	Asthma	2.6 (2.0–3.3)	0.00273 (0.00178–0.00396)	416.9 (269.8–622.7)	23.5 (16.0–30.9)	6.1 (-0.2–13.1)
11	Osteoarthritis	2.6 (1.5–4.8)	0.00272 (0.00132–0.00550)	289.8 (139.6–582.8)	31.8 (28.3–35.8)	5.3 (2.4–8.4)
12	Alcohol use disorders	2.5 (2.1–3.1)	0.00269 (0.00183–0.00370)	319.1 (217.0–440.0)	-14.8 (-22.3–-6.3)	-24.6 (-31.0–-18.3)
13	Oral disorders	2.3 (1.6–3.2)	0.00242 (0.00141–0.00366)	273.0 (159.2–415.0)	16.2 (11.0–21.4)	-4.6 (-8.4–-0.5)
14	Gynecological diseases	2.0 (1.6–2.4)	0.00207 (0.00145–0.00295)	243.0 (168.5–348.8)	2.1 (-6.3–11.1)	-12.6 (-20.2–-4.7)
15	Neck pain	1.9 (1.5–2.4)	0.00201 (0.00133–0.00289)	239.2 (158.9–344.1)	17.0 (10.5–22.6)	-0.8 (-3.6–2.1)
16	Schizophrenia	1.9 (1.4–2.5)	0.00197 (0.00144–0.00250)	221.0 (160.7–279.8)	14.9 (5.7–24.9)	-4.7 (-11.9–2.7)
17	Stroke	1.5 (1.3–1.8)	0.00162 (0.00117–0.00206)	171.0 (124.5–219.2)	8.6 (-0.2–18.2)	-12.4 (-19.1–-4.8)
18	Alzheimer's disease and other dementias	1.5 (1.2–1.9)	0.00158 (0.00110–0.00210)	147.3 (101.3–194.8)	20.5 (13.8–27.2)	-5.9 (-10.9–-1.1)
19	Chronic obstructive pulmonary disease	1.4 (1.1–1.8)	0.00150 (0.00126–0.00177)	154.2 (129.5–182.2)	15.3 (5.9–27.5)	-7.4 (-14.7–2.1)
20	Chronic kidney disease	1.4 (1.2–1.7)	0.00150 (0.00108–0.00191)	158.1 (113.8–201.8)	37.2 (27.3–48.6)	9.6 (1.4–18.9)
21	Neonatal disorders	1.3 (1.1–1.7)	0.00142 (0.000976–0.00186)	224.6 (156.5–297.5)	-7.7 (-26.1–12.8)	-17.4 (-33.4–1.8)
22	HIV/AIDS	1.3 (0.7–2.1)	0.00141 (0.000650–0.00249)	156.8 (72.2–275.7)	-12.3 (-34.6–12.4)	-25.8 (-44.6–-4.9)
23	Dermatitis	1.3 (0.8–1.8)	0.00135 (0.000804–0.00213)	213.9 (124.7–343.0)	3.2 (-1.5–7.8)	-6.8 (-10.8–-2.7)
24	Autism spectrum disorders	1.2 (0.8–1.8)	0.00128 (0.000871–0.00182)	191.9 (130.8–273.0)	17.2 (7.4–28.3)	4.6 (-4.4–13.9)
25	Blindness and vision loss	1.1 (0.8–1.6)	0.00115 (0.000735–0.00175)	133.1 (83.8–204.5)	22.5 (14.7–31.7)	1.9 (-4.4–8.9)

Table S3: Cause-specific YLDs by US state in 2021 and percentage change between 1990 and 2021, Florida						
**Rank**	**Cause Name**	**2021 Percentage of all cause YLDs**	**2021 YLDs (millions)**	**2021 Age Standardised Rate (per 100 000)**	**Percentage change YLD count 1990 to 2021**	**Percentage change age-standardised YLD rate 1990 to 2021**
	All causes	100.0 (100.0–100.0)	3.79 (2.88–4.87)	13565.7 (10248.9–17490.7)	104.7 (98.6–112.4)	15.9 (11.4–21.2)
1	Low back pain	9.3 (8.2–10.5)	0.353 (0.256–0.457)	1195.0 (869.7–1554.3)	68.0 (56.8–79.6)	-9.6 (-15.4–-3.0)
2	Diabetes mellitus	7.0 (6.0–7.9)	0.267 (0.190–0.361)	693.2 (489.2–933.6)	424.8 (386.2–464.1)	163.6 (145.1–182.9)
3	Depressive disorders	6.6 (5.0–8.5)	0.249 (0.166–0.353)	1139.9 (765.3–1615.7)	126.4 (84.7–172.1)	44.6 (17.0–74.0)
4	Other musculoskeletal disorders	6.0 (4.7–7.4)	0.227 (0.160–0.305)	809.4 (575.4–1085.3)	150.8 (122.9–183.9)	39.0 (23.8–57.9)
5	Drug use disorders	5.9 (4.5–7.3)	0.222 (0.154–0.289)	1087.2 (752.0–1417.1)	564.1 (480.3–657.1)	328.0 (271.9–390.3)
6	Anxiety disorders	5.0 (3.4–6.9)	0.189 (0.121–0.280)	829.2 (522.4–1222.8)	118.9 (70.0–175.6)	36.2 (5.6–70.0)
7	Age-related and other hearing loss	4.4 (3.5–5.3)	0.166 (0.116–0.227)	416.7 (294.6–572.2)	80.7 (74.0–88.0)	-6.1 (-8.8–-2.7)
8	Headache disorders	4.1 (1.0–7.9)	0.155 (0.0354–0.325)	684.9 (138.4–1442.7)	60.3 (52.3–72.9)	-3.2 (-7.8–2.4)
9	Falls	3.3 (2.7–3.9)	0.126 (0.0888–0.177)	318.5 (222.1–441.5)	115.8 (101.8–129.5)	-2.3 (-8.7–4.0)
10	Osteoarthritis	3.3 (1.9–6.1)	0.125 (0.0606–0.252)	307.8 (147.8–616.3)	110.5 (105.0–116.9)	5.9 (3.0–9.1)
11	Chronic obstructive pulmonary disease	2.8 (2.2–3.6)	0.107 (0.0915–0.122)	246.1 (211.5–280.2)	114.2 (98.1–133.0)	7.3 (-0.4–16.3)
12	Oral disorders	2.4 (1.7–3.2)	0.0906 (0.0544–0.135)	272.6 (159.3–416.0)	72.7 (65.4–80.3)	-5.3 (-9.6–-1.1)
13	Asthma	2.1 (1.6–2.7)	0.0790 (0.0517–0.117)	388.6 (251.4–586.1)	65.6 (53.8–79.1)	5.3 (-2.5–15.1)
14	Alzheimer's disease and other dementias	2.0 (1.6–2.5)	0.0746 (0.0516–0.0992)	147.3 (101.1–195.1)	88.5 (78.4–98.5)	-8.5 (-12.9–-4.1)
15	Stroke	1.8 (1.5–2.1)	0.0663 (0.0477–0.0841)	169.6 (122.3–216.8)	89.7 (74.6–107.9)	-0.5 (-7.3–7.8)
16	Neck pain	1.6 (1.3–2.1)	0.0619 (0.0414–0.0865)	237.0 (155.6–340.5)	72.6 (64.9–81.1)	-1.1 (-3.8–1.9)
17	Road injuries	1.6 (1.4–1.8)	0.0606 (0.0433–0.0812)	200.5 (144.3–268.7)	23.6 (20.0–27.1)	-34.6 (-36.3–-32.8)
18	Gynecological diseases	1.5 (1.2–1.8)	0.0577 (0.0407–0.0792)	257.4 (180.1–357.0)	35.0 (25.7–45.9)	-13.7 (-20.6–-6.1)
19	Schizophrenia	1.4 (1.1–1.9)	0.0540 (0.0400–0.0681)	219.8 (162.5–280.9)	58.4 (47.2–70.7)	-6.0 (-13.6–1.4)
20	Alcohol use disorders	1.3 (1.1–1.6)	0.0505 (0.0348–0.0693)	218.5 (151.4–297.5)	27.9 (17.3–41.0)	-23.2 (-29.0–-15.6)
21	Chronic kidney disease	1.2 (1.0–1.5)	0.0451 (0.0316–0.0585)	119.1 (84.7–153.1)	144.8 (125.2–166.1)	25.5 (14.9–36.0)
22	Blindness and vision loss	1.1 (0.8–1.6)	0.0432 (0.0279–0.0643)	130.5 (82.1–200.5)	90.3 (78.6–103.2)	1.3 (-4.8–7.7)
23	COVID-19	1.1 (0.4–2.4)	0.0405 (0.0160–0.0907)	168.3 (60.7–393.4)	0.0 (0.0–0.0)	0.0 (0.0–0.0)
24	Autism spectrum disorders	1.1 (0.7–1.6)	0.0402 (0.0276–0.0554)	199.8 (137.2–276.3)	63.6 (51.3–78.8)	2.2 (-5.7–11.6)
25	Neonatal disorders	1.1 (0.9–1.3)	0.0400 (0.0285–0.0521)	217.8 (153.6–283.1)	65.5 (38.6–101.2)	5.9 (-11.9–29.3)

Table S3: Cause-specific YLDs by US state in 2021 and percentage change between 1990 and 2021, Georgia						
Rank	Cause Name	2021 Percentage of all cause YLDs	2021 YLDs (millions)	2021 Age Standardised Rate (per 100 000)	Percentage change YLD count 1990 to 2021	Percentage change age-standardised YLD rate 1990 to 2021
	All causes	100.0 (100.0–100.0)	1.69 (1.28–2.17)	13200.8 (9970.4–16911.6)	108.9 (101.6–117.0)	14.2 (10.1–18.9)
1	Low back pain	9.3 (8.2–10.6)	0.158 (0.114–0.206)	1188.8 (862.2–1551.9)	71.3 (59.6–85.6)	-8.0 (-13.9–-1.0)
2	Depressive disorders	7.0 (5.2–9.3)	0.118 (0.0771–0.168)	1055.0 (681.8–1508.7)	140.2 (91.1–193.2)	53.0 (22.2–87.4)
3	Diabetes mellitus	6.8 (5.8–7.7)	0.116 (0.0813–0.156)	722.7 (509.5–977.9)	443.0 (402.8–488.5)	135.3 (118.3–153.7)
4	Other musculoskeletal disorders	6.6 (5.3–8.2)	0.112 (0.0796–0.150)	865.3 (616.3–1168.3)	169.9 (138.5–200.7)	48.4 (32.0–65.9)
5	Anxiety disorders	5.6 (3.8–7.6)	0.0946 (0.0584–0.137)	829.3 (504.9–1197.7)	117.3 (66.4–179.6)	35.5 (3.6–74.3)
6	Drug use disorders	5.4 (4.0–6.8)	0.0900 (0.0626–0.117)	835.9 (576.6–1091.7)	490.7 (419.7–573.8)	304.6 (254.3–362.1)
7	Headache disorders	4.6 (1.0–9.0)	0.0790 (0.0172–0.165)	691.2 (139.3–1457.8)	56.3 (48.1–73.8)	-2.7 (-7.4–3.3)
8	Age-related and other hearing loss	3.9 (3.1–4.8)	0.0663 (0.0463–0.0913)	418.3 (294.1–580.6)	111.8 (103.2–121.0)	-5.8 (-9.1–-2.5)
9	Osteoarthritis	3.0 (1.7–5.6)	0.0511 (0.0248–0.103)	307.5 (148.1–618.1)	154.2 (146.3–161.8)	6.1 (2.8–9.1)
10	Falls	2.8 (2.3–3.3)	0.0475 (0.0331–0.0664)	301.7 (208.7–418.3)	111.0 (95.2–125.7)	-5.7 (-11.1–-0.4)
11	Oral disorders	2.4 (1.7–3.3)	0.0407 (0.0239–0.0617)	280.3 (161.7–425.9)	99.1 (81.2–115.9)	-4.4 (-12.0–3.9)
12	Asthma	2.4 (1.8–3.0)	0.0402 (0.0264–0.0588)	391.2 (253.2–580.2)	70.4 (55.8–85.0)	4.8 (-3.2–14.4)
13	Chronic obstructive pulmonary disease	2.3 (1.8–2.9)	0.0388 (0.0331–0.0446)	229.9 (196.0–264.5)	148.4 (132.3–166.8)	4.1 (-2.4–11.7)
14	Neck pain	1.8 (1.4–2.2)	0.0298 (0.0200–0.0423)	238.2 (157.7–341.7)	73.5 (62.0–86.7)	-0.8 (-3.7–2.1)
15	Stroke	1.7 (1.4–2.1)	0.0294 (0.0215–0.0372)	187.8 (137.6–239.4)	111.8 (95.2–132.3)	-4.9 (-12.2–3.8)
16	Gynecological diseases	1.6 (1.3–2.0)	0.0278 (0.0195–0.0389)	247.1 (171.4–348.4)	31.9 (21.0–42.1)	-13.6 (-20.9–-6.5)
17	Schizophrenia	1.6 (1.2–2.1)	0.0266 (0.0198–0.0339)	220.7 (164.2–281.2)	53.9 (41.9–67.7)	-5.8 (-13.0–2.0)
18	Alzheimer's disease and other dementias	1.5 (1.2–1.9)	0.0252 (0.0174–0.0336)	145.8 (100.1–193.6)	130.8 (118.9–141.9)	-7.4 (-11.9–-2.8)
19	Road injuries	1.4 (1.2–1.6)	0.0238 (0.0170–0.0320)	174.3 (125.0–234.3)	9.9 (6.1–14.0)	-42.8 (-44.3–-41.1)
20	Neonatal disorders	1.3 (1.1–1.6)	0.0223 (0.0158–0.0289)	231.0 (163.0–300.5)	62.1 (31.2–98.5)	8.4 (-12.6–31.9)
21	Alcohol use disorders	1.3 (1.0–1.6)	0.0216 (0.0149–0.0292)	186.5 (128.3–255.5)	14.5 (2.6–26.1)	-27.1 (-33.6–-20.2)
22	Chronic kidney disease	1.2 (1.0–1.5)	0.0204 (0.0143–0.0262)	135.0 (94.5–173.1)	162.8 (139.6–191.9)	22.7 (12.5–34.5)
23	Autism spectrum disorders	1.2 (0.8–1.8)	0.0202 (0.0140–0.0283)	197.2 (135.6–275.7)	59.3 (44.4–75.4)	1.9 (-7.4–12.0)
24	Blindness and vision loss	1.1 (0.8–1.6)	0.0194 (0.0126–0.0295)	135.6 (87.3–207.8)	110.1 (94.7–126.0)	1.4 (-5.3–8.8)
25	COVID-19	1.1 (0.4–2.5)	0.0180 (0.00640–0.0441)	153.7 (54.4–384.3)	0.0 (0.0–0.0)	0.0 (0.0–0.0)

Table S3: Cause-specific YLDs by US state in 2021 and percentage change between 1990 and 2021, Hawaii						
Rank	Cause Name	2021 Percentage of all cause YLDs	2021 YLDs (millions)	2021 Age Standardised Rate (per 100 000)	Percentage change YLD count 1990 to 2021	Percentage change age-standardised YLD rate 1990 to 2021
	All causes	100.0 (100.0–100.0)	0.223 (0.169–0.288)	12085.3 (9090.8–15557.1)	62.3 (57.4–67.9)	7.7 (4.6–11.6)
1	Low back pain	8.3 (7.3–9.4)	0.0185 (0.0134–0.0241)	967.9 (704.4–1277.8)	27.1 (15.9–39.0)	-16.7 (-22.8–-9.7)
2	Diabetes mellitus	6.8 (6.0–7.6)	0.0152 (0.0108–0.0207)	610.2 (430.8–823.7)	343.3 (308.5–379.5)	126.8 (110.0–144.4)
3	Depressive disorders	6.1 (4.6–8.0)	0.0135 (0.00878–0.0192)	923.8 (596.7–1326.9)	73.3 (41.8–108.5)	45.2 (18.4–76.4)
4	Other musculoskeletal disorders	6.0 (4.7–7.5)	0.0134 (0.00945–0.0181)	744.2 (526.9–1009.3)	89.3 (65.7–115.7)	31.3 (15.5–48.8)
5	Anxiety disorders	5.1 (3.6–6.9)	0.0115 (0.00713–0.0167)	750.7 (466.8–1102.1)	54.6 (20.5–92.2)	24.0 (-3.0–55.0)
6	Age-related and other hearing loss	4.9 (3.9–6.0)	0.0108 (0.00759–0.0149)	422.9 (296.1–584.1)	88.3 (80.2–96.9)	-5.8 (-9.2–-2.5)
7	Headache disorders	4.6 (1.1–8.9)	0.0103 (0.00229–0.0218)	686.3 (137.0–1455.4)	21.0 (14.3–35.2)	-2.2 (-6.9–3.4)
8	Drug use disorders	3.7 (2.8–4.8)	0.00829 (0.00570–0.0108)	602.8 (413.6–787.5)	205.1 (168.5–250.1)	178.9 (145.5–221.3)
9	Falls	3.7 (3.0–4.4)	0.00819 (0.00575–0.0113)	329.1 (228.6–453.2)	104.9 (90.5–118.4)	3.4 (-2.5–8.8)
10	Osteoarthritis	3.3 (1.9–6.1)	0.00733 (0.00357–0.0147)	282.0 (135.8–563.0)	117.1 (110.0–124.6)	6.8 (3.5–10.4)
11	Asthma	2.9 (2.3–3.7)	0.00649 (0.00420–0.00949)	433.9 (280.8–650.3)	42.8 (34.0–52.0)	4.1 (-2.3–10.8)
12	Oral disorders	2.6 (1.8–3.6)	0.00576 (0.00340–0.00875)	267.4 (153.6–413.7)	57.5 (42.2–70.3)	-8.3 (-16.3–-0.2)
13	Chronic obstructive pulmonary disease	2.3 (1.8–3.0)	0.00515 (0.00428–0.00601)	184.1 (153.7–214.0)	115.8 (101.2–134.0)	-0.1 (-6.9–8.1)
14	Alzheimer's disease and other dementias	2.3 (1.8–2.9)	0.00511 (0.00349–0.00675)	151.6 (102.2–201.8)	153.8 (140.5–168.1)	-5.7 (-9.5–-1.6)
15	Neck pain	1.8 (1.4–2.3)	0.00409 (0.00274–0.00576)	238.4 (158.9–347.1)	37.6 (27.5–47.5)	-0.4 (-3.2–2.3)
16	Stroke	1.8 (1.5–2.1)	0.00395 (0.00284–0.00500)	153.0 (111.5–194.8)	85.9 (70.9–100.8)	-8.7 (-15.4–-2.1)
17	Schizophrenia	1.7 (1.3–2.3)	0.00378 (0.00274–0.00479)	235.7 (171.1–302.3)	18.1 (8.5–28.5)	-4.8 (-12.2–2.7)
18	Alcohol use disorders	1.6 (1.3–2.0)	0.00361 (0.00248–0.00502)	238.2 (162.8–336.2)	-11.6 (-19.2–-1.5)	-26.2 (-32.5–-18.5)
19	Gynecological diseases	1.6 (1.3–1.9)	0.00361 (0.00251–0.00502)	241.4 (169.2–336.9)	2.9 (-4.6–10.7)	-12.2 (-18.8–-4.9)
20	Chronic kidney disease	1.4 (1.2–1.7)	0.00313 (0.00225–0.00402)	127.1 (89.8–162.8)	141.1 (119.8–168.4)	23.8 (13.7–34.4)
21	Road injuries	1.3 (1.1–1.5)	0.00294 (0.00211–0.00395)	151.4 (108.9–203.0)	-6.1 (-9.2–-2.7)	-39.2 (-40.9–-37.3)
22	Blindness and vision loss	1.3 (0.9–1.8)	0.00288 (0.00186–0.00431)	132.6 (82.8–205.6)	83.8 (73.2–95.6)	3.1 (-2.9–9.9)
23	Dermatitis	1.3 (0.9–1.8)	0.00282 (0.00167–0.00443)	207.5 (118.5–329.6)	17.7 (13.1–22.0)	-6.0 (-10.5–-1.9)
24	Autism spectrum disorders	1.3 (0.8–1.9)	0.00279 (0.00193–0.00389)	205.4 (141.6–286.6)	24.2 (14.7–35.3)	2.0 (-6.1–10.6)
25	Other mental disorders	1.0 (0.8–1.4)	0.00234 (0.00153–0.00341)	132.9 (85.8–196.3)	37.1 (30.4–45.2)	-0.4 (-4.7–4.9)

Table S3: Cause-specific YLDs by US state in 2021 and percentage change between 1990 and 2021, Idaho

Rank	Cause Name	2021 Percentage of all cause YLDs	2021 YLDs (millions)	2021 Age Standardised Rate (per 100 000)	Percentage change YLD count 1990 to 2021	Percentage change age-standardised YLD rate 1990 to 2021
	All causes	100.0 (100.0–100.0)	0.289 (0.220–0.369)	13152.2 (9890.5–16865.8)	128.6 (121.3–137.1)	13.3 (9.1–18.3)
1	Low back pain	9.5 (8.4–10.7)	0.0274 (0.0198–0.0357)	1222.4 (882.2–1600.7)	90.9 (77.9–104.9)	-7.1 (-13.6–-0.1)
2	Other musculoskeletal disorders	7.8 (6.1–9.6)	0.0225 (0.0161–0.0302)	1054.1 (749.3–1415.2)	207.4 (175.3–245.1)	53.9 (37.9–73.5)
3	Depressive disorders	7.3 (5.5–9.6)	0.0212 (0.0141–0.0313)	1127.9 (737.5–1678.2)	179.3 (124.8–240.2)	55.6 (24.9–88.2)
4	Diabetes mellitus	5.7 (5.0–6.5)	0.0166 (0.0117–0.0224)	584.7 (414.5–791.6)	461.4 (415.8–510.9)	128.6 (111.1–147.8)
5	Drug use disorders	5.3 (4.0–6.6)	0.0152 (0.0108–0.0197)	860.2 (611.7–1117.5)	530.9 (463.4–608.0)	262.7 (220.6–309.0)
6	Anxiety disorders	5.3 (3.8–7.2)	0.0152 (0.00943–0.0226)	789.8 (486.9–1170.3)	136.6 (84.1–195.4)	29.9 (1.5–62.4)
7	Headache disorders	4.5 (1.0–8.9)	0.0131 (0.00278–0.0276)	683.4 (136.1–1445.3)	77.3 (67.7–93.1)	-3.0 (-8.2–2.5)
8	Age-related and other hearing loss	4.1 (3.3–5.1)	0.0120 (0.00832–0.0165)	421.6 (294.5–584.6)	122.6 (114.2–131.9)	-5.7 (-9.0–-2.2)
9	Falls	3.4 (2.8–4.0)	0.00975 (0.00686–0.0136)	349.5 (244.1–483.1)	159.1 (140.2–175.8)	6.4 (-0.3–12.5)
10	Osteoarthritis	3.0 (1.7–5.6)	0.00868 (0.00421–0.0175)	299.8 (144.3–601.8)	165.3 (157.4–173.3)	7.6 (4.3–10.7)
11	Asthma	2.4 (1.8–3.0)	0.00685 (0.00446–0.0100)	356.7 (231.7–526.9)	73.7 (58.9–89.9)	-6.4 (-13.2–1.9)
12	Oral disorders	2.4 (1.7–3.3)	0.00683 (0.00413–0.0104)	274.5 (159.6–423.1)	107.3 (89.3–123.7)	-5.4 (-13.5–2.9)
13	Chronic obstructive pulmonary disease	2.3 (1.8–2.8)	0.00651 (0.00553–0.00749)	210.3 (179.6–241.3)	156.8 (137.5–179.9)	1.6 (-5.7–10.6)
14	Neck pain	1.7 (1.3–2.2)	0.00495 (0.00335–0.00706)	237.1 (158.0–342.0)	93.6 (83.0–105.3)	-1.0 (-3.9–1.5)
15	Alzheimer's disease and other dementias	1.7 (1.3–2.1)	0.00478 (0.00329–0.00637)	146.4 (100.7–194.5)	133.1 (122.6–145.5)	-6.8 (-10.9–-2.2)
16	Schizophrenia	1.5 (1.1–2.0)	0.00437 (0.00323–0.00554)	221.1 (162.8–284.5)	75.1 (62.2–90.1)	-6.0 (-12.9–1.7)
17	Gynecological diseases	1.5 (1.2–1.8)	0.00423 (0.00291–0.00593)	227.0 (156.4–319.3)	48.2 (36.6–60.1)	-15.1 (-22.1–-7.7)
18	Road injuries	1.5 (1.2–1.7)	0.00423 (0.00301–0.00567)	182.3 (131.0–244.2)	24.4 (20.4–28.6)	-41.4 (-43.1–-39.7)
19	Stroke	1.5 (1.2–1.7)	0.00421 (0.00302–0.00538)	149.9 (107.5–191.8)	114.6 (97.5–134.1)	-7.9 (-14.9–-0.2)
20	Alcohol use disorders	1.4 (1.2–1.8)	0.00419 (0.00288–0.00587)	219.2 (151.3–307.4)	44.0 (30.2–58.8)	-22.0 (-28.9–-13.6)
21	Autism spectrum disorders	1.3 (0.8–1.8)	0.00362 (0.00251–0.00507)	202.6 (140.7–283.1)	79.3 (65.2–96.0)	1.9 (-6.4–11.4)
22	Bipolar disorder	1.2 (0.8–1.6)	0.00338 (0.00225–0.00479)	173.0 (115.5–245.3)	88.7 (76.5–100.2)	0.7 (-5.9–7.0)
23	Blindness and vision loss	1.1 (0.8–1.6)	0.00325 (0.00209–0.00488)	132.8 (84.1–204.4)	123.6 (109.6–138.2)	3.0 (-3.7–9.8)
24	COVID-19	1.1 (0.4–2.5)	0.00314 (0.00115–0.00706)	156.9 (55.1–365.0)	0.0 (0.0–0.0)	0.0 (0.0–0.0)
25	Chronic kidney disease	1.0 (0.8–1.2)	0.00292 (0.00206–0.00379)	107.6 (77.1–139.3)	197.7 (169.5–229.6)	29.9 (18.7–42.0)

Table S3: Cause-specific YLDs by US state in 2021 and percentage change between 1990 and 2021, Illinois

Rank	Cause Name	2021 Percentage of all cause YLDs	2021 YLDs (millions)	2021 Age Standardised Rate (per 100 000)	Percentage change YLD count 1990 to 2021	Percentage change age-standardised YLD rate 1990 to 2021
	All causes	100.0 (100.0–100.0)	2.01 (1.53–2.58)	12915.7 (9754.1–16670.2)	36.8 (32.2–42.0)	13.1 (9.0–18.0)
1	Low back pain	9.3 (8.2–10.6)	0.188 (0.136–0.242)	1184.1 (856.3–1539.1)	15.3 (6.7–23.6)	-5.6 (-12.1–1.5)
2	Other musculoskeletal disorders	7.4 (5.9–9.2)	0.149 (0.106–0.199)	984.4 (700.1–1320.9)	82.9 (61.2–108.4)	55.0 (36.7–75.3)
3	Diabetes mellitus	6.2 (5.4–7.1)	0.125 (0.0887–0.170)	618.3 (437.5–839.1)	241.6 (216.8–269.1)	131.9 (115.6–150.0)
4	Depressive disorders	5.7 (4.3–7.3)	0.114 (0.0780–0.163)	876.0 (592.0–1244.2)	55.3 (24.5–86.1)	48.2 (18.6–77.6)
5	Drug use disorders	5.6 (4.2–7.1)	0.112 (0.0783–0.146)	898.6 (624.8–1178.1)	255.2 (215.6–302.6)	257.1 (215.7–305.4)
6	Anxiety disorders	5.4 (4.0–7.3)	0.107 (0.0690–0.156)	794.6 (510.0–1183.0)	40.5 (7.4–79.3)	29.6 (-1.0–64.1)
7	Headache disorders	4.6 (1.0–8.8)	0.0926 (0.0202–0.195)	687.3 (136.9–1449.0)	5.7 (-0.5–16.4)	-3.2 (-8.9–2.4)
8	Age-related and other hearing loss	4.2 (3.4–5.2)	0.0853 (0.0594–0.119)	419.0 (292.3–583.6)	34.4 (29.1–39.8)	-5.6 (-9.0–-2.1)
9	Osteoarthritis	3.2 (1.9–6.1)	0.0648 (0.0315–0.130)	308.5 (148.4–618.9)	58.3 (53.8–62.6)	5.7 (2.7–8.6)
10	Falls	3.0 (2.4–3.5)	0.0595 (0.0417–0.0833)	295.0 (204.6–409.8)	29.1 (20.3–37.3)	-13.1 (-18.3–-8.0)
11	Chronic obstructive pulmonary disease	2.6 (2.0–3.2)	0.0506 (0.0429–0.0581)	230.5 (195.3–264.1)	66.3 (53.8–79.4)	10.5 (2.5–19.2)
12	Oral disorders	2.4 (1.7–3.4)	0.0487 (0.0287–0.0749)	271.6 (157.5–419.0)	24.9 (14.4–34.8)	-6.3 (-14.4–2.6)
13	Asthma	2.4 (1.9–3.0)	0.0484 (0.0315–0.0702)	388.6 (251.3–572.0)	9.8 (1.5–18.7)	-0.3 (-6.9–7.8)
14	Alzheimer's disease and other dementias	1.8 (1.4–2.3)	0.0361 (0.0251–0.0480)	147.7 (102.5–196.5)	41.0 (33.9–48.8)	-8.9 (-13.5–-4.2)
15	Neck pain	1.8 (1.4–2.2)	0.0357 (0.0238–0.0510)	238.1 (157.8–345.8)	16.7 (9.7–23.7)	-0.9 (-3.6–2.2)
16	Stroke	1.7 (1.4–2.0)	0.0335 (0.0240–0.0428)	165.4 (118.8–211.8)	32.3 (21.9–44.2)	-6.6 (-13.7–1.2)
17	Schizophrenia	1.6 (1.2–2.1)	0.0320 (0.0237–0.0406)	222.3 (165.0–282.2)	5.4 (-2.2–13.0)	-5.5 (-12.3–1.4)
18	Gynecological diseases	1.5 (1.2–1.8)	0.0305 (0.0209–0.0427)	230.1 (158.3–328.3)	-12.6 (-19.6–-5.5)	-15.6 (-22.6–-8.0)
19	Alcohol use disorders	1.4 (1.2–1.8)	0.0288 (0.0199–0.0400)	211.4 (146.2–293.2)	-19.2 (-26.5–-12.1)	-24.9 (-31.5–-18.9)
20	Neonatal disorders	1.2 (1.0–1.5)	0.0244 (0.0171–0.0320)	215.8 (151.3–282.4)	-3.6 (-20.3–15.9)	-5.5 (-22.0–13.9)
21	Autism spectrum disorders	1.2 (0.8–1.8)	0.0244 (0.0172–0.0342)	202.1 (142.6–283.7)	8.5 (-0.5–18.2)	2.5 (-6.2–12.4)
22	Chronic kidney disease	1.2 (1.0–1.5)	0.0239 (0.0172–0.0307)	121.3 (87.1–157.4)	73.4 (58.8–91.1)	24.2 (14.8–35.5)
23	Blindness and vision loss	1.2 (0.8–1.7)	0.0234 (0.0149–0.0357)	131.5 (81.5–202.1)	36.7 (28.1–47.1)	2.1 (-3.9–9.2)
24	Road injuries	1.1 (1.0–1.3)	0.0229 (0.0163–0.0309)	136.6 (97.3–185.1)	-30.5 (-32.8–-28.0)	-46.0 (-47.6–-44.3)
25	COVID-19	1.1 (0.4–2.3)	0.0216 (0.00806–0.0503)	153.6 (54.4–362.5)	0.0 (0.0–0.0)	0.0 (0.0–0.0)

Table S3: Cause-specific YLDs by US state in 2021 and percentage change between 1990 and 2021, Indiana						
**Rank**	**Cause Name**	**2021 Percentage of all cause YLDs**	**2021 YLDs (millions)**	**2021 Age Standardised Rate (per 100 000)**	**Percentage change YLD count 1990 to 2021**	**Percentage change age-standardised YLD rate 1990 to 2021**
	All causes	100.0 (100.0–100.0)	1.12 (0.852–1.44)	13720.3 (10275.3–17599.9)	54.6 (49.9–61.1)	18.2 (13.9–23.4)
1	Low back pain	9.5 (8.4–10.8)	0.107 (0.0780–0.140)	1283.2 (942.6–1687.9)	27.2 (18.3–36.5)	-3.6 (-9.5–3.3)
2	Other musculoskeletal disorders	7.1 (5.8–8.8)	0.0800 (0.0568–0.107)	994.2 (703.7–1330.3)	96.5 (73.6–123.1)	52.1 (34.9–72.7)
3	Diabetes mellitus	7.0 (6.0–7.9)	0.0788 (0.0552–0.107)	735.1 (518.9–987.3)	290.5 (261.1–322.3)	144.9 (127.0–164.2)
4	Depressive disorders	6.8 (5.1–9.0)	0.0767 (0.0498–0.111)	1110.4 (719.4–1603.0)	79.0 (41.7–118.6)	56.3 (22.9–92.5)
5	Drug use disorders	6.7 (5.1–8.5)	0.0746 (0.0523–0.0968)	1148.7 (808.5–1493.0)	495.6 (419.6–580.3)	452.2 (379.1–533.3)
6	Anxiety disorders	5.0 (3.6–6.8)	0.0559 (0.0369–0.0802)	788.0 (516.6–1125.1)	51.5 (18.9–96.9)	29.0 (1.3–68.4)
7	Headache disorders	4.3 (1.0–8.3)	0.0483 (0.0104–0.102)	683.3 (135.8–1446.3)	13.8 (7.5–25.3)	-3.6 (-8.6–2.0)
8	Age-related and other hearing loss	4.0 (3.2–4.9)	0.0445 (0.0311–0.0615)	417.7 (293.7–580.6)	44.5 (39.4–50.7)	-5.6 (-8.5—2.1)
9	Osteoarthritis	3.2 (1.8–6.0)	0.0356 (0.0173–0.0717)	323.2 (156.6–648.8)	69.3 (64.7–74.6)	5.3 (2.4–8.4)
10	Falls	2.8 (2.2–3.3)	0.0310 (0.0217–0.0435)	295.4 (204.3–410.4)	57.3 (45.6–68.5)	-0.9 (-7.3–5.2)
11	Chronic obstructive pulmonary disease	2.6 (2.1–3.2)	0.0284 (0.0246–0.0326)	251.1 (218.6–286.7)	74.5 (60.9–90.8)	9.1 (0.7–19.1)
12	Asthma	2.3 (1.8–3.0)	0.0264 (0.0171–0.0382)	398.2 (260.8–588.4)	26.5 (17.3–37.9)	4.3 (-2.8–13.2)
13	Oral disorders	2.3 (1.6–3.3)	0.0262 (0.0156–0.0398)	278.5 (159.8–427.3)	37.0 (25.1–47.1)	-4.3 (-12.8–3.6)
14	Alzheimer's disease and other dementias	2.1 (1.6–2.6)	0.0230 (0.0160–0.0307)	185.6 (128.3–248.0)	49.2 (41.6–56.6)	-7.8 (-12.2—3.2)
15	Stroke	1.7 (1.4–2.0)	0.0190 (0.0139–0.0241)	180.2 (131.4–228.9)	52.2 (39.1–65.6)	0.4 (-7.4–8.7)
16	Neck pain	1.6 (1.3–2.1)	0.0185 (0.0125–0.0264)	236.7 (156.8–342.6)	25.0 (18.4–32.7)	-1.4 (-4.3–1.6)
17	Schizophrenia	1.5 (1.1–1.9)	0.0164 (0.0122–0.0211)	219.3 (163.0–285.4)	12.4 (4.2–23.7)	-6.4 (-13.3–1.8)
18	Gynecological diseases	1.4 (1.2–1.7)	0.0160 (0.0111–0.0224)	233.2 (159.6–328.9)	-7.5 (-14.2—0.8)	-16.8 (-23.3—10.3)
19	Road injuries	1.3 (1.1–1.5)	0.0146 (0.0104–0.0196)	167.2 (120.0–225.3)	-15.0 (-17.9—11.8)	-38.5 (-40.4—36.5)
20	Alcohol use disorders	1.3 (1.0–1.6)	0.0144 (0.0101–0.0197)	201.5 (140.1–279.0)	-6.4 (-14.6–2.9)	-20.0 (-27.1—12.5)
21	Autism spectrum disorders	1.2 (0.8–1.7)	0.0129 (0.00884–0.0178)	200.3 (137.4–278.6)	18.2 (9.8–28.1)	1.5 (-5.8–10.0)
22	Chronic kidney disease	1.1 (0.9–1.3)	0.0124 (0.00872–0.0160)	122.5 (87.2–157.3)	91.3 (74.3–110.1)	29.8 (19.1–41.7)
23	Blindness and vision loss	1.1 (0.8–1.6)	0.0121 (0.00777–0.0184)	130.9 (82.1–201.8)	45.8 (36.2–56.0)	1.9 (-4.9–8.9)
24	Neonatal disorders	1.0 (0.9–1.3)	0.0117 (0.00829–0.0156)	194.4 (137.4–260.4)	12.1 (-8.8–39.2)	0.0 (-19.0–24.5)
25	Other cardiovascular and circulatory diseases	0.9 (0.7–1.2)	0.0105 (0.00662–0.0161)	105.4 (66.1–159.8)	133.4 (86.9–190.8)	53.8 (26.2–88.5)

Table S3: Cause-specific YLDs by US state in 2021 and percentage change between 1990 and 2021, Iowa

Rank	Cause Name	2021 Percentage of all cause YLDs	2021 YLDs (millions)	2021 Age Standardised Rate (per 100 000)	Percentage change YLD count 1990 to 2021	Percentage change age-standardised YLD rate 1990 to 2021
	All causes	100.0 (100.0–100.0)	0.500 (0.378–0.642)	12812.8 (9594.7–16587.9)	36.4 (32.4–41.2)	13.2 (9.4–17.8)
1	Low back pain	10.6 (9.3–11.9)	0.0528 (0.0379–0.0692)	1374.3 (980.7–1805.8)	13.3 (6.9–20.3)	-5.5 (-11.2–0.4)
2	Other musculoskeletal disorders	8.2 (6.5–10.1)	0.0408 (0.0285–0.0548)	1127.3 (787.8–1525.6)	79.6 (63.1–98.1)	54.8 (40.6–71.9)
3	Diabetes mellitus	6.3 (5.4–7.1)	0.0314 (0.0222–0.0428)	606.9 (429.4–825.3)	224.8 (200.6–251.5)	133.2 (115.9–150.3)
4	Depressive disorders	5.8 (4.5–7.7)	0.0292 (0.0193–0.0420)	911.7 (595.7–1316.2)	56.6 (28.6–87.0)	44.6 (18.6–73.7)
5	Anxiety disorders	5.0 (3.5–6.7)	0.0250 (0.0160–0.0351)	757.0 (485.4–1049.4)	37.5 (7.1–73.7)	24.1 (-3.4–57.2)
6	Age-related and other hearing loss	4.5 (3.6–5.5)	0.0225 (0.0156–0.0309)	421.2 (294.5–585.5)	26.6 (22.0–32.2)	-5.2 (-8.2–-1.4)
7	Headache disorders	4.4 (1.0–8.7)	0.0223 (0.00490–0.0469)	683.5 (136.4–1445.0)	7.8 (2.0–18.6)	-3.7 (-8.7–1.5)
8	Falls	3.9 (3.3–4.7)	0.0198 (0.0140–0.0277)	371.9 (259.7–517.1)	57.9 (48.3–67.1)	9.5 (2.9–15.9)
9	Drug use disorders	3.9 (2.9–4.9)	0.0192 (0.0133–0.0250)	647.0 (449.2–844.9)	288.3 (240.2–343.5)	271.5 (223.4–326.0)
10	Osteoarthritis	3.2 (1.9–5.9)	0.0160 (0.00778–0.0322)	293.2 (141.8–585.2)	50.4 (46.0–55.7)	7.0 (3.7–10.7)
11	Chronic obstructive pulmonary disease	2.6 (2.1–3.3)	0.0128 (0.0110–0.0147)	221.2 (190.3–252.9)	46.8 (37.6–57.2)	5.0 (-1.7–12.7)
12	Oral disorders	2.2 (1.5–3.1)	0.0111 (0.00663–0.0174)	246.5 (144.3–392.1)	27.5 (9.5–46.5)	-0.4 (-14.9–15.6)
13	Asthma	2.2 (1.7–2.8)	0.0111 (0.00722–0.0162)	347.4 (227.0–518.2)	15.2 (6.3–25.4)	1.6 (-6.3–11.0)
14	Alzheimer's disease and other dementias	2.1 (1.6–2.6)	0.0102 (0.00706–0.0135)	151.8 (104.2–200.1)	23.6 (17.2–30.5)	-6.6 (-11.1–-1.8)
15	Neck pain	1.7 (1.3–2.2)	0.00871 (0.00583–0.0124)	237.5 (157.3–347.4)	17.1 (10.8–23.4)	-1.2 (-4.3–1.6)
16	Stroke	1.6 (1.3–1.9)	0.00793 (0.00573–0.0101)	148.1 (106.8–188.0)	30.5 (19.1–41.7)	-0.8 (-8.3–7.4)
17	Schizophrenia	1.6 (1.1–2.1)	0.00768 (0.00568–0.00971)	223.1 (165.9–283.7)	7.3 (-0.6–15.8)	-5.8 (-12.3–1.6)
18	Road injuries	1.5 (1.3–1.7)	0.00752 (0.00530–0.0101)	181.0 (128.6–243.1)	-15.2 (-17.6–-12.6)	-33.8 (-35.5–-32.0)
19	Alcohol use disorders	1.4 (1.1–1.7)	0.00700 (0.00480–0.00973)	212.8 (145.4–296.6)	-11.5 (-19.8–-2.0)	-21.3 (-28.1–-13.4)
20	Gynecological diseases	1.4 (1.1–1.7)	0.00688 (0.00476–0.00958)	216.8 (148.9–307.8)	-14.5 (-21.3–-7.5)	-19.0 (-26.1–-11.9)
21	Autism spectrum disorders	1.2 (0.8–1.8)	0.00615 (0.00425–0.00839)	204.1 (141.1–281.1)	12.8 (4.6–23.2)	2.0 (-5.5–11.7)
22	Blindness and vision loss	1.2 (0.9–1.7)	0.00601 (0.00389–0.00896)	131.9 (82.8–203.0)	30.0 (22.7–38.7)	2.3 (-3.6–9.2)
23	COVID-19	1.1 (0.4–2.6)	0.00572 (0.00217–0.0130)	162.9 (55.1–376.7)	0.0 (0.0–0.0)	0.0 (0.0–0.0)
24	Chronic kidney disease	1.1 (0.9–1.4)	0.00559 (0.00392–0.00731)	106.9 (74.8–137.8)	69.9 (54.8–86.3)	30.8 (19.1–44.0)
25	Neonatal disorders	1.0 (0.9–1.3)	0.00523 (0.00369–0.00681)	185.2 (131.1–240.3)	10.7 (-11.3–35.8)	2.7 (-17.7–26.3)

Table S3: Cause-specific YLDs by US state in 2021 and percentage change between 1990 and 2021, Kansas						
Rank	Cause Name	2021 Percentage of all cause YLDs	2021 YLDs (millions)	2021 Age Standardised Rate (per 100 000)	Percentage change YLD count 1990 to 2021	Percentage change age-standardised YLD rate 1990 to 2021
	All causes	100.0 (100.0–100.0)	0.460 (0.349–0.588)	13126.1 (9836.1–16856.3)	42.6 (37.8–47.6)	14.2 (9.9–18.7)
1	Low back pain	9.8 (8.6–11.1)	0.0452 (0.0331–0.0591)	1279.7 (933.6–1686.7)	17.7 (10.8–25.8)	-7.1 (-12.2—0.7)
2	Other musculoskeletal disorders	7.3 (5.8–9.0)	0.0334 (0.0236–0.0449)	990.3 (693.3–1322.6)	84.8 (66.3–105.9)	49.1 (33.9–65.9)
3	Depressive disorders	6.6 (5.0–8.7)	0.0303 (0.0203–0.0432)	1022.5 (688.1–1459.0)	69.0 (37.8–105.7)	50.3 (22.3–84.3)
4	Diabetes mellitus	6.3 (5.4–7.1)	0.0290 (0.0205–0.0391)	642.6 (453.4–865.5)	252.6 (225.5–279.7)	140.0 (121.6–157.8)
5	Anxiety disorders	5.2 (3.6–7.2)	0.0239 (0.0149–0.0350)	785.7 (488.9–1147.3)	48.0 (16.6–89.0)	28.9 (2.1–64.4)
6	Drug use disorders	5.1 (3.9–6.4)	0.0233 (0.0163–0.0298)	831.5 (580.5–1068.3)	294.1 (246.7–345.5)	267.2 (222.5–317.8)
7	Headache disorders	4.4 (1.0–8.6)	0.0207 (0.00445–0.0434)	682.7 (135.3–1441.8)	12.2 (6.4–22.3)	-3.3 (-8.2–2.3)
8	Age-related and other hearing loss	4.2 (3.3–5.1)	0.0192 (0.0135–0.0268)	419.4 (296.4–584.3)	31.0 (25.5–36.7)	-5.7 (-9.0—2.0)
9	Falls	3.3 (2.8–3.9)	0.0154 (0.0108–0.0215)	335.8 (233.4–465.8)	62.2 (50.8–72.1)	9.3 (1.9–15.7)
10	Osteoarthritis	3.0 (1.7–5.5)	0.0138 (0.00674–0.0277)	294.0 (142.6–585.0)	57.3 (52.8–62.0)	6.6 (3.5–9.8)
11	Oral disorders	2.5 (1.7–3.3)	0.0113 (0.00691–0.0168)	281.8 (168.9–427.3)	28.9 (23.8–34.0)	-3.4 (-7.4–0.6)
12	Chronic obstructive pulmonary disease	2.5 (2.0–3.1)	0.0112 (0.00964–0.0128)	227.6 (195.2–259.4)	54.0 (43.4–66.5)	5.5 (-1.5–13.8)
13	Asthma	2.4 (1.8–3.1)	0.0111 (0.00719–0.0165)	389.3 (253.0–589.8)	23.7 (14.1–33.4)	6.7 (-1.6–15.7)
14	Alzheimer's disease and other dementias	1.8 (1.4–2.2)	0.00811 (0.00566–0.0108)	146.1 (102.0–195.7)	25.3 (18.6–31.9)	-8.9 (-13.3—4.4)
15	Neck pain	1.7 (1.3–2.2)	0.00791 (0.00532–0.0113)	237.2 (157.8–344.2)	21.3 (14.9–28.2)	-1.1 (-3.9–1.6)
16	Stroke	1.7 (1.4–2.0)	0.00779 (0.00563–0.00987)	170.1 (122.4–214.7)	37.2 (26.8–49.9)	0.2 (-6.2–8.3)
17	Schizophrenia	1.5 (1.1–2.0)	0.00705 (0.00521–0.00900)	222.1 (164.1–285.9)	10.2 (1.9–18.4)	-5.7 (-12.7–1.2)
18	Road injuries	1.5 (1.3–1.8)	0.00704 (0.00499–0.00947)	189.7 (135.0–256.5)	-14.0 (-16.5—11.0)	-34.7 (-36.5—32.7)
19	Alcohol use disorders	1.5 (1.2–1.8)	0.00682 (0.00463–0.00955)	224.2 (150.2–314.9)	-10.3 (-19.7—0.8)	-21.7 (-29.3—13.6)
20	Gynecological diseases	1.4 (1.2–1.8)	0.00668 (0.00460–0.00936)	226.5 (155.3–322.8)	-8.2 (-15.2—1.0)	-16.0 (-23.1—8.7)
21	Autism spectrum disorders	1.2 (0.8–1.8)	0.00564 (0.00391–0.00794)	202.4 (140.2–285.6)	15.6 (7.0–24.9)	1.6 (-5.8–10.4)
22	Blindness and vision loss	1.1 (0.8–1.6)	0.00524 (0.00336–0.00790)	131.6 (82.7–201.1)	35.2 (26.2–45.7)	2.2 (-4.1–9.3)
23	Chronic kidney disease	1.1 (0.9–1.3)	0.00506 (0.00358–0.00650)	114.4 (80.0–146.8)	73.0 (58.1–90.7)	28.9 (17.5–40.0)
24	Neonatal disorders	1.0 (0.8–1.2)	0.00459 (0.00327–0.00606)	174.9 (123.6–232.1)	16.8 (-6.1–44.2)	5.8 (-15.1–31.3)
25	COVID-19	1.0 (0.4–2.3)	0.00454 (0.00162–0.0106)	142.6 (48.7–343.6)	0.0 (0.0–0.0)	0.0 (0.0–0.0)

Table S3: Cause-specific YLDs by US state in 2021 and percentage change between 1990 and 2021, Kentucky						
Rank	Cause Name	2021 Percentage of all cause YLDs	2021 YLDs (millions)	2021 Age Standardised Rate (per 100 000)	Percentage change YLD count 1990 to 2021	Percentage change age-standardised YLD rate 1990 to 2021
	All causes	100.0 (100.0–100.0)	0.790 (0.598–0.996)	14549.3 (10933.1–18396.6)	60.1 (54.4–66.8)	23.1 (17.9–28.8)
1	Low back pain	10.0 (8.9–11.2)	0.0787 (0.0571–0.102)	1416.6 (1028.1–1839.4)	32.8 (23.4–43.2)	0.5 (-6.1–8.4)
2	Drug use disorders	8.5 (6.4–10.6)	0.0665 (0.0480–0.0853)	1548.3 (1118.8–1993.0)	603.5 (520.5–696.5)	557.0 (475.0–649.3)
3	Diabetes mellitus	6.8 (5.9–7.7)	0.0540 (0.0382–0.0731)	749.9 (532.9–1006.5)	284.1 (255.8–316.1)	140.3 (122.3–159.7)
4	Other musculoskeletal disorders	6.7 (5.4–8.3)	0.0531 (0.0375–0.0710)	1000.7 (711.4–1340.1)	101.1 (79.6–128.4)	57.7 (40.3–79.7)
5	Depressive disorders	6.7 (5.1–8.6)	0.0528 (0.0349–0.0737)	1170.0 (768.7–1627.6)	87.7 (55.5–124.8)	67.8 (38.7–102.0)
6	Anxiety disorders	4.9 (3.4–6.6)	0.0390 (0.0247–0.0565)	835.3 (530.8–1223.0)	57.7 (24.6–99.3)	36.4 (6.6–74.6)
7	Headache disorders	4.0 (0.9–7.8)	0.0316 (0.00701–0.0670)	676.9 (135.1–1434.3)	11.7 (5.6–24.7)	-4.4 (-9.3–1.0)
8	Age-related and other hearing loss	3.8 (3.0–4.7)	0.0302 (0.0211–0.0418)	417.4 (292.5–574.8)	46.5 (40.8–52.4)	-5.7 (-9.0–-2.4)
9	Osteoarthritis	3.1 (1.8–5.8)	0.0245 (0.0120–0.0492)	325.5 (158.9–650.5)	72.3 (68.0–77.4)	5.3 (2.3–8.5)
10	Falls	3.0 (2.5–3.6)	0.0240 (0.0168–0.0335)	336.4 (232.8–464.6)	59.8 (47.8–70.7)	-0.9 (-7.5–5.1)
11	Chronic obstructive pulmonary disease	2.5 (2.0–3.1)	0.0193 (0.0167–0.0219)	251.4 (218.4–286.3)	69.3 (58.1–82.4)	4.6 (-2.1–12.3)
12	Oral disorders	2.4 (1.7–3.3)	0.0191 (0.0114–0.0291)	297.3 (173.7–456.2)	43.5 (30.3–55.5)	-1.7 (-9.9–7.4)
13	Asthma	2.2 (1.7–2.8)	0.0172 (0.0112–0.0252)	394.2 (254.1–585.3)	24.9 (15.1–37.2)	3.7 (-4.1–13.3)
14	Stroke	1.8 (1.5–2.1)	0.0141 (0.0103–0.0179)	197.5 (143.6–249.2)	59.1 (45.1–73.1)	3.9 (-4.4–12.6)
15	Neck pain	1.6 (1.2–2.0)	0.0123 (0.00824–0.0174)	235.3 (156.3–344.0)	24.4 (17.2–32.1)	-1.8 (-4.8–1.1)
16	Alzheimer's disease and other dementias	1.6 (1.2–2.0)	0.0123 (0.00857–0.0163)	146.6 (102.0–194.4)	51.0 (43.2–59.2)	-8.1 (-12.9–-3.3)
17	Road injuries	1.5 (1.2–1.7)	0.0116 (0.00824–0.0156)	196.9 (141.1–265.5)	-4.3 (-7.3–-0.9)	-31.4 (-33.2–-29.4)
18	Gynecological diseases	1.4 (1.1–1.7)	0.0108 (0.00742–0.0148)	236.9 (162.2–333.4)	-9.4 (-16.2–-2.3)	-18.0 (-24.6–-10.6)
19	Schizophrenia	1.4 (1.0–1.8)	0.0108 (0.00793–0.0137)	216.6 (159.4–278.5)	11.8 (2.7–21.3)	-6.8 (-14.2–0.9)
20	Alcohol use disorders	1.1 (0.9–1.3)	0.00852 (0.00596–0.0118)	180.6 (125.5–251.2)	-7.3 (-16.3–2.7)	-20.0 (-27.6–-12.4)
21	Autism spectrum disorders	1.1 (0.7–1.6)	0.00843 (0.00584–0.0118)	198.6 (137.5–277.6)	17.2 (8.0–27.1)	1.3 (-6.8–10.0)
22	Chronic kidney disease	1.1 (0.9–1.3)	0.00838 (0.00594–0.0109)	122.4 (86.7–159.0)	98.6 (80.5–118.5)	32.8 (21.5–45.5)
23	Blindness and vision loss	1.0 (0.7–1.5)	0.00820 (0.00524–0.0125)	131.1 (82.4–202.6)	46.6 (37.2–58.1)	1.5 (-4.8–8.8)
24	COVID-19	1.0 (0.4–2.1)	0.00752 (0.00286–0.0167)	153.2 (55.2–353.5)	0.0 (0.0–0.0)	0.0 (0.0–0.0)
25	Neonatal disorders	0.9 (0.8–1.2)	0.00739 (0.00524–0.00974)	189.7 (133.9–250.0)	17.5 (-4.2–43.3)	7.0 (-12.6–30.6)

Table S3: Cause-specific YLDs by US state in 2021 and percentage change between 1990 and 2021, Louisiana						
Rank	Cause Name	2021 Percentage of all cause YLDs	2021 YLDs (millions)	2021 Age Standardised Rate (per 100 000)	Percentage change YLD count 1990 to 2021	Percentage change age-standardised YLD rate 1990 to 2021
	All causes	100.0 (100.0–100.0)	0.761 (0.583–0.973)	13779.8 (10508.9–17656.2)	41.7 (37.0–47.6)	17.0 (12.7–22.1)
1	Low back pain	9.5 (8.4–10.7)	0.0723 (0.0524–0.0939)	1266.4 (914.1–1649.7)	16.9 (9.6–25.6)	-6.2 (-11.7–0.3)
2	Drug use disorders	7.2 (5.5–9.1)	0.0546 (0.0385–0.0708)	1210.6 (852.5–1573.1)	372.6 (319.9–435.4)	377.2 (323.4–441.5)
3	Diabetes mellitus	6.8 (5.8–7.7)	0.0518 (0.0364–0.0695)	730.2 (515.2–978.6)	250.1 (220.8–279.4)	132.1 (113.7–150.1)
4	Other musculoskeletal disorders	6.5 (5.1–8.1)	0.0492 (0.0351–0.0657)	890.1 (636.9–1190.7)	81.3 (62.1–103.5)	49.3 (32.9–67.6)
5	Depressive disorders	6.0 (4.6–7.7)	0.0456 (0.0309–0.0656)	973.4 (654.1–1413.6)	58.3 (30.2–93.5)	52.3 (24.8–85.5)
6	Anxiety disorders	5.1 (3.6–7.1)	0.0389 (0.0252–0.0572)	804.4 (520.9–1194.3)	40.9 (9.1–76.9)	31.2 (1.6–64.6)
7	Headache disorders	4.3 (1.0–8.4)	0.0329 (0.00714–0.0693)	682.4 (135.9–1441.5)	3.3 (-2.8–15.5)	-4.2 (-9.4–1.8)
8	Age-related and other hearing loss	3.9 (3.1–4.8)	0.0295 (0.0206–0.0410)	418.5 (294.1–580.5)	37.7 (32.6–43.7)	-5.4 (-8.6–-1.8)
9	Osteoarthritis	3.0 (1.8–5.6)	0.0231 (0.0113–0.0467)	316.0 (153.3–636.1)	62.3 (57.6–67.7)	5.5 (2.6–8.7)
10	Falls	2.5 (2.1–3.0)	0.0194 (0.0135–0.0270)	280.6 (194.5–387.6)	41.3 (30.7–51.4)	-4.1 (-9.9–2.0)
11	Oral disorders	2.5 (1.7–3.4)	0.0188 (0.0116–0.0279)	297.4 (178.3–446.4)	35.5 (30.2–41.5)	-0.1 (-4.5–4.5)
12	Chronic obstructive pulmonary disease	2.4 (1.9–3.0)	0.0182 (0.0157–0.0208)	243.1 (209.6–276.9)	65.5 (54.4–79.4)	8.2 (0.9–17.2)
13	Asthma	2.2 (1.7–2.8)	0.0165 (0.0107–0.0240)	362.6 (232.9–537.6)	4.8 (-3.5–14.1)	-2.9 (-9.7–5.5)
14	Stroke	1.8 (1.5–2.2)	0.0139 (0.0100–0.0178)	200.6 (144.2–256.1)	46.0 (35.6–57.3)	1.3 (-5.5–8.5)
15	Neck pain	1.6 (1.3–2.1)	0.0126 (0.00844–0.0177)	236.5 (157.7–342.3)	15.4 (8.7–22.3)	-1.5 (-4.3–1.3)
16	Gynecological diseases	1.5 (1.2–1.8)	0.0116 (0.00815–0.0161)	243.8 (171.1–343.8)	-13.5 (-19.5–-6.4)	-16.9 (-23.3–-9.2)
17	Alzheimer's disease and other dementias	1.5 (1.2–1.9)	0.0115 (0.00793–0.0153)	145.2 (99.9–192.5)	46.4 (39.3–53.5)	-7.1 (-11.2–-2.9)
18	Road injuries	1.5 (1.3–1.7)	0.0114 (0.00817–0.0155)	194.8 (139.9–263.1)	-14.9 (-17.7–-12.3)	-33.6 (-35.5–-31.9)
19	Schizophrenia	1.5 (1.1–1.9)	0.0112 (0.00836–0.0143)	217.8 (162.3–280.4)	4.4 (-3.9–13.9)	-6.5 (-13.6–2.2)
20	Neonatal disorders	1.3 (1.1–1.7)	0.0102 (0.00742–0.0136)	249.3 (179.2–331.4)	7.6 (-12.6–30.0)	9.3 (-11.2–32.4)
21	Alcohol use disorders	1.3 (1.1–1.6)	0.00997 (0.00681–0.0138)	205.1 (140.6–286.2)	-17.8 (-25.5–-8.7)	-22.9 (-29.8–-14.8)
22	Chronic kidney disease	1.3 (1.0–1.5)	0.00956 (0.00677–0.0121)	144.2 (102.5–184.3)	76.1 (60.8–93.4)	26.1 (15.8–36.6)
23	Autism spectrum disorders	1.2 (0.7–1.7)	0.00867 (0.00596–0.0121)	196.0 (135.8–273.8)	6.5 (-3.8–17.0)	2.3 (-7.4–12.1)
24	Blindness and vision loss	1.1 (0.7–1.5)	0.00805 (0.00516–0.0124)	130.9 (81.6–204.2)	35.4 (26.9–45.7)	1.4 (-4.7–8.7)
25	Dermatitis	1.0 (0.7–1.4)	0.00771 (0.00460–0.0122)	176.0 (102.8–278.2)	-5.1 (-9.6–-0.7)	-8.9 (-13.9–-4.0)

Table S3: Cause-specific YLDs by US state in 2021 and percentage change between 1990 and 2021, Maine

Rank	Cause Name	2021 Percentage of all cause YLDs	2021 YLDs (millions)	2021 Age Standardised Rate (per 100 000)	Percentage change YLD count 1990 to 2021	Percentage change age-standardised YLD rate 1990 to 2021
	All causes	100.0 (100.0–100.0)	0.240 (0.182–0.306)	13353.8 (9998.1–17052.8)	47.6 (43.2–53.0)	15.9 (11.7–20.6)
1	Low back pain	9.6 (8.4–10.9)	0.0230 (0.0167–0.0298)	1253.5 (913.0–1634.8)	20.8 (11.9–30.7)	-6.4 (-12.4–0.5)
2	Other musculoskeletal disorders	8.3 (6.6–10.3)	0.0199 (0.0140–0.0268)	1185.9 (843.0–1592.1)	90.6 (71.6–112.9)	58.1 (43.6–76.3)
3	Diabetes mellitus	7.3 (6.3–8.2)	0.0175 (0.0122–0.0238)	656.7 (459.9–889.4)	295.0 (262.5–329.9)	128.8 (111.6–146.3)
4	Depressive disorders	5.6 (4.1–7.2)	0.0133 (0.00859–0.0184)	980.3 (635.4–1376.9)	45.2 (16.6–77.2)	42.8 (15.3–73.7)
5	Drug use disorders	5.5 (4.1–6.9)	0.0131 (0.00902–0.0167)	1093.7 (745.8–1404.4)	367.3 (311.0–434.9)	410.1 (343.8–491.9)
6	Age-related and other hearing loss	4.6 (3.7–5.8)	0.0111 (0.00780–0.0153)	417.9 (295.3–578.2)	55.7 (49.4–62.5)	-5.7 (-8.9–-2.0)
7	Anxiety disorders	4.5 (3.2–6.1)	0.0108 (0.00646–0.0155)	755.4 (442.4–1101.4)	30.7 (0.5–64.1)	23.4 (-5.9–56.2)
8	Headache disorders	4.0 (1.0–7.7)	0.00960 (0.00226–0.0201)	683.8 (139.1–1457.5)	2.0 (-4.3–17.8)	-3.4 (-8.3–2.5)
9	Falls	3.8 (3.2–4.5)	0.00923 (0.00648–0.0128)	354.4 (246.4–486.9)	77.3 (64.7–88.3)	3.5 (-2.7–8.9)
10	Osteoarthritis	3.5 (2.0–6.5)	0.00841 (0.00411–0.0169)	303.6 (146.6–609.3)	86.1 (80.4–92.5)	6.2 (3.2–9.6)
11	Chronic obstructive pulmonary disease	2.9 (2.3–3.7)	0.00689 (0.00593–0.00789)	234.8 (203.6–269.4)	89.4 (76.3–104.8)	6.7 (-0.3–15.1)
12	Oral disorders	2.6 (1.9–3.5)	0.00627 (0.00380–0.00946)	281.7 (163.3–431.2)	42.1 (29.4–52.4)	-4.2 (-12.6–4.3)
13	Asthma	2.2 (1.7–2.8)	0.00525 (0.00343–0.00763)	398.7 (259.2–592.5)	15.5 (5.6–25.7)	6.0 (-1.6–15.4)
14	Alzheimer's disease and other dementias	2.1 (1.6–2.6)	0.00494 (0.00340–0.00656)	148.3 (102.6–196.7)	63.0 (54.1–71.2)	-9.0 (-13.7–-4.3)
15	Stroke	1.7 (1.4–2.0)	0.00395 (0.00291–0.00505)	150.3 (110.6–191.9)	56.8 (44.4–70.6)	-3.3 (-10.2–4.7)
16	Neck pain	1.6 (1.3–2.1)	0.00394 (0.00266–0.00549)	237.2 (157.0–340.5)	18.6 (8.5–29.5)	-1.1 (-3.8–1.7)
17	Schizophrenia	1.4 (1.0–1.9)	0.00339 (0.00250–0.00428)	221.4 (163.8–282.1)	3.0 (-4.9–12.1)	-5.9 (-13.0–2.0)
18	Road injuries	1.4 (1.2–1.6)	0.00330 (0.00237–0.00443)	168.6 (121.3–227.9)	-11.3 (-14.1–-8.1)	-35.1 (-36.8–-33.2)
19	Gynecological diseases	1.3 (1.0–1.6)	0.00306 (0.00210–0.00422)	227.2 (156.6–319.7)	-21.2 (-27.7–-15.1)	-18.7 (-25.5–-12.2)
20	Alcohol use disorders	1.3 (1.0–1.5)	0.00304 (0.00209–0.00422)	215.1 (145.4–302.4)	-16.5 (-24.0–-6.6)	-20.2 (-27.1–-11.6)
21	Blindness and vision loss	1.2 (0.9–1.7)	0.00286 (0.00184–0.00431)	131.6 (82.0–201.5)	49.4 (39.4–59.3)	1.8 (-4.8–8.3)
22	Chronic kidney disease	1.2 (0.9–1.4)	0.00275 (0.00191–0.00356)	108.6 (76.3–140.5)	107.3 (86.9–130.9)	32.5 (20.4–46.6)
23	Atrial fibrillation and flutter	1.1 (0.9–1.3)	0.00273 (0.00191–0.00362)	85.8 (59.8–113.7)	120.6 (73.0–181.1)	21.9 (-3.4–54.4)
24	Autism spectrum disorders	1.1 (0.7–1.6)	0.00254 (0.00177–0.00356)	204.1 (143.9–286.1)	4.2 (-4.5–13.1)	1.7 (-6.0–10.4)
25	Other mental disorders	0.9 (0.7–1.3)	0.00223 (0.00146–0.00323)	130.9 (83.8–191.4)	19.3 (13.2–25.6)	-0.7 (-5.3–4.8)

Table S3: Cause-specific YLDs by US state in 2021 and percentage change between 1990 and 2021, Maryland

Rank	Cause Name	2021 Percentage of all cause YLDs	2021 YLDs (millions)	2021 Age Standardised Rate (per 100 000)	Percentage change YLD count 1990 to 2021	Percentage change age-standardised YLD rate 1990 to 2021
	All causes	100.0 (100.0–100.0)	0.968 (0.738–1.24)	12772.5 (9648.0–16439.6)	61.7 (56.0–67.5)	12.9 (9.1–17.3)
1	Low back pain	8.8 (7.7–10.0)	0.0852 (0.0618–0.111)	1078.1 (782.2–1400.5)	30.1 (20.4–40.9)	-10.3 (-16.3–-3.0)
2	Diabetes mellitus	6.9 (6.0–7.8)	0.0670 (0.0468–0.0902)	680.3 (478.4–918.0)	331.6 (299.9–367.5)	138.5 (122.1–157.3)
3	Other musculoskeletal disorders	6.7 (5.3–8.4)	0.0652 (0.0457–0.0874)	870.0 (615.8–1163.0)	99.6 (76.4–125.9)	43.3 (28.1–61.1)
4	Depressive disorders	6.1 (4.7–8.1)	0.0593 (0.0387–0.0857)	950.6 (610.5–1373.5)	131.4 (86.9–179.2)	98.4 (60.0–140.9)
5	Anxiety disorders	5.5 (3.9–7.8)	0.0532 (0.0341–0.0773)	812.8 (512.7–1192.0)	62.2 (21.3–103.6)	31.9 (-1.6–64.9)
6	Headache disorders	4.7 (1.1–9.1)	0.0456 (0.0101–0.0960)	699.3 (138.8–1499.1)	21.0 (14.2–34.2)	-2.0 (-6.5–3.2)
7	Drug use disorders	4.3 (3.3–5.5)	0.0415 (0.0295–0.0538)	693.8 (489.7–902.3)	250.2 (211.3–299.0)	217.4 (181.1–262.4)
8	Age-related and other hearing loss	4.2 (3.4–5.1)	0.0409 (0.0288–0.0564)	415.9 (290.0–573.8)	69.4 (62.4–78.0)	-5.7 (-9.0–-1.8)
9	Osteoarthritis	3.4 (2.0–6.4)	0.0334 (0.0163–0.0671)	326.7 (158.3–654.2)	98.0 (92.5–104.5)	5.7 (2.7–9.1)
10	Falls	3.1 (2.6–3.7)	0.0303 (0.0212–0.0421)	310.2 (214.7–428.9)	83.7 (70.3–96.5)	1.8 (-4.2–8.0)
11	Asthma	2.6 (2.0–3.3)	0.0251 (0.0163–0.0372)	421.4 (273.6–628.3)	42.8 (32.6–54.7)	10.4 (2.4–20.1)
12	Chronic obstructive pulmonary disease	2.5 (2.0–3.2)	0.0243 (0.0207–0.0277)	229.2 (194.3–260.9)	101.8 (87.9–119.9)	5.1 (-2.0–14.1)
13	Oral disorders	2.4 (1.7–3.2)	0.0230 (0.0141–0.0330)	266.1 (163.2–386.9)	47.8 (21.8–81.6)	-7.7 (-23.1–10.9)
14	Neck pain	1.8 (1.4–2.3)	0.0174 (0.0117–0.0247)	238.1 (156.8–343.1)	33.4 (25.3–43.3)	-1.0 (-3.7–2.0)
15	Stroke	1.8 (1.5–2.1)	0.0172 (0.0125–0.0222)	175.7 (126.5–225.5)	76.3 (62.7–92.4)	-1.9 (-9.1–5.8)
16	Alzheimer's disease and other dementias	1.8 (1.4–2.2)	0.0170 (0.0117–0.0223)	145.2 (100.6–191.7)	97.2 (87.1–107.8)	-8.4 (-12.6–-3.8)
17	Schizophrenia	1.6 (1.2–2.2)	0.0157 (0.0116–0.0201)	223.0 (166.8–282.2)	17.9 (8.6–27.9)	-5.6 (-12.2–1.9)
18	Gynecological diseases	1.6 (1.3–1.9)	0.0155 (0.0108–0.0213)	239.8 (165.8–339.2)	-2.8 (-10.2–5.1)	-15.9 (-23.2–-8.6)
19	Alcohol use disorders	1.3 (1.0–1.6)	0.0124 (0.00835–0.0173)	187.8 (126.6–264.2)	-10.1 (-18.0–-0.9)	-24.7 (-31.0–-17.5)
20	Road injuries	1.3 (1.1–1.4)	0.0122 (0.00873–0.0163)	149.5 (107.4–200.2)	-10.7 (-13.5–-7.6)	-40.2 (-41.7–-38.4)
21	Chronic kidney disease	1.2 (1.0–1.5)	0.0120 (0.00843–0.0153)	125.6 (88.4–161.8)	112.7 (92.2–134.7)	22.3 (12.2–32.7)
22	Neonatal disorders	1.2 (1.0–1.5)	0.0118 (0.00851–0.0158)	215.9 (155.4–291.6)	-2.0 (-18.3–17.2)	-15.8 (-29.9–0.6)
23	Blindness and vision loss	1.2 (0.8–1.7)	0.0113 (0.00717–0.0173)	131.1 (81.6–202.5)	68.4 (57.5–80.8)	2.9 (-3.0–9.8)
24	Dermatitis	1.1 (0.8–1.6)	0.0109 (0.00643–0.0172)	190.7 (109.4–303.6)	12.8 (8.0–17.9)	-8.2 (-12.7–-3.1)
25	Autism spectrum disorders	1.0 (0.7–1.6)	0.00977 (0.00653–0.0138)	167.7 (111.3–237.5)	22.9 (12.2–34.6)	1.2 (-7.8–11.1)

Table S3: Cause-specific YLDs by US state in 2021 and percentage change between 1990 and 2021, Massachusetts						
**Rank**	**Cause Name**	**2021 Percentage of all cause YLDs**	**2021 YLDs (millions)**	**2021 Age Standardised Rate (per 100 000)**	**Percentage change YLD count 1990 to 2021**	**Percentage change age-standardised YLD rate 1990 to 2021**
	All causes	100.0<br>(100.0–100.0)	1.16<br>(0.877–1.48)	13136.9<br>(9823.4–16858.5)	43.1<br>(38.5–48.2)	12.7<br>(8.5–17.2)
1	Low back pain	8.2<br>(7.3–9.2)	0.0951<br>(0.0689–0.123)	1021.7<br>(744.5–1331.8)	8.8<br>(1.2–17.0)	-18.0<br>(-23.9–-12.2)
2	Other musculoskeletal disorders	7.2<br>(5.6–8.9)	0.0829<br>(0.0585–0.111)	946.0<br>(670.4–1271.3)	79.3<br>(61.3–103.4)	41.2<br>(26.8–59.7)
3	Drug use disorders	7.1<br>(5.4–8.9)	0.0822<br>(0.0569–0.106)	1171.8<br>(805.6–1519.3)	416.0<br>(346.1–496.7)	402.5<br>(331.9–486.5)
4	Diabetes mellitus	6.1<br>(5.3–6.9)	0.0711<br>(0.0499–0.0953)	596.9<br>(420.3–803.9)	252.8<br>(226.2–280.0)	122.3<br>(106.2–138.8)
5	Depressive disorders	6.0<br>(4.5–8.0)	0.0693<br>(0.0440–0.101)	953.7<br>(605.2–1395.5)	54.6<br>(27.5–87.5)	43.0<br>(17.6–73.0)
6	Anxiety disorders	5.2<br>(3.6–7.0)	0.0597<br>(0.0373–0.0868)	787.5<br>(495.0–1148.3)	45.5<br>(11.6–85.8)	28.6<br>(-1.2–63.5)
7	Headache disorders	4.4<br>(1.0–8.7)	0.0520<br>(0.0115–0.109)	690.7<br>(137.7–1467.7)	10.6<br>(4.8–23.9)	-2.9<br>(-8.1–3.5)
8	Age-related and other hearing loss	4.2<br>(3.3–5.2)	0.0489<br>(0.0343–0.0679)	413.3<br>(287.8–575.8)	40.9<br>(36.0–46.9)	-5.3<br>(-8.2–-2.0)
9	Falls	3.6<br>(2.9–4.2)	0.0415<br>(0.0291–0.0577)	356.3<br>(247.0–488.8)	53.8<br>(43.7–62.9)	-1.0<br>(-6.8–4.1)
10	Osteoarthritis	3.3<br>(1.9–6.2)	0.0383<br>(0.0186–0.0774)	311.8<br>(150.0–629.6)	68.2<br>(61.3–75.3)	5.8<br>(1.3–10.1)
11	Oral disorders	2.4<br>(1.7–3.4)	0.0279<br>(0.0165–0.0420)	270.1<br>(157.8–410.7)	31.4<br>(20.6–42.6)	-6.5<br>(-14.4–2.6)
12	Asthma	2.4<br>(1.8–3.0)	0.0275<br>(0.0180–0.0406)	412.8<br>(267.8–619.4)	23.2<br>(14.3–33.2)	7.2<br>(-0.9–15.7)
13	Chronic obstructive pulmonary disease	2.4<br>(1.9–3.0)	0.0274<br>(0.0234–0.0317)	211.4<br>(179.9–242.9)	58.2<br>(47.4–71.6)	-0.6<br>(-7.2–7.9)
14	Alzheimer's disease and other dementias	1.9<br>(1.5–2.4)	0.0222<br>(0.0153–0.0296)	156.5<br>(106.8–207.6)	44.2<br>(36.8–50.9)	-6.1<br>(-10.9–-1.7)
15	Neck pain	1.7<br>(1.4–2.2)	0.0202<br>(0.0135–0.0285)	238.4<br>(158.9–340.6)	22.6<br>(14.5–31.1)	-0.7<br>(-3.7–2.3)
16	Schizophrenia	1.6<br>(1.2–2.1)	0.0183<br>(0.0135–0.0234)	223.3<br>(163.5–285.4)	10.2<br>(1.8–18.7)	-5.7<br>(-13.0–1.6)
17	Gynecological diseases	1.6<br>(1.3–1.9)	0.0180<br>(0.0125–0.0254)	241.9<br>(165.8–345.5)	-9.8<br>(-15.9–-2.5)	-16.2<br>(-22.7–-9.0)
18	Alcohol use disorders	1.4<br>(1.2–1.8)	0.0168<br>(0.0116–0.0230)	216.7<br>(151.7–300.5)	-9.1<br>(-18.5–0.6)	-18.6<br>(-26.1–-10.5)
19	Stroke	1.4<br>(1.2–1.7)	0.0166<br>(0.0120–0.0208)	142.1<br>(102.6–178.3)	35.3<br>(24.3–46.1)	-7.0<br>(-14.0–0.1)
20	Autism spectrum disorders	1.2<br>(0.8–1.8)	0.0137<br>(0.00959–0.0193)	206.7<br>(143.6–290.3)	14.2<br>(4.2–24.4)	2.1<br>(-6.2–11.4)
21	Blindness and vision loss	1.2<br>(0.8–1.6)	0.0133<br>(0.00842–0.0203)	130.7<br>(80.9–201.5)	44.2<br>(33.8–55.7)	3.2<br>(-3.6–9.6)
22	Bipolar disorder	1.1<br>(0.8–1.5)	0.0125<br>(0.00816–0.0177)	162.5<br>(105.6–229.9)	14.8<br>(7.5–21.9)	-1.0<br>(-7.5–5.7)
23	Neonatal disorders	1.0<br>(0.8–1.3)	0.0118<br>(0.00818–0.0155)	190.1<br>(131.4–251.2)	-0.4<br>(-19.2–24.4)	-7.8<br>(-25.6–15.2)
24	Dermatitis	1.0<br>(0.7–1.4)	0.0117<br>(0.00699–0.0183)	181.3<br>(105.1–287.7)	1.5<br>(-3.1–6.4)	-8.7<br>(-13.6–-3.5)
25	Chronic kidney disease	1.0<br>(0.8–1.3)	0.0117<br>(0.00825–0.0154)	101.2<br>(70.5–132.6)	75.3<br>(56.9–94.6)	20.6<br>(9.2–32.5)

Table S3: Cause-specific YLDs by US state in 2021 and percentage change between 1990 and 2021, Michigan						
Rank	Cause Name	2021 Percentage of all cause YLDs	2021 YLDs (millions)	2021 Age Standardised Rate (per 100 000)	Percentage change YLD count 1990 to 2021	Percentage change age-standardised YLD rate 1990 to 2021
	All causes	100.0 (100.0–100.0)	1.72 (1.31–2.19)	13721.5 (10436.0–17571.2)	42.9 (38.1–48.3)	17.4 (13.2–22.5)
1	Low back pain	9.0 (8.0–10.1)	0.154 (0.112–0.201)	1184.4 (857.3–1550.3)	14.5 (6.4–23.4)	-8.2 (-14.1–-1.3)
2	Other musculoskeletal disorders	7.3 (5.8–9.1)	0.126 (0.0894–0.168)	1018.2 (728.1–1354.8)	74.5 (57.7–94.2)	46.2 (32.4–62.5)
3	Diabetes mellitus	7.1 (6.1–8.0)	0.123 (0.0862–0.166)	702.0 (495.9–948.3)	301.2 (269.0–337.8)	150.9 (131.1–172.1)
4	Drug use disorders	6.4 (4.9–8.1)	0.110 (0.0768–0.143)	1167.1 (812.8–1521.4)	333.9 (284.8–392.6)	365.0 (310.4–430.8)
5	Depressive disorders	6.1 (4.6–8.1)	0.105 (0.0714–0.146)	1040.9 (705.3–1448.5)	57.4 (27.7–90.3)	58.1 (28.6–89.9)
6	Anxiety disorders	4.9 (3.4–6.5)	0.0842 (0.0550–0.122)	803.1 (525.5–1160.8)	36.0 (3.6–74.2)	31.2 (0.0–68.1)
7	Age-related and other hearing loss	4.2 (3.3–5.2)	0.0721 (0.0504–0.0993)	416.2 (291.7–575.7)	45.2 (39.2–51.4)	-6.0 (-9.5–-2.6)
8	Headache disorders	3.9 (1.0–7.6)	0.0673 (0.0160–0.139)	646.6 (137.1–1362.2)	0.8 (-4.9–14.3)	-2.9 (-7.2–2.2)
9	Osteoarthritis	3.6 (2.1–6.8)	0.0631 (0.0308–0.128)	351.4 (169.3–712.7)	66.9 (61.7–72.8)	3.0 (-0.1–6.4)
10	Falls	3.0 (2.5–3.5)	0.0513 (0.0361–0.0719)	298.6 (207.7–414.9)	63.0 (51.0–74.7)	2.2 (-4.2–8.4)
11	Chronic obstructive pulmonary disease	2.7 (2.2–3.5)	0.0467 (0.0404–0.0533)	250.1 (217.1–285.0)	87.5 (72.9–106.8)	14.5 (5.8–25.5)
12	Asthma	2.4 (1.8–3.0)	0.0409 (0.0268–0.0595)	426.1 (277.3–633.0)	14.4 (5.1–23.4)	9.0 (0.7–17.6)
13	Oral disorders	2.3 (1.6–3.2)	0.0399 (0.0231–0.0613)	272.0 (158.9–421.0)	26.3 (15.5–37.0)	-7.7 (-15.3–0.7)
14	Stroke	1.8 (1.5–2.1)	0.0312 (0.0227–0.0395)	183.7 (133.0–232.4)	54.1 (42.1–67.9)	1.5 (-5.8–9.4)
15	Alzheimer's disease and other dementias	1.8 (1.4–2.3)	0.0309 (0.0215–0.0405)	147.5 (102.5–193.4)	63.1 (54.3–71.9)	-7.6 (-12.7–-2.9)
16	Neck pain	1.6 (1.3–2.0)	0.0281 (0.0189–0.0398)	236.9 (156.7–342.1)	13.8 (5.7–23.0)	-1.3 (-3.9–1.3)
17	Schizophrenia	1.4 (1.1–1.9)	0.0244 (0.0182–0.0312)	219.4 (162.4–281.5)	-0.1 (-7.8–8.3)	-6.3 (-13.6–1.2)
18	Gynecological diseases	1.4 (1.2–1.7)	0.0244 (0.0169–0.0336)	242.5 (167.5–343.5)	-18.5 (-24.7–-12.3)	-15.8 (-22.1–-8.5)
19	Alcohol use disorders	1.4 (1.1–1.7)	0.0237 (0.0165–0.0327)	225.3 (155.6–312.4)	-20.0 (-27.4–-11.3)	-22.2 (-28.5–-13.8)
20	Road injuries	1.2 (1.0–1.3)	0.0199 (0.0142–0.0267)	146.4 (104.1–196.3)	-23.6 (-26.2–-20.9)	-41.2 (-42.9–-39.6)
21	Chronic kidney disease	1.2 (1.0–1.4)	0.0199 (0.0140–0.0258)	121.4 (85.7–157.6)	84.8 (69.6–101.6)	24.8 (15.6–35.7)
22	Blindness and vision loss	1.1 (0.8–1.6)	0.0191 (0.0122–0.0289)	130.4 (81.5–201.4)	41.3 (32.4–50.9)	1.5 (-4.5–8.2)
23	Neonatal disorders	1.1 (0.9–1.4)	0.0191 (0.0136–0.0253)	221.1 (156.0–294.0)	-0.2 (-18.0–21.5)	4.4 (-13.8–28.0)
24	Autism spectrum disorders	1.1 (0.7–1.7)	0.0188 (0.0132–0.0264)	200.2 (141.2–279.8)	3.1 (-4.5–11.6)	1.8 (-5.8–10.3)
25	Bipolar disorder	1.0 (0.7–1.4)	0.0177 (0.0119–0.0248)	162.6 (108.6–228.1)	6.7 (-0.2–14.1)	-1.7 (-7.7–5.1)

Table S3: Cause-specific YLDs by US state in 2021 and percentage change between 1990 and 2021, Minnesota

Rank	Cause Name	2021 Percentage of all cause YLDs	2021 YLDs (millions)	2021 Age Standardised Rate (per 100 000)	Percentage change YLD count 1990 to 2021	Percentage change age-standardised YLD rate 1990 to 2021
	All causes	100.0 (100.0–100.0)	0.869 (0.652–1.12)	12398.1 (9259.8–16035.0)	56.8 (52.0–62.4)	9.2 (5.7–13.8)
1	Low back pain	8.9 (7.8–10.1)	0.0775 (0.0563–0.101)	1082.4 (788.9–1415.8)	31.5 (21.9–41.4)	-10.1 (-16.4–-3.4)
2	Other musculoskeletal disorders	6.9 (5.4–8.6)	0.0602 (0.0423–0.0817)	911.9 (645.7–1228.9)	107.6 (82.9–138.2)	49.0 (32.2–70.5)
3	Depressive disorders	6.3 (4.8–8.4)	0.0546 (0.0365–0.0782)	945.4 (627.5–1380.9)	73.8 (44.5–112.5)	41.4 (18.4–71.2)
4	Diabetes mellitus	6.2 (5.4–7.1)	0.0544 (0.0382–0.0749)	586.6 (411.6–804.1)	318.0 (286.2–357.0)	131.3 (114.3–151.4)
5	Anxiety disorders	5.3 (3.8–7.3)	0.0463 (0.0290–0.0669)	772.5 (482.0–1114.2)	60.5 (23.2–103.2)	26.5 (-4.0–59.0)
6	Headache disorders	4.7 (1.1–9.1)	0.0409 (0.00900–0.0860)	688.0 (137.2–1460.5)	23.5 (16.7–38.7)	-2.9 (-7.7–2.9)
7	Age-related and other hearing loss	4.5 (3.6–5.5)	0.0389 (0.0272–0.0536)	419.2 (290.8–576.5)	60.8 (54.0–68.6)	-5.3 (-8.6–-1.1)
8	Falls	4.3 (3.6–5.1)	0.0376 (0.0265–0.0523)	400.8 (278.5–554.1)	84.5 (72.2–95.3)	4.0 (-2.1–9.6)
9	Drug use disorders	4.1 (3.1–5.2)	0.0356 (0.0248–0.0460)	660.3 (458.4–860.1)	303.2 (250.9–373.2)	251.4 (205.1–314.5)
10	Osteoarthritis	3.2 (1.9–6.1)	0.0281 (0.0137–0.0570)	291.8 (140.7–590.4)	95.0 (89.5–101.3)	6.5 (3.7–9.6)
11	Oral disorders	2.5 (1.7–3.4)	0.0217 (0.0128–0.0332)	267.7 (155.1–411.9)	47.5 (33.8–59.7)	-7.7 (-16.3–0.8)
12	Asthma	2.3 (1.8–3.0)	0.0205 (0.0134–0.0304)	356.8 (232.1–533.5)	29.7 (19.0–41.0)	-0.9 (-8.2–7.5)
13	Chronic obstructive pulmonary disease	2.1 (1.7–2.7)	0.0180 (0.0151–0.0210)	175.4 (148.6–203.4)	74.6 (61.5–91.1)	-5.1 (-12.1–3.8)
14	Alzheimer's disease and other dementias	2.0 (1.6–2.5)	0.0173 (0.0120–0.0229)	156.5 (107.8–206.3)	63.0 (55.1–71.4)	-4.5 (-9.2–0.2)
15	Neck pain	1.8 (1.4–2.3)	0.0159 (0.0107–0.0225)	238.2 (158.6–347.9)	37.2 (28.1–47.2)	-0.8 (-3.6–2.1)
16	Schizophrenia	1.7 (1.2–2.2)	0.0143 (0.0106–0.0180)	225.1 (165.2–285.3)	22.5 (12.8–31.9)	-5.4 (-12.2–1.3)
17	Alcohol use disorders	1.6 (1.3–2.0)	0.0141 (0.00979–0.0194)	233.7 (160.4–326.5)	-0.5 (-9.0–10.6)	-20.6 (-27.2–-12.7)
18	Stroke	1.5 (1.3–1.8)	0.0134 (0.00953–0.0171)	145.3 (103.7–186.0)	56.4 (44.0–72.0)	-4.9 (-12.3–4.0)
19	Gynecological diseases	1.5 (1.2–1.8)	0.0126 (0.00878–0.0176)	219.7 (151.9–312.7)	-1.9 (-9.7–6.5)	-16.9 (-24.1–-9.8)
20	Road injuries	1.3 (1.1–1.5)	0.0116 (0.00825–0.0155)	153.0 (109.7–205.3)	-12.1 (-15.4–-8.6)	-42.7 (-44.6–-40.7)
21	Autism spectrum disorders	1.3 (0.9–1.9)	0.0113 (0.00765–0.0158)	207.2 (139.9–291.8)	27.2 (17.2–39.7)	2.1 (-5.7–12.3)
22	Blindness and vision loss	1.2 (0.9–1.7)	0.0105 (0.00672–0.0160)	131.4 (82.1–203.3)	62.2 (50.6–74.5)	3.6 (-2.6–10.5)
23	Neonatal disorders	1.2 (1.0–1.4)	0.0101 (0.00701–0.0132)	195.9 (135.8–258.5)	-5.7 (-23.3–17.2)	-21.6 (-36.4–-2.2)
24	Chronic kidney disease	1.0 (0.8–1.3)	0.00898 (0.00635–0.0118)	99.8 (69.7–130.6)	107.7 (88.4–130.5)	28.9 (17.4–42.2)
25	Other mental disorders	1.0 (0.8–1.4)	0.00895 (0.00581–0.0131)	131.9 (85.0–193.9)	37.4 (30.2–45.0)	-0.3 (-5.0–5.2)

Table S3: Cause-specific YLDs by US state in 2021 and percentage change between 1990 and 2021, Mississippi						
Rank	Cause Name	2021 Percentage of all cause YLDs	2021 YLDs (millions)	2021 Age Standardised Rate (per 100 000)	Percentage change YLD count 1990 to 2021	Percentage change age-standardised YLD rate 1990 to 2021
	All causes	100.0 (100.0–100.0)	0.461 (0.350–0.592)	13068.7 (9837.9–16835.5)	43.8 (38.8–50.0)	15.7 (11.2–21.0)
1	Low back pain	8.6 (7.6–9.7)	0.0397 (0.0287–0.0515)	1092.4 (790.5–1414.5)	15.3 (7.8–23.3)	-10.1 (-15.7–-4.1)
2	Depressive disorders	7.0 (5.4–9.2)	0.0323 (0.0212–0.0454)	1077.6 (707.7–1523.9)	72.5 (38.1–109.1)	57.9 (26.0–91.7)
3	Diabetes mellitus	6.9 (5.9–7.8)	0.0318 (0.0222–0.0433)	700.4 (491.7–947.7)	242.3 (215.8–271.0)	128.1 (112.1–146.7)
4	Drug use disorders	6.4 (4.8–8.0)	0.0293 (0.0212–0.0381)	1043.4 (748.1–1355.4)	336.1 (280.9–399.5)	321.3 (267.2–385.4)
5	Anxiety disorders	5.5 (4.0–7.4)	0.0253 (0.0167–0.0373)	828.5 (543.8–1215.2)	50.3 (17.2–89.9)	34.4 (5.7–72.4)
6	Other musculoskeletal disorders	5.1 (4.0–6.4)	0.0234 (0.0166–0.0312)	673.7 (481.1–906.5)	71.4 (51.4–95.0)	38.9 (22.5–58.4)
7	Headache disorders	4.5 (1.0–8.8)	0.0211 (0.00460–0.0442)	688.8 (137.2–1460.4)	8.5 (2.9–22.0)	-3.8 (-8.6–2.6)
8	Age-related and other hearing loss	4.2 (3.4–5.2)	0.0194 (0.0135–0.0271)	421.5 (292.6–586.3)	37.0 (32.0–42.9)	-5.4 (-8.4–-1.8)
9	Falls	3.0 (2.5–3.6)	0.0138 (0.00971–0.0194)	304.6 (212.3–423.2)	50.5 (38.6–60.4)	-0.5 (-7.5–5.3)
10	Osteoarthritis	3.0 (1.7–5.6)	0.0138 (0.00663–0.0277)	288.0 (137.8–578.3)	65.2 (60.3–70.6)	7.5 (4.2–11.0)
11	Oral disorders	2.6 (1.8–3.5)	0.0119 (0.00713–0.0181)	290.5 (169.3–442.1)	33.7 (22.5–44.4)	-2.7 (-11.4–5.3)
12	Chronic obstructive pulmonary disease	2.5 (2.0–3.1)	0.0113 (0.00963–0.0130)	228.9 (195.5–262.5)	62.2 (51.6–75.7)	6.4 (-0.6–15.0)
13	Asthma	2.3 (1.8–3.0)	0.0108 (0.00711–0.0158)	374.0 (244.5–555.5)	15.9 (6.3–27.6)	3.3 (-4.7–13.4)
14	Stroke	2.2 (1.8–2.6)	0.00997 (0.00722–0.0127)	219.4 (159.3–278.2)	52.6 (40.2–65.4)	6.2 (-1.8–14.5)
15	Road injuries	1.8 (1.6–2.1)	0.00852 (0.00609–0.0115)	226.6 (162.2–305.1)	-8.7 (-11.6–-5.9)	-31.4 (-33.4–-29.7)
16	Neck pain	1.7 (1.4–2.2)	0.00806 (0.00540–0.0115)	238.1 (156.7–344.7)	20.4 (13.8–27.0)	-1.3 (-4.1–2.0)
17	Alzheimer's disease and other dementias	1.7 (1.4–2.1)	0.00779 (0.00540–0.0104)	147.9 (102.0–197.1)	39.7 (32.6–47.2)	-5.9 (-10.3–-1.4)
18	Schizophrenia	1.5 (1.2–2.0)	0.00706 (0.00522–0.00898)	219.2 (161.0–279.1)	10.6 (2.6–19.9)	-5.8 (-12.5–1.7)
19	Gynecological diseases	1.4 (1.2–1.7)	0.00669 (0.00463–0.00940)	225.0 (155.7–315.5)	-10.7 (-17.0–-4.0)	-17.7 (-24.4–-10.6)
20	Chronic kidney disease	1.4 (1.1–1.7)	0.00642 (0.00459–0.00823)	149.4 (106.7–191.2)	83.5 (67.6–99.4)	31.1 (20.3–41.3)
21	Neonatal disorders	1.4 (1.1–1.7)	0.00639 (0.00462–0.00866)	249.5 (179.3–338.6)	7.8 (-10.3–31.8)	5.5 (-12.6–28.8)
22	Alcohol use disorders	1.3 (1.0–1.6)	0.00581 (0.00403–0.00802)	189.4 (130.1–264.4)	-13.3 (-21.9–-3.9)	-23.0 (-29.7–-15.3)
23	Autism spectrum disorders	1.2 (0.8–1.8)	0.00538 (0.00367–0.00754)	193.5 (131.5–272.1)	10.1 (2.1–19.5)	1.8 (-5.8–10.6)
24	Blindness and vision loss	1.2 (0.8–1.7)	0.00532 (0.00337–0.00809)	133.0 (82.5–206.7)	37.1 (28.3–48.0)	1.4 (-4.8–9.5)
25	COVID-19	1.1 (0.4–2.5)	0.00516 (0.00194–0.0116)	163.3 (58.3–379.0)	0.0 (0.0–0.0)	0.0 (0.0–0.0)

Table S3: Cause-specific YLDs by US state in 2021 and percentage change between 1990 and 2021, Missouri						
**Rank**	**Cause Name**	**2021 Percentage of all cause YLDs**	**2021 YLDs (millions)**	**2021 Age Standardised Rate (per 100 000)**	**Percentage change YLD count 1990 to 2021**	**Percentage change age-standardised YLD rate 1990 to 2021**
	All causes	100.0 (100.0–100.0)	1.02 (0.770–1.31)	13577.6 (10171.0–17426.5)	51.7 (47.1–57.5)	18.4 (14.0–23.8)
1	Low back pain	9.6 (8.5–10.8)	0.0980 (0.0715–0.127)	1273.6 (919.7–1659.1)	24.2 (16.3–33.4)	-4.9 (-11.0–2.1)
2	Other musculoskeletal disorders	7.1 (5.7–8.8)	0.0731 (0.0513–0.0981)	1006.6 (704.2–1351.8)	98.1 (75.2–125.5)	56.5 (39.4–78.5)
3	Diabetes mellitus	6.7 (5.8–7.6)	0.0687 (0.0484–0.0932)	684.8 (484.2–923.6)	291.7 (260.4–322.4)	154.9 (134.8–175.0)
4	Drug use disorders	6.3 (4.8–8.0)	0.0644 (0.0459–0.0830)	1109.4 (789.0–1433.4)	456.5 (394.0–539.5)	416.3 (356.2–495.8)
5	Depressive disorders	5.8 (4.4–7.7)	0.0590 (0.0387–0.0835)	954.5 (619.9–1350.0)	100.6 (64.2–147.4)	78.6 (45.9–121.3)
6	Anxiety disorders	5.0 (3.5–6.9)	0.0513 (0.0341–0.0761)	800.7 (525.4–1188.8)	50.9 (16.3–87.0)	30.3 (1.0–61.5)
7	Headache disorders	4.2 (1.0–8.3)	0.0436 (0.00961–0.0916)	683.4 (134.9–1444.0)	12.5 (6.4–23.3)	-3.9 (-9.1–2.0)
8	Age-related and other hearing loss	4.1 (3.3–5.0)	0.0425 (0.0298–0.0583)	417.4 (293.3–575.6)	38.3 (33.5–43.9)	-5.8 (-8.7–-2.3)
9	Falls	3.4 (2.8–4.0)	0.0344 (0.0241–0.0479)	340.4 (236.3–470.6)	48.8 (38.7–58.1)	-4.8 (-11.0–0.9)
10	Osteoarthritis	3.2 (1.9–6.0)	0.0330 (0.0161–0.0659)	315.8 (152.8–628.7)	62.7 (58.4–67.9)	5.4 (2.6–8.5)
11	Chronic obstructive pulmonary disease	2.7 (2.1–3.3)	0.0268 (0.0233–0.0307)	246.0 (214.5–280.3)	70.6 (57.6–86.4)	11.0 (2.2–20.9)
12	Oral disorders	2.4 (1.7–3.3)	0.0246 (0.0146–0.0376)	278.8 (160.5–428.6)	32.3 (20.4–42.5)	-4.8 (-13.6–3.2)
13	Asthma	2.3 (1.8–2.9)	0.0237 (0.0155–0.0344)	403.4 (262.4–602.9)	25.3 (16.0–35.9)	8.0 (0.4–17.5)
14	Alzheimer's disease and other dementias	1.8 (1.4–2.3)	0.0183 (0.0125–0.0241)	146.8 (101.0–193.2)	38.6 (31.6–45.4)	-8.7 (-13.2–-4.4)
15	Stroke	1.8 (1.5–2.1)	0.0182 (0.0132–0.0230)	179.7 (129.1–227.3)	47.2 (35.1–58.7)	1.1 (-6.6–9.0)
16	Neck pain	1.7 (1.3–2.1)	0.0170 (0.0115–0.0237)	237.0 (157.0–340.0)	23.5 (16.8–30.7)	-1.4 (-4.5–1.7)
17	Schizophrenia	1.5 (1.1–1.9)	0.0149 (0.0111–0.0191)	219.7 (163.8–281.4)	12.2 (2.9–22.3)	-6.0 (-13.9–2.8)
18	Road injuries	1.5 (1.2–1.7)	0.0149 (0.0106–0.0201)	184.1 (132.0–248.4)	-12.3 (-15.3–-9.4)	-35.7 (-37.6–-33.7)
19	Gynecological diseases	1.4 (1.1–1.7)	0.0143 (0.00983–0.0198)	230.0 (158.4–325.5)	-8.0 (-14.5–-0.8)	-16.5 (-22.7–-9.5)
20	Alcohol use disorders	1.3 (1.0–1.5)	0.0129 (0.00901–0.0176)	200.4 (137.5–277.6)	-7.8 (-16.0–1.8)	-20.5 (-27.6–-12.5)
21	Chronic kidney disease	1.1 (0.9–1.4)	0.0116 (0.00819–0.0149)	119.2 (84.6–153.6)	81.7 (66.6–98.5)	27.1 (17.4–37.4)
22	Autism spectrum disorders	1.1 (0.7–1.7)	0.0116 (0.00793–0.0164)	200.1 (138.5–283.1)	16.8 (7.4–26.6)	2.0 (-6.1–11.0)
23	Blindness and vision loss	1.1 (0.8–1.6)	0.0115 (0.00735–0.0174)	131.5 (82.5–201.9)	40.9 (32.5–50.7)	1.8 (-4.2–8.8)
24	Bipolar disorder	1.1 (0.8–1.5)	0.0115 (0.00760–0.0159)	178.3 (118.4–248.1)	16.5 (9.6–24.1)	-1.6 (-7.5–4.9)
25	Neonatal disorders	1.1 (0.9–1.3)	0.0109 (0.00779–0.0149)	204.2 (145.1–278.8)	15.3 (-5.9–42.9)	4.8 (-14.6–29.9)

Table S3: Cause-specific YLDs by US state in 2021 and percentage change between 1990 and 2021, Montana						
Rank	Cause Name	2021 Percentage of all cause YLDs	2021 YLDs (millions)	2021 Age Standardised Rate (per 100 000)	Percentage change YLD count 1990 to 2021	Percentage change age-standardised YLD rate 1990 to 2021
	All causes	100.0 (100.0–100.0)	0.181 (0.138–0.232)	13196.6 (9887.8–16948.9)	73.6 (68.6–79.9)	13.6 (9.9–18.4)
1	Low back pain	9.5 (8.4–10.8)	0.0172 (0.0125–0.0225)	1214.7 (883.7–1593.4)	45.0 (34.9–55.7)	-7.5 (-13.3–-0.9)
2	Other musculoskeletal disorders	7.5 (6.0–9.3)	0.0137 (0.00958–0.0185)	1050.7 (744.2–1417.1)	133.8 (107.4–163.7)	58.3 (40.6–78.8)
3	Depressive disorders	6.3 (4.6–8.2)	0.0114 (0.00732–0.0159)	1054.1 (684.7–1481.9)	89.1 (50.4–132.8)	47.5 (17.3–80.0)
4	Diabetes mellitus	5.7 (5.0–6.6)	0.0104 (0.00734–0.0142)	530.5 (375.6–715.6)	315.8 (277.6–358.4)	110.6 (93.1–129.9)
5	Drug use disorders	4.9 (3.8–6.2)	0.00889 (0.00622–0.0115)	905.0 (628.1–1173.0)	332.1 (284.9–384.9)	252.8 (213.4–296.8)
6	Anxiety disorders	4.8 (3.4–6.6)	0.00867 (0.00557–0.0126)	769.6 (487.9–1132.6)	67.2 (31.5–113.2)	26.6 (-1.5–61.6)
7	Age-related and other hearing loss	4.5 (3.6–5.6)	0.00823 (0.00577–0.0113)	420.4 (295.8–576.5)	78.3 (71.4–85.7)	-5.6 (-8.9–-2.3)
8	Headache disorders	4.1 (1.0–8.1)	0.00757 (0.00175–0.0160)	679.5 (139.9–1438.2)	27.5 (20.2–42.4)	-3.6 (-8.4–1.9)
9	Falls	4.1 (3.4–4.8)	0.00740 (0.00519–0.0103)	391.0 (271.6–539.4)	115.5 (99.9–129.7)	10.8 (4.1–17.1)
10	Osteoarthritis	3.3 (2.0–6.2)	0.00602 (0.00294–0.0123)	298.7 (144.1–604.7)	112.7 (105.7–120.1)	7.8 (4.5–11.6)
11	Chronic obstructive pulmonary disease	2.7 (2.1–3.3)	0.00478 (0.00409–0.00550)	220.4 (189.2–252.8)	108.8 (94.6–128.3)	2.5 (-3.9–11.1)
12	Oral disorders	2.5 (1.8–3.5)	0.00458 (0.00278–0.00682)	278.8 (164.6–426.4)	64.1 (57.1–70.9)	-3.6 (-8.1–1.2)
13	Asthma	2.5 (1.9–3.2)	0.00456 (0.00298–0.00669)	416.1 (269.9–624.5)	38.0 (26.6–49.6)	2.0 (-5.8–9.9)
14	Alzheimer's disease and other dementias	1.9 (1.5–2.5)	0.00352 (0.00245–0.00469)	147.5 (101.6–194.9)	92.3 (82.9–102.1)	-6.1 (-10.8–-1.4)
15	Road injuries	1.8 (1.6–2.1)	0.00332 (0.00236–0.00447)	225.8 (161.4–305.3)	14.1 (10.6–17.5)	-29.2 (-31.0–-27.4)
16	Alcohol use disorders	1.8 (1.4–2.1)	0.00318 (0.00223–0.00435)	284.6 (198.0–390.2)	7.9 (-2.6–18.6)	-19.1 (-26.2–-12.2)
17	Stroke	1.7 (1.4–2.0)	0.00308 (0.00222–0.00389)	159.0 (114.5–201.9)	76.7 (63.7–91.5)	-5.0 (-11.5–2.4)
18	Neck pain	1.7 (1.3–2.1)	0.00302 (0.00205–0.00418)	236.6 (157.9–340.3)	43.6 (33.6–55.0)	-1.1 (-3.7–1.9)
19	Schizophrenia	1.5 (1.1–1.9)	0.00263 (0.00193–0.00332)	220.3 (163.9–281.5)	27.8 (17.7–39.4)	-5.8 (-13.3–2.5)
20	Gynecological diseases	1.3 (1.0–1.6)	0.00232 (0.00161–0.00323)	217.0 (148.8–307.3)	0.3 (-7.6–9.2)	-17.9 (-25.0–-10.0)
21	Blindness and vision loss	1.2 (0.8–1.7)	0.00214 (0.00139–0.00319)	132.9 (84.1–203.5)	73.4 (63.9–83.8)	2.3 (-3.8–8.9)
22	Autism spectrum disorders	1.1 (0.8–1.7)	0.00204 (0.00140–0.00285)	200.8 (137.1–280.0)	30.8 (18.8–43.1)	1.9 (-7.6–12.0)
23	Chronic kidney disease	1.1 (0.9–1.4)	0.00199 (0.00141–0.00258)	106.9 (76.7–137.6)	134.1 (112.2–158.6)	29.1 (17.9–41.9)
24	Atrial fibrillation and flutter	1.0 (0.8–1.1)	0.00173 (0.00120–0.00228)	74.2 (51.9–97.8)	133.2 (86.2–198.9)	13.1 (-10.5–43.4)
25	Other mental disorders	1.0 (0.7–1.3)	0.00173 (0.00112–0.00253)	132.1 (85.6–195.1)	45.8 (38.6–53.0)	-0.1 (-4.8–4.8)

Table S3: Cause-specific YLDs by US state in 2021 and percentage change between 1990 and 2021, Nebraska

Rank	Cause Name	2021 Percentage of all cause YLDs	2021 YLDs (millions)	2021 Age Standardised Rate (per 100 000)	Percentage change YLD count 1990 to 2021	Percentage change age-standardised YLD rate 1990 to 2021
	All causes	100.0 (100.0–100.0)	0.299 (0.227–0.386)	12639.9 (9512.7–16407.3)	45.3 (41.3–50.2)	10.6 (7.2–14.8)
1	Low back pain	10.6 (9.4–12.1)	0.0317 (0.0231–0.0413)	1355.6 (985.5–1776.1)	24.0 (16.9–31.4)	-5.8 (-11.1–0.1)
2	Other musculoskeletal disorders	6.9 (5.5–8.6)	0.0206 (0.0146–0.0277)	909.6 (646.7–1217.8)	88.3 (69.2–110.8)	43.5 (29.1–60.9)
3	Diabetes mellitus	6.3 (5.5–7.1)	0.0189 (0.0133–0.0256)	619.2 (438.8–830.5)	260.2 (233.8–292.5)	135.1 (118.8–154.7)
4	Depressive disorders	5.8 (4.5–7.7)	0.0175 (0.0115–0.0247)	884.3 (581.2–1261.1)	63.1 (32.3–97.9)	38.0 (11.4–67.9)
5	Anxiety disorders	5.2 (3.6–7.0)	0.0155 (0.00970–0.0228)	762.8 (473.2–1123.8)	50.7 (15.0–91.3)	24.8 (-4.2–58.8)
6	Headache disorders	4.6 (1.0–9.0)	0.0138 (0.00300–0.0291)	685.5 (135.7–1455.8)	17.8 (12.1–27.9)	-3.2 (-8.1–1.9)
7	Age-related and other hearing loss	4.3 (3.4–5.3)	0.0129 (0.00901–0.0179)	419.8 (291.7–585.6)	35.9 (31.0–41.7)	-5.3 (-8.4–-1.7)
8	Falls	3.6 (3.0–4.3)	0.0109 (0.00769–0.0152)	356.9 (249.2–494.9)	61.0 (50.5–71.0)	5.8 (-0.6–11.9)
9	Drug use disorders	3.4 (2.6–4.3)	0.0102 (0.00726–0.0131)	549.2 (388.5–710.5)	263.1 (211.8–318.3)	219.3 (173.1–269.8)
10	Osteoarthritis	3.2 (1.9–6.0)	0.00971 (0.00469–0.0197)	309.3 (148.1–625.4)	63.5 (59.1–68.2)	7.2 (4.1–10.3)
11	Asthma	2.6 (2.0–3.3)	0.00774 (0.00505–0.0113)	381.7 (246.1–566.4)	22.2 (13.2–32.4)	-2.5 (-9.7–5.8)
12	Chronic obstructive pulmonary disease	2.6 (2.0–3.2)	0.00761 (0.00652–0.00874)	230.1 (197.1–262.0)	61.5 (51.2–73.7)	7.7 (0.9–15.6)
13	Oral disorders	2.4 (1.7–3.2)	0.00721 (0.00444–0.0103)	270.6 (167.3–392.2)	28.3 (6.3–55.7)	-6.8 (-21.6–12.1)
14	Alzheimer's disease and other dementias	1.9 (1.5–2.4)	0.00565 (0.00393–0.00750)	149.5 (104.3–197.4)	30.5 (23.1–37.2)	-7.0 (-11.4–-2.3)
15	Neck pain	1.8 (1.4–2.2)	0.00528 (0.00357–0.00753)	237.6 (158.8–344.6)	27.2 (20.9–33.7)	-1.0 (-3.5–2.0)
16	Schizophrenia	1.6 (1.2–2.1)	0.00473 (0.00345–0.00612)	223.9 (162.5–289.5)	16.5 (8.2–26.3)	-5.4 (-12.6–2.3)
17	Stroke	1.6 (1.3–1.8)	0.00468 (0.00333–0.00600)	152.2 (109.1–194.7)	32.6 (22.6–43.0)	-5.9 (-12.7–1.2)
18	Road injuries	1.6 (1.3–1.8)	0.00465 (0.00330–0.00625)	188.1 (134.2–254.6)	-8.5 (-11.0–-5.9)	-33.4 (-35.2–-31.6)
19	Gynecological diseases	1.4 (1.2–1.7)	0.00425 (0.00293–0.00598)	216.5 (148.5–310.7)	-5.6 (-12.9–2.2)	-17.9 (-24.5–-10.5)
20	Alcohol use disorders	1.4 (1.1–1.7)	0.00406 (0.00278–0.00567)	200.9 (137.3–284.2)	-5.4 (-13.2–3.8)	-21.7 (-28.0–-14.6)
21	Autism spectrum disorders	1.3 (0.9–1.9)	0.00383 (0.00267–0.00536)	204.1 (141.5–285.6)	22.6 (13.4–34.2)	2.0 (-5.7–11.4)
22	Blindness and vision loss	1.2 (0.8–1.7)	0.00351 (0.00227–0.00530)	131.5 (82.6–202.3)	39.9 (31.0–50.7)	2.1 (-4.0–9.2)
23	COVID-19	1.2 (0.5–2.6)	0.00347 (0.00129–0.00751)	163.0 (58.9–367.7)	0.0 (0.0–0.0)	0.0 (0.0–0.0)
24	Chronic kidney disease	1.1 (0.9–1.4)	0.00332 (0.00234–0.00428)	111.0 (77.9–142.6)	80.5 (63.5–98.2)	30.0 (18.9–41.6)
25	Other cardiovascular and circulatory diseases	1.1 (0.8–1.4)	0.00323 (0.00206–0.00495)	113.5 (72.9–170.5)	106.4 (73.2–149.2)	39.1 (17.6–66.3)

Table S3: Cause-specific YLDs by US state in 2021 and percentage change between 1990 and 2021, Nevada						
Rank	Cause Name	2021 Percentage of all cause YLDs	2021 YLDs (millions)	2021 Age Standardised Rate (per 100 000)	Percentage change YLD count 1990 to 2021	Percentage change age-standardised YLD rate 1990 to 2021
	All causes	100.0 (100.0–100.0)	0.507 (0.385–0.648)	13408.7 (10089.3–17183.4)	225.4 (214.2–238.6)	14.9 (10.6–20.0)
1	Low back pain	9.5 (8.4–10.7)	0.0482 (0.0349–0.0628)	1220.9 (879.0–1593.6)	174.2 (155.5–194.4)	-4.4 (-10.7–2.8)
2	Depressive disorders	7.2 (5.6–9.7)	0.0367 (0.0242–0.0518)	1126.0 (742.5–1593.8)	264.9 (199.5–339.1)	48.8 (21.9–80.2)
3	Other musculoskeletal disorders	7.0 (5.6–8.6)	0.0354 (0.0250–0.0473)	929.3 (657.0–1239.7)	333.6 (282.5–391.9)	55.8 (37.9–76.9)
4	Drug use disorders	6.6 (5.0–8.2)	0.0331 (0.0232–0.0426)	1029.8 (718.6–1335.9)	807.5 (707.5–922.4)	281.9 (236.6–333.1)
5	Diabetes mellitus	6.1 (5.3–7.0)	0.0312 (0.0223–0.0420)	656.0 (464.0–881.2)	726.7 (660.8–795.2)	143.4 (124.7–161.8)
6	Anxiety disorders	5.4 (3.9–7.4)	0.0273 (0.0171–0.0402)	815.3 (513.2–1206.0)	237.1 (163.5–326.1)	34.6 (4.4–69.9)
7	Headache disorders	4.5 (1.0–8.9)	0.0229 (0.00500–0.0477)	681.8 (136.4–1437.2)	146.3 (133.7–169.6)	-2.4 (-7.4–3.4)
8	Age-related and other hearing loss	3.9 (3.1–4.8)	0.0200 (0.0140–0.0276)	421.0 (295.9–584.3)	226.9 (214.8–239.7)	-6.2 (-9.3—3.0)
9	Osteoarthritis	3.0 (1.7–5.6)	0.0151 (0.00719–0.0303)	307.0 (145.4–615.8)	273.2 (262.3–285.0)	5.9 (2.9–8.9)
10	Falls	2.6 (2.1–3.1)	0.0132 (0.00921–0.0184)	284.2 (196.3–393.2)	244.8 (216.7–270.9)	-0.7 (-7.0–5.3)
11	Chronic obstructive pulmonary disease	2.5 (2.0–3.1)	0.0123 (0.0106–0.0140)	243.8 (211.0–276.9)	287.0 (262.0–317.6)	5.5 (-1.1–13.2)
12	Asthma	2.4 (1.8–3.0)	0.0121 (0.00789–0.0178)	400.7 (262.5–600.6)	154.5 (135.9–173.9)	0.2 (-6.4–7.6)
13	Oral disorders	2.2 (1.6–3.1)	0.0113 (0.00670–0.0173)	263.8 (152.6–404.1)	194.9 (165.6–222.2)	-5.9 (-14.8–2.8)
14	Alcohol use disorders	1.8 (1.5–2.2)	0.00897 (0.00620–0.0125)	260.7 (179.3–363.5)	88.8 (71.7–109.0)	-24.5 (-30.7—17.3)
15	Neck pain	1.7 (1.3–2.2)	0.00872 (0.00589–0.0124)	236.6 (159.0–341.2)	168.6 (155.0–183.6)	-0.8 (-3.7–2.4)
16	Stroke	1.7 (1.4–2.0)	0.00850 (0.00619–0.0110)	183.9 (133.8–236.2)	229.1 (204.5–256.2)	-6.3 (-12.8–1.1)
17	Gynecological diseases	1.6 (1.3–1.9)	0.00812 (0.00567–0.0113)	242.3 (168.7–339.7)	111.0 (96.4–128.4)	-11.9 (-18.7—4.4)
18	Schizophrenia	1.6 (1.2–2.1)	0.00794 (0.00592–0.0100)	220.8 (165.0–281.2)	138.4 (119.7–157.2)	-5.9 (-12.8–1.6)
19	Road injuries	1.5 (1.3–1.8)	0.00786 (0.00562–0.0105)	193.6 (138.2–260.0)	50.9 (46.0–56.3)	-48.6 (-50.0—47.0)
20	Alzheimer's disease and other dementias	1.5 (1.2–1.9)	0.00774 (0.00535–0.0102)	145.5 (100.4–192.3)	307.2 (285.3–331.0)	-9.5 (-13.9—5.2)
21	Autism spectrum disorders	1.2 (0.8–1.8)	0.00601 (0.00417–0.00847)	201.0 (138.9–283.2)	147.5 (127.9–168.7)	1.8 (-6.4–10.4)
22	COVID-19	1.1 (0.5–2.7)	0.00579 (0.00217–0.0133)	168.4 (59.6–398.8)	0.0 (0.0–0.0)	0.0 (0.0–0.0)
23	Blindness and vision loss	1.1 (0.8–1.6)	0.00556 (0.00354–0.00843)	132.3 (83.3–203.7)	228.6 (207.9–249.8)	2.4 (-3.7–9.7)
24	Chronic kidney disease	1.1 (0.9–1.3)	0.00555 (0.00394–0.00715)	121.4 (87.0–156.2)	330.6 (291.7–377.1)	25.3 (14.9–37.4)
25	Other mental disorders	1.0 (0.7–1.3)	0.00494 (0.00321–0.00724)	131.2 (83.9–192.2)	164.9 (151.7–180.4)	-1.6 (-6.1–3.7)

Table S3: Cause-specific YLDs by US state in 2021 and percentage change between 1990 and 2021, New Hampshire						
Rank	Cause Name	2021 Percentage of all cause YLDs	2021 YLDs (millions)	2021 Age Standardised Rate (per 100 000)	Percentage change YLD count 1990 to 2021	Percentage change age-standardised YLD rate 1990 to 2021
	All causes	100.0 (100.0–100.0)	0.241 (0.185–0.308)	13633.2 (10315.5–17390.8)	68.7 (63.0–75.1)	17.8 (13.4–23.1)
1	Low back pain	9.1 (8.0–10.3)	0.0218 (0.0158–0.0283)	1174.6 (857.5–1525.1)	33.9 (23.3–46.8)	-9.5 (-15.4—2.0)
2	Drug use disorders	7.6 (5.7–9.5)	0.0182 (0.0129–0.0237)	1436.0 (1020.6–1880.1)	405.7 (345.1–482.2)	400.7 (339.2–478.5)
3	Other musculoskeletal disorders	7.2 (5.7–9.0)	0.0174 (0.0122–0.0236)	997.3 (703.7–1343.4)	111.2 (88.8–140.3)	49.5 (34.3–69.7)
4	Diabetes mellitus	6.9 (5.9–7.8)	0.0166 (0.0116–0.0227)	648.5 (452.3–884.4)	384.3 (343.1–426.5)	138.3 (118.9–156.8)
5	Depressive disorders	6.3 (4.8–8.4)	0.0153 (0.0101–0.0227)	1095.5 (719.1–1640.2)	73.2 (41.6–109.8)	51.4 (24.8–83.9)
6	Anxiety disorders	4.8 (3.4–6.4)	0.0115 (0.00743–0.0161)	785.6 (506.2–1122.1)	53.5 (19.3–94.6)	28.6 (0.6–62.5)
7	Age-related and other hearing loss	4.3 (3.4–5.3)	0.0104 (0.00725–0.0145)	415.9 (289.7–578.0)	81.4 (73.9–89.9)	-5.6 (-9.0—1.7)
8	Headache disorders	4.1 (1.0–7.9)	0.00990 (0.00226–0.0207)	682.3 (135.2–1442.2)	15.0 (7.6–32.5)	-3.5 (-8.7–2.6)
9	Falls	3.8 (3.1–4.5)	0.00916 (0.00646–0.0128)	372.9 (259.9–513.0)	100.7 (85.4–114.6)	4.5 (-1.6–10.4)
10	Osteoarthritis	3.5 (2.1–6.5)	0.00851 (0.00414–0.0172)	320.9 (155.8–646.2)	119.6 (113.0–127.0)	5.4 (2.4–8.7)
11	Chronic obstructive pulmonary disease	2.6 (2.1–3.2)	0.00623 (0.00533–0.00717)	225.6 (193.3–258.3)	117.5 (103.2–133.9)	3.9 (-2.5–11.8)
12	Oral disorders	2.4 (1.7–3.3)	0.00589 (0.00365–0.00864)	275.3 (171.0–408.4)	61.5 (34.8–96.0)	-5.1 (-19.9–12.6)
13	Asthma	2.2 (1.7–2.8)	0.00533 (0.00350–0.00767)	399.5 (261.7–596.5)	34.0 (21.3–46.6)	7.5 (-1.0–16.2)
14	Alzheimer's disease and other dementias	1.8 (1.4–2.3)	0.00435 (0.00300–0.00576)	147.3 (101.7–194.3)	94.2 (84.0–106.0)	-7.8 (-12.1—2.6)
15	Neck pain	1.7 (1.3–2.1)	0.00400 (0.00267–0.00558)	237.0 (158.3–342.7)	34.1 (21.3–46.9)	-1.1 (-3.8–1.8)
16	Stroke	1.5 (1.3–1.7)	0.00359 (0.00261–0.00461)	146.4 (106.1–185.9)	64.0 (50.4–79.3)	-12.1 (-18.8—4.5)
17	Schizophrenia	1.5 (1.1–2.0)	0.00351 (0.00259–0.00448)	221.5 (165.5–283.4)	15.0 (5.6–25.5)	-6.1 (-13.4–1.6)
18	Alcohol use disorders	1.4 (1.2–1.7)	0.00340 (0.00237–0.00469)	231.6 (159.8–325.8)	-5.8 (-14.9–4.8)	-19.0 (-25.6—10.4)
19	Gynecological diseases	1.3 (1.1–1.6)	0.00316 (0.00219–0.00437)	226.4 (156.4–318.1)	-10.5 (-17.5—3.0)	-17.7 (-24.1—11.1)
20	Road injuries	1.2 (1.1–1.4)	0.00300 (0.00215–0.00405)	152.8 (109.6–206.2)	-4.2 (-7.7—0.3)	-38.6 (-40.6—36.8)
21	Blindness and vision loss	1.1 (0.8–1.7)	0.00276 (0.00175–0.00423)	130.8 (81.8–202.4)	72.7 (59.3–88.7)	2.0 (-4.3–8.9)
22	Autism spectrum disorders	1.1 (0.7–1.6)	0.00264 (0.00182–0.00370)	206.1 (142.3–290.0)	17.7 (6.3–27.7)	2.0 (-7.9–10.2)
23	Atrial fibrillation and flutter	1.0 (0.8–1.2)	0.00251 (0.00173–0.00337)	86.3 (59.6–115.7)	163.2 (108.6–235.3)	23.4 (-1.3–55.2)
24	Chronic kidney disease	1.0 (0.8–1.3)	0.00250 (0.00175–0.00323)	104.0 (73.7–134.4)	136.2 (109.5–161.4)	30.4 (18.1–41.8)
25	Other mental disorders	0.9 (0.7–1.3)	0.00226 (0.00148–0.00328)	130.8 (85.3–192.0)	33.9 (27.0–41.7)	-0.7 (-5.3–4.2)

Table S3: Cause-specific YLDs by US state in 2021 and percentage change between 1990 and 2021, New Jersey

Rank	Cause Name	2021 Percentage of all cause YLDs	2021 YLDs (millions)	2021 Age Standardised Rate (per 100 000)	Percentage change YLD count 1990 to 2021	Percentage change age-standardised YLD rate 1990 to 2021
	All causes	100.0 (100.0–100.0)	1.49 (1.14–1.90)	12926.6 (9841.0–16510.0)	44.8 (40.4–49.9)	12.5 (8.6–16.9)
1	Low back pain	9.1 (8.0–10.2)	0.135 (0.0973–0.177)	1135.6 (812.4–1486.9)	20.9 (12.8–30.5)	-7.5 (-13.6–0.3)
2	Other musculoskeletal disorders	7.4 (5.9–9.2)	0.110 (0.0776–0.147)	956.1 (679.6–1283.4)	80.3 (61.2–103.9)	41.9 (27.2–60.0)
3	Diabetes mellitus	6.5 (5.7–7.4)	0.0974 (0.0687–0.131)	638.4 (448.5–854.7)	267.6 (234.4–302.9)	138.0 (117.0–160.9)
4	Drug use disorders	5.7 (4.3–7.2)	0.0845 (0.0608–0.109)	954.0 (677.3–1237.4)	284.3 (237.3–338.0)	271.0 (224.1–323.4)
5	Depressive disorders	5.3 (3.9–7.1)	0.0788 (0.0511–0.111)	832.4 (544.3–1195.0)	64.1 (33.5–100.8)	48.1 (20.1–80.4)
6	Anxiety disorders	5.3 (3.6–7.2)	0.0786 (0.0504–0.111)	798.3 (512.1–1132.4)	49.0 (16.7–87.8)	30.3 (1.7–63.3)
7	Headache disorders	4.5 (1.1–8.6)	0.0674 (0.0149–0.142)	688.4 (136.5–1447.3)	11.8 (5.9–22.2)	-3.2 (-8.4–2.4)
8	Age-related and other hearing loss	4.3 (3.4–5.3)	0.0636 (0.0444–0.0878)	415.9 (289.5–573.0)	42.3 (36.9–48.4)	-5.5 (-8.8–-1.8)
9	Osteoarthritis	3.3 (2.0–6.2)	0.0502 (0.0242–0.101)	316.0 (151.3–635.6)	65.4 (61.2–70.9)	5.4 (2.8–8.6)
10	Falls	2.8 (2.3–3.4)	0.0423 (0.0296–0.0589)	281.4 (195.5–388.6)	55.2 (43.8–64.8)	-1.5 (-7.6–3.9)
11	Asthma	2.6 (1.9–3.3)	0.0380 (0.0250–0.0560)	431.0 (282.2–651.6)	30.8 (22.5–40.5)	11.3 (3.8–20.3)
12	Chronic obstructive pulmonary disease	2.5 (2.0–3.1)	0.0373 (0.0318–0.0431)	225.6 (192.8–259.3)	67.5 (55.5–82.9)	5.6 (-1.8–14.5)
13	Oral disorders	2.4 (1.7–3.3)	0.0360 (0.0214–0.0549)	269.0 (156.4–411.2)	30.7 (19.5–42.0)	-6.7 (-14.8–2.3)
14	Alzheimer's disease and other dementias	1.8 (1.5–2.3)	0.0273 (0.0187–0.0363)	147.2 (100.2–196.2)	56.0 (48.9–63.8)	-8.3 (-12.2–-4.1)
15	Neck pain	1.8 (1.3–2.2)	0.0263 (0.0176–0.0374)	238.0 (158.0–344.1)	22.9 (16.3–30.5)	-1.0 (-3.6–1.6)
16	Gynecological diseases	1.6 (1.3–1.9)	0.0237 (0.0165–0.0329)	245.6 (167.1–348.1)	-8.3 (-15.6–-1.3)	-15.3 (-22.4–-8.5)
17	Schizophrenia	1.6 (1.2–2.1)	0.0236 (0.0173–0.0299)	223.0 (163.6–284.8)	10.6 (2.2–20.6)	-5.4 (-12.6–2.5)
18	Stroke	1.5 (1.3–1.8)	0.0225 (0.0162–0.0288)	148.4 (106.8–191.3)	34.5 (23.1–45.5)	-10.7 (-17.5–-4.2)
19	Chronic kidney disease	1.2 (1.0–1.4)	0.0176 (0.0124–0.0228)	118.7 (83.9–152.9)	77.3 (63.6–93.4)	18.6 (10.0–28.6)
20	Blindness and vision loss	1.2 (0.8–1.7)	0.0174 (0.0111–0.0269)	130.7 (81.8–203.5)	47.7 (38.2–58.9)	2.9 (-3.2–10.5)
21	Alcohol use disorders	1.1 (0.9–1.4)	0.0171 (0.0116–0.0238)	172.6 (116.8–243.2)	-14.0 (-21.8–-3.9)	-23.8 (-30.3–-15.4)
22	Dermatitis	1.1 (0.8–1.6)	0.0169 (0.00995–0.0265)	196.3 (113.3–313.7)	5.8 (1.0–10.1)	-7.7 (-12.7–-3.9)
23	Road injuries	1.1 (0.9–1.2)	0.0159 (0.0113–0.0214)	127.5 (91.0–171.6)	-25.3 (-27.8–-22.6)	-45.3 (-46.9–-43.4)
24	Neonatal disorders	1.1 (0.9–1.3)	0.0159 (0.0111–0.0211)	193.2 (134.1–257.0)	-8.7 (-26.4–12.0)	-18.3 (-34.1–-0.2)
25	Other mental disorders	1.0 (0.7–1.3)	0.0147 (0.00952–0.0215)	130.8 (84.5–191.5)	22.9 (16.9–28.8)	-0.3 (-5.0–4.7)

Table S3: Cause-specific YLDs by US state in 2021 and percentage change between 1990 and 2021, New Mexico						
Rank	Cause Name	2021 Percentage of all cause YLDs	2021 YLDs (millions)	2021 Age Standardised Rate (per 100 000)	Percentage change YLD count 1990 to 2021	Percentage change age-standardised YLD rate 1990 to 2021
	All causes	100.0 (100.0–100.0)	0.361 (0.275–0.461)	13985.0 (10556.1–17961.4)	87.1 (81.0–94.7)	17.3 (13.0–22.3)
1	Low back pain	9.4 (8.4–10.6)	0.0341 (0.0251–0.0442)	1282.0 (931.8–1675.2)	55.1 (43.9–67.3)	-4.7 (-11.4–2.7)
2	Depressive disorders	7.4 (5.6–9.5)	0.0267 (0.0178–0.0383)	1258.2 (825.2–1778.4)	114.8 (73.2–160.4)	61.9 (30.1–97.2)
3	Drug use disorders	7.1 (5.4–8.9)	0.0255 (0.0177–0.0332)	1281.8 (886.3–1679.7)	375.8 (326.8–442.2)	285.3 (246.4–340.7)
4	Diabetes mellitus	6.7 (5.8–7.6)	0.0243 (0.0171–0.0329)	699.5 (491.0–939.1)	401.4 (361.4–449.8)	139.6 (121.5–160.4)
5	Other musculoskeletal disorders	6.7 (5.3–8.3)	0.0242 (0.0171–0.0326)	962.3 (680.5–1294.8)	149.1 (119.6–182.4)	59.5 (41.8–81.6)
6	Anxiety disorders	4.9 (3.5–6.6)	0.0178 (0.0114–0.0252)	811.0 (517.3–1151.2)	81.7 (40.1–131.9)	33.0 (2.6–67.2)
7	Age-related and other hearing loss	4.1 (3.3–5.1)	0.0150 (0.0105–0.0208)	419.2 (294.2–581.0)	98.3 (89.9–107.0)	-6.2 (-9.4—2.9)
8	Headache disorders	4.1 (0.9–8.0)	0.0148 (0.00327–0.0309)	678.2 (136.0–1431.2)	31.3 (24.2–48.4)	-4.0 (-8.7–2.2)
9	Falls	4.0 (3.3–4.7)	0.0144 (0.0101–0.0199)	407.8 (284.1–562.4)	111.1 (96.7–125.2)	-0.8 (-6.1–4.6)
10	Osteoarthritis	3.1 (1.8–5.7)	0.0113 (0.00553–0.0227)	310.2 (150.8–621.2)	131.6 (123.9–139.4)	6.5 (3.3–9.5)
11	Asthma	2.5 (1.9–3.2)	0.00895 (0.00583–0.0130)	433.2 (279.3–638.8)	38.6 (26.5–51.7)	2.2 (-5.6–10.8)
12	Chronic obstructive pulmonary disease	2.5 (2.0–3.1)	0.00878 (0.00754–0.0101)	224.3 (193.3–256.1)	137.9 (121.9–157.1)	4.0 (-2.9–12.5)
13	Oral disorders	2.3 (1.6–3.2)	0.00830 (0.00495–0.0124)	271.9 (158.2–411.8)	73.8 (56.7–88.4)	-6.1 (-14.1–1.9)
14	Alcohol use disorders	2.0 (1.7–2.5)	0.00731 (0.00506–0.0101)	334.3 (228.7–464.6)	12.5 (2.1–23.5)	-16.5 (-23.8—8.8)
15	Alzheimer's disease and other dementias	1.8 (1.4–2.3)	0.00658 (0.00453–0.00877)	148.9 (102.4–197.6)	142.5 (130.2–154.0)	-6.3 (-10.3—2.0)
16	Neck pain	1.6 (1.2–2.0)	0.00579 (0.00393–0.00812)	235.9 (157.2–341.4)	49.6 (40.3–61.2)	-1.4 (-4.2–1.7)
17	Road injuries	1.5 (1.3–1.8)	0.00556 (0.00398–0.00748)	200.0 (143.5–269.1)	0.1 (-3.4–3.6)	-41.0 (-42.7—39.3)
18	Stroke	1.5 (1.2–1.8)	0.00544 (0.00396–0.00697)	153.4 (112.3–196.7)	106.3 (90.9–125.2)	-2.0 (-9.2–5.7)
19	Gynecological diseases	1.4 (1.2–1.7)	0.00513 (0.00359–0.00706)	239.7 (165.8–335.6)	7.7 (-0.2–16.0)	-17.2 (-23.5—10.2)
20	Schizophrenia	1.4 (1.0–1.8)	0.00502 (0.00371–0.00638)	217.3 (161.1–274.3)	31.3 (22.1–43.4)	-6.3 (-12.5–1.5)
21	Chronic kidney disease	1.2 (1.0–1.4)	0.00415 (0.00294–0.00535)	121.9 (86.9–157.6)	154.5 (128.8–181.6)	24.8 (14.3–35.2)
22	Blindness and vision loss	1.1 (0.8–1.6)	0.00398 (0.00257–0.00600)	131.9 (83.4–205.2)	88.6 (77.1–99.8)	1.6 (-4.5–8.5)
23	Autism spectrum disorders	1.1 (0.7–1.6)	0.00391 (0.00273–0.00549)	198.1 (137.6–277.2)	31.9 (21.1–43.5)	2.4 (-6.4–10.7)
24	Neonatal disorders	0.9 (0.8–1.1)	0.00336 (0.00236–0.00436)	183.6 (128.4–238.5)	17.1 (-8.4–43.9)	-3.2 (-24.1–19.0)
25	Other mental disorders	0.9 (0.7–1.2)	0.00326 (0.00211–0.00474)	130.5 (83.5–189.2)	51.3 (43.8–59.8)	-0.6 (-5.6–4.7)

Table S3: Cause-specific YLDs by US state in 2021 and percentage change between 1990 and 2021, New York

Rank	Cause Name	2021 Percentage of all cause YLDs	2021 YLDs (millions)	2021 Age Standardised Rate (per 100 000)	Percentage change YLD count 1990 to 2021	Percentage change age-standardised YLD rate 1990 to 2021
	All causes	100.0 (100.0–100.0)	3.36 (2.54–4.28)	13422.3 (10101.7–17216.5)	37.4 (33.5–42.0)	12.9 (9.2–17.0)
1	Low back pain	8.8 (7.7–9.9)	0.295 (0.216–0.384)	1136.5 (828.1–1491.6)	18.2 (9.7–27.1)	-5.1 (-11.2–1.8)
2	Other musculoskeletal disorders	7.8 (6.2–9.7)	0.263 (0.188–0.349)	1051.9 (750.8–1402.9)	79.1 (60.2–104.0)	48.8 (33.9–69.1)
3	Diabetes mellitus	7.0 (6.0–7.9)	0.234 (0.164–0.317)	713.1 (498.2–972.5)	300.3 (267.2–337.2)	173.5 (151.4–198.0)
4	Depressive disorders	6.5 (5.0–8.6)	0.218 (0.147–0.313)	1055.8 (710.1–1528.0)	54.5 (27.2–88.8)	47.8 (21.6–81.4)
5	Anxiety disorders	5.1 (3.6–7.0)	0.172 (0.108–0.251)	803.4 (502.2–1164.6)	41.3 (8.7–84.6)	30.9 (1.3–68.7)
6	Drug use disorders	5.1 (3.9–6.4)	0.170 (0.121–0.221)	854.3 (600.6–1110.2)	172.5 (141.8–208.5)	172.9 (140.9–210.1)
7	Headache disorders	4.4 (1.0–8.5)	0.148 (0.0322–0.309)	693.7 (135.7–1467.7)	5.3 (-0.1–17.0)	-3.3 (-8.5–3.1)
8	Age-related and other hearing loss	4.1 (3.3–5.1)	0.138 (0.0962–0.189)	414.7 (288.8–569.7)	34.4 (29.2–40.5)	-5.6 (-9.1—1.9)
9	Osteoarthritis	3.3 (1.9–6.1)	0.111 (0.0539–0.222)	326.7 (157.5–652.7)	54.9 (50.4–59.7)	5.0 (1.7–8.4)
10	Falls	2.8 (2.4–3.4)	0.0953 (0.0665–0.132)	291.8 (201.8–401.0)	40.8 (31.9–50.2)	-5.3 (-10.9–0.1)
11	Asthma	2.7 (2.1–3.5)	0.0922 (0.0607–0.135)	475.8 (313.1–715.8)	16.3 (8.7–24.7)	5.8 (-0.8–14.0)
12	Chronic obstructive pulmonary disease	2.7 (2.1–3.4)	0.0889 (0.0767–0.101)	252.1 (217.9–285.3)	65.4 (53.8–80.9)	11.9 (3.8–22.3)
13	Oral disorders	2.5 (1.7–3.5)	0.0829 (0.0483–0.125)	289.4 (167.1–439.6)	28.0 (17.4–39.2)	-2.4 (-11.1–7.9)
14	Alzheimer's disease and other dementias	1.9 (1.5–2.4)	0.0633 (0.0435–0.0840)	152.6 (104.3–203.3)	50.8 (43.0–58.6)	-5.7 (-10.6–-1.4)
15	Neck pain	1.7 (1.3–2.1)	0.0568 (0.0380–0.0797)	237.3 (157.0–341.9)	15.7 (9.6–22.2)	-1.2 (-4.1–1.5)
16	Gynecological diseases	1.7 (1.4–2.0)	0.0568 (0.0392–0.0784)	265.1 (183.0–374.6)	-10.4 (-16.7–-3.3)	-14.2 (-20.6–-6.7)
17	Schizophrenia	1.5 (1.1–2.0)	0.0513 (0.0384–0.0650)	221.6 (165.7–282.6)	5.5 (-2.2–15.1)	-5.6 (-12.5–2.7)
18	Stroke	1.4 (1.1–1.7)	0.0471 (0.0343–0.0602)	143.6 (104.7–182.3)	28.8 (17.7–39.7)	-8.8 (-15.9–-1.1)
19	Alcohol use disorders	1.4 (1.1–1.7)	0.0465 (0.0327–0.0645)	213.1 (149.1–296.9)	-19.4 (-27.3–-10.5)	-25.1 (-31.9—18.2)
20	Autism spectrum disorders	1.2 (0.8–1.7)	0.0384 (0.0264–0.0535)	202.0 (138.9–282.4)	9.8 (-0.4–20.0)	2.8 (-6.4–12.6)
21	Chronic kidney disease	1.1 (0.9–1.4)	0.0378 (0.0269–0.0489)	116.1 (83.7–148.5)	67.2 (53.0–83.3)	17.8 (8.3–27.7)
22	Blindness and vision loss	1.1 (0.8–1.6)	0.0375 (0.0238–0.0570)	130.1 (80.5–201.3)	36.1 (27.9–45.6)	1.6 (-4.8–8.4)
23	Neonatal disorders	1.1 (0.9–1.3)	0.0354 (0.0251–0.0463)	197.7 (139.8–259.3)	-8.8 (-26.3–10.6)	-12.6 (-29.1–6.7)
24	Dermatitis	1.0 (0.7–1.5)	0.0348 (0.0208–0.0544)	185.4 (108.5–293.1)	-2.4 (-6.4–1.8)	-8.7 (-13.0–-4.4)
25	Other mental disorders	1.0 (0.7–1.3)	0.0320 (0.0206–0.0469)	130.1 (83.7–192.3)	16.8 (11.2–23.0)	-0.3 (-5.3–4.6)

Table S3: Cause-specific YLDs by US state in 2021 and percentage change between 1990 and 2021, North Carolina						
**Rank**	**Cause Name**	**2021 Percentage of all cause YLDs**	**2021 YLDs (millions)**	**2021 Age Standardised Rate (per 100 000)**	**Percentage change YLD count 1990 to 2021**	**Percentage change age-standardised YLD rate 1990 to 2021**
	All causes	100.0 (100.0–100.0)	1.69 (1.29–2.18)	13152.3 (9961.6–17064.6)	99.6 (92.6–107.4)	16.3 (11.8–20.9)
1	Low back pain	8.8 (7.8–10.0)	0.149 (0.108–0.193)	1118.1 (807.2–1454.2)	54.8 (42.5–66.9)	-11.6 (-18.2–-5.2)
2	Diabetes mellitus	7.2 (6.2–8.1)	0.122 (0.0861–0.165)	726.9 (512.0–979.4)	417.0 (378.3–458.9)	146.2 (128.1–163.3)
3	Drug use disorders	6.9 (5.2–8.7)	0.116 (0.0802–0.149)	1141.3 (788.6–1480.7)	499.2 (422.6–586.2)	341.7 (284.6–405.8)
4	Other musculoskeletal disorders	6.5 (5.1–8.0)	0.109 (0.0779–0.147)	857.6 (609.9–1158.6)	141.5 (114.5–174.5)	43.6 (27.6–62.9)
5	Depressive disorders	5.5 (4.2–7.3)	0.0939 (0.0619–0.137)	876.7 (569.7–1286.2)	167.5 (117.2–228.6)	84.6 (49.4–126.5)
6	Anxiety disorders	5.3 (3.8–7.2)	0.0893 (0.0572–0.128)	811.1 (521.5–1179.7)	87.2 (44.3–138.9)	25.2 (-3.2–59.2)
7	Headache disorders	4.4 (1.0–8.7)	0.0759 (0.0167–0.160)	690.8 (137.4–1464.3)	46.4 (38.6–61.3)	-2.8 (-7.3–2.3)
8	Age-related and other hearing loss	4.2 (3.4–5.1)	0.0708 (0.0495–0.0982)	418.1 (293.7–580.2)	96.1 (88.3–104.1)	-5.7 (-8.8–-2.5)
9	Falls	3.2 (2.7–3.8)	0.0546 (0.0382–0.0765)	321.9 (224.0–447.0)	109.3 (93.7–122.9)	-3.2 (-9.4–2.4)
10	Osteoarthritis	2.9 (1.7–5.3)	0.0487 (0.0238–0.0988)	277.5 (134.5–563.5)	129.2 (121.5–137.8)	6.3 (2.7–10.1)
11	Oral disorders	2.7 (1.9–3.7)	0.0460 (0.0281–0.0682)	308.5 (182.3–469.6)	104.2 (95.4–115.8)	6.8 (2.4–11.7)
12	Chronic obstructive pulmonary disease	2.4 (1.9–3.0)	0.0406 (0.0349–0.0467)	221.4 (190.7–253.8)	132.0 (115.6–150.8)	4.9 (-2.9–12.9)
13	Asthma	2.3 (1.7–2.9)	0.0385 (0.0253–0.0555)	377.2 (247.4–559.6)	62.2 (51.5–74.7)	3.7 (-3.9–12.7)
14	Alzheimer's disease and other dementias	2.2 (1.7–2.7)	0.0367 (0.0254–0.0487)	184.0 (127.3–243.6)	128.5 (116.3–140.5)	-3.7 (-8.6–1.3)
15	Stroke	1.9 (1.6–2.2)	0.0323 (0.0235–0.0414)	193.9 (140.5–245.1)	102.5 (86.9–117.4)	-1.7 (-8.6–5.5)
16	Gynecological diseases	1.5 (1.2–1.9)	0.0259 (0.0179–0.0363)	240.3 (166.4–343.2)	22.2 (12.7–32.3)	-14.4 (-21.2–-6.9)
17	Schizophrenia	1.5 (1.2–2.0)	0.0258 (0.0190–0.0328)	220.9 (163.7–282.9)	43.9 (33.2–55.4)	-6.0 (-12.6–1.0)
18	Neck pain	1.5 (1.1–2.0)	0.0256 (0.0166–0.0373)	209.2 (135.1–316.5)	61.4 (50.9–72.6)	-1.0 (-3.9–2.1)
19	Road injuries	1.5 (1.3–1.7)	0.0250 (0.0179–0.0338)	182.0 (130.3–246.7)	11.6 (8.0–15.4)	-38.2 (-39.8–-36.5)
20	Alcohol use disorders	1.3 (1.1–1.6)	0.0220 (0.0151–0.0304)	197.9 (136.0–277.1)	7.8 (-1.8–18.4)	-27.0 (-33.0–-20.2)
21	Chronic kidney disease	1.2 (1.0–1.5)	0.0204 (0.0145–0.0258)	126.6 (90.7–160.5)	147.8 (126.8–170.3)	22.8 (13.5–32.9)
22	Neonatal disorders	1.2 (1.0–1.5)	0.0199 (0.0141–0.0262)	216.6 (152.2–285.6)	38.3 (14.9–72.5)	-3.3 (-19.8–20.7)
23	Autism spectrum disorders	1.2 (0.8–1.7)	0.0197 (0.0136–0.0273)	199.2 (137.1–277.7)	51.9 (40.8–65.0)	1.9 (-5.4–10.9)
24	Blindness and vision loss	1.1 (0.8–1.6)	0.0193 (0.0124–0.0295)	131.3 (82.1–202.3)	95.3 (81.9–109.3)	1.5 (-5.0–8.5)
25	Other mental disorders	1.0 (0.7–1.3)	0.0162 (0.0106–0.0240)	130.1 (84.0–194.0)	61.0 (54.1–68.9)	-1.1 (-5.7–3.8)

Table S3: Cause-specific YLDs by US state in 2021 and percentage change between 1990 and 2021, North Dakota						
Rank	Cause Name	2021 Percentage of all cause YLDs	2021 YLDs (millions)	2021 Age Standardised Rate (per 100 000)	Percentage change YLD count 1990 to 2021	Percentage change age-standardised YLD rate 1990 to 2021
	All causes	100.0 (100.0–100.0)	0.122 (0.0924–0.156)	12852.1 (9661.0–16571.0)	48.1 (43.7–53.0)	13.4 (9.5–17.7)
1	Other musculoskeletal disorders	10.4 (8.3–12.7)	0.0127 (0.00908–0.0169)	1442.1 (1034.9–1927.2)	104.4 (88.3–124.2)	63.2 (50.3–79.3)
2	Low back pain	9.2 (8.1–10.3)	0.0112 (0.00819–0.0147)	1173.3 (856.2–1547.1)	19.0 (11.5–26.4)	-10.4 (-15.4–-4.7)
3	Diabetes mellitus	6.4 (5.6–7.3)	0.00788 (0.00557–0.0108)	633.0 (446.5–864.9)	262.7 (233.1–297.8)	133.8 (116.0–154.5)
4	Depressive disorders	5.3 (4.0–6.9)	0.00647 (0.00427–0.00936)	825.7 (538.3–1206.5)	61.4 (31.8–96.1)	39.7 (13.9–69.0)
5	Anxiety disorders	4.9 (3.4–6.8)	0.00598 (0.00384–0.00876)	735.7 (471.7–1083.7)	44.9 (12.9–83.6)	21.0 (-6.7–52.6)
6	Headache disorders	4.4 (1.0–8.6)	0.00543 (0.00121–0.0114)	677.9 (137.0–1436.2)	15.8 (10.0–27.4)	-3.6 (-8.1–1.6)
7	Age-related and other hearing loss	4.3 (3.5–5.3)	0.00531 (0.00372–0.00726)	420.9 (294.5–578.4)	37.2 (31.4–43.3)	-5.5 (-9.2–-1.8)
8	Falls	3.6 (3.0–4.3)	0.00438 (0.00308–0.00607)	355.3 (246.7–489.8)	78.1 (67.2–88.9)	16.0 (9.0–22.9)
9	Drug use disorders	3.6 (2.7–4.5)	0.00434 (0.00305–0.00568)	585.1 (410.0–767.5)	231.8 (189.2–280.8)	199.5 (161.3–246.3)
10	Osteoarthritis	3.1 (1.8–6.0)	0.00385 (0.00187–0.00781)	302.3 (145.0–611.0)	66.6 (61.8–72.1)	8.3 (5.0–11.9)
11	Asthma	2.5 (2.0–3.2)	0.00309 (0.00200–0.00457)	386.2 (248.9–582.3)	31.3 (21.0–41.7)	5.9 (-2.0–15.1)
12	Oral disorders	2.5 (1.8–3.4)	0.00303 (0.00179–0.00451)	279.1 (162.0–422.1)	32.8 (22.3–43.2)	-4.8 (-13.0–3.9)
13	Chronic obstructive pulmonary disease	2.4 (1.9–3.1)	0.00294 (0.00249–0.00340)	216.3 (183.3–249.3)	61.8 (50.5–75.5)	6.6 (-0.5–15.2)
14	Alzheimer's disease and other dementias	2.0 (1.6–2.5)	0.00239 (0.00165–0.00316)	149.6 (104.2–197.9)	37.1 (29.7–45.7)	-7.2 (-11.7–-2.1)
15	Neck pain	1.7 (1.3–2.2)	0.00210 (0.00142–0.00296)	236.3 (157.6–340.6)	26.2 (19.3–32.9)	-1.3 (-4.3–1.4)
16	Road injuries	1.7 (1.5–2.0)	0.00210 (0.00150–0.00282)	211.7 (152.0–286.5)	7.2 (4.2–10.4)	-21.7 (-23.7–-19.5)
17	Stroke	1.6 (1.3–1.8)	0.00190 (0.00137–0.00247)	149.8 (107.1–192.5)	44.7 (34.2–56.1)	0.2 (-6.5–7.7)
18	Schizophrenia	1.6 (1.2–2.1)	0.00190 (0.00140–0.00242)	222.6 (163.5–283.3)	16.6 (7.8–26.1)	-5.9 (-13.2–1.8)
19	Alcohol use disorders	1.5 (1.3–1.9)	0.00189 (0.00132–0.00264)	232.9 (160.7–327.0)	0.9 (-8.2–11.0)	-15.5 (-22.8–-7.0)
20	Gynecological diseases	1.4 (1.2–1.7)	0.00172 (0.00118–0.00239)	219.2 (149.8–311.2)	-6.6 (-14.0–1.4)	-18.5 (-25.6–-11.2)
21	Autism spectrum disorders	1.3 (0.8–1.8)	0.00152 (0.00105–0.00211)	203.2 (140.7–282.0)	21.5 (11.4–32.1)	2.2 (-6.2–11.1)
22	Blindness and vision loss	1.2 (0.8–1.7)	0.00142 (0.000911–0.00213)	131.7 (82.3–203.3)	41.1 (32.7–50.8)	2.2 (-4.0–9.1)
23	Chronic kidney disease	1.1 (0.9–1.4)	0.00136 (0.000966–0.00176)	110.6 (79.1–143.1)	84.0 (69.5–103.3)	30.0 (19.8–42.0)
24	Other mental disorders	1.0 (0.7–1.4)	0.00121 (0.000781–0.00178)	132.7 (85.1–195.6)	28.3 (22.4–34.4)	-0.1 (-5.0–4.9)
25	Neonatal disorders	1.0 (0.8–1.2)	0.00116 (0.000824–0.00153)	164.4 (116.8–217.8)	29.2 (2.3–59.7)	11.8 (-11.4–38.3)

Table S3: Cause-specific YLDs by US state in 2021 and percentage change between 1990 and 2021, Ohio

Rank	Cause Name	2021 Percentage of all cause YLDs	2021 YLDs (millions)	2021 Age Standardised Rate (per 100 000)	Percentage change YLD count 1990 to 2021	Percentage change age-standardised YLD rate 1990 to 2021
	All causes	100.0 (100.0–100.0)	2.03 (1.56–2.59)	13979.8 (10602.4–17949.1)	41.6 (36.5–47.5)	19.8 (14.9–25.9)
1	Low back pain	9.0 (8.0–10.1)	0.182 (0.132–0.237)	1215.7 (878.7–1589.4)	12.2 (4.5–21.2)	-7.1 (-13.0–-0.3)
2	Drug use disorders	7.6 (5.8–9.7)	0.154 (0.108–0.199)	1400.2 (974.7–1817.3)	494.5 (410.7–591.0)	527.1 (439.5–634.4)
3	Diabetes mellitus	7.3 (6.3–8.2)	0.148 (0.104–0.197)	743.9 (524.1–991.7)	259.1 (229.5–290.8)	143.4 (124.4–162.9)
4	Other musculoskeletal disorders	6.9 (5.5–8.6)	0.140 (0.0984–0.187)	981.5 (696.8–1318.8)	67.9 (50.0–88.8)	44.9 (28.1–63.2)
5	Depressive disorders	6.0 (4.5–7.6)	0.122 (0.0803–0.174)	1036.5 (681.7–1465.6)	57.9 (30.1–89.9)	57.1 (29.4–87.9)
6	Anxiety disorders	4.9 (3.5–6.7)	0.0989 (0.0615–0.142)	807.1 (499.8–1175.1)	36.9 (6.3–75.3)	31.9 (2.3–69.0)
7	Headache disorders	4.1 (1.0–8.0)	0.0832 (0.0185–0.175)	682.5 (136.3–1452.3)	0.3 (-5.4–11.9)	-4.1 (-9.0–1.5)
8	Age-related and other hearing loss	4.1 (3.2–5.0)	0.0825 (0.0580–0.114)	416.1 (293.4–574.2)	34.6 (29.4–39.7)	-5.8 (-8.6–-2.3)
9	Osteoarthritis	3.5 (2.1–6.5)	0.0713 (0.0351–0.144)	348.5 (169.4–704.3)	56.5 (51.8–61.9)	5.2 (2.1–8.7)
10	Falls	3.2 (2.6–3.9)	0.0653 (0.0460–0.0910)	329.6 (230.6–456.5)	50.8 (39.0–61.6)	0.1 (-6.8–6.3)
11	Chronic obstructive pulmonary disease	2.7 (2.1–3.4)	0.0535 (0.0465–0.0611)	251.5 (218.3–286.1)	64.6 (52.3–81.0)	9.8 (1.5–20.2)
12	Oral disorders	2.3 (1.6–3.2)	0.0466 (0.0278–0.0707)	271.7 (159.3–416.3)	23.3 (12.8–33.6)	-6.1 (-14.4–2.0)
13	Asthma	2.3 (1.7–2.9)	0.0460 (0.0299–0.0674)	400.9 (261.3–599.4)	14.4 (5.6–24.2)	6.4 (-1.6–16.1)
14	Stroke	1.8 (1.5–2.1)	0.0356 (0.0254–0.0457)	181.1 (129.5–231.6)	43.0 (31.5–55.1)	0.7 (-6.8–8.3)
15	Alzheimer's disease and other dementias	1.7 (1.4–2.2)	0.0351 (0.0244–0.0461)	146.7 (101.8–193.5)	45.3 (37.8–53.3)	-8.0 (-12.6–-3.0)
16	Neck pain	1.6 (1.2–2.0)	0.0326 (0.0219–0.0459)	236.6 (156.5–341.8)	12.0 (4.5–19.1)	-1.5 (-4.2–1.6)
17	Schizophrenia	1.4 (1.0–1.9)	0.0284 (0.0209–0.0363)	218.7 (160.8–280.5)	-0.3 (-6.8–8.4)	-6.5 (-12.6–0.9)
18	Gynecological diseases	1.4 (1.1–1.7)	0.0279 (0.0193–0.0390)	237.1 (163.8–337.3)	-19.4 (-25.6–-13.4)	-17.4 (-24.2–-10.1)
19	Alcohol use disorders	1.3 (1.0–1.6)	0.0259 (0.0180–0.0356)	210.3 (144.4–291.8)	-17.6 (-25.0–-9.3)	-20.6 (-27.5–-12.5)
20	Road injuries	1.1 (1.0–1.3)	0.0231 (0.0165–0.0312)	146.9 (105.2–197.7)	-25.7 (-28.3–-22.8)	-41.2 (-43.0–-39.4)
21	Chronic kidney disease	1.1 (0.9–1.4)	0.0228 (0.0162–0.0292)	121.1 (87.4–154.6)	75.2 (58.8–94.4)	26.6 (16.0–38.2)
22	Autism spectrum disorders	1.1 (0.7–1.6)	0.0221 (0.0151–0.0309)	199.7 (137.2–279.1)	4.2 (-4.3–15.2)	1.4 (-6.9–11.9)
23	Blindness and vision loss	1.1 (0.8–1.6)	0.0221 (0.0141–0.0336)	130.5 (81.6–202.2)	34.5 (25.8–43.5)	1.7 (-4.8–8.8)
24	COVID-19	1.1 (0.4–2.4)	0.0219 (0.00810–0.0476)	168.5 (59.8–379.7)	0.0 (0.0–0.0)	0.0 (0.0–0.0)
25	Neonatal disorders	1.1 (0.9–1.3)	0.0218 (0.0153–0.0292)	215.2 (151.2–289.6)	7.2 (-13.7–32.7)	9.8 (-11.7–35.6)

Table S3: Cause-specific YLDs by US state in 2021 and percentage change between 1990 and 2021, Oklahoma						
Rank	Cause Name	2021 Percentage of all cause YLDs	2021 YLDs (millions)	2021 Age Standardised Rate (per 100 000)	Percentage change YLD count 1990 to 2021	Percentage change age-standardised YLD rate 1990 to 2021
	All causes	100.0 (100.0–100.0)	0.663 (0.500–0.841)	14147.8 (10559.1–17975.3)	58.9 (53.1–65.5)	20.8 (16.0–26.6)
1	Low back pain	9.6 (8.5–10.8)	0.0637 (0.0462–0.0822)	1325.4 (960.8–1723.2)	29.7 (21.5–39.1)	-3.7 (-9.7–3.3)
2	Drug use disorders	8.1 (6.1–10.1)	0.0532 (0.0376–0.0685)	1382.6 (971.5–1783.3)	540.0 (467.3–619.7)	450.3 (386.0–519.4)
3	Depressive disorders	7.1 (5.4–9.3)	0.0470 (0.0306–0.0664)	1169.2 (762.6–1653.1)	91.6 (56.5–133.8)	60.2 (31.1–94.3)
4	Other musculoskeletal disorders	6.8 (5.4–8.4)	0.0451 (0.0318–0.0603)	1000.9 (711.7–1351.5)	105.2 (82.8–128.8)	59.4 (41.2–78.6)
5	Diabetes mellitus	6.4 (5.5–7.2)	0.0423 (0.0299–0.0575)	704.4 (500.3–952.1)	287.0 (259.9–319.0)	153.0 (136.4–172.1)
6	Anxiety disorders	5.0 (3.5–6.8)	0.0334 (0.0213–0.0495)	810.2 (515.5–1200.7)	61.7 (24.7–102.2)	32.7 (2.6–66.5)
7	Headache disorders	4.2 (1.0–8.2)	0.0278 (0.00610–0.0588)	677.6 (136.5–1438.8)	18.1 (12.2–28.2)	-4.2 (-8.7–1.6)
8	Age-related and other hearing loss	3.9 (3.1–4.8)	0.0256 (0.0179–0.0354)	419.0 (292.8–583.9)	39.0 (34.3–44.3)	-5.8 (-8.8–-2.7)
9	Falls	3.2 (2.6–3.8)	0.0211 (0.0149–0.0296)	346.3 (243.4–480.9)	70.7 (58.2–83.0)	8.2 (0.2–15.5)
10	Osteoarthritis	3.0 (1.7–5.7)	0.0201 (0.00981–0.0405)	321.7 (156.0–646.9)	63.0 (58.7–67.7)	5.7 (2.9–9.0)
11	Chronic obstructive pulmonary disease	2.5 (2.0–3.1)	0.0161 (0.0140–0.0184)	248.6 (215.5–283.5)	64.8 (53.8–79.4)	7.9 (0.6–17.5)
12	Oral disorders	2.4 (1.7–3.2)	0.0159 (0.00973–0.0233)	294.3 (174.3–442.4)	39.9 (35.1–45.7)	-0.8 (-4.6–3.6)
13	Asthma	2.4 (1.8–3.0)	0.0158 (0.0104–0.0232)	410.2 (270.3–619.6)	27.8 (19.3–38.2)	3.8 (-2.9–12.5)
14	Stroke	1.8 (1.5–2.1)	0.0116 (0.00838–0.0149)	191.2 (137.4–244.4)	50.1 (39.3–64.8)	2.8 (-4.1–12.1)
15	Neck pain	1.6 (1.2–2.0)	0.0106 (0.00718–0.0152)	235.7 (157.0–340.9)	27.8 (21.6–33.7)	-1.7 (-4.6–1.4)
16	Alzheimer's disease and other dementias	1.6 (1.3–2.0)	0.0105 (0.00716–0.0139)	145.9 (99.7–192.2)	37.9 (30.9–44.4)	-8.1 (-12.7–-3.9)
17	Road injuries	1.5 (1.3–1.7)	0.0100 (0.00712–0.0135)	199.6 (143.1–269.1)	-7.4 (-9.9–-4.4)	-33.6 (-35.2–-31.8)
18	Gynecological diseases	1.5 (1.2–1.8)	0.00973 (0.00678–0.0135)	239.9 (166.7–337.7)	-0.5 (-8.3–8.5)	-15.8 (-22.8–-7.4)
19	Schizophrenia	1.4 (1.0–1.9)	0.00941 (0.00688–0.0119)	217.7 (159.0–275.7)	17.0 (8.3–26.3)	-6.5 (-13.7–1.2)
20	Alcohol use disorders	1.4 (1.1–1.7)	0.00919 (0.00631–0.0126)	221.7 (153.7–307.3)	-1.8 (-10.4–8.4)	-20.3 (-27.0–-12.7)
21	Autism spectrum disorders	1.1 (0.7–1.7)	0.00741 (0.00512–0.0103)	196.6 (135.2–275.1)	22.3 (12.4–33.4)	1.0 (-7.4–10.1)
22	Chronic kidney disease	1.1 (0.9–1.3)	0.00722 (0.00516–0.00922)	123.8 (88.3–158.1)	90.6 (73.4–109.9)	32.5 (20.5–45.3)
23	Neonatal disorders	1.1 (0.9–1.3)	0.00711 (0.00491–0.00936)	202.4 (139.0–266.5)	29.4 (4.6–59.1)	10.8 (-10.4–36.7)
24	Blindness and vision loss	1.1 (0.8–1.5)	0.00699 (0.00451–0.0106)	131.6 (82.8–204.4)	42.0 (33.1–51.1)	1.3 (-4.8–8.2)
25	COVID-19	1.0 (0.4–2.5)	0.00675 (0.00250–0.0160)	158.2 (56.9–386.2)	0.0 (0.0–0.0)	0.0 (0.0–0.0)

Table S3: Cause-specific YLDs by US state in 2021 and percentage change between 1990 and 2021, Oregon						
Rank	Cause Name	2021 Percentage of all cause YLDs	2021 YLDs (millions)	2021 Age Standardised Rate (per 100 000)	Percentage change YLD count 1990 to 2021	Percentage change age-standardised YLD rate 1990 to 2021
	All causes	100.0 (100.0–100.0)	0.692 (0.529–0.892)	12842.8 (9758.9–16542.8)	86.3 (80.4–93.6)	12.9 (8.7–18.1)
1	Low back pain	9.6 (8.5–10.8)	0.0664 (0.0487–0.0866)	1200.9 (880.2–1575.9)	59.1 (47.8–70.3)	-5.2 (-11.4–1.5)
2	Other musculoskeletal disorders	7.7 (6.1–9.4)	0.0531 (0.0374–0.0720)	1044.5 (742.6–1418.2)	161.1 (131.4–195.6)	64.3 (46.2–84.7)
3	Depressive disorders	6.8 (5.2–8.8)	0.0474 (0.0306–0.0667)	1100.5 (712.0–1553.8)	114.9 (78.8–159.1)	53.4 (27.2–84.8)
4	Diabetes mellitus	6.0 (5.2–6.7)	0.0413 (0.0289–0.0564)	565.6 (397.9–760.6)	368.1 (332.1–407.6)	129.5 (112.2–147.9)
5	Drug use disorders	5.2 (3.9–6.6)	0.0359 (0.0250–0.0462)	875.4 (608.3–1130.5)	388.4 (331.1–452.4)	256.4 (212.0–304.9)
6	Age-related and other hearing loss	4.5 (3.6–5.5)	0.0312 (0.0218–0.0431)	419.2 (291.1–581.0)	86.5 (79.2–94.2)	-5.7 (-8.9–2.3)
7	Headache disorders	4.4 (1.0–8.5)	0.0307 (0.00696–0.0646)	686.1 (139.2–1466.1)	41.6 (34.1–55.8)	-3.0 (-7.6–2.7)
8	Anxiety disorders	4.0 (2.8–5.6)	0.0276 (0.0180–0.0409)	615.9 (395.7–908.3)	77.3 (36.5–124.6)	23.1 (-4.7–54.9)
9	Falls	4.0 (3.3–4.7)	0.0275 (0.0193–0.0381)	372.1 (259.3–513.0)	112.9 (99.1–125.7)	1.9 (-4.0–7.5)
10	Osteoarthritis	3.3 (1.9–6.1)	0.0228 (0.0111–0.0460)	299.6 (144.5–601.4)	121.0 (114.4–128.0)	7.0 (3.7–10.4)
11	Asthma	2.5 (1.9–3.2)	0.0174 (0.0113–0.0253)	393.5 (256.0–586.5)	48.8 (36.1–60.4)	-2.4 (-10.1–5.2)
12	Oral disorders	2.4 (1.7–3.4)	0.0169 (0.0101–0.0258)	267.3 (155.0–409.4)	68.8 (54.7–83.4)	-7.0 (-15.5–1.9)
13	Chronic obstructive pulmonary disease	2.3 (1.8–2.9)	0.0157 (0.0133–0.0181)	187.4 (160.3–214.9)	108.2 (91.6–129.5)	-3.7 (-11.2–5.6)
14	Alzheimer's disease and other dementias	1.9 (1.5–2.4)	0.0134 (0.00929–0.0179)	147.9 (102.8–196.8)	98.0 (87.1–108.4)	-7.2 (-12.0—2.5)
15	Stroke	1.8 (1.5–2.1)	0.0123 (0.00893–0.0155)	164.7 (119.1–208.6)	86.7 (71.7–104.0)	-4.9 (-12.0–3.0)
16	Neck pain	1.7 (1.3–2.2)	0.0121 (0.00815–0.0170)	237.9 (159.9–345.6)	57.2 (47.8–66.8)	-0.8 (-3.7–2.0)
17	Alcohol use disorders	1.7 (1.4–2.1)	0.0118 (0.00816–0.0160)	256.9 (175.5–349.6)	18.4 (7.1–31.9)	-20.2 (-26.9—11.2)
18	Schizophrenia	1.6 (1.2–2.1)	0.0107 (0.00782–0.0136)	222.6 (164.0–281.1)	40.8 (29.6–52.4)	-5.7 (-13.1–1.9)
19	Gynecological diseases	1.5 (1.2–1.8)	0.0101 (0.00700–0.0142)	228.6 (159.6–326.3)	15.1 (6.5–24.9)	-16.1 (-23.0—8.4)
20	Road injuries	1.4 (1.2–1.6)	0.00948 (0.00671–0.0127)	161.1 (114.5–214.8)	-3.5 (-6.6—0.1)	-45.5 (-47.0—43.9)
21	Blindness and vision loss	1.2 (0.8–1.7)	0.00825 (0.00529–0.0125)	132.3 (82.2–203.3)	85.7 (74.9–98.4)	2.3 (-4.1–9.2)
22	Autism spectrum disorders	1.2 (0.8–1.8)	0.00819 (0.00565–0.0115)	205.1 (142.2–287.5)	44.1 (33.5–58.8)	2.1 (-5.8–12.5)
23	Chronic kidney disease	1.1 (0.9–1.3)	0.00761 (0.00540–0.00991)	106.3 (75.7–136.5)	143.5 (121.1–168.9)	26.6 (16.5–38.6)
24	Other mental disorders	1.0 (0.7–1.3)	0.00683 (0.00443–0.00999)	131.3 (84.3–194.6)	57.8 (50.9–66.0)	-0.6 (-5.0–4.5)
25	Dermatitis	1.0 (0.6–1.4)	0.00667 (0.00395–0.0105)	169.8 (97.8–271.2)	27.4 (21.5–33.6)	-8.7 (-13.7–3.4)

Table S3: Cause-specific YLDs by US state in 2021 and percentage change between 1990 and 2021, Pennsylvania						
**Rank**	**Cause Name**	**2021 Percentage of all cause YLDs**	**2021 YLDs (millions)**	**2021 Age Standardised Rate (per 100 000)**	**Percentage change YLD count 1990 to 2021**	**Percentage change age-standardised YLD rate 1990 to 2021**
	All causes	100.0 (100.0–100.0)	2.26 (1.72–2.87)	14004.7 (10475.5–17840.0)	36.7 (32.4–41.7)	18.3 (14.2–23.5)
1	Low back pain	9.4 (8.4–10.6)	0.212 (0.155–0.272)	1280.5 (934.8–1657.8)	15.5 (8.3–24.2)	-1.3 (-7.7–5.8)
2	Other musculoskeletal disorders	7.5 (5.9–9.3)	0.169 (0.119–0.225)	1082.9 (769.1–1447.4)	76.5 (57.0–99.1)	57.9 (40.9–77.9)
3	Drug use disorders	7.2 (5.5–9.0)	0.162 (0.114–0.209)	1336.7 (935.4–1727.4)	365.8 (312.4–426.3)	382.0 (325.9–449.9)
4	Diabetes mellitus	6.4 (5.5–7.3)	0.146 (0.103–0.194)	650.6 (463.6–873.0)	223.4 (198.2–253.6)	135.3 (117.1–155.5)
5	Depressive disorders	5.5 (4.2–7.2)	0.126 (0.0839–0.178)	968.5 (641.2–1382.2)	53.6 (24.3–88.6)	52.9 (23.4–85.8)
6	Anxiety disorders	4.8 (3.3–6.4)	0.108 (0.0684–0.154)	798.4 (500.6–1137.4)	36.4 (6.3–70.4)	30.7 (2.5–62.8)
7	Age-related and other hearing loss	4.2 (3.3–5.1)	0.0950 (0.0664–0.131)	416.7 (290.3–575.9)	26.8 (22.2–32.0)	-5.3 (-8.6–-1.7)
8	Headache disorders	4.0 (0.9–7.8)	0.0916 (0.0210–0.190)	678.5 (137.4–1429.4)	2.0 (-3.4–10.9)	-3.6 (-8.5–1.9)
9	Osteoarthritis	3.6 (2.1–6.7)	0.0822 (0.0398–0.165)	352.9 (169.5–707.6)	42.6 (38.6–47.2)	3.1 (0.3–6.3)
10	Falls	3.3 (2.7–3.9)	0.0739 (0.0518–0.103)	323.9 (226.1–449.0)	53.7 (42.9–63.8)	4.7 (-2.5–10.9)
11	Chronic obstructive pulmonary disease	2.7 (2.1–3.3)	0.0596 (0.0515–0.0681)	242.6 (211.3–276.0)	52.4 (41.5–64.9)	9.4 (1.7–18.8)
12	Oral disorders	2.4 (1.7–3.2)	0.0538 (0.0334–0.0790)	276.6 (164.5–412.5)	21.4 (17.6–25.6)	-2.7 (-6.4–1.2)
13	Asthma	2.3 (1.7–2.9)	0.0513 (0.0336–0.0746)	415.7 (270.0–617.3)	16.4 (8.1–26.2)	10.4 (2.1–19.1)
14	Alzheimer's disease and other dementias	1.9 (1.5–2.3)	0.0420 (0.0291–0.0561)	146.5 (101.1–195.3)	35.7 (29.2–43.2)	-8.6 (-13.2–-4.2)
15	Stroke	1.7 (1.4–2.0)	0.0379 (0.0277–0.0479)	167.5 (122.3–211.0)	28.3 (18.7–40.1)	-4.6 (-11.2–2.8)
16	Neck pain	1.6 (1.2–2.0)	0.0364 (0.0244–0.0510)	236.3 (156.4–343.1)	11.7 (6.0–17.8)	-1.5 (-4.2–1.3)
17	Autism spectrum disorders	1.6 (1.1–2.4)	0.0363 (0.0258–0.0505)	299.1 (212.6–417.0)	6.8 (-2.1–16.0)	2.3 (-5.7–11.2)
18	Schizophrenia	1.4 (1.0–1.9)	0.0318 (0.0235–0.0403)	219.5 (161.4–280.6)	1.1 (-6.6–9.1)	-6.4 (-13.2–1.2)
19	Gynecological diseases	1.3 (1.1–1.6)	0.0306 (0.0213–0.0428)	234.4 (161.8–329.9)	-19.4 (-26.2–-12.3)	-18.3 (-25.0–-10.1)
20	Dermatitis	1.2 (0.8–1.8)	0.0281 (0.0162–0.0442)	239.1 (135.0–379.2)	-3.7 (-7.8–0.0)	-6.3 (-10.8–-1.9)
21	Alcohol use disorders	1.2 (1.0–1.4)	0.0264 (0.0180–0.0369)	193.3 (132.8–270.4)	-15.7 (-23.9–-6.1)	-20.2 (-27.6–-11.9)
22	Road injuries	1.1 (1.0–1.3)	0.0257 (0.0183–0.0347)	145.0 (103.3–195.3)	-26.5 (-29.1–-23.8)	-41.1 (-43.0–-39.2)
23	Chronic kidney disease	1.1 (0.9–1.4)	0.0254 (0.0177–0.0332)	115.8 (81.7–150.7)	62.2 (48.4–77.1)	21.3 (10.9–32.1)
24	Blindness and vision loss	1.1 (0.8–1.6)	0.0253 (0.0162–0.0383)	130.9 (82.0–204.5)	32.0 (23.8–41.2)	3.0 (-3.2–9.6)
25	Atrial fibrillation and flutter	1.0 (0.9–1.2)	0.0235 (0.0164–0.0311)	88.9 (62.1–117.3)	27.9 (1.7–62.8)	-8.8 (-26.7–15.5)

Table S3: Cause-specific YLDs by US state in 2021 and percentage change between 1990 and 2021, Rhode Island

Rank	Cause Name	2021 Percentage of all cause YLDs	2021 YLDs (millions)	2021 Age Standardised Rate (per 100 000)	Percentage change YLD count 1990 to 2021	Percentage change age-standardised YLD rate 1990 to 2021
	All causes	100.0 (100.0–100.0)	0.184 (0.141–0.236)	13288.6 (10044.7–17033.1)	35.2 (31.0–40.0)	14.6 (10.2–19.4)
1	Low back pain	8.7 (7.7–9.8)	0.0160 (0.0116–0.0209)	1110.4 (810.2–1448.5)	9.2 (1.8–17.8)	-10.7 (-16.5–-3.1)
2	Depressive disorders	7.0 (5.3–9.3)	0.0129 (0.00858–0.0182)	1126.2 (740.0–1588.8)	55.5 (27.9–89.8)	50.2 (23.5–83.2)
3	Drug use disorders	6.7 (5.1–8.5)	0.0123 (0.00847–0.0159)	1122.4 (765.2–1454.8)	293.5 (238.6–358.0)	299.2 (241.6–366.0)
4	Other musculoskeletal disorders	6.6 (5.3–8.2)	0.0122 (0.00855–0.0165)	903.6 (636.9–1222.7)	72.8 (52.9–94.2)	47.6 (31.8–65.9)
5	Diabetes mellitus	6.4 (5.5–7.2)	0.0118 (0.00841–0.0160)	625.7 (440.7–852.1)	240.7 (212.8–269.8)	137.8 (118.4–157.7)
6	Anxiety disorders	5.0 (3.5–6.9)	0.00920 (0.00598–0.0135)	778.3 (508.1–1147.5)	35.3 (6.7–69.4)	27.1 (0.4–59.4)
7	Headache disorders	4.3 (1.0–8.5)	0.00807 (0.00183–0.0169)	687.8 (140.0–1463.0)	4.2 (-1.3–16.4)	-3.2 (-7.9–3.3)
8	Age-related and other hearing loss	4.3 (3.4–5.3)	0.00789 (0.00554–0.0109)	416.4 (292.5–575.0)	27.9 (22.7–33.8)	-5.3 (-8.6–-1.5)
9	Falls	3.8 (3.1–4.5)	0.00693 (0.00485–0.00966)	365.8 (253.4–507.7)	48.9 (39.5–57.8)	2.1 (-3.9–7.8)
10	Osteoarthritis	3.3 (1.9–6.1)	0.00605 (0.00297–0.0122)	308.0 (149.8–620.0)	51.6 (47.6–56.5)	5.6 (2.7–8.7)
11	Chronic obstructive pulmonary disease	2.6 (2.1–3.3)	0.00481 (0.00414–0.00553)	236.2 (204.2–271.2)	53.7 (43.3–66.3)	9.3 (2.0–17.9)
12	Oral disorders	2.4 (1.6–3.3)	0.00437 (0.00258–0.00658)	267.0 (155.1–405.9)	18.0 (7.9–28.2)	-8.1 (-16.2–0.1)
13	Asthma	2.3 (1.7–2.9)	0.00421 (0.00271–0.00615)	409.9 (265.0–614.4)	19.0 (9.2–29.8)	11.4 (2.7–22.3)
14	Alzheimer's disease and other dementias	1.9 (1.5–2.4)	0.00348 (0.00242–0.00460)	149.7 (103.7–197.8)	28.3 (22.3–34.3)	-9.0 (-13.0–-4.8)
15	Alcohol use disorders	1.7 (1.4–2.1)	0.00320 (0.00219–0.00442)	264.1 (182.0–370.0)	-18.5 (-26.0–-8.5)	-23.9 (-30.4–-15.9)
16	Neck pain	1.7 (1.3–2.2)	0.00316 (0.00212–0.00442)	237.7 (158.6–344.9)	14.9 (8.0–22.0)	-1.1 (-4.0–2.0)
17	Schizophrenia	1.6 (1.2–2.1)	0.00283 (0.00209–0.00355)	222.2 (165.0–282.9)	4.3 (-3.9–13.6)	-6.1 (-13.4–2.1)
18	Stroke	1.4 (1.2–1.7)	0.00264 (0.00191–0.00337)	142.0 (102.4–180.6)	20.7 (10.9–31.0)	-9.1 (-15.5–-1.5)
19	Gynecological diseases	1.4 (1.2–1.7)	0.00262 (0.00182–0.00363)	227.8 (156.6–320.6)	-15.3 (-21.1–-8.5)	-16.7 (-23.3–-9.5)
20	Blindness and vision loss	1.2 (0.8–1.7)	0.00214 (0.00136–0.00323)	131.6 (81.8–203.7)	32.3 (23.2–43.0)	2.3 (-4.1–9.6)
21	Autism spectrum disorders	1.2 (0.8–1.7)	0.00210 (0.00146–0.00292)	203.9 (142.2–282.8)	6.6 (-1.4–17.0)	2.1 (-5.7–11.4)
22	Chronic kidney disease	1.1 (0.9–1.3)	0.00204 (0.00143–0.00264)	110.2 (77.3–142.7)	67.3 (53.5–83.4)	24.9 (15.1–36.1)
23	Bipolar disorder	1.0 (0.7–1.4)	0.00190 (0.00126–0.00268)	157.4 (104.9–222.9)	5.9 (-1.0–13.7)	-2.6 (-9.1–4.6)
24	Road injuries	1.0 (0.9–1.1)	0.00183 (0.00131–0.00245)	121.7 (87.0–163.1)	-28.0 (-30.2–-25.4)	-43.2 (-44.8–-41.3)
25	Other mental disorders	1.0 (0.7–1.3)	0.00178 (0.00117–0.00261)	130.3 (83.8–192.9)	15.3 (9.9–21.5)	-0.7 (-5.5–4.1)

Table S3: Cause-specific YLDs by US state in 2021 and percentage change between 1990 and 2021, South Carolina						
Rank	Cause Name	2021 Percentage of all cause YLDs	2021 YLDs (millions)	2021 Age Standardised Rate (per 100 000)	Percentage change YLD count 1990 to 2021	Percentage change age-standardised YLD rate 1990 to 2021
	All causes	100.0 (100.0–100.0)	0.872 (0.665–1.12)	13736.6 (10390.1–17722.1)	92.8 (86.5–100.8)	16.5 (12.1–21.7)
1	Low back pain	9.3 (8.3–10.5)	0.0813 (0.0589–0.106)	1234.7 (895.8–1605.3)	60.3 (48.7–73.1)	-5.0 (-11.1–1.9)
2	Diabetes mellitus	7.1 (6.2–8.0)	0.0624 (0.0443–0.0843)	741.6 (524.7–999.6)	419.3 (382.1–457.1)	146.9 (130.1–164.0)
3	Other musculoskeletal disorders	6.7 (5.3–8.3)	0.0584 (0.0416–0.0789)	917.8 (657.6–1232.4)	144.9 (118.9–178.3)	50.0 (33.0–70.8)
4	Depressive disorders	6.7 (5.1–8.7)	0.0582 (0.0395–0.0833)	1113.9 (761.4–1590.2)	115.8 (75.5–164.9)	57.8 (28.2–94.5)
5	Drug use disorders	6.3 (4.8–7.9)	0.0546 (0.0386–0.0712)	1105.4 (781.1–1451.7)	428.3 (365.8–498.1)	315.3 (264.6–371.9)
6	Anxiety disorders	5.0 (3.7–6.7)	0.0439 (0.0288–0.0636)	815.5 (527.4–1190.9)	87.8 (46.2–134.1)	33.1 (5.0–65.8)
7	Headache disorders	4.2 (1.0–8.1)	0.0367 (0.00816–0.0765)	684.1 (137.5–1445.2)	36.1 (29.0–52.7)	-3.6 (-8.2–1.8)
8	Age-related and other hearing loss	4.1 (3.3–5.1)	0.0358 (0.0250–0.0494)	417.3 (291.6–577.8)	100.0 (92.1–109.4)	-5.8 (-9.1–-2.6)
9	Osteoarthritis	3.1 (1.8–5.7)	0.0270 (0.0131–0.0547)	304.1 (146.0–614.4)	132.1 (125.5–140.1)	5.6 (2.6–9.3)
10	Falls	2.8 (2.3–3.4)	0.0248 (0.0173–0.0347)	294.4 (204.6–408.4)	113.5 (97.5–129.6)	-0.4 (-6.5–5.8)
11	Chronic obstructive pulmonary disease	2.5 (2.0–3.2)	0.0218 (0.0187–0.0250)	234.7 (203.3–267.5)	145.4 (127.3–167.3)	8.5 (0.5–17.9)
12	Asthma	2.4 (1.8–3.0)	0.0207 (0.0135–0.0300)	409.6 (267.0–610.0)	60.2 (48.1–72.5)	9.1 (1.5–17.1)
13	Oral disorders	2.3 (1.6–3.2)	0.0204 (0.0121–0.0309)	273.3 (158.6–416.9)	78.4 (62.2–93.4)	-5.7 (-14.2–2.2)
14	Stroke	1.9 (1.6–2.2)	0.0165 (0.0119–0.0210)	195.9 (141.5–248.3)	101.5 (87.0–119.9)	-4.0 (-10.7–4.6)
15	Alzheimer's disease and other dementias	1.7 (1.3–2.1)	0.0145 (0.00993–0.0194)	146.2 (100.0–194.1)	133.0 (121.6–145.1)	-7.3 (-11.6–-2.2)
16	Neck pain	1.6 (1.3–2.1)	0.0143 (0.00969–0.0203)	236.7 (157.5–342.3)	54.2 (44.3–65.8)	-1.4 (-4.1–1.5)
17	Road injuries	1.6 (1.3–1.8)	0.0137 (0.00983–0.0185)	200.8 (144.8–270.2)	10.3 (6.2–14.1)	-37.2 (-39.1–-35.4)
18	Alcohol use disorders	1.5 (1.2–1.9)	0.0133 (0.00936–0.0182)	245.2 (172.5–341.0)	1.9 (-7.6–12.2)	-26.9 (-33.5–-19.5)
19	Gynecological diseases	1.4 (1.2–1.8)	0.0127 (0.00884–0.0176)	240.6 (166.6–339.3)	13.0 (5.0–21.6)	-16.1 (-22.5–-8.6)
20	Schizophrenia	1.4 (1.1–1.9)	0.0125 (0.00929–0.0159)	218.5 (160.1–280.0)	37.0 (26.7–47.5)	-6.0 (-12.8–1.0)
21	Chronic kidney disease	1.2 (1.0–1.5)	0.0106 (0.00754–0.0138)	133.2 (94.9–172.2)	149.3 (125.4–177.6)	23.4 (12.4–33.8)
22	Neonatal disorders	1.1 (0.9–1.4)	0.00983 (0.00700–0.0133)	222.1 (156.9–303.0)	29.6 (2.6–62.4)	-0.1 (-20.7–25.6)
23	Blindness and vision loss	1.1 (0.8–1.6)	0.00954 (0.00615–0.0144)	131.3 (82.4–203.2)	90.8 (78.7–102.9)	0.9 (-5.2–7.6)
24	Autism spectrum disorders	1.1 (0.7–1.6)	0.00951 (0.00662–0.0133)	197.0 (136.6–276.7)	40.7 (30.0–53.4)	2.2 (-5.8–11.9)
25	COVID-19	0.9 (0.4–2.1)	0.00820 (0.00312–0.0183)	145.8 (53.6–342.4)	0.0 (0.0–0.0)	0.0 (0.0–0.0)

Table S3: Cause-specific YLDs by US state in 2021 and percentage change between 1990 and 2021, South Dakota						
Rank	Cause Name	2021 Percentage of all cause YLDs	2021 YLDs (millions)	2021 Age Standardised Rate (per 100 000)	Percentage change YLD count 1990 to 2021	Percentage change age-standardised YLD rate 1990 to 2021
	All causes	100.0 (100.0–100.0)	0.138 (0.104–0.177)	12771.1 (9589.9–16446.9)	54.5 (50.3–59.8)	13.2 (9.4–17.8)
1	Low back pain	9.9 (8.7–11.3)	0.0136 (0.00991–0.0179)	1272.6 (929.7–1676.1)	30.8 (23.3–39.9)	-5.4 (-10.9–1.0)
2	Other musculoskeletal disorders	8.8 (6.9–10.9)	0.0121 (0.00850–0.0161)	1226.7 (863.8–1643.5)	118.5 (97.7–144.0)	67.5 (50.8–87.4)
3	Diabetes mellitus	6.1 (5.3–7.0)	0.00848 (0.00602–0.0116)	595.4 (422.2–805.0)	276.6 (246.8–309.5)	134.4 (116.0–154.2)
4	Depressive disorders	5.0 (3.9–6.5)	0.00694 (0.00471–0.00992)	786.1 (529.1–1126.4)	64.7 (34.0–98.8)	35.0 (10.1–63.8)
5	Anxiety disorders	4.9 (3.5–6.8)	0.00675 (0.00423–0.00987)	743.0 (464.0–1091.3)	52.2 (18.4–92.1)	21.9 (-5.9–54.2)
6	Age-related and other hearing loss	4.5 (3.6–5.5)	0.00618 (0.00429–0.00851)	421.9 (294.2–579.4)	44.2 (38.2–50.0)	-5.4 (-8.6–-2.0)
7	Headache disorders	4.4 (1.0–8.6)	0.00611 (0.00133–0.0128)	681.6 (135.1–1436.6)	21.2 (14.7–34.1)	-3.6 (-8.4–2.2)
8	Falls	4.1 (3.4–4.8)	0.00569 (0.00402–0.00787)	400.1 (278.7–550.3)	83.3 (72.7–93.2)	13.7 (7.7–19.7)
9	Drug use disorders	3.6 (2.7–4.5)	0.00486 (0.00336–0.00635)	607.5 (417.1–794.9)	255.8 (215.2–307.4)	211.2 (173.7–258.1)
10	Osteoarthritis	3.1 (1.8–5.9)	0.00431 (0.00208–0.00873)	289.6 (138.7–582.0)	75.1 (70.1–80.4)	8.4 (5.2–11.6)
11	Chronic obstructive pulmonary disease	2.6 (2.1–3.3)	0.00353 (0.00301–0.00409)	222.0 (188.8–255.1)	73.1 (60.5–89.3)	7.8 (0.3–17.5)
12	Oral disorders	2.5 (1.8–3.4)	0.00347 (0.00207–0.00524)	277.3 (161.6–422.7)	38.5 (27.5–49.9)	-5.1 (-13.4–3.8)
13	Asthma	2.5 (1.9–3.2)	0.00342 (0.00221–0.00507)	376.5 (244.9–567.3)	33.5 (22.2–45.7)	5.0 (-3.3–14.4)
14	Alzheimer's disease and other dementias	2.0 (1.6–2.6)	0.00281 (0.00192–0.00373)	150.4 (102.6–200.1)	42.5 (35.7–50.2)	-6.8 (-11.0–-2.1)
15	Road injuries	1.8 (1.5–2.0)	0.00245 (0.00175–0.00330)	218.1 (157.0–295.2)	4.6 (2.3–7.4)	-27.0 (-28.5–-25.2)
16	Neck pain	1.7 (1.3–2.2)	0.00238 (0.00160–0.00333)	237.1 (156.4–342.4)	32.5 (25.5–39.9)	-1.1 (-4.1–1.9)
17	Alcohol use disorders	1.6 (1.3–2.0)	0.00225 (0.00157–0.00313)	252.7 (175.3–351.6)	1.4 (-8.5–10.7)	-18.6 (-25.6–-11.3)
18	Stroke	1.6 (1.3–1.9)	0.00223 (0.00161–0.00286)	151.9 (109.5–194.6)	46.2 (34.3–60.5)	-3.0 (-10.1–4.8)
19	Schizophrenia	1.5 (1.1–2.1)	0.00209 (0.00155–0.00268)	222.1 (164.5–284.1)	21.1 (11.3–31.0)	-5.7 (-12.6–1.7)
20	Gynecological diseases	1.4 (1.1–1.6)	0.00187 (0.00129–0.00258)	216.4 (149.3–304.5)	-3.5 (-11.0–4.8)	-18.6 (-25.7–-11.1)
21	Autism spectrum disorders	1.2 (0.8–1.8)	0.00168 (0.00117–0.00237)	200.2 (138.1–282.9)	24.5 (14.8–35.0)	1.6 (-6.3–10.3)
22	Blindness and vision loss	1.2 (0.9–1.7)	0.00166 (0.00108–0.00249)	133.0 (83.6–203.9)	47.7 (39.0–57.2)	2.1 (-4.3–8.4)
23	Chronic kidney disease	1.2 (1.0–1.4)	0.00161 (0.00112–0.00210)	112.9 (81.0–145.7)	90.9 (75.1–108.9)	28.4 (18.6–40.3)
24	COVID-19	1.1 (0.4–2.7)	0.00157 (0.000530–0.00379)	163.3 (51.9–411.0)	0.0 (0.0–0.0)	0.0 (0.0–0.0)
25	Other mental disorders	1.0 (0.7–1.3)	0.00135 (0.000880–0.00196)	132.1 (85.7–192.8)	33.3 (27.4–39.9)	-0.4 (-5.6–4.3)

Table S3: Cause-specific YLDs by US state in 2021 and percentage change between 1990 and 2021, Tennessee						
Rank	Cause Name	2021 Percentage of all cause YLDs	2021 YLDs (millions)	2021 Age Standardised Rate (per 100 000)	Percentage change YLD count 1990 to 2021	Percentage change age-standardised YLD rate 1990 to 2021
	All causes	100.0 (100.0–100.0)	1.17 (0.892–1.50)	13943.1 (10470.7–17766.4)	81.2 (75.4–88.7)	19.5 (14.9–25.0)
1	Low back pain	9.2 (8.2–10.4)	0.108 (0.0781–0.141)	1245.3 (896.3–1611.8)	44.4 (34.5–55.7)	-6.4 (-12.3–1.0)
2	Drug use disorders	8.1 (6.2–10.3)	0.0951 (0.0681–0.123)	1413.6 (1013.0–1839.3)	550.8 (475.7–639.0)	416.7 (355.3–490.1)
3	Depressive disorders	6.8 (5.2–9.0)	0.0796 (0.0528–0.113)	1127.8 (738.4–1600.9)	109.2 (69.1–155.3)	59.0 (28.7–93.6)
4	Diabetes mellitus	6.7 (5.7–7.6)	0.0786 (0.0555–0.107)	711.8 (502.5–962.7)	338.1 (305.5–372.9)	138.6 (122.7–155.1)
5	Other musculoskeletal disorders	6.2 (5.0–7.7)	0.0732 (0.0519–0.0980)	884.8 (632.2–1189.0)	118.3 (94.4–146.3)	46.9 (30.7–65.9)
6	Anxiety disorders	5.0 (3.6–6.9)	0.0586 (0.0386–0.0875)	805.7 (516.2–1203.4)	77.3 (40.0–122.5)	31.3 (3.9–65.5)
7	Headache disorders	4.2 (1.0–8.1)	0.0497 (0.0109–0.105)	685.4 (136.0–1450.5)	31.2 (24.3–47.0)	-3.6 (-8.4–2.4)
8	Age-related and other hearing loss	4.0 (3.2–4.9)	0.0469 (0.0328–0.0644)	418.2 (294.2–578.2)	70.4 (63.7–77.2)	-5.7 (-9.1–-2.3)
9	Osteoarthritis	3.2 (1.8–5.9)	0.0377 (0.0183–0.0765)	324.7 (156.6–658.0)	99.3 (93.2–105.8)	5.7 (2.4–8.9)
10	Falls	3.1 (2.6–3.7)	0.0370 (0.0258–0.0518)	331.1 (230.0–459.7)	103.1 (86.9–117.3)	7.2 (-0.2–13.9)
11	Oral disorders	2.5 (1.8–3.5)	0.0298 (0.0179–0.0454)	299.9 (175.0–461.2)	66.8 (51.8–80.8)	-1.1 (-9.2–7.6)
12	Chronic obstructive pulmonary disease	2.5 (2.0–3.2)	0.0294 (0.0254–0.0334)	245.5 (212.9–277.4)	108.2 (93.0–128.4)	9.8 (1.9–20.2)
13	Asthma	2.1 (1.6–2.6)	0.0243 (0.0158–0.0355)	359.9 (233.8–528.4)	43.8 (32.9–55.9)	1.8 (-6.0–11.3)
14	Stroke	1.9 (1.6–2.2)	0.0221 (0.0159–0.0280)	199.8 (143.5–254.2)	77.4 (62.7–91.6)	-0.7 (-8.1–6.4)
15	Neck pain	1.6 (1.3–2.1)	0.0192 (0.0128–0.0268)	236.8 (157.4–342.0)	44.7 (36.5–53.2)	-1.5 (-4.5–1.5)
16	Alzheimer's disease and other dementias	1.6 (1.3–2.0)	0.0190 (0.0131–0.0252)	146.8 (101.0–194.6)	79.4 (70.3–88.8)	-7.4 (-11.6–-2.8)
17	Gynecological diseases	1.5 (1.2–1.8)	0.0174 (0.0122–0.0244)	245.7 (170.3–347.5)	9.6 (1.6–18.1)	-14.7 (-21.4–-7.7)
18	Schizophrenia	1.5 (1.1–1.9)	0.0169 (0.0125–0.0215)	218.3 (162.9–281.1)	30.2 (20.3–40.6)	-6.2 (-12.9–1.2)
19	Road injuries	1.4 (1.2–1.6)	0.0165 (0.0117–0.0222)	180.8 (129.1–243.1)	-5.8 (-9.1–-2.2)	-41.7 (-43.5–-39.8)
20	Neonatal disorders	1.2 (1.0–1.5)	0.0140 (0.00996–0.0183)	232.1 (166.1–306.2)	36.4 (13.2–65.7)	6.2 (-12.0–28.8)
21	Alcohol use disorders	1.2 (1.0–1.5)	0.0139 (0.00958–0.0194)	189.7 (129.8–266.6)	5.0 (-4.7–16.2)	-22.2 (-29.0–-14.0)
22	Chronic kidney disease	1.1 (0.9–1.4)	0.0131 (0.00931–0.0169)	123.5 (87.6–158.2)	120.8 (100.4–142.8)	26.5 (15.4–37.5)
23	Autism spectrum disorders	1.1 (0.7–1.6)	0.0128 (0.00879–0.0178)	196.2 (135.3–270.8)	36.7 (25.6–47.4)	1.4 (-7.0–9.1)
24	Blindness and vision loss	1.1 (0.8–1.5)	0.0127 (0.00810–0.0193)	131.3 (82.7–204.5)	69.7 (59.1–80.6)	1.2 (-4.8–8.2)
25	COVID-19	1.0 (0.4–2.4)	0.0121 (0.00464–0.0285)	160.2 (57.4–389.5)	0.0 (0.0–0.0)	0.0 (0.0–0.0)

Table S3: Cause-specific YLDs by US state in 2021 and percentage change between 1990 and 2021, Texas

Rank	Cause Name	2021 Percentage of all cause YLDs	2021 YLDs (millions)	2021 Age Standardised Rate (per 100 000)	Percentage change YLD count 1990 to 2021	Percentage change age-standardised YLD rate 1990 to 2021
	All causes	100.0 (100.0–100.0)	4.36 (3.32–5.61)	12832.2 (9715.6–16463.0)	112.6 (105.3–120.9)	13.0 (9.0–17.6)
1	Low back pain	9.1 (8.0–10.2)	0.395 (0.290–0.514)	1127.3 (828.0–1474.7)	75.0 (62.3–87.1)	-9.1 (-15.4–-2.4)
2	Depressive disorders	6.8 (5.1–8.9)	0.297 (0.202–0.426)	974.9 (665.9–1391.5)	154.2 (110.6–207.0)	52.8 (26.3–83.8)
3	Other musculoskeletal disorders	6.7 (5.4–8.3)	0.292 (0.205–0.393)	853.0 (602.6–1145.2)	181.3 (147.8–222.3)	48.2 (31.0–68.8)
4	Diabetes mellitus	6.5 (5.6–7.3)	0.283 (0.198–0.383)	697.0 (489.5–946.7)	474.0 (433.1–523.0)	151.7 (133.8–173.6)
5	Anxiety disorders	5.8 (4.1–7.8)	0.252 (0.169–0.370)	813.1 (536.6–1203.3)	126.2 (74.7–184.3)	32.9 (2.7–67.3)
6	Drug use disorders	5.0 (3.8–6.4)	0.217 (0.149–0.286)	733.2 (500.5–963.9)	462.5 (393.7–543.9)	260.0 (215.6–312.1)
7	Headache disorders	4.8 (1.1–9.5)	0.214 (0.0449–0.454)	687.1 (135.5–1454.2)	65.6 (56.9–82.2)	-3.0 (-7.8–2.4)
8	Age-related and other hearing loss	3.9 (3.1–4.7)	0.170 (0.119–0.235)	420.4 (294.0–586.1)	107.4 (99.2–115.4)	-5.7 (-9.1–-2.2)
9	Osteoarthritis	2.9 (1.7–5.5)	0.128 (0.0621–0.258)	305.6 (147.3–614.2)	150.0 (141.8–158.4)	6.3 (2.7–9.8)
10	Falls	2.8 (2.3–3.3)	0.122 (0.0855–0.170)	305.4 (211.8–423.2)	97.2 (81.3–110.8)	-10.3 (-16.4–-5.1)
11	Asthma	2.5 (1.9–3.2)	0.109 (0.0699–0.163)	384.9 (247.4–576.9)	73.9 (61.4–89.9)	3.1 (-4.0–12.0)
12	Oral disorders	2.4 (1.6–3.2)	0.103 (0.0609–0.155)	274.4 (160.8–414.3)	98.1 (89.8–106.7)	-4.9 (-9.2–-0.4)
13	Chronic obstructive pulmonary disease	2.2 (1.7–2.8)	0.0955 (0.0821–0.110)	224.3 (192.9–257.4)	136.0 (122.1–156.3)	1.7 (-4.2–10.3)
14	Neck pain	1.8 (1.4–2.3)	0.0793 (0.0528–0.114)	237.9 (157.6–346.0)	81.1 (71.4–91.9)	-0.9 (-3.4–2.0)
15	Gynecological diseases	1.7 (1.4–2.1)	0.0755 (0.0522–0.106)	244.4 (169.1–347.2)	42.9 (32.9–53.5)	-13.3 (-19.7–-6.5)
16	Schizophrenia	1.7 (1.2–2.2)	0.0722 (0.0532–0.0917)	222.1 (164.4–283.9)	63.8 (51.4–77.7)	-5.4 (-12.5–2.5)
17	Stroke	1.6 (1.3–1.9)	0.0704 (0.0511–0.0902)	176.2 (126.9–226.0)	106.5 (91.6–124.9)	-5.1 (-11.6–3.1)
18	Road injuries	1.5 (1.3–1.8)	0.0672 (0.0483–0.0904)	187.5 (135.3–251.6)	24.8 (20.4–29.1)	-36.6 (-38.5–-34.7)
19	Alzheimer's disease and other dementias	1.4 (1.1–1.8)	0.0627 (0.0435–0.0831)	144.2 (99.8–190.5)	112.8 (102.0–122.7)	-8.4 (-12.9–-4.5)
20	Alcohol use disorders	1.4 (1.1–1.7)	0.0598 (0.0417–0.0836)	191.3 (132.2–270.4)	26.3 (14.5–39.9)	-24.0 (-31.0–-16.1)
21	Neonatal disorders	1.3 (1.1–1.6)	0.0575 (0.0401–0.0757)	211.3 (147.0–278.2)	45.1 (20.2–76.5)	-8.8 (-24.3–11.3)
22	Autism spectrum disorders	1.3 (0.9–1.9)	0.0568 (0.0392–0.0799)	199.9 (137.3–281.1)	67.7 (53.3–81.4)	1.8 (-6.8–9.8)
23	COVID-19	1.3 (0.5–2.8)	0.0544 (0.0202–0.125)	173.0 (62.8–402.2)	0.0 (0.0–0.0)	0.0 (0.0–0.0)
24	Chronic kidney disease	1.1 (0.9–1.4)	0.0492 (0.0351–0.0643)	126.4 (90.7–165.2)	167.0 (143.4–193.1)	25.7 (15.7–37.2)
25	Dermatitis	1.1 (0.8–1.6)	0.0490 (0.0282–0.0778)	177.4 (100.9–283.5)	49.4 (42.6–55.6)	-8.3 (-13.1–-3.9)

Table S3: Cause-specific YLDs by US state in 2021 and percentage change between 1990 and 2021, Utah						
Rank	Cause Name	2021 Percentage of all cause YLDs	2021 YLDs (millions)	2021 Age Standardised Rate (per 100 000)	Percentage change YLD count 1990 to 2021	Percentage change age-standardised YLD rate 1990 to 2021
	All causes	100.0 (100.0–100.0)	0.474 (0.357–0.604)	13178.6 (9882.1–16774.0)	145.3 (136.2–156.1)	15.1 (10.7–20.1)
1	Low back pain	9.5 (8.4–10.7)	0.0450 (0.0329–0.0585)	1233.1 (899.9–1606.0)	107.3 (93.0–123.2)	-6.1 (-12.2–0.7)
2	Depressive disorders	9.2 (7.1–11.6)	0.0439 (0.0285–0.0625)	1271.9 (823.1–1809.7)	207.7 (152.1–269.3)	55.8 (27.9–86.9)
3	Other musculoskeletal disorders	7.0 (5.7–8.7)	0.0334 (0.0239–0.0449)	922.5 (661.2–1239.5)	230.4 (193.4–274.3)	50.8 (33.7–70.3)
4	Drug use disorders	6.6 (5.0–8.3)	0.0310 (0.0215–0.0406)	921.0 (638.1–1205.5)	744.5 (632.8–872.7)	348.5 (288.2–415.9)
5	Anxiety disorders	5.6 (4.0–7.7)	0.0266 (0.0168–0.0389)	769.6 (484.3–1127.3)	151.6 (93.8–217.5)	25.8 (-3.1–58.5)
6	Diabetes mellitus	5.3 (4.5–6.0)	0.0251 (0.0177–0.0340)	633.5 (442.3–858.6)	453.9 (412.6–497.4)	117.1 (100.5–134.5)
7	Headache disorders	4.9 (1.1–9.9)	0.0236 (0.00489–0.0497)	683.2 (137.5–1440.0)	92.2 (82.6–114.7)	-3.5 (-8.2–2.4)
8	Age-related and other hearing loss	3.5 (2.8–4.4)	0.0167 (0.0117–0.0230)	419.8 (294.6–577.2)	131.6 (123.0–141.1)	-5.8 (-8.8–-2.3)
9	Falls	3.0 (2.5–3.6)	0.0143 (0.0100–0.0198)	363.2 (252.9–498.8)	162.7 (142.6–180.2)	9.0 (1.8–15.1)
10	Osteoarthritis	2.7 (1.6–5.1)	0.0129 (0.00625–0.0264)	322.5 (155.9–655.9)	175.9 (167.8–185.9)	5.9 (2.7–9.6)
11	Asthma	2.5 (1.9–3.3)	0.0121 (0.00783–0.0177)	360.3 (233.9–531.6)	85.6 (67.0–105.3)	-1.9 (-9.4–6.1)
12	Oral disorders	2.1 (1.5–3.0)	0.0101 (0.00589–0.0154)	267.2 (156.2–410.4)	116.8 (98.5–134.9)	-7.5 (-15.8–0.5)
13	Neck pain	1.8 (1.4–2.3)	0.00843 (0.00562–0.0121)	237.1 (157.8–343.3)	109.5 (98.8–120.9)	-1.2 (-4.1–2.0)
14	Gynecological diseases	1.6 (1.3–2.0)	0.00780 (0.00538–0.0109)	226.5 (156.0–316.0)	68.4 (55.5–82.4)	-16.0 (-22.4–-8.7)
15	Schizophrenia	1.6 (1.2–2.2)	0.00772 (0.00575–0.00987)	221.6 (165.0–284.1)	93.9 (78.1–112.1)	-5.9 (-13.3–2.7)
16	Chronic obstructive pulmonary disease	1.5 (1.2–2.0)	0.00713 (0.00605–0.00827)	172.3 (146.6–200.0)	142.8 (125.5–163.1)	-6.0 (-12.6–1.8)
17	Autism spectrum disorders	1.4 (0.9–2.1)	0.00664 (0.00456–0.00924)	203.2 (139.8–283.5)	87.9 (73.6–102.7)	2.4 (-5.5–10.7)
18	Alcohol use disorders	1.3 (1.1–1.7)	0.00639 (0.00434–0.00885)	185.3 (125.6–257.0)	55.6 (40.1–72.5)	-21.5 (-28.4–-13.5)
19	Alzheimer's disease and other dementias	1.3 (1.0–1.6)	0.00600 (0.00413–0.00794)	143.4 (98.7–189.5)	144.4 (133.0–157.2)	-6.7 (-11.0–-1.9)
20	Stroke	1.3 (1.1–1.5)	0.00599 (0.00436–0.00761)	151.3 (110.4–191.3)	124.6 (109.4–143.2)	-7.4 (-13.6–0.1)
21	Neonatal disorders	1.2 (1.0–1.5)	0.00585 (0.00414–0.00773)	182.8 (128.7–241.3)	88.2 (49.8–136.5)	6.0 (-15.7–33.2)
22	COVID-19	1.2 (0.5–2.7)	0.00547 (0.00203–0.0133)	157.8 (57.9–388.8)	0.0 (0.0–0.0)	0.0 (0.0–0.0)
23	Road injuries	1.1 (1.0–1.3)	0.00529 (0.00376–0.00709)	143.4 (102.1–192.5)	18.2 (14.1–22.9)	-47.2 (-48.8–-45.3)
24	Viral skin diseases	1.1 (0.8–1.4)	0.00502 (0.00323–0.00749)	153.5 (98.8–229.7)	74.9 (70.7–79.6)	-2.2 (-4.6–0.4)
25	Bipolar disorder	1.0 (0.7–1.4)	0.00496 (0.00325–0.00708)	142.0 (93.3–203.1)	95.1 (80.2–109.7)	-2.5 (-9.6–4.5)

Table S3: Cause-specific YLDs by US state in 2021 and percentage change between 1990 and 2021, Vermont						
**Rank**	**Cause Name**	**2021 Percentage of all cause YLDs**	**2021 YLDs (millions)**	**2021 Age Standardised Rate (per 100 000)**	**Percentage change YLD count 1990 to 2021**	**Percentage change age-standardised YLD rate 1990 to 2021**
	All causes	100.0 (100.0–100.0)	0.106 (0.0807–0.136)	12734.1 (9472.1–16331.3)	49.9 (45.3–55.2)	12.9 (8.7–17.1)
1	Low back pain	9.0 (7.9–10.2)	0.00958 (0.00694–0.0124)	1113.5 (807.8–1456.5)	20.9 (11.9–31.2)	-10.5 (-16.1–-3.3)
2	Depressive disorders	6.7 (5.1–8.5)	0.00710 (0.00475–0.0101)	1105.4 (723.0–1583.8)	62.9 (34.2–97.0)	56.7 (29.8–88.2)
3	Other musculoskeletal disorders	6.6 (5.1–8.2)	0.00697 (0.00486–0.00940)	883.7 (624.8–1187.5)	77.2 (57.2–102.0)	40.3 (25.6–58.0)
4	Diabetes mellitus	6.4 (5.5–7.3)	0.00680 (0.00478–0.00927)	563.9 (397.1–766.0)	291.9 (259.8–325.5)	111.9 (95.8–128.0)
5	Anxiety disorders	5.2 (3.6–7.0)	0.00552 (0.00340–0.00802)	825.1 (513.0–1220.0)	46.1 (12.0–82.6)	34.9 (4.4–67.8)
6	Drug use disorders	4.9 (3.7–6.2)	0.00523 (0.00377–0.00676)	899.2 (646.6–1166.4)	239.0 (196.8–285.2)	260.4 (215.1–312.2)
7	Age-related and other hearing loss	4.8 (3.8–5.8)	0.00507 (0.00353–0.00703)	418.6 (291.4–578.4)	68.4 (61.0–76.5)	-5.3 (-8.6–-1.8)
8	Falls	4.7 (3.9–5.6)	0.00501 (0.00354–0.00705)	417.7 (292.0–580.2)	94.3 (79.8–107.1)	7.4 (0.9–12.9)
9	Headache disorders	4.3 (1.0–8.2)	0.00457 (0.00105–0.00951)	686.2 (137.2–1453.1)	4.8 (-1.5–22.7)	-3.3 (-7.8–2.7)
10	Osteoarthritis	3.5 (2.1–6.7)	0.00379 (0.00184–0.00761)	300.4 (144.4–600.3)	102.9 (96.3–110.3)	6.6 (3.5–9.9)
11	Chronic obstructive pulmonary disease	2.8 (2.2–3.5)	0.00296 (0.00250–0.00340)	223.6 (190.8–255.9)	102.9 (91.3–116.3)	5.9 (-0.1–12.8)
12	Oral disorders	2.7 (1.9–3.7)	0.00288 (0.00175–0.00426)	282.4 (166.0–427.8)	52.0 (44.8–58.9)	-3.3 (-7.4–0.9)
13	Asthma	2.3 (1.8–2.9)	0.00246 (0.00159–0.00356)	386.4 (249.8–572.5)	23.3 (13.0–34.2)	6.6 (-0.3–14.5)
14	Alzheimer's disease and other dementias	2.1 (1.6–2.6)	0.00220 (0.00151–0.00292)	148.4 (102.5–195.5)	80.0 (70.4–89.6)	-7.7 (-12.1–-3.2)
15	Neck pain	1.7 (1.3–2.2)	0.00185 (0.00125–0.00257)	238.0 (158.8–344.9)	22.4 (11.0–35.3)	-1.0 (-3.8–2.0)
16	Stroke	1.6 (1.3–1.9)	0.00171 (0.00125–0.00219)	143.1 (103.7–183.0)	59.3 (47.3–73.2)	-8.6 (-15.4–-0.8)
17	Schizophrenia	1.5 (1.1–2.0)	0.00161 (0.00119–0.00206)	223.3 (164.4–283.5)	6.1 (-2.1–15.9)	-5.6 (-12.9–2.0)
18	Alcohol use disorders	1.4 (1.2–1.8)	0.00154 (0.00105–0.00213)	227.3 (153.2–317.9)	-16.7 (-24.5–-7.2)	-22.3 (-28.7–-15.1)
19	Road injuries	1.3 (1.2–1.5)	0.00143 (0.00102–0.00192)	156.4 (112.6–209.8)	-13.0 (-16.2–-10.0)	-39.2 (-40.8–-37.4)
20	Gynecological diseases	1.3 (1.0–1.6)	0.00137 (0.000957–0.00191)	215.9 (147.6–306.3)	-19.2 (-25.9–-12.3)	-18.5 (-25.7–-11.1)
21	Blindness and vision loss	1.2 (0.9–1.8)	0.00132 (0.000847–0.00199)	132.4 (82.3–202.7)	58.3 (48.0–70.6)	2.5 (-3.6–9.9)
22	Autism spectrum disorders	1.2 (0.7–1.7)	0.00122 (0.000829–0.00171)	205.2 (139.6–288.8)	8.2 (-2.0–19.5)	2.6 (-7.2–13.0)
23	Chronic kidney disease	1.1 (0.9–1.4)	0.00119 (0.000847–0.00155)	101.9 (72.1–131.8)	113.1 (91.9–138.0)	26.7 (16.0–40.2)
24	Atrial fibrillation and flutter	1.1 (0.9–1.3)	0.00113 (0.000786–0.00150)	79.3 (55.2–105.1)	133.8 (84.8–199.5)	18.3 (-5.0–49.8)
25	Other mental disorders	1.0 (0.7–1.3)	0.00105 (0.000676–0.00152)	131.6 (84.3–192.7)	23.9 (18.0–30.4)	-0.1 (-5.2–4.9)

Table S3: Cause-specific YLDs by US state in 2021 and percentage change between 1990 and 2021, Virginia						
Rank	Cause Name	2021 Percentage of all cause YLDs	2021 YLDs (millions)	2021 Age Standardised Rate (per 100 000)	Percentage change YLD count 1990 to 2021	Percentage change age-standardised YLD rate 1990 to 2021
	All causes	100.0 (100.0–100.0)	1.37 (1.04–1.76)	13016.4 (9750.6–16686.8)	74.5 (68.8–80.9)	13.3 (9.4–17.8)
1	Low back pain	9.1 (8.0–10.3)	0.125 (0.0914–0.163)	1145.5 (833.9–1498.6)	41.4 (31.4–53.2)	-9.4 (-15.1–-2.7)
2	Diabetes mellitus	6.9 (6.0–7.8)	0.0952 (0.0671–0.129)	700.1 (493.6–947.5)	392.0 (353.1–430.5)	152.1 (132.9–171.0)
3	Depressive disorders	6.8 (5.2–9.0)	0.0935 (0.0618–0.134)	1059.8 (699.6–1532.9)	99.8 (61.9–142.4)	56.5 (26.9–88.8)
4	Other musculoskeletal disorders	6.7 (5.4–8.3)	0.0925 (0.0657–0.124)	885.1 (630.8–1182.1)	121.5 (96.4–153.8)	47.9 (31.0–69.1)
5	Anxiety disorders	5.4 (3.8–7.3)	0.0737 (0.0462–0.108)	803.8 (505.0–1184.7)	74.5 (34.2–118.1)	31.4 (2.2–64.3)
6	Drug use disorders	5.1 (3.9–6.4)	0.0696 (0.0501–0.0908)	819.0 (585.2–1072.0)	344.4 (290.3–404.7)	274.3 (225.8–328.3)
7	Headache disorders	4.5 (1.0–8.9)	0.0629 (0.0142–0.132)	686.7 (139.9–1459.6)	29.2 (22.1–43.9)	-3.0 (-7.7–2.1)
8	Age-related and other hearing loss	4.1 (3.3–5.1)	0.0568 (0.0397–0.0779)	417.4 (291.6–572.8)	82.2 (75.4–89.4)	-5.6 (-8.7–-2.0)
9	Osteoarthritis	3.2 (1.9–6.0)	0.0443 (0.0212–0.0888)	313.3 (149.5–628.7)	113.5 (106.8–121.1)	5.6 (2.5–9.4)
10	Falls	3.1 (2.6–3.7)	0.0430 (0.0302–0.0596)	316.6 (220.5–435.6)	104.7 (89.4–118.8)	5.2 (-1.2–10.9)
11	Asthma	2.5 (1.9–3.2)	0.0345 (0.0224–0.0506)	410.8 (266.5–617.4)	47.6 (36.8–58.5)	5.1 (-2.5–13.3)
12	Chronic obstructive pulmonary disease	2.4 (1.9–3.0)	0.0325 (0.0277–0.0373)	221.8 (188.7–253.1)	117.2 (103.7–134.9)	5.2 (-1.0–13.5)
13	Oral disorders	2.3 (1.6–3.2)	0.0320 (0.0198–0.0459)	266.0 (165.7–385.2)	60.2 (33.8–96.9)	-7.7 (-22.2–12.0)
14	Neck pain	1.8 (1.3–2.2)	0.0242 (0.0162–0.0341)	237.8 (156.5–341.1)	44.3 (34.5–54.5)	-0.9 (-3.9–2.1)
15	Alzheimer's disease and other dementias	1.7 (1.3–2.1)	0.0232 (0.0159–0.0303)	147.2 (101.0–191.8)	106.7 (96.9–117.6)	-7.8 (-12.2–-2.9)
16	Stroke	1.7 (1.4–2.0)	0.0229 (0.0166–0.0294)	169.3 (122.9–217.6)	81.4 (67.2–96.6)	-5.8 (-12.9–1.5)
17	Schizophrenia	1.6 (1.2–2.1)	0.0218 (0.0159–0.0278)	221.9 (162.3–284.3)	27.5 (17.6–38.1)	-6.0 (-12.7–1.8)
18	Gynecological diseases	1.6 (1.3–1.9)	0.0216 (0.0150–0.0301)	238.7 (164.8–335.8)	7.0 (-1.7–16.5)	-14.8 (-22.1–-7.3)
19	Alcohol use disorders	1.4 (1.2–1.7)	0.0194 (0.0135–0.0275)	208.8 (144.0–294.8)	-0.9 (-10.7–9.9)	-23.6 (-30.1–-15.8)
20	Road injuries	1.3 (1.1–1.5)	0.0174 (0.0124–0.0234)	152.5 (109.3–206.2)	-4.9 (-8.2–-1.4)	-41.3 (-42.9–-39.5)
21	Autism spectrum disorders	1.2 (0.8–1.8)	0.0166 (0.0113–0.0235)	203.0 (138.3–287.2)	34.7 (23.5–46.1)	2.3 (-6.2–10.9)
22	Chronic kidney disease	1.2 (1.0–1.4)	0.0161 (0.0114–0.0209)	123.5 (86.8–158.4)	130.7 (110.6–153.6)	24.7 (14.9–35.7)
23	Blindness and vision loss	1.1 (0.8–1.6)	0.0156 (0.00994–0.0239)	131.0 (81.9–202.3)	78.8 (66.8–92.1)	2.3 (-3.8–10.3)
24	Dermatitis	1.1 (0.7–1.6)	0.0151 (0.00890–0.0238)	189.1 (108.9–304.5)	22.3 (16.3–28.0)	-8.0 (-12.7–-3.4)
25	Neonatal disorders	1.1 (0.9–1.4)	0.0151 (0.0108–0.0200)	196.2 (139.9–261.0)	16.9 (-3.5–42.1)	-7.4 (-23.4–13.8)

Table S3: Cause-specific YLDs by US state in 2021 and percentage change between 1990 and 2021, Washington						
Rank	Cause Name	2021 Percentage of all cause YLDs	2021 YLDs (millions)	2021 Age Standardised Rate (per 100 000)	Percentage change YLD count 1990 to 2021	Percentage change age-standardised YLD rate 1990 to 2021
	All causes	100.0 (100.0–100.0)	1.24 (0.937–1.58)	13066.1 (9859.3–16688.1)	97.3 (91.6–104.0)	12.5 (8.8–16.8)
1	Low back pain	9.0 (7.9–10.2)	0.112 (0.0802–0.145)	1136.6 (824.5–1486.8)	62.6 (51.8–74.1)	-9.3 (-14.6–-3.1)
2	Other musculoskeletal disorders	8.6 (6.8–10.6)	0.106 (0.0750–0.142)	1165.6 (824.2–1556.8)	183.8 (155.4–217.4)	67.6 (50.4–87.6)
3	Depressive disorders	6.9 (5.1–8.8)	0.0852 (0.0544–0.122)	1076.1 (679.9–1551.6)	120.6 (77.9–170.1)	47.5 (18.9–79.8)
4	Drug use disorders	5.9 (4.5–7.4)	0.0722 (0.0506–0.0929)	946.3 (659.6–1214.8)	365.8 (308.2–434.5)	224.7 (182.8–275.1)
5	Diabetes mellitus	5.8 (5.0–6.5)	0.0716 (0.0501–0.0971)	579.3 (407.5–777.4)	411.8 (368.1–455.8)	132.3 (113.6–151.5)
6	Anxiety disorders	5.2 (3.7–7.0)	0.0638 (0.0407–0.0920)	773.7 (483.3–1120.3)	97.3 (50.5–151.0)	27.5 (-2.5–62.5)
7	Headache disorders	4.5 (1.0–8.6)	0.0559 (0.0126–0.117)	682.9 (137.5–1447.0)	50.1 (41.8–67.7)	-3.0 (-7.8–3.2)
8	Age-related and other hearing loss	4.2 (3.3–5.2)	0.0523 (0.0366–0.0721)	419.9 (293.7–575.4)	102.6 (94.8–111.0)	-5.5 (-9.0–-1.9)
9	Falls	3.6 (3.0–4.3)	0.0451 (0.0315–0.0627)	362.7 (250.9–499.4)	115.8 (99.5–130.1)	-2.0 (-8.2–3.5)
10	Osteoarthritis	3.2 (1.8–5.9)	0.0394 (0.0192–0.0793)	304.4 (147.4–610.2)	142.7 (135.7–150.4)	6.8 (3.7–10.3)
11	Oral disorders	2.3 (1.6–3.2)	0.0288 (0.0176–0.0416)	263.8 (162.7–385.1)	79.4 (48.3–120.9)	-8.4 (-23.6–10.6)
12	Asthma	2.3 (1.8–2.9)	0.0284 (0.0184–0.0413)	361.1 (234.6–536.3)	58.3 (46.3–71.1)	-2.5 (-9.4–5.1)
13	Chronic obstructive pulmonary disease	2.1 (1.7–2.7)	0.0256 (0.0219–0.0297)	187.1 (160.3–216.0)	123.2 (109.1–142.0)	-3.7 (-10.1–4.5)
14	Neck pain	1.7 (1.4–2.2)	0.0217 (0.0146–0.0307)	237.2 (158.2–344.8)	67.1 (56.5–78.2)	-0.9 (-3.5–2.1)
15	Alzheimer's disease and other dementias	1.7 (1.3–2.1)	0.0210 (0.0146–0.0281)	146.2 (101.3–194.8)	113.2 (101.9–125.0)	-8.0 (-12.6–-3.1)
16	Schizophrenia	1.6 (1.2–2.1)	0.0196 (0.0143–0.0251)	222.8 (163.0–283.2)	49.0 (38.1–61.8)	-5.5 (-12.1–2.1)
17	Gynecological diseases	1.6 (1.3–1.9)	0.0194 (0.0134–0.0269)	239.0 (164.2–335.6)	24.8 (15.6–34.8)	-14.6 (-21.9–-7.9)
18	Stroke	1.5 (1.3–1.8)	0.0188 (0.0137–0.0240)	151.9 (109.8–192.9)	99.7 (85.5–117.7)	-5.6 (-12.0–2.5)
19	Alcohol use disorders	1.4 (1.2–1.7)	0.0174 (0.0122–0.0247)	209.7 (145.5–297.6)	20.3 (8.6–34.0)	-21.5 (-28.0–-13.9)
20	Autism spectrum disorders	1.3 (0.8–1.8)	0.0152 (0.0106–0.0213)	205.9 (143.1–288.9)	54.6 (41.3–67.8)	2.4 (-6.4–11.0)
21	Road injuries	1.2 (1.0–1.3)	0.0143 (0.0102–0.0191)	138.4 (98.9–185.2)	-2.5 (-5.9–1.4)	-47.9 (-49.5–-46.1)
22	Blindness and vision loss	1.1 (0.8–1.6)	0.0141 (0.00897–0.0215)	131.4 (81.9–202.9)	101.7 (88.5–117.2)	3.2 (-3.1–10.6)
23	Dermatitis	1.0 (0.7–1.5)	0.0127 (0.00742–0.0202)	176.7 (100.9–283.6)	36.8 (30.0–42.6)	-8.7 (-14.2–-4.0)
24	Chronic kidney disease	1.0 (0.8–1.2)	0.0126 (0.00896–0.0164)	104.8 (74.4–134.7)	158.9 (134.0–185.8)	25.1 (15.1–36.4)
25	Neonatal disorders	1.0 (0.8–1.2)	0.0123 (0.00882–0.0166)	177.6 (126.0–239.5)	-0.5 (-17.2–22.7)	-31.4 (-42.8–-15.7)

Table S3: Cause-specific YLDs by US state in 2021 and percentage change between 1990 and 2021, West Virginia						
**Rank**	**Cause Name**	**2021 Percentage of all cause YLDs**	**2021 YLDs (millions)**	**2021 Age Standardised Rate (per 100 000)**	**Percentage change YLD count 1990 to 2021**	**Percentage change age-standardised YLD rate 1990 to 2021**
	All causes	100.0 (100.0–100.0)	0.333 (0.255–0.422)	14832.9 (11226.9–18882.5)	33.5 (28.7–39.3)	25.8 (20.0–32.9)
1	Drug use disorders	9.6 (7.3–11.8)	0.0317 (0.0226–0.0405)	1949.8 (1374.5–2495.8)	508.1 (437.9–588.4)	590.7 (507.6–685.8)
2	Low back pain	8.9 (8.0–10.0)	0.0297 (0.0218–0.0387)	1273.9 (930.6–1659.7)	4.4 (-3.0–12.6)	-4.7 (-10.5–3.0)
3	Diabetes mellitus	7.8 (6.7–8.8)	0.0259 (0.0183–0.0349)	806.5 (569.0–1088.4)	227.3 (202.8–252.9)	150.8 (132.3–168.7)
4	Depressive disorders	6.7 (5.1–8.7)	0.0224 (0.0146–0.0316)	1268.1 (820.2–1793.0)	52.9 (23.3–87.0)	68.7 (37.0–106.3)
5	Other musculoskeletal disorders	5.8 (4.6–7.2)	0.0194 (0.0139–0.0263)	890.8 (641.0–1202.1)	49.6 (34.0–70.9)	44.4 (28.5–63.5)
6	Anxiety disorders	4.6 (3.3–6.3)	0.0153 (0.00939–0.0224)	834.9 (512.5–1220.9)	27.3 (-1.6–60.4)	36.7 (5.9–71.9)
7	Age-related and other hearing loss	4.1 (3.3–5.1)	0.0137 (0.00958–0.0190)	418.5 (291.6–579.1)	21.8 (17.4–26.5)	-5.5 (-8.6–-1.7)
8	Headache disorders	3.7 (0.9–7.2)	0.0123 (0.00275–0.0260)	674.5 (134.5–1416.9)	-10.0 (-15.4–1.2)	-5.0 (-10.2–0.9)
9	Osteoarthritis	3.3 (2.0–6.1)	0.0112 (0.00545–0.0229)	333.0 (160.2–675.9)	38.5 (34.7–43.0)	4.6 (1.7–7.9)
10	Falls	3.3 (2.7–4.0)	0.0110 (0.00775–0.0154)	338.3 (235.3–470.6)	50.1 (38.2–60.5)	7.8 (-0.1–15.0)
11	Chronic obstructive pulmonary disease	2.7 (2.2–3.4)	0.00900 (0.00780–0.0103)	256.8 (222.2–292.0)	40.4 (31.4–51.7)	5.2 (-1.1–13.8)
12	Oral disorders	2.6 (1.9–3.4)	0.00872 (0.00543–0.0122)	314.7 (197.7–453.0)	17.9 (-3.0–45.1)	0.3 (-15.4–21.7)
13	Asthma	2.2 (1.7–2.8)	0.00727 (0.00480–0.0105)	424.0 (277.1–625.8)	6.7 (-1.1–16.6)	9.8 (1.8–19.5)
14	Alzheimer's disease and other dementias	1.8 (1.4–2.3)	0.00603 (0.00414–0.00806)	147.1 (101.2–195.5)	31.7 (24.7–38.1)	-7.6 (-12.0–-3.3)
15	Stroke	1.8 (1.5–2.1)	0.00600 (0.00437–0.00768)	185.1 (133.8–235.7)	34.2 (24.3–46.9)	4.4 (-3.4–13.6)
16	Neck pain	1.5 (1.2–1.9)	0.00499 (0.00338–0.00698)	234.7 (156.7–338.5)	1.4 (-4.6–7.4)	-2.2 (-5.1–0.8)
17	Road injuries	1.3 (1.1–1.5)	0.00444 (0.00317–0.00598)	179.5 (129.5–241.9)	-27.6 (-30.1–-24.9)	-37.4 (-39.4–-35.4)
18	Schizophrenia	1.3 (1.0–1.7)	0.00424 (0.00311–0.00539)	215.3 (158.7–275.1)	-8.9 (-15.5–-1.6)	-7.3 (-14.6–0.2)
19	Gynecological diseases	1.3 (1.0–1.5)	0.00418 (0.00292–0.00575)	236.9 (164.9–332.2)	-28.3 (-33.3–-22.6)	-19.2 (-25.5–-12.5)
20	Chronic kidney disease	1.2 (1.0–1.4)	0.00385 (0.00274–0.00493)	124.6 (88.1–158.3)	66.9 (51.5–84.9)	32.7 (21.3–45.1)
21	Alcohol use disorders	1.1 (0.9–1.4)	0.00381 (0.00262–0.00523)	206.1 (143.7–287.1)	-23.6 (-30.9–-15.4)	-20.7 (-27.7–-12.9)
22	Blindness and vision loss	1.1 (0.7–1.5)	0.00356 (0.00233–0.00535)	130.9 (82.9–203.2)	20.7 (14.0–28.9)	1.1 (-4.7–8.7)
23	Atrial fibrillation and flutter	1.0 (0.8–1.2)	0.00329 (0.00231–0.00432)	84.3 (59.3–111.2)	69.1 (32.6–115.7)	22.3 (-3.5–54.0)
24	Autism spectrum disorders	1.0 (0.6–1.5)	0.00323 (0.00222–0.00451)	197.2 (136.2–276.5)	-4.9 (-13.2–4.3)	1.7 (-7.4–11.9)
25	Bipolar disorder	1.0 (0.7–1.3)	0.00321 (0.00212–0.00452)	170.4 (112.2–240.1)	-8.8 (-14.0–-2.7)	-5.6 (-11.3–1.4)

Table S3: Cause-specific YLDs by US state in 2021 and percentage change between 1990 and 2021, Wisconsin						
**Rank**	**Cause Name**	**2021 Percentage of all cause YLDs**	**2021 YLDs (millions)**	**2021 Age Standardised Rate (per 100 000)**	**Percentage change YLD count 1990 to 2021**	**Percentage change age-standardised YLD rate 1990 to 2021**
	All causes	100.0 (100.0–100.0)	0.955 (0.721–1.22)	13133.1 (9880.8–16852.1)	49.6 (45.0–55.0)	13.6 (9.6–18.1)
1	Low back pain	9.6 (8.5–10.8)	0.0917 (0.0670–0.119)	1243.1 (912.2–1621.0)	22.2 (14.1–31.0)	-8.5 (-14.3–-2.0)
2	Other musculoskeletal disorders	6.5 (5.1–8.1)	0.0622 (0.0435–0.0829)	907.3 (640.6–1209.4)	83.1 (62.6–105.9)	43.6 (28.4–61.2)
3	Diabetes mellitus	6.2 (5.4–7.1)	0.0595 (0.0419–0.0811)	601.0 (423.0–812.5)	240.4 (213.7–272.9)	106.6 (90.7–124.1)
4	Depressive disorders	6.2 (4.7–8.1)	0.0591 (0.0404–0.0851)	997.6 (684.4–1439.3)	63.5 (34.0–99.7)	44.8 (18.6–77.6)
5	Drug use disorders	6.2 (4.7–7.8)	0.0589 (0.0402–0.0754)	1070.6 (727.4–1378.3)	320.8 (270.3–383.5)	298.6 (249.3–360.0)
6	Anxiety disorders	4.9 (3.5–6.7)	0.0468 (0.0306–0.0680)	757.3 (493.9–1104.4)	44.8 (12.3–86.7)	23.7 (-4.1–59.9)
7	Falls	4.4 (3.7–5.2)	0.0420 (0.0295–0.0584)	417.9 (292.5–574.8)	91.0 (77.4–102.9)	14.1 (7.0–20.8)
8	Headache disorders	4.3 (1.0–8.4)	0.0418 (0.00933–0.0876)	683.5 (136.0–1445.1)	13.6 (7.4–27.5)	-3.4 (-7.8–2.2)
9	Age-related and other hearing loss	4.4 (3.5–5.3)	0.0416 (0.0291–0.0575)	419.9 (291.7–582.0)	48.1 (42.4–54.3)	-5.1 (-8.2–-1.8)
10	Osteoarthritis	3.3 (1.9–6.2)	0.0321 (0.0155–0.0647)	312.7 (150.7–629.7)	76.9 (71.9–82.2)	6.3 (3.6–9.5)
11	Chronic obstructive pulmonary disease	2.4 (1.9–3.0)	0.0225 (0.0191–0.0260)	206.3 (175.0–237.8)	76.7 (63.5–92.5)	5.6 (-2.0–14.7)
12	Oral disorders	2.3 (1.6–3.0)	0.0216 (0.0134–0.0314)	252.6 (157.0–370.3)	32.8 (6.6–64.8)	-8.8 (-26.2–11.8)
13	Asthma	2.2 (1.7–2.8)	0.0214 (0.0139–0.0312)	362.4 (233.5–534.1)	20.9 (10.6–31.4)	0.4 (-7.5–9.1)
14	Alzheimer's disease and other dementias	1.9 (1.5–2.4)	0.0180 (0.0125–0.0238)	150.0 (103.1–198.3)	48.6 (41.8–56.1)	-8.0 (-11.8–-3.4)
15	Neck pain	1.7 (1.3–2.2)	0.0164 (0.0110–0.0231)	237.3 (157.9–342.1)	26.6 (18.6–35.6)	-1.1 (-4.0–1.9)
16	Schizophrenia	1.5 (1.1–2.0)	0.0145 (0.0107–0.0186)	222.4 (165.3–285.8)	14.1 (5.7–23.0)	-5.3 (-12.0–1.7)
17	Alcohol use disorders	1.4 (1.2–1.8)	0.0139 (0.00941–0.0193)	224.1 (150.4–314.9)	-5.9 (-14.7–4.1)	-19.3 (-26.1–-11.8)
18	Stroke	1.4 (1.2–1.7)	0.0134 (0.00961–0.0172)	136.3 (97.4–175.1)	42.8 (32.5–56.0)	-6.7 (-13.2–1.3)
19	Road injuries	1.4 (1.2–1.6)	0.0132 (0.00935–0.0177)	167.3 (119.6–224.7)	-8.2 (-10.9–-5.2)	-35.2 (-36.8–-33.4)
20	Gynecological diseases	1.4 (1.1–1.6)	0.0129 (0.00889–0.0181)	218.5 (149.0–313.1)	-10.6 (-18.1–-3.5)	-18.8 (-26.1–-11.5)
21	Autism spectrum disorders	1.2 (0.8–1.8)	0.0114 (0.00798–0.0158)	205.2 (143.7–284.9)	16.3 (5.5–25.7)	1.8 (-7.7–10.0)
22	Blindness and vision loss	1.2 (0.8–1.7)	0.0112 (0.00719–0.0169)	131.9 (82.4–202.6)	50.4 (40.8–61.9)	3.4 (-2.8–9.7)
23	Chronic kidney disease	1.1 (0.9–1.4)	0.0108 (0.00756–0.0142)	113.3 (79.6–147.1)	87.9 (71.1–106.9)	24.7 (14.5–37.0)
24	COVID-19	1.1 (0.4–2.4)	0.0103 (0.00398–0.0239)	157.4 (57.7–375.8)	0.0 (0.0–0.0)	0.0 (0.0–0.0)
25	Neonatal disorders	1.1 (0.9–1.4)	0.0103 (0.00713–0.0139)	199.0 (136.9–270.7)	18.6 (-4.6–46.3)	8.2 (-13.0–33.7)

Table S3: Cause-specific YLDs by US state in 2021 and percentage change between 1990 and 2021, Wyoming						
Rank	Cause Name	2021 Percentage of all cause YLDs	2021 YLDs (millions)	2021 Age Standardised Rate (per 100 000)	Percentage change YLD count 1990 to 2021	Percentage change age-standardised YLD rate 1990 to 2021
	All causes	100.0 (100.0–100.0)	0.0928 (0.0706–0.119)	13397.3 (10078.5–17225.3)	66.0 (60.3–72.8)	15.9 (11.7–21.0)
1	Low back pain	10.5 (9.2–11.9)	0.00975 (0.00705–0.0128)	1393.9 (1011.7–1824.0)	42.5 (32.5–53.3)	0.0 (-6.1–7.3)
2	Other musculoskeletal disorders	7.4 (5.8–9.1)	0.00684 (0.00485–0.00916)	1036.6 (740.6–1395.3)	121.6 (96.8–151.2)	61.6 (43.2–82.9)
3	Depressive disorders	6.8 (5.3–8.9)	0.00636 (0.00432–0.00931)	1102.7 (746.6–1611.8)	82.9 (50.1–120.2)	51.4 (23.2–82.5)
4	Drug use disorders	5.6 (4.3–7.0)	0.00515 (0.00358–0.00665)	959.6 (665.2–1246.2)	396.1 (339.4–462.6)	334.3 (281.3–392.5)
5	Diabetes mellitus	5.1 (4.4–5.8)	0.00477 (0.00339–0.00647)	515.8 (366.4–692.3)	295.3 (262.9–333.6)	110.1 (93.6–129.0)
6	Anxiety disorders	5.0 (3.5–6.9)	0.00467 (0.00286–0.00667)	782.3 (477.8–1121.3)	59.6 (24.9–98.3)	28.9 (1.5–60.0)
7	Headache disorders	4.3 (1.0–8.4)	0.00399 (0.000890–0.00840)	672.2 (134.3–1420.8)	18.1 (11.8–32.2)	-4.4 (-9.1–1.3)
8	Age-related and other hearing loss	4.2 (3.4–5.2)	0.00394 (0.00275–0.00544)	423.3 (297.5–584.4)	76.2 (69.2–83.9)	-5.2 (-8.4–-2.1)
9	Falls	3.6 (3.0–4.2)	0.00333 (0.00235–0.00465)	368.2 (257.3–509.5)	101.6 (86.1–116.2)	10.1 (3.3–16.5)
10	Osteoarthritis	3.1 (1.8–5.8)	0.00288 (0.00140–0.00586)	300.0 (144.9–605.9)	106.6 (100.0–113.5)	6.2 (3.0–9.7)
11	Chronic obstructive pulmonary disease	2.6 (2.1–3.3)	0.00242 (0.00208–0.00275)	242.8 (209.7–274.9)	114.9 (100.7–131.3)	8.4 (1.1–16.6)
12	Oral disorders	2.4 (1.7–3.3)	0.00227 (0.00141–0.00330)	281.8 (174.2–413.5)	58.6 (33.0–91.1)	-3.3 (-18.2–15.4)
13	Asthma	2.2 (1.7–2.8)	0.00208 (0.00137–0.00304)	361.0 (233.3–541.4)	19.3 (8.2–30.4)	-5.7 (-13.0–2.4)
14	Alzheimer's disease and other dementias	1.8 (1.4–2.2)	0.00163 (0.00114–0.00217)	148.9 (103.2–197.1)	98.4 (88.3–108.1)	-7.8 (-12.1–-3.3)
15	Road injuries	1.7 (1.5–2.0)	0.00160 (0.00115–0.00217)	215.5 (155.3–291.2)	-1.8 (-5.1–1.8)	-35.2 (-37.1–-33.3)
16	Alcohol use disorders	1.7 (1.4–2.1)	0.00159 (0.00111–0.00222)	267.3 (185.9–376.6)	2.0 (-7.7–13.1)	-17.2 (-24.3–-8.6)
17	Neck pain	1.7 (1.3–2.1)	0.00156 (0.00106–0.00218)	235.5 (157.7–337.9)	34.4 (25.0–44.2)	-1.5 (-4.2–1.3)
18	Schizophrenia	1.5 (1.1–2.0)	0.00139 (0.00103–0.00174)	220.2 (161.9–280.2)	19.0 (10.7–27.4)	-6.1 (-12.3–0.2)
19	Stroke	1.5 (1.2–1.7)	0.00137 (0.000975–0.00174)	147.7 (105.3–188.3)	69.0 (56.3–83.3)	-8.6 (-15.2–-1.1)
20	Gynecological diseases	1.3 (1.1–1.6)	0.00121 (0.000827–0.00169)	210.0 (144.2–294.6)	-8.4 (-16.5–-0.7)	-20.7 (-28.0–-13.0)
21	COVID-19	1.3 (0.5–3.3)	0.00120 (0.000421–0.00297)	192.6 (65.1–489.6)	0.0 (0.0–0.0)	0.0 (0.0–0.0)
22	Autism spectrum disorders	1.2 (0.8–1.8)	0.00111 (0.000756–0.00156)	202.8 (138.5–285.5)	22.2 (12.4–32.1)	2.6 (-5.6–10.8)
23	Blindness and vision loss	1.1 (0.8–1.6)	0.00105 (0.000680–0.00158)	132.6 (84.3–202.1)	67.5 (57.3–77.9)	2.3 (-3.3–9.2)
24	Other cardiovascular and circulatory diseases	1.1 (0.8–1.5)	0.00104 (0.000649–0.00162)	122.1 (77.8–188.7)	151.8 (110.1–206.2)	45.3 (23.6–72.2)
25	Chronic kidney disease	1.0 (0.8–1.2)	0.000924 (0.000647–0.00121)	102.7 (72.9–133.1)	132.8 (108.8–161.1)	30.0 (18.4–44.4)

Table S4: Cause-specific DALYs for the 25 leading Level 3 causes by US state in 2021 and percentage change between 1990 and 2021

Table S4: Cause-specific DALYs by US state in 2021 and percentage change between 1990 and 2021, Alabama						
Rank	Cause Name	2021 Percentage of all cause DALYs	2021 DALYs (millions)	2021 Age Standardised Rate (per 100 000)	Percentage change DALY count 1990 to 2021	Percentage change age-standardised DALY rate 1990 to 2021
	All causes	100.0 (100.0–100.0)	2.37 (2.09–2.64)	35359.1 (31328.3–39703.5)	58.8 (47.1–71.5)	10.7 (3.3–19.1)
1	COVID-19	11.5 (10.0–13.7)	0.272 (0.256–0.314)	3821.9 (3572.2–4440.2)	--	--
2	Ischaemic heart disease	7.2 (6.4–8.2)	0.171 (0.146–0.197)	2039.1 (1747.6–2361.1)	-10.3 (-22.2–3.6)	-43.8 (-51.4—34.8)
3	Drug use disorders	4.6 (4.0–5.3)	0.109 (0.0889–0.131)	2211.3 (1802.3–2643.5)	794.8 (675.6–956.9)	688.1 (584.0–828.3)
4	Chronic obstructive pulmonary disease	4.5 (4.0–5.0)	0.107 (0.0948–0.120)	1187.9 (1050.7–1330.4)	132.7 (109.2–160.5)	39.6 (25.6–56.2)
5	Stroke	3.8 (3.5–4.2)	0.0910 (0.0789–0.103)	1090.2 (944.8–1236.3)	34.1 (18.7–50.7)	-15.9 (-25.6—5.4)
6	Diabetes mellitus	3.7 (3.2–4.2)	0.0877 (0.0696–0.109)	1110.3 (888.6–1381.5)	135.9 (111.6–159.6)	51.3 (35.9–66.3)
7	Low back pain	3.5 (2.7–4.3)	0.0828 (0.0598–0.108)	1332.0 (954.4–1728.7)	31.6 (23.6–40.3)	-2.5 (-8.0–3.6)
8	Tracheal, bronchus, and lung cancer	3.4 (3.0–3.9)	0.0805 (0.0688–0.0948)	918.3 (785.4–1085.9)	6.2 (-9.9–25.2)	-38.5 (-48.0—27.2)
9	Chronic kidney disease	2.6 (2.3–2.9)	0.0612 (0.0533–0.0700)	754.5 (660.5–861.3)	231.4 (192.7–276.4)	105.1 (82.4–132.1)
10	Road injuries	2.5 (2.4–2.7)	0.0603 (0.0527–0.0686)	1148.1 (1012.3–1301.4)	-25.3 (-34.3—16.0)	-39.6 (-46.9—31.8)
11	Other musculoskeletal disorders	2.5 (1.9–3.2)	0.0597 (0.0432–0.0792)	993.6 (722.2–1329.4)	98.6 (77.3–122.1)	51.5 (34.4–70.0)
12	Depressive disorders	2.2 (1.5–3.0)	0.0522 (0.0337–0.0742)	1031.0 (668.8–1475.9)	85.8 (52.6–123.2)	61.0 (31.7–93.7)
13	Alzheimer's disease and other dementias	2.0 (0.9–4.3)	0.0470 (0.0219–0.102)	491.5 (229.8–1067.0)	65.2 (51.6–80.9)	-4.1 (-11.7–4.9)
14	Anxiety disorders	1.9 (1.3–2.6)	0.0440 (0.0285–0.0636)	839.7 (549.7–1239.7)	63.1 (26.9–108.0)	36.9 (6.5–74.1)
15	Cirrhosis and other chronic liver diseases	1.6 (1.4–1.8)	0.0379 (0.0319–0.0442)	530.1 (447.6–618.5)	98.6 (69.4–131.8)	30.5 (11.5–52.7)
16	Self-harm	1.6 (1.4–1.8)	0.0367 (0.0311–0.0425)	704.9 (599.3–813.2)	47.2 (24.3–72.3)	24.5 (5.5–45.0)
17	Headache disorders	1.5 (0.3–3.0)	0.0358 (0.00795–0.0748)	684.3 (137.6–1447.3)	15.6 (9.4–28.5)	-3.9 (-8.4–1.5)
18	Falls	1.5 (1.3–1.7)	0.0348 (0.0274–0.0445)	440.4 (347.9–557.7)	69.7 (57.6–83.8)	4.4 (-2.7–12.8)
19	Age-related and other hearing loss	1.4 (1.0–1.9)	0.0341 (0.0240–0.0476)	419.0 (294.5–583.9)	49.1 (43.7–55.0)	-5.3 (-8.4—1.8)
20	Colon and rectum cancer	1.4 (1.2–1.6)	0.0329 (0.0284–0.0387)	407.8 (350.7–482.0)	30.2 (9.8–53.3)	-16.5 (-29.9—1.4)
21	Interpersonal violence	1.3 (1.2–1.5)	0.0315 (0.0271–0.0361)	685.0 (593.1–780.7)	-9.3 (-21.3–4.6)	-17.1 (-27.8—4.9)
22	Hypertensive heart disease	1.2 (1.1–1.4)	0.0290 (0.0245–0.0335)	367.4 (311.2–424.8)	158.4 (120.4–200.8)	67.9 (42.5–96.4)
23	Neonatal disorders	1.1 (1.0–1.3)	0.0269 (0.0234–0.0306)	825.8 (728.9–930.7)	-34.8 (-41.8—26.3)	-36.0 (-43.0—27.6)
24	Osteoarthritis	1.1 (0.6–2.2)	0.0264 (0.0128–0.0535)	313.8 (151.0–633.2)	74.0 (68.8–79.0)	6.1 (2.8–9.0)
25	Breast cancer	1.1 (0.9–1.3)	0.0262 (0.0209–0.0322)	337.4 (268.2–416.3)	4.6 (-16.3–27.6)	-34.7 (-48.1—20.0)

Table S4: Cause-specific DALYs by US state in 2021 and percentage change between 1990 and 2021, Alaska						
Rank	Cause Name	2021 Percentage of all cause DALYs	2021 DALYs (millions)	2021 Age Standardised Rate (per 100 000)	Percentage change DALY count 1990 to 2021	Percentage change age-standardised DALY rate 1990 to 2021
	All causes	100.0 (100.0–100.0)	0.268 (0.238–0.301)	31270.4 (27632.6–35282.6)	91.9 (81.6–104.3)	4.0 (-1.8–10.0)
1	COVID-19	10.9 (8.6–13.1)	0.0293 (0.0237–0.0347)	3177.7 (2565.7–3773.3)	--	--
2	Drug use disorders	5.7 (4.9–6.5)	0.0153 (0.0126–0.0183)	2086.0 (1711.9–2497.3)	549.8 (466.3–667.8)	472.8 (396.7–575.6)
3	Ischaemic heart disease	4.8 (4.2–5.6)	0.0129 (0.0113–0.0149)	1252.0 (1089.8–1442.3)	41.7 (25.0–62.2)	-57.0 (-62.0–-51.1)
4	Low back pain	4.1 (3.2–4.9)	0.0110 (0.00790–0.0144)	1261.5 (913.2–1645.0)	50.5 (38.1–64.6)	-5.7 (-11.3–0.4)
5	Diabetes mellitus	3.4 (2.9–3.9)	0.00912 (0.00724–0.0115)	890.5 (715.4–1116.4)	289.3 (248.9–327.6)	43.1 (27.8–57.3)
6	Self-harm	3.4 (3.0–3.9)	0.00903 (0.00785–0.0103)	1244.4 (1082.2–1420.4)	74.3 (50.3–101.6)	41.8 (22.3–63.7)
7	Other musculoskeletal disorders	3.2 (2.5–4.1)	0.00872 (0.00630–0.0115)	1047.9 (750.7–1389.0)	147.1 (118.3–178.8)	59.8 (42.2–78.8)
8	Chronic obstructive pulmonary disease	2.8 (2.4–3.1)	0.00743 (0.00667–0.00837)	696.3 (624.2–781.0)	215.9 (185.4–252.0)	-13.6 (-21.6–-4.3)
9	Depressive disorders	2.7 (2.0–3.7)	0.00720 (0.00483–0.0103)	959.9 (639.9–1376.5)	80.4 (46.1–118.7)	42.4 (16.0–74.1)
10	Tracheal, bronchus, and lung cancer	2.6 (2.2–2.9)	0.00693 (0.00600–0.00788)	636.5 (552.4–719.6)	58.9 (38.2–82.8)	-50.1 (-56.4–-42.7)
11	Alcohol use disorders	2.5 (2.3–2.7)	0.00667 (0.00573–0.00779)	808.7 (692.2–961.2)	64.2 (44.8–91.2)	21.0 (8.7–39.0)
12	Stroke	2.5 (2.2–2.7)	0.00663 (0.00588–0.00739)	684.0 (608.1–762.0)	109.7 (91.6–134.3)	-31.4 (-37.2–-24.5)
13	Anxiety disorders	2.1 (1.5–3.1)	0.00570 (0.00373–0.00853)	737.4 (483.2–1110.6)	61.5 (26.7–105.8)	23.6 (-2.6–57.1)
14	Falls	2.0 (1.7–2.4)	0.00542 (0.00430–0.00684)	568.7 (454.6–710.1)	120.9 (100.5–144.5)	4.1 (-2.7–12.7)
15	Headache disorders	1.9 (0.4–3.8)	0.00515 (0.00111–0.0109)	670.0 (132.5–1415.2)	23.9 (15.9–43.8)	-2.9 (-7.5–2.6)
16	Road injuries	1.9 (1.8–2.1)	0.00512 (0.00452–0.00576)	696.0 (615.3–785.4)	-31.9 (-37.9–-23.9)	-47.1 (-52.0–-40.3)
17	Alzheimer's disease and other dementias	1.8 (0.9–3.8)	0.00486 (0.00228–0.0105)	503.7 (233.7–1084.2)	361.1 (325.2–404.3)	-3.8 (-10.4–4.0)
18	Cirrhosis and other chronic liver diseases	1.8 (1.6–2.1)	0.00481 (0.00427–0.00549)	512.0 (454.4–586.4)	134.8 (106.9–168.1)	18.0 (4.0–34.2)
19	Age-related and other hearing loss	1.6 (1.2–2.1)	0.00425 (0.00296–0.00585)	425.4 (299.2–582.4)	151.5 (135.5–171.0)	-5.9 (-9.4–-2.2)
20	Chronic kidney disease	1.6 (1.4–1.8)	0.00423 (0.00374–0.00476)	439.6 (388.9–492.1)	407.6 (348.6–482.3)	94.4 (77.1–119.8)
21	Osteoarthritis	1.2 (0.6–2.3)	0.00322 (0.00156–0.00654)	302.1 (145.4–613.1)	216.5 (201.3–232.1)	6.9 (3.8–10.1)
22	Colon and rectum cancer	1.2 (1.0–1.4)	0.00317 (0.00273–0.00369)	311.0 (268.3–359.0)	95.8 (67.1–129.8)	-34.6 (-43.8–-24.0)
23	Asthma	1.2 (0.9–1.6)	0.00317 (0.00221–0.00449)	437.4 (300.4–628.4)	36.8 (24.0–48.0)	-1.4 (-7.8–4.9)
24	Interpersonal violence	1.1 (1.0–1.2)	0.00291 (0.00258–0.00324)	428.6 (378.2–480.4)	-6.5 (-17.6–4.8)	-17.0 (-27.0–-6.8)
25	Neonatal disorders	1.0 (0.9–1.2)	0.00276 (0.00230–0.00325)	577.6 (489.8–679.8)	-41.1 (-50.3–-30.6)	-30.9 (-41.6–-18.2)

Table S4: Cause-specific DALYs by US state in 2021 and percentage change between 1990 and 2021, Arizona

Rank	Cause Name	2021 Percentage of all cause DALYs	2021 DALYs (millions)	2021 Age Standardised Rate (per 100 000)	Percentage change DALY count 1990 to 2021	Percentage change age-standardised DALY rate 1990 to 2021
	All causes	100.0 (100.0–100.0)	2.96 (2.59–3.34)	31028.1 (27055.8–35352.3)	149.0 (132.7–167.3)	8.6 (1.9–15.8)
1	COVID-19	12.1 (10.4–14.3)	0.356 (0.337–0.409)	3470.2 (3260.0–4000.1)	--	--
2	Ischaemic heart disease	6.0 (5.2–6.9)	0.176 (0.150–0.205)	1399.2 (1187.1–1631.8)	29.7 (11.6–50.3)	-52.5 (-59.3–-45.0)
3	Drug use disorders	5.4 (4.7–6.2)	0.159 (0.132–0.188)	2219.6 (1835.7–2626.0)	805.0 (673.6–965.0)	402.5 (331.2–487.6)
4	Low back pain	4.0 (3.2–4.8)	0.118 (0.0852–0.152)	1292.1 (938.8–1681.6)	114.0 (100.3–129.9)	-3.8 (-9.8–3.3)
5	Diabetes mellitus	3.8 (3.3–4.3)	0.112 (0.0890–0.141)	983.2 (776.3–1225.8)	349.6 (305.1–386.8)	74.7 (57.5–89.1)
6	Chronic obstructive pulmonary disease	3.5 (3.1–3.9)	0.104 (0.0923–0.117)	783.3 (693.2–882.7)	161.2 (134.6–190.2)	-4.8 (-14.4–5.7)
7	Other musculoskeletal disorders	3.1 (2.3–3.9)	0.0907 (0.0649–0.121)	1041.1 (745.9–1379.3)	215.9 (184.6–251.5)	46.4 (33.1–62.4)
8	Depressive disorders	2.9 (2.1–4.0)	0.0872 (0.0565–0.122)	1188.8 (771.8–1659.1)	204.6 (145.9–271.0)	62.7 (31.1–97.2)
9	Stroke	2.5 (2.2–2.8)	0.0745 (0.0647–0.0846)	605.2 (524.8–684.6)	105.1 (81.2–128.7)	-23.1 (-31.8–-14.3)
10	Alzheimer's disease and other dementias	2.5 (1.2–5.1)	0.0725 (0.0330–0.157)	505.4 (231.1–1085.9)	193.8 (167.4–219.0)	-2.9 (-11.0–4.7)
11	Tracheal, bronchus, and lung cancer	2.3 (2.0–2.6)	0.0678 (0.0576–0.0788)	531.1 (451.5–618.6)	37.1 (16.4–60.4)	-51.2 (-58.7–-42.6)
12	Self-harm	2.2 (1.9–2.6)	0.0658 (0.0560–0.0770)	865.5 (738.0–1009.7)	105.3 (74.0–141.0)	6.3 (-10.1–24.2)
13	Road injuries	2.2 (2.0–2.4)	0.0652 (0.0566–0.0746)	837.7 (730.5–950.8)	9.3 (-3.5–21.2)	-46.5 (-53.0–-40.4)
14	Falls	2.2 (1.9–2.5)	0.0647 (0.0523–0.0819)	550.3 (443.2–688.8)	235.6 (210.3–263.5)	22.3 (13.5–32.0)
15	Anxiety disorders	2.1 (1.5–3.0)	0.0631 (0.0408–0.0908)	833.8 (534.8–1204.9)	164.1 (103.4–233.7)	37.3 (6.1–74.3)
16	Chronic kidney disease	2.0 (1.8–2.2)	0.0586 (0.0509–0.0665)	494.2 (432.3–559.2)	479.2 (412.9–560.0)	116.0 (92.2–145.5)
17	Cirrhosis and other chronic liver diseases	1.8 (1.6–2.1)	0.0532 (0.0447–0.0630)	542.4 (457.6–641.4)	185.9 (140.9–237.3)	18.8 (0.4–40.1)
18	Headache disorders	1.7 (0.4–3.5)	0.0516 (0.0113–0.108)	682.6 (136.9–1437.3)	88.8 (78.7–107.1)	-3.1 (-7.8–2.4)
19	Age-related and other hearing loss	1.7 (1.2–2.1)	0.0492 (0.0345–0.0677)	418.0 (295.4–575.3)	141.2 (132.2–151.0)	-6.3 (-9.5–-3.0)
20	Alcohol use disorders	1.5 (1.3–1.7)	0.0434 (0.0353–0.0522)	539.1 (437.2–657.3)	109.2 (86.0–140.7)	3.4 (-6.9–18.3)
21	Osteoarthritis	1.3 (0.7–2.4)	0.0374 (0.0181–0.0759)	312.0 (150.4–628.4)	182.1 (173.8–192.3)	5.8 (2.5–9.6)
22	Colon and rectum cancer	1.2 (1.0–1.4)	0.0357 (0.0303–0.0424)	307.0 (259.8–364.6)	97.3 (64.0–138.0)	-22.0 (-35.5–-5.9)
23	Asthma	1.2 (0.9–1.6)	0.0347 (0.0239–0.0493)	484.9 (328.3–704.0)	84.1 (71.9–96.4)	-4.2 (-10.3–2.5)
24	Breast cancer	1.0 (0.8–1.2)	0.0299 (0.0240–0.0362)	270.4 (216.2–330.7)	63.6 (31.9–97.9)	-37.1 (-49.6–-23.3)
25	Endocrine, metabolic, blood, and immune disorders	1.0 (0.9–1.1)	0.0289 (0.0247–0.0337)	304.3 (260.0–355.4)	320.1 (258.7–392.7)	74.3 (51.9–101.6)

Table S4: Cause-specific DALYs by US state in 2021 and percentage change between 1990 and 2021, Arkansas						
Rank	Cause Name	2021 Percentage of all cause DALYs	2021 DALYs (millions)	2021 Age Standardised Rate (per 100 000)	Percentage change DALY count 1990 to 2021	Percentage change age-standardised DALY rate 1990 to 2021
	All causes	100.0 (100.0–100.0)	1.35 (1.18–1.50)	33866.4 (29589.5–38035.3)	53.6 (41.4–66.3)	10.0 (2.0–18.5)
1	COVID-19	10.2 (8.8–12.5)	0.138 (0.127–0.162)	3255.7 (2986.7–3816.3)	--	--
2	Ischaemic heart disease	9.2 (8.0–10.3)	0.124 (0.106–0.144)	2481.6 (2127.0–2892.2)	-2.3 (-15.6–13.7)	-33.6 (-42.8–-22.4)
3	Chronic obstructive pulmonary disease	4.6 (4.1–5.1)	0.0617 (0.0545–0.0688)	1150.3 (1018.6–1281.6)	119.4 (94.0–145.0)	43.6 (26.7–60.4)
4	Diabetes mellitus	3.8 (3.4–4.3)	0.0515 (0.0417–0.0627)	1103.2 (892.5–1339.8)	151.7 (128.9–175.7)	70.6 (55.2–86.9)
5	Tracheal, bronchus, and lung cancer	3.5 (3.0–4.1)	0.0477 (0.0405–0.0559)	924.7 (783.9–1085.0)	-1.8 (-17.5–15.9)	-40.2 (-49.9–-29.2)
6	Stroke	3.3 (3.0–3.6)	0.0451 (0.0386–0.0508)	914.7 (794.0–1032.2)	4.6 (-7.0–17.0)	-27.5 (-35.5–-19.0)
7	Low back pain	3.3 (2.6–4.0)	0.0440 (0.0321–0.0567)	1197.0 (870.7–1547.3)	26.5 (18.4–36.2)	-6.4 (-12.4–0.9)
8	Drug use disorders	3.2 (2.6–3.9)	0.0438 (0.0337–0.0537)	1512.8 (1156.5–1848.5)	531.9 (423.4–676.4)	426.0 (336.5–544.6)
9	Depressive disorders	2.8 (2.0–3.9)	0.0380 (0.0256–0.0542)	1258.0 (842.9–1791.4)	98.1 (63.6–138.9)	64.3 (36.0–98.2)
10	Road injuries	2.6 (2.4–2.8)	0.0347 (0.0304–0.0394)	1107.8 (975.4–1253.8)	-21.1 (-29.4–-11.8)	-39.2 (-45.8–-31.9)
11	Chronic kidney disease	2.5 (2.2–2.7)	0.0332 (0.0292–0.0376)	684.1 (600.8–775.4)	243.6 (197.9–286.8)	125.3 (96.6–153.0)
12	Alzheimer's disease and other dementias	2.2 (1.0–4.6)	0.0296 (0.0139–0.0623)	500.3 (235.3–1048.4)	48.7 (36.4–63.0)	-3.2 (-11.2–6.1)
13	Other musculoskeletal disorders	2.1 (1.6–2.7)	0.0284 (0.0204–0.0375)	803.2 (575.6–1068.3)	83.7 (62.1–108.8)	39.5 (22.9–58.4)
14	Self-harm	2.0 (1.8–2.3)	0.0276 (0.0233–0.0328)	908.7 (766.7–1074.8)	78.5 (51.0–111.5)	45.6 (23.3–72.1)
15	Anxiety disorders	1.9 (1.2–2.7)	0.0258 (0.0157–0.0375)	827.5 (502.3–1204.0)	66.0 (28.6–113.1)	34.7 (4.2–72.7)
16	Hypertensive heart disease	1.6 (1.4–1.8)	0.0218 (0.0184–0.0255)	457.7 (384.1–535.4)	363.2 (291.6–449.2)	216.1 (165.0–276.1)
17	Falls	1.6 (1.4–1.9)	0.0214 (0.0170–0.0274)	454.7 (363.6–579.3)	71.9 (59.8–87.3)	9.4 (1.5–19.2)
18	Headache disorders	1.6 (0.4–3.2)	0.0213 (0.00465–0.0451)	685.5 (136.5–1453.2)	20.8 (15.3–30.5)	-3.5 (-8.1–1.7)
19	Age-related and other hearing loss	1.5 (1.1–2.0)	0.0205 (0.0143–0.0284)	422.5 (295.7–589.1)	39.1 (34.0–44.8)	-5.5 (-8.7–-2.1)
20	Cirrhosis and other chronic liver diseases	1.5 (1.3–1.7)	0.0199 (0.0169–0.0235)	487.8 (413.1–574.0)	111.1 (77.2–149.3)	45.5 (22.1–70.7)
21	Colon and rectum cancer	1.4 (1.2–1.6)	0.0193 (0.0161–0.0227)	405.1 (337.7–475.7)	23.0 (3.0–45.6)	-15.6 (-29.9–0.4)
22	Interpersonal violence	1.2 (1.0–1.3)	0.0157 (0.0137–0.0180)	568.9 (494.0–650.3)	-8.8 (-21.8–4.6)	-22.4 (-33.8–-11.3)
23	Neonatal disorders	1.2 (1.0–1.4)	0.0157 (0.0133–0.0184)	800.7 (682.6–942.6)	-17.1 (-28.5–-4.3)	-22.1 (-32.8–-9.1)
24	Osteoarthritis	1.1 (0.6–2.1)	0.0146 (0.00710–0.0294)	293.1 (141.9–588.8)	64.2 (58.7–69.9)	7.1 (3.4–11.0)
25	Breast cancer	1.0 (0.8–1.2)	0.0138 (0.0111–0.0168)	304.4 (245.6–371.7)	2.3 (-18.4–24.1)	-32.9 (-46.6–-17.6)

Table S4: Cause-specific DALYs by US state in 2021 and percentage change between 1990 and 2021, California

Rank	Cause Name	2021 Percentage of all cause DALYs	2021 DALYs (millions)	2021 Age Standardised Rate (per 100 000)	Percentage change DALY count 1990 to 2021	Percentage change age-standardised DALY rate 1990 to 2021
	All causes	100.0 (100.0–100.0)	13.0 (11.3–14.6)	25116.9 (21783.8–28758.6)	42.5 (32.9–51.6)	-11.5 (-17.2–-5.9)
1	COVID-19	9.3 (8.1–10.6)	1.19 (1.16–1.31)	2191.0 (2103.5–2412.7)	--	--
2	Ischaemic heart disease	6.4 (5.4–7.5)	0.834 (0.709–0.955)	1271.3 (1088.3–1455.4)	-16.5 (-27.7—5.2)	-58.5 (-64.2—52.7)
3	Drug use disorders	4.2 (3.7–4.9)	0.550 (0.449–0.649)	1304.8 (1064.3–1542.8)	244.6 (204.9–295.0)	185.0 (153.0–224.4)
4	Low back pain	4.2 (3.4–5.1)	0.550 (0.402–0.713)	1104.0 (808.0–1447.2)	35.3 (25.5–45.8)	-11.6 (-17.5–-5.1)
5	Diabetes mellitus	3.8 (3.4–4.3)	0.495 (0.391–0.620)	820.3 (647.3–1025.9)	223.7 (193.6–254.2)	72.6 (56.3–88.8)
6	Other musculoskeletal disorders	3.4 (2.6–4.2)	0.436 (0.313–0.583)	903.0 (648.3–1205.1)	105.7 (84.7–132.2)	37.6 (23.2–54.5)
7	Stroke	3.1 (2.7–3.5)	0.405 (0.349–0.454)	630.0 (550.0–706.7)	30.1 (15.9–43.0)	-33.5 (-40.6–-26.9)
8	Alzheimer's disease and other dementias	3.1 (1.6–6.1)	0.398 (0.198–0.818)	541.3 (271.1–1112.3)	112.3 (96.5–128.9)	-3.9 (-10.8–3.1)
9	Chronic obstructive pulmonary disease	2.9 (2.5–3.3)	0.375 (0.331–0.416)	556.4 (492.7–616.6)	57.4 (42.8–71.1)	-22.4 (-29.4–-15.7)
10	Depressive disorders	2.8 (1.9–3.7)	0.365 (0.234–0.515)	892.0 (564.1–1263.8)	107.7 (66.4–152.8)	65.3 (34.1–103.2)
11	Anxiety disorders	2.7 (1.8–3.8)	0.350 (0.218–0.516)	827.2 (513.0–1232.3)	76.6 (36.3–126.9)	35.8 (4.6–73.9)
12	Chronic kidney disease	2.4 (2.1–2.7)	0.314 (0.270–0.354)	500.8 (432.1–563.3)	268.3 (224.2–317.7)	91.1 (68.9–115.4)
13	Headache disorders	2.2 (0.5–4.5)	0.291 (0.0640–0.616)	687.5 (139.0–1463.3)	27.7 (20.5–41.2)	-2.3 (-7.0–3.2)
14	Road injuries	2.2 (2.0–2.3)	0.280 (0.242–0.319)	643.7 (563.6–729.1)	-29.7 (-36.5—22.4)	-48.7 (-53.5—43.4)
15	Tracheal, bronchus, and lung cancer	2.0 (1.7–2.4)	0.262 (0.218–0.305)	400.5 (336.5–465.6)	-25.2 (-36.9—11.8)	-63.8 (-69.5—57.3)
16	Age-related and other hearing loss	1.9 (1.5–2.5)	0.254 (0.179–0.351)	420.5 (294.6–581.9)	73.4 (67.2–80.4)	-5.8 (-8.9—2.5)
17	Cirrhosis and other chronic liver diseases	1.8 (1.5–2.0)	0.228 (0.196–0.260)	415.9 (357.1–474.4)	43.4 (23.6–64.8)	-16.9 (-28.2–-4.7)
18	Falls	1.7 (1.4–2.0)	0.220 (0.169–0.283)	366.7 (281.4–470.7)	75.2 (62.4–88.4)	-5.1 (-11.4–0.9)
19	Self-harm	1.6 (1.4–1.9)	0.213 (0.182–0.245)	494.4 (424.7–567.9)	18.0 (1.1–35.0)	-9.2 (-22.0–3.7)
20	Osteoarthritis	1.3 (0.7–2.6)	0.172 (0.0828–0.346)	276.0 (132.5–557.4)	112.7 (105.4–122.0)	9.2 (5.5–14.0)
21	Colon and rectum cancer	1.3 (1.1–1.5)	0.167 (0.141–0.194)	275.5 (232.7–321.6)	29.0 (9.4–53.8)	-31.0 (-41.5–-17.7)
22	Alcohol use disorders	1.2 (1.1–1.4)	0.161 (0.127–0.203)	356.1 (278.5–454.8)	19.1 (7.0–35.0)	-11.4 (-18.8–-1.9)
23	Asthma	1.2 (0.9–1.6)	0.158 (0.107–0.223)	405.9 (272.2–583.4)	20.7 (12.5–28.5)	-8.9 (-14.9–-3.0)
24	Breast cancer	1.1 (0.9–1.4)	0.149 (0.121–0.179)	252.3 (203.8–305.5)	5.1 (-14.2–27.2)	-43.5 (-53.9–-31.4)
25	Oral disorders	1.1 (0.7–1.6)	0.146 (0.0861–0.219)	271.1 (158.1–408.2)	57.5 (51.5–63.9)	-6.3 (-10.2–-1.8)

Table S4: Cause-specific DALYs by US state in 2021 and percentage change between 1990 and 2021, Colorado						
**Rank**	**Cause Name**	**2021 Percentage of all cause DALYs**	**2021 DALYs (millions)**	**2021 Age Standardised Rate (per 100 000)**	**Percentage change DALY count 1990 to 2021**	**Percentage change age-standardised DALY rate 1990 to 2021**
	All causes	100.0 (100.0–100.0)	1.98 (1.69–2.27)	26543.5 (22529.7–30469.4)	110.3 (95.1–127.0)	-1.0 (-8.0–6.2)
1	COVID-19	7.4 (6.2–9.0)	0.147 (0.136–0.177)	1840.2 (1690.4–2224.5)	--	--
2	Drug use disorders	5.3 (4.5–6.1)	0.104 (0.0854–0.124)	1766.2 (1444.5–2098.8)	790.5 (659.4–951.1)	463.0 (377.2–566.0)
3	Ischaemic heart disease	4.9 (4.1–5.7)	0.0962 (0.0787–0.113)	1016.8 (830.5–1197.8)	4.2 (-12.4–22.0)	-60.8 (-67.1–-54.0)
4	Low back pain	4.3 (3.4–5.1)	0.0845 (0.0612–0.110)	1146.7 (829.9–1501.0)	80.5 (67.2–95.3)	-10.4 (-16.3–-4.0)
5	Chronic obstructive pulmonary disease	3.8 (3.2–4.3)	0.0750 (0.0641–0.0843)	760.9 (649.9–856.1)	139.2 (110.6–169.3)	-12.6 (-23.0–-1.6)
6	Other musculoskeletal disorders	3.5 (2.7–4.5)	0.0703 (0.0501–0.0931)	1003.0 (719.7–1329.8)	169.9 (140.8–201.8)	39.0 (24.5–56.1)
7	Depressive disorders	3.3 (2.4–4.4)	0.0660 (0.0440–0.0925)	1098.0 (732.5–1542.9)	143.5 (99.7–194.9)	46.6 (19.9–76.8)
8	Diabetes mellitus	3.2 (2.8–3.6)	0.0628 (0.0496–0.0789)	699.3 (552.2–873.8)	267.9 (226.6–305.0)	45.5 (29.3–60.5)
9	Self-harm	3.0 (2.6–3.5)	0.0598 (0.0507–0.0703)	988.7 (839.9–1156.8)	107.9 (74.6–143.8)	25.2 (5.6–46.6)
10	Falls	2.8 (2.5–3.3)	0.0562 (0.0448–0.0706)	637.8 (508.6–800.3)	194.0 (171.7–222.5)	20.1 (11.9–30.9)
11	Stroke	2.6 (2.3–3.0)	0.0522 (0.0440–0.0594)	566.3 (480.1–642.4)	86.5 (64.6–110.0)	-27.1 (-35.3–-18.1)
12	Alzheimer's disease and other dementias	2.5 (1.2–5.3)	0.0499 (0.0230–0.106)	502.9 (231.6–1062.2)	163.2 (138.8–190.6)	-4.2 (-13.1–5.7)
13	Anxiety disorders	2.4 (1.6–3.4)	0.0485 (0.0303–0.0697)	778.5 (484.1–1139.6)	120.1 (70.6–173.0)	28.2 (-0.7–56.8)
14	Road injuries	2.2 (2.0–2.4)	0.0434 (0.0375–0.0495)	685.1 (591.1–776.7)	2.6 (-9.0–16.4)	-44.0 (-50.7–-36.2)
15	Headache disorders	2.1 (0.5–4.2)	0.0424 (0.00936–0.0891)	683.6 (137.2–1442.2)	66.2 (56.8–85.3)	-3.0 (-7.5–3.0)
16	Tracheal, bronchus, and lung cancer	2.0 (1.7–2.4)	0.0404 (0.0333–0.0477)	415.2 (342.1–489.7)	32.0 (7.8–56.6)	-52.6 (-61.3–-44.0)
17	Chronic kidney disease	2.0 (1.7–2.2)	0.0387 (0.0325–0.0444)	428.4 (361.3–490.5)	419.1 (351.0–502.8)	105.0 (79.1–135.9)
18	Cirrhosis and other chronic liver diseases	1.9 (1.6–2.3)	0.0377 (0.0313–0.0451)	468.6 (391.0–557.7)	199.5 (147.8–262.4)	33.6 (10.7–60.6)
19	Age-related and other hearing loss	1.9 (1.4–2.4)	0.0372 (0.0261–0.0514)	417.6 (293.3–574.6)	134.0 (125.4–144.4)	-5.7 (-8.7–-2.2)
20	Alcohol use disorders	1.8 (1.6–2.0)	0.0350 (0.0288–0.0418)	523.7 (429.2–628.6)	106.8 (79.0–141.6)	15.2 (1.8–31.6)
21	Osteoarthritis	1.5 (0.8–2.9)	0.0300 (0.0146–0.0598)	322.1 (155.7–644.0)	180.7 (172.0–189.2)	5.8 (2.7–8.8)
22	Asthma	1.3 (0.9–1.7)	0.0252 (0.0171–0.0363)	437.8 (293.8–639.2)	65.4 (53.2–76.7)	-6.4 (-12.7–-0.2)
23	Colon and rectum cancer	1.2 (1.0–1.4)	0.0230 (0.0191–0.0274)	254.9 (210.7–303.3)	62.2 (32.4–96.8)	-36.3 (-48.0–-22.9)
24	Oral disorders	1.1 (0.7–1.5)	0.0220 (0.0132–0.0329)	273.6 (160.6–414.0)	114.2 (106.1–124.2)	-5.1 (-9.1–-1.3)
25	Neonatal disorders	1.1 (1.0–1.3)	0.0219 (0.0189–0.0253)	567.1 (501.7–646.1)	-4.8 (-15.6–9.4)	-32.0 (-40.1–-22.5)

Table S4: Cause-specific DALYs by US state in 2021 and percentage change between 1990 and 2021, Connecticut

Rank	Cause Name	2021 Percentage of all cause DALYs	2021 DALYs (millions)	2021 Age Standardised Rate (per 100 000)	Percentage change DALY count 1990 to 2021	Percentage change age-standardised DALY rate 1990 to 2021
	All causes	100.0 (100.0–100.0)	1.24 (1.07–1.42)	24457.8 (20813.7–28368.3)	18.8 (9.9–28.0)	-7.3 (-14.3—0.7)
1	Ischaemic heart disease	6.6 (5.6–7.7)	0.0822 (0.0670–0.0962)	1143.9 (943.9–1338.1)	-37.0 (-46.6—27.4)	-59.9 (-66.5—53.4)
2	Drug use disorders	6.3 (5.5–7.1)	0.0783 (0.0646–0.0932)	2199.8 (1815.8–2619.6)	444.6 (361.3–545.5)	462.6 (378.1–562.4)
3	COVID-19	5.6 (4.9–6.5)	0.0691 (0.0668–0.0740)	1260.2 (1202.7–1388.9)	--	--
4	Diabetes mellitus	4.3 (3.7–4.9)	0.0530 (0.0410–0.0677)	849.3 (657.1–1080.4)	140.8 (113.1–165.8)	65.5 (47.3–81.9)
5	Low back pain	4.1 (3.3–5.0)	0.0508 (0.0371–0.0651)	1075.4 (787.9–1386.3)	10.4 (2.2–19.3)	-9.7 (-15.9—3.0)
6	Chronic obstructive pulmonary disease	3.6 (3.0–4.0)	0.0439 (0.0382–0.0498)	607.9 (532.3–685.8)	55.2 (39.2–72.3)	-0.5 (-10.7–10.3)
7	Other musculoskeletal disorders	3.5 (2.7–4.4)	0.0434 (0.0308–0.0578)	968.2 (693.9–1289.1)	64.0 (45.5–84.8)	39.8 (25.0–57.3)
8	Alzheimer's disease and other dementias	3.4 (1.6–7.2)	0.0422 (0.0194–0.0913)	506.1 (237.5–1100.1)	61.2 (45.9–77.1)	-4.0 (-13.4–5.1)
9	Tracheal, bronchus, and lung cancer	2.9 (2.4–3.4)	0.0355 (0.0294–0.0419)	516.0 (427.1–608.9)	-24.9 (-38.2—10.2)	-53.2 (-61.8—43.8)
10	Stroke	2.8 (2.5–3.2)	0.0350 (0.0293–0.0404)	497.2 (424.0–571.8)	-2.1 (-12.9–9.8)	-36.7 (-43.6—29.0)
11	Anxiety disorders	2.4 (1.6–3.4)	0.0293 (0.0190–0.0444)	765.2 (489.3–1150.3)	30.8 (3.8–69.8)	24.7 (-1.5–61.9)
12	Chronic kidney disease	2.3 (2.0–2.6)	0.0284 (0.0241–0.0328)	424.3 (361.7–487.9)	181.9 (148.7–222.2)	82.6 (61.7–106.9)
13	Falls	2.3 (2.0–2.6)	0.0284 (0.0224–0.0359)	444.1 (349.8–563.6)	72.9 (60.1–87.2)	11.9 (4.2–20.7)
14	Depressive disorders	2.3 (1.6–3.1)	0.0281 (0.0186–0.0405)	758.6 (499.2–1102.0)	55.6 (28.3–90.0)	52.5 (25.8–89.3)
15	Age-related and other hearing loss	2.1 (1.6–2.7)	0.0262 (0.0182–0.0362)	416.6 (290.0–576.7)	36.3 (31.5–41.9)	-5.3 (-8.8—2.0)
16	Headache disorders	2.1 (0.5–4.2)	0.0261 (0.00585–0.0548)	687.9 (137.6–1463.5)	2.1 (-3.5–14.0)	-3.1 (-8.1–2.8)
17	Osteoarthritis	1.6 (0.9–3.2)	0.0205 (0.00991–0.0413)	316.7 (151.8–634.0)	59.1 (55.0–63.9)	5.7 (2.7–8.7)
18	Road injuries	1.5 (1.4–1.7)	0.0191 (0.0163–0.0223)	482.3 (415.2–561.6)	-47.0 (-53.0—40.2)	-53.4 (-59.0—47.3)
19	Cirrhosis and other chronic liver diseases	1.4 (1.2–1.6)	0.0173 (0.0141–0.0205)	311.9 (253.1–368.9)	21.2 (-1.5–43.7)	-12.7 (-29.4–3.2)
20	Self-harm	1.4 (1.2–1.6)	0.0172 (0.0140–0.0205)	444.6 (365.6–532.1)	1.1 (-17.7–20.9)	-4.1 (-22.1–14.8)
21	Colon and rectum cancer	1.3 (1.1–1.5)	0.0161 (0.0134–0.0191)	250.1 (207.1–296.5)	-22.8 (-36.4—7.5)	-46.7 (-56.5—35.9)
22	Asthma	1.2 (0.9–1.7)	0.0154 (0.0105–0.0223)	449.3 (302.6–667.1)	10.5 (3.2–18.5)	3.6 (-3.0–11.4)
23	Alcohol use disorders	1.2 (1.1–1.4)	0.0147 (0.0117–0.0182)	353.9 (278.3–444.0)	11.8 (-3.4–29.3)	0.9 (-11.3–15.5)
24	Breast cancer	1.2 (1.0–1.4)	0.0146 (0.0117–0.0179)	242.9 (192.3–300.1)	-26.5 (-40.9—11.0)	-49.0 (-59.2—37.4)
25	Oral disorders	1.2 (0.8–1.6)	0.0146 (0.00875–0.0219)	272.2 (159.0–414.1)	23.6 (19.2–27.9)	-6.1 (-9.9—2.0)

Table S4: Cause-specific DALYs by US state in 2021 and percentage change between 1990 and 2021, Delaware

Rank	Cause Name	2021 Percentage of all cause DALYs	2021 DALYs (millions)	2021 Age Standardised Rate (per 100 000)	Percentage change DALY count 1990 to 2021	Percentage change age-standardised DALY rate 1990 to 2021
	All causes	100.0 (100.0–100.0)	0.409 (0.360–0.463)	29847.1 (26152.7–34145.6)	80.5 (70.3–91.8)	0.1 (-5.8–5.9)
1	COVID-19	7.4 (6.4–8.8)	0.0302 (0.0277–0.0345)	2033.1 (1849.2–2312.8)	--	--
2	Ischaemic heart disease	6.6 (5.7–7.6)	0.0270 (0.0236–0.0308)	1425.4 (1247.9–1620.9)	-7.1 (-17.2–4.3)	-59.6 (-64.3—54.4)
3	Drug use disorders	6.2 (5.3–7.1)	0.0252 (0.0208–0.0296)	2607.5 (2171.0–3059.2)	722.1 (600.1–878.4)	537.8 (446.8–653.9)
4	Diabetes mellitus	4.3 (3.8–4.9)	0.0177 (0.0141–0.0223)	1043.9 (829.9–1313.1)	188.0 (154.8–220.9)	38.1 (23.0–53.3)
5	Low back pain	3.8 (3.0–4.6)	0.0155 (0.0112–0.0201)	1193.0 (873.5–1552.9)	58.7 (46.8–70.9)	-7.1 (-13.9–0.3)
6	Chronic obstructive pulmonary disease	3.7 (3.3–4.2)	0.0152 (0.0138–0.0166)	773.6 (707.2–847.7)	144.8 (125.7–166.6)	6.5 (-1.8–16.0)
7	Tracheal, bronchus, and lung cancer	3.3 (2.9–3.8)	0.0135 (0.0117–0.0155)	721.1 (624.2–825.4)	16.2 (1.6–32.3)	-49.0 (-55.7—41.6)
8	Stroke	3.3 (2.9–3.6)	0.0134 (0.0119–0.0149)	718.3 (640.6–803.3)	78.0 (63.2–96.1)	-21.7 (-28.2—14.1)
9	Other musculoskeletal disorders	3.2 (2.4–4.0)	0.0129 (0.00929–0.0172)	1037.4 (747.4–1382.3)	145.6 (122.2–172.0)	49.4 (33.9–66.0)
10	Alzheimer's disease and other dementias	2.6 (1.2–5.5)	0.0108 (0.00502–0.0223)	500.5 (234.7–1032.1)	147.4 (131.5–165.5)	-4.5 (-10.5–2.3)
11	Depressive disorders	2.6 (1.8–3.5)	0.0107 (0.00727–0.0150)	1071.2 (723.4–1503.6)	117.2 (75.8–160.0)	58.6 (27.9–90.9)
12	Chronic kidney disease	2.4 (2.1–2.6)	0.00976 (0.00870–0.0110)	562.0 (502.8–627.6)	298.1 (261.7–343.8)	81.9 (65.7–100.9)
13	Anxiety disorders	2.1 (1.4–3.0)	0.00846 (0.00536–0.0126)	811.8 (514.8–1229.3)	88.5 (47.1–140.0)	32.8 (4.0–67.7)
14	Road injuries	2.0 (1.8–2.1)	0.00804 (0.00712–0.00907)	773.8 (691.4–866.4)	-13.7 (-21.6—5.0)	-40.9 (-46.2—34.8)
15	Age-related and other hearing loss	1.8 (1.3–2.3)	0.00720 (0.00506–0.00990)	415.4 (291.0–574.0)	100.7 (92.8–109.1)	-5.9 (-9.0—2.5)
16	Headache disorders	1.7 (0.4–3.6)	0.00712 (0.00160–0.0148)	686.3 (136.9–1447.0)	37.9 (30.2–53.4)	-3.4 (-8.2–2.3)
17	Falls	1.7 (1.5–2.0)	0.00706 (0.00567–0.00896)	419.3 (335.0–530.6)	124.8 (109.1–143.6)	4.3 (-2.5–12.4)
18	Self-harm	1.5 (1.3–1.7)	0.00607 (0.00524–0.00703)	591.4 (512.8–687.4)	52.9 (31.1–78.2)	11.1 (-4.9–29.7)
19	Osteoarthritis	1.5 (0.8–2.8)	0.00607 (0.00296–0.0121)	341.2 (164.9–679.8)	131.0 (123.7–138.9)	5.4 (2.1–8.7)
20	Cirrhosis and other chronic liver diseases	1.4 (1.2–1.6)	0.00579 (0.00510–0.00663)	399.7 (350.9–458.1)	94.5 (69.9–124.2)	2.9 (-10.3–18.4)
21	Colon and rectum cancer	1.4 (1.2–1.5)	0.00552 (0.00472–0.00639)	317.6 (271.6–368.1)	24.8 (5.5–47.1)	-40.8 (-50.2—30.7)
22	Hypertensive heart disease	1.2 (1.1–1.4)	0.00507 (0.00444–0.00573)	305.7 (269.8–346.2)	208.2 (170.5–248.0)	49.1 (30.9–68.3)
23	Neonatal disorders	1.2 (1.0–1.4)	0.00497 (0.00415–0.00583)	849.8 (717.1–1005.6)	-29.9 (-41.1—17.2)	-35.1 (-45.6—22.5)
24	Endocrine, metabolic, blood, and immune disorders	1.2 (1.1–1.3)	0.00485 (0.00425–0.00555)	358.3 (314.4–411.9)	227.3 (190.0–272.2)	76.0 (57.7–98.2)
25	Breast cancer	1.2 (1.0–1.4)	0.00483 (0.00415–0.00564)	301.9 (259.1–350.9)	15.2 (-1.1–33.0)	-43.0 (-51.1—34.4)

Table S4: Cause-specific DALYs by US state in 2021 and percentage change between 1990 and 2021, District of Columbia						
Rank	Cause Name	2021 Percentage of all cause DALYs	2021 DALYs (millions)	2021 Age Standardised Rate (per 100 000)	Percentage change DALY count 1990 to 2021	Percentage change age-standardised DALY rate 1990 to 2021
	All causes	100.0 (100.0–100.0)	0.245 (0.214–0.278)	28932.1 (25273.2–32849.3)	-21.5 (-27.8–-15.2)	-37.7 (-42.9–-32.7)
1	Drug use disorders	8.0 (6.9–9.0)	0.0196 (0.0160–0.0231)	2333.5 (1902.3–2752.8)	273.0 (225.1–323.2)	232.1 (189.7–277.2)
2	Ischaemic heart disease	6.6 (5.6–7.9)	0.0162 (0.0135–0.0191)	1680.6 (1400.5–1988.7)	-38.7 (-48.6–-27.8)	-51.7 (-59.7–-43.1)
3	COVID-19	5.9 (4.2–8.1)	0.0144 (0.0105–0.0189)	1557.2 (1136.0–2040.9)	--	--
4	Low back pain	3.2 (2.5–3.8)	0.00777 (0.00567–0.0102)	889.0 (650.1–1164.1)	-3.9 (-11.5–5.5)	-20.8 (-27.1–-13.5)
5	Diabetes mellitus	3.2 (2.8–3.5)	0.00774 (0.00629–0.00942)	834.7 (678.3–1013.5)	13.8 (-0.4–27.7)	-8.8 (-20.3–2.1)
6	Interpersonal violence	3.0 (2.6–3.5)	0.00741 (0.00644–0.00850)	1158.3 (1001.7–1329.2)	-70.9 (-75.0–-66.3)	-70.1 (-74.4–-65.3)
7	Other musculoskeletal disorders	2.8 (2.2–3.6)	0.00695 (0.00500–0.00924)	837.1 (601.7–1113.6)	40.4 (27.3–55.2)	21.9 (9.4–35.1)
8	Stroke	2.8 (2.4–3.1)	0.00673 (0.00574–0.00773)	689.5 (593.4–792.6)	-33.8 (-41.8–-25.4)	-48.9 (-54.9–-42.5)
9	Anxiety disorders	2.6 (1.7–3.5)	0.00627 (0.00395–0.00894)	791.8 (493.9–1117.9)	45.2 (13.2–80.8)	28.2 (0.1–59.2)
10	Depressive disorders	2.5 (1.8–3.5)	0.00626 (0.00403–0.00896)	821.6 (538.3–1182.4)	51.0 (23.2–82.3)	39.9 (13.8–70.2)
11	Chronic kidney disease	2.3 (1.5–2.8)	0.00561 (0.00351–0.00702)	593.2 (371.5–741.3)	34.5 (-14.2–66.5)	2.2 (-34.4–26.8)
12	Hypertensive heart disease	2.3 (1.8–2.7)	0.00555 (0.00453–0.00680)	584.6 (478.3–722.0)	-0.4 (-18.6–23.4)	-22.9 (-37.3–-3.4)
13	Tracheal, bronchus, and lung cancer	2.3 (1.9–2.7)	0.00554 (0.00470–0.00656)	589.7 (500.3–699.0)	-52.4 (-59.7–-42.9)	-63.2 (-69.0–-55.9)
14	Alzheimer's disease and other dementias	2.3 (1.1–4.7)	0.00553 (0.00264–0.0113)	489.0 (238.5–1008.2)	25.8 (14.6–38.5)	-5.9 (-15.0–2.8)
15	Headache disorders	2.2 (0.5–4.5)	0.00552 (0.00119–0.0116)	699.0 (138.9–1471.2)	11.1 (4.6–22.3)	-2.5 (-7.5–2.9)
16	Chronic obstructive pulmonary disease	2.0 (1.7–2.2)	0.00482 (0.00423–0.00538)	493.1 (433.7–549.6)	4.7 (-5.4–16.6)	-15.0 (-23.4–-5.4)
17	Alcohol use disorders	1.9 (1.7–2.2)	0.00473 (0.00379–0.00571)	552.7 (443.6–669.5)	-7.0 (-14.7–2.6)	-20.7 (-27.2–-12.9)
18	Falls	1.9 (1.6–2.2)	0.00467 (0.00370–0.00586)	499.4 (394.9–625.6)	17.2 (8.8–25.1)	-9.3 (-15.8–-3.3)
19	Cirrhosis and other chronic liver diseases	1.7 (1.4–1.9)	0.00404 (0.00344–0.00468)	453.2 (386.3–525.1)	-47.4 (-55.7–-38.7)	-59.2 (-65.7–-52.6)
20	Neonatal disorders	1.6 (1.4–1.9)	0.00397 (0.00330–0.00474)	828.7 (687.3–991.8)	-76.2 (-80.2–-71.4)	-79.1 (-82.8–-74.7)
21	Age-related and other hearing loss	1.6 (1.2–2.1)	0.00395 (0.00275–0.00545)	419.4 (291.0–581.8)	16.7 (12.7–21.1)	-4.6 (-7.6–-1.0)
22	HIV/AIDS	1.6 (1.3–1.9)	0.00390 (0.00313–0.00498)	442.9 (358.2–562.0)	-84.9 (-87.6–-81.4)	-87.3 (-89.5–-84.4)
23	Colon and rectum cancer	1.3 (1.1–1.5)	0.00315 (0.00265–0.00373)	337.4 (283.7–401.1)	-43.5 (-52.3–-32.1)	-54.8 (-61.9–-45.8)
24	Breast cancer	1.3 (1.0–1.5)	0.00307 (0.00252–0.00363)	332.3 (273.2–394.8)	-43.4 (-53.3–-31.7)	-56.2 (-64.0–-47.2)
25	Asthma	1.2 (0.9–1.6)	0.00299 (0.00204–0.00423)	451.2 (304.0–655.6)	5.2 (-3.2–12.4)	-7.4 (-14.5–-1.2)

Table S4: Cause-specific DALYs by US state in 2021 and percentage change between 1990 and 2021, Florida						
**Rank**	**Cause Name**	**2021 Percentage of all cause DALYs**	**2021 DALYs (millions)**	**2021 Age Standardised Rate (per 100 000)**	**Percentage change DALY count 1990 to 2021**	**Percentage change age-standardised DALY rate 1990 to 2021**
	All causes	100.0 (100.0–100.0)	9.10 (7.96–10.3)	29425.1 (25535.9–33748.6)	85.4 (72.9–99.0)	0.0 (-6.5–6.3)
1	COVID-19	10.0 (8.7–11.6)	0.909 (0.865–1.03)	2699.9 (2536.5–3073.9)	--	--
2	Ischaemic heart disease	6.9 (5.8–8.0)	0.630 (0.528–0.735)	1457.5 (1220.9–1703.1)	-6.0 (-18.7–7.7)	-52.9 (-59.5—45.8)
3	Drug use disorders	5.2 (4.5–5.9)	0.475 (0.391–0.564)	2241.9 (1845.2–2667.5)	977.0 (811.1–1159.6)	580.3 (477.7–691.9)
4	Diabetes mellitus	4.2 (3.6–4.8)	0.379 (0.298–0.475)	990.3 (773.2–1239.5)	237.9 (202.3–268.5)	72.0 (54.3–87.9)
5	Low back pain	3.9 (3.1–4.7)	0.353 (0.256–0.457)	1195.0 (869.7–1554.3)	68.0 (56.8–79.6)	-9.6 (-15.4—3.0)
6	Chronic obstructive pulmonary disease	3.8 (3.3–4.2)	0.345 (0.302–0.385)	761.4 (672.9–851.9)	124.0 (101.0–148.1)	11.9 (0.4–23.7)
7	Stroke	3.3 (2.8–3.6)	0.298 (0.255–0.339)	697.3 (601.7–789.0)	69.3 (50.5–88.6)	-17.3 (-26.5—7.7)
8	Tracheal, bronchus, and lung cancer	3.0 (2.5–3.5)	0.272 (0.230–0.318)	632.4 (536.9–737.8)	5.7 (-9.3–23.6)	-51.7 (-58.6—43.0)
9	Alzheimer's disease and other dementias	2.9 (1.4–5.9)	0.262 (0.122–0.557)	502.0 (233.3–1067.7)	105.8 (88.3–126.2)	-3.6 (-11.0–5.4)
10	Depressive disorders	2.7 (1.9–3.7)	0.249 (0.166–0.353)	1139.9 (765.3–1615.7)	126.4 (84.7–172.1)	44.6 (17.0–74.0)
11	Other musculoskeletal disorders	2.6 (2.0–3.3)	0.237 (0.171–0.315)	841.3 (605.7–1115.8)	141.4 (117.0–170.7)	33.6 (19.4–50.4)
12	Road injuries	2.3 (2.1–2.5)	0.208 (0.178–0.238)	888.0 (772.1–1009.8)	4.0 (-7.5–15.8)	-41.1 (-47.6—33.9)
13	Chronic kidney disease	2.1 (1.8–2.3)	0.190 (0.163–0.216)	474.2 (409.6–540.1)	318.3 (269.6–374.7)	101.4 (79.7–128.4)
14	Anxiety disorders	2.1 (1.4–3.0)	0.189 (0.121–0.280)	829.2 (522.4–1222.8)	118.9 (70.0–175.6)	36.2 (5.6–70.0)
15	Falls	2.1 (1.8–2.4)	0.187 (0.150–0.237)	463.1 (367.7–588.0)	153.6 (133.8–178.0)	11.8 (3.2–21.8)
16	Age-related and other hearing loss	1.8 (1.4–2.4)	0.166 (0.116–0.227)	416.7 (294.6–572.2)	80.7 (74.0–88.0)	-6.1 (-8.8—2.7)
17	Headache disorders	1.7 (0.4–3.4)	0.155 (0.0354–0.325)	684.9 (138.4–1442.7)	60.3 (52.3–72.9)	-3.2 (-7.8–2.4)
18	Self-harm	1.6 (1.4–1.9)	0.148 (0.126–0.172)	628.4 (536.9–728.1)	61.0 (36.8–88.0)	-4.2 (-18.3–11.9)
19	Cirrhosis and other chronic liver diseases	1.5 (1.3–1.8)	0.137 (0.115–0.161)	416.7 (348.5–489.7)	112.0 (76.7–149.2)	6.5 (-11.3–25.0)
20	Osteoarthritis	1.4 (0.7–2.5)	0.125 (0.0606–0.252)	307.8 (147.8–616.3)	110.5 (105.0–116.9)	5.9 (3.0–9.1)
21	Colon and rectum cancer	1.3 (1.1–1.6)	0.122 (0.102–0.145)	317.1 (264.0–378.3)	37.4 (16.2–65.3)	-25.9 (-37.5—10.7)
22	Breast cancer	1.1 (0.9–1.3)	0.100 (0.0820–0.122)	276.9 (225.4–338.9)	23.5 (1.0–49.8)	-39.4 (-50.8—25.7)
23	Neonatal disorders	1.1 (0.9–1.2)	0.0969 (0.0836–0.111)	765.8 (677.4–865.2)	-14.8 (-24.1—4.0)	-35.7 (-42.5—27.8)
24	Alcohol use disorders	1.0 (0.9–1.2)	0.0952 (0.0774–0.114)	381.9 (306.7–466.2)	66.6 (49.1–89.4)	-5.5 (-14.8–5.8)
25	Oral disorders	1.0 (0.6–1.4)	0.0906 (0.0544–0.135)	272.6 (159.3–416.0)	72.7 (65.4–80.3)	-5.3 (-9.6—1.1)

Table S4: Cause-specific DALYs by US state in 2021 and percentage change between 1990 and 2021, Georgia

Rank	Cause Name	2021 Percentage of all cause DALYs	2021 DALYs (millions)	2021 Age Standardised Rate (per 100 000)	Percentage change DALY count 1990 to 2021	Percentage change age-standardised DALY rate 1990 to 2021
	All causes	100.0 (100.0–100.0)	4.24 (3.73–4.78)	31111.8 (27306.4–35274.4)	93.9 (80.1–108.4)	-2.2 (-8.8–4.5)
1	COVID-19	11.9 (10.5–13.5)	0.504 (0.491–0.539)	3516.8 (3404.0–3814.7)	--	--
2	Ischaemic heart disease	6.3 (5.4–7.3)	0.268 (0.228–0.313)	1631.0 (1382.2–1903.7)	5.8 (-9.5–22.9)	-55.2 (-61.8–-47.9)
3	Drug use disorders	4.0 (3.4–4.6)	0.169 (0.135–0.203)	1531.3 (1225.1–1837.1)	720.8 (608.8–858.5)	453.0 (377.1–543.9)
4	Diabetes mellitus	3.9 (3.4–4.5)	0.167 (0.132–0.208)	1046.7 (829.5–1309.3)	250.4 (215.0–283.1)	53.5 (38.0–67.5)
5	Low back pain	3.7 (3.0–4.5)	0.158 (0.114–0.206)	1188.8 (862.2–1551.9)	71.3 (59.6–85.6)	-8.0 (-13.9–-1.0)
6	Chronic obstructive pulmonary disease	3.7 (3.3–4.1)	0.157 (0.139–0.177)	910.1 (804.1–1020.2)	173.3 (144.5–205.8)	11.9 (0.0–25.2)
7	Stroke	3.3 (2.9–3.7)	0.141 (0.122–0.160)	868.6 (755.2–985.9)	56.3 (38.0–77.3)	-32.4 (-40.5–-23.5)
8	Tracheal, bronchus, and lung cancer	2.9 (2.5–3.3)	0.123 (0.104–0.142)	707.8 (600.6–817.5)	22.8 (3.7–43.9)	-51.3 (-58.9–-42.9)
9	Depressive disorders	2.8 (1.9–3.8)	0.118 (0.0771–0.168)	1055.0 (681.8–1508.7)	140.2 (91.1–193.2)	53.0 (22.2–87.4)
10	Other musculoskeletal disorders	2.7 (2.1–3.4)	0.117 (0.0842–0.155)	900.7 (650.0–1202.9)	157.9 (130.8–185.9)	41.4 (26.9–57.4)
11	Chronic kidney disease	2.6 (2.3–2.9)	0.111 (0.0963–0.125)	697.0 (608.1–784.0)	329.9 (278.9–386.3)	87.0 (65.6–110.6)
12	Road injuries	2.3 (2.1–2.4)	0.0955 (0.0828–0.109)	829.9 (725.6–939.9)	-13.3 (-23.4–-2.4)	-47.9 (-54.0–-41.3)
13	Anxiety disorders	2.2 (1.5–3.1)	0.0946 (0.0584–0.137)	829.3 (504.9–1197.7)	117.3 (66.4–179.6)	35.5 (3.6–74.3)
14	Alzheimer's disease and other dementias	2.0 (1.0–4.3)	0.0852 (0.0390–0.184)	494.7 (226.9–1069.0)	141.6 (120.4–164.0)	-4.1 (-12.3–4.4)
15	Headache disorders	1.8 (0.4–3.7)	0.0790 (0.0172–0.165)	691.2 (139.3–1457.8)	56.3 (48.1–73.8)	-2.7 (-7.4–3.3)
16	Self-harm	1.7 (1.5–2.0)	0.0737 (0.0622–0.0854)	646.5 (548.3–748.1)	66.8 (40.8–94.3)	6.0 (-10.3–23.3)
17	Hypertensive heart disease	1.6 (1.4–1.9)	0.0687 (0.0577–0.0819)	437.8 (367.9–520.9)	344.9 (274.3–425.6)	97.3 (65.9–132.9)
18	Falls	1.6 (1.4–1.9)	0.0686 (0.0541–0.0879)	437.9 (345.2–558.7)	125.7 (110.8–142.8)	1.1 (-5.0–8.1)
19	Age-related and other hearing loss	1.6 (1.2–2.0)	0.0663 (0.0463–0.0913)	418.3 (294.1–580.6)	111.8 (103.2–121.0)	-5.8 (-9.1–-2.5)
20	Neonatal disorders	1.4 (1.2–1.6)	0.0590 (0.0514–0.0670)	850.8 (757.8–955.8)	-25.8 (-33.7–-16.1)	-40.2 (-46.5–-33.2)
21	Cirrhosis and other chronic liver diseases	1.4 (1.2–1.6)	0.0582 (0.0494–0.0681)	384.6 (327.4–447.9)	104.2 (73.0–140.9)	-5.7 (-20.2–10.9)
22	Colon and rectum cancer	1.4 (1.2–1.6)	0.0575 (0.0481–0.0671)	355.6 (297.6–415.3)	81.6 (50.8–115.9)	-21.4 (-34.7–-6.8)
23	Interpersonal violence	1.2 (1.1–1.4)	0.0517 (0.0448–0.0588)	509.4 (444.5–576.5)	-7.0 (-19.9–5.8)	-35.2 (-43.6–-26.5)
24	Osteoarthritis	1.2 (0.6–2.3)	0.0511 (0.0248–0.103)	307.5 (148.1–618.1)	154.2 (146.3–161.8)	6.1 (2.8–9.1)
25	Breast cancer	1.1 (0.9–1.4)	0.0486 (0.0397–0.0589)	311.0 (251.8–378.4)	42.5 (16.3–72.3)	-36.4 (-48.2–-23.0)

Table S4: Cause-specific DALYs by US state in 2021 and percentage change between 1990 and 2021, Hawaii						
Rank	Cause Name	2021 Percentage of all cause DALYs	2021 DALYs (millions)	2021 Age Standardised Rate (per 100 000)	Percentage change DALY count 1990 to 2021	Percentage change age-standardised DALY rate 1990 to 2021
	All causes	100.0<br>(100.0–100.0)	0.474<br>(0.412–0.542)	23323.2<br>(20113.4–26912.4)	54.6<br>(43.1–67.6)	-7.3<br>(-14.1—0.6)
1	Ischaemic heart disease	6.5<br>(5.4–7.8)	0.0307<br>(0.0253–0.0362)	1104.3<br>(906.3–1305.8)	5.3<br>(-11.3–23.1)	-52.0<br>(-60.3—43.6)
2	Diabetes mellitus	4.3<br>(3.6–4.9)	0.0203<br>(0.0160–0.0260)	814.5<br>(641.3–1039.7)	187.0<br>(157.5–215.0)	47.1<br>(31.6–62.1)
3	Low back pain	3.9<br>(3.1–4.7)	0.0185<br>(0.0134–0.0241)	967.9<br>(704.4–1277.8)	27.1<br>(15.9–39.0)	-16.7<br>(-22.8—9.7)
4	Alzheimer's disease and other dementias	3.9<br>(1.8–7.9)	0.0183<br>(0.00867–0.0374)	509.0<br>(246.6–1044.6)	184.0<br>(158.6–209.3)	-2.3<br>(-9.9–6.3)
5	Stroke	3.9<br>(3.3–4.4)	0.0182<br>(0.0155–0.0208)	646.2<br>(555.6–737.3)	51.1<br>(33.7–70.5)	-32.2<br>(-40.1—23.6)
6	Drug use disorders	3.7<br>(3.2–4.2)	0.0176<br>(0.0149–0.0207)	1220.9<br>(1028.8–1438.7)	400.4<br>(328.7–493.2)	338.9<br>(277.1–417.8)
7	COVID-19	3.3<br>(2.8–4.2)	0.0155<br>(0.0147–0.0195)	690.9<br>(645.9–875.1)	--	--
8	Other musculoskeletal disorders	2.9<br>(2.3–3.7)	0.0139<br>(0.0101–0.0186)	770.4<br>(553.5–1034.5)	85.1<br>(62.7–109.1)	27.6<br>(13.3–43.3)
9	Tracheal, bronchus, and lung cancer	2.9<br>(2.4–3.5)	0.0137<br>(0.0115–0.0161)	501.9<br>(422.4–591.3)	21.6<br>(1.7–44.6)	-42.5<br>(-51.6—32.0)
10	Depressive disorders	2.8<br>(2.1–3.9)	0.0135<br>(0.00878–0.0192)	923.8<br>(596.7–1326.9)	73.3<br>(41.8–108.5)	45.2<br>(18.4–76.4)
11	Chronic obstructive pulmonary disease	2.8<br>(2.3–3.1)	0.0130<br>(0.0114–0.0147)	445.9<br>(394.0–504.3)	113.0<br>(92.4–135.4)	-4.0<br>(-12.8–5.9)
12	Chronic kidney disease	2.6<br>(2.3–2.9)	0.0125<br>(0.0107–0.0143)	472.2<br>(411.1–535.3)	312.0<br>(263.5–376.1)	94.8<br>(73.1–123.7)
13	Falls	2.5<br>(2.1–2.9)	0.0117<br>(0.00929–0.0149)	460.7<br>(362.1–587.5)	127.4<br>(109.5–147.3)	11.9<br>(4.6–20.1)
14	Anxiety disorders	2.4<br>(1.6–3.3)	0.0115<br>(0.00713–0.0167)	750.7<br>(466.8–1102.1)	54.6<br>(20.5–92.2)	24.0<br>(-3.0–55.0)
15	Age-related and other hearing loss	2.3<br>(1.7–2.9)	0.0108<br>(0.00759–0.0149)	422.9<br>(296.1–584.1)	88.3<br>(80.2–96.9)	-5.8<br>(-9.2—2.5)
16	Headache disorders	2.2<br>(0.5–4.4)	0.0103<br>(0.00229–0.0218)	686.3<br>(137.0–1455.4)	21.0<br>(14.3–35.2)	-2.2<br>(-6.9–3.4)
17	Self-harm	2.0<br>(1.6–2.3)	0.00926<br>(0.00770–0.0109)	610.4<br>(505.4–720.7)	28.4<br>(5.7–52.7)	5.6<br>(-13.1–25.5)
18	Road injuries	1.8<br>(1.7–1.9)	0.00843<br>(0.00726–0.00972)	544.2<br>(473.9–623.4)	-30.8<br>(-38.6—22.6)	-47.4<br>(-53.3—41.1)
19	Osteoarthritis	1.5<br>(0.8–2.9)	0.00733<br>(0.00357–0.0147)	282.0<br>(135.8–563.0)	117.1<br>(110.0–124.6)	6.8<br>(3.5–10.4)
20	Colon and rectum cancer	1.5<br>(1.3–1.8)	0.00722<br>(0.00603–0.00865)	284.1<br>(236.4–339.3)	33.9<br>(8.6–60.0)	-32.5<br>(-45.1—19.1)
21	Asthma	1.5<br>(1.1–2.0)	0.00711<br>(0.00483–0.0101)	466.3<br>(313.4–681.9)	34.6<br>(27.0–42.5)	-2.2<br>(-7.9–3.4)
22	Cirrhosis and other chronic liver diseases	1.4<br>(1.1–1.6)	0.00644<br>(0.00544–0.00757)	308.0<br>(261.3–363.3)	81.9<br>(50.9–117.3)	9.3<br>(-9.5–30.1)
23	Neonatal disorders	1.3<br>(1.1–1.5)	0.00610<br>(0.00515–0.00709)	702.7<br>(593.9–817.2)	-34.6<br>(-43.7—23.3)	-27.9<br>(-38.6—13.8)
24	Oral disorders	1.2<br>(0.8–1.7)	0.00576<br>(0.00340–0.00875)	267.4<br>(153.6–413.7)	57.5<br>(42.2–70.3)	-8.3<br>(-16.3—0.2)
25	Breast cancer	1.2<br>(1.0–1.4)	0.00572<br>(0.00475–0.00680)	243.9<br>(200.8–290.3)	31.5<br>(9.5–57.2)	-29.9<br>(-41.9—15.4)

Table S4: Cause-specific DALYs by US state in 2021 and percentage change between 1990 and 2021, Idaho

Rank	Cause Name	2021 Percentage of all cause DALYs	2021 DALYs (millions)	2021 Age Standardised Rate (per 100 000)	Percentage change DALY count 1990 to 2021	Percentage change age-standardised DALY rate 1990 to 2021
	All causes	100.0 (100.0–100.0)	0.664 (0.583–0.761)	27820.4 (24175.1–31963.7)	120.5 (104.5–134.6)	2.8 (-4.2–9.4)
1	COVID-19	10.6 (9.2–12.8)	0.0703 (0.0661–0.0803)	2825.7 (2639.8–3239.3)	--	--
2	Ischaemic heart disease	5.6 (4.7–6.5)	0.0372 (0.0311–0.0429)	1235.2 (1037.7–1425.3)	11.3 (-5.2–27.7)	-54.6 (-61.4—47.7)
3	Low back pain	4.1 (3.3–5.0)	0.0274 (0.0198–0.0357)	1222.4 (882.2–1600.7)	90.9 (77.9–104.9)	-7.1 (-13.6—0.1)
4	Chronic obstructive pulmonary disease	4.0 (3.5–4.6)	0.0267 (0.0234–0.0298)	835.7 (732.8–936.1)	168.6 (139.2–198.2)	6.7 (-5.0–18.6)
5	Drug use disorders	3.9 (3.3–4.5)	0.0258 (0.0206–0.0310)	1438.2 (1150.1–1722.4)	758.2 (647.4–909.2)	390.1 (330.0–473.4)
6	Diabetes mellitus	3.6 (3.1–4.1)	0.0237 (0.0183–0.0302)	835.6 (646.3–1062.6)	259.5 (217.5–298.3)	49.2 (32.4–64.7)
7	Other musculoskeletal disorders	3.5 (2.7–4.5)	0.0235 (0.0170–0.0313)	1095.4 (791.4–1455.8)	197.1 (168.7–229.9)	48.3 (34.3–65.6)
8	Depressive disorders	3.2 (2.2–4.3)	0.0212 (0.0141–0.0313)	1127.9 (737.5–1678.2)	179.3 (124.8–240.2)	55.6 (24.9–88.2)
9	Stroke	2.7 (2.4–3.0)	0.0182 (0.0159–0.0205)	610.8 (535.8–689.2)	64.6 (47.1–82.8)	-31.1 (-38.4—23.8)
10	Alzheimer's disease and other dementias	2.5 (1.2–5.3)	0.0165 (0.00762–0.0359)	502.4 (232.1–1092.1)	146.0 (125.6–165.4)	-2.5 (-10.6–5.3)
11	Self-harm	2.5 (2.1–2.9)	0.0164 (0.0137–0.0192)	862.0 (720.5–1004.6)	101.5 (68.1–138.6)	9.9 (-8.1–30.0)
12	Tracheal, bronchus, and lung cancer	2.3 (2.0–2.7)	0.0155 (0.0130–0.0179)	503.4 (424.1–584.9)	42.1 (18.5–67.9)	-45.6 (-54.9—35.7)
13	Anxiety disorders	2.3 (1.5–3.2)	0.0152 (0.00943–0.0226)	789.8 (486.9–1170.3)	136.6 (84.1–195.4)	29.9 (1.5–62.4)
14	Road injuries	2.3 (2.1–2.5)	0.0151 (0.0130–0.0175)	770.7 (667.9–885.1)	-3.9 (-14.4–7.7)	-49.7 (-55.4—43.3)
15	Falls	2.2 (1.9–2.5)	0.0146 (0.0115–0.0184)	517.9 (407.9–652.9)	193.4 (173.2–215.5)	19.8 (12.3–28.3)
16	Headache disorders	2.0 (0.4–3.9)	0.0131 (0.00278–0.0276)	683.4 (136.1–1445.3)	77.3 (67.7–93.1)	-3.0 (-8.2–2.5)
17	Chronic kidney disease	2.0 (1.7–2.2)	0.0130 (0.0113–0.0147)	447.2 (392.6–503.4)	400.3 (341.0–464.7)	105.4 (81.6–131.2)
18	Age-related and other hearing loss	1.8 (1.3–2.3)	0.0120 (0.00832–0.0165)	421.6 (294.5–584.6)	122.6 (114.2–131.9)	-5.7 (-9.0—2.2)
19	Cirrhosis and other chronic liver diseases	1.5 (1.3–1.7)	0.00987 (0.00830–0.0114)	398.1 (334.4–459.8)	231.6 (178.9–285.5)	46.6 (22.9–70.8)
20	Osteoarthritis	1.3 (0.7–2.5)	0.00868 (0.00421–0.0175)	299.8 (144.3–601.8)	165.3 (157.4–173.3)	7.6 (4.3–10.7)
21	Colon and rectum cancer	1.2 (1.0–1.3)	0.00767 (0.00639–0.00909)	268.8 (224.3–319.0)	68.9 (38.1–100.0)	-28.4 (-41.7—15.2)
22	Asthma	1.1 (0.8–1.5)	0.00754 (0.00516–0.0107)	389.8 (264.5–560.2)	65.7 (53.3–79.5)	-10.7 (-16.4—3.8)
23	Alcohol use disorders	1.1 (1.0–1.3)	0.00739 (0.00592–0.00915)	366.6 (290.4–457.7)	91.0 (70.4–118.5)	-1.1 (-11.1–11.7)
24	Neonatal disorders	1.1 (0.9–1.2)	0.00698 (0.00588–0.00819)	519.7 (437.5–610.8)	-5.8 (-18.8–9.5)	-38.4 (-47.2—27.8)
25	Oral disorders	1.0 (0.7–1.4)	0.00683 (0.00413–0.0104)	274.5 (159.6–423.1)	107.3 (89.3–123.7)	-5.4 (-13.5–2.9)

Table S4: Cause-specific DALYs by US state in 2021 and percentage change between 1990 and 2021, Illinois

Rank	Cause Name	2021 Percentage of all cause DALYs	2021 DALYs (millions)	2021 Age Standardised Rate (per 100 000)	Percentage change DALY count 1990 to 2021	Percentage change age-standardised DALY rate 1990 to 2021
	All causes	100.0 (100.0–100.0)	4.62 (4.08–5.26)	27225.0 (23752.3–31172.9)	18.5 (9.9–27.3)	-8.2 (-14.5–-2.0)
1	Ischaemic heart disease	7.1 (6.0–8.3)	0.328 (0.280–0.381)	1485.6 (1274.5–1729.6)	-37.7 (-45.8–-28.8)	-58.5 (-64.1–-52.3)
2	COVID-19	6.7 (5.8–7.9)	0.310 (0.293–0.350)	1725.7 (1609.7–1987.2)	--	--
3	Drug use disorders	5.1 (4.4–5.8)	0.235 (0.195–0.279)	1820.2 (1503.9–2158.4)	393.5 (333.3–469.1)	385.7 (327.9–457.5)
4	Low back pain	4.0 (3.2–4.9)	0.188 (0.136–0.242)	1184.1 (856.3–1539.1)	15.3 (6.7–23.6)	-5.6 (-12.1–1.5)
5	Diabetes mellitus	3.9 (3.4–4.5)	0.180 (0.143–0.226)	888.2 (706.9–1109.4)	105.1 (82.9–125.0)	41.5 (26.1–54.9)
6	Chronic obstructive pulmonary disease	3.7 (3.2–4.1)	0.168 (0.149–0.190)	738.8 (656.5–830.2)	63.5 (46.6–82.8)	7.2 (-4.0–19.8)
7	Stroke	3.4 (2.9–3.8)	0.155 (0.135–0.177)	707.0 (621.5–809.7)	7.0 (-4.8–19.4)	-28.4 (-36.3–-20.2)
8	Other musculoskeletal disorders	3.3 (2.5–4.3)	0.154 (0.111–0.205)	1014.8 (732.8–1350.7)	75.8 (56.2–98.2)	48.6 (32.4–65.9)
9	Tracheal, bronchus, and lung cancer	3.2 (2.7–3.7)	0.146 (0.122–0.171)	659.0 (553.4–773.1)	-17.8 (-31.6–-2.9)	-48.6 (-57.3–-39.0)
10	Alzheimer's disease and other dementias	2.7 (1.3–5.7)	0.127 (0.0588–0.267)	500.6 (234.3–1053.3)	52.8 (40.2–66.9)	-4.5 (-12.2–4.3)
11	Chronic kidney disease	2.6 (2.3–3.0)	0.121 (0.105–0.139)	573.4 (500.7–656.9)	182.9 (147.4–224.7)	88.0 (65.8–115.0)
12	Depressive disorders	2.5 (1.7–3.4)	0.114 (0.0780–0.163)	876.0 (592.0–1244.2)	55.3 (24.5–86.1)	48.2 (18.6–77.6)
13	Anxiety disorders	2.3 (1.6–3.2)	0.107 (0.0690–0.156)	794.6 (510.0–1183.0)	40.5 (7.4–79.3)	29.6 (-1.0–64.1)
14	Headache disorders	2.0 (0.5–4.0)	0.0926 (0.0202–0.195)	687.3 (136.9–1449.0)	5.7 (-0.5–16.4)	-3.2 (-8.9–2.4)
15	Falls	1.9 (1.6–2.2)	0.0865 (0.0691–0.109)	423.4 (336.8–532.7)	42.5 (31.8–55.3)	-5.5 (-11.7–2.1)
16	Age-related and other hearing loss	1.8 (1.4–2.4)	0.0853 (0.0594–0.119)	419.0 (292.3–583.6)	34.4 (29.1–39.8)	-5.6 (-9.0–-2.1)
17	Road injuries	1.6 (1.5–1.8)	0.0756 (0.0662–0.0858)	545.6 (479.9–616.1)	-44.3 (-49.9–-38.4)	-51.7 (-56.8–-46.3)
18	Colon and rectum cancer	1.5 (1.3–1.7)	0.0680 (0.0566–0.0804)	327.5 (271.3–387.8)	-10.1 (-25.1–6.6)	-38.0 (-48.6–-26.4)
19	Self-harm	1.5 (1.3–1.7)	0.0680 (0.0577–0.0799)	505.3 (429.8–592.6)	11.2 (-6.5–31.0)	3.5 (-13.1–21.4)
20	Osteoarthritis	1.4 (0.7–2.7)	0.0648 (0.0315–0.130)	308.5 (148.4–618.9)	58.3 (53.8–62.6)	5.7 (2.7–8.6)
21	Interpersonal violence	1.4 (1.2–1.6)	0.0635 (0.0547–0.0719)	550.6 (474.6–622.7)	-30.3 (-39.6–-20.2)	-28.7 (-38.3–-18.5)
22	Cirrhosis and other chronic liver diseases	1.3 (1.1–1.5)	0.0611 (0.0523–0.0715)	332.2 (286.5–390.1)	6.5 (-9.5–25.1)	-24.2 (-35.7–-10.9)
23	Neonatal disorders	1.3 (1.1–1.5)	0.0598 (0.0517–0.0685)	781.6 (691.6–881.3)	-51.3 (-56.7–-45.6)	-37.6 (-44.3–-30.2)
24	Breast cancer	1.3 (1.0–1.5)	0.0579 (0.0466–0.0704)	290.5 (232.8–356.6)	-18.8 (-34.3–-1.7)	-45.1 (-55.8–-33.3)
25	Hypertensive heart disease	1.3 (1.0–1.4)	0.0577 (0.0482–0.0679)	285.8 (238.7–336.3)	115.3 (81.2–153.9)	49.2 (25.9–76.7)

Table S4: Cause-specific DALYs by US state in 2021 and percentage change between 1990 and 2021, Indiana						
Rank	Cause Name	2021 Percentage of all cause DALYs	2021 DALYs (millions)	2021 Age Standardised Rate (per 100 000)	Percentage change DALY count 1990 to 2021	Percentage change age-standardised DALY rate 1990 to 2021
	All causes	100.0 (100.0–100.0)	2.80 (2.46–3.14)	31532.1 (27567.8–35645.5)	51.6 (42.1–63.0)	9.4 (2.9–16.9)
1	COVID-19	8.3 (7.2–9.9)	0.230 (0.217–0.271)	2451.1 (2289.6–2874.0)	--	--
2	Ischaemic heart disease	7.1 (6.0–8.1)	0.198 (0.169–0.229)	1757.9 (1494.6–2023.7)	-21.8 (-32.0—10.7)	-50.3 (-57.0—43.0)
3	Drug use disorders	6.1 (5.3–6.8)	0.169 (0.143–0.197)	2549.2 (2152.7–2964.5)	995.1 (830.2–1241.8)	897.2 (747.7–1115.3)
4	Chronic obstructive pulmonary disease	4.5 (4.0–5.0)	0.127 (0.112–0.141)	1072.4 (950.1–1191.5)	119.2 (95.7–145.3)	34.6 (20.2–50.5)
5	Diabetes mellitus	4.1 (3.6–4.8)	0.116 (0.0917–0.145)	1090.5 (860.0–1356.8)	146.4 (118.5–169.6)	58.4 (41.2–73.2)
6	Low back pain	3.8 (3.0–4.7)	0.107 (0.0780–0.140)	1283.2 (942.6–1687.9)	27.2 (18.3–36.5)	-3.6 (-9.5–3.3)
7	Tracheal, bronchus, and lung cancer	3.4 (2.9–3.9)	0.0953 (0.0801–0.111)	823.6 (692.3–962.7)	3.5 (-11.8–20.1)	-38.9 (-48.1—28.8)
8	Stroke	3.2 (2.9–3.5)	0.0904 (0.0789–0.102)	810.9 (707.1–911.1)	21.4 (8.5–35.7)	-21.7 (-29.7—12.6)
9	Other musculoskeletal disorders	3.0 (2.3–3.8)	0.0831 (0.0599–0.110)	1027.9 (737.8–1362.8)	91.6 (70.4–115.2)	48.0 (32.3–66.5)
10	Depressive disorders	2.7 (1.9–3.8)	0.0767 (0.0498–0.111)	1110.4 (719.4–1603.0)	79.0 (41.7–118.6)	56.3 (22.9–92.5)
11	Chronic kidney disease	2.6 (2.3–2.8)	0.0714 (0.0628–0.0800)	653.9 (576.2–733.1)	268.5 (225.8–318.4)	130.9 (105.4–160.5)
12	Alzheimer's disease and other dementias	2.4 (1.2–5.0)	0.0675 (0.0328–0.137)	536.8 (261.3–1083.6)	57.7 (45.0–70.9)	-3.6 (-11.2–4.5)
13	Anxiety disorders	2.0 (1.4–2.8)	0.0559 (0.0369–0.0802)	788.0 (516.6–1125.1)	51.5 (18.9–96.9)	29.0 (1.3–68.4)
14	Road injuries	1.9 (1.8–2.1)	0.0543 (0.0471–0.0621)	756.0 (660.0–858.0)	-28.9 (-36.2—20.9)	-42.3 (-48.3—35.7)
15	Headache disorders	1.7 (0.4–3.5)	0.0483 (0.0104–0.102)	683.3 (135.8–1446.3)	13.8 (7.5–25.3)	-3.6 (-8.6–2.0)
16	Self-harm	1.7 (1.5–2.0)	0.0482 (0.0411–0.0565)	688.0 (587.5–803.9)	30.7 (10.9–52.5)	13.0 (-4.2–32.1)
17	Falls	1.7 (1.4–1.9)	0.0464 (0.0373–0.0591)	440.1 (353.5–557.5)	81.5 (68.2–97.9)	14.4 (6.5–24.4)
18	Age-related and other hearing loss	1.6 (1.2–2.1)	0.0445 (0.0311–0.0615)	417.7 (293.7–580.6)	44.5 (39.4–50.7)	-5.6 (-8.5—2.1)
19	Cirrhosis and other chronic liver diseases	1.5 (1.3–1.7)	0.0420 (0.0357–0.0483)	441.9 (376.8–510.9)	120.9 (89.8–158.1)	49.8 (28.5–74.6)
20	Colon and rectum cancer	1.4 (1.2–1.6)	0.0388 (0.0327–0.0459)	360.4 (304.4–426.1)	13.2 (-5.8–34.6)	-26.4 (-38.9—12.6)
21	Osteoarthritis	1.3 (0.7–2.5)	0.0356 (0.0173–0.0717)	323.2 (156.6–648.8)	69.3 (64.7–74.6)	5.3 (2.4–8.4)
22	Neonatal disorders	1.1 (1.0–1.3)	0.0321 (0.0280–0.0366)	718.9 (634.8–811.0)	-31.6 (-39.0—23.0)	-30.8 (-38.4—22.1)
23	Endocrine, metabolic, blood, and immune disorders	1.1 (1.0–1.2)	0.0302 (0.0260–0.0347)	336.7 (290.9–390.1)	163.2 (129.6–204.9)	78.5 (57.5–103.6)
24	Breast cancer	1.1 (0.9–1.3)	0.0295 (0.0241–0.0357)	285.8 (231.0–348.3)	-7.0 (-25.1–13.5)	-40.3 (-52.4—26.5)
25	Asthma	1.0 (0.8–1.4)	0.0292 (0.0200–0.0411)	436.4 (297.6–626.0)	21.9 (14.6–30.4)	1.1 (-5.1–8.3)

Table S4: Cause-specific DALYs by US state in 2021 and percentage change between 1990 and 2021, Iowa

Rank	Cause Name	2021 Percentage of all cause DALYs	2021 DALYs (millions)	2021 Age Standardised Rate (per 100 000)	Percentage change DALY count 1990 to 2021	Percentage change age-standardised DALY rate 1990 to 2021
	All causes	100.0 (100.0–100.0)	1.16 (1.02–1.33)	26403.7 (22824.1–30498.0)	30.2 (20.6–40.0)	3.6 (-3.4–10.5)
1	Ischaemic heart disease	8.2 (7.0–9.4)	0.0946 (0.0799–0.110)	1623.3 (1366.4–1899.7)	-27.7 (-38.1–-16.4)	-46.1 (-54.2–-37.3)
2	COVID-19	7.5 (6.6–8.6)	0.0870 (0.0835–0.0943)	1889.9 (1782.3–2103.9)	--	--
3	Low back pain	4.5 (3.6–5.4)	0.0528 (0.0379–0.0692)	1374.3 (980.7–1805.8)	13.3 (6.9–20.3)	-5.5 (-11.2–0.4)
4	Chronic obstructive pulmonary disease	4.3 (3.7–4.8)	0.0497 (0.0435–0.0555)	814.7 (711.7–909.1)	70.0 (50.1–89.4)	19.9 (6.0–33.5)
5	Diabetes mellitus	3.8 (3.3–4.4)	0.0446 (0.0353–0.0564)	854.8 (675.2–1084.8)	122.0 (98.4–143.7)	63.5 (47.0–78.9)
6	Other musculoskeletal disorders	3.6 (2.8–4.6)	0.0424 (0.0300–0.0562)	1160.0 (819.5–1557.0)	75.9 (60.3–93.4)	51.2 (37.3–67.1)
7	Tracheal, bronchus, and lung cancer	3.3 (2.8–3.8)	0.0384 (0.0318–0.0449)	667.7 (553.1–782.9)	0.5 (-16.6–17.1)	-33.2 (-44.8–-21.9)
8	Alzheimer's disease and other dementias	3.1 (1.5–6.6)	0.0362 (0.0166–0.0795)	513.1 (240.1–1119.4)	31.6 (18.5–45.2)	-1.8 (-11.0–7.8)
9	Stroke	3.0 (2.7–3.4)	0.0353 (0.0302–0.0399)	606.3 (520.8–687.7)	-1.0 (-12.0–11.3)	-24.0 (-32.6–-14.6)
10	Drug use disorders	2.9 (2.4–3.3)	0.0333 (0.0269–0.0399)	1098.3 (886.0–1320.6)	457.5 (379.7–554.9)	424.9 (350.7–518.6)
11	Falls	2.6 (2.2–3.0)	0.0298 (0.0242–0.0379)	548.5 (439.3–699.8)	80.1 (67.6–95.3)	24.4 (15.6–34.8)
12	Depressive disorders	2.5 (1.7–3.5)	0.0292 (0.0193–0.0420)	911.7 (595.7–1316.2)	56.6 (28.6–87.0)	44.6 (18.6–73.7)
13	Chronic kidney disease	2.2 (2.0–2.5)	0.0261 (0.0224–0.0295)	456.8 (392.1–514.0)	223.9 (182.0–272.2)	132.5 (102.9–167.0)
14	Anxiety disorders	2.1 (1.4–2.9)	0.0250 (0.0160–0.0351)	757.0 (485.4–1049.4)	37.5 (7.1–73.7)	24.1 (-3.4–57.2)
15	Road injuries	2.0 (1.9–2.2)	0.0238 (0.0204–0.0273)	698.4 (601.1–796.2)	-32.7 (-41.0–-25.1)	-43.4 (-50.9–-36.6)
16	Self-harm	2.0 (1.6–2.3)	0.0228 (0.0188–0.0272)	712.1 (591.8–847.3)	48.1 (21.9–76.1)	36.1 (12.2–62.0)
17	Age-related and other hearing loss	1.9 (1.5–2.5)	0.0225 (0.0156–0.0309)	421.2 (294.5–585.5)	26.6 (22.0–32.2)	-5.2 (-8.2–-1.4)
18	Headache disorders	1.9 (0.4–3.8)	0.0223 (0.00490–0.0469)	683.5 (136.4–1445.0)	7.8 (2.0–18.6)	-3.7 (-8.7–-1.5)
19	Colon and rectum cancer	1.4 (1.2–1.6)	0.0166 (0.0135–0.0196)	305.2 (249.6–360.0)	-7.9 (-25.1–9.8)	-31.4 (-44.1–-18.4)
20	Osteoarthritis	1.4 (0.7–2.7)	0.0160 (0.00778–0.0322)	293.2 (141.8–585.2)	50.4 (46.0–55.7)	7.0 (3.7–10.7)
21	Cirrhosis and other chronic liver diseases	1.3 (1.1–1.5)	0.0148 (0.0124–0.0174)	332.0 (275.1–390.8)	105.6 (70.7–144.9)	55.1 (28.5–83.8)
22	Endocrine, metabolic, blood, and immune disorders	1.1 (1.0–1.3)	0.0130 (0.0111–0.0151)	293.5 (248.7–343.1)	152.0 (113.2–195.1)	80.6 (55.8–109.2)
23	Breast cancer	1.1 (0.9–1.3)	0.0125 (0.00989–0.0154)	248.7 (195.3–307.0)	-15.0 (-32.0–5.2)	-39.1 (-52.0–-24.3)
24	Asthma	1.1 (0.8–1.4)	0.0123 (0.00838–0.0174)	379.8 (259.1–553.2)	10.3 (2.6–19.4)	-2.0 (-9.1–6.6)
25	Alcohol use disorders	1.0 (0.9–1.2)	0.0121 (0.00971–0.0151)	346.6 (276.0–441.3)	23.1 (7.6–45.5)	4.4 (-7.8–21.7)

Table S4: Cause-specific DALYs by US state in 2021 and percentage change between 1990 and 2021, Kansas						
Rank	Cause Name	2021 Percentage of all cause DALYs	2021 DALYs (millions)	2021 Age Standardised Rate (per 100 000)	Percentage change DALY count 1990 to 2021	Percentage change age-standardised DALY rate 1990 to 2021
	All causes	100.0 (100.0–100.0)	1.11 (0.970–1.25)	28937.9 (25089.0–32706.5)	41.1 (30.7–52.7)	8.2 (1.0–15.7)
1	COVID-19	8.8 (7.7–10.0)	0.0970 (0.0940–0.104)	2398.6 (2303.2–2617.9)	--	--
2	Ischaemic heart disease	7.4 (6.3–8.4)	0.0817 (0.0690–0.0945)	1648.1 (1386.4–1927.9)	-20.5 (-32.9—7.4)	-44.1 (-53.2—34.4)
3	Chronic obstructive pulmonary disease	4.3 (3.8–4.8)	0.0477 (0.0419–0.0538)	925.5 (816.1–1042.4)	84.7 (63.5–108.2)	25.3 (11.0–41.1)
4	Low back pain	4.1 (3.2–5.0)	0.0452 (0.0331–0.0591)	1279.7 (933.6–1686.7)	17.7 (10.8–25.8)	-7.1 (-12.2—0.7)
5	Diabetes mellitus	3.9 (3.5–4.5)	0.0438 (0.0347–0.0545)	965.4 (770.1–1201.0)	141.9 (116.8–165.3)	68.2 (51.3–84.5)
6	Drug use disorders	3.6 (3.0–4.2)	0.0406 (0.0325–0.0487)	1422.8 (1133.7–1706.3)	468.2 (387.4–573.4)	422.7 (348.7–516.7)
7	Tracheal, bronchus, and lung cancer	3.2 (2.7–3.7)	0.0352 (0.0295–0.0415)	715.4 (598.3–843.2)	1.4 (-15.2–19.8)	-36.1 (-46.8—24.3)
8	Other musculoskeletal disorders	3.1 (2.4–4.0)	0.0349 (0.0251–0.0464)	1029.0 (733.7–1365.9)	80.1 (64.0–99.3)	45.1 (31.2–60.4)
9	Stroke	3.1 (2.7–3.4)	0.0340 (0.0296–0.0387)	695.1 (608.0–786.8)	8.1 (-4.3–21.0)	-21.4 (-30.6—11.9)
10	Depressive disorders	2.7 (2.0–3.7)	0.0303 (0.0203–0.0432)	1022.5 (688.1–1459.0)	69.0 (37.8–105.7)	50.3 (22.3–84.3)
11	Alzheimer's disease and other dementias	2.6 (1.2–5.4)	0.0287 (0.0133–0.0622)	498.8 (231.5–1083.2)	34.0 (21.3–48.1)	-3.7 (-12.6–6.3)
12	Chronic kidney disease	2.4 (2.1–2.6)	0.0264 (0.0228–0.0301)	554.3 (483.2–628.3)	240.7 (196.2–288.2)	138.7 (108.0–170.7)
13	Road injuries	2.3 (2.1–2.5)	0.0255 (0.0218–0.0293)	825.9 (711.1–942.1)	-25.0 (-34.4—15.0)	-38.3 (-46.2—29.9)
14	Self-harm	2.2 (1.8–2.5)	0.0242 (0.0200–0.0285)	822.1 (677.9–966.6)	61.1 (31.7–92.4)	43.7 (17.9–71.5)
15	Anxiety disorders	2.1 (1.4–3.1)	0.0239 (0.0149–0.0350)	785.7 (488.9–1147.3)	48.0 (16.6–89.0)	28.9 (2.1–64.4)
16	Falls	2.1 (1.9–2.5)	0.0239 (0.0191–0.0302)	513.9 (411.1–651.9)	92.5 (77.1–110.2)	29.1 (18.4–40.9)
17	Headache disorders	1.8 (0.4–3.7)	0.0207 (0.00445–0.0434)	682.7 (135.3–1441.8)	12.2 (6.4–22.3)	-3.3 (-8.2–2.3)
18	Age-related and other hearing loss	1.7 (1.3–2.2)	0.0192 (0.0135–0.0268)	419.4 (296.4–584.3)	31.0 (25.5–36.7)	-5.7 (-9.0—2.0)
19	Cirrhosis and other chronic liver diseases	1.5 (1.3–1.7)	0.0163 (0.0135–0.0194)	409.9 (337.8–486.1)	121.0 (79.5–161.7)	57.0 (28.2–86.5)
20	Colon and rectum cancer	1.3 (1.1–1.5)	0.0148 (0.0123–0.0176)	320.3 (264.5–381.5)	3.0 (-14.6–23.8)	-26.4 (-39.2—11.0)
21	Osteoarthritis	1.2 (0.6–2.4)	0.0138 (0.00674–0.0277)	294.0 (142.6–585.0)	57.3 (52.8–62.0)	6.6 (3.5–9.8)
22	Neonatal disorders	1.2 (1.0–1.4)	0.0132 (0.0111–0.0153)	693.0 (591.7–806.0)	-21.5 (-32.1—8.5)	-15.9 (-28.1—1.0)
23	Breast cancer	1.1 (0.9–1.4)	0.0127 (0.0103–0.0158)	287.5 (233.5–360.6)	-0.3 (-20.0–23.4)	-32.0 (-45.7—15.2)
24	Asthma	1.1 (0.8–1.5)	0.0122 (0.00828–0.0176)	422.0 (284.7–621.7)	18.5 (10.5–27.0)	2.5 (-4.8–9.8)
25	Alcohol use disorders	1.1 (1.0–1.2)	0.0120 (0.00962–0.0147)	371.6 (295.3–462.4)	23.2 (7.8–43.3)	2.1 (-10.0–17.2)

Table S4: Cause-specific DALYs by US state in 2021 and percentage change between 1990 and 2021, Kentucky

Rank	Cause Name	2021 Percentage of all cause DALYs	2021 DALYs (millions)	2021 Age Standardised Rate (per 100 000)	Percentage change DALY count 1990 to 2021	Percentage change age-standardised DALY rate 1990 to 2021
	All causes	100.0 (100.0–100.0)	2.13 (1.88–2.37)	35579.4 (31286.0–39980.1)	63.4 (51.2–76.9)	17.0 (8.4–26.3)
1	COVID-19	10.0 (8.7–11.8)	0.212 (0.202–0.241)	3351.4 (3163.0–3796.6)	--	--
2	Ischaemic heart disease	7.4 (6.4–8.4)	0.157 (0.132–0.184)	2104.1 (1765.4–2453.4)	-14.8 (-26.6—1.6)	-46.0 (-53.6—37.2)
3	Drug use disorders	6.9 (6.0–8.0)	0.148 (0.120–0.174)	3340.8 (2722.5–3960.9)	1072.3 (890.4–1290.9)	971.1 (807.6–1159.6)
4	Chronic obstructive pulmonary disease	4.7 (4.2–5.3)	0.100 (0.0877–0.114)	1251.7 (1095.9–1421.9)	124.2 (98.6–152.0)	35.2 (19.6–51.9)
5	Diabetes mellitus	3.9 (3.5–4.5)	0.0838 (0.0682–0.104)	1173.5 (959.6–1441.6)	155.8 (130.1–179.9)	64.3 (48.0–79.5)
6	Tracheal, bronchus, and lung cancer	3.9 (3.3–4.5)	0.0825 (0.0699–0.0973)	1050.7 (887.5–1240.4)	8.2 (-9.7–27.5)	-37.5 (-47.8—26.3)
7	Low back pain	3.7 (2.9–4.5)	0.0787 (0.0571–0.102)	1416.6 (1028.1–1839.4)	32.8 (23.4–43.2)	0.5 (-6.1–8.4)
8	Stroke	3.1 (2.8–3.5)	0.0667 (0.0573–0.0759)	894.1 (765.8–1016.8)	29.5 (15.4–45.4)	-16.6 (-25.8—6.2)
9	Other musculoskeletal disorders	2.6 (2.0–3.3)	0.0554 (0.0398–0.0731)	1039.5 (752.0–1374.9)	96.2 (76.5–120.9)	53.4 (37.6–73.6)
10	Depressive disorders	2.5 (1.7–3.3)	0.0528 (0.0349–0.0737)	1170.0 (768.7–1627.6)	87.7 (55.5–124.8)	67.8 (38.7–102.0)
11	Chronic kidney disease	2.4 (2.2–2.7)	0.0517 (0.0437–0.0590)	702.9 (601.4–801.3)	286.8 (236.8–343.7)	139.8 (109.9–173.4)
12	Road injuries	2.1 (2.0–2.3)	0.0452 (0.0389–0.0512)	949.8 (825.1–1074.6)	-23.9 (-33.2—14.9)	-38.0 (-45.9—30.0)
13	Alzheimer's disease and other dementias	2.0 (0.9–4.2)	0.0417 (0.0194–0.0894)	493.7 (229.9–1050.8)	59.1 (46.6–74.0)	-4.3 (-11.8–5.0)
14	Anxiety disorders	1.8 (1.2–2.5)	0.0390 (0.0247–0.0565)	835.3 (530.8–1223.0)	57.7 (24.6–99.3)	36.4 (6.6–74.6)
15	Self-harm	1.7 (1.5–2.0)	0.0363 (0.0309–0.0427)	784.0 (668.5–919.2)	45.9 (22.4–71.4)	27.9 (7.7–49.9)
16	Falls	1.7 (1.5–2.0)	0.0362 (0.0292–0.0460)	507.2 (409.2–643.0)	85.4 (70.2–102.6)	15.2 (6.3–25.6)
17	Cirrhosis and other chronic liver diseases	1.6 (1.4–1.9)	0.0350 (0.0295–0.0410)	544.2 (458.1–637.3)	144.7 (106.3–187.0)	63.4 (37.5–92.5)
18	Headache disorders	1.5 (0.3–3.0)	0.0316 (0.00701–0.0670)	676.9 (135.1–1434.3)	11.7 (5.6–24.7)	-4.4 (-9.3–1.0)
19	Colon and rectum cancer	1.4 (1.2–1.6)	0.0303 (0.0253–0.0362)	415.4 (346.5–497.3)	22.7 (0.3–45.0)	-21.3 (-35.5—6.5)
20	Age-related and other hearing loss	1.4 (1.0–1.9)	0.0302 (0.0211–0.0418)	417.4 (292.5–574.8)	46.5 (40.8–52.4)	-5.7 (-9.0—2.4)
21	Osteoarthritis	1.1 (0.6–2.2)	0.0245 (0.0120–0.0492)	325.5 (158.9–650.5)	72.3 (68.0–77.4)	5.3 (2.3–8.5)
22	Breast cancer	1.1 (0.9–1.3)	0.0230 (0.0187–0.0286)	330.8 (268.0–413.2)	6.9 (-12.1–32.1)	-32.2 (-44.8—15.0)
23	Hypertensive heart disease	1.0 (0.9–1.2)	0.0221 (0.0187–0.0263)	318.5 (268.6–379.1)	248.8 (197.3–315.1)	134.3 (99.6–180.4)
24	Endocrine, metabolic, blood, and immune disorders	1.0 (0.9–1.1)	0.0208 (0.0177–0.0239)	348.3 (298.5–404.4)	176.9 (135.6–226.3)	88.4 (62.6–119.2)
25	Neonatal disorders	0.9 (0.8–1.1)	0.0195 (0.0161–0.0229)	669.2 (560.7–790.3)	-19.7 (-32.1—5.2)	-20.8 (-33.8—5.3)

Table S4: Cause-specific DALYs by US state in 2021 and percentage change between 1990 and 2021, Louisiana						
**Rank**	**Cause Name**	**2021 Percentage of all cause DALYs**	**2021 DALYs (millions)**	**2021 Age Standardised Rate (per 100 000)**	**Percentage change DALY count 1990 to 2021**	**Percentage change age-standardised DALY rate 1990 to 2021**
	All causes	100.0 (100.0–100.0)	2.08 (1.84–2.34)	35065.7 (30892.0–39666.2)	38.5 (28.3–50.5)	6.7 (-0.3–15.1)
1	COVID-19	10.2 (8.2–12.8)	0.212 (0.178–0.259)	3373.3 (2832.4–4116.3)	--	--
2	Ischaemic heart disease	7.4 (6.6–8.4)	0.155 (0.133–0.178)	2115.7 (1824.2–2435.2)	-19.9 (-30.8–-8.0)	-47.1 (-54.3–-39.0)
3	Drug use disorders	5.5 (4.6–6.3)	0.114 (0.0935–0.137)	2453.1 (2009.4–2940.1)	645.6 (534.5–779.5)	635.4 (528.2–766.5)
4	Diabetes mellitus	3.9 (3.5–4.5)	0.0822 (0.0654–0.101)	1170.8 (934.9–1446.1)	93.4 (68.7–115.4)	31.4 (14.8–46.6)
5	Chronic obstructive pulmonary disease	3.6 (3.2–4.0)	0.0745 (0.0659–0.0830)	959.8 (853.3–1070.7)	103.7 (81.2–127.9)	30.4 (16.1–45.9)
6	Low back pain	3.5 (2.7–4.2)	0.0723 (0.0524–0.0939)	1266.4 (914.1–1649.7)	16.9 (9.6–25.6)	-6.2 (-11.7–0.3)
7	Stroke	3.3 (3.0–3.6)	0.0684 (0.0599–0.0777)	949.1 (832.0–1079.4)	18.3 (6.0–32.6)	-20.9 (-29.3–-11.0)
8	Tracheal, bronchus, and lung cancer	3.3 (2.8–3.7)	0.0677 (0.0580–0.0796)	886.2 (759.2–1042.2)	-8.2 (-22.2–9.9)	-43.2 (-51.9–-31.8)
9	Chronic kidney disease	2.8 (2.5–3.1)	0.0581 (0.0509–0.0661)	827.7 (726.2–938.2)	203.0 (166.0–239.9)	103.7 (78.9–128.3)
10	Other musculoskeletal disorders	2.5 (1.9–3.2)	0.0518 (0.0379–0.0681)	933.6 (683.1–1231.0)	73.4 (57.1–92.3)	42.4 (28.7–58.0)
11	Road injuries	2.3 (2.1–2.5)	0.0481 (0.0421–0.0553)	997.5 (878.3–1141.5)	-30.0 (-37.5–-20.7)	-36.1 (-42.9–-27.3)
12	Depressive disorders	2.2 (1.5–3.0)	0.0456 (0.0309–0.0656)	973.4 (654.1–1413.6)	58.3 (30.2–93.5)	52.3 (24.8–85.5)
13	Alzheimer's disease and other dementias	1.9 (0.9–4.1)	0.0393 (0.0182–0.0865)	494.1 (228.8–1082.4)	54.3 (42.0–70.1)	-3.4 (-11.0–6.1)
14	Anxiety disorders	1.9 (1.2–2.7)	0.0389 (0.0252–0.0572)	804.4 (520.9–1194.3)	40.9 (9.1–76.9)	31.2 (1.6–64.6)
15	Interpersonal violence	1.8 (1.6–2.0)	0.0367 (0.0318–0.0425)	864.3 (753.6–999.5)	-23.0 (-33.6–-11.2)	-20.0 (-30.7–-7.9)
16	Self-harm	1.7 (1.5–1.9)	0.0352 (0.0298–0.0415)	737.9 (627.1–868.6)	25.1 (5.8–47.2)	18.5 (0.3–39.5)
17	Headache disorders	1.6 (0.4–3.3)	0.0329 (0.00714–0.0693)	682.4 (135.9–1441.5)	3.3 (-2.8–15.5)	-4.2 (-9.4–1.8)
18	Hypertensive heart disease	1.5 (1.3–1.8)	0.0318 (0.0269–0.0379)	469.9 (399.2–563.3)	114.8 (79.5–156.0)	51.0 (26.3–79.9)
19	Colon and rectum cancer	1.5 (1.3–1.7)	0.0306 (0.0257–0.0360)	429.9 (359.4–506.7)	23.5 (3.4–46.5)	-16.4 (-30.2–-0.6)
20	Age-related and other hearing loss	1.4 (1.0–1.9)	0.0295 (0.0206–0.0410)	418.5 (294.1–580.5)	37.7 (32.6–43.7)	-5.4 (-8.6–-1.8)
21	Falls	1.4 (1.2–1.6)	0.0289 (0.0232–0.0367)	421.0 (337.5–530.5)	59.0 (47.1–72.8)	8.4 (1.1–17.3)
22	Cirrhosis and other chronic liver diseases	1.4 (1.2–1.6)	0.0289 (0.0246–0.0339)	450.2 (383.4–528.4)	54.4 (31.7–82.3)	9.4 (-6.9–29.6)
23	Neonatal disorders	1.3 (1.1–1.5)	0.0270 (0.0234–0.0304)	842.3 (740.1–936.2)	-40.9 (-47.2–-34.2)	-33.2 (-40.2–-25.7)
24	Breast cancer	1.2 (0.9–1.4)	0.0240 (0.0195–0.0291)	351.5 (285.3–427.7)	-3.0 (-21.9–19.4)	-34.2 (-47.3–-18.4)
25	Osteoarthritis	1.1 (0.6–2.1)	0.0231 (0.0113–0.0467)	316.0 (153.3–636.1)	62.3 (57.6–67.7)	5.5 (2.6–8.7)

Table S4: Cause-specific DALYs by US state in 2021 and percentage change between 1990 and 2021, Maine						
**Rank**	**Cause Name**	**2021 Percentage of all cause DALYs**	**2021 DALYs (millions)**	**2021 Age Standardised Rate (per 100 000)**	**Percentage change DALY count 1990 to 2021**	**Percentage change age-standardised DALY rate 1990 to 2021**
	All causes	100.0 (100.0–100.0)	0.576 (0.505–0.661)	28327.4 (24719.2–32739.7)	45.6 (34.3–56.8)	5.4 (-2.4–13.0)
1	Ischaemic heart disease	6.6 (5.7–7.6)	0.0381 (0.0323–0.0443)	1296.6 (1094.2–1507.1)	-28.7 (-39.3—18.0)	-58.9 (-65.1—52.3)
2	COVID-19	5.6 (4.1–7.5)	0.0320 (0.0243–0.0425)	1436.9 (1084.9–1921.1)	--	--
3	Drug use disorders	5.1 (4.5–5.8)	0.0295 (0.0246–0.0345)	2375.7 (1977.6–2774.8)	707.2 (591.2–865.1)	758.7 (638.2–920.7)
4	Chronic obstructive pulmonary disease	4.7 (4.2–5.3)	0.0272 (0.0240–0.0304)	881.5 (780.1–985.9)	103.2 (81.7–126.1)	13.0 (1.1–25.7)
5	Diabetes mellitus	4.4 (3.9–5.1)	0.0256 (0.0205–0.0321)	968.0 (776.1–1218.1)	161.4 (133.7–190.0)	56.5 (40.0–73.7)
6	Low back pain	4.0 (3.1–4.8)	0.0230 (0.0167–0.0298)	1253.5 (913.0–1634.8)	20.8 (11.9–30.7)	-6.4 (-12.4–0.5)
7	Tracheal, bronchus, and lung cancer	4.0 (3.4–4.7)	0.0229 (0.0192–0.0269)	785.8 (660.0–923.6)	11.9 (-7.8–32.3)	-39.8 (-50.5—28.5)
8	Other musculoskeletal disorders	3.6 (2.7–4.6)	0.0207 (0.0148–0.0276)	1221.3 (877.1–1626.0)	86.3 (69.3–107.0)	54.0 (40.2–70.9)
9	Alzheimer's disease and other dementias	3.0 (1.4–6.3)	0.0172 (0.00783–0.0362)	502.2 (231.9–1060.3)	74.4 (59.7–92.5)	-3.8 (-11.8–5.7)
10	Stroke	2.9 (2.6–3.2)	0.0169 (0.0146–0.0191)	595.3 (516.4–671.9)	19.7 (7.7–33.1)	-27.8 (-35.5—19.7)
11	Falls	2.5 (2.2–2.9)	0.0145 (0.0118–0.0182)	541.6 (437.7–681.4)	118.8 (99.7–141.8)	25.3 (15.4–38.5)
12	Depressive disorders	2.3 (1.6–3.2)	0.0133 (0.00859–0.0184)	980.3 (635.4–1376.9)	45.2 (16.6–77.2)	42.8 (15.3–73.7)
13	Chronic kidney disease	2.3 (2.0–2.6)	0.0131 (0.0113–0.0148)	479.1 (416.4–537.8)	258.9 (219.4–307.3)	113.4 (90.8–141.1)
14	Self-harm	1.9 (1.7–2.3)	0.0112 (0.00933–0.0134)	817.2 (683.8–972.6)	41.9 (16.9–70.3)	38.3 (14.8–65.3)
15	Age-related and other hearing loss	1.9 (1.5–2.5)	0.0111 (0.00780–0.0153)	417.9 (295.3–578.2)	55.7 (49.4–62.5)	-5.7 (-8.9—2.0)
16	Anxiety disorders	1.9 (1.2–2.6)	0.0108 (0.00646–0.0155)	755.4 (442.4–1101.4)	30.7 (0.5–64.1)	23.4 (-5.9–56.2)
17	Road injuries	1.8 (1.6–1.9)	0.0101 (0.00863–0.0117)	695.9 (599.5–791.5)	-32.5 (-39.9—25.1)	-40.7 (-47.7—33.7)
18	Headache disorders	1.7 (0.4–3.4)	0.00960 (0.00226–0.0201)	683.8 (139.1–1457.5)	2.0 (-4.3–17.8)	-3.4 (-8.3–2.5)
19	Cirrhosis and other chronic liver diseases	1.5 (1.3–1.8)	0.00882 (0.00739–0.0103)	404.7 (338.7–469.4)	103.3 (71.9–136.3)	37.5 (15.8–60.3)
20	Osteoarthritis	1.5 (0.8–2.7)	0.00841 (0.00411–0.0169)	303.6 (146.6–609.3)	86.1 (80.4–92.5)	6.2 (3.2–9.6)
21	Colon and rectum cancer	1.4 (1.2–1.7)	0.00817 (0.00680–0.00969)	306.8 (254.7–363.2)	2.8 (-15.2–21.9)	-36.6 (-47.9—24.6)
22	Endocrine, metabolic, blood, and immune disorders	1.3 (1.1–1.4)	0.00720 (0.00612–0.00836)	349.6 (298.9–408.1)	180.9 (141.2–229.0)	88.3 (63.9–116.6)
23	Alcohol use disorders	1.1 (1.0–1.3)	0.00651 (0.00538–0.00789)	416.2 (337.1–513.9)	29.0 (11.6–52.7)	12.6 (0.3–30.9)
24	Breast cancer	1.1 (0.9–1.4)	0.00650 (0.00536–0.00784)	262.8 (214.8–320.9)	-4.0 (-20.6–16.5)	-40.6 (-51.4—27.7)
25	Oral disorders	1.1 (0.7–1.5)	0.00627 (0.00380–0.00946)	281.7 (163.3–431.2)	42.1 (29.4–52.4)	-4.2 (-12.6–4.3)

Table S4: Cause-specific DALYs by US state in 2021 and percentage change between 1990 and 2021, Maryland						
**Rank**	**Cause Name**	**2021 Percentage of all cause DALYs**	**2021 DALYs (millions)**	**2021 Age Standardised Rate (per 100 000)**	**Percentage change DALY count 1990 to 2021**	**Percentage change age-standardised DALY rate 1990 to 2021**
	All causes	100.0 (100.0–100.0)	2.22 (1.93–2.55)	27233.6 (23547.6–31289.4)	42.8 (32.4–54.1)	-7.7 (-14.5—1.0)
1	Ischaemic heart disease	6.8 (5.7–7.9)	0.151 (0.126–0.176)	1404.9 (1170.1–1641.0)	-13.5 (-26.4—0.5)	-55.8 (-62.7—48.8)
2	COVID-19	5.8 (5.0–6.8)	0.129 (0.124–0.142)	1479.2 (1406.8–1664.1)	--	--
3	Drug use disorders	4.6 (4.0–5.3)	0.102 (0.0837–0.121)	1631.1 (1342.8–1933.5)	576.9 (453.9–741.5)	494.2 (387.7–631.0)
4	Diabetes mellitus	4.3 (3.7–4.9)	0.0950 (0.0749–0.119)	970.0 (762.1–1208.5)	142.0 (111.5–169.7)	35.5 (18.3–51.0)
5	Low back pain	3.8 (3.0–4.6)	0.0852 (0.0618–0.111)	1078.1 (782.2–1400.5)	30.1 (20.4–40.9)	-10.3 (-16.3—3.0)
6	Stroke	3.4 (3.0–3.8)	0.0751 (0.0647–0.0858)	716.0 (622.3–817.5)	42.5 (25.6–61.5)	-25.8 (-34.5—16.1)
7	Chronic obstructive pulmonary disease	3.2 (2.8–3.5)	0.0702 (0.0622–0.0789)	641.0 (570.3–717.9)	76.9 (59.3–95.8)	-10.0 (-18.8–0.0)
8	Other musculoskeletal disorders	3.1 (2.4–3.9)	0.0682 (0.0487–0.0905)	904.3 (650.9–1195.8)	93.0 (72.3–116.5)	38.0 (23.9–53.9)
9	Tracheal, bronchus, and lung cancer	2.8 (2.3–3.3)	0.0620 (0.0520–0.0731)	580.9 (485.4–684.9)	-20.0 (-32.8—5.6)	-59.1 (-65.7—51.7)
10	Alzheimer's disease and other dementias	2.7 (1.3–5.6)	0.0597 (0.0276–0.125)	493.2 (229.5–1037.9)	113.5 (94.2–135.6)	-5.2 (-13.4–4.1)
11	Depressive disorders	2.7 (1.9–3.7)	0.0593 (0.0387–0.0857)	950.6 (610.5–1373.5)	131.4 (86.9–179.2)	98.4 (60.0–140.9)
12	Self-harm	2.5 (2.1–2.9)	0.0548 (0.0452–0.0646)	819.6 (679.1–962.0)	66.5 (36.9–99.5)	35.2 (12.0–61.3)
13	Anxiety disorders	2.4 (1.7–3.4)	0.0532 (0.0341–0.0773)	812.8 (512.7–1192.0)	62.2 (21.3–103.6)	31.9 (-1.6–64.9)
14	Chronic kidney disease	2.4 (2.1–2.7)	0.0531 (0.0460–0.0608)	531.5 (464.3–607.2)	198.0 (161.5–241.8)	61.2 (42.2–84.8)
15	Headache disorders	2.0 (0.5–4.1)	0.0456 (0.0101–0.0960)	699.3 (138.8–1499.1)	21.0 (14.2–34.2)	-2.0 (-6.5–3.2)
16	Falls	2.0 (1.8–2.4)	0.0453 (0.0363–0.0571)	459.8 (367.1–580.3)	104.8 (89.5–123.0)	12.1 (4.7–20.9)
17	Age-related and other hearing loss	1.8 (1.4–2.4)	0.0409 (0.0288–0.0564)	415.9 (290.0–573.8)	69.4 (62.4–78.0)	-5.7 (-9.0—1.8)
18	Road injuries	1.7 (1.6–1.9)	0.0386 (0.0336–0.0445)	581.2 (506.7–667.5)	-32.0 (-39.5—24.5)	-48.2 (-54.2—42.4)
19	Interpersonal violence	1.7 (1.5–2.0)	0.0382 (0.0323–0.0442)	665.1 (562.0–766.8)	-12.7 (-27.1–0.8)	-22.8 (-35.3—10.5)
20	Hypertensive heart disease	1.5 (1.3–1.8)	0.0343 (0.0285–0.0406)	354.7 (293.6–419.7)	145.1 (103.5–188.7)	37.4 (13.8–62.3)
21	Osteoarthritis	1.5 (0.8–2.9)	0.0334 (0.0163–0.0671)	326.7 (158.3–654.2)	98.0 (92.5–104.5)	5.7 (2.7–9.1)
22	Colon and rectum cancer	1.4 (1.2–1.7)	0.0317 (0.0263–0.0375)	319.8 (265.9–380.4)	4.8 (-13.5–25.4)	-41.8 (-51.9—30.2)
23	Neonatal disorders	1.4 (1.2–1.6)	0.0309 (0.0266–0.0355)	794.9 (692.9–898.6)	-38.5 (-45.5—31.1)	-35.1 (-42.1—27.8)
24	Cirrhosis and other chronic liver diseases	1.3 (1.1–1.5)	0.0279 (0.0234–0.0330)	312.4 (262.5–369.7)	37.0 (14.4–63.0)	-15.9 (-29.4–0.3)
25	Asthma	1.2 (0.9–1.6)	0.0273 (0.0185–0.0393)	452.3 (301.7–657.9)	34.1 (25.0–44.7)	4.0 (-3.2–12.1)

Table S4: Cause-specific DALYs by US state in 2021 and percentage change between 1990 and 2021, Massachusetts

Rank	Cause Name	2021 Percentage of all cause DALYs	2021 DALYs (millions)	2021 Age Standardised Rate (per 100 000)	Percentage change DALY count 1990 to 2021	Percentage change age-standardised DALY rate 1990 to 2021
	All causes	100.0 (100.0–100.0)	2.35 (2.00–2.70)	24067.4 (20231.8–27961.4)	20.6 (11.1–29.8)	-10.4 (-17.0—4.2)
1	Drug use disorders	7.2 (6.2–8.1)	0.169 (0.138–0.198)	2338.6 (1901.2–2741.3)	759.7 (625.2–929.4)	716.1 (588.1–875.5)
2	COVID-19	5.9 (5.1–7.0)	0.138 (0.134–0.146)	1313.5 (1261.6–1420.9)	--	--
3	Ischaemic heart disease	5.8 (4.8–6.7)	0.135 (0.113–0.157)	1022.4 (854.2–1198.0)	-46.6 (-54.6—37.8)	-66.5 (-71.8—60.6)
4	Diabetes mellitus	4.1 (3.5–4.7)	0.0953 (0.0727–0.121)	798.0 (608.7–1009.4)	108.9 (83.9–131.3)	35.9 (19.7–49.9)
5	Low back pain	4.0 (3.2–4.8)	0.0951 (0.0689–0.123)	1021.7 (744.5–1331.8)	8.8 (1.2–17.0)	-18.0 (-23.9—12.2)
6	Other musculoskeletal disorders	3.6 (2.8–4.6)	0.0852 (0.0608–0.113)	967.9 (692.4–1291.9)	75.1 (58.2–95.8)	37.6 (24.5–54.1)
7	Chronic obstructive pulmonary disease	3.6 (3.1–4.1)	0.0840 (0.0734–0.0943)	623.6 (546.7–700.5)	54.3 (38.4–72.4)	-3.5 (-13.4–7.9)
8	Alzheimer's disease and other dementias	3.2 (1.5–6.5)	0.0750 (0.0357–0.155)	511.4 (245.7–1051.5)	50.7 (38.0–65.5)	-3.4 (-11.7–5.8)
9	Tracheal, bronchus, and lung cancer	3.0 (2.5–3.5)	0.0710 (0.0583–0.0831)	541.8 (446.6–635.7)	-21.4 (-36.0—6.8)	-54.4 (-63.0—45.9)
10	Depressive disorders	2.9 (2.0–4.1)	0.0693 (0.0440–0.101)	953.7 (605.2–1395.5)	54.6 (27.5–87.5)	43.0 (17.6–73.0)
11	Stroke	2.7 (2.3–3.0)	0.0635 (0.0536–0.0728)	492.3 (418.9–564.3)	-5.5 (-16.7–6.0)	-38.4 (-45.6—30.7)
12	Anxiety disorders	2.5 (1.7–3.5)	0.0597 (0.0373–0.0868)	787.5 (495.0–1148.3)	45.5 (11.6–85.8)	28.6 (-1.2–63.5)
13	Falls	2.5 (2.1–2.9)	0.0579 (0.0457–0.0748)	484.5 (378.7–623.6)	71.1 (58.9–84.4)	8.1 (0.9–15.8)
14	Headache disorders	2.2 (0.5–4.5)	0.0520 (0.0115–0.109)	690.7 (137.7–1467.7)	10.6 (4.8–23.9)	-2.9 (-8.1–3.5)
15	Chronic kidney disease	2.2 (1.9–2.5)	0.0518 (0.0439–0.0601)	412.9 (353.6–479.7)	168.3 (134.3–205.9)	71.7 (50.9–95.6)
16	Age-related and other hearing loss	2.1 (1.6–2.7)	0.0489 (0.0343–0.0679)	413.3 (287.8–575.8)	40.9 (36.0–46.9)	-5.3 (-8.2—2.0)
17	Osteoarthritis	1.6 (0.9–3.1)	0.0383 (0.0186–0.0774)	311.8 (150.0–629.6)	68.2 (61.3–75.3)	5.8 (1.3–10.1)
18	Cirrhosis and other chronic liver diseases	1.3 (1.1–1.5)	0.0304 (0.0250–0.0365)	284.1 (234.7–341.0)	10.2 (-9.7–31.5)	-26.6 (-39.9—12.7)
19	Alcohol use disorders	1.3 (1.1–1.5)	0.0303 (0.0243–0.0373)	367.5 (292.6–460.8)	25.1 (10.2–45.6)	4.8 (-6.2–19.5)
20	Colon and rectum cancer	1.3 (1.1–1.5)	0.0302 (0.0246–0.0356)	249.8 (204.2–295.1)	-29.5 (-42.7—14.9)	-53.0 (-62.2—42.8)
21	Asthma	1.3 (0.9–1.7)	0.0295 (0.0198–0.0425)	435.2 (288.8–640.7)	15.4 (7.7–23.7)	0.9 (-6.3–8.4)
22	Self-harm	1.2 (1.0–1.5)	0.0290 (0.0239–0.0348)	370.6 (304.6–444.3)	-19.8 (-34.4—2.9)	-29.7 (-42.4—14.8)
23	Road injuries	1.2 (1.1–1.3)	0.0285 (0.0238–0.0335)	350.3 (293.4–412.9)	-48.5 (-54.7—42.2)	-59.1 (-64.0—53.7)
24	Oral disorders	1.2 (0.8–1.7)	0.0279 (0.0165–0.0420)	270.1 (157.8–410.7)	31.4 (20.6–42.6)	-6.5 (-14.4–2.6)
25	Breast cancer	1.1 (0.8–1.3)	0.0246 (0.0190–0.0302)	210.1 (161.8–260.0)	-37.5 (-51.6—23.2)	-60.4 (-69.6—51.0)

Table S4: Cause-specific DALYs by US state in 2021 and percentage change between 1990 and 2021, Michigan						
**Rank**	**Cause Name**	**2021 Percentage of all cause DALYs**	**2021 DALYs (millions)**	**2021 Age Standardised Rate (per 100 000)**	**Percentage change DALY count 1990 to 2021**	**Percentage change age-standardised DALY rate 1990 to 2021**
	All causes	100.0 (100.0–100.0)	4.15 (3.64–4.74)	29868.8 (25921.1–34256.4)	34.4 (25.0–44.0)	1.3 (-5.4–8.0)
1	COVID-19	8.7 (7.7–9.9)	0.362 (0.350–0.393)	2426.1 (2323.9–2680.2)	--	--
2	Ischaemic heart disease	8.0 (6.8–9.0)	0.331 (0.282–0.383)	1762.9 (1513.9–2045.7)	-23.5 (-33.6–-12.5)	-53.4 (-59.7–-46.1)
3	Drug use disorders	5.4 (4.7–6.2)	0.226 (0.183–0.264)	2333.2 (1888.5–2730.7)	531.3 (449.9–634.0)	567.3 (479.9–671.9)
4	Diabetes mellitus	4.2 (3.7–4.8)	0.175 (0.139–0.222)	1010.9 (802.8–1269.5)	134.6 (108.1–161.2)	50.2 (33.7–67.1)
5	Chronic obstructive pulmonary disease	4.0 (3.5–4.4)	0.165 (0.146–0.182)	839.6 (744.5–927.7)	100.0 (79.5–120.7)	18.4 (6.4–30.9)
6	Low back pain	3.7 (2.9–4.5)	0.154 (0.112–0.201)	1184.4 (857.3–1550.3)	14.5 (6.4–23.4)	-8.2 (-14.1–-1.3)
7	Tracheal, bronchus, and lung cancer	3.2 (2.8–3.8)	0.135 (0.113–0.157)	706.9 (595.2–828.0)	-1.7 (-17.5–17.0)	-43.0 (-52.2–-32.0)
8	Other musculoskeletal disorders	3.1 (2.4–4.0)	0.131 (0.0941–0.173)	1050.1 (760.3–1387.4)	69.5 (54.1–86.9)	41.6 (28.6–56.3)
9	Stroke	3.1 (2.7–3.4)	0.127 (0.110–0.144)	693.8 (599.1–788.2)	16.1 (3.8–29.0)	-28.2 (-35.8–-20.2)
10	Alzheimer's disease and other dementias	2.6 (1.2–5.3)	0.107 (0.0486–0.228)	497.4 (228.0–1063.2)	75.2 (60.1–91.6)	-3.6 (-11.8–5.0)
11	Depressive disorders	2.5 (1.8–3.4)	0.105 (0.0714–0.146)	1040.9 (705.3–1448.5)	57.4 (27.7–90.3)	58.1 (28.6–89.9)
12	Chronic kidney disease	2.3 (2.1–2.5)	0.0953 (0.0819–0.109)	537.4 (463.0–610.9)	220.2 (181.8–261.5)	98.6 (76.0–122.8)
13	Anxiety disorders	2.0 (1.4–2.8)	0.0842 (0.0550–0.122)	803.1 (525.5–1160.8)	36.0 (3.6–74.2)	31.2 (0.0–68.1)
14	Falls	1.8 (1.6–2.1)	0.0769 (0.0614–0.0976)	441.9 (354.4–562.1)	83.5 (68.7–99.1)	13.6 (5.6–22.1)
15	Self-harm	1.8 (1.5–2.0)	0.0728 (0.0623–0.0855)	709.8 (606.2–837.4)	27.4 (7.9–50.1)	25.8 (7.0–48.5)
16	Age-related and other hearing loss	1.7 (1.3–2.2)	0.0721 (0.0504–0.0993)	416.2 (291.7–575.7)	45.2 (39.2–51.4)	-6.0 (-9.5–-2.6)
17	Headache disorders	1.6 (0.4–3.2)	0.0673 (0.0160–0.139)	646.6 (137.1–1362.2)	0.8 (-4.9–14.3)	-2.9 (-7.2–2.2)
18	Road injuries	1.6 (1.5–1.7)	0.0671 (0.0583–0.0777)	622.5 (545.8–715.3)	-42.3 (-48.3–-35.2)	-47.8 (-53.3–-41.2)
19	Osteoarthritis	1.5 (0.8–2.9)	0.0631 (0.0308–0.128)	351.4 (169.3–712.7)	66.9 (61.7–72.8)	3.0 (-0.1–6.4)
20	Cirrhosis and other chronic liver diseases	1.4 (1.2–1.6)	0.0577 (0.0489–0.0669)	389.4 (330.3–452.6)	30.6 (10.7–53.0)	-6.8 (-21.6–9.1)
21	Colon and rectum cancer	1.3 (1.1–1.5)	0.0530 (0.0445–0.0625)	305.8 (257.2–361.1)	-1.0 (-18.4–19.2)	-35.3 (-46.5–-22.4)
22	Hypertensive heart disease	1.3 (1.1–1.4)	0.0524 (0.0446–0.0612)	315.1 (267.6–368.8)	206.6 (161.9–258.2)	105.0 (73.6–138.7)
23	Neonatal disorders	1.2 (1.1–1.4)	0.0498 (0.0432–0.0570)	789.5 (698.6–889.1)	-48.8 (-54.0–-42.1)	-35.7 (-42.4–-27.5)
24	Asthma	1.1 (0.8–1.5)	0.0447 (0.0306–0.0634)	461.2 (312.7–669.8)	8.1 (0.0–15.4)	3.5 (-3.8–10.8)
25	Breast cancer	1.1 (0.9–1.3)	0.0443 (0.0358–0.0540)	271.0 (216.0–332.0)	-15.8 (-32.4–2.9)	-44.5 (-55.9–-32.1)

Table S4: Cause-specific DALYs by US state in 2021 and percentage change between 1990 and 2021, Minnesota

Rank	Cause Name	2021 Percentage of all cause DALYs	2021 DALYs (millions)	2021 Age Standardised Rate (per 100 000)	Percentage change DALY count 1990 to 2021	Percentage change age-standardised DALY rate 1990 to 2021
	All causes	100.0 (100.0–100.0)	1.88 (1.61–2.16)	24376.5 (20772.2–27993.4)	46.9 (36.2–58.1)	-3.5 (-10.0–3.3)
1	COVID-19	6.7 (5.8–7.7)	0.124 (0.119–0.135)	1534.9 (1453.5–1698.7)	--	--
2	Ischaemic heart disease	5.5 (4.5–6.3)	0.103 (0.0846–0.119)	1012.4 (833.2–1175.6)	-34.4 (-44.6–-23.9)	-62.9 (-68.9–-56.5)
3	Low back pain	4.1 (3.3–5.0)	0.0775 (0.0563–0.101)	1082.4 (788.9–1415.8)	31.5 (21.9–41.4)	-10.1 (-16.4–-3.4)
4	Diabetes mellitus	4.1 (3.6–4.7)	0.0768 (0.0605–0.0968)	825.6 (648.6–1037.5)	177.4 (147.3–205.5)	58.2 (41.3–73.6)
5	Drug use disorders	3.5 (3.0–4.1)	0.0665 (0.0537–0.0781)	1205.4 (972.6–1419.8)	515.6 (414.5–634.3)	428.9 (341.5–528.3)
6	Chronic obstructive pulmonary disease	3.5 (3.0–4.0)	0.0660 (0.0565–0.0742)	621.5 (532.0–698.1)	86.9 (65.4–109.4)	1.7 (-10.0–13.8)
7	Other musculoskeletal disorders	3.3 (2.6–4.2)	0.0631 (0.0452–0.0844)	947.4 (680.6–1261.9)	101.5 (79.2–129.2)	44.2 (28.9–63.6)
8	Stroke	3.2 (2.8–3.6)	0.0598 (0.0505–0.0676)	595.2 (508.3–674.0)	16.1 (2.4–29.2)	-30.8 (-39.1–-22.9)
9	Falls	3.1 (2.8–3.6)	0.0590 (0.0479–0.0745)	617.5 (498.6–780.5)	117.1 (99.2–137.4)	21.6 (12.1–32.6)
10	Alzheimer's disease and other dementias	3.1 (1.5–6.6)	0.0586 (0.0279–0.126)	514.3 (246.9–1105.2)	67.9 (52.7–85.3)	-2.0 (-10.9–7.6)
11	Tracheal, bronchus, and lung cancer	3.0 (2.5–3.6)	0.0570 (0.0472–0.0677)	557.8 (461.7–664.7)	14.3 (-3.9–36.8)	-42.6 (-51.7–-30.9)
12	Depressive disorders	2.9 (2.1–3.9)	0.0546 (0.0365–0.0782)	945.4 (627.5–1380.9)	73.8 (44.5–112.5)	41.4 (18.4–71.2)
13	Anxiety disorders	2.5 (1.6–3.4)	0.0463 (0.0290–0.0669)	772.5 (482.0–1114.2)	60.5 (23.2–103.2)	26.5 (-4.0–59.0)
14	Chronic kidney disease	2.3 (2.0–2.6)	0.0428 (0.0366–0.0485)	438.3 (377.4–496.9)	275.2 (228.9–327.1)	115.1 (88.8–145.1)
15	Headache disorders	2.2 (0.5–4.4)	0.0409 (0.00900–0.0860)	688.0 (137.2–1460.5)	23.5 (16.7–38.7)	-2.9 (-7.7–2.9)
16	Age-related and other hearing loss	2.1 (1.5–2.7)	0.0389 (0.0272–0.0536)	419.2 (290.8–576.5)	60.8 (54.0–68.6)	-5.3 (-8.6–-1.1)
17	Self-harm	2.0 (1.7–2.4)	0.0377 (0.0309–0.0446)	649.0 (532.1–764.9)	40.2 (14.2–66.7)	13.1 (-7.3–34.3)
18	Road injuries	1.7 (1.6–1.9)	0.0326 (0.0278–0.0377)	521.0 (447.1–596.9)	-32.3 (-39.9–-24.9)	-50.9 (-56.7–-45.0)
19	Osteoarthritis	1.5 (0.8–2.8)	0.0281 (0.0137–0.0570)	291.8 (140.7–590.4)	95.0 (89.5–101.3)	6.5 (3.7–9.6)
20	Cirrhosis and other chronic liver diseases	1.5 (1.2–1.7)	0.0276 (0.0231–0.0327)	336.7 (282.0–398.6)	142.5 (100.4–187.3)	45.4 (20.2–71.9)
21	Alcohol use disorders	1.3 (1.2–1.5)	0.0254 (0.0206–0.0314)	392.1 (312.8–489.0)	36.1 (20.0–57.4)	1.0 (-9.1–15.4)
22	Colon and rectum cancer	1.3 (1.1–1.6)	0.0252 (0.0208–0.0303)	264.9 (219.3–318.1)	6.4 (-12.2–29.6)	-39.0 (-50.0–-25.7)
23	Asthma	1.2 (0.9–1.6)	0.0225 (0.0153–0.0324)	386.5 (261.2–562.9)	23.9 (14.8–33.4)	-5.1 (-11.4–1.8)
24	Breast cancer	1.2 (0.9–1.4)	0.0221 (0.0176–0.0271)	244.0 (192.7–299.7)	-5.1 (-22.8–16.7)	-46.7 (-57.0–-33.8)
25	Neonatal disorders	1.2 (1.0–1.3)	0.0218 (0.0183–0.0256)	562.3 (476.9–655.4)	-23.8 (-34.4–-12.9)	-26.7 (-37.4–-15.3)

Table S4: Cause-specific DALYs by US state in 2021 and percentage change between 1990 and 2021, Mississippi						
**Rank**	**Cause Name**	**2021 Percentage of all cause DALYs**	**2021 DALYs (millions)**	**2021 Age Standardised Rate (per 100 000)**	**Percentage change DALY count 1990 to 2021**	**Percentage change age-standardised DALY rate 1990 to 2021**
	All causes	100.0 (100.0–100.0)	1.39 (1.22–1.57)	36508.5 (31955.3–41080.5)	48.5 (37.3–62.1)	12.2 (4.0–21.4)
1	COVID-19	10.7 (8.7–13.3)	0.148 (0.131–0.182)	3664.7 (3215.1–4511.0)	--	--
2	Ischaemic heart disease	8.0 (7.1–9.0)	0.112 (0.0961–0.131)	2402.8 (2063.4–2800.6)	-17.7 (-28.1—4.3)	-43.4 (-50.9—34.1)
3	Chronic obstructive pulmonary disease	4.2 (3.8–4.6)	0.0583 (0.0513–0.0658)	1145.0 (1008.9–1289.3)	129.0 (104.1–157.5)	48.6 (32.1–67.1)
4	Drug use disorders	3.8 (3.2–4.4)	0.0530 (0.0418–0.0639)	1845.5 (1456.2–2224.7)	537.9 (437.3–673.7)	503.9 (410.8–626.2)
5	Stroke	3.8 (3.4–4.1)	0.0526 (0.0453–0.0596)	1126.2 (969.7–1277.7)	28.4 (13.2–46.0)	-11.7 (-22.3–0.2)
6	Diabetes mellitus	3.8 (3.3–4.3)	0.0524 (0.0421–0.0647)	1163.9 (934.0–1434.1)	150.1 (125.8–173.1)	69.5 (53.2–85.1)
7	Tracheal, bronchus, and lung cancer	3.5 (3.0–4.1)	0.0493 (0.0417–0.0587)	997.4 (843.2–1186.0)	10.8 (-7.2–32.2)	-31.5 (-42.6—18.3)
8	Road injuries	3.0 (2.8–3.2)	0.0422 (0.0368–0.0483)	1394.0 (1226.0–1591.9)	-23.1 (-31.3—13.4)	-31.9 (-38.9—23.1)
9	Chronic kidney disease	3.0 (2.7–3.3)	0.0419 (0.0367–0.0478)	927.2 (814.6–1054.8)	247.9 (209.4–298.9)	137.0 (111.0–171.3)
10	Low back pain	2.8 (2.2–3.5)	0.0397 (0.0287–0.0515)	1092.4 (790.5–1414.5)	15.3 (7.8–23.3)	-10.1 (-15.7—4.1)
11	Depressive disorders	2.3 (1.6–3.1)	0.0323 (0.0212–0.0454)	1077.6 (707.7–1523.9)	72.5 (38.1–109.1)	57.9 (26.0–91.7)
12	Hypertensive heart disease	1.9 (1.7–2.2)	0.0271 (0.0229–0.0319)	609.3 (514.2–722.3)	220.0 (169.5–277.7)	123.8 (87.4–166.1)
13	Alzheimer's disease and other dementias	1.9 (0.9–4.0)	0.0263 (0.0121–0.0580)	498.2 (229.1–1096.5)	44.6 (32.6–59.2)	-2.8 (-10.5–6.8)
14	Anxiety disorders	1.8 (1.2–2.5)	0.0253 (0.0167–0.0373)	828.5 (543.8–1215.2)	50.3 (17.2–89.9)	34.4 (5.7–72.4)
15	Other musculoskeletal disorders	1.8 (1.4–2.3)	0.0251 (0.0183–0.0329)	717.8 (525.0–950.8)	66.2 (49.2–87.0)	34.1 (20.2–50.8)
16	Interpersonal violence	1.7 (1.5–1.9)	0.0232 (0.0201–0.0266)	862.8 (747.4–983.6)	-0.8 (-14.6–13.6)	-2.0 (-15.2–12.1)
17	Colon and rectum cancer	1.6 (1.3–1.8)	0.0216 (0.0179–0.0261)	473.8 (392.0–570.1)	45.8 (19.9–77.6)	0.1 (-17.4–21.2)
18	Falls	1.5 (1.3–1.8)	0.0215 (0.0173–0.0267)	477.5 (383.9–590.4)	79.0 (65.4–94.8)	19.3 (10.6–29.9)
19	Self-harm	1.5 (1.3–1.7)	0.0214 (0.0182–0.0252)	711.9 (606.5–837.2)	48.4 (25.3–74.2)	35.2 (14.2–58.1)
20	Cirrhosis and other chronic liver diseases	1.5 (1.3–1.7)	0.0213 (0.0180–0.0250)	527.1 (445.8–616.4)	105.1 (73.2–141.2)	43.0 (20.3–67.6)
21	Headache disorders	1.5 (0.3–3.1)	0.0211 (0.00460–0.0442)	688.8 (137.2–1460.4)	8.5 (2.9–22.0)	-3.8 (-8.6–2.6)
22	Age-related and other hearing loss	1.4 (1.0–1.8)	0.0194 (0.0135–0.0271)	421.5 (292.6–586.3)	37.0 (32.0–42.9)	-5.4 (-8.4—1.8)
23	Neonatal disorders	1.3 (1.2–1.6)	0.0187 (0.0159–0.0216)	991.7 (844.5–1152.1)	-36.9 (-45.3—26.5)	-28.3 (-38.6—15.5)
24	Breast cancer	1.1 (0.9–1.3)	0.0154 (0.0124–0.0188)	353.9 (283.3–434.4)	10.4 (-10.8–37.2)	-26.1 (-40.9—7.7)
25	Lower respiratory infections	1.0 (0.9–1.2)	0.0145 (0.0121–0.0172)	335.8 (283.3–395.2)	-10.3 (-24.4–6.6)	-37.0 (-46.1—25.6)

Table S4: Cause-specific DALYs by US state in 2021 and percentage change between 1990 and 2021, Missouri

Rank	Cause Name	2021 Percentage of all cause DALYs	2021 DALYs (millions)	2021 Age Standardised Rate (per 100 000)	Percentage change DALY count 1990 to 2021	Percentage change age-standardised DALY rate 1990 to 2021
	All causes	100.0 (100.0–100.0)	2.60 (2.27–2.92)	31380.6 (27342.5–35383.1)	44.7 (33.8–56.2)	7.6 (0.0–15.5)
1	COVID-19	8.1 (7.1–9.3)	0.210 (0.200–0.236)	2367.9 (2232.3–2678.6)	--	--
2	Ischaemic heart disease	7.9 (6.8–9.1)	0.206 (0.177–0.238)	1910.3 (1630.0–2215.0)	-20.0 (-31.7—7.5)	-46.2 (-54.3—37.4)
3	Drug use disorders	5.4 (4.6–6.1)	0.139 (0.115–0.162)	2337.9 (1922.7–2707.5)	805.6 (668.1–977.8)	727.7 (600.0–884.9)
4	Chronic obstructive pulmonary disease	4.3 (3.8–4.8)	0.112 (0.0996–0.126)	982.7 (872.9–1103.3)	95.9 (74.1–118.8)	25.5 (11.3–39.8)
5	Diabetes mellitus	3.9 (3.4–4.4)	0.100 (0.0797–0.124)	1002.5 (799.2–1233.4)	145.8 (116.5–169.8)	64.9 (46.4–80.8)
6	Low back pain	3.8 (3.1–4.6)	0.0980 (0.0715–0.127)	1273.6 (919.7–1659.1)	24.2 (16.3–33.4)	-4.9 (-11.0–2.1)
7	Tracheal, bronchus, and lung cancer	3.5 (3.0–4.1)	0.0914 (0.0773–0.107)	829.8 (698.4–970.1)	-1.0 (-17.3–16.5)	-40.8 (-50.7—30.5)
8	Stroke	3.2 (2.9–3.5)	0.0828 (0.0724–0.0939)	767.4 (668.1–865.7)	17.7 (3.6–31.4)	-20.8 (-30.2—12.0)
9	Other musculoskeletal disorders	2.9 (2.3–3.8)	0.0762 (0.0546–0.101)	1043.0 (740.6–1386.4)	91.7 (70.8–114.1)	51.2 (35.3–68.6)
10	Alzheimer's disease and other dementias	2.5 (1.2–5.0)	0.0638 (0.0297–0.135)	497.0 (235.9–1051.0)	48.2 (35.0–61.9)	-4.4 (-12.6–4.4)
11	Chronic kidney disease	2.4 (2.1–2.6)	0.0617 (0.0536–0.0705)	588.9 (515.4–670.5)	240.0 (199.6–287.0)	122.7 (96.9–154.0)
12	Depressive disorders	2.3 (1.6–3.2)	0.0590 (0.0387–0.0835)	954.5 (619.9–1350.0)	100.6 (64.2–147.4)	78.6 (45.9–121.3)
13	Road injuries	2.2 (2.0–2.3)	0.0568 (0.0493–0.0645)	882.8 (771.2–991.9)	-25.5 (-33.3—16.0)	-39.1 (-45.4—31.0)
14	Falls	2.0 (1.7–2.4)	0.0524 (0.0421–0.0660)	514.8 (412.5–650.6)	73.4 (60.5–89.8)	10.7 (2.9–21.2)
15	Self-harm	2.0 (1.7–2.3)	0.0519 (0.0433–0.0612)	828.2 (691.6–974.0)	53.0 (27.3–81.2)	34.7 (11.9–59.4)
16	Anxiety disorders	2.0 (1.4–2.8)	0.0513 (0.0341–0.0761)	800.7 (525.4–1188.8)	50.9 (16.3–87.0)	30.3 (1.0–61.5)
17	Headache disorders	1.7 (0.4–3.3)	0.0436 (0.00961–0.0916)	683.4 (134.9–1444.0)	12.5 (6.4–23.3)	-3.9 (-9.1–2.0)
18	Age-related and other hearing loss	1.6 (1.2–2.1)	0.0425 (0.0298–0.0583)	417.4 (293.3–575.6)	38.3 (33.5–43.9)	-5.8 (-8.7—2.3)
19	Colon and rectum cancer	1.4 (1.2–1.6)	0.0357 (0.0304–0.0421)	351.4 (299.4–413.4)	6.0 (-11.8–27.4)	-27.5 (-39.6—12.7)
20	Cirrhosis and other chronic liver diseases	1.3 (1.2–1.6)	0.0350 (0.0294–0.0411)	400.7 (337.5–471.1)	89.2 (57.6–124.7)	31.2 (10.1–55.5)
21	Interpersonal violence	1.3 (1.2–1.5)	0.0341 (0.0297–0.0388)	615.5 (537.6–701.2)	3.3 (-10.4–18.2)	-3.4 (-16.1–10.4)
22	Osteoarthritis	1.3 (0.7–2.4)	0.0330 (0.0161–0.0659)	315.8 (152.8–628.7)	62.7 (58.4–67.9)	5.4 (2.6–8.5)
23	Breast cancer	1.1 (0.9–1.3)	0.0282 (0.0226–0.0342)	291.7 (234.3–354.3)	-11.7 (-29.0–6.3)	-41.8 (-53.4—29.4)
24	Neonatal disorders	1.1 (0.9–1.2)	0.0280 (0.0240–0.0323)	723.0 (628.9–812.9)	-31.4 (-39.3—22.4)	-26.5 (-35.4—17.0)
25	Hypertensive heart disease	1.1 (0.9–1.2)	0.0273 (0.0231–0.0318)	274.5 (232.4–320.6)	167.1 (124.6–212.1)	86.7 (56.0–118.6)

Table S4: Cause-specific DALYs by US state in 2021 and percentage change between 1990 and 2021, Montana						
Rank	Cause Name	2021 Percentage of all cause DALYs	2021 DALYs (millions)	2021 Age Standardised Rate (per 100 000)	Percentage change DALY count 1990 to 2021	Percentage change age-standardised DALY rate 1990 to 2021
	All causes	100.0 (100.0–100.0)	0.439 (0.386–0.498)	28895.0 (25241.1–32981.7)	71.6 (59.9–85.1)	4.7 (-1.7–12.5)
1	COVID-19	9.8 (8.6–11.0)	0.0429 (0.0417–0.0456)	2601.2 (2503.4–2812.9)	--	--
2	Ischaemic heart disease	6.9 (5.8–8.0)	0.0301 (0.0252–0.0351)	1427.8 (1196.4–1680.4)	6.9 (-8.8–25.0)	-45.2 (-53.5–-35.6)
3	Chronic obstructive pulmonary disease	4.6 (4.1–5.2)	0.0202 (0.0179–0.0227)	899.9 (799.1–1009.3)	112.3 (89.7–137.9)	5.3 (-5.8–18.0)
4	Low back pain	3.9 (3.1–4.8)	0.0172 (0.0125–0.0225)	1214.7 (883.7–1593.4)	45.0 (34.9–55.7)	-7.5 (-13.3–-0.9)
5	Diabetes mellitus	3.5 (3.1–4.0)	0.0156 (0.0124–0.0194)	807.4 (646.8–1004.6)	168.0 (139.9–198.6)	41.7 (27.2–56.9)
6	Drug use disorders	3.5 (3.0–4.1)	0.0156 (0.0122–0.0183)	1551.2 (1212.8–1821.6)	527.6 (437.4–645.4)	405.7 (331.4–503.1)
7	Other musculoskeletal disorders	3.3 (2.5–4.2)	0.0144 (0.0103–0.0192)	1100.9 (792.6–1469.4)	124.8 (102.0–150.1)	51.7 (36.5–69.2)
8	Stroke	2.8 (2.5–3.1)	0.0122 (0.0106–0.0138)	598.3 (526.2–674.8)	25.0 (12.4–39.9)	-33.3 (-39.8–-24.9)
9	Alzheimer's disease and other dementias	2.8 (1.3–5.8)	0.0122 (0.00574–0.0257)	497.6 (235.7–1048.5)	101.5 (86.2–118.7)	-3.2 (-10.4–5.1)
10	Self-harm	2.7 (2.3–3.2)	0.0120 (0.0101–0.0143)	1133.2 (956.2–1343.1)	64.1 (36.6–96.4)	27.2 (6.4–51.8)
11	Tracheal, bronchus, and lung cancer	2.6 (2.2–3.1)	0.0116 (0.00992–0.0136)	554.6 (471.0–650.6)	8.5 (-8.6–29.8)	-46.7 (-55.3–-36.0)
12	Depressive disorders	2.6 (1.8–3.5)	0.0114 (0.00732–0.0159)	1054.1 (684.7–1481.9)	89.1 (50.4–132.8)	47.5 (17.3–80.0)
13	Falls	2.6 (2.2–3.0)	0.0113 (0.00905–0.0143)	593.8 (473.8–753.1)	143.4 (125.4–163.3)	24.8 (16.0–34.7)
14	Road injuries	2.6 (2.4–2.7)	0.0113 (0.00989–0.0129)	1011.8 (899.4–1149.6)	-15.3 (-23.6–-4.5)	-39.3 (-45.4–-31.4)
15	Anxiety disorders	2.0 (1.3–2.8)	0.00867 (0.00557–0.0126)	769.6 (487.9–1132.6)	67.2 (31.5–113.2)	26.6 (-1.5–61.6)
16	Chronic kidney disease	2.0 (1.7–2.2)	0.00860 (0.00744–0.00979)	433.2 (377.1–493.9)	292.8 (247.8–345.7)	104.3 (80.5–130.6)
17	Age-related and other hearing loss	1.9 (1.4–2.4)	0.00823 (0.00577–0.0113)	420.4 (295.8–576.5)	78.3 (71.4–85.7)	-5.6 (-8.9–-2.3)
18	Headache disorders	1.7 (0.4–3.4)	0.00757 (0.00175–0.0160)	679.5 (139.9–1438.2)	27.5 (20.2–42.4)	-3.6 (-8.4–1.9)
19	Cirrhosis and other chronic liver diseases	1.6 (1.4–1.9)	0.00720 (0.00615–0.00836)	472.8 (404.4–549.8)	128.7 (94.3–171.7)	40.8 (19.7–67.4)
20	Alcohol use disorders	1.5 (1.3–1.7)	0.00651 (0.00540–0.00785)	539.5 (445.6–661.0)	59.3 (40.2–86.7)	12.5 (0.0–29.8)
21	Osteoarthritis	1.4 (0.7–2.6)	0.00602 (0.00294–0.0123)	298.7 (144.1–604.7)	112.7 (105.7–120.1)	7.8 (4.5–11.6)
22	Colon and rectum cancer	1.3 (1.0–1.5)	0.00556 (0.00465–0.00664)	284.2 (239.6–338.9)	26.1 (3.2–53.4)	-32.2 (-44.3–-17.5)
23	Asthma	1.2 (0.9–1.5)	0.00509 (0.00351–0.00721)	460.4 (314.4–670.4)	31.0 (21.5–40.4)	-2.5 (-8.9–3.8)
24	Breast cancer	1.1 (0.9–1.2)	0.00466 (0.00394–0.00546)	258.8 (217.9–304.1)	12.5 (-4.5–33.3)	-38.8 (-48.6–-27.0)
25	Oral disorders	1.0 (0.7–1.4)	0.00458 (0.00278–0.00682)	278.8 (164.6–426.4)	64.1 (57.1–70.9)	-3.6 (-8.1–1.2)

Table S4: Cause-specific DALYs by US state in 2021 and percentage change between 1990 and 2021, Nebraska						
**Rank**	**Cause Name**	**2021 Percentage of all cause DALYs**	**2021 DALYs (millions)**	**2021 Age Standardised Rate (per 100 000)**	**Percentage change DALY count 1990 to 2021**	**Percentage change age-standardised DALY rate 1990 to 2021**
	All causes	100.0 (100.0–100.0)	0.680 (0.591–0.778)	26142.1 (22498.6–30098.0)	33.7 (23.4–43.7)	-1.8 (-8.9–5.3)
1	Ischaemic heart disease	6.5 (5.5–7.5)	0.0439 (0.0370–0.0510)	1300.8 (1089.7–1520.6)	-34.6 (-43.8—24.9)	-55.4 (-62.0—48.3)
2	COVID-19	6.0 (5.2–7.0)	0.0408 (0.0383–0.0463)	1514.6 (1401.6–1741.8)	--	--
3	Low back pain	4.7 (3.6–5.7)	0.0317 (0.0231–0.0413)	1355.6 (985.5–1776.1)	24.0 (16.9–31.4)	-5.8 (-11.1–0.1)
4	Chronic obstructive pulmonary disease	4.6 (4.0–5.2)	0.0312 (0.0275–0.0348)	897.3 (795.5–1001.6)	78.7 (59.3–99.6)	18.3 (5.3–32.0)
5	Diabetes mellitus	4.1 (3.6–4.6)	0.0278 (0.0222–0.0349)	911.9 (728.8–1135.0)	150.0 (125.9–172.4)	67.7 (52.1–81.9)
6	Other musculoskeletal disorders	3.2 (2.5–4.0)	0.0217 (0.0157–0.0287)	949.5 (687.9–1259.5)	83.0 (66.0–102.2)	39.1 (26.3–54.5)
7	Stroke	3.1 (2.8–3.5)	0.0212 (0.0184–0.0240)	638.0 (552.6–714.6)	2.4 (-8.8–12.9)	-27.8 (-35.8—20.2)
8	Tracheal, bronchus, and lung cancer	3.1 (2.6–3.6)	0.0209 (0.0174–0.0243)	633.7 (529.7–741.7)	-6.1 (-20.4–11.2)	-42.8 (-51.6—32.1)
9	Alzheimer's disease and other dementias	3.0 (1.4–6.3)	0.0201 (0.00924–0.0414)	509.6 (237.3–1051.0)	38.9 (28.0–51.0)	-2.1 (-9.3–6.3)
10	Drug use disorders	2.6 (2.2–3.0)	0.0176 (0.0139–0.0210)	927.2 (732.8–1110.5)	416.1 (346.6–520.2)	347.1 (285.3–435.9)
11	Depressive disorders	2.6 (1.9–3.5)	0.0175 (0.0115–0.0247)	884.3 (581.2–1261.1)	63.1 (32.3–97.9)	38.0 (11.4–67.9)
12	Chronic kidney disease	2.5 (2.2–2.9)	0.0171 (0.0148–0.0195)	527.7 (456.6–599.0)	228.4 (190.9–272.8)	122.2 (97.3–152.0)
13	Road injuries	2.4 (2.2–2.6)	0.0164 (0.0141–0.0186)	805.1 (700.8–907.8)	-21.9 (-31.3—13.8)	-38.0 (-45.5—31.1)
14	Falls	2.4 (2.1–2.8)	0.0162 (0.0131–0.0206)	522.9 (420.5–663.0)	83.5 (70.7–98.3)	20.3 (12.0–29.9)
15	Anxiety disorders	2.3 (1.6–3.2)	0.0155 (0.00970–0.0228)	762.8 (473.2–1123.8)	50.7 (15.0–91.3)	24.8 (-4.2–58.8)
16	Headache disorders	2.0 (0.4–4.1)	0.0138 (0.00300–0.0291)	685.5 (135.7–1455.8)	17.8 (12.1–27.9)	-3.2 (-8.1–1.9)
17	Self-harm	2.0 (1.7–2.2)	0.0133 (0.0109–0.0156)	663.7 (546.7–775.7)	36.9 (11.7–61.4)	12.8 (-7.4–32.6)
18	Age-related and other hearing loss	1.9 (1.4–2.5)	0.0129 (0.00901–0.0179)	419.8 (291.7–585.6)	35.9 (31.0–41.7)	-5.3 (-8.4—1.7)
19	Colon and rectum cancer	1.5 (1.3–1.8)	0.0102 (0.00852–0.0119)	324.8 (269.0–381.2)	4.7 (-12.5–25.3)	-28.6 (-40.5—14.2)
20	Cirrhosis and other chronic liver diseases	1.5 (1.3–1.7)	0.0100 (0.00844–0.0116)	381.5 (320.0–442.9)	107.1 (73.7–141.5)	41.7 (18.7–65.1)
21	Osteoarthritis	1.4 (0.8–2.7)	0.00971 (0.00469–0.0197)	309.3 (148.1–625.4)	63.5 (59.1–68.2)	7.2 (4.1–10.3)
22	Asthma	1.3 (1.0–1.6)	0.00869 (0.00600–0.0123)	423.6 (288.1–608.1)	15.2 (7.3–22.6)	-7.5 (-13.8—1.0)
23	Breast cancer	1.3 (1.1–1.5)	0.00864 (0.00732–0.0102)	292.7 (245.1–345.7)	-3.2 (-18.1–14.1)	-36.0 (-46.3—24.0)
24	Endocrine, metabolic, blood, and immune disorders	1.2 (1.0–1.3)	0.00796 (0.00692–0.00924)	307.6 (265.6–360.0)	162.3 (124.0–205.0)	79.0 (54.4–106.1)
25	Neonatal disorders	1.2 (1.0–1.4)	0.00794 (0.00667–0.00940)	573.7 (486.7–679.5)	-17.9 (-30.3—3.4)	-23.2 (-35.1—8.4)

Table S4: Cause-specific DALYs by US state in 2021 and percentage change between 1990 and 2021, Nevada						
Rank	Cause Name	2021 Percentage of all cause DALYs	2021 DALYs (millions)	2021 Age Standardised Rate (per 100 000)	Percentage change DALY count 1990 to 2021	Percentage change age-standardised DALY rate 1990 to 2021
	All causes	100.0 (100.0–100.0)	1.23 (1.08–1.39)	30252.6 (26310.2–34486.9)	204.4 (183.0–226.5)	-1.2 (-8.2–5.5)
1	COVID-19	11.4 (10.1–12.9)	0.140 (0.135–0.149)	3264.6 (3142.0–3516.5)	--	--
2	Ischaemic heart disease	7.1 (6.1–8.2)	0.0875 (0.0735–0.101)	1723.8 (1445.8–1997.4)	94.9 (65.1–127.5)	-48.9 (-56.9—40.4)
3	Drug use disorders	5.4 (4.6–6.2)	0.0667 (0.0543–0.0787)	2006.7 (1636.4–2353.6)	1175.6 (1001.3–1394.8)	427.8 (355.7–512.7)
4	Low back pain	3.9 (3.1–4.7)	0.0482 (0.0349–0.0628)	1220.9 (879.0–1593.6)	174.2 (155.5–194.4)	-4.4 (-10.7–2.8)
5	Chronic obstructive pulmonary disease	3.9 (3.4–4.3)	0.0477 (0.0425–0.0530)	916.8 (817.8–1017.1)	219.4 (186.5–251.1)	-14.7 (-23.3—6.5)
6	Diabetes mellitus	3.6 (3.1–4.1)	0.0442 (0.0348–0.0553)	924.5 (726.5–1150.4)	466.1 (406.1–519.3)	65.3 (47.9–80.9)
7	Depressive disorders	3.0 (2.1–4.0)	0.0367 (0.0242–0.0518)	1126.0 (742.5–1593.8)	264.9 (199.5–339.1)	48.8 (21.9–80.2)
8	Other musculoskeletal disorders	3.0 (2.3–3.7)	0.0367 (0.0263–0.0485)	958.1 (687.6–1268.5)	318.7 (272.4–371.7)	49.9 (34.0–68.3)
9	Stroke	2.8 (2.5–3.1)	0.0350 (0.0306–0.0394)	715.4 (622.9–806.3)	160.4 (131.6–189.9)	-30.8 (-38.5—23.3)
10	Tracheal, bronchus, and lung cancer	2.8 (2.3–3.2)	0.0339 (0.0285–0.0398)	659.6 (556.3–774.0)	65.6 (38.8–95.7)	-53.9 (-61.3—45.8)
11	Self-harm	2.4 (2.0–2.7)	0.0290 (0.0243–0.0337)	846.6 (711.9–984.1)	105.6 (70.9–140.9)	-18.7 (-31.8—5.1)
12	Anxiety disorders	2.2 (1.5–3.2)	0.0273 (0.0171–0.0402)	815.3 (513.2–1206.0)	237.1 (163.5–326.1)	34.6 (4.4–69.9)
13	Alzheimer's disease and other dementias	2.1 (1.0–4.5)	0.0264 (0.0124–0.0543)	491.8 (231.1–1014.5)	356.7 (315.0–397.9)	-3.5 (-11.1–4.8)
14	Chronic kidney disease	2.1 (1.8–2.3)	0.0253 (0.0220–0.0288)	527.2 (455.9–596.8)	594.9 (513.1–692.1)	89.6 (68.0–113.9)
15	Headache disorders	1.8 (0.4–3.9)	0.0229 (0.00500–0.0477)	681.8 (136.4–1437.2)	146.3 (133.7–169.6)	-2.4 (-7.4–3.4)
16	Road injuries	1.9 (1.7–2.0)	0.0228 (0.0193–0.0261)	660.4 (564.2–749.9)	10.7 (-2.3–21.9)	-59.0 (-63.6—54.6)
17	Cirrhosis and other chronic liver diseases	1.8 (1.5–2.0)	0.0216 (0.0180–0.0251)	490.5 (409.9–568.8)	201.9 (149.2–252.3)	-3.3 (-19.9–12.4)
18	Age-related and other hearing loss	1.6 (1.2–2.1)	0.0200 (0.0140–0.0276)	421.0 (295.9–584.3)	226.9 (214.8–239.7)	-6.2 (-9.3—3.0)
19	Falls	1.6 (1.4–1.9)	0.0198 (0.0155–0.0250)	421.4 (330.7–529.6)	290.5 (258.5–328.3)	10.7 (2.6–19.7)
20	Hypertensive heart disease	1.5 (1.3–1.8)	0.0190 (0.0157–0.0231)	396.4 (327.2–482.7)	261.3 (196.0–353.3)	3.4 (-15.3–29.8)
21	Alcohol use disorders	1.4 (1.3–1.6)	0.0176 (0.0140–0.0213)	476.1 (378.7–583.6)	140.0 (113.9–171.6)	-9.8 (-18.9–0.5)
22	Colon and rectum cancer	1.3 (1.1–1.5)	0.0158 (0.0131–0.0185)	324.2 (268.9–380.8)	130.5 (89.6–175.3)	-34.4 (-46.0—21.7)
23	Osteoarthritis	1.2 (0.6–2.4)	0.0151 (0.00719–0.0303)	307.0 (145.4–615.8)	273.2 (262.3–285.0)	5.9 (2.9–8.9)
24	Breast cancer	1.2 (1.0–1.4)	0.0143 (0.0120–0.0169)	306.3 (258.3–362.8)	126.6 (89.8–170.4)	-32.1 (-43.3—18.8)
25	Asthma	1.1 (0.8–1.4)	0.0132 (0.00905–0.0190)	435.0 (296.5–636.8)	138.8 (123.8–155.4)	-5.7 (-11.3–1.0)

Table S4: Cause-specific DALYs by US state in 2021 and percentage change between 1990 and 2021, New Hampshire						
Rank	Cause Name	2021 Percentage of all cause DALYs	2021 DALYs (millions)	2021 Age Standardised Rate (per 100 000)	Percentage change DALY count 1990 to 2021	Percentage change age-standardised DALY rate 1990 to 2021
	All causes	100.0 (100.0–100.0)	0.519 (0.447–0.593)	26333.3 (22369.9–30440.1)	57.4 (46.5–69.2)	-0.8 (-7.3–5.9)
1	Drug use disorders	6.8 (5.8–7.7)	0.0351 (0.0284–0.0407)	2701.0 (2184.4–3134.8)	693.1 (582.0–857.2)	668.1 (565.6–818.3)
2	Ischaemic heart disease	6.5 (5.4–7.5)	0.0336 (0.0281–0.0395)	1211.2 (1014.8–1425.2)	-20.4 (-31.8–-7.1)	-62.0 (-67.6–-55.3)
3	COVID-19	5.3 (4.6–6.3)	0.0274 (0.0263–0.0317)	1277.6 (1213.3–1489.5)	--	--
4	Diabetes mellitus	4.4 (3.8–5.0)	0.0229 (0.0177–0.0290)	898.4 (691.8–1130.5)	200.7 (165.3–230.4)	51.6 (34.2–66.5)
5	Low back pain	4.2 (3.3–5.1)	0.0218 (0.0158–0.0283)	1174.6 (857.5–1525.1)	33.9 (23.3–46.8)	-9.5 (-15.4–-2.0)
6	Chronic obstructive pulmonary disease	4.2 (3.6–4.7)	0.0215 (0.0189–0.0240)	753.0 (661.8–840.1)	116.3 (93.4–139.4)	1.6 (-9.1–12.3)
7	Other musculoskeletal disorders	3.5 (2.6–4.4)	0.0180 (0.0128–0.0241)	1025.5 (733.8–1368.9)	106.4 (85.9–133.1)	45.5 (31.2–63.3)
8	Tracheal, bronchus, and lung cancer	3.3 (2.7–3.9)	0.0171 (0.0143–0.0203)	613.2 (514.8–728.6)	14.3 (-4.0–35.5)	-48.2 (-56.6–-38.7)
9	Depressive disorders	2.9 (2.0–4.1)	0.0153 (0.0101–0.0227)	1095.5 (719.1–1640.2)	73.2 (41.6–109.8)	51.4 (24.8–83.9)
10	Alzheimer's disease and other dementias	2.9 (1.3–6.2)	0.0150 (0.00703–0.0326)	500.3 (234.5–1081.7)	105.5 (90.1–124.5)	-3.6 (-11.2–5.0)
11	Stroke	2.8 (2.4–3.1)	0.0143 (0.0123–0.0160)	539.7 (467.9–604.1)	24.9 (12.5–39.0)	-36.5 (-42.5–-29.4)
12	Falls	2.6 (2.2–3.0)	0.0135 (0.0108–0.0171)	542.0 (433.1–687.4)	131.4 (113.7–151.0)	19.4 (11.6–28.7)
13	Anxiety disorders	2.2 (1.5–3.0)	0.0115 (0.00743–0.0161)	785.6 (506.2–1122.1)	53.5 (19.3–94.6)	28.6 (0.6–62.5)
14	Chronic kidney disease	2.1 (1.8–2.4)	0.0109 (0.00923–0.0127)	419.5 (356.6–486.8)	288.5 (237.8–358.3)	96.7 (72.5–130.7)
15	Age-related and other hearing loss	2.0 (1.5–2.6)	0.0104 (0.00725–0.0145)	415.9 (289.7–578.0)	81.4 (73.9–89.9)	-5.6 (-9.0–-1.7)
16	Headache disorders	1.9 (0.4–3.7)	0.00990 (0.00226–0.0207)	682.3 (135.2–1442.2)	15.0 (7.6–32.5)	-3.5 (-8.7–2.6)
17	Self-harm	1.9 (1.6–2.2)	0.00989 (0.00813–0.0118)	691.5 (570.8–820.7)	30.8 (7.0–57.5)	12.5 (-7.5–34.8)
18	Osteoarthritis	1.6 (0.9–3.1)	0.00851 (0.00414–0.0172)	320.9 (155.8–646.2)	119.6 (113.0–127.0)	5.4 (2.4–8.7)
19	Road injuries	1.5 (1.4–1.7)	0.00805 (0.00674–0.00932)	527.0 (447.7–604.5)	-32.9 (-40.6–-25.3)	-48.8 (-54.3–-42.6)
20	Cirrhosis and other chronic liver diseases	1.4 (1.2–1.6)	0.00720 (0.00599–0.00850)	331.4 (277.0–391.2)	84.4 (51.8–117.2)	5.6 (-13.1–24.8)
21	Colon and rectum cancer	1.3 (1.1–1.6)	0.00690 (0.00578–0.00827)	267.0 (224.2–318.8)	13.0 (-5.5–35.0)	-42.9 (-52.1–-31.7)
22	Alcohol use disorders	1.2 (1.1–1.4)	0.00644 (0.00511–0.00779)	402.0 (317.1–496.6)	33.6 (16.6–55.6)	4.9 (-5.8–19.7)
23	Breast cancer	1.2 (1.0–1.5)	0.00637 (0.00525–0.00763)	261.4 (215.9–314.7)	2.5 (-14.2–23.6)	-47.1 (-56.1–-36.1)
24	Oral disorders	1.1 (0.7–1.6)	0.00589 (0.00365–0.00864)	275.3 (171.0–408.4)	61.5 (34.8–96.0)	-5.1 (-19.9–12.6)
25	Endocrine, metabolic, blood, and immune disorders	1.1 (1.0–1.3)	0.00582 (0.00489–0.00682)	289.3 (242.7–339.4)	177.7 (136.6–226.0)	66.6 (44.6–90.7)

Table S4: Cause-specific DALYs by US state in 2021 and percentage change between 1990 and 2021, New Jersey

Rank	Cause Name	2021 Percentage of all cause DALYs	2021 DALYs (millions)	2021 Age Standardised Rate (per 100 000)	Percentage change DALY count 1990 to 2021	Percentage change age-standardised DALY rate 1990 to 2021
	All causes	100.0 (100.0–100.0)	3.16 (2.74–3.61)	24895.9 (21319.8–28733.6)	18.9 (10.5–27.3)	-13.5 (-19.3—7.8)
1	COVID-19	7.3 (6.3–8.3)	0.228 (0.221–0.243)	1693.7 (1618.6–1850.8)	--	--
2	Ischaemic heart disease	7.0 (5.8–8.2)	0.221 (0.185–0.257)	1288.5 (1083.3–1500.6)	-37.3 (-46.1—27.1)	-61.6 (-67.4—55.3)
3	Drug use disorders	6.1 (5.3–6.9)	0.192 (0.158–0.223)	2080.8 (1723.1–2436.0)	478.4 (398.7–582.7)	445.8 (373.6–539.6)
4	Low back pain	4.3 (3.4–5.1)	0.135 (0.0973–0.177)	1135.6 (812.4–1486.9)	20.9 (12.8–30.5)	-7.5 (-13.6–0.3)
5	Diabetes mellitus	4.2 (3.7–4.8)	0.135 (0.106–0.170)	878.8 (688.5–1113.6)	89.1 (64.3–112.1)	24.6 (8.4–40.1)
6	Other musculoskeletal disorders	3.6 (2.7–4.5)	0.113 (0.0813–0.150)	981.6 (706.1–1308.9)	73.0 (55.8–93.4)	35.8 (22.3–52.2)
7	Chronic obstructive pulmonary disease	3.3 (2.8–3.7)	0.103 (0.0909–0.115)	596.6 (528.4–665.6)	55.8 (41.7–71.6)	-3.2 (-12.0–6.8)
8	Alzheimer's disease and other dementias	3.1 (1.4–6.5)	0.0965 (0.0434–0.205)	500.7 (227.0–1066.7)	70.5 (54.9–88.4)	-4.2 (-12.7–5.6)
9	Stroke	2.9 (2.6–3.3)	0.0920 (0.0785–0.105)	558.9 (479.9–634.3)	2.9 (-8.9–15.2)	-35.8 (-43.1—28.1)
10	Tracheal, bronchus, and lung cancer	2.7 (2.2–3.2)	0.0857 (0.0704–0.102)	512.4 (422.5–611.7)	-31.6 (-43.7—19.4)	-58.7 (-66.0—51.3)
11	Depressive disorders	2.5 (1.7–3.4)	0.0788 (0.0511–0.111)	832.4 (544.3–1195.0)	64.1 (33.5–100.8)	48.1 (20.1–80.4)
12	Anxiety disorders	2.5 (1.6–3.4)	0.0786 (0.0504–0.111)	798.3 (512.1–1132.4)	49.0 (16.7–87.8)	30.3 (1.7–63.3)
13	Chronic kidney disease	2.3 (2.0–2.7)	0.0733 (0.0618–0.0842)	457.8 (389.3–525.0)	165.1 (128.6–205.7)	64.1 (42.1–88.3)
14	Headache disorders	2.1 (0.5–4.2)	0.0674 (0.0149–0.142)	688.4 (136.5–1447.3)	11.8 (5.9–22.2)	-3.2 (-8.4–2.4)
15	Age-related and other hearing loss	2.0 (1.5–2.6)	0.0636 (0.0444–0.0878)	415.9 (289.5–573.0)	42.3 (36.9–48.4)	-5.5 (-8.8—1.8)
16	Falls	1.8 (1.6–2.1)	0.0577 (0.0447–0.0754)	378.2 (291.3–491.7)	63.0 (51.5–74.0)	1.9 (-4.6–8.3)
17	Osteoarthritis	1.6 (0.8–3.0)	0.0502 (0.0242–0.101)	316.0 (151.3–635.6)	65.4 (61.2–70.9)	5.4 (2.8–8.6)
18	Colon and rectum cancer	1.4 (1.2–1.7)	0.0452 (0.0370–0.0542)	288.8 (237.2–347.3)	-19.8 (-34.4—4.4)	-46.8 (-56.6—36.5)
19	Road injuries	1.4 (1.3–1.5)	0.0430 (0.0368–0.0493)	415.7 (358.1–475.6)	-45.2 (-51.7—39.3)	-56.7 (-61.9—51.6)
20	Breast cancer	1.3 (1.1–1.6)	0.0418 (0.0329–0.0517)	279.2 (217.2–345.9)	-24.2 (-40.0—4.7)	-50.5 (-61.3—37.6)
21	Asthma	1.3 (0.9–1.7)	0.0409 (0.0279–0.0590)	456.7 (307.6–677.1)	22.3 (14.1–30.6)	4.8 (-2.3–12.7)
22	Cirrhosis and other chronic liver diseases	1.3 (1.1–1.5)	0.0397 (0.0328–0.0471)	286.4 (236.5–338.9)	-3.7 (-20.2–14.9)	-34.6 (-46.2—21.9)
23	Oral disorders	1.1 (0.7–1.6)	0.0360 (0.0214–0.0549)	269.0 (156.4–411.2)	30.7 (19.5–42.0)	-6.7 (-14.8–2.3)
24	Endocrine, metabolic, blood, and immune disorders	1.1 (0.9–1.2)	0.0338 (0.0286–0.0397)	261.6 (220.6–310.0)	96.8 (73.7–127.0)	32.9 (18.9–51.0)
25	Neonatal disorders	1.1 (0.9–1.2)	0.0338 (0.0281–0.0392)	541.9 (468.1–618.8)	-53.2 (-59.0—47.6)	-53.4 (-59.1—48.2)

Table S4: Cause-specific DALYs by US state in 2021 and percentage change between 1990 and 2021, New Mexico						
**Rank**	**Cause Name**	**2021 Percentage of all cause DALYs**	**2021 DALYs (millions)**	**2021 Age Standardised Rate (per 100 000)**	**Percentage change DALY count 1990 to 2021**	**Percentage change age-standardised DALY rate 1990 to 2021**
	All causes	100.0 (100.0–100.0)	0.927 (0.807–1.05)	33298.9 (28839.2–37604.5)	95.5 (79.8–111.4)	13.7 (4.9–22.1)
1	COVID-19	9.3 (7.4–11.3)	0.0857 (0.0725–0.105)	2786.6 (2342.4–3421.2)	--	--
2	Ischaemic heart disease	6.8 (5.8–7.8)	0.0626 (0.0527–0.0730)	1633.5 (1376.0–1909.4)	35.4 (15.9–56.0)	-40.1 (-49.1—30.5)
3	Drug use disorders	6.1 (5.3–6.9)	0.0566 (0.0465–0.0661)	2784.2 (2285.1–3254.6)	560.4 (474.0–682.5)	431.3 (362.6–524.6)
4	Diabetes mellitus	4.0 (3.5–4.6)	0.0373 (0.0302–0.0463)	1095.1 (888.1–1346.4)	206.5 (171.6–240.9)	51.2 (34.3–68.2)
5	Low back pain	3.7 (2.9–4.4)	0.0341 (0.0251–0.0442)	1282.0 (931.8–1675.2)	55.1 (43.9–67.3)	-4.7 (-11.4–2.7)
6	Chronic obstructive pulmonary disease	3.5 (3.1–3.9)	0.0324 (0.0286–0.0365)	805.3 (710.6–907.5)	133.0 (109.6–162.2)	0.9 (-9.3–13.3)
7	Depressive disorders	2.9 (2.1–3.8)	0.0267 (0.0178–0.0383)	1258.2 (825.2–1778.4)	114.8 (73.2–160.4)	61.9 (30.1–97.2)
8	Other musculoskeletal disorders	2.8 (2.2–3.5)	0.0257 (0.0186–0.0340)	1016.2 (732.1–1348.3)	139.8 (113.8–169.2)	52.9 (37.0–72.1)
9	Cirrhosis and other chronic liver diseases	2.7 (2.4–3.1)	0.0255 (0.0215–0.0298)	929.1 (787.0–1087.1)	171.6 (128.5–220.9)	61.7 (36.4–91.2)
10	Road injuries	2.6 (2.4–2.8)	0.0239 (0.0208–0.0274)	1100.4 (961.6–1251.6)	-20.3 (-30.3—10.5)	-43.0 (-50.0—35.8)
11	Self-harm	2.6 (2.2–2.9)	0.0236 (0.0200–0.0274)	1119.5 (947.4–1296.4)	62.3 (35.9–90.1)	22.9 (2.9–43.7)
12	Falls	2.5 (2.1–2.9)	0.0229 (0.0187–0.0287)	657.9 (531.9–820.5)	149.6 (130.7–170.0)	18.1 (9.7–26.9)
13	Alzheimer's disease and other dementias	2.5 (1.2–5.0)	0.0229 (0.0108–0.0465)	503.9 (237.7–1021.6)	160.3 (138.0–185.1)	-3.4 (-11.0–6.1)
14	Stroke	2.5 (2.2–2.7)	0.0228 (0.0195–0.0260)	612.0 (524.5–698.2)	65.7 (46.4–86.8)	-24.9 (-33.4—15.5)
15	Alcohol use disorders	2.3 (2.1–2.5)	0.0212 (0.0179–0.0249)	913.7 (769.8–1087.6)	94.2 (66.5–127.2)	36.2 (18.1–58.5)
16	Chronic kidney disease	2.1 (1.9–2.3)	0.0196 (0.0169–0.0224)	555.0 (486.0–633.5)	363.3 (308.8–428.7)	118.7 (95.0–146.9)
17	Tracheal, bronchus, and lung cancer	2.0 (1.7–2.3)	0.0183 (0.0153–0.0216)	491.5 (410.8–580.2)	22.5 (2.7–43.5)	-44.7 (-53.6—35.1)
18	Anxiety disorders	1.9 (1.3–2.6)	0.0178 (0.0114–0.0252)	811.0 (517.3–1151.2)	81.7 (40.1–131.9)	33.0 (2.6–67.2)
19	Age-related and other hearing loss	1.6 (1.2–2.1)	0.0150 (0.0105–0.0208)	419.2 (294.2–581.0)	98.3 (89.9–107.0)	-6.2 (-9.4—2.9)
20	Headache disorders	1.6 (0.4–3.2)	0.0148 (0.00327–0.0309)	678.2 (136.0–1431.2)	31.3 (24.2–48.4)	-4.0 (-8.7–2.2)
21	Osteoarthritis	1.2 (0.6–2.3)	0.0113 (0.00553–0.0227)	310.2 (150.8–621.2)	131.6 (123.9–139.4)	6.5 (3.3–9.5)
22	Colon and rectum cancer	1.2 (1.0–1.4)	0.0111 (0.00914–0.0131)	325.0 (268.6–385.0)	64.7 (33.9–96.6)	-18.3 (-33.4—2.3)
23	Interpersonal violence	1.2 (1.1–1.3)	0.0110 (0.00945–0.0125)	570.8 (492.7–648.0)	9.4 (-5.4–25.4)	-11.4 (-23.6–1.5)
24	Asthma	1.1 (0.8–1.4)	0.0101 (0.00692–0.0140)	482.4 (327.7–687.2)	33.6 (23.1–43.6)	-1.9 (-8.8–5.5)
25	Breast cancer	1.1 (0.9–1.3)	0.00993 (0.00795–0.0121)	311.1 (247.9–384.0)	51.2 (20.2–90.5)	-21.5 (-38.0—0.4)

Table S4: Cause-specific DALYs by US state in 2021 and percentage change between 1990 and 2021, New York						
Rank	Cause Name	2021 Percentage of all cause DALYs	2021 DALYs (millions)	2021 Age Standardised Rate (per 100 000)	Percentage change DALY count 1990 to 2021	Percentage change age-standardised DALY rate 1990 to 2021
	All causes	100.0 (100.0–100.0)	6.97 (5.97–7.94)	25205.4 (21418.9–29069.6)	7.1 (-0.5–14.3)	-18.1 (-23.8–-12.8)
1	Ischaemic heart disease	8.4 (7.0–9.8)	0.584 (0.484–0.675)	1563.9 (1299.6–1810.3)	-38.3 (-47.0–-29.3)	-60.0 (-65.8–-54.0)
2	COVID-19	7.0 (6.1–8.2)	0.489 (0.472–0.533)	1657.3 (1583.2–1840.0)	--	--
3	Drug use disorders	5.0 (4.4–5.6)	0.348 (0.293–0.404)	1677.5 (1409.2–1964.2)	252.2 (208.7–307.0)	244.9 (202.2–296.5)
4	Diabetes mellitus	4.4 (3.8–5.1)	0.311 (0.239–0.393)	942.7 (723.6–1196.4)	145.7 (117.0–171.8)	69.4 (49.8–87.0)
5	Low back pain	4.2 (3.4–5.1)	0.295 (0.216–0.384)	1136.5 (828.1–1491.6)	18.2 (9.7–27.1)	-5.1 (-11.2–1.8)
6	Other musculoskeletal disorders	3.9 (3.0–4.9)	0.270 (0.195–0.356)	1075.2 (774.7–1424.8)	73.1 (56.0–96.0)	43.4 (29.7–61.4)
7	Chronic obstructive pulmonary disease	3.2 (2.8–3.7)	0.225 (0.200–0.250)	608.6 (543.5–673.6)	44.4 (30.8–59.4)	-4.6 (-13.2–5.2)
8	Alzheimer's disease and other dementias	3.2 (1.5–6.5)	0.220 (0.104–0.443)	505.7 (240.5–1023.9)	61.2 (46.8–78.0)	-3.5 (-11.7–6.1)
9	Depressive disorders	3.1 (2.2–4.2)	0.218 (0.147–0.313)	1055.8 (710.1–1528.0)	54.5 (27.2–88.8)	47.8 (21.6–81.4)
10	Tracheal, bronchus, and lung cancer	2.7 (2.3–3.2)	0.190 (0.158–0.224)	531.7 (440.7–626.8)	-28.6 (-41.2–-15.2)	-54.5 (-62.3–-46.0)
11	Anxiety disorders	2.5 (1.7–3.4)	0.172 (0.108–0.251)	803.4 (502.2–1164.6)	41.3 (8.7–84.6)	30.9 (1.3–68.7)
12	Stroke	2.4 (2.1–2.6)	0.165 (0.143–0.189)	471.2 (409.8–538.4)	-15.0 (-23.8–-5.2)	-42.8 (-48.9–-36.2)
13	Headache disorders	2.1 (0.5–4.2)	0.148 (0.0322–0.309)	693.7 (135.7–1467.7)	5.3 (-0.1–17.0)	-3.3 (-8.5–3.1)
14	Chronic kidney disease	2.1 (1.8–2.3)	0.143 (0.123–0.164)	412.3 (356.7–469.3)	121.6 (95.6–147.5)	44.3 (27.9–60.4)
15	Age-related and other hearing loss	2.0 (1.5–2.5)	0.138 (0.0962–0.189)	414.7 (288.8–569.7)	34.4 (29.2–40.5)	-5.6 (-9.1–-1.9)
16	Falls	1.9 (1.6–2.2)	0.132 (0.104–0.169)	395.8 (309.7–509.1)	45.2 (35.8–55.7)	-4.5 (-9.9–1.8)
17	Osteoarthritis	1.6 (0.9–3.1)	0.111 (0.0539–0.222)	326.7 (157.5–652.7)	54.9 (50.4–59.7)	5.0 (1.7–8.4)
18	Asthma	1.4 (1.1–1.9)	0.100 (0.0689–0.142)	509.8 (347.6–748.5)	6.2 (-0.6–12.5)	-2.8 (-9.2–3.5)
19	Colon and rectum cancer	1.3 (1.1–1.5)	0.0902 (0.0754–0.107)	270.1 (225.4–321.5)	-24.9 (-37.3–-11.0)	-47.0 (-56.0–-37.3)
20	Self-harm	1.2 (1.0–1.4)	0.0840 (0.0714–0.0987)	392.1 (334.1–458.7)	-0.4 (-16.1–17.3)	-6.7 (-21.6–9.5)
21	Oral disorders	1.2 (0.8–1.7)	0.0829 (0.0483–0.125)	289.4 (167.1–439.6)	28.0 (17.4–39.2)	-2.4 (-11.1–7.9)
22	Breast cancer	1.2 (1.0–1.4)	0.0822 (0.0661–0.100)	257.0 (206.9–315.2)	-31.7 (-44.5–-16.1)	-52.9 (-62.1–-42.1)
23	Road injuries	1.2 (1.1–1.3)	0.0821 (0.0701–0.0953)	365.5 (315.5–421.6)	-54.6 (-59.5–-49.8)	-61.5 (-65.6–-57.5)
24	Hypertensive heart disease	1.2 (0.9–1.4)	0.0821 (0.0652–0.102)	237.6 (189.1–296.0)	75.9 (42.2–120.0)	16.3 (-6.2–45.6)
25	Alcohol use disorders	1.1 (1.0–1.3)	0.0776 (0.0628–0.0969)	335.9 (268.0–423.5)	-5.7 (-15.0–6.6)	-16.8 (-24.1–-7.6)

Table S4: Cause-specific DALYs by US state in 2021 and percentage change between 1990 and 2021, North Carolina						
Rank	Cause Name	2021 Percentage of all cause DALYs	2021 DALYs (millions)	2021 Age Standardised Rate (per 100 000)	Percentage change DALY count 1990 to 2021	Percentage change age-standardised DALY rate 1990 to 2021
	All causes	100.0 (100.0–100.0)	4.24 (3.73–4.79)	30494.0 (26676.8–34672.9)	86.8 (72.5–100.7)	1.4 (-5.6–8.4)
1	COVID-19	8.3 (6.5–10.7)	0.351 (0.292–0.444)	2367.8 (1953.9–2997.6)	--	--
2	Ischaemic heart disease	6.5 (5.6–7.5)	0.277 (0.235–0.323)	1546.3 (1309.6–1793.9)	-8.0 (-21.0–6.2)	-57.7 (-63.9–-51.0)
3	Drug use disorders	6.1 (5.3–6.8)	0.258 (0.218–0.297)	2487.9 (2091.0–2881.5)	960.4 (791.7–1174.5)	669.0 (548.0–820.1)
4	Diabetes mellitus	4.3 (3.7–4.9)	0.182 (0.145–0.226)	1090.8 (864.5–1348.4)	227.0 (191.9–257.7)	57.4 (40.9–72.0)
5	Chronic obstructive pulmonary disease	3.8 (3.4–4.3)	0.163 (0.144–0.182)	858.9 (762.6–959.4)	172.6 (141.7–204.5)	21.9 (8.1–36.3)
6	Low back pain	3.5 (2.8–4.3)	0.149 (0.108–0.193)	1118.1 (807.2–1454.2)	54.8 (42.5–66.9)	-11.6 (-18.2–-5.2)
7	Stroke	3.4 (3.0–3.8)	0.146 (0.127–0.163)	823.8 (724.0–920.8)	49.4 (32.3–66.7)	-30.9 (-38.5–-23.2)
8	Tracheal, bronchus, and lung cancer	3.2 (2.7–3.8)	0.137 (0.115–0.161)	745.5 (625.8–875.8)	27.1 (7.1–50.6)	-44.1 (-53.0–-33.8)
9	Alzheimer's disease and other dementias	2.7 (1.4–5.6)	0.117 (0.0565–0.242)	575.1 (278.4–1191.3)	139.3 (119.8–160.8)	-1.8 (-9.6–6.2)
10	Other musculoskeletal disorders	2.7 (2.1–3.5)	0.115 (0.0831–0.152)	895.5 (646.0–1194.5)	132.3 (107.8–160.4)	37.6 (22.5–54.0)
11	Chronic kidney disease	2.5 (2.3–2.8)	0.107 (0.0934–0.121)	627.7 (546.7–703.2)	332.1 (278.6–391.1)	100.5 (75.6–126.5)
12	Road injuries	2.3 (2.1–2.5)	0.0972 (0.0842–0.111)	868.8 (759.6–983.1)	-8.1 (-18.5–2.7)	-41.6 (-48.3–-34.9)
13	Depressive disorders	2.2 (1.5–3.0)	0.0939 (0.0619–0.137)	876.7 (569.7–1286.2)	167.5 (117.2–228.6)	84.6 (49.4–126.5)
14	Anxiety disorders	2.1 (1.4–3.0)	0.0893 (0.0572–0.128)	811.1 (521.5–1179.7)	87.2 (44.3–138.9)	25.2 (-3.2–59.2)
15	Falls	2.0 (1.7–2.3)	0.0844 (0.0681–0.107)	495.7 (398.6–627.3)	145.2 (125.8–166.0)	12.5 (4.6–21.3)
16	Headache disorders	1.8 (0.4–3.6)	0.0759 (0.0167–0.160)	690.8 (137.4–1464.3)	46.4 (38.6–61.3)	-2.8 (-7.3–2.3)
17	Age-related and other hearing loss	1.7 (1.2–2.2)	0.0708 (0.0495–0.0982)	418.1 (293.7–580.2)	96.1 (88.3–104.1)	-5.7 (-8.8–-2.5)
18	Self-harm	1.5 (1.3–1.8)	0.0649 (0.0545–0.0753)	588.7 (494.9–683.5)	51.1 (26.5–76.3)	2.1 (-14.0–19.1)
19	Cirrhosis and other chronic liver diseases	1.5 (1.3–1.7)	0.0645 (0.0545–0.0751)	423.3 (356.7–493.2)	113.3 (80.5–148.2)	7.5 (-9.2–25.6)
20	Neonatal disorders	1.4 (1.2–1.6)	0.0593 (0.0521–0.0672)	932.2 (831.5–1041.9)	-12.9 (-21.8–-2.6)	-27.1 (-34.6–-18.7)
21	Colon and rectum cancer	1.3 (1.1–1.5)	0.0548 (0.0463–0.0642)	323.3 (273.7–378.8)	49.6 (25.5–80.0)	-27.8 (-39.5–-12.9)
22	Osteoarthritis	1.1 (0.6–2.2)	0.0487 (0.0238–0.0988)	277.5 (134.5–563.5)	129.2 (121.5–137.8)	6.3 (2.7–10.1)
23	Endocrine, metabolic, blood, and immune disorders	1.1 (1.0–1.3)	0.0483 (0.0411–0.0555)	346.9 (295.7–400.2)	224.4 (180.7–275.2)	69.3 (49.4–92.6)
24	Breast cancer	1.1 (0.9–1.3)	0.0478 (0.0380–0.0583)	294.8 (234.7–361.6)	27.2 (1.7–56.5)	-38.4 (-51.2–-24.0)
25	Oral disorders	1.1 (0.7–1.5)	0.0460 (0.0281–0.0682)	308.5 (182.3–469.6)	104.2 (95.4–115.8)	6.8 (2.4–11.7)

Table S4: Cause-specific DALYs by US state in 2021 and percentage change between 1990 and 2021, North Dakota						
Rank	Cause Name	2021 Percentage of all cause DALYs	2021 DALYs (millions)	2021 Age Standardised Rate (per 100 000)	Percentage change DALY count 1990 to 2021	Percentage change age-standardised DALY rate 1990 to 2021
	All causes	100.0 (100.0–100.0)	0.261 (0.225–0.298)	25471.6 (21758.7–29290.8)	32.7 (24.2–41.7)	-1.1 (-7.4–5.3)
1	Ischaemic heart disease	6.7 (5.6–7.8)	0.0174 (0.0147–0.0199)	1267.7 (1070.6–1458.4)	-34.2 (-43.3—25.0)	-56.0 (-62.4—49.1)
2	COVID-19	6.6 (5.8–7.6)	0.0172 (0.0165–0.0189)	1549.2 (1461.8–1753.6)	--	--
3	Other musculoskeletal disorders	5.0 (3.8–6.3)	0.0131 (0.00943–0.0173)	1477.5 (1068.1–1959.3)	100.0 (85.0–118.3)	59.4 (47.3–74.5)
4	Low back pain	4.3 (3.4–5.1)	0.0112 (0.00819–0.0147)	1173.3 (856.2–1547.1)	19.0 (11.5–26.4)	-10.4 (-15.4—4.7)
5	Diabetes mellitus	4.3 (3.7–4.9)	0.0111 (0.00872–0.0142)	903.9 (706.1–1136.0)	124.1 (99.4–147.5)	51.4 (35.4–66.2)
6	Chronic obstructive pulmonary disease	3.5 (3.0–3.9)	0.00902 (0.00793–0.0100)	632.1 (556.0–700.9)	62.4 (46.9–78.4)	6.3 (-3.7–16.8)
7	Alzheimer's disease and other dementias	3.3 (1.5–6.7)	0.00852 (0.00405–0.0174)	505.8 (241.9–1031.2)	48.4 (36.4–60.9)	-2.8 (-10.7–5.2)
8	Stroke	3.0 (2.6–3.4)	0.00793 (0.00687–0.00899)	587.2 (515.5–663.0)	1.0 (-8.9–12.7)	-29.4 (-36.3—21.0)
9	Drug use disorders	2.7 (2.3–3.2)	0.00717 (0.00566–0.00856)	950.8 (747.0–1138.7)	381.0 (313.0–473.4)	327.9 (267.1–409.0)
10	Tracheal, bronchus, and lung cancer	2.7 (2.3–3.2)	0.00703 (0.00590–0.00812)	528.3 (443.6–613.1)	-5.7 (-20.6–9.9)	-42.3 (-51.5—32.5)
11	Road injuries	2.6 (2.4–2.8)	0.00685 (0.00598–0.00775)	850.7 (751.7–959.3)	-16.8 (-25.1—7.9)	-32.4 (-39.4—24.9)
12	Depressive disorders	2.5 (1.8–3.3)	0.00647 (0.00427–0.00936)	825.7 (538.3–1206.5)	61.4 (31.8–96.1)	39.7 (13.9–69.0)
13	Falls	2.4 (2.1–2.8)	0.00629 (0.00494–0.00801)	502.5 (397.7–637.2)	94.5 (82.3–107.8)	26.4 (19.1–35.2)
14	Self-harm	2.4 (2.0–2.8)	0.00627 (0.00528–0.00735)	809.6 (682.1–948.7)	68.1 (41.7–99.7)	45.3 (22.7–72.7)
15	Anxiety disorders	2.3 (1.5–3.2)	0.00598 (0.00384–0.00876)	735.7 (471.7–1083.7)	44.9 (12.9–83.6)	21.0 (-6.7–52.6)
16	Chronic kidney disease	2.1 (1.6–2.5)	0.00544 (0.00403–0.00651)	416.8 (314.7–493.3)	177.1 (112.4–230.1)	87.1 (46.4–121.7)
17	Headache disorders	2.1 (0.5–4.2)	0.00543 (0.00121–0.0114)	677.9 (137.0–1436.2)	15.8 (10.0–27.4)	-3.6 (-8.1–1.6)
18	Age-related and other hearing loss	2.0 (1.5–2.6)	0.00531 (0.00372–0.00726)	420.9 (294.5–578.4)	37.2 (31.4–43.3)	-5.5 (-9.2—1.8)
19	Osteoarthritis	1.5 (0.8–2.8)	0.00385 (0.00187–0.00781)	302.3 (145.0–611.0)	66.6 (61.8–72.1)	8.3 (5.0–11.9)
20	Cirrhosis and other chronic liver diseases	1.4 (1.2–1.7)	0.00376 (0.00318–0.00435)	372.0 (314.4–428.7)	82.7 (55.9–112.8)	28.8 (9.2–50.4)
21	Alcohol use disorders	1.4 (1.3–1.6)	0.00366 (0.00301–0.00446)	429.5 (352.8–530.2)	42.3 (24.6–65.9)	13.3 (0.7–30.5)
22	Colon and rectum cancer	1.4 (1.1–1.6)	0.00353 (0.00293–0.00409)	279.6 (235.8–325.3)	-6.0 (-21.1–10.3)	-36.0 (-46.5—24.7)
23	Asthma	1.3 (1.0–1.7)	0.00339 (0.00230–0.00486)	421.0 (284.4–616.0)	24.2 (15.7–32.6)	1.2 (-5.7–8.1)
24	Neonatal disorders	1.3 (1.1–1.5)	0.00332 (0.00282–0.00389)	607.8 (519.7–710.3)	-14.2 (-27.5–1.3)	-23.1 (-35.5—7.5)
25	Endocrine, metabolic, blood, and immune disorders	1.3 (1.1–1.4)	0.00329 (0.00282–0.00374)	326.6 (281.2–372.0)	163.0 (126.9–209.3)	83.2 (58.8–112.2)

Table S4: Cause-specific DALYs by US state in 2021 and percentage change between 1990 and 2021, Ohio						
**Rank**	**Cause Name**	**2021 Percentage of all cause DALYs**	**2021 DALYs (millions)**	**2021 Age Standardised Rate (per 100 000)**	**Percentage change DALY count 1990 to 2021**	**Percentage change age-standardised DALY rate 1990 to 2021**
	All causes	100.0 (100.0–100.0)	5.13 (4.51–5.76)	31980.3 (27846.8–36084.6)	39.1 (29.9–49.7)	10.2 (3.4–17.2)
1	COVID-19	8.4 (7.3–10.0)	0.429 (0.404–0.496)	2509.5 (2335.3–2884.9)	--	--
2	Ischaemic heart disease	7.4 (6.4–8.5)	0.380 (0.326–0.434)	1776.8 (1519.7–2037.7)	-28.7 (-37.9–-18.6)	-52.2 (-58.6–-45.1)
3	Drug use disorders	7.1 (6.3–8.0)	0.365 (0.312–0.419)	3219.8 (2749.6–3681.3)	962.6 (804.0–1190.3)	1002.2 (838.8–1231.5)
4	Diabetes mellitus	4.2 (3.7–4.8)	0.218 (0.175–0.270)	1107.2 (882.2–1364.7)	116.3 (94.2–137.2)	51.2 (35.8–65.5)
5	Chronic obstructive pulmonary disease	4.2 (3.7–4.7)	0.214 (0.189–0.237)	958.0 (847.2–1064.6)	84.6 (65.8–105.9)	21.0 (8.7–34.6)
6	Low back pain	3.5 (2.8–4.4)	0.182 (0.132–0.237)	1215.7 (878.7–1589.4)	12.2 (4.5–21.2)	-7.1 (-13.0–-0.3)
7	Tracheal, bronchus, and lung cancer	3.4 (2.9–3.9)	0.172 (0.144–0.199)	796.6 (669.3–924.7)	-7.8 (-22.9–8.2)	-41.3 (-51.1–-31.0)
8	Stroke	3.3 (2.9–3.6)	0.167 (0.145–0.188)	787.3 (689.2–885.9)	23.3 (10.9–37.3)	-17.0 (-25.5–-7.6)
9	Other musculoskeletal disorders	2.8 (2.1–3.6)	0.146 (0.105–0.193)	1018.0 (730.5–1353.5)	63.9 (47.6–83.5)	40.9 (25.5–57.4)
10	Chronic kidney disease	2.4 (2.2–2.7)	0.124 (0.109–0.140)	611.0 (538.0–683.1)	227.2 (189.5–269.8)	119.0 (95.0–147.7)
11	Depressive disorders	2.4 (1.7–3.1)	0.122 (0.0803–0.174)	1036.5 (681.7–1465.6)	57.9 (30.1–89.9)	57.1 (29.4–87.9)
12	Alzheimer's disease and other dementias	2.4 (1.1–5.0)	0.122 (0.0569–0.265)	496.2 (234.2–1076.2)	55.5 (43.7–69.4)	-3.9 (-11.2–4.4)
13	Falls	1.9 (1.7–2.3)	0.0993 (0.0794–0.125)	496.4 (396.6–626.9)	74.8 (61.1–90.7)	15.2 (6.6–25.1)
14	Anxiety disorders	1.9 (1.3–2.7)	0.0989 (0.0615–0.142)	807.1 (499.8–1175.1)	36.9 (6.3–75.3)	31.9 (2.3–69.0)
15	Headache disorders	1.6 (0.4–3.2)	0.0832 (0.0185–0.175)	682.5 (136.3–1452.3)	0.3 (-5.4–11.9)	-4.1 (-9.0–1.5)
16	Age-related and other hearing loss	1.6 (1.2–2.1)	0.0825 (0.0580–0.114)	416.1 (293.4–574.2)	34.6 (29.4–39.7)	-5.8 (-8.6–-2.3)
17	Self-harm	1.6 (1.4–1.8)	0.0818 (0.0699–0.0950)	682.7 (588.5–791.1)	30.2 (11.0–52.7)	28.0 (9.3–49.6)
18	Road injuries	1.6 (1.4–1.7)	0.0795 (0.0690–0.0910)	630.8 (555.0–717.5)	-40.5 (-47.0–-33.5)	-46.8 (-52.5–-40.7)
19	Osteoarthritis	1.4 (0.7–2.7)	0.0713 (0.0351–0.144)	348.5 (169.4–704.3)	56.5 (51.8–61.9)	5.2 (2.1–8.7)
20	Cirrhosis and other chronic liver diseases	1.4 (1.2–1.5)	0.0692 (0.0588–0.0806)	395.7 (337.7–459.4)	76.0 (48.8–104.5)	29.8 (9.6–50.7)
21	Colon and rectum cancer	1.4 (1.1–1.6)	0.0691 (0.0575–0.0806)	346.1 (288.0–402.7)	-4.6 (-21.7–15.1)	-32.9 (-44.8–-19.0)
22	Neonatal disorders	1.2 (1.1–1.3)	0.0611 (0.0535–0.0688)	823.4 (734.9–916.4)	-34.0 (-41.2–-26.2)	-22.2 (-30.6–-12.9)
23	Endocrine, metabolic, blood, and immune disorders	1.2 (1.0–1.3)	0.0590 (0.0509–0.0682)	359.2 (311.0–416.1)	155.2 (120.9–196.6)	84.9 (63.2–111.9)
24	Breast cancer	1.1 (0.9–1.3)	0.0569 (0.0461–0.0688)	301.0 (243.2–368.0)	-17.5 (-32.7–-1.1)	-42.3 (-53.3–-30.0)
25	Hypertensive heart disease	1.1 (0.9–1.2)	0.0538 (0.0457–0.0621)	278.1 (235.6–321.8)	121.9 (90.1–155.7)	59.8 (36.5–84.9)

Table S4: Cause-specific DALYs by US state in 2021 and percentage change between 1990 and 2021, Oklahoma						
Rank	Cause Name	2021 Percentage of all cause DALYs	2021 DALYs (millions)	2021 Age Standardised Rate (per 100 000)	Percentage change DALY count 1990 to 2021	Percentage change age-standardised DALY rate 1990 to 2021
	All causes	100.0 (100.0–100.0)	1.78 (1.57–2.02)	34606.2 (30292.6–39520.7)	62.3 (51.5–74.5)	17.0 (9.7–24.7)
1	COVID-19	10.6 (9.2–12.5)	0.188 (0.179–0.218)	3484.8 (3285.6–4031.6)	--	--
2	Ischaemic heart disease	7.3 (6.4–8.4)	0.130 (0.112–0.152)	2035.7 (1763.9–2378.8)	-21.1 (-31.1—9.4)	-47.4 (-54.2—39.1)
3	Drug use disorders	5.2 (4.3–6.1)	0.0922 (0.0733–0.110)	2342.9 (1869.5–2806.4)	805.8 (677.9–979.7)	665.2 (560.2–808.9)
4	Chronic obstructive pulmonary disease	4.4 (3.9–4.9)	0.0776 (0.0695–0.0869)	1148.0 (1027.3–1284.5)	112.1 (91.5–137.3)	36.2 (22.6–52.7)
5	Diabetes mellitus	3.6 (3.1–4.1)	0.0642 (0.0511–0.0790)	1077.1 (853.6–1323.8)	167.1 (138.2–193.1)	78.1 (59.1–95.6)
6	Low back pain	3.6 (2.8–4.4)	0.0637 (0.0462–0.0822)	1325.4 (960.8–1723.2)	29.7 (21.5–39.1)	-3.7 (-9.7–3.3)
7	Tracheal, bronchus, and lung cancer	3.2 (2.8–3.7)	0.0574 (0.0491–0.0674)	880.0 (751.4–1037.6)	5.2 (-11.6–22.4)	-35.7 (-46.2—25.1)
8	Hypertensive heart disease	2.9 (2.5–3.3)	0.0522 (0.0447–0.0605)	847.0 (725.1–984.1)	815.4 (688.1–953.4)	507.7 (421.3–600.4)
9	Stroke	2.9 (2.6–3.2)	0.0517 (0.0457–0.0588)	822.5 (726.3–937.3)	13.6 (1.7–26.6)	-22.1 (-30.1—12.8)
10	Other musculoskeletal disorders	2.6 (2.0–3.4)	0.0471 (0.0340–0.0623)	1038.5 (750.8–1387.7)	98.6 (78.2–120.7)	54.1 (37.6–71.6)
11	Depressive disorders	2.6 (1.8–3.6)	0.0470 (0.0306–0.0664)	1169.2 (762.6–1653.1)	91.6 (56.5–133.8)	60.2 (31.1–94.3)
12	Chronic kidney disease	2.5 (2.3–2.8)	0.0453 (0.0396–0.0517)	726.4 (639.1–830.4)	307.5 (260.3–365.7)	167.0 (137.7–206.3)
13	Road injuries	2.3 (2.1–2.4)	0.0403 (0.0351–0.0463)	959.0 (845.0–1101.0)	-19.9 (-27.7—10.2)	-38.3 (-44.5—30.8)
14	Self-harm	2.1 (1.8–2.4)	0.0370 (0.0313–0.0430)	909.3 (771.6–1054.8)	78.6 (50.4–110.2)	47.0 (23.9–72.8)
15	Alzheimer's disease and other dementias	2.0 (1.0–4.3)	0.0362 (0.0167–0.0785)	495.5 (229.5–1067.8)	45.2 (33.4–58.4)	-4.3 (-11.8–3.9)
16	Falls	1.9 (1.7–2.2)	0.0345 (0.0282–0.0437)	561.5 (458.8–709.0)	112.7 (94.7–134.5)	33.8 (22.8–47.5)
17	Anxiety disorders	1.9 (1.3–2.6)	0.0334 (0.0213–0.0495)	810.2 (515.5–1200.7)	61.7 (24.7–102.2)	32.7 (2.6–66.5)
18	Cirrhosis and other chronic liver diseases	1.7 (1.5–2.0)	0.0309 (0.0265–0.0358)	580.4 (498.0–671.5)	150.2 (114.8–191.1)	72.8 (48.5–101.4)
19	Headache disorders	1.6 (0.3–3.2)	0.0278 (0.00610–0.0588)	677.6 (136.5–1438.8)	18.1 (12.2–28.2)	-4.2 (-8.7–1.6)
20	Age-related and other hearing loss	1.4 (1.1–1.9)	0.0256 (0.0179–0.0354)	419.0 (292.8–583.9)	39.0 (34.3–44.3)	-5.8 (-8.8—2.7)
21	Colon and rectum cancer	1.3 (1.1–1.5)	0.0228 (0.0193–0.0266)	374.8 (317.2–435.8)	26.3 (4.4–48.5)	-14.0 (-28.7–1.5)
22	Osteoarthritis	1.1 (0.6–2.1)	0.0201 (0.00981–0.0405)	321.7 (156.0–646.9)	63.0 (58.7–67.7)	5.7 (2.9–9.0)
23	Neonatal disorders	1.1 (0.9–1.3)	0.0195 (0.0167–0.0223)	722.9 (628.5–815.6)	-13.2 (-23.5—2.8)	-19.8 (-29.8—10.4)
24	Breast cancer	1.0 (0.8–1.2)	0.0182 (0.0147–0.0222)	312.3 (250.9–383.5)	3.8 (-17.5–26.1)	-32.4 (-46.4—17.3)
25	Alcohol use disorders	1.0 (0.9–1.1)	0.0180 (0.0148–0.0219)	409.0 (334.4–502.9)	45.5 (26.9–72.0)	12.2 (-0.9–31.1)

Table S4: Cause-specific DALYs by US state in 2021 and percentage change between 1990 and 2021, Oregon						
**Rank**	**Cause Name**	**2021 Percentage of all cause DALYs**	**2021 DALYs (millions)**	**2021 Age Standardised Rate (per 100 000)**	**Percentage change DALY count 1990 to 2021**	**Percentage change age-standardised DALY rate 1990 to 2021**
	All causes	100.0 (100.0–100.0)	1.60 (1.40–1.84)	26656.5 (23210.6–30845.7)	73.0 (60.1–87.5)	-2.1 (-9.2–5.6)
1	COVID-19	7.4 (5.6–9.9)	0.117 (0.0935–0.157)	1832.9 (1449.9–2433.9)	--	--
2	Ischaemic heart disease	5.3 (4.5–6.2)	0.0843 (0.0708–0.100)	1033.5 (866.2–1234.9)	-25.7 (-36.1—12.4)	-64.3 (-69.4—57.7)
3	Drug use disorders	4.3 (3.7–5.1)	0.0694 (0.0554–0.0824)	1636.4 (1303.0–1952.5)	569.3 (480.1–693.8)	381.5 (319.4–467.9)
4	Low back pain	4.1 (3.3–5.0)	0.0664 (0.0487–0.0866)	1200.9 (880.2–1575.9)	59.1 (47.8–70.3)	-5.2 (-11.4–1.5)
5	Diabetes mellitus	3.9 (3.5–4.4)	0.0624 (0.0499–0.0780)	853.9 (681.2–1068.1)	214.0 (181.3–247.1)	57.3 (41.4–73.4)
6	Chronic obstructive pulmonary disease	3.8 (3.4–4.3)	0.0610 (0.0539–0.0698)	716.4 (631.8–819.1)	100.0 (79.4–124.5)	-5.9 (-15.5–5.5)
7	Other musculoskeletal disorders	3.5 (2.7–4.4)	0.0553 (0.0395–0.0742)	1079.4 (779.7–1450.5)	152.2 (125.0–182.7)	58.2 (41.6–77.1)
8	Stroke	3.4 (3.0–3.8)	0.0538 (0.0463–0.0620)	668.6 (576.6–770.7)	42.3 (26.2–60.6)	-29.8 (-37.9—20.7)
9	Depressive disorders	3.0 (2.1–4.0)	0.0474 (0.0306–0.0667)	1100.5 (712.0–1553.8)	114.9 (78.8–159.1)	53.4 (27.2–84.8)
10	Tracheal, bronchus, and lung cancer	3.0 (2.5–3.4)	0.0473 (0.0394–0.0565)	584.8 (486.2–699.6)	4.2 (-14.5–25.2)	-52.8 (-61.1—43.3)
11	Alzheimer's disease and other dementias	2.9 (1.4–6.1)	0.0465 (0.0219–0.0975)	499.4 (238.8–1042.8)	110.8 (93.0–131.9)	-3.3 (-11.6–6.5)
12	Falls	2.6 (2.2–3.0)	0.0413 (0.0331–0.0521)	548.6 (434.4–692.1)	146.4 (127.2–166.1)	15.8 (7.8–24.5)
13	Self-harm	2.4 (2.0–2.8)	0.0377 (0.0320–0.0449)	846.4 (721.0–1007.0)	69.7 (42.7–102.0)	17.1 (-1.6–38.9)
14	Chronic kidney disease	2.2 (1.9–2.4)	0.0347 (0.0298–0.0402)	446.5 (385.5–515.3)	335.2 (281.4–401.3)	110.5 (85.8–140.9)
15	Age-related and other hearing loss	1.9 (1.5–2.5)	0.0312 (0.0218–0.0431)	419.2 (291.1–581.0)	86.5 (79.2–94.2)	-5.7 (-8.9–2.3)
16	Road injuries	1.9 (1.8–2.1)	0.0311 (0.0269–0.0362)	668.0 (580.8–765.0)	-20.6 (-28.8—11.3)	-50.2 (-55.5—44.2)
17	Headache disorders	1.9 (0.4–3.8)	0.0307 (0.00696–0.0646)	686.1 (139.2–1466.1)	41.6 (34.1–55.8)	-3.0 (-7.6–2.7)
18	Cirrhosis and other chronic liver diseases	1.8 (1.6–2.1)	0.0294 (0.0250–0.0352)	463.2 (394.2–553.7)	171.8 (128.3–226.8)	46.2 (22.5–74.7)
19	Anxiety disorders	1.7 (1.2–2.4)	0.0276 (0.0180–0.0409)	615.9 (395.7–908.3)	77.3 (36.5–124.6)	23.1 (-4.7–54.9)
20	Alcohol use disorders	1.5 (1.4–1.7)	0.0244 (0.0202–0.0297)	485.8 (398.4–592.7)	70.6 (49.1–98.4)	6.5 (-5.4–21.7)
21	Osteoarthritis	1.4 (0.8–2.6)	0.0228 (0.0111–0.0460)	299.6 (144.5–601.4)	121.0 (114.4–128.0)	7.0 (3.7–10.4)
22	Colon and rectum cancer	1.3 (1.1–1.5)	0.0209 (0.0172–0.0252)	278.1 (227.2–336.8)	34.1 (9.8–61.8)	-32.4 (-44.6—18.1)
23	Asthma	1.2 (0.9–1.5)	0.0191 (0.0131–0.0271)	427.4 (289.4–621.1)	38.4 (28.7–48.5)	-8.4 (-15.0—1.7)
24	Breast cancer	1.1 (0.9–1.4)	0.0182 (0.0145–0.0229)	256.9 (204.0–324.7)	23.2 (-1.7–52.3)	-38.5 (-51.2—23.6)
25	Endocrine, metabolic, blood, and immune disorders	1.1 (1.0–1.3)	0.0181 (0.0155–0.0213)	299.2 (256.6–352.9)	212.1 (166.7–269.4)	64.9 (44.4–90.0)

Table S4: Cause-specific DALYs by US state in 2021 and percentage change between 1990 and 2021, Pennsylvania						
Rank	Cause Name	2021 Percentage of all cause DALYs	2021 DALYs (millions)	2021 Age Standardised Rate (per 100 000)	Percentage change DALY count 1990 to 2021	Percentage change age-standardised DALY rate 1990 to 2021
	All causes	100.0 (100.0–100.0)	5.36 (4.69–6.12)	29553.4 (25666.0–33940.9)	23.5 (15.2–32.1)	1.5 (-5.1–7.9)
1	COVID-19	8.5 (7.4–9.8)	0.452 (0.440–0.477)	2300.7 (2218.6–2481.1)	--	--
2	Ischaemic heart disease	7.2 (6.2–8.2)	0.386 (0.325–0.446)	1528.0 (1290.8–1759.2)	-39.4 (-47.6–-30.8)	-57.2 (-63.2–-51.1)
3	Drug use disorders	7.1 (6.2–8.0)	0.380 (0.314–0.443)	3027.0 (2486.9–3516.1)	626.8 (543.4–741.4)	637.1 (554.0–756.1)
4	Low back pain	3.9 (3.2–4.7)	0.212 (0.155–0.272)	1280.5 (934.8–1657.8)	15.5 (8.3–24.2)	-1.3 (-7.7–5.8)
5	Diabetes mellitus	3.9 (3.4–4.5)	0.210 (0.167–0.263)	933.8 (739.1–1166.5)	85.2 (65.3–105.2)	38.1 (23.5–53.0)
6	Chronic obstructive pulmonary disease	3.6 (3.2–4.0)	0.194 (0.169–0.217)	745.0 (655.0–833.2)	50.1 (35.6–66.2)	6.6 (-3.7–17.9)
7	Other musculoskeletal disorders	3.3 (2.5–4.1)	0.175 (0.125–0.232)	1113.7 (798.7–1475.6)	71.2 (53.1–91.9)	52.7 (37.0–70.6)
8	Tracheal, bronchus, and lung cancer	3.1 (2.6–3.6)	0.167 (0.138–0.198)	667.3 (554.1–791.2)	-20.8 (-33.9–-6.2)	-46.6 (-55.6–-36.7)
9	Alzheimer's disease and other dementias	3.0 (1.4–6.4)	0.162 (0.0723–0.342)	543.0 (246.0–1143.2)	49.5 (37.2–63.9)	-3.5 (-10.8–5.3)
10	Stroke	3.0 (2.7–3.3)	0.162 (0.139–0.185)	646.4 (560.1–739.4)	-0.6 (-11.0–11.4)	-29.9 (-37.3–-21.4)
11	Depressive disorders	2.3 (1.7–3.2)	0.126 (0.0839–0.178)	968.5 (641.2–1382.2)	53.6 (24.3–88.6)	52.9 (23.4–85.8)
12	Chronic kidney disease	2.3 (2.0–2.6)	0.123 (0.106–0.139)	513.4 (442.9–581.8)	167.6 (136.4–203.6)	84.7 (64.0–108.7)
13	Falls	2.1 (1.8–2.4)	0.112 (0.0889–0.142)	478.4 (379.0–608.2)	75.9 (62.6–90.5)	17.3 (9.2–26.4)
14	Anxiety disorders	2.0 (1.4–2.8)	0.108 (0.0684–0.154)	798.4 (500.6–1137.4)	36.4 (6.3–70.4)	30.7 (2.5–62.8)
15	Age-related and other hearing loss	1.8 (1.3–2.3)	0.0950 (0.0664–0.131)	416.7 (290.3–575.9)	26.8 (22.2–32.0)	-5.3 (-8.6–-1.7)
16	Headache disorders	1.7 (0.4–3.4)	0.0916 (0.0210–0.190)	678.5 (137.4–1429.4)	2.0 (-3.4–10.9)	-3.6 (-8.5–1.9)
17	Osteoarthritis	1.5 (0.8–3.0)	0.0822 (0.0398–0.165)	352.9 (169.5–707.6)	42.6 (38.6–47.2)	3.1 (0.3–6.3)
18	Self-harm	1.5 (1.3–1.8)	0.0816 (0.0687–0.0946)	600.4 (506.2–694.8)	10.6 (-7.6–27.3)	4.7 (-12.1–20.2)
19	Road injuries	1.4 (1.3–1.5)	0.0761 (0.0656–0.0882)	534.6 (464.5–612.4)	-44.2 (-49.9–-37.8)	-51.5 (-56.9–-46.1)
20	Colon and rectum cancer	1.4 (1.1–1.6)	0.0733 (0.0601–0.0880)	319.2 (261.7–381.9)	-21.1 (-35.7–-4.0)	-39.9 (-51.1–-27.0)
21	Cirrhosis and other chronic liver diseases	1.2 (1.1–1.4)	0.0664 (0.0567–0.0772)	330.8 (282.9–385.3)	25.7 (7.4–46.8)	-6.2 (-19.9–9.7)
22	Breast cancer	1.1 (0.9–1.3)	0.0601 (0.0478–0.0734)	275.8 (218.7–339.3)	-30.6 (-43.8–-15.9)	-49.8 (-59.8–-38.2)
23	Neonatal disorders	1.1 (1.0–1.2)	0.0584 (0.0503–0.0668)	766.4 (674.7–860.9)	-42.2 (-48.5–-35.2)	-31.7 (-39.0–-23.6)
24	Asthma	1.0 (0.8–1.4)	0.0553 (0.0377–0.0790)	443.3 (299.3–645.4)	9.5 (2.7–17.2)	4.9 (-2.3–12.6)
25	Oral disorders	1.0 (0.7–1.4)	0.0538 (0.0334–0.0790)	276.6 (164.5–412.5)	21.4 (17.6–25.6)	-2.7 (-6.4–1.2)

Table S4: Cause-specific DALYs by US state in 2021 and percentage change between 1990 and 2021, Rhode Island						
Rank	Cause Name	2021 Percentage of all cause DALYs	2021 DALYs (millions)	2021 Age Standardised Rate (per 100 000)	Percentage change DALY count 1990 to 2021	Percentage change age-standardised DALY rate 1990 to 2021
	All causes	100.0 (100.0–100.0)	0.393 (0.338–0.454)	25354.0 (21654.8–29422.4)	15.6 (7.5–24.1)	-7.3 (-13.8–-1.1)
1	Ischaemic heart disease	7.5 (6.3–8.8)	0.0294 (0.0247–0.0347)	1360.2 (1140.1–1603.1)	-42.0 (-49.9–-32.9)	-60.5 (-66.1–-53.9)
2	COVID-19	6.9 (6.0–7.9)	0.0268 (0.0260–0.0285)	1613.2 (1545.7–1755.0)	--	--
3	Drug use disorders	6.1 (5.2–7.1)	0.0241 (0.0198–0.0285)	2131.0 (1737.7–2525.2)	436.2 (363.1–525.3)	432.3 (357.3–513.5)
4	Diabetes mellitus	4.1 (3.6–4.7)	0.0161 (0.0126–0.0203)	847.6 (659.8–1074.1)	100.9 (77.0–124.7)	44.0 (27.1–60.7)
5	Low back pain	4.1 (3.3–5.0)	0.0160 (0.0116–0.0209)	1110.4 (810.2–1448.5)	9.2 (1.8–17.8)	-10.7 (-16.5–-3.1)
6	Chronic obstructive pulmonary disease	3.6 (3.1–4.1)	0.0142 (0.0125–0.0160)	660.3 (583.8–741.7)	48.4 (33.3–65.2)	4.1 (-6.4–15.8)
7	Depressive disorders	3.3 (2.3–4.4)	0.0129 (0.00858–0.0182)	1126.2 (740.0–1588.8)	55.5 (27.9–89.8)	50.2 (23.5–83.2)
8	Other musculoskeletal disorders	3.2 (2.4–4.0)	0.0126 (0.00891–0.0169)	925.0 (657.3–1245.2)	67.2 (49.1–86.3)	42.6 (28.2–59.1)
9	Tracheal, bronchus, and lung cancer	3.2 (2.6–3.7)	0.0125 (0.0105–0.0148)	603.0 (509.8–711.0)	-24.4 (-35.4–-11.8)	-50.9 (-58.1–-42.5)
10	Alzheimer's disease and other dementias	3.1 (1.5–6.5)	0.0124 (0.00585–0.0265)	507.0 (242.9–1095.2)	40.1 (28.7–53.5)	-4.0 (-11.5–5.0)
11	Stroke	2.6 (2.3–2.9)	0.0103 (0.00874–0.0118)	499.1 (425.5–570.4)	-10.1 (-19.8–0.1)	-35.9 (-43.2–-28.8)
12	Falls	2.6 (2.3–3.1)	0.0102 (0.00809–0.0129)	524.8 (414.8–665.8)	67.9 (56.6–80.8)	13.2 (5.9–21.4)
13	Anxiety disorders	2.3 (1.6–3.3)	0.00920 (0.00598–0.0135)	778.3 (508.1–1147.5)	35.3 (6.7–69.4)	27.1 (0.4–59.4)
14	Chronic kidney disease	2.1 (1.8–2.3)	0.00808 (0.00693–0.00918)	403.8 (345.6–459.4)	147.2 (120.7–180.3)	71.5 (53.6–94.0)
15	Headache disorders	2.0 (0.5–4.1)	0.00807 (0.00183–0.0169)	687.8 (140.0–1463.0)	4.2 (-1.3–16.4)	-3.2 (-7.9–3.3)
16	Age-related and other hearing loss	2.0 (1.5–2.6)	0.00789 (0.00554–0.0109)	416.4 (292.5–575.0)	27.9 (22.7–33.8)	-5.3 (-8.6–-1.5)
17	Osteoarthritis	1.5 (0.8–2.8)	0.00605 (0.00297–0.0122)	308.0 (149.8–620.0)	51.6 (47.6–56.5)	5.6 (2.7–8.7)
18	Cirrhosis and other chronic liver diseases	1.4 (1.2–1.7)	0.00561 (0.00466–0.00667)	335.2 (280.7–397.9)	29.4 (7.0–55.6)	-6.6 (-23.2–12.5)
19	Alcohol use disorders	1.3 (1.2–1.5)	0.00527 (0.00424–0.00667)	409.5 (326.1–523.3)	2.0 (-9.3–18.1)	-10.1 (-18.7–2.9)
20	Colon and rectum cancer	1.3 (1.1–1.5)	0.00518 (0.00433–0.00612)	262.6 (219.8–310.6)	-28.7 (-39.4–-16.1)	-48.6 (-56.5–-39.2)
21	Self-harm	1.3 (1.1–1.5)	0.00508 (0.00421–0.00612)	414.8 (345.4–500.7)	-18.2 (-32.5–-0.6)	-25.1 (-38.0–-9.1)
22	Road injuries	1.2 (1.1–1.3)	0.00477 (0.00403–0.00563)	384.1 (328.7–451.5)	-48.5 (-54.4–-42.4)	-55.0 (-60.4–-49.1)
23	Asthma	1.1 (0.8–1.5)	0.00445 (0.00296–0.00639)	428.1 (283.6–634.2)	12.6 (4.4–21.5)	6.1 (-1.6–15.2)
24	Breast cancer	1.1 (0.9–1.3)	0.00438 (0.00351–0.00534)	236.6 (188.8–291.0)	-34.2 (-45.9–-20.4)	-54.7 (-63.1–-44.9)
25	Oral disorders	1.1 (0.7–1.6)	0.00437 (0.00258–0.00658)	267.0 (155.1–405.9)	18.0 (7.9–28.2)	-8.1 (-16.2–0.1)

Table S4: Cause-specific DALYs by US state in 2021 and percentage change between 1990 and 2021, South Carolina						
Rank	Cause Name	2021 Percentage of all cause DALYs	2021 DALYs (millions)	2021 Age Standardised Rate (per 100 000)	Percentage change DALY count 1990 to 2021	Percentage change age-standardised DALY rate 1990 to 2021
	All causes	100.0 (100.0–100.0)	2.31 (2.05–2.62)	33347.4 (29252.1–37921.5)	87.6 (72.8–103.0)	3.7 (-3.9–11.2)
1	COVID-19	10.4 (8.5–13.1)	0.239 (0.206–0.295)	3227.4 (2764.6–3981.4)	--	--
2	Ischaemic heart disease	6.5 (5.7–7.5)	0.151 (0.129–0.174)	1721.5 (1466.4–1984.0)	-1.4 (-15.2–12.8)	-54.5 (-61.2–-47.8)
3	Drug use disorders	4.8 (4.1–5.6)	0.112 (0.0916–0.132)	2210.1 (1806.4–2619.8)	732.5 (606.6–894.4)	541.4 (447.9–661.5)
4	Diabetes mellitus	4.1 (3.5–4.6)	0.0943 (0.0759–0.118)	1127.4 (906.4–1405.6)	214.0 (179.7–245.4)	51.1 (35.4–66.3)
5	Chronic obstructive pulmonary disease	3.9 (3.5–4.4)	0.0904 (0.0793–0.102)	944.8 (831.3–1060.8)	188.0 (153.7–223.7)	25.4 (10.5–41.2)
6	Low back pain	3.5 (2.7–4.3)	0.0813 (0.0589–0.106)	1234.7 (895.8–1605.3)	60.3 (48.7–73.1)	-5.0 (-11.1–1.9)
7	Stroke	3.5 (3.1–3.8)	0.0807 (0.0692–0.0920)	924.5 (795.2–1055.1)	42.6 (25.3–61.3)	-34.4 (-42.2–-25.7)
8	Tracheal, bronchus, and lung cancer	3.2 (2.7–3.7)	0.0741 (0.0616–0.0859)	795.2 (661.8–925.2)	33.8 (12.1–57.8)	-41.8 (-51.4–-31.6)
9	Other musculoskeletal disorders	2.7 (2.0–3.3)	0.0614 (0.0446–0.0817)	960.6 (698.5–1272.3)	134.9 (112.0–165.1)	43.4 (28.9–62.3)
10	Chronic kidney disease	2.6 (2.4–2.9)	0.0609 (0.0518–0.0687)	715.7 (612.2–806.3)	318.6 (267.0–379.0)	93.9 (70.4–120.3)
11	Road injuries	2.6 (2.4–2.8)	0.0593 (0.0522–0.0674)	1095.0 (968.0–1238.9)	-8.9 (-19.3–2.0)	-37.3 (-44.6–-30.0)
12	Depressive disorders	2.5 (1.8–3.5)	0.0582 (0.0395–0.0833)	1113.9 (761.4–1590.2)	115.8 (75.5–164.9)	57.8 (28.2–94.5)
13	Alzheimer's disease and other dementias	2.1 (1.0–4.7)	0.0494 (0.0229–0.108)	498.5 (230.7–1098.1)	147.2 (125.7–171.0)	-3.0 (-11.4–6.1)
14	Anxiety disorders	1.9 (1.3–2.7)	0.0439 (0.0288–0.0636)	815.5 (527.4–1190.9)	87.8 (46.2–134.1)	33.1 (5.0–65.8)
15	Falls	1.7 (1.5–2.0)	0.0389 (0.0316–0.0491)	463.3 (376.7–580.5)	151.7 (132.7–175.8)	17.2 (9.0–27.9)
16	Self-harm	1.7 (1.5–1.9)	0.0384 (0.0322–0.0451)	729.4 (615.8–850.8)	77.5 (47.6–108.1)	30.5 (9.2–53.1)
17	Cirrhosis and other chronic liver diseases	1.6 (1.4–1.8)	0.0367 (0.0306–0.0431)	495.2 (412.5–581.0)	120.8 (86.0–159.3)	16.1 (-2.0–36.1)
18	Headache disorders	1.6 (0.4–3.2)	0.0367 (0.00816–0.0765)	684.1 (137.5–1445.2)	36.1 (29.0–52.7)	-3.6 (-8.2–1.8)
19	Age-related and other hearing loss	1.5 (1.1–2.0)	0.0358 (0.0250–0.0494)	417.3 (291.6–577.8)	100.0 (92.1–109.4)	-5.8 (-9.1–-2.6)
20	Colon and rectum cancer	1.3 (1.1–1.5)	0.0305 (0.0256–0.0362)	361.3 (301.9–427.4)	64.2 (37.4–94.7)	-21.2 (-34.3–-6.4)
21	Interpersonal violence	1.2 (1.1–1.4)	0.0284 (0.0244–0.0329)	609.4 (523.9–701.0)	9.5 (-6.2–26.8)	-12.0 (-24.8–2.4)
22	Osteoarthritis	1.2 (0.6–2.2)	0.0270 (0.0131–0.0547)	304.1 (146.0–614.4)	132.1 (125.5–140.1)	5.6 (2.6–9.3)
23	Neonatal disorders	1.2 (1.0–1.3)	0.0268 (0.0233–0.0304)	830.1 (733.9–926.7)	-34.6 (-41.5–-26.4)	-41.3 (-47.4–-34.4)
24	Breast cancer	1.1 (0.9–1.4)	0.0261 (0.0211–0.0320)	323.6 (260.0–400.1)	33.6 (7.8–62.5)	-34.9 (-48.2–-20.8)
25	Endocrine, metabolic, blood, and immune disorders	1.1 (1.0–1.2)	0.0254 (0.0221–0.0292)	366.8 (319.9–424.6)	217.9 (175.3–270.9)	71.1 (50.5–96.5)

Table S4: Cause-specific DALYs by US state in 2021 and percentage change between 1990 and 2021, South Dakota						
**Rank**	**Cause Name**	**2021 Percentage of all cause DALYs**	**2021 DALYs (millions)**	**2021 Age Standardised Rate (per 100 000)**	**Percentage change DALY count 1990 to 2021**	**Percentage change age-standardised DALY rate 1990 to 2021**
	All causes	100.0 (100.0–100.0)	0.321 (0.282–0.361)	27268.6 (23754.5–30996.0)	42.0 (33.3–51.6)	1.0 (-5.0–7.5)
1	Ischaemic heart disease	7.7 (6.6–8.8)	0.0245 (0.0212–0.0281)	1557.6 (1351.9–1785.5)	-23.8 (-33.2–-12.5)	-50.2 (-56.7–-42.3)
2	COVID-19	6.9 (6.1–8.0)	0.0222 (0.0211–0.0244)	1756.8 (1645.3–2005.0)	--	--
3	Low back pain	4.2 (3.3–5.1)	0.0136 (0.00991–0.0179)	1272.6 (929.7–1676.1)	30.8 (23.3–39.9)	-5.4 (-10.9–1.0)
4	Other musculoskeletal disorders	3.9 (3.0–4.9)	0.0126 (0.00898–0.0167)	1272.6 (909.7–1694.4)	112.4 (92.5–135.0)	62.3 (46.4–80.6)
5	Chronic obstructive pulmonary disease	3.9 (3.4–4.4)	0.0126 (0.0112–0.0139)	753.7 (673.4–830.8)	82.8 (65.0–102.2)	13.5 (2.4–25.4)
6	Diabetes mellitus	3.9 (3.4–4.4)	0.0125 (0.00989–0.0156)	882.3 (704.1–1099.4)	152.3 (126.9–175.2)	63.4 (47.3–77.4)
7	Alzheimer's disease and other dementias	3.1 (1.5–6.5)	0.0100 (0.00475–0.0212)	508.2 (242.9–1080.2)	53.3 (42.2–66.1)	-1.9 (-9.1–6.6)
8	Tracheal, bronchus, and lung cancer	3.0 (2.5–3.4)	0.00970 (0.00829–0.0112)	624.9 (530.9–718.3)	13.2 (-3.0–31.7)	-34.0 (-43.3–-23.1)
9	Stroke	2.9 (2.6–3.2)	0.00934 (0.00815–0.0105)	599.5 (527.3–672.9)	7.5 (-2.8–18.6)	-27.8 (-34.8–-20.3)
10	Falls	2.7 (2.4–3.1)	0.00871 (0.00701–0.0110)	601.3 (478.5–755.0)	109.8 (95.8–125.6)	29.1 (20.8–38.7)
11	Road injuries	2.7 (2.5–2.9)	0.00868 (0.00764–0.00981)	969.7 (862.2–1084.7)	-19.5 (-26.9–-11.8)	-37.0 (-43.1–-30.8)
12	Drug use disorders	2.5 (2.1–2.9)	0.00805 (0.00643–0.00963)	989.1 (788.8–1188.5)	407.2 (338.0–498.1)	337.7 (278.4–415.3)
13	Self-harm	2.4 (2.1–2.8)	0.00784 (0.00674–0.00908)	919.6 (792.2–1065.3)	64.5 (40.1–91.6)	35.8 (15.4–58.3)
14	Chronic kidney disease	2.2 (1.9–2.4)	0.00698 (0.00606–0.00792)	461.4 (401.4–520.2)	233.8 (197.3–274.9)	113.0 (90.6–139.7)
15	Depressive disorders	2.2 (1.6–2.9)	0.00694 (0.00471–0.00992)	786.1 (529.1–1126.4)	64.7 (34.0–98.8)	35.0 (10.1–63.8)
16	Anxiety disorders	2.1 (1.4–3.0)	0.00675 (0.00423–0.00987)	743.0 (464.0–1091.3)	52.2 (18.4–92.1)	21.9 (-5.9–54.2)
17	Age-related and other hearing loss	1.9 (1.4–2.5)	0.00618 (0.00429–0.00851)	421.9 (294.2–579.4)	44.2 (38.2–50.0)	-5.4 (-8.6–-2.0)
18	Headache disorders	1.9 (0.4–3.8)	0.00611 (0.00133–0.0128)	681.6 (135.1–1436.6)	21.2 (14.7–34.1)	-3.6 (-8.4–2.2)
19	Cirrhosis and other chronic liver diseases	1.7 (1.5–2.0)	0.00559 (0.00488–0.00643)	495.1 (430.2–569.1)	124.7 (95.0–159.4)	54.1 (33.5–78.8)
20	Colon and rectum cancer	1.4 (1.2–1.6)	0.00438 (0.00372–0.00509)	302.4 (257.2–349.9)	3.5 (-10.8–19.4)	-32.1 (-41.6–-21.5)
21	Osteoarthritis	1.3 (0.7–2.6)	0.00431 (0.00208–0.00873)	289.6 (138.7–582.0)	75.1 (70.1–80.4)	8.4 (5.2–11.6)
22	Alcohol use disorders	1.3 (1.2–1.5)	0.00429 (0.00353–0.00513)	455.4 (372.2–553.1)	41.0 (25.5–61.4)	8.0 (-3.0–21.8)
23	Neonatal disorders	1.2 (1.0–1.4)	0.00383 (0.00325–0.00451)	642.7 (547.6–761.8)	-15.8 (-28.7–0.3)	-19.1 (-30.9–-3.3)
24	Asthma	1.2 (0.9–1.6)	0.00379 (0.00257–0.00542)	414.1 (282.7–601.5)	27.0 (17.4–36.7)	0.5 (-6.8–8.6)
25	Oral disorders	1.1 (0.7–1.5)	0.00347 (0.00207–0.00524)	277.3 (161.6–422.7)	38.5 (27.5–49.9)	-5.1 (-13.4–3.8)

Table S4: Cause-specific DALYs by US state in 2021 and percentage change between 1990 and 2021, Tennessee

Rank	Cause Name	2021 Percentage of all cause DALYs	2021 DALYs (millions)	2021 Age Standardised Rate (per 100 000)	Percentage change DALY count 1990 to 2021	Percentage change age-standardised DALY rate 1990 to 2021
	All causes	100.0 (100.0–100.0)	3.19 (2.83–3.60)	34629.1 (30610.7–39153.6)	81.6 (68.1–96.1)	11.6 (3.9–19.3)
1	COVID-19	10.3 (8.7–12.5)	0.329 (0.298–0.393)	3350.2 (3020.9–4007.1)	--	--
2	Ischaemic heart disease	8.0 (6.9–9.1)	0.255 (0.220–0.297)	2191.3 (1875.0–2550.8)	1.0 (-13.1–16.8)	-45.2 (-53.1—36.2)
3	Drug use disorders	6.3 (5.3–7.3)	0.200 (0.162–0.236)	2905.5 (2342.7–3409.4)	965.6 (788.5–1181.3)	731.9 (599.2–899.2)
4	Chronic obstructive pulmonary disease	4.2 (3.7–4.7)	0.134 (0.119–0.148)	1076.5 (961.2–1196.6)	156.3 (127.8–187.0)	32.7 (18.4–48.8)
5	Diabetes mellitus	3.8 (3.3–4.3)	0.121 (0.0956–0.148)	1100.3 (874.8–1343.9)	202.4 (172.0–228.7)	68.2 (52.2–82.7)
6	Tracheal, bronchus, and lung cancer	3.4 (2.9–3.9)	0.110 (0.0940–0.129)	906.1 (779.4–1068.4)	19.3 (1.1–40.4)	-40.3 (-49.2—29.3)
7	Low back pain	3.4 (2.6–4.2)	0.108 (0.0781–0.141)	1245.3 (896.3–1611.8)	44.4 (34.5–55.7)	-6.4 (-12.3–1.0)
8	Stroke	3.2 (2.9–3.5)	0.102 (0.0894–0.115)	890.1 (780.7–1004.1)	33.4 (20.3–48.3)	-26.2 (-33.5—17.6)
9	Depressive disorders	2.5 (1.7–3.4)	0.0796 (0.0528–0.113)	1127.8 (738.4–1600.9)	109.2 (69.1–155.3)	59.0 (28.7–93.6)
10	Other musculoskeletal disorders	2.4 (1.8–3.0)	0.0771 (0.0555–0.102)	927.0 (675.6–1229.9)	110.8 (89.8–136.2)	41.5 (28.2–58.2)
11	Chronic kidney disease	2.2 (2.0–2.4)	0.0710 (0.0618–0.0800)	629.0 (545.9–709.1)	297.9 (249.4–351.2)	113.4 (87.9–142.1)
12	Road injuries	2.2 (2.0–2.4)	0.0696 (0.0607–0.0787)	946.6 (830.0–1066.3)	-17.2 (-26.1—7.2)	-41.9 (-48.5—34.8)
13	Alzheimer's disease and other dementias	2.0 (1.0–4.4)	0.0647 (0.0305–0.142)	496.9 (233.9–1090.6)	89.1 (73.3–105.7)	-3.5 (-11.5–5.0)
14	Anxiety disorders	1.8 (1.3–2.6)	0.0586 (0.0386–0.0875)	805.7 (516.2–1203.4)	77.3 (40.0–122.5)	31.3 (3.9–65.5)
15	Falls	1.8 (1.5–2.1)	0.0573 (0.0459–0.0729)	515.9 (413.5–652.2)	132.9 (113.8–153.4)	23.4 (13.8–34.2)
16	Self-harm	1.8 (1.5–2.0)	0.0562 (0.0481–0.0656)	773.5 (664.4–904.5)	71.0 (45.9–102.5)	27.1 (8.3–50.0)
17	Cirrhosis and other chronic liver diseases	1.7 (1.5–1.9)	0.0529 (0.0448–0.0617)	537.7 (454.5–627.6)	159.5 (119.8–202.0)	51.9 (28.7–77.1)
18	Headache disorders	1.5 (0.3–3.1)	0.0497 (0.0109–0.105)	685.4 (136.0–1450.5)	31.2 (24.3–47.0)	-3.6 (-8.4–2.4)
19	Age-related and other hearing loss	1.5 (1.1–1.9)	0.0469 (0.0328–0.0644)	418.2 (294.2–578.2)	70.4 (63.7–77.2)	-5.7 (-9.1—2.3)
20	Colon and rectum cancer	1.3 (1.1–1.6)	0.0429 (0.0359–0.0512)	385.1 (322.5–459.4)	47.2 (21.0–78.8)	-17.1 (-31.6–0.8)
21	Hypertensive heart disease	1.3 (1.1–1.5)	0.0425 (0.0351–0.0498)	397.6 (330.5–465.3)	236.1 (179.6–294.4)	94.0 (60.3–128.2)
22	Osteoarthritis	1.2 (0.6–2.2)	0.0377 (0.0183–0.0765)	324.7 (156.6–658.0)	99.3 (93.2–105.8)	5.7 (2.4–8.9)
23	Interpersonal violence	1.1 (1.0–1.3)	0.0363 (0.0316–0.0419)	572.5 (498.7–659.8)	1.5 (-12.4–17.5)	-17.7 (-28.9—5.1)
24	Neonatal disorders	1.1 (1.0–1.3)	0.0363 (0.0316–0.0416)	798.2 (703.1–904.7)	-19.8 (-28.8—10.0)	-32.1 (-39.8—23.7)
25	Breast cancer	1.1 (0.9–1.3)	0.0338 (0.0274–0.0409)	312.4 (252.2–381.2)	19.7 (-2.6–47.8)	-34.7 (-47.4—18.8)

Table S4: Cause-specific DALYs by US state in 2021 and percentage change between 1990 and 2021, Texas

Rank	Cause Name	2021 Percentage of all cause DALYs	2021 DALYs (millions)	2021 Age Standardised Rate (per 100 000)	Percentage change DALY count 1990 to 2021	Percentage change age-standardised DALY rate 1990 to 2021
	All causes	100.0 (100.0–100.0)	10.4 (9.16–11.8)	29183.9 (25587.5–32983.5)	98.9 (86.2–110.9)	-0.2 (-6.1–5.5)
1	COVID-19	11.3 (9.7–13.5)	1.18 (1.10–1.35)	3172.2 (2933.8–3623.4)	--	--
2	Ischaemic heart disease	6.7 (5.8–7.7)	0.703 (0.612–0.808)	1674.8 (1459.8–1924.4)	13.3 (-1.1–29.1)	-50.9 (-57.3—44.1)
3	Diabetes mellitus	3.9 (3.4–4.5)	0.409 (0.322–0.510)	1008.4 (790.2–1256.3)	220.5 (182.5–256.8)	42.1 (25.6–57.7)
4	Low back pain	3.8 (3.0–4.5)	0.395 (0.290–0.514)	1127.3 (828.0–1474.7)	75.0 (62.3–87.1)	-9.1 (-15.4—2.4)
5	Drug use disorders	3.7 (3.1–4.3)	0.382 (0.308–0.455)	1260.8 (1012.7–1504.7)	584.4 (503.8–686.7)	332.4 (281.3–396.2)
6	Chronic obstructive pulmonary disease	3.2 (2.8–3.6)	0.334 (0.302–0.368)	768.5 (695.1–845.7)	143.2 (118.6–167.7)	3.4 (-6.8–13.9)
7	Stroke	3.1 (2.7–3.4)	0.319 (0.280–0.357)	774.8 (682.3–869.6)	68.1 (50.7–86.4)	-24.9 (-32.6—16.9)
8	Other musculoskeletal disorders	2.9 (2.3–3.7)	0.304 (0.218–0.405)	886.3 (635.8–1179.1)	168.8 (137.8–203.2)	41.3 (25.8–58.7)
9	Depressive disorders	2.8 (2.1–3.8)	0.297 (0.202–0.426)	974.9 (665.9–1391.5)	154.2 (110.6–207.0)	52.8 (26.3–83.8)
10	Road injuries	2.5 (2.3–2.7)	0.259 (0.227–0.295)	830.8 (729.6–937.2)	5.1 (-5.2–16.0)	-39.7 (-45.5—33.5)
11	Chronic kidney disease	2.4 (2.2–2.7)	0.254 (0.224–0.289)	624.9 (551.0–708.3)	390.0 (336.8–453.6)	118.4 (95.5–146.1)
12	Anxiety disorders	2.4 (1.7–3.4)	0.252 (0.169–0.370)	813.1 (536.6–1203.3)	126.2 (74.7–184.3)	32.9 (2.7–67.3)
13	Tracheal, bronchus, and lung cancer	2.3 (2.0–2.6)	0.240 (0.206–0.276)	551.0 (474.5–633.6)	3.5 (-11.8–19.7)	-57.9 (-64.0—51.1)
14	Alzheimer's disease and other dementias	2.1 (1.0–4.3)	0.215 (0.100–0.455)	493.8 (231.0–1043.6)	125.0 (107.4–144.1)	-3.9 (-11.4–4.2)
15	Headache disorders	2.0 (0.4–4.2)	0.214 (0.0449–0.454)	687.1 (135.5–1454.2)	65.6 (56.9–82.2)	-3.0 (-7.8–2.4)
16	Self-harm	1.9 (1.6–2.1)	0.195 (0.168–0.225)	632.5 (547.3–728.5)	75.0 (50.9–102.0)	4.7 (-9.4–20.8)
17	Cirrhosis and other chronic liver diseases	1.8 (1.6–2.0)	0.186 (0.160–0.217)	478.9 (411.1–557.6)	158.5 (120.0–202.0)	17.6 (0.1–37.3)
18	Falls	1.7 (1.5–2.0)	0.176 (0.139–0.224)	439.3 (347.3–557.0)	120.9 (104.5–138.2)	0.3 (-6.2–7.7)
19	Age-related and other hearing loss	1.6 (1.2–2.1)	0.170 (0.119–0.235)	420.4 (294.0–586.1)	107.4 (99.2–115.4)	-5.7 (-9.1—2.2)
20	Neonatal disorders	1.4 (1.2–1.6)	0.146 (0.127–0.166)	709.9 (626.3–791.6)	2.2 (-8.7–13.7)	-22.0 (-30.0—13.2)
21	Colon and rectum cancer	1.3 (1.2–1.5)	0.140 (0.119–0.163)	342.4 (292.0–397.5)	80.5 (53.5–113.7)	-20.4 (-32.3—5.6)
22	Osteoarthritis	1.2 (0.6–2.3)	0.128 (0.0621–0.258)	305.6 (147.3–614.2)	150.0 (141.8–158.4)	6.3 (2.7–9.8)
23	Hypertensive heart disease	1.1 (1.0–1.3)	0.118 (0.0987–0.141)	294.9 (247.1–352.5)	240.0 (184.1–306.3)	52.8 (27.6–82.8)
24	Asthma	1.1 (0.8–1.5)	0.118 (0.0786–0.172)	412.3 (275.9–605.9)	63.7 (52.6–76.2)	-3.1 (-9.7–4.3)
25	Breast cancer	1.1 (0.9–1.3)	0.112 (0.0902–0.136)	281.3 (225.6–340.8)	43.8 (15.8–74.1)	-36.0 (-48.8—22.2)

Table S4: Cause-specific DALYs by US state in 2021 and percentage change between 1990 and 2021, Utah						
Rank	Cause Name	2021 Percentage of all cause DALYs	2021 DALYs (millions)	2021 Age Standardised Rate (per 100 000)	Percentage change DALY count 1990 to 2021	Percentage change age-standardised DALY rate 1990 to 2021
	All causes	100.0 (100.0–100.0)	0.978 (0.844–1.12)	26650.1 (22908.5–30615.3)	130.1 (115.3–144.8)	4.4 (-2.1–10.8)
1	COVID-19	7.7 (6.4–9.5)	0.0745 (0.0663–0.0897)	1978.8 (1753.1–2382.5)	--	--
2	Drug use disorders	6.1 (5.3–7.0)	0.0600 (0.0491–0.0705)	1772.2 (1452.3–2084.0)	1181.5 (1000.7–1410.6)	573.7 (480.5–687.5)
3	Low back pain	4.6 (3.7–5.5)	0.0450 (0.0329–0.0585)	1233.1 (899.9–1606.0)	107.3 (93.0–123.2)	-6.1 (-12.2–0.7)
4	Ischaemic heart disease	4.5 (3.8–5.3)	0.0439 (0.0370–0.0508)	1079.0 (908.1–1246.1)	12.2 (-3.8–28.5)	-56.6 (-62.9–-50.3)
5	Depressive disorders	4.5 (3.2–5.9)	0.0439 (0.0285–0.0625)	1271.9 (823.1–1809.7)	207.7 (152.1–269.3)	55.8 (27.9–86.9)
6	Diabetes mellitus	3.8 (3.4–4.3)	0.0376 (0.0300–0.0468)	947.1 (755.3–1171.5)	242.5 (204.0–276.5)	36.2 (21.0–49.9)
7	Other musculoskeletal disorders	3.6 (2.8–4.5)	0.0352 (0.0257–0.0468)	971.7 (708.4–1288.7)	216.0 (183.9–251.6)	43.9 (29.3–60.5)
8	Self-harm	3.2 (2.7–3.8)	0.0314 (0.0266–0.0365)	907.0 (770.6–1055.2)	116.0 (82.7–153.5)	9.3 (-7.7–28.1)
9	Chronic obstructive pulmonary disease	2.8 (2.4–3.2)	0.0272 (0.0241–0.0305)	650.7 (576.7–727.8)	168.1 (141.0–198.6)	3.5 (-7.0–15.4)
10	Anxiety disorders	2.7 (1.9–3.9)	0.0266 (0.0168–0.0389)	769.6 (484.3–1127.3)	151.6 (93.8–217.5)	25.8 (-3.1–58.5)
11	Stroke	2.6 (2.3–2.9)	0.0255 (0.0226–0.0289)	631.4 (559.7–715.0)	77.9 (61.0–97.6)	-29.2 (-35.9–-21.3)
12	Headache disorders	2.4 (0.5–4.8)	0.0236 (0.00489–0.0497)	683.2 (137.5–1440.0)	92.2 (82.6–114.7)	-3.5 (-8.2–2.4)
13	Falls	2.2 (1.9–2.5)	0.0215 (0.0173–0.0273)	544.3 (437.2–689.3)	194.5 (172.8–219.2)	22.2 (13.8–31.9)
14	Alzheimer's disease and other dementias	2.1 (1.0–4.4)	0.0204 (0.00938–0.0443)	489.2 (224.1–1056.4)	159.0 (139.3–180.0)	-2.2 (-9.7–5.5)
15	Chronic kidney disease	2.0 (1.8–2.3)	0.0198 (0.0173–0.0225)	495.8 (436.2–562.9)	438.6 (381.8–507.2)	118.3 (95.9–146.4)
16	Road injuries	1.8 (1.7–2.0)	0.0181 (0.0159–0.0208)	518.5 (457.7–593.8)	-11.0 (-20.0–-1.6)	-54.9 (-59.2–-49.9)
17	Age-related and other hearing loss	1.7 (1.3–2.2)	0.0167 (0.0117–0.0230)	419.8 (294.6–577.2)	131.6 (123.0–141.1)	-5.8 (-8.8–-2.3)
18	Neonatal disorders	1.7 (1.4–2.0)	0.0164 (0.0140–0.0192)	639.0 (545.0–743.2)	50.9 (28.6–75.8)	5.4 (-10.6–24.3)
19	Tracheal, bronchus, and lung cancer	1.4 (1.2–1.6)	0.0134 (0.0114–0.0156)	324.9 (276.9–378.6)	49.9 (26.5–78.5)	-44.0 (-52.9–-33.2)
20	Asthma	1.3 (1.0–1.8)	0.0132 (0.00894–0.0188)	390.3 (264.8–561.7)	78.1 (62.6–94.0)	-7.1 (-13.5–-0.6)
21	Osteoarthritis	1.3 (0.7–2.5)	0.0129 (0.00625–0.0264)	322.5 (155.9–655.9)	175.9 (167.8–185.9)	5.9 (2.7–9.6)
22	Alcohol use disorders	1.3 (1.1–1.4)	0.0123 (0.0101–0.0153)	350.7 (287.7–438.4)	108.6 (84.4–139.8)	0.7 (-9.5–14.4)
23	Cirrhosis and other chronic liver diseases	1.3 (1.1–1.5)	0.0123 (0.0105–0.0142)	326.3 (278.5–376.9)	164.6 (123.4–209.3)	9.4 (-7.7–28.1)
24	Congenital birth defects	1.1 (1.0–1.3)	0.0107 (0.00931–0.0125)	411.8 (354.7–479.9)	-0.5 (-13.1–14.0)	-31.2 (-40.9–-20.4)
25	Breast cancer	1.0 (0.9–1.3)	0.0102 (0.00873–0.0121)	261.6 (223.1–309.7)	71.5 (45.4–102.7)	-32.4 (-43.0–-19.9)

Table S4: Cause-specific DALYs by US state in 2021 and percentage change between 1990 and 2021, Vermont

Rank	Cause Name	2021 Percentage of all cause DALYs	2021 DALYs (millions)	2021 Age Standardised Rate (per 100 000)	Percentage change DALY count 1990 to 2021	Percentage change age-standardised DALY rate 1990 to 2021
	All causes	100.0 (100.0–100.0)	0.240 (0.210–0.272)	25536.7 (22240.1–29405.3)	40.5 (32.3–50.4)	-4.2 (-9.6–1.8)
1	Ischaemic heart disease	7.4 (6.3–8.6)	0.0178 (0.0152–0.0202)	1338.9 (1156.6–1526.0)	-15.6 (-25.2—4.2)	-55.5 (-60.8—49.1)
2	COVID-19	5.1 (3.3–6.9)	0.0122 (0.00757–0.0163)	1190.7 (750.0–1580.0)	--	--
3	Drug use disorders	4.5 (3.9–5.1)	0.0107 (0.00887–0.0125)	1791.6 (1481.8–2096.2)	436.1 (366.6–539.5)	459.6 (386.8–570.3)
4	Chronic obstructive pulmonary disease	4.2 (3.6–4.7)	0.00997 (0.00891–0.0110)	716.6 (641.7–791.9)	83.0 (67.6–99.3)	-7.0 (-14.7–1.4)
5	Low back pain	4.0 (3.2–4.8)	0.00958 (0.00694–0.0124)	1113.5 (807.8–1456.5)	20.9 (11.9–31.2)	-10.5 (-16.1—3.3)
6	Diabetes mellitus	4.0 (3.4–4.5)	0.00952 (0.00749–0.0120)	797.3 (627.0–1003.3)	137.9 (110.4–161.9)	32.8 (17.7–45.8)
7	Tracheal, bronchus, and lung cancer	3.3 (2.8–3.9)	0.00790 (0.00680–0.00923)	602.5 (519.3–707.0)	3.0 (-12.2–20.3)	-48.5 (-56.1—39.8)
8	Falls	3.2 (2.8–3.7)	0.00763 (0.00612–0.00966)	628.8 (502.9–791.8)	122.7 (106.1–138.8)	22.4 (14.9–30.6)
9	Alzheimer's disease and other dementias	3.2 (1.5–6.6)	0.00763 (0.00357–0.0164)	502.1 (236.4–1074.2)	89.9 (76.9–104.5)	-3.6 (-10.3–3.7)
10	Other musculoskeletal disorders	3.0 (2.3–3.8)	0.00729 (0.00521–0.00974)	917.4 (659.0–1224.8)	72.7 (54.6–95.1)	36.0 (22.1–52.2)
11	Depressive disorders	3.0 (2.1–3.9)	0.00710 (0.00475–0.0101)	1105.4 (723.0–1583.8)	62.9 (34.2–97.0)	56.7 (29.8–88.2)
12	Stroke	2.8 (2.5–3.1)	0.00672 (0.00585–0.00752)	520.4 (457.1–582.1)	15.3 (5.5–27.7)	-36.4 (-41.9—29.5)
13	Anxiety disorders	2.3 (1.5–3.2)	0.00552 (0.00340–0.00802)	825.1 (513.0–1220.0)	46.1 (12.0–82.6)	34.9 (4.4–67.8)
14	Age-related and other hearing loss	2.1 (1.6–2.7)	0.00507 (0.00353–0.00703)	418.6 (291.4–578.4)	68.4 (61.0–76.5)	-5.3 (-8.6—1.8)
15	Self-harm	2.1 (1.8–2.4)	0.00498 (0.00433–0.00577)	761.8 (666.2–880.2)	21.6 (4.8–40.8)	14.9 (-0.6–32.1)
16	Chronic kidney disease	2.0 (1.7–2.2)	0.00468 (0.00411–0.00530)	374.6 (329.9–422.2)	221.7 (187.3–263.8)	77.8 (60.6–99.4)
17	Headache disorders	1.9 (0.4–3.8)	0.00457 (0.00105–0.00951)	686.2 (137.2–1453.1)	4.8 (-1.5–22.7)	-3.3 (-7.8–2.7)
18	Road injuries	1.8 (1.7–2.0)	0.00439 (0.00383–0.00501)	648.8 (574.3–733.5)	-36.1 (-42.1—29.9)	-44.1 (-49.4—38.1)
19	Osteoarthritis	1.6 (0.8–2.9)	0.00379 (0.00184–0.00761)	300.4 (144.4–600.3)	102.9 (96.3–110.3)	6.6 (3.5–9.9)
20	Colon and rectum cancer	1.4 (1.2–1.7)	0.00344 (0.00297–0.00404)	279.7 (242.3–327.9)	8.0 (-8.1–27.6)	-40.4 (-49.4—29.9)
21	Cirrhosis and other chronic liver diseases	1.4 (1.2–1.5)	0.00324 (0.00285–0.00372)	331.2 (291.7–382.0)	61.8 (41.4–89.7)	4.3 (-9.1–22.3)
22	Alcohol use disorders	1.2 (1.1–1.4)	0.00298 (0.00246–0.00363)	406.5 (334.0–503.8)	20.1 (5.9–38.8)	3.1 (-6.7–16.4)
23	Endocrine, metabolic, blood, and immune disorders	1.2 (1.1–1.4)	0.00297 (0.00259–0.00342)	319.1 (281.2–371.2)	152.0 (119.5–186.3)	66.3 (48.9–86.5)
24	Oral disorders	1.2 (0.8–1.6)	0.00288 (0.00175–0.00426)	282.4 (166.0–427.8)	52.0 (44.8–58.9)	-3.3 (-7.4–0.9)
25	Breast cancer	1.1 (1.0–1.4)	0.00275 (0.00234–0.00326)	246.1 (208.3–292.3)	-4.7 (-18.6–11.4)	-44.2 (-52.6—34.3)

Table S4: Cause-specific DALYs by US state in 2021 and percentage change between 1990 and 2021, Virginia

Rank	Cause Name	2021 Percentage of all cause DALYs	2021 DALYs (millions)	2021 Age Standardised Rate (per 100 000)	Percentage change DALY count 1990 to 2021	Percentage change age-standardised DALY rate 1990 to 2021
	All causes	100.0 (100.0–100.0)	3.17 (2.78–3.60)	27747.8 (24205.0–31727.8)	61.6 (49.3–74.6)	-4.2 (-11.0–2.6)
1	COVID-19	8.1 (6.7–10.1)	0.256 (0.233–0.312)	2103.4 (1905.9–2560.1)	--	--
2	Ischaemic heart disease	6.5 (5.5–7.4)	0.204 (0.173–0.237)	1413.5 (1188.9–1641.0)	-12.5 (-26.6–1.4)	-57.4 (-64.5—50.6)
3	Diabetes mellitus	4.4 (3.8–5.0)	0.139 (0.110–0.175)	1023.2 (809.0–1275.5)	233.9 (199.4–263.6)	71.9 (54.1–87.2)
4	Drug use disorders	4.4 (3.7–5.0)	0.138 (0.113–0.163)	1582.8 (1288.5–1864.0)	570.2 (471.8–692.0)	453.6 (373.2–552.6)
5	Low back pain	3.9 (3.1–4.7)	0.125 (0.0914–0.163)	1145.5 (833.9–1498.6)	41.4 (31.4–53.2)	-9.4 (-15.1—2.7)
6	Chronic obstructive pulmonary disease	3.6 (3.1–4.0)	0.114 (0.0998–0.129)	750.9 (659.6–848.3)	115.0 (91.7–142.0)	1.6 (-9.5–14.5)
7	Stroke	3.3 (2.9–3.6)	0.104 (0.0890–0.118)	723.4 (624.0–820.9)	35.7 (18.9–52.2)	-33.3 (-41.4—25.1)
8	Other musculoskeletal disorders	3.0 (2.3–3.8)	0.0963 (0.0692–0.128)	916.4 (660.7–1212.8)	113.7 (91.5–143.2)	42.0 (26.6–61.7)
9	Tracheal, bronchus, and lung cancer	3.0 (2.5–3.6)	0.0962 (0.0808–0.113)	651.0 (545.7–767.1)	0.4 (-16.0–18.6)	-52.6 (-60.4—44.0)
10	Depressive disorders	2.9 (2.1–4.0)	0.0935 (0.0618–0.134)	1059.8 (699.6–1532.9)	99.8 (61.9–142.4)	56.5 (26.9–88.8)
11	Chronic kidney disease	2.6 (2.3–2.8)	0.0812 (0.0697–0.0924)	581.8 (502.0–663.8)	278.9 (232.8–333.5)	88.1 (66.2–113.3)
12	Alzheimer's disease and other dementias	2.5 (1.2–5.3)	0.0801 (0.0371–0.168)	499.5 (233.3–1047.3)	121.7 (103.1–144.1)	-3.7 (-11.2–5.5)
13	Anxiety disorders	2.3 (1.6–3.3)	0.0737 (0.0462–0.108)	803.8 (505.0–1184.7)	74.5 (34.2–118.1)	31.4 (2.2–64.3)
14	Falls	2.0 (1.7–2.3)	0.0639 (0.0511–0.0806)	466.9 (370.8–586.5)	128.7 (111.9–146.5)	16.2 (8.9–24.7)
15	Headache disorders	2.0 (0.4–3.9)	0.0629 (0.0142–0.132)	686.7 (139.9–1459.6)	29.2 (22.1–43.9)	-3.0 (-7.7–2.1)
16	Road injuries	1.8 (1.7–2.0)	0.0578 (0.0496–0.0662)	613.0 (526.2–698.4)	-25.0 (-33.3—16.0)	-47.5 (-53.3—41.2)
17	Age-related and other hearing loss	1.8 (1.4–2.3)	0.0568 (0.0397–0.0779)	417.4 (291.6–572.8)	82.2 (75.4–89.4)	-5.6 (-8.7—2.0)
18	Self-harm	1.7 (1.5–2.0)	0.0551 (0.0462–0.0647)	607.6 (510.0–712.5)	38.8 (15.4–65.0)	7.3 (-10.2–27.2)
19	Cirrhosis and other chronic liver diseases	1.4 (1.2–1.7)	0.0456 (0.0381–0.0534)	361.7 (302.8–422.9)	96.7 (63.0–131.6)	8.7 (-10.1–28.3)
20	Osteoarthritis	1.4 (0.7–2.7)	0.0443 (0.0212–0.0888)	313.3 (149.5–628.7)	113.5 (106.8–121.1)	5.6 (2.5–9.4)
21	Colon and rectum cancer	1.4 (1.2–1.6)	0.0441 (0.0368–0.0523)	318.4 (265.9–378.0)	28.9 (6.0–52.5)	-34.4 (-46.4—22.0)
22	Neonatal disorders	1.3 (1.1–1.4)	0.0396 (0.0346–0.0455)	738.8 (657.4–837.6)	-35.9 (-42.6—28.6)	-38.9 (-44.8—31.5)
23	Breast cancer	1.2 (1.0–1.5)	0.0387 (0.0314–0.0471)	290.9 (234.8–357.3)	7.1 (-14.3–31.2)	-43.9 (-55.5—31.4)
24	Asthma	1.2 (0.9–1.6)	0.0378 (0.0258–0.0541)	443.5 (297.5–649.3)	38.8 (29.7–47.6)	-1.0 (-7.9–6.2)
25	Alcohol use disorders	1.1 (1.0–1.3)	0.0345 (0.0279–0.0425)	347.2 (279.3–434.1)	29.6 (15.6–46.9)	-6.9 (-15.7–3.9)

Table S4: Cause-specific DALYs by US state in 2021 and percentage change between 1990 and 2021, Washington

Rank	Cause Name	2021 Percentage of all cause DALYs	2021 DALYs (millions)	2021 Age Standardised Rate (per 100 000)	Percentage change DALY count 1990 to 2021	Percentage change age-standardised DALY rate 1990 to 2021
	All causes	100.0 (100.0–100.0)	2.64 (2.26–3.03)	25589.8 (21721.3–29545.1)	78.5 (64.6–91.3)	-5.3 (-12.1–1.1)
1	COVID-19	6.0 (4.8–7.9)	0.157 (0.139–0.207)	1422.3 (1258.9–1876.4)	--	--
2	Ischaemic heart disease	5.8 (4.9–6.7)	0.152 (0.128–0.177)	1125.5 (949.4–1306.4)	-7.9 (-21.9–6.8)	-59.6 (-65.8—53.0)
3	Drug use disorders	5.2 (4.4–6.0)	0.137 (0.110–0.164)	1747.9 (1408.5–2101.0)	556.1 (464.2–684.8)	352.4 (289.5–441.4)
4	Low back pain	4.2 (3.3–5.1)	0.112 (0.0802–0.145)	1136.6 (824.5–1486.8)	62.6 (51.8–74.1)	-9.3 (-14.6—3.1)
5	Other musculoskeletal disorders	4.1 (3.2–5.2)	0.110 (0.0784–0.145)	1196.5 (853.3–1584.4)	175.9 (150.0–205.8)	62.4 (46.6–79.9)
6	Diabetes mellitus	3.8 (3.4–4.4)	0.101 (0.0798–0.127)	817.2 (641.6–1022.0)	223.8 (187.2–257.7)	48.8 (32.2–64.2)
7	Chronic obstructive pulmonary disease	3.4 (2.9–3.8)	0.0894 (0.0791–0.0998)	637.2 (565.6–710.4)	92.0 (69.9–114.3)	-17.6 (-27.1—7.9)
8	Depressive disorders	3.2 (2.2–4.4)	0.0852 (0.0544–0.122)	1076.1 (679.9–1551.6)	120.6 (77.9–170.1)	47.5 (18.9–79.8)
9	Stroke	2.9 (2.5–3.3)	0.0769 (0.0652–0.0875)	585.6 (500.1–666.9)	43.4 (26.7–59.3)	-34.7 (-42.4—27.6)
10	Tracheal, bronchus, and lung cancer	2.8 (2.3–3.3)	0.0729 (0.0608–0.0858)	533.5 (444.2–627.4)	10.0 (-8.3–30.0)	-54.5 (-62.4—46.2)
11	Alzheimer's disease and other dementias	2.8 (1.3–5.7)	0.0728 (0.0340–0.160)	499.0 (233.5–1093.4)	127.1 (107.2–147.7)	-3.1 (-11.5–5.3)
12	Falls	2.5 (2.1–2.9)	0.0659 (0.0518–0.0841)	526.8 (412.7–668.7)	143.8 (127.1–165.2)	10.0 (2.8–18.8)
13	Anxiety disorders	2.4 (1.7–3.3)	0.0638 (0.0407–0.0920)	773.7 (483.3–1120.3)	97.3 (50.5–151.0)	27.5 (-2.5–62.5)
14	Self-harm	2.2 (1.8–2.6)	0.0574 (0.0484–0.0671)	707.9 (598.0–825.9)	59.3 (32.5–86.9)	5.3 (-12.2–23.6)
15	Headache disorders	2.1 (0.5–4.2)	0.0559 (0.0126–0.117)	682.9 (137.5–1447.0)	50.1 (41.8–67.7)	-3.0 (-7.8–3.2)
16	Age-related and other hearing loss	2.0 (1.5–2.5)	0.0523 (0.0366–0.0721)	419.9 (293.7–575.4)	102.6 (94.8–111.0)	-5.5 (-9.0—1.9)
17	Chronic kidney disease	1.9 (1.7–2.2)	0.0513 (0.0445–0.0582)	400.6 (346.6–453.9)	332.0 (279.1–386.0)	95.9 (72.9–119.0)
18	Cirrhosis and other chronic liver diseases	1.7 (1.4–2.0)	0.0447 (0.0370–0.0523)	401.5 (332.0–469.4)	164.6 (119.1–211.6)	30.9 (8.7–54.5)
19	Road injuries	1.7 (1.5–1.8)	0.0444 (0.0379–0.0512)	524.9 (450.6–601.1)	-28.8 (-37.1—21.2)	-57.3 (-62.2—52.6)
20	Osteoarthritis	1.5 (0.8–2.8)	0.0394 (0.0192–0.0793)	304.4 (147.4–610.2)	142.7 (135.7–150.4)	6.8 (3.7–10.3)
21	Colon and rectum cancer	1.3 (1.1–1.5)	0.0329 (0.0273–0.0396)	259.5 (215.4–311.1)	45.4 (18.6–78.2)	-33.2 (-45.5—18.2)
22	Asthma	1.2 (0.9–1.6)	0.0311 (0.0212–0.0441)	390.9 (264.4–567.9)	48.8 (38.0–59.4)	-8.1 (-14.4—1.6)
23	Alcohol use disorders	1.2 (1.0–1.3)	0.0310 (0.0254–0.0379)	347.1 (281.5–431.6)	62.1 (43.4–87.7)	-1.2 (-10.8–12.1)
24	Breast cancer	1.2 (0.9–1.4)	0.0305 (0.0242–0.0377)	250.1 (197.8–310.9)	29.4 (4.1–59.3)	-40.4 (-52.3—26.5)
25	Endocrine, metabolic, blood, and immune disorders	1.1 (1.0–1.2)	0.0288 (0.0245–0.0332)	274.5 (232.9–319.4)	215.3 (170.6–267.9)	59.0 (39.8–81.6)

Table S4: Cause-specific DALYs by US state in 2021 and percentage change between 1990 and 2021, West Virginia						
**Rank**	**Cause Name**	**2021 Percentage of all cause DALYs**	**2021 DALYs (millions)**	**2021 Age Standardised Rate (per 100 000)**	**Percentage change DALY count 1990 to 2021**	**Percentage change age-standardised DALY rate 1990 to 2021**
	All causes	100.0<br>(100.0–100.0)	0.966<br>(0.855–1.08)	38242.4<br>(33550.3–43107.6)	42.5<br>(32.0–54.3)	25.5<br>(16.8–35.5)
1	COVID-19	11.1<br>(8.8–13.7)	0.107<br>(0.0861–0.131)	3864.5<br>(3098.7–4735.5)	--	--
2	Ischaemic heart disease	7.9<br>(7.0–9.0)	0.0765<br>(0.0659–0.0880)	2203.1<br>(1889.2–2542.4)	-28.5<br>(-38.0–-17.5)	-45.4<br>(-52.8–-36.5)
3	Drug use disorders	7.8<br>(6.7–8.8)	0.0752<br>(0.0622–0.0885)	4474.5<br>(3688.9–5233.4)	983.5<br>(825.4–1200.4)	1105.1<br>(935.0–1338.2)
4	Chronic obstructive pulmonary disease	4.7<br>(4.2–5.2)	0.0454<br>(0.0404–0.0511)	1237.0<br>(1107.3–1392.2)	79.9<br>(60.0–102.5)	34.0<br>(19.1–50.7)
5	Diabetes mellitus	4.4<br>(3.9–5.0)	0.0428<br>(0.0346–0.0525)	1344.7<br>(1092.9–1650.3)	124.1<br>(102.7–143.9)	76.1<br>(59.8–92.3)
6	Tracheal, bronchus, and lung cancer	3.7<br>(3.2–4.2)	0.0353<br>(0.0299–0.0417)	1024.1<br>(864.8–1216.1)	-7.5<br>(-22.0–9.8)	-32.0<br>(-42.9–-18.8)
7	Low back pain	3.1<br>(2.4–3.8)	0.0297<br>(0.0218–0.0387)	1273.9<br>(930.6–1659.7)	4.4<br>(-3.0–12.6)	-4.7<br>(-10.5–3.0)
8	Stroke	3.0<br>(2.7–3.2)	0.0286<br>(0.0248–0.0323)	844.1<br>(732.8–953.6)	11.6<br>(-0.2–25.0)	-13.2<br>(-23.2–-2.2)
9	Chronic kidney disease	2.5<br>(2.3–2.7)	0.0239<br>(0.0208–0.0271)	726.9<br>(636.1–827.0)	228.1<br>(190.4–274.8)	147.1<br>(118.8–180.1)
10	Depressive disorders	2.3<br>(1.6–3.1)	0.0224<br>(0.0146–0.0316)	1268.1<br>(820.2–1793.0)	52.9<br>(23.3–87.0)	68.7<br>(37.0–106.3)
11	Alzheimer's disease and other dementias	2.2<br>(1.0–4.5)	0.0208<br>(0.00952–0.0446)	495.9<br>(228.5–1061.2)	40.3<br>(28.8–53.8)	-4.0<br>(-11.2–5.5)
12	Other musculoskeletal disorders	2.1<br>(1.6–2.7)	0.0204<br>(0.0149–0.0274)	931.0<br>(682.1–1246.9)	47.1<br>(32.9–65.0)	41.7<br>(27.1–59.4)
13	Falls	1.9<br>(1.6–2.2)	0.0182<br>(0.0149–0.0224)	557.9<br>(454.5–687.4)	87.0<br>(70.5–106.1)	33.9<br>(21.9–47.5)
14	Road injuries	1.8<br>(1.7–2.0)	0.0178<br>(0.0156–0.0201)	958.6<br>(837.2–1082.4)	-39.7<br>(-46.1–-32.2)	-40.1<br>(-46.9–-32.4)
15	Cirrhosis and other chronic liver diseases	1.7<br>(1.5–2.0)	0.0168<br>(0.0144–0.0196)	630.0<br>(539.6–735.0)	109.4<br>(77.7–144.6)	77.3<br>(50.2–108.4)
16	Self-harm	1.6<br>(1.4–1.9)	0.0159<br>(0.0133–0.0188)	895.7<br>(750.0–1054.3)	39.5<br>(16.8–66.0)	52.2<br>(27.4–81.2)
17	Anxiety disorders	1.6<br>(1.0–2.3)	0.0153<br>(0.00939–0.0224)	834.9<br>(512.5–1220.9)	27.3<br>(-1.6–60.4)	36.7<br>(5.9–71.9)
18	Colon and rectum cancer	1.4<br>(1.3–1.6)	0.0139<br>(0.0117–0.0164)	435.2<br>(363.9–513.1)	7.5<br>(-10.5–28.0)	-12.0<br>(-26.5–5.5)
19	Age-related and other hearing loss	1.4<br>(1.0–1.9)	0.0137<br>(0.00958–0.0190)	418.5<br>(291.6–579.1)	21.8<br>(17.4–26.5)	-5.5<br>(-8.6–-1.7)
20	Headache disorders	1.3<br>(0.3–2.5)	0.0123<br>(0.00275–0.0260)	674.5<br>(134.5–1416.9)	-10.0<br>(-15.4–1.2)	-5.0<br>(-10.2–0.9)
21	Endocrine, metabolic, blood, and immune disorders	1.2<br>(1.1–1.3)	0.0116<br>(0.0101–0.0132)	463.7<br>(403.0–530.0)	168.3<br>(131.7–216.1)	118.8<br>(91.0–154.8)
22	Osteoarthritis	1.2<br>(0.6–2.2)	0.0112<br>(0.00545–0.0229)	333.0<br>(160.2–675.9)	38.5<br>(34.7–43.0)	4.6<br>(1.7–7.9)
23	Breast cancer	1.0<br>(0.9–1.3)	0.0101<br>(0.00821–0.0124)	342.0<br>(273.6–421.1)	-5.4<br>(-24.6–17.0)	-23.8<br>(-40.0–-4.3)
24	Hypertensive heart disease	0.9<br>(0.8–1.1)	0.00901<br>(0.00770–0.0105)	302.7<br>(259.8–352.3)	136.5<br>(102.4–176.7)	101.4<br>(71.6–137.7)
25	Oral disorders	0.9<br>(0.6–1.2)	0.00872<br>(0.00543–0.0122)	314.7<br>(197.7–453.0)	17.9<br>(-3.0–45.1)	0.3<br>(-15.4–21.7)

Table S4: Cause-specific DALYs by US state in 2021 and percentage change between 1990 and 2021, Wisconsin						
Rank	Cause Name	2021 Percentage of all cause DALYs	2021 DALYs (millions)	2021 Age Standardised Rate (per 100 000)	Percentage change DALY count 1990 to 2021	Percentage change age-standardised DALY rate 1990 to 2021
	All causes	100.0 (100.0–100.0)	2.16 (1.88–2.49)	26802.6 (23056.4–31138.0)	39.3 (28.6–50.9)	-0.1 (-6.7–6.9)
1	Ischaemic heart disease	7.0 (5.9–8.0)	0.150 (0.125–0.177)	1378.1 (1145.3–1639.0)	-28.9 (-39.0–-17.0)	-56.1 (-62.7–-48.5)
2	COVID-19	6.4 (5.5–7.5)	0.138 (0.132–0.155)	1623.1 (1518.4–1865.5)	--	--
3	Drug use disorders	5.0 (4.3–5.8)	0.108 (0.0876–0.128)	1923.5 (1549.9–2274.5)	537.0 (452.3–653.3)	493.1 (412.2–598.2)
4	Low back pain	4.2 (3.4–5.1)	0.0917 (0.0670–0.119)	1243.1 (912.2–1621.0)	22.2 (14.1–31.0)	-8.5 (-14.3–-2.0)
5	Diabetes mellitus	3.9 (3.4–4.4)	0.0841 (0.0661–0.108)	850.3 (664.5–1082.6)	117.7 (93.0–139.7)	36.1 (21.1–49.7)
6	Chronic obstructive pulmonary disease	3.7 (3.2–4.1)	0.0792 (0.0687–0.0894)	696.5 (607.0–784.6)	87.8 (67.5–111.0)	11.9 (-0.3–26.0)
7	Falls	3.2 (2.8–3.7)	0.0693 (0.0567–0.0865)	672.1 (546.9–838.2)	137.7 (118.4–163.4)	38.7 (28.0–53.0)
8	Tracheal, bronchus, and lung cancer	3.1 (2.7–3.6)	0.0671 (0.0560–0.0803)	615.6 (515.5–737.2)	5.1 (-12.4–26.2)	-41.6 (-51.5–-29.8)
9	Other musculoskeletal disorders	3.0 (2.3–3.8)	0.0650 (0.0463–0.0854)	939.6 (674.4–1237.9)	79.1 (60.0–100.4)	39.8 (25.7–55.4)
10	Alzheimer's disease and other dementias	2.9 (1.4–6.1)	0.0634 (0.0290–0.130)	511.0 (233.6–1043.5)	59.8 (45.7–77.0)	-2.7 (-11.7–7.6)
11	Stroke	2.9 (2.5–3.2)	0.0619 (0.0533–0.0707)	578.5 (499.3–660.1)	4.3 (-8.1–17.2)	-33.7 (-41.5–-25.4)
12	Depressive disorders	2.7 (2.0–3.8)	0.0591 (0.0404–0.0851)	997.6 (684.4–1439.3)	63.5 (34.0–99.7)	44.8 (18.6–77.6)
13	Chronic kidney disease	2.4 (2.1–2.6)	0.0507 (0.0433–0.0586)	488.9 (417.8–562.6)	240.7 (197.9–291.3)	109.9 (84.3–139.4)
14	Anxiety disorders	2.2 (1.5–3.1)	0.0468 (0.0306–0.0680)	757.3 (493.9–1104.4)	44.8 (12.3–86.7)	23.7 (-4.1–59.9)
15	Headache disorders	1.9 (0.4–3.9)	0.0418 (0.00933–0.0876)	683.5 (136.0–1445.1)	13.6 (7.4–27.5)	-3.4 (-7.8–2.2)
16	Age-related and other hearing loss	1.9 (1.5–2.5)	0.0416 (0.0291–0.0575)	419.9 (291.7–582.0)	48.1 (42.4–54.3)	-5.1 (-8.2–-1.8)
17	Road injuries	1.9 (1.7–2.0)	0.0405 (0.0345–0.0470)	638.7 (552.5–732.6)	-31.0 (-39.1–-22.7)	-44.8 (-51.3–-37.9)
18	Self-harm	1.9 (1.6–2.2)	0.0401 (0.0331–0.0478)	663.5 (552.3–787.7)	30.2 (8.1–54.8)	12.4 (-6.5–32.7)
19	Osteoarthritis	1.5 (0.8–2.8)	0.0321 (0.0155–0.0647)	312.7 (150.7–629.7)	76.9 (71.9–82.2)	6.3 (3.6–9.5)
20	Cirrhosis and other chronic liver diseases	1.3 (1.1–1.6)	0.0288 (0.0241–0.0342)	331.2 (276.3–393.2)	89.4 (59.2–126.3)	23.0 (2.9–47.0)
21	Colon and rectum cancer	1.3 (1.1–1.5)	0.0277 (0.0226–0.0329)	273.9 (224.2–326.3)	-9.1 (-27.1–11.3)	-42.3 (-53.7–-29.2)
22	Alcohol use disorders	1.3 (1.1–1.4)	0.0273 (0.0224–0.0333)	404.8 (328.7–498.4)	39.7 (21.4–66.3)	10.7 (-1.8–28.1)
23	Neonatal disorders	1.2 (1.1–1.4)	0.0262 (0.0226–0.0299)	732.4 (647.5–817.4)	-17.0 (-25.8–-7.2)	-8.6 (-18.6–2.2)
24	Endocrine, metabolic, blood, and immune disorders	1.1 (1.0–1.3)	0.0242 (0.0207–0.0281)	292.7 (249.4–341.6)	171.5 (128.5–227.4)	77.8 (52.2–110.6)
25	Breast cancer	1.1 (0.9–1.3)	0.0238 (0.0186–0.0289)	251.3 (198.0–308.8)	-15.5 (-32.8–3.1)	-47.3 (-58.5–-35.1)

Table S4: Cause-specific DALYs by US state in 2021 and percentage change between 1990 and 2021, Wyoming						
Rank	Cause Name	2021 Percentage of all cause DALYs	2021 DALYs (millions)	2021 Age Standardised Rate (per 100 000)	Percentage change DALY count 1990 to 2021	Percentage change age-standardised DALY rate 1990 to 2021
	All causes	100.0 (100.0–100.0)	0.229 (0.201–0.258)	30297.6 (26299.0–34258.5)	71.1 (61.5–82.2)	7.9 (2.1–13.9)
1	COVID-19	11.1 (9.9–12.7)	0.0254 (0.0245–0.0277)	3139.1 (2998.1–3496.7)	--	--
2	Ischaemic heart disease	6.0 (5.2–6.9)	0.0138 (0.0120–0.0158)	1382.8 (1211.3–1581.4)	-3.4 (-15.4–9.6)	-51.5 (-57.6–-44.8)
3	Chronic obstructive pulmonary disease	4.6 (4.0–5.2)	0.0106 (0.00947–0.0117)	1017.8 (909.4–1118.3)	113.9 (95.5–135.9)	4.6 (-4.1–14.8)
4	Low back pain	4.2 (3.4–5.2)	0.00975 (0.00705–0.0128)	1393.9 (1011.7–1824.0)	42.5 (32.5–53.3)	0.0 (-6.1–7.3)
5	Drug use disorders	3.9 (3.3–4.6)	0.00903 (0.00721–0.0107)	1644.4 (1304.3–1938.9)	574.0 (484.3–690.1)	482.3 (403.9–577.8)
6	Diabetes mellitus	3.2 (2.8–3.6)	0.00725 (0.00577–0.00896)	787.4 (625.0–968.5)	162.5 (135.6–187.4)	41.6 (27.0–55.2)
7	Other musculoskeletal disorders	3.1 (2.4–3.9)	0.00714 (0.00515–0.00946)	1076.8 (781.7–1432.8)	113.5 (91.7–138.4)	54.9 (38.1–72.9)
8	Depressive disorders	2.8 (2.0–3.8)	0.00636 (0.00432–0.00931)	1102.7 (746.6–1611.8)	82.9 (50.1–120.2)	51.4 (23.2–82.5)
9	Road injuries	2.7 (2.5–2.9)	0.00621 (0.00551–0.00696)	1060.7 (944.3–1181.7)	-21.7 (-28.9–-13.1)	-39.2 (-45.0–-32.3)
10	Self-harm	2.7 (2.3–3.1)	0.00617 (0.00541–0.00714)	1060.7 (929.3–1222.1)	53.9 (31.7–78.4)	23.8 (6.3–43.1)
11	Stroke	2.6 (2.3–2.9)	0.00599 (0.00521–0.00673)	613.2 (538.7–691.7)	37.1 (24.0–50.3)	-29.7 (-36.6–-23.0)
12	Tracheal, bronchus, and lung cancer	2.6 (2.2–3.0)	0.00594 (0.00507–0.00678)	592.0 (507.0–673.8)	20.2 (2.4–39.0)	-40.8 (-49.6–-31.6)
13	Alzheimer's disease and other dementias	2.5 (1.2–5.1)	0.00565 (0.00264–0.0118)	503.5 (235.7–1049.0)	112.3 (97.6–127.3)	-4.0 (-10.5–2.6)
14	Falls	2.3 (2.0–2.6)	0.00519 (0.00419–0.00647)	569.4 (455.8–705.9)	132.5 (114.2–151.6)	25.4 (17.1–35.1)
15	Anxiety disorders	2.0 (1.4–2.8)	0.00467 (0.00286–0.00667)	782.3 (477.8–1121.3)	59.6 (24.9–98.3)	28.9 (1.5–60.0)
16	Cirrhosis and other chronic liver diseases	1.9 (1.6–2.2)	0.00436 (0.00381–0.00502)	561.8 (492.9–644.0)	139.0 (107.7–177.0)	50.1 (30.4–74.6)
17	Chronic kidney disease	1.9 (1.7–2.1)	0.00430 (0.00371–0.00486)	451.3 (396.9–509.0)	318.8 (270.1–375.7)	117.0 (95.1–145.0)
18	Headache disorders	1.7 (0.4–3.5)	0.00399 (0.000890–0.00840)	672.2 (134.3–1420.8)	18.1 (11.8–32.2)	-4.4 (-9.1–1.3)
19	Age-related and other hearing loss	1.7 (1.3–2.2)	0.00394 (0.00275–0.00544)	423.3 (297.5–584.4)	76.2 (69.2–83.9)	-5.2 (-8.4–-2.1)
20	Alcohol use disorders	1.6 (1.4–1.7)	0.00357 (0.00296–0.00429)	555.8 (458.2–674.6)	56.1 (38.8–80.8)	17.8 (6.7–35.3)
21	Osteoarthritis	1.2 (0.6–2.4)	0.00288 (0.00140–0.00586)	300.0 (144.9–605.9)	106.6 (100.0–113.5)	6.2 (3.0–9.7)
22	Colon and rectum cancer	1.2 (1.1–1.4)	0.00280 (0.00240–0.00319)	297.6 (255.0–339.6)	34.4 (14.7–54.6)	-28.9 (-39.5–-18.2)
23	Asthma	1.0 (0.7–1.3)	0.00230 (0.00159–0.00326)	397.1 (270.0–575.9)	14.8 (4.8–24.1)	-9.6 (-16.0–-3.4)
24	Breast cancer	1.0 (0.9–1.2)	0.00230 (0.00194–0.00270)	265.1 (224.2–311.9)	13.6 (-3.5–32.1)	-35.2 (-45.0–-24.6)
25	Neonatal disorders	1.0 (0.9–1.2)	0.00228 (0.00192–0.00271)	664.7 (563.6–782.0)	-23.9 (-35.6–-10.3)	-21.5 (-33.5–-6.3)

Table S5: Risk-specific age-standardised SEVs for the USA and by US state and Washington, DC in 1990, 2000, 2010, 2021, and annualised rate of change for 1990–2021, 2000–2021, and 2010–2021

USA

Risk Names	SEV 1990	SEV 2000	SEV 2010	SEV 2021	Annualised rate of change 1990 to 2021	Annualised rate of change 2000 to 2021	Annualised rate of change 2010 to 2021
**All risk factors**	**27·1 (24·5–30·0)**	**27·0 (24·5–29·8)**	**27·5 (25·1–30·2)**	**27·4 (24·8–30·3)**	**0·0 (-0·2–0·2)**	**0·1 (-0·1–0·2)**	**0·0 (-0·3–0·2)**
**Environmental/occupational risks**	**18·2 (12·1–28·3)**	**17·9 (12·3–27·6)**	**15·5 (10·3–24·2)**	**13·1 (8·5–20·4)**	**-1·1 (-1·8–-0·4)**	**-1·5 (-1·9–-0·9)**	**-1·5 (-1·9–-1·1)**
Unsafe water, sanitation, and handwashing	2·0 (1·1–3·0)	1·8 (0·9–2·7)	1·4 (0·5–2·1)	1·1 (0·4–1·8)	-1·8 (-3·8–-0·4)	-2·3 (-4·6–-0·2)	-1·9 (-4·8–1·0)
Unsafe water source	2·8 (1·2–5·1)	2·5 (1·1–4·6)	1·0 (0·5–1·9)	0·8 (0·4–1·4)	-4·0 (-6·0–-2·0)	-5·4 (-7·9–-2·8)	-2·1 (-5·9–1·9)
Unsafe sanitation	6·5 (4·4–9·3)	5·1 (3·2–7·7)	3·8 (2·3–5·8)	2·8 (1·8–4·3)	-2·7 (-4·3–-1·4)	-2·8 (-4·7–-1·0)	-2·7 (-5·0–-0·1)
No access to handwashing facility	1·7 (0·6–2·8)	1·7 (0·6–2·7)	1·7 (0·7–2·7)	1·4 (0·6–2·4)	-0·5 (-2·4–1·7)	-0·8 (-3·4–2·3)	-1·6 (-5·6–2·3)
Air pollution	23·8 (7·8–34·9)	22·1 (9·1–29·7)	13·5 (5·1–22·6)	8·0 (2·6–16·7)	-3·5 (-6·1–-1·5)	-4·8 (-8·1–-2·5)	-4·7 (-8·1–-2·6)
Particulate matter pollution	13·3 (5·0–23·3)	12·0 (8·7–16·0)	7·5 (4·7–10·9)	4·6 (2·1–7·5)	-3·4 (-6·1–-0·9)	-4·6 (-6·8–-3·4)	-4·5 (-7·3–-3·2)
Ambient particulate matter pollution	20·7 (8·0–35·9)	18·6 (14·0–23·7)	11·5 (7·4–16·2)	7·0 (3·3–11·2)	-3·5 (-6·2–-0·9)	-4·7 (-6·9–-3·5)	-4·5 (-7·3–-3·2)
Household air pollution from solid fuels	0·0 (0·0–0·1)	0·0 (0·0–0·0)	0·0 (0·0–0·0)	0·0 (0·0–0·0)	-3·9 (-27·1–-6·1)	-4·5 (-24·2–-5·8)	-4·1 (-18·2–-4·9)
Ambient ozone pollution	22·0 (18·8–26·4)	22·7 (19·4–27·4)	18·0 (15·2–22·4)	13·7 (11·2–17·1)	-1·5 (-1·7–-1·4)	-2·4 (-2·6–-2·2)	-2·5 (-2·8–-2·1)
Ambient nitrogen dioxide pollution	67·6 (0·0–98·6)	64·6 (0·0–97·9)	38·1 (0·0–85·2)	21·9 (0·0–68·5)	-3·6 (-9·5–0·0)	-5·1 (-13·5–0·0)	-5·0 (-14·6–0·0)
Non-optimal temperature	30·0 (24·8–37·1)	30·8 (26·3–36·8)	34·9 (29·9–41·6)	31·0 (26·3–37·2)	0·1 (0·0–0·3)	0·0 (-0·2–0·2)	-1·1 (-1·3–-0·8)
High temperature	28·7 (22·1–35·9)	27·6 (21·4–34·7)	36·2 (28·4–43·8)	32·9 (25·9–40·0)	0·4 (0·3–0·6)	0·8 (0·5–1·2)	-0·9 (-1·1–-0·6)
Low temperature	25·9 (23·0–29·5)	27·0 (24·4–30·3)	29·4 (27·2–32·3)	26·0 (23·6–29·2)	0·0 (-0·1–0·1)	-0·2 (-0·3–-0·1)	-1·1 (-1·3–-0·9)
Other environmental risks	28·3 (10·1–36·0)	26·5 (10·1–33·9)	23·7 (9·9–30·8)	20·8 (9·9–27·3)	-1·0 (-1·6–-0·1)	-1·2 (-1·9–-0·1)	-1·2 (-2·1–0·0)
Residential radon	26·6 (0·0–43·5)	26·4 (0·0–43·4)	26·3 (0·0–43·4)	26·2 (0·0–43·4)	0·0 (-0·2–0·1)	0·0 (-0·2–0·1)	0·0 (-0·1–0·1)
Lead exposure	29·1 (0·0–35·2)	26·5 (0·0–32·5)	22·5 (0·0–27·5)	18·2 (0·0–22·2)	-1·5 (-1·8–-0·7)	-1·8 (-2·3–-0·5)	-1·9 (-2·4–18·9)
Occupational risks	2·9 (2·4–3·8)	3·0 (2·5–3·9)	2·8 (2·2–3·6)	2·8 (2·2–3·7)	-0·2 (-0·3–0·0)	-0·3 (-0·6–-0·2)	0·0 (-0·2–0·3)
Occupational carcinogens	1·0 (0·5–1·8)	1·0 (0·5–2·0)	1·0 (0·4–1·9)	1·0 (0·4–1·9)	0·1 (-0·2–0·3)	-0·1 (-0·5–0·0)	0·1 (-0·2–0·4)
Occupational exposure to asbestos	4·2 (3·9–4·4)	4·5 (4·3–4·7)	3·9 (3·7–4·1)	3·6 (3·3–3·8)	-0·5 (-0·7–-0·3)	-1·1 (-1·3–-0·8)	-0·8 (-1·2–-0·4)
Occupational exposure to arsenic	0·5 (0·0–1·2)	0·5 (0·0–1·2)	0·5 (0·0–1·1)	0·5 (0·0–1·1)	-0·3 (-0·5–0·0)	-0·4 (-0·8–0·0)	0·1 (-0·5–0·7)
Occupational exposure to benzene	1·1 (0·1–3·1)	1·2 (0·1–3·4)	1·2 (0·1–3·4)	1·2 (0·1–3·5)	0·4 (0·1–0·5)	0·1 (-0·1–0·3)	0·3 (0·0–0·7)
Occupational exposure to beryllium	0·0 (0·0–0·0)	0·0 (0·0–0·0)	0·0 (0·0–0·0)	0·0 (0·0–0·0)	0·2 (0·1–0·3)	0·1 (0·0–0·2)	0·5 (0·3–0·7)
Occupational exposure to cadmium	0·1 (0·1–0·1)	0·1 (0·1–0·1)	0·1 (0·1–0·1)	0·1 (0·1–0·1)	-0·2 (-0·4–0·0)	-0·3 (-0·6–0·0)	0·1 (-0·4–0·7)
Occupational exposure to chromium	0·1 (0·1–0·2)	0·2 (0·1–0·2)	0·1 (0·1–0·1)	0·1 (0·1–0·2)	-0·1 (-0·3–0·1)	-0·2 (-0·5–0·0)	0·2 (-0·2–0·6)
Occupational exposure to diesel engine exhaust	0·7 (0·7–0·7)	0·7 (0·7–0·7)	0·7 (0·7–0·7)	0·7 (0·7–0·8)	0·3 (0·1–0·5)	0·2 (0·0–0·5)	0·7 (0·2–1·1)
Occupational exposure to formaldehyde	0·3 (0·3–0·3)	0·3 (0·3–0·3)	0·3 (0·3–0·3)	0·3 (0·3–0·3)	-0·2 (-0·5–0·0)	-0·4 (-0·7–-0·1)	0·0 (-0·5–0·6)
Occupational exposure to nickel	0·4 (0·0–1·7)	0·4 (0·0–1·6)	0·4 (0·0–1·4)	0·4 (0·0–1·5)	-0·4 (-0·7–0·0)	-0·4 (-0·9–0·0)	0·0 (-0·6–0·7)
Occupational exposure to polycyclic aromatic hydrocarbons	0·3 (0·3–0·3)	0·3 (0·3–0·3)	0·3 (0·3–0·3)	0·3 (0·3–0·3)	0·0 (-0·2–0·1)	-0·2 (-0·5–0·0)	0·2 (-0·2–0·6)
Occupational exposure to silica	3·6 (0·6–11·9)	3·5 (0·5–11·3)	3·2 (0·5–10·2)	3·2 (0·5–10·1)	-0·4 (-0·7–-0·1)	-0·4 (-0·8–0·0)	0·1 (-0·5–0·8)
Occupational exposure to sulphuric acid	0·8 (0·2–2·5)	0·8 (0·2–2·6)	0·7 (0·1–2·3)	0·7 (0·1–2·3)	-0·2 (-0·4–0·0)	-0·3 (-0·7–0·0)	0·2 (-0·4–0·6)
Occupational exposure to trichloroethylene	0·1 (0·1–0·1)	0·1 (0·1–0·1)	0·1 (0·1–0·1)	0·1 (0·1–0·1)	0·1 (-0·1–0·2)	0·0 (-0·2–0·2)	0·4 (0·1–0·7)
Occupational asthmagens	17·3 (15·3–19·3)	17·6 (15·7–19·5)	16·1 (14·4–18·1)	16·5 (14·6–18·5)	-0·2 (-0·4–0·0)	-0·3 (-0·6–-0·1)	0·2 (-0·2–0·6)
Occupational particulate matter, gases, and fumes	6·6 (5·1–8·8)	6·4 (5·0–8·5)	6·0 (4·6–7·8)	5·6 (4·3–7·2)	-0·6 (-0·7–-0·4)	-0·7 (-0·9–-0·5)	-0·7 (-1·0–-0·5)
Occupational noise	6·7 (6·4–7·2)	6·8 (6·4–7·3)	6·6 (6·3–7·1)	6·4 (6·1–6·9)	-0·2 (-0·2–-0·1)	-0·3 (-0·3–-0·2)	-0·3 (-0·4–-0·2)
Occupational injuries	--	--	--	--	--	--	--
Occupational ergonomic factors	8·1 (6·9–9·4)	8·1 (6·9–9·6)	7·2 (6·1–8·7)	7·4 (6·2–9·0)	-0·3 (-0·5–0·0)	-0·4 (-0·7–-0·1)	0·2 (-0·2–0·7)
**Behavioural risks**	**30·3 (27·8–33·4)**	**28·2 (25·7–31·5)**	**26·4 (24·0–29·8)**	**24·6 (22·2–28·0)**	**-0·7 (-0·8–-0·5)**	**-0·7 (-0·8–-0·5)**	**-0·7 (-0·9–-0·4)**
Child and maternal malnutrition	10·4 (7·3–14·1)	8·9 (6·3–11·9)	9·0 (6·5–12·1)	9·1 (6·5–12·3)	-0·4 (-0·9–0·0)	0·1 (-0·5–0·7)	0·2 (-0·9–1·2)
Suboptimal breastfeeding	68·9 (67·0–71·0)	66·8 (65·1–68·7)	62·9 (61·0–65·2)	62·3 (60·1–64·7)	-0·3 (-0·4–-0·2)	-0·3 (-0·4–-0·2)	-0·1 (-0·3–0·1)
Non-exclusive breastfeeding	64·4 (58·3–70·1)	59·7 (55·6–64·5)	48·8 (44·6–54·2)	46·4 (41·4–52·1)	-1·1 (-1·4–-0·7)	-1·2 (-1·6–-0·9)	-0·5 (-1·0–0·1)
Discontinued breastfeeding	88·8 (87·5–90·3)	86·4 (85·0–88·2)	84·5 (82·2–86·7)	84·7 (82·2–87·0)	-0·2 (-0·2–-0·1)	-0·1 (-0·2–0·0)	0·0 (-0·2–0·2)
Child growth failure	1·3 (0·4–3·0)	1·2 (0·4–2·7)	1·0 (0·3–2·3)	1·0 (0·3–2·1)	-1·0 (-1·3–-0·7)	-1·1 (-1·5–-0·8)	-0·7 (-1·2–-0·2)
Child underweight	2·8 (1·8–3·8)	2·5 (1·7–3·4)	2·2 (1·4–2·9)	2·0 (1·3–2·7)	-1·0 (-1·4–-0·7)	-1·1 (-1·6–-0·7)	-0·6 (-1·3–0·1)
Child wasting	0·9 (0·6–1·4)	0·8 (0·5–1·3)	0·8 (0·5–1·3)	0·8 (0·5–1·2)	-0·3 (-0·6–-0·1)	-0·2 (-0·4–0·2)	-0·3 (-0·7–0·2)
Child stunting	3·8 (3·0–4·7)	3·5 (2·8–4·3)	3·0 (2·4–3·7)	2·7 (2·1–3·4)	-1·1 (-1·5–-0·7)	-1·2 (-1·8–-0·7)	-0·9 (-1·6–-0·1)
Low birth weight and short gestation	20·3 (18·3–22·3)	21·9 (19·6–24·2)	23·1 (20·9–25·5)	23·2 (20·9–25·6)	0·4 (0·4–0·5)	0·3 (0·2–0·4)	0·0 (-0·1–0·2)
Short gestation	36·7 (34·3–39·2)	39·8 (37·2–42·5)	40·1 (37·4–42·6)	39·5 (36·8–42·0)	0·2 (0·2–0·3)	0·0 (-0·1–0·1)	-0·1 (-0·3–0·1)
Low birth weight	15·5 (14·8–16·2)	16·3 (15·5–17·1)	17·6 (16·8–18·5)	17·9 (17·1–18·8)	0·5 (0·4–0·5)	0·5 (0·4–0·5)	0·2 (0·0–0·3)

Iron deficiency	3·7 (2·7–4·9)	3·2 (2·3–4·1)	3·2 (2·4–4·2)	3·3 (2·4–4·3)	-0·4 (-0·8–0·0)	0·1 (-0·4–0·7)	0·1 (-0·9–1·1)
Vitamin A deficiency	1·0 (0·0–1·9)	1·4 (0·0–2·5)	0·9 (0·0–1·6)	0·5 (0·0–1·0)	-2·2 (-3·9–0·0)	-4·7 (-6·5–0·0)	-4·7 (-7·3–0·0)
Zinc deficiency	1·8 (0·0–7·6)	1·1 (0·0–4·5)	1·0 (0·0–4·2)	1·0 (0·0–4·1)	-2·0 (-12·6–0·0)	-0·5 (-1·9–1·0)	0·0 (-3·0–5·5)
Tobacco	40·5 (39·1–41·6)	35·3 (34·2–36·4)	28·8 (28·0–29·6)	24·7 (23·5–26·0)	-1·6 (-1·7–-1·4)	-1·7 (-1·9–-1·5)	-1·4 (-1·8–-1·0)
Smoking	37·3 (35·3–39·2)	32·0 (30·3–33·6)	26·0 (24·9–27·4)	22·1 (20·8–23·8)	-1·7 (-1·9–-1·5)	-1·8 (-2·0–-1·5)	-1·5 (-2·0–-1·0)
Chewing tobacco	2·2 (1·9–2·6)	2·2 (1·9–2·5)	2·1 (1·8–2·6)	2·0 (1·6–2·5)	-0·2 (-1·0–0·5)	-0·3 (-1·4–0·8)	-0·4 (-2·0–1·2)
Second-hand smoke	33·6 (32·0–34·5)	29·8 (28·3–30·6)	24·5 (23·3–25·1)	21·4 (20·0–22·4)	-1·5 (-1·6–-1·3)	-1·6 (-1·8–-1·3)	-1·2 (-1·7–-0·8)
High alcohol use	27·4 (22·9–36·2)	26·0 (21·8–34·1)	27·0 (22·5–35·5)	27·4 (22·6–36·0)	0·0 (-0·2–0·2)	0·2 (0·0–0·5)	0·1 (-0·3–0·5)
Drug use	0·9 (0·6–1·6)	1·1 (0·9–1·4)	1·9 (1·6–2·2)	4·7 (3·3–6·0)	5·3 (2·6–7·4)	6·8 (4·4–8·1)	8·3 (6·0–9·4)
Dietary risks	36·5 (27·3–47·2)	39·2 (30·1–50·9)	41·5 (32·5–53·7)	41·5 (32·5–53·4)	0·4 (0·2–0·6)	0·3 (0·1–0·5)	0·0 (-0·3–0·3)
Diet low in fruits	36·7 (31·8–39·3)	33·8 (29·2–36·1)	33·5 (29·6–35·8)	34·7 (30·2–37·9)	-0·2 (-0·4–0·1)	0·1 (-0·2–0·5)	0·3 (-0·3–0·8)
Diet low in vegetables	24·4 (15·2–31·9)	24·5 (15·0–31·8)	27·9 (15·9–34·9)	27·5 (16·4–34·6)	0·4 (0·2–0·6)	0·5 (0·2–0·9)	-0·2 (-0·7–0·4)
Diet low in legumes	37·8 (0·0–46·5)	38·0 (0·0–46·8)	42·1 (0·0–51·8)	42·6 (0·0–53·0)	0·4 (0·0–0·5)	0·5 (0·0–0·8)	0·1 (-0·4–0·5)
Diet low in whole grains	45·0 (37·8–50·7)	48·4 (40·4–54·5)	50·1 (41·8–56·9)	50·4 (42·3–57·0)	0·4 (0·2–0·5)	0·2 (0·0–0·4)	0·1 (-0·2–0·3)
Diet low in nuts and seeds	12·2 (10·7–13·9)	7·1 (6·1–8·3)	5·1 (4·3–6·1)	5·4 (4·0–7·0)	-2·7 (-3·6–-1·8)	-1·3 (-2·7–0·1)	0·5 (-1·8–2·5)
Diet low in milk	35·9 (33·2–38·9)	33·1 (30·3–36·1)	31·7 (28·8–34·8)	31·3 (28·0–35·2)	-0·4 (-0·7–-0·2)	-0·3 (-0·7–0·1)	-0·1 (-0·7–0·4)
Diet high in red meat	50·5 (0·0–67·9)	51·4 (0·0–68·8)	51·6 (0·0–69·0)	51·7 (0·0–69·5)	0·1 (-0·2–0·3)	0·0 (-0·3–0·3)	0·0 (-0·5–0·7)
Diet high in processed meat	45·8 (36·2–49·7)	57·0 (46·2–61·5)	63·9 (51·6–69·0)	62·0 (50·2–67·7)	1·0 (0·7–1·2)	0·4 (0·1–0·7)	-0·3 (-0·8–0·2)
Diet high in sugar-sweetened beverages	36·8 (29·5–41·8)	51·2 (41·4–55·2)	56·6 (46·3–60·9)	56·2 (46·4–62·3)	1·4 (0·9–1·9)	0·4 (0·0–0·9)	-0·1 (-0·9–0·7)
Diet low in fibre	38·6 (20·4–41·6)	29·2 (15·4–31·6)	25·8 (13·7–28·1)	22·4 (12·4–26·3)	-1·8 (-2·2–-1·3)	-1·3 (-2·0–-0·6)	-1·3 (-2·6–-0·2)
Diet low in calcium	5·6 (4·6–6·8)	4·7 (3·8–5·8)	5·0 (4·0–6·1)	4·9 (4·0–6·0)	-0·4 (-0·6–-0·3)	0·2 (0·0–0·4)	-0·2 (-0·4–0·1)
Diet low in seafood omega-3 fatty acids	36·9 (28·2–46·1)	44·0 (35·0–54·0)	47·1 (37·4–57·3)	41·6 (32·6–51·4)	0·4 (0·1–0·7)	-0·3 (-0·6–0·1)	-1·1 (-1·6–-0·6)
Diet low in omega-6 polyunsaturated fatty acids	35·1 (22·9–43·3)	30·8 (20·8–38·2)	23·2 (16·4–28·8)	21·4 (15·0–27·1)	-1·6 (-2·2–-0·9)	-1·7 (-2·4–-1·0)	-0·7 (-1·4–-0·1)
Diet high in trans fatty acids	71·2 (65·7–77·2)	71·7 (66·5–77·3)	63·4 (57·9–68·8)	0·0 (0·0–0·0)	-65·8 (-66·0–-65·5)	-97·1 (-97·5–-96·7)	-184·2 (-185·0–-183·4)
Diet high in sodium	23·4 (0·8–69·8)	28·2 (2·1–75·9)	31·2 (3·6–75·6)	31·6 (3·4–77·7)	1·0 (0·3–5·4)	0·5 (0·1–2·9)	0·1 (-1·6–0·8)
Intimate partner violence	27·3 (17·6–30·4)	27·4 (18·5–30·6)	24·3 (16·8–28·6)	23·4 (14·2–29·7)	-0·5 (-1·4–0·3)	-0·8 (-2·0–0·4)	-0·3 (-1·9–1·0)
Childhood sexual abuse and bullying	9·3 (5·9–14·5)	10·8 (7·0–16·7)	12·1 (7·9–18·9)	9·4 (6·6–13·7)	0·1 (-0·3–0·4)	-0·6 (-1·1–-0·2)	-2·3 (-3·1–-1·5)
Childhood sexual abuse	7·9 (7·2–8·7)	8·8 (8·2–9·5)	10·2 (9·6–10·8)	9·3 (8·5–10·5)	0·5 (0·1–1·0)	0·3 (-0·3–0·9)	-0·8 (-1·5–0·0)
Bullying victimization	8·8 (3·9–17·0)	10·3 (4·7–19·5)	11·4 (5·3–21·7)	7·9 (3·7–14·3)	-0·3 (-0·6–0·1)	-1·2 (-1·6–-0·9)	-3·3 (-3·9–-2·7)
Unsafe sex	--	--	--	--	--	--	--
Low physical activity	19·7 (15·8–24·8)	19·0 (15·0–23·3)	19·7 (15·8–24·1)	22·2 (17·7–27·4)	0·4 (-0·2–1·0)	0·7 (-0·1–1·6)	1·1 (-0·1–2·3)
**Metabolic risks**	**25·5 (22·4–28·5)**	**30·8 (27·2–33·3)**	**34·3 (30·7–36·7)**	**37·5 (33·1–39·9)**	**1·3 (1·0–1·5)**	**0·9 (0·7–1·2)**	**0·8 (0·5–1·1)**
High fasting plasma glucose	12·6 (9·8–14·3)	16·4 (12·7–18·6)	21·2 (16·2–23·7)	24·6 (18·7–27·6)	2·1 (1·8–2·5)	1·9 (1·4–2·5)	1·4 (0·7–2·1)
High LDL cholesterol	63·8 (45·0–86·6)	53·8 (37·0–74·4)	48·2 (32·7–67·3)	46·6 (31·3–65·2)	-1·0 (-1·2–-0·8)	-0·7 (-0·8–-0·5)	-0·3 (-0·5–-0·1)
High systolic blood pressure	29·4 (20·8–39·5)	25·2 (17·8–34·5)	18·8 (12·4–26·8)	26·1 (17·6–35·7)	-0·4 (-1·0–0·1)	0·2 (-0·7–1·0)	3·0 (1·4–4·9)
High body-mass index	29·0 (24·5–33·2)	35·5 (30·5–39·0)	39·5 (33·8–42·7)	42·6 (36·2–45·7)	1·2 (1·0–1·5)	0·9 (0·6–1·1)	0·7 (0·4–1·0)
Low bone mineral density	21·5 (15·8–28·4)	19·9 (14·6–26·6)	20·7 (15·2–27·4)	22·2 (16·6–29·1)	0·1 (0·0–0·2)	0·5 (0·4–0·7)	0·7 (0·4–1·0)
Kidney dysfunction	2·9 (2·4–3·8)	3·0 (2·5–3·9)	2·9 (2·4–3·9)	3·1 (2·5–4·0)	0·1 (0·1–0·2)	0·1 (0·0–0·2)	0·3 (0·2–0·5)

Alabama

Risk Names	SEV 1990	SEV 2000	SEV 2010	SEV 2021	Annualised rate of change 1990 to 2021	Annualised rate of change 2000 to 2021	Annualised rate of change 2010 to 2021
**All risk factors**	**26·6 (23·9–29·7)**	**27·3 (24·7–30·3)**	**27·7 (25·0–30·8)**	**26·9 (24·0–29·9)**	**0·0 (-0·2–0·3)**	**-0·1 (-0·4–0·2)**	**-0·3 (-0·7–0·2)**
**Environmental/occupational risks**	**18·0 (12·2–27·4)**	**17·8 (12·7–26·6)**	**15·8 (10·9–23·9)**	**12·1 (8·1–17·7)**	**-1·3 (-2·1–-0·5)**	**-1·8 (-2·5–-1·3)**	**-2·4 (-3·2–-1·8)**
Unsafe water, sanitation, and handwashing	2·3 (1·3–3·8)	2·1 (1·1–3·4)	1·5 (0·6–2·5)	1·3 (0·5–2·2)	-2·0 (-4·1–0·1)	-2·4 (-5·2–0·3)	-1·6 (-5·0–2·3)
Unsafe water source	3·3 (1·3–6·7)	3·0 (1·1–6·3)	1·2 (0·5–2·3)	1·0 (0·4–1·9)	-4·0 (-6·6–-1·3)	-5·4 (-9·0–-1·5)	-1·6 (-7·3–4·1)
Unsafe sanitation	8·4 (4·7–14·0)	6·6 (3·6–11·2)	4·7 (2·5–8·2)	3·8 (2·0–6·7)	-2·5 (-4·8–-0·5)	-2·6 (-5·2–-0·1)	-2·0 (-5·3–1·2)
No access to handwashing facility	1·8 (0·5–3·5)	1·8 (0·6–3·4)	1·7 (0·6–3·3)	1·5 (0·5–3·0)	-0·5 (-3·6–2·8)	-0·8 (-5·1–3·5)	-1·3 (-7·1–4·2)
Air pollution	19·1 (8·9–30·5)	17·5 (11·4–26·8)	11·0 (6·5–19·2)	6·4 (2·7–13·1)	-3·5 (-5·7–-1·8)	-4·8 (-7·1–-3·3)	-4·9 (-8·5–-3·0)
Particulate matter pollution	15·9 (6·9–26·6)	14·8 (11·3–19·1)	9·7 (6·6–13·4)	5·5 (2·7–8·8)	-3·4 (-5·9–-1·1)	-4·7 (-7·0–-3·4)	-5·1 (-8·8–-3·4)
Ambient particulate matter pollution	24·8 (10·6–42·1)	22·9 (18·1–28·6)	14·8 (10·3–19·9)	8·4 (4·1–13·2)	-3·5 (-6·0–-1·1)	-4·8 (-7·1–-3·5)	-5·2 (-8·8–-3·4)
Household air pollution from solid fuels	0·0 (0·0–0·2)	0·0 (0·0–0·1)	0·0 (0·0–0·0)	0·0 (0·0–0·0)	-4·5 (-26·1–-5·1)	-4·6 (-22·2–-4·5)	-3·8 (-16·7–-2·8)
Ambient ozone pollution	24·0 (17·8–30·8)	28·4 (22·0–36·0)	17·1 (12·0–23·1)	6·5 (3·1–10·9)	-4·2 (-5·8–-3·2)	-7·0 (-9·6–-5·4)	-8·8 (-13·0–-6·2)
Ambient nitrogen dioxide pollution	28·2 (0·0–77·2)	23·8 (0·0–70·0)	13·1 (0·0–50·9)	8·4 (0·0–42·6)	-3·9 (-12·7–0·0)	-4·9 (-16·7–0·0)	-4·0 (-17·7–0·0)
Non-optimal temperature	29·7 (22·8–39·8)	31·4 (25·3–41·3)	37·5 (30·0–47·6)	25·4 (20·4–33·2)	-0·5 (-0·8–-0·2)	-1·0 (-1·4–-0·7)	-3·5 (-4·4–-2·6)
High temperature	24·7 (16·8–34·2)	25·2 (17·7–34·7)	31·4 (22·6–41·0)	18·1 (11·9–26·3)	-1·0 (-1·4–-0·6)	-1·6 (-2·4–-1·0)	-5·0 (-6·7–-3·5)
Low temperature	24·8 (21·5–28·9)	27·8 (24·7–31·3)	32·3 (29·4–35·6)	23·3 (20·5–27·2)	-0·2 (-0·4–0·0)	-0·8 (-1·1–-0·6)	-2·9 (-3·5–-2·3)
Other environmental risks	25·8 (6·0–42·2)	24·4 (6·0–40·6)	21·9 (6·0–37·8)	19·0 (6·0–35·2)	-1·0 (-1·6–0·0)	-1·2 (-2·0–0·0)	-1·3 (-2·3–0·0)
Residential radon	22·7 (0·0–75·9)	22·7 (0·0–75·9)	22·7 (0·0–75·9)	22·7 (0·0–75·9)	0·0 (0·0–0·0)	0·0 (0·0–0·0)	0·0 (0·0–0·0)
Lead exposure	27·2 (0·0–34·1)	25·1 (0·0–31·3)	21·5 (0·0–26·8)	17·3 (0·0–21·8)	-1·4 (-1·9–0·0)	-1·8 (-2·2–0·0)	-2·0 (-2·7–0·0)
Occupational risks	2·8 (2·4–3·7)	2·9 (2·4–3·7)	2·6 (2·2–3·5)	2·7 (2·2–3·5)	-0·2 (-0·5–0·1)	-0·4 (-0·8–0·0)	0·1 (-0·4–0·6)
Occupational carcinogens	0·9 (0·4–1·6)	0·9 (0·4–1·8)	0·9 (0·4–1·8)	0·9 (0·4–1·8)	0·2 (-0·2–0·5)	-0·1 (-0·5–0·4)	0·3 (-0·4–0·8)
Occupational exposure to asbestos	3·5 (3·1–4·0)	3·8 (3·4–4·2)	3·5 (3·1–3·9)	3·2 (2·6–4·1)	-0·2 (-1·1–0·6)	-0·8 (-2·1–0·4)	-0·8 (-3·1–1·5)
Occupational exposure to arsenic	0·5 (0·0–1·2)	0·5 (0·0–1·3)	0·5 (0·0–1·1)	0·5 (0·0–1·2)	-0·3 (-0·8–0·2)	-0·5 (-1·2–0·1)	0·1 (-0·8–1·0)
Occupational exposure to benzene	1·0 (0·1–2·8)	1·1 (0·1–3·1)	1·1 (0·1–3·1)	1·1 (0·1–3·2)	0·4 (0·0–0·7)	0·2 (-0·3–0·6)	0·5 (-0·1–1·1)
Occupational exposure to beryllium	0·0 (0·0–0·0)	0·0 (0·0–0·0)	0·0 (0·0–0·0)	0·0 (0·0–0·0)	0·1 (-0·1–0·4)	0·0 (-0·3–0·4)	0·6 (0·1–1·1)
Occupational exposure to cadmium	0·1 (0·1–0·1)	0·1 (0·1–0·1)	0·1 (0·1–0·1)	0·1 (0·1–0·1)	-0·3 (-0·7–0·2)	-0·4 (-1·0–0·1)	0·2 (-0·7–1·0)
Occupational exposure to chromium	0·1 (0·1–0·2)	0·2 (0·1–0·2)	0·1 (0·1–0·1)	0·1 (0·1–0·2)	-0·1 (-0·5–0·3)	-0·3 (-0·8–0·2)	0·3 (-0·5–1·0)
Occupational exposure to diesel engine exhaust	0·6 (0·6–0·7)	0·6 (0·6–0·7)	0·6 (0·6–0·7)	0·7 (0·6–0·7)	0·2 (-0·2–0·6)	0·2 (-0·4–0·8)	0·8 (0·1–1·5)
Occupational exposure to formaldehyde	0·3 (0·3–0·3)	0·3 (0·3–0·3)	0·3 (0·3–0·3)	0·3 (0·3–0·3)	-0·3 (-0·7–0·2)	-0·5 (-1·1–0·1)	0·1 (-0·8–0·9)
Occupational exposure to nickel	0·4 (0·0–1·7)	0·4 (0·0–1·7)	0·4 (0·0–1·5)	0·4 (0·0–1·5)	-0·4 (-1·0–0·2)	-0·6 (-1·3–0·2)	0·1 (-1·0–1·1)
Occupational exposure to polycyclic aromatic hydrocarbons	0·3 (0·3–0·3)	0·3 (0·3–0·3)	0·3 (0·3–0·3)	0·3 (0·3–0·3)	-0·1 (-0·4–0·3)	-0·3 (-0·7–0·2)	0·3 (-0·4–0·9)
Occupational exposure to silica	3·9 (0·6–13·0)	3·7 (0·6–12·1)	3·3 (0·5–10·9)	3·4 (0·5–10·8)	-0·4 (-0·9–0·0)	-0·5 (-1·2–0·1)	0·2 (-0·7–1·1)
Occupational exposure to sulphuric acid	0·8 (0·2–2·5)	0·8 (0·2–2·5)	0·7 (0·1–2·3)	0·7 (0·1–2·3)	-0·2 (-0·7–0·2)	-0·4 (-1·1–0·2)	0·2 (-0·7–1·0)
Occupational exposure to trichloroethylene	0·1 (0·1–0·1)	0·1 (0·1–0·1)	0·1 (0·1–0·1)	0·1 (0·1–0·1)	0·0 (-0·3–0·3)	-0·1 (-0·6–0·3)	0·4 (-0·1–1·0)
Occupational asthmagens	17·1 (14·9–19·4)	17·4 (15·3–19·7)	15·4 (13·5–17·6)	15·9 (13·8–18·2)	-0·2 (-0·6–0·2)	-0·4 (-0·9–0·1)	0·3 (-0·4–1·0)
Occupational particulate matter, gases, and fumes	6·7 (5·2–8·9)	6·6 (5·0–8·8)	6·2 (4·7–8·2)	5·7 (4·4–7·4)	-0·6 (-0·8–-0·3)	-0·7 (-1·0–-0·4)	-0·8 (-1·2–-0·4)
Occupational noise	6·6 (6·0–7·3)	6·7 (6·1–7·3)	6·5 (6·1–7·1)	6·3 (5·9–6·8)	-0·2 (-0·3–0·0)	-0·3 (-0·5–-0·1)	-0·4 (-0·6–-0·1)
Occupational injuries	--	--	--	--	--	--	--
Occupational ergonomic factors	8·2 (6·9–9·9)	8·1 (6·8–9·7)	7·0 (5·9–8·4)	7·3 (6·1–9·1)	-0·4 (-0·8–0·1)	-0·5 (-1·1–0·1)	0·4 (-0·3–1·1)
**Behavioural risks**	**29·8 (26·7–32·8)**	**29·2 (26·2–32·5)**	**28·5 (25·4–32·0)**	**25·6 (22·4–28·8)**	**-0·5 (-0·8–-0·2)**	**-0·6 (-1·0–-0·2)**	**-1·0 (-1·6–-0·4)**
Child and maternal malnutrition	9·8 (6·8–13·4)	8·6 (6·0–11·9)	8·7 (6·1–11·8)	8·9 (6·0–12·2)	-0·3 (-1·1–0·5)	0·2 (-1·0–1·3)	0·2 (-1·5–1·9)
Suboptimal breastfeeding	69·0 (66·5–71·6)	66·8 (64·3–69·7)	63·0 (60·3–65·7)	62·4 (59·7–65·4)	-0·3 (-0·5–-0·2)	-0·3 (-0·5–-0·2)	-0·1 (-0·4–0·2)
Non-exclusive breastfeeding	64·7 (56·5–71·9)	59·9 (53·1–66·8)	48·9 (42·6–56·0)	46·8 (40·0–54·4)	-1·0 (-1·6–-0·6)	-1·2 (-1·8–-0·5)	-0·4 (-1·3–0·5)
Discontinued breastfeeding	88·9 (86·9–91·0)	86·5 (83·9–89·1)	84·6 (81·2–87·6)	84·8 (81·3–87·8)	-0·2 (-0·3–0·0)	-0·1 (-0·3–0·1)	0·0 (-0·3–0·3)
Child growth failure	1·5 (0·5–3·4)	1·4 (0·4–3·1)	1·2 (0·4–2·7)	1·1 (0·4–2·5)	-0·8 (-1·2–-0·5)	-0·9 (-1·5–-0·4)	-0·4 (-1·2–0·3)
Child underweight	3·1 (1·9–4·3)	2·9 (1·9–3·9)	2·5 (1·6–3·4)	2·4 (1·5–3·2)	-0·9 (-1·4–-0·3)	-0·9 (-1·6–-0·3)	-0·4 (-1·4–0·6)
Child wasting	1·0 (0·6–1·5)	0·9 (0·6–1·4)	0·9 (0·6–1·4)	0·9 (0·6–1·4)	-0·2 (-0·6–0·1)	-0·1 (-0·5–0·4)	-0·1 (-0·8–0·5)
Child stunting	4·2 (3·2–5·3)	3·9 (3·1–4·8)	3·4 (2·6–4·3)	3·1 (2·4–4·0)	-0·9 (-1·5–-0·3)	-1·0 (-1·8–-0·3)	-0·6 (-1·8–0·6)
Low birth weight and short gestation	22·3 (19·5–25·5)	24·4 (21·3–27·6)	26·8 (23·4–30·6)	26·0 (22·5–29·8)	0·5 (0·2–0·7)	0·3 (-0·1–0·7)	-0·3 (-1·0–0·4)
Short gestation	37·7 (33·1–42·9)	41·8 (36·6–47·2)	44·7 (39·4–50·7)	42·9 (37·7–48·8)	0·4 (0·1–0·7)	0·1 (-0·3–0·5)	-0·4 (-1·2–0·5)
Low birth weight	16·8 (15·1–18·6)	17·8 (16·0–19·6)	19·7 (17·7–21·8)	19·3 (17·4–21·2)	0·4 (0·2–0·7)	0·4 (0·0–0·7)	-0·2 (-0·9–0·5)

Iron deficiency	3·6 (2·5–4·8)	3·1 (2·3–4·3)	3·2 (2·3–4·2)	3·3 (2·3–4·5)	-0·3 (-1·0–0·5)	0·2 (-0·8–1·3)	0·1 (-1·5–1·8)
Vitamin A deficiency	1·4 (0·0–2·8)	1·9 (0·0–4·2)	1·1 (0·0–2·5)	0·8 (0·0–1·6)	-1·9 (-4·0–0·2)	-4·4 (-7·4–0·0)	-3·7 (-7·9–0·0)
Zinc deficiency	2·1 (0·0–8·7)	1·2 (0·0–5·3)	1·1 (0·0–4·8)	1·1 (0·0–4·7)	-2·0 (-47·7–0·0)	-0·4 (-1·3–4·1)	0·2 (-3·5–2·9)
Tobacco	37·8 (36·3–39·3)	36·3 (34·9–37·5)	33·1 (32·1–34·2)	28·3 (26·5–30·1)	-0·9 (-1·1–-0·7)	-1·2 (-1·5–-0·9)	-1·4 (-2·0–-0·9)
Smoking	34·9 (32·7–36·9)	33·6 (31·7–35·3)	30·2 (28·8–31·8)	25·9 (23·8–28·2)	-1·0 (-1·3–-0·7)	-1·2 (-1·6–-0·8)	-1·4 (-2·2–-0·7)
Chewing tobacco	4·8 (4·0–5·7)	4·5 (3·7–5·4)	4·1 (3·3–5·3)	3·8 (2·9–5·1)	-0·7 (-1·8–0·3)	-0·8 (-2·2–0·7)	-0·7 (-3·1–1·5)
Second-hand smoke	31·0 (29·4–32·2)	29·5 (27·8–30·6)	27·0 (25·2–28·1)	23·3 (21·4–25·1)	-0·9 (-1·2–-0·7)	-1·1 (-1·5–-0·8)	-1·4 (-2·0–-0·7)
High alcohol use	23·6 (17·6–32·9)	22·6 (16·5–31·7)	23·6 (17·2–32·9)	23·4 (16·2–32·6)	0·0 (-1·0–1·0)	0·2 (-1·1–1·4)	-0·1 (-1·9–1·7)
Drug use	0·8 (0·5–1·3)	1·4 (1·2–1·7)	2·8 (2·0–3·7)	5·5 (3·7–7·2)	6·2 (3·5–8·3)	6·5 (4·8–7·3)	6·0 (5·1–6·9)
Dietary risks	38·1 (29·2–49·0)	42·2 (32·7–54·3)	46·6 (36·4–59·4)	46·3 (36·9–58·6)	0·6 (0·4–0·9)	0·4 (0·1–0·7)	-0·1 (-0·5–0·4)
Diet low in fruits	39·3 (34·2–42·9)	37·8 (33·0–41·1)	40·3 (35·5–43·9)	41·6 (35·2–46·4)	0·2 (-0·2–0·5)	0·5 (0·0–0·9)	0·3 (-0·5–1·1)
Diet low in vegetables	31·5 (19·4–39·4)	37·5 (22·9–45·8)	51·3 (27·1–59·6)	49·8 (27·7–58·9)	1·5 (1·1–1·9)	1·3 (0·8–1·9)	-0·3 (-0·9–0·4)
Diet low in legumes	39·4 (0·0–49·0)	39·6 (0·0–49·4)	43·9 (0·0–54·8)	44·7 (0·0–56·5)	0·4 (0·0–0·7)	0·6 (0·0–1·0)	0·2 (-0·6–0·8)
Diet low in whole grains	45·6 (37·7–52·2)	49·1 (41·1–56·0)	51·2 (43·0–58·6)	51·0 (42·4–58·4)	0·4 (0·1–0·6)	0·2 (-0·2–0·5)	0·0 (-0·6–0·5)
Diet low in nuts and seeds	15·0 (11·3–19·5)	9·0 (6·1–12·2)	6·6 (4·3–9·3)	7·1 (4·6–10·6)	-2·4 (-3·9–-1·0)	-1·1 (-3·3–1·0)	0·7 (-2·6–3·9)
Diet low in milk	39·0 (35·0–44·1)	36·0 (31·9–40·8)	34·5 (30·2–39·3)	34·4 (29·7–39·3)	-0·4 (-0·9–0·0)	-0·2 (-0·8–0·4)	0·0 (-0·9–0·9)
Diet high in red meat	48·6 (0·0–67·9)	49·3 (0·0–67·0)	49·4 (0·0–67·6)	49·1 (0·0–67·7)	0·0 (-0·6–0·5)	0·0 (-0·9–0·6)	-0·1 (-1·1–1·0)
Diet high in processed meat	45·8 (36·1–52·2)	57·7 (46·2–64·7)	65·3 (52·1–73·4)	62·4 (49·5–71·5)	1·0 (0·5–1·4)	0·4 (-0·2–0·9)	-0·4 (-1·2–0·3)
Diet high in sugar-sweetened beverages	33·5 (25·6–41·4)	48·2 (38·3–56·5)	53·9 (43·1–62·7)	51·9 (39·6–61·7)	1·4 (0·5–2·3)	0·4 (-0·7–1·3)	-0·3 (-1·7–1·0)
Diet low in fibre	42·4 (22·3–49·8)	32·4 (17·2–39·9)	28·9 (16·3–35·4)	25·7 (14·6–32·3)	-1·6 (-2·6–-0·8)	-1·1 (-2·4–0·0)	-1·1 (-2·9–0·6)
Diet low in calcium	6·3 (5·2–7·8)	5·2 (4·2–6·4)	5·6 (4·5–6·8)	5·5 (4·5–6·7)	-0·4 (-0·6–-0·2)	0·3 (0·0–0·6)	-0·1 (-0·5–0·4)
Diet low in seafood omega-3 fatty acids	40·2 (30·4–50·5)	46·5 (36·2–58·0)	49·8 (38·7–61·2)	45·2 (35·4–56·2)	0·4 (0·0–0·8)	-0·1 (-0·6–0·3)	-0·9 (-1·6–-0·2)
Diet low in omega-6 polyunsaturated fatty acids	39·6 (25·4–50·7)	34·3 (22·4–44·4)	25·8 (18·1–32·9)	24·3 (16·8–31·8)	-1·6 (-2·3–-0·8)	-1·6 (-2·6–-0·8)	-0·6 (-1·7–0·3)
Diet high in trans fatty acids	71·5 (63·0–78·0)	71·8 (62·8–78·7)	63·3 (55·4–71·6)	0·0 (0·0–0·0)	-65·8 (-66·0–-65·4)	-97·1 (-97·5–-96·5)	-184·2 (-185·4–-183·0)
Diet high in sodium	23·5 (0·6–69·7)	28·6 (1·9–76·3)	31·8 (3·4–79·0)	32·1 (3·3–80·0)	1·0 (0·3–6·3)	0·5 (-0·1–3·7)	0·1 (-2·2–1·7)
Intimate partner violence	25·3 (15·4–32·7)	25·3 (16·0–32·2)	22·9 (14·3–28·6)	22·1 (11·9–30·1)	-0·4 (-1·8–1·0)	-0·6 (-2·4–1·3)	-0·3 (-3·1–1·7)
Childhood sexual abuse and bullying	21·0 (12·4–30·2)	22·4 (14·5–31·3)	23·4 (15·5–32·1)	16·3 (11·0–22·6)	-0·8 (-1·7–0·0)	-1·5 (-2·5–-0·6)	-3·3 (-5·2–-1·5)
Childhood sexual abuse	12·0 (10·1–14·3)	13·6 (11·6–16·2)	15·4 (13·5–17·5)	13·7 (11·3–16·9)	0·4 (-0·4–1·3)	0·1 (-0·9–1·1)	-1·0 (-2·6–0·5)
Bullying victimization	23·1 (10·8–36·7)	24·0 (12·3–37·0)	24·4 (12·9–36·9)	15·1 (7·5–24·7)	-1·4 (-2·2–-0·4)	-2·2 (-3·4–-1·0)	-4·3 (-6·6–-2·2)
Unsafe sex	--	--	--	--	--	--	--
Low physical activity	20·8 (15·6–27·2)	20·5 (16·0–25·7)	21·7 (17·2–26·7)	23·7 (17·9–30·2)	0·4 (-0·5–1·5)	0·7 (-0·5–1·9)	0·8 (-0·8–2·4)
**Metabolic risks**	**25·5 (22·2–28·6)**	**33·4 (29·5–36·4)**	**37·5 (33·2–40·4)**	**41·4 (36·3–44·4)**	**1·6 (1·3–1·9)**	**1·0 (0·7–1·3)**	**0·9 (0·5–1·4)**
High fasting plasma glucose	13·7 (10·5–16·6)	18·6 (13·8–22·3)	24·2 (17·2–29·0)	26·6 (19·3–31·7)	2·1 (1·5–2·8)	1·7 (0·8–2·6)	0·8 (-0·4–2·1)
High LDL cholesterol	63·2 (44·8–86·4)	53·7 (37·3–74·5)	48·3 (32·6–67·1)	46·7 (31·1–65·2)	-1·0 (-1·2–-0·8)	-0·7 (-0·9–-0·5)	-0·3 (-0·6–-0·1)
High systolic blood pressure	30·8 (21·3–41·7)	27·2 (19·4–36·5)	20·9 (14·0–29·7)	28·6 (18·8–41·3)	-0·2 (-1·2–0·6)	0·2 (-0·9–1·3)	2·9 (0·8–5·0)
High body-mass index	29·0 (24·4–33·3)	38·2 (32·4–42·1)	42·9 (36·5–46·6)	47·0 (39·9–50·9)	1·6 (1·2–1·9)	1·0 (0·7–1·3)	0·8 (0·4–1·3)
Low bone mineral density	22·1 (16·0–29·2)	21·9 (16·0–28·7)	23·3 (16·8–30·2)	24·7 (18·2–31·9)	0·4 (-0·1–0·8)	0·6 (-0·2–1·3)	0·5 (-0·6–1·8)
Kidney dysfunction	3·0 (2·5–4·0)	3·2 (2·6–4·1)	3·1 (2·6–4·2)	3·3 (2·7–4·3)	0·2 (0·1–0·3)	0·2 (0·0–0·3)	0·4 (0·1–0·7)

Alaska

Risk Names	SEV 1990	SEV 2000	SEV 2010	SEV 2021	Annualised rate of change 1990 to 2021	Annualised rate of change 2000 to 2021	Annualised rate of change 2010 to 2021
**All risk factors**	**24·1 (21·3–27·0)**	**24·0 (21·4–26·8)**	**24·2 (21·4–27·1)**	**24·0 (21·0–27·1)**	**0·0 (-0·3–0·3)**	**0·0 (-0·4–0·3)**	**-0·1 (-0·6–0·4)**
**Environmental/occupational risks**	**14·8 (9·3–24·1)**	**15·2 (10·0–25·0)**	**14·1 (9·3–23·1)**	**14·2 (9·3–23·5)**	**-0·1 (-0·9–0·7)**	**-0·3 (-0·7–0·1)**	**0·0 (-0·5–0·7)**
Unsafe water, sanitation, and handwashing	2·7 (1·2–4·5)	2·4 (0·9–4·1)	2·0 (0·5–3·5)	1·6 (0·4–3·0)	-1·6 (-4·5–0·8)	-1·9 (-5·7–0·9)	-2·0 (-6·5–2·1)
Unsafe water source	3·1 (1·1–6·6)	2·6 (1·0–5·3)	1·1 (0·4–2·2)	0·8 (0·4–1·6)	-4·2 (-6·8–-1·3)	-5·4 (-9·2–-1·4)	-2·4 (-8·0–3·2)
Unsafe sanitation	7·3 (3·7–12·0)	5·3 (2·6–8·9)	4·1 (2·1–7·1)	2·9 (1·5–4·9)	-3·0 (-5·0–-0·8)	-2·9 (-5·6–-0·2)	-3·2 (-6·5–0·2)
No access to handwashing facility	2·9 (0·9–5·6)	2·9 (1·0–5·7)	2·9 (1·0–5·7)	2·4 (0·8–4·7)	-0·5 (-3·5–2·5)	-0·8 (-4·9–3·5)	-1·7 (-7·6–4·2)
Air pollution	12·9 (3·2–22·9)	11·4 (3·3–20·8)	8·7 (2·3–16·8)	7·7 (2·8–15·0)	-1·7 (-3·6–0·6)	-1·9 (-3·6–0·3)	-1·1 (-3·2–2·9)
Particulate matter pollution	7·2 (1·8–14·7)	5·1 (2·6–8·2)	4·4 (2·0–7·3)	5·2 (2·6–8·4)	-1·1 (-3·4–2·9)	0·0 (-1·2–1·4)	1·6 (-0·6–4·8)
Ambient particulate matter pollution	11·1 (2·8–23·0)	7·8 (4·0–12·1)	6·6 (3·0–10·9)	7·9 (4·1–12·7)	-1·1 (-3·5–2·9)	0·1 (-1·2–1·4)	1·6 (-0·6–4·8)
Household air pollution from solid fuels	0·0 (0·0–0·1)	0·0 (0·0–0·1)	0·0 (0·0–0·0)	0·0 (0·0–0·0)	-1·6 (-25·9–-4·2)	-2·3 (-34·3–-5·4)	-0·3 (-20·7–-2·0)
Ambient ozone pollution	5·1 (1·8–10·8)	7·7 (2·5–14·8)	3·6 (1·3–7·3)	2·0 (0·5–5·4)	-3·0 (-6·3–0·0)	-6·4 (-11·4–-1·5)	-5·1 (-13·3–1·9)
Ambient nitrogen dioxide pollution	37·2 (0·0–84·2)	38·8 (0·0–88·3)	27·5 (0·0–72·5)	18·0 (0·0–57·3)	-2·3 (-7·2–0·0)	-3·7 (-11·0–0·0)	-3·9 (-12·7–0·0)
Non-optimal temperature	28·8 (23·5–36·0)	30·2 (24·2–37·3)	29·8 (24·4–36·6)	29·9 (24·6–36·3)	0·1 (-0·4–0·6)	-0·1 (-0·4–0·3)	0·0 (-0·3–0·5)
High temperature	14·4 (6·2–24·6)	12·3 (5·1–22·3)	13·4 (5·9–23·5)	15·1 (7·4–25·4)	0·2 (-0·7–1·2)	1·0 (0·1–2·4)	1·1 (-0·4–3·1)
Low temperature	28·3 (24·3–34·7)	30·0 (24·3–37·1)	29·6 (24·5–35·8)	29·6 (24·7–35·8)	0·1 (-0·2–0·5)	-0·1 (-0·3–0·2)	0·0 (-0·3–0·5)
Other environmental risks	29·8 (11·3–50·7)	28·4 (11·3–49·1)	26·1 (11·1–46·7)	23·4 (10·0–43·4)	-0·8 (-1·5–0·0)	-0·9 (-1·8–0·0)	-1·0 (-2·1–0·0)
Residential radon	38·0 (0·0–100·0)	38·0 (0·0–100·0)	38·0 (0·0–100·0)	38·0 (0·0–100·0)	0·0 (0·0–0·0)	0·0 (0·0–0·0)	0·0 (0·0–0·0)
Lead exposure	26·1 (0·0–32·9)	24·0 (0·0–29·9)	20·7 (0·0–25·6)	16·8 (0·0–20·7)	-1·4 (-1·9–0·0)	-1·7 (-2·2–0·0)	-1·9 (-2·7–0·0)
Occupational risks	2·7 (2·2–3·6)	2·8 (2·3–3·7)	2·7 (2·2–3·5)	2·7 (2·2–3·6)	0·0 (-0·3–0·3)	-0·2 (-0·6–0·2)	0·1 (-0·4–0·6)
Occupational carcinogens	0·9 (0·4–1·7)	1·0 (0·5–1·9)	1·0 (0·5–2·0)	1·0 (0·4–2·0)	0·3 (-0·2–0·6)	0·0 (-0·5–0·4)	0·1 (-0·6–0·7)
Occupational exposure to asbestos	6·5 (5·9–7·1)	6·5 (5·9–7·0)	5·7 (5·2–6·3)	5·1 (4·0–6·2)	-0·8 (-1·5–0·0)	-1·1 (-2·2–-0·1)	-1·1 (-2·9–0·6)
Occupational exposure to arsenic	0·4 (0·0–0·9)	0·4 (0·0–1·0)	0·4 (0·0–0·9)	0·4 (0·0–1·0)	0·2 (-0·2–0·6)	0·1 (-0·5–0·6)	0·3 (-0·5–1·2)
Occupational exposure to benzene	1·0 (0·1–2·9)	1·1 (0·1–3·2)	1·2 (0·1–3·4)	1·2 (0·1–3·5)	0·6 (0·2–0·9)	0·3 (-0·2–0·7)	0·3 (-0·3–1·0)
Occupational exposure to beryllium	0·0 (0·0–0·0)	0·0 (0·0–0·0)	0·0 (0·0–0·0)	0·0 (0·0–0·0)	0·3 (0·0–0·5)	0·1 (-0·2–0·5)	0·4 (-0·1–0·8)
Occupational exposure to cadmium	0·0 (0·0–0·1)	0·0 (0·0–0·1)	0·0 (0·0–0·1)	0·1 (0·0–0·1)	0·3 (-0·1–0·6)	0·1 (-0·4–0·6)	0·3 (-0·3–1·1)
Occupational exposure to chromium	0·1 (0·1–0·1)	0·1 (0·1–0·1)	0·1 (0·1–0·1)	0·1 (0·1–0·1)	0·4 (0·0–0·7)	0·2 (-0·3–0·6)	0·4 (-0·2–1·1)
Occupational exposure to diesel engine exhaust	0·7 (0·6–0·7)	0·7 (0·7–0·8)	0·7 (0·7–0·8)	0·8 (0·7–0·8)	0·4 (0·0–0·7)	0·3 (-0·2–0·7)	0·6 (-0·1–1·2)
Occupational exposure to formaldehyde	0·2 (0·2–0·2)	0·2 (0·2–0·3)	0·2 (0·2–0·3)	0·2 (0·2–0·3)	0·3 (-0·1–0·7)	0·1 (-0·5–0·6)	0·3 (-0·5–1·1)
Occupational exposure to nickel	0·3 (0·0–1·3)	0·3 (0·0–1·4)	0·3 (0·0–1·3)	0·3 (0·0–1·3)	0·0 (-0·5–0·4)	-0·2 (-0·8–0·5)	0·1 (-0·8–1·2)
Occupational exposure to polycyclic aromatic hydrocarbons	0·2 (0·2–0·3)	0·3 (0·2–0·3)	0·3 (0·2–0·3)	0·3 (0·3–0·3)	0·4 (0·1–0·7)	0·2 (-0·3–0·7)	0·4 (-0·3–1·0)
Occupational exposure to silica	3·2 (0·5–10·3)	3·2 (0·5–10·3)	3·1 (0·5–9·9)	3·1 (0·5–9·8)	-0·1 (-0·7–0·3)	-0·2 (-0·9–0·4)	0·1 (-0·9–1·1)
Occupational exposure to sulphuric acid	0·6 (0·1–1·9)	0·6 (0·1–2·1)	0·6 (0·1–2·1)	0·7 (0·1–2·2)	0·3 (-0·1–0·7)	0·2 (-0·4–0·7)	0·4 (-0·4–1·2)
Occupational exposure to trichloroethylene	0·1 (0·1–0·1)	0·1 (0·1–0·1)	0·1 (0·1–0·1)	0·1 (0·1–0·1)	0·4 (0·1–0·7)	0·3 (-0·1–0·7)	0·5 (-0·1–1·1)
Occupational asthmagens	15·7 (13·9–18·2)	16·4 (14·5–18·5)	15·4 (13·5–17·6)	15·9 (13·9–18·3)	0·0 (-0·4–0·5)	-0·1 (-0·7–0·4)	0·3 (-0·4–1·1)
Occupational particulate matter, gases, and fumes	5·2 (4·0–6·8)	5·2 (4·0–6·9)	5·1 (3·9–6·6)	4·9 (3·8–6·4)	-0·2 (-0·5–0·1)	-0·3 (-0·6–0·0)	-0·3 (-0·7–0·0)
Occupational noise	6·0 (5·5–6·6)	6·1 (5·7–6·7)	6·2 (5·8–6·7)	6·2 (5·8–6·7)	0·1 (-0·1–0·3)	0·0 (-0·2–0·2)	0·0 (-0·3–0·2)
Occupational injuries	--	--	--	--	--	--	--
Occupational ergonomic factors	9·8 (8·1–12·0)	9·4 (8·0–11·4)	8·4 (7·1–10·0)	8·5 (7·1–10·2)	-0·5 (-1·1–0·0)	-0·5 (-1·2–0·1)	0·1 (-0·8–1·0)
**Behavioural risks**	**29·5 (26·2–32·9)**	**27·5 (24·3–31·1)**	**25·9 (22·9–29·8)**	**24·2 (20·9–28·2)**	**-0·6 (-1·0–-0·2)**	**-0·6 (-1·2–-0·1)**	**-0·6 (-1·3–0·1)**
Child and maternal malnutrition	9·5 (6·3–13·6)	8·1 (5·3–11·3)	8·2 (5·7–11·2)	8·4 (5·9–11·5)	-0·4 (-1·3–0·5)	0·1 (-1·1–1·3)	0·2 (-1·5–1·9)
Suboptimal breastfeeding	68·8 (66·0–71·4)	66·7 (64·3–69·7)	63·0 (60·3–65·9)	62·3 (59·7–65·6)	-0·3 (-0·5–-0·2)	-0·3 (-0·5–-0·1)	-0·1 (-0·4–0·2)
Non-exclusive breastfeeding	64·3 (56·3–71·5)	59·8 (52·4–66·5)	49·1 (42·5–56·1)	46·6 (39·7–54·4)	-1·0 (-1·6–-0·5)	-1·2 (-1·9–-0·5)	-0·5 (-1·5–0·5)
Discontinued breastfeeding	88·6 (86·3–91·0)	86·3 (83·8–89·2)	84·4 (81·2–87·6)	84·6 (81·2–88·3)	-0·1 (-0·3–0·0)	-0·1 (-0·3–0·1)	0·0 (-0·3–0·3)
Child growth failure	1·3 (0·4–3·1)	1·2 (0·4–2·7)	1·1 (0·3–2·4)	1·0 (0·3–2·2)	-1·0 (-1·5–-0·6)	-1·1 (-1·6–-0·6)	-0·8 (-1·6–0·0)
Child underweight	2·8 (1·7–3·8)	2·6 (1·6–3·5)	2·2 (1·4–3·0)	2·0 (1·2–2·8)	-1·1 (-1·6–-0·5)	-1·1 (-1·9–-0·5)	-0·7 (-1·8–0·3)
Child wasting	0·9 (0·6–1·4)	0·8 (0·5–1·3)	0·8 (0·5–1·3)	0·8 (0·5–1·2)	-0·3 (-0·7–0·0)	-0·2 (-0·6–0·3)	-0·3 (-1·0–0·4)
Child stunting	3·9 (3·0–4·9)	3·5 (2·8–4·5)	3·1 (2·4–3·9)	2·8 (2·1–3·5)	-1·1 (-1·7–-0·5)	-1·2 (-2·0–-0·4)	-1·0 (-2·1–0·2)
Low birth weight and short gestation	14·0 (12·2–16·0)	15·1 (13·2–17·1)	17·9 (15·6–20·5)	17·2 (15·1–19·6)	0·7 (0·4–0·9)	0·6 (0·2–1·0)	-0·3 (-1·1–0·4)
Short gestation	25·3 (22·1–28·5)	27·2 (23·8–30·9)	31·9 (27·9–36·3)	29·6 (26·0–34·0)	0·5 (0·2–0·8)	0·4 (-0·1–0·9)	-0·7 (-1·6–0·3)
Low birth weight	11·2 (10·2–12·5)	11·8 (10·7–13·0)	13·7 (12·4–15·2)	13·6 (12·3–14·9)	0·6 (0·4–0·9)	0·7 (0·3–1·0)	-0·1 (-0·9–0·7)

Iron deficiency	3·2<br>(2·2–4·5)	2·8<br>(1·9–3·8)	2·8<br>(2·0–3·8)	2·8<br>(2·1–3·8)	-0·4<br>(-1·2–0·5)	0·1<br>(-1·1–1·1)	0·0<br>(-1·6–1·6)
Vitamin A deficiency	1·2<br>(0·0–2·4)	1·5<br>(0·0–3·1)	1·0<br>(0·0–2·1)	0·5<br>(0·0–1·1)	-2·5<br>(-5·0–0·0)	-5·0<br>(-8·0–0·0)	-5·6<br>(-9·7–0·0)
Zinc deficiency	1·4<br>(0·0–6·3)	1·0<br>(0·0–4·2)	0·9<br>(0·0–3·8)	0·9<br>(0·0–3·7)	-1·7<br>(-39·8–4·1)	-0·7<br>(-7·2–6·2)	-0·1<br>(-3·8–7·8)
Tobacco	42·3<br>(40·4–44·1)	35·7<br>(34·2–37·0)	30·7<br>(29·6–31·9)	26·5<br>(24·9–28·3)	-1·5<br>(-1·7–-1·3)	-1·4<br>(-1·7–-1·1)	-1·3<br>(-1·9–-0·8)
Smoking	39·7<br>(36·9–42·0)	33·3<br>(31·1–35·4)	28·1<br>(26·7–29·8)	23·7<br>(21·7–26·0)	-1·7<br>(-2·0–-1·3)	-1·6<br>(-2·1–-1·2)	-1·6<br>(-2·4–-0·8)
Chewing tobacco	2·4<br>(1·9–3·0)	2·8<br>(2·2–3·5)	2·7<br>(2·0–3·5)	2·5<br>(1·8–3·3)	0·1<br>(-1·1–1·3)	-0·6<br>(-2·3–1·0)	-0·7<br>(-3·3–2·0)
Second-hand smoke	34·4<br>(32·6–36·1)	28·9<br>(27·0–30·2)	25·3<br>(23·6–26·5)	22·3<br>(20·3–24·1)	-1·4<br>(-1·7–-1·2)	-1·2<br>(-1·6–-0·9)	-1·1<br>(-1·7–-0·5)
High alcohol use	25·1<br>(16·9–35·9)	24·6<br>(16·9–34·6)	25·1<br>(16·5–35·7)	25·6<br>(17·5–36·2)	0·1<br>(-1·2–1·2)	0·2<br>(-1·5–1·7)	0·2<br>(-2·0–2·1)
Drug use	0·9<br>(0·5–1·6)	0·9<br>(0·6–1·4)	1·5<br>(1·2–1·8)	5·2<br>(3·8–6·7)	5·8<br>(3·0–8·4)	8·4<br>(5·2–10·7)	11·5<br>(7·6–14·3)
Dietary risks	34·9<br>(26·2–45·9)	38·5<br>(29·0–49·8)	41·3<br>(32·1–53·2)	41·3<br>(32·0–53·1)	0·5<br>(0·3–0·9)	0·3<br>(0·0–0·6)	0·0<br>(-0·5–0·4)
Diet low in fruits	34·8<br>(30·2–38·6)	33·5<br>(28·1–36·6)	34·6<br>(29·8–37·7)	35·6<br>(30·9–39·5)	0·1<br>(-0·3–0·4)	0·3<br>(-0·3–0·8)	0·3<br>(-0·6–1·1)
Diet low in vegetables	20·2<br>(11·8–27·9)	24·7<br>(14·7–32·6)	30·0<br>(16·9–37·8)	28·9<br>(17·1–36·7)	1·2<br>(0·6–1·8)	0·8<br>(0·3–1·3)	-0·3<br>(-1·2–0·5)
Diet low in legumes	35·7<br>(0·0–45·1)	37·2<br>(0·0–46·6)	41·0<br>(0·0–51·5)	41·7<br>(0·0–52·8)	0·5<br>(0·0–0·8)	0·5<br>(0·0–1·0)	0·1<br>(-0·6–0·8)
Diet low in whole grains	45·4<br>(38·3–52·2)	48·9<br>(40·8–55·6)	50·3<br>(42·0–57·7)	50·8<br>(42·0–58·3)	0·4<br>(0·1–0·6)	0·2<br>(-0·2–0·5)	0·1<br>(-0·5–0·7)
Diet low in nuts and seeds	9·2<br>(6·3–12·6)	6·3<br>(3·8–9·1)	4·2<br>(2·3–6·4)	4·7<br>(2·4–7·4)	-2·2<br>(-4·3–-0·3)	-1·4<br>(-4·2–1·2)	0·9<br>(-3·2–4·9)
Diet low in milk	30·5<br>(26·1–35·2)	30·7<br>(26·5–35·7)	28·9<br>(24·3–33·1)	28·5<br>(23·9–33·4)	-0·2<br>(-0·7–0·3)	-0·4<br>(-1·0–0·3)	-0·1<br>(-1·1–0·8)
Diet high in red meat	52·6<br>(0·0–71·2)	52·7<br>(0·0–71·3)	53·0<br>(0·0–71·9)	53·0<br>(0·0–72·1)	0·0<br>(-0·5–0·5)	0·0<br>(-0·6–0·6)	0·0<br>(-1·1–1·0)
Diet high in processed meat	40·6<br>(31·8–45·2)	49·6<br>(40·0–55·1)	56·1<br>(44·9–62·7)	54·7<br>(45·4–61·8)	1·0<br>(0·5–1·4)	0·5<br>(-0·1–1·0)	-0·2<br>(-1·1–0·6)
Diet high in sugar-sweetened beverages	42·3<br>(32·1–51·7)	54·0<br>(43·0–63·2)	59·8<br>(47·9–69·2)	59·3<br>(47·6–69·5)	1·1<br>(0·3–1·9)	0·4<br>(-0·5–1·3)	-0·1<br>(-1·3–1·1)
Diet low in fibre	38·3<br>(20·7–45·6)	33·5<br>(18·5–40·6)	29·6<br>(15·7–36·0)	26·1<br>(14·2–33·0)	-1·2<br>(-2·1–-0·4)	-1·2<br>(-2·3–-0·1)	-1·2<br>(-2·7–0·4)
Diet low in calcium	5·8<br>(4·8–7·3)	5·9<br>(4·8–7·3)	6·2<br>(5·1–7·6)	6·1<br>(5·0–7·4)	0·1<br>(-0·1–0·3)	0·1<br>(-0·1–0·4)	-0·2<br>(-0·6–0·1)
Diet low in seafood omega-3 fatty acids	29·6<br>(21·0–38·7)	40·5<br>(31·1–50·7)	42·7<br>(32·6–53·0)	38·2<br>(28·3–48·4)	0·8<br>(0·3–1·4)	-0·3<br>(-0·9–0·3)	-1·0<br>(-1·8–-0·2)
Diet low in omega-6 polyunsaturated fatty acids	30·4<br>(20·0–39·1)	30·1<br>(20·0–39·0)	22·6<br>(15·9–29·0)	21·2<br>(15·1–27·4)	-1·2<br>(-1·8–-0·6)	-1·7<br>(-2·6–-0·9)	-0·6<br>(-1·6–0·3)
Diet high in trans fatty acids	71·3<br>(63·4–79·1)	71·7<br>(64·0–78·8)	63·5<br>(56·5–71·6)	0·0<br>(0·0–0·0)	-65·8<br>(-66·1–-65·4)	-97·1<br>(-97·5–-96·6)	-184·3<br>(-185·4–-183·2)
Diet high in sodium	23·3<br>(0·5–69·4)	28·3<br>(2·0–76·2)	31·5<br>(3·6–75·7)	32·0<br>(3·4–77·4)	1·0<br>(0·2–7·0)	0·6<br>(-0·1–4·0)	0·1<br>(-2·7–2·0)
Intimate partner violence	31·7<br>(19·6–40·2)	31·6<br>(21·0–39·3)	28·8<br>(19·1–34·7)	27·1<br>(14·8–36·5)	-0·5<br>(-1·8–0·8)	-0·7<br>(-2·5–1·0)	-0·6<br>(-2·7–1·4)
Childhood sexual abuse and bullying	9·2<br>(5·9–14·7)	11·3<br>(7·4–17·8)	12·9<br>(8·4–20·2)	10·9<br>(7·3–16·9)	0·5<br>(-0·1–1·2)	-0·2<br>(-1·0–0·8)	-1·5<br>(-3·0–0·2)
Childhood sexual abuse	9·0<br>(7·6–10·5)	10·3<br>(8·8–12·0)	11·8<br>(10·2–13·8)	11·1<br>(8·7–14·2)	0·7<br>(-0·3–1·6)	0·4<br>(-0·8–1·5)	-0·5<br>(-2·1–1·1)
Bullying victimization	8·0<br>(3·5–16·1)	10·2<br>(4·7–19·9)	11·6<br>(5·2–22·5)	9·1<br>(4·1–17·8)	0·4<br>(-0·4–1·2)	-0·5<br>(-1·6–0·5)	-2·2<br>(-4·1–-0·2)
Unsafe sex	--	--	--	--	--	--	--
Low physical activity	18·2<br>(13·6–23·2)	17·1<br>(13·1–21·9)	17·2<br>(13·4–21·6)	19·5<br>(14·3–25·5)	0·2<br>(-0·7–1·2)	0·6<br>(-0·7–1·9)	1·1<br>(-0·8–2·9)
**Metabolic risks**	**28·7<br>(25·4–32·0)**	**33·0<br>(29·1–36·2)**	**35·1<br>(31·0–38·1)**	**38·6<br>(33·8–41·5)**	**1·0<br>(0·7–1·2)**	**0·7<br>(0·4–1·0)**	**0·9<br>(0·4–1·3)**
High fasting plasma glucose	13·3<br>(10·1–16·1)	16·9<br>(13·0–20·5)	21·0<br>(16·1–25·2)	25·0<br>(19·1–30·2)	2·0<br>(1·3–2·7)	1·9<br>(0·9–2·8)	1·6<br>(0·3–2·8)
High LDL cholesterol	63·7<br>(44·7–86·7)	54·1<br>(37·0–74·3)	48·2<br>(32·5–67·1)	46·8<br>(31·4–65·4)	-1·0<br>(-1·2–-0·8)	-0·7<br>(-0·9–-0·5)	-0·3<br>(-0·5–0·0)
High systolic blood pressure	29·2<br>(19·7–41·1)	24·9<br>(17·4–34·3)	18·7<br>(12·1–26·8)	26·4<br>(16·7–39·0)	-0·3<br>(-1·4–0·6)	0·3<br>(-1·0–1·5)	3·2<br>(0·7–5·6)
High body-mass index	32·6<br>(27·5–36·9)	37·9<br>(32·2–42·0)	40·4<br>(34·4–44·1)	43·3<br>(37·1–47·1)	0·9<br>(0·7–1·2)	0·6<br>(0·3–0·9)	0·6<br>(0·3–1·1)
Low bone mineral density	21·5<br>(15·8–28·6)	19·3<br>(13·2–26·7)	20·0<br>(14·2–26·8)	22·0<br>(16·0–29·1)	0·1<br>(-0·4–0·6)	0·6<br>(-0·1–1·4)	0·8<br>(-0·5–2·3)
Kidney dysfunction	2·9<br>(2·3–3·8)	2·9<br>(2·4–3·9)	2·9<br>(2·3–3·9)	3·0<br>(2·4–3·9)	0·1<br>(0·0–0·2)	0·1<br>(-0·1–0·2)	0·3<br>(0·0–0·7)

Arizona

Risk Names	SEV 1990	SEV 2000	SEV 2010	SEV 2021	Annualised rate of change 1990 to 2021	Annualised rate of change 2000 to 2021	Annualised rate of change 2010 to 2021
**All risk factors**	**26·8 (23·3–30·5)**	**27·1 (23·8–31·0)**	**27·7 (24·4–31·2)**	**27·3 (23·9–30·8)**	**0·1 (-0·3–0·4)**	**0·0 (-0·4–0·4)**	**-0·1 (-0·7–0·4)**
**Environmental/occupational risks**	**19·4 (12·7–29·3)**	**19·4 (12·7–30·3)**	**16·1 (10·4–27·2)**	**14·0 (8·7–24·6)**	**-1·1 (-1·8–-0·4)**	**-1·6 (-2·1–-0·9)**	**-1·2 (-1·8–-0·8)**
Unsafe water, sanitation, and handwashing	2·3 (1·2–3·7)	2·2 (1·0–3·6)	1·6 (0·5–2·7)	1·3 (0·4–2·2)	-2·0 (-4·4–0·0)	-2·7 (-5·8–0·1)	-2·1 (-5·7–1·9)
Unsafe water source	3·2 (1·2–6·3)	3·0 (1·1–6·0)	1·1 (0·4–2·2)	0·9 (0·4–1·7)	-4·2 (-6·8–-1·5)	-6·0 (-9·5–-2·0)	-2·3 (-7·7–3·1)
Unsafe sanitation	7·8 (4·3–12·4)	6·5 (3·5–10·4)	4·3 (2·4–7·1)	3·1 (1·7–5·4)	-3·0 (-5·3–-1·0)	-3·5 (-6·4–-0·9)	-3·0 (-6·5–0·3)
No access to handwashing facility	1·9 (0·6–3·5)	2·0 (0·6–3·7)	1·9 (0·7–3·5)	1·6 (0·5–3·1)	-0·5 (-3·4–2·3)	-1·0 (-5·1–2·9)	-1·6 (-6·9–3·9)
Air pollution	25·3 (7·9–37·1)	23·9 (8·8–30·8)	13·7 (3·8–23·9)	7·8 (1·8–17·5)	-3·8 (-7·3–-1·6)	-5·3 (-10·2–-2·4)	-5·1 (-11·1–-2·5)
Particulate matter pollution	13·7 (5·5–23·0)	11·4 (8·1–15·4)	5·7 (3·1–8·9)	3·5 (1·2–6·4)	-4·4 (-7·9–-1·8)	-5·6 (-9·4–-3·9)	-4·4 (-9·1–-2·4)
Ambient particulate matter pollution	21·2 (8·5–36·6)	17·6 (12·9–23·0)	8·7 (4·6–13·2)	5·3 (1·8–9·6)	-4·5 (-7·9–-1·8)	-5·7 (-9·4–-4·0)	-4·5 (-9·2–-2·4)
Household air pollution from solid fuels	0·0 (0·0–0·2)	0·0 (0·0–0·2)	0·0 (0·0–0·1)	0·0 (0·0–0·0)	-4·3 (-22·8–-5·0)	-4·8 (-22·1–-4·9)	-3·5 (-13·9–-1·6)
Ambient ozone pollution	25·8 (19·1–34·3)	30·7 (23·9–39·0)	27·3 (21·1–35·1)	24·7 (18·2–32·0)	-0·1 (-0·5–0·2)	-1·0 (-1·6–-0·5)	-0·9 (-1·8–0·0)
Ambient nitrogen dioxide pollution	74·5 (0·0–100·0)	76·9 (0·0–100·0)	47·6 (0·0–100·0)	25·0 (0·0–75·4)	-3·5 (-13·5–0·0)	-5·4 (-20·5–0·0)	-5·9 (-25·7–0·0)
Non-optimal temperature	59·9 (49·3–69·8)	68·1 (55·9–78·6)	66·6 (54·4–76·9)	63·9 (51·7–74·3)	0·2 (0·1–0·4)	-0·3 (-0·5–-0·1)	-0·4 (-0·6–-0·2)
High temperature	80·5 (63·8–94·0)	84·0 (66·2–98·3)	84·5 (67·4–98·7)	84·0 (67·1–98·0)	0·1 (0·0–0·3)	0·0 (-0·1–0·2)	-0·1 (-0·2–0·1)
Low temperature	41·8 (37·3–46·5)	39·5 (35·9–43·9)	34·6 (32·1–38·4)	31·1 (28·7–34·7)	-1·0 (-1·2–-0·6)	-1·1 (-1·3–-0·8)	-1·0 (-1·1–-0·7)
Other environmental risks	27·8 (5·6–43·6)	26·1 (5·6–40·4)	23·3 (5·6–37·8)	20·2 (5·6–34·6)	-1·0 (-1·6–0·0)	-1·2 (-1·9–0·0)	-1·3 (-2·2–0·0)
Residential radon	21·8 (0·0–66·4)	21·8 (0·0–66·4)	21·8 (0·0–66·4)	21·8 (0·0–66·4)	0·0 (0·0–0·0)	0·0 (0·0–0·0)	0·0 (0·0–0·0)
Lead exposure	30·5 (0·0–38·1)	27·9 (0·0–34·8)	23·9 (0·0–29·3)	19·4 (0·0–24·1)	-1·5 (-1·9–0·0)	-1·7 (-2·2–0·0)	-1·9 (-2·6–0·0)
Occupational risks	2·7 (2·2–3·6)	2·8 (2·3–3·6)	2·6 (2·1–3·3)	2·6 (2·1–3·4)	-0·2 (-0·4–0·1)	-0·3 (-0·7–0·0)	0·1 (-0·4–0·6)
Occupational carcinogens	0·9 (0·4–1·7)	1·0 (0·4–1·9)	0·9 (0·4–1·8)	0·9 (0·4–1·8)	0·2 (-0·1–0·4)	-0·1 (-0·4–0·3)	0·3 (-0·3–0·8)
Occupational exposure to asbestos	3·7 (3·3–4·1)	3·8 (3·4–4·2)	3·8 (3·4–4·2)	3·6 (2·8–4·6)	-0·1 (-0·9–0·6)	-0·3 (-1·5–0·8)	-0·5 (-2·8–1·7)
Occupational exposure to arsenic	0·4 (0·0–1·1)	0·5 (0·0–1·1)	0·4 (0·0–1·0)	0·4 (0·0–1·0)	-0·1 (-0·6–0·3)	-0·3 (-0·8–0·3)	0·2 (-0·6–1·1)
Occupational exposure to benzene	1·0 (0·1–3·0)	1·1 (0·1–3·3)	1·1 (0·1–3·2)	1·1 (0·1–3·2)	0·3 (0·0–0·6)	0·1 (-0·4–0·5)	0·4 (-0·3–1·0)
Occupational exposure to beryllium	0·0 (0·0–0·0)	0·0 (0·0–0·0)	0·0 (0·0–0·0)	0·0 (0·0–0·0)	0·2 (-0·1–0·4)	0·1 (-0·3–0·4)	0·5 (0·1–1·0)
Occupational exposure to cadmium	0·1 (0·0–0·1)	0·1 (0·1–0·1)	0·1 (0·0–0·1)	0·1 (0·0–0·1)	-0·1 (-0·5–0·3)	-0·2 (-0·7–0·3)	0·3 (-0·5–1·0)
Occupational exposure to chromium	0·1 (0·1–0·1)	0·1 (0·1–0·1)	0·1 (0·1–0·1)	0·1 (0·1–0·1)	0·0 (-0·3–0·3)	-0·2 (-0·6–0·3)	0·3 (-0·3–1·0)
Occupational exposure to diesel engine exhaust	0·7 (0·6–0·7)	0·7 (0·7–0·7)	0·7 (0·6–0·7)	0·7 (0·7–0·8)	0·1 (-0·2–0·5)	0·1 (-0·4–0·6)	0·7 (0·0–1·4)
Occupational exposure to formaldehyde	0·3 (0·2–0·3)	0·3 (0·3–0·3)	0·3 (0·2–0·3)	0·3 (0·2–0·3)	-0·1 (-0·5–0·3)	-0·3 (-0·8–0·2)	0·2 (-0·6–0·9)
Occupational exposure to nickel	0·4 (0·0–1·5)	0·4 (0·0–1·5)	0·3 (0·0–1·3)	0·3 (0·0–1·3)	-0·3 (-0·8–0·2)	-0·4 (-1·1–0·4)	0·2 (-0·8–1·1)
Occupational exposure to polycyclic aromatic hydrocarbons	0·3 (0·3–0·3)	0·3 (0·3–0·3)	0·3 (0·3–0·3)	0·3 (0·3–0·3)	0·0 (-0·3–0·4)	-0·1 (-0·6–0·3)	0·3 (-0·3–1·0)
Occupational exposure to silica	3·4 (0·5–10·9)	3·2 (0·5–10·2)	2·9 (0·5–9·1)	3·0 (0·5–9·8)	-0·4 (-0·9–0·0)	-0·3 (-0·9–0·3)	0·3 (-0·6–1·1)
Occupational exposure to sulphuric acid	0·7 (0·1–2·3)	0·7 (0·1–2·4)	0·6 (0·1–2·1)	0·7 (0·1–2·1)	-0·1 (-0·5–0·3)	-0·2 (-0·8–0·3)	0·3 (-0·5–1·0)
Occupational exposure to trichloroethylene	0·1 (0·1–0·1)	0·1 (0·1–0·1)	0·1 (0·1–0·1)	0·1 (0·1–0·1)	0·1 (-0·2–0·4)	0·0 (-0·4–0·4)	0·5 (-0·1–1·1)
Occupational asthmagens	16·4 (14·3–18·8)	16·6 (14·6–18·7)	15·0 (13·1–17·2)	15·5 (13·6–17·8)	-0·2 (-0·6–0·2)	-0·3 (-0·8–0·1)	0·3 (-0·4–1·0)
Occupational particulate matter, gases, and fumes	6·1 (4·7–7·9)	6·0 (4·6–7·7)	5·6 (4·3–7·3)	5·2 (4·0–6·7)	-0·5 (-0·8–-0·2)	-0·7 (-1·0–-0·3)	-0·7 (-1·1–-0·3)
Occupational noise	6·5 (5·9–7·1)	6·5 (6·0–7·1)	6·4 (5·9–7·0)	6·1 (5·7–6·7)	-0·2 (-0·4–0·0)	-0·3 (-0·5–-0·1)	-0·4 (-0·6–-0·1)
Occupational injuries	--	--	--	--	--	--	--
Occupational ergonomic factors	7·9 (6·5–9·5)	7·9 (6·6–9·6)	6·9 (5·9–8·4)	7·1 (5·9–8·7)	-0·3 (-0·8–0·0)	-0·5 (-1·1–0·0)	0·2 (-0·5–0·9)
**Behavioural risks**	**29·2 (25·7–33·1)**	**26·6 (23·4–30·1)**	**24·9 (21·5–28·3)**	**23·1 (19·9–26·7)**	**-0·8 (-1·1–-0·4)**	**-0·7 (-1·2–-0·2)**	**-0·7 (-1·4–0·0)**
Child and maternal malnutrition	9·4 (6·3–12·9)	8·2 (5·6–11·4)	8·2 (5·7–11·5)	8·4 (5·7–11·8)	-0·3 (-1·2–0·5)	0·1 (-1·0–1·2)	0·2 (-1·6–1·9)
Suboptimal breastfeeding	69·0 (66·5–71·5)	66·9 (64·4–69·7)	63·0 (60·4–65·8)	62·3 (59·6–65·1)	-0·3 (-0·5–-0·2)	-0·3 (-0·5–-0·1)	-0·1 (-0·4–0·2)
Non-exclusive breastfeeding	64·8 (57·0–71·8)	60·2 (53·6–67·3)	49·0 (42·2–56·2)	46·6 (39·9–53·9)	-1·1 (-1·6–-0·6)	-1·2 (-1·9–-0·6)	-0·5 (-1·5–0·5)
Discontinued breastfeeding	88·8 (86·3–91·1)	86·5 (83·6–89·4)	84·6 (81·5–88·0)	84·7 (81·5–87·9)	-0·2 (-0·3–0·0)	-0·1 (-0·3–0·1)	0·0 (-0·3–0·3)
Child growth failure	1·4 (0·4–3·1)	1·3 (0·4–2·9)	1·1 (0·3–2·4)	1·0 (0·3–2·2)	-1·0 (-1·4–-0·7)	-1·2 (-1·8–-0·7)	-0·7 (-1·5–0·1)
Child underweight	2·9 (1·9–4·0)	2·7 (1·7–3·7)	2·2 (1·4–3·1)	2·1 (1·3–2·9)	-1·1 (-1·6–-0·6)	-1·3 (-2·0–-0·6)	-0·6 (-1·7–0·4)
Child wasting	0·9 (0·6–1·4)	0·9 (0·6–1·3)	0·8 (0·5–1·3)	0·8 (0·5–1·3)	-0·4 (-0·7–-0·1)	-0·3 (-0·7–0·2)	-0·3 (-0·9–0·4)
Child stunting	3·9 (3·1–5·0)	3·7 (2·9–4·6)	3·1 (2·4–3·9)	2·8 (2·2–3·5)	-1·1 (-1·7–-0·6)	-1·3 (-2·2–-0·6)	-0·9 (-2·1–0·3)
Low birth weight and short gestation	17·7 (15·4–20·3)	18·5 (15·9–21·1)	19·1 (16·7–22·1)	19·6 (17·0–22·6)	0·3 (0·1–0·6)	0·3 (-0·1–0·7)	0·2 (-0·6–0·9)
Short gestation	29·5 (25·3–34·5)	31·4 (26·9–36·5)	32·0 (27·5–37·0)	32·6 (28·0–37·8)	0·3 (0·0–0·7)	0·2 (-0·3–0·7)	0·2 (-0·7–1·1)
Low birth weight	13·6 (12·2–15·0)	13·9 (12·4–15·4)	14·5 (13·0–16·1)	14·9 (13·3–16·6)	0·3 (0·0–0·6)	0·3 (-0·1–0·7)	0·2 (-0·5–0·9)

Iron deficiency	3·3 (2·3–4·5)	2·9 (2·0–3·9)	2·9 (2·1–4·0)	3·0 (2·1–4·1)	-0·3 (-1·1–0·5)	0·2 (-0·8–1·3)	0·3 (-1·4–1·8)
Vitamin A deficiency	1·3 (0·0–2·5)	1·9 (0·0–4·0)	1·1 (0·0–2·2)	0·6 (0·0–1·3)	-2·4 (-4·8–0·0)	-5·4 (-8·3–0·0)	-5·0 (-9·1–0·0)
Zinc deficiency	1·9 (0·0–8·1)	1·2 (0·0–5·1)	1·1 (0·0–4·7)	1·1 (0·0–4·6)	-1·7 (-41·9–0·0)	-0·3 (-2·4–3·7)	0·5 (-2·9–10·4)
Tobacco	40·6 (39·0–42·3)	34·5 (33·2–35·8)	27·5 (26·4–28·5)	23·7 (22·1–25·3)	-1·7 (-2·0–-1·5)	-1·8 (-2·1–-1·5)	-1·4 (-2·0–-0·8)
Smoking	36·3 (34·0–38·7)	30·6 (28·6–32·3)	24·8 (23·3–26·3)	20·6 (18·8–22·6)	-1·8 (-2·2–-1·5)	-1·9 (-2·3–-1·4)	-1·7 (-2·4–-0·9)
Chewing tobacco	1·4 (1·1–1·9)	1·5 (1·1–2·0)	1·6 (1·1–2·1)	1·6 (1·1–2·1)	0·2 (-1·1–1·6)	0·2 (-1·5–2·0)	0·0 (-2·7–2·9)
Second-hand smoke	36·2 (34·3–37·9)	31·7 (29·7–33·2)	26·1 (24·1–27·4)	23·0 (21·0–24·9)	-1·5 (-1·7–-1·2)	-1·5 (-1·9–-1·1)	-1·1 (-1·8–-0·5)
High alcohol use	25·7 (17·7–36·7)	23·6 (16·3–33·7)	25·1 (17·7–34·1)	25·5 (17·3–35·2)	0·0 (-1·2–1·0)	0·4 (-1·2–1·7)	0·1 (-1·9–1·9)
Drug use	1·1 (0·7–2·0)	1·8 (1·6–2·1)	2·0 (1·7–2·3)	5·2 (3·8–6·5)	4·9 (2·3–7·1)	5·1 (3·6–6·0)	9·0 (5·9–10·6)
Dietary risks	33·8 (25·1–45·1)	34·5 (25·1–45·8)	36·9 (27·5–49·2)	37·0 (27·7–49·6)	0·3 (0·0–0·6)	0·3 (0·0–0·7)	0·0 (-0·5–0·5)
Diet low in fruits	35·4 (30·7–38·6)	30·9 (26·5–33·6)	30·2 (26·4–33·0)	32·5 (27·7–36·4)	-0·3 (-0·7–0·2)	0·2 (-0·3–0·8)	0·7 (-0·4–1·5)
Diet low in vegetables	17·4 (10·7–25·0)	11·3 (6·5–16·1)	13·4 (7·8–17·4)	14·3 (8·7–19·3)	-0·6 (-1·3–0·2)	1·1 (0·1–2·5)	0·6 (-0·7–1·8)
Diet low in legumes	38·4 (0·0–48·4)	39·2 (0·0–48·7)	43·4 (0·0–54·0)	44·3 (0·0–55·6)	0·5 (0·0–0·8)	0·6 (0·0–1·1)	0·2 (-0·5–0·9)
Diet low in whole grains	45·0 (37·3–50·9)	48·1 (39·2–54·4)	50·3 (41·3–57·4)	50·2 (41·6–57·3)	0·4 (0·1–0·6)	0·2 (-0·1–0·5)	0·0 (-0·5–0·5)
Diet low in nuts and seeds	13·2 (9·9–17·2)	8·4 (5·7–11·6)	6·1 (3·8–9·0)	6·9 (3·9–10·4)	-2·1 (-3·7–-0·6)	-1·0 (-3·3–1·2)	1·1 (-2·4–4·2)
Diet low in milk	36·7 (32·4–41·3)	34·6 (30·3–39·2)	33·2 (29·1–38·0)	33·6 (29·0–38·6)	-0·3 (-0·8–0·1)	-0·1 (-0·8–0·4)	0·1 (-0·8–0·9)
Diet high in red meat	48·7 (0·0–67·2)	49·1 (0·0–67·3)	49·9 (0·0–69·0)	49·3 (0·0–68·1)	0·0 (-0·5–0·5)	0·0 (-0·6–0·7)	-0·1 (-1·0–0·9)
Diet high in processed meat	41·6 (32·7–46·1)	50·5 (40·3–56·4)	56·9 (46·0–63·4)	54·5 (43·7–62·0)	0·9 (0·4–1·3)	0·4 (-0·2–0·9)	-0·4 (-1·2–0·4)
Diet high in sugar-sweetened beverages	35·5 (27·1–43·5)	48·1 (37·8–56·7)	53·8 (43·3–63·4)	51·7 (40·6–61·9)	1·2 (0·4–2·1)	0·3 (-0·6–1·3)	-0·4 (-1·7–1·0)
Diet low in fibre	40·1 (22·5–47·5)	31·5 (17·6–38·5)	28·2 (15·1–34·6)	25·3 (13·9–32·2)	-1·5 (-2·3–-0·7)	-1·0 (-2·2–0·1)	-1·0 (-2·7–0·7)
Diet low in calcium	5·7 (4·7–7·2)	4·9 (3·9–6·1)	5·3 (4·2–6·5)	5·4 (4·3–6·6)	-0·2 (-0·5–0·0)	0·4 (0·1–0·7)	0·2 (-0·2–0·6)
Diet low in seafood omega-3 fatty acids	37·0 (27·6–47·2)	45·0 (35·0–55·7)	48·2 (37·7–58·5)	44·2 (34·1–55·6)	0·6 (0·1–1·1)	-0·1 (-0·6–0·5)	-0·8 (-1·5–-0·1)
Diet low in omega-6 polyunsaturated fatty acids	36·7 (23·9–47·1)	33·0 (22·4–42·7)	24·8 (17·1–32·4)	23·7 (15·9–31·1)	-1·4 (-2·1–-0·7)	-1·6 (-2·4–-0·8)	-0·4 (-1·4–0·4)
Diet high in trans fatty acids	71·4 (63·4–79·3)	71·9 (64·9–78·9)	63·2 (53·8–70·9)	0·0 (0·0–0·0)	-65·8 (-66·1–-65·4)	-97·1 (-97·6–-96·6)	-184·2 (-185·3–-182·7)
Diet high in sodium	23·1 (0·6–68·8)	27·9 (1·8–74·8)	30·9 (3·6–74·8)	31·2 (3·4–77·2)	1·0 (0·2–6·8)	0·5 (-0·2–4·1)	0·1 (-2·6–2·2)
Intimate partner violence	23·2 (13·5–30·8)	22·7 (13·4–28·9)	20·9 (13·3–26·6)	21·4 (11·8–28·6)	-0·3 (-1·8–1·1)	-0·3 (-2·2–1·3)	0·2 (-1·4–1·6)
Childhood sexual abuse and bullying	9·3 (5·9–14·5)	11·2 (7·3–17·3)	12·8 (8·4–20·2)	9·7 (6·7–14·4)	0·1 (-0·4–0·7)	-0·7 (-1·6–0·1)	-2·6 (-4·1–-1·1)
Childhood sexual abuse	9·2 (7·8–10·7)	10·3 (8·9–11·9)	12·0 (10·5–13·7)	10·9 (8·9–13·3)	0·5 (-0·2–1·2)	0·2 (-0·7–1·2)	-0·9 (-2·4–0·4)
Bullying victimization	8·0 (3·5–16·1)	9·9 (4·6–19·4)	11·3 (5·1–22·0)	7·3 (3·4–14·1)	-0·3 (-1·0–0·4)	-1·5 (-2·5–-0·5)	-3·9 (-5·8–-2·2)
Unsafe sex	--	--	--	--	--	--	--
Low physical activity	19·3 (14·3–24·8)	18·3 (14·0–23·3)	19·4 (15·2–24·0)	21·7 (16·3–27·7)	0·4 (-0·5–1·4)	0·8 (-0·4–2·0)	1·1 (-0·9–2·9)
**Metabolic risks**	**23·3 (20·1–26·2)**	**28·6 (25·0–31·7)**	**33·2 (29·2–36·0)**	**37·1 (32·3–40·2)**	**1·5 (1·2–1·8)**	**1·2 (0·9–1·6)**	**1·0 (0·5–1·5)**
High fasting plasma glucose	12·3 (9·4–14·9)	15·8 (12·2–18·8)	20·7 (15·8–24·8)	23·9 (18·3–28·3)	2·1 (1·5–2·8)	2·0 (1·2–2·9)	1·3 (0·2–2·5)
High LDL cholesterol	63·7 (44·9–87·2)	53·7 (37·0–74·3)	48·2 (32·6–67·5)	46·6 (31·3–65·3)	-1·0 (-1·2–-0·8)	-0·7 (-0·9–-0·5)	-0·3 (-0·6–0·0)
High systolic blood pressure	28·2 (18·8–39·2)	24·1 (16·8–33·4)	18·1 (11·8–26·1)	24·8 (15·7–35·6)	-0·4 (-1·3–0·5)	0·1 (-1·1–1·3)	2·9 (0·6–5·1)
High body-mass index	26·4 (22·4–30·4)	32·8 (27·8–36·9)	38·1 (32·3–42·0)	42·0 (35·2–45·9)	1·5 (1·2–1·8)	1·2 (0·8–1·5)	0·9 (0·4–1·3)
Low bone mineral density	21·7 (15·7–28·5)	19·7 (13·8–26·5)	20·3 (14·5–27·3)	22·3 (16·2–29·3)	0·1 (-0·4–0·5)	0·6 (-0·2–1·4)	0·9 (-0·6–2·3)
Kidney dysfunction	2·9 (2·3–3·8)	2·9 (2·4–3·9)	2·9 (2·3–3·8)	3·0 (2·4–4·0)	0·2 (0·1–0·3)	0·1 (0·0–0·2)	0·4 (0·1–0·7)

Arkansas

Risk Names	SEV 1990	SEV 2000	SEV 2010	SEV 2021	Annualised rate of change 1990 to 2021	Annualised rate of change 2000 to 2021	Annualised rate of change 2010 to 2021
**All risk factors**	**26·5 (23·6–29·8)**	**26·4 (23·5–29·3)**	**26·8 (24·1–29·7)**	**26·9 (24·1–30·0)**	**0·0 (-0·2–0·3)**	**0·1 (-0·2–0·4)**	**0·0 (-0·4–0·5)**
**Environmental/occupational risks**	**18·1 (12·2–28·3)**	**18·1 (12·7–28·0)**	**16·1 (10·7–25·2)**	**13·4 (8·9–20·4)**	**-1·0 (-1·7–-0·3)**	**-1·4 (-1·9–-0·9)**	**-1·6 (-2·2–-1·2)**
Unsafe water, sanitation, and handwashing	2·7 (1·4–4·4)	2·4 (1·2–3·8)	1·7 (0·6–2·9)	1·3 (0·5–2·3)	-2·2 (-4·5–0·2)	-2·7 (-5·7–0·2)	-2·1 (-5·8–1·4)
Unsafe water source	3·7 (1·4–8·0)	3·4 (1·2–6·7)	1·3 (0·5–2·6)	1·0 (0·4–2·0)	-4·3 (-7·1–-1·4)	-5·8 (-9·7–-1·7)	-2·3 (-8·0–3·0)
Unsafe sanitation	9·8 (5·6–15·3)	7·7 (4·2–12·4)	5·5 (2·9–9·5)	4·0 (2·2–7·2)	-2·8 (-5·1–-0·9)	-3·0 (-5·9–-0·7)	-2·8 (-6·2–0·5)
No access to handwashing facility	1·9 (0·6–3·7)	1·9 (0·7–3·7)	1·9 (0·7–3·6)	1·6 (0·6–3·2)	-0·6 (-3·6–2·7)	-1·0 (-4·5–3·4)	-1·7 (-7·0–3·3)
Air pollution	17·3 (6·9–28·6)	16·1 (9·1–25·6)	10·2 (5·6–18·0)	6·2 (2·4–13·6)	-3·3 (-5·8–-1·5)	-4·6 (-7·2–-3·0)	-4·6 (-8·4–-2·5)
Particulate matter pollution	12·8 (4·4–23·0)	12·2 (8·7–16·2)	8·5 (5·3–12·1)	5·0 (2·2–8·3)	-3·0 (-5·8–-0·3)	-4·2 (-6·7–-2·9)	-4·8 (-8·8–-2·8)
Ambient particulate matter pollution	19·8 (6·9–35·5)	18·8 (13·9–24·4)	13·0 (8·4–18·1)	7·6 (3·4–12·3)	-3·1 (-5·9–-0·3)	-4·3 (-6·7–-3·0)	-4·8 (-8·8–-2·9)
Household air pollution from solid fuels	0·0 (0·0–0·2)	0·0 (0·0–0·1)	0·0 (0·0–0·0)	0·0 (0·0–0·0)	-4·6 (-29·1–-6·0)	-4·6 (-27·4–-5·8)	-3·9 (-24·5–-5·0)
Ambient ozone pollution	20·8 (15·3–28·2)	27·5 (21·3–35·3)	18·2 (12·9–24·7)	9·1 (5·0–14·3)	-2·7 (-3·7–-1·9)	-5·2 (-7·1–-4·0)	-6·2 (-9·1–-4·5)
Ambient nitrogen dioxide pollution	33·5 (0·0–87·7)	28·6 (0·0–78·8)	14·4 (0·0–54·4)	9·5 (0·0–46·8)	-4·1 (-14·7–0·0)	-5·2 (-19·3–0·0)	-3·7 (-16·3–0·0)
Non-optimal temperature	32·0 (25·0–42·3)	35·0 (27·8–45·5)	37·7 (29·6–48·5)	30·1 (23·8–39·2)	-0·2 (-0·4–0·0)	-0·7 (-1·1–-0·5)	-2·0 (-2·7–-1·4)
High temperature	32·9 (24·0–42·5)	39·5 (29·7–49·6)	40·9 (30·7–50·7)	28·1 (20·0–37·1)	-0·5 (-0·8–-0·3)	-1·6 (-2·2–-1·1)	-3·4 (-4·4–-2·5)
Low temperature	25·9 (22·9–29·7)	28·1 (25·2–31·6)	29·6 (26·9–32·8)	25·8 (23·0–29·3)	0·0 (-0·2–0·1)	-0·4 (-0·6–-0·3)	-1·2 (-1·6–-0·9)
Other environmental risks	25·9 (5·8–42·1)	24·6 (5·9–40·3)	22·5 (5·8–38·1)	19·8 (5·8–35·8)	-0·9 (-1·5–0·0)	-1·0 (-1·7–0·0)	-1·2 (-2·1–0·0)
Residential radon	23·7 (0·0–73·0)	23·7 (0·0–73·0)	23·7 (0·0–73·0)	23·7 (0·0–73·0)	0·0 (0·0–0·0)	0·0 (0·0–0·0)	0·0 (0·0–0·0)
Lead exposure	26·8 (0·0–33·7)	24·9 (0·0–30·9)	21·8 (0·0–27·1)	17·9 (0·0–22·5)	-1·3 (-1·7–0·0)	-1·6 (-2·1–0·0)	-1·8 (-2·6–0·0)
Occupational risks	2·9 (2·4–3·8)	3·0 (2·5–3·9)	2·7 (2·3–3·6)	2·8 (2·2–3·6)	-0·2 (-0·5–0·1)	-0·4 (-0·8–0·0)	0·1 (-0·4–0·5)
Occupational carcinogens	0·9 (0·4–1·7)	0·9 (0·4–1·8)	0·9 (0·4–1·8)	0·9 (0·4–1·9)	0·2 (-0·1–0·5)	0·1 (-0·4–0·4)	0·4 (-0·2–0·9)
Occupational exposure to asbestos	2·6 (2·3–2·9)	2·7 (2·4–3·0)	2·6 (2·3–2·9)	2·7 (2·1–3·4)	0·1 (-0·8–0·9)	0·0 (-1·2–1·2)	0·3 (-2·1–2·3)
Occupational exposure to arsenic	0·5 (0·0–1·3)	0·6 (0·0–1·4)	0·5 (0·0–1·2)	0·5 (0·0–1·2)	-0·3 (-0·8–0·1)	-0·6 (-1·2–0·1)	0·0 (-0·9–0·9)
Occupational exposure to benzene	1·0 (0·1–2·9)	1·1 (0·1–3·2)	1·1 (0·1–3·2)	1·2 (0·1–3·4)	0·4 (0·0–0·8)	0·2 (-0·3–0·6)	0·5 (-0·1–1·2)
Occupational exposure to beryllium	0·0 (0·0–0·0)	0·0 (0·0–0·0)	0·0 (0·0–0·0)	0·0 (0·0–0·0)	0·2 (-0·1–0·4)	0·0 (-0·3–0·4)	0·5 (0·0–0·9)
Occupational exposure to cadmium	0·1 (0·1–0·1)	0·1 (0·1–0·1)	0·1 (0·1–0·1)	0·1 (0·1–0·1)	-0·2 (-0·7–0·2)	-0·5 (-1·0–0·1)	0·1 (-0·7–0·9)
Occupational exposure to chromium	0·2 (0·1–0·2)	0·2 (0·1–0·2)	0·1 (0·1–0·2)	0·1 (0·1–0·2)	-0·1 (-0·5–0·2)	-0·3 (-0·9–0·2)	0·2 (-0·5–0·9)
Occupational exposure to diesel engine exhaust	0·6 (0·6–0·7)	0·7 (0·6–0·7)	0·6 (0·6–0·7)	0·7 (0·6–0·8)	0·3 (-0·1–0·6)	0·3 (-0·2–0·8)	0·8 (0·1–1·5)
Occupational exposure to formaldehyde	0·3 (0·3–0·3)	0·3 (0·3–0·3)	0·3 (0·3–0·3)	0·3 (0·3–0·3)	-0·3 (-0·7–0·1)	-0·5 (-1·1–0·0)	0·0 (-0·8–0·8)
Occupational exposure to nickel	0·4 (0·0–1·7)	0·4 (0·0–1·7)	0·4 (0·0–1·6)	0·4 (0·0–1·6)	-0·4 (-0·9–0·0)	-0·6 (-1·4–0·1)	0·0 (-1·1–1·1)
Occupational exposure to polycyclic aromatic hydrocarbons	0·3 (0·3–0·3)	0·3 (0·3–0·3)	0·3 (0·3–0·3)	0·3 (0·3–0·3)	-0·1 (-0·4–0·3)	-0·3 (-0·8–0·2)	0·2 (-0·4–0·9)
Occupational exposure to silica	3·9 (0·6–13·0)	3·8 (0·6–12·7)	3·4 (0·5–11·1)	3·4 (0·6–11·1)	-0·4 (-0·9–0·0)	-0·5 (-1·1–0·1)	0·1 (-0·8–1·1)
Occupational exposure to sulphuric acid	0·8 (0·2–2·7)	0·8 (0·2–2·7)	0·7 (0·2–2·4)	0·7 (0·1–2·4)	-0·2 (-0·7–0·2)	-0·5 (-1·1–0·1)	0·1 (-0·8–0·9)
Occupational exposure to trichloroethylene	0·1 (0·1–0·1)	0·1 (0·1–0·1)	0·1 (0·1–0·1)	0·1 (0·1–0·1)	0·0 (-0·3–0·3)	-0·1 (-0·6–0·3)	0·4 (-0·2–1·0)
Occupational asthmagens	17·4 (14·9–20·0)	17·9 (15·7–20·3)	16·0 (14·2–18·1)	16·4 (14·1–18·7)	-0·2 (-0·6–0·1)	-0·4 (-1·0–0·0)	0·2 (-0·6–0·9)
Occupational particulate matter, gases, and fumes	7·2 (5·6–9·5)	7·1 (5·5–9·4)	6·7 (5·1–8·8)	6·1 (4·7–7·9)	-0·5 (-0·8–-0·3)	-0·7 (-1·0–-0·4)	-0·8 (-1·2–-0·4)
Occupational noise	7·0 (6·5–7·7)	7·1 (6·5–7·7)	6·9 (6·5–7·5)	6·6 (6·2–7·2)	-0·2 (-0·3–0·0)	-0·3 (-0·5–-0·1)	-0·4 (-0·6–-0·2)
Occupational injuries	--	--	--	--	--	--	--
Occupational ergonomic factors	8·7 (7·2–10·6)	8·6 (7·2–10·4)	7·5 (6·3–9·0)	7·7 (6·4–9·3)	-0·4 (-1·0–0·1)	-0·5 (-1·2–0·0)	0·3 (-0·5–1·0)
**Behavioural risks**	**29·8 (26·7–33·2)**	**28·3 (25·4–31·6)**	**27·2 (24·0–30·4)**	**26·0 (22·9–29·3)**	**-0·4 (-0·8–-0·1)**	**-0·4 (-0·8–0·0)**	**-0·4 (-1·1–0·2)**
Child and maternal malnutrition	10·5 (7·4–14·5)	9·1 (6·3–12·7)	9·3 (6·5–12·7)	9·5 (6·3–13·2)	-0·3 (-1·2–0·5)	0·2 (-1·0–1·2)	0·2 (-1·6–2·1)
Suboptimal breastfeeding	69·0 (66·7–71·7)	66·9 (64·4–69·6)	63·1 (60·6–66·0)	62·4 (59·4–65·3)	-0·3 (-0·5–-0·2)	-0·3 (-0·5–-0·1)	-0·1 (-0·4–0·2)
Non-exclusive breastfeeding	64·9 (56·8–72·2)	60·2 (52·2–67·4)	49·3 (41·7–56·2)	46·9 (39·5–53·5)	-1·0 (-1·6–-0·5)	-1·2 (-1·9–-0·6)	-0·4 (-1·4–0·5)
Discontinued breastfeeding	88·8 (86·8–91·3)	86·5 (83·7–89·4)	84·6 (81·7–88·0)	84·8 (81·4–88·3)	-0·2 (-0·3–0·0)	-0·1 (-0·3–0·1)	0·0 (-0·3–0·3)
Child growth failure	1·5 (0·5–3·4)	1·4 (0·5–3·1)	1·2 (0·4–2·7)	1·1 (0·4–2·4)	-0·9 (-1·3–-0·5)	-1·0 (-1·5–-0·5)	-0·7 (-1·4–0·1)
Child underweight	3·2 (2·0–4·4)	3·0 (1·9–4·0)	2·6 (1·6–3·5)	2·4 (1·4–3·3)	-0·9 (-1·5–-0·4)	-1·0 (-1·7–-0·4)	-0·6 (-1·6–0·4)
Child wasting	1·0 (0·6–1·5)	0·9 (0·6–1·4)	0·9 (0·6–1·4)	0·9 (0·6–1·4)	-0·3 (-0·5–0·0)	-0·1 (-0·6–0·3)	-0·3 (-1·0–0·4)
Child stunting	4·2 (3·2–5·2)	4·0 (3·1–4·9)	3·5 (2·7–4·4)	3·2 (2·4–4·0)	-0·9 (-1·5–-0·4)	-1·1 (-1·9–-0·3)	-0·9 (-2·1–0·4)
Low birth weight and short gestation	24·3 (21·4–27·3)	25·4 (22·1–28·5)	27·3 (23·8–30·8)	26·8 (23·7–30·3)	0·3 (0·1–0·6)	0·3 (-0·1–0·6)	-0·2 (-0·9–0·5)
Short gestation	42·6 (38·2–47·3)	44·5 (39·8–49·4)	46·8 (41·8–51·8)	44·6 (40·5–49·4)	0·1 (-0·1–0·4)	0·0 (-0·4–0·4)	-0·4 (-1·2–0·3)
Low birth weight	18·5 (17·1–20·0)	19·1 (17·7–20·7)	20·6 (19·1–22·2)	20·7 (19·1–22·5)	0·4 (0·1–0·6)	0·4 (0·0–0·7)	0·0 (-0·6–0·7)

Iron deficiency	3·8 (2·7–5·1)	3·3 (2·4–4·5)	3·4 (2·5–4·5)	3·5 (2·4–4·7)	-0·3 (-1·1–0·4)	0·2 (-0·9–1·3)	0·1 (-1·5–1·8)
Vitamin A deficiency	1·6 (0·0–3·5)	2·2 (0·0–4·8)	1·4 (0·0–3·0)	0·8 (0·0–1·8)	-2·2 (-4·5–0·0)	-4·7 (-7·9–0·0)	-4·5 (-8·2–0·0)
Zinc deficiency	2·2 (0·0–9·5)	1·3 (0·0–5·5)	1·1 (0·0–5·0)	1·1 (0·0–4·6)	-2·2 (-48·7–0·0)	-0·7 (-2·5–2·2)	-0·3 (-4·8–2·1)
Tobacco	44·1 (42·4–45·7)	40·1 (38·6–41·4)	35·3 (34·1–36·4)	33·2 (31·1–35·2)	-0·9 (-1·1–-0·7)	-0·9 (-1·2–-0·6)	-0·5 (-1·1–0·0)
Smoking	41·2 (38·4–44·1)	37·1 (34·9–39·0)	32·4 (30·8–34·2)	30·4 (27·7–33·1)	-1·0 (-1·3–-0·7)	-0·9 (-1·4–-0·5)	-0·6 (-1·4–0·1)
Chewing tobacco	3·3 (2·5–4·2)	3·8 (2·9–4·8)	4·0 (3·0–5·3)	4·0 (2·9–5·5)	0·7 (-0·7–2·0)	0·3 (-1·5–1·9)	0·0 (-2·7–2·3)
Second-hand smoke	35·2 (33·3–36·5)	32·1 (30·3–33·3)	28·3 (26·4–29·4)	26·8 (24·6–28·5)	-0·9 (-1·1–-0·7)	-0·9 (-1·2–-0·5)	-0·5 (-1·1–0·1)
High alcohol use	22·1 (15·0–31·7)	20·7 (13·9–30·0)	22·0 (15·7–30·7)	22·3 (15·0–31·7)	0·0 (-1·0–1·2)	0·4 (-1·1–1·9)	0·1 (-1·9–2·2)
Drug use	0·8 (0·2–1·8)	1·1 (0·8–1·5)	1·2 (0·9–1·7)	4·2 (3·1–5·2)	5·5 (2·1–9·8)	6·6 (3·8–8·4)	11·3 (6·5–14·5)
Dietary risks	37·6 (28·8–48·7)	41·2 (31·9–53·3)	44·5 (35·4–56·7)	44·0 (34·4–56·7)	0·5 (0·3–0·8)	0·3 (0·0–0·6)	-0·1 (-0·6–0·3)
Diet low in fruits	39·7 (33·6–43·7)	38·3 (33·0–41·4)	39·6 (34·8–42·6)	39·4 (33·6–43·7)	0·0 (-0·3–0·4)	0·1 (-0·3–0·6)	0·0 (-0·8–0·7)
Diet low in vegetables	27·6 (16·8–35·5)	30·5 (17·7–38·4)	36·5 (20·8–44·1)	35·4 (21·3–43·9)	0·8 (0·4–1·2)	0·7 (0·3–1·1)	-0·3 (-1·0–0·4)
Diet low in legumes	39·9 (0·0–50·3)	40·2 (0·0–49·9)	44·3 (0·0–55·5)	44·5 (0·0–56·2)	0·4 (0·0–0·7)	0·5 (0·0–1·0)	0·0 (-0·7–0·8)
Diet low in whole grains	45·7 (38·0–51·8)	49·5 (41·0–55·6)	51·7 (42·6–58·3)	51·3 (42·2–59·0)	0·4 (0·1–0·6)	0·2 (-0·2–0·5)	-0·1 (-0·6–0·4)
Diet low in nuts and seeds	15·6 (11·4–20·5)	9·6 (6·5–13·3)	7·1 (4·5–10·4)	7·1 (4·4–10·5)	-2·5 (-4·2–-1·1)	-1·4 (-3·7–0·8)	0·0 (-3·2–3·3)
Diet low in milk	39·4 (35·0–44·3)	36·5 (32·0–41·8)	34·8 (30·2–40·2)	34·0 (29·2–39·4)	-0·5 (-1·0–0·0)	-0·3 (-0·9–0·2)	-0·2 (-1·0–0·6)
Diet high in red meat	48·3 (0·0–66·6)	49·0 (0·0–67·9)	49·4 (0·0–68·7)	49·4 (0·0–68·4)	0·1 (-0·4–0·6)	0·0 (-0·6–0·7)	0·0 (-1·0–1·0)
Diet high in processed meat	45·5 (35·9–51·2)	57·5 (46·5–65·4)	65·3 (53·6–73·3)	62·9 (50·5–71·4)	1·0 (0·6–1·5)	0·4 (-0·1–1·0)	-0·3 (-1·2–0·5)
Diet high in sugar-sweetened beverages	32·9 (24·7–40·7)	47·3 (36·9–57·0)	53·3 (42·1–62·9)	52·8 (41·0–62·7)	1·5 (0·7–2·4)	0·5 (-0·5–1·4)	-0·1 (-1·6–1·2)
Diet low in fibre	43·1 (24·1–50·5)	33·6 (18·3–41·3)	30·0 (16·4–37·4)	25·8 (14·3–33·4)	-1·7 (-2·5–-0·8)	-1·3 (-2·4–-0·2)	-1·4 (-3·2–0·3)
Diet low in calcium	6·4 (5·2–8·0)	5·3 (4·3–6·5)	5·7 (4·5–6·9)	5·4 (4·3–6·7)	-0·5 (-0·8–-0·3)	0·1 (-0·2–0·4)	-0·3 (-0·8–0·1)
Diet low in seafood omega-3 fatty acids	53·9 (43·3–65·4)	60·0 (48·7–71·6)	62·6 (51·7–74·2)	57·3 (46·2–68·5)	0·2 (-0·1–0·5)	-0·2 (-0·6–0·1)	-0·8 (-1·3–-0·3)
Diet low in omega-6 polyunsaturated fatty acids	40·6 (25·7–50·9)	35·4 (23·3–45·0)	26·5 (18·4–34·7)	24·0 (16·5–31·1)	-1·7 (-2·4–-0·9)	-1·9 (-2·7–-1·0)	-0·9 (-1·9–0·0)
Diet high in trans fatty acids	71·4 (62·6–78·7)	71·9 (64·1–79·0)	63·3 (54·8–70·5)	0·0 (0·0–0·0)	-65·8 (-66·1–-65·3)	-97·1 (-97·6–-96·6)	-184·2 (-185·2–-182·9)
Diet high in sodium	23·7 (0·6–70·0)	28·5 (1·7–77·2)	31·8 (3·3–77·3)	32·2 (3·3–78·6)	1·0 (0·2–6·1)	0·6 (0·0–3·9)	0·1 (-2·8–2·1)
Intimate partner violence	30·2 (18·8–39·2)	30·3 (19·7–37·9)	27·5 (17·9–34·5)	26·1 (13·9–35·4)	-0·5 (-1·9–0·7)	-0·7 (-2·5–0·9)	-0·5 (-2·9–1·4)
Childhood sexual abuse and bullying	8·8 (5·7–14·0)	10·4 (6·7–16·4)	11·9 (7·6–18·8)	9·0 (6·1–13·8)	0·1 (-0·5–0·7)	-0·7 (-1·6–0·1)	-2·5 (-4·0–-1·1)
Childhood sexual abuse	8·1 (6·6–9·7)	8·7 (7·4–10·0)	10·0 (8·9–11·3)	9·1 (7·5–11·0)	0·4 (-0·4–1·2)	0·2 (-0·8–1·3)	-0·9 (-2·3–0·5)
Bullying victimization	7·9 (3·5–15·8)	9·9 (4·5–19·1)	11·2 (5·1–21·5)	7·5 (3·4–14·4)	-0·2 (-0·9–0·5)	-1·3 (-2·3–-0·4)	-3·6 (-5·5–-1·8)
Unsafe sex	--	--	--	--	--	--	--
Low physical activity	19·5 (14·9–25·2)	19·2 (14·8–24·1)	20·8 (16·2–25·7)	23·2 (17·5–29·2)	0·6 (-0·4–1·5)	0·9 (-0·4–2·2)	1·0 (-0·8–2·5)
**Metabolic risks**	**27·2 (23·7–30·4)**	**32·7 (28·7–35·9)**	**37·7 (33·1–40·9)**	**40·9 (36·0–43·8)**	**1·3 (1·0–1·6)**	**1·1 (0·7–1·4)**	**0·7 (0·3–1·3)**
High fasting plasma glucose	12·7 (9·5–15·5)	17·0 (13·0–20·4)	22·3 (16·6–26·7)	25·7 (19·3–30·5)	2·3 (1·6–2·9)	2·0 (1·1–2·8)	1·3 (0·1–2·5)
High LDL cholesterol	63·1 (44·5–86·0)	53·6 (37·0–74·0)	48·2 (32·6–67·1)	46·7 (31·2–65·5)	-1·0 (-1·2–-0·8)	-0·7 (-0·9–-0·5)	-0·3 (-0·6–0·0)
High systolic blood pressure	30·5 (21·0–42·8)	26·7 (19·1–36·4)	20·6 (13·6–29·6)	28·8 (18·2–42·1)	-0·2 (-1·1–0·7)	0·4 (-0·8–1·5)	3·0 (0·9–5·6)
High body-mass index	30·9 (26·0–35·3)	37·7 (31·7–41·6)	43·4 (37·3–47·3)	46·6 (40·0–50·5)	1·3 (1·0–1·6)	1·0 (0·7–1·3)	0·6 (0·2–1·1)
Low bone mineral density	22·0 (16·0–29·0)	19·9 (13·9–26·5)	20·6 (14·9–27·2)	22·8 (16·6–29·8)	0·1 (-0·4–0·7)	0·7 (0·0–1·4)	0·9 (-0·5–2·4)
Kidney dysfunction	3·0 (2·4–3·9)	3·1 (2·5–4·1)	3·1 (2·5–4·1)	3·2 (2·6–4·2)	0·2 (0·1–0·3)	0·2 (0·0–0·3)	0·4 (0·1–0·6)

California

Risk Names	SEV 1990	SEV 2000	SEV 2010	SEV 2021	Annualised rate of change 1990 to 2021	Annualised rate of change 2000 to 2021	Annualised rate of change 2010 to 2021
**All risk factors**	**26·4 (23·6–29·4)**	**27·0 (24·1–30·1)**	**27·1 (24·4–30·2)**	**26·3 (23·4–29·5)**	**0·0 (-0·3–0·2)**	**-0·1 (-0·5–0·2)**	**-0·3 (-0·8–0·2)**
**Environmental/occupational risks**	**19·7 (13·5–29·6)**	**19·0 (13·0–28·6)**	**16·3 (10·7–25·2)**	**13·9 (9·2–21·6)**	**-1·1 (-1·8–-0·4)**	**-1·5 (-1·9–-0·9)**	**-1·4 (-1·8–-1·1)**
Unsafe water, sanitation, and handwashing	2·1 (1·2–3·3)	1·8 (0·8–2·9)	1·3 (0·4–2·2)	1·1 (0·3–1·8)	-2·2 (-4·6–-0·1)	-2·5 (-5·4–0·5)	-2·2 (-5·9–1·6)
Unsafe water source	2·9 (1·1–5·7)	2·5 (1·0–4·7)	1·0 (0·4–1·8)	0·8 (0·3–1·5)	-4·3 (-6·9–-1·7)	-5·6 (-9·1–-2·0)	-2·3 (-7·1–2·4)
Unsafe sanitation	6·9 (4·1–10·9)	4·9 (2·7–8·0)	3·5 (1·9–6·0)	2·5 (1·4–4·2)	-3·3 (-5·3–-1·2)	-3·2 (-5·6–-0·8)	-3·1 (-6·2–0·1)
No access to handwashing facility	1·7 (0·5–3·4)	1·7 (0·5–3·0)	1·7 (0·6–3·0)	1·4 (0·5–2·5)	-0·7 (-3·4–2·3)	-1·0 (-4·6–3·1)	-1·7 (-7·2–3·9)
Air pollution	29·0 (10·0–40·2)	27·7 (11·4–33·8)	19·1 (6·6–27·8)	10·1 (4·1–19·5)	-3·4 (-5·8–-1·4)	-4·8 (-8·2–-2·3)	-5·8 (-9·6–-2·4)
Particulate matter pollution	16·2 (7·1–26·8)	14·8 (11·1–19·1)	9·2 (6·2–12·8)	6·3 (3·6–9·5)	-3·0 (-4·9–-0·9)	-4·0 (-5·5–-3·1)	-3·4 (-5·2–-2·4)
Ambient particulate matter pollution	25·5 (11·0–42·2)	23·0 (18·0–28·7)	14·1 (9·8–19·0)	9·7 (5·7–14·3)	-3·1 (-5·1–-0·9)	-4·1 (-5·6–-3·2)	-3·4 (-5·2–-2·4)
Household air pollution from solid fuels	0·0 (0·0–0·0)	0·0 (0·0–0·0)	0·0 (0·0–0·0)	0·0 (0·0–0·0)	-2·0 (-36·5–-5·8)	-2·9 (-41·5–-7·1)	-5·2 (-35·8–-6·2)
Ambient ozone pollution	32·5 (26·6–39·3)	24·1 (18·4–31·1)	22·6 (16·7–29·3)	24·2 (17·9–31·5)	-1·0 (-1·3–-0·6)	0·0 (-0·3–0·3)	0·6 (0·0–1·1)
Ambient nitrogen dioxide pollution	82·0 (0·0–100·0)	82·7 (0·0–100·0)	61·7 (0·0–100·0)	23·7 (0·0–72·5)	-4·0 (-13·9–0·0)	-6·0 (-20·9–0·0)	-8·7 (-29·7–0·0)
Non-optimal temperature	24·3 (20·3–30·5)	24·0 (20·2–29·8)	25·0 (21·2–30·8)	26·2 (21·8–32·8)	0·2 (0·0–0·5)	0·4 (0·1–0·8)	0·4 (-0·2–1·2)
High temperature	34·0 (24·2–45·7)	31·9 (22·7–43·9)	36·3 (27·3–47·3)	46·8 (35·6–58·9)	1·0 (0·6–1·5)	1·8 (1·3–2·5)	2·3 (1·5–3·3)
Low temperature	21·3 (18·4–26·4)	21·7 (18·7–26·4)	22·4 (19·2–27·2)	20·7 (17·7–25·3)	-0·1 (-0·2–0·0)	-0·2 (-0·3–-0·1)	-0·7 (-0·9–-0·5)
Other environmental risks	30·6 (7·8–50·0)	28·4 (8·0–48·2)	25·0 (7·8–45·0)	21·5 (7·9–40·4)	-1·1 (-1·8–0·0)	-1·3 (-2·1–0·0)	-1·4 (-2·4–0·0)
Residential radon	24·6 (0·0–78·3)	24·6 (0·0–78·3)	24·6 (0·0–78·3)	24·6 (0·0–78·3)	0·0 (0·0–0·0)	0·0 (0·0–0·0)	0·0 (0·0–0·0)
Lead exposure	33·2 (0·0–41·2)	30·0 (0·0–36·9)	25·1 (0·0–31·1)	20·0 (0·0–24·9)	-1·6 (-2·1–0·0)	-1·9 (-2·4–0·0)	-2·1 (-2·8–0·0)
Occupational risks	2·8 (2·3–3·6)	2·8 (2·4–3·7)	2·6 (2·2–3·5)	2·7 (2·2–3·6)	-0·2 (-0·4–0·1)	-0·3 (-0·7–0·0)	0·1 (-0·4–0·5)
Occupational carcinogens	0·9 (0·4–1·7)	1·0 (0·5–1·9)	0·9 (0·4–1·8)	1·0 (0·4–1·9)	0·1 (-0·3–0·3)	-0·2 (-0·8–0·1)	0·1 (-0·6–0·6)
Occupational exposure to asbestos	4·0 (3·6–4·4)	4·3 (3·9–4·6)	3·6 (3·2–3·9)	3·1 (2·5–3·8)	-0·8 (-1·5–-0·1)	-1·5 (-2·6–-0·6)	-1·4 (-3·3–0·4)
Occupational exposure to arsenic	0·5 (0·0–1·1)	0·5 (0·0–1·2)	0·4 (0·0–1·1)	0·4 (0·0–1·1)	-0·3 (-0·7–0·2)	-0·3 (-1·0–0·3)	0·1 (-0·7–1·0)
Occupational exposure to benzene	1·0 (0·1–3·0)	1·1 (0·1–3·3)	1·1 (0·1–3·3)	1·2 (0·1–3·4)	0·3 (0·0–0·6)	0·1 (-0·4–0·5)	0·4 (-0·2–0·9)
Occupational exposure to beryllium	0·0 (0·0–0·0)	0·0 (0·0–0·0)	0·0 (0·0–0·0)	0·0 (0·0–0·0)	0·1 (-0·1–0·4)	0·1 (-0·3–0·4)	0·5 (0·1–0·9)
Occupational exposure to cadmium	0·1 (0·1–0·1)	0·1 (0·1–0·1)	0·1 (0·0–0·1)	0·1 (0·0–0·1)	-0·2 (-0·6–0·2)	-0·3 (-0·8–0·2)	0·2 (-0·5–1·0)
Occupational exposure to chromium	0·1 (0·1–0·2)	0·1 (0·1–0·2)	0·1 (0·1–0·1)	0·1 (0·1–0·1)	-0·1 (-0·4–0·3)	-0·2 (-0·7–0·3)	0·3 (-0·4–0·9)
Occupational exposure to diesel engine exhaust	0·7 (0·6–0·7)	0·7 (0·6–0·7)	0·7 (0·6–0·7)	0·7 (0·7–0·8)	0·2 (-0·2–0·5)	0·2 (-0·2–0·7)	0·7 (0·1–1·3)
Occupational exposure to formaldehyde	0·3 (0·3–0·3)	0·3 (0·3–0·3)	0·3 (0·3–0·3)	0·3 (0·2–0·3)	-0·2 (-0·6–0·2)	-0·3 (-0·9–0·2)	0·1 (-0·7–0·9)
Occupational exposure to nickel	0·4 (0·0–1·5)	0·4 (0·0–1·5)	0·3 (0·0–1·3)	0·3 (0·0–1·4)	-0·4 (-0·8–0·2)	-0·4 (-1·1–0·3)	0·1 (-0·9–1·1)
Occupational exposure to polycyclic aromatic hydrocarbons	0·3 (0·3–0·3)	0·3 (0·3–0·3)	0·3 (0·3–0·3)	0·3 (0·3–0·3)	0·0 (-0·3–0·3)	-0·2 (-0·7–0·2)	0·3 (-0·3–0·9)
Occupational exposure to silica	3·4 (0·5–11·0)	3·2 (0·5–10·3)	2·9 (0·5–9·5)	3·0 (0·5–9·6)	-0·3 (-0·8–0·1)	-0·3 (-1·0–0·3)	0·2 (-0·7–1·1)
Occupational exposure to sulphuric acid	0·7 (0·1–2·3)	0·7 (0·2–2·5)	0·7 (0·1–2·2)	0·7 (0·1–2·2)	-0·2 (-0·6–0·2)	-0·3 (-0·8–0·3)	0·2 (-0·6–0·9)
Occupational exposure to trichloroethylene	0·1 (0·1–0·1)	0·1 (0·1–0·1)	0·1 (0·1–0·1)	0·1 (0·1–0·1)	0·0 (-0·2–0·3)	0·0 (-0·4–0·4)	0·4 (-0·1–1·1)
Occupational asthmagens	16·7 (14·6–19·1)	16·9 (14·9–19·1)	15·5 (13·7–17·5)	16·1 (14·2–18·1)	-0·1 (-0·5–0·2)	-0·2 (-0·7–0·2)	0·3 (-0·4–1·0)
Occupational particulate matter, gases, and fumes	6·2 (4·8–8·2)	6·0 (4·7–8·0)	5·7 (4·4–7·3)	5·2 (4·0–6·8)	-0·6 (-0·8–-0·3)	-0·7 (-1·0–-0·4)	-0·7 (-1·1–-0·4)
Occupational noise	6·4 (5·9–7·0)	6·5 (6·0–7·0)	6·3 (5·9–6·9)	6·1 (5·8–6·6)	-0·2 (-0·3–0·0)	-0·3 (-0·4–-0·1)	-0·3 (-0·5–-0·1)
Occupational injuries	--	--	--	--	--	--	--
Occupational ergonomic factors	7·6 (6·4–9·2)	7·6 (6·3–9·2)	6·8 (5·7–8·2)	7·0 (5·8–8·6)	-0·3 (-0·7–0·1)	-0·4 (-0·8–0·1)	0·3 (-0·4–0·9)
**Behavioural risks**	**28·6 (25·5–32·2)**	**25·9 (22·9–29·8)**	**23·7 (20·9–27·4)**	**22·5 (19·3–26·3)**	**-0·8 (-1·2–-0·4)**	**-0·7 (-1·1–-0·2)**	**-0·5 (-1·2–0·2)**
Child and maternal malnutrition	10·4 (7·0–14·5)	8·7 (6·1–12·0)	8·6 (6·1–11·7)	8·8 (6·1–12·0)	-0·6 (-1·3–0·2)	0·0 (-1·1–1·1)	0·1 (-1·6–1·8)
Suboptimal breastfeeding	69·0 (66·5–71·6)	66·9 (64·6–69·6)	63·0 (60·5–65·7)	62·4 (59·6–65·3)	-0·3 (-0·5–-0·2)	-0·3 (-0·5–-0·2)	-0·1 (-0·4–0·2)
Non-exclusive breastfeeding	64·7 (57·7–72·2)	60·0 (53·6–66·9)	49·0 (42·7–55·5)	46·5 (39·1–53·0)	-1·1 (-1·6–-0·6)	-1·2 (-1·9–-0·7)	-0·5 (-1·4–0·5)
Discontinued breastfeeding	88·9 (87·2–90·9)	86·6 (84·0–89·1)	84·7 (81·8–87·7)	84·9 (82·0–88·1)	-0·1 (-0·3–0·0)	-0·1 (-0·3–0·1)	0·0 (-0·3–0·3)
Child growth failure	1·3 (0·4–3·1)	1·2 (0·4–2·6)	1·0 (0·3–2·2)	0·9 (0·3–2·0)	-1·3 (-1·7–-1·0)	-1·3 (-1·8–-0·9)	-0·9 (-1·6–-0·2)
Child underweight	2·8 (1·8–3·9)	2·5 (1·6–3·4)	2·0 (1·3–2·8)	1·8 (1·2–2·5)	-1·4 (-1·8–-0·8)	-1·4 (-2·0–-0·8)	-0·8 (-1·7–0·1)
Child wasting	0·9 (0·6–1·4)	0·8 (0·5–1·3)	0·8 (0·5–1·2)	0·8 (0·5–1·1)	-0·5 (-0·9–-0·2)	-0·3 (-0·8–0·1)	-0·4 (-1·2–0·2)
Child stunting	3·9 (3·0–4·9)	3·4 (2·7–4·3)	2·9 (2·2–3·6)	2·6 (2·0–3·2)	-1·3 (-1·9–-0·8)	-1·4 (-2·1–-0·7)	-1·1 (-2·2–0·1)
Low birth weight and short gestation	18·6 (16·2–21·0)	20·2 (17·6–22·6)	19·9 (17·5–22·6)	19·7 (17·2–22·2)	0·2 (-0·1–0·4)	-0·1 (-0·5–0·3)	-0·1 (-0·8–0·7)
Short gestation	35·7 (31·8–39·6)	39·5 (35·5–44·0)	35·2 (31·5–39·6)	33·1 (29·1–37·3)	-0·2 (-0·6–0·1)	-0·8 (-1·4–-0·3)	-0·6 (-1·5–0·4)
Low birth weight	13·6 (12·3–14·9)	14·2 (12·9–15·5)	15·2 (13·9–16·6)	15·7 (14·1–17·0)	0·5 (0·2–0·7)	0·5 (0·0–0·9)	0·3 (-0·5–0·9)

Iron deficiency	3·6 (2·5–4·9)	3·0 (2·2–4·1)	3·0 (2·2–4·1)	3·1 (2·1–4·2)	-0·5 (-1·2–0·2)	0·1 (-1·0–1·0)	0·1 (-1·5–1·6)
Vitamin A deficiency	1·1 (0·0–2·2)	1·4 (0·0–2·8)	0·8 (0·0–1·6)	0·5 (0·0–0·9)	-2·8 (-5·0–0·0)	-5·3 (-8·1–0·0)	-5·1 (-8·8–0·0)
Zinc deficiency	1·6 (0·0–7·0)	1·0 (0·0–4·1)	0·9 (0·0–3·9)	0·9 (0·0–3·7)	-2·0 (-41·4–1·6)	-0·7 (-6·1–3·4)	-0·1 (-8·6–7·4)
Tobacco	36·8 (35·4–38·4)	28·8 (27·6–29·9)	20·9 (20·2–21·7)	18·4 (17·3–19·7)	-2·2 (-2·4–-2·0)	-2·1 (-2·4–-1·8)	-1·1 (-1·7–-0·6)
Smoking	33·3 (31·2–35·7)	25·6 (24·0–27·2)	18·8 (17·7–19·9)	16·2 (14·9–17·5)	-2·3 (-2·6–-2·0)	-2·2 (-2·6–-1·8)	-1·3 (-2·0–-0·7)
Chewing tobacco	0·9 (0·7–1·2)	1·0 (0·8–1·4)	1·1 (0·8–1·5)	1·1 (0·8–1·5)	0·5 (-0·8–1·8)	0·2 (-1·7–2·0)	0·2 (-2·7–2·9)
Second-hand smoke	30·9 (29·0–32·2)	24·8 (22·8–25·9)	18·2 (16·8–19·2)	16·4 (14·8–17·9)	-2·0 (-2·3–-1·8)	-1·9 (-2·4–-1·6)	-0·9 (-1·6–-0·3)
High alcohol use	27·2 (19·5–37·1)	26·3 (18·8–37·0)	27·1 (20·0–37·2)	28·1 (20·3–38·2)	0·1 (-0·8–1·1)	0·3 (-0·8–1·7)	0·3 (-1·3–2·0)
Drug use	1·0 (0·7–1·5)	0·8 (0·6–1·1)	1·1 (0·9–1·4)	3·1 (2·3–4·0)	3·7 (1·6–5·3)	6·5 (3·6–8·7)	9·5 (5·6–12·0)
Dietary risks	34·0 (24·7–45·0)	36·4 (27·2–48·5)	38·6 (29·6–50·5)	38·1 (29·0–50·2)	0·4 (0·1–0·7)	0·2 (-0·1–0·6)	-0·1 (-0·6–0·3)
Diet low in fruits	34·4 (29·7–37·7)	31·0 (26·7–33·8)	29·8 (25·7–32·6)	30·0 (25·2–33·7)	-0·4 (-0·8–0·0)	-0·2 (-0·7–0·4)	0·1 (-0·9–1·0)
Diet low in vegetables	14·8 (8·1–22·2)	12·4 (6·5–19·4)	13·0 (7·3–19·4)	12·0 (6·5–18·7)	-0·7 (-1·6–0·2)	-0·2 (-1·4–1·3)	-0·8 (-3·0–1·4)
Diet low in legumes	36·9 (0·0–46·3)	37·5 (0·0–47·0)	41·2 (0·0–51·1)	41·5 (0·0–52·4)	0·4 (0·0–0·7)	0·5 (0·0–0·9)	0·1 (-0·6–0·7)
Diet low in whole grains	44·3 (36·5–50·6)	48·5 (40·2–55·2)	50·5 (41·7–57·7)	50·5 (41·7–58·2)	0·4 (0·2–0·7)	0·2 (-0·1–0·5)	0·0 (-0·5–0·5)
Diet low in nuts and seeds	10·7 (7·6–14·1)	6·4 (4·3–9·0)	4·2 (2·6–6·5)	4·3 (2·5–7·0)	-3·0 (-4·9–-1·3)	-1·9 (-4·6–0·7)	0·2 (-3·5–3·7)
Diet low in milk	33·9 (30·0–38·7)	32·1 (28·3–36·9)	30·3 (26·1–34·7)	29·5 (25·1–34·6)	-0·4 (-0·9–0·0)	-0·4 (-1·0–0·2)	-0·2 (-1·1–0·6)
Diet high in red meat	50·2 (0·0–69·1)	50·9 (0·0–69·4)	51·4 (0·0–69·9)	51·4 (0·0–70·0)	0·1 (-0·4–0·5)	0·0 (-0·5–0·6)	0·0 (-0·9–0·9)
Diet high in processed meat	46·8 (36·6–52·5)	59·0 (47·4–65·4)	66·8 (54·0–74·1)	64·8 (52·7–73·0)	1·0 (0·7–1·5)	0·4 (-0·1–1·0)	-0·3 (-1·0–0·4)
Diet high in sugar-sweetened beverages	38·3 (29·6–46·5)	52·7 (42·6–60·9)	59·2 (47·5–67·8)	58·9 (46·6–68·8)	1·4 (0·6–2·1)	0·5 (-0·2–1·2)	-0·1 (-1·2–1·0)
Diet low in fibre	36·2 (19·3–42·8)	27·8 (15·3–33·9)	23·8 (12·9–29·7)	20·1 (10·9–25·7)	-1·9 (-2·9–-1·0)	-1·5 (-2·9–-0·5)	-1·5 (-3·5–0·2)
Diet low in calcium	5·1 (4·2–6·3)	4·5 (3·6–5·6)	4·7 (3·8–5·8)	4·6 (3·7–5·6)	-0·4 (-0·5–-0·2)	0·0 (-0·2–0·3)	-0·3 (-0·5–0·1)
Diet low in seafood omega-3 fatty acids	33·0 (24·2–42·5)	41·4 (32·3–52·1)	43·9 (34·3–54·2)	37·4 (27·6–47·0)	0·4 (0·0–0·9)	-0·5 (-1·1–0·0)	-1·5 (-2·3–-0·7)
Diet low in omega-6 polyunsaturated fatty acids	32·6 (21·5–41·5)	29·3 (19·8–37·5)	21·5 (15·1–27·9)	19·4 (13·6–24·8)	-1·7 (-2·4–-0·9)	-2·0 (-2·8–-1·1)	-0·9 (-1·8–-0·2)
Diet high in trans fatty acids	71·6 (64·4–78·8)	72·0 (64·4–78·9)	63·5 (55·3–71·3)	0·0 (0·0–0·0)	-65·8 (-66·1–-65·4)	-97·1 (-97·6–-96·6)	-184·3 (-185·3–-183·0)
Diet high in sodium	23·3 (0·6–71·0)	28·1 (1·7–77·4)	31·1 (3·3–77·9)	31·4 (2·9–79·0)	1·0 (0·2–5·9)	0·5 (-0·2–3·7)	0·1 (-2·8–1·6)
Intimate partner violence	22·0 (12·9–28·3)	21·6 (13·1–27·0)	20·1 (12·3–24·9)	20·8 (10·8–28·6)	-0·2 (-1·7–1·3)	-0·2 (-2·1–1·6)	0·3 (-2·1–2·4)
Childhood sexual abuse and bullying	8·0 (5·0–13·2)	9·8 (6·2–15·9)	11·2 (7·1–18·2)	8·3 (5·6–12·9)	0·1 (-0·5–0·7)	-0·8 (-1·6–0·0)	-2·8 (-4·3–-1·4)
Childhood sexual abuse	6·3 (5·4–7·4)	7·2 (6·2–8·4)	8·3 (7·1–9·5)	7·5 (6·2–9·0)	0·6 (-0·2–1·4)	0·2 (-0·8–1·2)	-0·9 (-2·5–0·5)
Bullying victimization	7·9 (3·5–15·7)	9·9 (4·5–19·3)	11·3 (5·1–22·0)	7·4 (3·3–14·6)	-0·2 (-0·9–0·5)	-1·4 (-2·4–-0·4)	-3·8 (-5·8–-2·1)
Unsafe sex	--	--	--	--	--	--	--
Low physical activity	17·2 (12·4–22·6)	16·6 (12·3–21·3)	18·0 (13·8–22·5)	20·5 (15·5–26·6)	0·6 (-0·4–1·5)	1·0 (-0·2–2·2)	1·2 (-0·5–2·8)
**Metabolic risks**	**23·9 (21·0–26·9)**	**30·0 (26·7–33·1)**	**32·4 (28·8–35·4)**	**34·8 (30·5–37·8)**	**1·2 (0·9–1·5)**	**0·7 (0·4–1·0)**	**0·7 (0·2–1·1)**
High fasting plasma glucose	11·3 (8·7–13·5)	14·9 (11·3–17·9)	19·3 (14·5–22·9)	23·0 (17·3–27·5)	2·3 (1·7–3·0)	2·1 (1·3–2·9)	1·6 (0·5–2·9)
High LDL cholesterol	65·4 (46·2–88·7)	53·9 (36·9–74·3)	47·8 (32·4–66·6)	46·2 (31·2–64·7)	-1·1 (-1·3–-0·9)	-0·7 (-0·9–-0·6)	-0·3 (-0·6–-0·1)
High systolic blood pressure	28·1 (18·9–38·3)	24·4 (17·1–33·5)	18·2 (11·7–26·1)	24·8 (15·6–35·4)	-0·4 (-1·4–0·5)	0·1 (-1·2–1·2)	2·8 (0·7–5·1)
High body-mass index	27·3 (23·3–31·9)	34·8 (29·7–38·8)	37·5 (32·1–41·4)	39·5 (33·8–43·3)	1·2 (0·9–1·5)	0·6 (0·3–0·9)	0·5 (0·1–0·9)
Low bone mineral density	22·0 (16·1–29·2)	21·8 (15·9–29·0)	22·9 (16·5–30·2)	24·1 (17·4–31·5)	0·3 (-0·2–0·7)	0·5 (-0·2–1·2)	0·5 (-0·8–1·9)
Kidney dysfunction	3·1 (2·6–4·0)	3·1 (2·6–4·0)	3·0 (2·5–4·0)	3·1 (2·6–4·1)	0·0 (-0·1–0·2)	0·1 (-0·1–0·2)	0·3 (0·0–0·6)

Colorado

Risk Names	SEV 1990	SEV 2000	SEV 2010	SEV 2021	Annualised rate of change 1990 to 2021	Annualised rate of change 2000 to 2021	Annualised rate of change 2010 to 2021
**All risk factors**	**25·1 (22·1–28·4)**	**24·6 (21·8–27·7)**	**25·3 (22·3–28·5)**	**24·7 (21·5–27·8)**	**-0·1 (-0·4–0·3)**	**0·0 (-0·4–0·5)**	**-0·2 (-0·8–0·4)**
**Environmental/occupational risks**	**16·2 (10·4–27·1)**	**15·9 (10·0–26·0)**	**14·2 (8·9–23·4)**	**12·5 (7·9–21·1)**	**-0·8 (-1·5–-0·3)**	**-1·1 (-1·5–-0·7)**	**-1·1 (-1·5–-0·7)**
Unsafe water, sanitation, and handwashing	1·7 (0·9–2·8)	1·7 (0·7–2·7)	1·3 (0·4–2·2)	1·0 (0·3–1·8)	-1·7 (-4·1–0·2)	-2·4 (-5·4–0·6)	-2·1 (-6·0–2·3)
Unsafe water source	2·5 (0·9–5·2)	2·4 (0·9–5·0)	1·0 (0·4–2·0)	0·7 (0·3–1·4)	-3·9 (-6·6–-1·0)	-5·5 (-9·1–-1·8)	-2·2 (-7·7–3·2)
Unsafe sanitation	5·3 (2·7–9·1)	4·5 (2·4–7·6)	3·3 (1·7–5·7)	2·4 (1·2–4·3)	-2·6 (-5·1–-0·5)	-3·0 (-5·7–-0·2)	-2·9 (-6·1–0·2)
No access to handwashing facility	1·5 (0·5–2·8)	1·6 (0·5–3·0)	1·6 (0·5–3·0)	1·3 (0·4–2·5)	-0·4 (-3·2–2·4)	-0·9 (-4·5–3·1)	-1·7 (-7·7–4·5)
Air pollution	19·2 (3·3–29·3)	18·0 (4·3–25·4)	11·1 (2·2–21·2)	5·7 (0·7–14·5)	-3·9 (-8·9–-1·4)	-5·5 (-12·0–-2·4)	-6·1 (-14·6–-3·4)
Particulate matter pollution	7·0 (1·3–14·8)	6·2 (3·4–9·5)	3·5 (1·2–6·4)	1·6 (0·2–3·9)	-4·8 (-11·5–-1·0)	-6·5 (-14·3–-4·0)	-7·3 (-18·4–-4·0)
Ambient particulate matter pollution	10·8 (1·9–22·4)	9·5 (5·4–14·0)	5·3 (1·8–9·5)	2·4 (0·3–5·8)	-4·9 (-11·5–-1·0)	-6·6 (-14·3–-4·0)	-7·3 (-18·5–-3·9)
Household air pollution from solid fuels	0·0 (0·0–0·0)	0·0 (0·0–0·0)	0·0 (0·0–0·0)	0·0 (0·0–0·0)	-4·9 (-40·1–-8·8)	-6·2 (-38·7–-9·1)	-6·7 (-42·9–-10·2)
Ambient ozone pollution	21·6 (15·8–28·6)	24·3 (18·1–31·6)	28·0 (21·7–36·3)	25·0 (18·6–32·8)	0·5 (0·1–0·9)	0·1 (-0·4–0·7)	-1·0 (-2·1–-0·1)
Ambient nitrogen dioxide pollution	72·6 (0·0–100·0)	70·0 (0·0–100·0)	43·5 (0·0–100·0)	22·4 (0·0–70·6)	-3·8 (-14·3–0·0)	-5·4 (-20·6–0·0)	-6·0 (-26·4–0·0)
Non-optimal temperature	30·2 (24·6–38·7)	30·8 (25·1–39·1)	30·0 (24·6–38·2)	30·3 (24·3–39·3)	0·0 (-0·2–0·2)	-0·1 (-0·3–0·2)	0·1 (-0·3–0·7)
High temperature	26·3 (15·7–39·1)	24·1 (13·8–35·0)	22·4 (12·6–33·5)	27·3 (16·3–40·2)	0·1 (-0·3–0·6)	0·6 (0·1–1·2)	1·8 (0·7–3·4)
Low temperature	28·1 (24·2–33·0)	28·9 (24·9–33·8)	28·5 (24·4–33·5)	27·9 (23·8–33·0)	0·0 (-0·1–0·1)	-0·2 (-0·3–-0·1)	-0·2 (-0·4–-0·1)
Other environmental risks	29·0 (11·2–47·2)	27·2 (11·2–45·3)	24·5 (11·2–42·7)	21·7 (9·9–39·7)	-0·9 (-1·7–0·0)	-1·1 (-2·0–0·0)	-1·1 (-2·3–0·0)
Residential radon	31·0 (0·0–89·5)	31·0 (0·0–89·5)	31·0 (0·0–89·5)	31·0 (0·0–89·5)	0·0 (0·0–0·0)	0·0 (0·0–0·0)	0·0 (0·0–0·0)
Lead exposure	28·1 (0·0–34·9)	25·5 (0·0–31·8)	21·5 (0·0–26·7)	17·4 (0·0–21·6)	-1·5 (-2·0–0·0)	-1·8 (-2·3–0·0)	-1·9 (-2·7–0·0)
Occupational risks	2·9 (2·4–3·8)	3·0 (2·5–3·9)	2·8 (2·3–3·6)	2·8 (2·3–3·7)	-0·1 (-0·3–0·1)	-0·3 (-0·6–0·0)	0·0 (-0·4–0·4)
Occupational carcinogens	1·0 (0·4–1·8)	1·0 (0·4–2·0)	1·0 (0·4–1·9)	1·0 (0·4–2·0)	0·2 (-0·2–0·5)	-0·1 (-0·5–0·3)	0·2 (-0·3–0·7)
Occupational exposure to asbestos	3·5 (3·1–4·0)	3·7 (3·3–4·1)	3·1 (2·7–3·4)	3·2 (2·5–4·0)	-0·3 (-1·1–0·5)	-0·7 (-1·9–0·5)	0·4 (-1·8–2·6)
Occupational exposure to arsenic	0·5 (0·0–1·1)	0·5 (0·0–1·2)	0·5 (0·0–1·1)	0·5 (0·0–1·1)	-0·1 (-0·5–0·4)	-0·2 (-0·8–0·4)	0·1 (-0·8–1·0)
Occupational exposure to benzene	1·1 (0·1–3·2)	1·2 (0·1–3·6)	1·2 (0·1–3·5)	1·2 (0·1–3·6)	0·4 (0·0–0·7)	0·1 (-0·4–0·5)	0·3 (-0·5–0·9)
Occupational exposure to beryllium	0·0 (0·0–0·0)	0·0 (0·0–0·0)	0·0 (0·0–0·0)	0·0 (0·0–0·0)	0·2 (0·0–0·4)	0·1 (-0·2–0·4)	0·4 (0·0–0·9)
Occupational exposure to cadmium	0·1 (0·1–0·1)	0·1 (0·1–0·1)	0·1 (0·1–0·1)	0·1 (0·1–0·1)	0·0 (-0·4–0·4)	-0·2 (-0·7–0·4)	0·2 (-0·6–0·9)
Occupational exposure to chromium	0·1 (0·1–0·2)	0·1 (0·1–0·2)	0·1 (0·1–0·2)	0·1 (0·1–0·2)	0·1 (-0·3–0·4)	-0·1 (-0·6–0·4)	0·2 (-0·5–0·8)
Occupational exposure to diesel engine exhaust	0·8 (0·7–0·8)	0·8 (0·7–0·8)	0·8 (0·7–0·8)	0·8 (0·7–0·9)	0·2 (-0·2–0·5)	0·1 (-0·4–0·6)	0·5 (-0·1–1·2)
Occupational exposure to formaldehyde	0·3 (0·3–0·3)	0·3 (0·3–0·3)	0·3 (0·3–0·3)	0·3 (0·3–0·3)	-0·1 (-0·5–0·3)	-0·3 (-0·8–0·3)	0·0 (-0·7–0·8)
Occupational exposure to nickel	0·4 (0·0–1·5)	0·4 (0·0–1·5)	0·4 (0·0–1·4)	0·4 (0·0–1·4)	-0·2 (-0·8–0·3)	-0·3 (-0·9–0·4)	0·1 (-0·9–1·1)
Occupational exposure to polycyclic aromatic hydrocarbons	0·3 (0·3–0·3)	0·3 (0·3–0·3)	0·3 (0·3–0·3)	0·3 (0·3–0·3)	0·1 (-0·2–0·5)	-0·1 (-0·5–0·4)	0·2 (-0·4–0·8)
Occupational exposure to silica	3·5 (0·6–11·1)	3·3 (0·5–10·6)	3·1 (0·5–10·0)	3·2 (0·5–10·3)	-0·3 (-0·8–0·1)	-0·2 (-0·9–0·4)	0·2 (-0·9–1·2)
Occupational exposure to sulphuric acid	0·7 (0·1–2·3)	0·7 (0·2–2·5)	0·7 (0·1–2·3)	0·7 (0·1–2·3)	0·0 (-0·4–0·4)	-0·2 (-0·7–0·4)	0·2 (-0·6–0·9)
Occupational exposure to trichloroethylene	0·1 (0·1–0·1)	0·1 (0·1–0·1)	0·1 (0·1–0·1)	0·1 (0·1–0·1)	0·1 (-0·1–0·4)	0·0 (-0·4–0·4)	0·3 (-0·2–0·9)
Occupational asthmagens	17·2 (15·1–19·5)	17·8 (15·6–20·3)	16·6 (14·6–18·8)	16·8 (14·9–19·4)	-0·1 (-0·4–0·2)	-0·3 (-0·7–0·2)	0·1 (-0·6–0·8)
Occupational particulate matter, gases, and fumes	6·4 (4·9–8·6)	6·2 (4·8–8·2)	5·9 (4·5–7·8)	5·5 (4·2–7·2)	-0·5 (-0·7–-0·2)	-0·6 (-0·9–-0·3)	-0·6 (-1·0–-0·3)
Occupational noise	6·9 (6·3–7·6)	6·9 (6·4–7·5)	6·8 (6·4–7·4)	6·6 (6·2–7·1)	-0·1 (-0·3–0·0)	-0·2 (-0·4–0·0)	-0·3 (-0·5–-0·1)
Occupational injuries	--	--	--	--	--	--	--
Occupational ergonomic factors	8·2 (6·9–9·8)	8·3 (7·0–9·9)	7·6 (6·4–9·1)	7·7 (6·5–9·3)	-0·2 (-0·6–0·2)	-0·4 (-0·9–0·1)	0·1 (-0·6–0·7)
**Behavioural risks**	**30·1 (26·4–33·8)**	**27·6 (24·2–31·3)**	**25·8 (22·6–29·7)**	**24·6 (20·9–28·5)**	**-0·7 (-1·0–-0·3)**	**-0·5 (-1·1–-0·1)**	**-0·5 (-1·2–0·3)**
Child and maternal malnutrition	10·0 (6·9–14·0)	8·6 (5·8–12·1)	8·7 (6·1–12·0)	8·8 (5·9–12·4)	-0·4 (-1·4–0·5)	0·1 (-1·2–1·2)	0·1 (-2·0–1·7)
Suboptimal breastfeeding	68·7 (66·4–71·2)	66·6 (63·9–69·1)	62·8 (60·2–65·5)	62·2 (59·1–65·3)	-0·3 (-0·5–-0·2)	-0·3 (-0·5–-0·1)	-0·1 (-0·4–0·2)
Non-exclusive breastfeeding	63·9 (56·3–71·6)	59·4 (52·7–65·8)	48·5 (41·4–55·6)	46·2 (38·9–53·6)	-1·1 (-1·6–-0·5)	-1·2 (-1·9–-0·5)	-0·4 (-1·5–0·6)
Discontinued breastfeeding	88·6 (86·5–91·2)	86·3 (83·6–89·1)	84·3 (80·9–87·4)	84·6 (81·4–88·0)	-0·1 (-0·3–0·0)	-0·1 (-0·3–0·1)	0·0 (-0·3–0·3)
Child growth failure	1·2 (0·4–2·7)	1·1 (0·4–2·6)	1·0 (0·3–2·2)	0·9 (0·3–2·0)	-0·9 (-1·3–-0·5)	-1·1 (-1·6–-0·6)	-0·8 (-1·5–0·0)
Child underweight	2·5 (1·6–3·5)	2·4 (1·6–3·3)	2·1 (1·3–2·8)	1·9 (1·2–2·6)	-0·9 (-1·5–-0·4)	-1·1 (-1·9–-0·4)	-0·7 (-1·7–0·3)
Child wasting	0·8 (0·5–1·3)	0·8 (0·5–1·2)	0·8 (0·5–1·3)	0·8 (0·5–1·2)	-0·2 (-0·6–0·1)	-0·1 (-0·6–0·3)	-0·3 (-1·0–0·4)
Child stunting	3·5 (2·8–4·5)	3·4 (2·6–4·2)	2·9 (2·2–3·6)	2·6 (2·0–3·3)	-1·0 (-1·5–-0·4)	-1·2 (-2·0–-0·3)	-0·9 (-2·1–0·2)
Low birth weight and short gestation	20·7 (17·9–23·3)	21·6 (19·1–24·2)	22·4 (19·6–25·1)	24·6 (21·6–27·7)	0·6 (0·3–0·8)	0·6 (0·2–1·0)	0·8 (0·2–1·6)
Short gestation	32·0 (28·4–36·0)	34·1 (30·1–38·2)	35·0 (31·3–39·4)	36·7 (32·7–41·2)	0·4 (0·1–0·8)	0·4 (-0·1–0·8)	0·4 (-0·5–1·3)
Low birth weight	17·0 (15·5–18·6)	17·3 (15·9–18·8)	18·0 (16·6–19·4)	20·0 (18·4–21·9)	0·5 (0·3–0·8)	0·7 (0·3–1·1)	1·0 (0·3–1·7)

Iron deficiency	3·5 (2·5–4·8)	3·0 (2·1–4·1)	3·1 (2·2–4·2)	3·1 (2·1–4·3)	-0·4 (-1·2–0·4)	0·2 (-1·0–1·3)	0·1 (-1·8–1·7)
Vitamin A deficiency	0·9 (0·0–1·8)	1·2 (0·0–2·5)	0·7 (0·0–1·5)	0·4 (0·0–0·9)	-2·3 (-4·6–0·0)	-5·1 (-7·8–0·0)	-5·0 (-9·1–0·0)
Zinc deficiency	1·7 (0·0–7·4)	1·0 (0·0–4·2)	0·9 (0·0–4·1)	0·9 (0·0–3·9)	-2·0 (-44·6–0·6)	-0·5 (-2·5–4·0)	-0·3 (-4·9–18·7)
Tobacco	37·8 (36·2–39·4)	32·3 (30·9–33·4)	25·8 (24·8–26·7)	22·6 (21·1–24·2)	-1·7 (-1·9–-1·4)	-1·7 (-2·0–-1·4)	-1·2 (-1·8–-0·6)
Smoking	36·1 (33·6–38·6)	29·6 (27·7–31·5)	23·6 (22·3–25·0)	20·4 (18·7–22·1)	-1·8 (-2·1–-1·5)	-1·8 (-2·2–-1·4)	-1·3 (-2·1–-0·5)
Chewing tobacco	2·0 (1·4–2·6)	2·2 (1·6–2·8)	2·2 (1·6–3·0)	2·2 (1·5–3·0)	0·3 (-1·1–1·7)	0·0 (-1·9–1·8)	-0·2 (-2·9–2·4)
Second-hand smoke	30·2 (28·5–31·6)	26·3 (24·6–27·6)	21·1 (19·5–22·1)	18·8 (16·9–20·4)	-1·5 (-1·8–-1·3)	-1·6 (-2·0–-1·2)	-1·0 (-1·7–-0·3)
High alcohol use	29·6 (21·4–40·0)	27·7 (19·8–38·8)	29·1 (21·4–39·7)	30·2 (21·7–41·8)	0·1 (-0·9–1·0)	0·4 (-1·0–1·7)	0·3 (-1·6–2·2)
Drug use	0·8 (0·3–1·6)	0·8 (0·5–1·4)	1·4 (1·2–1·8)	4·6 (3·3–5·9)	5·7 (2·6–9·1)	8·2 (4·5–11·5)	10·6 (6·4–13·4)
Dietary risks	35·4 (26·4–46·1)	38·1 (29·0–49·0)	41·1 (32·0–53·2)	41·2 (32·0–53·0)	0·5 (0·3–0·8)	0·4 (0·1–0·6)	0·0 (-0·4–0·5)
Diet low in fruits	34·8 (29·9–38·0)	31·5 (27·3–34·4)	31·6 (27·8–34·3)	32·0 (27·1–35·9)	-0·3 (-0·6–0·1)	0·1 (-0·5–0·6)	0·1 (-0·8–1·0)
Diet low in vegetables	20·4 (11·9–28·2)	21·8 (13·1–29·3)	27·7 (15·3–35·2)	26·8 (15·4–34·6)	0·9 (0·4–1·5)	1·0 (0·4–1·7)	-0·3 (-1·2–0·5)
Diet low in legumes	37·4 (0·0–47·5)	37·5 (0·0–47·1)	41·7 (0·0–52·3)	41·9 (0·0–52·2)	0·4 (0·0–0·7)	0·5 (0·0–1·0)	0·0 (-0·7–0·7)
Diet low in whole grains	44·1 (36·4–50·6)	46·8 (39·4–53·8)	49·1 (41·0–56·6)	49·8 (41·8–57·2)	0·4 (0·1–0·7)	0·3 (-0·1–0·7)	0·1 (-0·4–0·7)
Diet low in nuts and seeds	11·7 (8·4–16·0)	6·6 (4·0–9·4)	4·8 (2·8–7·3)	4·8 (2·8–7·5)	-2·9 (-4·8–-1·3)	-1·5 (-4·2–1·0)	0·0 (-3·7–4·1)
Diet low in milk	34·9 (30·7–39·5)	31·6 (27·7–36·6)	30·6 (26·8–35·0)	30·0 (25·5–34·9)	-0·5 (-1·0–0·0)	-0·3 (-0·8–0·3)	-0·2 (-1·1–0·6)
Diet high in red meat	52·8 (0·0–71·5)	53·2 (0·0–72·1)	53·3 (0·0–72·7)	53·7 (0·0–72·9)	0·1 (-0·4–0·5)	0·0 (-0·5–0·6)	0·1 (-0·8–1·0)
Diet high in processed meat	45·1 (35·6–49·9)	55·5 (43·9–61·4)	62·5 (50·2–69·9)	61·6 (49·8–68·9)	1·0 (0·6–1·4)	0·5 (0·0–1·0)	-0·1 (-0·9–0·6)
Diet high in sugar-sweetened beverages	37·0 (28·8–45·1)	50·6 (40·1–58·6)	55·7 (43·3–64·7)	56·7 (44·5–66·7)	1·4 (0·6–2·1)	0·5 (-0·4–1·5)	0·2 (-1·2–1·4)
Diet low in fibre	37·7 (20·5–45·9)	28·0 (15·6–34·7)	25·0 (14·1–31·6)	21·0 (11·4–27·4)	-1·9 (-2·9–-1·0)	-1·4 (-2·7–-0·1)	-1·6 (-3·5–0·3)
Diet low in calcium	5·3 (4·3–6·5)	4·5 (3·6–5·5)	4·8 (3·8–5·9)	4·7 (3·7–5·7)	-0·4 (-0·7–-0·2)	0·2 (0·0–0·4)	-0·2 (-0·6–0·1)
Diet low in seafood omega-3 fatty acids	47·4 (36·4–58·3)	53·8 (43·1–65·0)	57·6 (46·7–68·8)	51·0 (40·2–62·3)	0·2 (-0·1–0·6)	-0·3 (-0·7–0·2)	-1·1 (-1·7–-0·5)
Diet low in omega-6 polyunsaturated fatty acids	33·8 (22·4–43·6)	29·2 (19·6–37·8)	22·3 (15·7–28·7)	20·0 (14·0–26·1)	-1·7 (-2·5–-0·9)	-1·8 (-2·8–-1·0)	-1·0 (-2·1–-0·1)
Diet high in trans fatty acids	71·4 (63·9–78·6)	71·7 (64·3–79·1)	63·4 (55·8–71·5)	0·0 (0·0–0·0)	-65·8 (-66·1–-65·4)	-97·1 (-97·6–-96·6)	-184·3 (-185·3–-183·1)
Diet high in sodium	22·9 (0·5–66·5)	27·6 (1·9–73·6)	30·6 (3·5–75·0)	31·1 (3·2–76·5)	1·0 (0·2–6·6)	0·6 (-0·1–4·0)	0·1 (-2·6–1·6)
Intimate partner violence	26·5 (16·0–34·7)	26·6 (17·6–33·3)	22·0 (13·6–27·8)	21·1 (10·3–29·3)	-0·7 (-2·2–0·6)	-1·1 (-3·3–0·7)	-0·4 (-3·2–1·8)
Childhood sexual abuse and bullying	8·6 (5·3–13·6)	10·4 (6·6–16·6)	11·9 (7·5–19·1)	9·6 (6·4–14·8)	0·4 (-0·2–0·9)	-0·4 (-1·3–0·4)	-2·0 (-3·6–-0·4)
Childhood sexual abuse	7·5 (6·3–8·9)	8·4 (7·1–9·9)	9·7 (8·4–11·2)	9·1 (7·5–11·3)	0·6 (-0·2–1·5)	0·4 (-0·7–1·5)	-0·6 (-2·0–1·0)
Bullying victimization	8·0 (3·5–15·9)	10·0 (4·6–19·6)	11·3 (5·1–22·1)	8·3 (3·7–15·7)	0·1 (-0·6–0·9)	-0·9 (-2·0–0·1)	-2·8 (-4·8–-1·0)
Unsafe sex	--	--	--	--	--	--	--
Low physical activity	17·9 (13·1–23·2)	17·0 (12·7–21·2)	17·6 (13·8–22·0)	20·5 (15·4–26·5)	0·4 (-0·5–1·3)	0·9 (-0·3–2·0)	1·4 (-0·3–3·2)
**Metabolic risks**	**23·4 (20·3–26·7)**	**26·6 (23·2–29·8)**	**29·3 (25·9–32·2)**	**32·4 (28·5–35·3)**	**1·1 (0·7–1·4)**	**0·9 (0·6–1·3)**	**0·9 (0·4–1·4)**
High fasting plasma glucose	11·2 (8·2–13·5)	13·6 (10·2–16·6)	17·1 (13·1–21·2)	21·1 (16·0–25·9)	2·1 (1·4–2·8)	2·1 (1·2–3·0)	1·9 (0·7–3·4)
High LDL cholesterol	63·8 (44·6–87·7)	53·7 (37·0–74·6)	48·1 (32·6–67·4)	46·6 (31·4–65·3)	-1·0 (-1·2–-0·8)	-0·7 (-0·9–-0·5)	-0·3 (-0·6–0·0)
High systolic blood pressure	27·1 (18·6–37·7)	22·9 (16·1–31·6)	16·9 (10·9–24·7)	24·0 (15·0–35·0)	-0·4 (-1·4–0·5)	0·2 (-1·0–1·4)	3·2 (1·0–5·6)
High body-mass index	26·3 (22·1–30·9)	30·6 (26·0–34·9)	33·9 (28·6–37·9)	36·7 (30·8–40·6)	1·1 (0·7–1·4)	0·9 (0·5–1·2)	0·7 (0·2–1·2)
Low bone mineral density	21·7 (15·9–28·4)	19·9 (14·3–26·8)	20·5 (15·0–27·4)	22·2 (15·9–28·6)	0·1 (-0·4–0·6)	0·5 (-0·1–1·4)	0·7 (-0·7–2·0)
Kidney dysfunction	2·8 (2·3–3·7)	2·9 (2·3–3·8)	2·8 (2·3–3·8)	2·9 (2·4–3·9)	0·1 (0·0–0·2)	0·1 (-0·1–0·2)	0·3 (0·0–0·6)

Connecticut

Risk Names	SEV 1990	SEV 2000	SEV 2010	SEV 2021	Annualised rate of change 1990 to 2021	Annualised rate of change 2000 to 2021	Annualised rate of change 2010 to 2021
**All risk factors**	**28·0 (24·8–31·4)**	**27·5 (24·5–30·9)**	**28·6 (25·5–31·7)**	**28·8 (25·6–32·2)**	**0·1 (-0·2–0·4)**	**0·2 (-0·1–0·6)**	**0·1 (-0·5–0·7)**
**Environmental/occupational risks**	**17·6 (11·7–28·3)**	**17·5 (12·1–28·4)**	**14·9 (9·7–24·4)**	**13·4 (8·6–22·4)**	**-0·9 (-1·6–-0·2)**	**-1·3 (-1·7–-0·7)**	**-1·0 (-1·5–-0·4)**
Unsafe water, sanitation, and handwashing	1·4 (0·6–2·4)	1·4 (0·5–2·3)	1·1 (0·3–1·9)	1·0 (0·3–1·7)	-1·3 (-3·7–0·6)	-1·6 (-4·8–1·0)	-1·5 (-5·3–2·3)
Unsafe water source	2·0 (0·7–4·1)	1·9 (0·8–3·8)	0·8 (0·4–1·5)	0·7 (0·3–1·2)	-3·5 (-6·4–-0·7)	-4·8 (-8·3–-1·1)	-1·6 (-6·5–3·9)
Unsafe sanitation	3·8 (2·0–6·3)	3·0 (1·5–5·2)	2·4 (1·2–4·3)	1·9 (0·9–3·3)	-2·1 (-4·5–0·1)	-2·1 (-4·9–0·2)	-2·0 (-5·6–1·3)
No access to handwashing facility	1·4 (0·5–2·7)	1·5 (0·5–2·8)	1·5 (0·5–2·8)	1·3 (0·5–2·5)	-0·2 (-3·3–2·8)	-0·5 (-4·4–3·5)	-1·3 (-6·7–4·2)
Air pollution	21·5 (6·5–33·5)	19·3 (7·0–28·0)	10·0 (3·9–19·3)	6·0 (1·6–13·8)	-4·1 (-7·2–-1·7)	-5·6 (-9·5–-3·0)	-4·7 (-9·0–-2·8)
Particulate matter pollution	10·9 (3·3–20·8)	9·6 (6·4–13·2)	6·1 (3·2–9·5)	3·6 (1·1–6·6)	-3·6 (-7·5–-0·2)	-4·6 (-8·8–-3·0)	-4·8 (-10·8–-2·5)
Ambient particulate matter pollution	16·9 (5·1–31·5)	14·7 (10·1–19·7)	9·3 (5·0–14·1)	5·5 (1·6–10·0)	-3·6 (-7·6–-0·3)	-4·7 (-8·9–-3·0)	-4·8 (-10·9–-2·5)
Household air pollution from solid fuels	0·0 (0·0–0·0)	0·0 (0·0–0·0)	0·0 (0·0–0·0)	0·0 (0·0–0·0)	-5·1 (-47·4–19·0)	-5·6 (-45·1–56·1)	-4·3 (-58·8–0·0)
Ambient ozone pollution	27·0 (20·3–36·2)	23·1 (17·0–31·6)	16·7 (10·7–24·3)	18·2 (12·1–25·9)	-1·3 (-1·8–-0·8)	-1·1 (-2·0–-0·4)	0·8 (-0·6–2·3)
Ambient nitrogen dioxide pollution	66·3 (0·0–100·0)	60·5 (0·0–100·0)	25·4 (0·0–73·7)	14·4 (0·0–54·5)	-4·9 (-14·1–0·0)	-6·8 (-19·8–0·0)	-5·2 (-18·9–0·0)
Non-optimal temperature	31·0 (23·6–42·4)	30·0 (24·0–40·0)	34·7 (26·2–46·4)	31·5 (24·2–42·5)	0·0 (-0·2–0·3)	0·2 (-0·2–0·7)	-0·9 (-1·6–-0·5)
High temperature	26·6 (16·2–38·0)	20·3 (10·9–31·5)	37·9 (24·7–50·7)	30·9 (19·6–42·8)	0·5 (0·1–0·9)	2·0 (1·1–3·4)	-1·8 (-2·7–-1·1)
Low temperature	27·4 (24·4–30·9)	28·5 (25·5–31·7)	28·7 (25·9–32·0)	27·4 (24·5–30·7)	0·0 (-0·1–0·1)	-0·2 (-0·4–-0·1)	-0·4 (-0·7–-0·3)
Other environmental risks	26·8 (10·5–47·1)	25·2 (10·5–46·7)	22·7 (10·3–43·7)	20·3 (9·5–40·5)	-0·9 (-1·6–0·0)	-1·0 (-1·9–0·0)	-1·0 (-2·1–0·0)
Residential radon	29·3 (0·0–92·9)	29·3 (0·0–92·9)	29·3 (0·0–92·9)	29·3 (0·0–92·9)	0·0 (0·0–0·0)	0·0 (0·0–0·0)	0·0 (0·0–0·0)
Lead exposure	25·7 (0·0–32·4)	23·3 (0·0–28·9)	19·7 (0·0–24·5)	16·2 (0·0–20·3)	-1·5 (-1·9–0·0)	-1·7 (-2·3–0·0)	-1·7 (-2·7–0·0)
Occupational risks	3·1 (2·6–4·1)	3·1 (2·6–4·1)	2·9 (2·4–3·8)	2·9 (2·4–3·8)	-0·2 (-0·4–0·1)	-0·3 (-0·6–0·0)	0·0 (-0·4–0·4)
Occupational carcinogens	1·0 (0·5–2·0)	1·1 (0·5–2·1)	1·1 (0·5–2·0)	1·1 (0·5–2·0)	0·1 (-0·3–0·4)	-0·1 (-0·5–0·2)	0·1 (-0·5–0·6)
Occupational exposure to asbestos	4·4 (3·9–5·0)	4·3 (3·9–4·8)	4·0 (3·5–4·4)	3·9 (3·0–4·9)	-0·4 (-1·3–0·4)	-0·5 (-1·6–0·8)	-0·1 (-2·4–2·1)
Occupational exposure to arsenic	0·6 (0·0–1·3)	0·5 (0·0–1·3)	0·5 (0·0–1·2)	0·5 (0·0–1·2)	-0·4 (-0·9–0·0)	-0·4 (-1·0–0·2)	0·0 (-0·8–0·8)
Occupational exposure to benzene	1·1 (0·1–3·2)	1·3 (0·1–3·7)	1·3 (0·1–3·7)	1·3 (0·1–3·7)	0·4 (0·0–0·7)	0·0 (-0·4–0·4)	0·2 (-0·4–0·7)
Occupational exposure to beryllium	0·0 (0·0–0·0)	0·0 (0·0–0·0)	0·0 (0·0–0·0)	0·0 (0·0–0·0)	0·2 (0·0–0·4)	0·1 (-0·3–0·4)	0·4 (-0·1–0·8)
Occupational exposure to cadmium	0·1 (0·1–0·1)	0·1 (0·1–0·1)	0·1 (0·1–0·1)	0·1 (0·1–0·1)	-0·3 (-0·7–0·1)	-0·3 (-0·8–0·3)	0·1 (-0·7–0·8)
Occupational exposure to chromium	0·2 (0·2–0·2)	0·2 (0·2–0·2)	0·2 (0·1–0·2)	0·2 (0·1–0·2)	-0·2 (-0·5–0·2)	-0·2 (-0·7–0·3)	0·1 (-0·5–0·7)
Occupational exposure to diesel engine exhaust	0·7 (0·6–0·8)	0·7 (0·7–0·8)	0·7 (0·7–0·8)	0·8 (0·7–0·9)	0·4 (0·1–0·8)	0·3 (-0·2–0·8)	0·6 (-0·1–1·3)
Occupational exposure to formaldehyde	0·3 (0·3–0·4)	0·3 (0·3–0·4)	0·3 (0·3–0·3)	0·3 (0·3–0·3)	-0·4 (-0·8–0·0)	-0·4 (-0·9–0·1)	-0·1 (-0·8–0·7)
Occupational exposure to nickel	0·5 (0·0–1·8)	0·4 (0·0–1·7)	0·4 (0·0–1·5)	0·4 (0·0–1·5)	-0·4 (-1·0–0·0)	-0·4 (-1·1–0·3)	0·0 (-1·1–1·0)
Occupational exposure to polycyclic aromatic hydrocarbons	0·3 (0·3–0·4)	0·3 (0·3–0·4)	0·3 (0·3–0·3)	0·3 (0·3–0·3)	-0·1 (-0·4–0·2)	-0·2 (-0·6–0·2)	0·1 (-0·5–0·7)
Occupational exposure to silica	3·9 (0·6–13·0)	3·6 (0·5–11·4)	3·3 (0·5–10·5)	3·4 (0·5–10·4)	-0·5 (-0·9–0·0)	-0·3 (-1·0–0·3)	0·1 (-0·8–1·0)
Occupational exposure to sulphuric acid	0·8 (0·2–2·8)	0·8 (0·2–2·7)	0·8 (0·2–2·5)	0·8 (0·2–2·6)	-0·3 (-0·7–0·1)	-0·3 (-0·8–0·3)	0·1 (-0·7–0·9)
Occupational exposure to trichloroethylene	0·1 (0·1–0·1)	0·1 (0·1–0·1)	0·1 (0·1–0·1)	0·1 (0·1–0·1)	0·0 (-0·2–0·3)	0·0 (-0·4–0·4)	0·3 (-0·2–0·8)
Occupational asthmagens	18·4 (15·9–20·7)	18·5 (16·3–20·9)	17·3 (15·1–19·7)	17·6 (15·3–20·0)	-0·1 (-0·5–0·2)	-0·2 (-0·7–0·2)	0·1 (-0·5–0·8)
Occupational particulate matter, gases, and fumes	7·2 (5·5–9·3)	6·8 (5·2–8·8)	6·3 (4·9–8·2)	5·8 (4·5–7·7)	-0·7 (-0·9–-0·4)	-0·8 (-1·1–-0·5)	-0·8 (-1·1–-0·4)
Occupational noise	7·0 (6·5–7·7)	7·0 (6·5–7·7)	6·9 (6·5–7·5)	6·7 (6·3–7·2)	-0·2 (-0·3–0·0)	-0·2 (-0·4–0·0)	-0·3 (-0·5–-0·1)
Occupational injuries	--	--	--	--	--	--	--
Occupational ergonomic factors	8·0 (6·7–9·6)	8·1 (6·8–9·8)	7·5 (6·3–9·1)	7·7 (6·4–9·3)	-0·1 (-0·5–0·3)	-0·3 (-0·8–0·3)	0·2 (-0·5–0·8)
**Behavioural risks**	**32·1 (28·8–36·1)**	**29·9 (26·6–33·6)**	**27·7 (24·5–31·4)**	**25·7 (22·5–29·7)**	**-0·7 (-1·1–-0·4)**	**-0·7 (-1·2–-0·2)**	**-0·7 (-1·3–0·0)**
Child and maternal malnutrition	9·9 (6·9–13·9)	8·5 (5·8–11·8)	8·5 (6·0–11·6)	8·7 (5·9–12·4)	-0·4 (-1·2–0·4)	0·1 (-1·1–1·4)	0·2 (-1·7–2·1)
Suboptimal breastfeeding	68·6 (66·0–71·3)	66·4 (64·1–69·1)	62·5 (60·2–65·3)	62·0 (59·4–65·2)	-0·3 (-0·5–-0·2)	-0·3 (-0·5–-0·1)	-0·1 (-0·4–0·2)
Non-exclusive breastfeeding	63·7 (56·3–71·5)	58·8 (52·6–65·4)	47·9 (41·6–54·8)	45·7 (38·9–53·4)	-1·1 (-1·6–-0·6)	-1·2 (-1·8–-0·6)	-0·4 (-1·4–0·6)
Discontinued breastfeeding	88·5 (86·2–90·9)	86·1 (83·8–88·7)	84·2 (80·9–87·6)	84·5 (81·2–87·8)	-0·2 (-0·3–0·0)	-0·1 (-0·3–0·1)	0·0 (-0·3–0·3)
Child growth failure	1·1 (0·4–2·4)	1·0 (0·4–2·4)	0·9 (0·3–2·0)	0·8 (0·3–1·8)	-0·9 (-1·3–-0·6)	-1·1 (-1·6–-0·7)	-0·6 (-1·4–0·2)
Child underweight	2·3 (1·4–3·2)	2·2 (1·4–3·0)	1·8 (1·2–2·5)	1·7 (1·1–2·3)	-1·0 (-1·5–-0·5)	-1·2 (-1·9–-0·5)	-0·5 (-1·5–0·5)
Child wasting	0·8 (0·5–1·2)	0·7 (0·5–1·1)	0·7 (0·5–1·1)	0·7 (0·5–1·1)	-0·3 (-0·6–0·1)	-0·1 (-0·6–0·3)	-0·2 (-0·9–0·4)
Child stunting	3·3 (2·5–4·1)	3·1 (2·5–3·9)	2·6 (2·0–3·3)	2·4 (1·8–3·0)	-1·0 (-1·6–-0·4)	-1·2 (-2·0–-0·5)	-0·8 (-2·0–0·5)
Low birth weight and short gestation	16·6 (14·3–19·3)	17·3 (14·9–19·8)	18·4 (15·9–21·3)	17·9 (15·4–20·3)	0·2 (0·0–0·5)	0·1 (-0·3–0·6)	-0·3 (-1·0–0·5)
Short gestation	28·6 (25·1–32·9)	28·2 (24·7–32·3)	28·8 (25·4–33·7)	28·0 (24·6–32·9)	-0·1 (-0·4–0·2)	0·0 (-0·5–0·4)	-0·3 (-1·2–0·7)
Low birth weight	12·3 (11·0–13·5)	13·2 (11·8–14·7)	14·3 (12·9–15·8)	13·9 (12·5–15·3)	0·4 (0·1–0·7)	0·3 (-0·2–0·7)	-0·2 (-0·9–0·5)

Iron deficiency	3·5 (2·5–4·9)	3·0 (2·1–4·1)	3·0 (2·2–4·0)	3·1 (2·2–4·3)	-0·4 (-1·2–0·4)	0·1 (-1·0–1·2)	0·1 (-1·6–1·9)
Vitamin A deficiency	0·6 (0·0–1·2)	0·7 (0·0–1·6)	0·5 (0·0–1·0)	0·3 (0·0–0·6)	-2·4 (-4·7–0·0)	-4·6 (-7·9–0·0)	-4·5 (-8·8–0·0)
Zinc deficiency	1·4 (0·0–6·3)	0·9 (0·0–3·5)	0·8 (0·0–3·2)	0·8 (0·0–3·2)	-2·0 (-23·9–14·9)	-0·5 (-4·3–2·1)	0·3 (-2·3–2·6)
Tobacco	42·0 (40·0–43·6)	35·2 (33·6–36·5)	27·7 (26·6–29·0)	23·6 (21·9–25·2)	-1·9 (-2·1–-1·7)	-1·9 (-2·2–-1·6)	-1·5 (-2·1–-0·9)
Smoking	37·9 (35·3–40·4)	31·0 (28·9–33·0)	24·4 (23·0–25·9)	20·4 (18·6–22·3)	-2·0 (-2·3–-1·7)	-2·0 (-2·4–-1·6)	-1·6 (-2·4–-0·9)
Chewing tobacco	1·0 (0·7–1·3)	1·1 (0·8–1·5)	1·2 (0·9–1·6)	1·2 (0·9–1·8)	0·7 (-0·7–2·3)	0·6 (-1·4–2·7)	0·4 (-2·3–3·3)
Second-hand smoke	37·9 (35·6–39·7)	32·9 (30·5–34·5)	26·8 (24·8–28·2)	23·2 (21·1–25·0)	-1·6 (-1·9–-1·3)	-1·7 (-2·0–-1·3)	-1·3 (-1·9–-0·7)
High alcohol use	33·4 (24·9–44·7)	32·6 (24·6–43·7)	33·5 (25·1–44·4)	33·2 (25·0–43·5)	0·0 (-0·9–0·9)	0·1 (-1·0–1·4)	-0·1 (-1·5–1·4)
Drug use	1·1 (0·7–2·0)	2·3 (1·8–2·7)	3·2 (2·4–4·0)	5·0 (3·7–6·4)	4·9 (2·3–7·2)	3·8 (2·7–4·5)	4·1 (3·1–5·0)
Dietary risks	35·5 (26·4–47·1)	38·5 (29·4–50·7)	41·5 (32·7–53·6)	41·7 (32·4–54·3)	0·5 (0·3–0·8)	0·4 (0·1–0·7)	0·0 (-0·4–0·5)
Diet low in fruits	32·7 (27·1–36·1)	29·6 (24·7–32·4)	29·5 (25·0–31·9)	31·0 (25·8–34·9)	-0·2 (-0·7–0·2)	0·2 (-0·4–0·8)	0·5 (-0·5–1·4)
Diet low in vegetables	20·9 (11·9–28·3)	22·0 (12·9–29·8)	26·4 (14·9–33·7)	26·2 (15·4–34·7)	0·7 (0·2–1·4)	0·8 (0·2–1·6)	-0·1 (-1·2–0·8)
Diet low in legumes	35·7 (0·0–45·1)	35·4 (0·0–44·5)	38·7 (0·0–48·3)	39·7 (0·0–50·1)	0·3 (0·0–0·7)	0·5 (0·0–1·0)	0·2 (-0·5–0·9)
Diet low in whole grains	44·1 (36·5–50·4)	46·9 (39·0–53·4)	48·4 (40·4–55·6)	49·4 (40·8–57·4)	0·4 (0·1–0·6)	0·2 (-0·1–0·6)	0·2 (-0·4–0·7)
Diet low in nuts and seeds	9·1 (6·1–12·6)	4·5 (2·5–7·0)	2·7 (1·4–4·7)	3·3 (1·6–5·7)	-3·3 (-5·5–-1·3)	-1·5 (-4·8–1·9)	1·7 (-3·6–7·1)
Diet low in milk	32·4 (28·1–36·9)	29·1 (24·8–34·1)	26·9 (22·5–31·9)	27·2 (22·5–32·0)	-0·6 (-1·1–-0·1)	-0·3 (-1·0–0·3)	0·1 (-0·9–1·0)
Diet high in red meat	50·9 (0·0–70·4)	51·7 (0·0–71·5)	52·3 (0·0–71·8)	52·3 (0·0–71·4)	0·1 (-0·4–0·5)	0·1 (-0·6–0·6)	0·0 (-1·1–0·8)
Diet high in processed meat	47·9 (37·8–54·0)	59·4 (47·0–66·5)	67·0 (53·3–74·6)	65·5 (52·5–74·0)	1·0 (0·6–1·4)	0·5 (-0·1–1·0)	-0·2 (-0·9–0·6)
Diet high in sugar-sweetened beverages	41·0 (32·0–50·2)	55·9 (44·5–64·5)	62·8 (51·0–71·7)	62·3 (50·0–72·6)	1·3 (0·6–2·1)	0·5 (-0·2–1·3)	-0·1 (-1·1–1·1)
Diet low in fibre	33·3 (18·1–40·0)	23·5 (13·2–29·8)	19·6 (11·2–25·4)	17·5 (9·7–23·3)	-2·1 (-3·2–-1·1)	-1·4 (-3·0–-0·2)	-1·0 (-3·4–0·8)
Diet low in calcium	4·9 (3·9–6·0)	4·2 (3·4–5·2)	4·3 (3·4–5·2)	4·3 (3·4–5·2)	-0·4 (-0·6–-0·2)	0·0 (-0·1–0·2)	0·0 (-0·2–0·2)
Diet low in seafood omega-3 fatty acids	30·0 (22·0–39·3)	36·7 (28·4–46·9)	38·3 (29·5–49·1)	34·4 (25·0–44·6)	0·4 (-0·1–1·0)	-0·3 (-1·0–0·3)	-1·0 (-2·0–-0·2)
Diet low in omega-6 polyunsaturated fatty acids	29·7 (19·6–38·9)	25·2 (17·1–33·3)	18·4 (12·9–24·6)	17·7 (12·1–23·3)	-1·7 (-2·5–-0·9)	-1·7 (-2·8–-0·8)	-0·4 (-1·3–0·5)
Diet high in trans fatty acids	71·5 (64·0–78·9)	71·6 (63·4–78·7)	63·4 (56·3–71·9)	0·0 (0·0–0·0)	-65·8 (-66·1–-65·4)	-97·1 (-97·5–-96·5)	-184·2 (-185·4–-183·2)
Diet high in sodium	23·2 (0·6–68·2)	27·9 (1·8–73·8)	30·8 (3·4–74·6)	31·3 (3·2–77·0)	1·0 (0·2–6·2)	0·6 (-0·1–3·6)	0·2 (-2·3–1·9)
Intimate partner violence	26·9 (14·0–34·8)	26·9 (15·5–34·1)	24·2 (14·9–30·2)	22·9 (13·1–31·1)	-0·5 (-1·9–0·7)	-0·8 (-2·7–0·8)	-0·5 (-2·9–1·6)
Childhood sexual abuse and bullying	9·2 (6·0–14·4)	11·1 (7·3–17·2)	12·7 (8·3–19·8)	10·5 (7·1–15·9)	0·4 (-0·1–1·1)	-0·3 (-1·1–0·6)	-1·7 (-3·2–-0·2)
Childhood sexual abuse	9·0 (7·4–10·8)	10·2 (8·5–12·1)	11·7 (10·1–13·4)	11·0 (9·1–13·3)	0·6 (-0·2–1·4)	0·3 (-0·7–1·4)	-0·6 (-2·0–0·9)
Bullying victimization	8·0 (3·5–16·1)	10·0 (4·6–19·4)	11·4 (5·1–22·1)	8·6 (3·9–16·9)	0·2 (-0·5–1·0)	-0·7 (-1·7–0·3)	-2·6 (-4·4–-0·6)
Unsafe sex	--	--	--	--	--	--	--
Low physical activity	19·7 (14·7–25·3)	18·7 (14·4–23·6)	19·4 (15·1–23·9)	22·0 (16·5–27·9)	0·4 (-0·6–1·2)	0·8 (-0·4–1·9)	1·1 (-0·8–2·7)
**Metabolic risks**	**24·1 (21·0–27·2)**	**29·0 (25·6–32·1)**	**32·4 (28·4–35·4)**	**35·9 (31·4–39·3)**	**1·3 (1·0–1·6)**	**1·0 (0·7–1·4)**	**1·0 (0·5–1·4)**
High fasting plasma glucose	12·0 (9·3–14·6)	15·1 (11·8–18·1)	19·8 (15·0–23·7)	24·0 (17·8–29·0)	2·2 (1·5–2·9)	2·2 (1·3–3·1)	1·8 (0·6–3·0)
High LDL cholesterol	64·0 (45·1–87·2)	54·0 (37·3–74·7)	48·2 (32·6–66·8)	46·7 (31·5–65·0)	-1·0 (-1·2–-0·8)	-0·7 (-0·9–-0·5)	-0·3 (-0·6–0·0)
High systolic blood pressure	28·1 (18·8–39·3)	23·8 (16·6–32·8)	17·5 (11·4–25·5)	24·7 (15·9–35·8)	-0·4 (-1·3–0·4)	0·2 (-1·0–1·3)	3·1 (0·9–5·5)
High body-mass index	27·4 (22·9–31·8)	33·3 (28·2–37·2)	37·0 (31·4–41·1)	40·5 (34·2–44·5)	1·3 (0·9–1·6)	0·9 (0·6–1·3)	0·8 (0·4–1·3)
Low bone mineral density	21·5 (15·4–28·4)	19·4 (13·7–26·1)	19·9 (13·9–26·9)	21·7 (15·5–28·6)	0·0 (-0·4–0·6)	0·5 (-0·1–1·4)	0·8 (-0·6–2·6)
Kidney dysfunction	2·8 (2·3–3·7)	2·9 (2·4–3·8)	2·8 (2·3–3·8)	2·9 (2·4–3·9)	0·1 (0·0–0·2)	0·1 (-0·1–0·2)	0·3 (0·0–0·6)

Delaware

Risk Names	SEV 1990	SEV 2000	SEV 2010	SEV 2021	Annualised rate of change 1990 to 2021	Annualised rate of change 2000 to 2021	Annualised rate of change 2010 to 2021
**All risk factors**	**28·0 (24·9–31·4)**	**27·5 (24·5–30·6)**	**27·9 (24·8–31·2)**	**27·4 (24·4–30·7)**	**-0·1 (-0·4–0·2)**	**0·0 (-0·3–0·3)**	**-0·1 (-0·7–0·4)**
**Environmental/occupational risks**	**19·5 (13·0–30·3)**	**19·1 (13·3–29·9)**	**16·4 (11·1–26·7)**	**14·1 (9·0–23·7)**	**-1·0 (-1·8–-0·3)**	**-1·4 (-1·9–-0·8)**	**-1·3 (-2·0–-0·7)**
Unsafe water, sanitation, and handwashing	1·7 (0·8–2·8)	1·6 (0·7–2·6)	1·3 (0·4–2·1)	1·1 (0·3–1·8)	-1·5 (-4·0–0·4)	-2·0 (-5·1–0·6)	-1·6 (-5·3–2·1)
Unsafe water source	2·5 (1·0–5·2)	2·3 (0·9–4·8)	1·0 (0·4–1·8)	0·8 (0·3–1·5)	-3·6 (-6·5–-0·9)	-5·1 (-9·0–-1·3)	-1·6 (-7·7–4·1)
Unsafe sanitation	5·4 (3·0–9·5)	4·4 (2·4–7·5)	3·3 (1·8–5·7)	2·6 (1·4–4·6)	-2·3 (-4·5–-0·1)	-2·4 (-5·2–0·3)	-2·0 (-5·4–1·6)
No access to handwashing facility	1·5 (0·5–2·8)	1·6 (0·5–3·0)	1·6 (0·5–3·0)	1·4 (0·4–2·7)	-0·3 (-3·6–3·1)	-0·6 (-4·3–3·3)	-1·4 (-6·8–4·3)
Air pollution	27·6 (9·1–39·0)	24·8 (10·0–31·9)	13·7 (5·9–23·9)	7·4 (2·6–15·4)	-4·2 (-6·8–-1·9)	-5·7 (-9·2–-3·1)	-5·5 (-9·5–-3·3)
Particulate matter pollution	15·1 (6·5–25·6)	13·0 (9·4–17·2)	8·4 (5·3–12·0)	4·8 (2·1–8·0)	-3·7 (-6·4–-1·2)	-4·7 (-7·3–-3·4)	-4·9 (-9·0–-2·8)
Ambient particulate matter pollution	23·6 (10·0–40·8)	20·1 (15·0–26·0)	12·8 (8·3–18·0)	7·4 (3·3–11·9)	-3·7 (-6·5–-1·2)	-4·8 (-7·4–-3·4)	-5·0 (-9·0–-2·8)
Household air pollution from solid fuels	0·0 (0·0–0·0)	0·0 (0·0–0·0)	0·0 (0·0–0·0)	0·0 (0·0–0·0)	-3·0 (-27·6–-6·0)	-4·1 (-20·4–-4·2)	-4·2 (-10·0–1·4)
Ambient ozone pollution	29·6 (20·3–39·3)	25·3 (17·2–35·6)	20·7 (13·7–30·0)	15·0 (9·0–22·8)	-2·2 (-3·2–-1·3)	-2·5 (-3·9–-1·4)	-3·0 (-5·4–-0·9)
Ambient nitrogen dioxide pollution	79·6 (0·0–100·0)	74·9 (0·0–100·0)	34·8 (0·0–89·6)	17·0 (0·0–59·7)	-5·0 (-14·2–0·0)	-7·1 (-19·4–0·0)	-6·5 (-20·4–0·0)
Non-optimal temperature	27·4 (21·1–37·2)	27·7 (22·5–36·3)	32·5 (25·5–43·6)	29·1 (23·0–38·9)	0·2 (-0·1–0·6)	0·2 (-0·2–0·7)	-1·0 (-1·6–-0·5)
High temperature	20·9 (11·1–34·0)	19·1 (10·5–30·3)	33·2 (21·9–44·9)	27·1 (16·8–38·1)	0·8 (0·2–1·8)	1·7 (0·8–2·9)	-1·8 (-2·8–-1·1)
Low temperature	25·8 (22·0–30·3)	27·0 (23·3–31·1)	28·7 (25·3–32·6)	26·4 (23·0–30·7)	0·1 (-0·1–0·3)	-0·1 (-0·3–0·1)	-0·8 (-1·0–-0·5)
Other environmental risks	27·3 (9·6–45·3)	25·6 (9·9–43·5)	23·1 (9·6–40·9)	20·3 (9·1–37·2)	-1·0 (-1·6–0·0)	-1·1 (-1·9–0·0)	-1·2 (-2·2–0·0)
Residential radon	27·1 (0·0–77·4)	27·1 (0·0–77·4)	27·1 (0·0–77·4)	27·1 (0·0–77·4)	0·0 (0·0–0·0)	0·0 (0·0–0·0)	0·0 (0·0–0·0)
Lead exposure	27·4 (0·0–34·6)	24·8 (0·0–30·9)	21·2 (0·0–26·4)	17·2 (0·0–21·4)	-1·5 (-1·9–0·0)	-1·8 (-2·3–0·0)	-1·9 (-2·7–0·0)
Occupational risks	2·9 (2·4–3·8)	3·0 (2·5–3·8)	2·8 (2·2–3·7)	2·8 (2·2–3·7)	-0·2 (-0·5–0·0)	-0·3 (-0·7–0·0)	0·0 (-0·4–0·5)
Occupational carcinogens	1·0 (0·5–1·9)	1·1 (0·5–1·9)	1·0 (0·5–1·9)	1·0 (0·5–2·0)	0·1 (-0·3–0·4)	-0·1 (-0·6–0·2)	0·2 (-0·5–0·7)
Occupational exposure to asbestos	5·9 (5·3–6·5)	6·0 (5·5–6·5)	5·1 (4·6–5·6)	4·9 (4·0–6·0)	-0·6 (-1·3–0·2)	-0·9 (-1·9–0·1)	-0·4 (-2·3–1·5)
Occupational exposure to arsenic	0·5 (0·0–1·3)	0·5 (0·0–1·3)	0·5 (0·0–1·2)	0·5 (0·0–1·1)	-0·3 (-0·8–0·2)	-0·3 (-1·0–0·3)	0·1 (-0·8–1·1)
Occupational exposure to benzene	1·1 (0·1–3·1)	1·2 (0·1–3·4)	1·2 (0·1–3·4)	1·2 (0·1–3·6)	0·4 (0·0–0·7)	0·1 (-0·3–0·5)	0·3 (-0·3–0·9)
Occupational exposure to beryllium	0·0 (0·0–0·0)	0·0 (0·0–0·0)	0·0 (0·0–0·0)	0·0 (0·0–0·0)	0·2 (0·0–0·5)	0·1 (-0·2–0·4)	0·4 (0·0–0·9)
Occupational exposure to cadmium	0·1 (0·1–0·1)	0·1 (0·1–0·1)	0·1 (0·1–0·1)	0·1 (0·1–0·1)	-0·2 (-0·6–0·2)	-0·2 (-0·8–0·3)	0·2 (-0·6–1·0)
Occupational exposure to chromium	0·2 (0·1–0·2)	0·2 (0·1–0·2)	0·1 (0·1–0·2)	0·1 (0·1–0·2)	-0·1 (-0·5–0·3)	-0·2 (-0·7–0·3)	0·2 (-0·5–0·9)
Occupational exposure to diesel engine exhaust	0·7 (0·6–0·8)	0·7 (0·7–0·8)	0·7 (0·7–0·8)	0·8 (0·7–0·8)	0·3 (-0·1–0·7)	0·2 (-0·3–0·6)	0·6 (-0·1–1·3)
Occupational exposure to formaldehyde	0·3 (0·3–0·3)	0·3 (0·3–0·3)	0·3 (0·3–0·3)	0·3 (0·3–0·3)	-0·3 (-0·7–0·2)	-0·3 (-0·9–0·2)	0·1 (-0·7–0·8)
Occupational exposure to nickel	0·4 (0·0–1·6)	0·4 (0·0–1·6)	0·4 (0·0–1·5)	0·4 (0·0–1·5)	-0·3 (-0·9–0·2)	-0·3 (-1·1–0·4)	0·1 (-1·0–1·1)
Occupational exposure to polycyclic aromatic hydrocarbons	0·3 (0·3–0·3)	0·3 (0·3–0·3)	0·3 (0·3–0·3)	0·3 (0·3–0·3)	0·0 (-0·4–0·3)	-0·1 (-0·6–0·3)	0·2 (-0·4–0·8)
Occupational exposure to silica	3·5 (0·5–11·7)	3·3 (0·5–10·9)	3·1 (0·5–10·0)	3·2 (0·5–10·3)	-0·3 (-0·9–0·2)	-0·2 (-1·0–0·4)	0·3 (-0·7–1·3)
Occupational exposure to sulphuric acid	0·8 (0·2–2·5)	0·8 (0·2–2·5)	0·7 (0·1–2·3)	0·7 (0·1–2·3)	-0·2 (-0·6–0·2)	-0·2 (-0·8–0·3)	0·2 (-0·6–1·0)
Occupational exposure to trichloroethylene	0·1 (0·1–0·1)	0·1 (0·1–0·1)	0·1 (0·1–0·1)	0·1 (0·1–0·1)	0·1 (-0·2–0·4)	0·0 (-0·4–0·3)	0·4 (-0·2–0·9)
Occupational asthmagens	17·2 (15·2–19·6)	17·4 (15·3–19·6)	16·0 (14·1–18·2)	16·4 (14·4–18·9)	-0·2 (-0·5–0·2)	-0·3 (-0·8–0·2)	0·2 (-0·5–0·9)
Occupational particulate matter, gases, and fumes	6·7 (5·1–8·8)	6·4 (4·9–8·3)	5·9 (4·5–7·6)	5·5 (4·2–7·0)	-0·6 (-0·9–-0·4)	-0·8 (-1·1–-0·4)	-0·8 (-1·2–-0·4)
Occupational noise	6·7 (6·1–7·3)	6·7 (6·2–7·3)	6·5 (6·1–7·1)	6·3 (5·9–6·8)	-0·2 (-0·4–0·0)	-0·3 (-0·5–-0·1)	-0·3 (-0·6–-0·1)
Occupational injuries	--	--	--	--	--	--	--
Occupational ergonomic factors	8·0 (6·6–9·5)	8·1 (6·8–9·7)	7·3 (6·1–8·9)	7·5 (6·2–9·2)	-0·2 (-0·6–0·2)	-0·3 (-0·9–0·2)	0·3 (-0·4–1·0)
**Behavioural risks**	**31·5 (28·0–35·4)**	**30·0 (26·9–33·6)**	**28·0 (24·9–31·8)**	**26·0 (22·6–29·5)**	**-0·6 (-1·0–-0·3)**	**-0·7 (-1·2–-0·2)**	**-0·7 (-1·4–0·0)**
Child and maternal malnutrition	10·5 (7·2–14·6)	9·0 (6·1–12·4)	9·1 (6·4–12·5)	9·2 (6·3–12·7)	-0·4 (-1·2–0·4)	0·1 (-1·2–1·2)	0·1 (-1·5–1·6)
Suboptimal breastfeeding	68·7 (66·1–71·5)	66·6 (64·2–69·1)	62·8 (60·2–65·4)	62·2 (59·2–65·2)	-0·3 (-0·5–-0·2)	-0·3 (-0·5–-0·1)	-0·1 (-0·4–0·2)
Non-exclusive breastfeeding	64·0 (56·7–71·6)	59·4 (53·6–65·7)	48·5 (42·5–55·5)	46·3 (39·6–53·3)	-1·0 (-1·5–-0·6)	-1·2 (-1·9–-0·6)	-0·4 (-1·4–0·5)
Discontinued breastfeeding	88·7 (86·7–91·1)	86·3 (83·9–88·8)	84·3 (81·3–87·4)	84·5 (81·6–87·8)	-0·2 (-0·3–0·0)	-0·1 (-0·3–0·1)	0·0 (-0·3–0·3)
Child growth failure	1·3 (0·4–3·0)	1·2 (0·4–2·7)	1·0 (0·3–2·3)	0·9 (0·3–2·1)	-1·0 (-1·4–-0·6)	-1·1 (-1·6–-0·6)	-0·6 (-1·4–0·2)
Child underweight	2·7 (1·7–3·8)	2·5 (1·6–3·4)	2·1 (1·3–2·9)	2·0 (1·1–2·7)	-1·0 (-1·6–-0·5)	-1·1 (-1·9–-0·5)	-0·6 (-1·7–0·5)
Child wasting	0·9 (0·5–1·3)	0·8 (0·5–1·3)	0·8 (0·5–1·3)	0·8 (0·5–1·2)	-0·3 (-0·7–0·0)	-0·1 (-0·6–0·3)	-0·3 (-0·8–0·4)
Child stunting	3·7 (2·9–4·7)	3·4 (2·7–4·3)	2·9 (2·3–3·7)	2·7 (2·1–3·3)	-1·1 (-1·7–-0·5)	-1·2 (-2·0–-0·5)	-0·9 (-2·0–0·3)
Low birth weight and short gestation	20·7 (17·9–23·9)	22·9 (20·1–26·1)	23·9 (20·8–27·4)	24·1 (21·0–27·4)	0·5 (0·2–0·7)	0·2 (-0·1–0·6)	0·1 (-0·6–0·8)
Short gestation	38·9 (33·7–44·3)	42·7 (37·6–48·6)	43·3 (38·0–49·3)	42·6 (37·1–47·8)	0·3 (0·0–0·6)	0·0 (-0·5–0·4)	-0·2 (-1·0–0·6)
Low birth weight	16·6 (14·8–18·5)	17·6 (16·0–19·5)	18·5 (16·8–20·5)	19·0 (17·1–20·9)	0·4 (0·2–0·7)	0·3 (0·0–0·7)	0·2 (-0·5–0·9)

Iron deficiency	3·8 (2·7–5·2)	3·2 (2·3–4·4)	3·3 (2·4–4·5)	3·3 (2·3–4·5)	-0·4 (-1·2–0·3)	0·1 (-1·0–1·2)	0·0 (-1·5–1·5)
Vitamin A deficiency	0·9 (0·0–1·8)	1·2 (0·0–2·6)	0·7 (0·0–1·6)	0·5 (0·0–1·0)	-1·9 (-4·3–0·3)	-4·4 (-7·5–0·0)	-4·0 (-7·9–0·0)
Zinc deficiency	1·7 (0·0–7·3)	1·0 (0·0–4·2)	0·9 (0·0–3·8)	1·0 (0·0–3·9)	-1·8 (-41·6–0·6)	-0·2 (-1·3–7·3)	0·6 (-2·7–64·7)
Tobacco	42·6 (40·7–44·4)	37·7 (36·1–39·1)	30·6 (29·4–31·8)	26·8 (25·0–28·7)	-1·5 (-1·7–-1·3)	-1·6 (-2·0–-1·3)	-1·2 (-1·8–-0·7)
Smoking	39·9 (37·1–42·5)	34·6 (32·3–36·6)	27·7 (26·0–29·4)	24·0 (21·9–26·0)	-1·6 (-2·0–-1·3)	-1·7 (-2·2–-1·3)	-1·3 (-2·1–-0·6)
Chewing tobacco	1·0 (0·8–1·4)	1·1 (0·9–1·5)	1·2 (0·9–1·7)	1·2 (0·9–1·7)	0·5 (-0·8–1·8)	0·3 (-1·6–2·1)	0·2 (-2·4–2·8)
Second-hand smoke	34·5 (32·7–36·0)	30·9 (28·7–32·1)	25·5 (23·7–26·5)	22·7 (20·8–24·4)	-1·4 (-1·6–-1·1)	-1·5 (-1·8–-1·1)	-1·1 (-1·7–-0·4)
High alcohol use	28·6 (20·2–39·1)	27·8 (20·0–37·4)	29·0 (21·1–39·3)	28·6 (20·1–39·0)	0·0 (-1·2–1·1)	0·1 (-1·3–1·4)	-0·1 (-2·1–1·7)
Drug use	1·0 (0·6–1·8)	1·5 (1·3–1·8)	1·9 (1·7–2·3)	5·5 (4·0–7·0)	5·5 (2·7–7·9)	6·3 (4·2–7·5)	9·5 (6·4–11·2)
Dietary risks	37·7 (28·1–49·0)	41·0 (31·4–53·0)	44·9 (34·5–57·2)	45·2 (35·3–57·2)	0·6 (0·3–0·9)	0·5 (0·1–0·8)	0·1 (-0·4–0·5)
Diet low in fruits	36·8 (31·6–40·0)	34·3 (29·8–37·1)	36·2 (32·3–39·2)	38·4 (33·1–43·1)	0·1 (-0·2–0·5)	0·5 (0·1–1·1)	0·5 (-0·2–1·3)
Diet low in vegetables	29·3 (17·6–37·4)	33·8 (20·0–41·6)	45·5 (24·3–53·4)	45·5 (25·2–54·3)	1·4 (1·0–1·9)	1·4 (0·9–1·9)	0·0 (-0·6–0·7)
Diet low in legumes	37·2 (0·0–46·5)	37·2 (0·0–46·4)	41·4 (0·0–51·6)	42·6 (0·0–53·7)	0·4 (0·0–0·8)	0·6 (0·0–1·1)	0·3 (-0·5–1·0)
Diet low in whole grains	45·0 (37·6–51·4)	47·4 (39·3–54·2)	49·4 (41·0–56·9)	50·1 (41·9–57·5)	0·3 (0·1–0·6)	0·3 (-0·1–0·6)	0·1 (-0·4–0·7)
Diet low in nuts and seeds	11·3 (8·1–15·4)	6·3 (3·9–9·1)	4·6 (2·8–7·0)	5·5 (3·1–8·5)	-2·3 (-4·2–-0·4)	-0·6 (-3·4–2·0)	1·6 (-2·3–5·4)
Diet low in milk	34·8 (30·3–39·7)	31·9 (27·6–37·0)	31·0 (26·7–35·7)	31·6 (26·8–37·1)	-0·3 (-0·8–0·2)	0·0 (-0·7–0·6)	0·2 (-0·7–1·1)
Diet high in red meat	50·2 (0·0–68·9)	50·4 (0·0–68·7)	50·5 (0·0–69·1)	50·2 (0·0–69·0)	0·0 (-0·4–0·4)	0·0 (-0·6–0·6)	-0·1 (-1·0–0·9)
Diet high in processed meat	47·2 (37·3–53·0)	58·2 (47·1–65·0)	65·6 (54·0–73·4)	63·3 (52·0–71·7)	0·9 (0·5–1·4)	0·4 (-0·2–1·0)	-0·3 (-1·1–0·5)
Diet high in sugar-sweetened beverages	38·3 (28·8–47·0)	52·4 (41·5–61·4)	57·6 (45·4–66·4)	55·7 (44·0–66·1)	1·2 (0·5–2·1)	0·3 (-0·6–1·2)	-0·3 (-1·6–1·0)
Diet low in fibre	36·9 (20·5–44·3)	27·5 (15·5–33·8)	24·6 (13·8–31·2)	22·5 (13·1–28·7)	-1·6 (-2·5–-0·7)	-0·9 (-2·2–0·4)	-0·8 (-2·7–1·2)
Diet low in calcium	5·3 (4·3–6·5)	4·5 (3·7–5·6)	4·9 (3·9–6·0)	5·0 (4·0–6·1)	-0·2 (-0·4–0·0)	0·4 (0·2–0·7)	0·2 (-0·1–0·6)
Diet low in seafood omega-3 fatty acids	34·0 (24·7–43·8)	41·0 (31·6–51·7)	44·4 (34·5–55·2)	40·9 (31·3–51·2)	0·6 (0·1–1·1)	0·0 (-0·6–0·5)	-0·7 (-1·5–-0·1)
Diet low in omega-6 polyunsaturated fatty acids	33·2 (21·6–42·1)	28·7 (19·3–37·2)	21·8 (15·2–28·5)	21·3 (14·7–28·4)	-1·4 (-2·2–-0·7)	-1·4 (-2·3–-0·6)	-0·2 (-1·1–0·8)
Diet high in trans fatty acids	71·5 (64·0–78·3)	71·7 (64·8–78·6)	63·4 (54·7–71·7)	0·0 (0·0–0·0)	-65·8 (-66·1–-65·4)	-97·1 (-97·5–-96·6)	-184·2 (-185·4–-182·9)
Diet high in sodium	23·3 (0·5–69·4)	28·0 (1·9–74·6)	31·1 (3·4–74·9)	31·5 (3·1–77·4)	1·0 (0·3–6·7)	0·6 (-0·1–3·5)	0·1 (-2·6–1·9)
Intimate partner violence	27·9 (13·0–36·0)	27·8 (13·8–35·2)	25·1 (17·1–30·9)	23·8 (13·1–32·0)	-0·5 (-1·9–0·9)	-0·7 (-2·6–1·1)	-0·5 (-2·9–1·6)
Childhood sexual abuse and bullying	9·0 (5·8–14·4)	10·9 (7·0–17·2)	12·5 (8·2–19·7)	9·7 (6·7–14·9)	0·2 (-0·4–0·8)	-0·6 (-1·4–0·3)	-2·3 (-3·8–-0·8)
Childhood sexual abuse	8·7 (7·4–10·3)	9·9 (8·5–11·4)	11·5 (10·0–13·2)	10·5 (8·5–13·0)	0·6 (-0·2–1·4)	0·3 (-0·7–1·4)	-0·9 (-2·3–0·5)
Bullying victimization	7·9 (3·5–15·9)	9·9 (4·5–19·4)	11·2 (5·1–21·9)	7·7 (3·5–15·1)	-0·1 (-0·8–0·6)	-1·2 (-2·2–-0·2)	-3·5 (-5·5–-1·6)
Unsafe sex	--	--	--	--	--	--	--
Low physical activity	22·3 (17·0–28·3)	21·4 (16·6–26·3)	21·2 (16·8–25·8)	23·4 (17·9–29·3)	0·2 (-0·6–1·1)	0·4 (-0·7–1·6)	0·9 (-0·7–2·4)
**Metabolic risks**	**27·1 (23·4–30·4)**	**30·9 (26·8–33·9)**	**35·3 (31·2–38·1)**	**38·5 (34·2–41·6)**	**1·1 (0·8–1·4)**	**1·0 (0·7–1·4)**	**0·8 (0·3–1·3)**
High fasting plasma glucose	13·7 (10·5–16·6)	17·6 (13·2–20·8)	22·3 (16·9–25·8)	25·0 (19·2–29·7)	1·9 (1·3–2·6)	1·7 (0·9–2·5)	1·1 (-0·1–2·2)
High LDL cholesterol	63·8 (44·7–87·7)	53·9 (37·1–74·8)	48·2 (32·6–67·0)	46·6 (31·1–65·0)	-1·0 (-1·2–-0·8)	-0·7 (-0·9–-0·5)	-0·3 (-0·6–-0·1)
High systolic blood pressure	29·3 (19·6–40·4)	25·2 (17·8–34·3)	18·9 (12·4–27·1)	26·3 (16·7–37·0)	-0·4 (-1·3–0·6)	0·2 (-0·9–1·4)	3·0 (0·9–5·5)
High body-mass index	30·7 (25·8–35·1)	35·2 (29·6–39·3)	40·3 (34·4–44·0)	43·6 (37·2–47·8)	1·1 (0·8–1·4)	1·0 (0·7–1·4)	0·7 (0·3–1·1)
Low bone mineral density	21·3 (15·1–28·1)	19·3 (13·1–26·1)	20·2 (14·3–27·1)	22·4 (16·5–29·7)	0·2 (-0·3–0·7)	0·7 (0·0–1·6)	1·0 (-0·4–2·5)
Kidney dysfunction	2·9 (2·4–3·8)	3·0 (2·5–4·0)	3·0 (2·4–4·0)	3·1 (2·5–4·0)	0·2 (0·0–0·3)	0·1 (-0·1–0·3)	0·3 (0·0–0·6)

Florida

Risk Names	SEV 1990	SEV 2000	SEV 2010	SEV 2021	Annualised rate of change 1990 to 2021	Annualised rate of change 2000 to 2021	Annualised rate of change 2010 to 2021
**All risk factors**	**27·8 (24·5–31·2)**	**26·9 (23·9–29·9)**	**27·1 (24·3–30·4)**	**27·0 (23·7–30·3)**	**-0·1 (-0·4–0·2)**	**0·0 (-0·4–0·4)**	**-0·1 (-0·6–0·5)**
**Environmental/occupational risks**	**12·5 (7·8–17·7)**	**12·8 (8·1–17·1)**	**12·1 (7·5–18·0)**	**8·2 (4·3–11·7)**	**-1·4 (-2·3–-0·5)**	**-2·1 (-3·1–-1·3)**	**-3·6 (-4·9–-2·6)**
Unsafe water, sanitation, and handwashing	2·0 (1·0–3·2)	1·8 (0·8–2·9)	1·3 (0·5–2·2)	1·1 (0·4–1·9)	-1·9 (-4·3–0·2)	-2·3 (-5·2–0·4)	-1·7 (-5·3–2·2)
Unsafe water source	2·8 (1·0–5·9)	2·5 (0·9–5·1)	1·0 (0·4–1·9)	0·8 (0·4–1·5)	-4·0 (-6·7–-1·5)	-5·3 (-9·2–-1·9)	-1·8 (-7·5–3·6)
Unsafe sanitation	6·6 (3·4–10·8)	5·0 (2·6–8·2)	3·6 (1·9–6·2)	2·8 (1·5–4·9)	-2·8 (-5·0–-0·7)	-2·8 (-5·5–0·0)	-2·3 (-5·7–1·2)
No access to handwashing facility	1·7 (0·6–3·2)	1·7 (0·5–3·2)	1·7 (0·5–3·1)	1·4 (0·4–2·7)	-0·5 (-3·4–2·7)	-0·8 (-4·5–3·2)	-1·4 (-6·7–4·0)
Air pollution	21·0 (5·3–32·3)	20·0 (7·6–28·7)	9·7 (3·3–19·2)	5·5 (0·6–13·9)	-4·3 (-9·3–-1·9)	-6·1 (-13·2–-3·3)	-5·2 (-15·7–-2·9)
Particulate matter pollution	10·3 (3·1–19·6)	10·4 (7·1–14·1)	5·6 (2·8–8·9)	2·6 (0·4–5·5)	-4·5 (-10·4–-1·0)	-6·6 (-14·3–-4·3)	-7·0 (-19·0–-3·9)
Ambient particulate matter pollution	15·9 (4·8–29·6)	15·9 (11·2–21·2)	8·5 (4·4–13·3)	3·9 (0·6–8·1)	-4·5 (-10·4–-1·1)	-6·7 (-14·4–-4·3)	-7·0 (-19·1–-4·0)
Household air pollution from solid fuels	0·0 (0·0–0·0)	0·0 (0·0–0·0)	0·0 (0·0–0·0)	0·0 (0·0–0·0)	-3·6 (-29·1–-6·4)	-4·1 (-28·7–-7·1)	-4·5 (-23·1–-2·8)
Ambient ozone pollution	14·5 (9·6–21·8)	17·8 (12·6–25·4)	14·0 (9·2–21·1)	4·0 (1·6–7·7)	-4·2 (-6·0–-3·0)	-7·1 (-9·9–-5·2)	-11·4 (-16·6–-7·6)
Ambient nitrogen dioxide pollution	67·4 (0·0–100·0)	61·0 (0·0–100·0)	26·5 (0·0–75·0)	18·3 (0·0–62·5)	-4·2 (-13·6–0·0)	-5·7 (-18·9–0·0)	-3·4 (-16·0–0·0)
Non-optimal temperature	20·6 (13·9–28·9)	22·8 (17·1–31·0)	37·7 (29·6–46·8)	22·3 (15·9–30·5)	0·2 (0·0–0·9)	-0·1 (-0·6–0·5)	-4·8 (-6·0–-3·3)
High temperature	21·5 (13·4–30·5)	19·3 (12·2–28·0)	29·6 (21·0–38·5)	21·3 (14·1–30·0)	0·0 (-0·3–0·6)	0·5 (-0·1–1·3)	-3·0 (-4·1–-1·9)
Low temperature	14·0 (11·5–17·7)	19·9 (16·8–23·1)	35·1 (30·9–39·6)	16·7 (14·8–19·9)	0·6 (0·3–1·0)	-0·8 (-1·1–-0·3)	-6·7 (-8·0–-5·0)
Other environmental risks	28·3 (5·5–44·5)	26·1 (5·5–41·4)	22·9 (5·5–38·3)	19·4 (5·5–33·7)	-1·2 (-1·8–0·0)	-1·4 (-2·2–0·0)	-1·5 (-2·5–0·0)
Residential radon	18·9 (0·0–63·5)	18·9 (0·0–63·5)	18·9 (0·0–63·5)	18·9 (0·0–63·5)	0·0 (0·0–0·0)	0·0 (0·0–0·0)	0·0 (0·0–0·0)
Lead exposure	32·4 (0·0–40·6)	29·3 (0·0–36·5)	24·6 (0·0–30·8)	19·5 (0·0–24·4)	-1·6 (-2·1–0·0)	-1·9 (-2·4–0·0)	-2·1 (-2·9–0·0)
Occupational risks	2·6 (2·2–3·5)	2·7 (2·2–3·6)	2·5 (2·0–3·4)	2·6 (2·0–3·3)	-0·1 (-0·4–0·2)	-0·3 (-0·7–0·1)	0·2 (-0·3–0·6)
Occupational carcinogens	0·9 (0·4–1·7)	1·0 (0·4–1·9)	0·9 (0·4–1·8)	0·9 (0·4–1·8)	0·1 (-0·3–0·4)	-0·1 (-0·6–0·2)	0·3 (-0·4–0·7)
Occupational exposure to asbestos	3·7 (3·2–4·3)	4·1 (3·7–4·5)	3·4 (3·0–3·7)	3·0 (2·4–3·8)	-0·7 (-1·5–0·1)	-1·5 (-2·7–-0·4)	-1·1 (-3·2–1·0)
Occupational exposure to arsenic	0·4 (0·0–1·0)	0·4 (0·0–1·1)	0·4 (0·0–1·0)	0·4 (0·0–1·0)	-0·1 (-0·5–0·4)	-0·2 (-0·7–0·5)	0·3 (-0·5–1·2)
Occupational exposure to benzene	1·1 (0·1–3·0)	1·2 (0·1–3·3)	1·1 (0·1–3·3)	1·2 (0·1–3·4)	0·3 (0·0–0·7)	0·1 (-0·4–0·5)	0·4 (-0·2–1·0)
Occupational exposure to beryllium	0·0 (0·0–0·0)	0·0 (0·0–0·0)	0·0 (0·0–0·0)	0·0 (0·0–0·0)	0·2 (0·0–0·4)	0·1 (-0·2–0·4)	0·6 (0·1–1·0)
Occupational exposure to cadmium	0·1 (0·0–0·1)	0·1 (0·0–0·1)	0·0 (0·0–0·1)	0·1 (0·0–0·1)	0·0 (-0·4–0·4)	-0·1 (-0·6–0·4)	0·4 (-0·4–1·1)
Occupational exposure to chromium	0·1 (0·1–0·1)	0·1 (0·1–0·1)	0·1 (0·1–0·1)	0·1 (0·1–0·1)	0·0 (-0·3–0·4)	-0·1 (-0·5–0·4)	0·4 (-0·2–1·0)
Occupational exposure to diesel engine exhaust	0·7 (0·6–0·8)	0·7 (0·7–0·8)	0·7 (0·6–0·7)	0·7 (0·7–0·8)	0·1 (-0·2–0·5)	0·1 (-0·3–0·6)	0·7 (0·0–1·4)
Occupational exposure to formaldehyde	0·3 (0·2–0·3)	0·3 (0·2–0·3)	0·2 (0·2–0·3)	0·3 (0·2–0·3)	-0·1 (-0·5–0·3)	-0·2 (-0·7–0·3)	0·2 (-0·5–1·0)
Occupational exposure to nickel	0·3 (0·0–1·4)	0·3 (0·0–1·3)	0·3 (0·0–1·2)	0·3 (0·0–1·3)	-0·2 (-0·7–0·3)	-0·2 (-0·9–0·5)	0·3 (-0·6–1·3)
Occupational exposure to polycyclic aromatic hydrocarbons	0·3 (0·2–0·3)	0·3 (0·3–0·3)	0·3 (0·3–0·3)	0·3 (0·3–0·3)	0·1 (-0·2–0·4)	-0·1 (-0·5–0·4)	0·4 (-0·2–1·0)
Occupational exposure to silica	3·0 (0·5–9·5)	2·9 (0·4–9·3)	2·7 (0·4–8·8)	2·8 (0·5–9·1)	-0·2 (-0·7–0·3)	-0·1 (-0·7–0·5)	0·4 (-0·5–1·4)
Occupational exposure to sulphuric acid	0·7 (0·1–2·2)	0·7 (0·1–2·2)	0·6 (0·1–2·0)	0·6 (0·1–2·1)	-0·1 (-0·5–0·4)	-0·1 (-0·7–0·4)	0·4 (-0·5–1·0)
Occupational exposure to trichloroethylene	0·1 (0·1–0·1)	0·1 (0·1–0·1)	0·1 (0·1–0·1)	0·1 (0·1–0·1)	0·1 (-0·2–0·4)	0·0 (-0·4–0·4)	0·5 (0·0–1·0)
Occupational asthmagens	15·6 (13·6–17·8)	16·0 (13·9–18·2)	14·7 (12·9–16·7)	15·4 (13·4–17·5)	-0·1 (-0·5–0·3)	-0·2 (-0·7–0·3)	0·4 (-0·3–1·0)
Occupational particulate matter, gases, and fumes	5·8 (4·5–7·9)	5·6 (4·3–7·6)	5·3 (4·1–7·0)	4·9 (3·8–6·4)	-0·5 (-0·8–-0·3)	-0·7 (-1·0–-0·4)	-0·7 (-1·1–-0·3)
Occupational noise	6·2 (5·7–6·9)	6·3 (5·8–6·9)	6·2 (5·7–6·7)	6·0 (5·6–6·4)	-0·1 (-0·3–0·0)	-0·2 (-0·4–-0·1)	-0·3 (-0·5–-0·1)
Occupational injuries	--	--	--	--	--	--	--
Occupational ergonomic factors	7·6 (6·3–9·2)	7·6 (6·4–9·3)	6·8 (5·6–8·2)	7·0 (5·8–8·7)	-0·2 (-0·7–0·2)	-0·4 (-0·9–0·1)	0·3 (-0·4–1·0)
**Behavioural risks**	**30·8 (27·3–34·6)**	**28·7 (25·4–32·5)**	**26·5 (23·4–30·1)**	**24·4 (21·0–28·4)**	**-0·8 (-1·2–-0·4)**	**-0·8 (-1·3–-0·3)**	**-0·8 (-1·4–-0·2)**
Child and maternal malnutrition	10·3 (6·9–14·3)	8·8 (5·9–12·3)	8·8 (5·9–12·1)	9·1 (6·2–12·5)	-0·4 (-1·2–0·4)	0·2 (-1·0–1·3)	0·2 (-1·5–1·9)
Suboptimal breastfeeding	68·9 (66·4–71·4)	66·8 (64·5–69·2)	62·9 (60·3–65·5)	62·2 (59·5–65·3)	-0·3 (-0·5–-0·2)	-0·3 (-0·5–-0·1)	-0·1 (-0·4–0·2)
Non-exclusive breastfeeding	64·4 (56·8–71·6)	59·8 (53·4–66·1)	48·6 (42·3–55·7)	46·3 (39·3–53·2)	-1·1 (-1·7–-0·5)	-1·2 (-1·9–-0·6)	-0·4 (-1·4–0·5)
Discontinued breastfeeding	88·8 (86·4–91·2)	86·4 (84·1–88·9)	84·5 (81·6–87·4)	84·6 (81·5–88·0)	-0·2 (-0·3–0·0)	-0·1 (-0·3–0·1)	0·0 (-0·3–0·3)
Child growth failure	1·3 (0·4–3·0)	1·2 (0·4–2·7)	1·0 (0·3–2·2)	1·0 (0·3–2·1)	-1·0 (-1·4–-0·7)	-1·0 (-1·6–-0·5)	-0·5 (-1·3–0·2)
Child underweight	2·7 (1·7–3·7)	2·5 (1·6–3·3)	2·1 (1·3–2·8)	2·0 (1·2–2·7)	-1·0 (-1·5–-0·6)	-1·1 (-1·8–-0·4)	-0·5 (-1·5–0·5)
Child wasting	0·9 (0·6–1·4)	0·8 (0·5–1·2)	0·8 (0·5–1·3)	0·8 (0·5–1·2)	-0·3 (-0·6–0·0)	-0·1 (-0·5–0·4)	-0·2 (-0·9–0·5)
Child stunting	3·8 (3·0–4·7)	3·4 (2·7–4·3)	2·9 (2·3–3·8)	2·7 (2·1–3·4)	-1·1 (-1·6–-0·5)	-1·2 (-2·0–-0·4)	-0·8 (-2·0–0·3)
Low birth weight and short gestation	20·6 (18·1–23·2)	22·5 (20·0–25·3)	24·2 (21·3–27·5)	23·8 (21·0–26·7)	0·5 (0·2–0·7)	0·3 (-0·2–0·7)	-0·1 (-0·8–0·6)
Short gestation	36·1 (32·2–40·5)	39·7 (35·3–44·4)	42·1 (37·4–47·5)	40·8 (36·1–45·9)	0·4 (0·1–0·7)	0·1 (-0·3–0·6)	-0·3 (-1·1–0·7)
Low birth weight	15·7 (14·4–17·1)	16·9 (15·5–18·4)	18·0 (16·5–19·6)	18·0 (16·5–19·5)	0·4 (0·2–0·7)	0·3 (-0·1–0·7)	0·0 (-0·7–0·7)

Iron deficiency	3·6<br>(2·5–5·0)	3·1<br>(2·1–4·3)	3·2<br>(2·2–4·3)	3·2<br>(2·3–4·4)	-0·4<br>(-1·1–0·4)	0·2<br>(-0·9–1·2)	0·2<br>(-1·3–1·8)
Vitamin A deficiency	1·1<br>(0·0–2·3)	1·4<br>(0·0–3·1)	0·8<br>(0·0–1·8)	0·5<br>(0·0–1·1)	-2·5<br>(-4·8–0·0)	-5·1<br>(-8·1–0·0)	-4·6<br>(-8·8–0·0)
Zinc deficiency	1·8<br>(0·0–7·8)	1·1<br>(0·0–4·6)	1·0<br>(0·0–4·2)	1·0<br>(0·0–4·3)	-1·8<br>(-41·0–0·3)	-0·3<br>(-2·5–3·9)	0·4<br>(-4·6–11·3)
Tobacco	42·3<br>(40·6–43·7)	36·9<br>(35·5–38·2)	29·1<br>(27·9–30·2)	24·6<br>(23·0–26·2)	-1·7<br>(-2·0–-1·5)	-1·9<br>(-2·2–-1·6)	-1·5<br>(-2·1–-0·9)
Smoking	40·0<br>(37·5–42·0)	34·1<br>(32·0–36·0)	27·1<br>(25·4–28·9)	22·7<br>(20·8–24·8)	-1·8<br>(-2·1–-1·5)	-1·9<br>(-2·4–-1·5)	-1·6<br>(-2·3–-0·8)
Chewing tobacco	1·9<br>(1·5–2·4)	1·9<br>(1·5–2·5)	2·0<br>(1·4–2·5)	2·0<br>(1·4–2·6)	0·2<br>(-1·0–1·4)	0·1<br>(-1·6–1·8)	0·0<br>(-2·6–2·6)
Second-hand smoke	33·4<br>(31·7–34·7)	29·7<br>(27·9–30·8)	23·6<br>(22·1–24·6)	20·4<br>(18·7–21·9)	-1·6<br>(-1·8–-1·4)	-1·8<br>(-2·1–-1·4)	-1·3<br>(-1·9–-0·7)
High alcohol use	27·5<br>(18·9–38·8)	26·1<br>(18·4–35·8)	27·1<br>(19·5–37·1)	26·9<br>(18·6–37·4)	-0·1<br>(-1·2–1·1)	0·1<br>(-1·3–1·6)	-0·1<br>(-1·9–1·6)
Drug use	0·9<br>(0·4–1·7)	1·1<br>(0·9–1·6)	1·8<br>(1·5–2·1)	5·2<br>(3·7–6·7)	5·7<br>(2·8–8·7)	7·4<br>(4·1–9·2)	9·8<br>(6·3–11·9)
Dietary risks	36·8<br>(27·5–47·6)	39·9<br>(30·4–51·8)	42·4<br>(33·0–54·7)	42·3<br>(33·1–54·9)	0·5<br>(0·2–0·7)	0·3<br>(0·0–0·6)	0·0<br>(-0·5–0·4)
Diet low in fruits	35·4<br>(31·2–39·0)	32·8<br>(28·2–35·9)	33·2<br>(29·3–36·3)	34·8<br>(29·1–39·1)	-0·1<br>(-0·4–0·3)	0·3<br>(-0·3–0·8)	0·4<br>(-0·4–1·3)
Diet low in vegetables	26·8<br>(16·7–34·2)	28·7<br>(17·2–36·3)	32·5<br>(19·3–40·7)	32·1<br>(19·4–40·6)	0·6<br>(0·2–1·0)	0·5<br>(0·1–1·1)	-0·1<br>(-0·9–0·7)
Diet low in legumes	37·7<br>(0·0–47·5)	38·1<br>(0·0–47·7)	42·1<br>(0·0–52·3)	43·1<br>(0·0–54·2)	0·4<br>(0·0–0·8)	0·6<br>(0·0–1·1)	0·2<br>(-0·5–0·9)
Diet low in whole grains	44·5<br>(37·0–50·7)	47·8<br>(40·4–54·9)	49·4<br>(41·3–56·5)	49·5<br>(41·5–56·6)	0·3<br>(0·1–0·6)	0·2<br>(-0·2–0·5)	0·0<br>(-0·4–0·6)
Diet low in nuts and seeds	12·0<br>(8·6–16·0)	7·1<br>(4·7–10·0)	5·1<br>(3·1–7·3)	5·7<br>(3·4–8·5)	-2·4<br>(-4·1–-1·0)	-1·1<br>(-3·4–1·2)	1·0<br>(-2·6–4·5)
Diet low in milk	35·8<br>(31·5–40·3)	33·2<br>(28·7–37·9)	31·8<br>(27·4–36·2)	32·0<br>(27·6–36·4)	-0·4<br>(-0·9–0·1)	-0·2<br>(-0·8–0·5)	0·1<br>(-0·7–1·0)
Diet high in red meat	49·5<br>(0·0–68·0)	49·9<br>(0·0–68·9)	49·9<br>(0·0–68·6)	49·7<br>(0·0–68·3)	0·0<br>(-0·4–0·5)	0·0<br>(-0·6–0·6)	0·0<br>(-0·9–0·9)
Diet high in processed meat	46·4<br>(37·0–52·2)	57·8<br>(46·3–64·7)	64·9<br>(51·4–72·7)	62·5<br>(49·0–70·5)	1·0<br>(0·5–1·4)	0·4<br>(-0·2–0·9)	-0·3<br>(-1·1–0·4)
Diet high in sugar-sweetened beverages	36·4<br>(28·6–44·5)	50·2<br>(39·7–58·1)	55·4<br>(44·1–64·9)	53·9<br>(42·8–63·2)	1·3<br>(0·5–2·1)	0·3<br>(-0·5–1·2)	-0·3<br>(-1·6–1·1)
Diet low in fibre	38·1<br>(20·3–46·1)	29·2<br>(15·7–36·3)	25·8<br>(14·1–32·1)	23·0<br>(12·8–29·8)	-1·6<br>(-2·5–-0·8)	-1·1<br>(-2·4–0·0)	-1·0<br>(-3·0–0·7)
Diet low in calcium	5·5<br>(4·4–6·8)	4·7<br>(3·8–5·8)	5·0<br>(4·0–6·1)	5·0<br>(4·0–6·1)	-0·3<br>(-0·5–-0·1)	0·3<br>(0·1–0·6)	0·1<br>(-0·3–0·4)
Diet low in seafood omega-3 fatty acids	35·0<br>(26·2–44·8)	42·8<br>(34·1–53·1)	45·9<br>(35·6–56·9)	41·6<br>(31·7–52·7)	0·6<br>(0·1–1·1)	-0·1<br>(-0·7–0·4)	-0·9<br>(-1·6–-0·3)
Diet low in omega-6 polyunsaturated fatty acids	34·5<br>(22·5–44·0)	30·6<br>(20·7–39·5)	22·8<br>(16·1–29·3)	21·7<br>(15·0–28·3)	-1·5<br>(-2·2–-0·8)	-1·6<br>(-2·4–-0·8)	-0·5<br>(-1·3–0·4)
Diet high in trans fatty acids	71·4<br>(63·9–78·7)	71·9<br>(64·4–78·8)	63·4<br>(55·4–71·4)	0·0<br>(0·0–0·0)	-65·8<br>(-66·1–-65·4)	-97·1<br>(-97·5–-96·6)	-184·2<br>(-185·3–-183·0)
Diet high in sodium	23·5<br>(0·6–71·5)	28·3<br>(1·8–74·5)	31·5<br>(3·5–75·5)	31·8<br>(3·1–78·5)	1·0<br>(0·2–6·0)	0·6<br>(-0·1–4·1)	0·1<br>(-2·9–1·7)
Intimate partner violence	28·2<br>(16·2–35·9)	28·1<br>(16·9–35·2)	25·4<br>(16·9–31·3)	23·9<br>(13·3–32·2)	-0·5<br>(-1·8–0·7)	-0·8<br>(-2·4–0·9)	-0·5<br>(-2·6–1·5)
Childhood sexual abuse and bullying	8·7<br>(5·5–14·0)	10·5<br>(6·8–16·8)	12·0<br>(7·7–19·1)	8·9<br>(6·0–13·9)	0·1<br>(-0·5–0·7)	-0·8<br>(-1·7–0·1)	-2·7<br>(-4·3–-1·1)
Childhood sexual abuse	7·8<br>(6·5–9·2)	8·8<br>(7·7–10·3)	10·1<br>(8·8–11·6)	9·1<br>(7·4–11·2)	0·5<br>(-0·3–1·4)	0·1<br>(-0·9–1·2)	-1·0<br>(-2·4–0·6)
Bullying victimization	8·0<br>(3·5–16·0)	9·9<br>(4·6–19·4)	11·3<br>(5·1–22·0)	7·4<br>(3·2–14·3)	-0·2<br>(-0·9–0·5)	-1·4<br>(-2·4–-0·4)	-3·8<br>(-5·7–-2·0)
Unsafe sex	--	--	--	--	--	--	--
Low physical activity	19·6<br>(14·6–25·1)	18·9<br>(14·5–23·6)	19·0<br>(14·7–23·7)	21·6<br>(16·7–27·8)	0·3<br>(-0·6–1·2)	0·6<br>(-0·5–1·8)	1·2<br>(-0·7–2·9)
**Metabolic risks**	**25·1**<br>**(22·2–28·1)**	**29·7**<br>**(26·4–32·5)**	**33·7**<br>**(29·9–36·4)**	**36·2**<br>**(31·8–39·1)**	**1·2**<br>**(0·9–1·5)**	**0·9**<br>**(0·6–1·2)**	**0·6**<br>**(0·2–1·1)**
High fasting plasma glucose	12·0<br>(9·1–14·5)	15·7<br>(11·7–18·6)	20·8<br>(15·6–24·9)	23·9<br>(18·5–28·9)	2·2<br>(1·6–2·8)	2·0<br>(1·3–2·8)	1·3<br>(0·1–2·4)
High LDL cholesterol	63·8<br>(45·0–86·8)	54·0<br>(37·2–74·5)	48·5<br>(32·7–67·3)	46·8<br>(31·7–65·3)	-1·0<br>(-1·2–-0·8)	-0·7<br>(-0·9–-0·5)	-0·3<br>(-0·6–-0·1)
High systolic blood pressure	29·1<br>(19·9–39·7)	25·2<br>(17·8–34·3)	19·3<br>(12·6–28·0)	26·9<br>(16·9–38·4)	-0·3<br>(-1·2–0·6)	0·3<br>(-0·9–1·4)	3·0<br>(0·8–5·5)
High body-mass index	28·6<br>(24·3–32·7)	34·3<br>(29·4–38·0)	38·7<br>(33·0–42·3)	40·7<br>(34·6–44·4)	1·1<br>(0·9–1·4)	0·8<br>(0·5–1·1)	0·5<br>(0·1–0·9)
Low bone mineral density	21·6<br>(15·6–28·0)	19·6<br>(13·5–26·5)	20·3<br>(14·2–27·4)	22·3<br>(16·1–29·4)	0·1<br>(-0·4–0·6)	0·6<br>(-0·2–1·4)	0·8<br>(-0·6–2·3)
Kidney dysfunction	2·9<br>(2·3–3·8)	2·9<br>(2·4–3·8)	2·9<br>(2·4–3·8)	3·0<br>(2·5–4·0)	0·2<br>(0·1–0·3)	0·1<br>(0·0–0·3)	0·4<br>(0·1–0·7)

Georgia

Risk Names	SEV 1990	SEV 2000	SEV 2010	SEV 2021	Annualised rate of change 1990 to 2021	Annualised rate of change 2000 to 2021	Annualised rate of change 2010 to 2021
**All risk factors**	**25·5 (22·8–28·5)**	**25·9 (23·2–28·8)**	**26·5 (23·6–29·4)**	**26·2 (23·0–29·7)**	**0·1 (-0·2–0·4)**	**0·1 (-0·3–0·4)**	**-0·1 (-0·6–0·4)**
**Environmental/occupational risks**	**18·5 (12·4–27·8)**	**18·5 (12·9–27·1)**	**16·2 (11·0–24·3)**	**12·7 (8·6–19·0)**	**-1·2 (-2·0–-0·5)**	**-1·8 (-2·4–-1·3)**	**-2·2 (-3·0–-1·6)**
Unsafe water, sanitation, and handwashing	2·2 (1·2–3·6)	2·0 (0·9–3·2)	1·5 (0·5–2·5)	1·2 (0·4–1·9)	-2·0 (-4·2–0·0)	-2·5 (-5·6–0·2)	-2·0 (-5·7–1·7)
Unsafe water source	3·1 (1·2–6·7)	2·8 (1·0–5·8)	1·1 (0·5–2·2)	0·9 (0·4–1·6)	-4·2 (-7·0–-1·3)	-5·7 (-9·7–-1·3)	-2·2 (-7·8–3·1)
Unsafe sanitation	7·6 (4·3–12·3)	5·9 (3·1–9·9)	4·3 (2·3–7·3)	3·1 (1·7–5·4)	-2·9 (-5·2–-0·9)	-3·0 (-5·6–-0·7)	-2·8 (-5·9–0·6)
No access to handwashing facility	1·7 (0·6–3·1)	1·8 (0·6–3·3)	1·7 (0·6–3·2)	1·4 (0·4–2·7)	-0·5 (-3·5–2·4)	-0·9 (-4·8–3·4)	-1·6 (-6·9–3·5)
Air pollution	24·5 (10·1–37·3)	22·4 (12·0–32·6)	12·8 (7·2–21·7)	7·4 (3·2–15·0)	-3·8 (-6·0–-1·9)	-5·2 (-7·7–-3·5)	-4·9 (-8·3–-3·1)
Particulate matter pollution	16·5 (6·7–27·4)	15·3 (11·4–19·8)	10·1 (6·6–14·0)	6·0 (3·1–9·3)	-3·3 (-5·4–-0·8)	-4·5 (-6·5–-3·3)	-4·8 (-8·0–-3·0)
Ambient particulate matter pollution	25·8 (10·7–43·3)	23·7 (18·4–29·5)	15·6 (10·5–20·7)	9·2 (4·8–13·9)	-3·3 (-5·5–-0·8)	-4·5 (-6·6–-3·4)	-4·8 (-8·1–-3·1)
Household air pollution from solid fuels	0·0 (0·0–0·1)	0·0 (0·0–0·0)	0·0 (0·0–0·0)	0·0 (0·0–0·0)	-3·3 (-25·4–-5·0)	-2·9 (-18·2–-3·1)	-2·7 (-13·0–-1·4)
Ambient ozone pollution	27·6 (21·3–35·5)	30·7 (24·4–39·0)	19·7 (14·0–27·2)	8·4 (4·5–13·4)	-3·8 (-5·1–-3·0)	-6·2 (-8·1–-4·8)	-7·8 (-10·8–-5·8)
Ambient nitrogen dioxide pollution	55·3 (0·0–100·0)	49·1 (0·0–100·0)	21·0 (0·0–65·9)	12·0 (0·0–50·1)	-4·9 (-15·9–0·0)	-6·7 (-22·2–0·0)	-5·1 (-23·3–0·0)
Non-optimal temperature	28·2 (21·8–38·0)	29·8 (24·4–38·4)	35·0 (28·4–44·4)	24·1 (19·9–31·2)	-0·5 (-0·9–-0·2)	-1·0 (-1·4–-0·7)	-3·4 (-4·3–-2·4)
High temperature	22·7 (15·3–32·1)	22·4 (15·5–31·1)	27·8 (19·7–37·2)	15·9 (9·9–24·5)	-1·1 (-1·7–-0·7)	-1·6 (-2·8–-0·9)	-5·1 (-7·3–-3·2)
Low temperature	24·2 (21·0–28·3)	27·3 (24·2–30·8)	31·1 (28·5–34·3)	22·7 (20·0–26·6)	-0·2 (-0·4–-0·1)	-0·9 (-1·2–-0·6)	-2·9 (-3·4–-2·2)
Other environmental risks	27·7 (7·5–42·8)	25·9 (7·5–40·8)	23·1 (7·5–37·8)	20·1 (7·5–34·7)	-1·0 (-1·7–0·0)	-1·2 (-2·0–0·0)	-1·3 (-2·3–0·0)
Residential radon	24·0 (0·0–70·2)	24·0 (0·0–70·2)	24·0 (0·0–70·2)	24·0 (0·0–70·2)	0·0 (0·0–0·0)	0·0 (0·0–0·0)	0·0 (0·0–0·0)
Lead exposure	29·2 (0·0–36·1)	26·7 (0·0–32·9)	22·7 (0·0–27·8)	18·3 (0·0–23·0)	-1·5 (-2·0–0·0)	-1·8 (-2·3–0·0)	-1·9 (-2·7–0·0)
Occupational risks	2·8 (2·3–3·5)	2·8 (2·3–3·7)	2·6 (2·1–3·4)	2·6 (2·1–3·4)	-0·2 (-0·5–0·1)	-0·4 (-0·8–0·0)	0·1 (-0·4–0·5)
Occupational carcinogens	0·9 (0·4–1·6)	0·9 (0·4–1·8)	0·9 (0·4–1·7)	0·9 (0·4–1·8)	0·1 (-0·3–0·4)	-0·1 (-0·6–0·3)	0·3 (-0·4–0·8)
Occupational exposure to asbestos	3·0 (2·6–3·3)	3·2 (2·9–3·6)	2·7 (2·4–3·0)	2·5 (1·9–3·1)	-0·6 (-1·4–0·2)	-1·3 (-2·5–-0·1)	-0·7 (-2·8–1·5)
Occupational exposure to arsenic	0·5 (0·0–1·2)	0·5 (0·0–1·2)	0·5 (0·0–1·1)	0·5 (0·0–1·1)	-0·3 (-0·8–0·2)	-0·4 (-1·1–0·3)	0·1 (-0·8–1·1)
Occupational exposure to benzene	1·0 (0·1–2·9)	1·1 (0·1–3·2)	1·1 (0·1–3·2)	1·1 (0·1–3·3)	0·4 (0·0–0·7)	0·2 (-0·3–0·6)	0·5 (-0·2–1·1)
Occupational exposure to beryllium	0·0 (0·0–0·0)	0·0 (0·0–0·0)	0·0 (0·0–0·0)	0·0 (0·0–0·0)	0·2 (-0·1–0·5)	0·1 (-0·2–0·4)	0·6 (0·1–1·1)
Occupational exposure to cadmium	0·1 (0·1–0·1)	0·1 (0·1–0·1)	0·1 (0·0–0·1)	0·1 (0·1–0·1)	-0·2 (-0·6–0·2)	-0·3 (-0·9–0·2)	0·2 (-0·6–1·0)
Occupational exposure to chromium	0·1 (0·1–0·2)	0·1 (0·1–0·2)	0·1 (0·1–0·1)	0·1 (0·1–0·2)	-0·1 (-0·5–0·3)	-0·2 (-0·7–0·3)	0·3 (-0·4–1·0)
Occupational exposure to diesel engine exhaust	0·6 (0·6–0·7)	0·7 (0·6–0·7)	0·6 (0·6–0·7)	0·7 (0·7–0·8)	0·3 (-0·1–0·7)	0·2 (-0·3–0·8)	0·8 (0·0–1·5)
Occupational exposure to formaldehyde	0·3 (0·3–0·3)	0·3 (0·3–0·3)	0·3 (0·2–0·3)	0·3 (0·2–0·3)	-0·3 (-0·7–0·1)	-0·4 (-0·9–0·2)	0·1 (-0·7–0·9)
Occupational exposure to nickel	0·4 (0·0–1·6)	0·4 (0·0–1·5)	0·4 (0·0–1·4)	0·4 (0·0–1·4)	-0·4 (-0·9–0·1)	-0·5 (-1·2–0·3)	0·1 (-1·0–1·2)
Occupational exposure to polycyclic aromatic hydrocarbons	0·3 (0·3–0·3)	0·3 (0·3–0·3)	0·3 (0·3–0·3)	0·3 (0·3–0·3)	-0·1 (-0·4–0·3)	-0·2 (-0·6–0·3)	0·3 (-0·3–1·0)
Occupational exposure to silica	3·5 (0·6–11·0)	3·4 (0·5–10·8)	3·1 (0·5–9·7)	3·1 (0·5–10·0)	-0·4 (-0·9–0·1)	-0·4 (-1·0–0·3)	0·2 (-0·8–1·2)
Occupational exposure to sulphuric acid	0·7 (0·2–2·4)	0·7 (0·2–2·4)	0·7 (0·1–2·2)	0·7 (0·1–2·2)	-0·2 (-0·6–0·2)	-0·4 (-0·9–0·3)	0·2 (-0·6–1·1)
Occupational exposure to trichloroethylene	0·1 (0·1–0·1)	0·1 (0·1–0·1)	0·1 (0·1–0·1)	0·1 (0·1–0·1)	0·0 (-0·2–0·3)	-0·1 (-0·4–0·3)	0·5 (-0·1–1·1)
Occupational asthmagens	16·5 (14·4–18·6)	16·8 (14·8–19·1)	15·1 (13·4–17·1)	15·6 (13·7–17·7)	-0·2 (-0·6–0·2)	-0·3 (-0·9–0·1)	0·3 (-0·4–1·0)
Occupational particulate matter, gases, and fumes	6·4 (4·9–8·5)	6·2 (4·7–8·2)	5·8 (4·5–7·6)	5·3 (4·1–6·9)	-0·6 (-0·9–-0·3)	-0·8 (-1·1–-0·5)	-0·8 (-1·2–-0·4)
Occupational noise	6·4 (5·8–7·0)	6·4 (5·9–6·9)	6·3 (5·8–6·8)	6·0 (5·6–6·5)	-0·2 (-0·4–0·0)	-0·3 (-0·5–-0·1)	-0·3 (-0·6–-0·1)
Occupational injuries	--	--	--	--	--	--	--
Occupational ergonomic factors	7·9 (6·6–9·6)	7·9 (6·7–9·5)	6·9 (5·9–8·4)	7·1 (6·0–8·8)	-0·3 (-0·8–0·1)	-0·5 (-1·0–0·1)	0·3 (-0·5–0·9)
**Behavioural risks**	**28·1 (25·1–31·5)**	**26·5 (23·3–30·3)**	**25·2 (22·2–29·2)**	**23·7 (20·4–27·7)**	**-0·5 (-0·9–-0·2)**	**-0·5 (-1·1–0·0)**	**-0·5 (-1·3–0·2)**
Child and maternal malnutrition	10·7 (7·1–14·7)	9·1 (6·2–12·4)	9·1 (6·4–12·3)	9·3 (6·3–12·6)	-0·4 (-1·3–0·3)	0·1 (-1·1–1·3)	0·2 (-1·6–1·9)
Suboptimal breastfeeding	68·9 (66·4–71·6)	66·7 (64·4–69·6)	62·9 (60·2–65·9)	62·3 (59·5–65·1)	-0·3 (-0·5–-0·2)	-0·3 (-0·5–-0·2)	-0·1 (-0·3–0·2)
Non-exclusive breastfeeding	64·5 (56·9–71·9)	59·8 (52·8–66·6)	48·7 (42·0–55·7)	46·3 (39·5–54·2)	-1·1 (-1·6–-0·6)	-1·2 (-1·9–-0·6)	-0·4 (-1·4–0·4)
Discontinued breastfeeding	88·8 (86·6–91·1)	86·3 (83·8–89·0)	84·4 (81·5–87·6)	84·7 (81·1–88·0)	-0·2 (-0·3–0·0)	-0·1 (-0·3–0·1)	0·0 (-0·3–0·3)
Child growth failure	1·4 (0·5–3·3)	1·3 (0·4–2·9)	1·1 (0·4–2·4)	1·0 (0·3–2·2)	-1·1 (-1·5–-0·7)	-1·1 (-1·7–-0·7)	-0·6 (-1·5–0·1)
Child underweight	3·1 (1·9–4·3)	2·8 (1·8–3·8)	2·3 (1·4–3·1)	2·2 (1·3–2·9)	-1·1 (-1·6–-0·6)	-1·2 (-1·9–-0·6)	-0·6 (-1·5–0·4)
Child wasting	0·9 (0·6–1·5)	0·9 (0·6–1·3)	0·9 (0·5–1·3)	0·8 (0·5–1·3)	-0·4 (-0·7–-0·1)	-0·2 (-0·7–0·2)	-0·3 (-1·0–0·3)
Child stunting	4·1 (3·2–5·2)	3·8 (3·0–4·7)	3·2 (2·4–4·0)	2·9 (2·2–3·6)	-1·2 (-1·8–-0·5)	-1·3 (-2·0–-0·5)	-0·8 (-2·0–0·3)
Low birth weight and short gestation	22·0 (19·4–25·2)	23·8 (20·8–26·9)	27·8 (24·5–31·5)	26·9 (23·7–30·6)	0·6 (0·4–0·9)	0·6 (0·2–0·9)	-0·3 (-1·0–0·4)
Short gestation	38·3 (33·7–43·7)	41·8 (36·7–47·2)	48·4 (42·6–55·1)	45·8 (40·6–52·0)	0·6 (0·3–0·9)	0·4 (0·0–0·9)	-0·5 (-1·4–0·4)
Low birth weight	17·2 (15·7–18·8)	18·0 (16·4–19·7)	20·7 (18·9–22·6)	20·3 (18·5–22·3)	0·5 (0·3–0·8)	0·6 (0·2–0·9)	-0·2 (-0·8–0·5)

Iron deficiency	3·8 (2·6–5·2)	3·2 (2·3–4·3)	3·3 (2·4–4·4)	3·4 (2·4–4·5)	-0·4 (-1·2–0·4)	0·2 (-0·8–1·3)	0·2 (-1·5–1·9)
Vitamin A deficiency	1·2 (0·0–2·7)	1·7 (0·0–3·6)	1·0 (0·0–2·1)	0·6 (0·0–1·3)	-2·3 (-4·8–0·1)	-5·0 (-8·0–0·0)	-5·0 (-8·8–0·0)
Zinc deficiency	1·9 (0·0–8·0)	1·1 (0·0–4·9)	1·0 (0·0–4·6)	1·1 (0·0–4·6)	-1·9 (-45·9–0·4)	-0·2 (-1·0–5·9)	0·4 (-5·6–4·8)
Tobacco	38·2 (36·8–39·7)	34·2 (32·8–35·4)	28·2 (27·2–29·1)	25·2 (23·6–26·9)	-1·3 (-1·6–-1·1)	-1·5 (-1·8–-1·1)	-1·0 (-1·6–-0·4)
Smoking	35·3 (33·1–37·5)	31·4 (29·4–33·1)	25·7 (24·4–27·1)	22·8 (20·8–25·0)	-1·4 (-1·7–-1·1)	-1·5 (-2·0–-1·1)	-1·1 (-1·9–-0·3)
Chewing tobacco	3·8 (3·2–4·6)	3·7 (3·1–4·4)	3·1 (2·5–4·0)	2·8 (2·1–3·7)	-1·0 (-2·2–0·1)	-1·2 (-2·8–0·2)	-0·9 (-3·2–1·3)
Second-hand smoke	31·4 (29·6–32·8)	28·3 (26·4–29·5)	23·7 (22·1–24·7)	21·3 (19·6–22·9)	-1·3 (-1·5–-1·0)	-1·4 (-1·7–-1·0)	-1·0 (-1·6–-0·3)
High alcohol use	23·7 (16·3–33·7)	22·4 (15·0–32·5)	24·0 (16·7–34·5)	24·3 (16·7–35·3)	0·1 (-1·1–1·3)	0·4 (-1·2–2·0)	0·1 (-2·1–2·4)
Drug use	0·7 (0·4–1·4)	0·7 (0·5–1·2)	1·4 (1·1–1·6)	4·0 (2·9–5·1)	5·5 (2·5–8·3)	8·1 (4·7–11·0)	9·8 (6·2–12·2)
Dietary risks	36·6 (28·0–48·1)	38·6 (29·8–50·6)	41·0 (31·2–53·9)	41·2 (31·6–53·6)	0·4 (0·1–0·7)	0·3 (0·0–0·6)	0·1 (-0·4–0·5)
Diet low in fruits	36·8 (32·9–40·2)	35·0 (30·3–38·1)	36·5 (32·7–39·4)	38·1 (33·6–42·5)	0·1 (-0·2–0·5)	0·4 (-0·1–0·9)	0·4 (-0·3–1·2)
Diet low in vegetables	25·7 (16·0–33·4)	21·5 (13·5–27·8)	23·0 (14·4–28·4)	23·9 (15·4–30·3)	-0·2 (-0·7–0·3)	0·5 (-0·2–1·3)	0·3 (-0·7–1·3)
Diet low in legumes	38·5 (0·0–48·9)	38·6 (0·0–48·4)	43·1 (0·0–53·8)	44·0 (0·0–55·3)	0·4 (0·0–0·7)	0·6 (0·0–1·1)	0·2 (-0·5–0·9)
Diet low in whole grains	44·5 (37·0–50·8)	47·8 (39·8–54·3)	49·5 (40·9–56·7)	50·0 (41·4–57·0)	0·4 (0·1–0·6)	0·2 (-0·1–0·6)	0·1 (-0·4–0·7)
Diet low in nuts and seeds	13·5 (9·6–17·4)	7·7 (4·9–10·7)	5·9 (3·6–8·4)	6·5 (4·0–9·6)	-2·4 (-4·1–-0·7)	-0·8 (-3·2–1·9)	0·9 (-2·7–4·4)
Diet low in milk	37·4 (33·3–41·8)	34·2 (29·7–38·9)	33·4 (28·8–37·9)	33·8 (28·9–38·9)	-0·3 (-0·8–0·1)	-0·1 (-0·7–0·5)	0·1 (-0·8–0·9)
Diet high in red meat	48·6 (0·0–67·2)	49·3 (0·0–67·5)	49·2 (0·0–67·4)	49·1 (0·0–67·3)	0·0 (-0·4–0·5)	0·0 (-0·7–0·7)	0·0 (-1·1–1·0)
Diet high in processed meat	45·4 (36·0–51·5)	57·1 (46·9–64·3)	64·2 (51·6–72·2)	62·1 (50·6–70·0)	1·0 (0·6–1·4)	0·4 (-0·2–1·0)	-0·3 (-1·0–0·5)
Diet high in sugar-sweetened beverages	34·4 (26·0–41·4)	49·1 (39·6–57·5)	53·6 (43·2–62·4)	52·5 (41·5–62·5)	1·4 (0·6–2·2)	0·3 (-0·7–1·3)	-0·2 (-1·6–1·1)
Diet low in fibre	40·3 (22·2–47·6)	30·3 (16·4–36·6)	27·5 (14·8–33·7)	24·7 (13·3–32·5)	-1·6 (-2·5–-0·8)	-1·0 (-2·2–0·3)	-1·0 (-2·9–0·7)
Diet low in calcium	5·9 (4·8–7·3)	4·9 (4·0–6·0)	5·3 (4·3–6·6)	5·4 (4·3–6·6)	-0·3 (-0·5–-0·1)	0·5 (0·2–0·8)	0·1 (-0·3–0·5)
Diet low in seafood omega-3 fatty acids	37·4 (28·2–47·4)	43·8 (33·8–54·6)	48·0 (37·8–59·4)	43·7 (34·0–54·4)	0·5 (0·1–1·0)	0·0 (-0·6–0·5)	-0·9 (-1·6–-0·1)
Diet low in omega-6 polyunsaturated fatty acids	37·0 (23·9–46·6)	31·8 (21·7–41·1)	24·4 (17·1–31·6)	23·1 (16·0–30·2)	-1·5 (-2·3–-0·8)	-1·5 (-2·5–-0·7)	-0·5 (-1·6–0·5)
Diet high in trans fatty acids	71·5 (63·5–78·7)	71·9 (63·3–78·7)	63·3 (53·3–71·1)	0·0 (0·0–0·0)	-65·8 (-66·1–-65·4)	-97·1 (-97·5–-96·5)	-184·2 (-185·3–-182·7)
Diet high in sodium	23·9 (0·6–72·7)	28·8 (1·8–79·0)	31·9 (3·5–77·9)	32·2 (2·9–79·9)	1·0 (0·2–6·1)	0·5 (-0·1–4·0)	0·1 (-2·9–2·3)
Intimate partner violence	27·6 (17·0–35·8)	28·0 (19·9–34·9)	23·4 (12·4–29·3)	22·4 (11·8–31·3)	-0·7 (-2·1–0·7)	-1·1 (-3·0–0·8)	-0·4 (-2·8–1·8)
Childhood sexual abuse and bullying	8·6 (5·4–13·8)	10·4 (6·6–16·6)	11·9 (7·6–19·2)	9·1 (5·9–13·1)	0·2 (-0·4–0·8)	-0·6 (-1·6–0·2)	-2·5 (-4·1–-1·0)
Childhood sexual abuse	7·5 (6·4–8·9)	8·5 (7·2–10·1)	9·9 (8·7–11·5)	9·1 (7·5–11·1)	0·6 (-0·2–1·3)	0·3 (-0·7–1·3)	-0·8 (-2·2–0·5)
Bullying victimization	7·9 (3·5–15·8)	9·9 (4·5–19·2)	11·3 (5·0–21·9)	7·6 (3·4–13·5)	-0·2 (-0·9–0·6)	-1·3 (-2·3–-0·2)	-3·6 (-5·6–-1·6)
Unsafe sex	--	--	--	--	--	--	--
Low physical activity	20·5 (15·7–26·5)	20·0 (15·4–24·8)	19·3 (15·1–24·1)	21·6 (16·5–27·8)	0·2 (-0·7–1·1)	0·4 (-0·8–1·5)	1·0 (-0·7–2·6)
**Metabolic risks**	**25·5 (22·2–29·0)**	**31·7 (27·8–34·7)**	**35·1 (31·0–37·9)**	**38·2 (33·6–41·2)**	**1·3 (1·0–1·6)**	**0·9 (0·6–1·2)**	**0·8 (0·3–1·2)**
High fasting plasma glucose	13·1 (10·0–15·7)	17·2 (12·8–20·3)	22·2 (16·7–25·9)	25·0 (18·7–29·8)	2·1 (1·5–2·7)	1·8 (1·0–2·5)	1·1 (-0·1–2·3)
High LDL cholesterol	63·3 (44·5–85·8)	53·5 (36·8–74·0)	48·2 (32·4–66·8)	46·7 (31·1–65·3)	-1·0 (-1·2–-0·8)	-0·7 (-0·8–-0·5)	-0·3 (-0·5–0·0)
High systolic blood pressure	31·4 (21·6–42·5)	27·4 (19·6–37·1)	20·3 (13·5–28·8)	27·9 (17·5–40·6)	-0·4 (-1·4–0·5)	0·1 (-1·1–1·1)	2·9 (0·6–5·1)
High body-mass index	28·7 (24·2–33·4)	36·3 (30·8–40·2)	40·3 (34·4–44·0)	43·4 (36·9–47·0)	1·3 (1·0–1·6)	0·9 (0·5–1·1)	0·7 (0·3–1·1)
Low bone mineral density	21·6 (15·4–28·3)	19·5 (13·9–26·7)	20·3 (13·9–27·5)	22·5 (16·5–29·7)	0·1 (-0·3–0·6)	0·7 (0·0–1·5)	0·9 (-0·4–2·5)
Kidney dysfunction	3·1 (2·5–4·0)	3·1 (2·6–4·2)	3·1 (2·5–4·1)	3·2 (2·6–4·2)	0·2 (0·1–0·3)	0·1 (0·0–0·2)	0·3 (0·0–0·6)

Hawaii

Risk Names	SEV 1990	SEV 2000	SEV 2010	SEV 2021	Annualised rate of change 1990 to 2021	Annualised rate of change 2000 to 2021	Annualised rate of change 2010 to 2021
**All risk factors**	**27·6 (24·6–31·0)**	**28·2 (25·1–31·4)**	**28·2 (25·2–31·5)**	**28·3 (25·1–31·5)**	**0·1 (-0·2–0·4)**	**0·0 (-0·4–0·4)**	**0·0 (-0·5–0·6)**
**Environmental/occupational risks**	**9·6 (5·3–13·2)**	**9·6 (5·1–12·7)**	**8·9 (4·5–11·6)**	**7·8 (3·8–10·5)**	**-0·7 (-1·3–-0·3)**	**-1·0 (-1·5–-0·6)**	**-1·2 (-1·8–-0·6)**
Unsafe water, sanitation, and handwashing	2·0 (1·0–3·2)	1·8 (0·8–2·8)	1·4 (0·5–2·4)	1·1 (0·4–2·0)	-1·8 (-4·0–0·3)	-2·0 (-5·0–0·9)	-1·8 (-5·9–2·5)
Unsafe water source	2·7 (1·1–5·5)	2·4 (1·0–4·8)	1·0 (0·4–2·0)	0·8 (0·3–1·5)	-4·0 (-7·0–-0·8)	-5·2 (-9·1–-1·4)	-2·0 (-8·0–3·3)
Unsafe sanitation	6·2 (3·3–10·3)	4·6 (2·4–8·1)	3·5 (1·9–5·9)	2·7 (1·4–4·5)	-2·7 (-4·9–-0·6)	-2·6 (-5·3–0·0)	-2·5 (-5·8–0·6)
No access to handwashing facility	1·7 (0·6–3·3)	1·8 (0·5–3·3)	1·8 (0·6–3·4)	1·5 (0·5–2·9)	-0·4 (-3·2–2·6)	-0·7 (-4·6–3·6)	-1·5 (-7·0–4·5)
Air pollution	7·4 (0·3–16·7)	7·5 (0·7–16·5)	4·2 (0·3–11·7)	2·8 (0·1–9·2)	-3·2 (-8·8–-0·8)	-4·8 (-11·9–-2·6)	-3·8 (-13·9–-1·6)
Particulate matter pollution	2·3 (0·0–7·5)	2·3 (0·3–5·1)	1·5 (0·1–3·9)	0·6 (0·0–2·3)	-4·3 (-13·3–6·6)	-6·3 (-15·2–-3·6)	-8·2 (-20·7–-4·2)
Ambient particulate matter pollution	3·5 (0·0–11·3)	3·5 (0·5–7·6)	2·3 (0·2–5·8)	0·9 (0·0–3·5)	-4·3 (-13·3–6·5)	-6·3 (-15·3–-3·6)	-8·2 (-20·6–-4·1)
Household air pollution from solid fuels	0·0 (0·0–0·0)	0·0 (0·0–0·0)	0·0 (0·0–0·0)	0·0 (0·0–0·0)	-4·3 (-33·2–-5·5)	-6·6 (-34·1–-7·6)	-7·3 (-32·4–-8·5)
Ambient ozone pollution	4·6 (0·3–14·1)	7·0 (0·4–17·4)	5·7 (0·2–15·3)	5·1 (0·1–16·0)	0·3 (-9·9–8·1)	-1·6 (-17·1–9·0)	-1·0 (-32·4–26·7)
Ambient nitrogen dioxide pollution	30·6 (0·0–78·1)	30·7 (0·0–78·1)	16·0 (0·0–54·9)	12·3 (0·0–49·0)	-2·9 (-12·0–0·0)	-4·4 (-17·9–0·0)	-2·4 (-13·4–0·0)
Non-optimal temperature	12·3 (9·8–17·8)	12·4 (9·9–17·6)	12·3 (9·8–17·4)	10·7 (8·4–15·6)	-0·4 (-0·6–-0·1)	-0·7 (-0·9–-0·2)	-1·2 (-1·5–-0·5)
High temperature	3·9 (0·0–9·5)	1·4 (0·0–7·1)	0·0 (0·0–0·0)	2·8 (0·0–8·1)	-1·0 (-4·2–53·2)	3·3 (-1·2–85·0)	45·3 (0·0–165·4)
Low temperature	12·1 (10·0–16·7)	12·1 (9·9–16·7)	12·0 (9·9–16·5)	10·6 (8·5–15·2)	-0·4 (-0·5–-0·2)	-0·6 (-0·8–-0·3)	-1·2 (-1·4–-0·7)
Other environmental risks	24·1 (4·8–37·4)	22·9 (4·8–36·0)	20·6 (4·8–33·5)	17·9 (4·8–30·5)	-1·0 (-1·5–0·0)	-1·2 (-1·8–0·0)	-1·3 (-2·2–0·0)
Residential radon	18·5 (0·0–53·9)	18·5 (0·0–53·9)	18·5 (0·0–53·9)	18·5 (0·0–53·9)	0·0 (0·0–0·0)	0·0 (0·0–0·0)	0·0 (0·0–0·0)
Lead exposure	26·5 (0·0–32·9)	24·8 (0·0–30·9)	21·4 (0·0–26·7)	17·5 (0·0–22·1)	-1·3 (-1·8–0·0)	-1·7 (-2·1–0·0)	-1·8 (-2·6–0·0)
Occupational risks	2·5 (2·0–3·2)	2·6 (2·1–3·4)	2·6 (2·1–3·4)	2·6 (2·1–3·5)	0·1 (-0·1–0·4)	0·0 (-0·4–0·3)	0·1 (-0·4–0·6)
Occupational carcinogens	0·8 (0·4–1·7)	0·9 (0·4–1·9)	0·9 (0·4–1·9)	1·0 (0·4–2·0)	0·4 (0·0–0·7)	0·1 (-0·3–0·5)	0·1 (-0·5–0·7)
Occupational exposure to asbestos	3·0 (2·6–3·3)	2·7 (2·3–3·0)	2·6 (2·3–2·9)	2·3 (1·7–2·9)	-0·8 (-1·8–0·1)	-0·7 (-2·0–0·6)	-1·3 (-3·6–1·2)
Occupational exposure to arsenic	0·4 (0·0–1·0)	0·4 (0·0–1·0)	0·4 (0·0–1·0)	0·4 (0·0–1·1)	0·1 (-0·4–0·5)	0·0 (-0·7–0·5)	0·2 (-0·7–1·1)
Occupational exposure to benzene	1·1 (0·1–3·0)	1·2 (0·1–3·5)	1·2 (0·1–3·6)	1·3 (0·1–3·8)	0·6 (0·2–0·9)	0·2 (-0·2–0·6)	0·2 (-0·4–0·9)
Occupational exposure to beryllium	0·0 (0·0–0·0)	0·0 (0·0–0·0)	0·0 (0·0–0·0)	0·0 (0·0–0·0)	0·3 (0·1–0·5)	0·1 (-0·2–0·5)	0·4 (-0·1–0·8)
Occupational exposure to cadmium	0·1 (0·0–0·1)	0·1 (0·0–0·1)	0·1 (0·0–0·1)	0·1 (0·0–0·1)	0·1 (-0·3–0·5)	0·0 (-0·5–0·6)	0·2 (-0·5–1·0)
Occupational exposure to chromium	0·1 (0·1–0·1)	0·1 (0·1–0·1)	0·1 (0·1–0·1)	0·1 (0·1–0·1)	0·2 (-0·1–0·6)	0·1 (-0·4–0·6)	0·3 (-0·4–0·9)
Occupational exposure to diesel engine exhaust	0·7 (0·6–0·7)	0·7 (0·7–0·8)	0·7 (0·7–0·8)	0·8 (0·7–0·8)	0·4 (0·0–0·7)	0·4 (-0·2–0·9)	0·6 (-0·2–1·3)
Occupational exposure to formaldehyde	0·2 (0·2–0·3)	0·3 (0·2–0·3)	0·3 (0·2–0·3)	0·3 (0·2–0·3)	0·2 (-0·3–0·5)	0·0 (-0·6–0·5)	0·1 (-0·7–0·9)
Occupational exposure to nickel	0·3 (0·0–1·2)	0·3 (0·0–1·3)	0·3 (0·0–1·3)	0·3 (0·0–1·3)	0·0 (-0·5–0·5)	-0·1 (-0·8–0·7)	0·2 (-0·8–1·2)
Occupational exposure to polycyclic aromatic hydrocarbons	0·3 (0·2–0·3)	0·3 (0·3–0·3)	0·3 (0·3–0·3)	0·3 (0·3–0·3)	0·3 (0·0–0·6)	0·1 (-0·4–0·6)	0·3 (-0·4–0·9)
Occupational exposure to silica	2·8 (0·4–8·7)	2·8 (0·4–8·7)	2·8 (0·5–8·8)	2·9 (0·5–9·1)	0·1 (-0·4–0·5)	0·1 (-0·6–0·8)	0·4 (-0·6–1·3)
Occupational exposure to sulphuric acid	0·6 (0·1–2·1)	0·7 (0·1–2·1)	0·6 (0·1–2·1)	0·7 (0·1–2·2)	0·2 (-0·3–0·6)	0·1 (-0·5–0·6)	0·3 (-0·5–1·0)
Occupational exposure to trichloroethylene	0·1 (0·1–0·1)	0·1 (0·1–0·1)	0·1 (0·1–0·1)	0·1 (0·1–0·1)	0·3 (0·0–0·6)	0·2 (-0·3–0·6)	0·4 (-0·2–1·0)
Occupational asthmagens	14·7 (12·8–16·7)	15·7 (13·6–17·8)	15·3 (13·3–17·5)	15·8 (13·8–18·2)	0·2 (-0·2–0·6)	0·0 (-0·5–0·6)	0·3 (-0·5–1·0)
Occupational particulate matter, gases, and fumes	5·3 (4·1–7·0)	5·2 (4·0–6·8)	5·0 (3·9–6·5)	4·8 (3·7–6·1)	-0·3 (-0·7–-0·1)	-0·4 (-0·7–-0·1)	-0·4 (-0·8–-0·1)
Occupational noise	5·8 (5·3–6·5)	5·9 (5·5–6·6)	6·0 (5·6–6·6)	6·0 (5·6–6·5)	0·1 (-0·1–0·3)	0·0 (-0·2–0·2)	0·0 (-0·3–0·2)
Occupational injuries	--	--	--	--	--	--	--
Occupational ergonomic factors	8·2 (6·8–9·9)	8·3 (7·0–10·2)	7·7 (6·5–9·4)	7·8 (6·5–9·6)	-0·1 (-0·5–0·2)	-0·3 (-0·8–0·1)	0·1 (-0·5–0·7)
**Behavioural risks**	**29·3 (26·0–33·4)**	**27·8 (24·6–31·3)**	**26·5 (23·6–29·9)**	**24·3 (21·1–28·0)**	**-0·6 (-1·0–-0·2)**	**-0·6 (-1·1–-0·1)**	**-0·8 (-1·5–-0·2)**
Child and maternal malnutrition	10·1 (6·9–13·9)	8·6 (6·1–11·9)	8·7 (6·2–11·7)	8·8 (6·1–12·1)	-0·4 (-1·3–0·5)	0·1 (-1·1–1·4)	0·2 (-1·7–1·9)
Suboptimal breastfeeding	68·7 (66·4–71·4)	66·6 (64·3–69·3)	62·8 (60·4–65·6)	62·1 (59·2–65·2)	-0·3 (-0·5–-0·2)	-0·3 (-0·5–-0·1)	-0·1 (-0·4–0·2)
Non-exclusive breastfeeding	64·1 (57·1–71·2)	59·5 (52·9–66·4)	48·7 (42·1–55·8)	46·3 (39·7–54·2)	-1·1 (-1·6–-0·5)	-1·2 (-1·9–-0·6)	-0·5 (-1·5–0·7)
Discontinued breastfeeding	88·6 (86·4–91·3)	86·2 (83·8–89·1)	84·3 (81·1–87·5)	84·5 (81·2–87·9)	-0·2 (-0·3–0·0)	-0·1 (-0·3–0·1)	0·0 (-0·3–0·3)
Child growth failure	1·2 (0·4–2·8)	1·1 (0·4–2·7)	1·0 (0·3–2·2)	0·9 (0·3–2·1)	-0·9 (-1·3–-0·5)	-1·0 (-1·5–-0·5)	-0·6 (-1·3–0·3)
Child underweight	2·6 (1·6–3·6)	2·4 (1·5–3·3)	2·1 (1·3–2·8)	1·9 (1·2–2·6)	-0·9 (-1·4–-0·4)	-1·0 (-1·8–-0·3)	-0·5 (-1·5–0·6)
Child wasting	0·8 (0·5–1·3)	0·8 (0·5–1·2)	0·8 (0·5–1·2)	0·8 (0·5–1·2)	-0·2 (-0·6–0·1)	-0·1 (-0·6–0·4)	-0·2 (-0·9–0·6)
Child stunting	3·6 (2·7–4·5)	3·4 (2·6–4·2)	2·9 (2·2–3·6)	2·7 (2·0–3·3)	-1·0 (-1·6–-0·4)	-1·1 (-2·0–-0·2)	-0·8 (-2·1–0·6)
Low birth weight and short gestation	22·4 (19·6–25·4)	23·5 (20·6–26·6)	23·6 (20·8–26·9)	23·5 (20·5–26·6)	0·2 (-0·1–0·4)	0·0 (-0·4–0·4)	0·0 (-0·8–0·7)
Short gestation	40·2 (35·5–44·9)	42·6 (37·5–48·0)	40·1 (35·5–45·7)	39·3 (34·8–44·1)	-0·1 (-0·4–0·2)	-0·4 (-0·8–0·0)	-0·2 (-1·0–0·7)
Low birth weight	17·1 (15·5–18·6)	17·7 (16·0–19·4)	18·6 (16·9–20·3)	18·7 (16·9–20·3)	0·3 (0·0–0·5)	0·3 (-0·1–0·6)	0·1 (-0·6–0·7)

Iron deficiency	3·4 (2·4–4·7)	3·0 (2·2–4·1)	3·0 (2·3–4·1)	3·1 (2·2–4·2)	-0·4 (-1·2–0·5)	0·2 (-1·0–1·4)	0·1 (-1·7–1·7)
Vitamin A deficiency	1·0 (0·0–2·2)	1·3 (0·0–2·7)	0·8 (0·0–1·7)	0·5 (0·0–1·0)	-2·5 (-4·7–0·0)	-4·8 (-7·9–0·0)	-5·0 (-9·4–0·0)
Zinc deficiency	1·6 (0·0–7·1)	1·0 (0·0–4·2)	0·9 (0·0–3·9)	0·9 (0·0–3·8)	-1·8 (-41·7–1·7)	-0·4 (-2·3–4·0)	0·1 (-1·8–18·7)
Tobacco	38·4 (36·6–39·9)	33·2 (31·9–34·5)	27·5 (26·5–28·6)	23·1 (21·5–24·7)	-1·6 (-1·9–-1·4)	-1·7 (-2·1–-1·4)	-1·6 (-2·3–-1·0)
Smoking	32·8 (30·4–35·1)	27·4 (25·7–29·2)	22·8 (21·5–24·2)	18·8 (17·2–20·8)	-1·8 (-2·1–-1·5)	-1·8 (-2·2–-1·4)	-1·8 (-2·5–-1·0)
Chewing tobacco	0·9 (0·6–1·1)	1·0 (0·8–1·4)	1·1 (0·8–1·6)	1·2 (0·9–1·7)	1·1 (-0·2–2·6)	0·8 (-1·1–2·7)	0·5 (-2·2–3·5)
Second-hand smoke	34·7 (32·6–36·3)	30·7 (28·7–32·0)	25·5 (23·8–26·6)	21·7 (19·8–23·5)	-1·5 (-1·8–-1·3)	-1·6 (-2·0–-1·3)	-1·5 (-2·2–-0·8)
High alcohol use	25·7 (17·9–36·1)	25·0 (18·1–34·2)	26·2 (19·0–35·2)	26·4 (18·8–36·5)	0·1 (-1·0–1·2)	0·3 (-1·1–1·6)	0·1 (-1·7–1·8)
Drug use	0·8 (0·3–1·6)	0·8 (0·5–1·3)	1·2 (1·0–1·6)	2·7 (2·3–3·3)	4·2 (1·5–7·3)	6·1 (3·2–8·8)	7·5 (4·6–9·6)
Dietary risks	34·4 (25·0–45·7)	38·2 (29·0–49·6)	41·2 (31·8–53·3)	41·8 (31·8–54·0)	0·6 (0·4–0·9)	0·4 (0·2–0·7)	0·1 (-0·2–0·6)
Diet low in fruits	34·0 (28·3–37·1)	30·3 (26·0–33·2)	31·4 (28·0–34·3)	34·0 (28·9–38·3)	0·0 (-0·4–0·4)	0·5 (0·0–1·1)	0·7 (0·0–1·7)
Diet low in vegetables	19·6 (11·4–26·9)	27·3 (16·0–34·5)	36·7 (20·8–44·1)	37·1 (21·3–45·5)	2·1 (1·5–2·8)	1·5 (1·0–2·1)	0·1 (-0·6–0·8)
Diet low in legumes	37·1 (0·0–46·6)	37·8 (0·0–47·5)	42·0 (0·0–52·2)	42·5 (0·0–54·0)	0·4 (0·0–0·8)	0·6 (0·0–1·0)	0·1 (-0·7–0·9)
Diet low in whole grains	43·2 (36·4–48·7)	46·1 (38·4–51·8)	47·8 (40·2–54·5)	48·4 (40·3–55·1)	0·4 (0·1–0·6)	0·2 (-0·1–0·6)	0·1 (-0·4–0·6)
Diet low in nuts and seeds	11·2 (8·0–14·9)	6·9 (4·4–10·1)	5·0 (3·1–7·7)	5·3 (3·0–8·4)	-2·4 (-4·2–-0·8)	-1·2 (-3·8–1·5)	0·5 (-3·3–4·0)
Diet low in milk	33·8 (29·7–38·3)	32·0 (28·0–36·4)	31·0 (27·0–35·3)	30·6 (26·1–35·2)	-0·3 (-0·8–0·2)	-0·2 (-0·9–0·4)	-0·1 (-1·0–0·8)
Diet high in red meat	54·2 (0·0–73·4)	54·2 (0·0–73·8)	54·3 (0·0–74·2)	54·2 (0·0–73·9)	0·0 (-0·4–0·3)	0·0 (-0·5–0·5)	0·0 (-0·7–0·8)
Diet high in processed meat	39·4 (31·0–43·9)	48·8 (39·2–54·2)	55·0 (43·9–61·9)	53·6 (42·1–61·3)	1·0 (0·6–1·4)	0·4 (-0·1–1·0)	-0·2 (-1·0–0·5)
Diet high in sugar-sweetened beverages	37·3 (28·5–45·5)	49·6 (38·7–57·7)	54·4 (43·6–63·0)	54·4 (43·6–64·3)	1·2 (0·5–2·0)	0·4 (-0·5–1·3)	0·0 (-1·3–1·1)
Diet low in fibre	36·9 (19·2–44·7)	28·6 (14·9–35·5)	25·4 (14·0–31·3)	22·0 (12·4–28·4)	-1·7 (-2·6–-0·8)	-1·3 (-2·6–-0·1)	-1·3 (-3·3–0·3)
Diet low in calcium	5·1 (4·1–6·3)	4·6 (3·7–5·5)	4·9 (3·9–5·9)	4·8 (3·8–5·8)	-0·2 (-0·4–0·0)	0·2 (0·0–0·4)	-0·2 (-0·5–0·2)
Diet low in seafood omega-3 fatty acids	33·4 (24·1–43·6)	41·6 (32·0–52·9)	44·9 (34·1–56·3)	40·2 (30·0–51·4)	0·6 (0·1–1·1)	-0·2 (-0·8–0·4)	-1·0 (-1·8–-0·3)
Diet low in omega-6 polyunsaturated fatty acids	33·0 (21·7–42·7)	29·7 (20·1–38·3)	22·5 (15·6–28·8)	21·0 (14·6–28·2)	-1·5 (-2·2–-0·7)	-1·6 (-2·6–-0·9)	-0·6 (-1·7–0·2)
Diet high in trans fatty acids	71·5 (63·3–78·8)	71·7 (64·3–79·0)	63·3 (54·3–70·2)	0·0 (0·0–0·0)	-65·8 (-66·1–-65·4)	-97·1 (-97·6–-96·6)	-184·2 (-185·2–-182·8)
Diet high in sodium	24·2 (0·8–69·1)	28·6 (2·3–74·6)	31·2 (4·0–73·6)	31·7 (3·7–75·6)	0·9 (0·2–5·5)	0·5 (-0·2–3·4)	0·1 (-2·1–1·6)
Intimate partner violence	20·8 (13·3–27·1)	20·6 (14·4–26·0)	19·5 (13·0–24·3)	20·4 (10·8–29·0)	-0·1 (-1·7–1·6)	-0·1 (-2·1–1·9)	0·4 (-1·3–2·0)
Childhood sexual abuse and bullying	16·8 (9·2–26·7)	18·9 (10·9–28·6)	20·5 (12·1–29·4)	16·1 (9·3–24·9)	-0·1 (-1·0–0·7)	-0·8 (-1·8–0·4)	-2·2 (-4·2–-0·1)
Childhood sexual abuse	6·1 (5·1–7·4)	7·2 (6·3–8·6)	8·5 (7·6–9·5)	7·8 (6·5–9·3)	0·8 (-0·1–1·7)	0·3 (-0·7–1·5)	-0·8 (-2·3–0·8)
Bullying victimization	20·7 (9·6–35·2)	22·9 (11·0–36·8)	24·5 (12·1–37·1)	18·5 (8·9–31·1)	-0·4 (-1·3–0·7)	-1·0 (-2·2–0·4)	-2·5 (-4·8–-0·1)
Unsafe sex	--	--	--	--	--	--	--
Low physical activity	19·0 (14·4–24·2)	17·7 (13·6–21·7)	17·7 (13·8–22·0)	20·1 (15·0–26·2)	0·2 (-0·7–1·1)	0·6 (-0·7–1·7)	1·2 (-0·6–2·9)
**Metabolic risks**	**24·5 (21·5–27·7)**	**28·9 (25·6–31·9)**	**31·9 (28·3–34·7)**	**34·6 (30·6–37·6)**	**1·1 (0·8–1·4)**	**0·9 (0·5–1·2)**	**0·8 (0·3–1·2)**
High fasting plasma glucose	11·4 (8·9–13·2)	14·7 (11·3–17·0)	18·1 (13·8–21·3)	21·3 (15·8–25·2)	2·0 (1·5–2·6)	1·8 (1·0–2·5)	1·5 (0·4–2·6)
High LDL cholesterol	63·0 (44·7–86·0)	53·3 (36·8–74·1)	47·8 (32·5–66·6)	46·3 (31·2–64·8)	-1·0 (-1·2–-0·8)	-0·7 (-0·9–-0·5)	-0·3 (-0·6–0·0)
High systolic blood pressure	29·4 (20·8–39·6)	23·5 (16·3–32·5)	17·4 (11·1–24·9)	24·4 (14·9–35·6)	-0·6 (-1·5–0·2)	0·2 (-1·1–1·3)	3·1 (0·7–5·4)
High body-mass index	27·1 (23·0–31·5)	32·7 (28·0–36·6)	36·4 (31·0–40·2)	38·9 (33·3–42·7)	1·2 (0·9–1·5)	0·8 (0·5–1·1)	0·6 (0·1–1·0)
Low bone mineral density	21·5 (15·9–28·8)	19·5 (13·6–26·2)	20·4 (14·1–27·3)	22·2 (15·9–29·5)	0·1 (-0·4–0·6)	0·6 (0·0–1·4)	0·7 (-0·6–2·3)
Kidney dysfunction	2·8 (2·3–3·7)	2·9 (2·4–3·9)	2·9 (2·3–3·8)	3·0 (2·4–4·0)	0·2 (0·1–0·3)	0·1 (0·0–0·3)	0·3 (0·0–0·7)

Idaho

Risk Names	SEV 1990	SEV 2000	SEV 2010	SEV 2021	Annualised rate of change 1990 to 2021	Annualised rate of change 2000 to 2021	Annualised rate of change 2010 to 2021
**All risk factors**	**24·6 (21·9–27·7)**	**25·0 (22·1–28·2)**	**25·0 (22·1–28·2)**	**25·0 (21·8–28·4)**	**0·0 (-0·2–0·3)**	**0·0 (-0·4–0·4)**	**0·0 (-0·6–0·6)**
**Environmental/occupational risks**	**16·0 (10·3–27·5)**	**16·1 (10·2–26·9)**	**14·8 (9·3–25·2)**	**13·5 (8·4–22·8)**	**-0·5 (-1·2–-0·1)**	**-0·8 (-1·2–-0·4)**	**-0·8 (-1·3–-0·3)**
Unsafe water, sanitation, and handwashing	2·2 (1·2–3·4)	2·0 (1·0–3·3)	1·5 (0·6–2·8)	1·3 (0·4–2·3)	-1·7 (-4·0–0·2)	-2·1 (-4·8–0·1)	-1·6 (-5·2–1·9)
Unsafe water source	3·1 (1·1–6·9)	2·8 (1·1–5·9)	1·1 (0·5–2·2)	0·9 (0·4–1·8)	-3·9 (-6·8–-1·1)	-5·3 (-9·5–-1·5)	-1·7 (-7·6–3·7)
Unsafe sanitation	7·5 (4·2–12·5)	6·0 (3·2–10·2)	4·5 (2·3–7·7)	3·5 (1·8–6·2)	-2·5 (-4·6–-0·4)	-2·5 (-5·1–0·2)	-2·2 (-5·6–1·3)
No access to handwashing facility	1·8 (0·6–3·2)	1·8 (0·6–3·5)	1·8 (0·6–3·3)	1·6 (0·5–3·2)	-0·4 (-3·2–2·4)	-0·7 (-4·6–2·8)	-1·3 (-6·7–3·9)
Air pollution	11·1 (1·8–21·8)	11·4 (3·1–21·8)	6·6 (1·5–15·3)	5·3 (0·6–13·6)	-2·4 (-6·8–-0·7)	-3·6 (-9·1–-2·1)	-2·0 (-9·6–-0·7)
Particulate matter pollution	5·4 (0·5–12·6)	5·1 (2·5–8·4)	3·2 (0·9–6·3)	1·8 (0·1–4·1)	-3·6 (-11·8–2·0)	-5·1 (-14·7–-2·9)	-5·5 (-20·5–-2·8)
Ambient particulate matter pollution	8·3 (0·8–19·0)	7·8 (3·7–12·4)	4·9 (1·5–9·5)	2·7 (0·2–6·2)	-3·6 (-11·8–1·9)	-5·1 (-14·7–-2·9)	-5·5 (-20·4–-2·7)
Household air pollution from solid fuels	0·0 (0·0–0·1)	0·0 (0·0–0·1)	0·0 (0·0–0·0)	0·0 (0·0–0·0)	-4·3 (-29·8–-6·3)	-4·5 (-28·1–-6·1)	-4·0 (-29·9–-5·7)
Ambient ozone pollution	16·2 (10·5–22·9)	18·9 (13·2–26·3)	18·3 (12·4–26·1)	15·4 (9·4–22·4)	-0·2 (-0·9–0·5)	-1·0 (-2·2–0·1)	-1·6 (-4·0–0·2)
Ambient nitrogen dioxide pollution	34·9 (0·0–89·5)	37·8 (0·0–93·2)	20·0 (0·0–65·9)	20·1 (0·0–65·8)	-1·8 (-6·7–0·0)	-3·0 (-11·4–0·0)	0·0 (-0·2–1·4)
Non-optimal temperature	33·8 (27·1–43·6)	34·9 (27·8–44·5)	32·7 (26·6–42·2)	37·8 (29·5–47·7)	0·4 (0·2–0·6)	0·4 (0·1–0·7)	1·3 (0·5–2·1)
High temperature	37·4 (25·1–50·0)	39·7 (27·2–52·9)	36·6 (24·8–48·8)	55·6 (39·8–70·8)	1·3 (0·8–1·8)	1·6 (1·0–2·3)	3·8 (2·4–5·4)
Low temperature	28·6 (26·1–32·4)	29·5 (26·4–33·9)	28·5 (25·2–32·9)	28·8 (25·6–33·2)	0·0 (-0·1–0·1)	-0·1 (-0·2–0·0)	0·1 (-0·1–0·3)
Other environmental risks	27·7 (13·0–48·4)	26·3 (13·0–47·4)	23·9 (12·2–44·6)	21·5 (10·4–41·5)	-0·8 (-1·5–0·0)	-1·0 (-1·8–0·0)	-1·0 (-2·0–0·0)
Residential radon	31·6 (0·0–100·0)	31·6 (0·0–100·0)	31·6 (0·0–100·0)	31·6 (0·0–100·0)	0·0 (0·0–0·0)	0·0 (0·0–0·0)	0·0 (0·0–0·0)
Lead exposure	25·9 (0·0–32·3)	23·9 (0·0–29·5)	20·4 (0·0–25·1)	16·9 (0·0–21·2)	-1·4 (-1·9–0·0)	-1·7 (-2·2–0·0)	-1·7 (-2·4–0·0)
Occupational risks	2·8 (2·4–3·6)	2·9 (2·4–3·8)	2·8 (2·3–3·6)	2·8 (2·3–3·6)	-0·1 (-0·3–0·2)	-0·3 (-0·6–0·1)	0·1 (-0·4–0·5)
Occupational carcinogens	0·9 (0·4–1·8)	1·0 (0·5–1·9)	1·0 (0·4–1·9)	1·0 (0·5–1·9)	0·3 (-0·1–0·5)	0·0 (-0·4–0·4)	0·2 (-0·4–0·8)
Occupational exposure to asbestos	4·5 (3·9–5·0)	4·6 (4·1–5·2)	4·6 (4·0–5·1)	4·3 (3·4–5·3)	-0·1 (-0·9–0·6)	-0·4 (-1·6–0·8)	-0·6 (-2·8–1·6)
Occupational exposure to arsenic	0·5 (0·0–1·1)	0·5 (0·0–1·2)	0·5 (0·0–1·1)	0·5 (0·0–1·1)	0·0 (-0·5–0·5)	-0·3 (-0·9–0·3)	0·2 (-0·7–1·1)
Occupational exposure to benzene	1·0 (0·1–3·0)	1·1 (0·1–3·3)	1·1 (0·1–3·3)	1·2 (0·1–3·4)	0·4 (0·1–0·8)	0·2 (-0·2–0·6)	0·4 (-0·3–1·0)
Occupational exposure to beryllium	0·0 (0·0–0·0)	0·0 (0·0–0·0)	0·0 (0·0–0·0)	0·0 (0·0–0·0)	0·2 (0·0–0·4)	0·1 (-0·3–0·4)	0·5 (0·0–0·9)
Occupational exposure to cadmium	0·1 (0·1–0·1)	0·1 (0·1–0·1)	0·1 (0·1–0·1)	0·1 (0·1–0·1)	0·0 (-0·4–0·4)	-0·2 (-0·7–0·4)	0·2 (-0·6–1·0)
Occupational exposure to chromium	0·1 (0·1–0·1)	0·1 (0·1–0·2)	0·1 (0·1–0·1)	0·1 (0·1–0·2)	0·1 (-0·2–0·5)	-0·1 (-0·6–0·4)	0·3 (-0·4–1·0)
Occupational exposure to diesel engine exhaust	0·7 (0·6–0·7)	0·7 (0·6–0·7)	0·7 (0·6–0·7)	0·7 (0·7–0·8)	0·2 (-0·1–0·6)	0·3 (-0·2–0·8)	0·7 (-0·1–1·4)
Occupational exposure to formaldehyde	0·3 (0·3–0·3)	0·3 (0·3–0·3)	0·3 (0·3–0·3)	0·3 (0·3–0·3)	0·0 (-0·4–0·5)	-0·2 (-0·8–0·3)	0·1 (-0·6–0·9)
Occupational exposure to nickel	0·4 (0·0–1·5)	0·4 (0·0–1·5)	0·4 (0·0–1·4)	0·4 (0·0–1·5)	-0·1 (-0·7–0·4)	-0·3 (-1·0–0·4)	0·2 (-0·9–1·3)
Occupational exposure to polycyclic aromatic hydrocarbons	0·3 (0·3–0·3)	0·3 (0·3–0·3)	0·3 (0·3–0·3)	0·3 (0·3–0·3)	0·2 (-0·2–0·5)	-0·1 (-0·5–0·4)	0·3 (-0·3–0·9)
Occupational exposure to silica	3·5 (0·5–11·1)	3·4 (0·5–11·2)	3·2 (0·5–10·3)	3·3 (0·5–10·4)	-0·2 (-0·6–0·3)	-0·2 (-0·9–0·4)	0·3 (-0·7–1·3)
Occupational exposure to sulphuric acid	0·7 (0·1–2·3)	0·8 (0·2–2·5)	0·7 (0·1–2·3)	0·7 (0·1–2·4)	0·0 (-0·4–0·4)	-0·2 (-0·8–0·3)	0·2 (-0·7–1·1)
Occupational exposure to trichloroethylene	0·1 (0·1–0·1)	0·1 (0·1–0·1)	0·1 (0·1–0·1)	0·1 (0·1–0·1)	0·2 (-0·1–0·4)	0·0 (-0·4–0·4)	0·4 (-0·1–1·0)
Occupational asthmagens	16·3 (14·2–18·9)	17·1 (15·1–19·5)	15·8 (13·9–17·9)	16·3 (14·1–18·6)	0·0 (-0·4–0·3)	-0·2 (-0·7–0·3)	0·3 (-0·5–1·0)
Occupational particulate matter, gases, and fumes	6·8 (5·1–8·9)	6·7 (5·1–8·8)	6·3 (4·9–8·3)	5·9 (4·5–7·5)	-0·5 (-0·7–-0·2)	-0·6 (-0·9–-0·3)	-0·7 (-1·1–-0·3)
Occupational noise	7·0 (6·5–7·7)	7·1 (6·6–7·7)	6·9 (6·5–7·5)	6·7 (6·3–7·2)	-0·2 (-0·3–0·0)	-0·3 (-0·4–-0·1)	-0·3 (-0·5–-0·1)
Occupational injuries	--	--	--	--	--	--	--
Occupational ergonomic factors	8·8 (7·3–10·7)	8·7 (7·3–10·5)	7·6 (6·5–9·1)	7·8 (6·5–9·5)	-0·4 (-0·9–0·1)	-0·5 (-1·2–0·1)	0·2 (-0·5–0·9)
**Behavioural risks**	**28·1 (24·9–31·8)**	**26·5 (23·3–30·4)**	**24·4 (21·2–28·3)**	**22·5 (19·3–26·5)**	**-0·7 (-1·1–-0·3)**	**-0·8 (-1·3–-0·3)**	**-0·7 (-1·5–0·0)**
Child and maternal malnutrition	10·1 (7·0–14·5)	8·8 (5·8–12·3)	8·9 (6·0–12·4)	9·1 (6·1–13·0)	-0·3 (-1·2–0·6)	0·2 (-1·0–1·3)	0·2 (-1·4–1·8)
Suboptimal breastfeeding	68·9 (66·3–71·6)	66·8 (64·1–69·4)	63·0 (60·2–65·7)	62·3 (59·6–65·1)	-0·3 (-0·5–-0·2)	-0·3 (-0·5–-0·2)	-0·1 (-0·4–0·2)
Non-exclusive breastfeeding	64·5 (56·3–72·2)	60·0 (52·8–66·5)	49·0 (42·3–55·7)	46·7 (40·0–54·1)	-1·0 (-1·5–-0·6)	-1·2 (-1·9–-0·6)	-0·4 (-1·4–0·6)
Discontinued breastfeeding	88·8 (86·7–91·0)	86·5 (83·8–89·1)	84·5 (81·4–87·6)	84·7 (81·5–87·7)	-0·2 (-0·3–0·0)	-0·1 (-0·3–0·1)	0·0 (-0·3–0·3)
Child growth failure	1·4 (0·5–3·1)	1·3 (0·4–2·9)	1·1 (0·4–2·5)	1·0 (0·3–2·3)	-1·0 (-1·4–-0·6)	-1·0 (-1·6–-0·6)	-0·7 (-1·5–0·1)
Child underweight	2·9 (1·9–4·0)	2·7 (1·7–3·6)	2·3 (1·4–3·1)	2·1 (1·3–3·0)	-1·0 (-1·5–-0·6)	-1·1 (-1·8–-0·5)	-0·6 (-1·5–0·4)
Child wasting	0·9 (0·6–1·4)	0·9 (0·5–1·3)	0·9 (0·5–1·3)	0·8 (0·5–1·3)	-0·4 (-0·7–0·0)	-0·2 (-0·6–0·3)	-0·3 (-1·0–0·5)
Child stunting	4·0 (3·1–4·9)	3·6 (2·9–4·5)	3·1 (2·4–3·9)	2·8 (2·2–3·6)	-1·1 (-1·6–-0·5)	-1·2 (-1·9–-0·5)	-0·8 (-2·0–0·4)
Low birth weight and short gestation	17·2 (14·9–19·5)	17·9 (15·6–20·5)	19·3 (16·7–21·6)	19·8 (17·4–22·4)	0·5 (0·2–0·7)	0·5 (0·1–0·9)	0·2 (-0·5–1·0)
Short gestation	32·5 (29·0–37·3)	31·9 (28·3–36·2)	32·2 (28·6–36·4)	32·7 (28·8–36·9)	0·0 (-0·3–0·3)	0·1 (-0·4–0·6)	0·1 (-0·7–1·0)
Low birth weight	13·0 (11·9–14·5)	13·9 (12·6–15·3)	15·4 (14·1–16·7)	15·9 (14·4–17·2)	0·6 (0·4–0·9)	0·6 (0·3–1·0)	0·3 (-0·4–1·0)

Iron deficiency	3·6 (2·5–5·0)	3·1 (2·1–4·2)	3·2 (2·2–4·4)	3·3 (2·3–4·5)	-0·3 (-1·1–0·6)	0·2 (-0·9–1·3)	0·2 (-1·4–1·6)
Vitamin A deficiency	1·3 (0·0–2·7)	1·8 (0·0–3·7)	1·1 (0·0–2·4)	0·7 (0·0–1·5)	-2·0 (-4·3–0·3)	-4·5 (-7·2–0·0)	-4·3 (-8·3–0·0)
Zinc deficiency	2·2 (0·0–8·9)	1·2 (0·0–5·1)	1·1 (0·0–4·9)	1·1 (0·0–4·8)	-2·1 (-46·9–0·0)	-0·3 (-3·5–2·4)	0·0 (-1·6–5·6)
Tobacco	35·8 (34·2–37·2)	32·7 (31·4–33·9)	26·7 (25·7–27·5)	22·7 (21·2–24·4)	-1·5 (-1·7–-1·2)	-1·7 (-2·0–-1·4)	-1·5 (-2·1–-0·9)
Smoking	33·5 (31·3–35·7)	29·7 (27·9–31·4)	23·6 (22·3–25·0)	20·0 (18·4–22·2)	-1·7 (-2·0–-1·3)	-1·9 (-2·3–-1·4)	-1·5 (-2·3–-0·7)
Chewing tobacco	2·5 (2·0–3·1)	2·8 (2·1–3·6)	3·1 (2·3–4·1)	3·1 (2·2–4·4)	0·7 (-0·6–2·0)	0·4 (-1·4–2·5)	-0·1 (-2·8–2·9)
Second-hand smoke	28·9 (27·2–30·0)	26·9 (25·3–28·0)	22·4 (21·1–23·4)	19·3 (17·5–21·0)	-1·3 (-1·6–-1·0)	-1·6 (-2·0–-1·2)	-1·4 (-2·1–-0·7)
High alcohol use	25·5 (18·2–37·1)	23·4 (16·0–33·9)	23·7 (16·4–33·9)	23·5 (15·2–33·0)	-0·3 (-1·4–0·9)	0·0 (-1·6–1·5)	-0·1 (-2·4–2·1)
Drug use	0·9 (0·6–1·4)	1·1 (0·9–1·3)	1·8 (1·5–2·2)	4·1 (2·9–5·2)	5·0 (2·5–6·9)	6·4 (4·2–7·9)	7·4 (5·4–8·8)
Dietary risks	36·6 (27·4–47·6)	40·4 (31·3–52·2)	43·2 (33·4–55·5)	43·6 (34·4–55·6)	0·6 (0·3–0·9)	0·4 (0·1–0·7)	0·1 (-0·3–0·5)
Diet low in fruits	38·4 (32·2–41·4)	35·2 (29·2–38·1)	35·2 (30·8–38·2)	37·3 (32·1–41·6)	-0·1 (-0·5–0·3)	0·3 (-0·3–0·8)	0·5 (-0·2–1·3)
Diet low in vegetables	23·7 (14·9–31·5)	30·1 (17·3–37·8)	39·8 (21·6–47·6)	39·9 (22·6–48·7)	1·7 (1·2–2·3)	1·3 (0·9–1·8)	0·0 (-0·5–0·7)
Diet low in legumes	39·4 (0·0–49·1)	39·5 (0·0–49·4)	44·1 (0·0–55·1)	44·6 (0·0–56·5)	0·4 (0·0–0·8)	0·6 (0·0–1·0)	0·1 (-0·7–0·8)
Diet low in whole grains	45·1 (37·8–51·3)	48·5 (40·6–55·3)	50·4 (42·0–57·9)	50·6 (41·7–58·4)	0·4 (0·1–0·6)	0·2 (-0·2–0·6)	0·0 (-0·5–0·6)
Diet low in nuts and seeds	14·9 (10·9–19·3)	8·9 (6·0–12·4)	6·8 (4·5–9·6)	7·1 (4·2–10·5)	-2·4 (-4·0–-1·1)	-1·1 (-3·4–0·8)	0·4 (-2·8–3·4)
Diet low in milk	38·0 (33·4–42·2)	34·9 (30·3–39·4)	34·0 (29·4–38·3)	33·7 (28·6–38·7)	-0·4 (-0·8–0·1)	-0·2 (-0·9–0·4)	-0·1 (-0·9–0·8)
Diet high in red meat	52·2 (0·0–71·2)	53·6 (0·0–72·1)	53·6 (0·0–73·2)	53·2 (0·0–72·9)	0·1 (-0·5–0·5)	0·0 (-0·7–0·5)	-0·1 (-1·2–0·7)
Diet high in processed meat	44·2 (35·3–49·3)	55·1 (43·7–61·7)	61·3 (49·6–68·4)	59·3 (47·2–67·4)	0·9 (0·5–1·4)	0·3 (-0·2–0·9)	-0·3 (-1·1–0·4)
Diet high in sugar-sweetened beverages	33·4 (25·1–41·4)	47·5 (37·3–56·5)	52·1 (41·4–61·8)	51·6 (41·0–61·5)	1·4 (0·6–2·3)	0·4 (-0·5–1·4)	-0·1 (-1·3–1·5)
Diet low in fibre	41·9 (21·7–49·9)	32·1 (17·4–39·2)	29·3 (15·8–36·3)	25·8 (14·4–33·2)	-1·6 (-2·6–-0·7)	-1·0 (-2·5–0·2)	-1·2 (-3·1–0·6)
Diet low in calcium	6·1 (4·9–7·5)	5·0 (4·0–6·2)	5·4 (4·4–6·8)	5·4 (4·3–6·5)	-0·4 (-0·6–-0·2)	0·4 (0·1–0·6)	-0·1 (-0·5–0·4)
Diet low in seafood omega-3 fatty acids	52·5 (42·2–63·6)	58·3 (47·3–69·9)	61·8 (50·6–73·3)	56·8 (46·3–68·2)	0·3 (0·0–0·5)	-0·1 (-0·5–0·2)	-0·8 (-1·2–-0·3)
Diet low in omega-6 polyunsaturated fatty acids	38·9 (24·9–49·3)	33·7 (22·6–43·0)	25·8 (18·1–33·2)	23·9 (16·6–30·7)	-1·6 (-2·3–-0·8)	-1·6 (-2·5–-0·7)	-0·7 (-1·7–0·2)
Diet high in trans fatty acids	71·4 (63·9–78·7)	71·8 (65·4–78·9)	63·3 (55·6–71·1)	0·0 (0·0–0·0)	-65·8 (-66·1–-65·4)	-97·1 (-97·6–-96·7)	-184·2 (-185·3–-183·1)
Diet high in sodium	23·2 (0·7–68·4)	28·0 (2·0–73·9)	30·8 (3·7–73·2)	31·4 (3·1–76·1)	1·0 (0·3–5·7)	0·5 (-0·1–3·7)	0·2 (-2·6–1·9)
Intimate partner violence	23·4 (12·2–30·4)	23·4 (12·4–29·8)	21·1 (13·3–26·1)	20·6 (11·2–28·6)	-0·4 (-1·8–1·0)	-0·6 (-2·4–1·3)	-0·2 (-2·6–1·9)
Childhood sexual abuse and bullying	9·6 (6·2–14·8)	11·5 (7·6–17·6)	13·0 (8·6–20·0)	10·5 (7·3–16·0)	0·3 (-0·3–0·9)	-0·4 (-1·3–0·4)	-2·0 (-3·6–-0·5)
Childhood sexual abuse	9·8 (8·3–11·5)	11·0 (9·4–12·6)	12·5 (11·0–14·3)	11·5 (9·6–14·1)	0·5 (-0·2–1·3)	0·2 (-0·7–1·3)	-0·7 (-2·2–0·7)
Bullying victimization	8·1 (3·5–16·2)	10·0 (4·6–19·4)	11·3 (5·1–21·9)	8·2 (3·6–15·9)	0·1 (-0·6–0·8)	-1·0 (-2·0–0·1)	-3·0 (-4·8–-1·0)
Unsafe sex	--	--	--	--	--	--	--
Low physical activity	17·7 (13·1–23·0)	16·8 (12·6–21·4)	17·4 (13·1–22·1)	20·3 (15·0–26·5)	0·4 (-0·6–1·5)	0·9 (-0·4–2·2)	1·4 (-0·6–3·3)
**Metabolic risks**	**24·3 (21·3–27·7)**	**29·5 (25·8–32·4)**	**33·4 (29·3–36·2)**	**36·4 (31·8–39·5)**	**1·3 (1·0–1·6)**	**1·0 (0·7–1·3)**	**0·8 (0·3–1·2)**
High fasting plasma glucose	12·1 (8·9–14·6)	15·7 (11·9–19·3)	19·8 (14·8–23·7)	22·8 (17·0–27·7)	2·1 (1·3–2·8)	1·8 (0·8–2·7)	1·3 (0·1–2·6)
High LDL cholesterol	63·2 (44·3–86·8)	53·7 (36·9–74·4)	48·2 (32·4–67·5)	46·5 (31·3–64·8)	-1·0 (-1·2–-0·8)	-0·7 (-0·9–-0·5)	-0·3 (-0·6–0·0)
High systolic blood pressure	27·8 (19·0–38·8)	24·0 (16·7–33·2)	17·8 (11·4–25·7)	24·5 (15·3–35·7)	-0·4 (-1·4–0·5)	0·1 (-1·1–1·2)	2·9 (0·7–5·3)
High body-mass index	27·7 (23·1–32·0)	34·1 (28·9–38·1)	38·7 (33·1–42·4)	41·5 (34·5–45·2)	1·3 (1·0–1·6)	0·9 (0·6–1·2)	0·6 (0·2–1·0)
Low bone mineral density	21·4 (15·3–28·2)	19·5 (13·7–26·4)	20·1 (14·5–26·4)	22·1 (15·9–29·6)	0·1 (-0·4–0·5)	0·6 (-0·3–1·4)	0·9 (-0·6–2·3)
Kidney dysfunction	2·8 (2·3–3·7)	2·9 (2·4–3·8)	2·8 (2·3–3·8)	3·0 (2·4–4·0)	0·2 (0·1–0·3)	0·1 (0·0–0·3)	0·4 (0·1–0·7)

Illinois

Risk Names	SEV 1990	SEV 2000	SEV 2010	SEV 2021	Annualised rate of change 1990 to 2021	Annualised rate of change 2000 to 2021	Annualised rate of change 2010 to 2021
**All risk factors**	**27·8 (24·8–31·4)**	**27·6 (24·5–31·1)**	**28·4 (25·2–31·8)**	**28·2 (24·6–31·7)**	**0·0 (-0·3–0·3)**	**0·1 (-0·3–0·5)**	**-0·1 (-0·6–0·5)**
**Environmental/occupational risks**	**20·5 (13·7–31·2)**	**19·3 (13·4–30·0)**	**16·9 (11·6–27·3)**	**15·0 (9·9–24·4)**	**-1·0 (-1·8–-0·3)**	**-1·2 (-1·6–-0·7)**	**-1·1 (-1·5–-0·6)**
Unsafe water, sanitation, and handwashing	1·9 (1·0–3·0)	1·7 (0·8–2·8)	1·3 (0·4–2·2)	1·0 (0·3–1·7)	-1·9 (-4·2–0·1)	-2·3 (-5·5–0·4)	-1·9 (-5·9–1·8)
Unsafe water source	2·6 (1·0–5·4)	2·3 (0·9–4·6)	0·9 (0·4–1·9)	0·8 (0·3–1·5)	-4·0 (-6·8–-1·2)	-5·4 (-9·0–-1·4)	-1·8 (-7·4–3·4)
Unsafe sanitation	5·9 (3·2–9·8)	4·5 (2·3–7·4)	3·2 (1·7–5·6)	2·5 (1·3–4·3)	-2·8 (-5·0–-0·7)	-2·8 (-5·6–0·1)	-2·4 (-6·0–1·3)
No access to handwashing facility	1·6 (0·5–3·1)	1·6 (0·4–3·1)	1·6 (0·5–2·9)	1·3 (0·4–2·4)	-0·6 (-3·2–2·3)	-0·9 (-4·6–3·3)	-1·6 (-7·3–3·8)
Air pollution	28·9 (9·7–40·6)	27·0 (10·9–33·1)	19·3 (7·0–28·1)	12·5 (4·6–22·8)	-2·7 (-4·9–-0·9)	-3·7 (-6·2–-1·5)	-4·0 (-6·2–-1·5)
Particulate matter pollution	16·2 (6·9–27·0)	14·3 (10·6–18·4)	9·8 (6·7–13·7)	6·9 (4·0–10·4)	-2·7 (-4·6–-0·5)	-3·5 (-5·0–-2·6)	-3·2 (-5·2–-2·1)
Ambient particulate matter pollution	25·3 (10·6–42·3)	22·2 (17·1–27·6)	15·1 (10·7–20·2)	10·6 (6·2–15·6)	-2·8 (-4·7–-0·5)	-3·5 (-5·0–-2·7)	-3·2 (-5·2–-2·1)
Household air pollution from solid fuels	0·0 (0·0–0·0)	0·0 (0·0–0·0)	0·0 (0·0–0·0)	0·0 (0·0–0·0)	-3·0 (-32·1–-6·1)	-3·5 (-27·6–-4·9)	-3·9 (-22·8–-3·5)
Ambient ozone pollution	15·8 (10·6–22·8)	15·8 (10·8–22·4)	14·3 (9·2–20·6)	15·7 (10·3–22·5)	0·0 (-0·4–0·4)	0·0 (-0·5–0·5)	0·8 (0·0–1·9)
Ambient nitrogen dioxide pollution	83·7 (0·0–100·0)	81·8 (0·0–100·0)	60·2 (0·0–100·0)	35·5 (0·0–90·3)	-2·8 (-7·7–0·0)	-4·0 (-10·3–0·0)	-4·8 (-11·0–0·0)
Non-optimal temperature	33·7 (25·9–45·8)	32·2 (25·0–44·2)	35·9 (27·7–48·1)	34·6 (26·5–47·1)	0·1 (-0·2–0·4)	0·3 (0·0–0·7)	-0·3 (-0·8–-0·1)
High temperature	32·6 (21·0–44·6)	27·5 (16·2–39·0)	38·6 (25·7–50·8)	34·2 (21·7–46·0)	0·2 (-0·1–0·4)	1·0 (0·5–1·9)	-1·1 (-1·9–-0·5)
Low temperature	29·0 (26·0–32·3)	28·7 (26·1–31·5)	29·8 (27·4–32·8)	30·0 (27·3–33·1)	0·1 (-0·1–0·2)	0·2 (0·0–0·4)	0·1 (-0·4–0·3)
Other environmental risks	31·1 (11·1–53·4)	28·9 (11·0–51·5)	25·7 (11·0–47·8)	22·2 (11·0–44·2)	-1·1 (-1·8–0·0)	-1·2 (-2·0–0·0)	-1·3 (-2·4–0·0)
Residential radon	28·7 (0·0–94·8)	28·7 (0·0–94·8)	28·7 (0·0–94·8)	28·7 (0·0–94·8)	0·0 (0·0–0·0)	0·0 (0·0–0·0)	0·0 (0·0–0·0)
Lead exposure	32·1 (0·0–39·7)	28·9 (0·0–35·5)	24·2 (0·0–30·1)	19·3 (0·0–23·8)	-1·6 (-2·1–0·0)	-1·9 (-2·4–0·0)	-2·1 (-2·9–0·0)
Occupational risks	3·0 (2·5–3·9)	3·1 (2·5–4·0)	2·8 (2·3–3·7)	2·8 (2·3–3·7)	-0·2 (-0·5–0·1)	-0·4 (-0·7–0·0)	0·0 (-0·5–0·4)
Occupational carcinogens	1·0 (0·5–1·9)	1·1 (0·5–2·1)	1·0 (0·5–2·0)	1·0 (0·5–1·9)	0·1 (-0·3–0·4)	-0·2 (-0·7–0·2)	0·1 (-0·6–0·6)
Occupational exposure to asbestos	4·8 (4·4–5·4)	5·3 (4·9–5·8)	4·4 (3·9–4·7)	3·8 (3·0–4·8)	-0·8 (-1·6–0·0)	-1·6 (-2·7–-0·6)	-1·2 (-3·2–0·7)
Occupational exposure to arsenic	0·5 (0·0–1·3)	0·5 (0·0–1·3)	0·5 (0·0–1·2)	0·5 (0·0–1·2)	-0·3 (-0·8–0·1)	-0·4 (-1·0–0·2)	0·0 (-0·8–0·9)
Occupational exposure to benzene	1·1 (0·1–3·2)	1·2 (0·1–3·5)	1·2 (0·1–3·4)	1·2 (0·1–3·5)	0·4 (0·0–0·7)	0·1 (-0·3–0·5)	0·3 (-0·2–0·9)
Occupational exposure to beryllium	0·0 (0·0–0·0)	0·0 (0·0–0·0)	0·0 (0·0–0·0)	0·0 (0·0–0·0)	0·2 (0·0–0·4)	0·1 (-0·3–0·4)	0·4 (0·0–0·8)
Occupational exposure to cadmium	0·1 (0·1–0·1)	0·1 (0·1–0·1)	0·1 (0·1–0·1)	0·1 (0·1–0·1)	-0·2 (-0·6–0·2)	-0·3 (-0·9–0·2)	0·1 (-0·6–0·8)
Occupational exposure to chromium	0·2 (0·1–0·2)	0·2 (0·1–0·2)	0·1 (0·1–0·2)	0·1 (0·1–0·2)	-0·1 (-0·5–0·2)	-0·3 (-0·7–0·2)	0·2 (-0·5–0·8)
Occupational exposure to diesel engine exhaust	0·7 (0·6–0·8)	0·7 (0·7–0·8)	0·7 (0·7–0·8)	0·8 (0·7–0·8)	0·3 (-0·1–0·7)	0·2 (-0·2–0·7)	0·6 (0·0–1·3)
Occupational exposure to formaldehyde	0·3 (0·3–0·3)	0·3 (0·3–0·3)	0·3 (0·3–0·3)	0·3 (0·3–0·3)	-0·3 (-0·8–0·1)	-0·4 (-1·0–0·1)	0·0 (-0·7–0·7)
Occupational exposure to nickel	0·4 (0·0–1·8)	0·4 (0·0–1·6)	0·4 (0·0–1·5)	0·4 (0·0–1·5)	-0·4 (-1·0–0·1)	-0·5 (-1·2–0·2)	-0·1 (-0·9–1·0)
Occupational exposure to polycyclic aromatic hydrocarbons	0·3 (0·3–0·3)	0·3 (0·3–0·3)	0·3 (0·3–0·3)	0·3 (0·3–0·3)	-0·1 (-0·4–0·2)	-0·2 (-0·7–0·2)	0·2 (-0·5–0·7)
Occupational exposure to silica	3·8 (0·6–12·2)	3·6 (0·6–11·5)	3·3 (0·5–10·6)	3·3 (0·5–10·5)	-0·4 (-0·9–0·0)	-0·4 (-1·0–0·2)	0·1 (-0·7–1·0)
Occupational exposure to sulphuric acid	0·8 (0·2–2·7)	0·8 (0·2–2·8)	0·7 (0·2–2·4)	0·8 (0·2–2·4)	-0·2 (-0·7–0·2)	-0·3 (-0·9–0·2)	0·1 (-0·7–0·9)
Occupational exposure to trichloroethylene	0·1 (0·1–0·1)	0·1 (0·1–0·1)	0·1 (0·1–0·1)	0·1 (0·1–0·1)	0·0 (-0·2–0·3)	-0·1 (-0·5–0·3)	0·3 (-0·2–0·9)
Occupational asthmagens	17·8 (15·6–20·3)	18·0 (15·8–20·6)	16·6 (14·6–18·8)	16·8 (14·7–19·0)	-0·2 (-0·5–0·2)	-0·3 (-0·8–0·1)	0·1 (-0·5–0·8)
Occupational particulate matter, gases, and fumes	6·9 (5·3–9·2)	6·7 (5·1–8·8)	6·2 (4·8–8·2)	5·7 (4·4–7·4)	-0·6 (-0·9–-0·3)	-0·7 (-1·0–-0·4)	-0·8 (-1·1–-0·4)
Occupational noise	6·8 (6·2–7·4)	6·8 (6·3–7·4)	6·7 (6·3–7·2)	6·4 (6·1–7·0)	-0·2 (-0·3–0·0)	-0·3 (-0·4–-0·1)	-0·3 (-0·5–-0·1)
Occupational injuries	--	--	--	--	--	--	--
Occupational ergonomic factors	7·9 (6·6–9·5)	7·9 (6·7–9·6)	7·2 (6·0–8·8)	7·4 (6·1–9·0)	-0·2 (-0·6–0·2)	-0·4 (-0·8–0·2)	0·2 (-0·5–0·9)
**Behavioural risks**	**31·2 (28·1–34·9)**	**29·0 (25·7–32·7)**	**27·2 (24·1–31·0)**	**25·1 (21·7–28·4)**	**-0·7 (-1·1–-0·4)**	**-0·7 (-1·2–-0·3)**	**-0·8 (-1·5–-0·1)**
Child and maternal malnutrition	10·3 (7·2–14·2)	8·8 (6·2–12·3)	8·8 (6·2–12·2)	8·9 (6·1–12·7)	-0·5 (-1·3–0·4)	0·1 (-1·2–1·3)	0·2 (-1·6–1·9)
Suboptimal breastfeeding	68·8 (66·2–71·3)	66·6 (64·0–69·1)	62·8 (60·2–66·0)	62·1 (59·2–65·1)	-0·3 (-0·5–-0·2)	-0·3 (-0·6–-0·1)	-0·1 (-0·4–0·2)
Non-exclusive breastfeeding	64·2 (55·5–71·4)	59·5 (52·4–65·8)	48·5 (41·8–55·4)	46·2 (38·7–53·5)	-1·1 (-1·6–-0·6)	-1·2 (-1·9–-0·6)	-0·4 (-1·5–0·4)
Discontinued breastfeeding	88·7 (86·4–91·1)	86·3 (83·6–89·3)	84·3 (80·9–87·5)	84·5 (81·0–87·7)	-0·2 (-0·3–0·0)	-0·1 (-0·3–0·1)	0·0 (-0·3–0·3)
Child growth failure	1·3 (0·4–3·0)	1·2 (0·4–2·7)	1·0 (0·3–2·2)	0·9 (0·3–2·0)	-1·1 (-1·5–-0·8)	-1·2 (-1·7–-0·8)	-0·7 (-1·5–0·0)
Child underweight	2·7 (1·7–3·8)	2·5 (1·6–3·3)	2·1 (1·3–2·8)	1·9 (1·2–2·6)	-1·2 (-1·7–-0·7)	-1·2 (-1·9–-0·6)	-0·7 (-1·6–0·4)
Child wasting	0·9 (0·6–1·4)	0·8 (0·5–1·3)	0·8 (0·5–1·2)	0·8 (0·5–1·2)	-0·4 (-0·7–-0·2)	-0·2 (-0·7–0·2)	-0·3 (-1·0–0·3)
Child stunting	3·8 (3·0–4·7)	3·5 (2·7–4·3)	2·9 (2·2–3·6)	2·6 (2·0–3·3)	-1·2 (-1·8–-0·7)	-1·3 (-2·1–-0·6)	-0·9 (-2·0–0·3)
Low birth weight and short gestation	21·9 (18·9–24·9)	23·1 (19·9–26·3)	24·8 (21·3–28·3)	23·9 (20·6–27·3)	0·3 (0·0–0·5)	0·2 (-0·2–0·5)	-0·3 (-1·0–0·4)
Short gestation	40·9 (35·2–47·0)	43·4 (37·0–49·2)	45·5 (39·2–51·5)	43·0 (37·0–48·9)	0·2 (-0·2–0·4)	-0·1 (-0·4–0·4)	-0·5 (-1·4–0·3)
Low birth weight	16·8 (15·0–18·6)	17·1 (15·3–19·0)	18·4 (16·5–20·2)	18·2 (16·3–20·2)	0·3 (0·0–0·5)	0·3 (-0·1–0·6)	-0·1 (-0·8–0·6)

Iron deficiency	3·7 (2·6–5·0)	3·1 (2·2–4·2)	3·1 (2·3–4·3)	3·2 (2·2–4·5)	-0·4 (-1·3–0·3)	0·1 (-1·1–1·3)	0·1 (-1·6–1·7)
Vitamin A deficiency	1·0 (0·0–2·0)	1·2 (0·0–2·5)	0·7 (0·0–1·4)	0·4 (0·0–0·9)	-2·8 (-4·8–0·0)	-5·2 (-7·8–0·0)	-5·1 (-9·2–0·0)
Zinc deficiency	1·7 (0·0–7·1)	1·0 (0·0–4·1)	0·9 (0·0–4·0)	0·9 (0·0–3·9)	-1·9 (-45·6–0·0)	-0·3 (-3·8–1·7)	0·0 (-5·9–1·5)
Tobacco	41·9 (40·3–43·4)	35·9 (34·6–37·1)	28·6 (27·6–29·7)	23·8 (22·3–25·2)	-1·8 (-2·0–-1·6)	-2·0 (-2·3–-1·7)	-1·6 (-2·2–-1·1)
Smoking	38·6 (36·2–40·7)	32·3 (30·4–34·1)	25·9 (24·6–27·4)	21·3 (19·4–23·1)	-1·9 (-2·2–-1·6)	-2·0 (-2·5–-1·6)	-1·8 (-2·6–-1·1)
Chewing tobacco	1·7 (1·3–2·2)	1·8 (1·4–2·4)	1·9 (1·4–2·5)	1·9 (1·4–2·5)	0·3 (-0·9–1·5)	0·1 (-1·5–1·7)	0·0 (-2·7–2·7)
Second-hand smoke	34·4 (32·6–35·7)	30·0 (28·2–31·3)	23·9 (22·3–25·0)	20·4 (18·6–22·0)	-1·7 (-1·9–-1·5)	-1·9 (-2·2–-1·5)	-1·4 (-2·1–-0·8)
High alcohol use	28·5 (20·6–39·4)	27·2 (19·4–37·1)	29·0 (21·7–40·0)	29·1 (20·4–38·6)	0·1 (-0·9–1·1)	0·3 (-0·9–1·6)	0·0 (-1·8–1·8)
Drug use	0·9 (0·6–1·5)	1·2 (1·0–1·4)	1·8 (1·5–2·1)	4·3 (3·0–5·5)	4·9 (2·4–6·8)	6·2 (4·0–7·4)	7·8 (5·5–9·1)
Dietary risks	38·6 (29·6–49·8)	42·0 (33·0–54·2)	45·0 (35·4–57·1)	45·3 (36·4–57·8)	0·5 (0·3–0·8)	0·4 (0·0–0·7)	0·1 (-0·4–0·6)
Diet low in fruits	40·6 (35·6–43·9)	37·2 (32·5–40·1)	37·0 (32·4–40·1)	39·9 (34·0–44·2)	-0·1 (-0·4–0·3)	0·3 (-0·1–0·8)	0·7 (-0·1–1·5)
Diet low in vegetables	30·4 (18·3–38·3)	35·8 (21·4–43·4)	46·9 (24·8–54·9)	46·3 (25·5–55·0)	1·4 (0·9–1·8)	1·2 (0·7–1·7)	-0·1 (-0·8–0·6)
Diet low in legumes	37·2 (0·0–47·2)	37·2 (0·0–46·9)	41·6 (0·0–52·3)	42·2 (0·0–53·2)	0·4 (0·0–0·7)	0·6 (0·0–1·1)	0·1 (-0·6–0·8)
Diet low in whole grains	44·9 (37·4–50·6)	48·4 (39·8–54·8)	50·2 (41·3–57·4)	50·2 (41·3–58·0)	0·4 (0·1–0·6)	0·2 (-0·2–0·5)	0·0 (-0·5–0·6)
Diet low in nuts and seeds	11·3 (8·0–15·3)	6·3 (4·0–9·0)	4·7 (3·0–7·0)	5·1 (2·9–7·9)	-2·6 (-4·4–-0·8)	-1·0 (-3·8–1·5)	0·7 (-3·1–4·5)
Diet low in milk	35·0 (30·4–39·4)	32·0 (27·7–37·0)	31·1 (26·8–36·1)	30·8 (25·9–35·4)	-0·4 (-0·9–0·0)	-0·2 (-0·8–0·4)	-0·1 (-1·1–0·8)
Diet high in red meat	52·6 (0·0–71·7)	53·8 (0·0–72·9)	53·9 (0·0–73·4)	53·5 (0·0–72·3)	0·1 (-0·4–0·5)	0·0 (-0·6–0·6)	-0·1 (-0·9–0·8)
Diet high in processed meat	45·8 (35·9–50·9)	57·1 (45·4–63·4)	63·4 (51·4–71·0)	61·1 (50·2–69·5)	0·9 (0·5–1·3)	0·3 (-0·3–0·8)	-0·3 (-1·1–0·4)
Diet high in sugar-sweetened beverages	38·0 (29·1–45·7)	53·5 (43·6–61·8)	58·3 (47·3–67·4)	57·0 (45·6–67·5)	1·3 (0·5–2·1)	0·3 (-0·6–1·2)	-0·2 (-1·5–1·1)
Diet low in fibre	37·1 (20·5–44·1)	27·3 (15·0–33·7)	24·6 (13·9–30·5)	21·4 (12·4–27·8)	-1·8 (-2·8–-0·9)	-1·2 (-2·6–0·1)	-1·2 (-3·3–0·5)
Diet low in calcium	5·4 (4·3–6·5)	4·5 (3·7–5·6)	4·8 (3·9–6·0)	4·8 (3·8–5·9)	-0·4 (-0·6–-0·2)	0·2 (0·0–0·5)	-0·1 (-0·4–0·2)
Diet low in seafood omega-3 fatty acids	34·3 (25·3–43·8)	40·9 (31·6–51·3)	44·8 (35·3–55·4)	39·6 (29·8–49·3)	0·5 (0·0–0·9)	-0·2 (-0·7–0·4)	-1·1 (-1·9–-0·3)
Diet low in omega-6 polyunsaturated fatty acids	33·5 (21·9–43·5)	28·9 (19·5–37·5)	22·0 (15·3–28·4)	20·5 (14·1–26·8)	-1·6 (-2·3–-0·8)	-1·6 (-2·5–-0·8)	-0·6 (-1·6–0·3)
Diet high in trans fatty acids	71·5 (63·2–78·8)	71·9 (64·3–79·1)	63·4 (54·2–71·2)	0·0 (0·0–0·0)	-65·8 (-66·1–-65·4)	-97·1 (-97·6–-96·6)	-184·2 (-185·3–-182·8)
Diet high in sodium	23·6 (0·6–70·3)	28·1 (2·0–74·9)	30·5 (3·3–74·5)	30·8 (3·1–77·0)	0·9 (0·1–6·2)	0·4 (-0·3–3·4)	0·1 (-2·8–1·8)
Intimate partner violence	30·0 (17·6–38·4)	30·0 (18·7–37·4)	27·2 (18·2–33·5)	25·4 (13·5–34·1)	-0·5 (-2·0–0·6)	-0·8 (-2·8–0·7)	-0·6 (-3·2–1·2)
Childhood sexual abuse and bullying	9·1 (5·9–14·2)	11·0 (7·2–17·0)	12·6 (8·3–19·8)	10·0 (6·9–15·5)	0·3 (-0·2–0·9)	-0·4 (-1·2–0·4)	-2·1 (-3·6–-0·7)
Childhood sexual abuse	8·8 (7·5–10·4)	9·9 (8·6–11·4)	11·5 (10·1–13·1)	10·7 (8·9–13·2)	0·6 (-0·1–1·4)	0·3 (-0·6–1·3)	-0·7 (-2·1–0·5)
Bullying victimization	8·0 (3·5–16·0)	9·9 (4·6–19·4)	11·3 (5·1–22·1)	8·0 (3·5–15·8)	0·0 (-0·7–0·8)	-1·0 (-2·1–0·1)	-3·1 (-5·1–-1·0)
Unsafe sex	--	--	--	--	--	--	--
Low physical activity	14·2 (10·4–19·1)	13·1 (9·5–16·9)	18·4 (14·2–23·3)	21·6 (16·3–27·9)	1·4 (0·4–2·4)	2·4 (1·1–3·7)	1·5 (-0·5–3·2)
**Metabolic risks**	**25·8 (22·5–28·9)**	**30·7 (26·8–33·5)**	**34·2 (30·2–37·2)**	**37·4 (32·7–40·5)**	**1·2 (0·9–1·5)**	**0·9 (0·6–1·3)**	**0·8 (0·3–1·2)**
High fasting plasma glucose	12·4 (9·7–14·8)	16·1 (12·6–19·2)	20·5 (15·7–24·0)	23·7 (18·4–28·2)	2·1 (1·4–2·7)	1·8 (1·1–2·7)	1·3 (0·2–2·5)
High LDL cholesterol	63·5 (44·7–86·4)	53·8 (36·8–73·9)	48·3 (32·7–67·4)	46·7 (31·5–65·6)	-1·0 (-1·2–-0·8)	-0·7 (-0·9–-0·5)	-0·3 (-0·5–0·0)
High systolic blood pressure	29·9 (19·5–41·2)	25·7 (18·0–35·5)	19·1 (12·6–27·1)	26·9 (16·8–38·4)	-0·3 (-1·3–0·6)	0·2 (-1·0–1·4)	3·1 (0·8–5·6)
High body-mass index	29·6 (24·8–34·1)	35·7 (30·3–39·6)	39·7 (33·9–43·5)	42·7 (36·2–46·4)	1·2 (0·9–1·5)	0·9 (0·5–1·1)	0·7 (0·2–1·1)
Low bone mineral density	22·7 (16·6–29·6)	20·4 (14·3–27·2)	20·2 (14·0–26·7)	22·1 (15·8–28·7)	-0·1 (-0·6–0·4)	0·4 (-0·4–1·1)	0·8 (-0·5–2·3)
Kidney dysfunction	3·0 (2·4–3·9)	3·0 (2·5–4·0)	3·0 (2·4–4·0)	3·1 (2·5–4·1)	0·1 (0·0–0·2)	0·1 (-0·1–0·2)	0·3 (0·0–0·6)

Indiana

Risk Names	SEV 1990	SEV 2000	SEV 2010	SEV 2021	Annualised rate of change 1990 to 2021	Annualised rate of change 2000 to 2021	Annualised rate of change 2010 to 2021
**All risk factors**	**27·3 (24·1–30·4)**	**27·0 (24·1–30·1)**	**27·7 (24·6–30·8)**	**27·5 (24·2–30·8)**	**0·0 (-0·3–0·3)**	**0·1 (-0·3–0·5)**	**-0·1 (-0·6–0·5)**
**Environmental/occupational risks**	**20·0 (13·3–30·6)**	**19·1 (13·3–29·8)**	**16·7 (11·4–26·9)**	**14·7 (9·7–23·8)**	**-1·0 (-1·7–-0·3)**	**-1·2 (-1·6–-0·7)**	**-1·1 (-1·7–-0·5)**
Unsafe water, sanitation, and handwashing	2·0 (1·1–3·2)	1·9 (0·9–3·2)	1·5 (0·5–2·6)	1·2 (0·4–1·9)	-1·8 (-4·1–0·2)	-2·3 (-5·3–0·3)	-2·0 (-5·7–2·0)
Unsafe water source	2·9 (1·1–5·9)	2·7 (1·1–5·5)	1·1 (0·5–2·3)	0·9 (0·4–1·7)	-3·9 (-6·7–-1·1)	-5·4 (-9·1–-1·4)	-2·1 (-7·7–3·4)
Unsafe sanitation	6·9 (3·7–11·2)	5·6 (3·0–9·5)	4·3 (2·3–7·7)	3·2 (1·7–5·7)	-2·5 (-4·6–-0·4)	-2·7 (-5·4–0·2)	-2·8 (-6·1–0·8)
No access to handwashing facility	1·6 (0·6–3·0)	1·7 (0·6–3·3)	1·7 (0·6–3·2)	1·5 (0·5–2·8)	-0·4 (-3·4–2·7)	-0·7 (-4·5–2·9)	-1·5 (-7·0–3·6)
Air pollution	25·2 (9·4–37·6)	22·5 (10·5–31·7)	14·4 (6·6–25·1)	9·1 (3·5–17·9)	-3·3 (-5·5–-1·3)	-4·3 (-6·7–-2·6)	-4·2 (-7·1–-2·6)
Particulate matter pollution	15·5 (6·5–26·1)	13·8 (10·2–17·7)	9·5 (6·3–13·0)	6·2 (3·4–9·6)	-3·0 (-5·1–-0·6)	-3·8 (-5·4–-2·8)	-3·9 (-6·2–-2·4)
Ambient particulate matter pollution	24·2 (10·0–41·1)	21·3 (16·4–26·8)	14·6 (9·9–19·5)	9·5 (5·3–14·3)	-3·0 (-5·1–-0·6)	-3·8 (-5·5–-2·8)	-3·9 (-6·3–-2·4)
Household air pollution from solid fuels	0·0 (0·0–0·0)	0·0 (0·0–0·0)	0·0 (0·0–0·0)	0·0 (0·0–0·0)	-3·0 (-28·1–-5·4)	-2·5 (-22·5–-4·4)	-1·4 (-21·7–-4·1)
Ambient ozone pollution	23·5 (17·7–30·7)	25·4 (19·3–32·4)	17·9 (12·7–24·1)	11·7 (7·1–17·7)	-2·3 (-3·0–-1·7)	-3·7 (-5·0–-2·8)	-3·9 (-5·6–-2·5)
Ambient nitrogen dioxide pollution	64·8 (0·0–100·0)	57·8 (0·0–100·0)	33·5 (0·0–89·2)	20·2 (0·0–67·2)	-3·8 (-13·2–0·0)	-5·0 (-18·0–0·0)	-4·6 (-21·0–0·0)
Non-optimal temperature	31·7 (24·3–43·4)	30·7 (24·3–41·9)	35·8 (27·4–47·8)	33·2 (25·5–44·9)	0·1 (-0·2–0·5)	0·4 (0·0–0·8)	-0·7 (-1·2–-0·3)
High temperature	28·5 (17·9–39·6)	23·2 (12·5–35·0)	37·4 (24·9–49·8)	30·6 (19·3–41·6)	0·2 (-0·1–0·5)	1·3 (0·6–2·6)	-1·8 (-2·8–-1·0)
Low temperature	28·0 (24·6–31·5)	28·3 (25·6–31·1)	30·0 (27·6–33·1)	29·3 (26·4–32·6)	0·1 (0·0–0·3)	0·2 (0·0–0·3)	-0·2 (-0·6–0·1)
Other environmental risks	27·9 (9·0–45·7)	26·4 (9·0–44·9)	23·7 (9·0–42·0)	21·0 (9·0–39·2)	-0·9 (-1·7–0·0)	-1·1 (-1·9–0·0)	-1·1 (-2·2–0·0)
Residential radon	28·7 (0·0–84·3)	28·7 (0·0–84·3)	28·7 (0·0–84·3)	28·7 (0·0–84·3)	0·0 (0·0–0·0)	0·0 (0·0–0·0)	0·0 (0·0–0·0)
Lead exposure	27·5 (0·0–34·4)	25·2 (0·0–31·1)	21·4 (0·0–26·7)	17·5 (0·0–21·5)	-1·5 (-1·9–0·0)	-1·8 (-2·3–0·0)	-1·8 (-2·6–0·0)
Occupational risks	3·1 (2·6–4·0)	3·2 (2·6–4·1)	3·0 (2·4–3·8)	2·9 (2·4–3·8)	-0·2 (-0·5–0·1)	-0·4 (-0·8–-0·1)	0·0 (-0·5–0·4)
Occupational carcinogens	1·0 (0·5–1·8)	1·0 (0·5–2·0)	1·0 (0·5–1·9)	1·0 (0·5–1·9)	0·1 (-0·2–0·4)	-0·1 (-0·5–0·3)	0·2 (-0·5–0·7)
Occupational exposure to asbestos	4·1 (3·6–4·5)	4·4 (4·0–4·8)	4·3 (3·8–4·6)	4·1 (3·3–5·1)	0·0 (-0·7–0·8)	-0·3 (-1·4–0·8)	-0·3 (-2·2–1·7)
Occupational exposure to arsenic	0·6 (0·0–1·4)	0·6 (0·0–1·5)	0·5 (0·0–1·3)	0·5 (0·0–1·3)	-0·4 (-0·9–0·1)	-0·7 (-1·2–0·1)	-0·1 (-1·0–0·7)
Occupational exposure to benzene	1·1 (0·1–2·9)	1·1 (0·1–3·4)	1·1 (0·1–3·4)	1·2 (0·1–3·5)	0·4 (0·0–0·7)	0·2 (-0·3–0·5)	0·4 (-0·2–1·0)
Occupational exposure to beryllium	0·0 (0·0–0·0)	0·0 (0·0–0·0)	0·0 (0·0–0·0)	0·0 (0·0–0·0)	0·2 (-0·1–0·4)	0·0 (-0·3–0·3)	0·4 (0·0–0·9)
Occupational exposure to cadmium	0·1 (0·1–0·1)	0·1 (0·1–0·1)	0·1 (0·1–0·1)	0·1 (0·1–0·1)	-0·3 (-0·7–0·1)	-0·5 (-1·1–0·0)	0·0 (-0·8–0·8)
Occupational exposure to chromium	0·2 (0·2–0·2)	0·2 (0·2–0·2)	0·2 (0·1–0·2)	0·2 (0·1–0·2)	-0·2 (-0·6–0·2)	-0·4 (-0·9–0·1)	0·1 (-0·6–0·7)
Occupational exposure to diesel engine exhaust	0·7 (0·6–0·7)	0·7 (0·6–0·7)	0·7 (0·6–0·7)	0·7 (0·7–0·8)	0·3 (-0·1–0·7)	0·3 (-0·3–0·8)	0·7 (0·0–1·4)
Occupational exposure to formaldehyde	0·4 (0·3–0·4)	0·4 (0·3–0·4)	0·3 (0·3–0·3)	0·3 (0·3–0·3)	-0·4 (-0·8–0·0)	-0·6 (-1·2–0·0)	-0·1 (-0·9–0·6)
Occupational exposure to nickel	0·5 (0·0–1·9)	0·5 (0·0–1·9)	0·4 (0·0–1·7)	0·4 (0·0–1·6)	-0·5 (-1·0–0·0)	-0·7 (-1·4–0·0)	-0·1 (-1·3–0·9)
Occupational exposure to polycyclic aromatic hydrocarbons	0·3 (0·3–0·4)	0·3 (0·3–0·4)	0·3 (0·3–0·3)	0·3 (0·3–0·3)	-0·2 (-0·5–0·2)	-0·4 (-0·8–0·1)	0·1 (-0·6–0·7)
Occupational exposure to silica	4·2 (0·7–13·8)	4·1 (0·7–13·4)	3·6 (0·6–11·8)	3·6 (0·6–11·5)	-0·5 (-1·0–0·0)	-0·6 (-1·2–0·1)	0·0 (-0·9–0·9)
Occupational exposure to sulphuric acid	0·9 (0·2–2·9)	0·9 (0·2–2·9)	0·8 (0·2–2·6)	0·8 (0·2–2·6)	-0·3 (-0·7–0·1)	-0·5 (-1·1–0·1)	0·0 (-0·8–0·8)
Occupational exposure to trichloroethylene	0·1 (0·1–0·1)	0·1 (0·1–0·1)	0·1 (0·1–0·1)	0·1 (0·1–0·1)	0·0 (-0·3–0·3)	-0·2 (-0·6–0·2)	0·3 (-0·2–0·9)
Occupational asthmagens	18·9 (16·4–21·7)	19·3 (16·9–21·9)	17·3 (15·3–19·4)	17·4 (15·3–19·9)	-0·3 (-0·6–0·1)	-0·5 (-1·0–0·0)	0·1 (-0·6–0·8)
Occupational particulate matter, gases, and fumes	7·6 (5·8–10·0)	7·5 (5·8–9·6)	7·0 (5·4–9·2)	6·4 (4·9–8·3)	-0·6 (-0·8–-0·3)	-0·7 (-1·0–-0·4)	-0·8 (-1·2–-0·4)
Occupational noise	7·1 (6·6–7·8)	7·2 (6·7–7·9)	7·1 (6·6–7·7)	6·8 (6·4–7·3)	-0·2 (-0·3–0·0)	-0·3 (-0·4–-0·1)	-0·4 (-0·5–-0·1)
Occupational injuries	--	--	--	--	--	--	--
Occupational ergonomic factors	8·3 (6·9–10·2)	8·3 (7·0–10·0)	7·4 (6·3–8·8)	7·6 (6·3–9·1)	-0·3 (-0·8–0·2)	-0·4 (-1·0–0·1)	0·2 (-0·5–0·9)
**Behavioural risks**	**30·6 (27·2–34·3)**	**29·1 (25·9–32·7)**	**27·6 (24·6–31·2)**	**25·6 (22·2–29·3)**	**-0·6 (-0·9–-0·3)**	**-0·6 (-1·1–-0·2)**	**-0·7 (-1·4–0·0)**
Child and maternal malnutrition	10·2 (6·9–14·2)	8·8 (6·0–12·4)	9·0 (6·2–12·3)	9·2 (6·2–12·6)	-0·3 (-1·2–0·5)	0·2 (-0·9–1·4)	0·2 (-1·6–1·8)
Suboptimal breastfeeding	69·0 (66·3–71·5)	66·8 (64·3–69·4)	63·0 (60·2–65·5)	62·3 (59·7–65·6)	-0·3 (-0·5–-0·2)	-0·3 (-0·5–-0·1)	-0·1 (-0·4–0·2)
Non-exclusive breastfeeding	64·5 (56·9–71·2)	59·8 (52·6–66·2)	48·9 (41·8–55·6)	46·6 (39·5–54·6)	-1·0 (-1·6–-0·5)	-1·2 (-1·9–-0·6)	-0·4 (-1·4–0·5)
Discontinued breastfeeding	88·8 (86·5–91·3)	86·4 (83·8–88·9)	84·5 (81·5–87·9)	84·7 (81·4–87·7)	-0·2 (-0·3–0·0)	-0·1 (-0·3–0·1)	0·0 (-0·3–0·3)
Child growth failure	1·3 (0·4–3·1)	1·2 (0·4–2·8)	1·1 (0·4–2·5)	1·0 (0·3–2·3)	-0·8 (-1·3–-0·5)	-0·9 (-1·5–-0·4)	-0·6 (-1·4–0·2)
Child underweight	2·8 (1·7–3·8)	2·6 (1·6–3·5)	2·3 (1·4–3·1)	2·1 (1·3–2·9)	-0·8 (-1·4–-0·4)	-1·0 (-1·7–-0·3)	-0·5 (-1·6–0·5)
Child wasting	0·9 (0·6–1·4)	0·8 (0·5–1·3)	0·9 (0·5–1·3)	0·8 (0·5–1·3)	-0·2 (-0·5–0·1)	0·0 (-0·5–0·4)	-0·2 (-0·9–0·4)
Child stunting	3·8 (2·9–4·8)	3·6 (2·8–4·5)	3·1 (2·5–4·0)	2·9 (2·2–3·6)	-0·9 (-1·5–-0·4)	-1·0 (-1·8–-0·3)	-0·8 (-1·9–0·3)
Low birth weight and short gestation	21·6 (18·8–24·7)	22·7 (20·0–25·9)	23·3 (20·5–26·5)	23·1 (20·3–26·2)	0·2 (0·0–0·5)	0·1 (-0·3–0·5)	-0·1 (-0·8–0·6)
Short gestation	36·4 (32·2–41·1)	39·1 (34·7–43·6)	39·4 (35·0–44·6)	38·5 (34·0–43·2)	0·2 (-0·1–0·5)	-0·1 (-0·5–0·4)	-0·2 (-1·1–0·6)
Low birth weight	16·9 (15·5–18·5)	17·3 (16·0–18·9)	17·9 (16·4–19·5)	18·0 (16·5–19·8)	0·2 (0·0–0·5)	0·2 (-0·2–0·5)	0·1 (-0·7–0·8)

Iron deficiency	3·6 (2·5–5·0)	3·1 (2·2–4·3)	3·3 (2·3–4·3)	3·3 (2·3–4·4)	-0·3 (-1·1–0·5)	0·3 (-0·8–1·4)	0·2 (-1·6–1·6)
Vitamin A deficiency	1·1 (0·0–2·3)	1·6 (0·0–3·4)	1·0 (0·0–2·2)	0·6 (0·0–1·4)	-1·9 (-3·9–0·1)	-4·4 (-7·1–0·0)	-4·5 (-8·4–0·0)
Zinc deficiency	2·0 (0·0–8·1)	1·1 (0·0–4·9)	1·1 (0·0–4·6)	1·0 (0·0–4·5)	-2·0 (-41·6–0·0)	-0·3 (-1·8–3·5)	-0·1 (-2·2–5·5)
Tobacco	45·1 (43·3–46·7)	42·7 (41·1–44·1)	37·6 (36·3–38·9)	32·0 (29·9–34·1)	-1·1 (-1·3–-0·9)	-1·4 (-1·7–-1·1)	-1·5 (-2·1–-0·9)
Smoking	40·5 (38·2–42·6)	36·8 (34·9–38·6)	32·3 (30·6–33·8)	27·5 (25·4–30·0)	-1·2 (-1·5–-1·0)	-1·4 (-1·8–-1·0)	-1·4 (-2·1–-0·7)
Chewing tobacco	2·4 (2·0–2·9)	2·4 (1·9–3·0)	2·3 (1·7–3·1)	2·1 (1·6–3·0)	-0·3 (-1·4–0·8)	-0·6 (-2·4–1·0)	-0·6 (-3·3–1·9)
Second-hand smoke	40·3 (37·4–42·2)	39·6 (36·8–41·2)	35·6 (33·0–37·1)	30·3 (27·8–32·6)	-0·9 (-1·2–-0·7)	-1·3 (-1·6–-0·9)	-1·5 (-2·1–-0·8)
High alcohol use	26·1 (18·5–36·3)	24·3 (17·5–34·8)	25·0 (17·7–34·9)	25·3 (17·3–36·1)	-0·1 (-1·1–0·9)	0·2 (-1·4–1·5)	0·1 (-2·2–1·9)
Drug use	0·8 (0·5–1·2)	0·9 (0·8–1·1)	2·8 (1·9–3·6)	5·7 (3·7–7·4)	6·4 (3·8–8·3)	8·5 (6·1–9·9)	6·5 (5·6–7·4)
Dietary risks	38·0 (28·8–49·2)	40·9 (31·5–52·5)	43·1 (33·5–54·8)	43·2 (34·1–55·4)	0·4 (0·2–0·6)	0·3 (0·0–0·6)	0·0 (-0·4–0·5)
Diet low in fruits	39·7 (34·2–43·0)	36·0 (31·2–39·2)	35·6 (30·9–38·7)	37·3 (31·5–41·2)	-0·2 (-0·6–0·1)	0·2 (-0·3–0·6)	0·4 (-0·4–1·1)
Diet low in vegetables	29·4 (17·2–37·5)	31·6 (18·5–39·6)	36·2 (20·3–43·8)	35·3 (20·3–43·3)	0·6 (0·2–1·0)	0·5 (0·1–1·0)	-0·2 (-1·1–0·5)
Diet low in legumes	38·7 (0·0–48·4)	38·7 (0·0–48·2)	43·3 (0·0–53·5)	43·9 (0·0–55·1)	0·4 (0·0–0·7)	0·6 (0·0–1·0)	0·1 (-0·6–0·8)
Diet low in whole grains	46·1 (38·0–52·5)	49·0 (40·9–55·7)	50·6 (41·9–58·1)	51·2 (42·4–58·8)	0·3 (0·1–0·6)	0·2 (-0·1–0·6)	0·1 (-0·4–0·7)
Diet low in nuts and seeds	13·6 (9·7–18·4)	7·9 (5·2–11·1)	6·1 (4·0–9·0)	6·4 (3·8–9·5)	-2·5 (-4·3–-1·0)	-1·1 (-3·6–1·1)	0·4 (-3·2–3·5)
Diet low in milk	37·3 (33·5–41·9)	34·3 (30·0–38·7)	33·4 (29·0–38·1)	32·9 (28·4–38·0)	-0·4 (-0·9–0·0)	-0·2 (-0·8–0·4)	-0·1 (-1·0–0·7)
Diet high in red meat	49·5 (0·0–68·2)	49·9 (0·0–68·8)	49·6 (0·0–68·7)	50·0 (0·0–69·1)	0·0 (-0·5–0·5)	0·0 (-0·6–0·7)	0·1 (-1·1–1·0)
Diet high in processed meat	45·4 (35·9–51·1)	56·6 (45·5–63·4)	63·5 (51·4–71·0)	62·0 (50·9–71·0)	1·0 (0·6–1·4)	0·4 (-0·1–1·0)	-0·2 (-1·0–0·6)
Diet high in sugar-sweetened beverages	35·8 (27·0–43·7)	50·1 (39·1–58·9)	54·3 (43·1–63·5)	54·3 (43·3–64·2)	1·3 (0·5–2·3)	0·4 (-0·5–1·4)	0·0 (-1·3–1·1)
Diet low in fibre	40·6 (21·4–47·8)	30·8 (16·8–37·6)	27·9 (15·2–34·4)	24·2 (13·2–30·5)	-1·7 (-2·5–-0·9)	-1·1 (-2·3–0·0)	-1·3 (-2·9–0·4)
Diet low in calcium	5·9 (4·8–7·2)	4·9 (4·0–6·0)	5·3 (4·3–6·6)	5·2 (4·2–6·4)	-0·4 (-0·6–-0·2)	0·3 (0·0–0·6)	-0·2 (-0·6–0·2)
Diet low in seafood omega-3 fatty acids	37·7 (27·7–48·2)	44·0 (33·8–54·7)	48·5 (37·8–59·2)	42·9 (32·6–53·7)	0·4 (0·0–0·9)	-0·1 (-0·6–0·5)	-1·1 (-1·9–-0·4)
Diet low in omega-6 polyunsaturated fatty acids	37·0 (23·9–47·0)	31·9 (21·4–41·1)	24·5 (16·8–31·6)	22·6 (15·7–29·3)	-1·6 (-2·3–-0·8)	-1·6 (-2·7–-0·8)	-0·7 (-1·8–0·2)
Diet high in trans fatty acids	71·5 (64·3–78·4)	71·8 (64·2–79·1)	63·3 (55·7–71·4)	0·0 (0·0–0·0)	-65·8 (-66·1–-65·4)	-97·1 (-97·6–-96·6)	-184·2 (-185·3–-183·1)
Diet high in sodium	23·4 (0·7–68·7)	28·3 (1·8–75·0)	31·3 (3·4–76·3)	31·6 (3·2–78·0)	1·0 (0·2–6·2)	0·5 (-0·1–4·1)	0·1 (-2·6–1·8)
Intimate partner violence	31·2 (20·6–40·3)	32·5 (21·3–40·5)	28·0 (17·6–34·8)	26·4 (16·5–35·4)	-0·5 (-1·9–0·8)	-1·0 (-2·7–0·6)	-0·5 (-2·8–1·3)
Childhood sexual abuse and bullying	6·9 (4·5–10·8)	7·7 (5·2–12·0)	8·8 (5·9–13·6)	7·4 (5·1–10·8)	0·2 (-0·4–0·8)	-0·2 (-1·0–0·7)	-1·6 (-3·0–-0·2)
Childhood sexual abuse	7·5 (6·3–8·7)	8·5 (7·3–10·1)	9·9 (8·5–11·6)	9·2 (7·6–11·4)	0·7 (-0·1–1·4)	0·4 (-0·7–1·5)	-0·6 (-2·2–0·9)
Bullying victimization	5·6 (2·3–11·5)	6·1 (2·6–12·4)	6·9 (2·9–13·5)	5·1 (2·1–10·0)	-0·3 (-1·0–0·5)	-0·8 (-1·8–0·3)	-2·7 (-4·6–-0·8)
Unsafe sex	--	--	--	--	--	--	--
Low physical activity	20·1 (15·0–26·1)	19·2 (14·5–24·1)	20·3 (16·0–25·2)	23·4 (18·1–29·7)	0·5 (-0·4–1·4)	0·9 (-0·3–2·1)	1·3 (-0·6–3·1)
**Metabolic risks**	**26·3 (23·1–29·3)**	**31·8 (27·8–34·7)**	**36·0 (31·8–38·7)**	**39·6 (34·6–42·4)**	**1·3 (1·0–1·6)**	**1·1 (0·8–1·4)**	**0·9 (0·5–1·3)**
High fasting plasma glucose	13·7 (10·5–16·6)	17·9 (13·6–21·5)	22·7 (17·3–27·3)	25·8 (19·6–30·8)	2·0 (1·4–2·7)	1·7 (1·0–2·6)	1·2 (0·0–2·3)
High LDL cholesterol	63·7 (44·9–86·5)	54·0 (37·1–74·1)	48·3 (32·9–67·0)	46·8 (31·6–65·3)	-1·0 (-1·2–-0·8)	-0·7 (-0·9–-0·5)	-0·3 (-0·5–-0·1)
High systolic blood pressure	29·6 (20·0–41·3)	25·5 (18·1–35·0)	19·4 (12·7–27·6)	26·4 (16·5–37·5)	-0·4 (-1·3–0·5)	0·2 (-1·1–1·3)	2·8 (0·5–5·0)
High body-mass index	30·2 (25·5–34·4)	36·6 (31·2–40·6)	41·4 (35·4–44·8)	45·1 (38·6–48·5)	1·3 (1·0–1·6)	1·0 (0·7–1·3)	0·8 (0·4–1·2)
Low bone mineral density	21·8 (15·9–29·0)	19·7 (14·1–26·7)	20·6 (14·8–27·2)	22·7 (16·9–29·6)	0·1 (-0·3–0·6)	0·7 (0·0–1·4)	0·9 (-0·5–2·3)
Kidney dysfunction	3·0 (2·4–3·9)	3·1 (2·5–4·0)	3·0 (2·5–4·0)	3·2 (2·6–4·2)	0·2 (0·1–0·3)	0·2 (0·0–0·3)	0·4 (0·1–0·7)

Iowa

Risk Names	SEV 1990	SEV 2000	SEV 2010	SEV 2021	Annualised rate of change 1990 to 2021	Annualised rate of change 2000 to 2021	Annualised rate of change 2010 to 2021
**All risk factors**	**27·2 (24·3–30·3)**	**27·2 (24·2–30·3)**	**27·5 (24·5–30·8)**	**28·3 (24·6–31·7)**	**0·1 (-0·2–0·4)**	**0·2 (-0·3–0·6)**	**0·2 (-0·4–0·9)**
**Environmental/occupational risks**	**17·9 (11·7–28·7)**	**16·8 (11·4–27·4)**	**15·1 (10·1–24·8)**	**13·7 (8·9–22·3)**	**-0·9 (-1·6–-0·2)**	**-1·0 (-1·4–-0·5)**	**-0·9 (-1·4–-0·4)**
Unsafe water, sanitation, and handwashing	1·8 (0·9–3·0)	1·7 (0·8–2·6)	1·3 (0·5–2·2)	1·1 (0·4–1·8)	-1·6 (-4·0–0·3)	-2·1 (-5·0–0·8)	-1·9 (-6·1–2·0)
Unsafe water source	2·5 (1·0–5·2)	2·4 (0·9–5·0)	1·0 (0·4–2·1)	0·8 (0·3–1·6)	-3·8 (-6·7–-0·8)	-5·2 (-9·0–-1·2)	-2·1 (-7·8–3·2)
Unsafe sanitation	5·7 (3·1–9·6)	4·6 (2·4–7·9)	3·6 (1·9–6·2)	2·7 (1·3–4·6)	-2·4 (-4·6–-0·3)	-2·6 (-5·2–0·0)	-2·8 (-6·3–0·5)
No access to handwashing facility	1·5 (0·5–2·9)	1·6 (0·5–2·9)	1·6 (0·5–3·1)	1·4 (0·5–2·6)	-0·3 (-3·2–2·5)	-0·6 (-4·4–3·4)	-1·4 (-7·1–3·9)
Air pollution	19·2 (6·1–30·9)	16·9 (7·2–27·5)	10·3 (4·5–19·1)	7·5 (2·5–15·8)	-3·1 (-5·5–-1·2)	-3·9 (-6·5–-2·4)	-2·9 (-5·8–-1·5)
Particulate matter pollution	11·6 (3·8–21·0)	10·0 (6·9–13·9)	7·1 (4·1–10·7)	4·7 (2·0–7·9)	-2·9 (-5·8–0·1)	-3·6 (-6·1–-2·4)	-3·7 (-7·4–-2·0)
Ambient particulate matter pollution	17·9 (5·7–31·8)	15·4 (10·9–20·8)	10·9 (6·5–15·9)	7·2 (3·1–11·9)	-2·9 (-5·8–0·1)	-3·6 (-6·1–-2·4)	-3·7 (-7·4–-2·0)
Household air pollution from solid fuels	0·0 (0·0–0·0)	0·0 (0·0–0·0)	0·0 (0·0–0·0)	0·0 (0·0–0·0)	-3·0 (-33·7–-7·3)	-2·9 (-31·7–-7·0)	-1·8 (-26·2–-4·9)
Ambient ozone pollution	9·5 (5·4–14·1)	18·0 (12·3–24·7)	15·3 (9·7–21·1)	12·8 (7·7–18·1)	1·0 (0·4–1·6)	-1·6 (-2·6–-0·8)	-1·6 (-3·1–0·0)
Ambient nitrogen dioxide pollution	51·5 (0·0–100·0)	45·1 (0·0–100·0)	22·0 (0·0–68·1)	17·9 (0·0–61·5)	-3·4 (-11·3–0·0)	-4·4 (-14·8–0·0)	-1·9 (-6·4–0·0)
Non-optimal temperature	35·0 (26·8–46·5)	33·1 (25·1–44·5)	34·9 (26·4–46·3)	35·8 (27·0–47·4)	0·1 (-0·2–0·3)	0·4 (0·1–0·8)	0·2 (-0·2–0·6)
High temperature	36·0 (24·1–48·5)	31·8 (20·3–43·4)	35·9 (23·2–48·1)	38·7 (25·7–51·8)	0·2 (0·0–0·5)	0·9 (0·5–1·5)	0·7 (0·2–1·4)
Low temperature	29·6 (26·9–32·6)	28·5 (26·2–30·9)	29·3 (27·4–31·6)	30·0 (27·7–32·6)	0·0 (-0·1–0·2)	0·2 (0·1–0·4)	0·2 (0·0–0·4)
Other environmental risks	24·9 (7·9–44·4)	23·6 (7·9–44·1)	21·6 (7·9–42·2)	19·5 (7·8–40·0)	-0·8 (-1·4–0·0)	-0·9 (-1·8–0·0)	-0·9 (-1·9–0·0)
Residential radon	29·2 (0·0–93·1)	29·2 (0·0–93·1)	29·2 (0·0–93·1)	29·2 (0·0–93·1)	0·0 (0·0–0·0)	0·0 (0·0–0·0)	0·0 (0·0–0·0)
Lead exposure	22·9 (0·0–28·3)	21·1 (0·0–26·4)	18·1 (0·0–22·8)	15·2 (0·0–19·3)	-1·3 (-1·7–0·0)	-1·6 (-2·1–0·0)	-1·6 (-2·4–0·0)
Occupational risks	3·2 (2·6–4·2)	3·3 (2·8–4·4)	3·2 (2·6–4·2)	3·1 (2·5–4·1)	0·0 (-0·3–0·2)	-0·2 (-0·6–0·1)	-0·1 (-0·5–0·3)
Occupational carcinogens	1·0 (0·5–2·0)	1·1 (0·5–2·1)	1·1 (0·5–2·1)	1·1 (0·5–2·1)	0·3 (-0·1–0·5)	0·1 (-0·4–0·4)	0·1 (-0·5–0·6)
Occupational exposure to asbestos	3·4 (3·0–3·7)	3·4 (3·1–3·8)	3·7 (3·2–4·0)	3·5 (2·7–4·4)	0·1 (-0·7–0·9)	0·1 (-1·0–1·2)	-0·4 (-2·5–1·8)
Occupational exposure to arsenic	0·6 (0·0–1·3)	0·6 (0·0–1·5)	0·6 (0·0–1·3)	0·5 (0·0–1·3)	-0·1 (-0·5–0·4)	-0·4 (-0·9–0·3)	-0·1 (-1·0–0·7)
Occupational exposure to benzene	1·2 (0·1–3·4)	1·3 (0·1–3·8)	1·3 (0·1–3·8)	1·3 (0·1–3·9)	0·5 (0·1–0·7)	0·2 (-0·3–0·5)	0·2 (-0·4–0·8)
Occupational exposure to beryllium	0·0 (0·0–0·0)	0·0 (0·0–0·0)	0·0 (0·0–0·0)	0·0 (0·0–0·0)	0·3 (0·0–0·5)	0·1 (-0·2–0·4)	0·3 (-0·1–0·7)
Occupational exposure to cadmium	0·1 (0·1–0·1)	0·1 (0·1–0·1)	0·1 (0·1–0·1)	0·1 (0·1–0·1)	0·0 (-0·4–0·4)	-0·3 (-0·8–0·2)	-0·1 (-0·8–0·7)
Occupational exposure to chromium	0·2 (0·2–0·2)	0·2 (0·2–0·2)	0·2 (0·2–0·2)	0·2 (0·2–0·2)	0·1 (-0·3–0·4)	-0·2 (-0·6–0·3)	0·0 (-0·6–0·6)
Occupational exposure to diesel engine exhaust	0·7 (0·7–0·8)	0·8 (0·7–0·8)	0·8 (0·7–0·8)	0·8 (0·8–0·9)	0·4 (0·1–0·8)	0·4 (0·0–0·8)	0·6 (0·0–1·3)
Occupational exposure to formaldehyde	0·3 (0·3–0·4)	0·4 (0·3–0·4)	0·3 (0·3–0·4)	0·3 (0·3–0·4)	-0·1 (-0·4–0·3)	-0·4 (-0·9–0·1)	-0·2 (-0·8–0·6)
Occupational exposure to nickel	0·5 (0·0–1·8)	0·5 (0·0–1·8)	0·4 (0·0–1·7)	0·4 (0·0–1·7)	-0·2 (-0·7–0·3)	-0·4 (-1·1–0·3)	-0·2 (-1·1–0·9)
Occupational exposure to polycyclic aromatic hydrocarbons	0·3 (0·3–0·4)	0·4 (0·3–0·4)	0·3 (0·3–0·4)	0·3 (0·3–0·4)	0·1 (-0·2–0·4)	-0·2 (-0·6–0·2)	0·0 (-0·6–0·6)
Occupational exposure to silica	3·9 (0·6–12·8)	4·0 (0·6–12·8)	3·7 (0·6–12·3)	3·7 (0·6–12·1)	-0·2 (-0·6–0·3)	-0·3 (-1·0–0·3)	0·0 (-0·9–0·9)
Occupational exposure to sulphuric acid	0·8 (0·2–2·9)	0·9 (0·2–3·0)	0·8 (0·2–2·7)	0·8 (0·2–2·8)	0·0 (-0·4–0·4)	-0·3 (-0·8–0·2)	0·0 (-0·8–0·7)
Occupational exposure to trichloroethylene	0·1 (0·1–0·1)	0·1 (0·1–0·1)	0·1 (0·1–0·1)	0·1 (0·1–0·1)	0·2 (-0·1–0·5)	0·0 (-0·4–0·4)	0·2 (-0·3–0·7)
Occupational asthmagens	18·4 (16·1–21·1)	19·5 (17·2–22·2)	18·5 (16·5–21·1)	18·5 (16·4–21·2)	0·0 (-0·3–0·3)	-0·3 (-0·7–0·2)	0·0 (-0·6–0·6)
Occupational particulate matter, gases, and fumes	7·6 (5·9–10·0)	7·5 (5·8–9·9)	7·2 (5·6–9·5)	6·7 (5·2–8·8)	-0·4 (-0·7–-0·2)	-0·6 (-0·9–-0·3)	-0·7 (-1·0–-0·3)
Occupational noise	7·6 (7·1–8·2)	7·7 (7·2–8·3)	7·6 (7·2–8·2)	7·4 (7·0–8·0)	-0·1 (-0·2–0·1)	-0·2 (-0·3–0·0)	-0·3 (-0·4–-0·1)
Occupational injuries	--	--	--	--	--	--	--
Occupational ergonomic factors	9·2 (7·7–11·0)	9·3 (7·9–11·1)	8·5 (7·2–10·2)	8·5 (7·1–10·3)	-0·2 (-0·7–0·2)	-0·4 (-1·0–0·1)	0·0 (-0·8–0·8)
**Behavioural risks**	**30·1 (26·8–33·5)**	**29·0 (25·7–32·9)**	**27·0 (23·7–30·9)**	**25·5 (22·2–29·4)**	**-0·5 (-0·9–-0·2)**	**-0·6 (-1·1–-0·1)**	**-0·5 (-1·2–-0·2)**
Child and maternal malnutrition	10·0 (6·9–14·1)	8·6 (5·9–11·8)	8·8 (6·1–12·1)	9·0 (6·2–12·7)	-0·3 (-1·2–0·5)	0·2 (-1·1–1·4)	0·2 (-1·5–2·0)
Suboptimal breastfeeding	68·8 (66·0–71·5)	66·7 (64·3–69·1)	62·8 (60·3–66·0)	62·1 (59·3–65·4)	-0·3 (-0·5–-0·2)	-0·3 (-0·5–-0·2)	-0·1 (-0·4–0·2)
Non-exclusive breastfeeding	64·1 (56·0–71·2)	59·6 (53·5–66·7)	48·6 (42·6–55·0)	46·2 (38·7–53·7)	-1·1 (-1·6–-0·6)	-1·2 (-1·9–-0·6)	-0·5 (-1·5–0·6)
Discontinued breastfeeding	88·8 (86·4–91·0)	86·3 (83·5–89·0)	84·3 (81·0–88·1)	84·5 (81·2–88·0)	-0·2 (-0·3–0·0)	-0·1 (-0·3–0·1)	0·0 (-0·3–0·3)
Child growth failure	1·2 (0·4–2·7)	1·2 (0·4–2·6)	1·0 (0·3–2·3)	0·9 (0·3–2·0)	-0·8 (-1·2–-0·5)	-1·0 (-1·6–-0·6)	-0·7 (-1·4–0·2)
Child underweight	2·5 (1·5–3·5)	2·5 (1·5–3·3)	2·1 (1·3–2·9)	2·0 (1·2–2·7)	-0·8 (-1·4–-0·3)	-1·1 (-1·8–-0·5)	-0·6 (-1·6–0·4)
Child wasting	0·8 (0·5–1·3)	0·8 (0·5–1·3)	0·8 (0·5–1·2)	0·8 (0·5–1·2)	-0·2 (-0·5–0·1)	-0·1 (-0·6–0·3)	-0·3 (-1·0–0·3)
Child stunting	3·5 (2·7–4·3)	3·4 (2·7–4·1)	2·9 (2·3–3·6)	2·6 (2·0–3·3)	-0·9 (-1·5–-0·3)	-1·2 (-2·0–-0·4)	-0·9 (-2·1–0·3)
Low birth weight and short gestation	17·6 (15·4–20·1)	18·5 (16·0–20·9)	20·2 (17·6–23·1)	20·4 (17·7–23·1)	0·5 (0·2–0·7)	0·5 (0·1–0·8)	0·1 (-0·7–0·8)
Short gestation	31·1 (27·3–35·4)	32·7 (28·3–36·7)	34·7 (29·9–39·5)	35·0 (30·4–39·8)	0·4 (0·1–0·7)	0·3 (-0·1–0·7)	0·1 (-0·8–1·0)
Low birth weight	13·8 (12·4–15·2)	14·3 (12·9–15·7)	15·7 (14·2–17·4)	15·8 (14·3–17·5)	0·4 (0·2–0·7)	0·5 (0·1–0·8)	0·1 (-0·6–0·8)

Iron deficiency	3·5 (2·5–4·9)	3·0 (2·1–4·1)	3·1 (2·2–4·2)	3·2 (2·2–4·4)	-0·4 (-1·1–0·5)	0·2 (-0·9–1·3)	0·1 (-1·6–1·8)
Vitamin A deficiency	0·9 (0·0–1·9)	1·3 (0·0–2·7)	0·8 (0·0–1·7)	0·5 (0·0–1·0)	-2·1 (-4·3–0·0)	-4·6 (-7·3–0·0)	-4·8 (-8·7–0·0)
Zinc deficiency	2·0 (0·0–8·4)	1·1 (0·0–4·6)	1·0 (0·0–4·4)	1·0 (0·0–4·2)	-2·2 (-47·7–0·0)	-0·6 (-2·7–2·5)	-0·1 (-4·5–2·5)
Tobacco	38·4 (36·6–39·9)	36·2 (34·8–37·4)	29·6 (28·6–30·6)	26·2 (24·6–27·8)	-1·2 (-1·4–-1·0)	-1·5 (-1·9–-1·2)	-1·1 (-1·7–-0·5)
Smoking	36·2 (33·7–38·4)	32·6 (30·5–34·5)	27·0 (25·7–28·5)	24·1 (22·0–26·4)	-1·3 (-1·6–-1·0)	-1·4 (-1·9–-1·0)	-1·1 (-1·8–-0·3)
Chewing tobacco	2·4 (1·9–3·1)	2·8 (2·2–3·5)	2·9 (2·1–3·9)	2·9 (2·0–4·0)	0·6 (-0·8–1·7)	0·2 (-1·5–1·8)	-0·1 (-2·9–2·7)
Second-hand smoke	30·5 (28·8–31·8)	29·6 (27·9–30·7)	24·0 (22·5–25·0)	21·5 (19·6–23·1)	-1·1 (-1·4–-0·9)	-1·5 (-1·9–-1·2)	-1·0 (-1·8–-0·4)
High alcohol use	28·5 (20·7–39·1)	27·1 (20·2–36·9)	27·6 (20·4–37·7)	28·1 (19·6–38·5)	0·0 (-1·1–1·0)	0·2 (-1·2–1·7)	0·1 (-1·7–1·9)
Drug use	0·6 (0·3–1·1)	0·5 (0·3–0·9)	0·9 (0·8–1·2)	3·0 (2·2–3·8)	5·3 (2·3–8·3)	8·4 (4·5–12·2)	10·6 (6·5–13·6)
Dietary risks	38·5 (29·2–49·7)	41·4 (31·5–53·0)	42·9 (33·5–55·0)	42·8 (33·6–55·4)	0·3 (0·1–0·6)	0·2 (-0·2–0·5)	0·0 (-0·5–0·5)
Diet low in fruits	39·1 (32·7–42·4)	36·0 (30·5–39·0)	33·9 (29·8–36·8)	34·7 (29·8–38·9)	-0·4 (-0·7–0·0)	-0·2 (-0·7–0·4)	0·2 (-0·6–1·1)
Diet low in vegetables	27·0 (15·6–34·4)	28·4 (16·5–36·3)	30·8 (17·4–37·8)	29·7 (16·7–38·1)	0·3 (-0·1–0·7)	0·2 (-0·4–0·8)	-0·3 (-1·3–0·5)
Diet low in legumes	38·4 (0·0–48·4)	38·7 (0·0–48·8)	42·8 (0·0–53·2)	43·1 (0·0–54·1)	0·4 (0·0–0·7)	0·5 (0·0–0·9)	0·1 (-0·7–0·7)
Diet low in whole grains	46·0 (38·4–52·2)	49·5 (41·8–55·7)	51·3 (42·6–58·9)	51·5 (43·0–59·6)	0·4 (0·1–0·6)	0·2 (-0·2–0·6)	0·0 (-0·5–0·7)
Diet low in nuts and seeds	13·2 (9·7–17·3)	7·8 (5·2–10·9)	5·5 (3·5–8·2)	5·7 (3·4–8·9)	-2·7 (-4·5–-1·1)	-1·5 (-3·9–0·8)	0·3 (-3·3–3·6)
Diet low in milk	36·9 (32·5–41·8)	34·0 (29·4–38·5)	32·2 (27·0–36·6)	31·5 (26·2–36·5)	-0·5 (-1·0–-0·1)	-0·4 (-1·0–0·3)	-0·2 (-1·1–0·6)
Diet high in red meat	56·9 (0·0–76·3)	57·9 (0·0–77·5)	58·1 (0·0–77·6)	57·7 (0·0–77·4)	0·0 (-0·4–0·4)	0·0 (-0·5–0·4)	-0·1 (-1·0–0·6)
Diet high in processed meat	47·7 (39·0–53·1)	59·1 (48·5–65·6)	65·3 (54·0–72·8)	63·4 (52·5–71·7)	0·9 (0·5–1·4)	0·3 (-0·3–0·9)	-0·3 (-1·1–0·5)
Diet high in sugar-sweetened beverages	36·3 (27·7–44·5)	50·7 (39·5–60·0)	56·5 (45·4–65·7)	56·5 (45·1–66·6)	1·4 (0·6–2·2)	0·5 (-0·4–1·4)	0·0 (-1·3–1·3)
Diet low in fibre	40·1 (22·9–47·2)	30·6 (17·9–37·4)	26·9 (15·7–33·7)	23·1 (13·8–30·0)	-1·8 (-2·8–-0·9)	-1·3 (-2·8–0·0)	-1·4 (-3·4–0·2)
Diet low in calcium	5·8 (4·7–7·1)	4·8 (3·9–6·0)	5·1 (4·1–6·2)	4·9 (3·9–6·0)	-0·5 (-0·8–-0·3)	0·1 (-0·2–0·3)	-0·3 (-0·6–0·1)
Diet low in seafood omega-3 fatty acids	50·3 (39·2–60·7)	56·9 (45·8–68·8)	59·5 (48·5–71·1)	54·3 (42·9–65·7)	0·2 (-0·1–0·6)	-0·2 (-0·6–0·1)	-0·8 (-1·4–-0·3)
Diet low in omega-6 polyunsaturated fatty acids	36·6 (23·8–47·4)	32·1 (21·3–41·3)	23·8 (16·3–31·0)	21·9 (14·7–28·7)	-1·7 (-2·5–-0·9)	-1·8 (-2·8–-0·9)	-0·8 (-1·9–0·2)
Diet high in trans fatty acids	71·3 (63·5–79·2)	71·8 (65·2–79·3)	63·4 (55·3–71·7)	0·0 (0·0–0·0)	-65·8 (-66·1–-65·4)	-97·1 (-97·6–-96·6)	-184·3 (-185·4–-183·0)
Diet high in sodium	23·2 (0·5–66·7)	28·0 (1·8–74·6)	30·9 (3·2–75·3)	31·4 (3·1–75·7)	1·0 (0·2–6·1)	0·5 (-0·1–3·7)	0·2 (-2·3–2·1)
Intimate partner violence	27·3 (16·9–34·4)	24·7 (18·1–29·3)	20·5 (14·0–25·5)	19·9 (11·9–27·7)	-1·0 (-2·3–0·5)	-1·0 (-2·7–0·8)	-0·3 (-1·6–1·1)
Childhood sexual abuse and bullying	8·4 (5·4–13·8)	9·9 (6·3–16·2)	11·1 (6·9–18·0)	9·3 (5·9–15·1)	0·3 (-0·3–0·9)	-0·3 (-1·2–0·6)	-1·6 (-3·1–0·0)
Childhood sexual abuse	7·3 (6·1–8·6)	7·4 (6·4–8·6)	8·0 (7·2–8·9)	8·0 (6·6–9·4)	0·3 (-0·6–1·1)	0·4 (-0·7–1·4)	0·0 (-1·3–1·3)
Bullying victimization	8·0 (3·5–15·9)	9·9 (4·6–19·3)	11·3 (5·1–22·0)	8·7 (3·6–17·4)	0·3 (-0·4–1·0)	-0·6 (-1·7–0·3)	-2·3 (-4·3–-0·5)
Unsafe sex	--	--	--	--	--	--	--
Low physical activity	22·0 (16·5–27·6)	21·7 (16·9–26·5)	22·3 (17·7–27·5)	24·5 (18·8–31·1)	0·3 (-0·6–1·2)	0·6 (-0·6–1·6)	0·9 (-1·0–2·4)
**Metabolic risks**	**26·4 (22·9–29·5)**	**31·3 (27·6–34·3)**	**35·0 (30·8–37·9)**	**39·6 (34·4–42·8)**	**1·3 (1·0–1·6)**	**1·1 (0·8–1·5)**	**1·1 (0·7–1·6)**
High fasting plasma glucose	13·0 (10·0–16·1)	16·8 (13·0–20·1)	21·4 (16·5–25·3)	25·3 (19·0–30·1)	2·1 (1·5–2·8)	1·9 (1·1–2·8)	1·5 (0·2–2·8)
High LDL cholesterol	63·6 (44·8–85·8)	53·7 (36·9–74·6)	48·1 (32·5–67·4)	46·6 (31·5–65·4)	-1·0 (-1·2–-0·8)	-0·7 (-0·9–-0·5)	-0·3 (-0·6–0·0)
High systolic blood pressure	30·0 (19·9–41·3)	24·9 (17·7–34·1)	18·0 (11·7–26·3)	26·2 (16·1–37·6)	-0·4 (-1·6–0·6)	0·3 (-1·2–1·6)	3·4 (0·7–6·1)
High body-mass index	30·2 (25·2–34·5)	36·2 (30·8–40·2)	40·6 (34·2–44·3)	45·3 (38·2–49·4)	1·3 (1·0–1·6)	1·1 (0·7–1·4)	1·0 (0·6–1·4)
Low bone mineral density	21·0 (15·3–27·7)	19·2 (13·7–26·0)	20·0 (14·4–26·9)	21·9 (16·0–28·7)	0·1 (-0·3–0·6)	0·6 (-0·1–1·4)	0·8 (-0·5–2·3)
Kidney dysfunction	2·8 (2·3–3·7)	2·9 (2·3–3·8)	2·8 (2·3–3·8)	3·0 (2·4–3·9)	0·2 (0·1–0·3)	0·1 (0·0–0·3)	0·4 (0·1–0·7)

Kansas

Risk Names	SEV 1990	SEV 2000	SEV 2010	SEV 2021	Annualised rate of change 1990 to 2021	Annualised rate of change 2000 to 2021	Annualised rate of change 2010 to 2021
**All risk factors**	**27·6 (24·4–30·6)**	**26·9 (24·1–29·8)**	**27·1 (24·2–30·2)**	**27·7 (24·5–31·2)**	**0·0 (-0·3–0·3)**	**0·1 (-0·2–0·5)**	**0·2 (-0·3–0·7)**
**Environmental/occupational risks**	**18·3 (12·0–29·1)**	**18·0 (12·4–28·5)**	**16·0 (10·6–25·6)**	**14·0 (9·1–22·9)**	**-0·9 (-1·6–-0·3)**	**-1·2 (-1·7–-0·7)**	**-1·2 (-1·9–-0·7)**
Unsafe water, sanitation, and handwashing	1·9 (1·0–3·1)	1·8 (0·8–2·9)	1·4 (0·5–2·5)	1·1 (0·4–2·1)	-1·7 (-4·2–0·4)	-2·3 (-5·5–0·5)	-2·2 (-6·2–1·5)
Unsafe water source	2·7 (1·0–5·8)	2·5 (0·9–4·8)	1·1 (0·4–2·2)	0·8 (0·4–1·6)	-3·9 (-6·9–-1·1)	-5·4 (-8·9–-1·6)	-2·6 (-7·6–3·0)
Unsafe sanitation	6·1 (3·4–10·3)	5·1 (2·6–8·9)	4·0 (2·1–7·2)	2·8 (1·4–4·8)	-2·5 (-4·8–-0·5)	-2·9 (-5·8–-0·3)	-3·4 (-6·8–0·0)
No access to handwashing facility	1·6 (0·5–3·3)	1·6 (0·5–3·2)	1·7 (0·5–3·4)	1·4 (0·4–3·0)	-0·4 (-3·4–3·1)	-0·7 (-5·0–3·8)	-1·6 (-7·6–4·1)
Air pollution	19·6 (5·8–31·3)	17·8 (7·4–28·0)	10·7 (4·4–20·5)	6·7 (2·0–15·2)	-3·4 (-6·3–-1·3)	-4·6 (-8·0–-2·6)	-4·2 (-8·6–-2·3)
Particulate matter pollution	11·1 (3·4–20·3)	10·1 (6·7–13·9)	6·7 (3·8–10·2)	4·1 (1·6–7·3)	-3·2 (-6·3–-0·2)	-4·3 (-7·2–-2·8)	-4·4 (-9·3–-2·1)
Ambient particulate matter pollution	17·1 (5·4–32·2)	15·6 (10·6–21·0)	10·3 (5·9–15·1)	6·3 (2·5–10·8)	-3·2 (-6·4–-0·2)	-4·3 (-7·3–-2·8)	-4·5 (-9·4–-2·1)
Household air pollution from solid fuels	0·0 (0·0–0·0)	0·0 (0·0–0·0)	0·0 (0·0–0·0)	0·0 (0·0–0·0)	-2·6 (-31·5–-5·7)	-3·7 (-30·8–-5·9)	-3·9 (-26·2–-5·5)
Ambient ozone pollution	14·8 (9·5–21·1)	25·3 (18·7–32·6)	20·4 (14·1–27·4)	15·4 (10·1–21·7)	0·1 (-0·4–0·6)	-2·4 (-3·3–-1·5)	-2·5 (-4·3–-1·1)
Ambient nitrogen dioxide pollution	55·6 (0·0–100·0)	49·2 (0·0–100·0)	25·5 (0·0–75·2)	16·5 (0·0–59·9)	-3·9 (-15·8–0·0)	-5·2 (-21·8–0·0)	-4·0 (-22·8–0·0)
Non-optimal temperature	34·5 (26·8–45·6)	35·5 (28·0–46·7)	35·7 (28·2–47·3)	32·8 (25·9–44·1)	-0·2 (-0·4–0·1)	-0·4 (-0·6–-0·1)	-0·8 (-1·2–-0·5)
High temperature	43·1 (30·6–55·7)	43·4 (30·9–56·2)	41·5 (28·9–54·1)	35·4 (23·8–47·2)	-0·6 (-1·1–-0·2)	-1·0 (-1·6–-0·4)	-1·4 (-2·1–-0·9)
Low temperature	28·3 (25·0–32·4)	29·4 (26·5–33·1)	30·1 (27·6–33·7)	28·5 (25·8–32·2)	0·0 (-0·2–0·2)	-0·1 (-0·3–0·0)	-0·5 (-0·8–-0·3)
Other environmental risks	26·1 (9·8–45·4)	24·6 (9·8–44·0)	22·5 (9·8–41·7)	20·0 (9·3–38·3)	-0·9 (-1·6–0·0)	-1·0 (-1·8–0·0)	-1·1 (-2·1–0·0)
Residential radon	26·8 (0·0–83·4)	26·8 (0·0–83·4)	26·8 (0·0–83·4)	26·8 (0·0–83·4)	0·0 (0·0–0·0)	0·0 (0·0–0·0)	0·0 (0·0–0·0)
Lead exposure	25·7 (0·0–32·3)	23·6 (0·0–29·7)	20·5 (0·0–25·7)	16·9 (0·0–21·0)	-1·4 (-1·8–0·0)	-1·6 (-2·0–0·0)	-1·8 (-2·6–0·0)
Occupational risks	3·0 (2·5–4·0)	3·1 (2·6–4·1)	3·0 (2·4–3·9)	3·0 (2·4–3·9)	-0·1 (-0·3–0·2)	-0·3 (-0·6–0·0)	-0·1 (-0·5–0·4)
Occupational carcinogens	1·0 (0·4–1·9)	1·1 (0·5–2·1)	1·0 (0·5–2·0)	1·0 (0·4–2·0)	0·2 (0·0–0·5)	0·0 (-0·4–0·3)	0·1 (-0·5–0·6)
Occupational exposure to asbestos	3·4 (3·0–3·9)	3·7 (3·2–4·0)	3·3 (2·9–3·7)	3·1 (2·4–4·0)	-0·3 (-1·2–0·5)	-0·8 (-2·0–0·3)	-0·4 (-2·8–1·7)
Occupational exposure to arsenic	0·5 (0·0–1·3)	0·6 (0·0–1·3)	0·5 (0·0–1·2)	0·5 (0·0–1·3)	-0·1 (-0·5–0·4)	-0·3 (-0·9–0·3)	-0·1 (-0·9–0·8)
Occupational exposure to benzene	1·1 (0·1–3·3)	1·3 (0·1–3·7)	1·3 (0·1–3·7)	1·3 (0·1–3·8)	0·4 (0·0–0·7)	0·1 (-0·3–0·5)	0·2 (-0·4–0·7)
Occupational exposure to beryllium	0·0 (0·0–0·0)	0·0 (0·0–0·0)	0·0 (0·0–0·0)	0·0 (0·0–0·0)	0·3 (0·0–0·5)	0·1 (-0·2–0·4)	0·3 (-0·1–0·7)
Occupational exposure to cadmium	0·1 (0·1–0·1)	0·1 (0·1–0·1)	0·1 (0·1–0·1)	0·1 (0·1–0·1)	0·0 (-0·4–0·4)	-0·2 (-0·8–0·3)	0·0 (-0·8–0·7)
Occupational exposure to chromium	0·2 (0·1–0·2)	0·2 (0·2–0·2)	0·2 (0·1–0·2)	0·2 (0·1–0·2)	0·1 (-0·2–0·4)	-0·2 (-0·6–0·3)	0·1 (-0·6–0·7)
Occupational exposure to diesel engine exhaust	0·7 (0·7–0·8)	0·8 (0·7–0·8)	0·8 (0·7–0·8)	0·8 (0·7–0·9)	0·3 (0·0–0·7)	0·3 (-0·2–0·8)	0·6 (-0·1–1·2)
Occupational exposure to formaldehyde	0·3 (0·3–0·3)	0·3 (0·3–0·4)	0·3 (0·3–0·3)	0·3 (0·3–0·3)	-0·1 (-0·4–0·3)	-0·3 (-0·8–0·2)	-0·1 (-0·9–0·6)
Occupational exposure to nickel	0·4 (0·0–1·7)	0·4 (0·0–1·7)	0·4 (0·0–1·6)	0·4 (0·0–1·6)	-0·2 (-0·7–0·4)	-0·3 (-1·1–0·4)	-0·1 (-1·1–0·9)
Occupational exposure to polycyclic aromatic hydrocarbons	0·3 (0·3–0·3)	0·3 (0·3–0·4)	0·3 (0·3–0·3)	0·3 (0·3–0·3)	0·1 (-0·2–0·4)	-0·1 (-0·5–0·3)	0·1 (-0·6–0·7)
Occupational exposure to silica	3·8 (0·6–12·4)	3·8 (0·6–12·1)	3·6 (0·6–11·6)	3·6 (0·6–11·5)	-0·2 (-0·7–0·2)	-0·3 (-1·0–0·3)	0·0 (-1·0–0·9)
Occupational exposure to sulphuric acid	0·8 (0·2–2·6)	0·8 (0·2–2·6)	0·8 (0·2–2·5)	0·8 (0·2–2·5)	0·0 (-0·4–0·4)	-0·2 (-0·8–0·3)	0·0 (-0·8–0·8)
Occupational exposure to trichloroethylene	0·1 (0·1–0·1)	0·1 (0·1–0·1)	0·1 (0·1–0·1)	0·1 (0·1–0·1)	0·2 (-0·1–0·5)	0·0 (-0·4–0·4)	0·3 (-0·3–0·8)
Occupational asthmagens	18·0 (15·6–20·3)	18·8 (16·5–21·2)	17·6 (15·6–20·0)	17·7 (15·6–19·8)	-0·1 (-0·4–0·3)	-0·3 (-0·7–0·2)	0·1 (-0·6–0·7)
Occupational particulate matter, gases, and fumes	7·0 (5·3–9·1)	6·9 (5·3–9·0)	6·6 (5·1–8·6)	6·2 (4·8–7·9)	-0·4 (-0·7–-0·1)	-0·5 (-0·9–-0·2)	-0·6 (-1·0–-0·3)
Occupational noise	7·2 (6·6–7·9)	7·3 (6·7–7·9)	7·2 (6·7–7·8)	7·0 (6·6–7·6)	-0·1 (-0·2–0·1)	-0·2 (-0·3–0·0)	-0·3 (-0·4–0·0)
Occupational injuries	--	--	--	--	--	--	--
Occupational ergonomic factors	8·7 (7·2–10·4)	8·8 (7·3–10·5)	8·0 (6·8–9·7)	8·1 (6·6–10·0)	-0·2 (-0·7–0·1)	-0·4 (-0·9–0·1)	0·0 (-0·7–0·7)
**Behavioural risks**	**30·3 (27·1–34·1)**	**28·0 (25·2–31·7)**	**26·3 (23·0–29·9)**	**25·2 (21·7–28·8)**	**-0·6 (-0·9–-0·3)**	**-0·5 (-0·9–-0·1)**	**-0·4 (-1·0–0·3)**
Child and maternal malnutrition	13·3 (8·3–19·7)	12·5 (8·1–18·1)	12·3 (7·9–17·6)	12·2 (7·7–17·7)	-0·3 (-0·7–0·1)	-0·1 (-0·7–0·4)	-0·1 (-1·0–0·8)
Suboptimal breastfeeding	68·8 (66·1–71·6)	66·7 (64·0–69·2)	62·9 (60·2–65·8)	62·2 (59·4–65·2)	-0·3 (-0·5–-0·2)	-0·3 (-0·5–-0·1)	-0·1 (-0·4–0·2)
Non-exclusive breastfeeding	64·1 (56·4–72·0)	59·7 (52·9–66·1)	48·8 (42·0–55·4)	46·4 (39·0–53·6)	-1·0 (-1·6–-0·6)	-1·2 (-1·9–-0·5)	-0·5 (-1·4–0·5)
Discontinued breastfeeding	88·7 (86·5–91·0)	86·3 (83·6–88·9)	84·4 (81·5–87·3)	84·6 (81·4–88·2)	-0·2 (-0·3–0·0)	-0·1 (-0·3–0·1)	0·0 (-0·3–0·3)
Child growth failure	1·2 (0·4–2·7)	1·2 (0·4–2·7)	1·1 (0·3–2·4)	1·0 (0·3–2·1)	-0·8 (-1·2–-0·4)	-1·0 (-1·5–-0·5)	-0·8 (-1·6–-0·1)
Child underweight	2·6 (1·6–3·6)	2·5 (1·7–3·4)	2·2 (1·4–3·1)	2·0 (1·2–2·8)	-0·8 (-1·3–-0·3)	-1·0 (-1·7–-0·4)	-0·7 (-1·8–0·3)
Child wasting	0·9 (0·5–1·3)	0·8 (0·5–1·3)	0·8 (0·5–1·3)	0·8 (0·5–1·2)	-0·2 (-0·5–0·1)	-0·1 (-0·6–0·4)	-0·4 (-1·1–0·3)
Child stunting	3·6 (2·8–4·6)	3·5 (2·7–4·3)	3·1 (2·4–3·9)	2·7 (2·1–3·5)	-0·9 (-1·4–-0·3)	-1·1 (-2·0–-0·4)	-1·0 (-2·3–0·2)
Low birth weight and short gestation	19·5 (17·2–22·1)	20·7 (18·3–23·5)	21·5 (18·9–24·3)	21·6 (18·9–24·6)	0·3 (0·1–0·6)	0·2 (-0·2–0·6)	0·0 (-0·7–0·8)
Short gestation	34·8 (30·7–39·7)	37·2 (32·9–41·9)	38·6 (33·7–43·3)	38·1 (33·4–43·1)	0·3 (0·0–0·6)	0·1 (-0·3–0·6)	-0·1 (-1·0–0·8)
Low birth weight	15·5 (14·2–17·1)	16·2 (14·8–17·9)	16·7 (15·2–18·1)	17·0 (15·5–18·6)	0·3 (0·0–0·5)	0·2 (-0·1–0·6)	0·2 (-0·5–0·9)

Iron deficiency	4·6 (2·9–6·7)	4·3 (2·9–6·1)	4·3 (2·8–6·0)	4·2 (2·8–6·0)	-0·2 (-0·6–0·1)	-0·1 (-0·6–0·5)	-0·1 (-0·9–0·7)
Vitamin A deficiency	1·0 (0·0–2·1)	1·4 (0·0–3·1)	1·0 (0·0–2·0)	0·5 (0·0–1·1)	-2·1 (-4·5–0·2)	-4·8 (-7·7–0·0)	-5·6 (-9·8–0·0)
Zinc deficiency	1·8 (0·0–8·0)	1·1 (0·0–4·6)	1·0 (0·0–4·4)	1·0 (0·0–4·3)	-2·0 (-40·6–0·1)	-0·5 (-3·2–3·4)	-0·1 (-2·5–6·7)
Tobacco	39·3 (37·6–40·8)	33·8 (32·5–35·0)	28·5 (27·5–29·5)	26·1 (24·3–27·7)	-1·3 (-1·5–-1·1)	-1·2 (-1·5–-0·9)	-0·8 (-1·4–-0·3)
Smoking	37·0 (34·4–39·5)	31·1 (28·9–33·0)	26·2 (24·8–27·8)	23·9 (21·9–26·3)	-1·4 (-1·8–-1·1)	-1·2 (-1·7–-0·8)	-0·8 (-1·6–-0·1)
Chewing tobacco	2·7 (2·2–3·5)	3·2 (2·4–3·9)	3·3 (2·5–4·3)	3·2 (2·3–4·5)	0·5 (-0·7–1·8)	0·1 (-1·6–1·8)	-0·2 (-2·8–2·3)
Second-hand smoke	31·4 (29·8–32·7)	27·5 (25·7–28·5)	23·1 (21·8–24·0)	21·3 (19·4–23·0)	-1·3 (-1·5–-1·0)	-1·2 (-1·5–-0·9)	-0·8 (-1·4–-0·2)
High alcohol use	27·0 (19·5–36·7)	25·1 (18·6–34·5)	25·6 (18·3–34·8)	26·8 (18·9–37·2)	0·0 (-1·0–1·0)	0·3 (-1·0–1·6)	0·4 (-1·4–2·0)
Drug use	0·8 (0·5–1·4)	1·0 (0·8–1·2)	1·8 (1·4–2·2)	4·0 (2·8–5·1)	5·1 (2·6–6·9)	6·5 (4·5–7·7)	7·1 (5·4–8·3)
Dietary risks	36·9 (27·5–48·0)	40·1 (30·7–51·7)	43·0 (33·5–54·8)	43·1 (33·8–55·1)	0·5 (0·3–0·8)	0·3 (0·0–0·7)	0·0 (-0·4–0·5)
Diet low in fruits	38·3 (31·9–42·0)	35·9 (30·5–38·9)	36·4 (31·2–39·3)	37·3 (31·7–41·9)	-0·1 (-0·5–0·3)	0·2 (-0·3–0·7)	0·2 (-0·5–1·0)
Diet low in vegetables	22·6 (13·7–30·5)	25·1 (14·8–33·0)	30·5 (17·1–38·1)	30·0 (17·6–38·3)	0·9 (0·5–1·4)	0·8 (0·3–1·4)	-0·2 (-1·1–0·7)
Diet low in legumes	38·1 (0·0–48·6)	38·4 (0·0–48·4)	42·4 (0·0–53·1)	42·9 (0·0–54·5)	0·4 (0·0–0·7)	0·5 (0·0–1·0)	0·1 (-0·6–0·8)
Diet low in whole grains	45·2 (37·3–52·0)	48·5 (39·6–55·9)	50·6 (41·8–58·3)	51·1 (42·8–59·0)	0·4 (0·1–0·7)	0·3 (-0·1–0·6)	0·1 (-0·4–0·7)
Diet low in nuts and seeds	12·6 (9·0–16·5)	7·5 (4·7–10·8)	5·3 (3·3–7·9)	5·5 (3·1–8·6)	-2·6 (-4·5–-1·0)	-1·5 (-4·0–1·0)	0·3 (-3·7–4·0)
Diet low in milk	35·9 (31·6–40·7)	33·3 (29·2–38·0)	31·7 (27·2–36·1)	31·2 (26·8–36·0)	-0·5 (-0·9–0·0)	-0·3 (-1·0–0·3)	-0·1 (-1·0–0·7)
Diet high in red meat	54·1 (0·0–73·2)	54·8 (0·0–74·1)	55·0 (0·0–74·4)	55·0 (0·0–74·5)	0·1 (-0·4–0·5)	0·0 (-0·6–0·6)	0·0 (-1·1–0·8)
Diet high in processed meat	46·5 (36·9–52·1)	57·3 (47·1–63·6)	64·5 (53·1–72·1)	63·0 (51·1–71·7)	1·0 (0·5–1·4)	0·4 (-0·1–1·0)	-0·2 (-1·0–0·6)
Diet high in sugar-sweetened beverages	36·5 (27·9–44·6)	50·5 (40·8–59·2)	56·7 (45·5–65·5)	56·9 (45·5–66·5)	1·4 (0·7–2·2)	0·6 (-0·4–1·5)	0·0 (-1·3–1·3)
Diet low in fibre	39·1 (21·7–46·7)	29·9 (16·5–36·9)	26·3 (15·0–32·6)	22·8 (13·2–29·4)	-1·7 (-2·8–-0·9)	-1·3 (-2·7–-0·1)	-1·3 (-3·2–0·5)
Diet low in calcium	5·6 (4·5–6·9)	4·7 (3·8–5·9)	5·0 (4·0–6·2)	4·9 (3·9–6·1)	-0·4 (-0·7–-0·2)	0·1 (-0·1–0·4)	-0·2 (-0·6–0·2)
Diet low in seafood omega-3 fatty acids	49·0 (38·3–60·1)	56·3 (45·1–68·0)	59·1 (48·1–70·9)	54·1 (42·5–66·1)	0·3 (0·0–0·7)	-0·2 (-0·6–0·2)	-0·8 (-1·4–-0·2)
Diet low in omega-6 polyunsaturated fatty acids	35·4 (22·8–45·1)	31·4 (21·0–39·8)	23·4 (16·4–30·4)	21·6 (15·0–28·4)	-1·6 (-2·3–-0·8)	-1·8 (-2·7–-1·0)	-0·7 (-1·7–0·2)
Diet high in trans fatty acids	71·3 (64·0–78·6)	71·9 (63·6–79·0)	63·2 (55·5–70·5)	0·0 (0·0–0·0)	-65·8 (-66·1–-65·4)	-97·1 (-97·6–-96·5)	-184·2 (-185·2–-183·0)
Diet high in sodium	23·1 (0·6–65·9)	27·9 (1·9–73·3)	31·1 (3·4–74·9)	31·6 (3·0–77·2)	1·0 (0·3–5·9)	0·6 (-0·1–3·7)	0·1 (-2·6–1·9)
Intimate partner violence	22·9 (14·1–29·4)	23·1 (14·0–29·6)	21·2 (13·4–26·5)	20·3 (11·4–28·1)	-0·4 (-1·5–0·7)	-0·6 (-2·1–0·8)	-0·4 (-2·3–1·3)
Childhood sexual abuse and bullying	9·1 (6·0–14·2)	11·0 (7·3–17·2)	12·6 (8·3–19·8)	10·1 (6·8–15·4)	0·4 (-0·3–0·9)	-0·4 (-1·2–0·4)	-1·9 (-3·3–-0·5)
Childhood sexual abuse	8·8 (7·4–10·4)	9·9 (8·6–11·5)	11·5 (10·0–13·3)	10·7 (8·8–13·2)	0·6 (-0·2–1·5)	0·4 (-0·7–1·4)	-0·6 (-2·2–0·8)
Bullying victimization	7·9 (3·5–15·9)	10·0 (4·6–19·5)	11·3 (5·1–22·0)	8·2 (3·6–15·4)	0·1 (-0·7–0·8)	-0·9 (-2·0–0·1)	-2·9 (-4·8–-1·0)
Unsafe sex	--	--	--	--	--	--	--
Low physical activity	21·4 (16·2–27·4)	21·1 (16·2–26·3)	22·0 (17·6–27·2)	24·1 (18·7–30·7)	0·4 (-0·6–1·3)	0·6 (-0·5–1·8)	0·9 (-0·9–2·4)
**Metabolic risks**	**26·5 (22·9–29·9)**	**31·1 (27·8–34·1)**	**35·5 (31·6–38·6)**	**40·0 (35·4–43·0)**	**1·3 (1·0–1·7)**	**1·2 (0·9–1·6)**	**1·1 (0·6–1·5)**
High fasting plasma glucose	12·6 (9·7–15·4)	16·4 (12·3–19·7)	21·4 (15·9–25·7)	25·2 (18·8–30·5)	2·2 (1·5–3·0)	2·1 (1·2–3·0)	1·5 (0·3–2·7)
High LDL cholesterol	63·6 (44·4–86·5)	53·7 (36·9–73·8)	48·1 (32·5–67·0)	46·8 (31·4–65·5)	-1·0 (-1·2–-0·8)	-0·7 (-0·9–-0·5)	-0·2 (-0·5–0·0)
High systolic blood pressure	28·6 (19·0–39·5)	24·4 (16·9–33·1)	18·5 (12·0–26·7)	26·1 (16·3–36·9)	-0·3 (-1·2–0·5)	0·3 (-0·9–1·4)	3·1 (1·0–5·3)
High body-mass index	30·0 (25·0–34·2)	35·8 (30·7–39·8)	41·0 (35·0–45·0)	45·5 (38·9–49·2)	1·3 (1·0–1·7)	1·1 (0·8–1·5)	1·0 (0·5–1·4)
Low bone mineral density	21·5 (14·8–28·6)	19·3 (13·7–26·1)	20·0 (14·7–26·8)	22·0 (15·3–28·9)	0·1 (-0·5–0·6)	0·6 (-0·2–1·4)	0·9 (-0·5–2·4)
Kidney dysfunction	2·9 (2·4–3·8)	3·0 (2·4–3·9)	2·9 (2·4–3·9)	3·1 (2·5–4·0)	0·2 (0·1–0·3)	0·1 (0·0–0·3)	0·4 (0·1–0·7)

Kentucky

Risk Names	SEV 1990	SEV 2000	SEV 2010	SEV 2021	Annualised rate of change 1990 to 2021	Annualised rate of change 2000 to 2021	Annualised rate of change 2010 to 2021
**All risk factors**	**27·0 (23·9–30·0)**	**27·2 (24·7–30·1)**	**27·6 (25·0–30·5)**	**27·6 (24·6–30·7)**	**0·1 (-0·2–0·3)**	**0·1 (-0·2–0·4)**	**0·0 (-0·4–0·5)**
**Environmental/occupational risks**	**20·1 (13·4–30·6)**	**19·5 (13·7–30·1)**	**16·8 (11·5–26·9)**	**14·5 (9·4–23·8)**	**-1·1 (-1·9–-0·3)**	**-1·4 (-1·9–-0·8)**	**-1·4 (-2·0–-0·7)**
Unsafe water, sanitation, and handwashing	2·4 (1·2–4·0)	2·1 (1·0–3·4)	1·6 (0·5–2·7)	1·3 (0·5–2·4)	-1·9 (-4·2–0·1)	-2·3 (-5·2–0·3)	-1·8 (-5·6–2·0)
Unsafe water source	3·4 (1·2–6·9)	3·0 (1·2–5·9)	1·2 (0·5–2·5)	1·0 (0·4–2·0)	-4·0 (-6·9–-1·1)	-5·4 (-9·5–-1·8)	-2·1 (-7·6–3·7)
Unsafe sanitation	8·6 (4·6–14·0)	6·7 (3·5–11·1)	5·1 (2·6–8·8)	3·9 (2·1–6·7)	-2·6 (-4·8–-0·5)	-2·6 (-5·3–0·1)	-2·5 (-5·9–0·8)
No access to handwashing facility	1·8 (0·5–3·4)	1·8 (0·6–3·5)	1·8 (0·6–3·5)	1·6 (0·6–3·2)	-0·4 (-3·5–2·5)	-0·7 (-4·5–3·5)	-1·4 (-7·1–4·2)
Air pollution	24·9 (9·8–37·4)	22·3 (11·0–32·4)	13·1 (6·5–22·5)	8·0 (3·2–16·6)	-3·7 (-5·9–-1·8)	-4·9 (-7·6–-3·0)	-4·5 (-8·1–-2·9)
Particulate matter pollution	16·1 (6·8–26·9)	14·2 (10·5–18·4)	9·1 (6·0–12·9)	5·5 (2·7–8·8)	-3·4 (-5·8–-1·2)	-4·5 (-6·7–-3·3)	-4·5 (-8·2–-2·9)
Ambient particulate matter pollution	25·0 (10·7–42·6)	22·1 (16·9–27·6)	14·0 (9·5–19·3)	8·5 (4·2–13·1)	-3·5 (-5·9–-1·2)	-4·6 (-6·8–-3·4)	-4·6 (-8·3–-2·9)
Household air pollution from solid fuels	0·0 (0·0–0·1)	0·0 (0·0–0·1)	0·0 (0·0–0·0)	0·0 (0·0–0·0)	-4·7 (-27·2–-6·2)	-4·1 (-23·1–-4·7)	-3·6 (-18·3–-3·1)
Ambient ozone pollution	25·9 (19·8–33·8)	32·6 (25·8–39·9)	22·1 (16·6–29·6)	11·9 (7·5–18·0)	-2·5 (-3·4–-1·9)	-4·8 (-6·1–-3·7)	-5·6 (-7·8–-4·0)
Ambient nitrogen dioxide pollution	60·0 (0·0–100·0)	53·7 (0·0–100·0)	27·1 (0·0–76·5)	16·7 (0·0–60·0)	-4·1 (-13·3–0·0)	-5·5 (-18·5–0·0)	-4·4 (-19·9–0·0)
Non-optimal temperature	26·8 (20·7–36·2)	27·0 (21·8–35·9)	32·1 (25·4–43·0)	27·1 (21·7–36·6)	0·0 (-0·2–0·4)	0·0 (-0·3–0·4)	-1·5 (-2·4–-0·6)
High temperature	22·1 (13·8–32·5)	18·2 (10·8–28·9)	29·7 (19·3–41·4)	20·1 (12·0–30·8)	-0·3 (-1·0–0·1)	0·5 (-0·2–1·2)	-3·5 (-5·3–-2·4)
Low temperature	25·0 (20·9–29·3)	26·2 (22·7–30·0)	29·0 (26·0–32·7)	26·0 (22·6–30·2)	0·1 (-0·1–0·3)	0·0 (-0·3–0·2)	-1·0 (-1·4–-0·5)
Other environmental risks	27·2 (9·4–47·2)	25·8 (9·5–47·0)	23·4 (9·4–43·9)	20·5 (9·3–40·3)	-0·9 (-1·5–0·0)	-1·1 (-1·9–0·0)	-1·2 (-2·1–0·0)
Residential radon	26·5 (0·0–89·9)	26·5 (0·0–89·9)	26·5 (0·0–89·9)	26·5 (0·0–89·9)	0·0 (0·0–0·0)	0·0 (0·0–0·0)	0·0 (0·0–0·0)
Lead exposure	27·4 (0·0–34·5)	25·4 (0·0–31·4)	21·9 (0·0–27·0)	17·7 (0·0–21·9)	-1·4 (-1·8–0·0)	-1·7 (-2·2–0·0)	-1·9 (-2·7–0·0)
Occupational risks	2·9 (2·4–3·8)	3·0 (2·4–3·9)	2·7 (2·2–3·6)	2·7 (2·2–3·6)	-0·2 (-0·5–0·1)	-0·4 (-0·8–0·0)	0·1 (-0·4–0·6)
Occupational carcinogens	0·9 (0·4–1·7)	1·0 (0·4–1·8)	0·9 (0·4–1·7)	0·9 (0·4–1·8)	0·2 (-0·1–0·5)	0·0 (-0·5–0·4)	0·4 (-0·2–1·0)
Occupational exposure to asbestos	3·3 (3·0–3·7)	3·8 (3·4–4·2)	3·4 (3·1–3·7)	3·4 (2·7–4·2)	0·1 (-0·8–0·8)	-0·5 (-1·7–0·6)	0·0 (-2·1–1·9)
Occupational exposure to arsenic	0·5 (0·0–1·3)	0·5 (0·0–1·3)	0·5 (0·0–1·1)	0·5 (0·0–1·1)	-0·3 (-0·7–0·2)	-0·5 (-1·2–0·2)	0·1 (-0·8–1·1)
Occupational exposure to benzene	1·0 (0·1–2·8)	1·1 (0·1–3·2)	1·1 (0·1–3·1)	1·1 (0·1–3·4)	0·4 (0·0–0·8)	0·2 (-0·3–0·6)	0·5 (-0·2–1·2)
Occupational exposure to beryllium	0·0 (0·0–0·0)	0·0 (0·0–0·0)	0·0 (0·0–0·0)	0·0 (0·0–0·0)	0·2 (-0·1–0·4)	0·0 (-0·3–0·4)	0·6 (0·1–1·1)
Occupational exposure to cadmium	0·1 (0·1–0·1)	0·1 (0·1–0·1)	0·1 (0·1–0·1)	0·1 (0·1–0·1)	-0·2 (-0·6–0·2)	-0·4 (-1·0–0·2)	0·2 (-0·6–1·1)
Occupational exposure to chromium	0·1 (0·1–0·2)	0·1 (0·1–0·2)	0·1 (0·1–0·1)	0·1 (0·1–0·2)	-0·1 (-0·5–0·3)	-0·3 (-0·9–0·2)	0·3 (-0·4–1·0)
Occupational exposure to diesel engine exhaust	0·7 (0·6–0·7)	0·7 (0·6–0·7)	0·6 (0·6–0·7)	0·7 (0·6–0·8)	0·2 (-0·2–0·5)	0·2 (-0·3–0·7)	0·8 (0·1–1·5)
Occupational exposure to formaldehyde	0·3 (0·3–0·3)	0·3 (0·3–0·3)	0·3 (0·3–0·3)	0·3 (0·3–0·3)	-0·2 (-0·6–0·2)	-0·5 (-1·1–0·1)	0·1 (-0·7–0·9)
Occupational exposure to nickel	0·4 (0·0–1·7)	0·4 (0·0–1·6)	0·4 (0·0–1·4)	0·4 (0·0–1·5)	-0·4 (-1·0–0·1)	-0·6 (-1·4–0·2)	0·1 (-1·0–1·2)
Occupational exposure to polycyclic aromatic hydrocarbons	0·3 (0·3–0·3)	0·3 (0·3–0·3)	0·3 (0·3–0·3)	0·3 (0·3–0·3)	0·0 (-0·4–0·3)	-0·2 (-0·8–0·2)	0·3 (-0·3–1·0)
Occupational exposure to silica	4·0 (0·6–13·1)	3·8 (0·6–11·9)	3·3 (0·5–10·4)	3·4 (0·5–10·8)	-0·6 (-1·1–0·0)	-0·6 (-1·3–0·1)	0·1 (-0·8–1·0)
Occupational exposure to sulphuric acid	0·7 (0·2–2·4)	0·8 (0·2–2·5)	0·7 (0·1–2·3)	0·7 (0·1–2·3)	-0·2 (-0·6–0·2)	-0·4 (-1·0–0·1)	0·2 (-0·6–1·0)
Occupational exposure to trichloroethylene	0·1 (0·1–0·1)	0·1 (0·1–0·1)	0·1 (0·1–0·1)	0·1 (0·1–0·1)	0·1 (-0·2–0·4)	-0·1 (-0·5–0·3)	0·5 (-0·1–1·1)
Occupational asthmagens	17·7 (15·6–20·1)	17·9 (15·7–20·2)	15·8 (13·8–17·9)	16·2 (14·1–18·6)	-0·3 (-0·6–0·1)	-0·5 (-0·9–0·0)	0·3 (-0·5–1·0)
Occupational particulate matter, gases, and fumes	7·0 (5·4–9·3)	6·9 (5·3–9·1)	6·5 (5·0–8·5)	5·9 (4·5–7·7)	-0·5 (-0·8–-0·3)	-0·7 (-1·0–-0·4)	-0·8 (-1·2–-0·4)
Occupational noise	7·0 (6·5–7·7)	7·0 (6·5–7·7)	6·9 (6·5–7·4)	6·6 (6·2–7·1)	-0·2 (-0·4–0·0)	-0·3 (-0·5–-0·1)	-0·4 (-0·6–-0·2)
Occupational injuries	--	--	--	--	--	--	--
Occupational ergonomic factors	8·4 (6·9–10·2)	8·3 (7·0–10·0)	7·2 (6·1–8·7)	7·5 (6·2–9·2)	-0·4 (-0·9–0·1)	-0·5 (-1·1–0·1)	0·4 (-0·4–1·1)
**Behavioural risks**	**31·0 (28·0–34·1)**	**30·2 (27·2–33·3)**	**29·1 (26·3–32·3)**	**27·0 (23·8–30·4)**	**-0·4 (-0·8–-0·2)**	**-0·5 (-1·0–-0·2)**	**-0·7 (-1·3–-0·1)**
Child and maternal malnutrition	10·1 (6·9–14·2)	8·8 (6·0–12·4)	9·1 (6·4–12·8)	9·3 (6·4–13·2)	-0·3 (-1·1–0·6)	0·3 (-0·9–1·5)	0·3 (-1·4–2·0)
Suboptimal breastfeeding	69·1 (66·5–71·7)	66·9 (64·3–69·8)	63·0 (60·4–65·9)	62·4 (59·4–65·2)	-0·3 (-0·5–-0·2)	-0·3 (-0·5–-0·1)	-0·1 (-0·4–0·2)
Non-exclusive breastfeeding	64·8 (56·4–72·0)	60·0 (53·0–67·2)	49·0 (42·1–55·8)	46·7 (39·7–53·7)	-1·1 (-1·6–-0·6)	-1·2 (-1·9–-0·5)	-0·4 (-1·5–0·5)
Discontinued breastfeeding	88·9 (86·4–91·5)	86·5 (83·9–89·3)	84·6 (81·4–87·8)	84·7 (81·3–88·0)	-0·2 (-0·3–0·0)	-0·1 (-0·3–0·1)	0·0 (-0·3–0·3)
Child growth failure	1·4 (0·4–3·1)	1·3 (0·4–2·8)	1·1 (0·4–2·6)	1·1 (0·3–2·3)	-0·8 (-1·2–-0·4)	-0·9 (-1·5–-0·5)	-0·8 (-1·5–0·0)
Child underweight	2·9 (1·8–4·0)	2·7 (1·7–3·7)	2·4 (1·5–3·3)	2·2 (1·4–3·0)	-0·9 (-1·4–-0·4)	-1·0 (-1·7–-0·3)	-0·7 (-1·7–0·3)
Child wasting	0·9 (0·6–1·4)	0·9 (0·6–1·3)	0·9 (0·6–1·4)	0·8 (0·5–1·3)	-0·2 (-0·6–0·1)	-0·1 (-0·5–0·3)	-0·4 (-1·1–0·3)
Child stunting	3·9 (3·1–4·9)	3·7 (2·9–4·5)	3·3 (2·5–4·2)	2·9 (2·3–3·7)	-0·9 (-1·5–-0·3)	-1·1 (-1·8–-0·3)	-1·0 (-2·1–0·3)
Low birth weight and short gestation	24·0 (21·4–27·0)	26·3 (23·3–29·6)	29·3 (25·9–32·9)	27·8 (24·8–31·3)	0·5 (0·2–0·7)	0·3 (-0·1–0·7)	-0·5 (-1·2–0·2)
Short gestation	42·6 (38·0–47·4)	47·0 (42·3–52·7)	51·0 (45·5–57·5)	48·4 (43·4–54·0)	0·4 (0·1–0·7)	0·1 (-0·3–0·6)	-0·5 (-1·2–0·3)
Low birth weight	18·9 (17·4–20·5)	20·2 (18·5–22·2)	22·5 (20·5–24·6)	21·5 (19·7–23·5)	0·4 (0·2–0·6)	0·3 (-0·1–0·7)	-0·4 (-1·0–0·2)

Iron deficiency	3·6 (2·5–5·0)	3·1 (2·2–4·3)	3·3 (2·4–4·5)	3·3 (2·4–4·7)	-0·2 (-1·0–0·5)	0·3 (-0·8–1·4)	0·2 (-1·3–1·8)
Vitamin A deficiency	1·4 (0·0–2·9)	1·9 (0·0–4·0)	1·2 (0·0–2·5)	0·8 (0·0–1·6)	-1·9 (-4·0–0·3)	-4·2 (-6·8–0·0)	-4·3 (-8·1–0·0)
Zinc deficiency	2·1 (0·0–9·3)	1·2 (0·0–5·3)	1·1 (0·0–5·0)	1·1 (0·0–5·0)	-2·1 (-48·2–0·0)	-0·5 (-1·9–0·6)	-0·1 (-1·9–4·9)
Tobacco	46·6 (44·9–48·0)	45·5 (44·0–46·9)	40·3 (39·1–41·7)	35·0 (32·9–37·1)	-0·9 (-1·1–-0·7)	-1·2 (-1·5–-1·0)	-1·3 (-1·8–-0·8)
Smoking	44·7 (42·6–46·6)	41·9 (40·0–43·6)	36·9 (35·2–38·4)	32·5 (29·6–35·4)	-1·0 (-1·3–-0·7)	-1·2 (-1·6–-0·8)	-1·1 (-1·9–-0·4)
Chewing tobacco	4·4 (3·7–5·3)	4·6 (3·8–5·5)	4·5 (3·5–5·7)	4·4 (3·3–5·8)	0·0 (-1·2–1·0)	-0·2 (-1·7–1·3)	-0·3 (-2·7–1·8)
Second-hand smoke	36·5 (34·6–37·8)	36·5 (34·3–37·9)	32·2 (30·5–33·5)	27·8 (25·9–29·6)	-0·9 (-1·1–-0·7)	-1·3 (-1·6–-1·0)	-1·3 (-2·0–-0·8)
High alcohol use	22·1 (16·1–30·7)	20·5 (14·7–28·7)	22·1 (15·9–31·5)	22·7 (15·5–31·6)	0·1 (-1·1–1·0)	0·5 (-1·0–1·8)	0·3 (-1·8–2·0)
Drug use	0·9 (0·7–1·2)	1·4 (1·1–1·8)	3·1 (2·1–4·1)	7·7 (5·3–9·9)	6·9 (5·0–8·1)	8·0 (6·9–8·7)	8·3 (7·3–9·3)
Dietary risks	40·0 (30·7–51·3)	43·2 (33·6–55·5)	45·5 (35·9–58·1)	45·2 (35·5–57·6)	0·4 (0·2–0·7)	0·2 (-0·1–0·5)	-0·1 (-0·5–0·4)
Diet low in fruits	42·3 (35·8–45·7)	40·5 (34·9–43·8)	39·9 (34·8–43·2)	40·5 (35·2–44·6)	-0·1 (-0·5–0·2)	0·0 (-0·5–0·4)	0·1 (-0·6–0·9)
Diet low in vegetables	31·9 (19·3–39·5)	33·4 (19·9–41·2)	37·7 (21·2–45·8)	36·7 (21·3–45·2)	0·5 (0·1–0·8)	0·5 (0·0–0·9)	-0·2 (-1·1–0·5)
Diet low in legumes	39·6 (0·0–49·7)	39·7 (0·0–49·5)	43·8 (0·0–54·7)	44·6 (0·0–55·7)	0·4 (0·0–0·7)	0·6 (0·0–1·0)	0·2 (-0·6–0·9)
Diet low in whole grains	45·5 (38·0–51·8)	48·7 (40·4–55·4)	50·6 (41·7–57·8)	51·0 (41·8–58·5)	0·4 (0·1–0·7)	0·2 (-0·1–0·6)	0·1 (-0·4–0·6)
Diet low in nuts and seeds	15·1 (11·5–19·6)	9·1 (6·1–12·7)	6·7 (4·4–9·5)	7·1 (4·0–10·6)	-2·4 (-4·4–-1·0)	-1·2 (-3·7–1·0)	0·6 (-3·0–3·9)
Diet low in milk	38·9 (34·9–43·0)	35·9 (31·7–40·1)	34·2 (29·6–39·0)	33·8 (29·1–38·6)	-0·5 (-0·9–0·0)	-0·3 (-0·9–0·3)	-0·1 (-0·9–0·7)
Diet high in red meat	52·0 (0·0–70·4)	52·5 (0·0–71·8)	52·6 (0·0–71·8)	52·3 (0·0–71·3)	0·0 (-0·4–0·4)	0·0 (-0·7–0·6)	0·0 (-1·0–0·8)
Diet high in processed meat	51·8 (41·4–57·8)	63·2 (50·9–70·5)	69·6 (56·1–77·9)	67·2 (54·3–75·0)	0·8 (0·4–1·3)	0·3 (-0·2–0·8)	-0·3 (-1·1–0·4)
Diet high in sugar-sweetened beverages	33·3 (26·0–40·8)	47·4 (37·9–55·9)	52·9 (42·0–62·5)	52·2 (40·5–61·5)	1·4 (0·6–2·4)	0·5 (-0·6–1·4)	-0·1 (-1·5–1·2)
Diet low in fibre	42·7 (23·5–50·5)	32·8 (17·9–39·9)	29·1 (16·0–36·4)	25·8 (14·3–32·9)	-1·6 (-2·4–-0·8)	-1·1 (-2·3–0·0)	-1·1 (-2·8–0·6)
Diet low in calcium	6·3 (5·2–7·8)	5·2 (4·2–6·4)	5·5 (4·5–6·9)	5·4 (4·4–6·7)	-0·5 (-0·7–-0·3)	0·2 (-0·1–0·5)	-0·1 (-0·5–0·3)
Diet low in seafood omega-3 fatty acids	40·2 (30·8–50·0)	46·6 (36·2–57·1)	49·8 (39·6–60·7)	45·0 (34·7–55·9)	0·4 (0·0–0·8)	-0·2 (-0·6–0·4)	-0·9 (-1·6–-0·3)
Diet low in omega-6 polyunsaturated fatty acids	39·6 (24·8–50·2)	34·3 (22·5–44·1)	25·9 (17·6–33·7)	24·3 (16·5–32·1)	-1·6 (-2·2–-0·8)	-1·6 (-2·5–-0·8)	-0·6 (-1·6–0·4)
Diet high in trans fatty acids	71·3 (64·2–78·8)	71·7 (62·8–78·6)	63·2 (54·7–70·8)	0·0 (0·0–0·0)	-65·8 (-66·1–-65·4)	-97·1 (-97·5–-96·5)	-184·2 (-185·3–-182·9)
Diet high in sodium	23·5 (0·5–69·5)	28·4 (1·7–75·0)	31·7 (3·3–77·4)	32·0 (3·2–79·1)	1·0 (0·2–7·4)	0·6 (-0·1–4·2)	0·1 (-2·2–1·9)
Intimate partner violence	29·4 (18·5–36·6)	29·4 (18·3–36·2)	26·6 (18·9–32·6)	25·1 (14·8–34·1)	-0·5 (-1·9–0·8)	-0·7 (-2·6–0·9)	-0·5 (-2·9–1·4)
Childhood sexual abuse and bullying	9·5 (6·5–14·6)	11·4 (7·8–17·5)	13·0 (8·9–20·2)	9·8 (6·9–14·4)	0·1 (-0·5–0·7)	-0·7 (-1·5–0·1)	-2·6 (-4·1–-1·2)
Childhood sexual abuse	9·8 (8·4–11·6)	11·0 (9·5–12·7)	12·7 (11·1–14·7)	11·3 (9·3–14·1)	0·5 (-0·3–1·2)	0·1 (-1·0–1·1)	-1·1 (-2·5–0·3)
Bullying victimization	7·9 (3·5–15·8)	9·8 (4·5–19·2)	11·2 (5·0–21·9)	7·2 (3·2–14·1)	-0·3 (-1·1–0·5)	-1·5 (-2·5–-0·5)	-4·0 (-5·9–-2·3)
Unsafe sex	--	--	--	--	--	--	--
Low physical activity	23·8 (18·4–30·5)	23·6 (18·5–28·8)	21·7 (17·2–26·2)	23·6 (18·2–29·8)	0·0 (-0·9–0·9)	0·0 (-1·1–1·3)	0·8 (-0·9–2·5)
**Metabolic risks**	**26·7 (23·4–30·0)**	**33·4 (29·3–36·4)**	**36·8 (32·8–39·7)**	**40·7 (35·7–43·8)**	**1·4 (1·1–1·6)**	**0·9 (0·6–1·3)**	**0·9 (0·5–1·4)**
High fasting plasma glucose	13·7 (10·6–16·2)	18·4 (13·9–22·3)	23·5 (17·8–27·8)	26·3 (19·8–31·8)	2·1 (1·5–2·7)	1·7 (0·9–2·5)	1·0 (-0·1–2·3)
High LDL cholesterol	63·5 (44·6–86·9)	53·9 (37·2–73·7)	48·4 (32·6–67·2)	46·8 (31·6–64·9)	-1·0 (-1·2–-0·8)	-0·7 (-0·9–-0·5)	-0·3 (-0·6–-0·1)
High systolic blood pressure	30·5 (20·8–41·7)	26·4 (18·7–36·0)	20·5 (13·7–29·1)	28·5 (18·1–41·0)	-0·2 (-1·1–0·7)	0·4 (-0·8–1·4)	3·0 (0·8–5·2)
High body-mass index	30·2 (25·7–34·3)	38·0 (32·3–42·0)	42·1 (35·6–45·7)	46·1 (39·1–49·9)	1·4 (1·1–1·7)	0·9 (0·6–1·2)	0·8 (0·4–1·3)
Low bone mineral density	21·8 (15·6–29·2)	19·9 (13·9–26·9)	20·9 (15·4–27·8)	23·0 (16·6–29·8)	0·2 (-0·3–0·7)	0·7 (0·0–1·5)	0·9 (-0·5–2·2)
Kidney dysfunction	3·0 (2·4–3·9)	3·1 (2·5–4·0)	3·1 (2·5–4·1)	3·2 (2·6–4·2)	0·2 (0·1–0·3)	0·2 (0·0–0·3)	0·4 (0·1–0·7)

Louisiana

Risk Names	SEV 1990	SEV 2000	SEV 2010	SEV 2021	Annualised rate of change 1990 to 2021	Annualised rate of change 2000 to 2021	Annualised rate of change 2010 to 2021
**All risk factors**	**27·5 (24·5–31·1)**	**27·3 (24·1–30·9)**	**27·9 (24·9–31·3)**	**27·8 (24·4–31·3)**	**0·0 (-0·3–0·3)**	**0·1 (-0·3–0·4)**	**-0·1 (-0·6–0·5)**
**Environmental/occupational risks**	**16·2 (10·6–24·4)**	**16·5 (11·2–23·5)**	**14·6 (9·6–22·2)**	**11·3 (7·4–16·6)**	**-1·2 (-2·0–-0·4)**	**-1·8 (-2·5–-1·3)**	**-2·4 (-3·3–-1·7)**
Unsafe water, sanitation, and handwashing	2·4 (1·3–4·0)	2·2 (1·1–3·7)	1·6 (0·6–2·8)	1·3 (0·4–2·1)	-2·1 (-4·5–-0·1)	-2·6 (-5·5–0·0)	-2·1 (-6·3–1·8)
Unsafe water source	3·4 (1·3–6·9)	3·1 (1·1–6·3)	1·2 (0·5–2·6)	0·9 (0·4–1·8)	-4·2 (-7·1–-1·2)	-5·7 (-9·3–-1·6)	-2·3 (-7·4–3·6)
Unsafe sanitation	8·8 (4·8–14·3)	6·9 (3·6–11·4)	5·0 (2·6–8·7)	3·6 (1·9–6·6)	-2·9 (-5·0–-0·8)	-3·1 (-5·6–-0·3)	-2·9 (-6·0–0·7)
No access to handwashing facility	1·9 (0·6–3·6)	1·9 (0·6–4·1)	1·9 (0·6–3·6)	1·6 (0·4–3·1)	-0·5 (-3·5–2·6)	-0·9 (-4·7–3·1)	-1·7 (-7·5–4·5)
Air pollution	19·7 (6·9–31·8)	18·6 (9·6–29·3)	10·7 (5·2–19·5)	6·4 (2·2–14·1)	-3·6 (-6·0–-1·7)	-5·1 (-8·0–-3·4)	-4·6 (-8·8–-2·8)
Particulate matter pollution	12·7 (4·8–22·4)	12·7 (9·3–16·9)	7·9 (4·7–11·5)	4·8 (2·0–8·0)	-3·1 (-6·0–-0·4)	-4·6 (-7·7–-3·2)	-4·5 (-8·7–-2·6)
Ambient particulate matter pollution	19·7 (7·3–35·3)	19·7 (14·8–25·2)	12·1 (7·3–17·2)	7·3 (3·0–12·0)	-3·2 (-6·0–-0·3)	-4·7 (-7·7–-3·3)	-4·6 (-8·7–-2·7)
Household air pollution from solid fuels	0·0 (0·0–0·0)	0·0 (0·0–0·0)	0·0 (0·0–0·0)	0·0 (0·0–0·0)	-3·8 (-34·4–-6·9)	-4·0 (-36·1–-7·4)	-2·2 (-28·3–-5·8)
Ambient ozone pollution	17·7 (12·2–24·9)	22·6 (16·7–30·2)	15·8 (10·5–22·5)	6·2 (2·7–10·8)	-3·4 (-5·2–-2·5)	-6·2 (-9·1–-4·5)	-8·5 (-13·2–-5·9)
Ambient nitrogen dioxide pollution	48·0 (0·0–100·0)	41·0 (0·0–97·1)	20·4 (0·0–65·0)	12·3 (0·0–50·5)	-4·4 (-13·8–0·0)	-5·7 (-18·6–0·0)	-4·6 (-18·1–0·0)
Non-optimal temperature	36·6 (26·6–47·0)	41·3 (31·1–51·8)	47·8 (36·5–59·3)	35·5 (26·0–46·1)	-0·1 (-0·5–0·3)	-0·7 (-1·2–-0·3)	-2·7 (-3·6–-2·0)
High temperature	32·6 (21·4–43·3)	40·8 (30·4–51·6)	43·8 (31·5–55·4)	29·6 (21·1–39·6)	-0·3 (-0·5–0·1)	-1·5 (-2·1–-1·1)	-3·6 (-4·5–-2·7)
Low temperature	29·3 (25·1–33·1)	32·1 (28·4–35·6)	39·1 (35·4–42·9)	30·1 (25·8–33·9)	0·1 (-0·3–0·4)	-0·3 (-0·7–-0·1)	-2·4 (-3·2–-1·8)
Other environmental risks	28·6 (7·0–47·4)	26·9 (7·0–45·8)	24·1 (7·0–43·0)	20·6 (7·1–39·7)	-1·1 (-1·7–0·0)	-1·3 (-2·0–0·0)	-1·4 (-2·4–0·0)
Residential radon	23·1 (0·0–81·0)	23·1 (0·0–81·0)	23·1 (0·0–81·0)	23·1 (0·0–81·0)	0·0 (0·0–0·0)	0·0 (0·0–0·0)	0·0 (0·0–0·0)
Lead exposure	31·0 (0·0–38·7)	28·4 (0·0–35·3)	24·4 (0·0–30·1)	19·4 (0·0–24·1)	-1·5 (-1·9–0·0)	-1·8 (-2·3–0·0)	-2·1 (-2·8–0·0)
Occupational risks	2·9 (2·4–3·8)	3·0 (2·5–3·9)	2·7 (2·3–3·6)	2·8 (2·2–3·5)	-0·2 (-0·5–0·1)	-0·4 (-0·8–0·0)	0·1 (-0·4–0·5)
Occupational carcinogens	1·0 (0·5–1·8)	1·1 (0·5–2·0)	1·0 (0·5–1·9)	1·1 (0·5–1·9)	0·2 (-0·1–0·5)	-0·1 (-0·6–0·3)	0·3 (-0·4–0·9)
Occupational exposure to asbestos	5·2 (4·7–5·8)	6·3 (5·7–6·8)	5·3 (4·8–5·7)	5·2 (4·2–6·3)	0·0 (-0·7–0·7)	-0·9 (-2·0–0·2)	0·0 (-2·0–1·8)
Occupational exposure to arsenic	0·4 (0·0–1·1)	0·5 (0·0–1·1)	0·4 (0·0–1·0)	0·4 (0·0–1·1)	-0·1 (-0·6–0·4)	-0·2 (-0·8–0·4)	0·2 (-0·7–1·0)
Occupational exposure to benzene	1·1 (0·1–3·1)	1·2 (0·1–3·4)	1·1 (0·1–3·3)	1·2 (0·1–3·5)	0·4 (0·0–0·7)	0·1 (-0·3–0·6)	0·4 (-0·3–1·0)
Occupational exposure to beryllium	0·0 (0·0–0·0)	0·0 (0·0–0·0)	0·0 (0·0–0·0)	0·0 (0·0–0·0)	0·2 (0·0–0·5)	0·1 (-0·3–0·4)	0·5 (0·0–1·0)
Occupational exposure to cadmium	0·1 (0·0–0·1)	0·1 (0·1–0·1)	0·1 (0·0–0·1)	0·1 (0·0–0·1)	0·0 (-0·4–0·4)	-0·2 (-0·7–0·4)	0·3 (-0·4–1·0)
Occupational exposure to chromium	0·1 (0·1–0·1)	0·1 (0·1–0·1)	0·1 (0·1–0·1)	0·1 (0·1–0·1)	0·1 (-0·3–0·4)	-0·1 (-0·6–0·4)	0·3 (-0·3–1·0)
Occupational exposure to diesel engine exhaust	0·7 (0·7–0·8)	0·7 (0·7–0·8)	0·7 (0·6–0·7)	0·7 (0·7–0·8)	0·0 (-0·4–0·3)	0·0 (-0·5–0·5)	0·6 (-0·2–1·3)
Occupational exposure to formaldehyde	0·3 (0·2–0·3)	0·3 (0·3–0·3)	0·3 (0·2–0·3)	0·3 (0·2–0·3)	0·0 (-0·5–0·4)	-0·2 (-0·8–0·3)	0·2 (-0·5–0·9)
Occupational exposure to nickel	0·4 (0·0–1·6)	0·4 (0·0–1·6)	0·4 (0·0–1·4)	0·4 (0·0–1·4)	-0·3 (-0·9–0·2)	-0·5 (-1·3–0·3)	0·1 (-0·9–1·1)
Occupational exposure to polycyclic aromatic hydrocarbons	0·3 (0·3–0·3)	0·3 (0·3–0·3)	0·3 (0·3–0·3)	0·3 (0·3–0·3)	0·1 (-0·2–0·4)	-0·1 (-0·5–0·4)	0·3 (-0·3–0·9)
Occupational exposure to silica	3·9 (0·6–13·1)	3·7 (0·6–12·3)	3·3 (0·5–10·7)	3·3 (0·5–10·6)	-0·6 (-1·1–-0·1)	-0·6 (-1·3–0·0)	0·0 (-0·9–0·9)
Occupational exposure to sulphuric acid	0·7 (0·1–2·2)	0·7 (0·1–2·3)	0·6 (0·1–2·0)	0·7 (0·1–2·2)	0·0 (-0·4–0·4)	-0·2 (-0·7–0·4)	0·3 (-0·5–1·1)
Occupational exposure to trichloroethylene	0·1 (0·1–0·1)	0·1 (0·1–0·1)	0·1 (0·1–0·1)	0·1 (0·1–0·1)	0·1 (-0·2–0·4)	0·0 (-0·4–0·5)	0·5 (-0·1–1·0)
Occupational asthmagens	17·4 (15·4–19·6)	17·6 (15·5–19·9)	15·7 (13·7–17·7)	16·0 (14·0–18·6)	-0·3 (-0·7–0·1)	-0·4 (-1·0–0·1)	0·2 (-0·6–0·8)
Occupational particulate matter, gases, and fumes	6·2 (4·8–8·0)	6·1 (4·7–7·9)	5·8 (4·4–7·5)	5·3 (4·1–6·9)	-0·5 (-0·8–-0·2)	-0·6 (-1·0–-0·4)	-0·7 (-1·2–-0·4)
Occupational noise	6·8 (6·3–7·5)	6·9 (6·4–7·5)	6·7 (6·3–7·3)	6·5 (6·1–7·0)	-0·2 (-0·4–0·0)	-0·3 (-0·5–-0·1)	-0·4 (-0·6–-0·1)
Occupational injuries	--	--	--	--	--	--	--
Occupational ergonomic factors	8·2 (6·8–10·0)	8·1 (6·9–9·9)	7·3 (6·2–8·8)	7·5 (6·2–9·1)	-0·3 (-0·8–0·1)	-0·4 (-1·0–0·1)	0·3 (-0·5–1·0)
**Behavioural risks**	**29·6 (26·4–33·3)**	**27·9 (24·6–31·8)**	**27·3 (23·8–31·1)**	**25·7 (22·4–29·5)**	**-0·4 (-0·8–-0·1)**	**-0·4 (-0·9–0·1)**	**-0·5 (-1·2–0·1)**
Child and maternal malnutrition	10·7 (7·3–14·7)	9·2 (6·3–13·2)	9·3 (6·4–13·1)	9·5 (6·5–13·3)	-0·4 (-1·2–0·4)	0·1 (-1·1–1·2)	0·2 (-1·6–1·8)
Suboptimal breastfeeding	69·0 (66·6–71·8)	66·9 (64·4–69·4)	63·0 (60·3–65·7)	62·4 (59·7–65·3)	-0·3 (-0·5–-0·2)	-0·3 (-0·5–-0·1)	-0·1 (-0·4–0·2)
Non-exclusive breastfeeding	64·8 (57·3–72·9)	60·1 (53·3–67·1)	49·0 (42·2–55·9)	46·7 (39·4–53·8)	-1·1 (-1·6–-0·5)	-1·2 (-1·9–-0·6)	-0·5 (-1·4–0·5)
Discontinued breastfeeding	88·9 (86·9–91·3)	86·6 (84·0–89·2)	84·6 (81·6–87·7)	84·7 (81·5–88·0)	-0·2 (-0·3–0·0)	-0·1 (-0·3–0·1)	0·0 (-0·3–0·3)
Child growth failure	1·5 (0·5–3·5)	1·4 (0·5–3·1)	1·1 (0·4–2·5)	1·1 (0·3–2·4)	-1·1 (-1·5–-0·7)	-1·2 (-1·7–-0·7)	-0·5 (-1·3–0·2)
Child underweight	3·2 (2·0–4·4)	2·9 (1·9–4·0)	2·4 (1·4–3·2)	2·3 (1·4–3·1)	-1·1 (-1·6–-0·6)	-1·2 (-1·9–-0·5)	-0·5 (-1·4–0·6)
Child wasting	1·0 (0·6–1·5)	0·9 (0·6–1·4)	0·9 (0·6–1·3)	0·9 (0·5–1·3)	-0·4 (-0·7–-0·1)	-0·3 (-0·7–0·2)	-0·2 (-1·0–0·5)
Child stunting	4·2 (3·3–5·4)	3·9 (3·1–4·9)	3·3 (2·5–4·1)	3·0 (2·3–3·8)	-1·1 (-1·7–-0·6)	-1·3 (-2·0–-0·5)	-0·7 (-1·9–0·4)
Low birth weight and short gestation	25·3 (22·1–28·6)	27·4 (23·9–31·2)	29·0 (25·2–32·6)	30·6 (26·5–34·5)	0·6 (0·4–0·9)	0·5 (0·2–0·9)	0·5 (-0·2–1·2)
Short gestation	44·3 (39·3–49·9)	48·0 (42·2–54·8)	49·7 (44·0–55·9)	53·0 (46·5–59·9)	0·6 (0·3–0·9)	0·5 (0·1–0·9)	0·6 (-0·3–1·4)
Low birth weight	19·1 (17·3–20·8)	20·2 (18·5–22·4)	21·6 (19·7–23·8)	22·4 (20·4–24·8)	0·5 (0·3–0·8)	0·5 (0·2–0·8)	0·3 (-0·4–1·0)

Iron deficiency	3·9 (2·7–5·2)	3·4 (2·3–4·7)	3·4 (2·4–4·7)	3·4 (2·4–4·7)	-0·4 (-1·2–0·3)	0·1 (-1·1–1·1)	0·0 (-1·7–1·6)
Vitamin A deficiency	1·4 (0·0–3·0)	2·0 (0·0–4·4)	1·2 (0·0–2·6)	0·8 (0·0–1·7)	-2·0 (-4·3–0·1)	-4·6 (-7·7–0·0)	-4·2 (-8·2–0·0)
Zinc deficiency	2·1 (0·0–8·8)	1·2 (0·0–5·3)	1·1 (0·0–4·7)	1·0 (0·0–4·5)	-2·2 (-47·7–0·1)	-0·8 (-2·5–4·8)	-0·3 (-4·5–5·2)
Tobacco	42·2 (40·4–44·0)	37·9 (36·6–39·2)	34·6 (33·4–35·7)	31·1 (29·0–32·8)	-1·0 (-1·2–-0·8)	-0·9 (-1·3–-0·6)	-1·0 (-1·6–-0·5)
Smoking	37·4 (34·8–39·9)	32·5 (30·7–34·4)	29·5 (28·1–31·0)	26·9 (24·7–29·3)	-1·1 (-1·4–-0·7)	-0·9 (-1·3–-0·5)	-0·8 (-1·6–-0·1)
Chewing tobacco	2·0 (1·6–2·6)	2·3 (1·9–2·9)	2·4 (1·8–3·1)	2·3 (1·7–3·1)	0·4 (-0·8–1·5)	-0·1 (-1·8–1·7)	-0·3 (-3·0–2·0)
Second-hand smoke	38·4 (36·1–40·2)	36·0 (33·7–37·5)	33·5 (31·1–34·9)	29·6 (27·2–31·9)	-0·8 (-1·1–-0·6)	-0·9 (-1·3–-0·6)	-1·1 (-1·7–-0·5)
High alcohol use	24·1 (16·5–34·4)	22·8 (15·7–33·3)	24·1 (16·8–35·3)	24·6 (17·6–35·5)	0·1 (-1·3–1·2)	0·4 (-1·4–1·9)	0·2 (-2·0–2·1)
Drug use	1·0 (0·6–1·6)	1·4 (1·2–1·7)	2·6 (2·0–3·3)	6·0 (4·1–7·6)	5·9 (3·2–8·1)	6·9 (4·6–8·1)	7·5 (5·7–8·7)
Dietary risks	39·1 (30·0–50·3)	42·2 (32·7–54·3)	44·9 (35·4–57·0)	44·9 (35·4–57·1)	0·4 (0·2–0·7)	0·3 (0·0–0·6)	0·0 (-0·4–0·4)
Diet low in fruits	40·6 (34·4–44·5)	38·5 (33·1–42·0)	38·7 (34·0–42·1)	39·9 (34·5–44·8)	-0·1 (-0·4–0·3)	0·2 (-0·3–0·7)	0·3 (-0·5–1·0)
Diet low in vegetables	31·9 (19·5–39·9)	34·3 (20·8–42·0)	39·2 (22·6–47·4)	38·5 (23·1–47·3)	0·6 (0·3–1·0)	0·6 (0·1–1·0)	-0·2 (-0·9–0·5)
Diet low in legumes	39·3 (0·0–49·3)	39·8 (0·0–49·7)	43·5 (0·0–54·4)	43·7 (0·0–54·8)	0·3 (0·0–0·7)	0·4 (0·0–0·9)	0·0 (-0·7–0·7)
Diet low in whole grains	45·6 (37·8–51·9)	49·0 (40·1–55·3)	50·8 (41·6–58·7)	51·3 (42·7–58·8)	0·4 (0·1–0·7)	0·2 (-0·1–0·6)	0·1 (-0·4–0·6)
Diet low in nuts and seeds	14·8 (10·4–19·1)	9·2 (6·2–12·6)	6·3 (3·9–9·4)	6·3 (3·8–9·6)	-2·8 (-4·5–-1·2)	-1·8 (-4·1–0·3)	0·0 (-3·6–3·5)
Diet low in milk	38·7 (34·0–43·4)	36·2 (31·8–40·7)	33·6 (29·0–38·1)	32·6 (28·1–37·8)	-0·6 (-1·0–-0·2)	-0·5 (-1·1–0·0)	-0·3 (-1·2–0·5)
Diet high in red meat	55·1 (0·0–75·1)	55·1 (0·0–74·3)	55·4 (0·0–75·1)	55·4 (0·0–74·7)	0·0 (-0·4–0·4)	0·0 (-0·5–0·6)	0·0 (-0·8–0·9)
Diet high in processed meat	45·5 (36·2–50·8)	56·3 (45·0–62·8)	63·3 (50·7–72·2)	61·9 (50·1–70·6)	1·0 (0·5–1·4)	0·5 (-0·2–1·0)	-0·2 (-1·0–0·5)
Diet high in sugar-sweetened beverages	33·1 (24·5–41·8)	46·6 (36·4–55·1)	53·2 (42·9–61·6)	53·6 (41·0–64·4)	1·6 (0·6–2·3)	0·7 (-0·4–1·7)	0·1 (-1·3–1·5)
Diet low in fibre	42·2 (23·3–49·4)	32·9 (18·4–39·6)	28·2 (15·8–35·1)	24·1 (14·0–30·9)	-1·8 (-2·8–-1·0)	-1·5 (-2·8–-0·3)	-1·4 (-3·1–0·2)
Diet low in calcium	6·6 (5·4–8·0)	5·5 (4·4–6·6)	5·5 (4·4–6·8)	5·3 (4·2–6·5)	-0·7 (-1·0–-0·5)	-0·2 (-0·5–0·1)	-0·4 (-0·8–0·0)
Diet low in seafood omega-3 fatty acids	40·0 (30·9–50·9)	46·9 (37·3–58·1)	48·4 (38·8–59·8)	42·8 (33·0–53·8)	0·2 (-0·2–0·7)	-0·4 (-1·0–0·2)	-1·1 (-2·0–-0·4)
Diet low in omega-6 polyunsaturated fatty acids	39·2 (24·4–50·0)	34·6 (22·5–44·4)	24·9 (16·9–32·7)	22·5 (15·5–29·9)	-1·8 (-2·6–-1·0)	-2·0 (-3·0–-1·1)	-0·9 (-2·1–0·0)
Diet high in trans fatty acids	71·3 (61·8–78·9)	71·9 (63·7–79·2)	63·3 (56·3–70·8)	0·0 (0·0–0·0)	-65·8 (-66·1–-65·3)	-97·1 (-97·6–-96·5)	-184·2 (-185·3–-183·2)
Diet high in sodium	23·8 (0·6–71·3)	28·7 (1·7–77·5)	32·2 (3·4–77·3)	32·6 (3·2–80·0)	1·0 (0·3–6·4)	0·6 (0·0–4·0)	0·1 (-2·5–2·0)
Intimate partner violence	27·3 (16·5–35·6)	27·8 (18·1–34·4)	23·1 (13·0–29·0)	22·2 (12·1–31·0)	-0·7 (-2·0–0·7)	-1·1 (-3·0–0·6)	-0·4 (-2·8–1·9)
Childhood sexual abuse and bullying	7·7 (4·6–12·9)	9·5 (5·8–15·8)	10·8 (6·6–17·9)	8·2 (5·2–13·0)	0·2 (-0·4–0·8)	-0·7 (-1·6–0·1)	-2·6 (-4·2–-1·0)
Childhood sexual abuse	5·6 (4·8–6·5)	6·4 (5·6–7·3)	7·4 (6·8–8·2)	6·8 (5·7–8·1)	0·7 (-0·2–1·4)	0·3 (-0·7–1·4)	-0·8 (-2·2–0·7)
Bullying victimization	7·9 (3·5–15·9)	9·9 (4·5–19·3)	11·2 (5·1–21·9)	7·7 (3·4–14·6)	-0·1 (-0·8–0·6)	-1·2 (-2·2–-0·2)	-3·4 (-5·3–-1·6)
Unsafe sex	--	--	--	--	--	--	--
Low physical activity	22·4 (16·9–28·8)	22·1 (17·2–27·4)	22·4 (17·7–27·4)	24·4 (18·8–30·8)	0·3 (-0·5–1·2)	0·5 (-0·6–1·6)	0·8 (-0·8–2·4)
**Metabolic risks**	**27·7 (24·4–30·9)**	**32·7 (28·6–35·7)**	**37·3 (33·0–39·9)**	**40·4 (35·4–43·4)**	**1·2 (0·9–1·5)**	**1·0 (0·7–1·3)**	**0·7 (0·2–1·2)**
High fasting plasma glucose	13·7 (10·2–16·4)	18·1 (13·5–21·3)	23·4 (17·2–28·1)	26·5 (19·8–31·5)	2·1 (1·5–2·7)	1·8 (1·1–2·6)	1·1 (0·0–2·2)
High LDL cholesterol	63·3 (44·6–86·3)	53·7 (37·1–73·8)	48·4 (32·8–67·7)	46·9 (31·6–65·7)	-1·0 (-1·2–-0·8)	-0·6 (-0·8–-0·5)	-0·3 (-0·5–0·0)
High systolic blood pressure	31·7 (21·4–44·3)	27·4 (19·5–37·2)	21·4 (14·2–30·1)	30·1 (19·1–43·2)	-0·2 (-1·1–0·8)	0·4 (-0·8–1·6)	3·1 (0·9–5·5)
High body-mass index	31·6 (26·6–35·8)	37·7 (32·5–41·7)	42·9 (37·1–46·4)	46·1 (39·5–49·8)	1·2 (0·9–1·5)	1·0 (0·6–1·3)	0·6 (0·2–1·1)
Low bone mineral density	20·3 (14·5–26·8)	19·5 (13·9–26·3)	19·1 (13·4–25·8)	18·9 (12·9–25·3)	-0·2 (-0·7–0·2)	-0·2 (-1·0–0·5)	-0·1 (-1·5–1·3)
Kidney dysfunction	3·1 (2·5–4·1)	3·2 (2·6–4·2)	3·2 (2·6–4·2)	3·3 (2·7–4·3)	0·2 (0·1–0·3)	0·1 (0·0–0·3)	0·4 (0·0–0·7)

Maine

Risk Names	SEV 1990	SEV 2000	SEV 2010	SEV 2021	Annualised rate of change 1990 to 2021	Annualised rate of change 2000 to 2021	Annualised rate of change 2010 to 2021
**All risk factors**	**26·9 (23·9–30·1)**	**26·2 (23·4–29·3)**	**27·0 (24·0–30·5)**	**26·8 (23·5–30·2)**	**0·0 (-0·3–0·3)**	**0·1 (-0·2–0·5)**	**-0·1 (-0·7–0·5)**
**Environmental/occupational risks**	**15·0 (9·9–25·5)**	**14·6 (9·4–24·7)**	**13·0 (8·2–21·7)**	**12·0 (7·5–20·8)**	**-0·7 (-1·3–-0·2)**	**-0·9 (-1·3–-0·6)**	**-0·7 (-1·2–-0·2)**
Unsafe water, sanitation, and handwashing	1·9 (1·0–3·0)	1·7 (0·7–2·7)	1·3 (0·4–2·3)	1·1 (0·4–2·1)	-1·6 (-3·8–0·2)	-1·9 (-5·1–0·7)	-1·5 (-5·5–2·6)
Unsafe water source	2·6 (1·0–5·1)	2·3 (0·9–4·6)	1·0 (0·4–2·1)	0·8 (0·3–1·6)	-3·8 (-6·3–-1·3)	-5·0 (-8·9–-1·7)	-1·8 (-7·6–4·5)
Unsafe sanitation	5·9 (3·1–10·0)	4·4 (2·3–7·9)	3·4 (1·8–6·3)	2·7 (1·4–4·8)	-2·5 (-4·8–-0·5)	-2·3 (-5·2–0·2)	-2·2 (-5·8–1·3)
No access to handwashing facility	1·7 (0·5–3·3)	1·7 (0·5–3·3)	1·7 (0·6–3·0)	1·5 (0·5–2·9)	-0·4 (-3·4–2·8)	-0·6 (-4·8–3·7)	-1·3 (-6·5–4·5)
Air pollution	9·9 (1·7–20·0)	8·1 (1·9–17·4)	3·6 (0·6–10·4)	2·5 (0·1–8·6)	-4·4 (-11·1–-2·1)	-5·6 (-13·5–-3·1)	-3·2 (-16·8–0·0)
Particulate matter pollution	5·2 (0·4–12·2)	3·8 (1·2–7·1)	2·1 (0·3–4·8)	0·8 (0·0–2·7)	-6·1 (-15·5–-0·9)	-7·6 (-18·1–-4·1)	-9·2 (-23·7–-4·2)
Ambient particulate matter pollution	7·9 (0·6–18·7)	5·8 (1·9–10·5)	3·2 (0·5–7·2)	1·2 (0·1–4·1)	-6·1 (-15·6–-0·9)	-7·6 (-18·1–-4·1)	-9·2 (-23·6–-4·2)
Household air pollution from solid fuels	0·0 (0·0–0·2)	0·0 (0·0–0·1)	0·0 (0·0–0·0)	0·0 (0·0–0·0)	-7·0 (-37·0–-9·1)	-8·2 (-36·9–-9·5)	-7·2 (-31·1–-6·2)
Ambient ozone pollution	16·0 (10·5–23·1)	14·5 (9·2–20·6)	10·2 (5·7–16·1)	3·4 (1·0–6·9)	-5·0 (-7·5–-3·5)	-6·9 (-10·6–-4·8)	-10·0 (-16·0–-6·4)
Ambient nitrogen dioxide pollution	29·7 (0·0–80·0)	26·0 (0·0–73·9)	8·9 (0·0–43·1)	10·2 (0·0–45·6)	-3·4 (-9·4–0·0)	-4·5 (-12·7–0·0)	1·3 (0·0–8·4)
Non-optimal temperature	27·9 (22·9–35·6)	26·6 (22·5–32·6)	28·0 (22·5–36·0)	27·5 (22·3–35·6)	0·0 (-0·3–0·2)	0·2 (-0·2–0·7)	-0·2 (-0·5–0·0)
High temperature	18·6 (9·5–29·8)	14·3 (6·7–24·8)	24·0 (13·3–36·6)	29·5 (17·9–43·3)	1·5 (0·5–2·5)	3·5 (1·8–5·4)	1·9 (0·5–3·4)
Low temperature	26·8 (23·1–31·5)	26·3 (22·8–31·0)	26·3 (22·6–31·2)	25·5 (21·9–30·3)	-0·2 (-0·2–-0·1)	-0·1 (-0·2–-0·1)	-0·3 (-0·4–-0·1)
Other environmental risks	24·9 (9·6–45·2)	23·7 (9·6–44·6)	22·2 (9·0–42·8)	20·3 (8·6–40·8)	-0·7 (-1·3–0·0)	-0·7 (-1·5–0·0)	-0·8 (-1·9–0·0)
Residential radon	32·5 (0·0–100·0)	32·5 (0·0–100·0)	32·5 (0·0–100·0)	32·5 (0·0–100·0)	0·0 (0·0–0·0)	0·0 (0·0–0·0)	0·0 (0·0–0·0)
Lead exposure	21·4 (0·0–27·1)	19·8 (0·0–24·7)	17·5 (0·0–21·7)	14·8 (0·0–18·3)	-1·2 (-1·7–0·0)	-1·4 (-1·9–0·0)	-1·5 (-2·4–0·0)
Occupational risks	3·2 (2·7–4·1)	3·3 (2·7–4·2)	3·0 (2·5–4·0)	3·0 (2·5–3·9)	-0·2 (-0·5–0·1)	-0·3 (-0·7–0·0)	0·0 (-0·4–0·5)
Occupational carcinogens	1·1 (0·6–2·0)	1·2 (0·6–2·2)	1·2 (0·6–2·2)	1·2 (0·6–2·2)	0·1 (-0·3–0·4)	-0·1 (-0·6–0·2)	0·1 (-0·5–0·7)
Occupational exposure to asbestos	6·7 (6·1–7·2)	7·0 (6·5–7·6)	6·4 (5·8–7·0)	6·0 (5·0–7·4)	-0·4 (-1·0–0·3)	-0·8 (-1·7–0·3)	-0·7 (-2·3–1·2)
Occupational exposure to arsenic	0·6 (0·0–1·3)	0·5 (0·0–1·3)	0·5 (0·0–1·2)	0·5 (0·0–1·2)	-0·4 (-0·8–0·1)	-0·4 (-1·0–0·2)	0·0 (-0·9–0·8)
Occupational exposure to benzene	1·1 (0·1–3·2)	1·3 (0·1–3·7)	1·3 (0·1–3·8)	1·3 (0·1–3·8)	0·5 (0·1–0·8)	0·2 (-0·3–0·6)	0·3 (-0·4–0·9)
Occupational exposure to beryllium	0·0 (0·0–0·0)	0·0 (0·0–0·0)	0·0 (0·0–0·0)	0·0 (0·0–0·0)	0·2 (0·0–0·4)	0·1 (-0·2–0·4)	0·4 (0·0–0·8)
Occupational exposure to cadmium	0·1 (0·1–0·1)	0·1 (0·1–0·1)	0·1 (0·1–0·1)	0·1 (0·1–0·1)	-0·3 (-0·7–0·1)	-0·3 (-0·9–0·2)	0·1 (-0·7–0·9)
Occupational exposure to chromium	0·2 (0·1–0·2)	0·2 (0·1–0·2)	0·1 (0·1–0·2)	0·2 (0·1–0·2)	-0·1 (-0·5–0·2)	-0·2 (-0·7–0·2)	0·1 (-0·5–0·8)
Occupational exposure to diesel engine exhaust	0·7 (0·6–0·7)	0·7 (0·7–0·7)	0·7 (0·6–0·7)	0·7 (0·7–0·8)	0·4 (0·0–0·7)	0·3 (-0·2–0·8)	0·6 (0·0–1·4)
Occupational exposure to formaldehyde	0·3 (0·3–0·4)	0·3 (0·3–0·3)	0·3 (0·3–0·3)	0·3 (0·3–0·3)	-0·3 (-0·7–0·1)	-0·4 (-0·9–0·1)	0·0 (-0·8–0·7)
Occupational exposure to nickel	0·4 (0·0–1·8)	0·4 (0·0–1·7)	0·4 (0·0–1·5)	0·4 (0·0–1·5)	-0·5 (-1·0–0·1)	-0·4 (-1·1–0·3)	0·0 (-1·1–1·0)
Occupational exposure to polycyclic aromatic hydrocarbons	0·3 (0·3–0·3)	0·3 (0·3–0·3)	0·3 (0·3–0·3)	0·3 (0·3–0·3)	-0·1 (-0·4–0·3)	-0·2 (-0·6–0·2)	0·1 (-0·5–0·7)
Occupational exposure to silica	3·8 (0·6–12·1)	3·5 (0·6–11·3)	3·2 (0·5–10·6)	3·3 (0·5–10·6)	-0·5 (-0·9–0·0)	-0·4 (-1·0–0·3)	0·1 (-0·8–1·1)
Occupational exposure to sulphuric acid	0·8 (0·2–2·7)	0·8 (0·2–2·7)	0·7 (0·1–2·5)	0·7 (0·2–2·4)	-0·3 (-0·7–0·1)	-0·3 (-0·9–0·2)	0·1 (-0·7–0·9)
Occupational exposure to trichloroethylene	0·1 (0·1–0·1)	0·1 (0·1–0·1)	0·1 (0·1–0·1)	0·1 (0·1–0·1)	0·0 (-0·2–0·3)	0·0 (-0·4–0·3)	0·3 (-0·3–0·9)
Occupational asthmagens	18·2 (15·8–20·9)	18·5 (16·1–21·0)	17·0 (14·8–19·1)	17·4 (15·3–19·7)	-0·2 (-0·5–0·2)	-0·3 (-0·8–0·2)	0·2 (-0·5–0·9)
Occupational particulate matter, gases, and fumes	7·3 (5·5–9·4)	7·0 (5·3–9·0)	6·5 (5·0–8·4)	6·0 (4·6–7·7)	-0·6 (-0·9–-0·3)	-0·7 (-1·1–-0·4)	-0·8 (-1·2–-0·4)
Occupational noise	7·2 (6·6–7·9)	7·2 (6·7–7·8)	7·1 (6·7–7·7)	6·9 (6·5–7·5)	-0·1 (-0·3–0·0)	-0·2 (-0·4–0·0)	-0·3 (-0·5–-0·1)
Occupational injuries	--	--	--	--	--	--	--
Occupational ergonomic factors	9·2 (7·7–11·2)	9·0 (7·7–10·9)	8·0 (6·8–9·7)	8·2 (6·8–10·1)	-0·4 (-0·8–0·1)	-0·4 (-1·1–0·1)	0·2 (-0·6–0·9)
**Behavioural risks**	**31·6 (28·4–35·6)**	**30·0 (26·6–33·9)**	**28·9 (25·4–33·1)**	**27·1 (23·6–31·2)**	**-0·5 (-0·9–-0·1)**	**-0·5 (-1·0–0·0)**	**-0·6 (-1·3–0·1)**
Child and maternal malnutrition	9·9 (6·6–13·6)	8·5 (5·7–11·5)	8·7 (5·9–11·8)	9·0 (6·2–12·7)	-0·3 (-1·2–0·5)	0·3 (-0·9–1·5)	0·3 (-1·4–2·2)
Suboptimal breastfeeding	68·8 (66·0–71·4)	66·6 (63·9–69·2)	62·7 (60·1–65·7)	62·1 (59·4–65·3)	-0·3 (-0·5–-0·2)	-0·3 (-0·5–-0·1)	-0·1 (-0·4–0·2)
Non-exclusive breastfeeding	64·1 (56·3–71·9)	59·4 (52·1–66·2)	48·4 (41·5–55·2)	46·0 (38·3–53·7)	-1·1 (-1·6–-0·5)	-1·2 (-1·9–-0·6)	-0·5 (-1·4–0·5)
Discontinued breastfeeding	88·7 (86·6–91·1)	86·3 (83·3–89·2)	84·3 (81·1–87·7)	84·5 (81·2–88·0)	-0·2 (-0·3–0·0)	-0·1 (-0·3–0·1)	0·0 (-0·3–0·3)
Child growth failure	1·2 (0·4–2·8)	1·1 (0·4–2·6)	1·0 (0·3–2·1)	1·0 (0·3–2·0)	-0·8 (-1·1–-0·4)	-0·9 (-1·4–-0·3)	-0·5 (-1·3–0·3)
Child underweight	2·5 (1·6–3·6)	2·4 (1·6–3·3)	2·1 (1·3–2·8)	2·0 (1·2–2·7)	-0·8 (-1·3–-0·3)	-0·9 (-1·6–-0·2)	-0·4 (-1·4–0·6)
Child wasting	0·8 (0·5–1·3)	0·8 (0·5–1·2)	0·8 (0·5–1·2)	0·8 (0·5–1·2)	-0·2 (-0·5–0·2)	0·0 (-0·4–0·4)	-0·1 (-0·8–0·6)
Child stunting	3·5 (2·7–4·4)	3·3 (2·6–4·1)	2·9 (2·2–3·7)	2·7 (2·1–3·4)	-0·9 (-1·4–-0·3)	-1·0 (-1·8–-0·1)	-0·7 (-1·9–0·5)
Low birth weight and short gestation	14·6 (12·8–16·7)	15·9 (13·8–18·3)	18·6 (16·1–20·9)	19·4 (17·1–22·4)	0·9 (0·7–1·2)	1·0 (0·6–1·3)	0·4 (-0·3–1·2)
Short gestation	27·8 (24·4–31·4)	29·4 (25·7–34·0)	32·3 (27·8–37·1)	33·0 (28·5–38·2)	0·6 (0·3–0·8)	0·5 (0·1–0·9)	0·2 (-0·7–1·1)
Low birth weight	12·3 (11·1–13·7)	13·0 (11·7–14·5)	15·1 (13·6–16·8)	15·8 (14·5–17·5)	0·8 (0·6–1·1)	0·9 (0·6–1·3)	0·4 (-0·2–1·2)

Iron deficiency	3·5 (2·4–4·7)	3·0 (2·1–4·0)	3·1 (2·2–4·2)	3·2 (2·3–4·4)	-0·3 (-1·1–0·5)	0·3 (-0·9–1·4)	0·2 (-1·5–1·9)
Vitamin A deficiency	1·0 (0·0–2·0)	1·2 (0·0–2·4)	0·8 (0·0–1·6)	0·5 (0·0–1·0)	-2·3 (-4·5–0·0)	-4·5 (-7·1–0·0)	-4·2 (-8·1–0·0)
Zinc deficiency	2·0 (0·0–8·3)	1·2 (0·0–5·1)	1·0 (0·0–4·6)	1·0 (0·0–4·5)	-2·1 (-47·8–0·0)	-0·6 (-3·1–2·7)	0·0 (-3·1–5·7)
Tobacco	43·4 (41·9–44·9)	38·5 (37·0–39·7)	34·0 (32·9–35·2)	30·8 (28·8–32·5)	-1·1 (-1·3–-0·9)	-1·1 (-1·4–-0·8)	-0·9 (-1·5–-0·4)
Smoking	42·0 (39·4–44·1)	36·4 (34·1–38·4)	31·8 (30·0–33·8)	28·5 (26·2–31·3)	-1·2 (-1·5–-0·9)	-1·2 (-1·5–-0·7)	-1·0 (-1·6–-0·2)
Chewing tobacco	1·1 (0·8–1·4)	1·2 (0·9–1·6)	1·3 (0·9–1·8)	1·3 (0·9–1·8)	0·6 (-0·7–1·8)	0·4 (-1·3–2·3)	0·2 (-2·6–2·9)
Second-hand smoke	34·3 (32·4–35·7)	30·3 (28·6–31·5)	26·8 (25·2–27·8)	24·4 (22·4–26·1)	-1·1 (-1·3–-0·9)	-1·0 (-1·4–-0·7)	-0·9 (-1·5–-0·3)
High alcohol use	28·4 (20·4–40·2)	27·9 (20·4–39·2)	29·0 (21·3–39·7)	28·7 (19·6–39·7)	0·0 (-1·1–1·1)	0·1 (-1·3–1·5)	-0·1 (-2·1–1·7)
Drug use	0·7 (0·4–1·2)	0·9 (0·7–1·1)	1·8 (1·4–2·1)	5·3 (3·4–7·1)	6·5 (3·6–8·8)	8·5 (5·8–10·1)	10·0 (7·6–11·2)
Dietary risks	35·5 (26·2–46·8)	38·1 (28·6–49·7)	40·7 (31·2–52·7)	41·0 (31·8–53·1)	0·5 (0·2–0·8)	0·4 (0·0–0·7)	0·1 (-0·4–0·6)
Diet low in fruits	34·6 (30·6–37·9)	31·4 (27·1–34·2)	31·8 (27·2–34·7)	33·7 (28·7–38·0)	-0·1 (-0·5–0·3)	0·3 (-0·3–0·9)	0·5 (-0·4–1·4)
Diet low in vegetables	22·3 (13·1–29·4)	21·8 (13·0–28·5)	24·5 (14·3–30·8)	24·8 (14·6–32·0)	0·3 (-0·2–0·9)	0·6 (-0·1–1·3)	0·1 (-0·8–1·1)
Diet low in legumes	38·9 (0·0–48·5)	39·1 (0·0–48·8)	42·9 (0·0–53·2)	43·6 (0·0–55·2)	0·4 (0·0–0·7)	0·5 (0·0–1·0)	0·1 (-0·6–0·9)
Diet low in whole grains	45·1 (37·4–51·5)	48·2 (39·8–54·6)	50·2 (41·9–57·1)	51·1 (42·8–58·1)	0·4 (0·1–0·7)	0·3 (-0·1–0·7)	0·2 (-0·4–0·7)
Diet low in nuts and seeds	13·8 (9·7–17·5)	8·2 (5·5–11·7)	5·7 (3·6–8·7)	6·2 (3·5–9·6)	-2·6 (-4·3–-1·2)	-1·4 (-4·2–0·9)	0·7 (-3·3–4·0)
Diet low in milk	37·4 (33·1–42·0)	34·8 (30·5–39·4)	32·9 (28·2–37·8)	32·5 (27·5–37·9)	-0·5 (-1·0–0·0)	-0·3 (-0·9–0·3)	-0·1 (-1·0–0·8)
Diet high in red meat	49·0 (0·0–67·8)	49·3 (0·0–67·5)	49·8 (0·0–68·5)	50·2 (0·0–70·6)	0·1 (-0·5–0·6)	0·1 (-0·6–0·8)	0·1 (-0·9–1·2)
Diet high in processed meat	46·2 (36·9–52·3)	57·5 (46·4–64·7)	65·3 (53·6–73·4)	63·7 (53·4–71·9)	1·0 (0·7–1·5)	0·5 (-0·1–1·0)	-0·2 (-1·0–0·6)
Diet high in sugar-sweetened beverages	34·4 (25·8–42·5)	48·4 (38·6–57·4)	54·9 (44·9–64·1)	54·8 (43·1–65·5)	1·5 (0·7–2·4)	0·6 (-0·4–1·5)	0·0 (-1·4–1·2)
Diet low in fibre	40·8 (21·6–48·1)	31·3 (16·7–38·7)	27·4 (15·9–33·8)	24·2 (14·2–31·2)	-1·7 (-2·6–-0·8)	-1·2 (-2·6–0·0)	-1·1 (-3·1–0·7)
Diet low in calcium	5·9 (4·8–7·3)	5·0 (4·1–6·1)	5·2 (4·2–6·4)	5·1 (4·1–6·3)	-0·5 (-0·7–-0·2)	0·1 (-0·2–0·4)	-0·1 (-0·5–0·2)
Diet low in seafood omega-3 fatty acids	37·9 (28·4–48·0)	45·0 (34·9–55·4)	47·3 (36·8–57·5)	42·6 (31·8–53·2)	0·4 (-0·1–0·8)	-0·3 (-0·8–0·3)	-1·0 (-1·8–-0·2)
Diet low in omega-6 polyunsaturated fatty acids	37·5 (24·1–47·1)	32·7 (21·9–41·9)	23·9 (16·9–30·9)	22·5 (15·8–29·8)	-1·6 (-2·4–-0·9)	-1·8 (-2·6–-0·9)	-0·5 (-1·6–0·3)
Diet high in trans fatty acids	71·4 (62·8–78·9)	71·7 (64·2–78·9)	63·3 (55·5–70·2)	0·0 (0·0–0·0)	-65·8 (-66·1–-65·3)	-97·1 (-97·6–-96·6)	-184·2 (-185·2–-183·0)
Diet high in sodium	22·9 (0·6–66·9)	27·6 (1·9–73·8)	30·7 (3·6–75·0)	31·0 (3·1–76·6)	1·0 (0·2–6·1)	0·6 (-0·1–3·9)	0·1 (-2·5–1·8)
Intimate partner violence	29·1 (13·9–37·2)	28·8 (16·8–36·2)	25·9 (18·5–31·5)	24·4 (14·0–33·3)	-0·6 (-1·9–0·7)	-0·8 (-2·6–0·8)	-0·6 (-2·9–1·3)
Childhood sexual abuse and bullying	9·2 (6·0–14·3)	11·2 (7·3–17·3)	12·7 (8·2–19·7)	10·7 (7·1–16·2)	0·5 (-0·1–1·1)	-0·2 (-1·0–0·6)	-1·5 (-3·0–0·1)
Childhood sexual abuse	9·0 (7·7–10·7)	10·3 (8·9–12·0)	11·6 (10·2–13·6)	11·0 (9·0–13·4)	0·6 (-0·1–1·4)	0·3 (-0·7–1·3)	-0·5 (-2·1–1·0)
Bullying victimization	8·0 (3·5–16·0)	10·0 (4·6–19·5)	11·4 (5·1–22·1)	8·9 (3·8–16·8)	0·4 (-0·4–1·2)	-0·6 (-1·6–0·6)	-2·2 (-4·2–-0·1)
Unsafe sex	--	--	--	--	--	--	--
Low physical activity	18·5 (13·5–24·2)	17·0 (12·7–21·3)	17·8 (13·5–22·7)	20·8 (16·2–27·2)	0·4 (-0·6–1·4)	1·0 (-0·2–2·2)	1·4 (-0·5–3·3)
**Metabolic risks**	**26·7 (23·2–29·9)**	**30·8 (27·2–33·6)**	**34·4 (30·4–37·4)**	**37·5 (33·2–40·7)**	**1·1 (0·8–1·4)**	**0·9 (0·6–1·3)**	**0·8 (0·3–1·3)**
High fasting plasma glucose	13·0 (9·6–15·5)	16·7 (12·4–19·9)	21·4 (15·6–25·6)	25·0 (18·6–30·2)	2·1 (1·4–2·9)	1·9 (1·0–2·8)	1·4 (0·1–2·7)
High LDL cholesterol	63·8 (45·1–87·3)	54·0 (37·3–74·7)	48·3 (32·5–67·2)	46·9 (31·6–65·3)	-1·0 (-1·2–-0·8)	-0·7 (-0·9–-0·5)	-0·3 (-0·5–0·0)
High systolic blood pressure	28·7 (18·6–39·6)	24·4 (17·1–33·7)	17·8 (11·5–25·7)	25·3 (16·0–35·4)	-0·4 (-1·3–0·4)	0·2 (-1·0–1·3)	3·2 (1·1–5·7)
High body-mass index	30·0 (25·3–34·4)	35·1 (30·0–38·9)	39·2 (33·7–43·0)	42·2 (36·1–46·1)	1·1 (0·8–1·4)	0·9 (0·6–1·2)	0·7 (0·2–1·1)
Low bone mineral density	21·8 (15·5–28·8)	19·7 (13·7–26·9)	20·6 (14·7–27·5)	22·3 (15·8–29·5)	0·1 (-0·4–0·6)	0·6 (-0·2–1·4)	0·7 (-0·7–2·1)
Kidney dysfunction	2·9 (2·3–3·8)	3·0 (2·4–3·9)	2·9 (2·3–3·9)	3·0 (2·5–4·0)	0·2 (0·1–0·3)	0·1 (0·0–0·3)	0·4 (0·1–0·7)

Maryland

Risk Names	SEV 1990	SEV 2000	SEV 2010	SEV 2021	Annualised rate of change 1990 to 2021	Annualised rate of change 2000 to 2021	Annualised rate of change 2010 to 2021
**All risk factors**	**28·3 (25·4–31·4)**	**28·4 (25·5–31·5)**	**29·1 (26·2–32·3)**	**28·3 (25·1–31·7)**	**0·0 (-0·3–0·3)**	**0·0 (-0·4–0·4)**	**-0·3 (-0·8–0·3)**
**Environmental/occupational risks**	**19·5 (12·8–30·1)**	**19·1 (13·4–29·7)**	**16·6 (11·2–27·0)**	**14·1 (9·1–23·4)**	**-1·0 (-1·8–-0·3)**	**-1·4 (-1·9–-0·8)**	**-1·5 (-2·1–-0·9)**
Unsafe water, sanitation, and handwashing	1·6 (0·8–2·6)	1·5 (0·7–2·3)	1·2 (0·4–2·0)	1·0 (0·3–1·8)	-1·6 (-4·0–0·5)	-2·0 (-5·1–0·7)	-1·5 (-5·4–2·3)
Unsafe water source	2·3 (0·9–4·7)	2·1 (0·8–4·1)	0·9 (0·4–1·7)	0·7 (0·3–1·3)	-3·8 (-6·3–-1·2)	-5·1 (-8·8–-1·3)	-1·7 (-6·9–3·8)
Unsafe sanitation	4·8 (2·5–7·8)	3·7 (1·9–6·2)	2·7 (1·4–4·8)	2·2 (1·1–3·9)	-2·5 (-4·9–-0·1)	-2·4 (-5·2–0·3)	-2·0 (-5·4–1·3)
No access to handwashing facility	1·5 (0·5–2·8)	1·5 (0·6–2·9)	1·5 (0·5–2·9)	1·3 (0·4–2·6)	-0·4 (-3·2–2·7)	-0·7 (-4·3–3·3)	-1·3 (-6·3–4·3)
Air pollution	28·3 (9·6–39·7)	26·0 (10·4–32·6)	15·3 (6·2–25·8)	7·6 (2·7–15·9)	-4·3 (-7·1–-1·8)	-5·9 (-9·6–-3·1)	-6·4 (-10·8–-3·7)
Particulate matter pollution	15·4 (6·4–26·2)	13·4 (9·9–17·6)	8·7 (5·6–12·4)	4·9 (2·3–8·1)	-3·7 (-6·2–-1·2)	-4·8 (-7·3–-3·4)	-5·1 (-9·1–-3·3)
Ambient particulate matter pollution	24·1 (10·2–40·5)	20·8 (15·9–26·5)	13·3 (8·8–18·5)	7·6 (3·5–12·2)	-3·7 (-6·3–-1·2)	-4·8 (-7·4–-3·5)	-5·2 (-9·2–-3·3)
Household air pollution from solid fuels	0·0 (0·0–0·0)	0·0 (0·0–0·0)	0·0 (0·0–0·0)	0·0 (0·0–0·0)	-3·5 (-39·7–-6·7)	-5·6 (-39·6–-6·3)	-4·0 (-25·7–-3·4)
Ambient ozone pollution	31·6 (24·3–40·7)	27·0 (20·2–35·8)	21·9 (15·4–29·0)	16·2 (10·3–23·4)	-2·2 (-2·9–-1·6)	-2·4 (-3·5–-1·6)	-2·7 (-4·4–-1·2)
Ambient nitrogen dioxide pollution	82·1 (0·0–100·0)	79·5 (0·0–100·0)	42·4 (0·0–99·9)	17·1 (0·0–61·2)	-5·1 (-14·8–0·0)	-7·3 (-21·0–0·0)	-8·2 (-24·9–0·0)
Non-optimal temperature	27·6 (21·4–37·7)	27·4 (22·2–35·9)	33·7 (26·3–45·2)	29·6 (23·4–39·7)	0·2 (0·0–0·5)	0·4 (0·0–0·8)	-1·2 (-1·7–-0·7)
High temperature	22·2 (12·8–34·3)	18·3 (10·1–29·9)	36·6 (25·2–49·1)	27·3 (17·1–38·1)	0·7 (0·2–1·3)	1·9 (0·9–3·1)	-2·7 (-4·0–-1·6)
Low temperature	25·6 (22·0–30·0)	26·6 (23·0–30·6)	28·9 (25·5–32·9)	26·5 (23·0–30·7)	0·1 (0·0–0·2)	0·0 (-0·2–0·2)	-0·8 (-1·0–-0·5)
Other environmental risks	27·9 (7·8–47·0)	26·0 (7·8–45·0)	23·3 (7·8–42·0)	20·5 (7·9–39·2)	-1·0 (-1·7–0·0)	-1·1 (-2·0–0·0)	-1·1 (-2·2–0·0)
Residential radon	26·8 (0·0–85·5)	26·8 (0·0–85·5)	26·8 (0·0–85·5)	26·8 (0·0–85·5)	0·0 (0·0–0·0)	0·0 (0·0–0·0)	0·0 (0·0–0·0)
Lead exposure	28·3 (0·0–35·7)	25·5 (0·0–32·0)	21·6 (0·0–27·2)	17·7 (0·0–22·1)	-1·5 (-2·0–0·0)	-1·8 (-2·3–0·0)	-1·9 (-2·6–0·0)
Occupational risks	2·7 (2·2–3·5)	2·8 (2·3–3·8)	2·7 (2·2–3·7)	2·7 (2·2–3·7)	0·0 (-0·2–0·3)	-0·2 (-0·5–0·2)	0·1 (-0·4–0·6)
Occupational carcinogens	1·0 (0·5–1·8)	1·1 (0·5–2·1)	1·0 (0·4–2·0)	1·0 (0·4–2·0)	0·2 (-0·3–0·5)	-0·2 (-0·7–0·2)	0·1 (-0·6–0·6)
Occupational exposure to asbestos	4·6 (4·1–5·1)	4·9 (4·4–5·4)	3·8 (3·4–4·2)	3·5 (2·7–4·4)	-0·9 (-1·7–-0·1)	-1·6 (-2·7–-0·4)	-0·7 (-2·8–1·5)
Occupational exposure to arsenic	0·5 (0·0–1·1)	0·5 (0·0–1·1)	0·4 (0·0–1·1)	0·5 (0·0–1·1)	0·0 (-0·4–0·5)	-0·1 (-0·7–0·5)	0·2 (-0·7–1·0)
Occupational exposure to benzene	1·1 (0·1–3·2)	1·3 (0·1–3·7)	1·3 (0·1–3·6)	1·3 (0·1–3·7)	0·4 (0·0–0·8)	0·1 (-0·4–0·6)	0·1 (-0·6–0·8)
Occupational exposure to beryllium	0·0 (0·0–0·0)	0·0 (0·0–0·0)	0·0 (0·0–0·0)	0·0 (0·0–0·0)	0·3 (0·0–0·5)	0·1 (-0·2–0·5)	0·4 (-0·1–0·8)
Occupational exposure to cadmium	0·1 (0·0–0·1)	0·1 (0·1–0·1)	0·1 (0·1–0·1)	0·1 (0·1–0·1)	0·1 (-0·3–0·5)	0·0 (-0·6–0·5)	0·2 (-0·6–1·0)
Occupational exposure to chromium	0·1 (0·1–0·1)	0·1 (0·1–0·2)	0·1 (0·1–0·1)	0·1 (0·1–0·2)	0·2 (-0·2–0·5)	0·0 (-0·4–0·5)	0·3 (-0·5–0·9)
Occupational exposure to diesel engine exhaust	0·7 (0·6–0·7)	0·7 (0·7–0·8)	0·7 (0·7–0·8)	0·8 (0·7–0·9)	0·5 (0·1–0·8)	0·4 (-0·2–0·9)	0·6 (-0·1–1·3)
Occupational exposure to formaldehyde	0·3 (0·2–0·3)	0·3 (0·3–0·3)	0·3 (0·3–0·3)	0·3 (0·2–0·3)	0·0 (-0·3–0·4)	-0·1 (-0·7–0·4)	0·1 (-0·7–0·8)
Occupational exposure to nickel	0·4 (0·0–1·4)	0·4 (0·0–1·4)	0·3 (0·0–1·3)	0·3 (0·0–1·4)	-0·1 (-0·6–0·5)	-0·1 (-0·8–0·6)	0·2 (-0·9–1·3)
Occupational exposure to polycyclic aromatic hydrocarbons	0·3 (0·3–0·3)	0·3 (0·3–0·3)	0·3 (0·3–0·3)	0·3 (0·3–0·3)	0·2 (-0·1–0·6)	0·0 (-0·4–0·5)	0·2 (-0·5–0·8)
Occupational exposure to silica	3·0 (0·5–9·9)	3·0 (0·5–9·5)	2·9 (0·5–9·0)	3·0 (0·5–9·2)	-0·1 (-0·5–0·5)	0·0 (-0·6–0·6)	0·3 (-0·6–1·3)
Occupational exposure to sulphuric acid	0·7 (0·1–2·2)	0·7 (0·1–2·2)	0·7 (0·1–2·2)	0·7 (0·1–2·2)	0·1 (-0·3–0·5)	0·0 (-0·6–0·5)	0·3 (-0·5–1·1)
Occupational exposure to trichloroethylene	0·1 (0·1–0·1)	0·1 (0·1–0·1)	0·1 (0·1–0·1)	0·1 (0·1–0·1)	0·3 (0·0–0·6)	0·2 (-0·2–0·6)	0·4 (-0·2–0·9)
Occupational asthmagens	15·9 (13·8–17·9)	16·6 (14·6–18·9)	15·9 (14·0–18·0)	16·3 (14·2–18·8)	0·1 (-0·3–0·4)	-0·1 (-0·6–0·4)	0·2 (-0·5–0·9)
Occupational particulate matter, gases, and fumes	5·6 (4·2–7·2)	5·4 (4·2–7·0)	5·2 (3·9–6·7)	4·9 (3·8–6·5)	-0·4 (-0·7–-0·1)	-0·4 (-0·8–-0·1)	-0·5 (-0·8–-0·1)
Occupational noise	6·1 (5·6–6·7)	6·2 (5·7–6·8)	6·2 (5·8–6·8)	6·2 (5·8–6·7)	0·0 (-0·1–0·2)	0·0 (-0·2–0·2)	-0·1 (-0·3–0·1)
Occupational injuries	--	--	--	--	--	--	--
Occupational ergonomic factors	8·1 (6·7–9·9)	8·2 (6·9–10·0)	7·5 (6·4–9·1)	7·7 (6·4–9·4)	-0·2 (-0·6–0·3)	-0·3 (-0·8–0·2)	0·2 (-0·5–0·8)
**Behavioural risks**	**31·4 (28·1–35·5)**	**29·8 (26·7–33·5)**	**27·9 (24·7–31·6)**	**25·3 (22·0–29·0)**	**-0·7 (-1·1–-0·4)**	**-0·8 (-1·3–-0·3)**	**-0·9 (-1·6–-0·3)**
Child and maternal malnutrition	10·5 (7·1–14·8)	9·1 (6·1–12·4)	9·1 (6·3–12·4)	9·3 (6·4–13·3)	-0·4 (-1·3–0·4)	0·1 (-1·1–1·3)	0·2 (-1·6–2·1)
Suboptimal breastfeeding	68·7 (65·8–71·3)	66·5 (64·1–69·2)	62·7 (60·2–65·5)	62·1 (59·2–65·2)	-0·3 (-0·5–-0·2)	-0·3 (-0·5–-0·1)	-0·1 (-0·4–0·2)
Non-exclusive breastfeeding	63·9 (55·8–71·6)	59·2 (52·1–65·8)	48·3 (41·6–55·1)	46·1 (38·9–53·0)	-1·1 (-1·6–-0·6)	-1·2 (-1·8–-0·6)	-0·4 (-1·4–0·5)
Discontinued breastfeeding	88·6 (86·1–91·1)	86·2 (83·7–89·0)	84·2 (81·2–87·5)	84·5 (80·9–87·8)	-0·2 (-0·3–0·0)	-0·1 (-0·3–0·1)	0·0 (-0·3–0·3)
Child growth failure	1·2 (0·4–2·8)	1·2 (0·4–2·7)	1·0 (0·3–2·2)	0·9 (0·3–2·1)	-0·9 (-1·3–-0·6)	-1·1 (-1·7–-0·6)	-0·5 (-1·2–0·3)
Child underweight	2·6 (1·7–3·6)	2·5 (1·6–3·3)	2·0 (1·3–2·7)	1·9 (1·1–2·6)	-1·0 (-1·5–-0·5)	-1·2 (-1·9–-0·5)	-0·4 (-1·5–0·7)
Child wasting	0·8 (0·5–1·3)	0·8 (0·5–1·2)	0·8 (0·5–1·2)	0·8 (0·5–1·2)	-0·3 (-0·6–0·0)	-0·2 (-0·6–0·3)	-0·1 (-0·8–0·6)
Child stunting	3·6 (2·9–4·5)	3·4 (2·7–4·3)	2·8 (2·2–3·6)	2·6 (2·1–3·4)	-1·0 (-1·6–-0·5)	-1·2 (-2·0–-0·4)	-0·7 (-1·8–0·6)
Low birth weight and short gestation	20·6 (17·9–23·5)	22·2 (19·3–25·3)	23·2 (20·1–26·3)	21·9 (19·0–24·7)	0·2 (0·0–0·5)	-0·1 (-0·5–0·3)	-0·5 (-1·3–0·2)
Short gestation	37·9 (33·0–42·7)	39·8 (34·7–45·0)	39·5 (34·5–44·9)	37·5 (32·7–42·8)	0·0 (-0·3–0·3)	-0·3 (-0·7–0·2)	-0·5 (-1·4–0·4)
Low birth weight	15·0 (13·7–16·6)	16·2 (14·6–17·8)	17·4 (15·8–19·0)	16·5 (15·1–18·2)	0·3 (0·1–0·5)	0·1 (-0·3–0·5)	-0·5 (-1·1–0·2)

Iron deficiency	3·8 (2·6–5·2)	3·3 (2·3–4·4)	3·3 (2·4–4·4)	3·3 (2·4–4·7)	-0·4 (-1·2–0·4)	0·1 (-1·1–1·1)	0·1 (-1·6–1·9)
Vitamin A deficiency	0·8 (0·0–1·6)	1·0 (0·0–2·2)	0·6 (0·0–1·2)	0·3 (0·0–0·8)	-2·6 (-4·9–0·0)	-5·1 (-7·6–0·0)	-4·4 (-8·4–0·0)
Zinc deficiency	1·6 (0·0–7·0)	1·0 (0·0–4·0)	0·8 (0·0–3·5)	0·9 (0·0–3·6)	-2·0 (-48·2–1·6)	-0·6 (-6·8–3·1)	0·3 (-4·4–5·7)
Tobacco	38·5 (36·8–40·0)	32·6 (31·3–33·9)	25·8 (24·9–26·8)	22·4 (20·8–24·2)	-1·7 (-2·0–-1·5)	-1·8 (-2·1–-1·4)	-1·3 (-1·9–-0·7)
Smoking	35·2 (32·6–37·5)	29·9 (27·8–31·9)	23·3 (22·1–24·9)	19·7 (18·0–21·6)	-1·9 (-2·2–-1·6)	-2·0 (-2·4–-1·6)	-1·6 (-2·3–-0·8)
Chewing tobacco	0·7 (0·6–0·9)	0·9 (0·7–1·2)	1·0 (0·8–1·4)	1·1 (0·8–1·5)	1·3 (-0·1–2·5)	0·9 (-0·9–2·4)	0·4 (-2·1–3·0)
Second-hand smoke	32·2 (30·2–33·3)	27·1 (25·5–28·2)	21·8 (20·3–22·8)	19·4 (17·7–21·1)	-1·6 (-1·9–-1·4)	-1·6 (-1·9–-1·2)	-1·0 (-1·6–-0·3)
High alcohol use	29·5 (21·7–40·5)	29·1 (21·2–39·9)	30·7 (23·7–40·9)	30·5 (22·2–40·2)	0·1 (-0·9–1·1)	0·2 (-1·0–1·5)	-0·1 (-1·7–1·4)
Drug use	0·8 (0·4–1·4)	1·3 (1·1–1·5)	2·3 (1·7–2·9)	3·2 (2·4–4·0)	4·6 (2·0–7·1)	4·4 (3·0–5·4)	3·2 (2·1–4·1)
Dietary risks	35·3 (26·1–46·7)	34·9 (26·1–47·2)	36·0 (26·4–48·9)	36·3 (26·8–49·1)	0·1 (-0·3–0·4)	0·2 (-0·2–0·6)	0·1 (-0·4–0·7)
Diet low in fruits	37·4 (32·5–41·0)	33·4 (30·0–36·3)	31·8 (28·3–34·6)	33·7 (29·0–38·0)	-0·3 (-0·7–0·0)	0·0 (-0·5–0·6)	0·5 (-0·4–1·5)
Diet low in vegetables	23·4 (14·5–31·7)	9·9 (5·2–15·5)	5·9 (3·5–8·4)	6·6 (3·9–9·7)	-4·1 (-5·0–-3·2)	-2·0 (-3·6–-0·1)	0·9 (-1·3–3·3)
Diet low in legumes	36·6 (0·0–46·3)	36·8 (0·0–46·4)	40·3 (0·0–50·9)	41·1 (0·0–51·7)	0·4 (0·0–0·7)	0·5 (0·0–1·0)	0·2 (-0·6–0·9)
Diet low in whole grains	44·5 (37·4–50·5)	47·7 (39·7–54·1)	49·4 (41·0–56·8)	49·9 (41·6–57·2)	0·4 (0·1–0·6)	0·2 (-0·2–0·6)	0·1 (-0·5–0·6)
Diet low in nuts and seeds	10·3 (7·3–14·4)	5·7 (3·5–8·2)	3·6 (2·1–5·7)	4·1 (2·1–6·8)	-2·9 (-5·1–-1·1)	-1·5 (-4·6–1·2)	1·2 (-3·1–5·3)
Diet low in milk	34·0 (29·7–38·7)	31·5 (27·1–35·7)	29·4 (25·1–34·2)	29·3 (24·5–34·3)	-0·5 (-1·0–0·0)	-0·3 (-1·0–0·4)	0·0 (-0·9–1·0)
Diet high in red meat	45·7 (0·0–64·0)	47·3 (0·0–66·5)	48·8 (0·0–68·1)	49·2 (0·0–68·0)	0·2 (-0·3–0·9)	0·2 (-0·5–1·0)	0·1 (-0·9–1·3)
Diet high in processed meat	40·6 (32·4–45·7)	51·1 (41·3–57·5)	58·3 (46·3–65·7)	56·6 (46·0–65·2)	1·1 (0·6–1·5)	0·5 (-0·1–1·1)	-0·3 (-1·2–0·6)
Diet high in sugar-sweetened beverages	39·0 (30·6–47·0)	53·6 (42·6–62·1)	60·6 (48·8–69·2)	59·7 (48·0–69·9)	1·4 (0·7–2·1)	0·5 (-0·3–1·5)	-0·1 (-1·3–1·2)
Diet low in fibre	35·7 (20·4–42·5)	26·7 (15·2–32·5)	22·5 (12·7–28·3)	19·8 (11·0–26·0)	-1·9 (-3·0–-1·0)	-1·4 (-2·8–-0·2)	-1·2 (-3·1–0·7)
Diet low in calcium	5·2 (4·2–6·4)	4·5 (3·6–5·5)	4·6 (3·7–5·7)	4·6 (3·7–5·6)	-0·4 (-0·6–-0·2)	0·1 (-0·2–0·3)	-0·1 (-0·4–0·2)
Diet low in seafood omega-3 fatty acids	32·6 (23·8–41·5)	40·1 (30·6–49·6)	42·0 (32·6–51·8)	37·2 (27·2–47·1)	0·4 (0·0–1·0)	-0·3 (-1·0–0·2)	-1·1 (-2·0–-0·3)
Diet low in omega-6 polyunsaturated fatty acids	31·9 (21·1–41·1)	27·9 (19·3–36·0)	20·4 (14·2–26·3)	19·2 (13·2–25·0)	-1·6 (-2·4–-0·9)	-1·8 (-2·8–-1·0)	-0·5 (-1·4–0·3)
Diet high in trans fatty acids	71·3 (64·1–79·1)	71·8 (63·2–79·1)	63·3 (55·8–70·9)	0·0 (0·0–0·0)	-65·8 (-66·1–-65·4)	-97·1 (-97·6–-96·5)	-184·2 (-185·3–-183·1)
Diet high in sodium	23·5 (0·5–68·9)	28·3 (2·0–76·4)	31·3 (3·4–78·2)	31·9 (3·2–78·9)	1·0 (0·2–6·5)	0·6 (-0·1–3·8)	0·2 (-2·5–1·9)
Intimate partner violence	32·2 (17·3–40·3)	32·2 (19·1–39·6)	29·3 (18·8–35·8)	27·3 (16·0–37·3)	-0·5 (-1·8–0·7)	-0·8 (-2·5–0·8)	-0·6 (-2·9–1·3)
Childhood sexual abuse and bullying	18·5 (10·7–28·4)	21·4 (13·7–30·7)	23·1 (15·2–31·4)	16·2 (10·9–22·9)	-0·4 (-1·5–0·6)	-1·3 (-2·5–-0·2)	-3·2 (-5·2–-1·3)
Childhood sexual abuse	10·0 (8·5–11·9)	11·3 (9·8–13·3)	13·0 (11·4–14·9)	11·7 (9·6–14·2)	0·5 (-0·2–1·3)	0·2 (-0·8–1·1)	-1·0 (-2·4–0·7)
Bullying victimization	20·8 (9·4–34·9)	24·0 (12·5–36·9)	25·4 (14·4–37·0)	16·2 (9·0–25·2)	-0·8 (-2·1–0·6)	-1·9 (-3·3–-0·3)	-4·1 (-6·4–-1·8)
Unsafe sex	--	--	--	--	--	--	--
Low physical activity	20·3 (15·1–27·0)	19·4 (15·0–24·5)	20·5 (15·8–25·5)	23·2 (17·5–29·2)	0·4 (-0·5–1·3)	0·8 (-0·4–2·1)	1·1 (-0·6–2·9)
**Metabolic risks**	**26·1 (22·7–29·3)**	**31·1 (27·5–34·0)**	**34·6 (30·7–37·4)**	**37·6 (33·1–40·4)**	**1·2 (0·9–1·5)**	**0·9 (0·6–1·2)**	**0·8 (0·3–1·2)**
High fasting plasma glucose	12·8 (9·5–15·1)	16·5 (12·8–19·4)	21·5 (16·0–25·3)	25·0 (18·8–30·4)	2·2 (1·5–2·8)	2·0 (1·1–2·8)	1·4 (0·3–2·6)
High LDL cholesterol	63·8 (45·0–86·5)	53·8 (37·1–74·5)	48·2 (32·8–67·4)	46·6 (31·3–65·4)	-1·0 (-1·2–-0·8)	-0·7 (-0·9–-0·5)	-0·3 (-0·6–-0·1)
High systolic blood pressure	30·3 (20·4–42·6)	25·8 (18·1–35·4)	18·9 (12·5–27·1)	26·7 (17·2–37·9)	-0·4 (-1·3–0·5)	0·2 (-0·9–1·4)	3·1 (0·9–5·8)
High body-mass index	29·6 (24·9–34·2)	35·7 (30·5–39·5)	39·7 (34·0–43·4)	42·7 (36·5–46·6)	1·2 (0·9–1·5)	0·9 (0·5–1·2)	0·7 (0·2–1·1)
Low bone mineral density	21·3 (15·3–28·4)	19·2 (13·6–26·3)	19·9 (13·9–26·8)	21·8 (15·8–28·7)	0·1 (-0·4–0·6)	0·6 (-0·2–1·4)	0·8 (-0·4–2·3)
Kidney dysfunction	3·0 (2·4–3·9)	3·0 (2·5–4·0)	3·0 (2·4–3·9)	3·1 (2·5–4·0)	0·1 (0·0–0·2)	0·1 (-0·1–0·2)	0·3 (0·0–0·6)

Massachusetts

Risk Names	SEV 1990	SEV 2000	SEV 2010	SEV 2021	Annualised rate of change 1990 to 2021	Annualised rate of change 2000 to 2021	Annualised rate of change 2010 to 2021
**All risk factors**	**28·8 (25·7–31·8)**	**28·4 (25·3–31·5)**	**29·5 (26·5–32·6)**	**29·8 (26·3–33·1)**	**0·1 (-0·2–0·4)**	**0·2 (-0·1–0·6)**	**0·1 (-0·5–0·6)**
**Environmental/occupational risks**	**17·2 (11·0–28·2)**	**17·1 (11·1–27·9)**	**14·8 (9·6–24·5)**	**13·1 (8·1–22·0)**	**-0·9 (-1·6–-0·2)**	**-1·3 (-1·7–-0·7)**	**-1·1 (-1·6–-0·4)**
Unsafe water, sanitation, and handwashing	1·4 (0·6–2·3)	1·3 (0·5–2·0)	1·1 (0·3–1·9)	0·9 (0·3–1·7)	-1·3 (-3·8–1·0)	-1·6 (-5·0–1·2)	-1·5 (-5·6–2·3)
Unsafe water source	1·9 (0·7–4·0)	1·8 (0·6–3·6)	0·8 (0·3–1·6)	0·7 (0·3–1·3)	-3·5 (-5·9–-0·4)	-4·7 (-8·1–-1·0)	-1·5 (-6·9–3·4)
Unsafe sanitation	3·8 (2·0–6·5)	2·9 (1·4–5·2)	2·3 (1·1–4·1)	1·9 (0·9–3·4)	-2·3 (-4·6–-0·1)	-2·1 (-4·8–0·7)	-1·8 (-5·2–1·9)
No access to handwashing facility	1·4 (0·4–2·7)	1·4 (0·5–2·8)	1·5 (0·5–2·8)	1·3 (0·4–2·6)	-0·3 (-3·4–2·9)	-0·5 (-4·5–3·5)	-1·3 (-7·1–4·0)
Air pollution	23·0 (5·2–33·2)	21·2 (5·7–27·7)	11·6 (3·3–22·1)	7·5 (0·9–16·9)	-3·6 (-7·4–-1·3)	-4·9 (-9·9–-2·1)	-4·0 (-11·4–-2·2)
Particulate matter pollution	9·6 (2·4–18·4)	8·1 (5·1–11·8)	5·4 (2·5–8·8)	2·6 (0·4–5·5)	-4·2 (-9·7–-0·7)	-5·4 (-12·6–-3·3)	-6·5 (-18·0–-3·4)
Ambient particulate matter pollution	14·8 (3·6–28·7)	12·4 (7·9–17·7)	8·2 (3·9–13·0)	4·0 (0·7–8·3)	-4·2 (-9·8–-0·7)	-5·4 (-12·6–-3·4)	-6·5 (-18·0–-3·3)
Household air pollution from solid fuels	0·0 (0·0–0·0)	0·0 (0·0–0·0)	0·0 (0·0–0·0)	0·0 (0·0–0·0)	-4·0 (-51·3–2·6)	-5·2 (-53·7–14·6)	-5·1 (-54·4–39·3)
Ambient ozone pollution	19·9 (13·9–27·6)	17·1 (11·6–24·6)	13·9 (8·9–20·7)	9·5 (4·9–15·7)	-2·4 (-3·9–-1·4)	-2·8 (-4·8–-1·7)	-3·5 (-6·7–-1·3)
Ambient nitrogen dioxide pollution	81·8 (0·0–100·0)	79·2 (0·0–100·0)	38·7 (0·0–93·8)	29·2 (0·0–79·0)	-3·3 (-7·7–0·0)	-4·7 (-10·5–0·0)	-2·5 (-5·5–0·0)
Non-optimal temperature	30·5 (23·8–41·3)	29·4 (24·0–38·7)	33·6 (25·8–44·5)	31·1 (24·3–41·4)	0·1 (-0·2–0·3)	0·3 (-0·1–0·7)	-0·7 (-1·3–-0·3)
High temperature	26·1 (16·2–36·9)	19·5 (10·9–30·1)	35·6 (23·2–48·1)	32·0 (20·5–44·4)	0·7 (0·2–1·2)	2·4 (1·3–3·9)	-1·0 (-1·6–-0·1)
Low temperature	27·4 (24·3–30·8)	28·1 (25·2–31·5)	28·4 (25·5–31·7)	27·2 (24·3–30·6)	0·0 (-0·2–0·1)	-0·2 (-0·3–-0·1)	-0·4 (-0·7–-0·1)
Other environmental risks	27·3 (7·7–48·9)	25·5 (7·7–46·8)	23·0 (7·7–44·0)	20·5 (7·7–40·8)	-0·9 (-1·7–0·0)	-1·0 (-1·9–0·0)	-1·0 (-2·2–0·0)
Residential radon	29·2 (0·0–97·7)	29·2 (0·0–97·7)	29·2 (0·0–97·7)	29·2 (0·0–97·7)	0·0 (0·0–0·0)	0·0 (0·0–0·0)	0·0 (0·0–0·0)
Lead exposure	26·4 (0·0–33·1)	23·8 (0·0–29·9)	20·1 (0·0–25·3)	16·5 (0·0–20·7)	-1·5 (-1·9–0·0)	-1·7 (-2·3–0·0)	-1·8 (-2·6–0·0)
Occupational risks	3·0 (2·5–4·0)	3·1 (2·5–4·1)	2·9 (2·4–3·8)	2·9 (2·3–3·8)	-0·1 (-0·4–0·1)	-0·3 (-0·7–0·0)	0·0 (-0·5–0·5)
Occupational carcinogens	1·1 (0·5–2·1)	1·2 (0·5–2·2)	1·1 (0·5–2·1)	1·1 (0·5–2·1)	0·1 (-0·3–0·3)	-0·3 (-0·8–0·1)	0·1 (-0·5–0·7)
Occupational exposure to asbestos	5·4 (4·9–5·9)	6·3 (5·8–6·7)	4·8 (4·4–5·2)	4·6 (3·7–5·6)	-0·5 (-1·3–0·2)	-1·5 (-2·5–-0·5)	-0·4 (-2·4–1·4)
Occupational exposure to arsenic	0·5 (0·0–1·3)	0·5 (0·0–1·3)	0·5 (0·0–1·2)	0·5 (0·0–1·2)	-0·3 (-0·7–0·2)	-0·3 (-0·9–0·4)	0·1 (-0·8–1·1)
Occupational exposure to benzene	1·2 (0·1–3·5)	1·3 (0·1–3·8)	1·3 (0·1–3·7)	1·3 (0·1–3·8)	0·3 (0·0–0·6)	0·0 (-0·4–0·4)	0·2 (-0·5–0·8)
Occupational exposure to beryllium	0·0 (0·0–0·0)	0·0 (0·0–0·0)	0·0 (0·0–0·0)	0·0 (0·0–0·0)	0·2 (0·0–0·5)	0·1 (-0·2–0·4)	0·4 (0·0–0·8)
Occupational exposure to cadmium	0·1 (0·1–0·1)	0·1 (0·1–0·1)	0·1 (0·1–0·1)	0·1 (0·1–0·1)	-0·2 (-0·6–0·2)	-0·2 (-0·7–0·4)	0·1 (-0·6–0·9)
Occupational exposure to chromium	0·2 (0·1–0·2)	0·2 (0·1–0·2)	0·1 (0·1–0·2)	0·2 (0·1–0·2)	-0·1 (-0·4–0·3)	-0·2 (-0·6–0·4)	0·2 (-0·5–0·9)
Occupational exposure to diesel engine exhaust	0·7 (0·6–0·7)	0·7 (0·7–0·8)	0·7 (0·7–0·8)	0·8 (0·7–0·9)	0·4 (0·1–0·8)	0·3 (-0·2–0·9)	0·6 (-0·1–1·3)
Occupational exposure to formaldehyde	0·3 (0·3–0·4)	0·3 (0·3–0·3)	0·3 (0·3–0·3)	0·3 (0·3–0·3)	-0·3 (-0·7–0·2)	-0·3 (-0·8–0·3)	0·0 (-0·8–0·8)
Occupational exposure to nickel	0·4 (0·0–1·7)	0·4 (0·0–1·6)	0·4 (0·0–1·4)	0·4 (0·0–1·4)	-0·4 (-0·9–0·1)	-0·3 (-1·0–0·5)	0·1 (-1·0–1·2)
Occupational exposure to polycyclic aromatic hydrocarbons	0·3 (0·3–0·3)	0·3 (0·3–0·3)	0·3 (0·3–0·3)	0·3 (0·3–0·3)	0·0 (-0·4–0·3)	-0·2 (-0·6–0·3)	0·1 (-0·5–0·8)
Occupational exposure to silica	3·6 (0·6–11·6)	3·4 (0·5–11·0)	3·2 (0·5–10·2)	3·2 (0·5–10·7)	-0·4 (-0·8–0·1)	-0·2 (-0·9–0·4)	0·2 (-0·7–1·1)
Occupational exposure to sulphuric acid	0·8 (0·2–2·7)	0·8 (0·2–2·7)	0·7 (0·2–2·5)	0·8 (0·2–2·4)	-0·2 (-0·6–0·3)	-0·2 (-0·7–0·4)	0·2 (-0·6–1·1)
Occupational exposure to trichloroethylene	0·1 (0·1–0·1)	0·1 (0·1–0·1)	0·1 (0·1–0·1)	0·1 (0·1–0·1)	0·1 (-0·2–0·4)	0·0 (-0·3–0·4)	0·3 (-0·2–0·9)
Occupational asthmagens	17·8 (15·6–20·3)	18·1 (16·0–20·6)	16·9 (14·9–19·2)	17·2 (15·1–19·4)	-0·1 (-0·4–0·3)	-0·2 (-0·7–0·2)	0·1 (-0·5–0·8)
Occupational particulate matter, gases, and fumes	6·7 (5·1–8·7)	6·4 (4·9–8·4)	6·0 (4·6–7·7)	5·5 (4·3–7·2)	-0·6 (-0·9–-0·3)	-0·7 (-1·0–-0·4)	-0·7 (-1·1–-0·3)
Occupational noise	6·8 (6·2–7·5)	6·9 (6·3–7·5)	6·8 (6·3–7·3)	6·6 (6·2–7·1)	-0·1 (-0·3–0·0)	-0·2 (-0·4–0·0)	-0·3 (-0·5–-0·1)
Occupational injuries	--	--	--	--	--	--	--
Occupational ergonomic factors	7·9 (6·6–9·6)	8·0 (6·7–9·6)	7·4 (6·2–9·0)	7·5 (6·3–9·2)	-0·1 (-0·6–0·2)	-0·3 (-0·8–0·3)	0·2 (-0·5–0·9)
**Behavioural risks**	**33·0 (29·7–36·5)**	**30·3 (26·8–33·9)**	**28·1 (25·0–31·7)**	**26·2 (22·6–30·0)**	**-0·7 (-1·1–-0·4)**	**-0·7 (-1·2–-0·3)**	**-0·6 (-1·3–0·0)**
Child and maternal malnutrition	10·0 (6·7–13·9)	8·4 (5·5–11·4)	8·5 (5·8–11·5)	8·7 (6·1–12·2)	-0·4 (-1·3–0·4)	0·2 (-1·1–1·4)	0·3 (-1·5–1·9)
Suboptimal breastfeeding	68·6 (65·8–71·2)	66·4 (63·9–69·2)	62·5 (59·8–65·5)	61·9 (58·8–64·9)	-0·3 (-0·5–-0·2)	-0·3 (-0·5–-0·2)	-0·1 (-0·4–0·1)
Non-exclusive breastfeeding	63·4 (55·7–70·9)	58·7 (52·0–65·5)	47·9 (41·2–54·3)	45·6 (38·4–52·5)	-1·1 (-1·6–-0·6)	-1·2 (-1·9–-0·7)	-0·4 (-1·4–0·4)
Discontinued breastfeeding	88·6 (86·3–90·9)	86·2 (83·4–88·8)	84·2 (80·8–87·2)	84·3 (80·7–88·2)	-0·2 (-0·3–0·0)	-0·1 (-0·3–0·1)	0·0 (-0·3–0·3)
Child growth failure	1·1 (0·4–2·4)	1·0 (0·3–2·2)	0·9 (0·3–1·9)	0·8 (0·3–1·7)	-1·0 (-1·3–-0·6)	-1·0 (-1·6–-0·6)	-0·7 (-1·4–0·1)
Child underweight	2·3 (1·5–3·1)	2·1 (1·4–2·9)	1·8 (1·2–2·4)	1·7 (1·1–2·3)	-1·0 (-1·5–-0·4)	-1·1 (-1·8–-0·4)	-0·6 (-1·6–0·4)
Child wasting	0·8 (0·5–1·2)	0·7 (0·5–1·1)	0·7 (0·5–1·1)	0·7 (0·4–1·1)	-0·3 (-0·7–0·1)	-0·1 (-0·5–0·4)	-0·3 (-0·9–0·4)
Child stunting	3·2 (2·5–4·0)	3·0 (2·4–3·7)	2·6 (2·0–3·2)	2·3 (1·8–2·9)	-1·0 (-1·7–-0·5)	-1·2 (-2·0–-0·4)	-0·9 (-2·1–0·5)
Low birth weight and short gestation	18·6 (16·3–21·0)	21·3 (18·5–24·2)	22·9 (20·2–26·0)	22·1 (19·4–25·3)	0·6 (0·3–0·8)	0·2 (-0·2–0·6)	-0·3 (-1·1–0·4)
Short gestation	34·2 (30·0–39·0)	38·4 (33·1–43·8)	39·8 (34·6–44·9)	37·7 (33·0–43·0)	0·3 (0·0–0·6)	-0·1 (-0·5–0·4)	-0·5 (-1·4–0·4)
Low birth weight	14·6 (13·1–16·2)	16·3 (14·6–18·0)	17·7 (15·9–19·3)	17·5 (15·8–19·4)	0·6 (0·3–0·8)	0·3 (0·0–0·7)	-0·1 (-0·8–0·6)

Iron deficiency	3·6 (2·5–4·9)	3·0 (2·0–4·0)	3·1 (2·2–4·1)	3·1 (2·2–4·3)	-0·4 (-1·2–0·4)	0·2 (-1·0–1·3)	0·2 (-1·5–1·8)
Vitamin A deficiency	0·6 (0·0–1·2)	0·7 (0·0–1·4)	0·4 (0·0–0·9)	0·3 (0·0–0·6)	-2·5 (-5·0–0·0)	-4·6 (-7·6–0·0)	-4·1 (-8·4–0·1)
Zinc deficiency	1·6 (0·0–6·7)	0·9 (0·0–3·9)	0·8 (0·0–3·5)	0·8 (0·0–3·3)	-2·2 (-44·7–3·4)	-0·9 (-6·8–4·1)	-0·4 (-11·9–6·5)
Tobacco	41·6 (39·9–43·3)	33·6 (32·2–34·9)	26·4 (25·4–27·4)	22·3 (20·8–23·9)	-2·0 (-2·2–-1·8)	-2·0 (-2·3–-1·6)	-1·5 (-2·2–-0·9)
Smoking	40·1 (37·5–42·4)	32·1 (29·9–34·1)	25·2 (23·7–26·8)	21·0 (19·3–23·2)	-2·1 (-2·4–-1·7)	-2·0 (-2·5–-1·5)	-1·6 (-2·4–-0·8)
Chewing tobacco	0·8 (0·6–1·0)	1·0 (0·7–1·3)	1·1 (0·8–1·5)	1·2 (0·8–1·6)	1·4 (0·0–2·5)	0·9 (-0·9–2·5)	0·5 (-2·2–3·2)
Second-hand smoke	33·5 (31·6–34·9)	26·9 (25·3–28·0)	21·3 (20·0–22·2)	18·4 (16·7–20·0)	-1·9 (-2·2–-1·7)	-1·8 (-2·2–-1·4)	-1·4 (-2·1–-0·7)
High alcohol use	34·1 (25·5–45·3)	33·3 (25·8–44·6)	34·3 (26·5–44·4)	34·3 (25·5–45·3)	0·0 (-0·8–0·8)	0·1 (-1·0–1·2)	0·0 (-1·5–1·3)
Drug use	0·8 (0·4–1·6)	1·4 (1·3–1·7)	2·6 (2·0–3·2)	5·6 (4·1–7·2)	6·3 (3·3–9·2)	6·5 (4·7–7·5)	7·0 (5·7–7·9)
Dietary risks	34·7 (25·6–45·1)	37·1 (27·7–48·2)	39·4 (30·5–51·1)	39·7 (30·3–51·4)	0·4 (0·2–0·8)	0·3 (0·0–0·7)	0·1 (-0·4–0·6)
Diet low in fruits	32·3 (27·4–35·5)	29·5 (25·1–32·5)	29·6 (25·6–32·2)	30·5 (25·4–34·6)	-0·2 (-0·6–0·3)	0·2 (-0·4–0·7)	0·3 (-0·6–1·2)
Diet low in vegetables	25·0 (14·7–32·8)	24·6 (14·2–32·7)	26·6 (15·0–34·0)	25·7 (14·6–33·7)	0·1 (-0·4–0·6)	0·2 (-0·5–0·8)	-0·3 (-1·4–0·5)
Diet low in legumes	36·6 (0·0–46·5)	36·6 (0·0–46·3)	40·1 (0·0–49·8)	40·2 (0·0–50·7)	0·3 (0·0–0·6)	0·5 (0·0–1·0)	0·0 (-0·6–0·8)
Diet low in whole grains	43·8 (36·1–49·9)	46·5 (38·2–52·6)	48·2 (40·5–55·0)	49·1 (41·4–56·4)	0·4 (0·1–0·7)	0·3 (-0·1–0·7)	0·2 (-0·3–0·7)
Diet low in nuts and seeds	10·3 (7·3–14·3)	5·4 (3·3–7·8)	3·5 (2·0–5·6)	3·6 (1·8–5·8)	-3·4 (-5·6–-1·6)	-1·9 (-4·8–0·7)	0·3 (-4·1–4·5)
Diet low in milk	34·0 (29·3–38·6)	30·8 (26·8–35·2)	28·9 (24·3–33·5)	28·1 (23·7–32·9)	-0·6 (-1·1–-0·1)	-0·4 (-1·1–0·2)	-0·3 (-1·2–0·7)
Diet high in red meat	45·9 (0·0–65·0)	47·6 (0·0–66·1)	49·0 (0·0–68·2)	50·0 (0·0–68·7)	0·3 (-0·2–1·1)	0·2 (-0·4–0·9)	0·2 (-0·8–1·3)
Diet high in processed meat	40·0 (31·2–44·9)	50·2 (40·3–56·4)	57·5 (46·6–64·5)	57·0 (48·2–65·4)	1·1 (0·7–1·6)	0·6 (0·0–1·2)	-0·1 (-0·9–0·7)
Diet high in sugar-sweetened beverages	38·7 (29·7–47·4)	53·0 (41·5–61·9)	59·4 (48·0–68·5)	60·7 (48·8–70·4)	1·4 (0·8–2·2)	0·6 (-0·2–1·4)	0·2 (-1·0–1·3)
Diet low in fibre	36·5 (19·5–43·5)	26·9 (14·9–34·1)	23·3 (13·6–29·2)	19·7 (11·5–26·3)	-2·0 (-2·9–-1·1)	-1·5 (-2·8–-0·1)	-1·6 (-3·5–0·4)
Diet low in calcium	5·2 (4·2–6·3)	4·4 (3·5–5·4)	4·5 (3·6–5·5)	4·4 (3·5–5·4)	-0·5 (-0·7–-0·4)	0·0 (-0·2–0·2)	-0·2 (-0·5–0·0)
Diet low in seafood omega-3 fatty acids	32·4 (23·9–41·6)	39·2 (29·8–49·7)	41·1 (31·2–51·7)	34·7 (25·4–44·4)	0·2 (-0·3–0·8)	-0·6 (-1·2–0·0)	-1·5 (-2·5–-0·8)
Diet low in omega-6 polyunsaturated fatty acids	39·7 (25·3–49·7)	37·7 (24·3–47·4)	32·4 (21·5–40·7)	29·4 (19·5–37·6)	-1·0 (-1·5–-0·4)	-1·2 (-1·8–-0·5)	-0·9 (-1·8–-0·1)
Diet high in trans fatty acids	71·4 (64·2–78·6)	71·6 (62·5–79·1)	63·3 (54·5–71·0)	0·0 (0·0–0·0)	-65·8 (-66·1–-65·4)	-97·1 (-97·6–-96·4)	-184·2 (-185·3–-182·9)
Diet high in sodium	23·0 (0·7–66·7)	27·6 (2·0–74·3)	30·5 (3·6–75·0)	31·1 (3·2–76·4)	1·0 (0·2–6·6)	0·6 (-0·2–3·8)	0·2 (-2·4–2·2)
Intimate partner violence	26·6 (17·5–34·9)	26·9 (18·6–34·3)	22·2 (13·1–28·1)	21·3 (10·8–30·3)	-0·7 (-2·3–0·5)	-1·1 (-3·0–0·7)	-0·3 (-2·7–1·9)
Childhood sexual abuse and bullying	8·6 (5·6–13·7)	10·4 (6·7–16·4)	11·9 (7·6–19·0)	9·5 (6·2–14·9)	0·3 (-0·2–0·9)	-0·4 (-1·2–0·4)	-2·1 (-3·5–-0·7)
Childhood sexual abuse	7·5 (6·3–8·8)	8·5 (7·4–10·0)	9·9 (8·6–11·3)	9·2 (7·6–11·2)	0·6 (-0·3–1·5)	0·4 (-0·8–1·4)	-0·7 (-2·3–0·8)
Bullying victimization	7·9 (3·5–15·9)	9·9 (4·5–19·3)	11·3 (5·1–22·0)	8·1 (3·6–15·5)	0·1 (-0·7–0·8)	-0·9 (-2·0–0·0)	-3·0 (-4·8–-1·1)
Unsafe sex	--	--	--	--	--	--	--
Low physical activity	18·2 (13·6–23·1)	17·3 (13·2–21·6)	17·8 (13·8–22·2)	20·6 (15·7–26·4)	0·4 (-0·5–1·4)	0·8 (-0·4–2·1)	1·3 (-0·5–3·2)
**Metabolic risks**	**24·7 (21·4–28·1)**	**28·7 (25·3–31·8)**	**31·8 (28·1–34·8)**	**34·5 (30·0–37·5)**	**1·1 (0·8–1·4)**	**0·9 (0·5–1·2)**	**0·7 (0·2–1·2)**
High fasting plasma glucose	12·2 (9·3–14·5)	15·2 (12·0–18·2)	18·9 (14·0–23·2)	22·7 (17·3–27·7)	2·0 (1·4–2·7)	1·9 (1·1–2·9)	1·7 (0·5–2·9)
High LDL cholesterol	63·9 (45·4–87·0)	53·8 (37·1–74·3)	48·0 (32·7–67·1)	46·4 (31·5–64·6)	-1·0 (-1·2–-0·9)	-0·7 (-0·9–-0·5)	-0·3 (-0·6–-0·1)
High systolic blood pressure	27·6 (18·2–38·2)	23·6 (16·2–32·3)	17·5 (11·3–25·6)	24·6 (15·1–36·0)	-0·4 (-1·3–0·5)	0·2 (-0·9–1·3)	3·1 (0·8–5·4)
High body-mass index	27·8 (23·3–32·5)	32·7 (27·9–36·9)	36·4 (31·2–40·5)	38·7 (32·6–42·9)	1·1 (0·8–1·4)	0·8 (0·5–1·1)	0·6 (0·1–1·0)
Low bone mineral density	19·2 (13·4–25·8)	19·4 (14·0–26·3)	18·1 (12·5–24·3)	18·6 (13·0–25·2)	-0·1 (-0·6–0·5)	-0·2 (-0·9–0·5)	0·2 (-1·2–1·8)
Kidney dysfunction	3·0 (2·4–4·1)	3·0 (2·4–4·0)	3·0 (2·3–4·0)	3·1 (2·5–4·2)	0·1 (0·0–0·2)	0·2 (0·0–0·3)	0·5 (0·2–0·8)

Michigan

Risk Names	SEV 1990	SEV 2000	SEV 2010	SEV 2021	Annualised rate of change 1990 to 2021	Annualised rate of change 2000 to 2021	Annualised rate of change 2010 to 2021
**All risk factors**	**27·3 (24·0–30·6)**	**27·0 (23·9–30·1)**	**27·6 (24·4–30·8)**	**27·7 (24·3–31·2)**	**0·0 (-0·3–0·3)**	**0·1 (-0·3–0·5)**	**0·0 (-0·6–0·6)**
**Environmental/occupational risks**	**18·6 (12·2–29·4)**	**17·8 (12·1–28·6)**	**15·5 (10·1–25·5)**	**14·1 (9·1–23·8)**	**-0·9 (-1·7–-0·2)**	**-1·1 (-1·5–-0·6)**	**-0·9 (-1·3–-0·4)**
Unsafe water, sanitation, and handwashing	1·9 (1·0–3·1)	1·7 (0·8–2·8)	1·3 (0·5–2·3)	1·1 (0·4–1·9)	-1·7 (-4·0–0·3)	-2·0 (-4·9–0·6)	-1·9 (-6·0–2·1)
Unsafe water source	2·7 (0·9–5·6)	2·4 (0·9–4·8)	1·0 (0·4–1·8)	0·8 (0·4–1·5)	-3·9 (-6·7–-1·2)	-5·2 (-9·4–-1·5)	-2·0 (-7·2–3·4)
Unsafe sanitation	6·0 (3·2–10·0)	4·5 (2·4–7·8)	3·5 (1·8–6·1)	2·6 (1·3–4·6)	-2·6 (-4·9–-0·5)	-2·5 (-5·2–0·0)	-2·6 (-5·9–0·5)
No access to handwashing facility	1·6 (0·5–2·9)	1·6 (0·5–3·0)	1·7 (0·6–3·0)	1·4 (0·5–2·7)	-0·3 (-3·3–2·5)	-0·6 (-4·6–3·1)	-1·5 (-7·0–4·1)
Air pollution	24·8 (7·0–35·8)	22·7 (8·2–29·9)	13·3 (4·7–23·4)	9·2 (3·0–17·9)	-3·2 (-5·6–-1·1)	-4·3 (-6·9–-2·1)	-3·4 (-6·1–-1·6)
Particulate matter pollution	12·4 (4·2–22·1)	11·1 (7·7–14·9)	7·0 (4·1–10·3)	5·2 (2·5–8·3)	-2·8 (-5·4–0·0)	-3·6 (-5·7–-2·5)	-2·6 (-5·5–-1·1)
Ambient particulate matter pollution	19·3 (6·5–34·7)	17·1 (12·3–22·4)	10·7 (6·2–15·4)	8·0 (3·8–12·4)	-2·8 (-5·4–0·0)	-3·6 (-5·8–-2·5)	-2·7 (-5·5–-1·1)
Household air pollution from solid fuels	0·0 (0·0–0·0)	0·0 (0·0–0·0)	0·0 (0·0–0·0)	0·0 (0·0–0·0)	-2·3 (-26·8–-5·6)	-2·3 (-20·7–-4·4)	-2·5 (-22·4–-4·3)
Ambient ozone pollution	19·6 (13·6–26·5)	19·2 (13·1–25·5)	14·7 (9·5–21·2)	15·8 (10·4–22·0)	-0·7 (-1·2–-0·3)	-0·9 (-1·6–-0·4)	0·6 (-0·3–1·6)
Ambient nitrogen dioxide pollution	78·1 (0·0–100·0)	73·1 (0·0–100·0)	40·0 (0·0–94·1)	24·9 (0·0–72·1)	-3·7 (-9·1–0·0)	-5·1 (-12·3–0·0)	-4·3 (-12·1–0·0)
Non-optimal temperature	31·3 (24·5–41·4)	29·5 (23·7–39·4)	33·8 (25·8–44·7)	31·6 (24·5–42·2)	0·0 (-0·2–0·3)	0·3 (0·0–0·7)	-0·6 (-1·3–-0·2)
High temperature	24·3 (14·2–35·9)	19·9 (9·8–32·5)	32·8 (20·8–45·2)	29·7 (18·5–41·3)	0·6 (0·2–1·3)	1·9 (0·8–3·9)	-0·9 (-1·9–0·0)
Low temperature	28·7 (25·3–32·5)	27·9 (24·8–31·9)	29·3 (26·0–33·5)	28·3 (24·8–32·5)	0·0 (-0·2–0·1)	0·1 (-0·1–0·2)	-0·3 (-0·7–0·0)
Other environmental risks	29·9 (9·4–51·4)	28·0 (9·6–49·5)	25·2 (9·3–46·6)	22·2 (9·0–43·2)	-1·0 (-1·7–0·0)	-1·1 (-2·0–0·0)	-1·2 (-2·3–0·0)
Residential radon	31·4 (0·0–100·0)	31·4 (0·0–100·0)	31·4 (0·0–100·0)	31·4 (0·0–100·0)	0·0 (0·0–0·0)	0·0 (0·0–0·0)	0·0 (0·0–0·0)
Lead exposure	29·2 (0·0–36·3)	26·5 (0·0–33·1)	22·4 (0·0–28·2)	18·0 (0·0–22·3)	-1·6 (-2·0–0·0)	-1·8 (-2·3–0·0)	-2·0 (-2·9–0·0)
Occupational risks	3·0 (2·5–4·0)	3·1 (2·6–4·0)	2·8 (2·3–3·7)	2·8 (2·3–3·7)	-0·3 (-0·6–0·0)	-0·5 (-0·8–-0·1)	0·0 (-0·5–0·5)
Occupational carcinogens	1·0 (0·5–1·8)	1·0 (0·5–2·0)	1·0 (0·4–1·8)	1·0 (0·4–1·9)	0·0 (-0·3–0·3)	-0·2 (-0·7–0·2)	0·2 (-0·5–0·7)
Occupational exposure to asbestos	4·2 (3·8–4·7)	4·9 (4·5–5·3)	4·4 (3·9–4·7)	4·1 (3·3–5·0)	-0·1 (-0·8–0·6)	-0·9 (-1·9–0·1)	-0·6 (-2·5–1·2)
Occupational exposure to arsenic	0·6 (0·0–1·3)	0·6 (0·0–1·4)	0·5 (0·0–1·2)	0·5 (0·0–1·2)	-0·5 (-1·0–0·0)	-0·7 (-1·3–0·0)	0·0 (-0·9–1·0)
Occupational exposure to benzene	1·0 (0·1–2·9)	1·1 (0·1–3·2)	1·1 (0·1–3·2)	1·1 (0·1–3·4)	0·3 (0·0–0·7)	0·1 (-0·4–0·6)	0·5 (-0·2–1·1)
Occupational exposure to beryllium	0·0 (0·0–0·0)	0·0 (0·0–0·0)	0·0 (0·0–0·0)	0·0 (0·0–0·0)	0·1 (-0·2–0·3)	0·0 (-0·4–0·4)	0·5 (0·1–1·0)
Occupational exposure to cadmium	0·1 (0·1–0·1)	0·1 (0·1–0·1)	0·1 (0·1–0·1)	0·1 (0·1–0·1)	-0·4 (-0·8–0·0)	-0·5 (-1·1–0·1)	0·1 (-0·7–0·9)
Occupational exposure to chromium	0·2 (0·1–0·2)	0·2 (0·1–0·2)	0·1 (0·1–0·2)	0·1 (0·1–0·2)	-0·3 (-0·6–0·1)	-0·4 (-0·9–0·1)	0·2 (-0·5–0·9)
Occupational exposure to diesel engine exhaust	0·6 (0·6–0·7)	0·6 (0·6–0·7)	0·6 (0·6–0·7)	0·7 (0·6–0·7)	0·3 (-0·1–0·7)	0·3 (-0·2–0·8)	0·9 (0·1–1·6)
Occupational exposure to formaldehyde	0·3 (0·3–0·4)	0·3 (0·3–0·4)	0·3 (0·3–0·3)	0·3 (0·3–0·3)	-0·5 (-0·9–-0·1)	-0·6 (-1·2–-0·1)	0·0 (-0·8–0·8)
Occupational exposure to nickel	0·5 (0·0–1·8)	0·4 (0·0–1·8)	0·4 (0·0–1·5)	0·4 (0·0–1·5)	-0·5 (-1·1–0·0)	-0·7 (-1·4–0·1)	0·0 (-1·0–1·0)
Occupational exposure to polycyclic aromatic hydrocarbons	0·3 (0·3–0·3)	0·3 (0·3–0·3)	0·3 (0·3–0·3)	0·3 (0·3–0·3)	-0·2 (-0·6–0·1)	-0·4 (-0·9–0·1)	0·2 (-0·5–0·9)
Occupational exposure to silica	3·9 (0·6–12·7)	3·8 (0·6–12·0)	3·3 (0·5–10·4)	3·3 (0·5–10·6)	-0·5 (-1·1–-0·1)	-0·6 (-1·3–0·0)	0·1 (-0·9–1·0)
Occupational exposure to sulphuric acid	0·8 (0·2–2·8)	0·8 (0·2–2·8)	0·7 (0·2–2·4)	0·7 (0·2–2·4)	-0·4 (-0·8–0·0)	-0·5 (-1·2–0·0)	0·1 (-0·7–1·0)
Occupational exposure to trichloroethylene	0·1 (0·1–0·1)	0·1 (0·1–0·1)	0·1 (0·1–0·1)	0·1 (0·1–0·1)	-0·1 (-0·4–0·2)	-0·2 (-0·6–0·2)	0·4 (-0·2–1·0)
Occupational asthmagens	18·3 (16·0–21·1)	18·5 (16·4–20·9)	16·3 (14·4–18·5)	16·7 (14·6–19·0)	-0·3 (-0·7–0·0)	-0·5 (-0·9–0·0)	0·2 (-0·5–0·8)
Occupational particulate matter, gases, and fumes	7·3 (5·4–9·6)	7·1 (5·3–9·3)	6·6 (5·0–8·6)	6·0 (4·6–7·8)	-0·6 (-0·9–-0·3)	-0·8 (-1·1–-0·5)	-0·9 (-1·2–-0·5)
Occupational noise	6·9 (6·3–7·6)	7·0 (6·4–7·6)	6·8 (6·3–7·3)	6·5 (6·1–7·0)	-0·2 (-0·4–0·0)	-0·3 (-0·5–-0·1)	-0·4 (-0·6–-0·2)
Occupational injuries	--	--	--	--	--	--	--
Occupational ergonomic factors	7·8 (6·5–9·5)	7·7 (6·5–9·3)	6·9 (5·7–8·2)	7·1 (5·9–8·7)	-0·3 (-0·7–0·1)	-0·4 (-0·9–0·1)	0·3 (-0·4–1·0)
**Behavioural risks**	**31·5 (28·0–34·9)**	**29·9 (26·8–33·6)**	**28·3 (25·0–32·0)**	**26·8 (23·2–30·6)**	**-0·5 (-0·8–-0·2)**	**-0·5 (-1·0–0·0)**	**-0·5 (-1·1–0·1)**
Child and maternal malnutrition	10·3 (7·0–14·4)	8·9 (5·9–12·4)	9·0 (6·1–12·2)	9·2 (6·2–12·6)	-0·4 (-1·3–0·5)	0·2 (-1·1–1·4)	0·2 (-1·9–2·1)
Suboptimal breastfeeding	68·8 (66·4–71·4)	66·7 (64·3–69·2)	62·8 (60·1–65·7)	62·2 (59·3–64·9)	-0·3 (-0·5–-0·2)	-0·3 (-0·5–-0·2)	-0·1 (-0·4–0·2)
Non-exclusive breastfeeding	64·3 (56·5–71·9)	59·6 (53·1–66·4)	48·6 (41·9–55·1)	46·3 (39·7–53·1)	-1·1 (-1·6–-0·6)	-1·2 (-1·8–-0·6)	-0·4 (-1·3–0·6)
Discontinued breastfeeding	88·7 (86·5–90·9)	86·3 (83·8–89·2)	84·4 (81·1–87·7)	84·6 (81·4–87·8)	-0·2 (-0·3–0·0)	-0·1 (-0·3–0·1)	0·0 (-0·3–0·3)
Child growth failure	1·3 (0·4–3·0)	1·2 (0·4–2·7)	1·1 (0·3–2·4)	1·0 (0·3–2·2)	-1·0 (-1·4–-0·6)	-1·0 (-1·5–-0·5)	-0·9 (-1·6–-0·1)
Child underweight	2·8 (1·7–3·9)	2·6 (1·6–3·5)	2·2 (1·4–3·0)	2·1 (1·3–2·8)	-1·0 (-1·6–-0·5)	-1·1 (-1·7–-0·4)	-0·8 (-1·8–0·2)
Child wasting	0·9 (0·6–1·4)	0·8 (0·5–1·3)	0·8 (0·5–1·3)	0·8 (0·5–1·3)	-0·3 (-0·6–0·0)	-0·1 (-0·5–0·3)	-0·4 (-1·2–0·2)
Child stunting	3·9 (3·0–4·8)	3·5 (2·8–4·4)	3·1 (2·4–3·9)	2·8 (2·1–3·5)	-1·1 (-1·7–-0·5)	-1·2 (-2·0–-0·4)	-1·0 (-2·2–0·2)
Low birth weight and short gestation	23·4 (20·4–26·2)	25·2 (21·8–28·7)	26·9 (23·6–30·5)	27·8 (24·2–31·5)	0·6 (0·3–0·8)	0·5 (0·1–0·8)	0·3 (-0·4–1·0)
Short gestation	43·2 (38·6–48·8)	46·7 (41·5–52·1)	48·4 (43·1–54·4)	48·7 (42·8–54·9)	0·4 (0·1–0·6)	0·2 (-0·1–0·6)	0·1 (-0·7–0·8)
Low birth weight	18·3 (16·7–19·9)	19·1 (17·4–20·8)	20·5 (18·6–22·5)	21·3 (19·5–23·3)	0·5 (0·2–0·7)	0·5 (0·2–0·9)	0·4 (-0·2–1·0)

Iron deficiency	3·7 (2·6–5·1)	3·2 (2·2–4·3)	3·3 (2·3–4·3)	3·3 (2·3–4·5)	-0·4 (-1·2–0·4)	0·2 (-0·9–1·4)	0·1 (-1·8–1·8)
Vitamin A deficiency	1·0 (0·0–1·9)	1·2 (0·0–2·6)	0·8 (0·0–1·8)	0·5 (0·0–1·1)	-2·2 (-4·3–0·3)	-4·4 (-7·3–0·0)	-4·6 (-8·7–0·0)
Zinc deficiency	1·8 (0·0–7·8)	1·1 (0·0–4·5)	1·0 (0·0–4·5)	1·0 (0·0–4·2)	-1·9 (-43·7–0·7)	-0·3 (-3·7–1·4)	-0·2 (-5·8–4·3)
Tobacco	43·3 (41·8–44·8)	38·6 (37·2–39·8)	33·2 (32·1–34·3)	30·2 (28·3–32·0)	-1·2 (-1·4–-1·0)	-1·2 (-1·4–-0·9)	-0·9 (-1·4–-0·3)
Smoking	40·6 (38·1–42·9)	35·2 (33·1–37·2)	29·8 (28·3–31·5)	27·1 (24·9–29·6)	-1·3 (-1·6–-1·0)	-1·3 (-1·6–-0·9)	-0·9 (-1·6–-0·2)
Chewing tobacco	1·7 (1·3–2·2)	1·9 (1·5–2·5)	2·1 (1·5–2·8)	2·1 (1·5–2·8)	0·7 (-0·7–2·0)	0·4 (-1·3–2·2)	0·2 (-2·4–2·7)
Second-hand smoke	34·9 (33·1–36·3)	31·3 (29·5–32·6)	27·1 (25·5–28·2)	24·8 (22·8–26·7)	-1·1 (-1·3–-0·9)	-1·1 (-1·5–-0·8)	-0·8 (-1·5–-0·2)
High alcohol use	27·8 (19·6–38·2)	27·0 (18·9–37·5)	27·8 (19·5–37·9)	28·2 (20·1–39·1)	0·0 (-1·0–1·2)	0·2 (-1·1–1·7)	0·1 (-1·6–2·2)
Drug use	0·9 (0·5–1·5)	1·1 (0·9–1·4)	1·8 (1·6–2·1)	5·6 (4·1–7·1)	6·0 (3·4–8·0)	7·7 (5·3–9·0)	10·1 (7·4–11·7)
Dietary risks	36·3 (27·2–47·5)	39·8 (30·5–51·7)	42·6 (33·1–54·6)	42·8 (33·8–55·4)	0·5 (0·3–0·8)	0·3 (0·0–0·7)	0·0 (-0·4–0·5)
Diet low in fruits	34·1 (30·1–37·6)	32·2 (27·6–35·3)	33·7 (30·3–36·9)	35·1 (30·7–39·6)	0·1 (-0·3–0·5)	0·4 (-0·2–1·0)	0·4 (-0·6–1·3)
Diet low in vegetables	24·2 (14·6–32·2)	25·8 (15·3–33·5)	31·2 (18·3–38·8)	31·2 (18·6–40·0)	0·8 (0·4–1·4)	0·9 (0·3–1·5)	0·0 (-0·9–0·9)
Diet low in legumes	38·0 (0·0–47·2)	38·0 (0·0–47·8)	42·8 (0·0–53·2)	43·3 (0·0–55·3)	0·4 (0·0–0·7)	0·6 (0·0–1·0)	0·1 (-0·6–0·9)
Diet low in whole grains	45·8 (37·9–52·2)	49·4 (41·3–56·1)	50·8 (42·4–58·4)	51·5 (43·1–59·1)	0·4 (0·1–0·7)	0·2 (-0·1–0·6)	0·1 (-0·4–0·7)
Diet low in nuts and seeds	12·4 (8·6–16·3)	7·1 (4·5–10·4)	5·7 (3·4–8·3)	5·9 (3·4–9·0)	-2·4 (-4·1–-0·8)	-0·8 (-3·4–1·7)	0·4 (-3·4–3·5)
Diet low in milk	36·3 (31·9–41·8)	33·3 (29·2–38·3)	32·8 (28·6–37·3)	32·4 (27·8–37·1)	-0·4 (-0·8–0·1)	-0·1 (-0·7–0·5)	-0·1 (-1·0–0·7)
Diet high in red meat	50·1 (0·0–68·9)	51·0 (0·0–69·6)	50·2 (0·0–68·2)	50·6 (0·0–70·4)	0·0 (-0·5–0·5)	0·0 (-0·8–0·6)	0·1 (-1·0–1·1)
Diet high in processed meat	47·2 (37·0–53·1)	59·5 (48·7–66·2)	65·9 (54·3–73·2)	64·1 (52·5–72·3)	1·0 (0·6–1·4)	0·4 (-0·2–0·9)	-0·2 (-1·1–0·5)
Diet high in sugar-sweetened beverages	37·1 (28·5–45·8)	52·4 (41·0–61·2)	55·7 (45·3–65·6)	55·8 (44·2–65·9)	1·3 (0·5–2·1)	0·3 (-0·8–1·2)	0·0 (-1·5–1·3)
Diet low in fibre	38·8 (21·4–46·0)	29·2 (15·7–35·8)	27·3 (15·0–33·8)	23·6 (13·8–30·8)	-1·6 (-2·5–-0·8)	-1·0 (-2·3–0·2)	-1·3 (-3·1–0·6)
Diet low in calcium	5·6 (4·5–6·9)	4·7 (3·8–5·8)	5·2 (4·2–6·4)	5·1 (4·1–6·3)	-0·3 (-0·5–-0·1)	0·4 (0·1–0·6)	-0·2 (-0·6–0·2)
Diet low in seafood omega-3 fatty acids	36·0 (26·9–46·1)	42·6 (32·6–53·3)	47·8 (37·4–59·1)	42·0 (31·9–53·1)	0·5 (0·0–1·0)	-0·1 (-0·6–0·5)	-1·2 (-1·9–-0·4)
Diet low in omega-6 polyunsaturated fatty acids	35·3 (23·1–45·4)	30·6 (20·8–39·4)	24·1 (16·7–31·3)	22·2 (15·3–29·4)	-1·5 (-2·2–-0·8)	-1·5 (-2·4–-0·7)	-0·8 (-1·9–0·2)
Diet high in trans fatty acids	71·4 (64·6–78·8)	71·8 (63·9–79·1)	63·4 (54·8–70·6)	0·0 (0·0–0·0)	-65·8 (-66·1–-65·4)	-97·1 (-97·6–-96·5)	-184·3 (-185·2–-182·9)
Diet high in sodium	23·5 (0·6–68·7)	28·3 (1·9–76·5)	31·4 (3·4–76·8)	31·7 (2·9–77·1)	1·0 (0·2–6·3)	0·5 (-0·2–3·6)	0·1 (-2·8–1·5)
Intimate partner violence	32·7 (19·7–42·4)	32·8 (21·3–41·2)	30·1 (20·8–36·9)	28·2 (17·8–37·4)	-0·5 (-1·7–0·8)	-0·7 (-2·4–1·0)	-0·6 (-2·8–1·4)
Childhood sexual abuse and bullying	9·6 (6·5–14·7)	11·5 (7·9–17·8)	13·2 (8·9–20·4)	10·4 (7·2–15·9)	0·3 (-0·3–0·8)	-0·5 (-1·2–0·3)	-2·1 (-3·6–-0·7)
Childhood sexual abuse	10·0 (8·4–11·7)	11·2 (9·8–12·8)	13·0 (11·5–14·9)	12·0 (9·8–14·8)	0·6 (-0·2–1·4)	0·3 (-0·7–1·3)	-0·8 (-2·2–0·8)
Bullying victimization	8·0 (3·5–15·9)	9·9 (4·5–19·4)	11·3 (5·1–22·2)	7·8 (3·4–15·2)	-0·1 (-0·8–0·7)	-1·1 (-2·1–-0·1)	-3·4 (-5·2–-1·6)
Unsafe sex	--	--	--	--	--	--	--
Low physical activity	20·4 (15·6–26·3)	19·9 (15·3–25·0)	19·5 (15·2–24·4)	22·2 (17·0–28·0)	0·3 (-0·6–1·3)	0·5 (-0·6–1·7)	1·2 (-0·6–3·0)
**Metabolic risks**	**27·8 (24·3–31·4)**	**32·2 (28·4–35·2)**	**35·5 (31·5–38·3)**	**39·5 (34·6–42·7)**	**1·1 (0·8–1·4)**	**1·0 (0·7–1·3)**	**1·0 (0·5–1·4)**
High fasting plasma glucose	13·3 (10·0–16·0)	17·6 (13·4–20·7)	22·6 (16·9–26·9)	25·9 (18·9–30·7)	2·2 (1·4–2·8)	1·8 (1·1–2·7)	1·2 (0·1–2·5)
High LDL cholesterol	63·5 (44·7–86·3)	54·0 (37·2–74·2)	48·3 (32·6–67·6)	46·8 (31·4–65·3)	-1·0 (-1·2–-0·8)	-0·7 (-0·9–-0·5)	-0·3 (-0·6–-0·1)
High systolic blood pressure	29·8 (19·9–41·7)	25·8 (18·2–35·2)	19·4 (12·8–27·8)	26·8 (17·4–36·8)	-0·3 (-1·3–0·5)	0·2 (-1·0–1·2)	3·0 (0·7–5·3)
High body-mass index	31·8 (27·0–36·4)	37·3 (32·0–41·0)	41·0 (35·2–44·6)	44·9 (38·3–48·5)	1·1 (0·8–1·4)	0·9 (0·6–1·2)	0·8 (0·4–1·3)
Low bone mineral density	21·5 (15·5–28·3)	19·4 (13·5–26·1)	20·3 (14·4–26·7)	22·4 (16·4–29·5)	0·1 (-0·4–0·7)	0·7 (0·0–1·4)	0·9 (-0·4–2·4)
Kidney dysfunction	2·9 (2·4–3·9)	3·0 (2·5–4·0)	3·0 (2·4–3·9)	3·1 (2·5–4·1)	0·2 (0·1–0·3)	0·1 (0·0–0·3)	0·4 (0·0–0·7)

Minnesota

Risk Names	SEV 1990	SEV 2000	SEV 2010	SEV 2021	Annualised rate of change 1990 to 2021	Annualised rate of change 2000 to 2021	Annualised rate of change 2010 to 2021
**All risk factors**	**26·1 (23·1–29·0)**	**25·1 (22·1–28·0)**	**26·0 (23·1–29·0)**	**26·9 (23·7–30·3)**	**0·1 (-0·2–0·4)**	**0·3 (-0·1–0·7)**	**0·3 (-0·3–0·8)**
**Environmental/occupational risks**	**17·1 (11·2–27·7)**	**16·1 (10·7–26·4)**	**14·3 (9·2–23·8)**	**13·0 (8·4–21·8)**	**-0·9 (-1·6–-0·3)**	**-1·0 (-1·4–-0·5)**	**-0·8 (-1·3–-0·3)**
Unsafe water, sanitation, and handwashing	1·6 (0·8–2·6)	1·5 (0·6–2·6)	1·2 (0·4–2·1)	1·0 (0·3–1·9)	-1·5 (-4·0–0·6)	-1·9 (-5·4–0·7)	-1·7 (-5·5–2·3)
Unsafe water source	2·3 (0·9–4·7)	2·1 (0·7–4·3)	0·9 (0·4–1·7)	0·7 (0·3–1·4)	-3·7 (-6·4–-0·8)	-5·2 (-8·9–-1·3)	-1·8 (-7·2–4·0)
Unsafe sanitation	4·7 (2·6–7·9)	3·8 (2·0–6·5)	2·9 (1·6–5·2)	2·2 (1·1–3·9)	-2·4 (-4·9–-0·2)	-2·5 (-5·6–0·4)	-2·4 (-5·9–0·9)
No access to handwashing facility	1·5 (0·4–2·9)	1·6 (0·5–2·9)	1·6 (0·5–2·9)	1·4 (0·5–2·9)	-0·3 (-3·3–2·9)	-0·6 (-4·8–3·8)	-1·4 (-7·0–4·1)
Air pollution	20·5 (4·5–31·3)	18·1 (5·3–26·6)	11·3 (3·1–21·6)	7·2 (1·5–16·3)	-3·4 (-6·8–-1·2)	-4·4 (-8·6–-2·0)	-4·1 (-9·4–-2·3)
Particulate matter pollution	9·1 (2·3–18·1)	7·7 (4·8–11·2)	5·2 (2·5–8·3)	3·2 (0·9–6·2)	-3·3 (-7·2–0·3)	-4·2 (-8·1–-2·6)	-4·3 (-10·3–-2·2)
Ambient particulate matter pollution	14·0 (3·4–27·8)	11·8 (7·5–16·7)	7·9 (3·8–12·5)	4·9 (1·4–9·2)	-3·4 (-7·3–0·2)	-4·2 (-8·1–-2·6)	-4·3 (-10·3–-2·2)
Household air pollution from solid fuels	0·0 (0·0–0·0)	0·0 (0·0–0·0)	0·0 (0·0–0·0)	0·0 (0·0–0·0)	-3·9 (-38·6–-7·4)	-4·0 (-35·3–-7·7)	-4·8 (-30·2–-5·2)
Ambient ozone pollution	9·1 (5·1–15·0)	13·5 (8·5–20·3)	12·8 (7·9–19·5)	7·4 (3·9–12·5)	-0·7 (-1·5–0·0)	-2·9 (-4·4–-1·7)	-5·0 (-7·8–-3·1)
Ambient nitrogen dioxide pollution	71·1 (0·0–100·0)	63·7 (0·0–100·0)	37·9 (0·0–91·9)	24·5 (0·0–72·4)	-3·4 (-9·6–0·0)	-4·5 (-12·8–0·0)	-4·0 (-14·5–0·0)
Non-optimal temperature	29·8 (24·4–38·2)	27·0 (22·7–34·4)	28·8 (22·8–37·4)	30·7 (24·1–40·2)	0·1 (-0·2–0·4)	0·6 (0·1–1·2)	0·6 (0·2–1·0)
High temperature	22·6 (12·3–34·5)	18·9 (9·8–30·1)	26·3 (14·8–38·6)	32·7 (19·6–46·6)	1·2 (0·6–1·9)	2·6 (1·5–3·8)	2·0 (0·9–3·3)
Low temperature	28·7 (25·6–32·6)	26·3 (23·8–30·1)	26·8 (24·1–30·7)	28·1 (25·4–31·8)	-0·1 (-0·2–0·1)	0·3 (0·1–0·5)	0·4 (0·1–0·7)
Other environmental risks	27·8 (8·6–48·9)	26·3 (8·6–47·0)	23·9 (8·6–44·6)	21·6 (8·6–42·1)	-0·8 (-1·6–0·0)	-0·9 (-1·9–0·0)	-0·9 (-2·1–0·0)
Residential radon	33·8 (0·0–100·0)	33·8 (0·0–100·0)	33·8 (0·0–100·0)	33·8 (0·0–100·0)	0·0 (0·0–0·0)	0·0 (0·0–0·0)	0·0 (0·0–0·0)
Lead exposure	25·1 (0·0–31·6)	22·9 (0·0–28·8)	19·5 (0·0–24·3)	16·1 (0·0–19·9)	-1·4 (-1·9–0·0)	-1·7 (-2·2–0·0)	-1·7 (-2·6–0·0)
Occupational risks	3·2 (2·6–4·2)	3·3 (2·7–4·3)	3·1 (2·6–4·1)	3·1 (2·5–4·1)	-0·1 (-0·3–0·2)	-0·3 (-0·6–0·0)	-0·1 (-0·6–0·3)
Occupational carcinogens	1·1 (0·5–2·0)	1·2 (0·6–2·2)	1·1 (0·5–2·2)	1·1 (0·5–2·2)	0·1 (-0·2–0·4)	-0·1 (-0·6–0·2)	0·0 (-0·7–0·5)
Occupational exposure to asbestos	5·5 (4·9–6·0)	5·9 (5·4–6·4)	5·3 (4·7–5·8)	4·9 (3·9–5·9)	-0·4 (-1·1–0·2)	-0·9 (-1·9–0·1)	-0·8 (-2·7–1·1)
Occupational exposure to arsenic	0·6 (0·0–1·4)	0·6 (0·0–1·4)	0·5 (0·0–1·3)	0·5 (0·0–1·2)	-0·2 (-0·6–0·3)	-0·4 (-1·1–0·2)	-0·1 (-1·1–0·8)
Occupational exposure to benzene	1·2 (0·1–3·3)	1·3 (0·1–3·8)	1·3 (0·1–3·8)	1·3 (0·1–3·9)	0·4 (0·0–0·7)	0·1 (-0·3–0·5)	0·2 (-0·4–0·7)
Occupational exposure to beryllium	0·0 (0·0–0·0)	0·0 (0·0–0·0)	0·0 (0·0–0·0)	0·0 (0·0–0·0)	0·2 (0·0–0·4)	0·1 (-0·2–0·4)	0·3 (-0·1–0·7)
Occupational exposure to cadmium	0·1 (0·1–0·1)	0·1 (0·1–0·1)	0·1 (0·1–0·1)	0·1 (0·1–0·1)	-0·1 (-0·5–0·3)	-0·3 (-0·8–0·2)	0·0 (-0·8–0·7)
Occupational exposure to chromium	0·2 (0·1–0·2)	0·2 (0·2–0·2)	0·2 (0·2–0·2)	0·2 (0·1–0·2)	0·0 (-0·3–0·4)	-0·2 (-0·7–0·2)	0·0 (-0·6–0·7)
Occupational exposure to diesel engine exhaust	0·7 (0·7–0·8)	0·8 (0·7–0·8)	0·8 (0·7–0·8)	0·8 (0·8–0·9)	0·4 (0·0–0·7)	0·3 (-0·2–0·8)	0·6 (-0·1–1·3)
Occupational exposure to formaldehyde	0·3 (0·3–0·4)	0·3 (0·3–0·4)	0·3 (0·3–0·3)	0·3 (0·3–0·3)	-0·2 (-0·5–0·3)	-0·4 (-0·9–0·2)	-0·2 (-0·9–0·6)
Occupational exposure to nickel	0·4 (0·0–1·8)	0·4 (0·0–1·8)	0·4 (0·0–1·7)	0·4 (0·0–1·6)	-0·2 (-0·8–0·3)	-0·4 (-1·2–0·3)	-0·1 (-1·1–0·9)
Occupational exposure to polycyclic aromatic hydrocarbons	0·3 (0·3–0·3)	0·3 (0·3–0·4)	0·3 (0·3–0·3)	0·3 (0·3–0·4)	0·0 (-0·3–0·4)	-0·2 (-0·6–0·2)	0·0 (-0·6–0·7)
Occupational exposure to silica	3·8 (0·6–12·3)	3·8 (0·6–12·0)	3·5 (0·6–11·2)	3·5 (0·6–11·0)	-0·3 (-0·8–0·2)	-0·4 (-1·0–0·3)	0·0 (-0·9–0·9)
Occupational exposure to sulphuric acid	0·8 (0·2–2·7)	0·9 (0·2–2·9)	0·8 (0·2–2·7)	0·8 (0·2–2·6)	-0·1 (-0·5–0·3)	-0·3 (-0·9–0·3)	0·0 (-0·8–0·8)
Occupational exposure to trichloroethylene	0·1 (0·1–0·1)	0·1 (0·1–0·1)	0·1 (0·1–0·1)	0·1 (0·1–0·1)	0·1 (-0·1–0·4)	0·0 (-0·4–0·4)	0·2 (-0·2–0·8)
Occupational asthmagens	18·4 (16·2–20·8)	19·3 (17·0–21·8)	18·2 (16·1–20·6)	18·2 (16·0–20·7)	-0·1 (-0·4–0·3)	-0·3 (-0·7–0·2)	0·0 (-0·7–0·6)
Occupational particulate matter, gases, and fumes	7·3 (5·6–9·6)	7·1 (5·5–9·4)	6·8 (5·2–8·9)	6·3 (4·9–8·3)	-0·4 (-0·7–-0·2)	-0·6 (-0·9–-0·3)	-0·7 (-1·0–-0·3)
Occupational noise	7·4 (6·9–8·0)	7·5 (7·0–8·1)	7·4 (7·0–8·0)	7·2 (6·8–7·8)	-0·1 (-0·2–0·1)	-0·2 (-0·3–0·0)	-0·3 (-0·5–-0·1)
Occupational injuries	--	--	--	--	--	--	--
Occupational ergonomic factors	8·6 (7·2–10·3)	8·8 (7·4–10·5)	8·1 (6·8–9·8)	8·2 (6·8–10·0)	-0·2 (-0·6–0·2)	-0·3 (-0·9–0·2)	0·1 (-0·6–0·8)
**Behavioural risks**	**30·8 (27·3–34·5)**	**27·6 (24·4–31·4)**	**26·1 (23·1–29·5)**	**24·9 (21·7–28·1)**	**-0·7 (-1·0–-0·4)**	**-0·5 (-1·0–-0·1)**	**-0·4 (-1·1–0·3)**
Child and maternal malnutrition	9·6 (6·4–13·5)	8·3 (5·4–11·8)	8·4 (5·7–11·6)	8·6 (5·7–12·1)	-0·3 (-1·3–0·6)	0·2 (-1·0–1·5)	0·3 (-1·4–2·0)
Suboptimal breastfeeding	68·6 (66·1–71·3)	66·5 (63·9–69·0)	62·7 (59·9–65·4)	62·0 (59·2–65·2)	-0·3 (-0·5–-0·2)	-0·3 (-0·5–-0·1)	-0·1 (-0·4–0·2)
Non-exclusive breastfeeding	63·7 (56·2–71·5)	59·1 (52·2–65·5)	48·2 (41·7–55·7)	45·9 (38·9–53·1)	-1·1 (-1·6–-0·6)	-1·2 (-1·9–-0·6)	-0·4 (-1·5–0·5)
Discontinued breastfeeding	88·6 (85·9–91·0)	86·2 (83·3–88·9)	84·2 (81·1–87·6)	84·5 (81·2–88·1)	-0·2 (-0·3–0·0)	-0·1 (-0·3–0·1)	0·0 (-0·3–0·3)
Child growth failure	1·1 (0·4–2·5)	1·1 (0·4–2·3)	0·9 (0·3–2·0)	0·9 (0·3–1·8)	-0·8 (-1·3–-0·5)	-1·0 (-1·7–-0·6)	-0·7 (-1·4–0·2)
Child underweight	2·3 (1·5–3·2)	2·2 (1·4–3·0)	1·9 (1·2–2·6)	1·8 (1·2–2·4)	-0·9 (-1·4–-0·4)	-1·1 (-1·8–-0·5)	-0·6 (-1·7–0·5)
Child wasting	0·8 (0·5–1·2)	0·7 (0·5–1·2)	0·8 (0·5–1·2)	0·7 (0·5–1·1)	-0·2 (-0·6–0·1)	-0·1 (-0·6–0·3)	-0·3 (-1·0–0·4)
Child stunting	3·3 (2·6–4·1)	3·1 (2·5–3·9)	2·7 (2·1–3·4)	2·4 (1·8–3·1)	-1·0 (-1·6–-0·5)	-1·2 (-2·1–-0·5)	-0·9 (-2·2–0·2)
Low birth weight and short gestation	19·5 (16·9–22·2)	21·0 (18·1–24·0)	22·5 (19·7–26·1)	23·1 (20·2–26·6)	0·6 (0·3–0·9)	0·5 (0·0–0·9)	0·3 (-0·5–0·9)
Short gestation	40·7 (35·6–46·7)	42·9 (37·1–48·8)	42·2 (36·8–48·2)	42·7 (37·3–48·7)	0·2 (-0·1–0·5)	0·0 (-0·5–0·4)	0·1 (-0·8–0·9)
Low birth weight	14·6 (13·1–16·1)	15·7 (14·0–17·3)	17·5 (15·8–19·3)	18·0 (16·2–19·9)	0·7 (0·4–0·9)	0·7 (0·3–1·0)	0·3 (-0·5–1·0)

Iron deficiency	3·4 (2·3–4·7)	2·9 (2·0–4·1)	3·0 (2·1–4·0)	3·0 (2·1–4·2)	-0·3 (-1·2–0·6)	0·2 (-1·0–1·5)	0·2 (-1·5–1·8)
Vitamin A deficiency	0·7 (0·0–1·6)	1·0 (0·0–2·1)	0·6 (0·0–1·3)	0·4 (0·0–0·8)	-2·4 (-4·5–0·0)	-4·8 (-7·7–0·0)	-4·8 (-8·8–0·0)
Zinc deficiency	1·8 (0·0–7·9)	1·0 (0·0–4·5)	0·9 (0·0–3·9)	0·9 (0·0–3·7)	-2·2 (-47·4–0·2)	-0·6 (-5·3–2·9)	0·0 (-4·1–9·8)
Tobacco	39·1 (37·5–40·5)	32·7 (31·4–33·9)	27·7 (26·7–28·7)	23·6 (22·0–25·2)	-1·6 (-1·8–-1·4)	-1·5 (-1·9–-1·2)	-1·4 (-2·1–-0·9)
Smoking	37·1 (34·9–39·2)	30·3 (28·3–32·2)	25·7 (24·2–27·2)	21·8 (19·8–23·7)	-1·7 (-2·0–-1·4)	-1·6 (-2·0–-1·2)	-1·5 (-2·2–-0·7)
Chewing tobacco	1·8 (1·5–2·4)	2·0 (1·5–2·6)	2·2 (1·6–3·0)	2·2 (1·6–3·0)	0·6 (-0·6–1·7)	0·4 (-1·2–2·1)	0·2 (-2·4–3·0)
Second-hand smoke	31·1 (29·4–32·5)	26·4 (24·9–27·5)	22·3 (20·8–23·3)	19·4 (17·8–21·0)	-1·5 (-1·8–-1·3)	-1·5 (-1·8–-1·1)	-1·3 (-2·0–-0·7)
High alcohol use	31·2 (22·1–42·2)	29·1 (21·2–39·8)	30·0 (22·6–40·0)	30·6 (22·8–39·7)	-0·1 (-1·0–0·9)	0·2 (-1·1–1·4)	0·2 (-1·6–1·9)
Drug use	0·6 (0·3–1·1)	0·7 (0·5–0·9)	1·0 (0·9–1·2)	3·1 (2·1–3·9)	5·4 (2·4–8·1)	7·2 (4·4–9·0)	10·0 (6·5–12·2)
Dietary risks	35·4 (26·3–46·2)	38·6 (29·4–50·5)	41·5 (32·0–53·8)	41·6 (31·8–53·9)	0·5 (0·3–0·8)	0·4 (0·1–0·6)	0·0 (-0·4–0·5)
Diet low in fruits	34·7 (29·8–38·2)	32·4 (27·9–35·3)	33·0 (28·4–35·9)	33·7 (28·0–38·1)	-0·1 (-0·5–0·3)	0·2 (-0·4–0·8)	0·2 (-0·7–1·0)
Diet low in vegetables	22·6 (13·8–30·3)	24·7 (14·3–32·5)	29·9 (16·5–37·3)	29·6 (16·8–37·4)	0·9 (0·4–1·4)	0·8 (0·4–1·4)	-0·1 (-0·9–0·7)
Diet low in legumes	37·7 (0·0–47·4)	37·7 (0·0–47·7)	41·6 (0·0–52·2)	42·0 (0·0–53·5)	0·4 (0·0–0·7)	0·5 (0·0–1·0)	0·1 (-0·6–0·8)
Diet low in whole grains	44·9 (37·6–50·9)	48·1 (40·6–55·0)	50·1 (42·1–57·3)	50·5 (42·0–57·8)	0·4 (0·1–0·6)	0·2 (-0·1–0·6)	0·1 (-0·5–0·6)
Diet low in nuts and seeds	12·1 (8·8–16·1)	6·7 (4·1–9·6)	4·7 (2·8–7·3)	4·9 (2·8–8·0)	-2·9 (-4·6–-1·3)	-1·5 (-4·2–0·8)	0·4 (-3·3–3·9)
Diet low in milk	35·5 (31·5–39·9)	32·2 (28·2–36·9)	30·6 (26·3–35·1)	30·1 (25·8–35·1)	-0·5 (-1·0–-0·1)	-0·3 (-0·9–0·3)	-0·2 (-1·1–0·8)
Diet high in red meat	49·7 (0·0–67·9)	50·4 (0·0–68·2)	50·9 (0·0–69·6)	51·2 (0·0–69·8)	0·1 (-0·4–0·5)	0·1 (-0·7–0·7)	0·1 (-0·9–0·8)
Diet high in processed meat	43·2 (34·5–48·9)	54·4 (44·7–60·6)	61·9 (51·8–69·1)	60·5 (49·0–68·8)	1·1 (0·6–1·6)	0·5 (-0·1–1·0)	-0·2 (-1·0–0·6)
Diet high in sugar-sweetened beverages	36·9 (28·4–44·0)	51·8 (40·9–59·6)	57·8 (47·0–66·7)	58·0 (45·9–67·3)	1·5 (0·8–2·2)	0·5 (-0·4–1·4)	0·0 (-1·2–1·2)
Diet low in fibre	38·0 (21·3–45·5)	28·2 (15·5–35·4)	24·7 (14·0–31·3)	21·3 (12·5–27·8)	-1·9 (-3·0–-0·9)	-1·3 (-2·8–-0·1)	-1·4 (-3·3–0·5)
Diet low in calcium	5·4 (4·4–6·7)	4·6 (3·7–5·6)	4·8 (3·8–5·9)	4·7 (3·8–5·8)	-0·5 (-0·7–-0·3)	0·1 (-0·1–0·4)	-0·2 (-0·5–0·1)
Diet low in seafood omega-3 fatty acids	35·3 (26·2–45·0)	41·7 (32·1–52·2)	44·7 (34·9–55·7)	39·1 (29·8–49·3)	0·3 (-0·1–0·8)	-0·3 (-0·9–0·3)	-1·2 (-2·0–-0·4)
Diet low in omega-6 polyunsaturated fatty acids	31·0 (20·7–40·2)	27·7 (19·1–36·0)	21·1 (15·0–27·3)	19·5 (13·5–25·6)	-1·5 (-2·2–-0·8)	-1·7 (-2·6–-0·8)	-0·7 (-1·9–0·1)
Diet high in trans fatty acids	78·1 (71·3–84·8)	76·1 (69·0–83·2)	63·7 (55·8–71·1)	0·0 (0·0–0·0)	-66·1 (-66·3–-65·8)	-97·4 (-97·8–-96·9)	-184·3 (-185·3–-183·1)
Diet high in sodium	23·0 (0·7–66·9)	27·7 (1·9–73·3)	30·6 (3·3–74·0)	31·0 (3·2–77·0)	1·0 (0·2–6·3)	0·5 (-0·1–3·9)	0·1 (-2·2–1·9)
Intimate partner violence	27·7 (17·6–35·7)	28·0 (19·4–34·5)	23·2 (15·9–28·6)	22·2 (12·1–30·3)	-0·7 (-2·3–0·8)	-1·1 (-3·2–0·6)	-0·4 (-3·0–1·6)
Childhood sexual abuse and bullying	6·5 (3·9–10·8)	6·2 (3·9–10·3)	6·3 (4·3–10·2)	6·0 (4·2–9·2)	-0·2 (-0·9–0·5)	-0·1 (-1·0–0·8)	-0·5 (-1·9–0·9)
Childhood sexual abuse	5·3 (4·4–6·2)	6·0 (5·3–6·9)	6·9 (6·3–7·6)	6·6 (5·5–7·9)	0·7 (0·0–1·5)	0·5 (-0·5–1·5)	-0·4 (-1·9–1·2)
Bullying victimization	6·3 (2·6–12·7)	5·5 (2·2–11·3)	5·1 (2·2–10·4)	4·7 (2·1–9·1)	-0·9 (-1·7–-0·2)	-0·7 (-1·8–0·4)	-0·8 (-2·7–1·0)
Unsafe sex	--	--	--	--	--	--	--
Low physical activity	20·7 (15·7–26·7)	20·4 (15·6–25·5)	21·4 (16·9–26·5)	23·3 (17·9–30·2)	0·4 (-0·6–1·4)	0·6 (-0·5–1·9)	0·8 (-0·9–2·6)
**Metabolic risks**	**24·5 (21·5–27·8)**	**30·3 (26·7–33·5)**	**33·1 (29·2–36·0)**	**36·5 (31·9–39·6)**	**1·3 (1·0–1·6)**	**0·9 (0·6–1·2)**	**0·9 (0·5–1·3)**
High fasting plasma glucose	12·0 (9·2–14·8)	15·4 (11·6–18·3)	19·6 (14·8–23·6)	23·4 (17·6–28·3)	2·1 (1·4–2·8)	2·0 (1·0–2·8)	1·6 (0·3–2·9)
High LDL cholesterol	63·6 (44·8–86·6)	53·7 (36·7–74·2)	48·0 (32·3–66·7)	46·6 (31·2–65·0)	-1·0 (-1·2–-0·8)	-0·7 (-0·9–-0·5)	-0·3 (-0·5–0·0)
High systolic blood pressure	28·0 (18·8–39·2)	23·7 (16·7–32·5)	16·7 (10·8–24·0)	23·5 (15·1–33·5)	-0·6 (-1·6–0·4)	0·0 (-1·2–1·0)	3·1 (0·9–5·6)
High body-mass index	28·0 (23·6–32·3)	34·9 (29·8–39·3)	38·2 (32·7–42·1)	41·5 (35·2–45·3)	1·3 (0·9–1·6)	0·8 (0·5–1·1)	0·8 (0·3–1·2)
Low bone mineral density	24·7 (18·2–31·9)	24·6 (18·3–31·8)	25·1 (18·2–32·8)	26·3 (20·0–34·2)	0·2 (-0·2–0·7)	0·3 (-0·3–1·1)	0·4 (-0·7–1·8)
Kidney dysfunction	2·6 (2·2–3·5)	2·7 (2·2–3·6)	2·7 (2·2–3·6)	2·8 (2·3–3·8)	0·2 (0·1–0·3)	0·2 (0·0–0·4)	0·3 (0·0–0·7)

Mississippi

Risk Names	SEV 1990	SEV 2000	SEV 2010	SEV 2021	Annualised rate of change 1990 to 2021	Annualised rate of change 2000 to 2021	Annualised rate of change 2010 to 2021
**All risk factors**	**26·2 (23·6–29·4)**	**26·0 (23·3–28·9)**	**26·6 (23·7–29·6)**	**26·2 (23·3–29·2)**	**0·0 (-0·3–0·2)**	**0·0 (-0·3–0·3)**	**-0·1 (-0·6–0·3)**
**Environmental/occupational risks**	**17·1 (11·4–25·6)**	**17·3 (11·9–25·5)**	**15·1 (10·1–22·5)**	**11·8 (7·7–17·0)**	**-1·2 (-1·9–-0·4)**	**-1·8 (-2·5–-1·3)**	**-2·2 (-3·1–-1·6)**
Unsafe water, sanitation, and handwashing	2·7 (1·5–4·4)	2·4 (1·2–4·0)	1·8 (0·7–2·9)	1·4 (0·6–2·3)	-2·2 (-4·4–-0·2)	-2·8 (-5·8–0·0)	-2·3 (-6·2–1·6)
Unsafe water source	3·8 (1·4–8·0)	3·5 (1·2–6·9)	1·4 (0·5–2·7)	1·0 (0·4–1·8)	-4·2 (-7·1–-1·4)	-5·8 (-9·7–-1·9)	-2·6 (-7·9–3·0)
Unsafe sanitation	10·1 (5·6–16·0)	7·9 (4·3–13·0)	5·9 (3·1–10·0)	4·1 (2·2–7·2)	-2·9 (-4·9–-0·8)	-3·1 (-5·6–-0·6)	-3·2 (-6·6–0·2)
No access to handwashing facility	2·0 (0·6–3·8)	2·0 (0·6–3·9)	1·9 (0·6–3·5)	1·6 (0·5–3·2)	-0·7 (-3·6–2·6)	-1·1 (-5·0–3·2)	-1·7 (-7·6–4·2)
Air pollution	16·1 (6·8–26·9)	15·3 (9·8–23·8)	9·0 (4·8–16·7)	5·6 (2·2–12·1)	-3·4 (-5·9–-1·5)	-4·8 (-7·5–-3·1)	-4·3 (-8·3–-2·3)
Particulate matter pollution	13·6 (5·2–23·7)	13·2 (9·5–17·5)	8·1 (4·8–11·8)	4·7 (2·1–8·0)	-3·4 (-6·3–-0·6)	-4·9 (-7·7–-3·3)	-4·8 (-8·9–-2·7)
Ambient particulate matter pollution	21·1 (8·1–37·6)	20·4 (15·0–26·1)	12·3 (7·5–17·5)	7·2 (3·2–11·9)	-3·5 (-6·3–-0·6)	-4·9 (-7·8–-3·3)	-4·9 (-9·0–-2·7)
Household air pollution from solid fuels	0·0 (0·0–0·2)	0·0 (0·0–0·1)	0·0 (0·0–0·0)	0·0 (0·0–0·0)	-5·4 (-27·1–-5·8)	-5·6 (-24·1–-5·7)	-4·9 (-18·5–-3·7)
Ambient ozone pollution	20·0 (14·8–27·4)	27·0 (20·9–35·5)	16·0 (11·0–22·7)	6·1 (3·0–10·3)	-3·8 (-5·3–-2·8)	-7·1 (-9·6–-5·4)	-8·8 (-12·7–-6·0)
Ambient nitrogen dioxide pollution	22·9 (0·0–68·0)	19·3 (0·0–62·6)	9·8 (0·0–45·4)	7·8 (0·0–40·7)	-3·5 (-13·3–0·0)	-4·3 (-17·4–0·0)	-2·1 (-7·9–0·0)
Non-optimal temperature	31·1 (23·6–40·9)	35·1 (27·6–45·3)	39·1 (31·2–49·2)	27·7 (22·2–36·1)	-0·4 (-0·7–0·0)	-1·1 (-1·5–-0·8)	-3·1 (-3·8–-2·3)
High temperature	25·6 (17·2–35·4)	32·2 (23·5–42·2)	33·3 (24·2–43·3)	20·7 (14·3–29·1)	-0·7 (-1·0–-0·3)	-2·1 (-2·9–-1·4)	-4·3 (-5·6–-3·0)
Low temperature	26·1 (22·5–30·2)	29·0 (25·7–32·7)	33·4 (30·2–37·0)	25·0 (22·0–28·8)	-0·1 (-0·4–0·1)	-0·7 (-1·0–-0·5)	-2·6 (-3·1–-2·1)
Other environmental risks	26·6 (7·5–43·5)	25·1 (7·5–41·4)	22·8 (7·5–38·6)	19·8 (7·5–35·5)	-1·0 (-1·6–0·0)	-1·1 (-1·8–0·0)	-1·3 (-2·2–0·0)
Residential radon	22·2 (0·0–70·6)	22·2 (0·0–70·6)	22·2 (0·0–70·6)	22·2 (0·0–70·6)	0·0 (0·0–0·0)	0·0 (0·0–0·0)	0·0 (0·0–0·0)
Lead exposure	28·6 (0·0–35·7)	26·4 (0·0–32·4)	23·0 (0·0–28·5)	18·7 (0·0–23·3)	-1·4 (-1·8–0·0)	-1·6 (-2·1–0·0)	-1·9 (-2·7–0·0)
Occupational risks	2·8 (2·3–3·6)	2·8 (2·3–3·7)	2·5 (2·1–3·3)	2·6 (2·1–3·4)	-0·2 (-0·5–0·1)	-0·4 (-0·8–0·0)	0·1 (-0·4–0·6)
Occupational carcinogens	0·9 (0·4–1·6)	0·9 (0·4–1·8)	0·9 (0·4–1·7)	0·9 (0·4–1·8)	0·2 (-0·2–0·5)	0·0 (-0·4–0·4)	0·4 (-0·2–1·0)
Occupational exposure to asbestos	3·2 (2·9–3·6)	3·1 (2·8–3·4)	3·1 (2·8–3·4)	3·2 (2·4–4·1)	0·0 (-0·8–0·8)	0·1 (-1·0–1·4)	0·3 (-2·1–2·5)
Occupational exposure to arsenic	0·5 (0·0–1·2)	0·5 (0·0–1·2)	0·5 (0·0–1·1)	0·5 (0·0–1·1)	-0·4 (-0·8–0·2)	-0·6 (-1·2–0·1)	0·0 (-0·8–1·0)
Occupational exposure to benzene	1·0 (0·1–2·8)	1·1 (0·1–3·1)	1·0 (0·1–3·0)	1·1 (0·1–3·2)	0·4 (0·0–0·8)	0·2 (-0·3–0·7)	0·6 (-0·2–1·2)
Occupational exposure to beryllium	0·0 (0·0–0·0)	0·0 (0·0–0·0)	0·0 (0·0–0·0)	0·0 (0·0–0·0)	0·1 (-0·2–0·4)	0·0 (-0·4–0·4)	0·6 (0·1–1·1)
Occupational exposure to cadmium	0·1 (0·1–0·1)	0·1 (0·1–0·1)	0·1 (0·0–0·1)	0·1 (0·0–0·1)	-0·3 (-0·7–0·2)	-0·5 (-1·0–0·1)	0·1 (-0·6–1·0)
Occupational exposure to chromium	0·1 (0·1–0·2)	0·1 (0·1–0·2)	0·1 (0·1–0·1)	0·1 (0·1–0·1)	-0·2 (-0·5–0·2)	-0·4 (-0·8–0·1)	0·2 (-0·4–1·0)
Occupational exposure to diesel engine exhaust	0·6 (0·6–0·7)	0·6 (0·6–0·7)	0·6 (0·6–0·6)	0·7 (0·6–0·7)	0·2 (-0·2–0·6)	0·2 (-0·3–0·8)	0·9 (0·2–1·6)
Occupational exposure to formaldehyde	0·3 (0·3–0·3)	0·3 (0·3–0·3)	0·3 (0·2–0·3)	0·3 (0·2–0·3)	-0·3 (-0·7–0·2)	-0·5 (-1·1–0·0)	0·1 (-0·7–0·9)
Occupational exposure to nickel	0·4 (0·0–1·6)	0·4 (0·0–1·6)	0·4 (0·0–1·4)	0·4 (0·0–1·4)	-0·5 (-1·0–0·1)	-0·7 (-1·4–0·1)	0·0 (-1·0–1·1)
Occupational exposure to polycyclic aromatic hydrocarbons	0·3 (0·3–0·3)	0·3 (0·3–0·3)	0·3 (0·3–0·3)	0·3 (0·3–0·3)	-0·1 (-0·5–0·3)	-0·3 (-0·8–0·2)	0·3 (-0·4–0·9)
Occupational exposure to silica	3·8 (0·6–12·4)	3·7 (0·6–11·8)	3·2 (0·5–10·0)	3·3 (0·5–10·1)	-0·5 (-1·0–0·0)	-0·6 (-1·2–0·0)	0·1 (-0·8–1·1)
Occupational exposure to sulphuric acid	0·7 (0·1–2·4)	0·8 (0·2–2·5)	0·7 (0·1–2·1)	0·7 (0·1–2·2)	-0·3 (-0·7–0·2)	-0·5 (-1·1–0·1)	0·2 (-0·7–1·0)
Occupational exposure to trichloroethylene	0·1 (0·1–0·1)	0·1 (0·1–0·1)	0·1 (0·1–0·1)	0·1 (0·1–0·1)	0·0 (-0·3–0·3)	-0·2 (-0·6–0·3)	0·5 (-0·1–1·1)
Occupational asthmagens	16·6 (14·5–19·1)	16·8 (14·8–19·0)	14·8 (13·0–16·7)	15·3 (13·4–17·5)	-0·3 (-0·7–0·1)	-0·5 (-1·0–0·1)	0·3 (-0·4–1·0)
Occupational particulate matter, gases, and fumes	6·6 (5·1–8·6)	6·4 (5·0–8·3)	6·0 (4·7–7·7)	5·4 (4·2–7·0)	-0·6 (-0·9–-0·4)	-0·8 (-1·1–-0·5)	-0·9 (-1·3–-0·5)
Occupational noise	6·6 (6·0–7·3)	6·6 (6·1–7·2)	6·4 (6·0–7·0)	6·1 (5·7–6·7)	-0·2 (-0·4–-0·1)	-0·4 (-0·6–-0·2)	-0·4 (-0·7–-0·2)
Occupational injuries	--	--	--	--	--	--	--
Occupational ergonomic factors	8·3 (6·8–10·0)	8·1 (6·8–9·7)	6·9 (5·8–8·2)	7·2 (6·0–8·9)	-0·4 (-1·0–0·1)	-0·5 (-1·1–0·1)	0·4 (-0·4–1·1)
**Behavioural risks**	**28·9 (25·9–32·4)**	**27·4 (24·3–30·8)**	**26·9 (23·9–30·4)**	**25·0 (22·0–28·4)**	**-0·5 (-0·8–-0·2)**	**-0·4 (-0·9–0·0)**	**-0·7 (-1·3–-0·1)**
Child and maternal malnutrition	10·8 (7·4–15·4)	9·5 (6·6–13·1)	9·6 (6·6–13·1)	9·8 (6·5–14·0)	-0·3 (-1·1–0·4)	0·1 (-1·0–1·2)	0·1 (-1·5–1·7)
Suboptimal breastfeeding	69·1 (66·1–71·8)	66·9 (64·5–69·5)	63·0 (60·2–65·7)	62·4 (59·4–65·3)	-0·3 (-0·5–-0·2)	-0·3 (-0·5–-0·1)	-0·1 (-0·4–0·2)
Non-exclusive breastfeeding	64·9 (56·9–72·4)	60·1 (53·1–67·1)	49·1 (42·8–56·0)	46·7 (39·9–54·3)	-1·1 (-1·6–-0·6)	-1·2 (-1·9–-0·6)	-0·5 (-1·4–0·5)
Discontinued breastfeeding	88·8 (86·0–91·4)	86·4 (83·5–89·4)	84·5 (81·1–87·7)	84·7 (81·4–88·2)	-0·2 (-0·3–0·0)	-0·1 (-0·3–0·1)	0·0 (-0·3–0·3)
Child growth failure	1·6 (0·5–3·6)	1·5 (0·5–3·4)	1·3 (0·4–2·9)	1·2 (0·4–2·7)	-0·9 (-1·3–-0·5)	-1·0 (-1·6–-0·6)	-0·7 (-1·4–0·0)
Child underweight	3·3 (2·1–4·7)	3·1 (2·1–4·3)	2·7 (1·6–3·7)	2·5 (1·5–3·4)	-0·9 (-1·4–-0·4)	-1·1 (-1·8–-0·4)	-0·6 (-1·6–0·4)
Child wasting	1·0 (0·6–1·6)	1·0 (0·6–1·5)	1·0 (0·6–1·5)	0·9 (0·6–1·4)	-0·3 (-0·6–0·0)	-0·2 (-0·6–0·2)	-0·3 (-1·0–0·3)
Child stunting	4·4 (3·4–5·5)	4·2 (3·3–5·3)	3·6 (2·8–4·6)	3·3 (2·5–4·2)	-1·0 (-1·5–-0·4)	-1·1 (-1·9–-0·4)	-0·9 (-2·0–0·4)
Low birth weight and short gestation	23·7 (20·7–27·2)	26·3 (23·1–29·9)	30·0 (26·4–34·4)	28·9 (25·3–32·9)	0·6 (0·4–0·9)	0·5 (0·1–0·8)	-0·3 (-1·0–0·3)
Short gestation	40·6 (35·3–46·3)	45·5 (39·9–51·4)	49·1 (43·0–55·7)	47·2 (41·8–53·9)	0·5 (0·2–0·8)	0·2 (-0·3–0·6)	-0·4 (-1·2–0·4)
Low birth weight	17·6 (15·7–19·5)	19·1 (17·0–21·2)	22·2 (20·1–24·4)	21·6 (19·5–23·7)	0·7 (0·4–0·9)	0·6 (0·2–0·9)	-0·3 (-0·9–0·3)

Iron deficiency	4·0 (2·8–5·5)	3·5 (2·5–4·7)	3·6 (2·6–4·8)	3·6 (2·5–5·0)	-0·3 (-1·1–0·4)	0·1 (-0·9–1·1)	0·0 (-1·6–1·5)
Vitamin A deficiency	1·7 (0·0–3·8)	2·3 (0·0–4·7)	1·5 (0·0–3·1)	0·9 (0·0–1·9)	-2·0 (-4·1–0·2)	-4·4 (-7·1–0·0)	-4·4 (-8·6–0·0)
Zinc deficiency	2·4 (0·0–9·3)	1·3 (0·0–5·7)	1·2 (0·0–5·1)	1·2 (0·0–5·0)	-2·2 (-47·7–0·0)	-0·5 (-2·3–0·5)	0·2 (-4·7–2·8)
Tobacco	42·4 (40·6–44·1)	39·2 (37·6–40·5)	36·3 (35·0–37·6)	31·6 (29·6–33·4)	-1·0 (-1·2–-0·7)	-1·0 (-1·3–-0·7)	-1·3 (-1·8–-0·7)
Smoking	36·5 (34·0–39·0)	33·1 (31·1–34·8)	30·1 (28·6–31·6)	26·2 (24·1–28·6)	-1·1 (-1·4–-0·8)	-1·1 (-1·5–-0·7)	-1·2 (-2·0–-0·5)
Chewing tobacco	3·8 (2·9–4·6)	3·9 (3·2–4·9)	4·0 (3·1–5·1)	3·9 (2·8–5·1)	0·1 (-1·1–1·2)	-0·1 (-1·6–1·4)	-0·3 (-2·6–1·8)
Second-hand smoke	39·6 (36·9–41·3)	38·1 (35·3–39·8)	36·3 (33·8–38·0)	31·4 (28·6–33·6)	-0·7 (-1·0–-0·5)	-0·9 (-1·3–-0·6)	-1·3 (-2·0–-0·7)
High alcohol use	21·4 (15·2–29·7)	20·1 (13·4–29·2)	21·2 (14·2–31·1)	21·7 (14·5–31·6)	0·0 (-1·1–1·1)	0·4 (-1·1–1·8)	0·2 (-1·8–2·2)
Drug use	0·9 (0·5–1·5)	1·2 (0·9–1·4)	2·1 (1·7–2·5)	5·0 (3·4–6·4)	5·6 (2·9–7·8)	7·0 (4·5–8·5)	7·9 (6·0–9·4)
Dietary risks	40·3 (31·0–51·6)	43·9 (34·1–56·3)	47·3 (37·0–59·9)	46·9 (37·4–59·9)	0·5 (0·2–0·8)	0·3 (0·0–0·6)	-0·1 (-0·5–0·4)
Diet low in fruits	40·5 (35·1–44·1)	39·2 (34·2–42·1)	41·2 (36·5–44·5)	42·3 (36·4–47·0)	0·1 (-0·2–0·5)	0·4 (-0·1–0·8)	0·2 (-0·5–0·9)
Diet low in vegetables	35·5 (22·0–43·4)	38·1 (23·0–46·1)	46·2 (25·5–55·0)	44·7 (25·9–53·9)	0·7 (0·4–1·1)	0·8 (0·3–1·3)	-0·3 (-1·0–0·4)
Diet low in legumes	40·6 (0·0–50·9)	40·6 (0·0–50·8)	44·7 (0·0–55·5)	45·3 (0·0–57·4)	0·4 (0·0–0·7)	0·5 (0·0–1·0)	0·1 (-0·7–0·9)
Diet low in whole grains	46·2 (38·0–52·7)	50·1 (41·3–56·9)	51·9 (42·4–59·6)	51·8 (42·5–60·0)	0·4 (0·1–0·7)	0·2 (-0·2–0·5)	0·0 (-0·6–0·5)
Diet low in nuts and seeds	16·9 (13·0–21·5)	10·4 (7·0–14·0)	7·6 (4·7–10·8)	7·9 (5·0–11·6)	-2·4 (-4·0–-1·0)	-1·3 (-3·6–0·9)	0·4 (-3·0–3·8)
Diet low in milk	40·8 (36·5–45·5)	37·6 (33·4–42·1)	35·7 (31·5–40·6)	35·4 (30·6–40·2)	-0·5 (-0·9–-0·1)	-0·3 (-0·9–0·2)	-0·1 (-1·0–0·6)
Diet high in red meat	53·8 (0·0–73·2)	54·6 (0·0–74·4)	54·4 (0·0–73·7)	53·8 (0·0–73·6)	0·0 (-0·5–0·4)	-0·1 (-0·7–0·5)	-0·1 (-0·9–0·9)
Diet high in processed meat	50·5 (40·5–56·1)	62·9 (50·0–69·8)	69·4 (56·0–78·2)	66·5 (54·9–75·5)	0·9 (0·5–1·3)	0·3 (-0·2–0·8)	-0·4 (-1·1–0·4)
Diet high in sugar-sweetened beverages	32·0 (23·9–40·7)	47·2 (37·4–57·2)	53·0 (41·8–63·7)	51·6 (40·9–62·7)	1·5 (0·6–2·4)	0·4 (-0·7–1·5)	-0·2 (-1·7–1·3)
Diet low in fibre	45·1 (24·4–53·1)	34·7 (18·8–42·4)	30·8 (16·0–37·7)	27·1 (15·8–34·1)	-1·6 (-2·5–-0·9)	-1·2 (-2·3–-0·1)	-1·2 (-2·7–0·4)
Diet low in calcium	6·8 (5·6–8·4)	5·6 (4·5–6·9)	5·9 (4·7–7·3)	5·8 (4·7–7·1)	-0·5 (-0·8–-0·3)	0·2 (-0·1–0·5)	-0·2 (-0·6–0·3)
Diet low in seafood omega-3 fatty acids	43·2 (32·5–53·9)	49·1 (38·9–60·3)	51·8 (41·2–63·6)	47·0 (37·2–58·3)	0·3 (-0·1–0·7)	-0·2 (-0·7–0·3)	-0·9 (-1·5–-0·3)
Diet low in omega-6 polyunsaturated fatty acids	42·5 (26·8–53·5)	36·7 (24·1–46·7)	27·4 (18·7–34·6)	25·4 (17·4–33·1)	-1·7 (-2·4–-0·9)	-1·8 (-2·6–-0·9)	-0·7 (-1·7–0·3)
Diet high in trans fatty acids	71·4 (62·8–78·6)	71·7 (64·1–79·2)	63·4 (54·9–71·2)	0·0 (0·0–0·0)	-65·8 (-66·1–-65·3)	-97·1 (-97·6–-96·6)	-184·2 (-185·3–-182·9)
Diet high in sodium	23·8 (0·6–70·2)	28·8 (1·7–76·6)	32·3 (3·1–77·7)	32·6 (3·0–79·5)	1·0 (0·2–6·1)	0·6 (-0·1–4·1)	0·1 (-2·5–1·8)
Intimate partner violence	31·5 (18·8–40·4)	31·5 (19·9–39·8)	28·6 (19·6–35·3)	26·7 (16·3–35·7)	-0·5 (-1·8–0·6)	-0·8 (-2·5–0·7)	-0·6 (-2·9–1·2)
Childhood sexual abuse and bullying	12·0 (7·8–18·5)	13·1 (8·6–20·0)	14·7 (9·7–22·5)	11·6 (8·2–17·3)	-0·1 (-0·7–0·5)	-0·6 (-1·4–0·3)	-2·1 (-3·6–-0·6)
Childhood sexual abuse	10·0 (8·4–11·8)	11·3 (9·7–13·1)	13·1 (11·3–15·2)	12·0 (10·0–14·3)	0·6 (-0·2–1·3)	0·3 (-0·6–1·2)	-0·8 (-2·2–0·5)
Bullying victimization	11·4 (5·4–21·0)	12·2 (5·8–22·2)	13·3 (6·3–24·3)	9·5 (4·5–17·5)	-0·6 (-1·3–0·1)	-1·2 (-2·2–-0·2)	-3·1 (-5·0–-1·1)
Unsafe sex	--	--	--	--	--	--	--
Low physical activity	22·3 (17·1–27·9)	22·5 (17·7–28·1)	23·3 (19·0–28·3)	25·1 (19·5–31·6)	0·4 (-0·5–1·3)	0·5 (-0·7–1·6)	0·7 (-1·0–2·2)
**Metabolic risks**	**28·3 (24·6–31·8)**	**34·9 (30·8–38·0)**	**39·1 (34·7–42·1)**	**42·7 (37·3–45·8)**	**1·3 (1·0–1·6)**	**1·0 (0·6–1·3)**	**0·8 (0·3–1·3)**
High fasting plasma glucose	13·8 (10·5–16·5)	18·8 (14·6–22·3)	24·2 (18·8–29·1)	27·0 (20·3–32·6)	2·2 (1·5–2·9)	1·7 (0·8–2·7)	1·0 (-0·1–2·2)
High LDL cholesterol	63·2 (44·3–86·2)	53·8 (36·8–74·3)	48·4 (32·7–67·2)	47·0 (31·5–65·7)	-1·0 (-1·2–-0·8)	-0·6 (-0·8–-0·5)	-0·3 (-0·5–0·0)
High systolic blood pressure	32·4 (22·7–43·4)	28·6 (20·3–38·3)	22·0 (14·9–30·8)	30·2 (19·5–42·9)	-0·2 (-1·1–0·6)	0·2 (-0·9–1·4)	2·9 (0·6–5·4)
High body-mass index	32·3 (27·5–37·0)	40·2 (34·7–44·1)	45·1 (38·6–48·5)	48·8 (41·3–52·9)	1·3 (1·0–1·6)	0·9 (0·6–1·2)	0·7 (0·3–1·2)
Low bone mineral density	21·5 (15·7–28·4)	19·4 (13·8–26·3)	20·4 (14·9–27·3)	22·5 (16·5–29·9)	0·1 (-0·3–0·6)	0·7 (0·0–1·5)	0·9 (-0·3–2·2)
Kidney dysfunction	3·1 (2·5–4·0)	3·2 (2·6–4·2)	3·2 (2·6–4·2)	3·4 (2·8–4·4)	0·3 (0·2–0·4)	0·2 (0·1–0·4)	0·4 (0·2–0·7)

Missouri

Risk Names	SEV 1990	SEV 2000	SEV 2010	SEV 2021	Annualised rate of change 1990 to 2021	Annualised rate of change 2000 to 2021	Annualised rate of change 2010 to 2021
**All risk factors**	**27·3 (24·3–30·5)**	**27·4 (24·4–30·5)**	**27·9 (24·7–31·2)**	**27·5 (24·4–30·9)**	**0·0 (-0·3–0·3)**	**0·0 (-0·4–0·4)**	**-0·1 (-0·7–0·4)**
**Environmental/occupational risks**	**19·3 (12·8–29·9)**	**18·5 (13·1–29·2)**	**16·6 (11·2–26·8)**	**14·4 (9·3–23·5)**	**-1·0 (-1·7–-0·3)**	**-1·2 (-1·6–-0·7)**	**-1·3 (-1·9–-0·8)**
Unsafe water, sanitation, and handwashing	2·1 (1·1–3·4)	1·9 (0·9–3·1)	1·5 (0·5–2·5)	1·2 (0·4–2·1)	-1·7 (-4·1–0·3)	-2·1 (-5·1–0·7)	-1·8 (-5·9–2·3)
Unsafe water source	2·9 (1·1–6·0)	2·7 (0·9–5·4)	1·1 (0·4–2·3)	0·9 (0·4–1·8)	-3·9 (-6·7–-1·2)	-5·4 (-9·0–-1·3)	-2·2 (-7·5–3·7)
Unsafe sanitation	6·8 (3·8–10·9)	5·3 (2·9–9·1)	4·1 (2·2–7·4)	3·1 (1·6–5·4)	-2·5 (-4·8–-0·5)	-2·6 (-5·4–0·0)	-2·6 (-5·9–0·6)
No access to handwashing facility	1·7 (0·5–3·4)	1·8 (0·5–3·5)	1·8 (0·6–3·3)	1·5 (0·5–3·0)	-0·4 (-3·4–2·8)	-0·7 (-4·5–3·2)	-1·4 (-7·3–3·8)
Air pollution	22·0 (7·8–34·2)	19·3 (9·0–29·8)	12·6 (5·9–22·0)	7·3 (2·8–15·4)	-3·5 (-5·9–-1·5)	-4·6 (-7·3–-2·9)	-4·9 (-8·7–-2·9)
Particulate matter pollution	13·6 (5·3–23·6)	11·9 (8·4–15·8)	8·6 (5·3–12·1)	5·2 (2·5–8·6)	-3·1 (-5·8–-0·5)	-3·9 (-6·3–-2·8)	-4·5 (-8·2–-2·6)
Ambient particulate matter pollution	21·2 (8·3–37·0)	18·4 (13·3–23·9)	13·1 (8·4–18·2)	8·0 (3·8–12·7)	-3·1 (-5·9–-0·5)	-4·0 (-6·4–-2·8)	-4·5 (-8·3–-2·7)
Household air pollution from solid fuels	0·0 (0·0–0·1)	0·0 (0·0–0·0)	0·0 (0·0–0·0)	0·0 (0·0–0·0)	-3·4 (-25·8–-5·3)	-3·4 (-21·7–-4·9)	-2·5 (-15·6–-2·6)
Ambient ozone pollution	17·5 (12·1–23·9)	25·2 (19·2–33·3)	16·4 (11·0–23·4)	14·5 (9·3–20·9)	-0·6 (-1·0–-0·2)	-2·6 (-3·6–-1·8)	-1·1 (-2·3–0·0)
Ambient nitrogen dioxide pollution	56·6 (0·0–100·0)	48·8 (0·0–100·0)	27·8 (0·0–76·5)	14·6 (0·0–54·9)	-4·4 (-14·5–0·0)	-5·7 (-19·8–0·0)	-5·9 (-26·8–0·0)
Non-optimal temperature	31·0 (24·0–41·6)	31·6 (25·1–42·4)	34·2 (26·8–45·9)	30·7 (24·2–41·8)	0·0 (-0·3–0·2)	-0·1 (-0·4–0·1)	-1·0 (-1·6–-0·5)
High temperature	32·6 (22·3–43·8)	32·3 (22·0–43·5)	37·0 (25·4–48·9)	28·9 (18·4–40·4)	-0·4 (-0·8–0·0)	-0·5 (-1·1–0·0)	-2·2 (-3·3–-1·4)
Low temperature	27·0 (23·3–31·2)	27·9 (24·8–31·4)	29·3 (26·7–32·9)	27·8 (24·7–31·5)	0·1 (-0·1–0·3)	0·0 (-0·2–0·2)	-0·5 (-0·8–-0·2)
Other environmental risks	27·5 (7·8–46·5)	25·9 (7·9–44·1)	23·4 (7·8–41·5)	20·4 (7·8–38·7)	-1·0 (-1·6–0·0)	-1·1 (-1·9–0·0)	-1·2 (-2·2–0·0)
Residential radon	26·2 (0·0–83·6)	26·2 (0·0–83·6)	26·2 (0·0–83·6)	26·2 (0·0–83·6)	0·0 (0·0–0·0)	0·0 (0·0–0·0)	0·0 (0·0–0·0)
Lead exposure	28·0 (0·0–34·8)	25·7 (0·0–31·8)	22·0 (0·0–27·5)	17·8 (0·0–22·3)	-1·5 (-1·9–0·0)	-1·7 (-2·2–0·0)	-1·9 (-2·8–0·0)
Occupational risks	3·0 (2·5–3·8)	3·0 (2·5–3·9)	2·8 (2·3–3·7)	2·8 (2·3–3·7)	-0·1 (-0·4–0·1)	-0·3 (-0·6–0·0)	0·0 (-0·4–0·5)
Occupational carcinogens	1·0 (0·4–1·8)	1·0 (0·5–2·0)	1·0 (0·4–1·9)	1·0 (0·4–1·9)	0·2 (-0·1–0·4)	-0·1 (-0·5–0·3)	0·1 (-0·5–0·6)
Occupational exposure to asbestos	3·6 (3·2–4·0)	3·9 (3·5–4·3)	3·6 (3·2–3·9)	3·3 (2·6–4·1)	-0·3 (-1·1–0·5)	-0·8 (-1·9–0·3)	-0·8 (-2·9–1·3)
Occupational exposure to arsenic	0·5 (0·0–1·2)	0·5 (0·0–1·3)	0·5 (0·0–1·2)	0·5 (0·0–1·2)	-0·2 (-0·7–0·2)	-0·4 (-1·0–0·3)	0·1 (-0·7–0·8)
Occupational exposure to benzene	1·1 (0·1–3·1)	1·2 (0·1–3·4)	1·2 (0·1–3·4)	1·2 (0·1–3·6)	0·4 (0·0–0·7)	0·1 (-0·3–0·5)	0·3 (-0·3–0·9)
Occupational exposure to beryllium	0·0 (0·0–0·0)	0·0 (0·0–0·0)	0·0 (0·0–0·0)	0·0 (0·0–0·0)	0·2 (0·0–0·4)	0·1 (-0·2–0·4)	0·4 (0·0–0·9)
Occupational exposure to cadmium	0·1 (0·1–0·1)	0·1 (0·1–0·1)	0·1 (0·1–0·1)	0·1 (0·1–0·1)	-0·2 (-0·6–0·3)	-0·3 (-0·9–0·2)	0·1 (-0·6–0·8)
Occupational exposure to chromium	0·2 (0·1–0·2)	0·2 (0·1–0·2)	0·1 (0·1–0·2)	0·1 (0·1–0·2)	-0·1 (-0·4–0·3)	-0·2 (-0·7–0·2)	0·2 (-0·5–0·8)
Occupational exposure to diesel engine exhaust	0·7 (0·6–0·7)	0·7 (0·7–0·8)	0·7 (0·7–0·8)	0·8 (0·7–0·8)	0·3 (0·0–0·7)	0·2 (-0·2–0·7)	0·7 (0·1–1·4)
Occupational exposure to formaldehyde	0·3 (0·3–0·3)	0·3 (0·3–0·3)	0·3 (0·3–0·3)	0·3 (0·3–0·3)	-0·2 (-0·6–0·2)	-0·4 (-1·0–0·2)	0·0 (-0·7–0·7)
Occupational exposure to nickel	0·4 (0·0–1·7)	0·4 (0·0–1·6)	0·4 (0·0–1·5)	0·4 (0·0–1·5)	-0·3 (-0·8–0·2)	-0·4 (-1·1–0·3)	0·0 (-0·9–1·1)
Occupational exposure to polycyclic aromatic hydrocarbons	0·3 (0·3–0·3)	0·3 (0·3–0·3)	0·3 (0·3–0·3)	0·3 (0·3–0·3)	0·0 (-0·3–0·3)	-0·2 (-0·6–0·3)	0·2 (-0·4–0·7)
Occupational exposure to silica	3·7 (0·6–11·7)	3·6 (0·5–11·7)	3·3 (0·5–11·1)	3·3 (0·5–10·9)	-0·3 (-0·8–0·1)	-0·3 (-1·0–0·3)	0·1 (-0·7–1·1)
Occupational exposure to sulphuric acid	0·8 (0·2–2·6)	0·8 (0·2–2·6)	0·7 (0·2–2·3)	0·7 (0·2–2·4)	-0·2 (-0·6–0·3)	-0·3 (-0·8–0·3)	0·1 (-0·6–0·9)
Occupational exposure to trichloroethylene	0·1 (0·1–0·1)	0·1 (0·1–0·1)	0·1 (0·1–0·1)	0·1 (0·1–0·1)	0·1 (-0·2–0·4)	-0·1 (-0·4–0·3)	0·4 (-0·2–0·9)
Occupational asthmagens	17·5 (15·3–19·9)	18·0 (15·9–20·4)	16·5 (14·4–18·8)	16·8 (14·7–19·2)	-0·1 (-0·5–0·2)	-0·3 (-0·8–0·1)	0·2 (-0·5–0·9)
Occupational particulate matter, gases, and fumes	6·9 (5·3–9·0)	6·7 (5·3–8·7)	6·4 (4·9–8·2)	5·9 (4·5–7·6)	-0·5 (-0·8–-0·2)	-0·6 (-0·9–-0·3)	-0·7 (-1·1–-0·3)
Occupational noise	6·9 (6·3–7·6)	7·0 (6·4–7·6)	6·9 (6·4–7·4)	6·7 (6·3–7·2)	-0·1 (-0·3–0·1)	-0·2 (-0·4–0·0)	-0·3 (-0·5–-0·1)
Occupational injuries	--	--	--	--	--	--	--
Occupational ergonomic factors	8·4 (7·0–10·2)	8·4 (7·0–10·1)	7·6 (6·3–9·2)	7·7 (6·4–9·4)	-0·2 (-0·7–0·2)	-0·4 (-0·9–0·1)	0·2 (-0·5–0·9)
**Behavioural risks**	**31·1 (28·0–34·5)**	**30·1 (26·7–33·8)**	**28·7 (25·1–32·6)**	**26·5 (23·3–30·7)**	**-0·5 (-0·9–-0·2)**	**-0·6 (-1·1–-0·2)**	**-0·7 (-1·4–-0·1)**
Child and maternal malnutrition	10·1 (6·9–14·1)	8·8 (5·9–12·2)	8·9 (6·2–12·2)	9·2 (6·2–13·0)	-0·3 (-1·2–0·6)	0·2 (-1·0–1·2)	0·2 (-1·6–1·8)
Suboptimal breastfeeding	68·9 (66·4–71·7)	66·7 (64·2–69·5)	62·9 (60·0–65·7)	62·2 (59·4–65·1)	-0·3 (-0·5–-0·2)	-0·3 (-0·5–-0·1)	-0·1 (-0·4–0·2)
Non-exclusive breastfeeding	64·6 (56·6–71·6)	59·8 (52·8–66·4)	48·9 (42·2–56·2)	46·5 (39·7–54·1)	-1·1 (-1·5–-0·6)	-1·2 (-1·8–-0·5)	-0·5 (-1·6–0·5)
Discontinued breastfeeding	88·7 (86·4–91·3)	86·3 (83·6–88·9)	84·3 (81·1–87·6)	84·6 (81·1–87·9)	-0·2 (-0·3–0·0)	-0·1 (-0·3–0·1)	0·0 (-0·3–0·3)
Child growth failure	1·3 (0·4–3·0)	1·2 (0·4–2·9)	1·1 (0·3–2·4)	1·0 (0·3–2·3)	-0·8 (-1·2–-0·5)	-0·8 (-1·4–-0·3)	-0·4 (-1·2–0·3)
Child underweight	2·8 (1·8–3·8)	2·6 (1·6–3·6)	2·2 (1·4–3·1)	2·2 (1·3–2·9)	-0·8 (-1·3–-0·4)	-0·9 (-1·5–-0·2)	-0·4 (-1·5–0·6)
Child wasting	0·9 (0·6–1·4)	0·8 (0·5–1·3)	0·8 (0·5–1·3)	0·8 (0·5–1·3)	-0·2 (-0·5–0·1)	0·0 (-0·4–0·5)	-0·1 (-0·8–0·5)
Child stunting	3·8 (2·9–4·8)	3·6 (2·8–4·4)	3·1 (2·4–4·0)	2·9 (2·2–3·7)	-0·9 (-1·5–-0·3)	-1·0 (-1·8–-0·1)	-0·7 (-1·8–0·6)
Low birth weight and short gestation	22·0 (18·6–25·1)	23·3 (19·9–26·4)	24·7 (21·4–27·7)	25·7 (22·6–29·0)	0·5 (0·3–0·8)	0·5 (0·1–0·8)	0·4 (-0·4–1·2)
Short gestation	40·4 (35·3–45·7)	43·6 (38·0–49·3)	44·9 (39·3–51·1)	45·5 (39·8–51·5)	0·4 (0·1–0·7)	0·2 (-0·2–0·6)	0·1 (-0·8–0·9)
Low birth weight	17·2 (15·4–19·0)	17·7 (16·0–19·4)	18·9 (17·0–20·8)	19·9 (18·0–22·0)	0·5 (0·2–0·7)	0·6 (0·2–0·9)	0·5 (-0·2–1·2)

Iron deficiency	3·6 (2·5–4·9)	3·2 (2·2–4·3)	3·2 (2·3–4·3)	3·3 (2·3–4·5)	-0·3 (-1·1–0·5)	0·2 (-0·9–1·2)	0·1 (-1·7–1·6)
Vitamin A deficiency	1·1 (0·0–2·4)	1·5 (0·0–3·1)	1·0 (0·0–2·1)	0·6 (0·0–1·3)	-2·0 (-4·2–0·2)	-4·4 (-7·2–0·0)	-4·3 (-8·1–0·0)
Zinc deficiency	1·9 (0·0–8·2)	1·1 (0·0–4·8)	1·0 (0·0–4·4)	1·0 (0·0–4·5)	-1·9 (-46·1–0·7)	-0·3 (-1·6–2·5)	0·1 (-4·3–5·9)
Tobacco	42·4 (41·0–43·9)	40·4 (38·9–41·7)	35·5 (34·3–36·7)	30·7 (28·8–32·7)	-1·0 (-1·2–-0·8)	-1·3 (-1·6–-1·0)	-1·3 (-1·9–-0·8)
Smoking	40·0 (37·8–42·0)	37·2 (35·2–39·1)	32·5 (31·0–34·4)	28·3 (26·1–30·8)	-1·1 (-1·4–-0·8)	-1·3 (-1·7–-0·9)	-1·3 (-2·0–-0·5)
Chewing tobacco	2·3 (1·9–2·9)	2·6 (2·0–3·3)	2·9 (2·1–3·8)	2·8 (2·0–3·8)	0·7 (-0·6–1·8)	0·3 (-1·3–2·1)	0·0 (-2·6–2·9)
Second-hand smoke	33·9 (32·0–35·2)	32·5 (30·8–33·9)	28·6 (26·8–29·7)	24·8 (23·0–26·7)	-1·0 (-1·2–-0·8)	-1·3 (-1·6–-1·0)	-1·3 (-1·9–-0·7)
High alcohol use	26·8 (18·8–36·7)	25·4 (17·9–35·4)	26·0 (18·0–36·0)	26·0 (18·2–36·9)	-0·1 (-1·2–1·0)	0·1 (-1·2–1·5)	0·0 (-2·2–1·8)
Drug use	0·8 (0·6–1·1)	1·1 (0·9–1·3)	2·2 (1·5–2·8)	5·4 (3·5–7·0)	6·3 (4·1–7·7)	7·6 (5·9–8·4)	8·3 (7·0–9·3)
Dietary risks	39·3 (29·7–50·9)	42·6 (33·5–54·9)	44·6 (35·2–57·0)	44·4 (35·2–56·9)	0·4 (0·1–0·7)	0·2 (-0·1–0·5)	0·0 (-0·5–0·4)
Diet low in fruits	38·8 (33·5–42·3)	36·4 (32·0–39·6)	35·9 (30·9–38·8)	37·4 (32·4–42·1)	-0·1 (-0·5–0·3)	0·1 (-0·4–0·7)	0·4 (-0·5–1·2)
Diet low in vegetables	28·8 (17·1–36·4)	30·8 (17·9–38·5)	34·8 (19·7–42·7)	34·3 (20·1–42·7)	0·6 (0·2–1·0)	0·5 (0·0–1·0)	-0·1 (-1·0–0·7)
Diet low in legumes	38·3 (0·0–48·1)	38·4 (0·0–48·0)	42·8 (0·0–53·2)	43·7 (0·0–54·7)	0·4 (0·0–0·7)	0·6 (0·0–1·1)	0·2 (-0·5–0·9)
Diet low in whole grains	45·4 (37·7–51·8)	49·0 (41·0–55·5)	50·7 (42·1–57·9)	51·0 (42·3–58·4)	0·4 (0·1–0·6)	0·2 (-0·2–0·5)	0·1 (-0·5–0·6)
Diet low in nuts and seeds	13·0 (9·1–17·1)	7·6 (4·7–10·7)	5·7 (3·4–8·4)	6·3 (3·5–9·5)	-2·3 (-4·2–-0·8)	-0·9 (-3·5–1·3)	0·9 (-3·0–4·5)
Diet low in milk	36·9 (32·7–41·2)	34·1 (29·7–39·7)	32·8 (29·1–37·7)	32·8 (28·5–37·8)	-0·4 (-0·8–0·1)	-0·2 (-0·8–0·4)	0·0 (-0·8–0·8)
Diet high in red meat	56·3 (0·0–75·0)	56·7 (0·0–76·3)	56·1 (0·0–76·4)	55·5 (0·0–76·4)	0·0 (-0·5–0·3)	-0·1 (-0·7–0·4)	-0·1 (-1·0–0·6)
Diet high in processed meat	51·7 (41·5–57·3)	63·2 (51·0–70·6)	69·3 (56·1–76·7)	66·6 (55·2–74·7)	0·8 (0·4–1·2)	0·3 (-0·3–0·7)	-0·3 (-1·1–0·4)
Diet high in sugar-sweetened beverages	36·1 (27·9–43·6)	50·9 (39·6–58·7)	55·8 (42·9–64·4)	54·1 (41·8–65·0)	1·3 (0·5–2·2)	0·3 (-0·7–1·3)	-0·3 (-1·6–1·2)
Diet low in fibre	39·7 (21·9–46·9)	30·1 (17·1–36·4)	27·0 (15·6–33·1)	24·1 (13·7–30·8)	-1·6 (-2·5–-0·8)	-1·1 (-2·3–0·2)	-1·0 (-2·8–0·7)
Diet low in calcium	5·8 (4·6–7·1)	4·9 (3·9–5·9)	5·2 (4·1–6·4)	5·2 (4·1–6·4)	-0·3 (-0·6–-0·1)	0·3 (0·0–0·6)	0·0 (-0·4–0·3)
Diet low in seafood omega-3 fatty acids	49·7 (39·6–59·9)	56·6 (45·7–67·8)	59·9 (48·7–71·0)	55·7 (45·7–66·7)	0·4 (0·0–0·7)	-0·1 (-0·5–0·3)	-0·7 (-1·2–-0·2)
Diet low in omega-6 polyunsaturated fatty acids	36·3 (23·4–45·5)	31·9 (21·1–40·5)	24·1 (16·5–30·8)	22·8 (15·8–29·5)	-1·5 (-2·3–-0·8)	-1·6 (-2·4–-0·8)	-0·5 (-1·5–0·6)
Diet high in trans fatty acids	71·4 (64·3–78·5)	71·9 (63·7–79·9)	63·3 (55·5–70·6)	0·0 (0·0–0·0)	-65·8 (-66·1–-65·4)	-97·1 (-97·6–-96·5)	-184·2 (-185·2–-183·0)
Diet high in sodium	23·4 (0·7–69·5)	28·4 (1·8–76·1)	31·5 (3·4–78·5)	31·8 (3·2–78·8)	1·0 (0·3–6·3)	0·5 (-0·1–3·7)	0·1 (-2·4–2·0)
Intimate partner violence	24·0 (14·9–30·1)	23·7 (15·8–28·4)	21·9 (15·6–27·0)	22·4 (13·0–30·5)	-0·2 (-1·7–1·2)	-0·3 (-2·3–1·5)	0·2 (-2·1–2·2)
Childhood sexual abuse and bullying	9·2 (6·0–14·7)	11·1 (7·4–17·5)	12·8 (8·6–19·9)	10·2 (7·0–15·6)	0·3 (-0·3–0·9)	-0·4 (-1·3–0·3)	-2·1 (-3·5–-0·7)
Childhood sexual abuse	9·2 (7·8–10·8)	10·3 (9·0–11·9)	12·0 (10·7–13·7)	11·1 (9·1–13·5)	0·6 (-0·2–1·5)	0·4 (-0·7–1·4)	-0·7 (-2·2–0·7)
Bullying victimization	7·9 (3·5–16·0)	9·9 (4·6–19·3)	11·3 (5·1–21·9)	7·9 (3·5–15·9)	0·0 (-0·7–0·6)	-1·1 (-2·0–-0·1)	-3·2 (-5·0–-1·5)
Unsafe sex	--	--	--	--	--	--	--
Low physical activity	20·7 (15·7–26·2)	20·1 (15·4–25·0)	20·1 (15·8–25·1)	22·5 (16·7–28·7)	0·3 (-0·6–1·2)	0·5 (-0·7–1·7)	1·0 (-0·9–2·9)
**Metabolic risks**	**26·0 (22·7–29·2)**	**32·0 (28·2–35·0)**	**35·5 (31·4–38·5)**	**39·2 (34·5–42·5)**	**1·3 (1·0–1·6)**	**1·0 (0·6–1·3)**	**0·9 (0·5–1·4)**
High fasting plasma glucose	12·8 (10·0–15·4)	17·1 (13·1–20·5)	22·2 (16·2–26·5)	25·3 (18·4–30·2)	2·2 (1·5–2·9)	1·9 (0·9–2·8)	1·2 (0·0–2·4)
High LDL cholesterol	63·4 (44·6–86·5)	53·8 (37·0–74·3)	48·4 (32·8–67·4)	46·8 (31·7–65·0)	-1·0 (-1·2–-0·8)	-0·7 (-0·9–-0·5)	-0·3 (-0·6–0·0)
High systolic blood pressure	30·0 (19·9–41·7)	25·9 (18·2–35·4)	19·3 (12·7–27·9)	26·7 (17·1–37·5)	-0·4 (-1·4–0·5)	0·1 (-1·2–1·2)	2·9 (0·6–5·1)
High body-mass index	29·7 (25·0–34·1)	36·9 (31·4–40·9)	40·8 (35·0–44·6)	44·6 (38·1–48·5)	1·3 (1·0–1·6)	0·9 (0·6–1·2)	0·8 (0·4–1·3)
Low bone mineral density	21·8 (15·5–28·9)	19·8 (14·0–26·5)	20·6 (14·6–27·2)	22·7 (16·4–29·9)	0·1 (-0·3–0·6)	0·7 (-0·1–1·4)	0·9 (-0·5–2·4)
Kidney dysfunction	2·9 (2·4–3·8)	3·0 (2·5–4·0)	3·0 (2·4–3·9)	3·1 (2·6–4·1)	0·2 (0·1–0·3)	0·1 (0·0–0·3)	0·4 (0·1–0·7)

Montana

Risk Names	SEV 1990	SEV 2000	SEV 2010	SEV 2021	Annualised rate of change 1990 to 2021	Annualised rate of change 2000 to 2021	Annualised rate of change 2010 to 2021
**All risk factors**	**24·5 (21·6–27·6)**	**24·1 (21·4–27·1)**	**24·3 (21·6–27·2)**	**24·5 (21·5–27·7)**	**0·0 (-0·3–0·3)**	**0·1 (-0·3–0·4)**	**0·1 (-0·4–0·6)**
**Environmental/occupational risks**	**14·9 (9·8–24·6)**	**15·2 (9·9–25·1)**	**13·3 (8·6–22·3)**	**12·1 (7·7–20·3)**	**-0·7 (-1·3–-0·2)**	**-1·1 (-1·5–-0·7)**	**-0·9 (-1·3–-0·5)**
Unsafe water, sanitation, and handwashing	2·0 (1·1–3·3)	1·8 (0·8–3·1)	1·4 (0·5–2·4)	1·2 (0·4–2·1)	-1·8 (-4·0–0·3)	-2·2 (-4·9–0·6)	-2·0 (-5·9–1·7)
Unsafe water source	2·8 (1·1–5·8)	2·5 (1·0–5·2)	1·1 (0·4–2·0)	0·8 (0·4–1·6)	-4·0 (-6·7–-1·0)	-5·4 (-8·9–-1·6)	-2·3 (-8·1–3·1)
Unsafe sanitation	6·5 (3·5–10·4)	5·0 (2·5–8·1)	4·0 (2·1–6·7)	2·8 (1·4–4·9)	-2·7 (-5·0–-0·4)	-2·7 (-5·5–-0·1)	-3·1 (-6·7–0·3)
No access to handwashing facility	1·7 (0·6–3·5)	1·8 (0·6–3·5)	1·8 (0·5–3·3)	1·5 (0·5–3·0)	-0·5 (-3·1–2·7)	-0·7 (-4·3–3·4)	-1·5 (-6·8–3·5)
Air pollution	11·6 (2·3–22·1)	11·7 (3·5–21·8)	5·9 (1·0–14·1)	5·4 (0·5–13·7)	-2·5 (-6·8–-1·0)	-3·7 (-9·0–-2·2)	-0·9 (-6·6–0·1)
Particulate matter pollution	5·7 (1·1–13·0)	5·4 (2·9–8·5)	2·3 (0·7–4·7)	1·4 (0·2–3·4)	-4·5 (-10·2–-0·5)	-6·4 (-12·8–-4·1)	-4·5 (-11·8–-2·5)
Ambient particulate matter pollution	8·7 (1·7–19·7)	8·2 (4·5–12·6)	3·5 (1·0–7·0)	2·1 (0·3–5·1)	-4·6 (-10·2–-0·5)	-6·4 (-12·8–-4·2)	-4·5 (-11·6–-2·5)
Household air pollution from solid fuels	0·0 (0·0–0·1)	0·0 (0·0–0·1)	0·0 (0·0–0·0)	0·0 (0·0–0·0)	-4·9 (-35·1–-7·6)	-6·3 (-39·6–-9·9)	-3·8 (-33·4–-8·4)
Ambient ozone pollution	12·4 (7·2–17·9)	17·4 (11·6–24·4)	14·9 (9·1–21·9)	12·0 (6·8–18·2)	-0·1 (-0·9–0·8)	-1·8 (-3·2–-0·5)	-1·9 (-4·5–0·4)
Ambient nitrogen dioxide pollution	36·9 (0·0–90·2)	38·9 (0·0–92·8)	20·9 (0·0–64·5)	22·6 (0·0–67·7)	-1·6 (-4·8–0·0)	-2·6 (-7·9–0·0)	0·7 (0·0–1·5)
Non-optimal temperature	31·4 (25·1–40·7)	32·3 (26·0–41·1)	29·7 (24·2–37·6)	32·8 (25·6–42·2)	0·1 (0·0–0·3)	0·1 (-0·2–0·4)	0·9 (0·0–2·0)
High temperature	27·4 (16·0–40·4)	31·7 (19·2–46·0)	24·1 (13·4–37·2)	35·3 (22·1–49·1)	0·8 (0·4–1·4)	0·5 (0·1–1·0)	3·5 (1·9–5·7)
Low temperature	29·1 (25·4–34·1)	30·0 (25·7–35·3)	28·4 (24·0–34·1)	29·5 (25·4–34·8)	0·1 (-0·1–0·1)	-0·1 (-0·2–0·0)	0·3 (0·0–0·6)
Other environmental risks	26·4 (10·4–47·2)	25·2 (10·9–45·9)	23·2 (10·4–44·0)	20·9 (9·2–41·5)	-0·8 (-1·5–0·0)	-0·9 (-1·8–0·0)	-0·9 (-2·2–0·0)
Residential radon	33·7 (0·0–99·3)	33·7 (0·0–99·3)	33·7 (0·0–99·3)	33·7 (0·0–99·3)	0·0 (0·0–0·0)	0·0 (0·0–0·0)	0·0 (0·0–0·0)
Lead exposure	23·1 (0·0–29·0)	21·4 (0·0–26·9)	18·4 (0·0–23·2)	15·2 (0·0–19·1)	-1·4 (-1·8–0·0)	-1·6 (-2·2–0·0)	-1·8 (-2·6–0·0)
Occupational risks	2·9 (2·4–3·7)	3·0 (2·4–3·9)	2·8 (2·3–3·8)	2·8 (2·3–3·8)	0·0 (-0·3–0·3)	-0·2 (-0·5–0·2)	0·1 (-0·4–0·6)
Occupational carcinogens	1·0 (0·5–1·9)	1·1 (0·5–2·1)	1·1 (0·5–2·0)	1·1 (0·5–2·1)	0·3 (0·0–0·6)	0·1 (-0·4–0·4)	0·2 (-0·4–0·8)
Occupational exposure to asbestos	5·3 (4·5–5·9)	5·5 (4·8–6·2)	5·0 (4·3–5·5)	5·0 (3·8–6·2)	-0·2 (-1·1–0·7)	-0·5 (-1·8–0·7)	0·0 (-2·3–2·4)
Occupational exposure to arsenic	0·4 (0·0–1·0)	0·4 (0·0–1·1)	0·4 (0·0–1·0)	0·4 (0·0–1·1)	0·1 (-0·3–0·5)	-0·1 (-0·7–0·5)	0·2 (-0·7–1·0)
Occupational exposure to benzene	1·1 (0·1–3·1)	1·2 (0·1–3·5)	1·2 (0·1–3·6)	1·3 (0·1–3·7)	0·5 (0·1–0·8)	0·2 (-0·3–0·6)	0·3 (-0·3–0·8)
Occupational exposure to beryllium	0·0 (0·0–0·0)	0·0 (0·0–0·0)	0·0 (0·0–0·0)	0·0 (0·0–0·0)	0·2 (0·0–0·5)	0·1 (-0·2–0·4)	0·4 (-0·1–0·8)
Occupational exposure to cadmium	0·1 (0·0–0·1)	0·1 (0·1–0·1)	0·1 (0·0–0·1)	0·1 (0·0–0·1)	0·2 (-0·2–0·5)	0·0 (-0·5–0·5)	0·2 (-0·5–1·0)
Occupational exposure to chromium	0·1 (0·1–0·1)	0·1 (0·1–0·1)	0·1 (0·1–0·1)	0·1 (0·1–0·1)	0·2 (-0·1–0·6)	0·0 (-0·4–0·5)	0·3 (-0·3–0·9)
Occupational exposure to diesel engine exhaust	0·7 (0·7–0·8)	0·7 (0·7–0·8)	0·7 (0·7–0·8)	0·8 (0·7–0·8)	0·2 (-0·2–0·6)	0·2 (-0·3–0·7)	0·5 (-0·2–1·3)
Occupational exposure to formaldehyde	0·3 (0·2–0·3)	0·3 (0·2–0·3)	0·3 (0·2–0·3)	0·3 (0·2–0·3)	0·2 (-0·2–0·5)	-0·1 (-0·6–0·4)	0·1 (-0·6–0·9)
Occupational exposure to nickel	0·4 (0·0–1·4)	0·4 (0·0–1·4)	0·3 (0·0–1·3)	0·3 (0·0–1·3)	-0·1 (-0·6–0·4)	-0·2 (-0·8–0·5)	0·1 (-0·8–1·2)
Occupational exposure to polycyclic aromatic hydrocarbons	0·3 (0·2–0·3)	0·3 (0·3–0·3)	0·3 (0·3–0·3)	0·3 (0·3–0·3)	0·3 (0·0–0·6)	0·1 (-0·4–0·5)	0·3 (-0·3–0·9)
Occupational exposure to silica	3·3 (0·5–10·7)	3·3 (0·5–10·4)	3·1 (0·5–9·9)	3·1 (0·5–9·9)	-0·2 (-0·7–0·2)	-0·2 (-0·8–0·4)	0·2 (-0·7–1·1)
Occupational exposure to sulphuric acid	0·7 (0·1–2·2)	0·7 (0·1–2·4)	0·7 (0·1–2·3)	0·7 (0·1–2·3)	0·1 (-0·3–0·5)	0·0 (-0·6–0·5)	0·3 (-0·6–1·1)
Occupational exposure to trichloroethylene	0·1 (0·1–0·1)	0·1 (0·1–0·1)	0·1 (0·1–0·1)	0·1 (0·1–0·1)	0·3 (0·0–0·6)	0·1 (-0·3–0·5)	0·4 (-0·2–1·0)
Occupational asthmagens	16·1 (14·0–18·2)	16·7 (14·7–19·1)	15·8 (13·8–18·0)	16·2 (14·0–18·8)	0·0 (-0·4–0·4)	-0·2 (-0·7–0·3)	0·3 (-0·5–1·0)
Occupational particulate matter, gases, and fumes	6·4 (4·9–8·4)	6·3 (4·8–8·3)	6·0 (4·6–7·8)	5·5 (4·3–7·2)	-0·5 (-0·7–-0·2)	-0·6 (-0·9–-0·3)	-0·7 (-1·0–-0·3)
Occupational noise	7·1 (6·6–7·7)	7·1 (6·6–7·7)	7·0 (6·6–7·5)	6·8 (6·4–7·3)	-0·1 (-0·3–0·0)	-0·2 (-0·4–0·0)	-0·3 (-0·5–-0·1)
Occupational injuries	--	--	--	--	--	--	--
Occupational ergonomic factors	9·2 (7·7–11·1)	9·1 (7·6–10·9)	8·1 (6·9–9·8)	8·2 (6·8–10·0)	-0·4 (-0·9–0·1)	-0·5 (-1·1–0·1)	0·1 (-0·7–0·9)
**Behavioural risks**	**29·8 (26·4–33·5)**	**28·2 (24·6–31·9)**	**26·9 (23·9–30·8)**	**25·5 (22·0–29·3)**	**-0·5 (-0·9–-0·1)**	**-0·5 (-1·0–0·1)**	**-0·5 (-1·2–0·3)**
Child and maternal malnutrition	10·1 (6·8–14·0)	8·7 (5·9–11·6)	8·8 (6·1–11·7)	9·0 (6·1–12·7)	-0·3 (-1·2–0·5)	0·2 (-1·1–1·3)	0·2 (-1·6–2·0)
Suboptimal breastfeeding	68·8 (66·2–71·4)	66·7 (64·4–69·0)	62·8 (60·0–65·6)	62·2 (59·2–65·2)	-0·3 (-0·5–-0·2)	-0·3 (-0·5–-0·1)	-0·1 (-0·4–0·2)
Non-exclusive breastfeeding	64·1 (56·4–71·6)	59·6 (52·8–66·1)	48·7 (42·0–54·7)	46·2 (38·7–52·8)	-1·1 (-1·6–-0·5)	-1·2 (-1·9–-0·6)	-0·5 (-1·6–0·5)
Discontinued breastfeeding	88·7 (86·5–90·9)	86·4 (83·4–89·0)	84·4 (80·9–87·5)	84·6 (81·2–87·9)	-0·2 (-0·3–0·0)	-0·1 (-0·3–0·1)	0·0 (-0·3–0·3)
Child growth failure	1·3 (0·4–3·0)	1·2 (0·4–2·8)	1·1 (0·3–2·5)	1·0 (0·3–2·2)	-0·9 (-1·3–-0·6)	-1·1 (-1·6–-0·6)	-0·8 (-1·6–0·0)
Child underweight	2·8 (1·7–3·8)	2·6 (1·7–3·6)	2·3 (1·4–3·1)	2·1 (1·3–2·9)	-1·0 (-1·5–-0·4)	-1·1 (-1·8–-0·4)	-0·8 (-1·9–0·3)
Child wasting	0·9 (0·6–1·4)	0·8 (0·5–1·3)	0·9 (0·5–1·3)	0·8 (0·5–1·3)	-0·3 (-0·6–0·0)	-0·2 (-0·6–0·3)	-0·4 (-1·1–0·2)
Child stunting	3·8 (3·0–4·8)	3·6 (2·8–4·5)	3·1 (2·4–3·9)	2·8 (2·1–3·5)	-1·0 (-1·6–-0·4)	-1·2 (-2·0–-0·4)	-1·0 (-2·2–0·2)
Low birth weight and short gestation	16·0 (14·0–17·9)	17·0 (14·9–19·2)	20·4 (17·6–23·1)	21·1 (18·3–23·8)	0·9 (0·6–1·1)	1·0 (0·6–1·4)	0·3 (-0·5–1·1)
Short gestation	27·3 (24·1–30·7)	29·0 (25·5–32·9)	33·9 (29·5–38·1)	33·8 (29·6–38·6)	0·7 (0·4–1·0)	0·7 (0·3–1·2)	0·0 (-1·0–0·9)
Low birth weight	13·4 (12·3–14·7)	14·0 (12·6–15·3)	16·4 (14·9–17·9)	17·2 (15·8–18·8)	0·8 (0·6–1·1)	1·0 (0·6–1·4)	0·5 (-0·2–1·2)

Iron deficiency	3·5 (2·5–4·8)	3·1 (2·2–4·1)	3·1 (2·3–4·1)	3·2 (2·2–4·4)	-0·4 (-1·2–0·5)	0·2 (-1·0–1·2)	0·1 (-1·7–1·8)
Vitamin A deficiency	1·1 (0·0–2·2)	1·4 (0·0–3·0)	0·9 (0·0–2·0)	0·5 (0·0–1·2)	-2·3 (-4·6–0·0)	-4·7 (-7·4–0·0)	-5·1 (-9·1–0·0)
Zinc deficiency	2·1 (0·0–8·6)	1·3 (0·0–5·3)	1·1 (0·0–4·8)	1·0 (0·0–4·5)	-2·3 (-48·4–0·0)	-1·0 (-4·1–3·3)	-0·5 (-5·5–1·5)
Tobacco	37·4 (35·9–38·9)	33·5 (32·2–34·9)	29·8 (28·8–30·9)	26·6 (24·7–28·2)	-1·1 (-1·3–-0·9)	-1·1 (-1·4–-0·8)	-1·1 (-1·6–-0·5)
Smoking	36·9 (34·3–39·4)	31·9 (29·8–33·9)	27·6 (26·2–29·2)	24·7 (22·3–26·7)	-1·3 (-1·6–-1·0)	-1·2 (-1·7–-0·8)	-1·0 (-1·8–-0·3)
Chewing tobacco	4·2 (3·4–5·2)	4·9 (4·0–6·1)	4·8 (3·6–6·3)	4·4 (3·1–6·2)	0·2 (-1·1–1·4)	-0·5 (-2·3–1·2)	-0·8 (-3·3–1·9)
Second-hand smoke	28·7 (27·2–30·0)	26·3 (24·7–27·4)	23·8 (22·3–24·7)	21·2 (19·4–23·0)	-1·0 (-1·2–-0·7)	-1·0 (-1·4–-0·7)	-1·0 (-1·7–-0·4)
High alcohol use	27·8 (19·4–38·6)	26·4 (17·8–36·5)	27·0 (18·6–37·6)	27·8 (19·1–38·9)	0·0 (-1·1–1·2)	0·2 (-1·2–1·8)	0·3 (-1·8–2·3)
Drug use	0·9 (0·6–1·4)	1·2 (1·0–1·4)	2·1 (1·6–2·5)	4·2 (3·2–5·4)	5·0 (2·9–6·6)	6·0 (4·4–7·0)	6·6 (5·4–7·6)
Dietary risks	36·1 (26·8–46·8)	39·8 (30·4–51·4)	42·7 (33·8–54·5)	43·1 (33·9–55·3)	0·6 (0·3–0·9)	0·4 (0·1–0·7)	0·1 (-0·3–0·6)
Diet low in fruits	35·8 (31·2–39·2)	33·7 (29·8–36·6)	34·0 (30·2–37·3)	35·5 (31·2–39·8)	0·0 (-0·4–0·3)	0·2 (-0·3–0·8)	0·4 (-0·4–1·2)
Diet low in vegetables	22·0 (13·0–29·3)	26·8 (15·8–34·4)	33·1 (18·7–40·5)	33·0 (18·9–40·7)	1·3 (0·9–1·9)	1·0 (0·5–1·6)	0·0 (-0·9–0·7)
Diet low in legumes	39·2 (0·0–49·3)	39·8 (0·0–50·0)	43·6 (0·0–54·9)	43·6 (0·0–55·2)	0·3 (0·0–0·7)	0·4 (0·0–1·0)	0·0 (-0·8–0·8)
Diet low in whole grains	44·8 (37·3–50·8)	48·2 (40·2–54·7)	50·4 (41·6–57·6)	51·3 (41·7–58·9)	0·4 (0·1–0·7)	0·3 (-0·1–0·7)	0·1 (-0·4–0·7)
Diet low in nuts and seeds	14·4 (11·0–18·8)	9·2 (6·3–12·5)	6·4 (4·0–9·5)	6·2 (3·6–9·3)	-2·7 (-4·5–-1·2)	-1·9 (-4·3–0·3)	-0·3 (-3·8–3·2)
Diet low in milk	37·7 (33·8–42·5)	35·5 (31·1–40·5)	33·3 (29·1–38·0)	32·0 (27·4–37·2)	-0·5 (-1·0–-0·1)	-0·5 (-1·1–0·1)	-0·4 (-1·2–0·4)
Diet high in red meat	54·8 (0·0–74·4)	54·7 (0·0–73·7)	54·8 (0·0–74·1)	55·2 (0·0–74·6)	0·0 (-0·4–0·4)	0·0 (-0·6–0·6)	0·1 (-0·8–1·0)
Diet high in processed meat	45·7 (36·4–52·0)	56·5 (46·6–63·4)	64·0 (53·2–71·6)	63·1 (51·9–71·4)	1·0 (0·6–1·5)	0·5 (0·0–1·1)	-0·1 (-0·9–0·7)
Diet high in sugar-sweetened beverages	33·6 (25·3–41·8)	46·6 (36·3–54·6)	53·3 (43·4–61·7)	54·9 (42·9–66·0)	1·6 (0·8–2·4)	0·8 (-0·1–1·8)	0·3 (-1·0–1·5)
Diet low in fibre	41·6 (22·4–49·5)	32·8 (17·8–39·7)	28·4 (15·7–34·9)	23·9 (12·6–31·3)	-1·8 (-2·6–-0·9)	-1·5 (-2·7–-0·3)	-1·6 (-3·4–0·1)
Diet low in calcium	6·0 (4·8–7·3)	5·1 (4·1–6·3)	5·3 (4·2–6·5)	5·0 (4·0–6·2)	-0·6 (-0·8–-0·4)	-0·1 (-0·4–0·2)	-0·5 (-1·0–-0·1)
Diet low in seafood omega-3 fatty acids	39·3 (30·0–49·8)	46·8 (36·5–58·0)	48·8 (37·1–59·3)	42·3 (31·5–53·3)	0·2 (-0·2–0·7)	-0·5 (-1·1–0·1)	-1·3 (-2·1–-0·5)
Diet low in omega-6 polyunsaturated fatty acids	38·6 (24·5–49·2)	34·6 (22·6–43·8)	25·2 (17·1–32·5)	22·5 (15·2–29·2)	-1·7 (-2·5–-0·9)	-2·1 (-2·9–-1·1)	-1·0 (-2·0–-0·1)
Diet high in trans fatty acids	71·5 (64·0–78·6)	71·6 (63·8–78·9)	63·4 (55·3–70·8)	0·0 (0·0–0·0)	-65·8 (-66·1–-65·4)	-97·1 (-97·6–-96·5)	-184·3 (-185·3–-183·0)
Diet high in sodium	23·0 (0·6–67·0)	28·0 (1·8–72·7)	30·8 (3·1–74·8)	31·3 (3·1–76·4)	1·0 (0·2–6·4)	0·5 (-0·1–3·8)	0·1 (-2·4–2·1)
Intimate partner violence	31·6 (20·1–40·2)	31·7 (21·0–39·4)	28·8 (18·3–35·6)	27·0 (15·7–35·8)	-0·5 (-1·7–0·8)	-0·8 (-2·4–0·9)	-0·6 (-2·7–1·5)
Childhood sexual abuse and bullying	9·1 (5·9–14·3)	10·7 (6·9–16·9)	12·1 (7·8–19·0)	10·0 (6·5–15·1)	0·3 (-0·3–0·9)	-0·4 (-1·2–0·5)	-1·8 (-3·2–-0·3)
Childhood sexual abuse	8·6 (7·2–10·3)	9·1 (7·9–10·3)	10·3 (9·5–11·2)	9·9 (8·3–11·8)	0·4 (-0·3–1·3)	0·4 (-0·6–1·4)	-0·4 (-1·9–1·1)
Bullying victimization	8·0 (3·5–16·2)	10·1 (4·6–19·7)	11·4 (5·1–22·2)	8·5 (3·7–15·9)	0·2 (-0·6–0·9)	-0·8 (-2·0–0·2)	-2·7 (-4·8–-0·8)
Unsafe sex	--	--	--	--	--	--	--
Low physical activity	17·8 (13·0–22·9)	16·7 (12·6–21·2)	17·0 (13·1–21·8)	20·7 (15·6–27·0)	0·5 (-0·4–1·5)	1·0 (-0·2–2·3)	1·8 (0·0–3·7)
**Metabolic risks**	**25·2 (22·0–28·5)**	**29·3 (25·5–32·4)**	**32·7 (28·9–35·8)**	**36·4 (31·7–39·5)**	**1·2 (0·9–1·5)**	**1·0 (0·7–1·4)**	**1·0 (0·5–1·4)**
High fasting plasma glucose	11·8 (9·0–14·6)	15·1 (11·8–18·4)	18·8 (14·4–22·8)	23·1 (17·6–28·3)	2·2 (1·4–3·0)	2·0 (1·1–3·1)	1·8 (0·5–3·2)
High LDL cholesterol	63·7 (45·1–86·5)	54·0 (37·3–74·4)	48·3 (32·7–67·0)	47·1 (31·7–65·4)	-1·0 (-1·2–-0·8)	-0·7 (-0·9–-0·5)	-0·2 (-0·5–0·0)
High systolic blood pressure	28·4 (18·9–39·5)	24·1 (16·9–33·2)	17·5 (11·2–25·1)	25·2 (15·7–36·5)	-0·4 (-1·3–0·5)	0·2 (-1·1–1·3)	3·3 (0·9–5·5)
High body-mass index	28·3 (23·8–32·6)	33·6 (28·3–37·9)	37·7 (32·3–41·8)	41·0 (34·8–45·2)	1·2 (0·9–1·5)	1·0 (0·7–1·3)	0·8 (0·3–1·2)
Low bone mineral density	21·5 (15·5–28·2)	19·5 (13·7–26·4)	20·6 (14·4–26·9)	22·3 (15·8–29·3)	0·1 (-0·4–0·6)	0·6 (-0·1–1·4)	0·7 (-0·8–2·1)
Kidney dysfunction	2·8 (2·3–3·7)	2·9 (2·4–3·8)	2·9 (2·3–3·8)	3·0 (2·4–3·9)	0·2 (0·1–0·3)	0·1 (0·0–0·2)	0·4 (0·1–0·7)

Nebraska

Risk Names	SEV 1990	SEV 2000	SEV 2010	SEV 2021	Annualised rate of change 1990 to 2021	Annualised rate of change 2000 to 2021	Annualised rate of change 2010 to 2021
**All risk factors**	**26·9 (23·8–30·3)**	**27·0 (24·2–30·3)**	**27·5 (24·6–30·6)**	**27·3 (24·0–30·5)**	**0·1 (-0·3–0·4)**	**0·1 (-0·4–0·4)**	**-0·1 (-0·7–0·5)**
**Environmental/occupational risks**	**17·2 (11·1–28·5)**	**16·5 (11·0–27·0)**	**15·2 (10·1–25·2)**	**13·4 (8·5–22·6)**	**-0·8 (-1·5–-0·2)**	**-1·0 (-1·4–-0·5)**	**-1·2 (-1·7–-0·6)**
Unsafe water, sanitation, and handwashing	1·8 (0·9–2·9)	1·7 (0·8–2·8)	1·3 (0·5–2·2)	1·1 (0·4–1·8)	-1·7 (-4·2–0·3)	-2·2 (-5·3–0·4)	-2·1 (-5·9–1·8)
Unsafe water source	2·6 (0·9–5·1)	2·4 (0·8–4·9)	1·0 (0·4–2·0)	0·8 (0·4–1·4)	-3·9 (-6·5–-0·9)	-5·4 (-9·2–-1·5)	-2·2 (-7·5–3·6)
Unsafe sanitation	5·6 (3·1–9·3)	4·6 (2·5–8·0)	3·6 (2·0–6·4)	2·6 (1·3–4·6)	-2·4 (-4·9–-0·4)	-2·6 (-5·5–-0·2)	-2·9 (-6·4–0·4)
No access to handwashing facility	1·5 (0·5–3·0)	1·6 (0·5–3·0)	1·6 (0·5–3·0)	1·4 (0·4–2·7)	-0·4 (-3·6–2·6)	-0·7 (-4·8–3·5)	-1·7 (-7·3–4·0)
Air pollution	19·4 (4·6–30·2)	17·6 (5·8–26·7)	10·9 (3·6–21·3)	8·0 (1·6–17·5)	-2·9 (-6·0–-1·0)	-3·8 (-7·4–-1·9)	-2·8 (-8·4–-1·3)
Particulate matter pollution	9·2 (2·4–18·2)	8·3 (5·2–12·1)	5·9 (3·1–9·2)	3·4 (1·1–6·3)	-3·3 (-6·9–0·2)	-4·3 (-8·1–-2·6)	-5·1 (-11·0–-2·5)
Ambient particulate matter pollution	14·2 (3·6–27·6)	12·8 (8·2–18·1)	9·0 (4·9–13·9)	5·1 (1·7–9·3)	-3·3 (-6·9–0·2)	-4·3 (-8·1–-2·6)	-5·1 (-11·0–-2·5)
Household air pollution from solid fuels	0·0 (0·0–0·0)	0·0 (0·0–0·0)	0·0 (0·0–0·0)	0·0 (0·0–0·0)	-3·7 (-37·4–-7·7)	-4·7 (-37·4–-8·1)	-5·8 (-35·7–-7·9)
Ambient ozone pollution	11·9 (6·7–18·6)	20·2 (13·9–27·6)	17·2 (10·9–24·6)	14·7 (9·1–21·4)	0·7 (-0·1–1·5)	-1·5 (-2·8–-0·4)	-1·4 (-3·9–0·6)
Ambient nitrogen dioxide pollution	64·0 (0·0–100·0)	57·6 (0·0–100·0)	31·6 (0·0–86·4)	27·7 (0·0–79·0)	-2·7 (-9·2–0·0)	-3·5 (-12·4–0·0)	-1·2 (-6·5–0·0)
Non-optimal temperature	38·3 (28·7–50·8)	38·3 (28·8–50·7)	38·4 (28·9–50·6)	38·8 (28·6–50·9)	0·0 (-0·2–0·3)	0·1 (-0·3–0·3)	0·1 (-0·4–0·4)
High temperature	46·4 (32·5–59·8)	43·7 (29·4–57·0)	43·1 (28·4–56·0)	44·8 (29·8–58·5)	-0·1 (-0·5–0·1)	0·1 (-0·3–0·5)	0·4 (-0·2–0·7)
Low temperature	30·3 (27·7–33·1)	30·6 (28·3–33·2)	31·2 (29·2–33·5)	30·9 (29·0–33·3)	0·1 (-0·1–0·2)	0·0 (-0·1–0·2)	-0·1 (-0·4–0·1)
Other environmental risks	26·4 (9·4–44·7)	25·1 (9·4–43·1)	22·9 (9·4–40·5)	20·5 (9·0–37·9)	-0·8 (-1·5–0·0)	-1·0 (-1·8–0·0)	-1·0 (-2·0–0·0)
Residential radon	30·1 (0·0–89·0)	30·1 (0·0–89·0)	30·1 (0·0–89·0)	30·1 (0·0–89·0)	0·0 (0·0–0·0)	0·0 (0·0–0·0)	0·0 (0·0–0·0)
Lead exposure	24·7 (0·0–31·2)	22·7 (0·0–28·3)	19·6 (0·0–24·6)	16·2 (0·0–20·1)	-1·4 (-1·8–0·0)	-1·6 (-2·1–0·0)	-1·7 (-2·6–0·0)
Occupational risks	3·0 (2·5–3·9)	3·2 (2·6–4·2)	3·1 (2·5–4·1)	3·0 (2·5–4·0)	0·1 (-0·2–0·3)	-0·2 (-0·5–0·1)	-0·1 (-0·5–0·4)
Occupational carcinogens	1·0 (0·5–1·9)	1·1 (0·5–2·2)	1·1 (0·5–2·1)	1·1 (0·5–2·1)	0·3 (-0·1–0·5)	0·0 (-0·4–0·4)	0·0 (-0·6–0·5)
Occupational exposure to asbestos	4·1 (3·6–4·5)	4·2 (3·7–4·6)	4·1 (3·6–4·5)	3·7 (2·9–4·6)	-0·3 (-1·2–0·4)	-0·6 (-1·8–0·6)	-0·9 (-3·2–1·3)
Occupational exposure to arsenic	0·5 (0·0–1·2)	0·5 (0·0–1·3)	0·5 (0·0–1·3)	0·5 (0·0–1·3)	0·1 (-0·4–0·6)	-0·2 (-0·8–0·4)	-0·1 (-0·9–0·8)
Occupational exposure to benzene	1·2 (0·1–3·4)	1·3 (0·1–3·8)	1·3 (0·1–3·8)	1·3 (0·1–3·9)	0·4 (0·1–0·8)	0·2 (-0·3–0·6)	0·2 (-0·4–0·7)
Occupational exposure to beryllium	0·0 (0·0–0·0)	0·0 (0·0–0·0)	0·0 (0·0–0·0)	0·0 (0·0–0·0)	0·3 (0·1–0·5)	0·1 (-0·2–0·4)	0·3 (-0·1–0·6)
Occupational exposure to cadmium	0·1 (0·1–0·1)	0·1 (0·1–0·1)	0·1 (0·1–0·1)	0·1 (0·1–0·1)	0·1 (-0·3–0·6)	-0·2 (-0·7–0·4)	0·0 (-0·7–0·7)
Occupational exposure to chromium	0·2 (0·1–0·2)	0·2 (0·2–0·2)	0·2 (0·2–0·2)	0·2 (0·1–0·2)	0·2 (-0·1–0·6)	-0·1 (-0·5–0·4)	0·1 (-0·6–0·7)
Occupational exposure to diesel engine exhaust	0·8 (0·7–0·8)	0·8 (0·7–0·9)	0·8 (0·8–0·9)	0·9 (0·8–0·9)	0·4 (0·0–0·7)	0·3 (-0·2–0·8)	0·5 (-0·1–1·1)
Occupational exposure to formaldehyde	0·3 (0·3–0·3)	0·3 (0·3–0·3)	0·3 (0·3–0·3)	0·3 (0·3–0·3)	0·1 (-0·3–0·5)	-0·2 (-0·7–0·3)	-0·1 (-0·8–0·6)
Occupational exposure to nickel	0·4 (0·0–1·6)	0·4 (0·0–1·7)	0·4 (0·0–1·6)	0·4 (0·0–1·6)	0·0 (-0·5–0·6)	-0·2 (-1·0–0·5)	-0·1 (-1·1–0·9)
Occupational exposure to polycyclic aromatic hydrocarbons	0·3 (0·3–0·3)	0·3 (0·3–0·4)	0·3 (0·3–0·3)	0·3 (0·3–0·4)	0·2 (-0·1–0·6)	0·0 (-0·5–0·4)	0·1 (-0·5–0·6)
Occupational exposure to silica	3·7 (0·6–12·2)	3·7 (0·6–12·1)	3·6 (0·6–11·4)	3·6 (0·6–11·4)	-0·1 (-0·5–0·4)	-0·2 (-0·8–0·5)	0·0 (-0·8–0·9)
Occupational exposure to sulphuric acid	0·8 (0·2–2·6)	0·8 (0·2–2·8)	0·8 (0·2–2·7)	0·8 (0·2–2·6)	0·1 (-0·3–0·5)	-0·2 (-0·7–0·4)	0·0 (-0·7–0·8)
Occupational exposure to trichloroethylene	0·1 (0·1–0·1)	0·1 (0·1–0·1)	0·1 (0·1–0·1)	0·1 (0·1–0·1)	0·3 (0·0–0·6)	0·1 (-0·3–0·5)	0·2 (-0·3–0·7)
Occupational asthmagens	17·1 (14·9–19·3)	18·3 (16·2–20·7)	17·6 (15·5–19·9)	17·8 (15·6–20·1)	0·1 (-0·2–0·5)	-0·1 (-0·6–0·4)	0·1 (-0·6–0·7)
Occupational particulate matter, gases, and fumes	6·9 (5·3–9·2)	6·9 (5·3–9·1)	6·6 (5·1–8·8)	6·2 (4·8–8·2)	-0·4 (-0·6–-0·1)	-0·5 (-0·8–-0·2)	-0·6 (-0·9–-0·2)
Occupational noise	7·3 (6·8–7·9)	7·4 (6·9–8·0)	7·4 (6·9–7·9)	7·2 (6·8–7·7)	0·0 (-0·2–0·1)	-0·1 (-0·3–0·0)	-0·2 (-0·4–0·0)
Occupational injuries	--	--	--	--	--	--	--
Occupational ergonomic factors	9·0 (7·6–10·8)	9·1 (7·8–10·9)	8·4 (7·1–10·0)	8·4 (7·1–10·2)	-0·2 (-0·7–0·2)	-0·4 (-1·0–0·1)	0·0 (-0·7–0·7)
**Behavioural risks**	**29·7 (26·5–33·6)**	**28·1 (24·9–32·1)**	**26·2 (23·0–29·8)**	**24·6 (21·6–28·6)**	**-0·6 (-1·0–-0·2)**	**-0·6 (-1·2–-0·1)**	**-0·6 (-1·4–0·1)**
Child and maternal malnutrition	9·9 (6·4–13·9)	8·5 (5·8–11·9)	8·7 (5·9–11·9)	8·9 (6·0–12·6)	-0·3 (-1·2–0·6)	0·2 (-1·0–1·4)	0·3 (-1·6–2·0)
Suboptimal breastfeeding	68·8 (66·2–71·4)	66·7 (64·2–69·3)	62·8 (59·9–65·8)	62·2 (59·3–65·4)	-0·3 (-0·5–-0·2)	-0·3 (-0·5–-0·1)	-0·1 (-0·3–0·2)
Non-exclusive breastfeeding	64·0 (55·7–71·1)	59·5 (51·9–66·5)	48·6 (41·3–55·6)	46·4 (38·8–54·3)	-1·0 (-1·6–-0·6)	-1·2 (-1·8–-0·6)	-0·4 (-1·5–0·6)
Discontinued breastfeeding	88·7 (86·2–91·4)	86·3 (83·8–89·4)	84·4 (80·8–87·5)	84·6 (81·4–87·7)	-0·2 (-0·3–0·0)	-0·1 (-0·3–0·1)	0·0 (-0·3–0·3)
Child growth failure	1·2 (0·4–2·8)	1·1 (0·4–2·6)	1·0 (0·3–2·2)	0·9 (0·3–2·0)	-0·9 (-1·3–-0·6)	-1·1 (-1·7–-0·6)	-0·8 (-1·6–0·0)
Child underweight	2·5 (1·6–3·5)	2·4 (1·6–3·3)	2·1 (1·3–2·8)	1·9 (1·2–2·6)	-0·9 (-1·4–-0·5)	-1·2 (-1·9–-0·6)	-0·7 (-1·7–0·2)
Child wasting	0·8 (0·5–1·3)	0·8 (0·5–1·3)	0·8 (0·5–1·2)	0·8 (0·5–1·2)	-0·3 (-0·6–0·0)	-0·2 (-0·7–0·2)	-0·4 (-1·0–0·2)
Child stunting	3·5 (2·8–4·4)	3·4 (2·6–4·2)	2·9 (2·3–3·7)	2·6 (2·0–3·3)	-1·0 (-1·6–-0·4)	-1·2 (-2·1–-0·4)	-1·0 (-2·2–0·4)
Low birth weight and short gestation	21·1 (18·6–24·0)	21·9 (19·3–24·6)	23·1 (20·2–26·2)	24·1 (21·2–27·3)	0·4 (0·2–0·7)	0·5 (0·1–0·8)	0·4 (-0·3–1·1)
Short gestation	38·7 (34·3–43·4)	40·5 (35·6–45·1)	41·7 (36·9–46·8)	42·5 (38·1–48·0)	0·3 (0·0–0·6)	0·2 (-0·1–0·6)	0·2 (-0·6–1·0)
Low birth weight	16·9 (15·5–18·7)	17·3 (15·9–19·0)	18·3 (16·6–20·1)	19·4 (17·5–21·1)	0·4 (0·2–0·7)	0·5 (0·2–0·9)	0·5 (-0·1–1·2)

Iron deficiency	3·5 (2·3–4·8)	3·0 (2·1–4·0)	3·1 (2·2–4·1)	3·2 (2·2–4·3)	-0·3 (-1·1–0·5)	0·2 (-0·8–1·4)	0·2 (-1·5–1·9)
Vitamin A deficiency	0·9 (0·0–1·9)	1·3 (0·0–2·7)	0·8 (0·0–1·8)	0·5 (0·0–1·0)	-2·1 (-4·6–0·0)	-4·7 (-7·5–0·0)	-5·1 (-9·0–0·0)
Zinc deficiency	1·9 (0·0–7·6)	1·1 (0·0–4·7)	1·0 (0·0–4·2)	1·0 (0·0–4·0)	-2·2 (-47·5–1·0)	-0·6 (-2·7–5·3)	-0·3 (-3·4–5·4)
Tobacco	37·7 (36·1–39·1)	34·3 (33·0–35·5)	28·2 (27·3–29·3)	24·3 (22·6–26·0)	-1·4 (-1·6–-1·2)	-1·7 (-2·0–-1·3)	-1·4 (-2·0–-0·8)
Smoking	35·6 (33·3–37·5)	31·3 (29·4–33·2)	25·9 (24·5–27·4)	22·1 (20·1–24·3)	-1·5 (-1·9–-1·2)	-1·7 (-2·1–-1·2)	-1·4 (-2·3–-0·6)
Chewing tobacco	2·5 (2·0–3·1)	2·9 (2·3–3·6)	2·9 (2·2–4·1)	2·8 (1·9–4·0)	0·4 (-0·9–1·7)	-0·3 (-2·0–1·5)	-0·6 (-3·1–2·1)
Second-hand smoke	30·4 (28·9–31·6)	28·1 (26·6–29·2)	23·0 (21·5–23·9)	20·1 (18·5–21·7)	-1·3 (-1·6–-1·1)	-1·6 (-1·9–-1·2)	-1·2 (-1·8–-0·6)
High alcohol use	28·2 (20·5–39·7)	26·6 (18·8–37·3)	27·2 (19·8–38·3)	27·7 (19·0–38·1)	-0·1 (-1·1–1·0)	0·2 (-1·3–1·6)	0·2 (-1·9–2·0)
Drug use	0·6 (0·3–1·2)	0·5 (0·3–0·8)	0·8 (0·7–1·1)	2·5 (1·9–3·2)	4·8 (1·8–7·8)	7·7 (4·2–10·6)	10·1 (6·1–12·7)
Dietary risks	38·1 (28·9–49·3)	41·0 (31·4–52·9)	43·3 (34·1–55·7)	43·5 (34·5–55·5)	0·4 (0·2–0·7)	0·3 (0·0–0·6)	0·0 (-0·4–0·5)
Diet low in fruits	37·7 (31·9–41·0)	34·4 (29·8–37·3)	33·5 (29·0–36·5)	34·6 (28·9–38·8)	-0·3 (-0·7–0·1)	0·0 (-0·5–0·6)	0·3 (-0·6–1·2)
Diet low in vegetables	25·2 (15·0–33·3)	27·2 (15·7–35·2)	31·6 (17·8–39·2)	31·1 (17·7–38·8)	0·7 (0·3–1·1)	0·6 (0·2–1·1)	-0·1 (-1·0–0·6)
Diet low in legumes	38·1 (0·0–48·0)	38·3 (0·0–48·1)	42·3 (0·0–52·7)	42·4 (0·0–53·6)	0·3 (0·0–0·7)	0·5 (0·0–0·9)	0·0 (-0·7–0·8)
Diet low in whole grains	45·6 (37·7–52·0)	48·9 (40·5–55·9)	50·7 (42·2–57·7)	51·2 (42·3–58·5)	0·4 (0·1–0·6)	0·2 (-0·1–0·6)	0·1 (-0·5–0·7)
Diet low in nuts and seeds	12·8 (9·1–17·0)	7·5 (4·9–10·8)	5·3 (3·2–7·7)	5·2 (2·9–8·2)	-2·9 (-4·9–-1·3)	-1·7 (-4·8–0·9)	-0·1 (-4·0–3·6)
Diet low in milk	37·4 (33·4–41·5)	34·3 (30·1–39·2)	32·3 (28·0–36·9)	31·3 (26·7–36·3)	-0·6 (-1·1–-0·1)	-0·4 (-1·1–0·2)	-0·3 (-1·1–0·6)
Diet high in red meat	57·7 (0·0–77·1)	57·8 (0·0–77·8)	57·5 (0·0–76·7)	57·5 (0·0–76·7)	0·0 (-0·4–0·3)	0·0 (-0·6–0·5)	0·0 (-0·8–0·7)
Diet high in processed meat	49·4 (39·4–55·3)	60·3 (48·2–67·5)	66·7 (53·4–74·5)	65·4 (53·5–73·9)	0·9 (0·5–1·3)	0·4 (-0·1–0·9)	-0·2 (-0·9–0·5)
Diet high in sugar-sweetened beverages	36·7 (27·1–46·5)	50·8 (40·2–60·0)	56·5 (45·9–65·9)	57·6 (45·7–69·0)	1·5 (0·6–2·3)	0·6 (-0·3–1·6)	0·2 (-1·2–1·5)
Diet low in fibre	39·0 (21·9–46·1)	29·7 (16·2–36·3)	26·2 (14·1–32·7)	22·0 (12·4–28·9)	-1·8 (-2·8–-0·9)	-1·4 (-2·8–-0·1)	-1·6 (-3·4–0·2)
Diet low in calcium	5·6 (4·6–6·9)	4·7 (3·8–5·9)	5·0 (4·0–6·2)	4·8 (3·8–6·0)	-0·5 (-0·7–-0·3)	0·0 (-0·2–0·3)	-0·4 (-0·7–0·0)
Diet low in seafood omega-3 fatty acids	49·4 (39·2–59·7)	56·0 (45·3–67·2)	58·7 (47·6–70·2)	52·9 (42·4–64·2)	0·2 (-0·1–0·6)	-0·3 (-0·7–0·1)	-0·9 (-1·6–-0·4)
Diet low in omega-6 polyunsaturated fatty acids	35·9 (23·1–45·6)	31·2 (20·8–39·7)	23·1 (16·3–30·0)	20·9 (14·6–27·3)	-1·8 (-2·5–-1·0)	-1·9 (-2·8–-1·1)	-0·9 (-2·0–0·0)
Diet high in trans fatty acids	71·5 (63·6–78·6)	71·7 (63·8–79·1)	63·3 (54·5–71·1)	0·0 (0·0–0·0)	-65·8 (-66·1–-65·4)	-97·1 (-97·6–-96·5)	-184·2 (-185·3–-182·9)
Diet high in sodium	23·0 (0·7–66·9)	27·8 (1·9–73·7)	30·8 (3·2–72·9)	31·3 (3·1–77·1)	1·0 (0·3–5·8)	0·6 (-0·1–3·7)	0·1 (-2·3–1·8)
Intimate partner violence	30·8 (18·4–39·5)	30·8 (20·3–38·7)	27·9 (19·0–34·4)	26·2 (15·4–34·5)	-0·5 (-1·8–0·7)	-0·8 (-2·5–0·8)	-0·6 (-2·9–1·4)
Childhood sexual abuse and bullying	9·3 (6·2–14·4)	11·2 (7·5–17·4)	12·7 (8·4–19·9)	10·6 (7·0–16·4)	0·4 (-0·1–1·0)	-0·3 (-1·1–0·6)	-1·6 (-3·1–-0·1)
Childhood sexual abuse	9·2 (7·8–11·0)	10·4 (8·9–12·2)	11·8 (10·2–13·6)	11·0 (9·0–13·4)	0·6 (-0·2–1·4)	0·3 (-0·8–1·4)	-0·6 (-2·1–1·0)
Bullying victimization	8·0 (3·5–15·8)	10·0 (4·6–19·5)	11·3 (5·1–21·9)	8·7 (3·6–17·5)	0·3 (-0·4–1·1)	-0·6 (-1·7–0·4)	-2·3 (-4·4–-0·5)
Unsafe sex	--	--	--	--	--	--	--
Low physical activity	22·9 (17·5–29·9)	22·5 (17·4–28·1)	21·8 (17·2–26·9)	23·8 (18·4–30·4)	0·1 (-0·8–1·1)	0·3 (-1·0–1·5)	0·8 (-1·0–2·7)
**Metabolic risks**	**25·6 (22·4–29·0)**	**30·8 (27·3–33·9)**	**34·7 (30·6–37·7)**	**39·0 (34·1–42·2)**	**1·4 (1·1–1·7)**	**1·1 (0·8–1·5)**	**1·1 (0·6–1·5)**
High fasting plasma glucose	12·9 (9·4–15·6)	16·6 (12·8–19·9)	21·2 (16·0–25·0)	25·0 (18·7–30·4)	2·1 (1·5–2·9)	1·9 (1·0–2·9)	1·5 (0·4–2·7)
High LDL cholesterol	63·8 (45·4–86·8)	53·9 (37·3–74·9)	48·2 (32·4–66·8)	46·8 (31·5–65·3)	-1·0 (-1·2–-0·8)	-0·7 (-0·9–-0·5)	-0·3 (-0·5–0·0)
High systolic blood pressure	27·8 (18·4–39·0)	23·9 (16·6–33·0)	18·0 (11·7–26·0)	25·4 (15·8–36·7)	-0·3 (-1·3–0·7)	0·3 (-0·9–1·5)	3·1 (0·8–5·5)
High body-mass index	29·2 (24·6–33·7)	35·6 (30·3–39·6)	40·1 (34·2–44·1)	44·6 (37·6–48·5)	1·4 (1·0–1·7)	1·1 (0·8–1·4)	1·0 (0·5–1·4)
Low bone mineral density	21·0 (15·2–27·9)	19·2 (13·7–26·1)	20·1 (14·4–26·9)	22·0 (16·2–28·8)	0·1 (-0·3–0·7)	0·6 (-0·1–1·3)	0·8 (-0·6–2·3)
Kidney dysfunction	2·9 (2·3–3·7)	2·9 (2·4–3·9)	2·9 (2·4–3·9)	3·0 (2·4–4·0)	0·2 (0·1–0·3)	0·1 (0·0–0·3)	0·4 (0·1–0·7)

Nevada

Risk Names	SEV 1990	SEV 2000	SEV 2010	SEV 2021	Annualised rate of change 1990 to 2021	Annualised rate of change 2000 to 2021	Annualised rate of change 2010 to 2021
**All risk factors**	**27·0 (23·9–30·5)**	**27·4 (24·4–30·7)**	**27·5 (24·7–30·8)**	**28·0 (24·7–31·3)**	**0·1 (-0·2–0·5)**	**0·1 (-0·3–0·5)**	**0·2 (-0·4–0·7)**
**Environmental/occupational risks**	**17·6 (11·7–28·2)**	**17·1 (11·5–27·0)**	**15·3 (10·0–24·5)**	**14·1 (9·1–23·7)**	**-0·7 (-1·4–-0·2)**	**-0·9 (-1·3–-0·4)**	**-0·7 (-1·2–-0·3)**
Unsafe water, sanitation, and handwashing	2·1 (1·2–3·5)	1·9 (0·9–3·2)	1·4 (0·5–2·5)	1·2 (0·5–2·1)	-1·8 (-3·9–0·0)	-2·2 (-5·0–0·2)	-1·3 (-5·2–2·6)
Unsafe water source	3·1 (1·2–6·2)	2·8 (1·1–6·1)	1·1 (0·4–2·1)	0·9 (0·4–1·7)	-4·0 (-6·7–-1·0)	-5·3 (-9·1–-1·4)	-1·5 (-7·1–4·1)
Unsafe sanitation	7·2 (3·9–12·1)	5·6 (2·8–9·6)	4·0 (2·0–7·0)	3·4 (1·8–5·8)	-2·5 (-4·7–-0·5)	-2·5 (-5·2–0·1)	-1·5 (-4·6–1·8)
No access to handwashing facility	1·7 (0·5–3·3)	1·8 (0·6–3·4)	1·7 (0·6–3·2)	1·5 (0·5–2·9)	-0·4 (-3·2–2·3)	-0·7 (-4·7–2·9)	-1·1 (-7·0–4·3)
Air pollution	22·1 (4·9–32·3)	21·7 (5·9–27·8)	13·5 (2·8–23·2)	7·7 (1·3–17·4)	-3·4 (-7·6–-1·2)	-4·9 (-11·7–-1·9)	-5·1 (-12·9–-1·9)
Particulate matter pollution	9·3 (2·5–18·1)	8·1 (5·2–11·6)	4·2 (1·6–7·2)	2·6 (0·6–5·4)	-4·1 (-9·2–-0·9)	-5·5 (-10·9–-3·3)	-4·4 (-11·7–-1·7)
Ambient particulate matter pollution	14·4 (3·8–27·7)	12·5 (8·1–17·3)	6·4 (2·4–10·6)	3·9 (0·8–8·2)	-4·2 (-9·3–-0·9)	-5·5 (-11·0–-3·3)	-4·4 (-11·7–-1·7)
Household air pollution from solid fuels	0·0 (0·0–0·0)	0·0 (0·0–0·0)	0·0 (0·0–0·0)	0·0 (0·0–0·0)	-3·6 (-20·3–-3·8)	-5·8 (-13·1–-1·3)	-5·4 (-6·8–8·6)
Ambient ozone pollution	23·5 (16·3–30·5)	33·6 (26·8–41·0)	30·9 (24·2–38·1)	24·8 (17·9–32·5)	0·2 (-0·5–0·8)	-1·5 (-2·3–-0·7)	-2·0 (-3·5–-0·7)
Ambient nitrogen dioxide pollution	77·7 (0·0–100·0)	80·4 (0·0–100·0)	53·5 (0·0–100·0)	28·9 (0·0–82·6)	-3·2 (-13·1–0·0)	-4·9 (-20·3–0·0)	-5·6 (-26·0–0·0)
Non-optimal temperature	41·8 (34·7–51·7)	45·1 (37·0–55·6)	47·0 (39·3–57·3)	53·3 (43·9–64·2)	0·8 (0·5–1·0)	0·8 (0·5–1·0)	1·1 (0·6–1·6)
High temperature	51·2 (40·2–62·4)	55·4 (43·9–66·7)	61·5 (50·0–72·6)	72·4 (59·9–83·1)	1·1 (0·7–1·5)	1·3 (0·9–1·7)	1·5 (0·9–2·0)
Low temperature	31·9 (29·1–35·3)	31·9 (28·6–35·8)	32·9 (29·7–36·9)	31·6 (28·5–35·7)	0·0 (-0·2–0·1)	0·0 (-0·2–0·0)	-0·4 (-0·6–-0·2)
Other environmental risks	28·6 (6·1–46·7)	27·0 (6·0–45·1)	24·2 (6·0–42·0)	21·2 (6·0–38·7)	-1·0 (-1·5–0·0)	-1·2 (-1·9–0·0)	-1·2 (-2·2–0·0)
Residential radon	25·4 (0·0–80·7)	25·4 (0·0–80·7)	25·4 (0·0–80·7)	25·4 (0·0–80·7)	0·0 (0·0–0·0)	0·0 (0·0–0·0)	0·0 (0·0–0·0)
Lead exposure	30·0 (0·0–37·2)	27·7 (0·0–34·1)	23·5 (0·0–29·3)	19·3 (0·0–23·7)	-1·4 (-1·8–0·0)	-1·7 (-2·2–0·0)	-1·8 (-2·6–0·0)
Occupational risks	2·7 (2·2–3·6)	2·8 (2·3–3·7)	2·6 (2·1–3·4)	2·6 (2·1–3·4)	-0·1 (-0·4–0·1)	-0·3 (-0·7–0·0)	0·1 (-0·3–0·6)
Occupational carcinogens	1·0 (0·4–1·8)	1·0 (0·5–1·9)	0·9 (0·4–1·8)	1·0 (0·4–1·8)	0·0 (-0·3–0·3)	-0·2 (-0·7–0·1)	0·2 (-0·5–0·7)
Occupational exposure to asbestos	4·5 (3·9–5·0)	4·6 (4·1–5·0)	3·8 (3·4–4·2)	3·4 (2·7–4·2)	-0·9 (-1·7–-0·2)	-1·5 (-2·6–-0·4)	-1·0 (-3·3–0·8)
Occupational exposure to arsenic	0·4 (0·0–1·0)	0·4 (0·0–1·0)	0·4 (0·0–1·0)	0·4 (0·0–1·1)	0·0 (-0·5–0·5)	-0·1 (-0·7–0·5)	0·3 (-0·5–1·2)
Occupational exposure to benzene	1·1 (0·1–3·2)	1·2 (0·1–3·4)	1·1 (0·1–3·3)	1·2 (0·1–3·5)	0·3 (-0·1–0·6)	0·0 (-0·5–0·4)	0·3 (-0·3–0·9)
Occupational exposure to beryllium	0·0 (0·0–0·0)	0·0 (0·0–0·0)	0·0 (0·0–0·0)	0·0 (0·0–0·0)	0·2 (-0·1–0·5)	0·1 (-0·2–0·4)	0·5 (0·1–1·0)
Occupational exposure to cadmium	0·1 (0·0–0·1)	0·1 (0·0–0·1)	0·0 (0·0–0·1)	0·1 (0·0–0·1)	0·0 (-0·4–0·5)	-0·1 (-0·6–0·5)	0·4 (-0·3–1·1)
Occupational exposure to chromium	0·1 (0·1–0·1)	0·1 (0·1–0·1)	0·1 (0·1–0·1)	0·1 (0·1–0·1)	0·1 (-0·3–0·5)	-0·1 (-0·5–0·4)	0·4 (-0·3–1·1)
Occupational exposure to diesel engine exhaust	0·7 (0·7–0·8)	0·7 (0·7–0·8)	0·7 (0·6–0·7)	0·7 (0·7–0·8)	0·1 (-0·3–0·5)	0·1 (-0·4–0·6)	0·6 (-0·1–1·3)
Occupational exposure to formaldehyde	0·3 (0·2–0·3)	0·3 (0·2–0·3)	0·2 (0·2–0·3)	0·3 (0·2–0·3)	0·0 (-0·4–0·4)	-0·2 (-0·8–0·4)	0·2 (-0·5–1·0)
Occupational exposure to nickel	0·3 (0·0–1·3)	0·3 (0·0–1·3)	0·3 (0·0–1·2)	0·3 (0·0–1·3)	-0·1 (-0·6–0·4)	-0·2 (-0·9–0·5)	0·3 (-0·6–1·3)
Occupational exposure to polycyclic aromatic hydrocarbons	0·3 (0·2–0·3)	0·3 (0·3–0·3)	0·3 (0·3–0·3)	0·3 (0·3–0·3)	0·1 (-0·2–0·5)	-0·1 (-0·5–0·4)	0·4 (-0·2–1·0)
Occupational exposure to silica	3·2 (0·5–10·4)	3·1 (0·5–10·1)	2·8 (0·4–9·3)	3·0 (0·5–9·6)	-0·2 (-0·7–0·2)	-0·2 (-0·9–0·4)	0·4 (-0·6–1·4)
Occupational exposure to sulphuric acid	0·6 (0·1–2·1)	0·7 (0·1–2·2)	0·6 (0·1–2·1)	0·7 (0·1–2·2)	0·1 (-0·4–0·5)	-0·1 (-0·6–0·5)	0·4 (-0·4–1·2)
Occupational exposure to trichloroethylene	0·1 (0·1–0·1)	0·1 (0·1–0·1)	0·1 (0·1–0·1)	0·1 (0·1–0·1)	0·1 (-0·2–0·5)	0·0 (-0·4–0·4)	0·5 (-0·1–1·1)
Occupational asthmagens	16·4 (14·2–18·9)	16·8 (14·7–19·1)	15·2 (13·3–17·4)	15·7 (13·7–17·9)	-0·1 (-0·5–0·2)	-0·3 (-0·8–0·2)	0·3 (-0·4–1·0)
Occupational particulate matter, gases, and fumes	5·6 (4·3–7·4)	5·6 (4·2–7·3)	5·3 (4·1–6·9)	5·0 (3·9–6·4)	-0·4 (-0·7–-0·1)	-0·5 (-0·9–-0·2)	-0·5 (-1·0–-0·1)
Occupational noise	6·3 (5·7–6·9)	6·4 (5·9–7·0)	6·3 (5·9–6·8)	6·1 (5·7–6·6)	-0·1 (-0·3–0·1)	-0·2 (-0·4–0·0)	-0·3 (-0·5–0·0)
Occupational injuries	--	--	--	--	--	--	--
Occupational ergonomic factors	8·0 (6·6–9·7)	7·9 (6·6–9·6)	7·1 (6·0–8·7)	7·3 (6·0–8·9)	-0·3 (-0·8–0·1)	-0·4 (-1·0–0·1)	0·3 (-0·4–1·0)
**Behavioural risks**	**31·7 (28·0–35·9)**	**29·2 (25·8–33·2)**	**26·6 (23·4–30·3)**	**23·7 (20·5–27·4)**	**-0·9 (-1·4–-0·5)**	**-1·0 (-1·5–-0·5)**	**-1·1 (-1·7–-0·4)**
Child and maternal malnutrition	10·3 (7·2–15·1)	8·9 (6·1–12·5)	9·0 (6·1–12·1)	9·1 (6·2–13·0)	-0·4 (-1·3–0·4)	0·1 (-1·2–1·2)	0·2 (-1·6–1·9)
Suboptimal breastfeeding	68·9 (66·1–71·6)	66·9 (64·3–69·5)	63·1 (60·5–65·9)	62·4 (59·4–65·4)	-0·3 (-0·5–-0·2)	-0·3 (-0·6–-0·1)	-0·1 (-0·4–0·2)
Non-exclusive breastfeeding	64·6 (56·1–71·9)	60·2 (52·9–67·5)	49·2 (42·1–55·8)	46·9 (39·4–54·2)	-1·0 (-1·6–-0·5)	-1·2 (-2·0–-0·5)	-0·4 (-1·5–0·5)
Discontinued breastfeeding	88·8 (86·4–91·3)	86·5 (84·1–89·3)	84·6 (81·3–87·7)	84·8 (81·3–88·0)	-0·1 (-0·3–0·0)	-0·1 (-0·3–0·1)	0·0 (-0·3–0·3)
Child growth failure	1·4 (0·5–3·3)	1·3 (0·4–3·0)	1·1 (0·4–2·6)	1·0 (0·3–2·3)	-1·0 (-1·4–-0·6)	-1·1 (-1·7–-0·6)	-0·6 (-1·3–0·3)
Child underweight	3·0 (1·9–4·3)	2·8 (1·8–3·8)	2·3 (1·5–3·1)	2·2 (1·3–3·0)	-1·0 (-1·6–-0·5)	-1·1 (-1·9–-0·5)	-0·5 (-1·5–0·6)
Child wasting	0·9 (0·6–1·5)	0·9 (0·6–1·4)	0·9 (0·5–1·4)	0·8 (0·5–1·3)	-0·4 (-0·7–0·0)	-0·2 (-0·6–0·3)	-0·2 (-0·9–0·4)
Child stunting	4·1 (3·1–5·1)	3·8 (3·0–4·7)	3·2 (2·5–4·1)	2·9 (2·2–3·7)	-1·1 (-1·7–-0·5)	-1·2 (-2·0–-0·5)	-0·8 (-2·0–0·3)
Low birth weight and short gestation	20·7 (17·9–23·7)	22·2 (19·1–25·7)	23·9 (20·6–27·3)	22·0 (18·8–25·7)	0·2 (-0·1–0·5)	-0·1 (-0·5–0·4)	-0·8 (-1·6–0·0)
Short gestation	35·9 (30·9–41·5)	41·4 (36·1–48·1)	42·5 (36·9–48·7)	35·0 (30·3–40·7)	-0·1 (-0·4–0·2)	-0·8 (-1·3–-0·3)	-1·8 (-2·8–-0·9)
Low birth weight	15·6 (13·9–17·4)	15·7 (14·1–17·6)	17·4 (15·5–19·3)	17·3 (15·6–19·6)	0·3 (0·1–0·6)	0·5 (0·0–0·9)	0·0 (-0·8–0·7)

Iron deficiency	3·6 (2·5–5·1)	3·1 (2·2–4·3)	3·2 (2·3–4·2)	3·2 (2·3–4·5)	-0·3 (-1·1–0·5)	0·2 (-0·9–1·3)	0·2 (-1·6–1·8)
Vitamin A deficiency	1·2 (0·0–2·5)	1·6 (0·0–3·3)	0·9 (0·0–1·8)	0·6 (0·0–1·3)	-2·2 (-4·5–0·0)	-4·7 (-7·5–0·0)	-3·9 (-7·7–0·1)
Zinc deficiency	1·8 (0·0–7·6)	1·0 (0·0–4·3)	1·0 (0·0–4·1)	1·1 (0·0–4·5)	-1·6 (-38·9–0·0)	0·2 (-2·6–1·4)	1·0 (-9·8–8·9)
Tobacco	46·2 (44·3–47·9)	39·4 (37·8–40·8)	30·4 (29·3–31·5)	24·5 (22·9–26·2)	-2·0 (-2·3–-1·8)	-2·3 (-2·6–-2·0)	-1·9 (-2·5–-1·4)
Smoking	44·0 (41·0–46·7)	36·7 (34·6–38·6)	28·0 (26·5–29·6)	21·9 (20·1–24·0)	-2·2 (-2·5–-1·9)	-2·5 (-2·8–-2·1)	-2·2 (-2·9–-1·4)
Chewing tobacco	1·6 (1·2–2·1)	1·8 (1·3–2·3)	1·8 (1·3–2·5)	1·8 (1·2–2·5)	0·3 (-1·1–1·6)	0·0 (-1·9–1·9)	-0·2 (-3·1–2·6)
Second-hand smoke	36·3 (34·2–37·8)	31·7 (29·9–32·9)	25·1 (23·6–26·3)	21·0 (19·1–22·7)	-1·8 (-2·0–-1·5)	-2·0 (-2·4–-1·6)	-1·7 (-2·3–-1·0)
High alcohol use	26·0 (17·2–37·3)	24·6 (16·5–35·2)	25·7 (18·0–36·2)	24·6 (16·5–36·2)	-0·2 (-1·5–1·0)	0·0 (-1·5–1·5)	-0·4 (-2·4–1·3)
Drug use	1·0 (0·7–1·7)	1·1 (0·9–1·5)	1·9 (1·6–2·1)	5·0 (3·5–6·4)	5·2 (2·6–7·2)	7·2 (4·3–9·0)	9·0 (6·3–10·7)
Dietary risks	35·3 (26·6–46·5)	39·0 (29·6–50·8)	42·2 (32·6–54·5)	42·3 (33·0–54·6)	0·6 (0·3–0·9)	0·4 (0·1–0·7)	0·0 (-0·4–0·4)
Diet low in fruits	34·9 (29·5–38·2)	32·4 (27·4–35·4)	33·6 (29·3–36·4)	35·9 (30·6–40·2)	0·1 (-0·3–0·5)	0·5 (0·0–1·0)	0·6 (-0·3–1·5)
Diet low in vegetables	21·4 (13·7–28·9)	25·4 (15·4–32·8)	33·5 (19·7–40·5)	34·8 (20·7–42·5)	1·6 (1·0–2·2)	1·5 (1·0–2·2)	0·4 (-0·4–1·0)
Diet low in legumes	37·5 (0·0–46·4)	37·6 (0·0–47·2)	42·1 (0·0–53·0)	43·8 (0·0–54·8)	0·5 (0·0–0·8)	0·7 (0·0–1·2)	0·4 (-0·4–1·0)
Diet low in whole grains	44·4 (37·0–50·7)	47·2 (39·3–53·8)	49·0 (40·9–55·9)	48·9 (40·8–56·5)	0·3 (0·0–0·6)	0·2 (-0·2–0·5)	0·0 (-0·6–0·6)
Diet low in nuts and seeds	11·6 (7·8–15·5)	6·5 (4·2–9·3)	5·0 (3·0–7·6)	6·3 (3·8–9·8)	-2·0 (-3·8–-0·4)	-0·1 (-2·7–2·1)	2·0 (-1·5–5·3)
Diet low in milk	34·2 (30·0–39·1)	31·4 (27·4–36·2)	31·1 (26·8–35·6)	32·5 (28·1–37·7)	-0·2 (-0·6–0·3)	0·2 (-0·5–0·9)	0·4 (-0·4–1·3)
Diet high in red meat	49·5 (0·0–67·5)	50·0 (0·0–68·1)	49·8 (0·0–68·5)	48·8 (0·0–68·3)	0·0 (-0·5–0·4)	-0·1 (-0·7–0·5)	-0·2 (-1·2–0·8)
Diet high in processed meat	44·7 (34·7–50·4)	56·0 (44·2–62·4)	63·1 (49·2–70·9)	60·0 (48·1–67·8)	1·0 (0·5–1·4)	0·3 (-0·2–0·8)	-0·5 (-1·2–0·2)
Diet high in sugar-sweetened beverages	37·0 (27·5–45·2)	51·1 (39·8–59·6)	55·2 (43·6–64·1)	51·7 (40·4–61·8)	1·1 (0·3–1·9)	0·1 (-0·9–0·9)	-0·6 (-1·9–0·5)
Diet low in fibre	37·3 (20·1–44·5)	27·8 (15·2–34·6)	25·7 (13·8–31·9)	24·5 (13·3–31·4)	-1·4 (-2·3–-0·5)	-0·6 (-1·9–0·6)	-0·4 (-2·3–1·4)
Diet low in calcium	5·2 (4·1–6·5)	4·4 (3·5–5·4)	4·8 (3·9–6·0)	5·1 (4·1–6·3)	0·0 (-0·3–0·2)	0·7 (0·5–1·0)	0·6 (0·2–0·9)
Diet low in seafood omega-3 fatty acids	34·3 (25·2–43·5)	40·8 (31·3–51·0)	45·3 (35·0–56·1)	43·0 (33·2–53·8)	0·7 (0·3–1·2)	0·2 (-0·3–0·8)	-0·5 (-1·2–0·2)
Diet low in omega-6 polyunsaturated fatty acids	33·7 (22·5–42·9)	29·1 (19·5–37·2)	22·7 (15·7–29·0)	22·9 (15·9–29·5)	-1·2 (-1·8–-0·6)	-1·1 (-1·9–-0·4)	0·1 (-0·8–1·0)
Diet high in trans fatty acids	71·4 (63·9–78·4)	71·8 (64·2–78·7)	63·4 (55·9–71·0)	0·0 (0·0–0·0)	-65·8 (-66·1–-65·4)	-97·1 (-97·5–-96·6)	-184·3 (-185·3–-183·1)
Diet high in sodium	23·5 (0·5–70·3)	28·7 (1·7–76·5)	31·7 (3·5–76·7)	31·9 (3·3–76·3)	1·0 (0·2–6·6)	0·5 (-0·2–3·9)	0·1 (-2·9–1·8)
Intimate partner violence	32·9 (16·8–41·4)	32·7 (18·5–40·1)	29·5 (20·4–36·3)	28·4 (16·9–38·3)	-0·5 (-1·9–0·9)	-0·7 (-2·5–1·0)	-0·3 (-2·4–1·4)
Childhood sexual abuse and bullying	9·5 (6·3–14·8)	11·2 (7·4–17·2)	12·8 (8·4–20·1)	9·9 (6·9–14·7)	0·1 (-0·4–0·8)	-0·6 (-1·4–0·3)	-2·4 (-3·8–-0·9)
Childhood sexual abuse	9·6 (7·9–11·6)	10·3 (8·9–12·0)	11·9 (10·7–13·4)	10·8 (9·1–13·1)	0·4 (-0·5–1·2)	0·2 (-0·7–1·3)	-0·9 (-2·3–0·7)
Bullying victimization	8·0 (3·5–16·1)	10·0 (4·6–19·4)	11·4 (5·1–22·1)	7·7 (3·4–14·5)	-0·1 (-0·8–0·6)	-1·3 (-2·2–-0·2)	-3·6 (-5·4–-1·8)
Unsafe sex	--	--	--	--	--	--	--
Low physical activity	18·4 (13·9–23·9)	17·1 (13·0–21·6)	17·9 (14·0–22·5)	20·3 (14·7–26·3)	0·3 (-0·7–1·3)	0·8 (-0·5–2·0)	1·1 (-0·7–3·0)
**Metabolic risks**	**26·2 (22·9–29·4)**	**29·5 (26·1–32·4)**	**32·7 (28·9–35·6)**	**35·8 (31·3–38·7)**	**1·0 (0·7–1·3)**	**0·9 (0·6–1·3)**	**0·8 (0·3–1·3)**
High fasting plasma glucose	12·3 (9·5–15·0)	15·8 (12·0–19·1)	20·5 (15·3–24·4)	22·8 (17·2–26·9)	2·0 (1·3–2·6)	1·7 (1·0–2·5)	0·9 (-0·2–2·1)
High LDL cholesterol	63·6 (44·5–86·7)	53·9 (36·9–73·7)	48·3 (32·9–67·4)	46·2 (31·2–64·2)	-1·0 (-1·2–-0·8)	-0·7 (-0·9–-0·5)	-0·4 (-0·7–-0·1)
High systolic blood pressure	30·5 (20·7–42·0)	25·9 (18·2–35·7)	18·9 (12·3–27·1)	25·9 (16·5–36·9)	-0·5 (-1·6–0·4)	0·0 (-1·3–1·2)	2·8 (0·3–5·3)
High body-mass index	29·6 (25·2–34·3)	33·7 (28·7–37·8)	37·4 (31·9–41·5)	40·5 (34·2–44·5)	1·0 (0·7–1·3)	0·9 (0·5–1·2)	0·7 (0·2–1·2)
Low bone mineral density	22·1 (16·1–29·1)	19·9 (14·3–26·4)	20·5 (14·5–27·7)	22·8 (16·8–29·7)	0·1 (-0·4–0·6)	0·6 (0·0–1·4)	1·0 (-0·4–2·2)
Kidney dysfunction	3·0 (2·4–3·9)	3·0 (2·5–4·0)	3·0 (2·4–3·9)	3·1 (2·5–4·0)	0·1 (0·0–0·2)	0·0 (-0·1–0·2)	0·3 (0·0–0·6)

New Hampshire

Risk Names	SEV 1990	SEV 2000	SEV 2010	SEV 2021	Annualised rate of change 1990 to 2021	Annualised rate of change 2000 to 2021	Annualised rate of change 2010 to 2021
**All risk factors**	**27·8 (24·9–31·3)**	**27·7 (24·7–30·9)**	**27·9 (24·8–31·3)**	**28·2 (24·9–31·5)**	**0·0 (-0·3–0·4)**	**0·1 (-0·3–0·4)**	**0·1 (-0·5–0·6)**
**Environmental/occupational risks**	**15·9 (10·2–27·3)**	**15·8 (10·2–26·8)**	**13·9 (9·1–23·7)**	**12·6 (7·9–22·0)**	**-0·8 (-1·5–-0·2)**	**-1·1 (-1·5–-0·7)**	**-1·0 (-1·4–-0·4)**
Unsafe water, sanitation, and handwashing	1·6 (0·7–2·5)	1·5 (0·6–2·4)	1·2 (0·3–2·2)	1·0 (0·3–1·8)	-1·5 (-4·0–0·7)	-1·7 (-5·0–1·1)	-1·6 (-6·0–2·6)
Unsafe water source	2·2 (0·7–4·3)	1·9 (0·7–3·8)	0·8 (0·3–1·6)	0·7 (0·3–1·3)	-3·7 (-6·6–-0·5)	-5·0 (-8·7–-1·2)	-1·7 (-7·1–3·4)
Unsafe sanitation	4·3 (2·4–7·3)	3·3 (1·8–5·8)	2·6 (1·3–4·7)	2·0 (1·0–3·5)	-2·5 (-4·8–-0·3)	-2·4 (-5·1–0·3)	-2·3 (-5·7–1·1)
No access to handwashing facility	1·6 (0·5–3·3)	1·6 (0·5–3·1)	1·7 (0·6–3·1)	1·4 (0·4–2·7)	-0·3 (-3·3–2·8)	-0·5 (-4·6–3·5)	-1·3 (-7·2–4·4)
Air pollution	13·4 (3·4–24·6)	11·4 (3·9–22·0)	5·6 (1·8–12·9)	3·5 (0·3–10·6)	-4·3 (-10·4–-2·0)	-5·7 (-13·2–-3·3)	-4·3 (-16·8–-1·6)
Particulate matter pollution	7·4 (1·1–15·4)	6·1 (3·2–9·5)	3·9 (1·4–7·0)	1·6 (0·1–4·2)	-4·8 (-13·1–-0·6)	-6·2 (-17·3–-3·6)	-7·9 (-25·3–-3·9)
Ambient particulate matter pollution	11·3 (1·7–23·6)	9·3 (5·0–14·2)	5·9 (2·1–10·4)	2·5 (0·1–6·3)	-4·9 (-13·1–-0·6)	-6·3 (-17·3–-3·6)	-7·9 (-25·3–-3·9)
Household air pollution from solid fuels	0·0 (0·0–0·1)	0·0 (0·0–0·0)	0·0 (0·0–0·0)	0·0 (0·0–0·0)	-4·7 (-44·6–-9·2)	-5·2 (-45·3–-9·3)	-5·8 (-36·3–-6·7)
Ambient ozone pollution	18·1 (12·3–25·8)	16·4 (10·7–24·2)	13·9 (8·6–20·3)	5·9 (2·3–11·1)	-3·6 (-5·8–-2·4)	-4·9 (-7·7–-3·1)	-7·8 (-13·0–-4·8)
Ambient nitrogen dioxide pollution	38·1 (0·0–93·0)	33·5 (0·0–87·8)	11·2 (0·0–49·6)	11·2 (0·0–49·2)	-4·0 (-13·1–0·0)	-5·2 (-17·9–0·0)	0·0 (-1·7–0·3)
Non-optimal temperature	30·4 (24·2–40·3)	28·5 (23·9–36·8)	32·4 (25·2–42·5)	30·6 (24·1–40·2)	0·0 (-0·2–0·3)	0·3 (-0·1–0·9)	-0·5 (-1·0–-0·2)
High temperature	26·8 (16·3–38·4)	18·9 (10·3–29·6)	35·2 (23·3–48·4)	36·0 (23·8–50·1)	0·9 (0·4–1·7)	3·1 (1·6–5·0)	0·2 (-0·6–1·1)
Low temperature	27·4 (24·3–31·2)	27·4 (24·6–31·2)	27·9 (24·9–31·7)	26·6 (23·6–30·6)	-0·1 (-0·2–0·0)	-0·1 (-0·3–0·0)	-0·4 (-0·7–-0·2)
Other environmental risks	24·3 (7·3–44·7)	23·1 (7·3–43·4)	21·5 (7·7–41·8)	19·7 (7·3–39·3)	-0·7 (-1·4–0·0)	-0·7 (-1·6–0·0)	-0·8 (-1·8–0·0)
Residential radon	32·1 (0·0–91·3)	32·1 (0·0–91·3)	32·1 (0·0–91·3)	32·1 (0·0–91·3)	0·0 (0·0–0·0)	0·0 (0·0–0·0)	0·0 (0·0–0·0)
Lead exposure	20·7 (0·0–25·9)	19·0 (0·0–23·8)	16·7 (0·0–20·8)	14·2 (0·0–17·7)	-1·2 (-1·7–0·0)	-1·4 (-1·9–0·0)	-1·5 (-2·3–0·0)
Occupational risks	3·3 (2·7–4·3)	3·4 (2·8–4·4)	3·2 (2·6–4·2)	3·1 (2·5–4·1)	-0·2 (-0·4–0·1)	-0·4 (-0·7–-0·1)	-0·1 (-0·6–0·3)
Occupational carcinogens	1·1 (0·5–2·0)	1·1 (0·5–2·2)	1·1 (0·5–2·1)	1·1 (0·5–2·1)	0·1 (-0·3–0·4)	-0·2 (-0·6–0·1)	0·1 (-0·5–0·5)
Occupational exposure to asbestos	5·1 (4·5–5·7)	5·5 (5·0–6·0)	4·6 (4·2–5·1)	4·6 (3·6–5·6)	-0·4 (-1·2–0·3)	-0·9 (-2·1–0·1)	-0·2 (-2·4–1·7)
Occupational exposure to arsenic	0·6 (0·0–1·5)	0·6 (0·0–1·5)	0·6 (0·0–1·3)	0·5 (0·0–1·3)	-0·5 (-0·9–0·0)	-0·5 (-1·1–0·1)	-0·2 (-1·0–0·7)
Occupational exposure to benzene	1·2 (0·1–3·4)	1·3 (0·1–3·7)	1·3 (0·1–3·8)	1·3 (0·1–3·9)	0·4 (0·0–0·7)	0·1 (-0·3–0·5)	0·2 (-0·4–0·7)
Occupational exposure to beryllium	0·0 (0·0–0·0)	0·0 (0·0–0·0)	0·0 (0·0–0·0)	0·0 (0·0–0·0)	0·2 (0·0–0·4)	0·1 (-0·2–0·4)	0·3 (-0·1–0·7)
Occupational exposure to cadmium	0·1 (0·1–0·1)	0·1 (0·1–0·1)	0·1 (0·1–0·1)	0·1 (0·1–0·1)	-0·3 (-0·7–0·0)	-0·4 (-0·9–0·1)	-0·1 (-0·8–0·7)
Occupational exposure to chromium	0·2 (0·2–0·2)	0·2 (0·2–0·2)	0·2 (0·2–0·2)	0·2 (0·2–0·2)	-0·2 (-0·5–0·1)	-0·3 (-0·7–0·1)	0·0 (-0·7–0·6)
Occupational exposure to diesel engine exhaust	0·7 (0·7–0·8)	0·8 (0·7–0·8)	0·8 (0·7–0·8)	0·8 (0·7–0·9)	0·4 (0·1–0·8)	0·3 (-0·1–0·8)	0·6 (-0·1–1·3)
Occupational exposure to formaldehyde	0·4 (0·3–0·4)	0·4 (0·3–0·4)	0·3 (0·3–0·4)	0·3 (0·3–0·4)	-0·4 (-0·8–0·0)	-0·5 (-1·0–0·0)	-0·2 (-0·9–0·5)
Occupational exposure to nickel	0·5 (0·0–2·0)	0·5 (0·0–1·8)	0·4 (0·0–1·6)	0·4 (0·0–1·6)	-0·6 (-1·0–0·0)	-0·6 (-1·2–0·1)	-0·2 (-1·2–0·7)
Occupational exposure to polycyclic aromatic hydrocarbons	0·4 (0·3–0·4)	0·4 (0·3–0·4)	0·3 (0·3–0·4)	0·3 (0·3–0·4)	-0·2 (-0·5–0·2)	-0·3 (-0·7–0·1)	0·0 (-0·6–0·6)
Occupational exposure to silica	4·2 (0·7–13·9)	4·0 (0·6–13·1)	3·7 (0·6–11·5)	3·6 (0·5–11·6)	-0·5 (-1·0–0·0)	-0·5 (-1·1–0·1)	-0·1 (-0·9–0·8)
Occupational exposure to sulphuric acid	0·9 (0·2–3·0)	0·9 (0·2–2·9)	0·8 (0·2–2·7)	0·8 (0·2–2·7)	-0·3 (-0·7–0·0)	-0·4 (-0·9–0·1)	-0·1 (-0·9–0·7)
Occupational exposure to trichloroethylene	0·1 (0·1–0·1)	0·1 (0·1–0·1)	0·1 (0·1–0·1)	0·1 (0·1–0·1)	0·0 (-0·2–0·3)	-0·1 (-0·4–0·3)	0·2 (-0·3–0·7)
Occupational asthmagens	19·5 (17·2–22·2)	19·8 (17·6–22·5)	18·5 (16·3–21·1)	18·5 (16·0–21·0)	-0·2 (-0·5–0·1)	-0·3 (-0·8–0·1)	0·0 (-0·6–0·6)
Occupational particulate matter, gases, and fumes	7·8 (6·0–10·2)	7·5 (5·8–9·8)	7·1 (5·4–9·1)	6·5 (5·0–8·3)	-0·6 (-0·9–-0·4)	-0·7 (-1·0–-0·4)	-0·8 (-1·2–-0·4)
Occupational noise	7·5 (6·9–8·2)	7·5 (7·0–8·2)	7·4 (6·9–8·0)	7·2 (6·7–7·8)	-0·1 (-0·3–0·0)	-0·2 (-0·4–-0·1)	-0·3 (-0·5–-0·1)
Occupational injuries	--	--	--	--	--	--	--
Occupational ergonomic factors	8·9 (7·4–10·6)	8·9 (7·5–10·7)	8·1 (6·9–9·8)	8·2 (6·9–10·0)	-0·2 (-0·7–0·2)	-0·4 (-0·9–0·1)	0·1 (-0·6–0·7)
**Behavioural risks**	**32·1 (28·5–36·1)**	**30·7 (27·5–34·6)**	**28·6 (25·5–32·4)**	**27·0 (23·5–30·8)**	**-0·6 (-0·9–-0·2)**	**-0·6 (-1·1–-0·2)**	**-0·5 (-1·2–0·1)**
Child and maternal malnutrition	9·9 (6·8–14·1)	8·4 (5·9–11·6)	8·5 (5·9–11·6)	8·8 (5·9–12·4)	-0·4 (-1·3–0·5)	0·2 (-1·0–1·5)	0·3 (-1·6–2·1)
Suboptimal breastfeeding	68·7 (66·0–71·3)	66·5 (63·9–69·0)	62·7 (60·0–65·6)	62·0 (59·4–65·0)	-0·3 (-0·5–-0·2)	-0·3 (-0·5–-0·1)	-0·1 (-0·4–0·2)
Non-exclusive breastfeeding	63·8 (54·8–71·1)	59·1 (51·1–65·2)	48·3 (41·3–54·9)	45·8 (38·3–54·2)	-1·1 (-1·6–-0·6)	-1·2 (-2·0–-0·6)	-0·5 (-1·4–0·5)
Discontinued breastfeeding	88·6 (86·3–90·9)	86·2 (83·4–89·0)	84·2 (81·2–87·7)	84·4 (81·4–87·7)	-0·2 (-0·3–0·0)	-0·1 (-0·3–0·1)	0·0 (-0·3–0·3)
Child growth failure	1·1 (0·4–2·6)	1·1 (0·4–2·4)	0·9 (0·3–2·1)	0·9 (0·3–1·8)	-0·9 (-1·3–-0·6)	-1·0 (-1·6–-0·6)	-0·6 (-1·4–0·2)
Child underweight	2·4 (1·5–3·3)	2·2 (1·5–3·1)	1·9 (1·2–2·6)	1·8 (1·1–2·4)	-0·9 (-1·5–-0·4)	-1·1 (-1·9–-0·5)	-0·5 (-1·7–0·6)
Child wasting	0·8 (0·5–1·2)	0·7 (0·5–1·2)	0·7 (0·5–1·1)	0·7 (0·5–1·1)	-0·3 (-0·6–0·1)	-0·1 (-0·5–0·4)	-0·2 (-0·8–0·6)
Child stunting	3·4 (2·6–4·3)	3·1 (2·5–3·8)	2·7 (2·1–3·4)	2·5 (1·9–3·1)	-1·0 (-1·6–-0·5)	-1·2 (-2·0–-0·4)	-0·8 (-2·0–0·5)
Low birth weight and short gestation	13·5 (11·7–15·3)	16·2 (14·2–18·3)	19·0 (16·7–21·7)	18·5 (16·0–20·9)	1·0 (0·7–1·3)	0·6 (0·2–1·1)	-0·3 (-1·2–0·5)
Short gestation	29·7 (26·5–33·1)	31·0 (27·4–35·0)	33·9 (29·9–38·8)	32·5 (28·8–36·7)	0·3 (0·1–0·6)	0·2 (-0·1–0·6)	-0·4 (-1·3–0·4)
Low birth weight	11·4 (10·3–12·7)	13·1 (11·8–14·7)	15·2 (13·7–16·8)	15·1 (13·6–16·5)	0·9 (0·6–1·2)	0·7 (0·2–1·0)	0·0 (-0·8–0·7)

Iron deficiency	3·5 (2·5–4·9)	3·0 (2·1–4·1)	3·0 (2·2–4·1)	3·1 (2·2–4·3)	-0·4 (-1·2–0·5)	0·2 (-0·9–1·4)	0·2 (-1·5–1·9)
Vitamin A deficiency	0·7 (0·0–1·4)	0·8 (0·0–1·7)	0·5 (0·0–1·1)	0·3 (0·0–0·6)	-2·6 (-4·8–0·0)	-4·9 (-7·6–0·0)	-4·8 (-8·6–0·0)
Zinc deficiency	1·7 (0·0–7·4)	1·1 (0·0–4·4)	0·9 (0·0–3·8)	0·9 (0·0–3·8)	-2·1 (-46·1–1·8)	-0·7 (-4·5–5·0)	-0·1 (-4·5–3·0)
Tobacco	41·0 (39·2–42·6)	37·0 (35·6–38·3)	30·6 (29·5–31·7)	26·5 (24·7–28·2)	-1·4 (-1·6–-1·2)	-1·6 (-1·9–-1·3)	-1·3 (-1·8–-0·7)
Smoking	40·1 (37·6–42·4)	35·1 (32·7–37·1)	28·5 (26·9–30·2)	24·8 (22·5–27·0)	-1·6 (-1·9–-1·3)	-1·7 (-2·1–-1·2)	-1·3 (-2·0–-0·5)
Chewing tobacco	0·7 (0·5–0·9)	0·9 (0·6–1·2)	1·0 (0·7–1·4)	1·1 (0·7–1·5)	1·5 (0·1–3·0)	1·0 (-0·9–3·0)	0·5 (-2·8–3·3)
Second-hand smoke	32·3 (30·4–33·7)	29·5 (27·6–30·7)	24·6 (23·1–25·7)	21·5 (19·5–23·1)	-1·3 (-1·6–-1·1)	-1·5 (-1·8–-1·2)	-1·2 (-1·8–-0·6)
High alcohol use	32·1 (23·3–42·0)	31·4 (22·9–40·5)	32·2 (24·1–41·7)	32·6 (24·1–43·0)	0·1 (-0·9–1·0)	0·2 (-1·0–1·4)	0·1 (-1·4–1·8)
Drug use	1·1 (0·7–1·8)	1·5 (1·2–1·8)	2·9 (2·2–3·6)	7·1 (4·8–9·2)	6·0 (3·3–8·1)	7·5 (5·2–9·0)	8·3 (6·4–9·5)
Dietary risks	34·8 (26·1–46·0)	37·4 (28·0–49·2)	40·0 (30·8–52·3)	40·6 (31·2–52·7)	0·5 (0·3–0·8)	0·4 (0·1–0·7)	0·1 (-0·4–0·6)
Diet low in fruits	33·7 (29·0–37·1)	30·9 (26·3–33·9)	31·6 (27·4–34·4)	33·4 (28·2–37·6)	0·0 (-0·4–0·4)	0·4 (-0·2–0·9)	0·5 (-0·4–1·4)
Diet low in vegetables	24·6 (14·4–32·2)	25·2 (14·8–32·5)	28·1 (16·3–35·6)	28·2 (16·8–36·5)	0·4 (0·0–1·0)	0·5 (0·0–1·2)	0·0 (-1·0–1·0)
Diet low in legumes	37·4 (0·0–46·9)	37·3 (0·0–47·0)	40·8 (0·0–51·1)	41·6 (0·0–52·3)	0·3 (0·0–0·7)	0·5 (0·0–0·9)	0·2 (-0·5–0·8)
Diet low in whole grains	44·6 (37·2–50·8)	47·3 (39·0–53·8)	49·6 (41·4–56·3)	50·7 (41·9–57·7)	0·4 (0·1–0·7)	0·3 (-0·1–0·7)	0·2 (-0·4–0·7)
Diet low in nuts and seeds	11·3 (8·0–14·9)	6·2 (4·0–9·1)	4·1 (2·3–6·3)	4·5 (2·3–7·5)	-2·9 (-5·0–-1·3)	-1·5 (-4·6–1·1)	1·0 (-3·5–5·2)
Diet low in milk	34·8 (30·1–39·7)	31·8 (27·9–36·2)	29·7 (25·4–34·3)	29·6 (25·1–34·6)	-0·5 (-1·0–0·0)	-0·3 (-1·0–0·3)	0·0 (-1·0–0·9)
Diet high in red meat	45·3 (0·0–63·1)	46·9 (0·0–65·5)	48·6 (0·0–66·7)	49·3 (0·0–68·2)	0·3 (-0·2–1·1)	0·2 (-0·4–1·2)	0·1 (-0·8–1·4)
Diet high in processed meat	40·3 (32·4–45·7)	50·1 (41·1–56·4)	57·7 (47·0–65·1)	56·8 (47·1–64·3)	1·1 (0·7–1·5)	0·6 (0·0–1·1)	-0·1 (-0·9–0·7)
Diet high in sugar-sweetened beverages	37·4 (28·6–46·2)	51·7 (40·2–60·5)	59·1 (47·0–68·0)	59·4 (46·8–69·1)	1·5 (0·7–2·3)	0·7 (-0·3–1·6)	0·0 (-1·3–1·2)
Diet low in fibre	37·3 (19·8–44·4)	27·6 (14·5–34·5)	23·4 (13·0–30·4)	20·6 (12·1–26·9)	-1·9 (-2·8–-1·0)	-1·4 (-2·8–-0·1)	-1·2 (-3·3–0·9)
Diet low in calcium	5·3 (4·3–6·5)	4·5 (3·6–5·5)	4·6 (3·7–5·7)	4·6 (3·7–5·6)	-0·5 (-0·7–-0·3)	0·1 (-0·2–0·3)	-0·1 (-0·4–0·3)
Diet low in seafood omega-3 fatty acids	33·8 (25·1–43·4)	40·9 (31·8–51·8)	42·8 (32·9–53·8)	37·8 (28·6–48·2)	0·4 (-0·1–0·8)	-0·4 (-0·9–0·2)	-1·1 (-1·9–-0·4)
Diet low in omega-6 polyunsaturated fatty acids	33·6 (21·6–42·8)	29·0 (19·6–37·5)	21·0 (14·8–27·3)	19·7 (13·5–26·2)	-1·7 (-2·5–-0·9)	-1·8 (-2·8–-1·0)	-0·6 (-1·6–0·4)
Diet high in trans fatty acids	71·3 (64·0–78·7)	71·8 (63·6–78·9)	63·3 (55·6–71·1)	0·0 (0·0–0·0)	-65·8 (-66·1–-65·4)	-97·1 (-97·6–-96·5)	-184·2 (-185·3–-183·1)
Diet high in sodium	23·1 (0·5–67·4)	27·6 (1·9–73·5)	30·7 (3·5–75·4)	31·2 (3·0–75·9)	1·0 (0·2–6·5)	0·6 (-0·1–3·7)	0·1 (-2·5–1·7)
Intimate partner violence	31·3 (18·8–39·4)	31·2 (20·9–38·9)	28·4 (19·8–35·4)	26·8 (15·1–36·0)	-0·5 (-1·7–0·8)	-0·7 (-2·4–0·9)	-0·5 (-2·7–1·3)
Childhood sexual abuse and bullying	8·1 (5·0–13·3)	9·9 (6·2–16·0)	11·3 (7·1–18·3)	8·9 (5·8–14·3)	0·3 (-0·3–0·9)	-0·5 (-1·3–0·3)	-2·2 (-3·7–-0·7)
Childhood sexual abuse	6·4 (5·4–7·7)	7·3 (6·2–8·5)	8·4 (7·4–9·8)	7·8 (6·5–9·4)	0·6 (-0·2–1·5)	0·3 (-0·8–1·4)	-0·7 (-2·2–0·7)
Bullying victimization	8·0 (3·5–16·0)	10·0 (4·6–19·4)	11·4 (5·1–22·1)	8·2 (3·6–15·9)	0·1 (-0·5–0·8)	-1·0 (-1·9–0·0)	-3·0 (-4·8–-1·3)
Unsafe sex	--	--	--	--	--	--	--
Low physical activity	18·2 (13·5–24·1)	16·9 (12·5–21·4)	18·3 (14·1–23·4)	21·6 (15·6–28·1)	0·5 (-0·4–1·5)	1·2 (0·0–2·5)	1·5 (-0·4–3·3)
**Metabolic risks**	**24·8 (21·7–28·1)**	**29·4 (25·8–32·4)**	**32·3 (28·5–35·1)**	**36·4 (31·7–39·6)**	**1·2 (0·9–1·6)**	**1·0 (0·6–1·4)**	**1·1 (0·6–1·5)**
High fasting plasma glucose	12·6 (9·6–15·4)	15·9 (12·3–19·2)	20·8 (15·7–25·0)	25·2 (18·7–30·8)	2·2 (1·6–2·9)	2·2 (1·3–3·1)	1·7 (0·4–3·1)
High LDL cholesterol	64·0 (44·9–86·9)	54·1 (37·2–74·3)	48·4 (32·8–67·5)	47·0 (32·0–65·7)	-1·0 (-1·2–-0·8)	-0·7 (-0·9–-0·5)	-0·3 (-0·5–0·0)
High systolic blood pressure	28·0 (18·6–39·4)	23·6 (16·4–32·7)	17·7 (11·4–25·6)	24·9 (15·7–35·7)	-0·4 (-1·3–0·5)	0·3 (-1·0–1·5)	3·1 (1·0–5·5)
High body-mass index	27·9 (23·2–32·3)	33·6 (28·4–37·6)	37·1 (31·5–41·0)	41·0 (34·8–45·0)	1·2 (0·9–1·6)	1·0 (0·6–1·3)	0·9 (0·4–1·4)
Low bone mineral density	21·6 (15·9–29·0)	19·6 (13·7–26·6)	20·1 (14·3–27·3)	21·9 (15·8–29·2)	0·0 (-0·4–0·5)	0·5 (-0·2–1·4)	0·8 (-0·7–2·1)
Kidney dysfunction	2·8 (2·3–3·7)	2·9 (2·3–3·8)	2·8 (2·3–3·8)	2·9 (2·4–3·9)	0·1 (0·0–0·2)	0·1 (-0·1–0·2)	0·3 (0·1–0·7)

New Jersey

Risk Names	SEV 1990	SEV 2000	SEV 2010	SEV 2021	Annualised rate of change 1990 to 2021	Annualised rate of change 2000 to 2021	Annualised rate of change 2010 to 2021
**All risk factors**	**27·7 (24·8–31·1)**	**27·5 (24·8–30·9)**	**28·5 (25·6–31·9)**	**29·0 (25·8–32·3)**	**0·1 (-0·2–0·5)**	**0·2 (-0·2–0·6)**	**0·1 (-0·4–0·7)**
**Environmental/occupational risks**	**19·7 (12·9–30·1)**	**19·5 (13·3–30·1)**	**16·3 (10·9–26·4)**	**14·2 (9·2–23·3)**	**-1·0 (-1·8–-0·4)**	**-1·5 (-2·0–-1·0)**	**-1·2 (-1·7–-0·6)**
Unsafe water, sanitation, and handwashing	1·5 (0·7–2·6)	1·4 (0·6–2·3)	1·2 (0·3–2·0)	1·0 (0·3–1·7)	-1·4 (-3·5–0·7)	-1·7 (-4·4–1·2)	-1·5 (-5·6–2·5)
Unsafe water source	2·1 (0·7–4·2)	2·0 (0·7–3·9)	0·8 (0·4–1·6)	0·7 (0·3–1·4)	-3·6 (-6·2–-0·5)	-4·9 (-8·3–-1·3)	-1·6 (-6·5–3·3)
Unsafe sanitation	4·3 (2·4–7·3)	3·4 (1·7–5·9)	2·6 (1·3–4·7)	2·1 (1·1–3·6)	-2·3 (-4·5–-0·1)	-2·2 (-5·1–0·5)	-2·0 (-5·4–1·3)
No access to handwashing facility	1·5 (0·4–2·8)	1·5 (0·4–2·9)	1·6 (0·5–2·9)	1·4 (0·4–2·6)	-0·3 (-2·9–3·1)	-0·5 (-4·3–3·6)	-1·3 (-7·0–4·0)
Air pollution	28·1 (9·2–39·0)	26·4 (9·8–32·1)	17·0 (5·4–26·3)	9·6 (2·8–19·3)	-3·5 (-6·3–-1·4)	-4·8 (-8·8–-2·1)	-5·2 (-8·5–-2·4)
Particulate matter pollution	14·9 (5·7–25·2)	13·0 (9·5–17·1)	7·7 (4·7–11·3)	4·8 (2·2–7·8)	-3·6 (-6·3–-1·2)	-4·8 (-7·3–-3·4)	-4·3 (-7·8–-2·5)
Ambient particulate matter pollution	23·2 (9·0–39·4)	20·2 (15·1–25·6)	11·9 (7·5–16·8)	7·3 (3·3–11·7)	-3·7 (-6·4–-1·2)	-4·8 (-7·4–-3·5)	-4·4 (-7·9–-2·5)
Household air pollution from solid fuels	0·0 (0·0–0·0)	0·0 (0·0–0·0)	0·0 (0·0–0·0)	0·0 (0·0–0·0)	-3·4 (-39·1–18·3)	-3·9 (-35·0–50·6)	-3·1 (-26·1–0·0)
Ambient ozone pollution	24·2 (17·9–32·0)	21·8 (15·7–28·8)	18·4 (12·6–25·4)	13·4 (7·7–20·4)	-1·9 (-2·9–-1·1)	-2·3 (-3·7–-1·2)	-2·9 (-5·4–-1·0)
Ambient nitrogen dioxide pollution	84·4 (0·0–100·0)	84·2 (0·0–100·0)	57·0 (0·0–100·0)	29·7 (0·0–80·7)	-3·4 (-9·0–0·0)	-5·0 (-13·2–0·0)	-5·9 (-13·5–0·0)
Non-optimal temperature	28·5 (22·1–38·0)	28·9 (23·6–37·3)	34·2 (26·6–45·1)	30·6 (24·1–40·8)	0·2 (0·0–0·5)	0·3 (-0·2–0·7)	-1·0 (-1·5–-0·6)
High temperature	23·2 (12·7–35·3)	21·6 (12·4–32·8)	39·2 (26·5–51·4)	30·6 (19·5–41·9)	0·9 (0·4–1·7)	1·7 (0·9–2·7)	-2·2 (-3·3–-1·3)
Low temperature	26·3 (22·8–30·3)	27·6 (24·3–31·2)	28·7 (25·6–32·5)	27·0 (23·9–31·0)	0·1 (0·0–0·2)	-0·1 (-0·3–0·1)	-0·6 (-0·8–-0·4)
Other environmental risks	30·5 (8·6–48·5)	28·3 (8·5–46·8)	25·0 (8·5–43·4)	21·8 (8·5–39·6)	-1·1 (-1·8–0·0)	-1·2 (-2·1–0·0)	-1·3 (-2·3–0·0)
Residential radon	28·3 (0·0–83·6)	28·3 (0·0–83·6)	28·3 (0·0–83·6)	28·3 (0·0–83·6)	0·0 (0·0–0·0)	0·0 (0·0–0·0)	0·0 (0·0–0·0)
Lead exposure	31·4 (0·0–38·9)	28·2 (0·0–35·0)	23·4 (0·0–28·9)	18·8 (0·0–23·4)	-1·7 (-2·1–0·0)	-1·9 (-2·4–0·0)	-2·0 (-2·8–0·0)
Occupational risks	2·9 (2·4–3·8)	3·0 (2·4–3·9)	2·8 (2·3–3·7)	2·8 (2·2–3·6)	-0·1 (-0·4–0·1)	-0·3 (-0·6–0·0)	0·0 (-0·5–0·4)
Occupational carcinogens	1·0 (0·5–1·9)	1·1 (0·5–2·1)	1·1 (0·5–2·0)	1·0 (0·5–2·0)	0·0 (-0·4–0·3)	-0·3 (-0·8–0·1)	-0·1 (-0·8–0·4)
Occupational exposure to asbestos	5·7 (5·2–6·3)	6·2 (5·7–6·6)	5·1 (4·7–5·5)	4·3 (3·5–5·3)	-0·9 (-1·7–-0·2)	-1·7 (-2·8–-0·7)	-1·6 (-3·5–0·1)
Occupational exposure to arsenic	0·5 (0·0–1·2)	0·5 (0·0–1·2)	0·5 (0·0–1·2)	0·5 (0·0–1·2)	-0·3 (-0·7–0·2)	-0·3 (-0·9–0·4)	0·1 (-0·8–1·0)
Occupational exposure to benzene	1·1 (0·1–3·2)	1·2 (0·1–3·6)	1·2 (0·1–3·5)	1·2 (0·1–3·6)	0·3 (0·0–0·6)	0·0 (-0·4–0·4)	0·2 (-0·5–0·7)
Occupational exposure to beryllium	0·0 (0·0–0·0)	0·0 (0·0–0·0)	0·0 (0·0–0·0)	0·0 (0·0–0·0)	0·2 (0·0–0·5)	0·1 (-0·2–0·4)	0·4 (0·0–0·8)
Occupational exposure to cadmium	0·1 (0·1–0·1)	0·1 (0·1–0·1)	0·1 (0·1–0·1)	0·1 (0·1–0·1)	-0·2 (-0·6–0·2)	-0·2 (-0·7–0·3)	0·2 (-0·6–0·9)
Occupational exposure to chromium	0·2 (0·1–0·2)	0·2 (0·1–0·2)	0·1 (0·1–0·2)	0·1 (0·1–0·2)	-0·1 (-0·4–0·2)	-0·1 (-0·6–0·3)	0·2 (-0·4–0·8)
Occupational exposure to diesel engine exhaust	0·7 (0·7–0·8)	0·8 (0·7–0·8)	0·7 (0·7–0·8)	0·8 (0·7–0·9)	0·4 (0·0–0·7)	0·3 (-0·2–0·8)	0·6 (-0·1–1·3)
Occupational exposure to formaldehyde	0·3 (0·3–0·3)	0·3 (0·3–0·3)	0·3 (0·3–0·3)	0·3 (0·3–0·3)	-0·3 (-0·6–0·1)	-0·3 (-0·8–0·2)	0·0 (-0·7–0·7)
Occupational exposure to nickel	0·4 (0·0–1·6)	0·4 (0·0–1·5)	0·4 (0·0–1·4)	0·4 (0·0–1·5)	-0·3 (-0·9–0·2)	-0·2 (-1·0–0·5)	0·2 (-1·0–1·1)
Occupational exposure to polycyclic aromatic hydrocarbons	0·3 (0·3–0·3)	0·3 (0·3–0·3)	0·3 (0·3–0·3)	0·3 (0·3–0·3)	0·0 (-0·4–0·3)	-0·1 (-0·6–0·3)	0·2 (-0·4–0·8)
Occupational exposure to silica	3·5 (0·6–11·2)	3·3 (0·5–10·7)	3·1 (0·5–10·0)	3·2 (0·5–9·8)	-0·3 (-0·9–0·1)	-0·2 (-0·9–0·5)	0·2 (-0·7–1·2)
Occupational exposure to sulphuric acid	0·8 (0·2–2·5)	0·8 (0·2–2·6)	0·7 (0·1–2·3)	0·7 (0·2–2·4)	-0·2 (-0·5–0·2)	-0·2 (-0·8–0·4)	0·2 (-0·5–0·9)
Occupational exposure to trichloroethylene	0·1 (0·1–0·1)	0·1 (0·1–0·1)	0·1 (0·1–0·1)	0·1 (0·1–0·1)	0·1 (-0·2–0·3)	0·0 (-0·4–0·4)	0·4 (-0·1–0·9)
Occupational asthmagens	17·1 (14·9–19·6)	17·4 (15·4–19·6)	16·3 (14·3–18·5)	16·7 (14·6–18·9)	-0·1 (-0·4–0·2)	-0·2 (-0·6–0·2)	0·2 (-0·4–0·8)
Occupational particulate matter, gases, and fumes	6·5 (4·9–8·6)	6·2 (4·7–8·0)	5·8 (4·4–7·4)	5·4 (4·1–6·9)	-0·6 (-0·9–-0·4)	-0·7 (-1·0–-0·4)	-0·7 (-1·1–-0·3)
Occupational noise	6·5 (6·0–7·2)	6·5 (6·1–7·2)	6·4 (6·0–7·0)	6·2 (5·9–6·8)	-0·1 (-0·3–0·0)	-0·2 (-0·4–0·0)	-0·3 (-0·5–-0·1)
Occupational injuries	--	--	--	--	--	--	--
Occupational ergonomic factors	7·6 (6·4–9·2)	7·7 (6·6–9·3)	7·1 (6·0–8·6)	7·3 (6·0–8·9)	-0·1 (-0·5–0·2)	-0·2 (-0·8–0·2)	0·3 (-0·5–1·0)
**Behavioural risks**	**30·6 (27·6–34·5)**	**28·7 (25·8–32·3)**	**26·5 (23·7–30·2)**	**24·9 (21·7–28·8)**	**-0·7 (-1·1–-0·3)**	**-0·7 (-1·1–-0·3)**	**-0·6 (-1·3–0·0)**
Child and maternal malnutrition	10·2 (6·8–14·4)	8·7 (5·8–11·8)	8·7 (6·0–12·0)	8·9 (6·1–12·7)	-0·4 (-1·4–0·5)	0·1 (-1·1–1·3)	0·2 (-1·8–1·8)
Suboptimal breastfeeding	68·7 (66·3–71·2)	66·5 (63·8–69·0)	62·6 (60·0–65·5)	62·0 (59·1–65·3)	-0·3 (-0·5–-0·2)	-0·3 (-0·5–-0·1)	-0·1 (-0·4–0·2)
Non-exclusive breastfeeding	63·7 (55·9–71·6)	59·0 (52·1–66·1)	48·0 (41·4–55·0)	45·9 (38·8–53·4)	-1·1 (-1·6–-0·5)	-1·2 (-1·9–-0·5)	-0·4 (-1·3–0·7)
Discontinued breastfeeding	88·7 (86·6–91·1)	86·2 (83·5–89·3)	84·3 (81·1–87·6)	84·5 (81·3–87·6)	-0·2 (-0·3–0·0)	-0·1 (-0·3–0·1)	0·0 (-0·3–0·3)
Child growth failure	1·2 (0·4–2·7)	1·1 (0·4–2·4)	0·9 (0·3–2·1)	0·9 (0·3–1·9)	-1·0 (-1·3–-0·6)	-1·0 (-1·6–-0·5)	-0·5 (-1·3–0·3)
Child underweight	2·4 (1·5–3·3)	2·3 (1·5–3·1)	1·9 (1·2–2·6)	1·8 (1·2–2·4)	-1·0 (-1·5–-0·5)	-1·1 (-1·8–-0·4)	-0·5 (-1·4–0·6)
Child wasting	0·8 (0·5–1·2)	0·8 (0·5–1·2)	0·8 (0·5–1·2)	0·7 (0·5–1·1)	-0·3 (-0·6–0·0)	-0·1 (-0·5–0·4)	-0·2 (-0·9–0·6)
Child stunting	3·5 (2·7–4·3)	3·2 (2·5–4·0)	2·7 (2·1–3·4)	2·5 (1·9–3·1)	-1·1 (-1·6–-0·5)	-1·2 (-2·0–-0·4)	-0·8 (-2·0–0·4)
Low birth weight and short gestation	18·6 (16·4–21·0)	20·0 (17·5–22·6)	21·5 (18·7–24·3)	20·3 (17·5–23·1)	0·3 (0·0–0·6)	0·1 (-0·4–0·5)	-0·5 (-1·4–0·3)
Short gestation	32·9 (29·0–37·2)	34·2 (29·9–38·4)	34·8 (30·5–39·1)	33·4 (29·0–38·0)	0·0 (-0·2–0·4)	-0·1 (-0·7–0·4)	-0·4 (-1·3–0·5)
Low birth weight	13·9 (12·6–15·4)	14·9 (13·6–16·3)	16·5 (14·9–18·1)	15·6 (14·0–17·2)	0·4 (0·1–0·7)	0·2 (-0·2–0·7)	-0·5 (-1·3–0·3)

Iron deficiency	3·6 (2·5–5·0)	3·1 (2·2–4·1)	3·1 (2·2–4·2)	3·2 (2·2–4·4)	-0·4 (-1·4–0·4)	0·1 (-1·0–1·3)	0·1 (-1·7–1·7)
Vitamin A deficiency	0·7 (0·0–1·4)	0·9 (0·0–1·8)	0·5 (0·0–1·1)	0·3 (0·0–0·7)	-2·2 (-4·4–0·0)	-4·5 (-7·4–0·0)	-4·2 (-8·3–0·0)
Zinc deficiency	1·5 (0·0–6·5)	0·9 (0·0–3·6)	0·8 (0·0–3·5)	0·8 (0·0–3·4)	-1·9 (-37·3–3·0)	-0·5 (-3·7–1·8)	0·2 (-8·8–6·8)
Tobacco	39·4 (37·7–41·1)	33·1 (31·9–34·3)	25·2 (24·2–26·1)	21·8 (20·3–23·3)	-1·9 (-2·1–-1·7)	-2·0 (-2·3–-1·7)	-1·3 (-1·9–-0·7)
Smoking	35·3 (32·4–38·0)	29·7 (27·8–31·7)	22·8 (21·4–24·2)	19·6 (17·8–21·5)	-1·9 (-2·3–-1·5)	-2·0 (-2·4–-1·5)	-1·4 (-2·1–-0·6)
Chewing tobacco	0·8 (0·6–1·0)	0·9 (0·7–1·1)	1·0 (0·7–1·3)	1·0 (0·7–1·4)	0·7 (-0·6–2·1)	0·7 (-1·0–2·3)	0·4 (-2·0–2·8)
Second-hand smoke	33·6 (31·6–34·9)	28·1 (26·4–29·3)	21·3 (19·7–22·3)	18·7 (17·0–20·2)	-1·9 (-2·2–-1·7)	-1·9 (-2·3–-1·6)	-1·2 (-1·9–-0·5)
High alcohol use	31·2 (23·7–42·1)	30·4 (22·5–41·0)	31·5 (23·9–41·8)	31·5 (23·9–42·2)	0·0 (-0·9–0·9)	0·2 (-0·9–1·2)	0·0 (-1·6–1·4)
Drug use	1·0 (0·6–1·7)	1·2 (0·9–1·5)	1·5 (1·3–1·8)	4·6 (3·3–5·8)	5·0 (2·4–7·1)	6·6 (4·2–8·1)	10·2 (6·8–12·2)
Dietary risks	34·4 (25·5–45·1)	37·0 (28·2–48·5)	39·6 (30·4–51·8)	39·8 (30·3–51·9)	0·5 (0·2–0·7)	0·3 (0·0–0·6)	0·0 (-0·4–0·5)
Diet low in fruits	34·0 (29·5–37·4)	31·6 (27·0–34·5)	31·9 (27·9–34·6)	33·3 (27·9–37·2)	-0·1 (-0·4–0·3)	0·2 (-0·3–0·8)	0·4 (-0·4–1·2)
Diet low in vegetables	23·4 (14·4–31·6)	23·4 (14·2–31·4)	27·3 (15·8–34·7)	27·5 (16·4–36·0)	0·5 (0·0–1·0)	0·8 (0·1–1·4)	0·1 (-1·0–0·9)
Diet low in legumes	36·1 (0·0–45·6)	36·1 (0·0–45·3)	40·0 (0·0–50·2)	40·8 (0·0–51·8)	0·4 (0·0–0·7)	0·6 (0·0–1·1)	0·2 (-0·6–0·9)
Diet low in whole grains	44·1 (37·1–50·6)	47·1 (39·2–53·8)	48·8 (41·0–55·8)	49·1 (41·0–56·6)	0·3 (0·1–0·6)	0·2 (-0·2–0·6)	0·1 (-0·5–0·7)
Diet low in nuts and seeds	9·6 (6·6–13·0)	5·1 (3·0–7·5)	3·5 (2·0–5·5)	4·0 (2·1–6·4)	-2·8 (-4·9–-0·9)	-1·1 (-4·2–1·6)	1·2 (-2·9–5·3)
Diet low in milk	33·1 (28·8–37·7)	30·2 (26·1–34·7)	28·7 (24·9–32·9)	28·8 (24·3–33·6)	-0·4 (-1·0–0·0)	-0·2 (-0·9–0·4)	0·0 (-1·0–0·9)
Diet high in red meat	43·9 (0·0–61·5)	45·7 (0·0–63·7)	47·1 (0·0–65·2)	47·3 (0·0–65·8)	0·2 (-0·3–1·0)	0·2 (-0·5–1·0)	0·1 (-0·9–1·2)
Diet high in processed meat	38·8 (30·9–43·3)	48·4 (39·7–54·1)	54·7 (44·0–61·8)	53·0 (43·1–60·2)	1·0 (0·6–1·4)	0·4 (-0·1–1·0)	-0·3 (-1·1–0·5)
Diet high in sugar-sweetened beverages	39·7 (30·2–49·1)	54·5 (42·6–63·6)	60·2 (47·3–69·2)	59·1 (47·7–69·4)	1·3 (0·5–2·1)	0·4 (-0·5–1·3)	-0·2 (-1·5–1·0)
Diet low in fibre	34·4 (18·8–41·7)	25·0 (14·9–31·2)	21·8 (13·0–27·3)	19·2 (11·3–25·1)	-1·9 (-2·9–-1·0)	-1·3 (-2·7–0·1)	-1·2 (-3·4–0·9)
Diet low in calcium	5·0 (4·0–6·1)	4·3 (3·5–5·3)	4·5 (3·6–5·5)	4·5 (3·6–5·5)	-0·3 (-0·5–-0·1)	0·1 (-0·1–0·4)	-0·1 (-0·3–0·2)
Diet low in seafood omega-3 fatty acids	31·1 (22·0–40·3)	38·0 (28·8–48·4)	40·9 (30·9–50·9)	36·0 (26·3–45·7)	0·5 (0·0–1·0)	-0·3 (-0·9–0·4)	-1·2 (-2·0–-0·3)
Diet low in omega-6 polyunsaturated fatty acids	30·7 (20·5–39·9)	26·4 (18·2–34·5)	19·8 (14·0–25·3)	18·6 (12·8–24·4)	-1·6 (-2·3–-0·8)	-1·7 (-2·5–-0·8)	-0·5 (-1·5–0·3)
Diet high in trans fatty acids	71·5 (64·5–78·5)	71·8 (63·8–78·8)	63·2 (54·9–71·1)	0·0 (0·0–0·0)	-65·8 (-66·1–-65·4)	-97·1 (-97·5–-96·5)	-184·2 (-185·3–-182·9)
Diet high in sodium	23·5 (0·6–70·4)	28·2 (1·9–77·5)	31·1 (3·2–77·3)	31·6 (3·0–78·6)	1·0 (0·2–6·2)	0·5 (-0·1–4·1)	0·1 (-2·4–1·8)
Intimate partner violence	23·2 (12·0–30·9)	22·8 (14·3–28·9)	18·3 (11·4–23·3)	18·0 (8·9–25·2)	-0·8 (-2·5–0·7)	-1·1 (-3·4–0·7)	-0·1 (-3·1–2·2)
Childhood sexual abuse and bullying	8·4 (5·3–13·7)	10·3 (6·6–16·4)	11·7 (7·4–18·8)	9·0 (6·0–14·2)	0·2 (-0·4–0·8)	-0·6 (-1·4–0·3)	-2·3 (-4·0–-0·7)
Childhood sexual abuse	7·1 (5·9–8·5)	8·1 (7·0–9·4)	9·1 (8·0–10·4)	8·3 (6·7–10·1)	0·5 (-0·3–1·3)	0·1 (-0·9–1·1)	-0·8 (-2·3–0·8)
Bullying victimization	8·1 (3·6–16·2)	10·0 (4·6–19·5)	11·4 (5·1–22·3)	8·0 (3·5–15·6)	0·0 (-0·7–0·8)	-1·0 (-2·1–0·0)	-3·2 (-5·1–-1·3)
Unsafe sex	--	--	--	--	--	--	--
Low physical activity	20·4 (15·1–26·0)	19·8 (15·1–25·3)	19·3 (15·2–23·8)	20·8 (15·6–27·0)	0·1 (-0·9–0·9)	0·2 (-1·1–1·4)	0·7 (-1·1–2·4)
**Metabolic risks**	**24·8 (21·3–28·1)**	**29·2 (25·6–31·1)**	**32·6 (28·7–35·5)**	**35·7 (31·3–38·7)**	**1·2 (0·9–1·5)**	**1·0 (0·6–1·3)**	**0·8 (0·4–1·3)**
High fasting plasma glucose	12·3 (9·5–15·1)	15·3 (11·9–18·4)	19·5 (14·4–23·3)	23·3 (17·6–28·0)	2·1 (1·4–2·7)	2·0 (1·2–2·9)	1·6 (0·5–2·8)
High LDL cholesterol	63·8 (44·9–87·0)	53·9 (37·5–74·7)	48·3 (32·8–67·2)	46·5 (31·2–65·4)	-1·0 (-1·2–-0·8)	-0·7 (-0·9–-0·5)	-0·3 (-0·6–-0·1)
High systolic blood pressure	29·8 (20·2–40·1)	25·2 (17·6–34·1)	18·3 (12·0–26·1)	25·7 (15·7–37·7)	-0·5 (-1·5–0·4)	0·1 (-1·2–1·1)	3·1 (0·6–5·2)
High body-mass index	28·1 (23·4–32·7)	33·7 (28·6–37·8)	37·5 (32·0–41·5)	40·3 (33·8–44·3)	1·2 (0·8–1·5)	0·9 (0·5–1·1)	0·6 (0·2–1·0)
Low bone mineral density	21·2 (15·3–28·0)	19·2 (14·0–26·1)	20·0 (14·3–26·7)	21·8 (15·9–29·0)	0·1 (-0·4–0·6)	0·6 (-0·1–1·4)	0·8 (-0·5–2·1)
Kidney dysfunction	2·9 (2·4–3·8)	3·0 (2·4–4·0)	2·9 (2·4–3·9)	3·0 (2·5–4·0)	0·1 (0·0–0·2)	0·1 (-0·1–0·2)	0·3 (0·0–0·6)

New Mexico

Risk Names	SEV 1990	SEV 2000	SEV 2010	SEV 2021	Annualised rate of change 1990 to 2021	Annualised rate of change 2000 to 2021	Annualised rate of change 2010 to 2021
**All risk factors**	**24·6 (21·9–27·9)**	**25·2 (22·5–28·4)**	**25·4 (22·4–28·6)**	**25·0 (21·9–28·3)**	**0·1 (-0·2–0·3)**	**0·0 (-0·4–0·4)**	**-0·1 (-0·6–0·4)**
**Environmental/occupational risks**	**16·1 (10·7–26·6)**	**15·7 (10·2–24·4)**	**13·9 (8·6–22·6)**	**12·5 (7·6–20·3)**	**-0·8 (-1·4–-0·4)**	**-1·1 (-1·5–-0·6)**	**-1·0 (-1·4–-0·5)**
Unsafe water, sanitation, and handwashing	2·6 (1·4–4·4)	2·4 (1·2–3·9)	1·8 (0·6–3·0)	1·4 (0·5–2·4)	-2·1 (-4·4–-0·1)	-2·5 (-5·9–0·1)	-2·1 (-6·3–1·4)
Unsafe water source	3·5 (1·2–6·6)	3·2 (1·0–6·3)	1·2 (0·5–2·4)	0·9 (0·4–1·8)	-4·2 (-6·8–-1·5)	-5·7 (-9·3–-1·8)	-2·4 (-7·4–3·8)
Unsafe sanitation	9·1 (4·8–14·9)	7·0 (3·6–11·6)	5·0 (2·7–8·6)	3·5 (1·8–5·9)	-3·0 (-5·3–-1·1)	-3·3 (-6·0–-0·6)	-3·2 (-6·5–0·1)
No access to handwashing facility	2·1 (0·8–4·0)	2·2 (0·7–4·2)	2·1 (0·7–4·1)	1·8 (0·5–3·5)	-0·6 (-3·7–2·0)	-0·9 (-5·1–2·9)	-1·6 (-7·5–3·6)
Air pollution	15·9 (2·7–26·3)	16·2 (4·1–24·8)	8·9 (1·5–19·1)	6·1 (0·8–15·0)	-3·1 (-6·8–-1·3)	-4·7 (-9·3–-2·2)	-3·5 (-8·0–-1·9)
Particulate matter pollution	6·0 (1·1–13·0)	5·8 (3·1–9·1)	2·8 (1·0–5·5)	1·4 (0·3–3·5)	-4·6 (-9·6–-0·9)	-6·6 (-11·9–-4·2)	-6·1 (-12·4–-3·6)
Ambient particulate matter pollution	9·1 (1·7–19·6)	8·8 (4·9–13·3)	4·2 (1·5–8·1)	2·2 (0·4–5·2)	-4·6 (-9·6–-0·9)	-6·7 (-11·9–-4·2)	-6·1 (-12·4–-3·6)
Household air pollution from solid fuels	0·1 (0·0–0·7)	0·1 (0·0–0·5)	0·0 (0·0–0·2)	0·0 (0·0–0·1)	-4·9 (-25·9–-5·9)	-5·5 (-26·1–-6·3)	-4·3 (-18·4–-3·2)
Ambient ozone pollution	23·0 (17·0–30·3)	28·9 (22·6–36·5)	25·1 (19·0–32·4)	24·8 (18·6–32·3)	0·2 (-0·3–0·7)	-0·7 (-1·4–-0·1)	-0·1 (-1·5–1·1)
Ambient nitrogen dioxide pollution	58·7 (0·0–100·0)	61·4 (0·0–100·0)	34·7 (0·0–89·7)	25·3 (0·0–74·5)	-2·7 (-8·7–0·0)	-4·2 (-13·4–0·0)	-2·9 (-12·9–0·0)
Non-optimal temperature	31·6 (25·4–40·9)	33·1 (25·9–43·6)	32·7 (26·1–43·1)	31·1 (24·5–41·4)	0·0 (-0·3–0·2)	-0·3 (-0·6–-0·1)	-0·5 (-0·9–-0·2)
High temperature	30·5 (21·5–41·1)	26·1 (17·4–37·0)	29·8 (21·2–40·0)	30·5 (21·7–41·0)	0·0 (-0·5–0·3)	0·7 (0·2–1·7)	0·2 (-0·4–0·8)
Low temperature	27·5 (24·4–31·1)	29·7 (26·0–33·4)	28·6 (25·4–32·4)	26·7 (23·6–30·6)	-0·1 (-0·2–0·0)	-0·5 (-0·7–-0·3)	-0·6 (-0·9–-0·4)
Other environmental risks	27·1 (8·0–44·8)	25·7 (8·0–43·7)	23·5 (8·0–42·0)	20·8 (8·0–38·6)	-0·9 (-1·5–0·0)	-1·0 (-1·9–0·0)	-1·1 (-2·2–0·0)
Residential radon	27·1 (0·0–83·2)	27·1 (0·0–83·2)	27·1 (0·0–83·2)	27·1 (0·0–83·2)	0·0 (0·0–0·0)	0·0 (0·0–0·0)	0·0 (0·0–0·0)
Lead exposure	27·0 (0·0–34·0)	25·0 (0·0–30·7)	21·7 (0·0–27·1)	17·9 (0·0–22·4)	-1·3 (-1·8–0·0)	-1·6 (-2·1–0·0)	-1·8 (-2·6–0·0)
Occupational risks	2·7 (2·2–3·6)	2·7 (2·2–3·6)	2·5 (2·0–3·3)	2·5 (2·0–3·4)	-0·2 (-0·5–0·1)	-0·3 (-0·7–0·1)	0·2 (-0·4–0·8)
Occupational carcinogens	0·9 (0·4–1·7)	0·9 (0·4–1·9)	0·9 (0·4–1·8)	0·9 (0·4–1·8)	0·2 (-0·2–0·5)	0·0 (-0·5–0·3)	0·3 (-0·3–0·9)
Occupational exposure to asbestos	3·5 (3·0–4·0)	3·6 (3·2–4·0)	3·2 (2·8–3·5)	3·2 (2·5–4·0)	-0·3 (-1·2–0·5)	-0·6 (-1·9–0·5)	0·0 (-2·2–2·1)
Occupational exposure to arsenic	0·4 (0·0–0·9)	0·4 (0·0–1·0)	0·4 (0·0–1·0)	0·4 (0·0–1·0)	0·0 (-0·4–0·5)	-0·1 (-0·8–0·5)	0·3 (-0·6–1·2)
Occupational exposure to benzene	1·0 (0·1–3·0)	1·1 (0·1–3·3)	1·1 (0·1–3·3)	1·2 (0·1–3·4)	0·3 (0·0–0·7)	0·1 (-0·4–0·5)	0·4 (-0·4–1·0)
Occupational exposure to beryllium	0·0 (0·0–0·0)	0·0 (0·0–0·0)	0·0 (0·0–0·0)	0·0 (0·0–0·0)	0·2 (-0·1–0·4)	0·1 (-0·3–0·4)	0·5 (0·0–1·0)
Occupational exposure to cadmium	0·0 (0·0–0·1)	0·1 (0·0–0·1)	0·0 (0·0–0·1)	0·0 (0·0–0·1)	0·1 (-0·3–0·5)	-0·1 (-0·7–0·5)	0·4 (-0·4–1·2)
Occupational exposure to chromium	0·1 (0·1–0·1)	0·1 (0·1–0·1)	0·1 (0·1–0·1)	0·1 (0·1–0·1)	0·1 (-0·2–0·5)	0·0 (-0·5–0·5)	0·4 (-0·3–1·1)
Occupational exposure to diesel engine exhaust	0·7 (0·6–0·7)	0·7 (0·6–0·7)	0·6 (0·6–0·7)	0·7 (0·6–0·8)	0·0 (-0·3–0·4)	0·1 (-0·4–0·6)	0·7 (0·0–1·4)
Occupational exposure to formaldehyde	0·2 (0·2–0·3)	0·3 (0·2–0·3)	0·2 (0·2–0·3)	0·2 (0·2–0·3)	0·1 (-0·3–0·5)	-0·1 (-0·7–0·4)	0·2 (-0·6–1·0)
Occupational exposure to nickel	0·4 (0·0–1·4)	0·3 (0·0–1·4)	0·3 (0·0–1·2)	0·3 (0·0–1·2)	-0·3 (-0·8–0·2)	-0·3 (-1·1–0·4)	0·2 (-0·8–1·2)
Occupational exposure to polycyclic aromatic hydrocarbons	0·3 (0·2–0·3)	0·3 (0·2–0·3)	0·3 (0·2–0·3)	0·3 (0·2–0·3)	0·2 (-0·2–0·5)	0·0 (-0·5–0·4)	0·4 (-0·3–1·0)
Occupational exposure to silica	3·5 (0·6–11·5)	3·2 (0·5–10·5)	2·9 (0·5–9·1)	3·0 (0·5–9·3)	-0·5 (-1·1–0·0)	-0·4 (-1·1–0·3)	0·2 (-0·7–1·2)
Occupational exposure to sulphuric acid	0·6 (0·1–2·0)	0·6 (0·1–2·1)	0·6 (0·1–2·0)	0·6 (0·1–2·0)	0·1 (-0·3–0·5)	-0·1 (-0·7–0·6)	0·4 (-0·5–1·2)
Occupational exposure to trichloroethylene	0·1 (0·1–0·1)	0·1 (0·1–0·1)	0·1 (0·1–0·1)	0·1 (0·1–0·1)	0·2 (-0·1–0·5)	0·1 (-0·4–0·5)	0·6 (-0·1–1·2)
Occupational asthmagens	16·0 (14·1–18·3)	16·0 (14·1–18·2)	14·5 (12·8–16·5)	15·1 (13·0–17·3)	-0·2 (-0·6–0·2)	-0·3 (-0·8–0·3)	0·3 (-0·4–1·2)
Occupational particulate matter, gases, and fumes	5·7 (4·4–7·3)	5·5 (4·3–7·2)	5·2 (4·0–6·7)	4·9 (3·7–6·3)	-0·5 (-0·8–-0·2)	-0·6 (-1·0–-0·3)	-0·7 (-1·1–-0·2)
Occupational noise	6·6 (6·0–7·2)	6·6 (6·1–7·1)	6·4 (6·0–7·0)	6·1 (5·8–6·7)	-0·2 (-0·4–0·0)	-0·3 (-0·5–-0·1)	-0·4 (-0·6–-0·2)
Occupational injuries	--	--	--	--	--	--	--
Occupational ergonomic factors	8·4 (7·1–10·0)	8·2 (7·0–10·0)	7·2 (6·1–8·7)	7·4 (6·1–9·2)	-0·4 (-0·9–0·0)	-0·5 (-1·1–0·1)	0·3 (-0·5–1·1)
**Behavioural risks**	**27·7 (24·7–31·4)**	**26·4 (23·2–30·1)**	**24·8 (21·9–28·4)**	**23·1 (20·0–26·9)**	**-0·6 (-1·0–-0·2)**	**-0·6 (-1·1–-0·2)**	**-0·7 (-1·4–0·0)**
Child and maternal malnutrition	10·5 (7·1–14·6)	9·0 (6·1–12·4)	9·1 (6·3–12·7)	9·3 (6·5–13·4)	-0·4 (-1·2–0·4)	0·2 (-1·0–1·3)	0·3 (-1·4–1·8)
Suboptimal breastfeeding	69·0 (66·4–71·8)	66·9 (64·6–69·6)	63·1 (60·1–66·0)	62·4 (59·7–65·5)	-0·3 (-0·5–-0·2)	-0·3 (-0·5–-0·1)	-0·1 (-0·4–0·2)
Non-exclusive breastfeeding	64·9 (56·7–72·6)	60·2 (53·0–68·1)	49·2 (42·7–56·8)	46·8 (39·8–54·6)	-1·1 (-1·6–-0·5)	-1·2 (-1·9–-0·5)	-0·5 (-1·4–0·5)
Discontinued breastfeeding	88·8 (86·6–91·2)	86·5 (84·1–89·3)	84·6 (81·6–87·7)	84·8 (81·4–88·0)	-0·1 (-0·3–0·0)	-0·1 (-0·3–0·1)	0·0 (-0·3–0·3)
Child growth failure	1·5 (0·5–3·4)	1·4 (0·4–3·0)	1·2 (0·4–2·6)	1·1 (0·3–2·5)	-1·0 (-1·4–-0·6)	-1·0 (-1·6–-0·5)	-0·5 (-1·3–0·2)
Child underweight	3·2 (2·0–4·4)	2·9 (1·8–3·9)	2·4 (1·6–3·4)	2·3 (1·5–3·2)	-1·0 (-1·5–-0·5)	-1·0 (-1·7–-0·4)	-0·4 (-1·5–0·6)
Child wasting	1·0 (0·6–1·5)	0·9 (0·6–1·4)	0·9 (0·6–1·4)	0·9 (0·6–1·3)	-0·3 (-0·7–0·0)	-0·1 (-0·6–0·4)	-0·2 (-0·8–0·6)
Child stunting	4·3 (3·3–5·5)	3·9 (3·0–4·9)	3·3 (2·6–4·2)	3·1 (2·4–3·9)	-1·1 (-1·6–-0·5)	-1·1 (-1·9–-0·4)	-0·7 (-1·8–0·4)
Low birth weight and short gestation	22·3 (19·4–25·1)	23·9 (21·1–27·4)	26·0 (22·8–29·3)	25·0 (21·7–28·4)	0·4 (0·1–0·6)	0·2 (-0·2–0·6)	-0·4 (-1·2–0·4)
Short gestation	38·1 (33·6–43·0)	42·3 (37·1–48·0)	43·0 (37·9–48·7)	40·1 (35·0–45·7)	0·2 (-0·1–0·5)	-0·3 (-0·7–0·2)	-0·6 (-1·5–0·3)
Low birth weight	17·9 (16·2–19·6)	18·5 (16·7–20·4)	20·7 (18·8–23·1)	20·4 (18·6–22·5)	0·4 (0·2–0·7)	0·5 (0·1–0·9)	-0·1 (-0·9–0·6)

Iron deficiency	3·8 (2·6–5·1)	3·2 (2·3–4·3)	3·3 (2·4–4·5)	3·3 (2·4–4·7)	-0·4 (-1·2–0·4)	0·2 (-0·9–1·2)	0·1 (-1·3–1·6)
Vitamin A deficiency	1·5 (0·0–3·1)	2·0 (0·0–4·3)	1·2 (0·0–2·8)	0·7 (0·0–1·6)	-2·3 (-4·5–0·0)	-4·9 (-7·8–0·0)	-4·9 (-8·8–0·0)
Zinc deficiency	2·1 (0·0–9·0)	1·3 (0·0–5·4)	1·1 (0·0–4·8)	1·2 (0·0–5·0)	-1·9 (-48·1–0·0)	-0·4 (-5·1–1·0)	0·3 (-2·4–6·7)
Tobacco	36·5 (34·6–37·9)	32·4 (31·1–33·7)	27·4 (26·4–28·3)	23·7 (22·0–25·1)	-1·4 (-1·6–-1·2)	-1·5 (-1·8–-1·2)	-1·3 (-1·9–-0·8)
Smoking	34·0 (31·6–36·5)	30·3 (28·5–32·2)	25·2 (24·0–26·7)	21·3 (19·4–23·3)	-1·5 (-1·8–-1·2)	-1·7 (-2·1–-1·3)	-1·6 (-2·3–-0·8)
Chewing tobacco	2·1 (1·6–2·7)	2·3 (1·7–2·9)	2·3 (1·7–3·0)	2·3 (1·7–3·1)	0·2 (-1·1–1·6)	0·0 (-1·7–1·8)	-0·1 (-2·7–2·6)
Second-hand smoke	29·8 (28·0–31·0)	26·2 (24·4–27·4)	22·3 (20·7–23·5)	19·8 (18·1–21·3)	-1·3 (-1·6–-1·1)	-1·3 (-1·7–-1·0)	-1·1 (-1·7–-0·5)
High alcohol use	23·9 (16·5–33·5)	23·0 (15·9–32·9)	23·7 (16·2–34·0)	24·0 (15·8–34·4)	0·0 (-1·2–1·1)	0·2 (-1·4–1·6)	0·1 (-1·9–2·1)
Drug use	1·2 (0·8–1·9)	1·2 (1·0–1·6)	1·9 (1·6–2·3)	6·2 (4·5–8·0)	5·3 (2·9–7·2)	7·7 (5·2–9·5)	10·6 (7·4–12·5)
Dietary risks	35·9 (26·9–46·7)	38·9 (29·9–50·5)	41·2 (32·1–53·1)	41·3 (32·3–52·7)	0·5 (0·2–0·7)	0·3 (0·0–0·6)	0·0 (-0·4–0·4)
Diet low in fruits	35·6 (30·0–38·9)	32·8 (28·9–35·8)	31·1 (26·9–33·7)	32·2 (26·8–36·1)	-0·3 (-0·7–0·1)	-0·1 (-0·6–0·4)	0·3 (-0·5–1·1)
Diet low in vegetables	23·6 (14·5–31·0)	25·7 (14·9–33·0)	29·6 (16·9–37·3)	30·2 (17·7–38·3)	0·8 (0·4–1·3)	0·8 (0·3–1·4)	0·2 (-0·6–1·0)
Diet low in legumes	39·4 (0·0–49·2)	39·9 (0·0–49·4)	44·0 (0·0–55·5)	45·0 (0·0–57·0)	0·4 (0·0–0·8)	0·6 (0·0–1·0)	0·2 (-0·5–0·9)
Diet low in whole grains	44·9 (37·8–51·0)	48·2 (40·0–54·9)	50·1 (41·7–57·0)	50·7 (42·2–57·9)	0·4 (0·1–0·7)	0·2 (-0·1–0·6)	0·1 (-0·4–0·6)
Diet low in nuts and seeds	14·9 (11·0–19·0)	9·4 (6·1–13·0)	6·8 (4·2–9·9)	7·5 (4·6–11·2)	-2·2 (-3·8–-0·8)	-1·1 (-3·3–1·3)	0·9 (-2·5–4·0)
Diet low in milk	38·3 (33·9–43·6)	36·1 (31·7–40·6)	34·3 (29·9–38·8)	34·3 (29·5–39·6)	-0·4 (-0·8–0·1)	-0·2 (-0·8–0·4)	0·0 (-0·8–0·9)
Diet high in red meat	48·2 (0·0–66·4)	48·5 (0·0–67·9)	48·8 (0·0–67·7)	48·8 (0·0–66·8)	0·0 (-0·5–0·6)	0·0 (-0·6–1·0)	0·0 (-1·0–1·1)
Diet high in processed meat	45·5 (35·5–50·6)	56·6 (45·4–63·1)	64·0 (51·5–71·1)	61·9 (50·5–69·3)	1·0 (0·6–1·4)	0·4 (-0·1–1·0)	-0·3 (-1·1–0·5)
Diet high in sugar-sweetened beverages	33·5 (24·5–41·7)	46·3 (37·2–55·8)	51·9 (41·5–61·5)	50·8 (39·7–60·7)	1·3 (0·4–2·3)	0·4 (-0·5–1·4)	-0·2 (-1·6–1·2)
Diet low in fibre	42·3 (22·8–49·3)	33·1 (18·1–39·9)	29·2 (15·8–35·7)	26·3 (14·3–33·2)	-1·5 (-2·4–-0·7)	-1·1 (-2·3–0·1)	-0·9 (-2·8–0·7)
Diet low in calcium	6·2 (5·0–7·6)	5·2 (4·2–6·4)	5·5 (4·4–6·8)	5·5 (4·4–6·8)	-0·4 (-0·6–-0·1)	0·2 (-0·1–0·5)	0·0 (-0·4–0·4)
Diet low in seafood omega-3 fatty acids	52·5 (42·1–63·2)	59·5 (48·1–70·6)	61·9 (50·7–72·7)	58·2 (47·4–69·5)	0·3 (0·0–0·6)	-0·1 (-0·5–0·3)	-0·6 (-1·0–-0·1)
Diet low in omega-6 polyunsaturated fatty acids	39·2 (25·2–49·7)	35·0 (23·0–44·9)	26·1 (18·3–34·2)	24·8 (17·1–32·3)	-1·5 (-2·2–-0·8)	-1·6 (-2·5–-0·8)	-0·5 (-1·5–0·5)
Diet high in trans fatty acids	71·3 (63·0–79·1)	71·8 (64·4–78·8)	63·3 (56·5–70·8)	0·0 (0·0–0·0)	-65·8 (-66·1–-65·4)	-97·1 (-97·5–-96·6)	-184·2 (-185·3–-183·2)
Diet high in sodium	23·0 (0·6–67·6)	27·7 (1·8–73·3)	30·8 (3·4–75·4)	31·3 (3·2–77·5)	1·0 (0·2–6·0)	0·6 (-0·1–3·9)	0·2 (-2·6–1·8)
Intimate partner violence	28·1 (16·8–37·2)	28·6 (18·7–36·0)	23·9 (16·9–29·7)	22·8 (12·5–31·0)	-0·7 (-2·1–0·6)	-1·1 (-3·0–0·8)	-0·4 (-2·8–1·9)
Childhood sexual abuse and bullying	8·6 (5·4–13·8)	10·5 (6·7–16·6)	11·9 (7·6–18·7)	9·1 (6·0–13·9)	0·2 (-0·4–0·8)	-0·7 (-1·5–0·2)	-2·5 (-3·9–-1·0)
Childhood sexual abuse	7·5 (6·4–8·8)	8·5 (7·3–10·0)	9·9 (8·5–11·5)	9·0 (7·3–10·8)	0·6 (-0·2–1·4)	0·3 (-0·8–1·2)	-0·8 (-2·4–0·6)
Bullying victimization	8·0 (3·5–16·2)	10·0 (4·6–19·5)	11·3 (5·1–22·1)	7·6 (3·3–14·6)	-0·1 (-0·8–0·6)	-1·3 (-2·2–-0·2)	-3·6 (-5·4–-1·7)
Unsafe sex	--	--	--	--	--	--	--
Low physical activity	18·8 (14·0–24·2)	17·7 (13·2–22·6)	17·9 (13·9–22·6)	20·5 (15·7–26·8)	0·3 (-0·7–1·3)	0·7 (-0·6–1·9)	1·2 (-0·6–3·1)
**Metabolic risks**	**24·2 (21·0–27·6)**	**29·9 (26·2–32·9)**	**33·6 (29·5–36·4)**	**38·0 (33·2–41·0)**	**1·5 (1·1–1·8)**	**1·1 (0·8–1·5)**	**1·1 (0·6–1·6)**
High fasting plasma glucose	13·0 (10·1–15·8)	16·9 (13·0–20·3)	22·1 (16·8–26·3)	25·4 (19·2–30·4)	2·2 (1·5–2·9)	1·9 (1·0–2·8)	1·3 (0·1–2·4)
High LDL cholesterol	62·9 (44·2–86·0)	53·3 (36·6–73·2)	47·9 (32·6–66·6)	46·4 (31·3–65·2)	-1·0 (-1·2–-0·8)	-0·7 (-0·9–-0·5)	-0·3 (-0·6–0·0)
High systolic blood pressure	26·6 (17·7–38·2)	22·8 (15·8–31·7)	17·8 (11·5–26·0)	25·2 (15·7–36·6)	-0·2 (-1·2–0·8)	0·5 (-0·7–1·7)	3·2 (1·0–5·7)
High body-mass index	27·0 (22·7–31·6)	33·9 (28·8–38·1)	38·2 (32·5–41·7)	42·9 (36·2–46·7)	1·5 (1·1–1·8)	1·1 (0·8–1·4)	1·0 (0·6–1·5)
Low bone mineral density	21·6 (15·7–28·5)	19·5 (13·9–26·4)	20·5 (14·4–27·3)	22·4 (16·2–29·4)	0·1 (-0·4–0·6)	0·7 (-0·1–1·4)	0·8 (-0·5–2·5)
Kidney dysfunction	2·9 (2·4–3·8)	3·0 (2·4–3·9)	2·9 (2·4–3·9)	3·1 (2·5–4·0)	0·2 (0·1–0·3)	0·2 (0·0–0·3)	0·4 (0·1–0·7)

New York

Risk Names	SEV 1990	SEV 2000	SEV 2010	SEV 2021	Annualised rate of change 1990 to 2021	Annualised rate of change 2000 to 2021	Annualised rate of change 2010 to 2021
**All risk factors**	**27·2 (24·2–30·3)**	**27·5 (24·5–30·2)**	**28·2 (25·3–31·1)**	**29·1 (26·0–32·3)**	**0·2 (-0·1–0·6)**	**0·3 (-0·1–0·7)**	**0·3 (-0·4–0·8)**
**Environmental/occupational risks**	**18·9 (12·1–29·4)**	**18·7 (12·8–29·4)**	**15·7 (10·3–25·7)**	**13·9 (8·8–23·2)**	**-1·0 (-1·7–-0·3)**	**-1·4 (-1·9–-0·8)**	**-1·1 (-1·6–-0·5)**
Unsafe water, sanitation, and handwashing	1·7 (0·8–2·6)	1·5 (0·6–2·5)	1·2 (0·4–2·1)	1·0 (0·3–1·7)	-1·5 (-3·9–0·4)	-1·9 (-5·2–0·7)	-1·6 (-5·5–2·3)
Unsafe water source	2·3 (0·8–4·7)	2·1 (0·8–4·3)	0·9 (0·4–1·7)	0·7 (0·3–1·4)	-3·6 (-6·4–-0·9)	-4·9 (-8·4–-1·4)	-1·6 (-6·9–3·9)
Unsafe sanitation	4·8 (2·6–7·8)	3·7 (1·9–6·4)	2·8 (1·4–5·0)	2·2 (1·2–3·8)	-2·5 (-4·8–-0·3)	-2·4 (-5·2–0·1)	-2·2 (-5·6–1·3)
No access to handwashing facility	1·6 (0·6–2·8)	1·6 (0·5–3·0)	1·6 (0·5–2·9)	1·4 (0·5–2·6)	-0·3 (-3·3–2·7)	-0·6 (-4·4–3·2)	-1·4 (-6·5–4·1)
Air pollution	26·2 (7·3–36·9)	25·0 (8·6–30·7)	17·8 (4·4–25·8)	10·4 (2·2–20·6)	-3·0 (-5·7–-1·0)	-4·2 (-8·1–-1·7)	-4·9 (-8·8–-1·6)
Particulate matter pollution	12·8 (4·6–22·7)	11·5 (8·2–15·3)	6·8 (3·8–10·0)	4·1 (1·6–7·0)	-3·7 (-6·8–-0·9)	-4·9 (-7·9–-3·5)	-4·6 (-8·6–-2·8)
Ambient particulate matter pollution	19·9 (7·4–35·3)	17·8 (13·1–23·0)	10·4 (6·0–14·9)	6·2 (2·5–10·5)	-3·8 (-6·9–-0·9)	-5·0 (-8·0–-3·5)	-4·6 (-8·7–-2·8)
Household air pollution from solid fuels	0·0 (0·0–0·0)	0·0 (0·0–0·0)	0·0 (0·0–0·0)	0·0 (0·0–0·0)	-4·3 (-41·2–-4·9)	-6·1 (-41·7–-5·1)	-6·2 (-39·1–-3·4)
Ambient ozone pollution	19·6 (13·9–26·4)	16·8 (10·9–23·5)	15·1 (9·8–22·1)	13·1 (7·9–19·7)	-1·3 (-2·1–-0·7)	-1·2 (-2·4–-0·2)	-1·3 (-3·3–0·4)
Ambient nitrogen dioxide pollution	84·4 (0·0–100·0)	84·4 (0·0–100·0)	66·8 (0·0–100·0)	38·0 (0·0–91·6)	-2·6 (-6·7–0·0)	-3·8 (-9·9–0·0)	-5·1 (-11·2–0·0)
Non-optimal temperature	29·1 (22·9–39·3)	28·6 (23·6–37·5)	32·9 (25·5–43·2)	30·0 (23·6–40·1)	0·1 (-0·1–0·3)	0·2 (-0·2–0·7)	-0·8 (-1·3–-0·5)
High temperature	23·6 (12·8–35·0)	19·6 (10·2–31·1)	35·9 (23·7–48·0)	28·7 (18·2–40·1)	0·6 (0·2–1·5)	1·8 (0·8–3·4)	-2·0 (-3·2–-1·1)
Low temperature	26·5 (23·5–30·1)	27·4 (24·5–30·8)	28·0 (25·1–31·6)	26·8 (23·8–30·4)	0·0 (-0·1–0·1)	-0·1 (-0·3–0·0)	-0·4 (-0·6–-0·2)
Other environmental risks	29·5 (12·4–46·4)	27·6 (12·3–44·2)	24·6 (12·1–41·4)	21·6 (11·0–38·3)	-1·0 (-1·7–0·0)	-1·2 (-2·0–0·0)	-1·2 (-2·2–0·0)
Residential radon	27·9 (0·0–82·2)	27·9 (0·0–82·2)	27·9 (0·0–82·2)	27·9 (0·0–82·2)	0·0 (0·0–0·0)	0·0 (0·0–0·0)	0·0 (0·0–0·0)
Lead exposure	30·2 (0·0–37·9)	27·4 (0·0–33·9)	23·1 (0·0–28·8)	18·8 (0·0–23·3)	-1·5 (-2·0–0·0)	-1·8 (-2·3–0·0)	-1·9 (-2·7–0·0)
Occupational risks	2·7 (2·3–3·6)	2·8 (2·3–3·7)	2·6 (2·1–3·4)	2·6 (2·1–3·4)	-0·1 (-0·4–0·1)	-0·3 (-0·7–0·1)	0·1 (-0·4–0·6)
Occupational carcinogens	0·9 (0·4–1·8)	1·0 (0·4–2·0)	1·0 (0·4–1·9)	1·0 (0·4–1·9)	0·1 (-0·3–0·3)	-0·2 (-0·7–0·2)	0·1 (-0·6–0·6)
Occupational exposure to asbestos	3·5 (3·1–3·9)	3·6 (3·3–4·0)	3·2 (2·9–3·5)	2·8 (2·2–3·5)	-0·7 (-1·6–0·1)	-1·3 (-2·5–-0·1)	-1·4 (-3·6–0·8)
Occupational exposure to arsenic	0·5 (0·0–1·1)	0·5 (0·0–1·1)	0·4 (0·0–1·0)	0·4 (0·0–1·1)	-0·2 (-0·7–0·2)	-0·2 (-0·9–0·4)	0·2 (-0·7–1·1)
Occupational exposure to benzene	1·1 (0·1–3·2)	1·2 (0·1–3·6)	1·2 (0·1–3·5)	1·2 (0·1–3·6)	0·3 (0·0–0·6)	0·0 (-0·5–0·4)	0·3 (-0·4–0·8)
Occupational exposure to beryllium	0·0 (0·0–0·0)	0·0 (0·0–0·0)	0·0 (0·0–0·0)	0·0 (0·0–0·0)	0·2 (0·0–0·5)	0·1 (-0·2–0·5)	0·5 (0·0–0·9)
Occupational exposure to cadmium	0·1 (0·1–0·1)	0·1 (0·1–0·1)	0·1 (0·0–0·1)	0·1 (0·0–0·1)	-0·2 (-0·6–0·2)	-0·2 (-0·7–0·4)	0·3 (-0·6–1·1)
Occupational exposure to chromium	0·1 (0·1–0·2)	0·1 (0·1–0·2)	0·1 (0·1–0·1)	0·1 (0·1–0·2)	-0·1 (-0·4–0·3)	-0·1 (-0·6–0·4)	0·3 (-0·4–1·0)
Occupational exposure to diesel engine exhaust	0·7 (0·6–0·7)	0·7 (0·7–0·7)	0·7 (0·6–0·7)	0·7 (0·7–0·8)	0·3 (-0·1–0·7)	0·3 (-0·2–0·8)	0·7 (0·0–1·4)
Occupational exposure to formaldehyde	0·3 (0·3–0·3)	0·3 (0·3–0·3)	0·3 (0·2–0·3)	0·3 (0·2–0·3)	-0·2 (-0·6–0·2)	-0·3 (-0·9–0·3)	0·1 (-0·7–0·9)
Occupational exposure to nickel	0·4 (0·0–1·5)	0·4 (0·0–1·5)	0·3 (0·0–1·3)	0·3 (0·0–1·3)	-0·3 (-0·9–0·2)	-0·2 (-1·1–0·5)	0·2 (-1·0–1·3)
Occupational exposure to polycyclic aromatic hydrocarbons	0·3 (0·3–0·3)	0·3 (0·3–0·3)	0·3 (0·3–0·3)	0·3 (0·3–0·3)	0·0 (-0·4–0·3)	-0·1 (-0·6–0·4)	0·3 (-0·3–0·9)
Occupational exposure to silica	3·2 (0·5–10·7)	3·1 (0·5–9·9)	2·8 (0·5–9·0)	2·9 (0·5–9·2)	-0·3 (-0·8–0·2)	-0·2 (-0·8–0·5)	0·3 (-0·7–1·3)
Occupational exposure to sulphuric acid	0·7 (0·1–2·3)	0·7 (0·1–2·3)	0·7 (0·1–2·1)	0·7 (0·1–2·1)	-0·1 (-0·6–0·3)	-0·1 (-0·7–0·5)	0·3 (-0·5–1·2)
Occupational exposure to trichloroethylene	0·1 (0·1–0·1)	0·1 (0·1–0·1)	0·1 (0·1–0·1)	0·1 (0·1–0·1)	0·1 (-0·2–0·4)	0·0 (-0·4–0·4)	0·5 (-0·1–1·0)
Occupational asthmagens	16·4 (14·3–18·5)	16·6 (14·7–18·7)	15·4 (13·6–17·5)	15·9 (13·9–18·2)	-0·1 (-0·5–0·2)	-0·2 (-0·7–0·2)	0·3 (-0·4–1·0)
Occupational particulate matter, gases, and fumes	5·9 (4·5–7·7)	5·7 (4·3–7·3)	5·3 (4·1–6·8)	4·9 (3·8–6·3)	-0·6 (-0·9–-0·3)	-0·7 (-1·0–-0·3)	-0·7 (-1·0–-0·3)
Occupational noise	6·3 (5·7–6·9)	6·3 (5·8–6·9)	6·2 (5·8–6·8)	6·0 (5·7–6·5)	-0·1 (-0·3–0·1)	-0·2 (-0·4–0·0)	-0·3 (-0·5–0·0)
Occupational injuries	--	--	--	--	--	--	--
Occupational ergonomic factors	7·5 (6·4–9·1)	7·6 (6·4–9·1)	6·9 (5·8–8·4)	7·2 (6·0–8·9)	-0·2 (-0·6–0·2)	-0·3 (-0·8–0·2)	0·3 (-0·4–1·0)
**Behavioural risks**	**31·0 (27·8–34·5)**	**28·7 (25·5–32·3)**	**26·4 (23·4–29·8)**	**24·6 (21·5–28·2)**	**-0·7 (-1·1–-0·4)**	**-0·7 (-1·2–-0·3)**	**-0·6 (-1·3–0·0)**
Child and maternal malnutrition	10·4 (7·2–14·6)	8·7 (6·0–12·0)	8·7 (6·1–12·1)	8·9 (6·0–12·0)	-0·5 (-1·3–0·2)	0·1 (-1·1–1·2)	0·1 (-1·7–1·8)
Suboptimal breastfeeding	68·9 (66·4–71·3)	66·7 (64·4–69·1)	62·8 (60·0–65·5)	62·2 (59·4–65·0)	-0·3 (-0·5–-0·2)	-0·3 (-0·5–-0·1)	-0·1 (-0·4–0·2)
Non-exclusive breastfeeding	64·2 (56·3–71·4)	59·4 (52·6–65·6)	48·5 (42·2–54·2)	46·2 (39·7–52·1)	-1·1 (-1·6–-0·6)	-1·2 (-1·9–-0·6)	-0·4 (-1·4–0·5)
Discontinued breastfeeding	88·8 (86·8–91·1)	86·4 (83·9–89·2)	84·4 (81·2–87·5)	84·5 (81·2–87·9)	-0·2 (-0·3–0·0)	-0·1 (-0·3–0·1)	0·0 (-0·3–0·3)
Child growth failure	1·3 (0·4–2·9)	1·1 (0·4–2·5)	0·9 (0·3–2·1)	0·9 (0·3–1·9)	-1·2 (-1·5–-0·8)	-1·1 (-1·6–-0·6)	-0·7 (-1·4–0·1)
Child underweight	2·7 (1·7–3·7)	2·3 (1·5–3·2)	2·0 (1·2–2·7)	1·8 (1·1–2·5)	-1·2 (-1·7–-0·7)	-1·2 (-1·9–-0·5)	-0·6 (-1·6–0·4)
Child wasting	0·9 (0·5–1·3)	0·8 (0·5–1·2)	0·8 (0·5–1·2)	0·7 (0·5–1·2)	-0·5 (-0·8–-0·1)	-0·2 (-0·6–0·3)	-0·3 (-0·9–0·4)
Child stunting	3·7 (2·9–4·7)	3·3 (2·6–4·0)	2·8 (2·2–3·5)	2·5 (1·9–3·1)	-1·3 (-1·8–-0·6)	-1·2 (-2·0–-0·3)	-0·9 (-2·1–0·5)
Low birth weight and short gestation	19·0 (16·6–21·5)	20·2 (17·7–22·7)	21·1 (18·4–23·8)	21·4 (18·7–24·2)	0·4 (0·1–0·6)	0·3 (-0·1–0·7)	0·1 (-0·6–0·9)
Short gestation	33·4 (29·3–38·2)	35·9 (31·4–41·0)	35·5 (31·1–40·0)	35·7 (30·9–40·8)	0·2 (-0·1–0·5)	0·0 (-0·5–0·5)	0·1 (-0·8–0·9)
Low birth weight	14·6 (13·1–16·1)	15·2 (13·7–16·7)	16·3 (14·7–17·9)	16·6 (15·0–18·3)	0·4 (0·2–0·6)	0·4 (0·1–0·8)	0·2 (-0·5–0·9)

Iron deficiency	3·8 (2·7–5·1)	3·1 (2·2–4·2)	3·2 (2·3–4·3)	3·2 (2·2–4·2)	-0·5 (-1·3–0·1)	0·1 (-1·0–1·2)	0·1 (-1·6–1·6)
Vitamin A deficiency	0·8 (0·0–1·6)	1·0 (0·0–2·0)	0·6 (0·0–1·2)	0·4 (0·0–0·8)	-2·4 (-4·5–0·0)	-4·6 (-7·5–0·0)	-4·3 (-7·8–0·0)
Zinc deficiency	1·6 (0·0–6·7)	1·0 (0·0–4·0)	0·8 (0·0–3·7)	0·8 (0·0–3·4)	-2·1 (-41·5–1·9)	-0·7 (-11·9–5·7)	-0·3 (-4·1–8·5)
Tobacco	41·1 (39·5–42·5)	35·5 (34·0–36·7)	27·3 (26·4–28·3)	23·0 (21·5–24·6)	-1·9 (-2·1–-1·7)	-2·1 (-2·4–-1·7)	-1·6 (-2·2–-0·9)
Smoking	37·3 (34·9–39·3)	31·8 (29·9–33·6)	24·9 (23·5–26·4)	20·4 (18·8–22·2)	-1·9 (-2·3–-1·7)	-2·1 (-2·6–-1·7)	-1·8 (-2·6–-1·1)
Chewing tobacco	0·7 (0·5–0·9)	0·9 (0·6–1·1)	1·0 (0·8–1·4)	1·1 (0·8–1·5)	1·6 (0·2–2·9)	1·2 (-0·7–3·0)	0·5 (-1·9–3·2)
Second-hand smoke	34·3 (32·5–35·8)	29·9 (27·7–31·2)	22·8 (21·2–23·9)	19·9 (18·0–21·5)	-1·8 (-2·0–-1·5)	-1·9 (-2·3–-1·6)	-1·3 (-2·0–-0·6)
High alcohol use	30·7 (22·4–40·4)	29·6 (21·8–39·5)	30·7 (23·1–40·2)	30·6 (22·2–40·9)	0·0 (-0·9–0·8)	0·2 (-1·0–1·2)	0·0 (-1·5–1·3)
Drug use	1·2 (0·7–2·1)	1·5 (1·3–1·8)	2·1 (1·8–2·5)	4·0 (3·1–5·1)	4·0 (1·4–6·3)	4·8 (2·9–6·0)	5·9 (4·1–7·1)
Dietary risks	35·5 (26·3–46·7)	38·1 (28·9–49·5)	39·7 (30·5–51·8)	39·9 (30·4–51·8)	0·4 (0·2–0·6)	0·2 (-0·1–0·5)	0·0 (-0·4–0·5)
Diet low in fruits	34·6 (29·4–37·6)	32·0 (27·3–35·0)	31·4 (27·4–34·0)	32·5 (27·6–36·4)	-0·2 (-0·6–0·1)	0·1 (-0·5–0·6)	0·3 (-0·7–1·1)
Diet low in vegetables	24·8 (15·3–32·3)	24·9 (14·7–32·7)	26·9 (15·1–34·4)	26·0 (15·0–34·5)	0·2 (-0·3–0·6)	0·2 (-0·4–0·8)	-0·3 (-1·3–0·6)
Diet low in legumes	36·5 (0·0–46·0)	36·7 (0·0–46·0)	40·7 (0·0–51·1)	40·9 (0·0–51·6)	0·4 (0·0–0·7)	0·5 (0·0–1·0)	0·1 (-0·6–0·8)
Diet low in whole grains	44·3 (36·5–50·8)	47·8 (39·9–54·1)	48·8 (40·9–55·6)	49·2 (40·7–56·6)	0·3 (0·1–0·6)	0·1 (-0·2–0·5)	0·1 (-0·4–0·6)
Diet low in nuts and seeds	10·1 (6·8–13·9)	5·5 (3·3–8·2)	3·9 (2·1–5·8)	4·0 (2·2–6·3)	-3·0 (-5·1–-1·4)	-1·5 (-4·5–1·3)	0·3 (-4·1–4·7)
Diet low in milk	34·2 (29·9–38·6)	31·4 (27·3–35·5)	30·0 (25·8–34·1)	29·1 (24·4–33·9)	-0·5 (-1·0–0·0)	-0·4 (-1·0–0·3)	-0·3 (-1·2–0·6)
Diet high in red meat	48·1 (0·0–66·6)	49·5 (0·0–68·1)	49·9 (0·0–68·1)	50·5 (0·0–69·9)	0·2 (-0·3–0·7)	0·1 (-0·5–0·8)	0·1 (-0·9–1·4)
Diet high in processed meat	41·7 (33·1–46·3)	52·0 (41·2–57·8)	57·6 (46·5–64·5)	56·3 (45·5–63·7)	1·0 (0·6–1·4)	0·4 (-0·1–0·9)	-0·2 (-1·0–0·6)
Diet high in sugar-sweetened beverages	38·9 (29·5–47·8)	53·9 (42·1–61·9)	58·6 (45·7–67·2)	59·1 (45·3–69·0)	1·3 (0·6–2·1)	0·4 (-0·4–1·3)	0·1 (-1·1–1·2)
Diet low in fibre	35·5 (19·8–42·2)	26·4 (15·0–32·3)	23·3 (13·2–28·7)	19·5 (11·3–25·8)	-1·9 (-2·8–-1·0)	-1·4 (-2·7–-0·2)	-1·6 (-3·5–0·4)
Diet low in calcium	5·2 (4·2–6·3)	4·5 (3·6–5·5)	4·7 (3·8–5·7)	4·5 (3·7–5·5)	-0·4 (-0·6–-0·2)	0·1 (-0·1–0·3)	-0·3 (-0·6–0·0)
Diet low in seafood omega-3 fatty acids	32·3 (23·2–41·7)	39·6 (30·3–49·3)	42·7 (32·6–52·9)	36·4 (26·4–46·1)	0·4 (-0·1–0·8)	-0·4 (-1·0–0·1)	-1·4 (-2·3–-0·7)
Diet low in omega-6 polyunsaturated fatty acids	31·7 (20·9–40·5)	27·6 (18·7–35·3)	20·6 (14·3–26·8)	18·8 (13·1–24·4)	-1·7 (-2·5–-0·9)	-1·8 (-2·8–-1·0)	-0·9 (-1·8–0·0)
Diet high in trans fatty acids	71·5 (65·3–78·7)	71·9 (64·5–79·1)	63·5 (56·8–70·7)	0·0 (0·0–0·0)	-65·8 (-66·1–-65·5)	-97·1 (-97·6–-96·6)	-184·3 (-185·2–-183·3)
Diet high in sodium	23·2 (0·6–67·7)	27·9 (1·9–75·8)	30·8 (3·3–74·6)	31·2 (3·0–76·5)	1·0 (0·2–6·5)	0·5 (0·0–3·6)	0·1 (-2·5–1·4)
Intimate partner violence	25·6 (15·4–33·0)	25·5 (16·3–32·5)	22·9 (16·6–28·2)	21·9 (12·5–30·0)	-0·5 (-1·9–0·9)	-0·7 (-2·6–1·1)	-0·4 (-2·8–1·8)
Childhood sexual abuse and bullying	5·5 (3·8–8·4)	5·9 (4·2–8·7)	6·6 (4·9–9·6)	5·9 (4·3–8·2)	0·2 (-0·5–0·8)	0·0 (-0·8–0·8)	-1·1 (-2·4–0·1)
Childhood sexual abuse	7·6 (6·4–9·0)	8·6 (7·5–10·0)	10·1 (8·9–11·6)	9·3 (7·8–11·1)	0·7 (-0·1–1·4)	0·4 (-0·6–1·3)	-0·7 (-2·2–0·7)
Bullying victimization	3·5 (1·3–7·6)	3·5 (1·3–7·3)	3·7 (1·4–7·8)	2·8 (1·0–5·8)	-0·7 (-1·4–0·0)	-0·9 (-1·8–0·0)	-2·4 (-4·2–-0·7)
Unsafe sex	--	--	--	--	--	--	--
Low physical activity	20·5 (15·2–26·6)	20·0 (15·4–25·3)	19·6 (15·4–24·5)	22·2 (17·1–27·8)	0·2 (-0·6–1·1)	0·5 (-0·6–1·7)	1·1 (-0·4–2·7)
**Metabolic risks**	**24·6 (21·3–27·9)**	**30·1 (26·1–33·1)**	**33·0 (29·0–35·7)**	**36·0 (31·4–39·2)**	**1·2 (0·9–1·6)**	**0·9 (0·5–1·2)**	**0·8 (0·3–1·3)**
High fasting plasma glucose	12·1 (9·4–14·6)	15·6 (11·7–18·5)	20·5 (15·5–24·2)	24·3 (18·4–28·5)	2·2 (1·6–2·8)	2·1 (1·3–2·8)	1·5 (0·4–2·5)
High LDL cholesterol	64·0 (45·3–87·2)	54·1 (37·1–75·0)	48·3 (33·0–67·0)	46·6 (31·5–65·2)	-1·0 (-1·2–-0·8)	-0·7 (-0·9–-0·5)	-0·3 (-0·6–-0·1)
High systolic blood pressure	28·1 (19·2–39·4)	24·3 (17·1–33·4)	17·8 (11·6–25·7)	24·5 (15·5–35·7)	-0·5 (-1·3–0·4)	0·0 (-1·2–1·1)	2·9 (0·6–5·3)
High body-mass index	28·0 (23·2–32·8)	34·6 (29·2–38·6)	37·8 (32·1–41·4)	40·4 (34·1–44·1)	1·2 (0·9–1·5)	0·7 (0·4–1·1)	0·6 (0·1–1·0)
Low bone mineral density	21·5 (15·3–28·6)	19·5 (14·1–26·8)	20·3 (14·1–27·1)	22·1 (15·6–28·8)	0·1 (-0·4–0·6)	0·6 (-0·1–1·4)	0·8 (-0·6–2·3)
Kidney dysfunction	2·9 (2·4–3·8)	2·9 (2·4–3·9)	2·9 (2·3–3·9)	3·0 (2·4–4·0)	0·1 (0·0–0·2)	0·1 (-0·1–0·2)	0·3 (0·0–0·6)

North Carolina

Risk Names	SEV 1990	SEV 2000	SEV 2010	SEV 2021	Annualised rate of change 1990 to 2021	Annualised rate of change 2000 to 2021	Annualised rate of change 2010 to 2021
**All risk factors**	**26·6 (23·5–29·6)**	**26·5 (23·7–29·4)**	**27·0 (24·2–30·0)**	**26·7 (23·7–29·9)**	**0·0 (-0·3–0·3)**	**0·0 (-0·3–0·4)**	**-0·1 (-0·6–0·4)**
**Environmental/occupational risks**	**18·6 (12·7–28·9)**	**18·7 (13·3–28·7)**	**15·5 (10·4–24·2)**	**12·7 (8·3–20·2)**	**-1·2 (-2·1–-0·5)**	**-1·8 (-2·5–-1·2)**	**-1·8 (-2·4–-1·2)**
Unsafe water, sanitation, and handwashing	2·1 (1·1–3·5)	2·0 (0·9–3·2)	1·4 (0·5–2·4)	1·2 (0·4–2·0)	-1·9 (-4·2–0·0)	-2·4 (-5·5–0·1)	-1·9 (-5·7–1·8)
Unsafe water source	3·1 (1·2–6·8)	2·8 (1·1–5·9)	1·1 (0·5–2·3)	0·9 (0·4–1·7)	-4·0 (-6·8–-1·2)	-5·6 (-9·6–-1·3)	-2·1 (-7·9–3·3)
Unsafe sanitation	7·4 (4·1–11·8)	5·9 (3·2–10·1)	4·3 (2·3–7·8)	3·2 (1·6–5·5)	-2·7 (-4·9–-0·7)	-2·9 (-5·7–-0·4)	-2·6 (-5·9–0·6)
No access to handwashing facility	1·7 (0·6–3·1)	1·7 (0·6–3·1)	1·7 (0·6–3·0)	1·4 (0·4–2·6)	-0·5 (-3·2–2·6)	-0·8 (-4·7–2·8)	-1·5 (-6·9–3·7)
Air pollution	22·0 (9·4–34·7)	19·9 (11·2–30·6)	9·8 (5·2–17·9)	5·4 (1·8–12·6)	-4·5 (-7·4–-2·6)	-6·2 (-9·7–-4·1)	-5·4 (-9·9–-3·2)
Particulate matter pollution	16·1 (7·0–27·1)	14·3 (10·6–18·6)	7·9 (4·7–11·5)	4·1 (1·6–7·1)	-4·4 (-7·5–-1·9)	-5·9 (-9·3–-4·3)	-5·8 (-11·0–-3·7)
Ambient particulate matter pollution	25·1 (10·9–42·1)	22·2 (17·0–27·8)	12·0 (7·5–17·1)	6·3 (2·4–10·6)	-4·4 (-7·6–-1·9)	-6·0 (-9·4–-4·4)	-5·9 (-11·0–-3·7)
Household air pollution from solid fuels	0·0 (0·0–0·2)	0·0 (0·0–0·1)	0·0 (0·0–0·0)	0·0 (0·0–0·0)	-4·8 (-27·9–-6·5)	-5·0 (-22·1–-4·6)	-5·1 (-16·9–-2·3)
Ambient ozone pollution	27·7 (21·3–35·9)	31·2 (24·3–40·0)	21·3 (15·6–28·7)	12·1 (7·3–18·5)	-2·7 (-3·6–-2·0)	-4·5 (-5·9–-3·5)	-5·2 (-7·2–-3·7)
Ambient nitrogen dioxide pollution	43·6 (0·0–100·0)	39·6 (0·0–95·1)	15·2 (0·0–55·3)	9·4 (0·0–46·3)	-4·9 (-17·2–0·0)	-6·8 (-24·4–0·0)	-4·3 (-21·0–0·0)
Non-optimal temperature	25·8 (19·9–35·1)	25·9 (21·6–33·0)	32·0 (25·5–41·8)	25·0 (20·4–32·6)	-0·1 (-0·4–0·2)	-0·2 (-0·4–0·2)	-2·2 (-3·0–-1·5)
High temperature	21·4 (14·1–30·6)	15·5 (9·2–24·5)	27·2 (18·8–36·8)	17·7 (10·7–27·1)	-0·6 (-1·2–-0·2)	0·6 (0·2–1·1)	-3·9 (-5·9–-2·4)
Low temperature	22·9 (19·5–26·8)	25·4 (22·2–29·2)	28·5 (25·5–31·9)	23·5 (20·7–27·2)	0·1 (-0·1–0·3)	-0·4 (-0·6–-0·2)	-1·7 (-2·1–-1·3)
Other environmental risks	24·7 (9·7–41·0)	23·5 (10·0–40·0)	21·4 (9·7–36·9)	19·2 (9·0–34·8)	-0·8 (-1·4–0·0)	-0·9 (-1·7–0·0)	-1·0 (-1·9–0·0)
Residential radon	24·9 (0·0–71·5)	24·9 (0·0–71·5)	24·9 (0·0–71·5)	24·9 (0·0–71·5)	0·0 (0·0–0·0)	0·0 (0·0–0·0)	0·0 (0·0–0·0)
Lead exposure	24·6 (0·0–30·7)	22·8 (0·0–28·6)	19·7 (0·0–24·6)	16·7 (0·0–21·0)	-1·3 (-1·7–0·0)	-1·5 (-2·0–0·0)	-1·5 (-2·4–0·0)
Occupational risks	3·0 (2·5–3·8)	3·0 (2·5–3·9)	2·8 (2·2–3·6)	2·8 (2·2–3·6)	-0·3 (-0·6–0·0)	-0·4 (-0·8–-0·1)	0·0 (-0·6–0·5)
Occupational carcinogens	0·9 (0·4–1·7)	1·0 (0·4–1·8)	0·9 (0·4–1·8)	0·9 (0·4–1·8)	0·1 (-0·3–0·4)	-0·1 (-0·5–0·3)	0·2 (-0·4–0·8)
Occupational exposure to asbestos	3·2 (2·8–3·6)	3·4 (3·0–3·8)	3·2 (2·8–3·5)	2·8 (2·1–3·6)	-0·5 (-1·4–0·3)	-1·0 (-2·3–0·2)	-1·2 (-3·5–1·0)
Occupational exposure to arsenic	0·6 (0·0–1·4)	0·6 (0·0–1·4)	0·5 (0·0–1·2)	0·5 (0·0–1·2)	-0·5 (-1·0–0·0)	-0·6 (-1·2–0·0)	0·0 (-0·9–0·9)
Occupational exposure to benzene	1·0 (0·1–3·0)	1·1 (0·1–3·2)	1·1 (0·1–3·2)	1·2 (0·1–3·4)	0·4 (0·0–0·8)	0·2 (-0·3–0·7)	0·5 (-0·2–1·1)
Occupational exposure to beryllium	0·0 (0·0–0·0)	0·0 (0·0–0·0)	0·0 (0·0–0·0)	0·0 (0·0–0·0)	0·2 (-0·1–0·4)	0·0 (-0·3–0·4)	0·5 (0·0–1·0)
Occupational exposure to cadmium	0·1 (0·1–0·1)	0·1 (0·1–0·1)	0·1 (0·1–0·1)	0·1 (0·1–0·1)	-0·4 (-0·8–0·0)	-0·5 (-1·0–0·1)	0·1 (-0·8–0·9)
Occupational exposure to chromium	0·2 (0·1–0·2)	0·2 (0·1–0·2)	0·1 (0·1–0·2)	0·1 (0·1–0·2)	-0·2 (-0·6–0·1)	-0·4 (-0·8–0·1)	0·1 (-0·6–0·8)
Occupational exposure to diesel engine exhaust	0·6 (0·6–0·7)	0·7 (0·6–0·7)	0·6 (0·6–0·7)	0·7 (0·6–0·8)	0·4 (0·1–0·8)	0·3 (-0·2–0·9)	0·8 (0·0–1·5)
Occupational exposure to formaldehyde	0·3 (0·3–0·4)	0·3 (0·3–0·4)	0·3 (0·3–0·3)	0·3 (0·3–0·3)	-0·4 (-0·8–0·0)	-0·5 (-1·1–0·0)	0·0 (-0·9–0·8)
Occupational exposure to nickel	0·5 (0·0–1·8)	0·4 (0·0–1·8)	0·4 (0·0–1·5)	0·4 (0·0–1·5)	-0·6 (-1·1–0·0)	-0·6 (-1·3–0·1)	-0·1 (-1·1–1·2)
Occupational exposure to polycyclic aromatic hydrocarbons	0·3 (0·3–0·3)	0·3 (0·3–0·3)	0·3 (0·3–0·3)	0·3 (0·3–0·3)	-0·2 (-0·5–0·2)	-0·3 (-0·8–0·2)	0·2 (-0·5–0·9)
Occupational exposure to silica	4·0 (0·6–12·7)	3·8 (0·6–12·3)	3·4 (0·5–10·9)	3·4 (0·5–10·6)	-0·5 (-1·0–0·0)	-0·5 (-1·2–0·1)	0·1 (-1·0–1·1)
Occupational exposure to sulphuric acid	0·8 (0·2–2·7)	0·8 (0·2–2·6)	0·7 (0·2–2·4)	0·7 (0·2–2·4)	-0·4 (-0·8–0·0)	-0·5 (-1·0–0·1)	0·1 (-0·8–0·9)
Occupational exposure to trichloroethylene	0·1 (0·1–0·1)	0·1 (0·1–0·1)	0·1 (0·1–0·1)	0·1 (0·1–0·1)	0·0 (-0·3–0·3)	-0·1 (-0·5–0·3)	0·4 (-0·2–0·9)
Occupational asthmagens	17·8 (15·5–20·1)	18·0 (15·9–20·2)	16·1 (14·3–18·4)	16·5 (14·2–19·1)	-0·3 (-0·7–0·1)	-0·4 (-0·9–0·1)	0·2 (-0·6–1·0)
Occupational particulate matter, gases, and fumes	7·4 (5·7–9·6)	7·1 (5·5–9·4)	6·6 (5·1–8·6)	6·0 (4·7–7·8)	-0·7 (-0·9–-0·4)	-0·8 (-1·1–-0·5)	-0·9 (-1·3–-0·5)
Occupational noise	6·9 (6·4–7·6)	6·9 (6·4–7·6)	6·8 (6·4–7·4)	6·5 (6·1–7·0)	-0·2 (-0·4–0·0)	-0·3 (-0·5–-0·1)	-0·4 (-0·6–-0·2)
Occupational injuries	--	--	--	--	--	--	--
Occupational ergonomic factors	8·4 (7·0–10·1)	8·3 (6·9–9·9)	7·3 (6·2–8·9)	7·4 (6·1–9·2)	-0·4 (-0·9–0·0)	-0·5 (-1·0–0·0)	0·2 (-0·5–1·0)
**Behavioural risks**	**29·9 (26·8–33·3)**	**28·4 (25·4–31·8)**	**26·9 (23·9–30·4)**	**24·7 (21·7–28·3)**	**-0·6 (-0·9–-0·3)**	**-0·7 (-1·1–-0·2)**	**-0·8 (-1·4–-0·2)**
Child and maternal malnutrition	10·4 (6·9–14·5)	8·9 (6·1–12·2)	9·0 (6·2–12·2)	9·2 (6·0–12·8)	-0·4 (-1·2–0·4)	0·1 (-1·0–1·3)	0·2 (-1·5–2·1)
Suboptimal breastfeeding	68·9 (66·6–71·4)	66·8 (64·3–69·2)	62·9 (60·5–65·6)	62·3 (59·2–65·4)	-0·3 (-0·5–-0·2)	-0·3 (-0·5–-0·1)	-0·1 (-0·4–0·2)
Non-exclusive breastfeeding	64·5 (57·1–71·5)	59·8 (53·4–66·2)	48·7 (42·4–55·6)	46·5 (39·1–54·0)	-1·1 (-1·6–-0·6)	-1·2 (-1·9–-0·6)	-0·4 (-1·4–0·5)
Discontinued breastfeeding	88·8 (86·4–91·2)	86·4 (83·7–89·4)	84·5 (81·5–87·7)	84·7 (81·2–88·1)	-0·2 (-0·3–0·0)	-0·1 (-0·3–0·1)	0·0 (-0·2–0·3)
Child growth failure	1·4 (0·4–3·2)	1·3 (0·4–2·8)	1·1 (0·3–2·4)	1·0 (0·3–2·3)	-1·0 (-1·5–-0·7)	-1·0 (-1·6–-0·5)	-0·6 (-1·3–0·2)
Child underweight	3·0 (1·9–4·1)	2·7 (1·7–3·6)	2·2 (1·4–3·1)	2·1 (1·3–2·9)	-1·1 (-1·6–-0·5)	-1·1 (-1·7–-0·4)	-0·5 (-1·5–0·5)
Child wasting	0·9 (0·6–1·4)	0·9 (0·5–1·3)	0·8 (0·5–1·3)	0·8 (0·5–1·3)	-0·4 (-0·7–0·0)	-0·1 (-0·6–0·3)	-0·2 (-0·9–0·5)
Child stunting	4·0 (3·1–5·0)	3·6 (2·9–4·6)	3·1 (2·4–3·9)	2·8 (2·2–3·6)	-1·1 (-1·7–-0·5)	-1·2 (-1·9–-0·4)	-0·8 (-1·9–0·4)
Low birth weight and short gestation	22·0 (19·4–24·8)	23·2 (20·6–26·4)	24·1 (21·0–27·2)	24·3 (21·3–27·5)	0·3 (0·1–0·6)	0·2 (-0·2–0·6)	0·1 (-0·6–0·7)
Short gestation	38·6 (34·5–43·1)	41·0 (37·1–45·4)	41·7 (37·3–46·7)	41·3 (37·2–46·0)	0·2 (-0·1–0·5)	0·0 (-0·4–0·5)	-0·1 (-0·9–0·8)
Low birth weight	16·8 (15·5–18·3)	17·4 (15·9–18·9)	18·2 (16·8–19·7)	18·5 (17·0–20·2)	0·3 (0·1–0·6)	0·3 (0·0–0·6)	0·2 (-0·6–0·8)

Iron deficiency	3·7 (2·6–5·1)	3·2 (2·3–4·3)	3·3 (2·3–4·4)	3·4 (2·3–4·6)	-0·3 (-1·1–0·4)	0·2 (-0·8–1·3)	0·2 (-1·4–1·9)
Vitamin A deficiency	1·2 (0·0–2·5)	1·7 (0·0–3·5)	1·0 (0·0–2·1)	0·6 (0·0–1·3)	-2·1 (-4·5–0·1)	-4·7 (-7·8–0·0)	-4·5 (-8·5–0·0)
Zinc deficiency	2·0 (0·0–8·4)	1·1 (0·0–4·9)	1·0 (0·0–4·8)	1·1 (0·0–4·7)	-1·9 (-42·3–0·2)	-0·2 (-3·3–5·5)	0·2 (-4·2–4·2)
Tobacco	45·0 (43·3–46·6)	41·7 (40·2–43·1)	34·9 (33·7–36·1)	28·9 (26·9–30·9)	-1·4 (-1·6–-1·2)	-1·7 (-2·1–-1·4)	-1·7 (-2·3–-1·1)
Smoking	40·2 (37·8–42·4)	35·6 (33·4–37·4)	29·5 (27·8–31·2)	24·2 (22·2–26·3)	-1·6 (-1·9–-1·4)	-1·8 (-2·3–-1·4)	-1·8 (-2·6–-1·0)
Chewing tobacco	4·5 (3·7–5·3)	3·8 (3·1–4·7)	3·3 (2·6–4·2)	3·1 (2·3–4·1)	-1·2 (-2·3–-0·1)	-1·1 (-2·6–0·4)	-0·8 (-3·2–1·4)
Second-hand smoke	40·2 (37·8–42·0)	39·2 (36·6–40·8)	33·9 (31·5–35·4)	28·3 (25·9–30·7)	-1·1 (-1·4–-0·9)	-1·5 (-1·9–-1·2)	-1·6 (-2·2–-1·0)
High alcohol use	23·9 (17·1–34·0)	22·6 (16·0–32·3)	24·7 (17·4–34·8)	25·3 (18·2–35·6)	0·2 (-0·8–1·3)	0·6 (-0·7–1·9)	0·2 (-1·5–1·9)
Drug use	1·0 (0·6–1·6)	1·3 (1·1–1·6)	2·5 (1·9–3·1)	5·6 (3·7–7·2)	5·7 (2·8–7·9)	6·8 (4·5–8·1)	7·3 (5·4–8·6)
Dietary risks	37·1 (27·6–48·4)	39·9 (30·4–51·8)	42·2 (32·5–54·1)	42·6 (33·5–54·7)	0·5 (0·2–0·7)	0·3 (0·0–0·6)	0·1 (-0·3–0·6)
Diet low in fruits	39·7 (34·8–43·3)	36·8 (32·4–40·0)	36·0 (31·1–38·8)	37·1 (32·6–41·3)	-0·2 (-0·5–0·1)	0·0 (-0·4–0·5)	0·3 (-0·6–1·1)
Diet low in vegetables	26·0 (15·7–33·1)	26·6 (16·1–33·6)	30·9 (17·4–38·3)	33·1 (18·8–41·3)	0·8 (0·4–1·2)	1·0 (0·6–1·6)	0·6 (-0·2–1·5)
Diet low in legumes	38·7 (0·0–48·7)	38·7 (0·0–48·5)	43·0 (0·0–54·0)	44·0 (0·0–55·2)	0·4 (0·0–0·7)	0·6 (0·0–1·1)	0·2 (-0·6–0·9)
Diet low in whole grains	45·5 (37·8–51·9)	48·7 (40·3–55·5)	50·5 (40·9–58·0)	50·8 (41·7–58·1)	0·4 (0·1–0·6)	0·2 (-0·2–0·6)	0·1 (-0·5–0·7)
Diet low in nuts and seeds	13·7 (9·6–17·7)	7·9 (5·1–11·2)	5·9 (3·4–8·5)	6·5 (3·8–9·8)	-2·4 (-4·2–-0·8)	-0·9 (-3·2–1·3)	0·9 (-2·7–4·1)
Diet low in milk	37·7 (33·7–42·5)	34·3 (29·8–39·1)	33·3 (28·8–37·7)	33·6 (28·9–38·7)	-0·4 (-0·8–0·1)	-0·1 (-0·8–0·5)	0·1 (-0·8–0·9)
Diet high in red meat	50·9 (0·0–69·7)	51·9 (0·0–70·6)	51·6 (0·0–70·3)	51·3 (0·0–70·0)	0·0 (-0·4–0·4)	-0·1 (-0·6–0·6)	-0·1 (-0·9–0·9)
Diet high in processed meat	43·3 (34·6–48·5)	54·9 (44·2–60·9)	62·2 (49·7–70·2)	60·3 (49·3–68·7)	1·1 (0·6–1·5)	0·4 (-0·1–1·0)	-0·3 (-1·1–0·5)
Diet high in sugar-sweetened beverages	35·2 (26·6–42·9)	50·1 (40·3–58·8)	54·9 (44·5–65·0)	53·5 (42·3–64·2)	1·4 (0·5–2·2)	0·3 (-0·6–1·3)	-0·2 (-1·5–1·0)
Diet low in fibre	43·9 (24·7–50·8)	33·6 (18·2–40·7)	29·9 (15·6–36·5)	26·7 (14·8–33·5)	-1·6 (-2·4–-0·8)	-1·1 (-2·3–0·0)	-1·0 (-2·7–0·6)
Diet low in calcium	6·1 (5·0–7·5)	4·9 (4·0–6·1)	5·3 (4·3–6·5)	5·4 (4·3–6·6)	-0·4 (-0·7–-0·2)	0·4 (0·1–0·7)	0·1 (-0·3–0·5)
Diet low in seafood omega-3 fatty acids	38·2 (28·7–48·5)	44·3 (34·2–55·4)	48·0 (37·8–58·8)	43·8 (34·0–54·4)	0·4 (0·0–0·9)	-0·1 (-0·6–0·5)	-0·8 (-1·5–-0·2)
Diet low in omega-6 polyunsaturated fatty acids	37·5 (24·1–47·4)	32·2 (21·3–41·5)	24·4 (16·9–31·6)	23·1 (16·0–29·5)	-1·6 (-2·3–-0·8)	-1·6 (-2·5–-0·8)	-0·5 (-1·4–0·5)
Diet high in trans fatty acids	57·8 (49·2–65·9)	63·7 (56·0–70·9)	63·1 (53·9–70·5)	0·0 (0·0–0·0)	-65·1 (-65·5–-64·6)	-96·5 (-97·0–-95·9)	-184·2 (-185·2–-182·8)
Diet high in sodium	23·5 (0·5–69·8)	28·4 (1·7–77·0)	31·2 (3·2–75·8)	31·6 (3·3–77·8)	1·0 (0·2–6·7)	0·5 (-0·1–4·3)	0·1 (-2·2–2·0)
Intimate partner violence	33·7 (17·1–42·8)	33·8 (17·6–41·8)	31·0 (20·7–37·5)	28·9 (17·6–37·7)	-0·5 (-1·8–0·7)	-0·7 (-2·4–0·8)	-0·6 (-2·7–1·2)
Childhood sexual abuse and bullying	8·7 (5·6–13·9)	10·2 (6·5–16·2)	11·6 (7·3–18·6)	9·0 (6·0–14·2)	0·1 (-0·5–0·7)	-0·6 (-1·5–0·2)	-2·3 (-3·9–-0·9)
Childhood sexual abuse	8·0 (6·6–9·5)	8·2 (7·1–9·5)	9·3 (8·4–10·2)	8·7 (7·2–10·2)	0·3 (-0·6–1·1)	0·3 (-0·9–1·3)	-0·6 (-2·1–0·8)
Bullying victimization	7·9 (3·5–15·8)	9·8 (4·6–19·1)	11·2 (5·1–21·8)	7·7 (3·4–15·3)	-0·1 (-0·8–0·6)	-1·2 (-2·2–-0·2)	-3·4 (-5·3–-1·6)
Unsafe sex	--	--	--	--	--	--	--
Low physical activity	23·0 (17·9–29·2)	22·8 (17·9–28·1)	22·2 (17·5–27·3)	24·2 (18·4–30·2)	0·2 (-0·6–0·9)	0·3 (-0·9–1·4)	0·8 (-0·8–2·2)
**Metabolic risks**	**26·7 (23·4–29·9)**	**32·8 (28·9–35·6)**	**35·8 (31·2–38·7)**	**38·5 (33·7–41·4)**	**1·2 (0·9–1·5)**	**0·8 (0·5–1·1)**	**0·7 (0·3–1·1)**
High fasting plasma glucose	13·4 (10·2–16·4)	17·8 (13·5–21·2)	22·9 (17·5–27·4)	25·8 (19·5–30·6)	2·1 (1·5–2·7)	1·8 (1·0–2·6)	1·1 (0·0–2·3)
High LDL cholesterol	63·5 (44·6–86·5)	53·8 (37·2–74·0)	48·3 (32·5–66·8)	46·6 (31·6–65·2)	-1·0 (-1·2–-0·8)	-0·7 (-0·9–-0·5)	-0·3 (-0·6–-0·1)
High systolic blood pressure	29·7 (19·9–41·5)	25·7 (18·1–34·9)	19·1 (12·6–27·4)	26·3 (17·0–37·7)	-0·4 (-1·4–0·5)	0·1 (-1·1–1·2)	2·9 (0·6–5·1)
High body-mass index	30·2 (25·4–34·6)	37·5 (32·1–41·5)	41·0 (34·9–44·4)	43·8 (37·3–47·3)	1·2 (0·9–1·5)	0·7 (0·4–1·0)	0·6 (0·2–1·0)
Low bone mineral density	21·6 (15·6–28·4)	19·5 (14·0–26·7)	20·3 (14·3–27·7)	22·3 (16·3–29·1)	0·1 (-0·4–0·6)	0·6 (0·0–1·4)	0·9 (-0·5–2·2)
Kidney dysfunction	3·0 (2·4–3·9)	3·1 (2·5–4·0)	3·0 (2·5–4·0)	3·2 (2·6–4·2)	0·2 (0·1–0·3)	0·1 (0·0–0·3)	0·4 (0·1–0·6)

North Dakota

Risk Names	SEV 1990	SEV 2000	SEV 2010	SEV 2021	Annualised rate of change 1990 to 2021	Annualised rate of change 2000 to 2021	Annualised rate of change 2010 to 2021
**All risk factors**	**26·0 (23·2–29·3)**	**25·5 (22·9–28·6)**	**25·6 (22·7–28·6)**	**26·0 (23·1–29·2)**	**0·0 (-0·3–0·3)**	**0·1 (-0·3–0·5)**	**0·2 (-0·4–0·7)**
**Environmental/occupational risks**	**13·8 (9·1–22·2)**	**13·3 (8·5–20·7)**	**12·0 (7·5–19·2)**	**11·0 (6·8–17·8)**	**-0·7 (-1·4–-0·2)**	**-0·9 (-1·4–-0·5)**	**-0·8 (-1·5–-0·2)**
Unsafe water, sanitation, and handwashing	1·8 (0·9–2·9)	1·6 (0·7–2·6)	1·4 (0·5–2·3)	1·0 (0·3–1·8)	-1·8 (-4·1–0·2)	-2·1 (-5·1–0·6)	-2·5 (-6·7–1·7)
Unsafe water source	2·6 (0·9–5·8)	2·3 (0·8–4·8)	1·0 (0·4–2·2)	0·7 (0·3–1·5)	-4·0 (-6·8–-1·1)	-5·3 (-8·9–-1·5)	-2·9 (-8·1–2·6)
Unsafe sanitation	5·8 (3·1–10·0)	4·4 (2·3–7·6)	3·7 (2·0–6·4)	2·4 (1·2–4·0)	-2·9 (-5·2–-0·5)	-2·9 (-5·8–-0·1)	-4·1 (-7·5–-0·9)
No access to handwashing facility	1·5 (0·4–3·1)	1·6 (0·5–3·0)	1·6 (0·6–3·0)	1·4 (0·5–2·6)	-0·4 (-3·4–2·3)	-0·6 (-4·6–3·5)	-1·7 (-7·3–3·7)
Air pollution	15·5 (2·1–25·8)	13·9 (2·7–23·6)	8·4 (1·4–18·1)	9·2 (0·6–19·1)	-1·7 (-6·2–-0·4)	-2·0 (-7·6–-0·8)	0·8 (-8·9–3·1)
Particulate matter pollution	5·6 (0·4–13·0)	4·9 (2·0–8·3)	3·2 (0·8–6·2)	1·7 (0·2–4·3)	-3·8 (-10·6–1·9)	-5·1 (-14·1–-2·5)	-5·7 (-20·1–-0·7)
Ambient particulate matter pollution	8·5 (0·6–19·8)	7·5 (3·0–12·6)	4·8 (1·1–9·2)	2·6 (0·2–6·4)	-3·9 (-10·6–2·0)	-5·1 (-14·1–-2·5)	-5·7 (-20·2–-0·6)
Household air pollution from solid fuels	0·0 (0·0–0·0)	0·0 (0·0–0·0)	0·0 (0·0–0·0)	0·0 (0·0–0·0)	-6·6 (-57·4–-8·6)	-8·4 (-70·6–-11·8)	-9·9 (-81·2–-12·4)
Ambient ozone pollution	8·4 (4·3–14·2)	11·6 (6·5–17·4)	11·0 (5·9–16·3)	8·8 (4·4–14·7)	0·2 (-1·3–1·5)	-1·3 (-3·4–0·3)	-2·0 (-5·8–1·6)
Ambient nitrogen dioxide pollution	60·3 (0·0–100·0)	54·1 (0·0–100·0)	31·4 (0·0–83·3)	43·4 (0·0–99·9)	-1·1 (-2·3–0·0)	-1·1 (-2·2–0·0)	2·9 (0·0–7·9)
Non-optimal temperature	31·1 (23·6–40·5)	28·1 (21·9–36·1)	27·9 (21·4–36·3)	32·2 (24·2–42·3)	0·1 (-0·1–0·5)	0·6 (0·2–1·2)	1·3 (0·5–2·2)
High temperature	23·8 (13·1–35·7)	22·1 (12·1–33·9)	24·1 (14·1–36·2)	33·5 (20·7–46·8)	1·1 (0·6–1·7)	2·0 (1·1–3·1)	3·0 (1·7–4·2)
Low temperature	29·8 (24·5–34·9)	26·8 (22·4–32·5)	26·4 (21·7–32·1)	29·0 (24·1–34·4)	-0·1 (-0·2–0·1)	0·4 (0·1–0·6)	0·9 (0·4–1·3)
Other environmental risks	27·8 (13·9–48·5)	26·3 (13·1–46·9)	24·2 (11·9–44·5)	21·9 (9·7–41·9)	-0·8 (-1·6–0·0)	-0·9 (-1·8–0·0)	-0·9 (-2·2–0·0)
Residential radon	35·9 (0·0–100·0)	35·9 (0·0–100·0)	35·9 (0·0–100·0)	35·9 (0·0–100·0)	0·0 (0·0–0·0)	0·0 (0·0–0·0)	0·0 (0·0–0·0)
Lead exposure	24·1 (0·0–30·6)	21·9 (0·0–27·3)	18·9 (0·0–23·9)	15·5 (0·0–19·5)	-1·4 (-1·9–0·0)	-1·6 (-2·1–0·0)	-1·8 (-2·5–0·0)
Occupational risks	2·9 (2·4–3·9)	3·1 (2·5–4·1)	3·1 (2·5–4·1)	3·1 (2·5–4·1)	0·1 (-0·1–0·4)	-0·1 (-0·4–0·2)	0·0 (-0·5–0·4)
Occupational carcinogens	1·0 (0·5–1·9)	1·1 (0·5–2·2)	1·1 (0·5–2·2)	1·1 (0·5–2·3)	0·4 (0·1–0·6)	0·1 (-0·3–0·4)	0·1 (-0·4–0·7)
Occupational exposure to asbestos	4·9 (4·4–5·4)	4·9 (4·4–5·3)	4·6 (4·0–5·0)	4·5 (3·6–5·4)	-0·3 (-1·0–0·4)	-0·4 (-1·5–0·7)	-0·2 (-2·3–1·7)
Occupational exposure to arsenic	0·4 (0·0–1·1)	0·5 (0·0–1·2)	0·5 (0·0–1·2)	0·5 (0·0–1·2)	0·3 (-0·1–0·7)	0·0 (-0·5–0·6)	0·1 (-0·7–0·9)
Occupational exposure to benzene	1·2 (0·1–3·4)	1·3 (0·1–3·8)	1·4 (0·1–4·0)	1·4 (0·1–4·2)	0·5 (0·2–0·8)	0·2 (-0·2–0·6)	0·2 (-0·5–0·8)
Occupational exposure to beryllium	0·0 (0·0–0·0)	0·0 (0·0–0·0)	0·0 (0·0–0·0)	0·0 (0·0–0·0)	0·4 (0·2–0·6)	0·2 (-0·1–0·5)	0·3 (-0·1–0·7)
Occupational exposure to cadmium	0·1 (0·1–0·1)	0·1 (0·1–0·1)	0·1 (0·1–0·1)	0·1 (0·1–0·1)	0·3 (-0·1–0·7)	0·1 (-0·4–0·5)	0·1 (-0·6–0·8)
Occupational exposure to chromium	0·1 (0·1–0·1)	0·1 (0·1–0·2)	0·1 (0·1–0·2)	0·2 (0·1–0·2)	0·4 (0·1–0·7)	0·1 (-0·3–0·5)	0·2 (-0·4–0·8)
Occupational exposure to diesel engine exhaust	0·8 (0·7–0·8)	0·8 (0·7–0·9)	0·8 (0·8–0·9)	0·9 (0·8–0·9)	0·4 (0·1–0·8)	0·4 (-0·1–0·9)	0·5 (-0·1–1·2)
Occupational exposure to formaldehyde	0·3 (0·2–0·3)	0·3 (0·3–0·3)	0·3 (0·3–0·3)	0·3 (0·3–0·3)	0·3 (-0·1–0·7)	0·0 (-0·5–0·5)	0·0 (-0·7–0·7)
Occupational exposure to nickel	0·4 (0·0–1·5)	0·4 (0·0–1·6)	0·4 (0·0–1·5)	0·4 (0·0–1·6)	0·1 (-0·4–0·6)	-0·1 (-0·7–0·6)	0·0 (-0·9–0·9)
Occupational exposure to polycyclic aromatic hydrocarbons	0·3 (0·3–0·3)	0·3 (0·3–0·3)	0·3 (0·3–0·3)	0·3 (0·3–0·3)	0·4 (0·1–0·7)	0·1 (-0·3–0·5)	0·1 (-0·4–0·7)
Occupational exposure to silica	3·5 (0·5–11·2)	3·5 (0·5–11·3)	3·5 (0·5–11·2)	3·5 (0·6–11·0)	0·0 (-0·4–0·4)	-0·1 (-0·6–0·5)	0·1 (-0·7–0·9)
Occupational exposure to sulphuric acid	0·7 (0·1–2·3)	0·8 (0·2–2·5)	0·8 (0·2–2·5)	0·8 (0·2–2·5)	0·3 (-0·1–0·7)	0·1 (-0·4–0·5)	0·1 (-0·6–0·8)
Occupational exposure to trichloroethylene	0·1 (0·1–0·1)	0·1 (0·1–0·1)	0·1 (0·1–0·1)	0·1 (0·1–0·1)	0·4 (0·2–0·7)	0·2 (-0·1–0·6)	0·3 (-0·2–0·9)
Occupational asthmagens	16·4 (14·4–18·7)	17·8 (15·6–20·2)	17·4 (15·3–19·8)	17·7 (15·4–20·2)	0·2 (-0·1–0·6)	0·0 (-0·5–0·4)	0·1 (-0·5–0·8)
Occupational particulate matter, gases, and fumes	6·9 (5·3–9·1)	6·8 (5·3–9·0)	6·5 (5·0–8·6)	6·1 (4·7–8·0)	-0·4 (-0·6–-0·2)	-0·5 (-0·8–-0·2)	-0·6 (-0·9–-0·3)
Occupational noise	7·6 (7·0–8·3)	7·7 (7·2–8·3)	7·6 (7·2–8·2)	7·4 (7·0–8·0)	-0·1 (-0·2–0·1)	-0·1 (-0·3–0·0)	-0·2 (-0·4–-0·1)
Occupational injuries	--	--	--	--	--	--	--
Occupational ergonomic factors	9·6 (8·1–11·5)	9·6 (8·2–11·4)	9·0 (7·6–10·7)	8·9 (7·4–10·8)	-0·3 (-0·7–0·2)	-0·4 (-1·0–0·1)	-0·1 (-0·8–0·6)
**Behavioural risks**	**30·2 (27·2–33·9)**	**28·8 (25·9–32·6)**	**27·0 (23·6–30·9)**	**26·0 (22·5–29·7)**	**-0·5 (-0·9–-0·1)**	**-0·5 (-1·0–-0·1)**	**-0·4 (-1·1–0·3)**
Child and maternal malnutrition	10·0 (6·5–13·9)	8·6 (6·0–11·9)	8·9 (6·1–12·2)	9·1 (6·1–12·5)	-0·3 (-1·1–0·6)	0·2 (-1·0–1·3)	0·2 (-1·8–2·2)
Suboptimal breastfeeding	68·7 (66·1–71·3)	66·6 (64·0–69·4)	62·7 (60·1–65·8)	62·1 (59·2–65·5)	-0·3 (-0·5–-0·2)	-0·3 (-0·5–-0·1)	-0·1 (-0·4–0·2)
Non-exclusive breastfeeding	63·9 (56·3–71·8)	59·3 (52·6–66·7)	48·6 (42·0–55·0)	46·2 (39·1–53·9)	-1·0 (-1·6–-0·5)	-1·2 (-1·9–-0·5)	-0·5 (-1·5–0·5)
Discontinued breastfeeding	88·6 (86·3–91·1)	86·2 (83·3–89·0)	84·2 (81·0–87·1)	84·3 (80·6–87·7)	-0·2 (-0·3–0·0)	-0·1 (-0·3–0·1)	0·0 (-0·3–0·4)
Child growth failure	1·2 (0·4–2·9)	1·2 (0·4–2·6)	1·0 (0·3–2·4)	0·9 (0·3–2·0)	-1·0 (-1·4–-0·6)	-1·2 (-1·8–-0·6)	-1·0 (-1·9–-0·3)
Child underweight	2·6 (1·7–3·6)	2·5 (1·6–3·3)	2·1 (1·4–2·9)	1·9 (1·2–2·6)	-1·0 (-1·5–-0·5)	-1·2 (-2·0–-0·6)	-1·0 (-2·1–0·0)
Child wasting	0·8 (0·5–1·3)	0·8 (0·5–1·2)	0·8 (0·5–1·3)	0·8 (0·5–1·2)	-0·4 (-0·7–0·0)	-0·3 (-0·7–0·2)	-0·6 (-1·3–0·0)
Child stunting	3·6 (2·8–4·5)	3·4 (2·7–4·2)	3·0 (2·3–3·7)	2·6 (2·0–3·3)	-1·0 (-1·6–-0·4)	-1·2 (-1·9–-0·4)	-1·2 (-2·3–0·0)
Low birth weight and short gestation	15·0 (12·9–17·0)	15·7 (13·7–17·5)	17·0 (14·9–19·0)	19·6 (17·1–22·2)	0·9 (0·6–1·1)	1·1 (0·7–1·5)	1·3 (0·6–2·0)
Short gestation	27·7 (24·3–31·3)	28·9 (25·7–32·8)	30·1 (26·5–34·1)	33·1 (29·4–37·8)	0·6 (0·3–0·9)	0·6 (0·2–1·1)	0·9 (0·0–1·7)
Low birth weight	12·1 (10·9–13·3)	12·4 (11·3–13·5)	13·6 (12·4–14·9)	15·7 (14·3–17·3)	0·8 (0·6–1·1)	1·1 (0·8–1·5)	1·3 (0·6–2·0)

Iron deficiency	3·5 (2·3–4·7)	3·0 (2·2–4·0)	3·1 (2·3–4·1)	3·1 (2·2–4·3)	-0·3 (-1·1–0·5)	0·2 (-0·9–1·2)	0·1 (-1·7–1·9)
Vitamin A deficiency	1·0 (0·0–2·0)	1·2 (0·0–2·7)	0·9 (0·0–1·8)	0·4 (0·0–1·0)	-2·6 (-4·8–0·0)	-4·9 (-7·8–0·0)	-6·1 (-10·4–0·0)
Zinc deficiency	2·0 (0·0–8·7)	1·2 (0·0–5·2)	1·0 (0·0–4·2)	0·8 (0·0–3·5)	-2·8 (-47·8–0·2)	-1·7 (-33·8–7·0)	-1·2 (-6·3–30·4)
Tobacco	38·3 (36·7–39·8)	34·9 (33·5–36·2)	30·0 (28·9–31·1)	26·3 (24·5–28·0)	-1·2 (-1·4–-1·0)	-1·3 (-1·6–-1·0)	-1·2 (-1·7–-0·6)
Smoking	36·9 (34·6–39·2)	32·5 (30·4–34·5)	27·8 (26·4–29·6)	24·3 (22·2–26·7)	-1·3 (-1·6–-1·1)	-1·4 (-1·8–-1·0)	-1·2 (-1·9–-0·5)
Chewing tobacco	2·8 (2·3–3·5)	2·9 (2·3–3·6)	3·1 (2·3–4·2)	3·1 (2·3–4·2)	0·4 (-0·9–1·6)	0·4 (-1·3–2·0)	0·0 (-2·5–2·5)
Second-hand smoke	30·1 (28·4–31·3)	27·9 (26·1–29·0)	24·0 (22·4–25·0)	21·4 (19·2–23·1)	-1·1 (-1·4–-0·9)	-1·3 (-1·7–-0·9)	-1·0 (-1·7–-0·4)
High alcohol use	28·5 (20·6–38·7)	27·3 (19·7–38·1)	27·1 (19·0–38·3)	29·1 (20·5–39·6)	0·1 (-1·0–1·1)	0·3 (-1·1–1·6)	0·7 (-1·3–2·7)
Drug use	0·7 (0·4–1·3)	0·7 (0·5–1·1)	1·2 (1·0–1·4)	2·7 (2·1–3·5)	4·4 (1·8–6·8)	6·2 (3·6–8·2)	7·6 (5·0–9·4)
Dietary risks	37·3 (28·1–48·3)	39·5 (30·5–50·9)	41·2 (31·9–53·0)	41·7 (32·0–53·7)	0·4 (0·1–0·7)	0·3 (0·0–0·6)	0·1 (-0·3–0·6)
Diet low in fruits	38·5 (33·4–42·1)	35·4 (31·4–38·6)	34·5 (29·8–37·5)	35·3 (29·9–39·5)	-0·3 (-0·7–0·1)	0·0 (-0·6–0·5)	0·2 (-0·7–1·0)
Diet low in vegetables	23·3 (13·3–31·3)	21·6 (13·1–28·8)	21·5 (12·6–27·8)	19·8 (11·8–26·4)	-0·5 (-1·1–0·0)	-0·4 (-1·1–0·4)	-0·8 (-2·0–0·4)
Diet low in legumes	38·9 (0·0–48·4)	39·4 (0·0–48·9)	42·4 (0·0–52·7)	41·1 (0·0–51·3)	0·2 (-0·1–0·5)	0·2 (-0·2–0·6)	-0·3 (-0·9–0·4)
Diet low in whole grains	46·1 (38·7–52·6)	49·6 (40·9–56·5)	51·0 (42·5–58·5)	52·1 (42·8–60·0)	0·4 (0·1–0·7)	0·2 (-0·2–0·6)	0·2 (-0·4–0·7)
Diet low in nuts and seeds	14·1 (10·4–18·2)	8·7 (5·8–12·4)	5·3 (3·1–8·1)	4·2 (2·2–7·0)	-3·9 (-5·9–-2·1)	-3·4 (-6·3–-0·8)	-2·1 (-6·6–2·0)
Diet low in milk	37·2 (32·8–41·8)	34·7 (30·4–39·5)	31·3 (26·9–35·6)	28·2 (23·6–32·8)	-0·9 (-1·4–-0·4)	-1·0 (-1·8–-0·4)	-0·9 (-2·0–0·0)
Diet high in red meat	55·4 (0·0–75·1)	55·5 (0·0–74·8)	55·9 (0·0–75·1)	57·4 (0·0–76·9)	0·1 (-0·3–0·5)	0·2 (-0·4–0·7)	0·2 (-0·6–1·1)
Diet high in processed meat	47·9 (37·6–54·1)	59·1 (47·0–66·2)	66·5 (53·2–74·3)	67·0 (54·8–75·8)	1·1 (0·6–1·5)	0·6 (0·1–1·1)	0·1 (-0·7–0·8)
Diet high in sugar-sweetened beverages	35·3 (26·7–44·0)	49·2 (39·5–58·2)	57·1 (45·2–67·9)	62·1 (49·8–73·0)	1·8 (1·0–2·6)	1·1 (0·2–2·0)	0·8 (-0·4–2·0)
Diet low in fibre	41·1 (22·3–48·4)	31·8 (17·1–38·3)	26·0 (14·3–32·5)	19·6 (11·4–25·9)	-2·4 (-3·4–-1·5)	-2·3 (-3·7–-1·0)	-2·6 (-4·7–-0·7)
Diet low in calcium	5·9 (4·7–7·3)	5·0 (4·0–6·2)	4·9 (3·9–6·1)	4·3 (3·4–5·4)	-1·0 (-1·2–-0·8)	-0·6 (-0·9–-0·4)	-1·0 (-1·4–-0·7)
Diet low in seafood omega-3 fatty acids	38·4 (29·3–48·8)	45·5 (35·3–56·4)	46·5 (35·8–56·6)	37·4 (27·4–47·3)	-0·1 (-0·6–0·4)	-0·9 (-1·5–-0·4)	-2·0 (-2·9–-1·2)
Diet low in omega-6 polyunsaturated fatty acids	37·8 (23·8–48·3)	33·4 (22·1–42·7)	23·3 (16·1–29·9)	19·2 (13·4–25·2)	-2·2 (-3·0–-1·3)	-2·6 (-3·7–-1·5)	-1·7 (-2·9–-0·7)
Diet high in trans fatty acids	71·4 (62·0–78·9)	71·8 (64·6–79·6)	63·4 (55·1–70·8)	0·0 (0·0–0·0)	-65·8 (-66·1–-65·3)	-97·1 (-97·6–-96·6)	-184·3 (-185·3–-183·0)
Diet high in sodium	23·3 (0·6–68·2)	28·0 (2·0–74·3)	31·2 (3·5–73·8)	31·6 (3·2–77·1)	1·0 (0·2–6·1)	0·6 (-0·1–3·7)	0·1 (-2·2–1·8)
Intimate partner violence	21·3 (7·7–27·9)	21·1 (8·8–27·3)	18·8 (11·2–23·8)	18·3 (9·0–25·7)	-0·5 (-2·1–1·0)	-0·7 (-2·9–1·3)	-0·2 (-3·1–2·0)
Childhood sexual abuse and bullying	8·9 (5·6–14·2)	10·8 (6·9–17·0)	12·1 (7·7–19·2)	10·3 (6·7–16·0)	0·5 (0·0–1·1)	-0·2 (-1·0–0·6)	-1·5 (-2·9–0·1)
Childhood sexual abuse	8·3 (6·8–9·9)	9·4 (8·0–10·7)	10·6 (9·2–12·0)	10·0 (8·2–12·2)	0·6 (-0·2–1·5)	0·3 (-0·6–1·3)	-0·5 (-1·9–0·9)
Bullying victimization	8·0 (3·5–16·0)	10·0 (4·6–19·5)	11·2 (5·1–21·8)	9·0 (3·9–16·7)	0·4 (-0·3–1·1)	-0·5 (-1·5–0·5)	-2·1 (-3·9–-0·1)
Unsafe sex	--	--	--	--	--	--	--
Low physical activity	20·8 (15·8–26·6)	20·1 (15·5–24·8)	21·1 (16·4–26·1)	24·6 (18·5–31·1)	0·5 (-0·4–1·5)	1·0 (-0·2–2·0)	1·4 (-0·2–3·1)
**Metabolic risks**	**25·7 (22·3–28·9)**	**31·2 (27·1–34·3)**	**35·4 (31·1–38·6)**	**39·6 (34·5–42·8)**	**1·4 (1·1–1·7)**	**1·1 (0·8–1·5)**	**1·0 (0·5–1·5)**
High fasting plasma glucose	13·2 (9·8–16·0)	17·3 (13·2–21·3)	21·9 (16·4–27·0)	27·1 (20·3–33·0)	2·3 (1·6–3·0)	2·1 (1·2–3·1)	1·9 (0·6–3·4)
High LDL cholesterol	63·5 (44·8–86·2)	53·8 (37·2–74·1)	48·1 (32·7–66·7)	47·0 (31·7–65·6)	-1·0 (-1·2–-0·8)	-0·6 (-0·9–-0·4)	-0·2 (-0·4–0·1)
High systolic blood pressure	29·0 (20·0–39·6)	24·7 (17·4–34·0)	18·1 (11·8–26·4)	25·7 (16·2–37·1)	-0·4 (-1·3–0·4)	0·2 (-1·0–1·3)	3·2 (0·9–5·7)
High body-mass index	29·5 (24·4–34·0)	36·0 (30·2–40·2)	40·9 (34·6–45·2)	44·8 (38·0–49·2)	1·3 (1·0–1·6)	1·0 (0·7–1·3)	0·8 (0·4–1·3)
Low bone mineral density	21·2 (15·3–28·1)	19·3 (13·8–25·8)	20·0 (14·0–26·7)	21·5 (16·2–28·1)	0·1 (-0·4–0·5)	0·5 (-0·2–1·3)	0·7 (-0·6–2·1)
Kidney dysfunction	2·8 (2·3–3·8)	2·9 (2·4–3·8)	2·9 (2·3–3·8)	3·0 (2·4–4·0)	0·2 (0·1–0·3)	0·1 (0·0–0·3)	0·4 (0·1–0·7)

Ohio

Risk Names	SEV 1990	SEV 2000	SEV 2010	SEV 2021	Annualised rate of change 1990 to 2021	Annualised rate of change 2000 to 2021	Annualised rate of change 2010 to 2021
**All risk factors**	**29·0 (25·8–32·4)**	**28·8 (25·8–31·9)**	**29·3 (26·2–32·8)**	**29·3 (25·8–32·7)**	**0·0 (-0·3–0·3)**	**0·1 (-0·3–0·4)**	**0·0 (-0·6–0·5)**
**Environmental/occupational risks**	**20·0 (13·3–30·5)**	**19·4 (13·4–30·1)**	**16·7 (11·3–26·9)**	**14·5 (9·4–23·6)**	**-1·0 (-1·8–-0·3)**	**-1·4 (-1·9–-0·8)**	**-1·3 (-1·8–-0·6)**
Unsafe water, sanitation, and handwashing	2·0 (1·0–3·2)	1·8 (0·9–2·9)	1·4 (0·5–2·5)	1·2 (0·4–2·0)	-1·7 (-3·9–0·4)	-2·0 (-4·8–0·8)	-1·7 (-5·5–2·2)
Unsafe water source	2·8 (1·1–5·9)	2·5 (1·0–5·2)	1·0 (0·4–2·1)	0·8 (0·4–1·6)	-3·8 (-6·6–-0·9)	-5·2 (-8·7–-1·3)	-1·9 (-8·2–3·1)
Unsafe sanitation	6·4 (3·6–10·2)	5·0 (2·7–8·2)	3·9 (2·1–6·6)	3·0 (1·6–5·1)	-2·4 (-4·6–-0·3)	-2·4 (-4·9–0·3)	-2·3 (-5·5–1·0)
No access to handwashing facility	1·7 (0·5–3·2)	1·7 (0·5–3·2)	1·7 (0·6–3·3)	1·5 (0·5–2·9)	-0·4 (-3·4–2·6)	-0·6 (-4·3–3·3)	-1·4 (-6·8–3·9)
Air pollution	28·0 (9·6–40·0)	26·0 (10·8–33·0)	14·2 (6·2–25·0)	9·3 (3·5–18·2)	-3·6 (-6·0–-1·4)	-4·9 (-7·8–-2·5)	-3·9 (-6·7–-2·3)
Particulate matter pollution	15·9 (6·5–26·6)	14·3 (10·6–18·6)	9·1 (6·1–12·8)	6·0 (3·2–9·1)	-3·2 (-5·3–-0·9)	-4·2 (-6·0–-3·1)	-3·9 (-6·5–-2·4)
Ambient particulate matter pollution	24·8 (10·2–42·1)	22·1 (17·0–27·7)	14·0 (9·6–19·1)	9·1 (5·0–13·6)	-3·2 (-5·4–-0·9)	-4·2 (-6·1–-3·2)	-3·9 (-6·6–-2·4)
Household air pollution from solid fuels	0·0 (0·0–0·0)	0·0 (0·0–0·0)	0·0 (0·0–0·0)	0·0 (0·0–0·0)	-2·3 (-27·9–-5·9)	-3·1 (-24·2–-4·4)	0·5 (-22·8–-4·6)
Ambient ozone pollution	26·8 (20·7–34·8)	27·3 (21·0–36·4)	19·3 (13·6–27·1)	14·5 (9·4–22·1)	-2·0 (-2·6–-1·4)	-3·0 (-4·0–-2·3)	-2·6 (-3·9–-1·7)
Ambient nitrogen dioxide pollution	78·8 (0·0–100·0)	75·0 (0·0–100·0)	34·2 (0·0–89·9)	22·0 (0·0–69·7)	-4·1 (-13·0–0·0)	-5·8 (-18·1–0·0)	-4·0 (-14·5–0·0)
Non-optimal temperature	30·3 (23·3–41·7)	30·1 (23·8–40·6)	35·3 (26·9–47·3)	32·4 (24·6–44·5)	0·2 (-0·1–0·5)	0·3 (0·0–0·8)	-0·8 (-1·4–-0·5)
High temperature	24·4 (14·3–35·3)	20·8 (10·0–32·7)	34·6 (21·7–46·6)	28·3 (17·2–39·7)	0·5 (0·0–0·9)	1·5 (0·6–3·2)	-1·8 (-2·7–-1·0)
Low temperature	27·4 (24·0–30·8)	28·2 (25·6–31·1)	30·2 (27·7–33·2)	28·8 (25·9–32·1)	0·2 (0·0–0·3)	0·1 (-0·1–0·3)	-0·4 (-0·8–-0·1)
Other environmental risks	29·0 (10·9–47·9)	27·1 (11·1–45·1)	24·5 (10·8–42·0)	21·5 (10·0–39·1)	-1·0 (-1·7–0·0)	-1·1 (-1·9–0·0)	-1·2 (-2·2–0·0)
Residential radon	29·7 (0·0–85·1)	29·7 (0·0–85·1)	29·7 (0·0–85·1)	29·7 (0·0–85·1)	0·0 (0·0–0·0)	0·0 (0·0–0·0)	0·0 (0·0–0·0)
Lead exposure	28·6 (0·0–35·6)	25·9 (0·0–32·0)	22·1 (0·0–27·5)	17·8 (0·0–22·1)	-1·5 (-2·0–0·0)	-1·8 (-2·3–0·0)	-2·0 (-2·8–0·0)
Occupational risks	3·1 (2·6–4·0)	3·1 (2·6–4·0)	2·9 (2·4–3·8)	2·9 (2·4–3·8)	-0·2 (-0·5–0·0)	-0·4 (-0·7–-0·1)	0·0 (-0·4–0·5)
Occupational carcinogens	1·0 (0·5–1·9)	1·1 (0·5–2·0)	1·0 (0·5–1·9)	1·0 (0·5–2·0)	0·1 (-0·2–0·4)	-0·1 (-0·6–0·2)	0·2 (-0·4–0·8)
Occupational exposure to asbestos	4·5 (4·0–5·0)	5·0 (4·6–5·3)	4·4 (4·0–4·8)	4·4 (3·5–5·4)	0·0 (-0·8–0·6)	-0·5 (-1·7–0·5)	0·1 (-2·2–1·9)
Occupational exposure to arsenic	0·6 (0·0–1·3)	0·6 (0·0–1·4)	0·5 (0·0–1·3)	0·5 (0·0–1·3)	-0·4 (-0·8–0·0)	-0·5 (-1·1–0·0)	0·0 (-0·9–0·9)
Occupational exposure to benzene	1·1 (0·1–3·1)	1·2 (0·1–3·5)	1·1 (0·1–3·4)	1·2 (0·1–3·5)	0·4 (0·0–0·7)	0·1 (-0·3–0·5)	0·4 (-0·2–1·0)
Occupational exposure to beryllium	0·0 (0·0–0·0)	0·0 (0·0–0·0)	0·0 (0·0–0·0)	0·0 (0·0–0·0)	0·2 (-0·1–0·4)	0·0 (-0·3–0·4)	0·4 (0·0–0·9)
Occupational exposure to cadmium	0·1 (0·1–0·1)	0·1 (0·1–0·1)	0·1 (0·1–0·1)	0·1 (0·1–0·1)	-0·3 (-0·7–0·1)	-0·4 (-1·0–0·1)	0·0 (-0·7–0·8)
Occupational exposure to chromium	0·2 (0·2–0·2)	0·2 (0·2–0·2)	0·2 (0·1–0·2)	0·2 (0·1–0·2)	-0·2 (-0·5–0·1)	-0·3 (-0·8–0·1)	0·1 (-0·5–0·8)
Occupational exposure to diesel engine exhaust	0·7 (0·6–0·7)	0·7 (0·6–0·7)	0·7 (0·6–0·7)	0·7 (0·7–0·8)	0·3 (0·0–0·7)	0·3 (-0·2–0·8)	0·7 (0·0–1·4)
Occupational exposure to formaldehyde	0·3 (0·3–0·4)	0·3 (0·3–0·4)	0·3 (0·3–0·3)	0·3 (0·3–0·3)	-0·4 (-0·7–0·0)	-0·5 (-1·1–0·0)	-0·1 (-0·8–0·7)
Occupational exposure to nickel	0·5 (0·0–1·9)	0·5 (0·0–1·8)	0·4 (0·0–1·6)	0·4 (0·0–1·5)	-0·5 (-1·0–0·0)	-0·6 (-1·3–0·1)	-0·1 (-1·0–1·0)
Occupational exposure to polycyclic aromatic hydrocarbons	0·3 (0·3–0·4)	0·3 (0·3–0·4)	0·3 (0·3–0·3)	0·3 (0·3–0·3)	-0·1 (-0·4–0·2)	-0·3 (-0·7–0·1)	0·1 (-0·5–0·7)
Occupational exposure to silica	4·0 (0·7–12·9)	3·9 (0·6–12·4)	3·5 (0·6–11·1)	3·5 (0·6–11·1)	-0·5 (-0·9–0·0)	-0·5 (-1·1–0·1)	0·1 (-0·9–1·0)
Occupational exposure to sulphuric acid	0·9 (0·2–2·7)	0·9 (0·2–2·9)	0·8 (0·2–2·6)	0·8 (0·2–2·6)	-0·3 (-0·7–0·1)	-0·4 (-1·0–0·1)	0·1 (-0·7–0·9)
Occupational exposure to trichloroethylene	0·1 (0·1–0·1)	0·1 (0·1–0·1)	0·1 (0·1–0·1)	0·1 (0·1–0·1)	0·0 (-0·3–0·2)	-0·1 (-0·5–0·2)	0·3 (-0·2–0·9)
Occupational asthmagens	18·5 (16·3–21·2)	18·7 (16·5–21·2)	16·9 (14·9–19·1)	17·1 (15·0–19·6)	-0·3 (-0·6–0·1)	-0·4 (-0·9–0·0)	0·1 (-0·5–0·8)
Occupational particulate matter, gases, and fumes	7·3 (5·6–9·8)	7·1 (5·5–9·4)	6·7 (5·1–8·8)	6·1 (4·7–8·0)	-0·6 (-0·8–-0·3)	-0·7 (-1·0–-0·4)	-0·8 (-1·1–-0·4)
Occupational noise	7·0 (6·5–7·7)	7·1 (6·5–7·7)	7·0 (6·5–7·6)	6·7 (6·3–7·3)	-0·2 (-0·3–0·0)	-0·3 (-0·5–-0·1)	-0·3 (-0·6–-0·2)
Occupational injuries	--	--	--	--	--	--	--
Occupational ergonomic factors	8·1 (6·8–9·8)	8·1 (6·8–9·9)	7·3 (6·2–9·0)	7·5 (6·3–9·1)	-0·2 (-0·7–0·2)	-0·4 (-0·9–0·2)	0·3 (-0·5–1·0)
**Behavioural risks**	**32·4 (28·7–36·0)**	**30·8 (27·4–34·9)**	**29·3 (25·8–33·3)**	**27·5 (24·0–31·6)**	**-0·5 (-0·9–-0·2)**	**-0·5 (-1·0–-0·1)**	**-0·6 (-1·3–0·1)**
Child and maternal malnutrition	10·2 (6·9–14·5)	8·9 (6·1–12·3)	9·2 (6·4–12·6)	9·3 (6·4–13·1)	-0·3 (-1·2–0·5)	0·2 (-1·0–1·4)	0·2 (-1·5–1·8)
Suboptimal breastfeeding	69·0 (66·4–71·3)	66·8 (64·4–69·2)	62·9 (60·6–65·6)	62·2 (59·5–65·3)	-0·3 (-0·5–-0·2)	-0·3 (-0·5–-0·2)	-0·1 (-0·4–0·2)
Non-exclusive breastfeeding	64·5 (56·7–71·4)	59·7 (53·5–66·1)	48·6 (42·1–55·7)	46·4 (39·2–53·8)	-1·1 (-1·6–-0·6)	-1·2 (-1·8–-0·6)	-0·4 (-1·5–0·6)
Discontinued breastfeeding	88·8 (86·7–91·1)	86·5 (83·7–89·0)	84·5 (81·3–87·5)	84·6 (81·5–87·8)	-0·2 (-0·3–0·0)	-0·1 (-0·3–0·1)	0·0 (-0·3–0·3)
Child growth failure	1·3 (0·4–3·0)	1·2 (0·4–2·8)	1·1 (0·3–2·5)	1·0 (0·3–2·3)	-0·7 (-1·1–-0·3)	-0·8 (-1·3–-0·3)	-0·4 (-1·1–0·4)
Child underweight	2·7 (1·7–3·8)	2·6 (1·6–3·5)	2·2 (1·4–3·0)	2·2 (1·3–3·0)	-0·7 (-1·2–-0·2)	-0·8 (-1·5–-0·1)	-0·3 (-1·3–0·7)
Child wasting	0·9 (0·6–1·4)	0·8 (0·5–1·3)	0·8 (0·5–1·3)	0·8 (0·5–1·3)	-0·1 (-0·5–0·3)	0·1 (-0·4–0·6)	0·0 (-0·7–0·8)
Child stunting	3·8 (2·9–4·7)	3·5 (2·8–4·4)	3·1 (2·4–3·9)	2·9 (2·3–3·7)	-0·8 (-1·4–-0·3)	-0·9 (-1·7–-0·1)	-0·5 (-1·7–0·6)
Low birth weight and short gestation	21·2 (18·9–23·7)	22·8 (19·9–25·7)	24·5 (21·3–27·6)	24·8 (21·8–27·8)	0·5 (0·3–0·8)	0·4 (0·0–0·7)	0·1 (-0·6–0·9)
Short gestation	37·4 (33·5–41·8)	40·5 (36·2–44·8)	43·2 (38·7–48·7)	43·1 (38·6–47·9)	0·5 (0·2–0·7)	0·3 (-0·1–0·7)	0·0 (-0·8–0·9)
Low birth weight	16·6 (15·3–18·0)	17·5 (16·1–19·1)	18·5 (17·0–20·2)	18·9 (17·4–20·5)	0·4 (0·2–0·7)	0·4 (0·0–0·7)	0·2 (-0·5–0·9)

Iron deficiency	3·7 (2·6–5·1)	3·2 (2·3–4·3)	3·3 (2·4–4·4)	3·4 (2·4–4·6)	-0·3 (-1·1–0·4)	0·2 (-0·9–1·4)	0·1 (-1·4–1·6)
Vitamin A deficiency	1·0 (0·0–2·0)	1·4 (0·0–2·9)	0·9 (0·0–2·0)	0·6 (0·0–1·2)	-1·9 (-4·1–0·1)	-4·3 (-7·3–0·0)	-4·2 (-8·3–0·2)
Zinc deficiency	1·9 (0·0–8·1)	1·1 (0·0–4·6)	1·0 (0·0–4·5)	1·0 (0·0–4·4)	-2·0 (-43·4–0·1)	-0·4 (-2·3–1·3)	-0·2 (-8·1–7·3)
Tobacco	42·4 (40·8–43·8)	40·3 (38·8–41·6)	34·7 (33·5–35·8)	31·0 (29·3–33·1)	-1·0 (-1·2–-0·8)	-1·2 (-1·5–-1·0)	-1·0 (-1·6–-0·5)
Smoking	39·5 (37·2–41·6)	36·6 (34·5–38·4)	31·6 (30·0–33·3)	28·5 (26·1–31·0)	-1·1 (-1·4–-0·8)	-1·2 (-1·6–-0·8)	-0·9 (-1·7–-0·3)
Chewing tobacco	2·5 (2·0–3·1)	2·3 (1·9–2·8)	2·1 (1·6–2·7)	1·9 (1·4–2·5)	-0·8 (-1·9–0·2)	-0·8 (-2·4–0·7)	-0·7 (-3·3–1·6)
Second-hand smoke	34·1 (32·3–35·5)	32·9 (31·2–34·2)	28·1 (26·5–29·2)	25·1 (23·1–26·8)	-1·0 (-1·2–-0·8)	-1·3 (-1·7–-1·0)	-1·1 (-1·7–-0·4)
High alcohol use	27·2 (19·5–36·7)	25·7 (18·8–35·5)	26·7 (18·9–37·1)	26·7 (19·2–37·2)	-0·1 (-1·1–0·8)	0·2 (-1·3–1·5)	0·0 (-2·1–1·8)
Drug use	0·8 (0·3–1·6)	0·9 (0·6–1·4)	1·8 (1·6–2·2)	6·9 (5·0–8·8)	6·9 (3·6–10·3)	9·6 (6·1–12·2)	12·1 (8·4–14·3)
Dietary risks	36·3 (27·4–47·1)	37·6 (27·9–49·2)	39·0 (29·3–51·3)	39·2 (29·4–51·3)	0·2 (-0·1–0·6)	0·2 (-0·1–0·5)	0·0 (-0·5–0·5)
Diet low in fruits	38·5 (33·5–41·6)	35·5 (31·0–38·4)	35·1 (31·0–37·9)	36·1 (31·1–40·0)	-0·2 (-0·5–0·2)	0·1 (-0·4–0·6)	0·3 (-0·6–1·2)
Diet low in vegetables	18·1 (10·3–25·5)	11·9 (6·3–18·2)	10·6 (6·0–15·7)	10·6 (5·7–16·4)	-1·7 (-2·8–-0·9)	-0·5 (-1·9–0·8)	0·0 (-2·2–2·0)
Diet low in legumes	38·2 (0·0–47·6)	38·4 (0·0–47·9)	42·8 (0·0–52·9)	43·3 (0·0–55·5)	0·4 (0·0–0·7)	0·6 (0·0–1·0)	0·1 (-0·5–0·8)
Diet low in whole grains	45·5 (38·2–51·8)	48·6 (40·1–55·1)	50·2 (41·3–57·7)	50·9 (42·5–58·9)	0·4 (0·1–0·6)	0·2 (-0·1–0·6)	0·1 (-0·4–0·6)
Diet low in nuts and seeds	12·8 (9·1–16·6)	7·4 (4·7–10·9)	5·7 (3·4–8·5)	5·8 (3·6–9·1)	-2·5 (-4·2–-0·9)	-1·2 (-3·6–1·1)	0·2 (-3·2–3·6)
Diet low in milk	36·8 (32·8–40·7)	33·9 (29·3–38·3)	32·9 (28·5–37·6)	32·2 (27·2–37·3)	-0·4 (-0·9–0·1)	-0·2 (-0·9–0·3)	-0·2 (-1·1–0·6)
Diet high in red meat	51·9 (0·0–70·7)	52·3 (0·0–71·0)	52·0 (0·0–70·4)	52·2 (0·0–70·7)	0·0 (-0·4–0·4)	0·0 (-0·7–0·5)	0·0 (-1·0–0·9)
Diet high in processed meat	51·3 (40·5–57·3)	62·4 (49·6–69·6)	68·3 (54·8–76·1)	66·6 (52·9–75·0)	0·8 (0·4–1·3)	0·3 (-0·2–0·8)	-0·2 (-1·0–0·4)
Diet high in sugar-sweetened beverages	36·4 (28·1–44·9)	50·7 (40·5–59·1)	55·0 (44·4–64·6)	55·1 (43·6–64·6)	1·3 (0·6–2·2)	0·4 (-0·5–1·3)	0·0 (-1·3–1·3)
Diet low in fibre	39·4 (21·6–46·7)	29·7 (16·2–36·6)	27·0 (14·7–33·6)	23·3 (12·8–29·6)	-1·7 (-2·5–-0·9)	-1·2 (-2·4–0·0)	-1·4 (-3·2–0·3)
Diet low in calcium	5·7 (4·7–7·0)	4·8 (3·9–5·9)	5·2 (4·2–6·4)	5·1 (4·1–6·3)	-0·4 (-0·6–-0·1)	0·2 (0·0–0·5)	-0·2 (-0·6–0·1)
Diet low in seafood omega-3 fatty acids	36·6 (27·4–46·1)	43·5 (33·5–53·8)	47·6 (37·6–58·1)	42·1 (32·5–53·1)	0·4 (0·0–0·9)	-0·2 (-0·7–0·4)	-1·1 (-1·8–-0·4)
Diet low in omega-6 polyunsaturated fatty acids	35·9 (23·0–46·0)	31·4 (20·6–40·7)	24·1 (16·5–31·3)	22·1 (15·2–29·0)	-1·6 (-2·3–-0·8)	-1·7 (-2·6–-0·8)	-0·8 (-1·9–0·2)
Diet high in trans fatty acids	71·4 (64·0–78·6)	71·7 (63·2–79·0)	63·3 (54·8–70·6)	0·0 (0·0–0·0)	-65·8 (-66·1–-65·4)	-97·1 (-97·6–-96·5)	-184·2 (-185·2–-182·9)
Diet high in sodium	23·3 (0·6–70·5)	28·0 (1·8–75·6)	31·2 (3·2–75·7)	31·6 (3·0–78·5)	1·0 (0·2–6·3)	0·6 (-0·1–3·9)	0·1 (-2·6–1·5)
Intimate partner violence	26·7 (17·5–34·2)	26·6 (19·3–32·2)	24·0 (15·5–29·7)	23·6 (12·4–31·8)	-0·4 (-1·9–1·0)	-0·6 (-2·4–1·1)	-0·2 (-1·7–1·3)
Childhood sexual abuse and bullying	18·4 (10·3–28·1)	18·0 (10·6–27·7)	18·5 (11·3–28·5)	14·5 (9·3–21·6)	-0·8 (-1·6–0·1)	-1·1 (-2·2–0·0)	-2·2 (-4·3–-0·2)
Childhood sexual abuse	7·5 (6·4–8·9)	8·5 (7·3–9·9)	9·9 (8·5–11·3)	9·1 (7·5–11·1)	0·6 (-0·2–1·3)	0·3 (-0·7–1·4)	-0·7 (-2·1–0·9)
Bullying victimization	22·2 (10·2–36·1)	20·9 (10·1–34·3)	20·7 (10·4–34·6)	15·3 (8·0–25·1)	-1·2 (-2·2–-0·2)	-1·5 (-2·9–-0·3)	-2·8 (-5·3–-0·4)
Unsafe sex	--	--	--	--	--	--	--
Low physical activity	20·6 (15·5–26·3)	19·6 (15·2–24·5)	19·6 (15·4–24·2)	22·5 (17·3–29·0)	0·3 (-0·7–1·2)	0·6 (-0·5–1·8)	1·2 (-0·5–2·9)
**Metabolic risks**	**26·4 (23·0–29·6)**	**31·3 (27·7–34·4)**	**35·8 (31·7–38·5)**	**39·5 (34·9–42·4)**	**1·3 (1·0–1·6)**	**1·1 (0·8–1·4)**	**0·9 (0·4–1·3)**
High fasting plasma glucose	13·6 (10·2–16·3)	17·9 (13·7–21·4)	22·6 (16·9–26·8)	25·7 (19·2–30·6)	2·1 (1·4–2·7)	1·7 (0·8–2·5)	1·2 (0·0–2·3)
High LDL cholesterol	63·6 (45·1–86·4)	53·8 (37·0–74·6)	48·2 (32·5–67·2)	46·6 (31·0–65·0)	-1·0 (-1·2–-0·8)	-0·7 (-0·9–-0·5)	-0·3 (-0·6–0·0)
High systolic blood pressure	29·3 (19·6–40·0)	25·5 (17·9–34·4)	19·0 (12·4–27·1)	26·2 (16·8–36·9)	-0·4 (-1·3–0·5)	0·1 (-1·1–1·2)	2·9 (0·6–5·0)
High body-mass index	29·9 (25·3–34·3)	35·9 (30·5–40·0)	41·0 (35·2–44·7)	45·0 (38·1–48·6)	1·3 (1·0–1·6)	1·1 (0·7–1·3)	0·8 (0·4–1·3)
Low bone mineral density	21·6 (15·6–28·9)	19·8 (13·8–26·8)	20·5 (14·4–27·7)	22·6 (16·6–29·6)	0·1 (-0·3–0·6)	0·6 (0·0–1·5)	0·9 (-0·4–2·4)
Kidney dysfunction	2·9 (2·4–3·9)	3·0 (2·5–4·0)	3·0 (2·4–4·0)	3·1 (2·6–4·1)	0·2 (0·1–0·3)	0·1 (0·0–0·3)	0·4 (0·1–0·7)

Oklahoma

Risk Names	SEV 1990	SEV 2000	SEV 2010	SEV 2021	Annualised rate of change 1990 to 2021	Annualised rate of change 2000 to 2021	Annualised rate of change 2010 to 2021
**All risk factors**	**26·7 (23·8–29·5)**	**26·8 (24·1–29·8)**	**27·3 (24·5–30·4)**	**27·1 (24·1–30·2)**	**0·0 (-0·2–0·3)**	**0·0 (-0·3–0·4)**	**-0·1 (-0·6–0·4)**
**Environmental/occupational risks**	**18·3 (12·6–28·8)**	**18·4 (12·9–28·2)**	**16·2 (10·8–24·8)**	**13·6 (9·1–21·2)**	**-1·0 (-1·7–-0·3)**	**-1·4 (-1·9–-1·0)**	**-1·6 (-2·2–-1·2)**
Unsafe water, sanitation, and handwashing	2·3 (1·2–3·7)	2·2 (1·1–3·7)	1·6 (0·5–2·9)	1·3 (0·5–2·2)	-1·9 (-4·1–0·1)	-2·6 (-5·4–0·0)	-2·3 (-6·0–1·7)
Unsafe water source	3·3 (1·3–6·9)	3·1 (1·2–6·3)	1·2 (0·5–2·4)	0·9 (0·4–1·8)	-4·0 (-6·7–-0·8)	-5·8 (-9·6–-1·7)	-2·5 (-7·8–3·5)
Unsafe sanitation	8·0 (4·2–13·8)	6·8 (3·7–11·8)	5·0 (2·6–8·7)	3·6 (1·8–6·0)	-2·6 (-5·0–-0·5)	-3·1 (-5·8–-0·4)	-3·2 (-6·4–0·2)
No access to handwashing facility	1·7 (0·6–3·2)	1·8 (0·6–3·5)	1·8 (0·6–3·6)	1·5 (0·5–2·9)	-0·4 (-3·2–2·4)	-0·9 (-4·5–2·8)	-1·7 (-7·0–3·9)
Air pollution	20·6 (7·2–32·5)	18·7 (8·7–29·2)	11·3 (5·2–20·4)	7·1 (2·3–15·2)	-3·4 (-5·9–-1·6)	-4·6 (-7·4–-3·0)	-4·2 (-8·0–-2·5)
Particulate matter pollution	12·3 (4·3–21·7)	11·4 (7·9–15·1)	7·8 (4·8–11·2)	4·4 (1·8–7·5)	-3·3 (-6·3–-0·4)	-4·5 (-7·5–-3·1)	-5·1 (-9·6–-3·0)
Ambient particulate matter pollution	19·0 (6·5–33·8)	17·5 (12·6–22·7)	11·9 (7·6–16·7)	6·8 (2·8–11·3)	-3·3 (-6·4–-0·4)	-4·5 (-7·5–-3·2)	-5·1 (-9·7–-3·0)
Household air pollution from solid fuels	0·0 (0·0–0·0)	0·0 (0·0–0·0)	0·0 (0·0–0·0)	0·0 (0·0–0·0)	-3·1 (-30·6–-6·7)	-4·1 (-37·0–-7·6)	-3·6 (-28·8–-5·8)
Ambient ozone pollution	22·4 (16·4–29·7)	28·1 (21·5–36·2)	23·3 (17·4–30·8)	13·4 (8·4–18·8)	-1·7 (-2·4–-1·1)	-3·5 (-4·8–-2·6)	-5·1 (-7·2–-3·5)
Ambient nitrogen dioxide pollution	54·5 (0·0–100·0)	47·6 (0·0–100·0)	23·6 (0·0–70·1)	17·1 (0·0–59·1)	-3·7 (-9·2–0·0)	-4·9 (-12·1–0·0)	-2·9 (-7·3–0·0)
Non-optimal temperature	34·5 (26·6–44·7)	39·0 (30·5–49·8)	38·8 (30·3–49·4)	31·7 (25·2–41·3)	-0·3 (-0·5–0·0)	-1·0 (-1·3–-0·6)	-1·8 (-2·5–-1·2)
High temperature	39·7 (29·6–49·5)	50·5 (39·3–60·8)	46·2 (35·1–56·6)	31·4 (22·4–40·8)	-0·8 (-1·0–-0·4)	-2·3 (-2·9–-1·5)	-3·5 (-4·5–-2·6)
Low temperature	27·1 (24·2–30·6)	29·3 (26·4–32·6)	29·9 (27·4–33·1)	26·9 (24·3–30·1)	0·0 (-0·1–0·1)	-0·4 (-0·6–-0·2)	-1·0 (-1·3–-0·7)
Other environmental risks	26·4 (6·6–44·9)	25·0 (6·6–43·3)	22·5 (6·6–40·9)	19·8 (6·6–37·3)	-0·9 (-1·5–0·0)	-1·1 (-1·8–0·0)	-1·2 (-2·1–0·0)
Residential radon	24·3 (0·0–77·4)	24·3 (0·0–77·4)	24·3 (0·0–77·4)	24·3 (0·0–77·4)	0·0 (0·0–0·0)	0·0 (0·0–0·0)	0·0 (0·0–0·0)
Lead exposure	27·2 (0·0–33·8)	25·2 (0·0–31·0)	21·6 (0·0–26·8)	17·7 (0·0–21·9)	-1·4 (-1·8–0·0)	-1·7 (-2·1–0·0)	-1·8 (-2·6–0·0)
Occupational risks	2·9 (2·5–3·8)	3·0 (2·5–3·9)	2·8 (2·3–3·6)	2·8 (2·3–3·6)	-0·2 (-0·4–0·1)	-0·3 (-0·7–0·0)	0·0 (-0·4–0·6)
Occupational carcinogens	0·9 (0·4–1·7)	1·0 (0·4–1·9)	1·0 (0·4–1·8)	1·0 (0·4–1·9)	0·3 (0·0–0·5)	0·0 (-0·4–0·4)	0·3 (-0·3–0·8)
Occupational exposure to asbestos	3·0 (2·7–3·3)	3·3 (2·9–3·6)	3·0 (2·7–3·3)	3·0 (2·4–3·8)	0·0 (-0·8–0·8)	-0·4 (-1·5–0·7)	0·0 (-2·1–2·0)
Occupational exposure to arsenic	0·5 (0·0–1·2)	0·5 (0·0–1·2)	0·5 (0·0–1·1)	0·5 (0·0–1·1)	-0·1 (-0·6–0·3)	-0·3 (-0·9–0·3)	0·1 (-0·8–1·0)
Occupational exposure to benzene	1·1 (0·1–3·1)	1·2 (0·1–3·4)	1·2 (0·1–3·4)	1·2 (0·1–3·6)	0·4 (0·0–0·7)	0·2 (-0·3–0·6)	0·4 (-0·3–1·0)
Occupational exposure to beryllium	0·0 (0·0–0·0)	0·0 (0·0–0·0)	0·0 (0·0–0·0)	0·0 (0·0–0·0)	0·2 (0·0–0·4)	0·1 (-0·3–0·4)	0·5 (0·0–0·9)
Occupational exposure to cadmium	0·1 (0·1–0·1)	0·1 (0·1–0·1)	0·1 (0·1–0·1)	0·1 (0·1–0·1)	0·0 (-0·4–0·4)	-0·2 (-0·8–0·3)	0·2 (-0·6–1·0)
Occupational exposure to chromium	0·1 (0·1–0·2)	0·1 (0·1–0·2)	0·1 (0·1–0·1)	0·1 (0·1–0·2)	0·0 (-0·3–0·4)	-0·2 (-0·6–0·3)	0·3 (-0·4–0·9)
Occupational exposure to diesel engine exhaust	0·7 (0·7–0·8)	0·7 (0·7–0·8)	0·7 (0·6–0·7)	0·7 (0·7–0·8)	0·1 (-0·3–0·4)	0·1 (-0·4–0·7)	0·6 (0·0–1·4)
Occupational exposure to formaldehyde	0·3 (0·3–0·3)	0·3 (0·3–0·3)	0·3 (0·3–0·3)	0·3 (0·3–0·3)	-0·1 (-0·5–0·3)	-0·3 (-0·9–0·2)	0·1 (-0·7–0·9)
Occupational exposure to nickel	0·4 (0·0–1·7)	0·4 (0·0–1·6)	0·4 (0·0–1·5)	0·4 (0·0–1·5)	-0·3 (-0·9–0·1)	-0·4 (-1·3–0·2)	0·0 (-1·1–1·0)
Occupational exposure to polycyclic aromatic hydrocarbons	0·3 (0·3–0·3)	0·3 (0·3–0·3)	0·3 (0·3–0·3)	0·3 (0·3–0·3)	0·1 (-0·2–0·4)	-0·1 (-0·6–0·3)	0·2 (-0·4–0·9)
Occupational exposure to silica	4·0 (0·6–13·1)	3·7 (0·6–12·0)	3·3 (0·5–10·9)	3·4 (0·5–10·6)	-0·6 (-1·0–-0·1)	-0·5 (-1·2–0·1)	0·1 (-0·9–0·9)
Occupational exposure to sulphuric acid	0·7 (0·1–2·4)	0·7 (0·1–2·4)	0·7 (0·1–2·3)	0·7 (0·1–2·3)	0·0 (-0·4–0·4)	-0·2 (-0·8–0·3)	0·2 (-0·6–1·0)
Occupational exposure to trichloroethylene	0·1 (0·1–0·1)	0·1 (0·1–0·1)	0·1 (0·1–0·1)	0·1 (0·1–0·1)	0·1 (-0·2–0·4)	0·0 (-0·4–0·4)	0·4 (-0·2–1·0)
Occupational asthmagens	17·9 (15·9–20·2)	18·1 (16·0–20·3)	16·3 (14·4–18·4)	16·6 (14·6–18·9)	-0·2 (-0·6–0·1)	-0·4 (-0·9–0·1)	0·2 (-0·6–1·0)
Occupational particulate matter, gases, and fumes	6·7 (5·1–9·0)	6·6 (5·0–8·7)	6·2 (4·8–8·0)	5·7 (4·4–7·4)	-0·5 (-0·8–-0·2)	-0·6 (-1·0–-0·3)	-0·7 (-1·1–-0·3)
Occupational noise	7·1 (6·6–7·8)	7·2 (6·6–7·8)	7·0 (6·6–7·6)	6·7 (6·3–7·3)	-0·2 (-0·4–0·0)	-0·3 (-0·5–-0·1)	-0·4 (-0·5–-0·2)
Occupational injuries	--	--	--	--	--	--	--
Occupational ergonomic factors	8·6 (7·2–10·4)	8·7 (7·4–10·5)	7·7 (6·5–9·2)	7·9 (6·5–9·6)	-0·3 (-0·8–0·1)	-0·5 (-1·0–0·1)	0·2 (-0·6–0·9)
**Behavioural risks**	**29·9 (27·0–33·0)**	**28·4 (25·4–31·7)**	**27·5 (24·3–30·9)**	**24·6 (21·4–28·2)**	**-0·6 (-1·0–-0·3)**	**-0·7 (-1·1–-0·3)**	**-1·0 (-1·6–-0·4)**
Child and maternal malnutrition	10·3 (6·9–14·4)	9·0 (6·1–12·3)	9·3 (6·4–12·8)	9·5 (6·3–12·9)	-0·3 (-1·0–0·6)	0·2 (-0·8–1·3)	0·2 (-1·4–1·9)
Suboptimal breastfeeding	69·0 (66·4–71·4)	66·9 (64·4–69·4)	63·1 (60·4–65·9)	62·4 (59·3–65·6)	-0·3 (-0·5–-0·2)	-0·3 (-0·5–-0·1)	-0·1 (-0·4–0·2)
Non-exclusive breastfeeding	64·6 (57·5–71·6)	60·2 (53·5–66·7)	49·3 (42·4–55·7)	46·9 (39·2–54·0)	-1·0 (-1·6–-0·6)	-1·2 (-1·9–-0·6)	-0·5 (-1·3–0·4)
Discontinued breastfeeding	88·9 (86·5–91·3)	86·5 (83·7–89·2)	84·6 (81·7–87·7)	84·7 (81·5–88·1)	-0·2 (-0·3–0·0)	-0·1 (-0·3–0·1)	0·0 (-0·3–0·3)
Child growth failure	1·4 (0·4–3·1)	1·3 (0·4–3·0)	1·2 (0·4–2·7)	1·1 (0·3–2·3)	-0·8 (-1·2–-0·4)	-1·1 (-1·6–-0·6)	-0·9 (-1·6–-0·1)
Child underweight	2·9 (1·8–3·9)	2·9 (1·8–3·9)	2·5 (1·5–3·4)	2·3 (1·4–3·1)	-0·8 (-1·3–-0·2)	-1·1 (-1·8–-0·5)	-0·8 (-1·8–0·1)
Child wasting	0·9 (0·6–1·4)	0·9 (0·6–1·4)	0·9 (0·6–1·4)	0·9 (0·5–1·3)	-0·2 (-0·5–0·1)	-0·2 (-0·7–0·3)	-0·5 (-1·1–0·3)
Child stunting	3·9 (3·0–4·9)	3·9 (3·0–4·8)	3·4 (2·6–4·3)	3·0 (2·3–3·8)	-0·8 (-1·4–-0·3)	-1·2 (-2·0–-0·4)	-1·0 (-2·2–0·1)
Low birth weight and short gestation	21·9 (19·2–24·7)	22·6 (19·9–25·5)	24·7 (21·6–27·6)	24·5 (21·3–27·6)	0·4 (0·1–0·6)	0·4 (0·0–0·7)	-0·1 (-0·7–0·6)
Short gestation	38·7 (34·4–43·2)	40·3 (35·8–45·1)	43·2 (38·4–47·9)	43·0 (38·2–47·7)	0·3 (0·0–0·6)	0·3 (-0·1–0·7)	0·0 (-0·8–0·8)
Low birth weight	16·6 (15·1–17·9)	17·0 (15·6–18·6)	18·5 (17·0–20·2)	18·4 (16·8–20·0)	0·3 (0·1–0·6)	0·4 (0·0–0·7)	-0·1 (-0·8–0·5)

Iron deficiency	3·6 (2·5–5·0)	3·2 (2·3–4·3)	3·4 (2·4–4·5)	3·4 (2·3–4·6)	-0·2 (-0·9–0·6)	0·3 (-0·7–1·3)	0·1 (-1·4–1·7)
Vitamin A deficiency	1·3 (0·0–2·7)	2·0 (0·0–4·1)	1·2 (0·0–2·6)	0·7 (0·0–1·6)	-1·8 (-3·9–0·4)	-4·6 (-7·0–0·0)	-4·7 (-8·5–0·0)
Zinc deficiency	2·0 (0·0–8·8)	1·2 (0·0–5·4)	1·1 (0·0–4·5)	1·0 (0·0–4·4)	-2·2 (-47·3–0·0)	-0·9 (-2·9–3·9)	-0·3 (-8·2–5·6)
Tobacco	41·4 (39·9–42·8)	38·6 (37·1–40·0)	35·2 (34·1–36·3)	27·8 (26·1–29·6)	-1·3 (-1·5–-1·1)	-1·6 (-1·8–-1·3)	-2·2 (-2·7–-1·6)
Smoking	39·3 (36·9–41·5)	35·3 (33·3–37·3)	32·5 (30·9–34·1)	25·0 (23·1–27·3)	-1·5 (-1·7–-1·2)	-1·6 (-2·0–-1·3)	-2·4 (-3·1–-1·7)
Chewing tobacco	2·5 (2·1–3·0)	2·8 (2·3–3·4)	2·8 (2·2–3·6)	2·6 (1·9–3·5)	0·1 (-1·0–1·2)	-0·3 (-1·8–1·1)	-0·6 (-2·9–1·8)
Second-hand smoke	32·5 (31·0–33·8)	31·2 (29·5–32·4)	28·4 (26·7–29·6)	23·2 (21·4–24·9)	-1·1 (-1·3–-0·9)	-1·4 (-1·8–-1·1)	-1·9 (-2·5–-1·3)
High alcohol use	23·9 (16·9–33·3)	21·8 (15·6–31·1)	22·6 (16·1–31·8)	23·1 (15·8–33·2)	-0·1 (-1·2–0·9)	0·3 (-1·2–1·6)	0·2 (-1·6–2·2)
Drug use	1·0 (0·7–1·5)	1·5 (1·2–1·8)	3·0 (2·1–3·9)	6·9 (4·5–9·2)	6·3 (3·8–7·9)	7·2 (5·5–8·2)	7·6 (6·5–8·5)
Dietary risks	38·9 (29·8–50·1)	42·2 (32·8–54·0)	44·4 (35·2–56·6)	44·3 (35·0–57·0)	0·4 (0·2–0·7)	0·2 (0·0–0·5)	0·0 (-0·4–0·4)
Diet low in fruits	39·4 (33·9–42·7)	37·9 (32·7–40·8)	37·3 (33·2–40·3)	38·0 (33·3–42·1)	-0·1 (-0·5–0·2)	0·0 (-0·5–0·5)	0·2 (-0·7–0·9)
Diet low in vegetables	27·8 (16·5–35·7)	29·7 (17·5–37·7)	32·2 (18·3–39·9)	31·8 (18·4–40·5)	0·4 (0·0–0·8)	0·3 (-0·2–0·8)	-0·1 (-0·9–0·7)
Diet low in legumes	38·8 (0·0–48·5)	39·7 (0·0–49·9)	43·2 (0·0–53·8)	43·5 (0·0–54·6)	0·4 (0·0–0·7)	0·4 (0·0–0·9)	0·1 (-0·6–0·8)
Diet low in whole grains	45·7 (38·2–52·2)	49·1 (40·7–55·9)	51·0 (42·7–58·6)	51·4 (42·9–58·8)	0·4 (0·1–0·7)	0·2 (-0·2–0·6)	0·1 (-0·5–0·6)
Diet low in nuts and seeds	13·8 (10·1–18·0)	9·2 (6·2–12·5)	6·1 (3·7–9·2)	6·1 (3·5–9·9)	-2·7 (-4·6–-1·1)	-2·0 (-4·6–0·2)	0·0 (-3·8–3·4)
Diet low in milk	37·6 (33·4–42·1)	35·8 (31·2–40·7)	33·1 (28·7–37·7)	32·2 (27·6–37·3)	-0·5 (-0·9–0·0)	-0·5 (-1·1–0·1)	-0·2 (-1·1–0·7)
Diet high in red meat	53·5 (0·0–72·3)	53·8 (0·0–73·5)	54·7 (0·0–74·3)	54·8 (0·0–74·2)	0·1 (-0·3–0·6)	0·1 (-0·5–0·6)	0·0 (-0·7–1·0)
Diet high in processed meat	51·3 (41·5–57·5)	62·2 (49·7–69·8)	69·1 (55·8–77·8)	67·4 (54·7–75·8)	0·9 (0·5–1·3)	0·4 (-0·2–0·9)	-0·2 (-1·0–0·5)
Diet high in sugar-sweetened beverages	35·3 (27·1–43·4)	47·8 (37·8–56·7)	55·2 (43·5–64·6)	55·1 (43·7–65·0)	1·4 (0·6–2·2)	0·7 (-0·4–1·6)	0·0 (-1·4–1·4)
Diet low in fibre	40·9 (24·1–48·4)	32·7 (18·4–39·9)	27·7 (15·7–33·8)	23·7 (13·5–30·4)	-1·8 (-2·7–-0·9)	-1·5 (-2·8–-0·4)	-1·4 (-3·3–0·2)
Diet low in calcium	6·0 (4·9–7·4)	5·2 (4·2–6·4)	5·3 (4·2–6·5)	5·1 (4·1–6·2)	-0·5 (-0·8–-0·3)	-0·1 (-0·4–0·2)	-0·3 (-0·7–0·1)
Diet low in seafood omega-3 fatty acids	51·0 (40·7–62·3)	59·3 (48·6–70·8)	60·6 (49·5–72·0)	55·4 (44·9–66·6)	0·3 (0·0–0·6)	-0·3 (-0·7–0·0)	-0·8 (-1·4–-0·3)
Diet low in omega-6 polyunsaturated fatty acids	37·5 (23·9–48·2)	34·5 (23·1–43·8)	24·7 (17·2–32·0)	22·5 (15·9–29·8)	-1·6 (-2·4–-0·9)	-2·0 (-3·0–-1·1)	-0·8 (-1·8–0·1)
Diet high in trans fatty acids	71·4 (63·9–79·2)	71·8 (63·7–78·8)	63·3 (55·9–70·9)	0·0 (0·0–0·0)	-65·8 (-66·1–-65·4)	-97·1 (-97·5–-96·5)	-184·2 (-185·3–-183·1)
Diet high in sodium	23·5 (0·5–70·6)	28·4 (1·9–76·8)	31·5 (3·3–77·4)	31·9 (3·0–79·0)	1·0 (0·2–6·4)	0·5 (-0·1–4·1)	0·1 (-2·3–1·8)
Intimate partner violence	27·3 (13·3–35·2)	27·6 (13·2–33·7)	26·4 (17·4–32·5)	27·2 (14·8–36·1)	0·0 (-1·5–1·5)	-0·1 (-1·9–1·8)	0·3 (-1·8–2·2)
Childhood sexual abuse and bullying	8·7 (5·7–14·3)	10·4 (6·7–16·6)	11·8 (7·6–19·0)	9·1 (6·1–13·4)	0·1 (-0·4–0·7)	-0·6 (-1·4–0·1)	-2·4 (-3·7–-1·0)
Childhood sexual abuse	8·0 (6·7–9·6)	8·4 (7·4–9·7)	9·7 (8·7–10·9)	9·0 (7·5–10·7)	0·4 (-0·4–1·2)	0·3 (-0·6–1·3)	-0·7 (-2·0–0·5)
Bullying victimization	7·9 (3·5–15·9)	9·9 (4·6–19·4)	11·2 (5·1–22·0)	7·7 (3·5–14·4)	-0·1 (-0·8–0·6)	-1·2 (-2·2–-0·2)	-3·4 (-5·2–-1·7)
Unsafe sex	--	--	--	--	--	--	--
Low physical activity	22·7 (17·2–29·1)	22·3 (17·1–27·0)	22·4 (17·9–27·6)	24·6 (18·7–30·4)	0·3 (-0·6–1·2)	0·5 (-0·7–1·6)	0·9 (-0·9–2·5)
**Metabolic risks**	**25·3 (21·9–28·8)**	**31·3 (27·7–34·4)**	**37·3 (33·2–39·9)**	**41·3 (36·5–44·3)**	**1·6 (1·3–1·9)**	**1·3 (1·0–1·7)**	**0·9 (0·5–1·3)**
High fasting plasma glucose	13·2 (10·1–16·3)	17·6 (13·2–21·7)	23·2 (17·7–27·7)	26·7 (20·5–31·8)	2·3 (1·6–2·9)	2·0 (1·1–2·8)	1·3 (0·0–2·5)
High LDL cholesterol	63·5 (44·7–86·6)	53·8 (37·0–74·2)	48·4 (32·8–67·9)	47·0 (31·7–66·0)	-1·0 (-1·2–-0·8)	-0·6 (-0·8–-0·5)	-0·3 (-0·5–0·0)
High systolic blood pressure	30·6 (20·5–41·5)	26·1 (18·4–36·1)	20·0 (13·2–28·5)	28·1 (17·9–39·9)	-0·3 (-1·1–0·5)	0·3 (-0·8–1·4)	3·1 (0·8–5·6)
High body-mass index	28·7 (23·9–33·2)	35·9 (30·5–39·9)	42·8 (36·7–46·3)	46·9 (40·2–50·6)	1·6 (1·2–1·9)	1·3 (0·9–1·6)	0·8 (0·4–1·2)
Low bone mineral density	21·6 (15·7–28·4)	19·7 (13·8–26·4)	20·5 (14·7–27·5)	22·2 (16·3–28·9)	0·1 (-0·4–0·6)	0·6 (-0·1–1·3)	0·7 (-0·7–2·1)
Kidney dysfunction	2·9 (2·4–3·8)	3·1 (2·5–4·0)	3·0 (2·5–4·0)	3·2 (2·6–4·2)	0·2 (0·1–0·3)	0·2 (0·0–0·3)	0·4 (0·1–0·7)

Oregon

Risk Names	SEV 1990	SEV 2000	SEV 2010	SEV 2021	Annualised rate of change 1990 to 2021	Annualised rate of change 2000 to 2021	Annualised rate of change 2010 to 2021
**All risk factors**	**26·0 (23·1–29·2)**	**26·4 (23·5–29·7)**	**27·3 (24·1–30·8)**	**26·5 (23·1–30·0)**	**0·1 (-0·2–0·3)**	**0·0 (-0·4–0·4)**	**-0·3 (-0·8–0·3)**
**Environmental/occupational risks**	**16·3 (10·5–27·5)**	**16·5 (10·4–27·4)**	**14·7 (9·0–24·8)**	**13·1 (8·1–22·7)**	**-0·7 (-1·3–-0·2)**	**-1·1 (-1·5–-0·7)**	**-1·0 (-1·5–-0·6)**
Unsafe water, sanitation, and handwashing	1·9 (0·9–3·2)	1·7 (0·7–2·8)	1·3 (0·4–2·2)	1·1 (0·3–2·0)	-1·8 (-4·4–0·2)	-2·2 (-5·4–0·4)	-1·9 (-5·8–1·7)
Unsafe water source	2·7 (1·0–5·7)	2·4 (0·9–5·0)	1·0 (0·4–1·9)	0·8 (0·3–1·4)	-4·0 (-6·5–-1·2)	-5·3 (-8·8–-1·8)	-2·0 (-7·6–3·2)
Unsafe sanitation	6·0 (3·2–9·7)	4·6 (2·5–7·8)	3·4 (1·8–5·9)	2·5 (1·3–4·4)	-2·8 (-5·2–-0·5)	-2·9 (-5·4–-0·3)	-2·8 (-6·0–0·6)
No access to handwashing facility	1·7 (0·5–3·3)	1·7 (0·5–3·3)	1·7 (0·6–3·1)	1·4 (0·5–2·8)	-0·5 (-3·3–2·4)	-0·8 (-4·9–3·0)	-1·5 (-7·3–3·8)
Air pollution	12·8 (2·4–24·0)	13·2 (3·5–23·6)	7·5 (1·5–16·6)	4·4 (0·5–12·1)	-3·5 (-8·2–-1·1)	-5·3 (-11·7–-3·0)	-4·9 (-12·1–-2·7)
Particulate matter pollution	6·0 (0·7–13·1)	5·8 (3·1–9·0)	3·4 (1·1–6·2)	1·8 (0·2–4·4)	-3·8 (-10·9–1·2)	-5·4 (-14·1–-3·1)	-5·6 (-17·7–-2·7)
Ambient particulate matter pollution	9·2 (1·1–20·2)	8·8 (4·7–13·4)	5·2 (1·7–9·4)	2·8 (0·3–6·5)	-3·8 (-11·0–1·2)	-5·5 (-14·2–-3·1)	-5·6 (-17·7–-2·7)
Household air pollution from solid fuels	0·0 (0·0–0·1)	0·0 (0·0–0·0)	0·0 (0·0–0·0)	0·0 (0·0–0·0)	-4·5 (-34·0–-6·8)	-5·6 (-31·8–-6·3)	-5·3 (-34·7–-7·2)
Ambient ozone pollution	8·1 (4·0–12·7)	10·6 (5·9–16·0)	7·8 (4·0–12·4)	13·7 (8·6–19·5)	1·7 (0·8–2·9)	1·2 (0·1–2·5)	5·1 (2·9–8·3)
Ambient nitrogen dioxide pollution	42·9 (0·0–99·5)	46·1 (0·0–100·0)	25·1 (0·0–73·1)	14·5 (0·0–55·0)	-3·5 (-14·0–0·0)	-5·5 (-21·4–0·0)	-5·0 (-24·5–0·0)
Non-optimal temperature	26·9 (22·1–34·7)	26·1 (21·9–32·7)	25·5 (21·2–32·5)	28·4 (22·8–36·8)	0·2 (0·0–0·4)	0·4 (-0·2–1·0)	1·0 (-0·1–2·1)
High temperature	26·8 (17·1–38·5)	19·0 (10·9–28·9)	24·1 (15·0–34·7)	37·2 (25·3–51·6)	1·1 (0·6–1·8)	3·2 (1·9–4·8)	3·9 (2·3–6·1)
Low temperature	24·5 (21·6–29·2)	25·1 (22·1–30·3)	23·9 (20·7–29·5)	24·4 (21·4–28·9)	0·0 (-0·1–0·1)	-0·1 (-0·3–0·0)	0·2 (-0·2–0·6)
Other environmental risks	27·9 (11·7–49·1)	26·5 (11·7–47·5)	23·8 (11·1–45·0)	21·2 (9·7–42·2)	-0·9 (-1·6–0·0)	-1·1 (-1·9–0·0)	-1·1 (-2·2–0·0)
Residential radon	29·9 (0·0–98·4)	29·9 (0·0–98·4)	29·9 (0·0–98·4)	29·9 (0·0–98·4)	0·0 (0·0–0·0)	0·0 (0·0–0·0)	0·0 (0·0–0·0)
Lead exposure	27·0 (0·0–34·3)	24·9 (0·0–31·2)	21·0 (0·0–26·6)	17·2 (0·0–21·4)	-1·5 (-1·9–0·0)	-1·7 (-2·3–0·0)	-1·8 (-2·6–0·0)
Occupational risks	2·9 (2·4–3·7)	3·0 (2·5–3·9)	2·8 (2·2–3·6)	2·8 (2·2–3·7)	-0·1 (-0·4–0·1)	-0·3 (-0·7–0·0)	0·0 (-0·4–0·5)
Occupational carcinogens	1·0 (0·5–1·8)	1·1 (0·5–2·0)	1·0 (0·5–1·9)	1·0 (0·5–1·9)	0·1 (-0·3–0·4)	-0·2 (-0·7–0·2)	0·1 (-0·7–0·7)
Occupational exposure to asbestos	5·0 (4·5–5·6)	5·6 (5·1–6·1)	4·9 (4·5–5·4)	4·4 (3·4–5·4)	-0·5 (-1·2–0·2)	-1·2 (-2·3–-0·2)	-1·1 (-3·4–0·9)
Occupational exposure to arsenic	0·5 (0·0–1·2)	0·5 (0·0–1·2)	0·4 (0·0–1·1)	0·5 (0·0–1·1)	-0·2 (-0·7–0·2)	-0·4 (-1·0–0·3)	0·1 (-0·7–1·1)
Occupational exposure to benzene	1·1 (0·1–2·9)	1·2 (0·1–3·3)	1·1 (0·1–3·3)	1·2 (0·1–3·5)	0·4 (0·0–0·7)	0·1 (-0·4–0·6)	0·4 (-0·3–1·0)
Occupational exposure to beryllium	0·0 (0·0–0·0)	0·0 (0·0–0·0)	0·0 (0·0–0·0)	0·0 (0·0–0·0)	0·2 (-0·1–0·4)	0·0 (-0·3–0·4)	0·5 (0·0–1·0)
Occupational exposure to cadmium	0·1 (0·1–0·1)	0·1 (0·1–0·1)	0·1 (0·1–0·1)	0·1 (0·1–0·1)	-0·2 (-0·6–0·3)	-0·3 (-0·9–0·3)	0·2 (-0·5–1·0)
Occupational exposure to chromium	0·1 (0·1–0·2)	0·1 (0·1–0·2)	0·1 (0·1–0·1)	0·1 (0·1–0·2)	-0·1 (-0·4–0·3)	-0·2 (-0·7–0·3)	0·3 (-0·4–1·0)
Occupational exposure to diesel engine exhaust	0·7 (0·6–0·7)	0·7 (0·6–0·7)	0·7 (0·6–0·7)	0·7 (0·7–0·8)	0·2 (-0·2–0·6)	0·2 (-0·3–0·7)	0·7 (0·0–1·4)
Occupational exposure to formaldehyde	0·3 (0·3–0·3)	0·3 (0·3–0·3)	0·3 (0·3–0·3)	0·3 (0·2–0·3)	-0·2 (-0·6–0·2)	-0·4 (-1·0–0·2)	0·1 (-0·7–0·9)
Occupational exposure to nickel	0·4 (0·0–1·6)	0·4 (0·0–1·5)	0·4 (0·0–1·4)	0·4 (0·0–1·4)	-0·3 (-0·8–0·2)	-0·4 (-1·1–0·3)	0·1 (-1·0–1·2)
Occupational exposure to polycyclic aromatic hydrocarbons	0·3 (0·3–0·3)	0·3 (0·3–0·3)	0·3 (0·3–0·3)	0·3 (0·3–0·3)	0·0 (-0·4–0·4)	-0·2 (-0·7–0·3)	0·3 (-0·3–0·9)
Occupational exposure to silica	3·4 (0·6–11·2)	3·3 (0·5–10·9)	3·0 (0·5–10·1)	3·1 (0·5–9·9)	-0·3 (-0·8–0·1)	-0·3 (-0·9–0·2)	0·2 (-0·7–1·3)
Occupational exposure to sulphuric acid	0·7 (0·2–2·5)	0·7 (0·1–2·6)	0·7 (0·1–2·3)	0·7 (0·1–2·3)	-0·2 (-0·6–0·3)	-0·3 (-0·9–0·2)	0·2 (-0·6–1·0)
Occupational exposure to trichloroethylene	0·1 (0·1–0·1)	0·1 (0·1–0·1)	0·1 (0·1–0·1)	0·1 (0·1–0·1)	0·0 (-0·2–0·4)	0·0 (-0·5–0·3)	0·4 (-0·2–1·0)
Occupational asthmagens	16·8 (14·6–19·4)	17·3 (15·2–19·7)	15·8 (13·8–18·2)	16·3 (14·2–18·8)	-0·1 (-0·5–0·3)	-0·3 (-0·8–0·3)	0·3 (-0·4–1·0)
Occupational particulate matter, gases, and fumes	6·6 (5·1–8·8)	6·5 (5·0–8·6)	6·1 (4·7–8·0)	5·6 (4·3–7·3)	-0·6 (-0·9–-0·3)	-0·7 (-1·0–-0·4)	-0·8 (-1·2–-0·4)
Occupational noise	6·8 (6·2–7·4)	6·8 (6·3–7·4)	6·7 (6·3–7·1)	6·4 (6·0–6·9)	-0·2 (-0·3–0·0)	-0·3 (-0·5–-0·1)	-0·4 (-0·6–-0·1)
Occupational injuries	--	--	--	--	--	--	--
Occupational ergonomic factors	8·0 (6·7–9·6)	7·9 (6·8–9·5)	7·1 (6·0–8·5)	7·2 (6·1–8·9)	-0·3 (-0·8–0·2)	-0·4 (-1·0–0·1)	0·2 (-0·5–0·9)
**Behavioural risks**	**29·6 (26·1–33·3)**	**27·8 (24·4–31·8)**	**26·5 (22·9–30·6)**	**25·0 (21·6–28·7)**	**-0·5 (-0·9–-0·2)**	**-0·5 (-1·1–-0·1)**	**-0·5 (-1·3–0·2)**
Child and maternal malnutrition	9·8 (6·6–13·7)	8·4 (5·8–11·7)	8·6 (5·9–11·9)	8·7 (6·0–12·2)	-0·4 (-1·3–0·6)	0·2 (-1·0–1·4)	0·2 (-1·5–1·9)
Suboptimal breastfeeding	68·8 (66·5–71·3)	66·7 (64·7–69·4)	62·9 (60·3–65·8)	62·2 (59·6–65·0)	-0·3 (-0·5–-0·2)	-0·3 (-0·5–-0·2)	-0·1 (-0·4–0·2)
Non-exclusive breastfeeding	64·1 (56·7–71·8)	59·8 (53·4–67·1)	48·7 (42·6–55·3)	46·2 (39·2–53·2)	-1·1 (-1·6–-0·5)	-1·2 (-1·9–-0·6)	-0·5 (-1·4–0·5)
Discontinued breastfeeding	88·6 (86·6–90·9)	86·3 (83·7–89·1)	84·5 (81·2–87·4)	84·6 (81·3–88·0)	-0·1 (-0·3–0·0)	-0·1 (-0·3–0·1)	0·0 (-0·3–0·3)
Child growth failure	1·2 (0·4–2·8)	1·1 (0·4–2·6)	1·0 (0·3–2·2)	0·9 (0·3–2·1)	-1·0 (-1·3–-0·6)	-1·1 (-1·6–-0·6)	-0·8 (-1·6–0·0)
Child underweight	2·6 (1·7–3·6)	2·4 (1·5–3·2)	2·1 (1·3–2·8)	1·9 (1·2–2·7)	-1·0 (-1·4–-0·5)	-1·1 (-1·8–-0·4)	-0·6 (-1·7–0·4)
Child wasting	0·9 (0·5–1·3)	0·8 (0·5–1·2)	0·8 (0·5–1·3)	0·8 (0·5–1·2)	-0·3 (-0·6–0·0)	-0·1 (-0·5–0·3)	-0·3 (-1·0–0·4)
Child stunting	3·6 (2·8–4·5)	3·4 (2·6–4·1)	2·9 (2·2–3·7)	2·6 (2·0–3·3)	-1·1 (-1·6–-0·5)	-1·2 (-2·0–-0·4)	-0·9 (-2·1–0·3)
Low birth weight and short gestation	15·8 (13·9–17·9)	16·7 (14·6–19·0)	18·2 (16·0–20·6)	19·4 (17·0–22·3)	0·7 (0·4–0·9)	0·7 (0·3–1·1)	0·6 (-0·2–1·4)
Short gestation	27·1 (23·8–31·0)	29·1 (25·4–33·4)	30·6 (26·6–34·8)	30·9 (27·3–35·5)	0·4 (0·1–0·7)	0·3 (-0·2–0·8)	0·1 (-0·8–1·0)
Low birth weight	13·0 (11·8–14·4)	13·3 (12·1–14·8)	14·6 (13·2–16·2)	15·9 (14·3–17·6)	0·6 (0·4–0·9)	0·8 (0·4–1·2)	0·8 (0·0–1·5)

Iron deficiency	3·4 (2·4–4·7)	2·9 (2·1–4·0)	3·1 (2·2–4·2)	3·1 (2·2–4·3)	-0·3 (-1·3–0·6)	0·3 (-1·0–1·4)	0·2 (-1·5–1·8)
Vitamin A deficiency	1·0 (0·0–2·1)	1·3 (0·0–2·7)	0·8 (0·0–1·7)	0·5 (0·0–1·0)	-2·5 (-4·5–0·0)	-5·0 (-8·1–0·0)	-5·0 (-9·5–0·0)
Zinc deficiency	2·0 (0·0–8·3)	1·1 (0·0–4·7)	1·0 (0·0–4·4)	1·0 (0·0–4·3)	-2·2 (-47·3–0·3)	-0·4 (-4·6–1·4)	-0·2 (-9·5–2·6)
Tobacco	37·9 (36·3–39·4)	33·2 (31·9–34·4)	28·4 (27·4–29·3)	24·9 (23·2–26·7)	-1·4 (-1·6–-1·1)	-1·4 (-1·7–-1·0)	-1·2 (-1·8–-0·6)
Smoking	35·9 (33·5–38·4)	30·9 (29·0–33·0)	26·1 (24·7–27·6)	22·4 (20·5–24·6)	-1·5 (-1·8–-1·2)	-1·5 (-2·0–-1·1)	-1·4 (-2·1–-0·6)
Chewing tobacco	2·1 (1·6–2·7)	2·3 (1·8–3·0)	2·4 (1·8–3·4)	2·4 (1·7–3·3)	0·6 (-0·8–1·9)	0·2 (-1·5–1·9)	0·0 (-2·7–2·5)
Second-hand smoke	30·1 (28·2–31·4)	26·6 (25·1–27·8)	23·1 (21·6–24·1)	20·7 (18·9–22·5)	-1·2 (-1·5–-0·9)	-1·2 (-1·6–-0·8)	-1·0 (-1·7–-0·3)
High alcohol use	28·1 (19·4–38·2)	27·0 (19·0–36·6)	28·4 (20·0–38·6)	29·2 (20·3–40·4)	0·1 (-0·9–1·1)	0·4 (-1·0–1·7)	0·3 (-1·6–2·3)
Drug use	0·9 (0·5–1·6)	1·0 (0·8–1·3)	1·5 (1·3–1·7)	4·2 (3·1–5·3)	5·0 (2·4–7·3)	6·7 (4·3–8·2)	9·4 (6·5–11·4)
Dietary risks	36·3 (27·2–47·4)	39·6 (30·2–51·2)	41·7 (32·4–53·0)	41·8 (32·5–53·6)	0·5 (0·2–0·8)	0·3 (0·0–0·6)	0·0 (-0·5–0·5)
Diet low in fruits	36·5 (31·3–40·1)	33·2 (28·5–36·1)	32·2 (27·5–35·2)	33·3 (27·6–37·5)	-0·3 (-0·7–0·1)	0·0 (-0·5–0·6)	0·3 (-0·6–1·2)
Diet low in vegetables	23·3 (13·4–31·0)	24·9 (14·5–32·4)	28·9 (15·8–36·4)	28·4 (16·5–36·2)	0·6 (0·2–1·2)	0·6 (0·1–1·2)	-0·2 (-1·0–0·7)
Diet low in legumes	38·5 (0·0–49·1)	38·4 (0·0–47·9)	42·9 (0·0–54·3)	43·2 (0·0–53·8)	0·4 (0·0–0·7)	0·6 (0·0–1·0)	0·1 (-0·7–0·7)
Diet low in whole grains	45·3 (37·4–51·6)	48·9 (40·6–55·4)	50·9 (42·4–57·7)	51·0 (42·5–58·6)	0·4 (0·1–0·7)	0·2 (-0·1–0·6)	0·0 (-0·5–0·5)
Diet low in nuts and seeds	13·2 (9·7–17·0)	7·4 (4·9–10·9)	5·8 (3·5–8·6)	5·9 (3·4–8·6)	-2·6 (-4·5–-1·1)	-1·1 (-3·5–1·3)	0·2 (-3·6–3·4)
Diet low in milk	36·6 (32·0–41·6)	33·3 (28·8–37·5)	32·5 (28·2–37·1)	31·9 (27·2–37·0)	-0·4 (-0·9–0·0)	-0·2 (-0·9–0·4)	-0·2 (-1·0–0·6)
Diet high in red meat	49·3 (0·0–68·2)	50·3 (0·0–69·4)	50·2 (0·0–69·1)	50·3 (0·0–69·3)	0·1 (-0·5–0·5)	0·0 (-0·8–0·6)	0·0 (-0·8–1·0)
Diet high in processed meat	46·7 (37·6–52·6)	58·8 (47·9–65·7)	66·0 (54·2–73·3)	64·2 (52·1–73·4)	1·0 (0·6–1·5)	0·4 (-0·2–1·0)	-0·3 (-1·1–0·5)
Diet high in sugar-sweetened beverages	35·8 (27·5–44·3)	51·1 (39·8–60·5)	55·9 (43·6–65·8)	55·9 (43·2–66·3)	1·4 (0·6–2·3)	0·4 (-0·5–1·3)	0·0 (-1·3–1·2)
Diet low in fibre	39·9 (22·1–47·5)	29·7 (16·1–36·5)	26·9 (14·3–33·7)	23·1 (12·4–30·3)	-1·8 (-2·7–-0·9)	-1·2 (-2·5–0·0)	-1·4 (-3·4–0·3)
Diet low in calcium	5·7 (4·6–6·9)	4·7 (3·8–5·8)	5·1 (4·1–6·4)	5·0 (4·0–6·2)	-0·4 (-0·7–-0·2)	0·3 (0·0–0·6)	-0·2 (-0·7–0·1)
Diet low in seafood omega-3 fatty acids	37·3 (27·8–47·0)	43·3 (33·4–53·7)	47·7 (36·8–58·1)	41·3 (30·9–51·3)	0·3 (-0·1–0·8)	-0·2 (-0·8–0·3)	-1·3 (-2·2–-0·5)
Diet low in omega-6 polyunsaturated fatty acids	36·6 (23·6–46·2)	31·3 (20·9–40·3)	24·2 (16·5–31·2)	21·8 (15·3–28·6)	-1·7 (-2·3–-0·9)	-1·7 (-2·6–-0·8)	-0·9 (-2·0–0·0)
Diet high in trans fatty acids	71·3 (63·2–79·4)	71·7 (64·9–78·6)	63·5 (53·6–71·6)	0·0 (0·0–0·0)	-65·8 (-66·1–-65·4)	-97·1 (-97·5–-96·6)	-184·3 (-185·4–-182·7)
Diet high in sodium	23·1 (0·6–67·3)	27·9 (2·0–74·4)	30·6 (3·5–74·8)	31·1 (3·2–76·1)	1·0 (0·2–6·6)	0·5 (-0·2–3·7)	0·1 (-2·7–1·8)
Intimate partner violence	29·8 (18·9–39·2)	30·7 (20·5–38·3)	25·9 (18·0–32·3)	24·5 (14·3–33·3)	-0·6 (-2·1–0·6)	-1·1 (-3·1–0·5)	-0·5 (-3·0–1·5)
Childhood sexual abuse and bullying	8·6 (5·4–13·9)	10·4 (6·7–16·5)	11·9 (7·6–18·8)	9·3 (6·2–13·9)	0·3 (-0·4–0·8)	-0·5 (-1·3–0·3)	-2·2 (-3·9–-0·8)
Childhood sexual abuse	7·4 (6·3–8·7)	8·4 (7·2–9·8)	9·8 (8·5–11·4)	9·1 (7·4–11·0)	0·7 (-0·1–1·4)	0·4 (-0·6–1·4)	-0·7 (-2·1–0·7)
Bullying victimization	8·0 (3·5–16·2)	10·0 (4·6–19·5)	11·3 (5·1–22·0)	7·9 (3·5–15·2)	0·0 (-0·7–0·6)	-1·1 (-2·1–-0·1)	-3·2 (-5·1–-1·4)
Unsafe sex	--	--	--	--	--	--	--
Low physical activity	18·5 (14·0–24·2)	17·8 (13·4–22·7)	18·5 (14·5–23·4)	21·1 (15·8–28·0)	0·4 (-0·5–1·3)	0·8 (-0·4–2·1)	1·2 (-0·8–2·8)
**Metabolic risks**	**25·7 (22·3–28·9)**	**30·5 (26·7–33·4)**	**33·2 (29·3–36·2)**	**36·5 (32·2–39·6)**	**1·1 (0·8–1·4)**	**0·9 (0·5–1·2)**	**0·8 (0·4–1·3)**
High fasting plasma glucose	12·3 (9·6–15·1)	16·1 (12·2–19·8)	20·1 (15·2–24·7)	23·6 (17·4–28·4)	2·1 (1·4–2·9)	1·8 (0·9–2·8)	1·5 (0·1–2·8)
High LDL cholesterol	63·5 (44·7–87·0)	53·8 (36·9–74·2)	48·2 (32·7–67·1)	46·7 (31·5–65·3)	-1·0 (-1·2–-0·8)	-0·7 (-0·9–-0·5)	-0·3 (-0·6–0·0)
High systolic blood pressure	28·5 (18·6–39·7)	24·2 (16·9–33·4)	17·6 (11·3–25·4)	25·1 (15·6–36·9)	-0·4 (-1·5–0·5)	0·2 (-1·1–1·3)	3·2 (0·9–5·7)
High body-mass index	29·2 (24·6–33·7)	35·2 (29·8–39·0)	38·4 (32·6–42·3)	41·3 (35·1–45·3)	1·1 (0·8–1·4)	0·8 (0·4–1·1)	0·7 (0·2–1·1)
Low bone mineral density	21·9 (16·2–28·6)	21·8 (15·9–29·3)	23·3 (17·1–30·4)	24·3 (17·5–31·5)	0·3 (-0·1–0·8)	0·5 (-0·1–1·2)	0·4 (-0·9–1·8)
Kidney dysfunction	2·8 (2·3–3·7)	2·9 (2·4–3·8)	2·8 (2·3–3·8)	3·0 (2·4–3·9)	0·2 (0·0–0·3)	0·1 (0·0–0·3)	0·4 (0·0–0·7)

Pennsylvania

Risk Names	SEV 1990	SEV 2000	SEV 2010	SEV 2021	Annualised rate of change 1990 to 2021	Annualised rate of change 2000 to 2021	Annualised rate of change 2010 to 2021
**All risk factors**	**28·5 (25·5–31·8)**	**27·9 (25·0–31·1)**	**28·4 (25·3–31·7)**	**29·1 (25·9–32·3)**	**0·1 (-0·2–0·3)**	**0·2 (-0·2–0·5)**	**0·2 (-0·3–0·8)**
**Environmental/occupational risks**	**19·9 (13·2–30·4)**	**19·6 (13·8–30·2)**	**16·6 (11·3–26·6)**	**14·4 (9·6–23·4)**	**-1·0 (-1·8–-0·3)**	**-1·5 (-1·9–-0·9)**	**-1·3 (-1·8–-0·6)**
Unsafe water, sanitation, and handwashing	1·8 (0·9–2·9)	1·6 (0·7–2·7)	1·3 (0·4–2·2)	1·1 (0·3–1·9)	-1·6 (-3·9–0·5)	-1·9 (-4·9–1·1)	-1·7 (-5·2–1·8)
Unsafe water source	2·5 (1·0–5·2)	2·2 (0·8–4·9)	0·9 (0·4–1·9)	0·8 (0·3–1·4)	-3·8 (-6·4–-1·1)	-5·1 (-9·0–-1·2)	-1·9 (-7·4–4·1)
Unsafe sanitation	5·4 (3·0–8·7)	4·1 (2·2–7·1)	3·3 (1·7–5·6)	2·5 (1·3–4·3)	-2·5 (-4·7–-0·3)	-2·4 (-5·1–0·5)	-2·4 (-5·7–1·2)
No access to handwashing facility	1·6 (0·5–3·2)	1·6 (0·5–3·1)	1·7 (0·6–3·1)	1·4 (0·4–2·7)	-0·3 (-3·3–2·8)	-0·6 (-4·6–3·7)	-1·4 (-7·0–3·6)
Air pollution	28·6 (9·8–39·9)	26·3 (11·0–33·3)	14·2 (6·3–24·5)	8·5 (3·2–16·9)	-3·9 (-6·5–-1·7)	-5·4 (-8·4–-2·9)	-4·7 (-7·9–-2·9)
Particulate matter pollution	16·2 (7·2–27·0)	14·3 (10·6–18·6)	9·0 (5·8–12·5)	5·6 (2·9–8·9)	-3·4 (-5·7–-1·0)	-4·4 (-6·5–-3·3)	-4·2 (-7·1–-2·8)
Ambient particulate matter pollution	25·3 (10·8–42·5)	22·1 (17·0–28·0)	13·7 (9·0–18·7)	8·6 (4·5–13·2)	-3·5 (-5·8–-1·0)	-4·5 (-6·6–-3·4)	-4·3 (-7·1–-2·8)
Household air pollution from solid fuels	0·0 (0·0–0·1)	0·0 (0·0–0·0)	0·0 (0·0–0·0)	0·0 (0·0–0·0)	-3·5 (-36·0–-7·0)	-4·2 (-34·0–-7·2)	-3·9 (-26·8–-6·2)
Ambient ozone pollution	26·7 (20·3–34·7)	25·2 (18·7–33·7)	19·2 (13·5–27·1)	14·1 (8·9–21·2)	-2·1 (-2·7–-1·5)	-2·8 (-3·8–-2·0)	-2·8 (-4·4–-1·8)
Ambient nitrogen dioxide pollution	80·9 (0·0–100·0)	77·4 (0·0–100·0)	34·6 (0·0–89·1)	19·1 (0·0–64·1)	-4·7 (-13·3–0·0)	-6·7 (-18·3–0·0)	-5·4 (-18·4–0·0)
Non-optimal temperature	28·9 (22·3–39·7)	28·9 (23·4–38·2)	34·1 (26·2–45·3)	31·3 (24·3–42·3)	0·3 (0·0–0·6)	0·4 (-0·1–0·9)	-0·8 (-1·2–-0·4)
High temperature	22·2 (11·7–33·6)	19·9 (9·8–31·0)	35·0 (22·4–47·1)	29·8 (18·8–40·8)	1·0 (0·4–1·8)	1·9 (1·0–3·6)	-1·4 (-2·4–-0·7)
Low temperature	26·5 (23·2–30·1)	27·4 (24·5–30·7)	29·2 (26·4–32·5)	27·4 (24·5–31·0)	0·1 (0·0–0·3)	0·0 (-0·1–0·1)	-0·6 (-0·8–-0·3)
Other environmental risks	27·7 (9·3–45·7)	26·1 (9·3–44·4)	23·5 (9·3–41·8)	20·8 (9·3–38·7)	-0·9 (-1·6–0·0)	-1·1 (-1·9–0·0)	-1·1 (-2·2–0·0)
Residential radon	28·5 (0·0–85·1)	28·5 (0·0–85·1)	28·5 (0·0–85·1)	28·5 (0·0–85·1)	0·0 (0·0–0·0)	0·0 (0·0–0·0)	0·0 (0·0–0·0)
Lead exposure	27·3 (0·0–34·0)	25·0 (0·0–30·9)	21·2 (0·0–26·4)	17·3 (0·0–21·6)	-1·5 (-1·9–0·0)	-1·8 (-2·3–0·0)	-1·8 (-2·6–0·0)
Occupational risks	3·1 (2·6–4·0)	3·1 (2·6–4·0)	2·9 (2·3–3·8)	2·9 (2·3–3·7)	-0·2 (-0·5–0·0)	-0·4 (-0·7–-0·1)	0·0 (-0·5–0·4)
Occupational carcinogens	1·0 (0·5–1·9)	1·1 (0·5–2·1)	1·1 (0·5–2·0)	1·1 (0·5–2·0)	0·1 (-0·3–0·3)	-0·2 (-0·7–0·1)	0·0 (-0·6–0·5)
Occupational exposure to asbestos	5·2 (4·7–5·7)	5·8 (5·3–6·1)	5·1 (4·7–5·4)	4·5 (3·6–5·4)	-0·5 (-1·2–0·2)	-1·2 (-2·2–-0·3)	-1·1 (-2·8–0·7)
Occupational exposure to arsenic	0·6 (0·0–1·3)	0·5 (0·0–1·3)	0·5 (0·0–1·2)	0·5 (0·0–1·2)	-0·4 (-0·8–0·0)	-0·4 (-1·0–0·2)	0·0 (-0·8–0·8)
Occupational exposure to benzene	1·1 (0·1–3·2)	1·2 (0·1–3·5)	1·2 (0·1–3·5)	1·2 (0·1–3·7)	0·4 (0·0–0·7)	0·1 (-0·4–0·4)	0·3 (-0·3–0·8)
Occupational exposure to beryllium	0·0 (0·0–0·0)	0·0 (0·0–0·0)	0·0 (0·0–0·0)	0·0 (0·0–0·0)	0·2 (-0·1–0·4)	0·1 (-0·3–0·4)	0·4 (0·0–0·8)
Occupational exposure to cadmium	0·1 (0·1–0·1)	0·1 (0·1–0·1)	0·1 (0·1–0·1)	0·1 (0·1–0·1)	-0·3 (-0·7–0·1)	-0·3 (-0·9–0·2)	0·1 (-0·7–0·8)
Occupational exposure to chromium	0·2 (0·1–0·2)	0·2 (0·2–0·2)	0·1 (0·1–0·2)	0·2 (0·1–0·2)	-0·2 (-0·5–0·1)	-0·3 (-0·7–0·2)	0·2 (-0·5–0·8)
Occupational exposure to diesel engine exhaust	0·7 (0·6–0·7)	0·7 (0·7–0·8)	0·7 (0·7–0·8)	0·8 (0·7–0·8)	0·3 (0·0–0·7)	0·3 (-0·2–0·8)	0·7 (0·0–1·4)
Occupational exposure to formaldehyde	0·3 (0·3–0·4)	0·3 (0·3–0·3)	0·3 (0·3–0·3)	0·3 (0·3–0·3)	-0·4 (-0·7–0·0)	-0·4 (-1·0–0·1)	0·0 (-0·8–0·7)
Occupational exposure to nickel	0·5 (0·0–1·9)	0·4 (0·0–1·7)	0·4 (0·0–1·5)	0·4 (0·0–1·5)	-0·5 (-1·0–0·0)	-0·4 (-1·1–0·2)	0·0 (-1·0–0·9)
Occupational exposure to polycyclic aromatic hydrocarbons	0·3 (0·3–0·3)	0·3 (0·3–0·3)	0·3 (0·3–0·3)	0·3 (0·3–0·3)	-0·1 (-0·4–0·2)	-0·2 (-0·7–0·2)	0·2 (-0·5–0·7)
Occupational exposure to silica	3·9 (0·6–12·7)	3·7 (0·6–12·2)	3·3 (0·5–10·6)	3·4 (0·5–10·7)	-0·5 (-0·9–0·0)	-0·4 (-1·0–0·2)	0·1 (-0·7–1·0)
Occupational exposure to sulphuric acid	0·8 (0·2–2·7)	0·8 (0·2–2·6)	0·7 (0·2–2·4)	0·8 (0·1–2·5)	-0·3 (-0·7–0·1)	-0·3 (-0·8–0·2)	0·1 (-0·7–0·8)
Occupational exposure to trichloroethylene	0·1 (0·1–0·1)	0·1 (0·1–0·1)	0·1 (0·1–0·1)	0·1 (0·1–0·1)	0·0 (-0·3–0·3)	-0·1 (-0·4–0·3)	0·3 (-0·2–0·9)
Occupational asthmagens	18·2 (15·8–20·8)	18·3 (16·0–20·9)	16·7 (14·5–18·8)	17·0 (14·8–19·1)	-0·2 (-0·6–0·1)	-0·4 (-0·8–0·1)	0·2 (-0·6–0·8)
Occupational particulate matter, gases, and fumes	7·1 (5·4–9·3)	6·9 (5·3–9·0)	6·4 (4·9–8·2)	5·9 (4·5–7·6)	-0·6 (-0·9–-0·4)	-0·7 (-1·1–-0·4)	-0·8 (-1·2–-0·4)
Occupational noise	7·0 (6·4–7·6)	7·0 (6·5–7·6)	6·9 (6·5–7·4)	6·6 (6·2–7·2)	-0·2 (-0·3–0·0)	-0·3 (-0·4–-0·1)	-0·3 (-0·5–-0·1)
Occupational injuries	--	--	--	--	--	--	--
Occupational ergonomic factors	8·0 (6·7–9·9)	8·1 (6·7–9·8)	7·3 (6·2–8·9)	7·6 (6·3–9·3)	-0·2 (-0·7–0·2)	-0·3 (-1·0–0·1)	0·3 (-0·4–0·9)
**Behavioural risks**	**31·6 (28·6–35·5)**	**30·2 (27·3–33·8)**	**28·6 (25·6–32·3)**	**26·2 (23·0–29·8)**	**-0·6 (-1·0–-0·3)**	**-0·7 (-1·2–-0·2)**	**-0·8 (-1·4–-0·2)**
Child and maternal malnutrition	12·9 (8·4–18·4)	12·0 (8·0–16·6)	11·8 (7·7–16·7)	11·8 (7·5–16·7)	-0·3 (-0·7–0·1)	-0·1 (-0·7–0·5)	0·0 (-1·0–0·8)
Suboptimal breastfeeding	68·9 (66·1–71·5)	66·7 (64·5–69·5)	62·8 (60·0–65·7)	62·1 (59·2–65·2)	-0·3 (-0·5–-0·2)	-0·3 (-0·5–-0·2)	-0·1 (-0·4–0·2)
Non-exclusive breastfeeding	64·2 (55·4–71·6)	59·4 (52·8–66·8)	48·4 (42·0–55·3)	46·1 (39·1–53·8)	-1·1 (-1·6–-0·6)	-1·2 (-1·9–-0·6)	-0·4 (-1·4–0·5)
Discontinued breastfeeding	88·8 (86·4–91·1)	86·4 (83·7–89·2)	84·4 (81·1–87·6)	84·5 (81·4–87·6)	-0·2 (-0·3–0·0)	-0·1 (-0·3–0·1)	0·0 (-0·3–0·3)
Child growth failure	1·3 (0·4–2·9)	1·2 (0·4–2·7)	1·0 (0·3–2·3)	0·9 (0·3–2·2)	-0·9 (-1·3–-0·6)	-1·0 (-1·6–-0·5)	-0·6 (-1·4–0·1)
Child underweight	2·6 (1·7–3·7)	2·4 (1·6–3·3)	2·1 (1·3–2·8)	2·0 (1·2–2·7)	-1·0 (-1·5–-0·5)	-1·1 (-1·8–-0·4)	-0·6 (-1·5–0·5)
Child wasting	0·9 (0·5–1·3)	0·8 (0·5–1·2)	0·8 (0·5–1·2)	0·8 (0·5–1·2)	-0·3 (-0·6–0·1)	-0·1 (-0·5–0·4)	-0·3 (-0·8–0·4)
Child stunting	3·7 (2·8–4·6)	3·4 (2·6–4·2)	2·9 (2·3–3·7)	2·7 (2·1–3·3)	-1·0 (-1·6–-0·5)	-1·1 (-1·9–-0·4)	-0·9 (-2·0–0·4)
Low birth weight and short gestation	20·5 (17·8–23·2)	22·3 (19·4–25·3)	24·1 (21·2–27·4)	23·8 (20·9–27·0)	0·5 (0·2–0·7)	0·3 (0·0–0·7)	-0·1 (-0·8–0·6)
Short gestation	36·4 (32·2–41·2)	40·2 (34·9–45·8)	41·7 (36·9–47·5)	40·3 (35·4–45·8)	0·3 (0·0–0·6)	0·0 (-0·4–0·4)	-0·3 (-1·1–0·5)
Low birth weight	15·8 (14·5–17·5)	16·6 (15·2–18·2)	18·2 (16·6–20·1)	18·3 (16·7–20·2)	0·5 (0·2–0·7)	0·5 (0·1–0·8)	0·1 (-0·5–0·7)

Iron deficiency	4·6 (3·1–6·4)	4·2 (2·9–5·8)	4·2 (2·9–5·8)	4·1 (2·8–5·7)	-0·3 (-0·7–0·1)	-0·1 (-0·7–0·4)	-0·1 (-1·0–0·7)
Vitamin A deficiency	0·9 (0·0–1·8)	1·1 (0·0–2·4)	0·7 (0·0–1·6)	0·4 (0·0–1·0)	-2·2 (-4·8–0·0)	-4·5 (-7·4–0·0)	-4·4 (-8·2–0·0)
Zinc deficiency	1·8 (0·0–7·7)	1·1 (0·0–4·7)	1·0 (0·0–4·2)	0·9 (0·0–3·9)	-2·1 (-47·1–0·5)	-0·7 (-2·8–5·0)	-0·2 (-5·3–15·9)
Tobacco	41·6 (40·1–43·2)	37·6 (36·2–38·8)	32·8 (31·6–33·9)	27·4 (25·8–29·2)	-1·4 (-1·6–-1·1)	-1·5 (-1·8–-1·2)	-1·6 (-2·2–-1·1)
Smoking	38·0 (35·5–40·4)	33·7 (31·7–35·5)	29·4 (27·9–31·1)	24·3 (22·3–26·5)	-1·4 (-1·7–-1·1)	-1·5 (-1·9–-1·1)	-1·7 (-2·4–-1·0)
Chewing tobacco	1·7 (1·3–2·1)	1·8 (1·4–2·3)	2·0 (1·5–2·6)	2·0 (1·4–2·7)	0·6 (-0·6–1·8)	0·5 (-1·2–2·2)	0·2 (-2·5–2·9)
Second-hand smoke	34·5 (32·6–35·8)	31·1 (29·2–32·2)	26·9 (25·3–28·0)	22·9 (21·2–24·6)	-1·3 (-1·6–-1·1)	-1·5 (-1·8–-1·1)	-1·5 (-2·1–-0·9)
High alcohol use	29·1 (21·1–40·6)	28·2 (21·2–38·1)	28·7 (21·2–38·7)	28·8 (20·6–39·2)	0·0 (-1·1–1·0)	0·1 (-1·3–1·3)	0·0 (-1·7–1·6)
Drug use	1·1 (0·7–1·7)	1·5 (1·3–1·8)	2·8 (2·2–3·4)	6·6 (4·6–8·4)	5·9 (3·3–7·8)	7·1 (5·0–8·2)	7·8 (6·2–8·9)
Dietary risks	37·0 (27·8–48·1)	40·1 (31·0–51·6)	42·0 (32·8–53·9)	41·6 (32·4–53·9)	0·4 (0·1–0·7)	0·2 (-0·1–0·5)	-0·1 (-0·6–0·4)
Diet low in fruits	36·4 (30·9–39·7)	33·8 (29·1–36·9)	32·8 (28·3–35·4)	33·5 (28·5–37·5)	-0·3 (-0·6–0·1)	0·0 (-0·6–0·5)	0·2 (-0·7–1·1)
Diet low in vegetables	28·1 (16·5–35·7)	28·7 (16·8–36·5)	30·4 (17·2–38·3)	28·7 (17·2–37·3)	0·1 (-0·3–0·4)	0·0 (-0·6–0·5)	-0·5 (-1·5–0·4)
Diet low in legumes	37·8 (0·0–47·0)	37·9 (0·0–47·3)	41·8 (0·0–51·8)	42·3 (0·0–53·0)	0·4 (0·0–0·7)	0·5 (0·0–1·0)	0·1 (-0·6–0·9)
Diet low in whole grains	45·6 (38·1–51·9)	49·1 (41·0–55·8)	50·4 (42·4–57·9)	50·6 (41·8–58·1)	0·3 (0·0–0·6)	0·1 (-0·3–0·5)	0·0 (-0·6–0·6)
Diet low in nuts and seeds	12·3 (9·0–16·4)	7·1 (4·6–10·1)	4·9 (2·9–7·2)	5·0 (2·8–7·5)	-2·9 (-4·8–-1·1)	-1·6 (-4·3–0·8)	0·3 (-3·5–4·1)
Diet low in milk	36·2 (32·0–40·6)	33·1 (29·0–37·9)	31·3 (26·8–36·1)	30·7 (26·0–35·3)	-0·5 (-1·0–-0·1)	-0·4 (-1·0–0·2)	-0·2 (-1·1–0·7)
Diet high in red meat	49·5 (0·0–68·5)	50·9 (0·0–70·0)	51·2 (0·0–70·6)	51·3 (0·0–70·4)	0·1 (-0·4–0·6)	0·0 (-0·7–0·7)	0·0 (-0·9–1·0)
Diet high in processed meat	44·9 (35·9–50·0)	56·6 (45·9–63·3)	63·4 (51·7–70·9)	61·5 (50·9–69·6)	1·0 (0·6–1·5)	0·4 (-0·1–1·0)	-0·3 (-1·0–0·5)
Diet high in sugar-sweetened beverages	37·3 (27·7–45·9)	52·5 (42·1–62·1)	58·0 (47·5–68·4)	57·7 (45·8–69·7)	1·4 (0·5–2·2)	0·5 (-0·5–1·4)	0·0 (-1·2–1·2)
Diet low in fibre	38·6 (21·0–45·9)	29·0 (15·9–36·0)	25·3 (14·2–31·8)	21·8 (12·8–28·6)	-1·8 (-2·8–-1·0)	-1·4 (-2·7–-0·2)	-1·4 (-3·4–0·4)
Diet low in calcium	5·6 (4·5–6·9)	4·7 (3·8–5·8)	4·9 (3·9–6·0)	4·8 (3·8–5·8)	-0·5 (-0·7–-0·3)	0·0 (-0·2–0·3)	-0·3 (-0·6–0·1)
Diet low in seafood omega-3 fatty acids	35·9 (27·3–45·2)	42·6 (32·6–53·1)	45·4 (35·3–56·1)	39·8 (29·9–50·3)	0·3 (-0·1–0·8)	-0·3 (-0·9–0·2)	-1·2 (-1·9–-0·5)
Diet low in omega-6 polyunsaturated fatty acids	35·0 (22·4–45·2)	30·3 (20·4–39·2)	22·5 (15·4–28·6)	20·7 (14·3–27·2)	-1·7 (-2·4–-0·9)	-1·8 (-2·7–-1·0)	-0·7 (-1·8–0·2)
Diet high in trans fatty acids	71·4 (64·2–79·0)	71·8 (64·2–78·5)	63·4 (54·9–71·1)	0·0 (0·0–0·0)	-65·8 (-66·1–-65·4)	-97·1 (-97·5–-96·6)	-184·3 (-185·3–-182·9)
Diet high in sodium	23·4 (0·7–69·1)	28·2 (1·8–76·3)	31·2 (3·3–75·4)	31·7 (3·3–77·9)	1·0 (0·2–5·9)	0·6 (-0·1–3·9)	0·1 (-2·0–1·8)
Intimate partner violence	30·2 (18·3–39·3)	31·3 (20·2–39·1)	26·9 (18·1–33·4)	25·4 (16·0–33·8)	-0·6 (-1·8–0·6)	-1·0 (-2·7–0·5)	-0·5 (-2·7–1·4)
Childhood sexual abuse and bullying	8·6 (5·5–13·8)	10·4 (6·7–16·6)	11·9 (7·6–18·9)	9·3 (6·2–14·3)	0·3 (-0·3–0·8)	-0·5 (-1·4–0·2)	-2·3 (-3·7–-0·9)
Childhood sexual abuse	7·5 (6·4–8·8)	8·5 (7·3–9·9)	9·8 (8·5–11·4)	9·1 (7·4–11·0)	0·6 (-0·2–1·3)	0·3 (-0·7–1·4)	-0·7 (-2·1–0·7)
Bullying victimization	8·0 (3·5–15·9)	9·9 (4·6–19·2)	11·3 (5·1–21·9)	7·9 (3·6–15·0)	0·0 (-0·7–0·7)	-1·1 (-2·1–-0·1)	-3·2 (-5·0–-1·5)
Unsafe sex	--	--	--	--	--	--	--
Low physical activity	20·1 (15·1–25·8)	19·7 (15·3–24·1)	20·1 (15·8–25·2)	22·6 (17·2–29·0)	0·4 (-0·6–1·4)	0·7 (-0·5–2·0)	1·1 (-0·8–2·8)
**Metabolic risks**	**26·5 (23·0–29·7)**	**31·0 (27·2–33·9)**	**34·4 (30·3–37·1)**	**37·2 (32·7–40·2)**	**1·1 (0·8–1·4)**	**0·9 (0·6–1·2)**	**0·7 (0·3–1·2)**
High fasting plasma glucose	13·0 (9·9–16·0)	16·8 (12·6–20·4)	21·6 (16·5–26·3)	25·1 (19·6–30·2)	2·1 (1·4–2·8)	1·9 (1·0–2·9)	1·4 (0·1–2·6)
High LDL cholesterol	63·8 (45·0–87·0)	54·0 (37·2–74·6)	48·3 (32·7–67·5)	46·8 (31·4–65·6)	-1·0 (-1·2–-0·8)	-0·7 (-0·9–-0·5)	-0·3 (-0·6–-0·1)
High systolic blood pressure	29·7 (20·2–40·2)	25·6 (18·1–35·0)	19·0 (12·6–27·0)	25·8 (15·8–38·0)	-0·5 (-1·4–0·4)	0·0 (-1·2–1·2)	2·8 (0·7–5·1)
High body-mass index	30·4 (25·7–34·7)	35·9 (30·5–39·7)	39·7 (33·7–43·3)	42·3 (35·9–45·9)	1·1 (0·8–1·3)	0·8 (0·5–1·1)	0·6 (0·1–1·0)
Low bone mineral density	19·4 (13·7–26·1)	19·2 (13·7–25·6)	21·0 (15·1–28·1)	19·8 (14·1–26·8)	0·1 (-0·4–0·6)	0·1 (-0·5–0·8)	-0·5 (-1·9–1·0)
Kidney dysfunction	3·0 (2·4–3·9)	3·0 (2·5–4·0)	3·0 (2·4–3·9)	3·1 (2·5–4·1)	0·1 (0·0–0·2)	0·1 (-0·1–0·2)	0·3 (0·0–0·6)

Rhode Island

Risk Names	SEV 1990	SEV 2000	SEV 2010	SEV 2021	Annualised rate of change 1990 to 2021	Annualised rate of change 2000 to 2021	Annualised rate of change 2010 to 2021
**All risk factors**	**28·9 (25·8–32·4)**	**28·8 (25·6–31·9)**	**29·5 (26·4–32·8)**	**30·1 (26·8–33·4)**	**0·1 (-0·2–0·4)**	**0·2 (-0·2–0·6)**	**0·2 (-0·4–0·7)**
**Environmental/occupational risks**	**17·9 (11·5–28·6)**	**17·7 (11·5–28·6)**	**15·2 (9·9–25·4)**	**13·4 (8·3–22·4)**	**-0·9 (-1·7–-0·3)**	**-1·3 (-1·8–-0·8)**	**-1·1 (-1·7–-0·4)**
Unsafe water, sanitation, and handwashing	1·6 (0·8–2·6)	1·5 (0·7–2·4)	1·2 (0·4–2·2)	1·0 (0·3–1·8)	-1·6 (-4·1–0·6)	-1·9 (-5·1–0·7)	-1·6 (-5·0–2·3)
Unsafe water source	2·3 (0·9–4·5)	2·2 (0·8–4·6)	0·9 (0·4–1·7)	0·7 (0·3–1·4)	-3·7 (-6·4–-0·9)	-5·2 (-9·1–-0·9)	-1·8 (-7·1–3·4)
Unsafe sanitation	4·9 (2·6–8·3)	3·8 (1·9–6·5)	2·9 (1·4–5·1)	2·3 (1·1–4·1)	-2·4 (-5·0–-0·3)	-2·4 (-5·4–0·2)	-2·1 (-5·3–1·2)
No access to handwashing facility	1·4 (0·5–2·8)	1·5 (0·5–2·9)	1·5 (0·4–3·0)	1·3 (0·4–2·8)	-0·3 (-3·4–2·7)	-0·6 (-4·7–3·3)	-1·3 (-6·6–3·8)
Air pollution	23·1 (6·1–33·4)	20·9 (6·3–28·2)	11·1 (3·8–20·6)	6·4 (1·3–14·8)	-4·1 (-7·8–-1·5)	-5·6 (-10·5–-2·8)	-5·0 (-10·8–-2·8)
Particulate matter pollution	10·5 (2·8–20·3)	9·0 (5·7–12·7)	6·1 (3·1–9·3)	3·3 (0·9–6·2)	-3·8 (-7·7–-0·6)	-4·9 (-9·3–-3·1)	-5·7 (-12·8–-2·9)
Ambient particulate matter pollution	16·2 (4·1–30·9)	13·8 (9·0–19·3)	9·3 (4·8–14·1)	5·0 (1·3–9·4)	-3·8 (-7·8–-0·6)	-4·9 (-9·4–-3·2)	-5·7 (-12·8–-2·8)
Household air pollution from solid fuels	0·0 (0·0–0·0)	0·0 (0·0–0·0)	0·0 (0·0–0·0)	0·0 (0·0–0·0)	-3·5 (-36·4–-7·2)	-4·4 (-37·7–-7·5)	-5·1 (-28·0–-6·8)
Ambient ozone pollution	26·2 (17·9–36·5)	22·9 (14·9–32·1)	17·0 (10·0–25·7)	12·3 (6·3–19·5)	-2·4 (-4·1–-1·3)	-3·0 (-5·1–-1·3)	-2·9 (-6·8–0·0)
Ambient nitrogen dioxide pollution	77·2 (0·0–100·0)	72·6 (0·0–100·0)	31·8 (0·0–84·2)	19·2 (0·0–63·4)	-4·5 (-13·9–0·0)	-6·3 (-19·8–0·0)	-4·6 (-20·1–0·0)
Non-optimal temperature	29·9 (22·7–40·8)	29·8 (23·4–39·8)	33·3 (25·0–44·4)	31·1 (23·5–42·0)	0·1 (-0·2–0·4)	0·2 (-0·2–0·7)	-0·6 (-1·3–-0·3)
High temperature	23·7 (13·9–34·5)	19·4 (10·5–30·2)	32·7 (21·3–44·5)	28·0 (17·7–39·3)	0·5 (0·2–1·1)	1·8 (0·9–3·0)	-1·4 (-2·3–-0·8)
Low temperature	27·1 (23·8–30·5)	28·6 (25·1–31·7)	28·4 (25·5–31·6)	27·6 (24·6–30·9)	0·1 (-0·1–0·2)	-0·2 (-0·4–0·0)	-0·3 (-0·6–-0·1)
Other environmental risks	30·2 (7·5–48·6)	28·2 (7·5–47·0)	25·1 (7·5–44·0)	22·0 (7·5–40·9)	-1·0 (-1·8–0·0)	-1·2 (-2·1–0·0)	-1·2 (-2·3–0·0)
Residential radon	30·1 (0·0–86·7)	30·1 (0·0–86·7)	30·1 (0·0–86·7)	30·1 (0·0–86·7)	0·0 (0·0–0·0)	0·0 (0·0–0·0)	0·0 (0·0–0·0)
Lead exposure	30·2 (0·0–38·1)	27·3 (0·0–34·1)	22·8 (0·0–28·6)	18·3 (0·0–23·0)	-1·6 (-2·0–0·0)	-1·9 (-2·4–0·0)	-2·0 (-2·8–0·0)
Occupational risks	3·1 (2·6–4·1)	3·1 (2·6–4·1)	2·9 (2·4–3·8)	2·9 (2·3–3·8)	-0·2 (-0·5–0·0)	-0·4 (-0·7–0·0)	0·0 (-0·5–0·4)
Occupational carcinogens	1·0 (0·5–1·9)	1·1 (0·5–2·1)	1·0 (0·5–2·0)	1·0 (0·5–2·0)	0·1 (-0·3–0·4)	-0·1 (-0·6–0·2)	0·1 (-0·5–0·7)
Occupational exposure to asbestos	4·9 (4·4–5·5)	5·0 (4·5–5·5)	4·7 (4·2–5·1)	4·3 (3·4–5·4)	-0·4 (-1·2–0·4)	-0·7 (-1·9–0·5)	-0·6 (-2·8–1·4)
Occupational exposure to arsenic	0·6 (0·0–1·4)	0·6 (0·0–1·4)	0·5 (0·0–1·2)	0·5 (0·0–1·2)	-0·5 (-1·0–0·0)	-0·5 (-1·1–0·2)	0·0 (-0·9–0·9)
Occupational exposure to benzene	1·1 (0·1–3·1)	1·2 (0·1–3·6)	1·2 (0·1–3·5)	1·2 (0·1–3·5)	0·4 (0·0–0·7)	0·1 (-0·3–0·5)	0·3 (-0·3–0·9)
Occupational exposure to beryllium	0·0 (0·0–0·0)	0·0 (0·0–0·0)	0·0 (0·0–0·0)	0·0 (0·0–0·0)	0·2 (-0·1–0·4)	0·1 (-0·3–0·4)	0·4 (0·0–0·9)
Occupational exposure to cadmium	0·1 (0·1–0·1)	0·1 (0·1–0·1)	0·1 (0·1–0·1)	0·1 (0·1–0·1)	-0·4 (-0·8–0·0)	-0·4 (-1·0–0·2)	0·1 (-0·7–0·9)
Occupational exposure to chromium	0·2 (0·2–0·2)	0·2 (0·2–0·2)	0·2 (0·1–0·2)	0·2 (0·1–0·2)	-0·3 (-0·6–0·1)	-0·3 (-0·8–0·2)	0·2 (-0·5–0·9)
Occupational exposure to diesel engine exhaust	0·7 (0·6–0·7)	0·7 (0·7–0·8)	0·7 (0·7–0·7)	0·8 (0·7–0·8)	0·4 (0·1–0·8)	0·3 (-0·2–0·8)	0·7 (0·0–1·5)
Occupational exposure to formaldehyde	0·3 (0·3–0·4)	0·3 (0·3–0·4)	0·3 (0·3–0·3)	0·3 (0·3–0·3)	-0·5 (-0·9–0·0)	-0·5 (-1·1–0·1)	0·0 (-0·9–0·8)
Occupational exposure to nickel	0·5 (0·0–1·9)	0·4 (0·0–1·7)	0·4 (0·0–1·6)	0·4 (0·0–1·6)	-0·6 (-1·2–0·0)	-0·5 (-1·3–0·2)	0·0 (-1·1–1·0)
Occupational exposure to polycyclic aromatic hydrocarbons	0·3 (0·3–0·4)	0·3 (0·3–0·4)	0·3 (0·3–0·3)	0·3 (0·3–0·3)	-0·2 (-0·5–0·2)	-0·3 (-0·7–0·2)	0·2 (-0·5–0·8)
Occupational exposure to silica	4·0 (0·6–12·8)	3·7 (0·6–11·5)	3·3 (0·5–10·4)	3·4 (0·6–10·7)	-0·6 (-1·1–-0·1)	-0·4 (-1·1–0·2)	0·1 (-0·9–1·1)
Occupational exposure to sulphuric acid	0·9 (0·2–2·8)	0·8 (0·2–2·7)	0·8 (0·2–2·4)	0·8 (0·2–2·4)	-0·4 (-0·8–0·1)	-0·4 (-1·0–0·2)	0·1 (-0·7–1·0)
Occupational exposure to trichloroethylene	0·1 (0·1–0·1)	0·1 (0·1–0·1)	0·1 (0·1–0·1)	0·1 (0·1–0·1)	0·0 (-0·3–0·3)	-0·1 (-0·4–0·4)	0·4 (-0·2–1·0)
Occupational asthmagens	18·4 (16·1–20·9)	18·4 (16·1–20·8)	16·8 (14·8–19·0)	17·1 (15·0–19·5)	-0·2 (-0·6–0·1)	-0·3 (-0·8–0·1)	0·2 (-0·5–0·8)
Occupational particulate matter, gases, and fumes	7·4 (5·7–9·6)	7·1 (5·4–9·1)	6·5 (5·0–8·5)	6·0 (4·6–7·7)	-0·7 (-1·0–-0·4)	-0·8 (-1·2–-0·5)	-0·8 (-1·3–-0·4)
Occupational noise	7·0 (6·4–7·7)	7·1 (6·5–7·8)	6·9 (6·4–7·5)	6·7 (6·2–7·2)	-0·2 (-0·4–0·0)	-0·3 (-0·5–-0·1)	-0·3 (-0·5–-0·1)
Occupational injuries	--	--	--	--	--	--	--
Occupational ergonomic factors	7·8 (6·6–9·7)	7·9 (6·6–9·6)	7·2 (6·1–8·8)	7·4 (6·2–9·2)	-0·2 (-0·6–0·2)	-0·3 (-0·9–0·2)	0·2 (-0·5–1·0)
**Behavioural risks**	**32·1 (28·5–36·1)**	**30·2 (26·7–34·1)**	**28·0 (25·0–31·6)**	**25·9 (22·9–29·8)**	**-0·7 (-1·1–-0·3)**	**-0·7 (-1·2–-0·2)**	**-0·7 (-1·4–0·0)**
Child and maternal malnutrition	10·0 (6·8–14·1)	8·5 (5·9–11·9)	8·7 (6·1–12·0)	8·9 (6·0–12·8)	-0·4 (-1·2–0·5)	0·2 (-1·1–1·3)	0·2 (-1·7–2·1)
Suboptimal breastfeeding	68·8 (65·9–71·3)	66·6 (64·0–69·6)	62·8 (60·1–65·9)	62·1 (59·2–65·2)	-0·3 (-0·5–-0·2)	-0·3 (-0·5–-0·1)	-0·1 (-0·4–0·2)
Non-exclusive breastfeeding	64·1 (55·4–71·6)	59·4 (52·0–66·3)	48·5 (41·4–55·7)	46·0 (38·4–53·5)	-1·1 (-1·6–-0·5)	-1·2 (-2·0–-0·6)	-0·5 (-1·4–0·5)
Discontinued breastfeeding	88·7 (86·7–91·2)	86·3 (83·5–89·1)	84·3 (81·1–87·4)	84·5 (80·8–88·1)	-0·2 (-0·3–0·0)	-0·1 (-0·3–0·1)	0·0 (-0·3–0·3)
Child growth failure	1·2 (0·4–2·7)	1·1 (0·4–2·5)	1·0 (0·3–2·1)	0·9 (0·3–1·9)	-1·0 (-1·4–-0·6)	-1·1 (-1·6–-0·6)	-0·8 (-1·5–0·1)
Child underweight	2·5 (1·6–3·4)	2·3 (1·6–3·2)	2·0 (1·3–2·7)	1·8 (1·2–2·5)	-1·0 (-1·5–-0·4)	-1·2 (-1·8–-0·6)	-0·6 (-1·5–0·5)
Child wasting	0·8 (0·5–1·3)	0·8 (0·5–1·2)	0·8 (0·5–1·2)	0·7 (0·5–1·2)	-0·3 (-0·6–0·1)	-0·2 (-0·6–0·3)	-0·3 (-0·9–0·4)
Child stunting	3·5 (2·7–4·4)	3·2 (2·5–4·0)	2·8 (2·2–3·5)	2·5 (1·9–3·2)	-1·0 (-1·6–-0·5)	-1·2 (-2·0–-0·4)	-0·9 (-2·2–0·3)
Low birth weight and short gestation	16·7 (14·7–19·1)	18·8 (16·4–21·1)	19·7 (17·3–22·2)	19·8 (17·3–22·3)	0·6 (0·3–0·8)	0·3 (-0·1–0·7)	0·1 (-0·7–0·8)
Short gestation	30·0 (26·5–33·6)	33·7 (29·9–37·9)	33·2 (29·5–37·4)	32·8 (28·8–37·0)	0·3 (0·0–0·5)	-0·1 (-0·6–0·3)	-0·1 (-0·9–0·8)
Low birth weight	13·4 (12·3–14·6)	14·5 (13·3–15·8)	15·7 (14·3–16·9)	15·9 (14·5–17·4)	0·6 (0·3–0·8)	0·5 (0·0–0·9)	0·1 (-0·5–0·9)

Iron deficiency	3·6 (2·5–5·0)	3·1 (2·2–4·2)	3·1 (2·3–4·3)	3·2 (2·2–4·5)	-0·4 (-1·2–0·5)	0·2 (-1·0–1·3)	0·1 (-1·6–2·0)
Vitamin A deficiency	0·8 (0·0–1·7)	1·0 (0·0–2·2)	0·6 (0·0–1·4)	0·4 (0·0–0·9)	-2·3 (-4·6–0·0)	-4·8 (-7·8–0·0)	-4·4 (-8·6–0·0)
Zinc deficiency	1·8 (0·0–7·4)	1·1 (0·0–4·6)	0·9 (0·0–4·2)	0·9 (0·0–3·8)	-2·1 (-44·8–1·3)	-0·8 (-2·9–4·3)	-0·2 (-4·5–8·6)
Tobacco	42·4 (40·8–43·8)	36·8 (35·4–38·2)	28·9 (27·9–30·0)	24·4 (22·8–26·2)	-1·8 (-2·0–-1·6)	-1·9 (-2·3–-1·6)	-1·5 (-2·2–-0·9)
Smoking	40·6 (38·2–42·5)	34·8 (32·6–36·7)	27·7 (26·1–29·4)	23·3 (21·2–25·5)	-1·8 (-2·1–-1·5)	-1·9 (-2·3–-1·5)	-1·6 (-2·3–-0·8)
Chewing tobacco	0·7 (0·5–0·9)	0·9 (0·6–1·1)	0·9 (0·7–1·3)	1·0 (0·7–1·4)	1·1 (-0·3–2·5)	0·7 (-1·2–2·7)	0·4 (-2·3–3·2)
Second-hand smoke	34·0 (32·0–35·4)	29·5 (27·4–30·9)	23·0 (21·5–24·1)	19·8 (18·0–21·5)	-1·7 (-2·0–-1·5)	-1·9 (-2·3–-1·5)	-1·4 (-2·1–-0·6)
High alcohol use	30·5 (21·7–42·1)	29·8 (21·2–41·3)	30·8 (23·0–41·0)	30·6 (21·6–40·4)	0·0 (-1·0–1·0)	0·1 (-1·2–1·5)	-0·1 (-2·0–1·8)
Drug use	1·0 (0·3–2·3)	1·2 (0·8–2·0)	2·1 (1·8–2·7)	5·4 (4·4–6·6)	5·4 (2·3–9·5)	7·1 (4·0–9·8)	8·6 (5·3–11·0)
Dietary risks	36·4 (27·7–47·6)	39·8 (30·5–51·6)	42·5 (32·9–54·5)	42·8 (33·0–55·1)	0·5 (0·3–0·8)	0·3 (0·0–0·6)	0·1 (-0·4–0·5)
Diet low in fruits	34·2 (30·2–37·4)	33·7 (29·1–36·6)	34·8 (30·2–38·0)	35·7 (30·7–39·9)	0·1 (-0·2–0·5)	0·3 (-0·2–0·8)	0·2 (-0·6–1·0)
Diet low in vegetables	26·3 (15·3–33·8)	28·2 (16·6–36·1)	31·9 (17·6–39·5)	32·1 (18·0–40·5)	0·6 (0·3–1·1)	0·6 (0·2–1·1)	0·1 (-0·7–0·8)
Diet low in legumes	37·7 (0·0–47·1)	38·1 (0·0–47·4)	41·8 (0·0–52·0)	42·2 (0·0–53·4)	0·4 (0·0–0·7)	0·5 (0·0–0·9)	0·1 (-0·6–0·8)
Diet low in whole grains	44·4 (36·8–50·9)	47·3 (39·6–54·0)	49·3 (40·4–56·7)	49·9 (41·3–57·2)	0·4 (0·1–0·6)	0·3 (-0·1–0·6)	0·1 (-0·5–0·7)
Diet low in nuts and seeds	12·0 (8·5–16·5)	7·1 (4·5–10·4)	4·8 (3·0–7·4)	5·0 (2·9–7·8)	-2·8 (-4·6–-1·2)	-1·7 (-4·2–0·8)	0·4 (-3·8–3·9)
Diet low in milk	35·8 (31·1–40·4)	33·5 (29·0–38·1)	31·4 (27·1–35·5)	30·9 (26·5–36·2)	-0·5 (-1·0–0·1)	-0·4 (-1·1–0·3)	-0·1 (-1·1–0·7)
Diet high in red meat	49·4 (0·0–68·3)	49·5 (0·0–67·7)	50·2 (0·0–68·5)	50·6 (0·0–69·2)	0·1 (-0·4–0·5)	0·1 (-0·5–0·7)	0·1 (-1·0–1·0)
Diet high in processed meat	46·3 (36·7–51·9)	57·3 (46·1–63·6)	65·2 (51·8–73·2)	63·8 (50·3–72·0)	1·0 (0·6–1·5)	0·5 (-0·1–1·0)	-0·2 (-1·0–0·6)
Diet high in sugar-sweetened beverages	36·4 (27·5–45·3)	49·7 (38·8–58·5)	56·3 (44·2–66·4)	56·6 (45·4–66·6)	1·4 (0·6–2·3)	0·6 (-0·3–1·6)	0·0 (-1·3–1·3)
Diet low in fibre	38·2 (22·4–45·4)	29·2 (16·4–36·1)	25·1 (14·1–32·0)	21·7 (13·1–28·9)	-1·8 (-2·7–-0·9)	-1·4 (-2·7–-0·1)	-1·4 (-3·2–0·6)
Diet low in calcium	5·5 (4·5–6·7)	4·8 (3·9–5·9)	4·9 (3·9–6·0)	4·8 (3·9–5·9)	-0·4 (-0·7–-0·2)	0·1 (-0·2–0·3)	-0·2 (-0·5–0·2)
Diet low in seafood omega-3 fatty acids	35·0 (25·8–45·2)	43·0 (33·1–53·5)	45·0 (35·2–55·4)	39·8 (30·0–49·6)	0·4 (0·0–0·9)	-0·4 (-1·0–0·2)	-1·1 (-1·9–-0·4)
Diet low in omega-6 polyunsaturated fatty acids	34·6 (22·3–44·4)	30·6 (20·3–39·3)	22·2 (15·4–28·9)	20·6 (14·4–26·8)	-1·7 (-2·4–-0·9)	-1·9 (-2·8–-1·0)	-0·7 (-1·7–0·2)
Diet high in trans fatty acids	71·4 (64·9–78·6)	71·8 (63·5–78·7)	63·4 (55·1–70·6)	0·0 (0·0–0·0)	-65·8 (-66·1–-65·5)	-97·1 (-97·5–-96·5)	-184·2 (-185·2–-183·0)
Diet high in sodium	23·2 (0·6–69·0)	27·7 (1·9–73·6)	30·6 (3·3–74·1)	31·2 (3·4–78·1)	1·0 (0·2–6·2)	0·6 (-0·2–3·7)	0·2 (-2·2–2·0)
Intimate partner violence	21·8 (13·0–28·5)	21·6 (13·5–27·1)	19·4 (13·4–24·5)	19·7 (11·4–28·2)	-0·3 (-1·9–1·3)	-0·4 (-2·5–1·6)	0·1 (-2·2–2·5)
Childhood sexual abuse and bullying	8·9 (5·9–14·1)	10·7 (7·1–17·1)	12·3 (8·1–19·5)	10·0 (6·8–14·8)	0·4 (-0·2–1·0)	-0·3 (-1·3–0·5)	-1·9 (-3·5–-0·3)
Childhood sexual abuse	8·5 (7·0–10·1)	9·6 (8·2–11·4)	11·1 (9·5–12·8)	10·4 (8·5–12·8)	0·7 (-0·2–1·5)	0·4 (-0·8–1·4)	-0·6 (-2·1–0·9)
Bullying victimization	7·9 (3·5–15·7)	9·8 (4·5–19·0)	11·2 (5·1–21·7)	8·2 (3·6–15·4)	0·1 (-0·6–0·9)	-0·8 (-1·9–0·3)	-2·8 (-4·8–-0·8)
Unsafe sex	--	--	--	--	--	--	--
Low physical activity	19·4 (14·7–25·0)	18·5 (14·2–22·8)	20·1 (15·9–24·9)	22·9 (16·9–29·6)	0·5 (-0·4–1·5)	1·0 (-0·3–2·2)	1·2 (-0·8–2·8)
**Metabolic risks**	**24·0 (21·0–27·2)**	**29·3 (25·5–32·3)**	**33·0 (29·1–35·9)**	**35·9 (31·4–38·9)**	**1·3 (1·0–1·6)**	**1·0 (0·6–1·4)**	**0·8 (0·3–1·2)**
High fasting plasma glucose	12·0 (9·0–14·8)	15·4 (11·6–18·5)	19·9 (14·7–23·8)	23·6 (17·8–28·8)	2·2 (1·4–2·9)	2·0 (1·2–3·0)	1·6 (0·3–2·9)
High LDL cholesterol	63·8 (45·3–86·6)	53·9 (37·1–74·0)	48·2 (32·8–66·7)	46·6 (31·1–65·3)	-1·0 (-1·2–-0·8)	-0·7 (-0·9–-0·5)	-0·3 (-0·6–-0·1)
High systolic blood pressure	28·3 (18·8–39·0)	24·2 (17·1–33·0)	18·0 (11·8–26·0)	25·3 (16·0–37·3)	-0·4 (-1·3–0·6)	0·2 (-1·0–1·3)	3·1 (0·8–5·4)
High body-mass index	27·1 (22·9–31·7)	33·5 (28·4–37·6)	37·8 (32·3–41·7)	40·7 (34·4–44·6)	1·3 (1·0–1·6)	0·9 (0·6–1·3)	0·7 (0·2–1·1)
Low bone mineral density	21·9 (15·7–28·6)	19·8 (14·2–26·4)	20·4 (14·6–27·2)	22·1 (15·8–29·1)	0·0 (-0·4–0·6)	0·5 (-0·2–1·3)	0·8 (-0·7–2·3)
Kidney dysfunction	2·8 (2·3–3·7)	2·9 (2·4–3·8)	2·9 (2·3–3·8)	3·0 (2·4–4·0)	0·1 (0·0–0·2)	0·1 (-0·1–0·3)	0·3 (0·0–0·7)

South Carolina

Risk Names	SEV 1990	SEV 2000	SEV 2010	SEV 2021	Annualised rate of change 1990 to 2021	Annualised rate of change 2000 to 2021	Annualised rate of change 2010 to 2021
**All risk factors**	**26·3 (23·5–29·5)**	**26·0 (23·3–29·1)**	**26·6 (23·6–29·8)**	**26·2 (23·2–29·2)**	**0·0 (-0·3–0·3)**	**0·0 (-0·3–0·4)**	**-0·2 (-0·6–0·3)**
**Environmental/occupational risks**	**17·7 (12·0–27·0)**	**17·8 (12·5–26·7)**	**15·1 (10·3–22·6)**	**12·0 (7·9–17·9)**	**-1·3 (-2·1–-0·5)**	**-1·9 (-2·6–-1·3)**	**-2·1 (-2·9–-1·6)**
Unsafe water, sanitation, and handwashing	2·3 (1·1–3·8)	2·0 (1·0–3·2)	1·5 (0·5–2·5)	1·2 (0·4–2·0)	-2·1 (-4·3–0·1)	-2·5 (-5·4–0·1)	-2·0 (-5·6–1·6)
Unsafe water source	3·2 (1·2–6·5)	2·9 (1·1–5·7)	1·2 (0·4–2·4)	0·9 (0·4–1·8)	-4·0 (-6·7–-1·2)	-5·5 (-9·1–-1·8)	-2·1 (-7·6–3·5)
Unsafe sanitation	8·0 (4·4–13·3)	6·1 (3·3–10·5)	4·5 (2·3–7·7)	3·4 (1·7–5·7)	-2·8 (-5·0–-0·7)	-2·8 (-5·6–-0·5)	-2·6 (-6·1–0·9)
No access to handwashing facility	1·8 (0·6–3·2)	1·7 (0·5–3·3)	1·7 (0·6–3·2)	1·5 (0·5–2·8)	-0·6 (-3·5–2·3)	-0·9 (-4·8–3·1)	-1·6 (-7·2–3·1)
Air pollution	20·2 (9·1–31·9)	18·2 (10·7–28·8)	9·5 (5·3–17·1)	5·4 (1·9–12·0)	-4·3 (-7·0–-2·2)	-5·8 (-9·0–-3·9)	-5·2 (-9·6–-2·9)
Particulate matter pollution	15·6 (6·4–26·2)	14·1 (10·4–18·3)	8·3 (5·1–11·7)	4·5 (1·8–7·6)	-4·0 (-7·1–-1·4)	-5·5 (-8·6–-4·0)	-5·6 (-10·2–-3·4)
Ambient particulate matter pollution	24·3 (10·2–40·8)	21·8 (16·6–27·3)	12·7 (8·1–17·5)	6·8 (2·8–11·3)	-4·1 (-7·2–-1·4)	-5·5 (-8·6–-4·1)	-5·6 (-10·3–-3·4)
Household air pollution from solid fuels	0·0 (0·0–0·1)	0·0 (0·0–0·1)	0·0 (0·0–0·0)	0·0 (0·0–0·0)	-5·3 (-27·7–-6·1)	-6·1 (-24·6–-5·5)	-5·3 (-22·3–-4·5)
Ambient ozone pollution	24·8 (18·8–32·6)	30·0 (23·2–39·4)	18·6 (13·1–26·1)	9·9 (5·9–15·9)	-3·0 (-3·9–-2·2)	-5·3 (-6·8–-4·2)	-5·7 (-8·1–-4·1)
Ambient nitrogen dioxide pollution	35·6 (0·0–89·9)	31·4 (0·0–85·0)	11·5 (0·0–48·7)	7·7 (0·0–41·7)	-5·0 (-20·3–0·0)	-6·7 (-28·5–0·0)	-3·7 (-23·2–0·0)
Non-optimal temperature	27·1 (20·7–36·3)	28·2 (23·2–36·7)	34·2 (27·2–44·1)	25·0 (20·1–33·1)	-0·3 (-0·6–0·0)	-0·6 (-0·9–-0·3)	-2·9 (-3·7–-2·0)
High temperature	22·1 (14·7–31·8)	17·7 (11·1–26·7)	27·3 (19·0–37·2)	16·4 (9·5–25·6)	-1·0 (-1·8–-0·5)	-0·4 (-1·1–0·0)	-4·6 (-7·3–-3·0)
Low temperature	23·5 (20·1–27·6)	27·4 (24·1–31·1)	30·7 (27·4–34·4)	23·6 (20·7–27·5)	0·0 (-0·1–0·2)	-0·7 (-1·0–-0·4)	-2·4 (-2·9–-1·7)
Other environmental risks	24·7 (7·7–38·3)	23·4 (7·7–37·3)	21·2 (7·7–35·1)	18·8 (7·7–32·6)	-0·9 (-1·5–0·0)	-1·0 (-1·7–0·0)	-1·1 (-2·0–0·0)
Residential radon	23·1 (0·0–67·9)	23·1 (0·0–67·9)	23·1 (0·0–67·9)	23·1 (0·0–67·9)	0·0 (0·0–0·0)	0·0 (0·0–0·0)	0·0 (0·0–0·0)
Lead exposure	25·4 (0·0–31·7)	23·5 (0·0–29·4)	20·3 (0·0–25·3)	16·9 (0·0–20·9)	-1·3 (-1·8–0·0)	-1·6 (-2·0–0·0)	-1·7 (-2·6–0·0)
Occupational risks	3·0 (2·5–3·8)	3·0 (2·5–3·8)	2·7 (2·2–3·6)	2·7 (2·2–3·6)	-0·3 (-0·6–0·0)	-0·4 (-0·8–-0·1)	0·1 (-0·4–0·6)
Occupational carcinogens	0·9 (0·4–1·7)	1·0 (0·4–1·8)	0·9 (0·4–1·8)	1·0 (0·4–1·8)	0·1 (-0·2–0·4)	-0·1 (-0·5–0·3)	0·3 (-0·3–0·8)
Occupational exposure to asbestos	3·7 (3·3–4·1)	4·3 (3·9–4·7)	3·7 (3·3–4·1)	3·6 (2·8–4·4)	-0·1 (-0·9–0·7)	-0·9 (-2·0–0·2)	-0·3 (-2·4–1·7)
Occupational exposure to arsenic	0·6 (0·0–1·3)	0·5 (0·0–1·3)	0·5 (0·0–1·2)	0·5 (0·0–1·2)	-0·5 (-1·0–0·0)	-0·6 (-1·3–0·1)	0·0 (-0·9–0·9)
Occupational exposure to benzene	1·0 (0·1–2·8)	1·1 (0·1–3·1)	1·1 (0·1–3·1)	1·1 (0·1–3·2)	0·4 (0·0–0·8)	0·2 (-0·3–0·7)	0·5 (-0·1–1·2)
Occupational exposure to beryllium	0·0 (0·0–0·0)	0·0 (0·0–0·0)	0·0 (0·0–0·0)	0·0 (0·0–0·0)	0·1 (-0·1–0·4)	0·0 (-0·3–0·4)	0·5 (0·1–1·0)
Occupational exposure to cadmium	0·1 (0·1–0·1)	0·1 (0·1–0·1)	0·1 (0·1–0·1)	0·1 (0·1–0·1)	-0·4 (-0·8–0·0)	-0·5 (-1·0–0·1)	0·1 (-0·7–1·0)
Occupational exposure to chromium	0·2 (0·1–0·2)	0·2 (0·1–0·2)	0·1 (0·1–0·1)	0·1 (0·1–0·2)	-0·3 (-0·6–0·1)	-0·4 (-0·8–0·2)	0·2 (-0·5–0·9)
Occupational exposure to diesel engine exhaust	0·6 (0·6–0·7)	0·6 (0·6–0·7)	0·6 (0·6–0·7)	0·7 (0·6–0·7)	0·4 (0·0–0·7)	0·3 (-0·3–0·8)	0·8 (0·1–1·6)
Occupational exposure to formaldehyde	0·3 (0·3–0·4)	0·3 (0·3–0·3)	0·3 (0·3–0·3)	0·3 (0·3–0·3)	-0·4 (-0·9–0·0)	-0·6 (-1·1–0·0)	0·0 (-0·8–0·9)
Occupational exposure to nickel	0·5 (0·0–1·8)	0·4 (0·0–1·7)	0·4 (0·0–1·5)	0·4 (0·0–1·5)	-0·6 (-1·1–0·0)	-0·7 (-1·4–0·1)	0·0 (-1·0–1·0)
Occupational exposure to polycyclic aromatic hydrocarbons	0·3 (0·3–0·3)	0·3 (0·3–0·3)	0·3 (0·3–0·3)	0·3 (0·3–0·3)	-0·2 (-0·6–0·2)	-0·3 (-0·8–0·2)	0·2 (-0·4–0·9)
Occupational exposure to silica	4·0 (0·6–13·2)	3·8 (0·6–12·4)	3·4 (0·5–11·0)	3·4 (0·5–11·1)	-0·5 (-1·0–0·0)	-0·5 (-1·2–0·1)	0·2 (-0·7–1·1)
Occupational exposure to sulphuric acid	0·8 (0·2–2·6)	0·8 (0·2–2·7)	0·7 (0·1–2·3)	0·7 (0·1–2·4)	-0·4 (-0·8–0·1)	-0·5 (-1·1–0·1)	0·1 (-0·7–1·0)
Occupational exposure to trichloroethylene	0·1 (0·1–0·1)	0·1 (0·1–0·1)	0·1 (0·1–0·1)	0·1 (0·1–0·1)	0·0 (-0·3–0·3)	-0·1 (-0·5–0·3)	0·4 (-0·1–1·0)
Occupational asthmagens	17·7 (15·3–20·1)	17·7 (15·5–20·2)	15·7 (13·8–17·9)	16·2 (13·9–18·5)	-0·3 (-0·7–0·1)	-0·4 (-0·9–0·1)	0·3 (-0·4–1·0)
Occupational particulate matter, gases, and fumes	7·2 (5·5–9·4)	7·0 (5·4–9·1)	6·5 (5·0–8·4)	6·0 (4·5–7·8)	-0·6 (-0·9–-0·3)	-0·8 (-1·1–-0·5)	-0·9 (-1·3–-0·5)
Occupational noise	6·8 (6·2–7·5)	6·8 (6·3–7·5)	6·7 (6·2–7·2)	6·4 (6·0–6·9)	-0·2 (-0·4–0·0)	-0·3 (-0·5–-0·1)	-0·4 (-0·6–-0·1)
Occupational injuries	--	--	--	--	--	--	--
Occupational ergonomic factors	8·4 (7·0–10·1)	8·2 (6·9–10·0)	7·1 (6·0–8·6)	7·4 (6·1–9·0)	-0·4 (-0·9–0·0)	-0·5 (-1·1–0·1)	0·3 (-0·4–1·1)
**Behavioural risks**	**29·4 (26·3–32·8)**	**27·9 (24·5–31·4)**	**26·7 (23·3–30·1)**	**24·7 (21·6–28·1)**	**-0·6 (-0·9–-0·2)**	**-0·6 (-1·0–-0·1)**	**-0·7 (-1·4–-0·1)**
Child and maternal malnutrition	10·7 (7·2–15·0)	9·2 (6·3–12·7)	9·2 (6·3–12·8)	9·3 (6·2–13·5)	-0·4 (-1·4–0·4)	0·1 (-1·2–1·2)	0·1 (-1·7–1·8)
Suboptimal breastfeeding	69·0 (66·5–71·7)	66·8 (64·6–69·4)	62·9 (60·4–66·0)	62·3 (59·5–65·5)	-0·3 (-0·5–-0·2)	-0·3 (-0·5–-0·2)	-0·1 (-0·4–0·2)
Non-exclusive breastfeeding	64·7 (56·4–72·6)	59·9 (53·1–66·9)	48·9 (42·2–56·2)	46·5 (39·5–54·1)	-1·1 (-1·6–-0·5)	-1·2 (-1·9–-0·5)	-0·5 (-1·5–0·5)
Discontinued breastfeeding	88·9 (86·5–91·2)	86·4 (83·6–89·3)	84·5 (81·3–87·7)	84·7 (81·0–88·0)	-0·2 (-0·3–0·0)	-0·1 (-0·3–0·1)	0·0 (-0·3–0·3)
Child growth failure	1·5 (0·5–3·5)	1·3 (0·4–3·1)	1·2 (0·4–2·6)	1·1 (0·3–2·3)	-1·1 (-1·4–-0·7)	-1·1 (-1·6–-0·5)	-0·7 (-1·5–0·1)
Child underweight	3·2 (2·0–4·5)	2·9 (1·8–3·9)	2·4 (1·5–3·3)	2·3 (1·4–3·1)	-1·1 (-1·6–-0·6)	-1·1 (-1·8–-0·4)	-0·7 (-1·7–0·4)
Child wasting	1·0 (0·6–1·5)	0·9 (0·6–1·4)	0·9 (0·6–1·4)	0·9 (0·6–1·3)	-0·4 (-0·7–-0·1)	-0·2 (-0·6–0·3)	-0·3 (-1·1–0·4)
Child stunting	4·3 (3·3–5·4)	3·9 (3·1–4·8)	3·3 (2·6–4·2)	3·0 (2·3–3·8)	-1·1 (-1·7–-0·6)	-1·2 (-1·9–-0·4)	-0·9 (-2·1–0·3)
Low birth weight and short gestation	22·4 (19·5–25·4)	24·8 (21·8–28·1)	27·6 (23·9–31·0)	25·9 (22·4–29·5)	0·5 (0·2–0·7)	0·2 (-0·2–0·6)	-0·6 (-1·2–0·1)
Short gestation	37·6 (33·1–42·6)	42·1 (37·2–47·9)	46·0 (40·3–51·7)	43·3 (37·9–48·7)	0·5 (0·2–0·8)	0·1 (-0·3–0·6)	-0·5 (-1·4–0·3)
Low birth weight	17·3 (15·7–18·9)	18·7 (17·0–20·3)	20·7 (18·8–22·6)	19·6 (17·8–21·6)	0·4 (0·2–0·6)	0·2 (-0·1–0·6)	-0·5 (-1·1–0·2)

Iron deficiency	3·9 (2·7–5·3)	3·3 (2·3–4·5)	3·4 (2·4–4·6)	3·4 (2·4–4·7)	-0·4 (-1·3–0·3)	0·1 (-1·1–1·1)	0·0 (-1·7–1·6)
Vitamin A deficiency	1·3 (0·0–2·8)	1·7 (0·0–3·6)	1·1 (0·0–2·4)	0·7 (0·0–1·4)	-2·2 (-4·5–0·0)	-4·5 (-7·3–0·0)	-4·3 (-8·1–0·1)
Zinc deficiency	2·1 (0·0–8·9)	1·2 (0·0–5·3)	1·1 (0·0–4·9)	1·1 (0·0–4·9)	-2·0 (-48·1–0·0)	-0·4 (-4·9–0·9)	0·2 (-5·8–3·4)
Tobacco	41·0 (39·4–42·5)	36·4 (35·0–37·6)	31·3 (30·3–32·4)	27·2 (25·5–28·8)	-1·3 (-1·5–-1·1)	-1·4 (-1·7–-1·1)	-1·3 (-1·8–-0·7)
Smoking	37·8 (35·6–40·0)	33·5 (31·5–35·4)	28·9 (27·4–30·5)	25·1 (23·2–27·4)	-1·3 (-1·6–-1·0)	-1·4 (-1·8–-1·0)	-1·3 (-2·0–-0·5)
Chewing tobacco	3·5 (2·9–4·1)	3·0 (2·5–3·7)	2·8 (2·1–3·5)	2·6 (1·9–3·4)	-0·9 (-2·0–0·0)	-0·7 (-2·2–0·7)	-0·6 (-2·7–1·8)
Second-hand smoke	33·5 (31·7–34·8)	29·5 (27·8–30·7)	25·5 (23·7–26·4)	22·2 (20·2–23·8)	-1·3 (-1·5–-1·1)	-1·3 (-1·7–-1·0)	-1·2 (-1·8–-0·6)
High alcohol use	23·8 (16·7–33·7)	23·1 (16·3–33·0)	24·4 (17·0–34·2)	24·6 (16·8–34·8)	0·1 (-1·0–1·1)	0·3 (-1·1–1·6)	0·1 (-1·8–1·7)
Drug use	1·0 (0·6–1·8)	1·4 (1·1–1·7)	2·5 (2·0–3·0)	5·4 (3·8–6·9)	5·4 (2·7–7·6)	6·5 (4·3–7·9)	7·2 (5·2–8·5)
Dietary risks	39·1 (30·0–50·6)	41·7 (32·1–53·3)	43·9 (34·4–55·8)	43·9 (34·8–55·8)	0·4 (0·1–0·6)	0·2 (-0·1–0·5)	0·0 (-0·5–0·5)
Diet low in fruits	41·2 (36·8–44·6)	37·7 (32·8–40·7)	36·1 (32·0–39·5)	37·1 (32·2–41·7)	-0·3 (-0·7–0·0)	-0·1 (-0·6–0·4)	0·2 (-0·6–1·0)
Diet low in vegetables	31·4 (18·7–39·4)	31·1 (18·6–39·1)	35·1 (20·0–43·2)	35·6 (21·4–44·2)	0·4 (0·1–0·7)	0·6 (0·2–1·1)	0·1 (-0·6–0·9)
Diet low in legumes	39·5 (0·0–49·8)	39·5 (0·0–48·9)	43·9 (0·0–55·7)	44·4 (0·0–56·7)	0·4 (0·0–0·7)	0·6 (0·0–1·1)	0·1 (-0·6–0·8)
Diet low in whole grains	45·6 (38·1–52·2)	49·0 (40·9–56·0)	50·9 (42·4–59·0)	51·2 (42·8–59·1)	0·4 (0·1–0·6)	0·2 (-0·2–0·6)	0·0 (-0·5–0·6)
Diet low in nuts and seeds	15·0 (11·0–19·5)	9·1 (6·1–12·6)	6·8 (4·3–9·8)	6·9 (4·0–10·4)	-2·5 (-4·2–-0·9)	-1·3 (-3·5–1·0)	0·2 (-3·2–3·6)
Diet low in milk	38·9 (34·8–43·7)	35·9 (31·4–41·0)	34·6 (30·4–39·2)	33·9 (29·2–39·3)	-0·4 (-0·9–0·0)	-0·3 (-0·9–0·3)	-0·2 (-1·0–0·7)
Diet high in red meat	52·6 (0·0–71·2)	53·6 (0·0–73·2)	53·8 (0·0–74·1)	53·5 (0·0–74·1)	0·1 (-0·4–0·5)	0·0 (-0·5–0·6)	-0·1 (-0·9–0·8)
Diet high in processed meat	47·8 (37·8–53·5)	59·5 (48·1–66·5)	65·9 (54·7–73·4)	63·6 (50·2–72·5)	0·9 (0·5–1·3)	0·3 (-0·3–0·9)	-0·3 (-1·2–0·4)
Diet high in sugar-sweetened beverages	33·6 (25·7–42·0)	48·1 (37·8–56·9)	53·2 (41·4–63·3)	52·5 (40·2–63·0)	1·4 (0·6–2·3)	0·4 (-0·6–1·4)	-0·1 (-1·6–1·3)
Diet low in fibre	42·3 (23·1–49·7)	32·6 (17·5–39·7)	29·4 (15·7–36·0)	25·5 (14·5–32·9)	-1·6 (-2·4–-0·8)	-1·2 (-2·3–0·0)	-1·3 (-3·1–0·5)
Diet low in calcium	6·3 (5·1–7·7)	5·2 (4·2–6·4)	5·6 (4·5–6·9)	5·4 (4·4–6·7)	-0·5 (-0·7–-0·2)	0·2 (-0·1–0·5)	-0·3 (-0·6–0·1)
Diet low in seafood omega-3 fatty acids	39·8 (30·6–50·0)	46·4 (36·3–57·1)	50·0 (38·9–60·9)	44·5 (34·3–55·1)	0·4 (0·0–0·8)	-0·2 (-0·7–0·3)	-1·1 (-1·8–-0·4)
Diet low in omega-6 polyunsaturated fatty acids	39·2 (25·1–50·0)	34·1 (22·0–43·6)	25·9 (18·0–33·6)	23·8 (16·2–30·9)	-1·6 (-2·3–-0·9)	-1·7 (-2·6–-0·9)	-0·8 (-1·8–0·2)
Diet high in trans fatty acids	71·4 (63·9–78·5)	71·7 (63·9–78·9)	63·3 (54·7–70·9)	0·0 (0·0–0·0)	-65·8 (-66·1–-65·4)	-97·1 (-97·6–-96·6)	-184·2 (-185·3–-182·9)
Diet high in sodium	23·9 (0·6–69·8)	28·8 (1·8–78·0)	31·9 (3·4–77·5)	32·3 (3·1–78·7)	1·0 (0·2–6·3)	0·5 (-0·1–3·6)	0·1 (-2·4–1·7)
Intimate partner violence	32·2 (18·4–40·7)	32·2 (19·4–39·9)	29·2 (19·7–35·9)	27·1 (14·7–35·6)	-0·6 (-2·1–0·6)	-0·8 (-2·8–0·7)	-0·7 (-3·3–1·2)
Childhood sexual abuse and bullying	9·2 (6·1–14·3)	11·1 (7·4–17·1)	12·7 (8·3–19·6)	9·9 (6·8–15·0)	0·2 (-0·3–0·8)	-0·5 (-1·4–0·4)	-2·3 (-4·0–-0·8)
Childhood sexual abuse	9·2 (7·8–10·8)	10·4 (9·0–12·2)	12·1 (10·5–14·1)	11·0 (9·0–13·3)	0·6 (-0·2–1·3)	0·3 (-0·8–1·3)	-0·9 (-2·2–0·6)
Bullying victimization	7·9 (3·5–15·8)	9·8 (4·5–19·2)	11·2 (5·0–21·6)	7·6 (3·4–15·3)	-0·1 (-0·8–0·6)	-1·2 (-2·2–-0·1)	-3·5 (-5·4–-1·6)
Unsafe sex	--	--	--	--	--	--	--
Low physical activity	19·6 (14·9–25·2)	18·8 (14·4–23·8)	20·5 (16·3–25·8)	23·3 (18·0–30·2)	0·6 (-0·3–1·5)	1·0 (-0·2–2·4)	1·2 (-0·7–2·9)
**Metabolic risks**	**26·8 (23·5–30·1)**	**32·2 (28·5–35·4)**	**36·5 (32·4–39·6)**	**40·1 (35·4–43·0)**	**1·3 (1·0–1·6)**	**1·0 (0·7–1·4)**	**0·8 (0·4–1·3)**
High fasting plasma glucose	13·4 (10·4–16·1)	18·1 (13·8–21·7)	23·6 (17·7–28·1)	26·7 (19·9–31·7)	2·2 (1·6–2·9)	1·8 (1·0–2·8)	1·1 (0·0–2·2)
High LDL cholesterol	63·4 (44·3–86·8)	53·8 (37·1–74·2)	48·3 (32·7–67·5)	46·8 (31·4–65·0)	-1·0 (-1·2–-0·8)	-0·7 (-0·9–-0·5)	-0·3 (-0·5–-0·1)
High systolic blood pressure	31·0 (21·1–42·5)	26·8 (19·2–36·2)	20·3 (13·4–28·9)	28·8 (18·8–41·1)	-0·2 (-1·2–0·6)	0·3 (-0·8–1·4)	3·2 (0·9–5·5)
High body-mass index	30·5 (25·8–35·0)	36·9 (31·6–41·0)	41·8 (36·0–45·5)	45·4 (38·7–49·0)	1·3 (0·9–1·6)	1·0 (0·6–1·3)	0·7 (0·3–1·2)
Low bone mineral density	21·8 (15·4–28·6)	19·6 (13·7–26·2)	20·3 (14·5–27·6)	22·3 (16·1–29·2)	0·1 (-0·4–0·6)	0·6 (-0·1–1·4)	0·9 (-0·5–2·3)
Kidney dysfunction	3·0 (2·5–4·0)	3·1 (2·6–4·1)	3·1 (2·5–4·1)	3·2 (2·6–4·2)	0·2 (0·1–0·3)	0·1 (0·0–0·3)	0·4 (0·1–0·7)

South Dakota

Risk Names	SEV 1990	SEV 2000	SEV 2010	SEV 2021	Annualised rate of change 1990 to 2021	Annualised rate of change 2000 to 2021	Annualised rate of change 2010 to 2021
**All risk factors**	**25·6 (22·6–28·8)**	**25·2 (22·4–28·4)**	**25·4 (22·5–28·8)**	**25·6 (22·4–28·9)**	**0·0 (-0·3–0·3)**	**0·1 (-0·3–0·4)**	**0·1 (-0·5–0·6)**
**Environmental/occupational risks**	**15·9 (10·2–27·0)**	**15·4 (10·0–25·0)**	**14·0 (9·0–23·5)**	**12·9 (8·2–21·4)**	**-0·7 (-1·3–-0·2)**	**-0·8 (-1·2–-0·4)**	**-0·8 (-1·2–-0·3)**
Unsafe water, sanitation, and handwashing	2·1 (1·0–3·4)	1·9 (0·8–3·1)	1·5 (0·5–2·5)	1·2 (0·4–2·0)	-1·8 (-4·2–0·2)	-2·1 (-5·0–0·8)	-2·1 (-6·1–2·1)
Unsafe water source	2·9 (1·1–5·9)	2·6 (0·9–5·0)	1·1 (0·4–2·3)	0·8 (0·4–1·7)	-4·0 (-6·8–-1·1)	-5·3 (-8·8–-1·2)	-2·3 (-7·7–3·1)
Unsafe sanitation	6·9 (3·6–11·6)	5·1 (2·7–8·7)	4·2 (2·2–7·4)	2·9 (1·5–5·2)	-2·8 (-5·1–-0·7)	-2·7 (-5·6–-0·1)	-3·3 (-6·6–-0·2)
No access to handwashing facility	1·8 (0·6–3·3)	1·8 (0·6–3·4)	1·9 (0·6–3·5)	1·6 (0·5–3·1)	-0·4 (-3·4–2·4)	-0·7 (-4·6–3·5)	-1·6 (-6·9–4·2)
Air pollution	15·1 (3·2–26·3)	13·5 (3·9–23·6)	8·0 (2·0–17·0)	7·4 (1·2–16·7)	-2·3 (-5·2–-0·7)	-2·9 (-6·3–-1·6)	-0·7 (-5·7–0·3)
Particulate matter pollution	6·9 (1·1–14·5)	6·1 (3·3–9·6)	3·9 (1·5–6·9)	2·6 (0·6–5·4)	-3·1 (-7·6–1·2)	-4·0 (-8·5–-2·3)	-3·7 (-10·4–-0·9)
Ambient particulate matter pollution	10·5 (1·7–22·0)	9·3 (5·0–14·3)	5·9 (2·3–10·3)	4·0 (1·0–8·1)	-3·2 (-7·6–1·2)	-4·1 (-8·5–-2·3)	-3·7 (-10·4–-0·8)
Household air pollution from solid fuels	0·0 (0·0–0·1)	0·0 (0·0–0·0)	0·0 (0·0–0·0)	0·0 (0·0–0·0)	-5·8 (-41·9–-8·4)	-6·9 (-41·9–-9·1)	-6·6 (-30·0–-7·2)
Ambient ozone pollution	9·6 (5·1–15·3)	15·0 (9·3–21·7)	14·0 (8·7–20·5)	13·1 (7·8–20·3)	1·0 (0·0–2·2)	-0·6 (-1·9–0·6)	-0·6 (-3·5–1·8)
Ambient nitrogen dioxide pollution	51·6 (0·0–100·0)	45·6 (0·0–100·0)	25·0 (0·0–72·4)	28·0 (0·0–77·0)	-2·0 (-5·0–0·0)	-2·3 (-6·0–0·0)	1·0 (0·0–2·1)
Non-optimal temperature	34·0 (26·7–44·7)	32·2 (25·5–42·5)	31·9 (25·4–41·6)	33·9 (26·2–44·5)	0·0 (-0·2–0·2)	0·2 (0·0–0·6)	0·5 (0·1–1·1)
High temperature	36·3 (23·5–50·3)	32·3 (20·5–45·6)	33·3 (21·5–46·7)	39·5 (26·0–53·5)	0·3 (0·0–0·6)	1·0 (0·5–1·6)	1·6 (0·8–2·5)
Low temperature	29·3 (26·5–32·8)	28·3 (25·7–31·8)	28·0 (25·5–31·6)	28·8 (25·9–32·5)	-0·1 (-0·1–0·0)	0·1 (-0·1–0·2)	0·3 (0·0–0·5)
Other environmental risks	26·1 (13·3–46·3)	24·9 (12·3–45·5)	22·8 (10·9–43·3)	20·6 (9·4–40·7)	-0·8 (-1·5–0·0)	-0·9 (-1·8–0·0)	-0·9 (-2·0–0·0)
Residential radon	31·9 (0·0–100·0)	31·9 (0·0–100·0)	31·9 (0·0–100·0)	31·9 (0·0–100·0)	0·0 (0·0–0·0)	0·0 (0·0–0·0)	0·0 (0·0–0·0)
Lead exposure	23·5 (0·0–29·3)	21·7 (0·0–27·1)	18·7 (0·0–23·4)	15·5 (0·0–19·3)	-1·3 (-1·8–0·0)	-1·6 (-2·2–0·0)	-1·7 (-2·5–0·0)
Occupational risks	2·9 (2·4–3·7)	3·0 (2·5–4·0)	3·0 (2·4–3·9)	3·0 (2·4–3·9)	0·1 (-0·2–0·4)	-0·1 (-0·5–0·2)	0·0 (-0·4–0·5)
Occupational carcinogens	0·9 (0·4–1·8)	1·0 (0·4–2·0)	1·0 (0·4–2·1)	1·0 (0·4–2·0)	0·4 (0·1–0·6)	0·1 (-0·3–0·4)	0·2 (-0·3–0·7)
Occupational exposure to asbestos	3·4 (3·0–3·8)	3·6 (3·2–4·0)	3·2 (2·9–3·6)	3·4 (2·6–4·2)	0·0 (-0·8–0·8)	-0·3 (-1·4–0·8)	0·3 (-1·8–2·3)
Occupational exposure to arsenic	0·5 (0·0–1·1)	0·5 (0·0–1·3)	0·5 (0·0–1·2)	0·5 (0·0–1·2)	0·2 (-0·3–0·6)	-0·1 (-0·8–0·5)	0·0 (-0·8–0·9)
Occupational exposure to benzene	1·1 (0·1–3·2)	1·3 (0·1–3·8)	1·3 (0·1–3·8)	1·3 (0·1–3·8)	0·5 (0·1–0·8)	0·2 (-0·2–0·7)	0·3 (-0·4–0·8)
Occupational exposure to beryllium	0·0 (0·0–0·0)	0·0 (0·0–0·0)	0·0 (0·0–0·0)	0·0 (0·0–0·0)	0·3 (0·1–0·5)	0·1 (-0·2–0·4)	0·3 (-0·1–0·7)
Occupational exposure to cadmium	0·1 (0·1–0·1)	0·1 (0·1–0·1)	0·1 (0·1–0·1)	0·1 (0·1–0·1)	0·2 (-0·2–0·6)	-0·1 (-0·6–0·5)	0·1 (-0·6–0·9)
Occupational exposure to chromium	0·1 (0·1–0·2)	0·2 (0·1–0·2)	0·2 (0·1–0·2)	0·2 (0·1–0·2)	0·3 (0·0–0·6)	0·0 (-0·5–0·5)	0·2 (-0·5–0·8)
Occupational exposure to diesel engine exhaust	0·7 (0·7–0·8)	0·8 (0·7–0·8)	0·8 (0·7–0·8)	0·8 (0·8–0·9)	0·4 (0·1–0·8)	0·3 (-0·2–0·8)	0·5 (-0·1–1·3)
Occupational exposure to formaldehyde	0·3 (0·3–0·3)	0·3 (0·3–0·3)	0·3 (0·3–0·3)	0·3 (0·3–0·3)	0·2 (-0·2–0·6)	-0·1 (-0·7–0·4)	0·0 (-0·7–0·8)
Occupational exposure to nickel	0·4 (0·0–1·5)	0·4 (0·0–1·6)	0·4 (0·0–1·5)	0·4 (0·0–1·6)	0·0 (-0·5–0·6)	-0·2 (-1·0–0·7)	0·0 (-1·0–1·1)
Occupational exposure to polycyclic aromatic hydrocarbons	0·3 (0·3–0·3)	0·3 (0·3–0·3)	0·3 (0·3–0·3)	0·3 (0·3–0·3)	0·3 (0·0–0·6)	0·0 (-0·4–0·5)	0·1 (-0·4–0·8)
Occupational exposure to silica	3·5 (0·5–11·1)	3·5 (0·6–11·4)	3·4 (0·5–10·8)	3·4 (0·6–11·0)	0·0 (-0·5–0·4)	-0·1 (-0·8–0·5)	0·1 (-0·8–1·1)
Occupational exposure to sulphuric acid	0·7 (0·2–2·4)	0·8 (0·2–2·6)	0·8 (0·2–2·5)	0·8 (0·2–2·6)	0·2 (-0·2–0·6)	-0·1 (-0·7–0·4)	0·1 (-0·8–0·9)
Occupational exposure to trichloroethylene	0·1 (0·1–0·1)	0·1 (0·1–0·1)	0·1 (0·1–0·1)	0·1 (0·1–0·1)	0·4 (0·1–0·6)	0·1 (-0·3–0·5)	0·3 (-0·3–0·8)
Occupational asthmagens	16·4 (14·3–18·8)	17·7 (15·6–20·0)	17·1 (15·0–19·3)	17·5 (15·4–19·8)	0·2 (-0·2–0·6)	-0·1 (-0·6–0·4)	0·2 (-0·5–0·9)
Occupational particulate matter, gases, and fumes	7·0 (5·3–9·1)	6·9 (5·3–8·9)	6·6 (5·1–8·6)	6·1 (4·8–8·0)	-0·4 (-0·7–-0·2)	-0·5 (-0·8–-0·3)	-0·6 (-1·0–-0·3)
Occupational noise	7·4 (6·9–8·0)	7·5 (7·0–8·0)	7·4 (7·0–7·9)	7·2 (6·8–7·7)	-0·1 (-0·3–0·1)	-0·2 (-0·4–0·0)	-0·3 (-0·5–-0·1)
Occupational injuries	--	--	--	--	--	--	--
Occupational ergonomic factors	9·6 (8·0–11·5)	9·4 (7·9–11·4)	8·6 (7·3–10·3)	8·6 (7·2–10·5)	-0·3 (-0·8–0·1)	-0·4 (-1·0–0·1)	0·0 (-0·8–0·7)
**Behavioural risks**	**29·8 (26·4–33·5)**	**28·2 (25·0–31·8)**	**26·1 (22·7–29·9)**	**24·9 (21·5–28·9)**	**-0·6 (-0·9–-0·2)**	**-0·6 (-1·1–-0·1)**	**-0·4 (-1·1–0·2)**
Child and maternal malnutrition	10·1 (7·1–14·0)	8·8 (6·2–11·9)	9·0 (6·2–12·6)	9·2 (6·0–13·1)	-0·3 (-1·1–0·5)	0·2 (-0·8–1·4)	0·2 (-1·3–1·9)
Suboptimal breastfeeding	68·9 (66·1–71·3)	66·7 (64·3–69·4)	62·9 (60·0–65·7)	62·2 (59·1–65·3)	-0·3 (-0·5–-0·2)	-0·3 (-0·6–-0·1)	-0·1 (-0·4–0·2)
Non-exclusive breastfeeding	64·4 (56·7–71·6)	59·7 (52·9–66·8)	48·9 (42·3–55·5)	46·6 (39·6–54·5)	-1·0 (-1·6–-0·5)	-1·2 (-1·9–-0·5)	-0·4 (-1·5–0·6)
Discontinued breastfeeding	88·7 (86·4–91·2)	86·3 (83·4–89·3)	84·3 (81·3–87·6)	84·5 (81·2–88·2)	-0·2 (-0·3–0·0)	-0·1 (-0·3–0·1)	0·0 (-0·2–0·3)
Child growth failure	1·3 (0·4–3·0)	1·2 (0·4–2·7)	1·1 (0·3–2·4)	1·0 (0·3–2·2)	-0·9 (-1·3–-0·5)	-0·9 (-1·6–-0·4)	-0·6 (-1·4–0·2)
Child underweight	2·8 (1·7–3·8)	2·6 (1·7–3·5)	2·2 (1·4–3·0)	2·1 (1·3–2·9)	-0·9 (-1·4–-0·4)	-1·0 (-1·7–-0·3)	-0·5 (-1·5–0·4)
Child wasting	0·9 (0·6–1·4)	0·8 (0·5–1·3)	0·8 (0·5–1·3)	0·8 (0·5–1·3)	-0·3 (-0·6–0·0)	-0·1 (-0·5–0·4)	-0·2 (-0·9–0·6)
Child stunting	3·8 (3·0–4·8)	3·5 (2·8–4·4)	3·1 (2·4–4·0)	2·8 (2·2–3·7)	-1·0 (-1·5–-0·4)	-1·1 (-1·8–-0·3)	-0·8 (-1·9–0·4)
Low birth weight and short gestation	15·1 (13·3–17·0)	16·5 (14·5–18·6)	18·5 (16·1–21·0)	19·4 (17·0–21·6)	0·8 (0·5–1·1)	0·8 (0·4–1·1)	0·4 (-0·4–1·2)
Short gestation	28·1 (24·6–31·8)	30·0 (26·3–34·1)	33·0 (29·0–37·5)	32·7 (28·9–36·6)	0·5 (0·2–0·8)	0·4 (0·0–0·8)	-0·1 (-0·9–0·8)
Low birth weight	12·3 (11·4–13·4)	13·2 (12·1–14·4)	14·6 (13·3–16·0)	15·7 (14·5–17·1)	0·8 (0·5–1·0)	0·8 (0·4–1·2)	0·7 (-0·1–1·3)

Iron deficiency	3·6 (2·6–4·8)	3·1 (2·3–4·1)	3·2 (2·2–4·4)	3·3 (2·2–4·5)	-0·3 (-1·1–0·4)	0·2 (-0·8–1·3)	0·2 (-1·3–1·7)
Vitamin A deficiency	1·1 (0·0–2·5)	1·5 (0·0–3·1)	1·0 (0·0–2·2)	0·5 (0·0–1·2)	-2·4 (-4·6–0·0)	-4·8 (-7·8–0·0)	-5·4 (-9·3–0·0)
Zinc deficiency	2·1 (0·0–8·8)	1·2 (0·0–5·1)	1·0 (0·0–4·4)	1·0 (0·0–4·0)	-2·5 (-47·9–0·0)	-0·9 (-4·3–4·1)	-0·3 (-6·6–5·0)
Tobacco	37·8 (36·2–39·2)	34·5 (33·3–35·7)	28·8 (27·8–29·8)	25·9 (24·2–27·5)	-1·2 (-1·4–-1·0)	-1·4 (-1·7–-1·1)	-1·0 (-1·5–-0·5)
Smoking	36·2 (33·8–38·4)	31·8 (29·8–33·7)	26·6 (25·2–28·2)	23·9 (21·8–26·2)	-1·3 (-1·7–-1·1)	-1·4 (-1·8–-0·9)	-1·0 (-1·8–-0·3)
Chewing tobacco	2·8 (2·2–3·6)	3·5 (2·7–4·5)	3·7 (2·6–4·9)	3·5 (2·4–4·8)	0·7 (-0·8–1·9)	0·0 (-1·8–1·7)	-0·4 (-3·0–2·4)
Second-hand smoke	30·1 (28·4–31·3)	28·0 (26·3–29·1)	23·1 (21·7–24·1)	21·0 (19·1–22·6)	-1·2 (-1·4–-0·9)	-1·4 (-1·7–-1·0)	-0·9 (-1·5–-0·3)
High alcohol use	27·8 (19·6–38·1)	26·4 (18·7–36·5)	26·4 (18·1–35·9)	27·0 (18·5–38·0)	-0·1 (-1·2–0·9)	0·1 (-1·4–1·6)	0·2 (-1·6–2·2)
Drug use	0·7 (0·4–1·3)	0·7 (0·5–1·1)	1·2 (1·0–1·5)	2·8 (2·2–3·6)	4·5 (1·9–7·1)	6·5 (3·6–8·7)	7·7 (4·9–9·6)
Dietary risks	33·4 (24·0–44·2)	34·8 (25·3–47·2)	36·7 (27·1–49·1)	37·2 (27·1–49·7)	0·3 (0·0–0·6)	0·3 (-0·1–0·7)	0·1 (-0·4–0·7)
Diet low in fruits	37·6 (33·1–40·8)	35·2 (30·0–38·0)	35·3 (31·6–38·2)	36·2 (30·9–40·3)	-0·1 (-0·5–0·2)	0·1 (-0·4–0·6)	0·2 (-0·6–1·1)
Diet low in vegetables	13·2 (6·8–20·9)	5·9 (2·6–10·3)	3·8 (1·6–6·8)	4·8 (1·9–8·5)	-3·3 (-5·2–-1·8)	-1·0 (-3·8–1·4)	2·2 (-2·4–6·1)
Diet low in legumes	39·1 (0·0–48·8)	39·0 (0·0–49·3)	42·3 (0·0–53·0)	42·6 (0·0–53·7)	0·3 (-0·1–0·6)	0·4 (-0·1–0·9)	0·1 (-0·7–0·8)
Diet low in whole grains	45·9 (38·6–52·0)	49·4 (41·5–55·9)	51·4 (43·1–58·9)	51·9 (42·7–59·8)	0·4 (0·1–0·7)	0·2 (-0·1–0·6)	0·1 (-0·4–0·7)
Diet low in nuts and seeds	14·5 (10·7–18·6)	8·3 (5·5–11·8)	5·4 (3·4–8·3)	5·4 (3·1–8·4)	-3·2 (-4·9–-1·5)	-2·0 (-4·6–0·4)	0·0 (-3·8–3·5)
Diet low in milk	37·6 (33·5–42·3)	34·3 (29·5–38·8)	31·6 (27·5–35·9)	30·6 (26·2–35·5)	-0·7 (-1·2–-0·2)	-0·5 (-1·2–0·1)	-0·3 (-1·2–0·6)
Diet high in red meat	49·0 (0·0–66·6)	49·9 (0·0–68·0)	50·8 (0·0–69·5)	51·3 (0·0–69·8)	0·1 (-0·4–0·6)	0·1 (-0·5–0·8)	0·1 (-0·9–0·9)
Diet high in processed meat	42·4 (33·6–47·7)	53·7 (42·9–60·1)	61·7 (49·4–69·0)	60·8 (49·0–69·4)	1·2 (0·7–1·6)	0·6 (0·0–1·2)	-0·1 (-1·0–0·7)
Diet high in sugar-sweetened beverages	34·7 (26·9–42·9)	49·7 (40·4–58·3)	57·3 (46·4–66·7)	58·3 (46·2–68·5)	1·7 (0·8–2·5)	0·8 (-0·2–1·6)	0·1 (-1·1–1·3)
Diet low in fibre	41·5 (23·6–49·0)	31·2 (18·1–37·8)	26·4 (15·1–32·6)	22·4 (12·6–29·1)	-2·0 (-3·1–-1·1)	-1·6 (-3·2–-0·4)	-1·5 (-3·7–0·4)
Diet low in calcium	6·0 (4·9–7·4)	4·9 (3·9–6·0)	4·9 (3·9–6·1)	4·7 (3·8–5·8)	-0·7 (-1·0–-0·5)	-0·1 (-0·4–0·1)	-0·4 (-0·7–0·0)
Diet low in seafood omega-3 fatty acids	38·9 (28·9–48·9)	44·8 (35·0–54·9)	46·2 (36·3–56·3)	40·8 (30·2–51·3)	0·2 (-0·3–0·6)	-0·4 (-1·0–0·1)	-1·1 (-2·0–-0·3)
Diet low in omega-6 polyunsaturated fatty acids	38·4 (24·4–48·9)	32·8 (21·9–42·9)	23·3 (16·3–30·3)	21·3 (14·6–28·1)	-1·9 (-2·7–-1·0)	-2·1 (-3·0–-1·1)	-0·8 (-2·0–0·1)
Diet high in trans fatty acids	71·3 (64·2–78·7)	71·8 (63·0–79·0)	63·3 (55·7–71·2)	0·0 (0·0–0·0)	-65·8 (-66·1–-65·4)	-97·1 (-97·6–-96·5)	-184·2 (-185·3–-183·1)
Diet high in sodium	23·0 (0·7–67·6)	27·8 (2·0–73·9)	30·8 (3·5–74·8)	31·4 (3·3–76·4)	1·0 (0·2–6·2)	0·6 (-0·1–3·7)	0·2 (-2·2–2·0)
Intimate partner violence	27·2 (14·1–35·1)	27·1 (14·7–34·0)	24·4 (16·6–30·3)	23·0 (14·4–31·1)	-0·5 (-2·0–0·7)	-0·8 (-2·7–0·8)	-0·5 (-2·9–1·4)
Childhood sexual abuse and bullying	8·9 (5·8–14·0)	10·9 (7·1–17·2)	12·4 (8·0–19·5)	10·6 (6·9–16·8)	0·6 (-0·1–1·1)	-0·1 (-1·0–0·7)	-1·4 (-2·9–0·1)
Childhood sexual abuse	8·5 (7·2–10·1)	9·6 (8·3–11·1)	11·0 (9·6–12·6)	10·4 (8·4–12·8)	0·7 (-0·1–1·5)	0·4 (-0·6–1·5)	-0·5 (-2·1–1·1)
Bullying victimization	8·0 (3·5–15·9)	10·0 (4·6–19·6)	11·3 (5·1–22·2)	9·1 (3·8–17·8)	0·4 (-0·3–1·2)	-0·4 (-1·5–0·6)	-1·9 (-3·9–0·0)
Unsafe sex	--	--	--	--	--	--	--
Low physical activity	22·1 (16·8–28·0)	21·8 (16·9–27·0)	22·4 (17·6–27·6)	24·7 (18·9–31·5)	0·4 (-0·5–1·2)	0·6 (-0·5–1·7)	0·9 (-0·8–2·6)
**Metabolic risks**	**25·4 (21·9–29·0)**	**30·7 (26·8–33·7)**	**35·1 (31·0–38·3)**	**38·0 (33·2–41·2)**	**1·3 (0·9–1·6)**	**1·0 (0·7–1·4)**	**0·7 (0·2–1·2)**
High fasting plasma glucose	12·6 (9·6–15·4)	16·7 (12·8–20·0)	21·4 (16·5–25·8)	25·6 (19·6–30·6)	2·3 (1·5–3·0)	2·0 (1·1–3·0)	1·6 (0·2–3·1)
High LDL cholesterol	63·3 (44·8–86·4)	53·7 (36·8–74·1)	47·9 (32·4–66·8)	46·6 (31·6–64·9)	-1·0 (-1·2–-0·8)	-0·7 (-0·9–-0·5)	-0·2 (-0·5–0·0)
High systolic blood pressure	28·1 (18·9–39·7)	24·1 (16·6–33·4)	18·1 (11·7–26·5)	25·6 (15·8–37·1)	-0·3 (-1·2–0·5)	0·3 (-0·9–1·3)	3·2 (0·9–5·4)
High body-mass index	29·0 (24·1–34·0)	35·5 (29·8–39·6)	40·6 (34·6–44·6)	43·2 (36·6–47·2)	1·3 (0·9–1·6)	0·9 (0·6–1·3)	0·6 (0·1–1·0)
Low bone mineral density	21·3 (15·1–28·4)	19·3 (13·8–26·1)	20·0 (14·3–26·6)	21·9 (15·9–29·1)	0·1 (-0·4–0·6)	0·6 (-0·1–1·4)	0·9 (-0·4–2·2)
Kidney dysfunction	2·8 (2·3–3·7)	2·9 (2·4–3·8)	2·9 (2·3–3·8)	3·0 (2·4–4·0)	0·2 (0·1–0·3)	0·1 (0·0–0·3)	0·4 (0·1–0·7)

Tennessee

Risk Names	SEV 1990	SEV 2000	SEV 2010	SEV 2021	Annualised rate of change 1990 to 2021	Annualised rate of change 2000 to 2021	Annualised rate of change 2010 to 2021
**All risk factors**	**26·1 (23·5–29·0)**	**26·0 (23·3–28·6)**	**26·7 (24·0–29·6)**	**26·6 (23·8–29·4)**	**0·1 (-0·2–0·3)**	**0·1 (-0·2–0·4)**	**0·0 (-0·5–0·4)**
**Environmental/occupational risks**	**19·0 (12·9–29·1)**	**18·6 (13·1–28·7)**	**16·3 (11·1–25·4)**	**13·4 (8·8–21·3)**	**-1·1 (-1·9–-0·4)**	**-1·6 (-2·1–-1·0)**	**-1·8 (-2·3–-1·1)**
Unsafe water, sanitation, and handwashing	2·4 (1·2–3·9)	2·1 (1·0–3·5)	1·6 (0·6–2·8)	1·3 (0·4–2·2)	-2·0 (-4·4–-0·2)	-2·5 (-5·4–0·2)	-2·3 (-5·8–1·2)
Unsafe water source	3·3 (1·3–6·3)	3·0 (1·1–6·3)	1·2 (0·5–2·5)	0·9 (0·4–1·8)	-4·1 (-6·8–-1·3)	-5·6 (-9·4–-1·7)	-2·5 (-7·6–2·6)
Unsafe sanitation	8·3 (4·4–13·9)	6·4 (3·3–11·2)	4·9 (2·5–8·5)	3·4 (1·8–6·0)	-2·8 (-5·1–-0·8)	-2·9 (-5·7–-0·4)	-3·3 (-6·5–0·0)
No access to handwashing facility	1·8 (0·5–3·5)	1·9 (0·6–3·7)	1·9 (0·6–3·6)	1·6 (0·5–3·0)	-0·5 (-3·7–2·4)	-0·9 (-4·7–3·1)	-1·7 (-6·7–3·4)
Air pollution	21·2 (8·7–33·1)	18·9 (10·3–29·6)	10·8 (5·6–19·2)	6·2 (2·3–13·7)	-3·9 (-6·4–-2·1)	-5·3 (-8·3–-3·5)	-5·0 (-9·1–-2·9)
Particulate matter pollution	14·8 (6·0–24·8)	13·3 (9·8–17·4)	8·4 (5·4–12·0)	4·6 (1·9–7·8)	-3·7 (-6·5–-1·2)	-5·0 (-8·0–-3·6)	-5·4 (-9·8–-3·4)
Ambient particulate matter pollution	23·0 (9·7–39·1)	20·5 (15·6–25·9)	12·9 (8·6–17·9)	7·1 (2·9–11·7)	-3·8 (-6·7–-1·3)	-5·1 (-8·0–-3·7)	-5·4 (-9·9–-3·4)
Household air pollution from solid fuels	0·0 (0·0–0·1)	0·0 (0·0–0·1)	0·0 (0·0–0·0)	0·0 (0·0–0·0)	-4·4 (-31·1–-6·9)	-4·3 (-26·3–-5·9)	-3·8 (-24·4–-6·0)
Ambient ozone pollution	25·9 (19·4–35·0)	32·5 (25·3–42·5)	20·5 (14·7–29·5)	10·9 (6·3–17·0)	-2·8 (-3·7–-2·1)	-5·2 (-6·8–-4·1)	-5·8 (-8·1–-4·1)
Ambient nitrogen dioxide pollution	45·5 (0·0–100·0)	39·2 (0·0–95·2)	18·3 (0·0–62·1)	11·6 (0·0–49·9)	-4·4 (-15·4–0·0)	-5·8 (-21·2–0·0)	-4·1 (-21·6–0·0)
Non-optimal temperature	27·9 (21·6–37·4)	28·6 (22·9–37·5)	33·5 (26·4–44·1)	26·0 (21·0–34·6)	-0·2 (-0·5–0·0)	-0·4 (-0·7–-0·2)	-2·3 (-3·2–-1·4)
High temperature	24·8 (16·8–34·5)	24·0 (16·0–33·3)	30·9 (22·0–40·8)	20·3 (13·3–29·0)	-0·6 (-1·1–-0·4)	-0·8 (-1·7–-0·3)	-3·8 (-5·3–-2·5)
Low temperature	24·5 (20·9–28·6)	26·0 (22·6–29·8)	29·2 (26·1–32·6)	24·1 (20·8–28·1)	-0·1 (-0·2–0·1)	-0·4 (-0·6–-0·2)	-1·8 (-2·2–-1·3)
Other environmental risks	27·0 (10·2–45·3)	25·4 (10·2–44·0)	23·0 (10·5–41·8)	20·3 (9·7–38·7)	-0·9 (-1·6–0·0)	-1·1 (-1·9–0·0)	-1·2 (-2·2–0·0)
Residential radon	25·8 (0·0–83·4)	25·8 (0·0–83·4)	25·8 (0·0–83·4)	25·8 (0·0–83·4)	0·0 (0·0–0·0)	0·0 (0·0–0·0)	0·0 (0·0–0·0)
Lead exposure	27·5 (0·0–35·1)	25·2 (0·0–31·3)	21·7 (0·0–27·1)	17·7 (0·0–22·2)	-1·4 (-1·9–0·0)	-1·7 (-2·1–0·0)	-1·8 (-2·7–0·0)
Occupational risks	2·9 (2·4–3·8)	3·0 (2·5–3·8)	2·7 (2·2–3·6)	2·7 (2·2–3·6)	-0·2 (-0·5–0·1)	-0·4 (-0·8–-0·1)	0·0 (-0·4–0·5)
Occupational carcinogens	0·9 (0·4–1·7)	1·0 (0·4–1·9)	0·9 (0·4–1·8)	1·0 (0·4–1·8)	0·2 (-0·2–0·5)	0·0 (-0·5–0·3)	0·3 (-0·3–0·8)
Occupational exposure to asbestos	3·3 (3·0–3·6)	3·4 (3·1–3·8)	3·5 (3·1–3·8)	3·3 (2·7–4·1)	0·1 (-0·7–0·8)	-0·1 (-1·2–0·9)	-0·4 (-2·4–1·6)
Occupational exposure to arsenic	0·5 (0·0–1·3)	0·5 (0·0–1·4)	0·5 (0·0–1·2)	0·5 (0·0–1·2)	-0·4 (-0·8–0·1)	-0·6 (-1·2–0·1)	0·0 (-0·9–0·9)
Occupational exposure to benzene	1·0 (0·1–2·8)	1·1 (0·1–3·3)	1·1 (0·1–3·2)	1·2 (0·1–3·3)	0·4 (0·0–0·8)	0·2 (-0·2–0·6)	0·5 (-0·2–1·1)
Occupational exposure to beryllium	0·0 (0·0–0·0)	0·0 (0·0–0·0)	0·0 (0·0–0·0)	0·0 (0·0–0·0)	0·2 (-0·1–0·4)	0·0 (-0·3–0·4)	0·5 (0·1–1·0)
Occupational exposure to cadmium	0·1 (0·1–0·1)	0·1 (0·1–0·1)	0·1 (0·1–0·1)	0·1 (0·1–0·1)	-0·3 (-0·7–0·1)	-0·5 (-1·0–0·1)	0·1 (-0·7–1·0)
Occupational exposure to chromium	0·2 (0·1–0·2)	0·2 (0·1–0·2)	0·1 (0·1–0·2)	0·1 (0·1–0·2)	-0·2 (-0·6–0·2)	-0·4 (-0·8–0·1)	0·2 (-0·5–0·9)
Occupational exposure to diesel engine exhaust	0·6 (0·6–0·7)	0·7 (0·6–0·7)	0·6 (0·6–0·7)	0·7 (0·6–0·8)	0·3 (-0·1–0·6)	0·2 (-0·3–0·8)	0·8 (0·1–1·5)
Occupational exposure to formaldehyde	0·3 (0·3–0·3)	0·3 (0·3–0·3)	0·3 (0·3–0·3)	0·3 (0·3–0·3)	-0·3 (-0·7–0·1)	-0·5 (-1·1–0·0)	0·0 (-0·8–0·9)
Occupational exposure to nickel	0·5 (0·0–1·8)	0·4 (0·0–1·8)	0·4 (0·0–1·5)	0·4 (0·0–1·5)	-0·5 (-1·0–0·0)	-0·6 (-1·3–0·1)	0·0 (-1·1–1·0)
Occupational exposure to polycyclic aromatic hydrocarbons	0·3 (0·3–0·3)	0·3 (0·3–0·3)	0·3 (0·3–0·3)	0·3 (0·3–0·3)	-0·1 (-0·5–0·2)	-0·3 (-0·7–0·2)	0·2 (-0·4–0·9)
Occupational exposure to silica	4·0 (0·6–12·7)	3·8 (0·6–12·1)	3·3 (0·5–10·4)	3·4 (0·5–10·8)	-0·5 (-1·0–0·0)	-0·5 (-1·2–0·1)	0·1 (-0·8–1·1)
Occupational exposure to sulphuric acid	0·8 (0·2–2·6)	0·8 (0·2–2·7)	0·7 (0·2–2·4)	0·7 (0·1–2·4)	-0·3 (-0·7–0·1)	-0·5 (-1·0–0·1)	0·1 (-0·8–1·0)
Occupational exposure to trichloroethylene	0·1 (0·1–0·1)	0·1 (0·1–0·1)	0·1 (0·1–0·1)	0·1 (0·1–0·1)	0·0 (-0·3–0·3)	-0·1 (-0·5–0·3)	0·4 (-0·2–1·0)
Occupational asthmagens	17·5 (15·3–20·0)	17·8 (15·6–20·1)	15·9 (13·8–18·1)	16·3 (14·1–18·7)	-0·2 (-0·6–0·1)	-0·4 (-0·9–0·0)	0·2 (-0·4–0·9)
Occupational particulate matter, gases, and fumes	7·0 (5·3–9·1)	6·8 (5·2–8·9)	6·4 (4·9–8·5)	5·9 (4·5–7·7)	-0·6 (-0·9–-0·3)	-0·7 (-1·1–-0·4)	-0·8 (-1·3–-0·4)
Occupational noise	6·8 (6·2–7·4)	6·8 (6·3–7·4)	6·7 (6·2–7·2)	6·4 (6·1–6·9)	-0·2 (-0·4–0·0)	-0·3 (-0·5–-0·1)	-0·4 (-0·6–-0·1)
Occupational injuries	--	--	--	--	--	--	--
Occupational ergonomic factors	8·3 (6·9–10·1)	8·2 (6·9–10·1)	7·2 (6·1–8·7)	7·4 (6·1–9·0)	-0·4 (-0·9–0·0)	-0·5 (-1·1–0·1)	0·2 (-0·5–0·9)
**Behavioural risks**	**29·8 (27·2–33·1)**	**28·3 (25·4–31·4)**	**27·2 (24·3–30·6)**	**25·8 (22·9–29·2)**	**-0·5 (-0·8–-0·2)**	**-0·4 (-0·8–0·1)**	**-0·5 (-1·0–0·1)**
Child and maternal malnutrition	10·5 (7·1–14·5)	9·1 (6·2–12·7)	9·2 (6·5–12·5)	9·3 (6·3–13·4)	-0·4 (-1·3–0·5)	0·1 (-1·2–1·3)	0·1 (-1·8–1·8)
Suboptimal breastfeeding	69·0 (66·4–71·5)	66·9 (64·5–69·4)	63·1 (60·6–65·7)	62·3 (59·5–65·4)	-0·3 (-0·5–-0·2)	-0·3 (-0·5–-0·2)	-0·1 (-0·4–0·2)
Non-exclusive breastfeeding	64·7 (57·2–72·1)	60·0 (53·1–67·2)	49·2 (42·7–55·6)	46·6 (39·5–53·8)	-1·1 (-1·5–-0·5)	-1·2 (-1·9–-0·6)	-0·5 (-1·6–0·4)
Discontinued breastfeeding	88·9 (86·6–91·3)	86·5 (83·8–89·3)	84·6 (81·6–87·9)	84·7 (81·3–87·9)	-0·2 (-0·3–0·0)	-0·1 (-0·3–0·1)	0·0 (-0·3–0·3)
Child growth failure	1·4 (0·5–3·3)	1·3 (0·4–3·0)	1·2 (0·4–2·7)	1·1 (0·3–2·4)	-1·0 (-1·4–-0·6)	-1·0 (-1·6–-0·5)	-1·2 (-2·0–-0·5)
Child underweight	3·0 (1·9–4·3)	2·8 (1·8–3·8)	2·5 (1·6–3·5)	2·2 (1·3–3·0)	-1·0 (-1·5–-0·5)	-1·1 (-1·8–-0·3)	-1·2 (-2·1–-0·1)
Child wasting	0·9 (0·6–1·5)	0·9 (0·6–1·4)	0·9 (0·6–1·4)	0·9 (0·5–1·3)	-0·3 (-0·6–0·0)	-0·1 (-0·6–0·4)	-0·7 (-1·4–0·0)
Child stunting	4·1 (3·1–5·1)	3·8 (2·9–4·7)	3·4 (2·7–4·4)	3·0 (2·3–3·8)	-1·0 (-1·6–-0·5)	-1·1 (-1·9–-0·4)	-1·4 (-2·5–-0·3)
Low birth weight and short gestation	23·0 (20·2–26·2)	24·5 (21·6–27·8)	24·5 (21·6–28·0)	26·6 (23·5–29·9)	0·5 (0·2–0·7)	0·4 (0·0–0·8)	0·7 (0·0–1·4)
Short gestation	40·0 (35·5–44·7)	43·6 (38·7–48·9)	43·6 (38·6–49·3)	45·7 (40·6–51·4)	0·4 (0·1–0·7)	0·2 (-0·2–0·7)	0·4 (-0·5–1·3)
Low birth weight	17·6 (16·3–19·2)	18·3 (16·6–20·1)	18·3 (16·8–20·1)	20·1 (18·5–21·9)	0·4 (0·2–0·7)	0·5 (0·1–0·8)	0·8 (0·2–1·5)

Iron deficiency	3·8 (2·7–5·1)	3·3 (2·3–4·5)	3·4 (2·5–4·5)	3·4 (2·4–4·8)	-0·3 (-1·1–0·4)	0·2 (-1·0–1·3)	0·1 (-1·6–1·6)
Vitamin A deficiency	1·3 (0·0–2·8)	1·8 (0·0–3·7)	1·2 (0·0–2·6)	0·7 (0·0–1·5)	-2·0 (-4·1–0·2)	-4·5 (-7·3–0·0)	-4·8 (-8·5–0·0)
Zinc deficiency	2·0 (0·0–8·6)	1·1 (0·0–4·9)	1·1 (0·0–4·4)	1·0 (0·0–4·5)	-2·2 (-47·8–0·0)	-0·5 (-2·1–7·3)	-0·1 (-3·1–2·5)
Tobacco	44·7 (43·0–46·3)	41·6 (40·2–42·9)	36·2 (35·0–37·4)	31·7 (29·6–33·6)	-1·1 (-1·3–-0·9)	-1·3 (-1·6–-1·0)	-1·2 (-1·8–-0·7)
Smoking	40·7 (38·5–42·7)	36·5 (34·7–38·3)	32·0 (30·5–33·8)	28·5 (26·0–31·2)	-1·2 (-1·4–-0·9)	-1·2 (-1·6–-0·8)	-1·1 (-1·8–-0·3)
Chewing tobacco	4·0 (3·2–4·9)	4·1 (3·2–5·1)	4·1 (3·1–5·3)	4·0 (3·0–5·3)	0·0 (-1·1–1·1)	-0·1 (-1·5–1·6)	-0·2 (-2·4–2·0)
Second-hand smoke	38·5 (36·4–40·0)	36·9 (34·7–38·3)	32·4 (30·1–33·8)	28·3 (25·9–30·3)	-1·0 (-1·2–-0·8)	-1·3 (-1·6–-0·9)	-1·2 (-1·8–-0·7)
High alcohol use	22·4 (16·6–31·2)	20·6 (15·3–29·0)	22·2 (16·3–31·4)	24·0 (16·4–33·6)	0·2 (-0·9–1·1)	0·7 (-0·6–1·9)	0·7 (-0·9–2·4)
Drug use	1·0 (0·7–1·6)	1·5 (1·3–1·8)	2·9 (2·1–3·6)	7·0 (4·6–9·2)	6·2 (3·6–8·2)	7·4 (5·3–8·6)	8·0 (6·6–9·1)
Dietary risks	38·9 (29·5–50·2)	41·4 (32·0–52·7)	43·5 (34·2–55·7)	43·8 (34·3–56·6)	0·4 (0·2–0·6)	0·3 (0·0–0·6)	0·0 (-0·4–0·5)
Diet low in fruits	41·3 (35·8–44·8)	35·9 (31·6–39·0)	35·1 (30·8–38·1)	37·2 (31·9–41·5)	-0·3 (-0·7–0·0)	0·2 (-0·3–0·7)	0·5 (-0·2–1·4)
Diet low in vegetables	31·1 (18·7–39·0)	30·9 (18·7–39·3)	35·3 (20·6–43·7)	35·0 (21·3–43·5)	0·4 (0·0–0·7)	0·6 (0·1–1·1)	-0·1 (-0·8–0·8)
Diet low in legumes	39·0 (0·0–48·8)	38·8 (0·0–49·2)	43·3 (0·0–53·7)	43·6 (0·0–54·5)	0·4 (0·0–0·7)	0·5 (0·0–1·0)	0·1 (-0·6–0·7)
Diet low in whole grains	44·9 (36·6–51·5)	48·6 (40·2–55·3)	50·4 (41·3–58·0)	50·7 (41·5–58·4)	0·4 (0·1–0·6)	0·2 (-0·2–0·6)	0·1 (-0·5–0·6)
Diet low in nuts and seeds	14·0 (10·2–18·2)	7·9 (5·1–11·2)	6·0 (3·6–9·0)	6·3 (3·6–9·6)	-2·6 (-4·4–-1·2)	-1·1 (-3·8–1·1)	0·4 (-3·3–4·0)
Diet low in milk	38·1 (34·1–42·3)	34·6 (30·7–39·1)	33·6 (29·6–38·0)	33·1 (28·3–38·4)	-0·5 (-0·9–0·0)	-0·2 (-0·9–0·4)	-0·1 (-1·0–0·6)
Diet high in red meat	48·9 (0·0–67·4)	50·7 (0·0–69·7)	50·9 (0·0–70·1)	51·1 (0·0–71·0)	0·1 (-0·4–0·6)	0·0 (-0·7–0·6)	0·0 (-0·9–0·9)
Diet high in processed meat	47·5 (38·3–53·0)	59·2 (48·1–66·0)	65·0 (53·4–72·5)	63·1 (51·1–71·2)	0·9 (0·5–1·3)	0·3 (-0·2–0·8)	-0·3 (-1·0–0·4)
Diet high in sugar-sweetened beverages	34·0 (26·4–42·6)	49·6 (40·2–58·9)	54·3 (44·1–64·7)	54·2 (42·8–65·0)	1·5 (0·7–2·4)	0·4 (-0·5–1·4)	0·0 (-1·4–1·4)
Diet low in fibre	41·3 (23·9–49·3)	30·9 (16·9–38·0)	28·0 (14·6–35·0)	24·2 (14·0–31·3)	-1·7 (-2·6–-0·9)	-1·2 (-2·4–-0·1)	-1·3 (-3·1–0·4)
Diet low in calcium	6·1 (4·9–7·4)	5·0 (4·0–6·1)	5·4 (4·3–6·6)	5·3 (4·3–6·4)	-0·5 (-0·7–-0·2)	0·3 (0·0–0·6)	-0·2 (-0·6–0·2)
Diet low in seafood omega-3 fatty acids	38·9 (28·9–49·4)	44·6 (34·8–55·2)	48·5 (38·3–59·3)	42·9 (32·3–52·9)	0·3 (-0·1–0·7)	-0·2 (-0·7–0·3)	-1·1 (-1·9–-0·3)
Diet low in omega-6 polyunsaturated fatty acids	38·4 (24·6–48·9)	32·6 (22·0–41·7)	24·7 (17·3–31·6)	22·6 (15·5–29·6)	-1·7 (-2·4–-0·9)	-1·7 (-2·6–-0·9)	-0·8 (-1·7–0·2)
Diet high in trans fatty acids	71·4 (62·3–78·4)	71·8 (64·4–79·0)	63·4 (56·4–71·1)	0·0 (0·0–0·0)	-65·8 (-66·1–-65·3)	-97·1 (-97·6–-96·6)	-184·3 (-185·3–-183·2)
Diet high in sodium	23·8 (0·6–70·2)	28·7 (2·0–76·2)	31·8 (3·3–78·3)	32·2 (3·1–78·2)	1·0 (0·2–6·4)	0·5 (-0·2–3·7)	0·1 (-2·5–2·0)
Intimate partner violence	31·6 (19·7–39·8)	31·7 (20·1–39·5)	28·9 (18·1–35·5)	26·9 (15·3–35·7)	-0·5 (-1·8–0·7)	-0·8 (-2·4–0·8)	-0·6 (-2·8–1·3)
Childhood sexual abuse and bullying	8·7 (5·5–13·9)	10·2 (6·6–16·2)	11·7 (7·5–18·5)	9·2 (6·0–14·4)	0·2 (-0·4–0·7)	-0·5 (-1·3–0·3)	-2·2 (-3·6–-0·8)
Childhood sexual abuse	7·9 (6·6–9·5)	8·2 (7·2–9·5)	9·4 (8·5–10·5)	9·0 (7·6–10·8)	0·4 (-0·4–1·3)	0·4 (-0·6–1·5)	-0·4 (-1·8–0·9)
Bullying victimization	7·9 (3·5–15·8)	9·8 (4·6–19·2)	11·2 (5·1–21·9)	7·8 (3·5–15·2)	-0·1 (-0·8–0·6)	-1·1 (-2·1–-0·2)	-3·3 (-5·2–-1·7)
Unsafe sex	--	--	--	--	--	--	--
Low physical activity	23·7 (18·3–30·4)	23·6 (18·7–28·6)	24·6 (19·6–29·9)	26·3 (20·2–32·9)	0·3 (-0·5–1·2)	0·5 (-0·5–1·6)	0·6 (-0·9–2·1)
**Metabolic risks**	**25·6 (22·3–28·8)**	**32·2 (28·3–35·3)**	**36·9 (32·3–39·8)**	**39·9 (35·1–42·9)**	**1·4 (1·1–1·8)**	**1·0 (0·7–1·4)**	**0·7 (0·2–1·2)**
High fasting plasma glucose	13·1 (10·0–16·0)	17·8 (13·3–21·2)	23·0 (17·0–27·3)	26·1 (19·4–31·3)	2·2 (1·5–2·9)	1·8 (1·0–2·7)	1·2 (0·0–2·3)
High LDL cholesterol	63·1 (44·8–86·0)	53·7 (37·2–74·5)	48·3 (33·1–67·8)	46·9 (31·8–65·8)	-1·0 (-1·2–-0·8)	-0·7 (-0·9–-0·5)	-0·3 (-0·5–0·0)
High systolic blood pressure	32·6 (22·5–43·8)	27·7 (20·0–38·0)	20·8 (13·8–29·5)	29·5 (18·6–43·9)	-0·3 (-1·5–0·8)	0·3 (-1·2–1·7)	3·2 (0·6–5·9)
High body-mass index	28·8 (24·4–33·2)	36·9 (31·5–40·7)	42·2 (36·0–45·9)	45·1 (38·3–48·7)	1·4 (1·1–1·8)	1·0 (0·6–1·3)	0·6 (0·2–1·0)
Low bone mineral density	18·3 (12·6–24·9)	18·1 (12·5–25·0)	19·8 (13·8–26·8)	18·4 (13·0–25·0)	0·0 (-0·5–0·5)	0·1 (-0·7–0·9)	-0·7 (-2·2–0·8)
Kidney dysfunction	2·9 (2·4–3·9)	3·1 (2·5–4·0)	3·0 (2·5–4·0)	3·1 (2·6–4·2)	0·2 (0·1–0·3)	0·1 (0·0–0·3)	0·3 (0·1–0·7)

Texas

Risk Names	SEV 1990	SEV 2000	SEV 2010	SEV 2021	Annualised rate of change 1990 to 2021	Annualised rate of change 2000 to 2021	Annualised rate of change 2010 to 2021
**All risk factors**	**27·6 (24·6–30·9)**	**27·5 (24·4–31·2)**	**27·5 (24·6–31·2)**	**27·3 (24·1–30·8)**	**0·0 (-0·4–0·3)**	**0·0 (-0·4–0·4)**	**-0·1 (-0·6–0·5)**
**Environmental/occupational risks**	**16·8 (11·3–25·4)**	**17·1 (11·7–25·2)**	**15·0 (9·8–22·7)**	**11·9 (7·7–17·9)**	**-1·1 (-1·9–-0·4)**	**-1·7 (-2·3–-1·2)**	**-2·1 (-2·8–-1·6)**
Unsafe water, sanitation, and handwashing	2·3 (1·2–3·8)	2·2 (1·1–3·6)	1·6 (0·6–2·8)	1·2 (0·4–2·1)	-2·1 (-4·3–0·0)	-2·9 (-5·8–-0·4)	-2·6 (-6·0–0·9)
Unsafe water source	3·3 (1·1–6·6)	3·2 (1·2–6·4)	1·2 (0·5–2·4)	0·9 (0·4–1·7)	-4·2 (-6·9–-1·5)	-6·1 (-9·9–-2·4)	-2·9 (-8·2–2·2)
Unsafe sanitation	8·3 (4·7–13·1)	6·9 (3·9–11·3)	5·0 (2·6–8·1)	3·2 (1·6–5·5)	-3·0 (-5·0–-1·2)	-3·6 (-6·1–-1·3)	-3·9 (-7·0–-0·6)
No access to handwashing facility	1·8 (0·6–3·4)	1·9 (0·7–3·5)	1·9 (0·6–3·5)	1·5 (0·5–2·9)	-0·6 (-3·4–2·4)	-1·1 (-4·9–2·9)	-1·9 (-6·9–2·9)
Air pollution	23·6 (7·0–35·0)	22·6 (9·4–30·9)	14·3 (5·7–24·8)	9·8 (2·8–19·2)	-2·9 (-5·2–-1·1)	-4·0 (-6·6–-1·9)	-3·5 (-6·7–-2·0)
Particulate matter pollution	12·6 (4·5–22·4)	12·4 (8·9–16·3)	8·1 (5·2–11·5)	4·9 (2·2–7·9)	-3·0 (-5·7–-0·4)	-4·4 (-6·8–-3·1)	-4·5 (-8·1–-2·8)
Ambient particulate matter pollution	19·5 (7·1–34·7)	19·2 (14·3–24·5)	12·4 (8·1–17·4)	7·5 (3·4–11·9)	-3·1 (-5·7–-0·4)	-4·5 (-6·9–-3·2)	-4·6 (-8·2–-2·8)
Household air pollution from solid fuels	0·0 (0·0–0·0)	0·0 (0·0–0·0)	0·0 (0·0–0·0)	0·0 (0·0–0·0)	-3·6 (-32·4–-6·3)	-4·4 (-33·6–-6·6)	-4·7 (-29·5–-6·0)
Ambient ozone pollution	17·5 (12·2–24·2)	21·1 (15·2–28·1)	16·8 (11·5–23·4)	9·1 (5·3–14·1)	-2·1 (-2·8–-1·6)	-4·0 (-5·3–-3·2)	-5·5 (-7·6–-4·3)
Ambient nitrogen dioxide pollution	71·0 (0·0–100·0)	65·3 (0·0–100·0)	39·9 (0·0–95·6)	30·5 (0·0–83·3)	-2·7 (-6·7–0·0)	-3·6 (-8·8–0·0)	-2·4 (-6·4–0·0)
Non-optimal temperature	40·7 (30·7–51·6)	45·9 (35·7–57·1)	48·8 (38·5–59·9)	39·8 (30·5–50·9)	-0·1 (-0·4–0·3)	-0·7 (-1·0–-0·3)	-1·8 (-2·4–-1·4)
High temperature	41·2 (31·1–51·6)	51·0 (40·0–61·8)	50·1 (39·2–60·7)	37·7 (28·6–47·6)	-0·3 (-0·4–0·0)	-1·4 (-1·9–-1·0)	-2·6 (-3·4–-1·9)
Low temperature	30·4 (26·7–34·0)	32·4 (29·0–35·8)	36·9 (33·6–40·2)	31·3 (27·5–34·7)	0·1 (-0·2–0·4)	-0·2 (-0·5–0·2)	-1·5 (-2·0–-1·0)
Other environmental risks	28·2 (4·5–45·3)	26·4 (4·5–42·6)	23·6 (4·5–40·0)	20·4 (4·5–36·6)	-1·0 (-1·6–0·0)	-1·2 (-1·9–0·0)	-1·3 (-2·2–0·0)
Residential radon	21·6 (0·0–67·1)	21·6 (0·0–67·1)	21·6 (0·0–67·1)	21·6 (0·0–67·1)	0·0 (0·0–0·0)	0·0 (0·0–0·0)	0·0 (0·0–0·0)
Lead exposure	31·0 (0·0–39·2)	28·5 (0·0–35·7)	24·3 (0·0–30·7)	19·8 (0·0–24·6)	-1·5 (-1·8–0·0)	-1·7 (-2·2–0·0)	-1·9 (-2·6–0·0)
Occupational risks	2·9 (2·4–3·8)	3·0 (2·4–3·8)	2·7 (2·2–3·6)	2·7 (2·2–3·7)	-0·2 (-0·5–0·1)	-0·4 (-0·7–0·0)	0·0 (-0·5–0·4)
Occupational carcinogens	0·9 (0·4–1·7)	1·0 (0·4–1·8)	1·0 (0·4–1·8)	1·0 (0·4–1·9)	0·2 (-0·2–0·4)	-0·1 (-0·6–0·3)	0·2 (-0·4–0·6)
Occupational exposure to asbestos	3·6 (3·2–3·9)	3·7 (3·4–4·1)	3·2 (3·0–3·5)	3·0 (2·3–3·7)	-0·6 (-1·3–0·1)	-1·1 (-2·3–0·0)	-0·8 (-2·9–1·3)
Occupational exposure to arsenic	0·5 (0·0–1·1)	0·5 (0·0–1·2)	0·5 (0·0–1·1)	0·5 (0·0–1·1)	-0·1 (-0·5–0·3)	-0·3 (-0·9–0·3)	0·1 (-0·7–0·9)
Occupational exposure to benzene	1·1 (0·1–3·1)	1·2 (0·1–3·4)	1·2 (0·1–3·3)	1·2 (0·1–3·5)	0·4 (0·0–0·7)	0·1 (-0·3–0·5)	0·3 (-0·2–0·9)
Occupational exposure to beryllium	0·0 (0·0–0·0)	0·0 (0·0–0·0)	0·0 (0·0–0·0)	0·0 (0·0–0·0)	0·2 (-0·1–0·4)	0·1 (-0·2–0·4)	0·5 (0·0–0·9)
Occupational exposure to cadmium	0·1 (0·1–0·1)	0·1 (0·1–0·1)	0·1 (0·1–0·1)	0·1 (0·1–0·1)	-0·1 (-0·4–0·3)	-0·2 (-0·7–0·3)	0·2 (-0·6–0·9)
Occupational exposure to chromium	0·1 (0·1–0·2)	0·1 (0·1–0·2)	0·1 (0·1–0·1)	0·1 (0·1–0·2)	0·0 (-0·3–0·3)	-0·1 (-0·6–0·3)	0·2 (-0·4–0·9)
Occupational exposure to diesel engine exhaust	0·7 (0·7–0·8)	0·7 (0·7–0·8)	0·7 (0·7–0·7)	0·8 (0·7–0·8)	0·1 (-0·2–0·4)	0·1 (-0·3–0·5)	0·6 (-0·1–1·2)
Occupational exposure to formaldehyde	0·3 (0·3–0·3)	0·3 (0·3–0·3)	0·3 (0·3–0·3)	0·3 (0·3–0·3)	-0·1 (-0·5–0·3)	-0·3 (-0·8–0·2)	0·1 (-0·7–0·8)
Occupational exposure to nickel	0·4 (0·0–1·6)	0·4 (0·0–1·5)	0·4 (0·0–1·5)	0·4 (0·0–1·5)	-0·3 (-0·9–0·2)	-0·3 (-1·1–0·3)	0·1 (-1·0–1·1)
Occupational exposure to polycyclic aromatic hydrocarbons	0·3 (0·3–0·3)	0·3 (0·3–0·3)	0·3 (0·3–0·3)	0·3 (0·3–0·3)	0·1 (-0·3–0·4)	-0·1 (-0·6–0·3)	0·2 (-0·4–0·8)
Occupational exposure to silica	3·8 (0·6–12·4)	3·6 (0·6–11·5)	3·3 (0·5–10·5)	3·3 (0·5–10·5)	-0·5 (-1·0–0·0)	-0·4 (-1·1–0·1)	0·1 (-0·8–0·9)
Occupational exposure to sulphuric acid	0·7 (0·1–2·4)	0·7 (0·2–2·4)	0·7 (0·1–2·2)	0·7 (0·1–2·3)	-0·1 (-0·5–0·3)	-0·2 (-0·8–0·3)	0·2 (-0·7–0·9)
Occupational exposure to trichloroethylene	0·1 (0·1–0·1)	0·1 (0·1–0·1)	0·1 (0·1–0·1)	0·1 (0·1–0·1)	0·1 (-0·2–0·4)	0·0 (-0·4–0·3)	0·4 (-0·1–0·9)
Occupational asthmagens	17·5 (15·5–19·8)	17·8 (15·8–20·0)	16·2 (14·4–18·3)	16·5 (14·5–18·9)	-0·2 (-0·5–0·2)	-0·3 (-0·8–0·1)	0·2 (-0·5–0·8)
Occupational particulate matter, gases, and fumes	6·5 (5·0–8·7)	6·4 (4·9–8·4)	6·0 (4·6–7·8)	5·6 (4·3–7·3)	-0·5 (-0·8–-0·3)	-0·7 (-1·0–-0·3)	-0·7 (-1·1–-0·3)
Occupational noise	6·9 (6·3–7·5)	6·9 (6·3–7·5)	6·7 (6·3–7·3)	6·5 (6·1–7·0)	-0·2 (-0·3–0·0)	-0·3 (-0·5–-0·1)	-0·3 (-0·5–-0·1)
Occupational injuries	--	--	--	--	--	--	--
Occupational ergonomic factors	8·2 (6·9–9·9)	8·2 (6·9–9·9)	7·4 (6·2–8·9)	7·5 (6·2–9·1)	-0·3 (-0·8–0·1)	-0·5 (-1·0–0·0)	0·1 (-0·6–0·8)
**Behavioural risks**	**28·8 (25·5–32·6)**	**26·3 (23·2–29·9)**	**24·1 (21·3–27·7)**	**22·5 (19·5–26·4)**	**-0·8 (-1·2–-0·4)**	**-0·7 (-1·3–-0·2)**	**-0·6 (-1·3–0·1)**
Child and maternal malnutrition	10·1 (6·8–14·2)	8·6 (5·8–11·9)	8·7 (6·1–11·9)	8·9 (6·1–12·2)	-0·4 (-1·2–0·4)	0·1 (-0·9–1·2)	0·2 (-1·5–1·8)
Suboptimal breastfeeding	69·1 (66·6–71·7)	67·0 (64·8–69·6)	63·2 (60·5–65·9)	62·5 (59·4–65·5)	-0·3 (-0·5–-0·2)	-0·3 (-0·5–-0·2)	-0·1 (-0·4–0·2)
Non-exclusive breastfeeding	64·9 (57·6–71·8)	60·4 (54·1–67·6)	49·5 (43·1–56·3)	46·9 (39·7–53·8)	-1·0 (-1·5–-0·5)	-1·2 (-1·8–-0·6)	-0·5 (-1·5–0·4)
Discontinued breastfeeding	88·9 (86·7–91·2)	86·6 (84·4–89·5)	84·7 (81·8–88·0)	84·9 (81·9–88·1)	-0·2 (-0·3–0·0)	-0·1 (-0·3–0·1)	0·0 (-0·3–0·3)
Child growth failure	1·4 (0·4–3·1)	1·3 (0·4–2·9)	1·1 (0·4–2·5)	1·0 (0·3–2·2)	-1·1 (-1·5–-0·8)	-1·2 (-1·7–-0·8)	-0·8 (-1·5–-0·2)
Child underweight	3·0 (1·9–4·1)	2·7 (1·7–3·7)	2·3 (1·4–3·1)	2·1 (1·3–2·8)	-1·1 (-1·6–-0·7)	-1·3 (-2·0–-0·7)	-0·8 (-1·8–0·2)
Child wasting	0·9 (0·6–1·5)	0·9 (0·5–1·4)	0·9 (0·5–1·3)	0·8 (0·5–1·3)	-0·4 (-0·7–-0·1)	-0·3 (-0·7–0·1)	-0·4 (-1·0–0·2)
Child stunting	4·0 (3·1–5·1)	3·7 (2·9–4·6)	3·1 (2·4–3·9)	2·8 (2·2–3·5)	-1·1 (-1·7–-0·6)	-1·3 (-2·1–-0·5)	-1·0 (-2·1–0·1)
Low birth weight and short gestation	20·7 (18·3–23·3)	22·5 (19·7–25·4)	23·4 (20·4–26·5)	24·3 (21·3–27·4)	0·5 (0·2–0·8)	0·4 (0·0–0·7)	0·3 (-0·3–1·1)
Short gestation	38·4 (33·8–43·4)	42·4 (37·6–47·2)	39·8 (35·2–44·6)	40·8 (35·9–46·0)	0·2 (-0·1–0·5)	-0·2 (-0·6–0·3)	0·2 (-0·6–1·1)
Low birth weight	15·1 (13·9–16·6)	16·0 (14·6–17·4)	17·7 (16·3–19·4)	18·4 (16·8–20·0)	0·6 (0·4–0·9)	0·7 (0·3–1·0)	0·4 (-0·3–1·1)

Iron deficiency	3·6 (2·5–5·0)	3·0 (2·1–4·1)	3·1 (2·3–4·2)	3·2 (2·3–4·3)	-0·4 (-1·1–0·4)	0·2 (-0·8–1·2)	0·1 (-1·4–1·5)
Vitamin A deficiency	1·3 (0·0–2·6)	2·0 (0·0–3·9)	1·2 (0·0–2·4)	0·7 (0·0–1·4)	-2·1 (-4·1–0·0)	-5·0 (-7·7–0·0)	-5·1 (-9·2–0·0)
Zinc deficiency	1·9 (0·0–8·2)	1·1 (0·0–4·8)	1·0 (0·0–4·3)	1·0 (0·0–4·2)	-2·1 (-46·7–1·2)	-0·6 (-4·4–3·8)	-0·2 (-6·9–10·6)
Tobacco	38·3 (36·8–39·8)	32·1 (31·0–33·2)	24·9 (24·0–25·7)	20·8 (19·4–22·3)	-2·0 (-2·2–-1·7)	-2·1 (-2·4–-1·7)	-1·6 (-2·2–-1·0)
Smoking	35·4 (33·0–37·6)	29·2 (27·6–30·8)	22·6 (21·6–23·8)	18·6 (17·0–20·4)	-2·1 (-2·4–-1·8)	-2·1 (-2·6–-1·7)	-1·8 (-2·6–-1·0)
Chewing tobacco	5·8 (4·7–7·0)	3·9 (3·1–4·9)	2·7 (2·0–3·5)	2·2 (1·6–3·0)	-3·0 (-4·2–-1·9)	-2·7 (-4·2–-1·1)	-1·7 (-3·9–0·6)
Second-hand smoke	31·3 (29·5–32·7)	26·8 (25·0–28·1)	21·0 (19·4–22·0)	18·1 (16·5–19·7)	-1·8 (-2·0–-1·5)	-1·9 (-2·3–-1·5)	-1·4 (-2·1–-0·7)
High alcohol use	25·0 (17·8–35·3)	22·9 (15·5–33·1)	23·7 (16·9–33·8)	25·0 (17·1–35·2)	0·0 (-1·1–1·0)	0·4 (-1·0–2·0)	0·5 (-1·5–2·1)
Drug use	0·7 (0·4–1·3)	0·8 (0·6–1·1)	1·4 (1·2–1·6)	3·5 (2·5–4·6)	5·0 (2·3–7·2)	7·0 (4·0–8·9)	8·4 (5·6–10·2)
Dietary risks	37·5 (28·2–48·7)	40·5 (31·3–51·9)	42·6 (33·5–54·7)	42·4 (33·3–54·5)	0·4 (0·2–0·6)	0·2 (0·0–0·5)	-0·1 (-0·5–0·4)
Diet low in fruits	37·9 (32·6–41·4)	34·6 (29·7–37·8)	33·4 (28·9–36·3)	34·1 (29·0–38·4)	-0·3 (-0·7–0·0)	-0·1 (-0·7–0·5)	0·2 (-0·7–1·0)
Diet low in vegetables	24·9 (14·9–32·5)	26·6 (15·8–34·8)	30·3 (17·3–38·2)	29·4 (17·7–37·3)	0·5 (0·1–1·0)	0·5 (0·0–1·0)	-0·3 (-1·1–0·5)
Diet low in legumes	38·2 (0·0–47·8)	38·7 (0·0–48·3)	42·6 (0·0–53·1)	42·8 (0·0–53·5)	0·4 (0·0–0·7)	0·5 (0·0–0·9)	0·0 (-0·7–0·7)
Diet low in whole grains	45·6 (38·5–51·5)	49·0 (41·3–55·5)	50·8 (42·3–57·6)	50·9 (42·6–58·7)	0·4 (0·1–0·6)	0·2 (-0·1–0·5)	0·0 (-0·5–0·6)
Diet low in nuts and seeds	12·9 (9·4–16·9)	7·7 (4·9–10·9)	5·4 (3·3–8·0)	5·4 (3·2–8·4)	-2·8 (-4·5–-1·2)	-1·7 (-4·1–0·6)	0·1 (-3·6–3·8)
Diet low in milk	36·5 (32·3–40·7)	34·0 (29·9–38·1)	32·3 (28·0–37·2)	31·6 (26·9–36·8)	-0·5 (-1·0–0·0)	-0·3 (-1·0–0·3)	-0·2 (-1·0–0·7)
Diet high in red meat	55·6 (0·0–74·5)	56·6 (0·0–75·9)	56·5 (0·0–76·0)	56·2 (0·0–75·7)	0·0 (-0·4–0·4)	0·0 (-0·6–0·4)	0·0 (-0·8–0·7)
Diet high in processed meat	46·9 (37·2–52·7)	58·3 (46·7–65·2)	65·2 (52·6–72·4)	63·2 (50·9–71·5)	1·0 (0·5–1·4)	0·4 (-0·2–0·9)	-0·3 (-1·0–0·5)
Diet high in sugar-sweetened beverages	36·4 (28·7–44·7)	50·4 (40·4–58·4)	56·3 (46·4–65·1)	56·2 (45·7–65·9)	1·4 (0·6–2·2)	0·5 (-0·4–1·4)	0·0 (-1·3–1·2)
Diet low in fibre	39·7 (21·9–47·1)	30·4 (16·5–37·3)	26·5 (14·7–32·8)	22·5 (12·7–29·2)	-1·8 (-2·8–-1·0)	-1·4 (-2·8–-0·3)	-1·5 (-3·4–0·3)
Diet low in calcium	5·7 (4·6–7·1)	4·8 (3·9–6·1)	5·1 (4·1–6·3)	5·0 (3·9–6·1)	-0·5 (-0·7–-0·2)	0·1 (-0·2–0·4)	-0·3 (-0·7–0·1)
Diet low in seafood omega-3 fatty acids	36·8 (27·9–47·2)	44·3 (34·5–54·9)	47·2 (37·2–58·3)	41·3 (31·3–52·1)	0·4 (-0·1–0·8)	-0·3 (-0·9–0·2)	-1·2 (-2·0–-0·6)
Diet low in omega-6 polyunsaturated fatty acids	36·1 (23·3–45·6)	32·1 (21·6–41·2)	23·6 (16·5–30·3)	21·5 (14·8–27·9)	-1·7 (-2·4–-0·9)	-1·9 (-2·8–-1·0)	-0·9 (-2·0–0·1)
Diet high in trans fatty acids	71·4 (63·4–79·0)	71·9 (64·3–79·1)	63·4 (56·8–70·7)	0·0 (0·0–0·0)	-65·8 (-66·1–-65·4)	-97·1 (-97·6–-96·6)	-184·3 (-185·2–-183·2)
Diet high in sodium	23·5 (0·6–69·0)	28·4 (1·8–76·9)	31·4 (3·3–75·9)	31·7 (3·1–78·2)	1·0 (0·2–6·3)	0·5 (-0·1–4·1)	0·1 (-2·3–1·7)
Intimate partner violence	27·0 (15·9–34·6)	27·6 (18·7–34·4)	23·0 (14·9–28·5)	22·0 (13·3–29·2)	-0·7 (-2·2–0·6)	-1·1 (-2·9–0·7)	-0·4 (-2·7–1·5)
Childhood sexual abuse and bullying	8·5 (5·4–13·7)	10·3 (6·6–16·6)	11·8 (7·5–18·9)	9·1 (6·1–13·9)	0·2 (-0·3–0·7)	-0·6 (-1·3–0·2)	-2·4 (-3·8–-1·0)
Childhood sexual abuse	7·4 (6·3–8·9)	8·4 (7·2–9·8)	9·7 (8·5–11·2)	8·9 (7·5–10·8)	0·6 (-0·1–1·3)	0·3 (-0·7–1·2)	-0·8 (-2·1–0·5)
Bullying victimization	7·9 (3·5–15·8)	9·9 (4·5–19·2)	11·3 (5·1–21·8)	7·7 (3·5–14·2)	-0·1 (-0·8–0·6)	-1·2 (-2·2–-0·3)	-3·5 (-5·4–-1·6)
Unsafe sex	--	--	--	--	--	--	--
Low physical activity	21·0 (15·7–27·1)	19·9 (15·5–24·4)	20·9 (16·5–25·8)	23·5 (18·2–29·2)	0·4 (-0·5–1·3)	0·8 (-0·3–2·0)	1·1 (-0·6–2·7)
**Metabolic risks**	**25·7 (22·6–29·0)**	**31·6 (28·0–34·5)**	**35·8 (31·6–38·5)**	**39·7 (35·2–42·7)**	**1·4 (1·1–1·7)**	**1·1 (0·8–1·4)**	**1·0 (0·5–1·4)**
High fasting plasma glucose	13·2 (9·9–15·9)	17·4 (13·4–20·6)	22·3 (17·2–26·2)	25·7 (19·7–30·1)	2·2 (1·6–2·7)	1·9 (1·2–2·7)	1·3 (0·2–2·4)
High LDL cholesterol	63·6 (44·3–86·3)	53·8 (37·0–74·6)	48·1 (32·6–67·4)	46·6 (31·3–64·7)	-1·0 (-1·2–-0·8)	-0·7 (-0·9–-0·5)	-0·3 (-0·6–-0·1)
High systolic blood pressure	29·6 (20·4–40·9)	25·4 (17·8–34·9)	19·0 (12·2–27·2)	26·4 (16·4–38·1)	-0·4 (-1·3–0·6)	0·2 (-1·0–1·3)	3·0 (0·9–5·4)
High body-mass index	29·3 (24·7–33·5)	36·7 (31·4–40·5)	41·4 (35·3–45·0)	45·3 (38·3–48·9)	1·4 (1·1–1·7)	1·0 (0·7–1·3)	0·8 (0·4–1·3)
Low bone mineral density	21·4 (15·1–28·2)	19·3 (13·6–26·0)	19·9 (14·5–27·3)	21·7 (15·3–28·7)	0·0 (-0·5–0·5)	0·6 (-0·2–1·4)	0·8 (-0·8–2·2)
Kidney dysfunction	2·6 (2·1–3·3)	2·6 (2·2–3·4)	2·5 (2·1–3·3)	2·7 (2·3–3·6)	0·2 (0·1–0·3)	0·2 (0·0–0·4)	0·6 (0·3–0·9)

Utah

Risk Names	SEV 1990	SEV 2000	SEV 2010	SEV 2021	Annualised rate of change 1990 to 2021	Annualised rate of change 2000 to 2021	Annualised rate of change 2010 to 2021
**All risk factors**	**24·3 (21·4–27·4)**	**24·3 (21·4–27·6)**	**24·5 (21·6–27·8)**	**25·8 (22·8–29·1)**	**0·2 (-0·2–0·5)**	**0·3 (-0·1–0·7)**	**0·5 (-0·1–1·1)**
**Environmental/occupational risks**	**18·1 (11·7–29·4)**	**17·6 (11·4–28·4)**	**16·4 (10·3–27·4)**	**14·6 (9·0–24·6)**	**-0·7 (-1·4–0·1)**	**-0·9 (-1·3–-0·4)**	**-1·1 (-1·5–-0·5)**
Unsafe water, sanitation, and handwashing	2·1 (1·1–3·6)	1·9 (0·9–3·0)	1·4 (0·5–2·5)	1·1 (0·4–1·9)	-2·1 (-4·4–0·0)	-2·5 (-5·5–0·2)	-2·2 (-6·0–1·6)
Unsafe water source	3·0 (1·1–6·3)	2·7 (1·1–5·3)	1·1 (0·4–2·2)	0·8 (0·3–1·6)	-4·1 (-6·8–-1·3)	-5·6 (-9·3–-1·7)	-2·4 (-7·9–2·9)
Unsafe sanitation	7·3 (4·0–11·9)	5·6 (2·9–9·2)	4·1 (2·2–6·9)	2·9 (1·5–4·9)	-3·0 (-5·2–-1·0)	-3·2 (-5·8–-0·7)	-3·2 (-6·2–0·0)
No access to handwashing facility	1·7 (0·5–3·6)	1·7 (0·5–3·5)	1·7 (0·5–3·4)	1·4 (0·4–2·7)	-0·6 (-3·6–2·2)	-0·9 (-5·0–2·9)	-1·7 (-7·6–3·8)
Air pollution	18·7 (5·1–30·1)	18·1 (6·0–26·9)	11·1 (3·9–21·2)	7·7 (2·0–17·1)	-2·9 (-5·5–-1·0)	-4·1 (-7·4–-2·0)	-3·3 (-6·8–-1·9)
Particulate matter pollution	9·6 (2·6–18·1)	8·2 (5·2–11·7)	5·8 (3·2–8·9)	3·6 (1·4–6·5)	-3·2 (-6·4–0·0)	-4·0 (-6·8–-2·6)	-4·4 (-8·3–-2·5)
Ambient particulate matter pollution	14·8 (3·9–28·0)	12·6 (8·3–17·3)	8·8 (5·0–13·3)	5·5 (2·1–9·7)	-3·2 (-6·4–0·0)	-4·0 (-6·8–-2·6)	-4·4 (-8·4–-2·5)
Household air pollution from solid fuels	0·0 (0·0–0·1)	0·0 (0·0–0·0)	0·0 (0·0–0·0)	0·0 (0·0–0·0)	-5·0 (-30·4–-6·4)	-5·4 (-28·3–-6·0)	-5·2 (-26·5–-6·4)
Ambient ozone pollution	25·0 (18·5–32·1)	31·9 (24·9–40·9)	25·8 (19·1–33·7)	26·0 (19·6–33·6)	0·1 (-0·3–0·7)	-1·0 (-1·8–-0·1)	0·1 (-1·2–1·4)
Ambient nitrogen dioxide pollution	56·9 (0·0–100·0)	59·6 (0·0–100·0)	32·0 (0·0–88·0)	23·7 (0·0–72·9)	-2·8 (-9·8–0·0)	-4·4 (-15·2–0·0)	-2·7 (-10·3–0·0)
Non-optimal temperature	36·6 (28·1–47·7)	38·3 (29·2–49·1)	37·4 (29·3–47·6)	41·1 (31·3–52·3)	0·4 (0·2–0·6)	0·3 (0·1–0·6)	0·9 (0·2–1·5)
High temperature	38·4 (25·8–51·0)	45·2 (30·8–59·0)	41·2 (28·2–54·4)	54·7 (37·6–69·3)	1·1 (0·7–1·7)	0·9 (0·5–1·4)	2·6 (1·4–3·7)
Low temperature	30·2 (27·6–33·8)	30·8 (27·8–34·7)	31·5 (28·5–35·2)	31·4 (28·2–35·2)	0·1 (0·0–0·2)	0·1 (0·0–0·2)	0·0 (-0·2–0·1)
Other environmental risks	31·8 (8·9–52·1)	30·1 (8·9–50·1)	26·8 (8·9–47·1)	23·6 (8·9–43·3)	-1·0 (-1·7–0·0)	-1·2 (-1·9–0·0)	-1·2 (-2·2–0·0)
Residential radon	31·8 (0·0–98·4)	31·8 (0·0–98·4)	31·8 (0·0–98·4)	31·8 (0·0–98·4)	0·0 (0·0–0·0)	0·0 (0·0–0·0)	0·0 (0·0–0·0)
Lead exposure	31·8 (0·0–40·0)	29·2 (0·0–36·3)	24·6 (0·0–30·8)	19·9 (0·0–24·6)	-1·5 (-2·0–0·0)	-1·8 (-2·3–0·0)	-1·9 (-2·7–0·0)
Occupational risks	2·9 (2·4–3·7)	3·0 (2·5–3·9)	2·9 (2·3–3·7)	2·9 (2·3–3·7)	-0·1 (-0·4–0·2)	-0·3 (-0·7–0·0)	0·0 (-0·5–0·5)
Occupational carcinogens	0·9 (0·4–1·8)	1·0 (0·5–2·0)	1·0 (0·4–1·9)	1·0 (0·4–1·9)	0·2 (-0·2–0·4)	-0·1 (-0·6–0·2)	0·0 (-0·6–0·5)
Occupational exposure to asbestos	3·9 (3·5–4·4)	4·5 (4·0–4·9)	3·9 (3·5–4·4)	3·5 (2·7–4·3)	-0·4 (-1·3–0·4)	-1·2 (-2·4–0·0)	-1·2 (-3·5–1·0)
Occupational exposure to arsenic	0·5 (0·0–1·2)	0·5 (0·0–1·2)	0·5 (0·0–1·2)	0·5 (0·0–1·2)	-0·1 (-0·5–0·4)	-0·3 (-0·9–0·4)	0·1 (-0·8–1·0)
Occupational exposure to benzene	1·1 (0·1–3·1)	1·2 (0·1–3·5)	1·2 (0·1–3·5)	1·2 (0·1–3·5)	0·4 (0·0–0·7)	0·1 (-0·4–0·5)	0·3 (-0·4–0·9)
Occupational exposure to beryllium	0·0 (0·0–0·0)	0·0 (0·0–0·0)	0·0 (0·0–0·0)	0·0 (0·0–0·0)	0·2 (0·0–0·5)	0·1 (-0·3–0·4)	0·4 (-0·1–0·8)
Occupational exposure to cadmium	0·1 (0·1–0·1)	0·1 (0·1–0·1)	0·1 (0·1–0·1)	0·1 (0·1–0·1)	0·0 (-0·4–0·4)	-0·2 (-0·8–0·3)	0·1 (-0·7–0·9)
Occupational exposure to chromium	0·1 (0·1–0·2)	0·2 (0·1–0·2)	0·1 (0·1–0·2)	0·1 (0·1–0·2)	0·1 (-0·3–0·4)	-0·1 (-0·7–0·3)	0·2 (-0·6–0·8)
Occupational exposure to diesel engine exhaust	0·7 (0·7–0·8)	0·8 (0·7–0·8)	0·7 (0·7–0·8)	0·8 (0·7–0·9)	0·2 (-0·2–0·5)	0·2 (-0·4–0·7)	0·6 (-0·2–1·3)
Occupational exposure to formaldehyde	0·3 (0·3–0·3)	0·3 (0·3–0·3)	0·3 (0·3–0·3)	0·3 (0·3–0·3)	-0·1 (-0·4–0·4)	-0·3 (-0·9–0·2)	0·0 (-0·8–0·8)
Occupational exposure to nickel	0·4 (0·0–1·6)	0·4 (0·0–1·6)	0·4 (0·0–1·5)	0·4 (0·0–1·5)	-0·2 (-0·7–0·3)	-0·3 (-1·1–0·3)	0·0 (-1·0–1·1)
Occupational exposure to polycyclic aromatic hydrocarbons	0·3 (0·3–0·3)	0·3 (0·3–0·3)	0·3 (0·3–0·3)	0·3 (0·3–0·3)	0·1 (-0·2–0·5)	-0·1 (-0·6–0·3)	0·2 (-0·5–0·8)
Occupational exposure to silica	3·7 (0·6–12·0)	3·6 (0·5–11·5)	3·3 (0·5–10·8)	3·3 (0·5–10·8)	-0·3 (-0·9–0·1)	-0·3 (-1·0–0·3)	0·1 (-0·9–1·0)
Occupational exposure to sulphuric acid	0·7 (0·2–2·4)	0·8 (0·2–2·6)	0·7 (0·2–2·5)	0·7 (0·2–2·4)	0·0 (-0·4–0·4)	-0·2 (-0·8–0·3)	0·1 (-0·7–0·9)
Occupational exposure to trichloroethylene	0·1 (0·1–0·1)	0·1 (0·1–0·1)	0·1 (0·1–0·1)	0·1 (0·1–0·1)	0·2 (-0·1–0·4)	0·0 (-0·4–0·4)	0·3 (-0·2–0·9)
Occupational asthmagens	17·5 (15·4–19·7)	18·2 (16·1–20·5)	16·9 (14·8–19·1)	17·1 (14·8–19·4)	-0·1 (-0·5–0·3)	-0·3 (-0·8–0·1)	0·1 (-0·7–0·8)
Occupational particulate matter, gases, and fumes	6·6 (5·1–8·7)	6·5 (5·0–8·4)	6·2 (4·8–8·0)	5·8 (4·5–7·6)	-0·4 (-0·7–-0·1)	-0·5 (-0·8–-0·2)	-0·6 (-1·0–-0·2)
Occupational noise	6·9 (6·4–7·6)	7·0 (6·5–7·6)	6·9 (6·5–7·5)	6·7 (6·3–7·3)	-0·1 (-0·3–0·1)	-0·2 (-0·4–0·0)	-0·3 (-0·5–-0·1)
Occupational injuries	--	--	--	--	--	--	--
Occupational ergonomic factors	8·3 (7·0–10·0)	8·4 (7·1–10·2)	7·7 (6·5–9·2)	7·8 (6·5–9·4)	-0·2 (-0·7–0·2)	-0·4 (-1·0–0·1)	0·1 (-0·5–0·8)
**Behavioural risks**	**24·2 (21·4–27·4)**	**22·1 (19·6–25·0)**	**20·4 (18·0–23·4)**	**19·6 (17·1–22·8)**	**-0·7 (-1·0–-0·4)**	**-0·6 (-0·9–-0·2)**	**-0·3 (-0·9–0·2)**
Child and maternal malnutrition	10·2 (6·9–13·8)	8·8 (6·1–12·2)	8·8 (6·2–12·2)	9·0 (6·1–12·5)	-0·4 (-1·3–0·5)	0·1 (-1·1–1·2)	0·2 (-1·7–1·8)
Suboptimal breastfeeding	68·9 (66·4–71·7)	66·9 (64·3–69·6)	63·0 (60·3–65·9)	62·3 (59·0–65·5)	-0·3 (-0·5–-0·2)	-0·3 (-0·5–-0·2)	-0·1 (-0·4–0·2)
Non-exclusive breastfeeding	64·6 (56·3–71·9)	60·1 (53·1–66·3)	49·1 (42·1–56·4)	46·5 (38·7–54·2)	-1·1 (-1·6–-0·5)	-1·2 (-1·9–-0·6)	-0·5 (-1·5–0·5)
Discontinued breastfeeding	88·7 (86·5–91·1)	86·4 (83·6–89·4)	84·4 (81·1–87·6)	84·6 (81·1–88·0)	-0·2 (-0·3–0·0)	-0·1 (-0·3–0·1)	0·0 (-0·3–0·3)
Child growth failure	1·4 (0·4–3·1)	1·2 (0·4–2·8)	1·1 (0·3–2·4)	1·0 (0·3–2·1)	-1·0 (-1·4–-0·7)	-1·1 (-1·7–-0·6)	-0·6 (-1·4–0·1)
Child underweight	2·9 (1·8–4·0)	2·6 (1·7–3·6)	2·2 (1·4–3·0)	2·1 (1·3–2·8)	-1·1 (-1·6–-0·5)	-1·1 (-1·9–-0·5)	-0·5 (-1·5–0·4)
Child wasting	0·9 (0·6–1·4)	0·8 (0·5–1·3)	0·8 (0·5–1·3)	0·8 (0·5–1·3)	-0·4 (-0·7–0·0)	-0·2 (-0·7–0·4)	-0·2 (-0·9–0·5)
Child stunting	3·9 (3·1–5·0)	3·6 (2·8–4·5)	3·0 (2·4–3·9)	2·8 (2·1–3·6)	-1·1 (-1·6–-0·5)	-1·2 (-2·0–-0·5)	-0·8 (-2·0–0·4)
Low birth weight and short gestation	19·6 (17·5–22·1)	19·9 (17·4–22·5)	20·4 (18·0–23·1)	21·4 (19·0–24·0)	0·3 (0·0–0·5)	0·4 (0·0–0·7)	0·4 (-0·3–1·1)
Short gestation	32·8 (29·4–36·8)	34·2 (30·3–38·2)	34·4 (31·0–38·7)	35·8 (32·1–40·3)	0·3 (0·0–0·6)	0·2 (-0·2–0·7)	0·4 (-0·4–1·2)
Low birth weight	16·0 (14·8–17·2)	15·9 (14·6–17·3)	16·4 (15·1–17·7)	17·1 (15·9–18·5)	0·2 (0·0–0·5)	0·4 (0·0–0·7)	0·4 (-0·3–1·1)

Iron deficiency	3·6 (2·5–4·8)	3·1 (2·2–4·3)	3·2 (2·3–4·3)	3·2 (2·3–4·4)	-0·4 (-1·2–0·5)	0·1 (-1·0–1·2)	0·1 (-1·6–1·7)
Vitamin A deficiency	1·2 (0·0–2·5)	1·6 (0·0–3·4)	1·0 (0·0–2·1)	0·6 (0·0–1·3)	-2·4 (-4·6–0·0)	-5·1 (-7·7–0·0)	-5·3 (-9·3–0·0)
Zinc deficiency	2·2 (0·0–9·1)	1·2 (0·0–5·4)	1·1 (0·0–4·7)	1·1 (0·0–4·6)	-2·2 (-48·4–0·0)	-0·5 (-3·7–2·9)	-0·2 (-6·3–2·0)
Tobacco	29·2 (27·8–30·5)	24·6 (23·6–25·6)	18·4 (17·6–19·2)	16·1 (14·9–17·3)	-1·9 (-2·2–-1·7)	-2·0 (-2·4–-1·7)	-1·2 (-1·9–-0·6)
Smoking	24·9 (23·0–26·8)	20·9 (19·4–22·2)	16·0 (15·0–17·2)	13·5 (12·3–14·8)	-2·0 (-2·3–-1·6)	-2·1 (-2·5–-1·6)	-1·5 (-2·3–-0·7)
Chewing tobacco	1·3 (0·9–1·6)	1·5 (1·1–2·0)	1·7 (1·2–2·3)	1·7 (1·2–2·4)	1·0 (-0·3–2·5)	0·6 (-1·2–2·8)	0·3 (-2·4–3·2)
Second-hand smoke	25·9 (24·4–27·1)	22·0 (20·5–23·0)	16·4 (15·1–17·2)	14·7 (13·3–16·0)	-1·8 (-2·1–-1·6)	-1·9 (-2·3–-1·5)	-1·0 (-1·7–-0·2)
High alcohol use	22·3 (16·2–31·7)	20·1 (14·4–28·7)	20·4 (15·0–29·3)	21·9 (15·8–31·3)	-0·1 (-0·9–0·8)	0·4 (-0·6–1·6)	0·6 (-0·9–2·2)
Drug use	0·7 (0·4–1·4)	0·8 (0·6–1·2)	1·9 (1·6–2·2)	4·4 (3·3–5·6)	5·7 (2·9–8·5)	8·1 (5·1–10·2)	7·9 (6·1–9·1)
Dietary risks	34·9 (26·1–45·7)	37·4 (28·2–49·1)	39·9 (30·5–51·6)	40·4 (30·9–51·6)	0·5 (0·2–0·8)	0·4 (0·1–0·7)	0·1 (-0·3–0·6)
Diet low in fruits	35·0 (29·7–38·5)	31·2 (27·3–34·2)	31·5 (27·2–34·1)	33·5 (29·0–37·6)	-0·1 (-0·5–0·2)	0·3 (-0·2–0·9)	0·6 (-0·3–1·5)
Diet low in vegetables	22·8 (13·9–30·4)	24·0 (14·1–31·3)	28·4 (15·7–35·5)	28·8 (17·3–36·5)	0·8 (0·3–1·2)	0·9 (0·4–1·5)	0·1 (-0·6–1·0)
Diet low in legumes	39·7 (0·0–49·7)	39·8 (0·0–49·5)	44·0 (0·0–54·7)	44·2 (0·0–55·6)	0·3 (0·0–0·6)	0·5 (0·0–1·0)	0·0 (-0·7–0·8)
Diet low in whole grains	44·7 (36·9–51·0)	47·7 (39·3–54·3)	49·4 (41·1–56·1)	49·8 (41·4–57·2)	0·4 (0·1–0·6)	0·2 (-0·2–0·5)	0·1 (-0·5–0·6)
Diet low in nuts and seeds	15·5 (11·6–20·2)	9·4 (6·4–13·0)	6·9 (4·6–9·8)	6·9 (4·3–10·4)	-2·6 (-4·3–-1·2)	-1·5 (-3·9–0·7)	0·0 (-3·5–3·3)
Diet low in milk	38·6 (34·6–43·4)	35·5 (31·3–39·9)	34·1 (29·8–38·8)	33·3 (28·5–38·6)	-0·5 (-1·0–-0·1)	-0·3 (-0·9–0·3)	-0·2 (-1·0–0·6)
Diet high in red meat	47·8 (0·0–66·9)	48·9 (0·0–67·8)	49·2 (0·0–68·5)	49·6 (0·0–68·7)	0·1 (-0·3–0·6)	0·1 (-0·5–0·7)	0·1 (-0·8–1·0)
Diet high in processed meat	41·3 (32·3–46·5)	51·3 (41·1–57·6)	57·9 (46·6–65·4)	56·8 (47·3–64·3)	1·0 (0·6–1·4)	0·5 (-0·1–1·0)	-0·2 (-1·0–0·6)
Diet high in sugar-sweetened beverages	32·4 (24·3–40·2)	45·9 (37·1–54·4)	50·8 (40·5–60·2)	51·3 (41·6–61·7)	1·5 (0·6–2·3)	0·5 (-0·5–1·4)	0·1 (-1·4–1·6)
Diet low in fibre	42·4 (22·3–49·9)	32·3 (16·7–38·9)	28·9 (15·6–34·9)	24·8 (13·5–30·8)	-1·7 (-2·6–-0·9)	-1·3 (-2·5–-0·1)	-1·4 (-3·3–0·3)
Diet low in calcium	6·1 (4·9–7·6)	5·0 (4·0–6·2)	5·4 (4·3–6·7)	5·2 (4·1–6·3)	-0·5 (-0·8–-0·3)	0·2 (-0·1–0·5)	-0·3 (-0·7–0·1)
Diet low in seafood omega-3 fatty acids	40·3 (30·8–50·3)	46·5 (36·1–57·5)	49·9 (39·1–61·3)	43·7 (33·8–54·4)	0·3 (-0·2–0·6)	-0·3 (-0·8–0·1)	-1·2 (-2·0–-0·5)
Diet low in omega-6 polyunsaturated fatty acids	39·8 (25·3–50·4)	34·6 (22·9–44·6)	26·1 (18·2–33·7)	23·5 (16·4–30·4)	-1·7 (-2·4–-0·9)	-1·8 (-2·7–-1·0)	-0·9 (-2·1–0·0)
Diet high in trans fatty acids	71·4 (63·6–79·1)	71·8 (62·7–79·3)	63·4 (53·9–71·6)	0·0 (0·0–0·0)	-65·8 (-66·1–-65·4)	-97·1 (-97·6–-96·5)	-184·3 (-185·4–-182·8)
Diet high in sodium	23·0 (0·7–67·4)	27·7 (1·9–73·8)	30·5 (3·4–73·3)	31·1 (3·3–74·9)	1·0 (0·2–6·1)	0·6 (-0·1–3·8)	0·2 (-2·6–2·2)
Intimate partner violence	26·6 (16·9–33·9)	26·7 (17·7–33·1)	24·7 (16·9–30·6)	23·6 (14·3–31·5)	-0·4 (-1·4–0·5)	-0·6 (-2·0–0·6)	-0·4 (-2·2–1·1)
Childhood sexual abuse and bullying	8·7 (5·5–13·9)	10·5 (6·7–16·6)	11·9 (7·5–18·9)	9·8 (6·3–15·7)	0·4 (-0·2–0·9)	-0·4 (-1·1–0·4)	-1·8 (-3·4–-0·3)
Childhood sexual abuse	7·7 (6·6–9·1)	8·7 (7·4–10·1)	9·9 (8·4–11·5)	9·3 (7·7–11·4)	0·6 (-0·2–1·4)	0·3 (-0·7–1·3)	-0·6 (-2·0–0·9)
Bullying victimization	8·0 (3·5–16·1)	10·0 (4·6–19·5)	11·3 (5·1–21·8)	8·5 (3·7–17·1)	0·2 (-0·5–0·9)	-0·8 (-1·8–0·3)	-2·5 (-4·4–-0·6)
Unsafe sex	--	--	--	--	--	--	--
Low physical activity	18·3 (13·9–24·0)	17·2 (12·8–22·1)	18·3 (13·9–23·0)	20·8 (15·4–27·1)	0·4 (-0·5–1·4)	0·9 (-0·4–2·2)	1·1 (-0·7–2·9)
**Metabolic risks**	**22·9 (20·0–26·1)**	**28·1 (24·6–31·0)**	**31·5 (27·6–34·5)**	**34·9 (30·4–37·8)**	**1·4 (1·0–1·6)**	**1·0 (0·7–1·4)**	**1·0 (0·5–1·5)**
High fasting plasma glucose	12·5 (9·5–15·1)	16·1 (12·3–19·1)	19·8 (15·0–23·5)	23·0 (18·1–27·7)	2·0 (1·3–2·6)	1·7 (0·8–2·5)	1·4 (0·1–2·5)
High LDL cholesterol	62·7 (44·2–85·3)	53·2 (36·5–73·3)	47·6 (32·1–66·4)	46·2 (31·0–64·3)	-1·0 (-1·2–-0·8)	-0·7 (-0·9–-0·5)	-0·3 (-0·5–0·0)
High systolic blood pressure	27·6 (18·1–39·1)	23·4 (16·4–32·8)	17·2 (11·0–25·0)	24·2 (15·0–34·9)	-0·4 (-1·4–0·5)	0·2 (-1·0–1·3)	3·1 (1·0–5·6)
High body-mass index	25·9 (21·7–30·2)	32·3 (27·4–36·3)	36·2 (30·8–40·3)	39·6 (33·7–43·4)	1·4 (1·0–1·7)	1·0 (0·6–1·3)	0·8 (0·4–1·3)
Low bone mineral density	21·1 (15·1–27·7)	19·1 (13·3–26·0)	19·6 (14·1–26·5)	21·4 (15·1–28·0)	0·1 (-0·5–0·5)	0·6 (-0·2–1·4)	0·8 (-0·6–2·2)
Kidney dysfunction	2·9 (2·3–3·8)	2·9 (2·4–3·8)	2·9 (2·3–3·9)	3·0 (2·4–3·9)	0·2 (0·1–0·3)	0·1 (0·0–0·3)	0·4 (0·1–0·7)

Vermont

Risk Names	SEV 1990	SEV 2000	SEV 2010	SEV 2021	Annualised rate of change 1990 to 2021	Annualised rate of change 2000 to 2021	Annualised rate of change 2010 to 2021
**All risk factors**	**26·2 (23·3–29·3)**	**25·9 (22·9–28·8)**	**26·9 (23·9–30·0)**	**26·2 (23·1–29·6)**	**0·0 (-0·3–0·3)**	**0·1 (-0·3–0·4)**	**-0·3 (-0·8–0·3)**
**Environmental/occupational risks**	**15·4 (10·0–25·8)**	**15·2 (10·1–25·1)**	**13·5 (8·7–22·7)**	**12·0 (7·7–20·5)**	**-0·8 (-1·5–-0·2)**	**-1·1 (-1·6–-0·8)**	**-1·1 (-1·7–-0·6)**
Unsafe water, sanitation, and handwashing	1·7 (0·8–2·7)	1·5 (0·6–2·6)	1·2 (0·3–2·2)	1·0 (0·3–1·8)	-1·6 (-3·9–0·4)	-1·9 (-5·3–1·0)	-1·7 (-5·8–2·3)
Unsafe water source	2·3 (0·8–4·6)	2·1 (0·7–4·3)	0·9 (0·4–1·9)	0·7 (0·3–1·4)	-3·7 (-6·5–-1·0)	-5·0 (-8·7–-1·1)	-1·6 (-7·3–3·1)
Unsafe sanitation	4·8 (2·7–8·2)	3·7 (2·0–6·3)	2·8 (1·4–5·1)	2·2 (1·1–3·9)	-2·6 (-4·8–-0·4)	-2·5 (-5·4–0·0)	-2·3 (-6·3–1·1)
No access to handwashing facility	1·6 (0·5–3·1)	1·7 (0·5–3·4)	1·7 (0·5–3·2)	1·4 (0·5–2·8)	-0·4 (-3·5–2·6)	-0·7 (-4·6–3·4)	-1·5 (-6·7–3·8)
Air pollution	12·5 (3·0–23·4)	10·8 (3·8–20·7)	5·2 (1·5–12·5)	3·3 (0·2–10·4)	-4·3 (-11·6–-2·0)	-5·6 (-15·2–-3·2)	-4·1 (-21·3–-0·9)
Particulate matter pollution	7·2 (0·9–15·4)	6·1 (3·1–9·7)	3·9 (1·3–7·1)	1·5 (0·0–4·2)	-5·0 (-15·7–-0·4)	-6·5 (-20·8–-3·6)	-8·4 (-32·5–-3·7)
Ambient particulate matter pollution	11·0 (1·4–23·9)	9·3 (4·7–14·4)	6·0 (1·9–10·6)	2·3 (0·1–6·3)	-5·0 (-15·7–-0·5)	-6·5 (-20·9–-3·5)	-8·5 (-32·5–-3·7)
Household air pollution from solid fuels	0·0 (0·0–0·2)	0·0 (0·0–0·1)	0·0 (0·0–0·0)	0·0 (0·0–0·0)	-6·1 (-44·1–-9·7)	-7·3 (-46·6–-10·5)	-8·5 (-45·7–-8·9)
Ambient ozone pollution	16·6 (11·0–24·1)	15·7 (10·0–23·5)	11·4 (6·5–17·9)	5·9 (2·5–10·0)	-3·3 (-5·0–-2·3)	-4·7 (-7·2–-3·0)	-6·0 (-10·0–-3·3)
Ambient nitrogen dioxide pollution	34·1 (0·0–88·2)	30·0 (0·0–82·0)	9·1 (0·0–45·5)	10·7 (0·0–48·4)	-3·7 (-19·6–0·0)	-4·9 (-26·7–0·0)	1·5 (0·0–51·6)
Non-optimal temperature	27·7 (22·4–35·6)	26·6 (22·5–32·5)	28·9 (23·2–37·0)	28·1 (22·4–36·0)	0·0 (-0·1–0·3)	0·3 (-0·2–0·9)	-0·3 (-0·7–0·0)
High temperature	15·3 (7·1–26·2)	11·5 (4·7–21·4)	23·4 (13·1–35·8)	26·2 (15·1–38·4)	1·7 (0·7–2·9)	3·9 (2·0–6·3)	1·0 (0·2–2·0)
Low temperature	27·1 (23·1–32·0)	26·6 (23·0–31·3)	27·4 (23·5–32·3)	26·2 (22·3–31·0)	-0·1 (-0·2–0·0)	-0·1 (-0·2–0·0)	-0·4 (-0·6–-0·2)
Other environmental risks	23·7 (10·7–44·7)	22·7 (10·1–43·5)	21·1 (9·5–41·9)	19·6 (8·7–40·1)	-0·6 (-1·3–0·0)	-0·7 (-1·5–0·0)	-0·7 (-1·5–0·0)
Residential radon	32·2 (0·0–95·5)	32·2 (0·0–95·5)	32·2 (0·0–95·5)	32·2 (0·0–95·5)	0·0 (0·0–0·0)	0·0 (0·0–0·0)	0·0 (0·0–0·0)
Lead exposure	19·9 (0·0–25·3)	18·5 (0·0–23·0)	16·1 (0·0–19·9)	13·9 (0·0–17·7)	-1·2 (-1·6–0·0)	-1·3 (-1·9–0·0)	-1·3 (-2·2–0·0)
Occupational risks	3·2 (2·6–4·2)	3·3 (2·7–4·4)	3·1 (2·5–4·1)	3·1 (2·5–4·0)	-0·1 (-0·4–0·2)	-0·3 (-0·7–0·1)	0·0 (-0·5–0·4)
Occupational carcinogens	1·0 (0·5–2·0)	1·1 (0·5–2·2)	1·1 (0·5–2·1)	1·1 (0·5–2·2)	0·2 (-0·2–0·4)	0·0 (-0·5–0·3)	0·1 (-0·4–0·6)
Occupational exposure to asbestos	4·4 (3·9–5·0)	4·4 (3·9–4·9)	4·0 (3·5–4·5)	3·9 (3·1–4·8)	-0·4 (-1·2–0·3)	-0·6 (-1·7–0·3)	-0·3 (-2·2–1·5)
Occupational exposure to arsenic	0·6 (0·0–1·4)	0·6 (0·0–1·4)	0·5 (0·0–1·3)	0·5 (0·0–1·2)	-0·3 (-0·7–0·2)	-0·4 (-1·0–0·2)	-0·1 (-0·9–0·8)
Occupational exposure to benzene	1·2 (0·1–3·5)	1·3 (0·1–3·9)	1·3 (0·1–3·9)	1·4 (0·1–4·0)	0·4 (0·0–0·8)	0·1 (-0·3–0·6)	0·2 (-0·4–0·8)
Occupational exposure to beryllium	0·0 (0·0–0·0)	0·0 (0·0–0·0)	0·0 (0·0–0·0)	0·0 (0·0–0·0)	0·2 (0·0–0·5)	0·1 (-0·3–0·4)	0·3 (-0·1–0·7)
Occupational exposure to cadmium	0·1 (0·1–0·1)	0·1 (0·1–0·1)	0·1 (0·1–0·1)	0·1 (0·1–0·1)	-0·2 (-0·6–0·2)	-0·3 (-0·8–0·2)	0·0 (-0·8–0·7)
Occupational exposure to chromium	0·2 (0·1–0·2)	0·2 (0·2–0·2)	0·2 (0·1–0·2)	0·2 (0·1–0·2)	-0·1 (-0·4–0·3)	-0·2 (-0·7–0·2)	0·0 (-0·6–0·7)
Occupational exposure to diesel engine exhaust	0·7 (0·6–0·7)	0·7 (0·7–0·8)	0·7 (0·7–0·8)	0·8 (0·7–0·8)	0·4 (0·1–0·8)	0·3 (-0·1–0·9)	0·6 (-0·2–1·3)
Occupational exposure to formaldehyde	0·3 (0·3–0·4)	0·3 (0·3–0·4)	0·3 (0·3–0·3)	0·3 (0·3–0·3)	-0·2 (-0·6–0·2)	-0·4 (-0·9–0·1)	-0·1 (-0·9–0·6)
Occupational exposure to nickel	0·4 (0·0–1·8)	0·4 (0·0–1·7)	0·4 (0·0–1·6)	0·4 (0·0–1·6)	-0·3 (-0·9–0·2)	-0·4 (-1·2–0·3)	-0·1 (-1·2–0·9)
Occupational exposure to polycyclic aromatic hydrocarbons	0·3 (0·3–0·3)	0·3 (0·3–0·4)	0·3 (0·3–0·3)	0·3 (0·3–0·3)	0·0 (-0·3–0·3)	-0·2 (-0·6–0·3)	0·1 (-0·6–0·7)
Occupational exposure to silica	3·8 (0·6–12·6)	3·7 (0·6–11·8)	3·5 (0·6–11·2)	3·5 (0·6–11·1)	-0·3 (-0·8–0·2)	-0·4 (-1·0–0·3)	0·0 (-0·9–1·0)
Occupational exposure to sulphuric acid	0·8 (0·2–2·7)	0·8 (0·2–2·8)	0·8 (0·2–2·6)	0·8 (0·2–2·5)	-0·2 (-0·6–0·3)	-0·3 (-0·9–0·3)	0·0 (-0·8–0·8)
Occupational exposure to trichloroethylene	0·1 (0·1–0·1)	0·1 (0·1–0·1)	0·1 (0·1–0·1)	0·1 (0·1–0·1)	0·1 (-0·2–0·4)	0·0 (-0·4–0·4)	0·2 (-0·3–0·8)
Occupational asthmagens	18·8 (16·4–21·5)	19·3 (17·0–21·9)	18·0 (15·8–20·5)	18·2 (16·0–20·8)	-0·1 (-0·5–0·2)	-0·3 (-0·8–0·2)	0·1 (-0·7–0·8)
Occupational particulate matter, gases, and fumes	7·3 (5·5–9·5)	7·1 (5·5–9·2)	6·7 (5·1–8·6)	6·2 (4·7–8·0)	-0·5 (-0·8–-0·3)	-0·7 (-1·0–-0·4)	-0·7 (-1·1–-0·4)
Occupational noise	7·4 (6·8–8·0)	7·5 (7·0–8·1)	7·4 (7·0–8·0)	7·2 (6·8–7·7)	-0·1 (-0·2–0·1)	-0·2 (-0·4–0·0)	-0·3 (-0·5–-0·1)
Occupational injuries	--	--	--	--	--	--	--
Occupational ergonomic factors	9·2 (7·8–11·0)	9·2 (7·8–11·1)	8·3 (7·1–10·0)	8·5 (7·0–10·2)	-0·3 (-0·8–0·2)	-0·4 (-1·0–0·1)	0·2 (-0·6–0·9)
**Behavioural risks**	**31·0 (27·7–34·9)**	**30·0 (26·5–33·5)**	**28·3 (24·8–32·2)**	**26·6 (23·0–30·2)**	**-0·5 (-0·9–-0·1)**	**-0·6 (-1·1–-0·1)**	**-0·6 (-1·2–0·1)**
Child and maternal malnutrition	10·1 (6·8–14·2)	8·6 (5·8–12·6)	8·6 (6·1–12·3)	8·8 (5·9–12·4)	-0·4 (-1·4–0·4)	0·1 (-1·1–1·4)	0·2 (-1·7–1·9)
Suboptimal breastfeeding	68·7 (66·0–71·2)	66·5 (64·3–69·0)	62·6 (60·0–65·4)	62·0 (59·3–64·9)	-0·3 (-0·5–-0·2)	-0·3 (-0·5–-0·1)	-0·1 (-0·4–0·2)
Non-exclusive breastfeeding	63·8 (55·5–71·2)	59·1 (51·8–66·0)	48·2 (41·1–55·3)	45·9 (38·6–53·0)	-1·1 (-1·6–-0·6)	-1·2 (-1·9–-0·6)	-0·4 (-1·5–0·5)
Discontinued breastfeeding	88·6 (86·2–91·1)	86·2 (83·5–89·0)	84·2 (80·9–87·8)	84·4 (80·9–87·8)	-0·2 (-0·3–0·0)	-0·1 (-0·3–0·1)	0·0 (-0·3–0·4)
Child growth failure	1·2 (0·4–2·8)	1·1 (0·4–2·5)	1·0 (0·3–2·2)	0·9 (0·3–2·0)	-1·0 (-1·4–-0·7)	-1·1 (-1·6–-0·6)	-0·7 (-1·5–0·1)
Child underweight	2·6 (1·6–3·6)	2·4 (1·6–3·3)	2·0 (1·3–2·8)	1·9 (1·2–2·6)	-1·1 (-1·6–-0·6)	-1·1 (-1·8–-0·5)	-0·6 (-1·6–0·6)
Child wasting	0·8 (0·5–1·3)	0·8 (0·5–1·2)	0·8 (0·5–1·2)	0·8 (0·5–1·2)	-0·3 (-0·7–0·0)	-0·1 (-0·6–0·3)	-0·3 (-1·0–0·4)
Child stunting	3·6 (2·8–4·5)	3·3 (2·6–4·1)	2·8 (2·2–3·5)	2·6 (2·0–3·2)	-1·1 (-1·6–-0·5)	-1·2 (-2·0–-0·4)	-0·8 (-2·2–0·4)
Low birth weight and short gestation	14·5 (12·6–16·8)	15·8 (13·8–18·3)	20·3 (17·7–23·4)	20·2 (17·5–23·2)	1·1 (0·8–1·3)	1·2 (0·7–1·6)	-0·1 (-0·8–0·8)
Short gestation	31·4 (27·8–35·4)	32·4 (28·4–36·6)	36·2 (31·6–41·7)	35·6 (30·7–40·6)	0·4 (0·1–0·7)	0·5 (0·0–0·9)	-0·1 (-1·0–0·8)
Low birth weight	12·0 (10·9–13·3)	13·0 (11·6–14·4)	16·6 (15·1–18·5)	16·7 (15·2–18·3)	1·0 (0·8–1·3)	1·2 (0·8–1·6)	0·0 (-0·7–0·8)

Iron deficiency	3·6 (2·5–5·0)	3·1 (2·1–4·3)	3·1 (2·3–4·3)	3·1 (2·2–4·3)	-0·4 (-1·4–0·4)	0·1 (-1·1–1·3)	0·1 (-1·6–1·8)
Vitamin A deficiency	0·8 (0·0–1·6)	1·0 (0·0–2·0)	0·6 (0·0–1·2)	0·3 (0·0–0·7)	-2·6 (-4·9–0·0)	-5·0 (-7·8–0·0)	-4·9 (-8·5–0·0)
Zinc deficiency	2·0 (0·0–8·5)	1·2 (0·0–4·9)	1·0 (0·0–4·3)	1·0 (0·0–4·0)	-2·3 (-47·3–0·0)	-1·0 (-14·1–3·4)	-0·4 (-3·0–3·7)
Tobacco	38·4 (36·7–39·9)	34·7 (33·2–36·0)	28·8 (27·8–29·8)	25·5 (23·9–27·2)	-1·3 (-1·5–-1·1)	-1·5 (-1·8–-1·1)	-1·1 (-1·7–-0·5)
Smoking	37·2 (34·5–39·7)	33·3 (30·9–35·3)	27·5 (26·0–29·3)	23·9 (22·1–26·3)	-1·4 (-1·7–-1·1)	-1·6 (-2·0–-1·2)	-1·3 (-2·0–-0·6)
Chewing tobacco	1·5 (1·1–2·0)	1·8 (1·3–2·3)	1·9 (1·4–2·6)	2·0 (1·4–2·7)	0·9 (-0·6–2·2)	0·6 (-1·2–2·4)	0·4 (-2·5–3·4)
Second-hand smoke	30·3 (28·6–31·5)	27·3 (25·8–28·3)	22·7 (21·3–23·5)	20·5 (18·9–22·1)	-1·3 (-1·5–-1·0)	-1·4 (-1·7–-1·0)	-0·9 (-1·5–-0·3)
High alcohol use	30·8 (22·5–42·2)	30·5 (21·9–40·4)	31·7 (23·1–42·1)	31·9 (22·4–42·3)	0·1 (-1·0–1·2)	0·2 (-1·1–1·4)	0·1 (-1·5–1·7)
Drug use	0·8 (0·5–1·4)	1·1 (0·9–1·3)	1·8 (1·5–2·2)	4·2 (3·0–5·3)	5·2 (2·8–7·2)	6·5 (4·4–7·9)	7·7 (5·6–9·1)
Dietary risks	36·8 (28·0–47·8)	39·7 (30·4–51·3)	42·4 (33·2–54·3)	43·1 (33·6–55·0)	0·5 (0·2–0·8)	0·4 (0·1–0·7)	0·1 (-0·3–0·6)
Diet low in fruits	36·0 (31·4–39·2)	33·4 (28·9–36·2)	34·0 (29·0–36·8)	35·8 (30·2–39·8)	0·0 (-0·4–0·3)	0·3 (-0·2–0·9)	0·5 (-0·4–1·3)
Diet low in vegetables	26·6 (15·5–34·4)	27·9 (16·1–35·8)	31·3 (17·6–38·7)	31·5 (18·1–39·9)	0·5 (0·2–1·0)	0·6 (0·1–1·1)	0·1 (-0·8–0·8)
Diet low in legumes	38·5 (0·0–48·4)	38·6 (0·0–48·4)	42·1 (0·0–52·9)	42·5 (0·0–53·5)	0·3 (0·0–0·6)	0·5 (0·0–0·9)	0·1 (-0·6–0·9)
Diet low in whole grains	45·4 (38·1–52·0)	48·0 (40·0–54·3)	50·0 (41·3–57·0)	51·0 (42·3–58·5)	0·4 (0·1–0·6)	0·3 (-0·1–0·6)	0·2 (-0·4–0·8)
Diet low in nuts and seeds	13·4 (9·8–17·2)	7·8 (5·0–10·7)	5·2 (3·0–8·0)	5·3 (3·0–8·0)	-3·0 (-4·8–-1·4)	-1·8 (-4·3–0·5)	0·1 (-3·7–3·9)
Diet low in milk	37·0 (32·8–41·7)	34·0 (30·2–39·0)	31·8 (27·1–36·8)	30·9 (26·0–35·9)	-0·6 (-1·0–-0·1)	-0·5 (-1·1–0·1)	-0·3 (-1·2–0·6)
Diet high in red meat	49·3 (0·0–68·3)	49·5 (0·0–68·9)	50·4 (0·0–69·3)	51·2 (0·0–70·2)	0·1 (-0·4–0·6)	0·2 (-0·5–0·8)	0·2 (-0·7–1·1)
Diet high in processed meat	46·6 (36·8–52·4)	57·6 (46·3–65·2)	65·7 (53·0–73·6)	64·8 (52·9–73·1)	1·1 (0·6–1·5)	0·6 (0·0–1·1)	-0·1 (-0·9–0·6)
Diet high in sugar-sweetened beverages	35·6 (27·6–44·7)	49·4 (39·4–58·4)	56·5 (45·4–66·4)	57·7 (46·2–69·2)	1·6 (0·7–2·4)	0·7 (-0·2–1·7)	0·2 (-1·1–1·5)
Diet low in fibre	39·9 (21·5–47·4)	30·2 (16·1–36·6)	25·9 (14·2–32·1)	22·0 (12·3–28·5)	-1·9 (-2·9–-1·1)	-1·5 (-2·9–-0·2)	-1·5 (-3·2–0·4)
Diet low in calcium	5·8 (4·7–7·1)	4·9 (3·9–6·0)	5·0 (4·0–6·2)	4·8 (3·8–5·9)	-0·6 (-0·9–-0·4)	-0·1 (-0·3–0·2)	-0·4 (-0·7–0·0)
Diet low in seafood omega-3 fatty acids	37·4 (28·1–46·8)	44·1 (33·9–54·2)	45·9 (35·6–56·3)	40·2 (30·5–50·6)	0·2 (-0·2–0·7)	-0·4 (-1·0–0·1)	-1·2 (-2·1–-0·5)
Diet low in omega-6 polyunsaturated fatty acids	36·7 (23·7–47·4)	31·7 (21·4–41·0)	22·9 (16·0–30·1)	20·9 (14·1–27·6)	-1·8 (-2·6–-1·0)	-2·0 (-3·0–-1·1)	-0·8 (-1·9–0·1)
Diet high in trans fatty acids	71·4 (64·7–79·0)	71·7 (63·2–79·1)	63·2 (55·3–71·3)	0·0 (0·0–0·0)	-65·8 (-66·1–-65·4)	-97·1 (-97·6–-96·5)	-184·2 (-185·3–-183·0)
Diet high in sodium	23·2 (0·6–68·5)	27·9 (1·7–74·3)	30·8 (3·3–74·9)	31·3 (3·3–78·7)	1·0 (0·2–6·2)	0·6 (-0·2–4·3)	0·2 (-2·3–2·1)
Intimate partner violence	21·0 (13·0–27·6)	20·9 (14·1–26·0)	19·8 (12·0–24·8)	20·8 (9·7–28·2)	0·0 (-1·7–1·3)	0·0 (-2·0–1·6)	0·4 (-1·3–1·7)
Childhood sexual abuse and bullying	7·9 (4·9–13·1)	9·8 (6·1–15·8)	11·3 (7·1–18·2)	8·5 (5·5–12·9)	0·2 (-0·4–0·8)	-0·7 (-1·6–0·1)	-2·7 (-4·2–-1·1)
Childhood sexual abuse	6·1 (5·1–7·1)	7·2 (6·2–8·3)	8·6 (7·8–9·4)	7·7 (6·6–9·5)	0·8 (0·0–1·5)	0·4 (-0·7–1·4)	-0·9 (-2·3–0·7)
Bullying victimization	7·9 (3·5–15·8)	9·9 (4·6–19·4)	11·3 (5·1–21·9)	7·6 (3·4–14·4)	-0·2 (-0·9–0·6)	-1·3 (-2·4–-0·2)	-3·7 (-5·5–-1·7)
Unsafe sex	--	--	--	--	--	--	--
Low physical activity	17·9 (13·3–23·3)	16·5 (12·4–21·2)	17·4 (13·6–21·8)	20·3 (14·9–26·3)	0·4 (-0·6–1·3)	1·0 (-0·3–2·2)	1·4 (-0·5–3·3)
**Metabolic risks**	**25·9 (22·4–29·6)**	**29·3 (25·7–32·5)**	**32·7 (28·8–38·6)**	**36·2 (31·8–39·3)**	**1·1 (0·8–1·4)**	**1·0 (0·7–1·3)**	**0·9 (0·5–1·5)**
High fasting plasma glucose	12·7 (9·7–15·4)	15·7 (11·9–18·8)	19·8 (14·7–24·3)	24·3 (18·1–29·6)	2·1 (1·4–2·8)	2·1 (1·2–3·0)	1·9 (0·5–3·1)
High LDL cholesterol	63·8 (44·5–86·5)	53·9 (37·0–74·2)	48·3 (32·6–67·3)	46·9 (31·4–65·4)	-1·0 (-1·2–-0·8)	-0·7 (-0·9–-0·5)	-0·3 (-0·5–0·0)
High systolic blood pressure	28·3 (19·0–39·3)	23·9 (16·6–33·1)	17·4 (11·3–25·4)	24·9 (15·3–36·5)	-0·4 (-1·4–0·6)	0·2 (-1·1–1·4)	3·3 (0·9–5·6)
High body-mass index	29·3 (24·5–34·1)	33·5 (28·7–37·7)	37·5 (31·8–41·3)	40·6 (34·8–44·6)	1·1 (0·8–1·3)	0·9 (0·6–1·2)	0·7 (0·3–1·2)
Low bone mineral density	21·6 (15·7–28·7)	19·7 (14·3–26·4)	20·5 (14·4–27·2)	22·0 (16·2–28·9)	0·1 (-0·4–0·5)	0·5 (-0·2–1·4)	0·7 (-0·7–2·1)
Kidney dysfunction	2·8 (2·3–3·7)	2·9 (2·3–3·8)	2·8 (2·3–3·8)	2·9 (2·4–3·9)	0·1 (0·0–0·2)	0·1 (-0·1–0·2)	0·4 (0·0–0·7)

Virginia

Risk Names	SEV 1990	SEV 2000	SEV 2010	SEV 2021	Annualised rate of change 1990 to 2021	Annualised rate of change 2000 to 2021	Annualised rate of change 2010 to 2021
**All risk factors**	**27·0 (24·1–30·4)**	**26·7 (23·8–29·6)**	**27·6 (24·5–30·6)**	**27·8 (24·6–30·9)**	**0·1 (-0·2–0·4)**	**0·2 (-0·2–0·6)**	**0·1 (-0·5–0·6)**
**Environmental/occupational risks**	**18·7 (12·3–28·9)**	**18·6 (13·0–29·2)**	**15·9 (10·7–25·5)**	**13·5 (8·8–22·2)**	**-1·1 (-1·9–-0·3)**	**-1·5 (-2·1–-0·9)**	**-1·5 (-2·0–-0·9)**
Unsafe water, sanitation, and handwashing	1·7 (0·8–2·7)	1·6 (0·7–2·6)	1·2 (0·4–2·1)	1·0 (0·3–1·7)	-1·7 (-4·2–0·3)	-2·1 (-5·2–0·8)	-1·6 (-5·3–1·9)
Unsafe water source	2·4 (0·9–4·8)	2·2 (0·9–4·5)	0·9 (0·3–1·7)	0·8 (0·3–1·4)	-3·8 (-6·5–-1·1)	-5·2 (-9·0–-1·3)	-1·6 (-6·9–3·9)
Unsafe sanitation	5·3 (2·8–8·6)	4·2 (2·1–7·0)	3·1 (1·5–5·3)	2·4 (1·2–4·2)	-2·6 (-4·9–-0·5)	-2·7 (-5·5–0·2)	-2·2 (-5·5–1·2)
No access to handwashing facility	1·5 (0·4–2·8)	1·6 (0·5–2·9)	1·6 (0·5–2·9)	1·3 (0·5–2·6)	-0·4 (-3·5–2·5)	-0·7 (-4·7–3·2)	-1·4 (-7·2–3·9)
Air pollution	25·1 (8·4–37·2)	22·6 (9·7–31·3)	11·9 (5·0–21·7)	6·2 (1·7–14·1)	-4·5 (-8·0–-2·2)	-6·2 (-10·6–-3·5)	-6·0 (-12·3–-3·7)
Particulate matter pollution	14·2 (5·3–24·2)	12·5 (9·1–16·4)	7·3 (4·2–10·9)	3·8 (1·3–6·8)	-4·3 (-7·7–-1·5)	-5·7 (-9·5–-4·0)	-5·9 (-11·5–-3·4)
Ambient particulate matter pollution	22·1 (8·6–38·3)	19·3 (14·4–24·7)	11·1 (6·6–16·1)	5·8 (2·0–10·2)	-4·3 (-7·8–-1·5)	-5·7 (-9·6–-4·0)	-6·0 (-11·6–-3·4)
Household air pollution from solid fuels	0·0 (0·0–0·2)	0·0 (0·0–0·1)	0·0 (0·0–0·0)	0·0 (0·0–0·0)	-4·5 (-36·9–-7·9)	-5·1 (-35·3–-7·8)	-3·6 (-21·5–-4·4)
Ambient ozone pollution	29·2 (22·3–37·0)	28·4 (21·7–36·0)	21·5 (15·3–29·1)	12·1 (7·0–18·1)	-2·9 (-3·9–-2·2)	-4·1 (-5·6–-3·1)	-5·2 (-7·8–-3·6)
Ambient nitrogen dioxide pollution	70·2 (0·0–100·0)	64·3 (0·0–100·0)	29·9 (0·0–80·8)	15·4 (0·0–56·7)	-4·9 (-15·4–0·0)	-6·8 (-21·7–0·0)	-6·1 (-25·9–0·0)
Non-optimal temperature	26·4 (20·5–35·9)	26·2 (21·6–34·1)	32·8 (26·1–42·8)	29·0 (23·1–38·7)	0·3 (0·1–0·6)	0·5 (0·0–1·0)	-1·1 (-1·6–-0·7)
High temperature	21·0 (12·9–31·4)	15·6 (9·0–25·5)	33·5 (23·3–44·9)	26·9 (17·8–37·0)	0·8 (0·3–1·4)	2·6 (1·4–4·1)	-2·0 (-3·2–-1·2)
Low temperature	24·3 (20·6–28·5)	25·7 (22·3–29·7)	28·6 (25·5–32·3)	25·9 (22·6–29·8)	0·2 (0·1–0·4)	0·0 (-0·2–0·2)	-0·9 (-1·2–-0·6)
Other environmental risks	27·1 (6·9–43·7)	25·4 (6·9–41·8)	22·7 (6·9–38·5)	19·9 (6·9–35·3)	-1·0 (-1·7–0·0)	-1·2 (-2·0–0·0)	-1·2 (-2·2–0·0)
Residential radon	26·2 (0·0–74·6)	26·2 (0·0–74·6)	26·2 (0·0–74·6)	26·2 (0·0–74·6)	0·0 (0·0–0·0)	0·0 (0·0–0·0)	0·0 (0·0–0·0)
Lead exposure	27·4 (0·0–33·9)	25·0 (0·0–30·8)	21·1 (0·0–26·3)	17·1 (0·0–21·3)	-1·5 (-1·9–0·0)	-1·8 (-2·3–0·0)	-1·9 (-2·7–0·0)
Occupational risks	2·8 (2·3–3·7)	2·9 (2·4–3·9)	2·8 (2·2–3·6)	2·8 (2·2–3·6)	0·0 (-0·3–0·2)	-0·2 (-0·6–0·1)	0·1 (-0·5–0·5)
Occupational carcinogens	1·0 (0·5–1·9)	1·1 (0·5–2·0)	1·0 (0·5–1·9)	1·0 (0·5–2·0)	0·2 (-0·3–0·5)	-0·1 (-0·7–0·2)	0·1 (-0·7–0·6)
Occupational exposure to asbestos	5·1 (4·7–5·7)	5·7 (5·2–6·0)	4·7 (4·3–5·1)	4·1 (3·2–5·1)	-0·7 (-1·5–0·0)	-1·5 (-2·7–-0·5)	-1·3 (-3·4–0·7)
Occupational exposure to arsenic	0·5 (0·0–1·1)	0·5 (0·0–1·2)	0·5 (0·0–1·1)	0·5 (0·0–1·2)	-0·1 (-0·5–0·4)	-0·2 (-0·8–0·4)	0·1 (-0·8–1·1)
Occupational exposure to benzene	1·1 (0·1–3·1)	1·2 (0·1–3·5)	1·2 (0·1–3·5)	1·2 (0·1–3·5)	0·4 (0·0–0·8)	0·2 (-0·3–0·6)	0·3 (-0·3–0·9)
Occupational exposure to beryllium	0·0 (0·0–0·0)	0·0 (0·0–0·0)	0·0 (0·0–0·0)	0·0 (0·0–0·0)	0·3 (0·0–0·5)	0·1 (-0·2–0·4)	0·4 (0·0–0·8)
Occupational exposure to cadmium	0·1 (0·1–0·1)	0·1 (0·1–0·1)	0·1 (0·1–0·1)	0·1 (0·1–0·1)	0·0 (-0·4–0·4)	-0·2 (-0·7–0·4)	0·2 (-0·6–1·0)
Occupational exposure to chromium	0·1 (0·1–0·2)	0·1 (0·1–0·2)	0·1 (0·1–0·1)	0·1 (0·1–0·2)	0·1 (-0·3–0·5)	-0·1 (-0·5–0·4)	0·3 (-0·4–1·0)
Occupational exposure to diesel engine exhaust	0·7 (0·6–0·7)	0·7 (0·7–0·8)	0·7 (0·7–0·7)	0·8 (0·7–0·8)	0·4 (0·1–0·8)	0·4 (-0·2–0·8)	0·7 (-0·1–1·3)
Occupational exposure to formaldehyde	0·3 (0·3–0·3)	0·3 (0·3–0·3)	0·3 (0·3–0·3)	0·3 (0·3–0·3)	-0·1 (-0·5–0·4)	-0·2 (-0·8–0·4)	0·1 (-0·7–0·9)
Occupational exposure to nickel	0·4 (0·0–1·5)	0·4 (0·0–1·5)	0·4 (0·0–1·4)	0·4 (0·0–1·4)	-0·2 (-0·7–0·3)	-0·3 (-1·0–0·5)	0·1 (-0·9–1·3)
Occupational exposure to polycyclic aromatic hydrocarbons	0·3 (0·3–0·3)	0·3 (0·3–0·3)	0·3 (0·3–0·3)	0·3 (0·3–0·3)	0·1 (-0·2–0·5)	0·0 (-0·5–0·5)	0·2 (-0·4–0·9)
Occupational exposure to silica	3·4 (0·5–10·8)	3·3 (0·5–10·7)	3·1 (0·5–10·1)	3·1 (0·5–9·9)	-0·2 (-0·7–0·3)	-0·2 (-0·8–0·5)	0·2 (-0·8–1·3)
Occupational exposure to sulphuric acid	0·7 (0·1–2·3)	0·7 (0·1–2·4)	0·7 (0·1–2·3)	0·7 (0·1–2·3)	0·0 (-0·4–0·4)	-0·1 (-0·7–0·4)	0·2 (-0·7–1·1)
Occupational exposure to trichloroethylene	0·1 (0·1–0·1)	0·1 (0·1–0·1)	0·1 (0·1–0·1)	0·1 (0·1–0·1)	0·2 (-0·1–0·5)	0·1 (-0·3–0·5)	0·4 (-0·2–1·0)
Occupational asthmagens	16·5 (14·4–18·8)	17·1 (15·2–19·3)	16·1 (14·2–18·4)	16·6 (14·5–18·8)	0·0 (-0·3–0·4)	-0·2 (-0·7–0·3)	0·3 (-0·4–0·9)
Occupational particulate matter, gases, and fumes	6·1 (4·6–8·1)	5·9 (4·6–7·9)	5·7 (4·3–7·5)	5·3 (4·1–7·0)	-0·4 (-0·7–-0·1)	-0·5 (-0·9–-0·2)	-0·6 (-1·0–-0·2)
Occupational noise	6·4 (5·8–7·0)	6·5 (6·0–7·1)	6·4 (6·0–7·0)	6·3 (5·9–6·8)	0·0 (-0·2–0·2)	-0·1 (-0·3–0·1)	-0·2 (-0·4–0·1)
Occupational injuries	--	--	--	--	--	--	--
Occupational ergonomic factors	8·2 (6·9–9·8)	8·3 (7·0–10·0)	7·5 (6·4–9·1)	7·7 (6·4–9·4)	-0·2 (-0·6–0·1)	-0·3 (-0·8–0·1)	0·2 (-0·5–0·8)
**Behavioural risks**	**30·2 (27·0–33·9)**	**28·1 (25·0–31·8)**	**26·7 (23·5–30·4)**	**25·0 (21·5–28·6)**	**-0·6 (-0·9–-0·3)**	**-0·6 (-1·0–-0·1)**	**-0·6 (-1·2–0·0)**
Child and maternal malnutrition	10·3 (6·9–14·2)	8·8 (6·1–12·5)	8·8 (6·1–12·5)	9·0 (6·3–12·7)	-0·4 (-1·3–0·5)	0·1 (-1·3–1·4)	0·2 (-1·7–1·9)
Suboptimal breastfeeding	68·7 (66·0–71·3)	66·6 (64·2–69·1)	62·7 (60·2–65·5)	62·2 (59·4–65·1)	-0·3 (-0·5–-0·2)	-0·3 (-0·5–-0·1)	-0·1 (-0·4–0·2)
Non-exclusive breastfeeding	64·0 (55·8–70·7)	59·4 (52·1–65·8)	48·4 (41·6–55·2)	46·3 (39·2–53·8)	-1·0 (-1·5–-0·5)	-1·2 (-1·9–-0·5)	-0·4 (-1·5–0·6)
Discontinued breastfeeding	88·7 (86·5–91·2)	86·2 (83·8–89·1)	84·3 (81·3–87·4)	84·5 (81·3–87·9)	-0·2 (-0·3–0·0)	-0·1 (-0·3–0·1)	0·0 (-0·3–0·3)
Child growth failure	1·3 (0·4–2·8)	1·1 (0·4–2·5)	1·0 (0·3–2·2)	0·9 (0·3–2·1)	-1·0 (-1·5–-0·7)	-1·1 (-1·7–-0·6)	-0·5 (-1·3–0·2)
Child underweight	2·6 (1·7–3·6)	2·4 (1·6–3·3)	2·0 (1·2–2·7)	1·9 (1·2–2·6)	-1·1 (-1·6–-0·6)	-1·2 (-1·9–-0·5)	-0·5 (-1·5–0·6)
Child wasting	0·9 (0·5–1·3)	0·8 (0·5–1·2)	0·8 (0·5–1·2)	0·8 (0·5–1·2)	-0·4 (-0·6–-0·1)	-0·2 (-0·6–0·3)	-0·2 (-0·9–0·5)
Child stunting	3·7 (2·8–4·6)	3·4 (2·6–4·2)	2·8 (2·2–3·6)	2·6 (2·0–3·2)	-1·1 (-1·7–-0·6)	-1·2 (-2·1–-0·5)	-0·8 (-2·0–0·4)
Low birth weight and short gestation	21·5 (18·7–24·5)	23·1 (20·3–26·0)	24·2 (21·1–27·5)	23·5 (20·4–26·9)	0·3 (0·0–0·5)	0·1 (-0·3–0·4)	-0·3 (-1·0–0·5)
Short gestation	40·3 (34·6–46·3)	42·2 (36·7–48·4)	42·8 (36·8–48·9)	41·8 (35·9–47·7)	0·1 (-0·2–0·4)	0·0 (-0·5–0·4)	-0·2 (-1·1–0·7)
Low birth weight	16·5 (14·8–18·3)	17·6 (15·8–19·6)	18·7 (16·8–20·6)	18·3 (16·2–20·4)	0·3 (0·1–0·6)	0·2 (-0·2–0·5)	-0·2 (-0·9–0·5)

Iron deficiency	3·6 (2·5–4·9)	3·1 (2·2–4·3)	3·2 (2·3–4·4)	3·2 (2·3–4·5)	-0·4 (-1·2–0·5)	0·2 (-1·1–1·3)	0·2 (-1·5–1·8)
Vitamin A deficiency	0·8 (0·0–1·8)	1·1 (0·0–2·4)	0·7 (0·0–1·4)	0·4 (0·0–0·9)	-2·4 (-4·7–0·0)	-5·0 (-7·9–0·0)	-4·5 (-8·8–0·0)
Zinc deficiency	1·7 (0·0–7·2)	1·0 (0·0–4·3)	0·9 (0·0–3·8)	0·9 (0·0–3·8)	-2·0 (-43·1–2·0)	-0·5 (-3·2–2·6)	0·2 (-4·3–20·0)
Tobacco	40·8 (39·2–42·5)	36·4 (34·8–37·7)	31·2 (30·1–32·4)	26·7 (24·9–28·6)	-1·4 (-1·6–-1·1)	-1·5 (-1·8–-1·2)	-1·4 (-2·0–-0·8)
Smoking	36·0 (33·5–38·2)	31·2 (29·2–33·2)	26·0 (24·7–27·5)	21·9 (20·0–24·1)	-1·6 (-1·9–-1·3)	-1·7 (-2·1–-1·3)	-1·6 (-2·3–-0·7)
Chewing tobacco	2·0 (1·6–2·5)	2·3 (1·8–2·8)	2·3 (1·7–3·0)	2·2 (1·6–2·9)	0·3 (-1·1–1·4)	-0·2 (-1·9–1·2)	-0·4 (-3·0–1·7)
Second-hand smoke	37·5 (35·3–39·2)	34·6 (32·5–36·1)	30·9 (28·7–32·3)	26·7 (24·4–28·8)	-1·1 (-1·4–-0·9)	-1·2 (-1·6–-0·9)	-1·3 (-1·9–-0·7)
High alcohol use	28·9 (21·1–38·9)	27·3 (20·1–37·2)	28·9 (21·5–39·2)	29·2 (20·9–39·5)	0·0 (-0·9–0·9)	0·3 (-0·9–1·6)	0·1 (-1·5–1·6)
Drug use	0·8 (0·4–1·4)	1·0 (0·8–1·3)	1·7 (1·4–2·1)	3·9 (2·7–5·1)	5·1 (2·3–7·6)	6·4 (3·9–7·9)	7·5 (5·2–8·9)
Dietary risks	36·7 (27·6–47·8)	39·0 (29·7–50·8)	40·7 (31·6–52·8)	41·1 (31·8–53·1)	0·4 (0·1–0·7)	0·3 (-0·1–0·5)	0·1 (-0·4–0·5)
Diet low in fruits	37·4 (32·3–41·1)	34·4 (29·2–37·4)	33·3 (29·3–36·3)	35·2 (30·3–39·4)	-0·2 (-0·6–0·2)	0·1 (-0·4–0·6)	0·5 (-0·3–1·3)
Diet low in vegetables	25·0 (15·3–33·1)	23·7 (14·8–31·5)	24·6 (14·7–32·1)	25·1 (14·8–32·8)	0·0 (-0·5–0·5)	0·3 (-0·3–1·0)	0·2 (-0·8–1·3)
Diet low in legumes	37·2 (0·0–46·9)	37·4 (0·0–47·0)	41·0 (0·0–51·6)	41·8 (0·0–53·0)	0·4 (0·0–0·7)	0·5 (0·0–1·0)	0·2 (-0·6–0·9)
Diet low in whole grains	44·9 (37·1–50·6)	47·9 (39·6–54·3)	49·6 (41·5–57·0)	50·2 (42·1–58·0)	0·4 (0·1–0·6)	0·2 (-0·2–0·6)	0·1 (-0·5–0·7)
Diet low in nuts and seeds	11·2 (7·4–15·0)	6·3 (4·0–9·3)	4·3 (2·4–6·6)	4·7 (2·5–7·5)	-2·8 (-4·8–-1·1)	-1·4 (-4·0–1·1)	1·0 (-3·0–4·8)
Diet low in milk	34·8 (30·2–39·7)	32·1 (27·7–37·0)	30·3 (26·1–35·6)	30·4 (25·4–35·7)	-0·4 (-1·0–0·0)	-0·3 (-0·9–0·3)	0·0 (-0·9–0·9)
Diet high in red meat	49·7 (0·0–68·4)	50·9 (0·0–70·3)	51·7 (0·0–71·0)	51·7 (0·0–71·3)	0·1 (-0·3–0·6)	0·1 (-0·6–0·7)	0·0 (-1·0–0·9)
Diet high in processed meat	45·9 (37·0–51·3)	56·6 (45·5–63·2)	63·4 (50·6–72·0)	61·4 (49·0–69·8)	0·9 (0·5–1·4)	0·4 (-0·2–0·9)	-0·3 (-1·1–0·5)
Diet high in sugar-sweetened beverages	38·1 (29·1–46·1)	52·3 (41·2–60·6)	58·8 (47·1–67·9)	58·0 (45·8–67·5)	1·4 (0·6–2·2)	0·5 (-0·4–1·4)	-0·1 (-1·4–1·0)
Diet low in fibre	37·0 (20·3–44·1)	27·8 (14·9–34·1)	24·0 (12·9–29·9)	21·2 (11·6–27·3)	-1·8 (-2·8–-0·9)	-1·3 (-2·7–-0·1)	-1·1 (-3·3–0·7)
Diet low in calcium	5·3 (4·3–6·5)	4·6 (3·7–5·6)	4·7 (3·8–5·8)	4·7 (3·8–5·8)	-0·4 (-0·6–-0·2)	0·2 (-0·1–0·4)	0·0 (-0·4–0·3)
Diet low in seafood omega-3 fatty acids	34·2 (25·5–44·3)	41·4 (32·0–52·0)	43·5 (33·8–54·2)	39·1 (29·5–49·8)	0·4 (0·0–0·9)	-0·3 (-0·8–0·3)	-1·0 (-1·7–-0·3)
Diet low in omega-6 polyunsaturated fatty acids	33·6 (21·9–44·1)	29·3 (19·3–38·3)	21·4 (14·7–27·8)	20·3 (14·0–27·0)	-1·6 (-2·4–-0·9)	-1·7 (-2·6–-0·9)	-0·5 (-1·4–0·5)
Diet high in trans fatty acids	71·4 (64·1–78·6)	71·9 (64·0–78·9)	63·4 (56·0–71·1)	0·0 (0·0–0·0)	-65·8 (-66·1–-65·4)	-97·1 (-97·6–-96·6)	-184·2 (-185·3–-183·1)
Diet high in sodium	23·6 (0·6–68·7)	28·5 (1·9–77·1)	31·4 (3·1–77·8)	31·9 (2·9–78·1)	1·0 (0·2–6·3)	0·5 (-0·2–3·9)	0·1 (-2·5–1·8)
Intimate partner violence	24·0 (15·4–30·6)	23·3 (14·9–28·1)	20·2 (14·0–24·9)	20·0 (10·7–28·4)	-0·6 (-2·0–1·0)	-0·7 (-2·6–1·3)	-0·1 (-2·6–2·0)
Childhood sexual abuse and bullying	8·5 (5·3–13·7)	9·2 (5·9–14·5)	10·0 (6·7–15·5)	8·1 (5·7–12·3)	-0·2 (-0·9–0·6)	-0·6 (-1·6–0·4)	-1·9 (-3·3–-0·5)
Childhood sexual abuse	7·5 (6·3–8·8)	8·5 (7·3–9·9)	9·8 (8·5–11·3)	9·1 (7·4–11·0)	0·6 (-0·1–1·3)	0·3 (-0·7–1·2)	-0·8 (-2·2–0·7)
Bullying victimization	8·0 (3·5–16·0)	8·3 (3·6–16·4)	8·6 (4·1–16·6)	6·2 (3·0–12·6)	-0·8 (-1·7–0·2)	-1·4 (-2·6–0·0)	-3·0 (-5·0–-1·2)
Unsafe sex	--	--	--	--	--	--	--
Low physical activity	19·0 (14·3–24·3)	17·8 (13·2–22·6)	18·2 (14·1–23·1)	21·0 (15·7–26·7)	0·3 (-0·6–1·3)	0·8 (-0·3–2·0)	1·3 (-0·5–2·9)
**Metabolic risks**	**24·9 (21·9–28·1)**	**30·2 (26·5–33·3)**	**33·9 (29·9–37·0)**	**37·9 (33·1–40·9)**	**1·4 (1·1–1·6)**	**1·1 (0·8–1·4)**	**1·0 (0·5–1·5)**
High fasting plasma glucose	12·9 (9·6–15·7)	16·9 (12·8–20·1)	22·1 (16·8–26·1)	25·4 (19·8–30·3)	2·2 (1·5–2·8)	1·9 (1·1–2·8)	1·3 (0·1–2·4)
High LDL cholesterol	63·6 (44·3–86·7)	53·7 (36·7–74·2)	48·1 (32·5–67·0)	46·6 (31·2–65·2)	-1·0 (-1·2–-0·8)	-0·7 (-0·9–-0·5)	-0·3 (-0·6–0·0)
High systolic blood pressure	31·2 (20·5–43·8)	25·8 (18·0–35·6)	19·0 (12·5–27·4)	27·7 (16·3–40·4)	-0·4 (-1·5–0·7)	0·3 (-1·1–1·6)	3·5 (0·9–6·2)
High body-mass index	28·2 (23·7–32·9)	34·6 (29·5–38·6)	38·8 (33·1–42·6)	42·9 (35·9–46·6)	1·4 (1·0–1·6)	1·0 (0·7–1·3)	0·9 (0·5–1·3)
Low bone mineral density	21·7 (15·6–28·5)	19·6 (13·8–27·0)	20·2 (14·0–27·2)	22·2 (16·3–29·4)	0·1 (-0·4–0·6)	0·6 (-0·2–1·4)	0·8 (-0·5–2·3)
Kidney dysfunction	3·0 (2·4–3·8)	3·0 (2·5–4·0)	3·0 (2·4–4·0)	3·1 (2·5–4·1)	0·1 (0·0–0·3)	0·1 (-0·1–0·2)	0·3 (0·0–0·7)

Washington, DC

Risk Names	SEV 1990	SEV 2000	SEV 2010	SEV 2021	Annualised rate of change 1990 to 2021	Annualised rate of change 2000 to 2021	Annualised rate of change 2010 to 2021
**All risk factors**	**26·3 (23·2–29·4)**	**26·8 (23·9–29·9)**	**27·3 (24·0–30·5)**	**26·9 (23·7–30·2)**	**0·1 (-0·3–0·4)**	**0·0 (-0·4–0·4)**	**-0·1 (-0·7–0·5)**
**Environmental/occupational risks**	**16·6 (10·8–27·8)**	**16·7 (10·7–27·6)**	**14·8 (9·1–24·8)**	**13·4 (8·2–22·9)**	**-0·7 (-1·4–-0·2)**	**-1·1 (-1·4–-0·6)**	**-0·9 (-1·3–-0·4)**
Unsafe water, sanitation, and handwashing	1·8 (0·8–2·9)	1·6 (0·7–2·5)	1·3 (0·4–2·1)	1·0 (0·3–1·7)	-1·8 (-4·1–0·1)	-2·1 (-4·7–0·7)	-1·9 (-5·8–2·1)
Unsafe water source	2·5 (1·0–5·0)	2·2 (0·9–4·6)	0·9 (0·4–1·9)	0·7 (0·3–1·4)	-3·9 (-6·4–-1·2)	-5·2 (-8·8–-1·3)	-2·0 (-8·2–2·9)
Unsafe sanitation	5·6 (3·0–9·2)	4·2 (2·1–7·1)	3·2 (1·7–5·7)	2·4 (1·2–4·1)	-2·7 (-5·0–-0·6)	-2·6 (-5·2–0·0)	-2·6 (-6·1–0·8)
No access to handwashing facility	1·6 (0·4–3·1)	1·6 (0·4–3·0)	1·6 (0·5–2·8)	1·3 (0·5–2·5)	-0·5 (-3·5–2·7)	-0·7 (-4·4–3·5)	-1·6 (-7·2–3·8)
Air pollution	13·8 (2·9–24·9)	13·5 (4·2–23·6)	8·0 (1·8–17·0)	4·8 (0·7–12·5)	-3·4 (-7·3–-1·5)	-5·0 (-10·2–-2·9)	-4·7 (-10·5–-2·6)
Particulate matter pollution	7·3 (1·3–15·6)	6·4 (3·6–9·8)	4·0 (1·5–7·0)	2·2 (0·4–4·8)	-3·8 (-9·1–0·3)	-5·0 (-10·6–-3·1)	-5·2 (-12·9–-2·8)
Ambient particulate matter pollution	11·3 (2·0–23·6)	9·9 (5·5–14·4)	6·1 (2·4–10·4)	3·4 (0·6–7·2)	-3·8 (-9·1–0·2)	-5·0 (-10·7–-3·1)	-5·2 (-13·1–-2·8)
Household air pollution from solid fuels	0·0 (0·0–0·0)	0·0 (0·0–0·0)	0·0 (0·0–0·0)	0·0 (0·0–0·0)	-4·5 (-41·7–-7·8)	-6·6 (-40·1–-8·3)	-8·0 (-41·6–-8·7)
Ambient ozone pollution	5·7 (2·7–9·7)	8·6 (4·5–13·5)	5·6 (2·7–9·9)	10·1 (5·9–15·3)	1·9 (1·0–3·3)	0·8 (-0·3–2·0)	5·3 (3·2–8·7)
Ambient nitrogen dioxide pollution	41·9 (0·0–96·3)	44·6 (0·0–98·5)	25·4 (0·0–74·0)	15·3 (0·0–56·7)	-3·3 (-10·2–0·0)	-5·1 (-15·7–0·0)	-4·6 (-15·4–0·0)
Non-optimal temperature	26·8 (21·7–35·1)	26·1 (21·8–32·3)	25·2 (20·8–31·6)	28·2 (22·7–37·1)	0·2 (0·0–0·4)	0·4 (-0·1–1·0)	1·0 (0·2–2·1)
High temperature	28·3 (17·7–42·6)	23·4 (13·6–37·0)	26·4 (16·2–38·1)	43·4 (29·3–60·9)	1·4 (0·8–2·1)	2·9 (1·8–4·4)	4·5 (2·6–7·0)
Low temperature	24·1 (21·2–29·0)	24·8 (21·6–30·3)	23·3 (20·0–29·1)	24·0 (20·9–28·9)	0·0 (-0·1–0·1)	-0·2 (-0·3–0·0)	0·3 (-0·1–0·6)
Other environmental risks	28·2 (9·9–46·8)	26·6 (9·9–44·8)	23·9 (9·9–42·7)	21·2 (9·4–39·4)	-0·9 (-1·7–0·0)	-1·1 (-2·0–0·0)	-1·1 (-2·2–0·0)
Residential radon	29·9 (0·0–87·5)	29·9 (0·0–87·5)	29·9 (0·0–87·5)	29·9 (0·0–87·5)	0·0 (0·0–0·0)	0·0 (0·0–0·0)	0·0 (0·0–0·0)
Lead exposure	27·4 (0·0–34·3)	25·1 (0·0–31·1)	21·2 (0·0–26·3)	17·3 (0·0–21·6)	-1·5 (-1·9–0·0)	-1·8 (-2·2–0·0)	-1·9 (-2·6–0·0)
Occupational risks	2·9 (2·4–3·8)	3·0 (2·5–3·9)	2·8 (2·3–3·7)	2·8 (2·3–3·7)	-0·1 (-0·4–0·2)	-0·3 (-0·7–0·1)	0·0 (-0·5–0·5)
Occupational carcinogens	1·0 (0·5–1·9)	1·1 (0·5–2·1)	1·0 (0·5–2·0)	1·0 (0·5–2·0)	0·0 (-0·4–0·3)	-0·2 (-0·8–0·2)	0·0 (-0·8–0·6)
Occupational exposure to asbestos	6·1 (5·6–6·6)	6·4 (5·9–6·8)	5·7 (5·3–6·1)	5·0 (4·0–6·0)	-0·7 (-1·4–0·0)	-1·2 (-2·1–-0·2)	-1·3 (-3·1–0·5)
Occupational exposure to arsenic	0·5 (0·0–1·1)	0·5 (0·0–1·2)	0·5 (0·0–1·1)	0·5 (0·0–1·1)	-0·2 (-0·6–0·3)	-0·3 (-0·9–0·4)	0·2 (-0·6–1·0)
Occupational exposure to benzene	1·1 (0·1–3·1)	1·2 (0·1–3·4)	1·1 (0·1–3·4)	1·2 (0·1–3·5)	0·3 (0·0–0·7)	0·1 (-0·3–0·5)	0·3 (-0·3–0·9)
Occupational exposure to beryllium	0·0 (0·0–0·0)	0·0 (0·0–0·0)	0·0 (0·0–0·0)	0·0 (0·0–0·0)	0·2 (-0·1–0·4)	0·1 (-0·3–0·4)	0·5 (0·0–1·0)
Occupational exposure to cadmium	0·1 (0·1–0·1)	0·1 (0·1–0·1)	0·1 (0·1–0·1)	0·1 (0·1–0·1)	-0·1 (-0·5–0·3)	-0·2 (-0·8–0·4)	0·2 (-0·5–1·0)
Occupational exposure to chromium	0·1 (0·1–0·2)	0·1 (0·1–0·2)	0·1 (0·1–0·1)	0·1 (0·1–0·2)	0·0 (-0·4–0·4)	-0·1 (-0·6–0·4)	0·3 (-0·3–0·9)
Occupational exposure to diesel engine exhaust	0·7 (0·6–0·7)	0·7 (0·6–0·7)	0·7 (0·6–0·7)	0·7 (0·7–0·8)	0·2 (-0·1–0·6)	0·2 (-0·3–0·8)	0·7 (-0·1–1·4)
Occupational exposure to formaldehyde	0·3 (0·3–0·3)	0·3 (0·3–0·3)	0·3 (0·3–0·3)	0·3 (0·3–0·3)	-0·1 (-0·6–0·3)	-0·3 (-0·9–0·3)	0·1 (-0·6–0·9)
Occupational exposure to nickel	0·4 (0·0–1·5)	0·4 (0·0–1·5)	0·4 (0·0–1·4)	0·4 (0·0–1·4)	-0·2 (-0·8–0·3)	-0·3 (-1·1–0·4)	0·1 (-0·8–1·1)
Occupational exposure to polycyclic aromatic hydrocarbons	0·3 (0·3–0·3)	0·3 (0·3–0·3)	0·3 (0·3–0·3)	0·3 (0·3–0·3)	0·0 (-0·3–0·4)	-0·1 (-0·6–0·4)	0·3 (-0·4–0·9)
Occupational exposure to silica	3·4 (0·5–10·7)	3·3 (0·5–10·5)	3·0 (0·5–9·4)	3·1 (0·5–9·6)	-0·3 (-0·8–0·2)	-0·2 (-0·9–0·4)	0·3 (-0·7–1·1)
Occupational exposure to sulphuric acid	0·7 (0·1–2·4)	0·7 (0·1–2·4)	0·7 (0·1–2·2)	0·7 (0·1–2·2)	-0·1 (-0·5–0·3)	-0·2 (-0·8–0·4)	0·2 (-0·5–1·1)
Occupational exposure to trichloroethylene	0·1 (0·1–0·1)	0·1 (0·1–0·1)	0·1 (0·1–0·1)	0·1 (0·1–0·1)	0·1 (-0·2–0·4)	0·0 (-0·4–0·4)	0·4 (-0·1–1·0)
Occupational asthmagens	16·9 (14·7–19·4)	17·3 (15·1–19·7)	16·0 (14·1–18·2)	16·5 (14·4–18·9)	-0·1 (-0·4–0·3)	-0·2 (-0·7–0·2)	0·3 (-0·4–0·9)
Occupational particulate matter, gases, and fumes	6·5 (4·8–8·6)	6·3 (4·8–8·4)	5·9 (4·5–7·9)	5·5 (4·2–7·3)	-0·5 (-0·8–-0·2)	-0·6 (-1·0–-0·3)	-0·7 (-1·1–-0·3)
Occupational noise	6·7 (6·1–7·3)	6·7 (6·2–7·3)	6·6 (6·2–7·1)	6·4 (6·0–6·9)	-0·1 (-0·3–0·0)	-0·2 (-0·4–-0·1)	-0·3 (-0·5–-0·1)
Occupational injuries	--	--	--	--	--	--	--
Occupational ergonomic factors	8·1 (6·8–9·7)	8·0 (6·7–9·7)	7·2 (6·0–8·7)	7·4 (6·1–9·2)	-0·3 (-0·7–0·2)	-0·4 (-0·9–0·2)	0·2 (-0·5–1·0)
**Behavioural risks**	**30·4 (27·0–33·9)**	**28·6 (25·4–32·2)**	**26·1 (22·6–29·6)**	**24·5 (20·8–28·4)**	**-0·7 (-1·1–-0·4)**	**-0·7 (-1·3–-0·3)**	**-0·6 (-1·2–0·1)**
Child and maternal malnutrition	9·9 (6·7–13·7)	8·3 (5·7–11·6)	8·5 (5·8–11·8)	8·6 (5·8–12·1)	-0·4 (-1·3–0·4)	0·2 (-1·0–1·4)	0·2 (-1·6–1·8)
Suboptimal breastfeeding	68·7 (66·2–71·3)	66·6 (64·3–69·2)	62·8 (60·1–65·5)	62·2 (59·4–65·2)	-0·3 (-0·5–-0·2)	-0·3 (-0·5–-0·1)	-0·1 (-0·4–0·2)
Non-exclusive breastfeeding	63·9 (55·4–71·1)	59·4 (53·0–65·7)	48·7 (41·6–55·4)	46·4 (39·0–53·8)	-1·0 (-1·6–-0·5)	-1·2 (-1·9–-0·6)	-0·4 (-1·5–0·5)
Discontinued breastfeeding	88·6 (86·5–90·8)	86·3 (83·7–89·1)	84·3 (81·1–87·7)	84·6 (81·0–87·9)	-0·2 (-0·3–0·0)	-0·1 (-0·3–0·1)	0·0 (-0·2–0·3)
Child growth failure	1·2 (0·4–2·8)	1·1 (0·4–2·5)	1·0 (0·3–2·1)	0·9 (0·3–1·9)	-1·0 (-1·4–-0·7)	-1·1 (-1·7–-0·7)	-0·8 (-1·5–0·0)
Child underweight	2·6 (1·6–3·6)	2·4 (1·5–3·2)	2·0 (1·3–2·7)	1·8 (1·1–2·5)	-1·1 (-1·6–-0·6)	-1·2 (-1·9–-0·6)	-0·7 (-1·6–0·3)
Child wasting	0·8 (0·5–1·3)	0·8 (0·5–1·2)	0·8 (0·5–1·2)	0·8 (0·5–1·1)	-0·4 (-0·7–0·0)	-0·2 (-0·6–0·3)	-0·3 (-1·0–0·3)
Child stunting	3·6 (2·8–4·4)	3·3 (2·6–4·1)	2·8 (2·1–3·5)	2·5 (1·9–3·1)	-1·1 (-1·7–-0·5)	-1·2 (-2·1–-0·4)	-0·9 (-2·1–0·3)
Low birth weight and short gestation	17·3 (15·2–19·6)	18·9 (16·6–21·6)	19·4 (16·9–22·0)	20·5 (17·8–23·2)	0·5 (0·3–0·8)	0·4 (-0·1–0·8)	0·5 (-0·4–1·3)
Short gestation	34·2 (29·9–39·1)	36·9 (32·6–41·9)	34·0 (29·1–38·7)	34·6 (29·9–39·5)	0·0 (-0·3–0·4)	-0·3 (-0·8–0·2)	0·2 (-0·8–1·1)
Low birth weight	12·6 (11·4–14·0)	13·6 (12·3–15·0)	14·9 (13·5–16·4)	16·0 (14·5–17·4)	0·8 (0·5–1·0)	0·7 (0·3–1·1)	0·6 (-0·2–1·4)

Iron deficiency	3·4 (2·4–4·7)	2·9 (2·1–4·0)	3·0 (2·1–4·0)	3·0 (2·1–4·1)	-0·4 (-1·3–0·4)	0·2 (-1·0–1·3)	0·1 (-1·5–1·7)
Vitamin A deficiency	0·9 (0·0–1·8)	1·1 (0·0–2·3)	0·7 (0·0–1·5)	0·4 (0·0–0·9)	-2·4 (-4·8–0·0)	-4·7 (-7·7–0·0)	-4·8 (-8·9–0·0)
Zinc deficiency	1·8 (0·0–7·6)	1·0 (0·0–4·5)	0·9 (0·0–4·1)	0·9 (0·0–3·8)	-2·3 (-47·8–1·6)	-0·7 (-6·2–2·4)	-0·3 (-8·9–5·6)
Tobacco	38·7 (37·0–40·2)	33·7 (32·3–34·9)	25·6 (24·6–26·6)	22·3 (20·7–23·9)	-1·8 (-2·0–-1·6)	-2·0 (-2·3–-1·6)	-1·3 (-2·0–-0·7)
Smoking	36·9 (34·3–39·4)	31·4 (29·3–33·4)	23·9 (22·4–25·4)	20·2 (18·3–22·2)	-1·9 (-2·3–-1·6)	-2·1 (-2·5–-1·7)	-1·5 (-2·3–-0·8)
Chewing tobacco	1·5 (1·2–1·9)	1·8 (1·4–2·3)	1·8 (1·3–2·5)	1·7 (1·2–2·5)	0·5 (-0·9–1·9)	-0·1 (-2·0–1·8)	-0·3 (-3·2–2·8)
Second-hand smoke	30·7 (29·0–31·9)	27·1 (25·4–28·3)	20·8 (19·1–21·8)	18·6 (16·8–20·1)	-1·6 (-1·9–-1·4)	-1·8 (-2·2–-1·4)	-1·0 (-1·8–-0·4)
High alcohol use	29·6 (20·9–40·1)	28·7 (21·1–39·1)	29·5 (21·1–39·7)	29·9 (20·9–41·2)	0·0 (-1·0–1·0)	0·2 (-1·2–1·4)	0·1 (-1·6–1·9)
Drug use	1·0 (0·5–2·0)	1·3 (1·1–1·8)	1·8 (1·5–2·1)	4·4 (3·5–5·4)	4·8 (2·1–7·8)	5·7 (3·6–7·1)	8·4 (5·4–10·4)
Dietary risks	36·8 (27·5–47·2)	40·3 (30·9–51·7)	42·9 (33·7–55·1)	42·8 (33·7–54·6)	0·5 (0·2–0·8)	0·3 (0·0–0·6)	0·0 (-0·5–0·4)
Diet low in fruits	36·6 (31·0–40·1)	34·2 (29·1–37·0)	34·6 (30·1–37·4)	35·1 (29·5–39·3)	-0·1 (-0·5–0·2)	0·1 (-0·4–0·6)	0·1 (-0·6–1·0)
Diet low in vegetables	25·2 (15·2–32·6)	27·3 (15·9–34·8)	31·0 (17·1–38·5)	30·1 (17·1–38·5)	0·6 (0·2–1·0)	0·5 (0·0–1·0)	-0·3 (-1·1–0·6)
Diet low in legumes	37·6 (0·0–46·8)	37·5 (0·0–46·9)	41·5 (0·0–52·2)	41·7 (0·0–52·6)	0·3 (0·0–0·7)	0·5 (0·0–1·0)	0·0 (-0·7–0·8)
Diet low in whole grains	44·8 (37·2–51·1)	48·3 (40·3–55·0)	50·5 (42·0–57·4)	50·6 (42·2–58·1)	0·4 (0·1–0·7)	0·2 (-0·1–0·6)	0·0 (-0·5–0·5)
Diet low in nuts and seeds	12·0 (8·6–15·9)	6·5 (4·0–9·3)	4·6 (2·7–6·9)	4·7 (2·5–7·4)	-3·0 (-5·0–-1·2)	-1·6 (-4·3–1·2)	0·1 (-4·1–4·1)
Diet low in milk	35·1 (30·8–40·1)	31·7 (27·4–36·8)	30·4 (26·3–35·4)	29·7 (25·3–35·4)	-0·5 (-1·0–-0·1)	-0·3 (-1·0–0·3)	-0·2 (-1·2–0·6)
Diet high in red meat	49·6 (0·0–66·9)	50·7 (0·0–68·8)	51·2 (0·0–69·7)	51·4 (0·0–70·3)	0·1 (-0·4–0·6)	0·1 (-0·6–0·7)	0·0 (-0·8–1·0)
Diet high in processed meat	46·9 (37·5–52·3)	59·2 (49·0–65·8)	66·8 (54·5–75·2)	65·0 (53·9–73·7)	1·1 (0·6–1·5)	0·4 (-0·1–1·0)	-0·3 (-0·9–0·4)
Diet high in sugar-sweetened beverages	37·0 (28·4–45·5)	52·6 (42·2–60·9)	58·4 (47·8–68·2)	58·5 (47·5–68·9)	1·5 (0·7–2·3)	0·5 (-0·3–1·4)	0·0 (-1·2–1·2)
Diet low in fibre	38·1 (20·9–45·8)	27·9 (15·9–34·3)	24·7 (13·7–31·3)	20·8 (12·1–27·2)	-2·0 (-2·9–-1·0)	-1·4 (-2·7–-0·2)	-1·6 (-3·4–0·3)
Diet low in calcium	5·4 (4·3–6·7)	4·5 (3·6–5·5)	4·8 (3·8–5·9)	4·6 (3·7–5·6)	-0·5 (-0·7–-0·3)	0·1 (-0·1–0·3)	-0·3 (-0·6–0·1)
Diet low in seafood omega-3 fatty acids	35·0 (25·8–44·9)	41·1 (31·4–51·4)	44·3 (34·3–54·5)	37·5 (27·7–47·5)	0·2 (-0·2–0·7)	-0·4 (-1·0–0·1)	-1·5 (-2·3–-0·7)
Diet low in omega-6 polyunsaturated fatty acids	34·4 (22·4–43·2)	29·4 (19·9–37·1)	22·0 (15·3–28·5)	19·7 (13·7–26·0)	-1·8 (-2·6–-1·0)	-1·9 (-2·9–-1·0)	-1·0 (-1·9–-0·1)
Diet high in trans fatty acids	71·3 (63·6–78·6)	71·8 (65·3–78·8)	63·3 (55·4–71·1)	0·0 (0·0–0·0)	-65·8 (-66·1–-65·4)	-97·1 (-97·5–-96·7)	-184·2 (-185·3–-183·0)
Diet high in sodium	23·1 (0·5–69·0)	28·0 (1·9–75·6)	30·9 (3·0–75·0)	31·4 (3·1–77·2)	1·0 (0·3–6·4)	0·5 (-0·1–3·8)	0·1 (-2·4–2·2)
Intimate partner violence	31·9 (21·4–40·6)	33·5 (22·6–41·8)	29·0 (18·9–35·4)	27·1 (16·5–35·9)	-0·5 (-1·7–0·6)	-1·0 (-2·6–0·7)	-0·6 (-2·9–1·2)
Childhood sexual abuse and bullying	9·0 (5·9–14·2)	10·9 (7·1–17·0)	12·5 (8·2–19·7)	10·2 (6·7–15·7)	0·4 (-0·2–1·0)	-0·3 (-1·2–0·5)	-1·9 (-3·3–-0·4)
Childhood sexual abuse	8·6 (7·4–10·0)	9·7 (8·5–10·9)	11·2 (10·4–12·3)	10·6 (9·0–12·6)	0·7 (0·0–1·4)	0·4 (-0·5–1·4)	-0·6 (-1·9–0·8)
Bullying victimization	8·0 (3·5–16·0)	10·0 (4·6–19·5)	11·4 (5·1–22·1)	8·3 (3·6–16·2)	0·1 (-0·6–0·9)	-0·9 (-1·9–0·2)	-2·8 (-4·6–-0·8)
Unsafe sex	--	--	--	--	--	--	--
Low physical activity	18·5 (13·8–23·7)	18·0 (13·5–22·6)	19·8 (15·5–24·3)	22·5 (17·1–28·8)	0·6 (-0·3–1·6)	1·1 (-0·1–2·3)	1·2 (-0·4–3·0)
**Metabolic risks**	**24·7 (21·4–28·0)**	**30·2 (26·5–33·3)**	**33·9 (29·8–36·8)**	**36·1 (31·5–39·1)**	**1·2 (0·9–1·5)**	**0·8 (0·5–1·2)**	**0·6 (0·1–1·0)**
High fasting plasma glucose	12·0 (9·1–14·5)	15·5 (11·9–18·6)	19·9 (14·9–23·9)	23·8 (18·0–28·2)	2·2 (1·5–2·9)	2·0 (1·2–3·0)	1·6 (0·4–2·9)
High LDL cholesterol	63·5 (44·4–86·4)	53·8 (36·8–74·3)	48·1 (32·4–67·0)	46·6 (31·3–64·9)	-1·0 (-1·2–-0·8)	-0·7 (-0·9–-0·5)	-0·3 (-0·6–0·0)
High systolic blood pressure	28·2 (19·0–39·4)	24·1 (16·7–33·6)	17·9 (11·5–25·7)	25·1 (15·4–35·8)	-0·4 (-1·3–0·5)	0·2 (-1·1–1·4)	3·1 (0·9–5·2)
High body-mass index	28·1 (23·5–32·6)	34·8 (29·4–38·8)	39·2 (33·3–43·1)	40·8 (34·6–44·6)	1·2 (0·9–1·5)	0·7 (0·4–1·0)	0·4 (-0·1–0·7)
Low bone mineral density	21·7 (15·4–28·8)	19·4 (13·8–26·6)	19·7 (14·0–26·6)	21·7 (15·2–29·0)	0·0 (-0·5–0·5)	0·5 (-0·3–1·3)	0·9 (-0·5–2·2)
Kidney dysfunction	2·8 (2·3–3·7)	2·9 (2·3–3·8)	2·8 (2·3–3·8)	2·9 (2·4–3·8)	0·1 (0·0–0·2)	0·1 (0·0–0·2)	0·4 (0·1–0·7)

Washington, DC

Risk Names	SEV 1990	SEV 2000	SEV 2010	SEV 2021	Annualised rate of change 1990 to 2021	Annualised rate of change 2000 to 2021	Annualised rate of change 2010 to 2021
**All risk factors**	**26·2 (23·3–29·1)**	**26·5 (23·6–29·3)**	**28·4 (25·3–31·1)**	**28·2 (25·2–31·1)**	**0·2 (0·0–0·5)**	**0·3 (0·0–0·6)**	**-0·1 (-0·6–0·4)**
**Environmental/occupational risks**	**21·3 (13·4–31·9)**	**20·7 (13·7–31·1)**	**18·0 (11·7–28·5)**	**15·1 (9·4–24·5)**	**-1·1 (-1·8–-0·4)**	**-1·5 (-2·0–-0·9)**	**-1·6 (-2·2–-0·9)**
Unsafe water, sanitation, and handwashing	1·7 (0·9–2·9)	1·3 (0·6–2·3)	1·1 (0·3–1·9)	0·9 (0·3–1·6)	-2·0 (-4·5–0·1)	-1·7 (-4·7–1·0)	-1·3 (-5·4–2·7)
Unsafe water source	2·5 (1·0–5·2)	1·9 (0·8–4·0)	0·8 (0·3–1·7)	0·7 (0·3–1·4)	-4·0 (-6·9–-1·2)	-4·7 (-8·3–-0·9)	-1·2 (-6·8–3·8)
Unsafe sanitation	5·5 (2·9–8·9)	3·2 (1·7–5·5)	2·5 (1·3–4·4)	2·1 (1·1–3·6)	-3·1 (-5·3–-1·0)	-2·0 (-4·8–0·6)	-1·4 (-4·8–1·8)
No access to handwashing facility	1·5 (0·5–2·9)	1·4 (0·4–2·6)	1·4 (0·5–2·7)	1·2 (0·4–2·3)	-0·6 (-3·6–2·4)	-0·5 (-4·6–3·7)	-1·2 (-6·9–4·3)
Air pollution	30·1 (10·6–41·7)	28·2 (11·7–34·2)	23·1 (7·5–29·5)	12·8 (3·9–23·6)	-2·7 (-5·5–-1·0)	-3·8 (-7·7–-1·4)	-5·4 (-10·9–-1·4)
Particulate matter pollution	17·1 (7·3–27·9)	15·1 (11·2–19·6)	10·2 (6·8–13·9)	6·1 (3·1–9·2)	-3·3 (-5·6–-0·9)	-4·3 (-6·4–-3·2)	-4·7 (-8·0–-2·9)
Ambient particulate matter pollution	26·7 (11·4–44·7)	23·4 (18·0–29·0)	15·7 (10·8–20·8)	9·3 (4·8–13·9)	-3·4 (-5·8–-0·9)	-4·4 (-6·5–-3·3)	-4·7 (-8·1–-3·0)
Household air pollution from solid fuels	0·0 (0·0–0·0)	0·0 (0·0–0·0)	0·0 (0·0–0·0)	0·0 (0·0–0·0)	-4·2 (-58·0–20·8)	-4·8 (-72·2–60·2)	-2·3 (-110·2–170·3)
Ambient ozone pollution	30·4 (21·8–43·3)	23·8 (15·3–35·5)	21·3 (11·8–32·6)	15·4 (6·9–25·0)	-2·2 (-4·2–-0·8)	-2·1 (-5·5–0·2)	-3·0 (-8·5–1·3)
Ambient nitrogen dioxide pollution	84·2 (0·0–100·0)	84·4 (0·0–100·0)	79·6 (0·0–100·0)	41·7 (0·0–100·0)	-2·3 (-8·2–0·0)	-3·4 (-12·1–0·0)	-5·9 (-18·1–0·0)
Non-optimal temperature	27·7 (21·8–37·4)	27·2 (22·2–35·8)	33·7 (26·4–45·1)	29·8 (23·5–40·3)	0·2 (0·0–0·5)	0·4 (0·0–0·9)	-1·1 (-1·8–-0·6)
High temperature	22·3 (13·1–33·9)	17·8 (9·9–29·2)	36·8 (25·6–49·0)	28·5 (18·6–39·3)	0·8 (0·3–1·5)	2·2 (1·1–3·9)	-2·3 (-3·4–-1·5)
Low temperature	25·7 (22·3–30·1)	26·6 (23·2–30·5)	29·0 (25·7–33·0)	26·6 (23·2–30·9)	0·1 (0·0–0·2)	0·0 (-0·2–0·2)	-0·8 (-1·1–-0·5)
Other environmental risks	38·3 (7·2–58·1)	34·9 (7·3–54·3)	29·9 (7·2–48·9)	24·9 (7·2–43·7)	-1·4 (-2·1–0·0)	-1·6 (-2·5–0·0)	-1·7 (-2·7–0·0)
Residential radon	26·3 (0·0–80·2)	26·3 (0·0–80·2)	26·3 (0·0–80·2)	26·3 (0·0–80·2)	0·0 (0·0–0·0)	0·0 (0·0–0·0)	0·0 (0·0–0·0)
Lead exposure	43·6 (0·0–54·0)	38·6 (0·0–47·6)	31·4 (0·0–38·5)	24·2 (0·0–30·0)	-1·9 (-2·3–0·0)	-2·2 (-2·7–0·0)	-2·4 (-3·1–0·0)
Occupational risks	2·2 (1·8–2·9)	2·3 (1·8–3·1)	2·3 (1·8–3·1)	2·3 (1·8–3·2)	0·2 (-0·1–0·5)	0·0 (-0·4–0·3)	0·2 (-0·3–0·7)
Occupational carcinogens	0·8 (0·3–1·6)	0·9 (0·4–1·8)	0·9 (0·4–1·8)	0·9 (0·4–1·9)	0·3 (0·0–0·6)	0·0 (-0·5–0·3)	0·2 (-0·5–0·7)
Occupational exposure to asbestos	2·0 (1·8–2·3)	2·3 (2·1–2·6)	2·1 (1·9–2·3)	1·9 (1·5–2·4)	-0·2 (-1·1–0·6)	-0·9 (-2·2–0·4)	-0·9 (-3·3–1·5)
Occupational exposure to arsenic	0·4 (0·0–0·8)	0·4 (0·0–0·9)	0·4 (0·0–0·9)	0·4 (0·0–0·9)	0·2 (-0·2–0·7)	0·1 (-0·4–0·7)	0·4 (-0·5–1·2)
Occupational exposure to benzene	1·1 (0·1–3·1)	1·2 (0·1–3·6)	1·2 (0·1–3·6)	1·2 (0·1–3·7)	0·4 (0·0–0·8)	0·1 (-0·4–0·5)	0·3 (-0·5–0·9)
Occupational exposure to beryllium	0·0 (0·0–0·0)	0·0 (0·0–0·0)	0·0 (0·0–0·0)	0·0 (0·0–0·0)	0·3 (0·1–0·6)	0·2 (-0·1–0·6)	0·5 (0·1–1·0)
Occupational exposure to cadmium	0·0 (0·0–0·0)	0·0 (0·0–0·0)	0·0 (0·0–0·0)	0·0 (0·0–0·1)	0·3 (-0·1–0·6)	0·2 (-0·3–0·7)	0·5 (-0·3–1·2)
Occupational exposure to chromium	0·1 (0·1–0·1)	0·1 (0·1–0·1)	0·1 (0·1–0·1)	0·1 (0·1–0·1)	0·4 (0·1–0·7)	0·2 (-0·2–0·7)	0·5 (-0·2–1·2)
Occupational exposure to diesel engine exhaust	0·6 (0·5–0·6)	0·6 (0·6–0·7)	0·6 (0·6–0·7)	0·7 (0·6–0·8)	0·7 (0·3–1·1)	0·6 (0·0–1·2)	0·9 (0·1–1·7)
Occupational exposure to formaldehyde	0·2 (0·2–0·2)	0·2 (0·2–0·2)	0·2 (0·2–0·2)	0·2 (0·2–0·3)	0·3 (-0·1–0·6)	0·1 (-0·4–0·6)	0·3 (-0·5–1·1)
Occupational exposure to nickel	0·3 (0·0–1·1)	0·3 (0·0–1·1)	0·3 (0·0–1·1)	0·3 (0·0–1·1)	0·2 (-0·3–0·6)	0·1 (-0·6–0·8)	0·4 (-0·5–1·5)
Occupational exposure to polycyclic aromatic hydrocarbons	0·2 (0·2–0·2)	0·2 (0·2–0·3)	0·2 (0·2–0·3)	0·3 (0·2–0·3)	0·4 (0·1–0·7)	0·2 (-0·2–0·6)	0·4 (-0·2–1·0)
Occupational exposure to silica	2·2 (0·3–7·0)	2·2 (0·3–7·0)	2·2 (0·3–6·8)	2·3 (0·4–7·1)	0·2 (-0·2–0·7)	0·2 (-0·4–0·9)	0·6 (-0·4–1·5)
Occupational exposure to sulphuric acid	0·5 (0·1–1·7)	0·5 (0·1–1·8)	0·5 (0·1–1·8)	0·6 (0·1–1·9)	0·4 (0·0–0·8)	0·3 (-0·3–0·8)	0·6 (-0·3–1·4)
Occupational exposure to trichloroethylene	0·1 (0·1–0·1)	0·1 (0·1–0·1)	0·1 (0·1–0·1)	0·1 (0·1–0·1)	0·5 (0·2–0·8)	0·4 (-0·1–0·8)	0·6 (0·0–1·3)
Occupational asthmagens	13·1 (11·5–15·2)	14·1 (12·3–16·0)	13·6 (11·9–15·6)	14·2 (12·4–16·4)	0·3 (-0·1–0·7)	0·0 (-0·5–0·6)	0·4 (-0·4–1·2)
Occupational particulate matter, gases, and fumes	3·7 (2·8–4·9)	3·7 (2·8–4·9)	3·6 (2·8–4·7)	3·5 (2·7–4·7)	-0·1 (-0·4–0·2)	-0·2 (-0·5–0·2)	-0·2 (-0·6–0·2)
Occupational noise	4·9 (4·5–5·4)	5·1 (4·7–5·6)	5·2 (4·8–5·7)	5·2 (4·9–5·7)	0·2 (0·0–0·4)	0·1 (-0·1–0·3)	0·0 (-0·2–0·2)
Occupational injuries	--	--	--	--	--	--	--
Occupational ergonomic factors	7·1 (5·9–8·5)	7·1 (5·9–8·6)	6·6 (5·5–8·0)	6·8 (5·5–8·4)	-0·1 (-0·6–0·2)	-0·2 (-0·7–0·3)	0·3 (-0·5–0·9)
**Behavioural risks**	**28·6 (25·3–31·9)**	**27·4 (24·1–31·0)**	**26·8 (23·4–30·5)**	**25·4 (22·1–29·3)**	**-0·4 (-0·7–-0·1)**	**-0·4 (-0·9–0·0)**	**-0·5 (-1·2–0·1)**
Child and maternal malnutrition	11·9 (7·8–16·6)	9·9 (6·7–13·4)	9·8 (6·7–13·7)	9·7 (6·4–13·6)	-0·7 (-1·5–0·1)	-0·1 (-1·3–1·0)	-0·1 (-1·9–1·5)
Suboptimal breastfeeding	68·6 (66·0–71·3)	66·3 (63·8–68·9)	62·3 (59·6–65·2)	61·6 (58·7–64·7)	-0·3 (-0·5–-0·2)	-0·3 (-0·5–-0·2)	-0·1 (-0·4–0·2)
Non-exclusive breastfeeding	63·8 (56·4–71·2)	58·7 (51·7–65·9)	47·4 (40·8–54·2)	45·2 (38·3–52·7)	-1·1 (-1·6–-0·6)	-1·2 (-1·9–-0·5)	-0·4 (-1·5–0·6)
Discontinued breastfeeding	88·6 (86·2–90·9)	86·2 (83·4–89·0)	84·0 (80·7–87·5)	84·1 (80·6–87·5)	-0·2 (-0·3–0·0)	-0·1 (-0·3–0·1)	0·0 (-0·3–0·3)
Child growth failure	1·7 (0·5–4·1)	1·4 (0·4–3·1)	1·1 (0·4–2·5)	1·0 (0·3–2·2)	-1·8 (-2·3–-1·5)	-1·5 (-2·1–-1·0)	-0·8 (-1·7–-0·1)
Child underweight	3·7 (2·3–5·1)	2·9 (1·8–3·9)	2·2 (1·3–3·1)	2·1 (1·2–2·8)	-1·9 (-2·4–-1·4)	-1·6 (-2·3–-0·9)	-0·8 (-1·8–0·3)
Child wasting	1·1 (0·7–1·7)	0·9 (0·6–1·4)	0·8 (0·5–1·3)	0·8 (0·5–1·3)	-1·0 (-1·3–-0·7)	-0·5 (-1·0–-0·1)	-0·5 (-1·1–0·2)
Child stunting	5·0 (3·8–6·5)	3·9 (3·1–5·0)	3·2 (2·4–4·1)	2·8 (2·2–3·7)	-1·8 (-2·4–-1·3)	-1·6 (-2·3–-0·8)	-1·0 (-2·3–0·2)
Low birth weight and short gestation	26·5 (23·2–30·4)	27·0 (23·6–30·9)	27·2 (23·7–31·2)	26·0 (22·5–29·5)	-0·1 (-0·4–0·2)	-0·2 (-0·5–0·1)	-0·4 (-1·1–0·2)
Short gestation	47·2 (41·5–54·1)	47·9 (42·0–53·8)	47·4 (42·2–53·6)	43·9 (38·7–49·7)	-0·2 (-0·6–0·1)	-0·4 (-0·8–0·0)	-0·7 (-1·6–0·1)
Low birth weight	20·0 (17·9–22·3)	20·4 (18·4–22·4)	20·7 (18·7–22·9)	20·5 (18·4–22·6)	0·1 (-0·2–0·3)	0·0 (-0·3–0·4)	-0·1 (-0·6–0·6)

Iron deficiency	4·5 (3·1–6·2)	3·7 (2·6–4·9)	3·7 (2·6–5·0)	3·6 (2·5–5·0)	-0·7 (-1·5–0·1)	-0·1 (-1·2–1·0)	-0·1 (-1·7–1·4)
Vitamin A deficiency	0·9 (0·0–1·8)	0·8 (0·0–1·7)	0·4 (0·0–1·0)	0·3 (0·0–0·6)	-3·7 (-6·0–0·0)	-5·2 (-8·1–0·0)	-4·5 (-8·3–0·0)
Zinc deficiency	1·3 (0·0–6·1)	0·8 (0·0–3·5)	0·6 (0·0–2·8)	0·6 (0·0–2·6)	-2·5 (-47·5–35·5)	-1·2 (-9·4–7·3)	-0·3 (-10·9–59·5)
Tobacco	33·9 (32·3–35·4)	27·1 (26·0–28·3)	22·3 (21·4–23·2)	20·3 (18·8–21·9)	-1·7 (-1·9–-1·4)	-1·4 (-1·7–-1·0)	-0·8 (-1·5–-0·2)
Smoking	31·5 (29·4–33·7)	26·6 (24·9–28·3)	22·0 (20·8–23·3)	19·0 (17·4–20·8)	-1·6 (-1·9–-1·3)	-1·6 (-2·0–-1·2)	-1·3 (-2·1–-0·5)
Chewing tobacco	0·7 (0·5–0·9)	0·7 (0·6–0·9)	0·8 (0·6–1·0)	0·7 (0·6–1·0)	0·2 (-0·9–1·4)	-0·1 (-1·5–1·5)	-0·2 (-2·4–2·0)
Second-hand smoke	27·4 (25·7–28·8)	21·0 (19·5–22·2)	17·1 (15·9–18·2)	16·4 (14·8–18·2)	-1·7 (-2·0–-1·3)	-1·2 (-1·6–-0·7)	-0·4 (-1·1–0·4)
High alcohol use	29·0 (21·4–38·9)	30·8 (23·1–40·3)	33·2 (25·1–44·1)	32·8 (23·7–44·3)	0·4 (-0·5–1·2)	0·3 (-0·9–1·2)	-0·1 (-1·8–1·1)
Drug use	1·8 (1·1–3·1)	2·0 (1·5–2·8)	2·9 (2·4–3·4)	5·7 (4·6–6·9)	3·8 (1·5–5·8)	4·9 (2·5–6·6)	6·2 (3·9–7·7)
Dietary risks	35·7 (26·9–46·6)	40·6 (31·5–52·4)	44·5 (35·4–56·5)	44·4 (35·2–56·1)	0·7 (0·5–1·0)	0·4 (0·1–0·7)	0·0 (-0·4–0·4)
Diet low in fruits	29·4 (25·0–32·7)	28·1 (23·2–31·1)	31·2 (25·8–34·5)	32·1 (26·5–36·9)	0·3 (-0·2–0·8)	0·6 (0·0–1·2)	0·2 (-0·7–1·1)
Diet low in vegetables	23·6 (14·5–31·9)	31·2 (18·3–39·3)	41·6 (24·1–49·1)	40·6 (24·2–49·2)	1·8 (1·2–2·4)	1·2 (0·8–1·8)	-0·2 (-0·8–0·4)
Diet low in legumes	35·8 (0·0–44·9)	35·5 (0·0–45·1)	38·6 (0·0–48·8)	38·4 (0·0–48·7)	0·2 (-0·1–0·6)	0·4 (-0·1–0·8)	0·0 (-0·8–0·7)
Diet low in whole grains	44·0 (36·8–50·1)	47·5 (40·0–54·4)	48·9 (40·6–55·7)	48·2 (39·8–55·8)	0·3 (0·0–0·6)	0·1 (-0·3–0·4)	-0·1 (-0·7–0·4)
Diet low in nuts and seeds	9·1 (6·3–12·5)	4·5 (2·6–7·2)	2·7 (1·3–4·5)	2·7 (1·2–4·9)	-4·0 (-6·3–-2·0)	-2·5 (-6·0–0·8)	-0·1 (-5·1–4·6)
Diet low in milk	33·1 (28·3–37·9)	29·5 (25·0–34·0)	27·1 (22·8–31·7)	26·3 (21·9–31·3)	-0·7 (-1·3–-0·2)	-0·5 (-1·3–0·2)	-0·3 (-1·2–0·6)
Diet high in red meat	49·8 (0·0–68·5)	51·5 (0·0–71·2)	52·4 (0·0–71·9)	52·4 (0·0–72·4)	0·2 (-0·3–0·6)	0·1 (-0·6–0·6)	0·0 (-0·9–0·8)
Diet high in processed meat	48·8 (38·8–55·3)	61·2 (48·8–67·9)	67·7 (54·6–75·8)	65·5 (53·1–74·1)	1·0 (0·5–1·3)	0·3 (-0·2–0·8)	-0·3 (-1·1–0·4)
Diet high in sugar-sweetened beverages	40·5 (30·9–49·4)	56·8 (46·4–65·1)	63·3 (50·8–72·2)	63·3 (50·0–73·4)	1·4 (0·6–2·2)	0·5 (-0·3–1·3)	0·0 (-1·1–1·1)
Diet low in fibre	33·5 (19·8–40·9)	23·7 (13·5–29·7)	19·5 (10·8–25·7)	15·8 (9·3–21·3)	-2·4 (-3·6–-1·4)	-1·9 (-3·5–-0·5)	-1·9 (-4·1–-0·1)
Diet low in calcium	5·0 (4·1–6·1)	4·3 (3·5–5·3)	4·3 (3·5–5·3)	4·3 (3·4–5·2)	-0·5 (-0·7–-0·4)	-0·1 (-0·2–0·1)	-0·1 (-0·3–0·1)
Diet low in seafood omega-3 fatty acids	30·6 (22·7–39·8)	36·6 (27·6–46·4)	38·0 (28·6–48·3)	31·0 (22·1–40·7)	0·0 (-0·5–0·6)	-0·8 (-1·5–-0·1)	-1·9 (-2·9–-0·9)
Diet low in omega-6 polyunsaturated fatty acids	30·3 (20·0–40·1)	25·5 (17·5–33·4)	18·4 (12·8–23·7)	16·4 (11·4–21·8)	-2·0 (-2·9–-1·1)	-2·1 (-3·1–-1·2)	-1·0 (-2·0–-0·2)
Diet high in trans fatty acids	71·3 (62·5–78·8)	71·8 (64·8–79·0)	63·4 (55·1–70·6)	0·0 (0·0–0·0)	-65·8 (-66·1–-65·3)	-97·1 (-97·6–-96·6)	-184·2 (-185·2–-183·0)
Diet high in sodium	23·5 (0·6–68·8)	28·1 (1·9–76·2)	31·3 (3·3–76·9)	31·7 (3·2–77·7)	1·0 (0·2–6·2)	0·6 (-0·2–3·5)	0·1 (-2·3–1·9)
Intimate partner violence	27·0 (16·0–36·3)	27·0 (16·0–36·2)	22·6 (12·8–31·0)	21·7 (10·9–30·8)	-0·7 (-2·3–0·7)	-1·0 (-3·0–0·7)	-0·4 (-2·8–1·8)
Childhood sexual abuse and bullying	7·7 (4·8–12·6)	9·3 (5·8–14·8)	10·7 (6·7–17·1)	8·4 (5·3–13·1)	0·3 (-0·3–0·8)	-0·5 (-1·3–0·3)	-2·2 (-3·7–-0·7)
Childhood sexual abuse	5·9 (5·0–7·0)	6·7 (5·7–7·9)	7·8 (6·9–8·9)	7·2 (6·0–8·8)	0·7 (-0·1–1·4)	0·4 (-0·6–1·4)	-0·7 (-2·1–0·8)
Bullying victimization	7·7 (3·5–15·5)	9·5 (4·5–18·3)	10·9 (5·0–20·9)	7·8 (3·3–14·3)	0·0 (-0·7–0·7)	-0·9 (-1·9–0·0)	-3·0 (-4·9–-1·1)
Unsafe sex	--	--	--	--	--	--	--
Low physical activity	20·0 (14·9–25·6)	19·4 (14·9–23·9)	20·2 (15·8–24·9)	21·8 (16·5–28·1)	0·3 (-0·6–1·2)	0·6 (-0·6–1·8)	0·7 (-1·1–2·4)
**Metabolic risks**	**26·2 (23·0–29·4)**	**30·3 (26·9–33·5)**	**31·7 (27·9–34·6)**	**33·6 (29·4–36·5)**	**0·8 (0·5–1·1)**	**0·5 (0·1–0·8)**	**0·5 (0·0–1·0)**
High fasting plasma glucose	12·4 (9·5–15·0)	15·6 (12·0–18·8)	17·6 (13·1–21·2)	20·2 (15·4–24·2)	1·6 (0·9–2·2)	1·2 (0·3–2·1)	1·2 (0·0–2·5)
High LDL cholesterol	63·1 (44·5–86·3)	53·8 (37·2–74·1)	48·1 (32·6–66·4)	46·4 (30·9–64·4)	-1·0 (-1·2–-0·8)	-0·7 (-0·9–-0·5)	-0·3 (-0·6–-0·1)
High systolic blood pressure	30·2 (21·1–41·2)	26·1 (18·6–35·2)	18·6 (12·2–26·4)	26·3 (16·7–38·4)	-0·4 (-1·5–0·5)	0·0 (-1·3–1·2)	3·2 (0·9–5·6)
High body-mass index	30·1 (25·8–34·3)	35·0 (30·3–39·1)	36·7 (31·4–40·7)	38·3 (32·7–42·1)	0·8 (0·5–1·0)	0·4 (0·1–0·7)	0·4 (-0·1–0·8)
Low bone mineral density	21·3 (15·4–28·3)	19·1 (13·5–25·8)	20·3 (14·6–26·9)	22·4 (16·0–29·3)	0·2 (-0·4–0·6)	0·8 (0·0–1·6)	0·9 (-0·4–2·5)
Kidney dysfunction	3·2 (2·6–4·1)	3·2 (2·7–4·2)	3·1 (2·5–4·1)	3·2 (2·6–4·2)	0·0 (-0·1–0·1)	-0·1 (-0·2–0·1)	0·3 (0·0–0·6)

West Virginia

Risk Names	SEV 1990	SEV 2000	SEV 2010	SEV 2021	Annualised rate of change 1990 to 2021	Annualised rate of change 2000 to 2021	Annualised rate of change 2010 to 2021
**All risk factors**	**26·9 (24·0–30·0)**	**26·9 (24·1–29·7)**	**27·2 (24·4–30·2)**	**27·3 (24·4–30·6)**	**0·0 (-0·2–0·3)**	**0·1 (-0·3–0·4)**	**0·0 (-0·5–0·5)**
**Environmental/occupational risks**	**19·3 (12·8–29·5)**	**19·1 (13·2–29·5)**	**15·7 (10·6–25·3)**	**13·5 (8·7–22·3)**	**-1·1 (-2·0–-0·4)**	**-1·6 (-2·3–-0·8)**	**-1·4 (-2·0–-0·6)**
Unsafe water, sanitation, and handwashing	2·3 (1·2–3·8)	2·1 (1·0–3·4)	1·7 (0·6–2·8)	1·3 (0·5–2·3)	-1·8 (-4·1–0·0)	-2·2 (-5·0–0·4)	-2·2 (-6·1–1·6)
Unsafe water source	3·4 (1·2–6·6)	3·0 (1·2–6·3)	1·3 (0·5–2·7)	1·0 (0·4–2·1)	-3·9 (-6·6–-1·2)	-5·3 (-8·9–-1·1)	-2·3 (-7·9–3·6)
Unsafe sanitation	8·3 (4·6–12·9)	6·5 (3·4–10·9)	5·5 (2·9–9·4)	4·0 (2·0–7·0)	-2·4 (-4·6–-0·4)	-2·4 (-5·1–0·3)	-2·9 (-6·5–0·3)
No access to handwashing facility	1·8 (0·5–3·3)	1·8 (0·6–3·4)	1·9 (0·5–3·5)	1·6 (0·5–3·1)	-0·4 (-3·6–2·9)	-0·7 (-4·9–3·5)	-1·7 (-7·7–3·8)
Air pollution	21·7 (9·3–34·4)	19·6 (10·7–30·0)	9·3 (4·8–17·6)	5·2 (1·8–12·3)	-4·6 (-7·4–-2·6)	-6·3 (-9·7–-4·1)	-5·2 (-9·6–-3·1)
Particulate matter pollution	15·6 (6·3–26·3)	14·0 (10·4–18·6)	7·5 (4·3–11·1)	4·1 (1·5–7·2)	-4·3 (-7·6–-1·8)	-5·9 (-9·4–-4·2)	-5·5 (-10·5–-3·2)
Ambient particulate matter pollution	24·2 (10·1–41·9)	21·7 (16·4–27·6)	11·4 (6·7–16·5)	6·2 (2·3–10·6)	-4·4 (-7·7–-1·9)	-6·0 (-9·5–-4·3)	-5·5 (-10·5–-3·2)
Household air pollution from solid fuels	0·0 (0·0–0·3)	0·0 (0·0–0·2)	0·0 (0·0–0·1)	0·0 (0·0–0·0)	-4·6 (-27·1–-6·2)	-4·7 (-24·8–-6·0)	-4·3 (-19·6–-3·8)
Ambient ozone pollution	25·5 (19·2–33·3)	29·4 (23·0–37·2)	19·5 (13·7–26·5)	12·6 (7·4–19·3)	-2·3 (-3·1–-1·7)	-4·0 (-5·4–-3·1)	-3·9 (-5·9–-2·5)
Ambient nitrogen dioxide pollution	44·3 (0·0–100·0)	39·4 (0·0–95·8)	14·4 (0·0–54·1)	8·5 (0·0–43·5)	-5·3 (-19·8–0·0)	-7·3 (-27·9–0·0)	-4·8 (-26·9–0·0)
Non-optimal temperature	25·8 (20·0–35·4)	26·5 (21·7–34·8)	30·9 (24·3–42·0)	28·4 (22·3–38·8)	0·3 (0·0–0·7)	0·3 (-0·1–0·8)	-0·8 (-1·3–-0·4)
High temperature	17·7 (9·0–29·3)	15·2 (6·9–26·7)	25·1 (14·1–37·4)	22·3 (12·3–33·8)	0·8 (0·2–1·5)	1·8 (0·8–3·7)	-1·1 (-1·9–-0·5)
Low temperature	24·5 (20·7–28·5)	26·1 (22·7–29·6)	28·6 (25·9–32·1)	26·5 (23·3–30·2)	0·2 (0·1–0·5)	0·1 (-0·1–0·3)	-0·7 (-1·0–-0·4)
Other environmental risks	25·9 (9·4–45·8)	24·7 (9·5–44·8)	22·6 (9·4–42·7)	20·1 (9·3–40·8)	-0·8 (-1·4–0·0)	-1·0 (-1·8–0·0)	-1·1 (-2·0–0·0)
Residential radon	27·2 (0·0–84·2)	27·2 (0·0–84·2)	27·2 (0·0–84·2)	27·2 (0·0–84·2)	0·0 (0·0–0·0)	0·0 (0·0–0·0)	0·0 (0·0–0·0)
Lead exposure	25·3 (0·0–31·7)	23·6 (0·0–29·5)	20·4 (0·0–25·6)	16·9 (0·0–21·0)	-1·3 (-1·7–0·0)	-1·6 (-2·1–0·0)	-1·8 (-2·5–0·0)
Occupational risks	2·9 (2·4–3·7)	2·9 (2·4–3·7)	2·6 (2·1–3·4)	2·6 (2·1–3·5)	-0·4 (-0·7–0·0)	-0·5 (-0·9–-0·1)	0·1 (-0·5–0·6)
Occupational carcinogens	0·9 (0·4–1·7)	1·0 (0·4–1·8)	0·9 (0·4–1·7)	1·0 (0·4–1·8)	0·2 (-0·1–0·5)	0·0 (-0·4–0·4)	0·3 (-0·3–0·9)
Occupational exposure to asbestos	5·0 (4·4–5·6)	5·3 (4·8–5·9)	5·7 (5·2–6·1)	5·4 (4·4–6·6)	0·2 (-0·5–1·0)	0·1 (-1·1–1·2)	-0·5 (-2·5–1·4)
Occupational exposure to arsenic	0·4 (0·0–1·1)	0·4 (0·0–1·1)	0·4 (0·0–1·0)	0·4 (0·0–1·0)	-0·2 (-0·7–0·2)	-0·3 (-0·9–0·4)	0·3 (-0·6–1·2)
Occupational exposure to benzene	1·0 (0·1–2·8)	1·1 (0·1–3·1)	1·0 (0·1–3·0)	1·1 (0·1–3·2)	0·4 (0·0–0·7)	0·1 (-0·4–0·6)	0·6 (-0·2–1·2)
Occupational exposure to beryllium	0·0 (0·0–0·0)	0·0 (0·0–0·0)	0·0 (0·0–0·0)	0·0 (0·0–0·0)	0·1 (-0·2–0·4)	0·0 (-0·3–0·4)	0·6 (0·1–1·1)
Occupational exposure to cadmium	0·1 (0·0–0·1)	0·1 (0·0–0·1)	0·0 (0·0–0·1)	0·1 (0·0–0·1)	-0·1 (-0·6–0·3)	-0·2 (-0·8–0·4)	0·4 (-0·4–1·2)
Occupational exposure to chromium	0·1 (0·1–0·1)	0·1 (0·1–0·1)	0·1 (0·1–0·1)	0·1 (0·1–0·1)	-0·1 (-0·4–0·3)	-0·2 (-0·7–0·4)	0·4 (-0·3–1·1)
Occupational exposure to diesel engine exhaust	0·7 (0·6–0·8)	0·7 (0·6–0·7)	0·6 (0·6–0·7)	0·7 (0·6–0·7)	-0·2 (-0·5–0·2)	-0·1 (-0·6–0·4)	0·7 (0·0–1·5)
Occupational exposure to formaldehyde	0·3 (0·2–0·3)	0·3 (0·2–0·3)	0·2 (0·2–0·3)	0·2 (0·2–0·3)	-0·2 (-0·6–0·2)	-0·3 (-0·9–0·2)	0·3 (-0·6–1·0)
Occupational exposure to nickel	0·4 (0·0–1·7)	0·4 (0·0–1·5)	0·3 (0·0–1·3)	0·3 (0·0–1·4)	-0·7 (-1·2–0·0)	-0·6 (-1·3–0·1)	0·1 (-0·9–1·1)
Occupational exposure to polycyclic aromatic hydrocarbons	0·3 (0·2–0·3)	0·3 (0·3–0·3)	0·3 (0·2–0·3)	0·3 (0·2–0·3)	0·0 (-0·4–0·3)	-0·2 (-0·6–0·3)	0·4 (-0·3–1·0)
Occupational exposure to silica	4·3 (0·7–13·8)	3·8 (0·6–12·3)	3·2 (0·5–10·3)	3·2 (0·5–10·5)	-0·9 (-1·4–-0·4)	-0·8 (-1·5–-0·1)	0·0 (-0·9–1·1)
Occupational exposure to sulphuric acid	0·7 (0·1–2·1)	0·7 (0·1–2·2)	0·6 (0·1–2·0)	0·6 (0·1–2·1)	-0·1 (-0·5–0·3)	-0·2 (-0·8–0·3)	0·4 (-0·4–1·2)
Occupational exposure to trichloroethylene	0·1 (0·1–0·1)	0·1 (0·1–0·1)	0·1 (0·1–0·1)	0·1 (0·1–0·1)	0·1 (-0·3–0·4)	0·0 (-0·4–0·4)	0·6 (0·0–1·2)
Occupational asthmagens	17·9 (15·7–20·2)	17·2 (15·2–19·6)	14·9 (13·1–16·8)	15·2 (13·3–17·5)	-0·5 (-0·9–-0·1)	-0·6 (-1·1–0·0)	0·2 (-0·6–0·9)
Occupational particulate matter, gases, and fumes	6·6 (5·0–8·8)	6·4 (4·8–8·4)	5·9 (4·5–7·7)	5·4 (4·1–6·9)	-0·7 (-1·0–-0·4)	-0·8 (-1·1–-0·5)	-0·9 (-1·3–-0·5)
Occupational noise	7·2 (6·6–7·9)	7·1 (6·6–7·7)	6·8 (6·3–7·3)	6·4 (6·0–6·9)	-0·4 (-0·6–-0·2)	-0·5 (-0·7–-0·3)	-0·6 (-0·8–-0·3)
Occupational injuries	--	--	--	--	--	--	--
Occupational ergonomic factors	8·3 (6·8–10·2)	8·1 (6·8–9·8)	7·1 (6·0–8·5)	7·4 (6·1–9·1)	-0·4 (-0·9–0·1)	-0·5 (-1·2–0·2)	0·4 (-0·5–1·1)
**Behavioural risks**	**30·5 (27·3–33·8)**	**29·7 (27·0–33·1)**	**29·1 (26·3–32·5)**	**26·6 (23·7–30·1)**	**-0·4 (-0·7–-0·2)**	**-0·5 (-0·9–-0·2)**	**-0·8 (-1·3–-0·3)**
Child and maternal malnutrition	10·5 (7·2–15·1)	9·2 (6·3–12·8)	9·4 (6·6–13·1)	9·6 (6·6–13·8)	-0·3 (-1·0–0·5)	0·2 (-0·8–1·3)	0·2 (-1·4–1·7)
Suboptimal breastfeeding	69·1 (66·4–71·7)	66·9 (64·4–69·5)	63·1 (60·4–65·9)	62·4 (59·6–65·4)	-0·3 (-0·5–-0·2)	-0·3 (-0·5–-0·1)	-0·1 (-0·4–0·2)
Non-exclusive breastfeeding	64·9 (57·1–72·0)	60·2 (53·1–67·2)	49·2 (42·4–55·5)	46·7 (40·1–54·0)	-1·1 (-1·6–-0·5)	-1·2 (-1·8–-0·5)	-0·5 (-1·4–0·5)
Discontinued breastfeeding	88·9 (86·3–91·4)	86·5 (83·7–89·4)	84·5 (81·3–87·8)	84·7 (81·6–88·0)	-0·2 (-0·3–0·0)	-0·1 (-0·3–0·1)	0·0 (-0·3–0·3)
Child growth failure	1·4 (0·5–3·3)	1·3 (0·4–3·0)	1·2 (0·4–2·8)	1·1 (0·4–2·5)	-0·8 (-1·2–-0·4)	-0·8 (-1·4–-0·4)	-0·8 (-1·5–-0·1)
Child underweight	3·0 (1·9–4·2)	2·8 (1·8–3·9)	2·5 (1·6–3·5)	2·3 (1·4–3·2)	-0·8 (-1·4–-0·3)	-0·9 (-1·6–-0·2)	-0·7 (-1·7–0·3)
Child wasting	0·9 (0·6–1·5)	0·9 (0·6–1·4)	0·9 (0·6–1·4)	0·9 (0·6–1·4)	-0·2 (-0·5–0·1)	0·0 (-0·5–0·4)	-0·4 (-1·0–0·2)
Child stunting	4·1 (3·1–5·1)	3·8 (2·9–4·8)	3·4 (2·7–4·4)	3·1 (2·3–3·9)	-0·9 (-1·4–-0·3)	-1·0 (-1·8–-0·2)	-1·0 (-2·2–0·3)
Low birth weight and short gestation	20·8 (18·4–23·4)	22·4 (19·5–25·2)	24·1 (21·4–27·0)	24·2 (21·4–27·2)	0·5 (0·2–0·7)	0·4 (0·0–0·8)	0·0 (-0·6–0·8)
Short gestation	35·1 (31·3–39·1)	38·5 (34·1–43·2)	38·2 (34·2–42·8)	38·5 (34·3–43·2)	0·3 (0·0–0·6)	0·0 (-0·4–0·5)	0·1 (-0·7–0·9)
Low birth weight	16·5 (15·0–17·9)	17·2 (15·8–18·7)	19·3 (17·7–20·8)	19·3 (17·7–21·2)	0·5 (0·3–0·7)	0·6 (0·2–0·9)	0·0 (-0·6–0·7)

Iron deficiency	3·8 (2·6–5·4)	3·3 (2·4–4·5)	3·4 (2·5–4·6)	3·5 (2·5–4·8)	-0·3 (-1·0–0·5)	0·2 (-0·8–1·2)	0·1 (-1·4–1·5)
Vitamin A deficiency	1·4 (0·0–2·8)	1·9 (0·0–4·0)	1·4 (0·0–3·2)	0·8 (0·0–1·8)	-1·6 (-3·8–0·6)	-3·9 (-6·7–0·0)	-4·5 (-8·8–0·2)
Zinc deficiency	2·3 (0·0–9·5)	1·3 (0·0–5·5)	1·2 (0·0–5·1)	1·2 (0·0–4·9)	-2·1 (-48·0–0·0)	-0·6 (-3·3–3·9)	-0·1 (-3·6–7·4)
Tobacco	44·9 (43·3–46·5)	44·6 (42·9–46·0)	43·1 (41·6–44·3)	37·0 (34·9–39·2)	-0·6 (-0·8–-0·4)	-0·9 (-1·2–-0·6)	-1·4 (-1·9–-0·9)
Smoking	40·9 (38·9–42·8)	38·8 (36·6–40·5)	36·8 (35·0–38·5)	31·8 (29·3–34·5)	-0·8 (-1·1–-0·5)	-0·9 (-1·3–-0·5)	-1·3 (-2·0–-0·6)
Chewing tobacco	6·0 (5·1–7·0)	5·2 (4·4–6·2)	4·7 (3·6–6·1)	4·4 (3·3–5·8)	-1·0 (-2·1–0·0)	-0·8 (-2·2–0·6)	-0·6 (-2·7–1·6)
Second-hand smoke	39·1 (37·0–40·8)	40·2 (37·9–41·6)	39·3 (36·7–40·6)	33·4 (30·8–35·5)	-0·5 (-0·7–-0·3)	-0·9 (-1·2–-0·6)	-1·5 (-2·0–-0·9)
High alcohol use	23·0 (16·8–31·7)	21·2 (15·8–29·9)	21·0 (15·6–29·9)	21·4 (15·0–30·7)	-0·2 (-1·3–0·7)	0·0 (-1·4–1·2)	0·1 (-1·9–1·8)
Drug use	1·1 (0·8–1·7)	1·7 (1·4–2·0)	3·8 (2·7–4·8)	9·9 (6·5–12·7)	7·1 (4·6–8·8)	8·5 (6·6–9·5)	8·7 (7·6–9·8)
Dietary risks	38·1 (28·5–49·2)	41·0 (31·6–52·4)	43·3 (33·6–55·4)	43·5 (34·0–55·5)	0·4 (0·2–0·7)	0·3 (0·0–0·6)	0·0 (-0·4–0·5)
Diet low in fruits	37·6 (33·7–40·8)	33·7 (29·8–36·5)	33·4 (28·6–36·2)	35·6 (30·1–39·8)	-0·2 (-0·6–0·2)	0·3 (-0·3–0·8)	0·6 (-0·3–1·4)
Diet low in vegetables	27·3 (16·3–35·1)	28·8 (17·1–36·7)	32·6 (18·5–40·4)	32·5 (19·0–41·0)	0·6 (0·2–0·9)	0·6 (0·1–1·1)	0·0 (-0·8–0·8)
Diet low in legumes	40·0 (0·0–50·5)	40·2 (0·0–50·5)	44·3 (0·0–55·5)	45·1 (0·0–58·0)	0·4 (0·0–0·7)	0·5 (0·0–1·1)	0·2 (-0·6–0·9)
Diet low in whole grains	46·2 (38·5–52·5)	49·3 (40·7–56·2)	51·0 (42·2–58·4)	51·9 (42·7–59·8)	0·4 (0·1–0·7)	0·2 (-0·2–0·6)	0·1 (-0·4–0·7)
Diet low in nuts and seeds	15·8 (12·2–20·3)	9·8 (6·8–13·6)	7·1 (4·9–10·1)	7·5 (4·6–11·1)	-2·4 (-4·0–-1·0)	-1·3 (-3·6–0·9)	0·5 (-3·2–3·5)
Diet low in milk	39·5 (35·0–43·9)	36·7 (32·4–41·4)	34·8 (30·3–39·4)	34·4 (30·0–39·9)	-0·4 (-0·9–0·0)	-0·3 (-0·9–0·3)	-0·1 (-0·9–0·7)
Diet high in red meat	50·1 (0·0–67·9)	50·7 (0·0–69·4)	50·9 (0·0–69·5)	51·1 (0·0–70·0)	0·1 (-0·5–0·5)	0·0 (-0·6–0·6)	0·0 (-1·1–1·1)
Diet high in processed meat	51·4 (40·4–57·2)	62·8 (50·1–70·2)	69·1 (55·3–77·0)	67·0 (55·4–76·1)	0·9 (0·4–1·3)	0·3 (-0·2–0·8)	-0·3 (-1·0–0·5)
Diet high in sugar-sweetened beverages	33·2 (24·5–41·4)	47·0 (37·5–56·0)	52·4 (41·7–61·6)	52·3 (41·1–62·4)	1·5 (0·6–2·4)	0·5 (-0·6–1·4)	0·0 (-1·6–1·3)
Diet low in fibre	43·5 (23·1–50·9)	33·8 (17·9–40·6)	30·0 (15·9–37·3)	26·5 (14·7–33·8)	-1·6 (-2·5–-0·8)	-1·2 (-2·4–-0·1)	-1·1 (-3·1–0·4)
Diet low in calcium	6·5 (5·4–8·1)	5·4 (4·3–6·7)	5·6 (4·5–7·0)	5·5 (4·4–6·8)	-0·5 (-0·8–-0·3)	0·1 (-0·2–0·4)	-0·2 (-0·6–0·2)
Diet low in seafood omega-3 fatty acids	54·4 (44·3–65·8)	60·5 (49·7–72·7)	62·9 (52·0–75·2)	58·6 (47·3–70·3)	0·2 (-0·1–0·5)	-0·2 (-0·5–0·2)	-0·6 (-1·1–-0·2)
Diet low in omega-6 polyunsaturated fatty acids	40·9 (25·9–51·9)	35·8 (23·5–45·6)	26·8 (18·4–34·4)	24·9 (17·1–32·7)	-1·6 (-2·3–-0·8)	-1·7 (-2·6–-0·9)	-0·6 (-1·7–0·4)
Diet high in trans fatty acids	71·2 (62·7–78·5)	71·8 (64·4–79·2)	63·2 (54·0–70·8)	0·0 (0·0–0·0)	-65·8 (-66·1–-65·3)	-97·1 (-97·6–-96·6)	-184·2 (-185·3–-182·8)
Diet high in sodium	23·4 (0·6–68·5)	28·3 (1·9–75·2)	31·5 (3·5–75·3)	31·9 (3·2–78·6)	1·0 (0·2–5·8)	0·6 (-0·1–3·7)	0·1 (-2·6–1·9)
Intimate partner violence	28·1 (11·7–36·4)	28·1 (13·1–35·9)	25·3 (17·0–31·2)	23·8 (13·5–32·3)	-0·5 (-2·0–0·9)	-0·8 (-2·8–1·0)	-0·5 (-3·1–1·7)
Childhood sexual abuse and bullying	9·3 (6·1–14·6)	11·1 (7·4–17·6)	12·7 (8·5–19·9)	9·5 (6·7–13·9)	0·1 (-0·6–0·6)	-0·8 (-1·7–0·0)	-2·6 (-4·2–-1·1)
Childhood sexual abuse	9·3 (7·8–10·9)	10·4 (9·0–11·9)	11·9 (10·4–13·4)	10·6 (8·8–13·0)	0·5 (-0·3–1·2)	0·1 (-0·8–1·0)	-1·0 (-2·3–0·3)
Bullying victimization	8·0 (3·5–16·0)	9·9 (4·5–19·2)	11·2 (5·1–21·9)	7·2 (3·2–13·2)	-0·3 (-1·0–0·3)	-1·5 (-2·5–-0·6)	-4·0 (-5·9–-2·3)
Unsafe sex	--	--	--	--	--	--	--
Low physical activity	20·9 (15·8–27·0)	20·1 (15·2–25·1)	21·5 (16·9–26·3)	23·7 (18·1–30·6)	0·4 (-0·5–1·4)	0·8 (-0·4–2·0)	0·9 (-0·9–2·5)
**Metabolic risks**	**27·5 (24·2–30·7)**	**33·3 (29·4–36·1)**	**37·9 (33·8–40·8)**	**41·5 (36·8–44·8)**	**1·3 (1·0–1·6)**	**1·0 (0·7–1·4)**	**0·8 (0·4–1·3)**
High fasting plasma glucose	14·3 (11·0–17·1)	19·6 (14·8–23·3)	24·6 (18·4–29·6)	27·8 (20·9–33·7)	2·1 (1·5–2·9)	1·7 (0·7–2·5)	1·1 (-0·1–2·3)
High LDL cholesterol	63·6 (44·7–86·7)	54·1 (37·3–74·4)	48·5 (32·9–67·4)	47·0 (31·6–65·7)	-1·0 (-1·2–-0·8)	-0·7 (-0·9–-0·5)	-0·3 (-0·5–0·0)
High systolic blood pressure	30·7 (20·3–41·9)	26·7 (18·8–37·0)	19·9 (13·1–28·4)	27·5 (17·2–39·5)	-0·4 (-1·3–0·5)	0·1 (-1·0–1·4)	3·0 (0·5–5·5)
High body-mass index	31·1 (26·4–35·3)	38·0 (32·5–41·8)	43·3 (37·5–47·1)	47·2 (40·3–51·4)	1·3 (1·0–1·6)	1·0 (0·7–1·3)	0·8 (0·4–1·2)
Low bone mineral density	21·8 (16·1–29·0)	19·8 (13·9–26·7)	20·6 (14·6–27·4)	22·8 (16·6–29·7)	0·1 (-0·3–0·6)	0·7 (-0·1–1·4)	0·9 (-0·2–2·3)
Kidney dysfunction	3·0 (2·4–3·9)	3·1 (2·5–4·0)	3·1 (2·5–4·1)	3·2 (2·6–4·2)	0·2 (0·1–0·4)	0·2 (0·0–0·4)	0·4 (0·1–0·7)

Wisconsin

Risk Names	SEV 1990	SEV 2000	SEV 2010	SEV 2021	Annualised rate of change 1990 to 2021	Annualised rate of change 2000 to 2021	Annualised rate of change 2010 to 2021
**All risk factors**	**27·2 (24·3–30·4)**	**26·6 (23·8–29·9)**	**27·5 (24·4–30·7)**	**27·4 (24·0–30·8)**	**0·0 (-0·3–0·3)**	**0·1 (-0·3–0·6)**	**0·0 (-0·7–0·6)**
**Environmental/occupational risks**	**18·4 (12·0–29·1)**	**17·5 (11·9–28·1)**	**15·3 (10·1–25·5)**	**14·0 (9·0–23·9)**	**-0·9 (-1·6–-0·3)**	**-1·1 (-1·5–-0·6)**	**-0·8 (-1·3–-0·4)**
Unsafe water, sanitation, and handwashing	1·8 (0·9–2·8)	1·6 (0·7–2·7)	1·3 (0·4–2·3)	1·1 (0·3–2·0)	-1·5 (-3·9–0·4)	-1·8 (-4·9–0·5)	-1·7 (-5·6–2·6)
Unsafe water source	2·5 (0·9–5·2)	2·3 (0·8–4·7)	1·0 (0·4–1·9)	0·8 (0·3–1·6)	-3·7 (-6·3–-1·0)	-5·1 (-8·5–-1·2)	-1·9 (-7·0–3·5)
Unsafe sanitation	5·3 (2·9–8·7)	4·2 (2·2–7·1)	3·3 (1·7–5·8)	2·5 (1·2–4·4)	-2·3 (-4·7–-0·2)	-2·4 (-5·3–0·4)	-2·4 (-6·1–1·1)
No access to handwashing facility	1·6 (0·6–3·1)	1·7 (0·6–3·3)	1·7 (0·6–3·4)	1·5 (0·5–2·8)	-0·3 (-3·2–2·4)	-0·5 (-4·6–3·5)	-1·3 (-6·9–4·0)
Air pollution	22·1 (6·5–34·0)	19·5 (7·7–28·6)	11·4 (4·2–21·3)	8·1 (2·6–16·9)	-3·2 (-5·7–-1·2)	-4·2 (-7·0–-2·4)	-3·1 (-6·0–-1·6)
Particulate matter pollution	11·8 (3·9–21·3)	10·4 (7·1–14·3)	6·6 (3·7–10·0)	4·9 (2·2–8·1)	-2·8 (-5·5–0·1)	-3·6 (-6·0–-2·4)	-2·6 (-5·6–-1·1)
Ambient particulate matter pollution	18·3 (6·1–33·3)	16·0 (11·3–21·2)	10·1 (5·8–14·9)	7·5 (3·4–12·1)	-2·9 (-5·6–0·1)	-3·6 (-6·0–-2·5)	-2·7 (-5·6–-1·0)
Household air pollution from solid fuels	0·0 (0·0–0·1)	0·0 (0·0–0·0)	0·0 (0·0–0·0)	0·0 (0·0–0·0)	-3·4 (-32·0–-6·5)	-3·5 (-27·2–-5·7)	-3·2 (-21·2–-3·9)
Ambient ozone pollution	16·7 (11·0–23·8)	16·8 (11·0–24·5)	13·7 (8·1–20·2)	11·3 (6·5–17·7)	-1·3 (-2·1–-0·8)	-1·9 (-3·0–-1·2)	-1·7 (-3·5–-0·4)
Ambient nitrogen dioxide pollution	65·9 (0·0–100·0)	58·4 (0·0–100·0)	31·1 (0·0–84·8)	20·7 (0·0–67·5)	-3·7 (-10·9–0·0)	-4·9 (-14·9–0·0)	-3·7 (-14·3–0·0)
Non-optimal temperature	30·1 (24·2–39·2)	27·5 (22·8–35·8)	30·5 (24·1–40·1)	29·6 (23·5–38·9)	-0·1 (-0·2–0·2)	0·3 (0·0–0·9)	-0·3 (-0·7–-0·1)
High temperature	24·1 (13·5–36·5)	18·6 (9·5–30·4)	27·9 (16·4–40·5)	28·1 (16·6–41·0)	0·5 (0·0–1·0)	2·0 (0·8–3·3)	0·0 (-0·9–1·1)
Low temperature	28·2 (25·3–32·0)	26·6 (23·9–30·3)	27·8 (24·9–31·5)	27·3 (24·6–30·9)	-0·1 (-0·2–0·0)	0·1 (0·0–0·3)	-0·2 (-0·5–0·2)
Other environmental risks	26·7 (10·9–46·6)	25·5 (10·9–45·4)	23·2 (10·5–43·4)	20·9 (9·6–41·0)	-0·8 (-1·5–0·0)	-0·9 (-1·8–0·0)	-1·0 (-2·0–0·0)
Residential radon	32·1 (0·0–97·3)	32·1 (0·0–97·3)	32·1 (0·0–97·3)	32·1 (0·0–97·3)	0·0 (0·0–0·0)	0·0 (0·0–0·0)	0·0 (0·0–0·0)
Lead exposure	24·3 (0·0–30·9)	22·5 (0·0–28·0)	19·2 (0·0–24·0)	15·8 (0·0–19·8)	-1·4 (-1·8–0·0)	-1·7 (-2·2–0·0)	-1·7 (-2·5–0·0)
Occupational risks	3·3 (2·8–4·3)	3·4 (2·8–4·4)	3·2 (2·6–4·2)	3·2 (2·6–4·2)	-0·1 (-0·4–0·1)	-0·4 (-0·7–0·0)	-0·1 (-0·6–0·3)
Occupational carcinogens	1·1 (0·5–2·0)	1·2 (0·6–2·2)	1·1 (0·5–2·1)	1·1 (0·5–2·1)	0·1 (-0·2–0·4)	-0·1 (-0·6–0·2)	0·0 (-0·6–0·5)
Occupational exposure to asbestos	5·0 (4·5–5·5)	5·6 (5·1–6·1)	5·1 (4·6–5·6)	4·8 (3·9–5·8)	-0·1 (-0·8–0·5)	-0·7 (-1·8–0·2)	-0·6 (-2·4–1·2)
Occupational exposure to arsenic	0·6 (0·0–1·5)	0·6 (0·0–1·6)	0·6 (0·0–1·4)	0·6 (0·0–1·4)	-0·4 (-0·8–0·1)	-0·6 (-1·2–0·0)	-0·2 (-1·1–0·7)
Occupational exposure to benzene	1·1 (0·1–3·2)	1·2 (0·1–3·6)	1·2 (0·1–3·7)	1·3 (0·1–3·7)	0·4 (0·0–0·7)	0·2 (-0·3–0·6)	0·3 (-0·3–0·8)
Occupational exposure to beryllium	0·0 (0·0–0·0)	0·0 (0·0–0·0)	0·0 (0·0–0·0)	0·0 (0·0–0·0)	0·2 (0·0–0·4)	0·0 (-0·3–0·3)	0·3 (-0·1–0·7)
Occupational exposure to cadmium	0·1 (0·1–0·1)	0·1 (0·1–0·1)	0·1 (0·1–0·1)	0·1 (0·1–0·1)	-0·3 (-0·7–0·1)	-0·5 (-1·1–0·0)	-0·1 (-1·0–0·6)
Occupational exposure to chromium	0·2 (0·2–0·2)	0·2 (0·2–0·2)	0·2 (0·2–0·2)	0·2 (0·2–0·2)	-0·2 (-0·5–0·2)	-0·4 (-0·9–0·1)	0·0 (-0·7–0·6)
Occupational exposure to diesel engine exhaust	0·7 (0·6–0·7)	0·7 (0·7–0·8)	0·7 (0·7–0·8)	0·8 (0·7–0·8)	0·4 (0·1–0·8)	0·4 (-0·1–0·8)	0·6 (0·0–1·4)
Occupational exposure to formaldehyde	0·4 (0·3–0·4)	0·4 (0·3–0·4)	0·3 (0·3–0·4)	0·3 (0·3–0·4)	-0·3 (-0·7–0·1)	-0·6 (-1·1–0·0)	-0·3 (-1·0–0·5)
Occupational exposure to nickel	0·5 (0·0–2·0)	0·5 (0·0–2·0)	0·5 (0·0–1·7)	0·4 (0·0–1·7)	-0·4 (-0·9–0·1)	-0·7 (-1·3–0·1)	-0·3 (-1·3–0·7)
Occupational exposure to polycyclic aromatic hydrocarbons	0·3 (0·3–0·4)	0·4 (0·3–0·4)	0·3 (0·3–0·4)	0·3 (0·3–0·4)	-0·1 (-0·4–0·2)	-0·3 (-0·8–0·1)	0·0 (-0·6–0·6)
Occupational exposure to silica	4·3 (0·7–14·0)	4·2 (0·7–13·8)	3·8 (0·6–12·0)	3·8 (0·6–11·8)	-0·4 (-0·9–0·0)	-0·6 (-1·2–0·1)	-0·1 (-1·1–0·8)
Occupational exposure to sulphuric acid	0·9 (0·2–3·0)	0·9 (0·2–3·2)	0·9 (0·2–2·9)	0·9 (0·2–2·8)	-0·2 (-0·7–0·1)	-0·5 (-1·1–0·0)	-0·1 (-0·9–0·6)
Occupational exposure to trichloroethylene	0·1 (0·1–0·1)	0·1 (0·1–0·1)	0·1 (0·1–0·1)	0·1 (0·1–0·1)	0·0 (-0·2–0·3)	-0·1 (-0·5–0·2)	0·2 (-0·4–0·7)
Occupational asthmagens	19·5 (17·2–22·1)	20·1 (17·7–22·9)	18·6 (16·2–21·1)	18·5 (16·3–21·0)	-0·2 (-0·5–0·2)	-0·4 (-0·9–0·0)	0·0 (-0·7–0·6)
Occupational particulate matter, gases, and fumes	8·0 (6·2–10·6)	7·9 (6·1–10·4)	7·5 (5·8–9·9)	6·9 (5·3–9·0)	-0·5 (-0·7–-0·2)	-0·6 (-0·9–-0·4)	-0·7 (-1·1–-0·4)
Occupational noise	7·6 (7·0–8·3)	7·7 (7·2–8·4)	7·6 (7·1–8·3)	7·4 (7·0–7·9)	-0·1 (-0·3–0·0)	-0·2 (-0·4–0·0)	-0·3 (-0·5–-0·1)
Occupational injuries	--	--	--	--	--	--	--
Occupational ergonomic factors	8·7 (7·2–10·6)	8·8 (7·4–10·4)	8·0 (6·7–9·6)	8·2 (6·7–9·8)	-0·2 (-0·7–0·2)	-0·4 (-0·9–0·1)	0·1 (-0·6–0·8)
**Behavioural risks**	**31·6 (28·1–35·2)**	**29·8 (26·4–33·4)**	**27·7 (24·3–31·8)**	**25·5 (21·9–29·5)**	**-0·7 (-1·1–-0·3)**	**-0·7 (-1·3–-0·2)**	**-0·8 (-1·6–0·0)**
Child and maternal malnutrition	9·8 (6·6–13·6)	8·6 (5·9–11·7)	8·7 (6·1–12·0)	8·9 (6·2–12·3)	-0·3 (-1·1–0·5)	0·2 (-1·0–1·3)	0·2 (-1·7–1·9)
Suboptimal breastfeeding	68·8 (66·2–71·3)	66·6 (64·0–69·2)	62·7 (59·8–65·6)	62·1 (59·4–65·4)	-0·3 (-0·5–-0·2)	-0·3 (-0·5–-0·2)	-0·1 (-0·4–0·2)
Non-exclusive breastfeeding	64·1 (55·9–71·6)	59·3 (51·8–66·2)	48·4 (41·0–55·3)	46·1 (39·2–53·5)	-1·1 (-1·6–-0·5)	-1·2 (-1·9–-0·5)	-0·4 (-1·5–0·7)
Discontinued breastfeeding	88·7 (86·3–91·0)	86·3 (83·5–89·0)	84·3 (80·9–87·6)	84·5 (81·0–88·1)	-0·2 (-0·3–0·0)	-0·1 (-0·3–0·1)	0·0 (-0·3–0·3)
Child growth failure	1·2 (0·4–2·6)	1·1 (0·4–2·6)	1·0 (0·3–2·2)	0·9 (0·3–2·1)	-0·7 (-1·1–-0·4)	-0·9 (-1·4–-0·4)	-0·5 (-1·3–0·3)
Child underweight	2·5 (1·6–3·4)	2·4 (1·5–3·3)	2·0 (1·3–2·9)	1·9 (1·2–2·7)	-0·8 (-1·3–-0·2)	-0·9 (-1·7–-0·2)	-0·5 (-1·6–0·6)
Child wasting	0·8 (0·5–1·3)	0·8 (0·5–1·2)	0·8 (0·5–1·2)	0·8 (0·5–1·2)	-0·1 (-0·5–0·2)	0·0 (-0·4–0·5)	-0·2 (-0·8–0·5)
Child stunting	3·4 (2·7–4·3)	3·3 (2·6–4·1)	2·9 (2·2–3·6)	2·6 (2·1–3·3)	-0·8 (-1·4–-0·3)	-1·0 (-1·9–-0·2)	-0·7 (-2·0–0·4)
Low birth weight and short gestation	18·2 (16·0–20·6)	19·6 (17·3–22·1)	21·4 (18·8–24·1)	21·9 (19·3–24·7)	0·6 (0·3–0·9)	0·5 (0·1–0·9)	0·2 (-0·6–1·0)
Short gestation	35·0 (31·5–39·3)	37·1 (33·5–41·5)	39·0 (34·8–43·6)	38·9 (34·9–44·1)	0·3 (0·1–0·6)	0·2 (-0·2–0·7)	0·0 (-0·8–0·9)
Low birth weight	14·4 (13·1–15·8)	15·2 (13·9–16·5)	16·6 (15·2–18·2)	17·2 (15·6–18·9)	0·6 (0·3–0·8)	0·6 (0·2–1·0)	0·3 (-0·4–1·1)

Iron deficiency	3·4 (2·4–4·7)	3·0 (2·2–4·0)	3·1 (2·3–4·2)	3·2 (2·2–4·3)	-0·3 (-1·0–0·5)	0·2 (-1·0–1·3)	0·2 (-1·5–1·8)
Vitamin A deficiency	0·9 (0·0–1·8)	1·1 (0·0–2·3)	0·7 (0·0–1·6)	0·4 (0·0–0·9)	-2·1 (-4·5–0·0)	-4·5 (-7·3–0·0)	-4·6 (-8·4–0·0)
Zinc deficiency	1·9 (0·0–8·3)	1·1 (0·0–4·7)	1·0 (0·0–4·4)	1·0 (0·0–4·3)	-2·1 (-45·4–0·0)	-0·5 (-2·7–2·9)	-0·1 (-6·0–2·2)
Tobacco	41·3 (39·4–43·1)	36·1 (34·7–37·5)	29·6 (28·6–30·7)	24·5 (23·0–26·2)	-1·7 (-1·9–-1·5)	-1·8 (-2·2–-1·5)	-1·7 (-2·3–-1·2)
Smoking	39·0 (36·5–41·2)	33·3 (31·2–35·4)	27·6 (26·1–29·2)	22·8 (20·8–24·9)	-1·7 (-2·0–-1·5)	-1·8 (-2·2–-1·4)	-1·8 (-2·5–-1·0)
Chewing tobacco	1·6 (1·2–2·0)	1·8 (1·4–2·3)	2·0 (1·5–2·6)	2·0 (1·4–2·8)	0·8 (-0·6–2·1)	0·5 (-1·5–2·3)	0·2 (-2·5–2·7)
Second-hand smoke	33·0 (30·8–34·8)	29·0 (27·4–30·2)	23·5 (21·9–24·6)	19·8 (18·2–21·3)	-1·7 (-1·9–-1·4)	-1·8 (-2·2–-1·5)	-1·6 (-2·3–-0·9)
High alcohol use	30·4 (22·1–40·9)	29·0 (20·7–39·1)	29·7 (21·0–40·3)	29·7 (20·4–40·1)	-0·1 (-1·1–1·0)	0·1 (-1·3–1·5)	0·0 (-2·0–2·0)
Drug use	1·0 (0·6–1·7)	1·2 (0·9–1·6)	2·3 (1·8–2·8)	5·2 (3·5–6·7)	5·4 (2·5–7·8)	6·9 (4·1–8·6)	7·5 (5·2–8·9)
Dietary risks	36·8 (27·9–47·3)	39·9 (31·1–51·4)	42·3 (32·8–54·5)	42·3 (33·1–54·6)	0·5 (0·2–0·7)	0·3 (-0·1–0·6)	0·0 (-0·5–0·5)
Diet low in fruits	36·5 (31·3–40·3)	33·7 (29·0–36·9)	33·9 (30·4–36·8)	35·1 (30·4–39·2)	-0·1 (-0·5–0·3)	0·2 (-0·4–0·7)	0·3 (-0·5–1·1)
Diet low in vegetables	24·8 (14·3–32·5)	26·5 (15·2–34·3)	30·7 (17·0–37·8)	30·1 (17·3–38·0)	0·6 (0·2–1·1)	0·6 (0·1–1·1)	-0·2 (-1·1–0·6)
Diet low in legumes	38·2 (0·0–48·1)	38·2 (0·0–48·2)	42·5 (0·0–53·1)	42·9 (0·0–54·2)	0·4 (0·0–0·7)	0·6 (0·0–1·0)	0·1 (-0·6–0·8)
Diet low in whole grains	45·8 (37·7–52·3)	49·0 (40·7–55·8)	50·6 (41·7–57·9)	51·0 (42·4–58·4)	0·3 (0·1–0·6)	0·2 (-0·1–0·6)	0·1 (-0·4–0·6)
Diet low in nuts and seeds	12·9 (9·1–17·1)	7·4 (4·8–11·0)	5·5 (3·3–8·3)	5·9 (3·1–9·5)	-2·5 (-4·5–-0·9)	-1·1 (-3·9–1·2)	0·6 (-3·6–4·2)
Diet low in milk	36·3 (31·8–40·8)	33·2 (29·1–37·9)	31·9 (27·6–36·2)	31·5 (26·8–36·2)	-0·5 (-0·9–0·0)	-0·2 (-0·9–0·4)	-0·1 (-0·9–0·8)
Diet high in red meat	49·8 (0·0–68·5)	50·4 (0·0–68·8)	50·3 (0·0–69·5)	50·4 (0·0–69·3)	0·0 (-0·5–0·6)	0·0 (-0·8–0·6)	0·0 (-0·9–1·1)
Diet high in processed meat	47·0 (36·9–52·5)	58·7 (47·5–65·4)	65·7 (53·4–73·1)	63·9 (51·7–72·0)	1·0 (0·5–1·5)	0·4 (-0·2–1·0)	-0·3 (-1·1–0·6)
Diet high in sugar-sweetened beverages	36·4 (27·8–45·5)	51·1 (40·4–60·4)	56·2 (45·4–66·7)	55·7 (43·3–66·2)	1·4 (0·6–2·2)	0·4 (-0·5–1·3)	-0·1 (-1·5–1·2)
Diet low in fibre	39·6 (21·9–47·1)	29·6 (17·2–36·1)	26·3 (15·6–33·0)	22·9 (13·2–30·0)	-1·8 (-2·7–-0·9)	-1·2 (-2·5–0·0)	-1·3 (-3·3–0·4)
Diet low in calcium	5·6 (4·6–7·0)	4·7 (3·8–5·8)	5·0 (4·0–6·1)	4·9 (3·9–6·1)	-0·4 (-0·7–-0·2)	0·2 (-0·1–0·5)	-0·1 (-0·5–0·2)
Diet low in seafood omega-3 fatty acids	36·7 (27·0–46·5)	43·0 (32·5–53·1)	46·5 (35·5–56·9)	41·3 (31·4–52·2)	0·4 (-0·1–0·8)	-0·2 (-0·8–0·4)	-1·1 (-1·9–-0·4)
Diet low in omega-6 polyunsaturated fatty acids	36·0 (23·2–46·3)	30·9 (20·7–39·7)	23·3 (16·1–29·7)	21·7 (14·9–28·6)	-1·6 (-2·3–-0·8)	-1·7 (-2·6–-0·8)	-0·6 (-1·7–0·3)
Diet high in trans fatty acids	71·3 (63·9–78·8)	71·8 (64·4–79·2)	63·2 (54·2–70·9)	0·0 (0·0–0·0)	-65·8 (-66·1–-65·4)	-97·1 (-97·6–-96·6)	-184·2 (-185·3–-182·8)
Diet high in sodium	23·2 (0·8–67·1)	28·2 (2·0–74·4)	31·0 (3·3–75·3)	31·3 (3·1–76·3)	1·0 (0·2–5·5)	0·5 (-0·2–3·7)	0·1 (-2·5–1·9)
Intimate partner violence	25·7 (14·0–33·2)	26·0 (15·6–33·0)	23·9 (15·5–29·6)	22·7 (12·7–30·0)	-0·4 (-1·4–0·6)	-0·6 (-2·0–0·7)	-0·5 (-2·2–1·2)
Childhood sexual abuse and bullying	9·0 (5·9–14·2)	10·5 (6·7–16·5)	11·4 (7·1–18·4)	9·6 (6·1–15·4)	0·2 (-0·3–0·8)	-0·4 (-1·2–0·4)	-1·6 (-2·9–-0·2)
Childhood sexual abuse	8·6 (7·4–9·9)	8·6 (7·6–9·8)	8·7 (8·0–9·6)	8·6 (7·4–10·1)	0·0 (-0·6–0·6)	0·0 (-0·8–0·8)	-0·1 (-1·2–1·1)
Bullying victimization	8·0 (3·5–15·7)	10·0 (4·6–19·4)	11·3 (5·1–22·0)	8·7 (3·8–17·0)	0·3 (-0·4–1·0)	-0·6 (-1·7–0·3)	-2·4 (-4·2–-0·5)
Unsafe sex	--	--	--	--	--	--	--
Low physical activity	18·5 (13·6–24·1)	17·6 (13·3–21·9)	18·4 (14·2–23·0)	21·4 (15·6–27·3)	0·5 (-0·5–1·5)	0·9 (-0·2–2·2)	1·4 (-0·3–3·3)
**Metabolic risks**	**26·0 (22·5–29·4)**	**30·6 (26·8–33·6)**	**34·3 (30·3–37·2)**	**38·0 (33·1–41·1)**	**1·2 (0·9–1·5)**	**1·0 (0·7–1·4)**	**1·0 (0·5–1·4)**
High fasting plasma glucose	13·4 (10·5–16·1)	16·4 (12·8–19·4)	20·8 (15·7–24·8)	24·5 (18·4–29·6)	2·0 (1·3–2·7)	1·9 (1·0–2·8)	1·5 (0·3–2·8)
High LDL cholesterol	63·6 (44·6–86·8)	53·8 (36·8–74·2)	48·1 (32·7–66·6)	46·6 (31·2–64·8)	-1·0 (-1·2–-0·8)	-0·7 (-0·9–-0·5)	-0·3 (-0·5–0·0)
High systolic blood pressure	29·1 (19·9–40·8)	25·1 (17·5–34·7)	18·2 (11·9–26·0)	24·8 (16·1–35·3)	-0·5 (-1·4–0·3)	0·0 (-1·2–1·0)	2·8 (0·6–5·0)
High body-mass index	29·7 (24·9–33·9)	35·3 (30·1–39·5)	39·6 (33·5–43·6)	43·2 (36·3–47·3)	1·2 (0·9–1·5)	1·0 (0·7–1·3)	0·8 (0·4–1·2)
Low bone mineral density	21·5 (15·6–28·6)	19·5 (13·3–26·6)	20·2 (14·2–27·0)	22·0 (16·0–29·3)	0·1 (-0·4–0·5)	0·6 (-0·1–1·4)	0·8 (-0·6–2·3)
Kidney dysfunction	3·0 (2·4–3·9)	3·0 (2·5–4·0)	3·0 (2·4–4·0)	3·1 (2·5–4·0)	0·1 (0·0–0·2)	0·0 (-0·1–0·2)	0·1 (-0·2–0·4)

Wyoming

Risk Names	SEV 1990	SEV 2000	SEV 2010	SEV 2021	Annualised rate of change 1990 to 2021	Annualised rate of change 2000 to 2021	Annualised rate of change 2010 to 2021
**All risk factors**	**25·1 (22·2–27·9)**	**24·3 (21·7–27·1)**	**24·6 (21·9–27·4)**	**24·7 (21·9–28·0)**	**0·0 (-0·3–0·2)**	**0·1 (-0·3–0·4)**	**0·0 (-0·5–0·6)**
**Environmental/occupational risks**	**14·6 (9·5–24·9)**	**14·5 (9·3–24·0)**	**13·2 (8·5–22·6)**	**12·1 (7·6–20·8)**	**-0·6 (-1·2–-0·2)**	**-0·8 (-1·3–-0·4)**	**-0·7 (-1·2–-0·3)**
Unsafe water, sanitation, and handwashing	2·1 (1·0–3·4)	1·9 (0·9–3·1)	1·4 (0·5–2·4)	1·1 (0·4–1·9)	-1·9 (-4·3–-0·1)	-2·3 (-5·7–0·2)	-1·9 (-5·6–2·2)
Unsafe water source	2·9 (1·0–6·1)	2·6 (1·0–5·3)	1·0 (0·4–2·0)	0·8 (0·4–1·6)	-4·1 (-7·0–-1·0)	-5·4 (-9·2–-1·3)	-1·9 (-7·2–3·6)
Unsafe sanitation	6·7 (3·6–11·6)	5·1 (2·7–9·0)	3·7 (1·9–6·6)	2·8 (1·4–4·9)	-2·8 (-5·1–-0·8)	-2·8 (-5·7–-0·1)	-2·5 (-5·7–0·8)
No access to handwashing facility	1·8 (0·6–3·4)	1·8 (0·6–3·3)	1·8 (0·6–3·3)	1·5 (0·5–2·9)	-0·5 (-3·4–2·3)	-0·9 (-4·9–2·8)	-1·5 (-7·0–4·1)
Air pollution	12·2 (1·1–23·0)	11·7 (1·9–21·6)	5·9 (0·6–14·8)	4·8 (0·3–13·4)	-3·0 (-7·9–-1·3)	-4·3 (-9·9–-2·1)	-1·9 (-8·3–-0·7)
Particulate matter pollution	4·1 (0·2–10·7)	3·4 (1·1–6·2)	1·4 (0·1–3·7)	0·6 (0·0–2·1)	-6·4 (-17·6–-0·6)	-8·6 (-22·8–-5·0)	-8·2 (-24·3–-4·7)
Ambient particulate matter pollution	6·2 (0·3–15·8)	5·2 (1·6–9·3)	2·1 (0·2–5·5)	0·9 (0·0–3·2)	-6·4 (-17·6–-0·6)	-8·6 (-22·8–-5·0)	-8·2 (-24·3–-4·7)
Household air pollution from solid fuels	0·0 (0·0–0·0)	0·0 (0·0–0·0)	0·0 (0·0–0·0)	0·0 (0·0–0·0)	-7·2 (-52·6–-11·5)	-10·1 (-63·4–-15·2)	-8·4 (-52·8–-11·4)
Ambient ozone pollution	18·4 (12·3–25·4)	25·2 (18·5–33·8)	23·3 (17·4–31·1)	19·6 (13·3–27·2)	0·2 (-0·4–0·8)	-1·2 (-2·1–-0·4)	-1·6 (-3·1–-0·1)
Ambient nitrogen dioxide pollution	47·8 (0·0–100·0)	47·8 (0·0–100·0)	24·5 (0·0–73·9)	22·8 (0·0–70·4)	-2·4 (-8·0–0·0)	-3·5 (-11·6–0·0)	-0·7 (-1·5–0·0)
Non-optimal temperature	31·0 (25·3–39·5)	33·3 (26·4–43·0)	31·0 (25·2–39·0)	32·3 (25·6–41·5)	0·1 (0·0–0·3)	-0·1 (-0·3–0·0)	0·4 (-0·2–1·0)
High temperature	24·7 (14·5–37·3)	30·0 (18·8–43·2)	25·3 (15·2–38·0)	30·5 (19·0–44·5)	0·7 (0·3–1·2)	0·1 (-0·2–0·4)	1·7 (0·8–2·8)
Low temperature	29·2 (25·6–33·9)	30·5 (26·5–35·5)	29·2 (25·0–34·4)	29·6 (25·6–34·6)	0·0 (-0·1–0·1)	-0·1 (-0·3–-0·1)	0·1 (-0·1–0·3)
Other environmental risks	26·4 (9·6–46·4)	25·1 (9·6–45·4)	23·4 (9·2–43·4)	21·2 (8·6–41·4)	-0·7 (-1·4–0·0)	-0·8 (-1·8–0·0)	-0·9 (-2·0–0·0)
Residential radon	34·6 (0·0–100·0)	34·6 (0·0–100·0)	34·6 (0·0–100·0)	34·6 (0·0–100·0)	0·0 (0·0–0·0)	0·0 (0·0–0·0)	0·0 (0·0–0·0)
Lead exposure	22·7 (0·0–28·3)	20·9 (0·0–26·5)	18·3 (0·0–23·2)	15·2 (0·0–19·2)	-1·3 (-1·8–0·0)	-1·5 (-2·1–0·0)	-1·7 (-2·6–0·0)
Occupational risks	3·2 (2·7–4·1)	3·3 (2·7–4·2)	3·0 (2·5–3·9)	2·9 (2·4–3·9)	-0·3 (-0·7–0·0)	-0·5 (-0·9–-0·1)	-0·1 (-0·7–0·3)
Occupational carcinogens	1·0 (0·5–1·8)	1·1 (0·5–2·0)	1·1 (0·5–2·0)	1·1 (0·5–2·0)	0·3 (-0·1–0·6)	0·0 (-0·4–0·4)	0·1 (-0·5–0·6)
Occupational exposure to asbestos	5·6 (5·0–6·2)	6·1 (5·5–6·6)	5·5 (4·9–6·0)	5·0 (4·1–6·0)	-0·4 (-1·0–0·3)	-0·9 (-1·9–0·0)	-0·9 (-2·9–0·8)
Occupational exposure to arsenic	0·4 (0·0–1·0)	0·4 (0·0–1·1)	0·4 (0·0–1·1)	0·4 (0·0–1·1)	0·1 (-0·3–0·5)	0·0 (-0·6–0·5)	0·2 (-0·7–1·0)
Occupational exposure to benzene	1·1 (0·1–3·0)	1·2 (0·1–3·5)	1·2 (0·1–3·5)	1·2 (0·1–3·6)	0·5 (0·1–0·8)	0·2 (-0·2–0·6)	0·3 (-0·4–0·9)
Occupational exposure to beryllium	0·0 (0·0–0·0)	0·0 (0·0–0·0)	0·0 (0·0–0·0)	0·0 (0·0–0·0)	0·2 (-0·1–0·4)	0·1 (-0·2–0·4)	0·4 (-0·1–0·8)
Occupational exposure to cadmium	0·1 (0·0–0·1)	0·1 (0·1–0·1)	0·1 (0·0–0·1)	0·1 (0·0–0·1)	0·2 (-0·2–0·5)	0·0 (-0·5–0·5)	0·3 (-0·5–1·0)
Occupational exposure to chromium	0·1 (0·1–0·1)	0·1 (0·1–0·1)	0·1 (0·1–0·1)	0·1 (0·1–0·1)	0·2 (-0·1–0·5)	0·1 (-0·4–0·5)	0·3 (-0·4–0·9)
Occupational exposure to diesel engine exhaust	0·9 (0·8–0·9)	0·8 (0·8–0·9)	0·8 (0·7–0·8)	0·8 (0·7–0·9)	-0·2 (-0·6–0·1)	-0·2 (-0·6–0·4)	0·3 (-0·4–1·1)
Occupational exposure to formaldehyde	0·3 (0·2–0·3)	0·3 (0·2–0·3)	0·3 (0·2–0·3)	0·3 (0·2–0·3)	0·2 (-0·2–0·5)	0·0 (-0·6–0·5)	0·1 (-0·6–0·9)
Occupational exposure to nickel	0·5 (0·0–1·8)	0·4 (0·0–1·7)	0·4 (0·0–1·5)	0·4 (0·0–1·5)	-0·7 (-1·3–0·0)	-0·8 (-1·5–0·0)	-0·2 (-1·2–0·8)
Occupational exposure to polycyclic aromatic hydrocarbons	0·3 (0·3–0·3)	0·3 (0·3–0·3)	0·3 (0·3–0·3)	0·3 (0·3–0·3)	0·2 (-0·1–0·5)	0·0 (-0·4–0·4)	0·2 (-0·4–0·8)
Occupational exposure to silica	5·3 (0·8–17·7)	4·7 (0·8–15·3)	3·8 (0·6–12·1)	3·6 (0·6–11·7)	-1·2 (-1·8–-0·7)	-1·2 (-2·0–-0·4)	-0·4 (-1·5–0·6)
Occupational exposure to sulphuric acid	0·7 (0·1–2·2)	0·7 (0·1–2·3)	0·7 (0·1–2·2)	0·7 (0·1–2·2)	0·2 (-0·2–0·6)	0·0 (-0·5–0·6)	0·3 (-0·6–1·0)
Occupational exposure to trichloroethylene	0·1 (0·1–0·1)	0·1 (0·1–0·1)	0·1 (0·1–0·1)	0·1 (0·1–0·1)	0·3 (0·0–0·6)	0·2 (-0·1–0·6)	0·4 (-0·1–1·0)
Occupational asthmagens	20·0 (17·9–22·7)	19·6 (17·6–21·9)	17·1 (14·9–19·5)	17·0 (14·7–19·4)	-0·5 (-1·0–-0·1)	-0·7 (-1·3–-0·1)	-0·1 (-0·9–0·7)
Occupational particulate matter, gases, and fumes	7·2 (5·5–9·4)	7·0 (5·4–9·0)	6·5 (5·0–8·5)	6·0 (4·6–7·7)	-0·6 (-0·8–-0·4)	-0·7 (-1·0–-0·4)	-0·8 (-1·2–-0·5)
Occupational noise	8·3 (7·7–9·1)	8·2 (7·6–8·9)	7·9 (7·4–8·5)	7·4 (7·0–7·9)	-0·4 (-0·6–-0·2)	-0·5 (-0·7–-0·3)	-0·6 (-0·8–-0·4)
Occupational injuries	--	--	--	--	--	--	--
Occupational ergonomic factors	9·4 (7·8–11·2)	9·3 (7·9–11·1)	8·4 (7·1–10·0)	8·5 (7·1–10·4)	-0·3 (-0·9–0·1)	-0·4 (-1·1–0·1)	0·2 (-0·6–0·9)
**Behavioural risks**	**30·0 (26·7–33·8)**	**28·1 (25·0–32·1)**	**26·8 (23·6–31·0)**	**24·7 (21·6–28·8)**	**-0·6 (-1·0–-0·3)**	**-0·6 (-1·1–-0·2)**	**-0·7 (-1·5–0·0)**
Child and maternal malnutrition	10·2 (7·0–14·6)	8·8 (6·1–12·4)	9·0 (6·2–12·3)	9·1 (6·1–12·7)	-0·4 (-1·2–0·4)	0·1 (-1·1–1·3)	0·1 (-1·8–1·8)
Suboptimal breastfeeding	68·8 (66·4–71·7)	66·7 (64·3–69·3)	62·9 (60·2–66·1)	62·3 (59·5–65·4)	-0·3 (-0·5–-0·2)	-0·3 (-0·5–-0·1)	-0·1 (-0·4–0·2)
Non-exclusive breastfeeding	64·3 (56·3–71·6)	59·7 (52·6–67·1)	48·7 (42·1–55·7)	46·5 (39·0–54·4)	-1·0 (-1·6–-0·5)	-1·2 (-1·9–-0·6)	-0·4 (-1·6–0·5)
Discontinued breastfeeding	88·7 (86·4–91·1)	86·4 (83·7–89·3)	84·4 (81·0–87·6)	84·6 (81·6–88·2)	-0·1 (-0·3–0·0)	-0·1 (-0·3–0·1)	0·0 (-0·3–0·3)
Child growth failure	1·3 (0·4–2·9)	1·2 (0·4–2·8)	1·1 (0·3–2·3)	1·0 (0·3–2·2)	-1·0 (-1·4–-0·6)	-1·1 (-1·7–-0·6)	-0·7 (-1·6–0·0)
Child underweight	2·7 (1·7–3·8)	2·6 (1·7–3·5)	2·2 (1·4–3·0)	2·0 (1·2–2·8)	-1·0 (-1·5–-0·4)	-1·2 (-2·0–-0·6)	-0·7 (-1·8–0·3)
Child wasting	0·9 (0·6–1·4)	0·8 (0·5–1·3)	0·8 (0·5–1·3)	0·8 (0·5–1·2)	-0·3 (-0·6–0·1)	-0·2 (-0·7–0·3)	-0·3 (-1·0–0·4)
Child stunting	3·8 (2·9–4·7)	3·6 (2·8–4·4)	3·1 (2·4–3·8)	2·8 (2·1–3·5)	-1·0 (-1·6–-0·4)	-1·2 (-2·0–-0·4)	-0·9 (-2·1–0·2)
Low birth weight and short gestation	17·8 (15·5–20·1)	18·5 (16·4–20·9)	22·2 (19·6–25·2)	21·9 (19·2–24·7)	0·7 (0·4–0·9)	0·8 (0·4–1·2)	-0·1 (-0·9–0·5)
Short gestation	28·8 (25·6–32·5)	29·9 (26·5–33·8)	33·0 (29·3–37·2)	32·0 (28·2–36·4)	0·3 (0·0–0·7)	0·3 (-0·1–0·8)	-0·3 (-1·1–0·7)
Low birth weight	14·5 (13·1–15·9)	15·0 (13·7–16·5)	18·4 (16·8–20·1)	18·4 (16·6–20·0)	0·8 (0·5–1·0)	1·0 (0·6–1·3)	0·0 (-0·8–0·6)

Iron deficiency	3·6 (2·5–5·0)	3·1 (2·2–4·3)	3·2 (2·3–4·2)	3·1 (2·2–4·3)	-0·4 (-1·2–0·3)	0·0 (-1·1–1·1)	-0·1 (-1·8–1·5)
Vitamin A deficiency	1·1 (0·0–2·4)	1·5 (0·0–3·2)	0·9 (0·0–1·8)	0·5 (0·0–1·1)	-2·6 (-4·6–0·0)	-5·1 (-8·1–0·0)	-4·9 (-8·6–0·0)
Zinc deficiency	1·9 (0·0–8·2)	1·1 (0·0–4·8)	0·9 (0·0–4·0)	0·9 (0·0–3·7)	-2·4 (-47·6–0·0)	-1·0 (-4·6–4·6)	-0·2 (-8·8–15·4)
Tobacco	40·9 (38·7–42·9)	35·4 (33·9–36·8)	31·2 (30·1–32·3)	26·9 (25·2–28·6)	-1·3 (-1·6–-1·1)	-1·3 (-1·6–-1·0)	-1·3 (-1·9–-0·8)
Smoking	38·1 (35·1–40·9)	32·3 (30·1–34·2)	27·8 (26·3–29·4)	23·9 (21·7–26·4)	-1·5 (-1·8–-1·2)	-1·4 (-1·8–-1·0)	-1·4 (-2·2–-0·7)
Chewing tobacco	3·9 (2·9–5·0)	4·5 (3·5–5·6)	4·7 (3·6–6·0)	4·7 (3·2–6·3)	0·6 (-0·7–1·9)	0·2 (-1·5–1·9)	-0·1 (-2·7–2·4)
Second-hand smoke	32·6 (30·4–34·5)	28·4 (26·6–29·6)	25·8 (23·9–26·9)	22·4 (20·5–24·1)	-1·2 (-1·5–-0·9)	-1·1 (-1·5–-0·8)	-1·3 (-1·9–-0·7)
High alcohol use	26·4 (18·2–37·0)	25·3 (17·7–35·4)	25·8 (17·7–36·2)	25·6 (17·8–36·2)	-0·1 (-1·3–1·1)	0·1 (-1·4–1·4)	-0·1 (-2·1–2·0)
Drug use	0·8 (0·5–1·4)	1·0 (0·8–1·3)	1·9 (1·5–2·2)	4·7 (3·4–5·9)	5·6 (3·0–7·7)	7·1 (4·9–8·6)	8·4 (6·3–9·7)
Dietary risks	36·2 (27·0–47·2)	39·6 (30·4–50·8)	43·3 (34·0–55·2)	43·8 (34·4–55·7)	0·6 (0·4–0·9)	0·5 (0·2–0·8)	0·1 (-0·4–0·6)
Diet low in fruits	34·0 (29·5–37·3)	30·6 (26·6–33·5)	32·8 (28·0–35·6)	34·9 (29·1–39·1)	0·1 (-0·3–0·5)	0·6 (0·0–1·2)	0·6 (-0·3–1·4)
Diet low in vegetables	24·6 (13·8–32·3)	28·2 (15·7–35·9)	33·7 (18·2–41·4)	33·9 (19·0–42·2)	1·0 (0·6–1·5)	0·9 (0·4–1·4)	0·0 (-0·7–0·7)
Diet low in legumes	38·3 (0·0–48·2)	38·5 (0·0–47·9)	41·4 (0·0–51·8)	41·7 (0·0–52·8)	0·3 (0·0–0·6)	0·4 (-0·1–0·8)	0·1 (-0·6–0·8)
Diet low in whole grains	45·4 (37·8–52·0)	48·5 (40·6–55·1)	51·1 (42·7–58·7)	51·5 (42·6–59·2)	0·4 (0·1–0·7)	0·3 (-0·1–0·7)	0·1 (-0·6–0·6)
Diet low in nuts and seeds	12·8 (9·3–16·7)	7·6 (5·0–10·6)	4·5 (2·7–6·8)	4·6 (2·4–7·2)	-3·3 (-5·4–-1·7)	-2·4 (-5·2–0·2)	0·1 (-3·7–4·5)
Diet low in milk	35·9 (31·7–40·9)	33·2 (29·0–37·9)	29·8 (25·8–34·2)	28·6 (24·5–33·6)	-0·7 (-1·2–-0·3)	-0·7 (-1·4–0·0)	-0·4 (-1·3–0·6)
Diet high in red meat	49·5 (0·0–67·5)	50·0 (0·0–68·2)	51·6 (0·0–70·7)	52·1 (0·0–71·6)	0·2 (-0·3–0·7)	0·2 (-0·5–1·0)	0·1 (-0·9–1·3)
Diet high in processed meat	46·9 (37·4–52·5)	58·3 (47·7–65·2)	67·5 (55·5–76·1)	66·0 (53·9–75·1)	1·1 (0·7–1·5)	0·6 (0·0–1·1)	-0·2 (-1·0–0·6)
Diet high in sugar-sweetened beverages	36·4 (27·6–45·1)	50·2 (39·3–60·1)	59·5 (47·7–69·8)	60·2 (48·3–70·3)	1·6 (0·9–2·5)	0·9 (0·0–1·8)	0·1 (-1·2–1·3)
Diet low in fibre	39·2 (20·8–45·9)	29·8 (16·2–36·7)	24·1 (13·7–29·9)	20·5 (11·6–27·7)	-2·1 (-3·2–-1·1)	-1·8 (-3·2–-0·5)	-1·5 (-3·5–0·3)
Diet low in calcium	5·5 (4·5–6·8)	4·7 (3·8–5·8)	4·6 (3·7–5·7)	4·4 (3·5–5·4)	-0·8 (-1·0–-0·5)	-0·3 (-0·6–-0·1)	-0·4 (-0·7–-0·1)
Diet low in seafood omega-3 fatty acids	49·3 (39·1–60·4)	56·0 (45·3–67·4)	56·4 (45·6–67·8)	51·1 (41·1–62·6)	0·1 (-0·2–0·5)	-0·4 (-0·8–0·0)	-0·9 (-1·5–-0·3)
Diet low in omega-6 polyunsaturated fatty acids	35·6 (23·5–45·6)	31·1 (21·1–40·5)	21·6 (14·8–28·0)	19·8 (13·8–26·1)	-1·9 (-2·7–-1·1)	-2·2 (-3·2–-1·2)	-0·8 (-1·9–0·2)
Diet high in trans fatty acids	71·4 (64·3–79·0)	71·7 (63·7–78·9)	63·5 (56·7–70·9)	0·0 (0·0–0·0)	-65·8 (-66·1–-65·4)	-97·1 (-97·6–-96·5)	-184·3 (-185·3–-183·2)
Diet high in sodium	22·9 (0·5–66·6)	27·7 (1·8–72·6)	30·8 (3·4–73·8)	31·5 (3·4–75·8)	1·0 (0·3–6·4)	0·6 (-0·1–4·2)	0·2 (-2·2–2·2)
Intimate partner violence	28·2 (15·7–36·5)	28·2 (16·1–35·5)	25·6 (17·8–32·3)	24·4 (13·4–33·1)	-0·5 (-1·9–0·7)	-0·7 (-2·6–0·9)	-0·4 (-3·0–1·7)
Childhood sexual abuse and bullying	10·0 (6·7–15·2)	12·0 (8·1–18·3)	13·7 (9·2–20·7)	11·1 (7·7–16·6)	0·3 (-0·3–1·0)	-0·4 (-1·3–0·5)	-1·9 (-3·6–-0·4)
Childhood sexual abuse	10·8 (9·2–12·6)	12·2 (10·6–14·2)	14·1 (12·4–16·3)	12·9 (10·4–16·0)	0·6 (-0·2–1·4)	0·3 (-0·8–1·4)	-0·8 (-2·2–0·9)
Bullying victimization	8·1 (3·6–16·1)	10·1 (4·6–19·8)	11·4 (5·1–22·1)	8·2 (3·6–15·6)	0·0 (-0·7–0·7)	-1·0 (-2·1–0·0)	-3·0 (-4·9–-1·0)
Unsafe sex	--	--	--	--	--	--	--
Low physical activity	17·8 (13·4–23·0)	16·4 (12·3–20·8)	18·0 (13·8–22·2)	20·7 (15·5–26·3)	0·5 (-0·6–1·4)	1·1 (-0·2–2·4)	1·2 (-0·8–3·1)
**Metabolic risks**	**26·9 (23·6–30·4)**	**29·9 (26·3–33·2)**	**33·7 (29·6–36·7)**	**37·2 (32·4–40·5)**	**1·0 (0·8–1·4)**	**1·0 (0·7–1·4)**	**0·9 (0·4–1·4)**
High fasting plasma glucose	12·3 (9·5–15·3)	15·4 (11·4–19·0)	20·0 (14·7–24·4)	24·1 (18·1–29·5)	2·2 (1·4–3·0)	2·1 (1·2–3·2)	1·7 (0·4–3·1)
High LDL cholesterol	63·7 (45·0–86·7)	53·9 (36·8–74·8)	48·4 (32·7–67·9)	47·0 (31·8–66·0)	-1·0 (-1·2–-0·8)	-0·6 (-0·8–-0·5)	-0·3 (-0·5–0·0)
High systolic blood pressure	27·7 (18·4–38·4)	23·5 (16·2–32·6)	17·6 (11·4–25·4)	24·6 (15·6–34·7)	-0·4 (-1·3–0·6)	0·2 (-0·9–1·4)	3·1 (0·9–5·6)
High body-mass index	30·7 (25·7–35·2)	34·3 (29·2–38·6)	38·9 (33·2–42·7)	42·1 (35·7–46·1)	1·0 (0·7–1·3)	1·0 (0·6–1·3)	0·7 (0·3–1·2)
Low bone mineral density	21·7 (15·8–28·7)	19·7 (13·8–26·7)	20·3 (14·3–27·3)	22·0 (15·8–29·1)	0·0 (-0·4–0·5)	0·5 (-0·2–1·3)	0·7 (-0·6–2·3)
Kidney dysfunction	2·8 (2·3–3·8)	2·9 (2·4–3·8)	2·9 (2·3–3·8)	3·0 (2·4–3·9)	0·1 (0·0–0·2)	0·1 (-0·1–0·2)	0·3 (0·0–0·7)

Table S6: Age-standardised risk-attributable DALY rates per 100 000 in the USA and by US state and Washington DC for all risk–outcome pairs combined and excluding one- and two-star pairs in 1990, 2010, and 2021						
Risk-outcome pairs are given a one- to five-star rating in the burden of proof risk function framework based on strength of evidence for an association. One- and two-star pairs are those with weak evidence of an association						
	Age-standardised risk-attributable DALY rate (per 100 000) for all risk–outcome pairs (95% UI)			Age-standardised risk-attributable DALY rate (per 100 000) excluding one- and two-star pairs (95% UI)		
Location	1990	2010	2021	1990	2010	2021
United States of America	12297.0 (11327.2–13431.8)	10294.7 (9241.7–11451.3)	10940.8 (9755.4–12320.2)	10964.3 (10177.6–11776.9)	8901.9 (8085.7–9744.7)	9595.7 (8644.8–10635.3)
Alabama	13638.8 (12578.0–14935.3)	13403.8 (12116.4–14785.1)	13794.3 (11999.4–15774.2)	12182.4 (11356.7–13129.6)	11688.4 (10732.2–12682.5)	12119.9 (10663.2–13681.9)
Alaska	11527.2 (10444.2–12717.2)	9992.3 (8861.4–11236.8)	10999.9 (9589.8–12581.3)	10529.8 (9681.0–11404.3)	8835.7 (7962.1–9746.2)	9924.8 (8724.0–11200.3)
Arizona	11443.8 (10449.9–12552.4)	10065.1 (9036.8–11188.3)	11321.0 (9682.8–13044.2)	10161.4 (9350.8–11048.8)	8678.1 (7811.9–9573.2)	9906.5 (8650.7–11297.3)
Arkansas	12942.7 (11895.9–14136.5)	12175.3 (11069.8–13487.1)	13166.7 (11375.5–14921.7)	11581.8 (10749.9–12449.5)	10610.4 (9746.1–11554.9)	11603.7 (10194.2–13017.2)
California	11860.2 (10856.0–12990.5)	8566.4 (7632.1–9645.5)	8886.0 (7655.2–10255.0)	10572.7 (9736.8–11371.2)	7270.5 (6558.2–8027.6)	7643.2 (6732.4–8636.4)
Colorado	10240.1 (9285.2–11299.3)	8679.4 (7698.6–9792.7)	9483.6 (8113.2–11001.1)	9130.2 (8361.7–9923.6)	7510.6 (6778.4–8307.0)	8339.9 (7178.5–9526.0)
Connecticut	10946.1 (10056.5–12043.2)	9222.8 (8189.8–10381.8)	9635.5 (8264.6–11131.6)	9652.2 (8924.0–10449.1)	7959.8 (7154.5–8796.4)	8442.5 (7260.8–9732.5)
Delaware	13174.9 (12030.1–14411.1)	11187.9 (10033.1–12545.3)	12167.7 (10550.7–13998.8)	11722.1 (10864.2–12655.4)	9718.2 (8831.9–10727.8)	10750.5 (9481.0–12149.2)
District of Columbia	21761.6 (20364.4–23175.9)	12546.3 (11418.9–13863.9)	12065.5 (10487.9–13759.6)	19958.0 (18956.3–21083.5)	11137.4 (10262.9–12088.6)	10763.1 (9374.6–12121.4)
Florida	12267.1 (11268.0–13376.6)	10378.7 (9304.6–11531.8)	11226.9 (9717.1–12980.2)	11048.1 (10262.5–11851.7)	9068.4 (8262.3–9936.4)	9959.1 (8675.1–11286.1)
Georgia	13705.0 (12649.4–14845.9)	11231.9 (10108.1–12420.5)	11562.7 (9926.1–13218.1)	12336.2 (11549.3–13205.3)	9761.9 (8947.6–10621.3)	10154.2 (8920.4–11471.3)
Hawaii	9665.4 (8670.4–10783.0)	8488.3 (7533.7–9632.8)	8764.7 (7500.8–10200.4)	8550.1 (7778.9–9384.4)	7295.7 (6553.3–8137.0)	7596.0 (6619.9–8664.9)
Idaho	10040.7 (9061.7–11177.3)	9062.4 (8027.5–10274.7)	9356.8 (7914.0–10842.1)	8912.1 (8062.5–9725.8)	7769.9 (7002.8–8619.8)	8072.8 (6895.4–9235.1)
Illinois	12762.0 (11757.6–13885.0)	10315.9 (9221.2–11447.6)	10732.5 (9337.6–12371.6)	11352.4 (10570.8–12149.6)	8932.2 (8152.1–9777.6)	9392.1 (8316.3–10687.6)
Indiana	12150.5 (11092.6–13355.4)	11571.4 (10354.5–12819.0)	12698.7 (11093.6–14368.8)	10833.6 (10003.0–11673.7)	10124.3 (9268.7–11088.3)	11259.2 (9957.2–12651.5)
Iowa	10087.2 (9128.4–11204.1)	9157.8 (8147.6–10273.8)	9659.2 (8201.1–11217.8)	8915.1 (8160.4–9710.3)	7844.9 (7103.6–8608.0)	8349.5 (7226.0–9517.1)
Kansas	10673.4 (9683.0–11799.9)	10105.9 (9088.3–11276.7)	10808.4 (9247.5–12425.0)	9436.9 (8669.1–10269.2)	8679.6 (7844.5–9496.5)	9386.7 (8156.1–10645.7)
Kentucky	13229.5 (12089.6–14455.1)	13267.9 (11997.0–14646.8)	14954.7 (12979.8–17083.5)	11796.1 (10975.2–12651.7)	11595.5 (10620.9–12580.9)	13275.9 (11635.6–14974.7)
Louisiana	14445.8 (13332.8–15773.5)	13115.0 (11920.8–14434.1)	14099.6 (12229.6–16216.3)	12935.3 (12023.2–13903.9)	11438.1 (10500.1–12474.8)	12477.8 (11008.3–14132.6)
Maine	10928.7 (9963.2–12125.2)	9669.6 (8606.6–10833.1)	11216.2 (9601.9–12849.7)	9699.3 (8910.4–10588.6)	8385.0 (7565.2–9268.7)	9952.5 (8710.9–11272.8)
Maryland	12747.6 (11674.0–13935.6)	10329.6 (9247.7–11490.7)	10643.4 (9217.9–12309.2)	11297.5 (10491.6–12132.0)	8939.5 (8149.3–9807.8)	9325.0 (8136.0–10697.3)
Massachusetts	11222.3 (10227.4–12391.0)	9244.1 (8249.6–10354.2)	9468.7 (7949.4–10953.1)	9851.1 (9068.6–10709.5)	7970.5 (7223.4–8824.3)	8317.5 (7038.4–9475.6)
Michigan	12596.7 (11548.2–13789.7)	11173.3 (10039.1–12364.7)	11966.2 (10368.0–13815.3)	11256.6 (10406.3–12101.5)	9747.3 (8893.4–10650.7)	10599.2 (9312.5–12039.9)
Minnesota	9796.8 (8834.8–10889.8)	7980.0 (7040.0–9033.0)	8592.7 (7267.1–9959.2)	8673.8 (7956.3–9458.1)	6852.7 (6151.1–7634.4)	7473.6 (6411.9–8489.1)
Mississippi	14193.4 (13134.4–15382.0)	13479.7 (12329.6–14796.9)	14487.7 (12475.8–16594.8)	12748.5 (11902.8–13618.8)	11819.7 (10876.6–12816.1)	12830.1 (11253.8–14626.6)
Missouri	12256.4 (11269.8–13422.0)	11548.9 (10457.4–12753.0)	12611.1 (10884.9–14300.0)	10903.3 (10081.2–11768.2)	10043.5 (9210.3–10941.1)	11135.7 (9738.8–12504.6)
Montana	10302.6 (9273.9–11464.2)	9504.6 (8435.5–10670.5)	10139.5 (8834.1–11616.0)	9255.1 (8432.4–10106.3)	8319.7 (7488.7–9187.4)	8931.8 (7800.7–10119.9)
Nebraska	10424.3 (9409.8–11549.8)	8931.4 (7939.2–10104.5)	9426.9 (8016.0–10844.3)	9172.2 (8441.6–9980.8)	7596.0 (6811.6–8445.7)	8060.8 (6995.5–9213.7)
Nevada	13213.8 (12119.2–14417.5)	11190.4 (10092.4–12427.1)	11670.6 (10088.0–13227.6)	11829.1 (10947.9–12761.1)	9700.9 (8807.5–10627.4)	10228.5 (8938.2–11472.7)
New Hampshire	10969.7 (9971.1–12090.3)	9306.3 (8249.2–10487.8)	10724.5 (9039.5–12354.0)	9686.4 (8900.6–10520.4)	8057.9 (7224.8–9009.1)	9499.3 (8076.1–10737.6)
New Jersey	12617.1 (11649.3–13762.5)	9345.9 (8356.5–10473.6)	9664.5 (8216.2–11159.5)	11206.0 (10437.6–12041.7)	7999.0 (7252.9–8764.2)	8438.2 (7271.5–9550.2)
New Mexico	11420.3 (10399.9–12678.9)	10765.4 (9685.7–11962.8)	13151.3 (11327.2–14988.9)	10183.9 (9350.0–11029.5)	9355.8 (8496.8–10236.9)	11621.1 (10056.7–13249.5)
New York	13650.9 (12594.5–14823.4)	9564.1 (8517.3–10684.6)	9517.3 (8171.1–10950.0)	12264.9 (11485.2–13128.7)	8268.4 (7493.8–9093.9)	8336.3 (7263.4–9437.1)
North Carolina	13008.4 (11979.5–14256.5)	11030.9 (9942.6–12203.5)	12262.0 (10615.2–13851.6)	11661.1 (10823.5–12554.6)	9576.3 (8732.0–10426.0)	10820.8 (9505.6–12180.6)
North Dakota	9829.1 (8858.8–10928.9)	8893.6 (7887.0–10004.5)	8837.8 (7486.6–10100.4)	8745.4 (8001.3–9488.1)	7708.2 (6992.6–8529.4)	7714.3 (6683.6–8727.0)
Ohio	12585.9 (11567.7–13811.3)	11646.0 (10481.3–12836.2)	13525.1 (11782.2–15498.0)	11153.6 (10347.6–12010.4)	10116.5 (9265.0–11019.2)	12020.1 (10498.1–13619.9)
Oklahoma	12490.6 (11426.4–13744.7)	13179.5 (11959.2–14502.9)	13971.1 (12102.2–16034.2)	11127.9 (10330.1–12054.5)	11502.7 (10578.3–12521.7)	12362.6 (10854.5–14149.9)
Oregon	10749.8 (9797.0–11896.7)	9161.5 (8154.5–10243.8)	9709.5 (8337.1–11323.7)	9553.2 (8781.8–10406.1)	7857.3 (7134.5–8615.9)	8448.5 (7288.5–9815.6)
Pennsylvania	12440.1 (11400.3–13668.0)	10933.5 (9814.8–12143.4)	11970.3 (10306.7–13808.0)	11011.0 (10204.8–11876.2)	9477.0 (8630.4–10401.9)	10593.1 (9173.8–12129.7)

Rhode Island	11740.7 (10686.6–12885.2)	10059.8 (9017.0–11278.3)	10091.2 (8591.4–11669.8)	10408.8 (9554.0–11324.2)	8715.1 (7899.5–9591.7)	8869.5 (7645.8–10153.8)
South Carolina	14056.3 (13037.8–15262.2)	12084.1 (10944.5–13353.6)	12911.5 (11126.8–14724.8)	12652.1 (11834.2–13572.3)	10538.2 (9633.6–11491.0)	11401.1 (9964.9–12997.7)
South Dakota	10292.7 (9334.0–11456.9)	9074.8 (8069.8–10191.8)	9717.3 (8445.4–11273.3)	9178.8 (8415.1–9935.7)	7858.7 (7120.3–8675.3)	8463.0 (7425.6–9664.9)
Tennessee	13467.8 (12406.3–14670.9)	12694.9 (11540.8–13986.0)	14203.4 (12312.3–16067.4)	12069.1 (11222.2–12954.9)	11102.9 (10191.9–12105.5)	12614.5 (11019.9–14107.0)
Texas	12330.0 (11386.7–13484.9)	10236.5 (9204.8–11417.9)	10497.1 (8954.2–12024.7)	11001.6 (10263.0–11824.2)	8775.9 (7974.1–9621.4)	9085.2 (7952.5–10279.7)
Utah	9117.2 (8194.4–10192.4)	8582.8 (7598.1–9753.9)	9393.4 (8055.3–10798.6)	7995.7 (7284.7–8805.8)	7308.4 (6597.1–8117.0)	8110.6 (7070.8–9230.9)
Vermont	10634.0 (9641.6–11767.6)	8722.3 (7682.1–9852.8)	9605.6 (8315.6–10948.1)	9442.2 (8664.7–10250.1)	7547.8 (6757.1–8430.3)	8460.5 (7403.8–9523.9)
Virginia	12311.7 (11315.8–13480.6)	9923.0 (8903.0–11118.0)	10465.9 (9039.1–12014.8)	10940.4 (10168.8–11754.9)	8544.8 (7771.2–9397.7)	9128.2 (7941.4–10371.9)
Washington	10677.5 (9743.3–11748.9)	8874.9 (7870.9–9981.9)	9358.9 (7914.7–10791.0)	9505.4 (8755.0–10345.5)	7623.6 (6907.4–8442.5)	8141.5 (7034.8–9299.4)
West Virginia	13299.2 (12122.4–14528.6)	13771.3 (12499.9–15146.2)	16567.5 (14369.2–18762.4)	11880.5 (11000.7–12796.8)	12113.5 (11131.0–13182.4)	14849.0 (12993.2–16618.8)
Wisconsin	10867.3 (9807.9–12055.1)	9545.3 (8466.0–10779.4)	10396.6 (8929.2–12049.8)	9610.6 (8777.0–10478.5)	8271.1 (7432.4–9150.2)	9151.7 (7934.6–10530.6)
Wyoming	10793.7 (9821.4–12020.4)	9914.6 (8839.2–11029.8)	10669.9 (9215.8–12204.9)	9672.7 (8899.8–10566.7)	8640.4 (7823.0–9507.3)	9372.1 (8106.9–10572.2)

Table S7. GATHER checklist

Item #	Checklist item	Reporting location
**Objectives and funding**		
**1**	Define the indicator(s), populations (including age, sex, and geographic entities), and time period(s) for which estimates were made.	Main text methods
**2**	List the funding sources for the work.	Main text (acknowledgement section); primary funder given in the "funding" section of the summary
**Data Inputs**		
For all data inputs from multiple sources that are synthesized as part of the study:		
**3**	Describe how the data were identified and how the data were accessed.	Main text methods, with references provided for access to full details
**4**	Specify the inclusion and exclusion criteria. Identify all ad-hoc exclusions.	References provided in the main text methods for access to full details
**5**	Provide information on all included data sources and their main characteristics. For each data source used, report reference information or contact name/institution, population represented, data collection method, year(s) of data collection, sex and age range, diagnostic criteria or measurement method, and sample size, as relevant.	Overview in main text methods; citations for all references available at http://ghdx.healthdata.org/gbd-2021/sources
**6**	Identify and describe any categories of input data that have potentially important biases (e.g., based on characteristics listed in item 5).	Main text methods, with references provided for access to full details
For data inputs that contribute to the analysis but were not synthesized as part of the study:		
**7**	Describe and give sources for any other data inputs.	N/A
For all data inputs:		
**8**	Provide all data inputs in a file format from which data can be efficiently extracted (e.g., a spreadsheet rather than a PDF), including all relevant meta-data listed in item 5. For any data inputs that cannot be shared because of ethical or legal reasons, such as third-party ownership, provide a contact name or the name of the institution that retains the right to the data.	http://ghdx.healthdata.org/gbd-2021/sources Availability of data is dependent on data use agreements; contact information provided when not directly available.
**Data analysis**		
**9**	Provide a conceptual overview of the data analysis method. A diagram may be helpful.	Main text methods, with references provided for access to full details
**10**	Provide a detailed description of all steps of the analysis, including mathematical formulae. This description should cover, as relevant, data cleaning, data pre-processing, data adjustments and weighting of data sources, and mathematical or statistical model(s).	Main text methods, with references provided for access to full details
**11**	Describe how candidate models were evaluated and how the final model(s) were selected.	References provided in the main text methods for access to full details
**12**	Provide the results of an evaluation of model performance, if done, as well as the results of any relevant sensitivity analysis.	N/A
**13**	Describe methods for calculating uncertainty of the estimates. State which sources of uncertainty were, and were not, accounted for in the uncertainty analysis.	Main text methods
**14**	State how analytic or statistical source code used to generate estimates can be accessed.	http://ghdx.healthdata.org/gbd-2021/code
**Results and Discussion**		
**15**	Provide published estimates in a file format from which data can be efficiently extracted.	All estimates are available for download at https://vizhub.healthdata.org/gbd-results/ and https://vizhub.healthdata.org/gbd-compare/
**16**	Report a quantitative measure of the uncertainty of the estimates (e.g. uncertainty intervals).	UIs given in all results, including in figures (as appropriate) and tables
**17**	Interpret results in light of existing evidence. If updating a previous set of estimates, describe the reasons for changes in estimates.	Main text discussion
**18**	Discuss limitations of the estimates. Include a discussion of any modelling assumptions or data limitations that affect interpretation of the estimates.	Main text limitations section of discussion
