# Supplementary material for: The burden of diseases, injuries, and risk factors by state in the USA, 1990–2021: a systematic analysis for the Global Burden of Disease Study 2021
Source: Lancet. 2024 Dec 7;404(10469):2314–40. doi: 10.1016/S0140-6736(24)01446-6 (PMC11694014; doi:10.1016/S0140-6736(24)01446-6)
Supplement: Supplementary appendix 2 [file mmc2.pdf]

THE LANCET

Supplementary appendix 2

This appendix formed part of the original submission and has been peer reviewed. We post it as supplied by the authors.

Supplement to: GBD 2021 US Burden of Disease Collaborators. The burden of diseases, injuries, and risk factors by state in the USA, 1990–2021: a systematic analysis for the Global Burden of Disease Study 2021. *Lancet* 2024; **404:** 2334–40.

Appendix 2: authorship information for "The burden of diseases, injuries, and risk factors by state in the USA, 1990–2021: a systematic analysis for the Global Burden of Disease Study 2021"

This appendix provides further authorship details for "The burden of diseases, injuries, and risk factors by state in the USA, 1990–2021: a systematic analysis for the Global Burden of Disease Study 2021"

Table of Contents

GBD 2021 US Burden of Disease Collaborators 3

Affiliations 5

Authors' Contributions 20

Managing the overall research enterprise 20

Writing the first draft of the manuscript 20

Primary responsibility for applying analytical methods to produce estimates 20

Primary responsibility for seeking, cataloguing, extracting, or cleaning data; designing or coding figures and tables 20

Providing data or critical feedback on data sources 20

Developing methods or computational machinery 21

Providing critical feedback on methods or results 21

Drafting the work or revising it critically for important intellectual content 23

Managing the estimation or publications process 25

GBD 2021 US Burden of Disease Collaborators

Ali H Mokdad*, Catherine Bisignano, Johnathan M Hsu, Hazim S Ababneh, Rouzbeh Abbasgholizadeh, Atef Abdelkader, Michael Abdelmasseh, Olugbenga Olusola Abiodun, Richard Gyan Aboagye, Ahmed Abu-Zaid, Hana J Abukhadijah, Isaac Yeboah Addo, Oluwafemi Atanda Adeagbo, Oyelola A Adegboye, Victor Adekanmbi, Temitayo Esther Adeyeoluwa, Leticia Akua Adzigbli, Aanuoluwapo Adeyimika Afolabi, Williams Agyemang-Duah, Shahzaib Ahmad, Danish Ahmad, Ayman Ahmed, Syed Anees Ahmed, Mohammed Ahmed Akkaif, Ashley E Akrami, Ema Akter, Syed Mahfuz Al Hasan, Omar Al Ta'ani, Yazan Al-Ajlouni, Ziyad Al-Aly, Rami Hani Al-Rifai, Jaffar A Al-Tawfiq, Mohammad Al-Wardat, Walid Adnan Al-Zyoud, Manjurul Alam, Almaza Albakri, Wafa A Aldhaleei, Robert W Aldridge, Mohammed Usman Ali, Abid Ali, Rafat Ali, Waad Ali, Sami Almustanyir, Ahmed Yaseen Alqutaibi, Ahmad Alrawashdeh, Mohammed A Alsabri, Hany Aly, Reza Amani, Prince M Amegbor, Alireza Amindarolzarbi, Sohrab Amiri, Abhishek Anil, Francis Appiah, Jalal Arabloo, Elshaimaa A Arafa, Mosab Arafat, Aleksandr Y Aravkin, Ali Ardekani, Demelash Areda, Sait Ashina, Alok Atreya, Fekadu Belay Ayalew, Ahmed Y Azzam, Giridhara Rathnaiah Babu, Soroush Baghdadi, Sara Bagherieh, Saeed Bahramian, Razieh Bahreini, Abdulaziz T Bako, Kannu Bansal, Till Winfried Bärnighausen, Amadou Barrow, Mohammad-Mahdi Bastan, Sanjay Basu, Ravi Batra, Kavita Batra, Mohsen Bayati, Maryam Beiranvand, Michelle L Bell, Apostolos Beloukas, Maryam Bemanalizadeh, Fiona B Bennitt, Habib Benzian, Azizullah Beran, Amiel Nazer C Bermudez, Robert S Bernstein, Habtamu B B Beyene, Kebede A Beyene, Akshaya Srikanth Bhagavathula, Neeraj Bhala, Ashish Bhargava, Sonu Bhaskar, Vivek Bhat, Aadam Olalekan Bodunrin, Sri Harsha Boppana, Hamed Borhany, Samuel Adolf Bosoka, Christopher Boxe, Edward J Boyko, Dejana Braithwaite, Michael Brauer, Dana Bryazka, Raffaele Bugiardini, Yasser Bustanji, Zahid A Butt, Florentino Luciano Caetano dos Santos, Jack Cagney, Chao Cao, Angelo Capodici, Joao Mauricio Castaldelli-Maia, Francieli Cembranel, Edina Cenko, Eeshwar K Chandrasekar, Anis Ahmad Chaudhary, An-Tian Chen, Meng Xuan Chen, Gerald Chi, Bryan Chong, Sonali Gajanan Choudhari, Rajiv Chowdhury, Sheng-Chia Chung, Rebecca M Cogen, Joao Conde, Leslie Trumbull Cooper, Samuele Cortese, Michael H Criqui, Natalia Cruz-Martins, Garland T Culbreth, Mario D'Oria, Bashir Dabo, Zhaoli Dai, Xiaochen Dai, Giovanni Damiani, Farah Daoud, Samuel D D Darcho, Aso Mohammad Darwesh, Saswati Das, Nihar Ranjan Dash, Mohsen Dashti, Louisa Degenhardt, Don C Des Jarlais, Vinoth Gnana Chellaiyan Devanbu, Syed Masudur Rahman Dewan, Kuldeep Dhama, Daniel Diaz, Luis Antonio Diaz, Michael J Diaz, Delaney D Ding, Thao Huynh Phuong Do, Thanh Chi Do, Khanh Duy Doan, Deepa Dongarwar, E. Ray Dorsey, Ojas Prakashbhai Doshi, Rajkumar Prakashbhai Doshi, Abdel Douiri, Robert Kokou Dowou, John Dube, Siddhartha Dutta, Laura Dwyer-Lindgren, Arkadiusz Marian Dziedzic, Abdel Rahman E'mar, Alireza Ebrahimi, Joshua R R Ehrlich, Temitope Cyrus Ekundayo, Rabie Adel El Arab, Ibrahim Farahat El Bayoumy, Muhammed Elhadi, Adel B Elmoselhi, Gihan ELNahas, Mohammed Elshaer, Chadi Eltaha, Mehdi Emamverdi, Francesco Esposito, Farshid Etaee, Elochukwu Fortune Ezenwankwo, Ayesha Fahim, Aliasghar Fakhri-Demeshghieh, Abidemi Omolara Fasanmi, Timur Fazylov, Valery L Feigin, Ginenus Fekadu, Abdullah Hamid Feroze, Nuno Ferreira, Irina Filip, Florian Fischer, Luisa S Flor, Weijia Fu, Takeshi Fukumoto, Muktar A Gadanya, Avi A Gajjar, Balasankar Ganesan, Mohammad Arfat Ganiyani, Xiang Gao, William M Gardner, Miglas Welay Gebregergis, Mesfin Gebrehiwot, Teferi Gebru Gebremeskel, Delaram J Ghadimi, Afsaneh Ghasemzadeh, Ali Gholamrezanezhad, Elena Ghotbi, Laszlo Göbölös, Mohamad Goldust, Mahaveer Golechha, Davide Golinelli, Ayman Grada, Avirup Guha, Stefano Guicciardi, Ishita Gupta, Veer Bala Gupta, Vivek Kumar Gupta, Annie Haakenstad, Parham Habibzadeh, Nils Haep, Demewoz Haile, Arvin Haj-Mirzaian, Aram Halimi, Erin B Hamilton, Obaid I Haque, Ahmed I Hasaballah, Md. Kamrul Hasan, Md Saquib Hasnain, Abbas M Hassan, Rasmus J Havmoeller, Simon I Hay, Jeffrey J Hebert, Zohreh Heidary, Mehdi Hemmati, Irma Hidayana, Thomas Kwadwo Hinneh, Yuta Hiraike, Nguyen Quoc Hoan, Nobuyuki Horita, Md. Belal Hossain, Md Mahbub Hossain, Mehdi

Hosseinzadeh, Sorin Hostiuc, Chengxi Hu, Junjie Huang, Tsegaye Gebreyes Hundie, Kiavash Hushmandi, Hong-Han Huynh, Kevin S Ikuta, Sheikh Mohammed Shariful Islam, Md. Rabiul Islam, Louis Jacob, Kathryn H Jacobsen, Akhil Jain, Ammar Abdulrahman Jairoun, Mihajlo Jakovljevic, Elham Jamshidi, Tahereh Javaheri, Bijay Mukesh Jeswani, Angeline Jeyakumar, Emily Katherine Johnson, Kehinde Kazeem Kanmodi, Rami S Kantar, Shama D Karanth, Ibraheem M Karaye, Nicholas J Kassebaum, Adarsh Katamreddy, Foad Kazemi, Jessica A Kerr, Yousef Saleh Khader, Faham Khamesipour, Mohammad Jobair Khan, Zeeshan Ali Khan, Fayaz Khan, Ajmal Khan, Khaled Khatab, Fatemeh Khatami, Moawiah Mohammad Khatatbeh, Moein Khormali, Atulya Aman Khosla, Sepehr Khosravi, Majid Khosravi, Jagdish Khubchandani, Grace Kim, Min Seo Kim, Ruth W Kimokoti, Adnan Kisa, Sonali Kochhar, Gerbrand Koren, Vijay Krishnamoorthy, Connor M Kubeisy, Md Abdul Kuddus, Mukhtar Kulimbet, Vishnutheertha Kulkarni, Vijay Kumar, Ashish Kumar, Rakesh Kumar, Satyajit Kundu, Om P Kurmi, Evans F Kyei, Hanpeng Lai, Qing Lan, Van Charles Lansingh, Trang Diep Thanh Le, Huu-Hoai Le, Nhi Huu Hanh Le, Thao Thi Thu Le, Janet L Leasher, Seung Won Lee, Wei-Chen Lee, Wei Li, Massimo Libra, Stephen S Lim, Jialing Lin, John C Lin, Vasileios-Arsenios Lioutas, Xuefeng Liu, Richard T Liu, Xiaofeng Liu, Jie Liu, José Francisco López-Gil, Platon D Lopukhov, Giancarlo Lucchetti, Raimundas Lunevicius, Jay B Lusk, Asma Mafhoumi, Elaheh Malakan Rad, Yosef Manla, Vahid Mansouri, Emmanuel Manu, Agustina M Marconi, Mirko Marino, Randall V Martin, Ramon Martinez-Piedra, Wolfgang Marx, Roy Rillera Marzo, Yasith Mathangasinghe, Fernanda Penido Matozinhos, Steven M McPhail, Tesfahun Mekene Meto, Hadush Negash Meles, Endalkachew Belayneh Melese, George A Mensah, Laverne G Mensah, Sultan Ayoub Meo, Tomislav Mestrovic, Laurette Mhlanga, Adquate Mhlanga, Irmina Maria Michalek, Ted R Miller, Mohammad Mirza-Aghazadeh-Attari, Ajay Kumar Mishra, Madeline E Moberg, Nouh Saad Mohamed, Mouhand F H Mohamed, Jama Mohamed, Ibrahim Mohammadzadeh, Salahuddin Mohammed, Shafiu Mohammed, Hossein Molavi Vardanjani, Sara Momtazmanesh, Mohammad Ali Moni, Fateme Montazeri, Yousef Moradi, Maziar Moradi-Lakeh, Paula Moraga, Shane Douglas Morrison, Rohith Motappa, Vincent Mougin, Efren Murillo-Zamora, Mohsen Naghavi, Pirouz Naghavi, Gurudatta Naik, Soroush Najdaghi, Dhairya P Nanavaty, Delaram Narimani Davani, Gustavo G Nascimento, Abdulqadir J Nashwan, Zuhair S Natto, Sabina Onyinye Nduaguba, Henok Biresaw Netsere, Ahmadreza Nezameslami, Hau Thi Hien Nguyen, Tuan Thanh Nguyen, Dang H Nguyen, Hien Quang Nguyen, Anh Hoang Nguyen, Van Thanh Nguyen, Yeshambel T Nigatu, Nasrin Nikravangolsefid, Syed Toukir Ahmed Noor, Amanda Novotney, Fred Nugen, Jerry John Nutor, Ogochukwu Janet Nzoputam, Bogdan Oancea, Michael Safo Oduro, Oluwaseun Adeolu Ogundijo, Sylvester Reuben Okeke, Osaretin Christabel Okonji, Andrew T Olagunju, Abdulhakeem Abayomi Olorukooba, Isaac Iyinoluwa Olufadewa, Hany A Omar, Kenneth Ikenna Onyedibe, Abdulahi Opejin, Doris V Ortega-Altamirano, Samuel M Ostroff, Mahesh Padukudru P A, Sujogya Kumar Panda, Romil R Parikh, Sungchul Park, Eun-Kee Park, Seoyeon Park, Ava Pashaei, Maja Pasovic, Jenil R. Patel, Shankargouda Patil, Shrikant Pawar, Emmanuel K Peprah, Gavin Pereira, Richard G Pestell, Hoang Tran Pham, Anil K. Philip, Michael R Phillips, Manon Pigeolet, Maarten J Postma, Ghazaleh Pourali, Reza Pourbabaki, Disha Prabhu, Pranil Man Singh Pradhan, Jalandhar Pradhan, Jagadeesh Puvvula, Mehrdad Rabiee Rad, Amir Radfar, Quinn Rafferty, Vafa Rahimi-Movaghar, Muhammad Aziz Rahman, Mohammad Rahmanian, Majed Ramadan, Shakthi Kumaran Ramasamy, Sheena Ramazanu, Amey Rane, Ahmed Mustafa Rashid, Reza Rawassizadeh, Elrashdy Moustafa Mohamed Redwan, Robert C Reiner Jr., Taeho Gregory Rhee, Jennifer Rickard, Monica Rodrigues, Jefferson Antonio Buendia Rodriguez, Himanshu Sekhar Rout, Tilleye Runghien, Aly M A Saad, Cameron John Sabet, Siamak Sabour, Umar Saeed, Mehdi Safari, Dominic Sagoe, Md Refat Uz Zaman Sajib, Giovanni A Salum, Vijaya Paul Samuel, Abdallah M Samy, Aswini Saravanan, Babak Saravi, Nikolaos Scarmeas, Markus P Schlaich, Art Schuermans, Austin E Schumacher, David C Schwebel, Allen Seylani, Mahan Shafie, Nilay S Shah, Ataollah Shahbandi, Ahmed Shaikh, Masood Ali Shaikh,

Husain Shakil, Muhammad Aaqib Shamim, Mohammad Ali Shamshirgaran, Medha Sharath, Amin Sharifan, Manoj Sharma, Pavanchand H Shetty, Premalatha K Shetty, Peilin Shi, Aminu Shittu, Velizar Shivarov, Sina Shool, Kerem Shuval, Emmanuel Edwar Siddig, Surjit Singh, Sarah Brooke Sirota, David A Sleet, Ranjan Solanki, Shipra Solanki, Sameh S M Soliman, Yi Song, Lauryn K Stafford, Abida Sultana, Jing Sun, Chandan Kumar Swain, Lukasz Szarpak, Mindy D Szeto, Seyyed Mohammad Tabatabaei, Celine Tabche, Jabeen Taiba, Nathan Y Tat, Mohamad-Hani Temsah, Masayuki Teramoto, James Douglas Thornton, Marcos Roberto Tovani-Palone, Khai Hoan Tram, Thang Huu Tran, Jasmine T Tran, Ngoc Ha Tran, Samuel Joseph Tromans, Thien Tan Tri Tai Truyen, Munkhtuya Tumurkhuu, Stefanos Tyrovolas, Arit Udoh, Sana Ullah, Saeed Ullah, Atta Ullah, Sanaz Vahdati, Asokan Govindaraj Vaithinathan, Omid Vakili, Jef Van den Eynde, Aaron van Donkelaar, Dominique Vervoort, Manish Vinayak, Avina Vongpradith, Theo Vos, Muhammad Waqas, Kosala Gayan Weerakoon, Ronny Westerman, Caroline Wilkerson, Chenkai Wu, Felicia Wu, Suowen Xu, Lin Yang, Danting Yang, Yuichiro Yano, Metin Yesiltepe, Dong Keon Yon, Mustafa Z Younis, Chuanhua Yu, Siddhesh Zadey, Michael Zastrozhin, Mohammed G M Zeariya, Haijun Zhang, Zhiqiang Zhang, Meixin Zhang, Claire Chenwen Zhong, Bin Zhu, Abzal Zhumagaliuly, Hafsa Zia, Makan Ziafati, Magdalena Zielińska, Sa'ed H Zyoud, and Christopher J L Murray**

*First Author
**Senior Author

Affiliations

Institute for Health Metrics and Evaluation (Prof A H Mokdad PhD, C Bisignano MPH, J M Hsu BA, R W Aldridge PhD, A Y Aravkin PhD, Prof M Brauer DSc, D Bryazka BA, J Cagney MSc, R M Cogen BA, G T Culbreth PhD, X Dai PhD, F Daoud BS, Prof L Degenhardt PhD, L Dwyer-Lindgren PhD, Prof V L Feigin PhD, L S Flor MPH, W Fu MS, W M Gardner MPH, A Haakenstad ScD, D Haile PhD, E B Hamilton MPH, Prof S I Hay FMedSci, K S Ikuta MD, N J Kassebaum MD, Prof S S Lim PhD, T Mestrovic PhD, M E Moberg MS, V Mougin BA, Prof M Naghavi PhD, A Novotney MPH, S M Ostroff PhD, M Pasovic Med, Q Rafferty BA, R C Reiner Jr. PhD, T Runghien MSc, A E Schumacher PhD, S B Sirota MA, L K Stafford MS, A Vongpradith BA, Prof T Vos PhD, C Wilkerson MPH, M Zhang MS, Prof C J L Murray DPhil), Department of Health Metrics Sciences, School of Medicine (Prof A H Mokdad PhD, R W Aldridge PhD, A Y Aravkin PhD, X Dai PhD, L Dwyer-Lindgren PhD, L S Flor MPH, Prof S I Hay FMedSci, N J Kassebaum MD, Prof S S Lim PhD, Prof M Naghavi PhD, R C Reiner Jr. PhD, Prof T Vos PhD, Prof C J L Murray DPhil), Department of Applied Mathematics (A Y Aravkin PhD), School of Medicine (E J Boyko MD), Department of Anesthesiology & Pain Medicine (N J Kassebaum MD, V Krishnamoorthy MD), Department of Global Health (S Kochhar MD), Division of Plastic and Reconstructive Surgery (S D Morrison MD), Henry M Jackson School of International Studies (S M Ostroff PhD), Division of Allergy and Infectious Diseases, Department of Medicine (K Tram MD), Department of Epidemiology (H Zia BDS), University of Washington, Seattle, WA, USA; Department of Radiation Oncology (H S Ababneh MD), Department of Orthopaedic Surgery (A Ebrahimi MD), Department of Radiology (A Haj-Mirzaian MD, X Liu PhD), Department of Psychiatry (R T Liu PhD), Division of Cardiology (D H Nguyen BS), Cardiovascular Research Center (A Schuermans BSc), Massachusetts General Hospital, Boston, MA, USA (M Kim MD); Doheny Eye Institute (R Abbasgholizadeh MD), Department of Ophthalmology (M Emamverdi MD), University of California Los Angeles, Los Angeles, CA, USA; Department of Mathematics and Sciences (A Abdelkader PhD), Department of Clinical Sciences (Prof E A Arafa PhD), Ajman University, Ajman, United Arab Emirates; Department of Surgery (M Abdelmasseh MD), Marshall University, Huntington, WV, USA; Department of Internal Medicine (O O Abiodun FWACP), Federal Medical Centre, Abuja, Nigeria; Department of Family and Community Health (R G Aboagye MPH), Department of Epidemiology and Biostatistics (L A

Adzigbli BSc, S A Bosoka MPhil, R K Dowou MPhil), Department of Population and Behavioural Sciences (E Manu PhD), University of Health and Allied Sciences, Ho, Ghana; Department of Biochemistry and Molecular Medicine (A Abu-Zaid PhD), College of Medicine (S Almustanyir MD), Alfaisal University, Riyadh, Saudi Arabia; College of Graduate Health Sciences (A Abu-Zaid PhD), University of Tennessee, Memphis, TN, USA; Academic Health System (H J Abukhadijah MPH), Nursing and Midwifery Research Department (NMRD) (A J Nashwan PhD), Hamad Medical Corporation, Doha, Qatar; Centre for Social Research in Health (I Y Addo PhD, S R Okeke PhD), School of Population Health (Z Dai PhD, J Lin PhD), National Drug and Alcohol Research Centre (Prof L Degenhardt PhD), University of New South Wales, Sydney, NSW, Australia; Quality and Systems Performance Unit (I Y Addo PhD), Cancer Institute NSW, Sydney, NSW, Australia; Department of Health Promotion, Education and Behavior (O A Adeagbo PhD), University of South Carolina, Columbia, SC, USA; Department of Public Health (O A Adeagbo PhD), University of KwaZulu-Natal, Durban, South Africa; Menzies School of Health Research (Prof O A Adegboye PhD), Charles Darwin University, Darwin, NT, Australia; Department of Obstetrics and Gynecology (V Adekanmbi PhD), The Department of Family Medicine (W Lee PhD), University of Texas Medical Branch, Galveston, TX, USA; Department of Pharmacology and Therapeutics (T E Adeyeoluwa PhD), Department of Microbiology (T C Ekundayo PhD), University of Medical Sciences, Ondo, Ondo, Nigeria; Department of Veterinary Medicine (T E Adeyeoluwa PhD), Department of Veterinary Public Health and Preventive Medicine (O A Ogundijo MSc), Faculty of Public Health (I I Olufadewa MHS), University of Ibadan, Ibadan, Nigeria; Technical Services Directorate (A A Afolabi MPH), MSI Nigeria Reproductive Choices, Abuja, Nigeria; Department of Geography and Planning (W Agyemang-Duah PhD), Queen's University, Kingston, ON, Canada; Department of Medical Oncology (S Ahmad MD, A A Khosla MD), Department of Medicine (M Ganiyani MD), Miami Cancer Institute, Miami, FL, USA; Department of Community Medicine and Preventive Health (S Ahmad MD), King Edward Medical University Lahore, Lahore, Pakistan; School of Medicine and Psychology (D Ahmad PhD), Australian National University, Canberra, ACT, Australia; Public Health Foundation of India, Gandhinagar, India (D Ahmad PhD); Institute of Endemic Diseases (A Ahmed MSc), Unit of Basic Medical Sciences (E E Siddig MD), University of Khartoum, Khartoum, Sudan; Swiss Tropical and Public Health Institute (A Ahmed MSc), University of Basel, Basel, Switzerland; Brody School of Medicine (S Ahmed PhD), Department of Computer Science (A O Bodunrin MSc), Department of Geography (A Opejin MSc), Department of Physiology (M Tumurkhuu PhD), East Carolina University, Greenville, NC, USA; Department of Cardiology (M Akkaif PhD), Fudan University, Shanghai, China; Chicago College of Osteopathic Medicine (A E Akrami BS), Midwestern University, Downers Grove, IL, USA; Feinberg School of Medicine (A E Akrami BS), Department of Medicine (N S Shah MD), Northwestern University, Chicago, IL, USA (L Mhlanga PhD, M D Szeto MS); Maternal and Child Health Division (E Akter MSc, S Noor MS), International Centre for Diarrhoeal Disease Research, Bangladesh, Dhaka, Bangladesh; Department of Surgery (S Al Hasan PhD, G Pourali MD), Department of Research and Development (Z Al-Aly MD), Department of Medicine (J Liu MPH), Department of Energy, Environmental, and Chemical Engineering (Prof R V Martin PhD, A van Donkelaar PhD), Washington University in St. Louis, St. Louis, MO, USA; Department of Internal Medicine (O Al Ta'ani MD), Allegheny Health Network, Pittsburgh, PA, USA; School of Medicine (Y Al-Ajlouni MD), New York Medical College, Valhalla, NY, USA; Department of Epidemiology (Y Al-Ajlouni MD), Departments of Psychiatry and Epidemiology (Prof M R Phillips MD), Department of Neurology (Prof N Scarmeas PhD), Columbia University, New York, NY, USA; Clinical Epidemiology Center (Z Al-Aly MD), US Department of Veterans Affairs (VA), St. Louis, MO, USA; Institute of Public Health (R H Al-Rifai PhD), United Arab Emirates University, Al Ain, United Arab Emirates; Department of Specialty Internal Medicine (Prof J A Al-Tawfiq MD), Johns Hopkins Aramco Healthcare, Dhahran, Saudi Arabia; Department of Medicine (Prof J A Al-Tawfiq MD), Indiana University School of

Medicine, Indianapolis, IN, USA; Department of Rehabilitation Sciences (M Al-Wardat PhD), Department of Allied Medical Sciences (A Alrawashdeh PhD), Department of Public Health (Prof Y S Khader PhD), Jordan University of Science and Technology, Irbid, Jordan; Department of Biomedical Engineering (W A Al-Zyoud PhD), German Jordanian University, Amman, Jordan; Department of Bioengineering (M Alam PhD), Department of Nutrition and Food Studies (S Tyrovolas PhD), George Mason University, Fairfax, VA, USA; Department of Medicine (A Albakri MD), Royal Jordanian Medical Services, Amman, Jordan; Division of Gastroenterology and Hepatology (W A Aldhaleei MD), Department of Cardiovascular Medicine (L T Cooper MD), Mayo Clinic, Jacksonville, FL, USA; Institute of Health Informatics (R W Aldridge PhD), Department of Health Informatics (S Chung PhD), University College London, London, United Kingdom; Department of Medical Rehabilitation (Physiotherapy) (M U Ali MSc), University of Maiduguri, Maiduguri, Nigeria; Department of Rehabilitation Sciences (M U Ali MSc, M Khan MPH), School of Nursing (S Tyrovolas PhD), Hong Kong Polytechnic University, Hong Kong, China; Department of Zoology (A Ali PhD), Abdul Wali Khan University Mardan, Mardan, Pakistan; Department of Biosciences (R Ali MPhil), Jamia Millia Islamia, New Delhi, India; Department of Geography (W Ali PhD), Sultan Qaboos University, Muscat, Oman; Ministry of Health, Riyadh, Saudi Arabia (S Almustanyir MD); Department of Prosthodontics and Implant Dentistry (A Alqutaibi PhD), Taibah University, Medinah, Saudi Arabia; Department of Prosthodontics and Implant Dentistry (A Alqutaibi PhD), Ibb University, Ibb, Yemen; Department of Emergency Medicine (M A Alsabri MD), Sana'a University, Sana’a, Yemen; Pediatric Emergency Medicine Department (M A Alsabri MD), St. Christopher’s Hospital for Children, Philadelphia, PA, USA; Department of Pediatrics (Prof H Aly MD, A E’mar MD), Lerner Research Institute (X Liu PhD), Cleveland Clinic, Cleveland, OH, USA; Interdisciplinary Graduate Program in Human Toxicology (R Amani DVM), University of Iowa, Iowa City, IA, USA; Health Policy Research Center (R Amani DVM, A Ardekani MD), Health Human Resources Research Center (M Bayati PhD), Department of Biostatistics (H Molavi Vardanjani PhD), Department of Occupational Health and Safety Engineering (R Pourbabaki PhD), Shiraz University of Medical Sciences, Shiraz, Iran; School of Global Public Health (P M Amegbor PhD, E K Peprah PhD), Department of Epidemiology and Health Promotion (Prof H Benzian PhD), Department of Child and Adolescent Psychiatry (Prof S Cortese PhD), New York University, New York, NY, USA; Department of Radiology and Radiological Science (A Amindarolzarbi MD), Department of Anesthesia and Critical Care Medicine (S Boppana MD), School of Nursing (T K Hinneh MSc), Department of Epidemiology (T G Hundie MD), Department of Neurosurgery (F Kazemi MD), Department of Health Policy and Management (D Vervoort MD), Department of International Health (H Zhang MS), Johns Hopkins University, Baltimore, MD, USA (E Jamshidi PharmD, E Melese MD); Medicine, Quran and Hadith Research Center (S Amiri PhD), Nephrology and Urology Research Center (K Hushmandi PhD), Baqiyatallah University of Medical Sciences, Tehran, Iran; Department of Pharmacology (A Anil MD, M Shamim MBBS, S Singh MD), Department of Pharmacology and Research (A Saravanan MD), All India Institute of Medical Sciences, Jodhpur, India; All India Institute of Medical Sciences, Bhubaneswar, India (A Anil MD); Department of Social Sciences (F Appiah MPhil), Berekum College of Education, Berekum, Ghana; School of Public Health (F Appiah MPhil), Kwame Nkrumah University of Science and Technology, Kumasi, Ghana; Health Management and Economics Research Center (J Arabloo PhD), School of Medicine (M Bastan MD), Department of Health Economics (M Khosravi PhD), Gastrointestinal and Liver Diseases Research Center (Prof M Moradi-Lakeh MD), Preventive Medicine and Public Health Research Center (Prof M Moradi-Lakeh MD), Department of Ophthalmology (M Ziafati MD), Iran University of Medical Sciences, Tehran, Iran; Department of Pharmacology and Toxicology (Prof E A Arafa PhD, Prof H A Omar PhD), Beni-Suef University, Beni-Suef, Egypt; College of Pharmacy (M Arafat PhD), Al Ain University, Abu Dhabi, United Arab Emirates; College of Art and Science (D Areda PhD), Ottawa University, Surprise, AZ, USA; School of Life Sciences (D Areda PhD),

Arizona State University, Tempe, AZ, USA; Department of Anesthesia, Critical Care and Pain Medicine (S Ashina MD), T.H. Chan School of Public Health (Prof T W Bärnighausen MD, P M S Pradhan MD), Center for Primary Care (S Basu PhD), Harvard Business School (F Caetano dos Santos PhD), Division of Cardiovascular Medicine (G Chi MD), Department of Neurological Surgery (A H Feroze MD), Department of Health Policy and Management (C M Kubeisy BA), Department of Health Policy and Oral Epidemiology (Z S Natto DrPH), Department of Global Health and Social Medicine (M Pigeolet MD), Harvard University, Boston, MA, USA; Department of Clinical Medicine (S Ashina MD), University of Copenhagen, Copenhagen, Denmark; Department of Forensic Medicine (A Atreya MD), Lumbini Medical College, Palpa, Nepal; Bloomberg School of Public Health (F B Ayalew MD), Johns Hopkins University, Baltimore, MA, USA; Montefiore-Einstein Cerebrovascular Research Lab (A Azzam MBBCh), Albert Einstein College of Medicine, Bronx, NY, USA; Faculty of Medicine (A Azzam MBBCh), October 6 University, 6th of October City, Egypt; Department of Population Medicine (Prof G Babu PhD), Qatar University, Doha, Qatar; Division of Orthopaedics (S Baghdadi MD), Children's Hospital of Philadelphia, Philadelphia, PA, USA; School of Medicine (S Bagherieh BSc, S Bahramian MD, M Rabiee Rad MD), Department of Pediatrics (M Bemanalizadeh MD), Heart Failure Research Center (S Najdaghi MD, D Narimani Davani MD), Neuroscience Research Center (S Najdaghi MD), Department of Clinical Biochemistry (O Vakili PhD), Isfahan University of Medical Sciences, Isfahan, Iran; College of Optometry (R Bahreini MS), Pacific University, Forest Grove, OR, USA; Department of Neurosurgery (A T Bako PhD), Houston Methodist Hospital, Houston, TX, USA (M Elhadi MD); Department of Medicine (K Bansal MD), University of Massachusetts Medical School, Worcester, MA, USA; Department of Medicine (K Bansal MD), Saint Vincent Hospital, Worcester, MA, USA; Heidelberg Institute of Global Health (HIGH) (Prof T W Bärnighausen MD), Heidelberg University, Heidelberg, Germany; Department of Public and Environmental Health (A Barrow MPH), University of The Gambia, Banjul, The Gambia; Department of Epidemiology (A Barrow MPH, D Braithwaite PhD, D D Ding BS, D Yang MPH), Division of Pulmonary, Critical Care, and Sleep (M Beiranvand PhD), College of Medicine (M J Diaz BS), UF Health Cancer Center (S D Karanth PhD), University of Florida, Gainesville, FL, USA; Non-communicable Diseases Research Center (M Bastan MD, S Momtazmanesh MD), Department of Pediatric Neurology (M Bemanalizadeh MD), Department of Reproductive Health (Z Heidary PhD), Urology Research Center (Prof F Khatami PhD), Sina Trauma and Surgery Research Center (M Khormali MD, Prof V Rahimi-Movaghar MD, S Shool MD), Orthopedic Department (A Mafhoumi MD), Department of Pediatric Cardiology (Prof E Malakan Rad MD), Digestive Diseases Research Institute (V Mansouri MD), School of Medicine (S Momtazmanesh MD), Department of Neurology (M Shafie MD), Department of Medicine (A Shahbandi MD), Sina Hospital (A Sharifan PharmD), Research Center for Rational Use of Drugs (A Sharifan PharmD), Tehran University of Medical Sciences, Tehran, Iran; School of Public Health (S Basu PhD), Department of Primary Care and Public Health (C Tabche MSc), Imperial College London, London, United Kingdom; School of Public Health (R Batra MS), Department of Medical Education (K Batra PhD), Department of Social and Behavioral Health (Prof M Sharma PhD), University of Nevada Las Vegas, Las Vegas, NV, USA; IT Department (R Batra MS), Coforge, Georgia, GA, USA; School of the Environment (Prof M L Bell PhD), Department of Internal Medicine (F Etaee MD), Department of Dermatology (M Goldust MD), Department of Psychiatry (W Li PhD, T Rhee PhD), Department of Radiology and Biomedical Imaging (X Liu PhD), Department of Genetics (S Pawar PhD), Yale University, New Haven, CT, USA; Department of Biomedical Sciences (Prof A Beloukas PhD), National AIDS Reference Center of Southern Greece (Prof A Beloukas PhD), University of West Attica, Athens, Greece; Department of Civil and Environmental Engineering (F B Bennitt BA), University of Massachusetts Amherst, Amherst, MA, USA; School of Medicine (A Beran MD, J T Tran BS), Indiana University, Indianapolis, IN, USA; Department of Epidemiology and Biostatistics (Prof A C Bermudez MD), University of the Philippines

Manila, Manila, Philippines; Department of Epidemiology (Prof A C Bermudez MD), Department of Internal Medicine (M F H Mohamed MSc), Brown University, Providence, RI, USA; Hubert Department of Global Health (R S Bernstein MD), School of Medicine (A O Fasanmi PhD), Rollins School of Public Health (Prof D A Sleet PhD), Emory University, Atlanta, GA, USA; Department of Global Health (R S Bernstein MD), George Washington University, Washington, DC, USA; Metabolomics Laboratory (H B Beyene PhD), Hypertension and Kidney Disease Laboratory (Prof M P Schlaich MD), Baker Heart and Diabetes Institute, Melbourne, VIC, Australia; Department of Microbiology (H B Beyene PhD), Addis Ababa University, Addis Ababa, Ethiopia; Department of Pharmaceutical and Administrative Sciences (K A Beyene PhD), University of Health Sciences and Pharmacy in St. Louis, St. Louis, MO, USA; School of Pharmacy (K A Beyene PhD), University of Aukland, Auckland, New Zealand; Department of Public Health (A S Bhagavathula PhD), North Dakota State University, Fargo, ND, USA; Institutes of Applied Health Research and Translational Medicine (N Bhala PhD), Queen Elizabeth Hospital Birmingham, Nottingham, United Kingdom; Institute of Applied Health Research (N Bhala PhD), University of Birmingham, Birmingham, United Kingdom; Department of Internal Medicine (A Bhargava MD), Wayne State University, Detroit, MI, USA; Global Health Neurology Lab (S Bhaskar PhD), NSW Brain Clot Bank, Sydney, NSW, Australia; Division of Cerebrovascular Medicine and Neurology (S Bhaskar PhD), National Cerebral and Cardiovascular Center, Suita, Japan; Department of Internal Medicine (V Bhat MBBS), St. John's National Academy of Health Sciences, Bangalore, India; Internal Medicine Department (H Borhany MD), School of Medicine (D J Ghadimi MD), Obstetrics and Gynecology Department (E Ghotbi MD), Obesity Research Center (A Haj-Mirzaian MD), Research Center for Social Determinants of Health (A Halimi MSc), Skull Base Research Center (I Mohammadzadeh MD), Student Research Committee (M Rahmanian MD), Department of Epidemiology (S Sabour PhD), Department of Health (M Safari PhD), Emergency Department (S Shool MD), Shahid Beheshti University of Medical Sciences, Tehran, Iran; Disease Surveillance Department (S A Bosoka MPhil), Ghana Health Service, Ho, Ghana; Department of Earth, Environment, and Equity (C Boxe PhD), Howard University, Washington, DC, USA; General Medicine Service (E J Boyko MD), Department of Veterans Affairs, Seattle, WA, USA; Cancer Population Sciences Program (D Braithwaite PhD), University of Florida Health Cancer Center, Gainesville, FL, USA; School of Population and Public Health (Prof M Brauer DSc, M Hossain MSc), School of Nursing (A Pashaei MSc), University of British Columbia, Vancouver, BC, Canada; Department of Medical and Surgical Sciences (Prof R Bugiardini MD, E Cenko MD), Department of Biomedical and Neuromotor Sciences (A Capodici MD, S Guicciardi MD), Dipartimento di Scienze Biomediche e Neuromotorie (DIBINEM) (F Esposito MD), University of Bologna, Bologna, Italy; School of Pharmacy (Prof Y Bustanji PhD), The University of Jordan, Amman, Jordan; Department of Basic Biomedical Sciences (Prof Y Bustanji PhD), Clinical Sciences Department (N R Dash MD), Basic Medical Sciences Department (A B Elmoselhi PhD), Research Institute of Medical & Health Sciences (A B Elmoselhi PhD), Department of Pharmacy Practice and Pharmacotherapeutics (Prof H A Omar PhD), Department of Medicinal Chemistry (S S M Soliman PhD), University of Sharjah, Sharjah, United Arab Emirates; School of Public Health Sciences (Z A Butt PhD), University of Waterloo, Waterloo, ON, Canada; Al Shifa School of Public Health (Z A Butt PhD), Al Shifa Trust Eye Hospital, Rawalpindi, Pakistan; Dana-Farber Cancer Institute, Boston, MA, USA (C Cao MPH); Department of Management and Healthcare (EMbeDS) (A Capodici MD), Sant'Anna School of Advanced Studies, Pisa, Italy; Department of Psychiatry (Prof J Castaldelli-Maia PhD), University of São Paulo, São Paulo, Brazil; Department of Nutrition (Prof F Cembranel DSc), Federal University of Santa Catarina, Florianópolis, Brazil; Department of Anesthesiology and Perioperative Medicine (E K Chandrasekar MD), School of Medicine (Prof S Xu PhD), University of Rochester, Rochester, NY, USA (E Dorsey MD); Department of Biology (A A Chaudhary PhD), Al-Imam Mohammad Ibn Saud Islamic University, Riyadh, Saudi Arabia; Fuwai Hospital (A Chen PhD), Chinese Academy of Medical Sciences & Peking Union Medical

College, Beijing, China; Department of Computer Science (A Chen PhD), University of Texas at Austin, Austin, TX, USA; Department of Ophthalmology and Visual Sciences (J R R Ehrlich MD), Institute for Social Research (J R R Ehrlich MD), University of Michigan, Ann Arbor, MI, USA (M Chen DDS); Department of Medicine (B Chong MBBS), Saw Swee Hock School of Public Health (S Ramazanu PhD), National University of Singapore, Singapore, Singapore; Department of Community Medicine (Prof S G Choudhari MD), Jawaharlal Nehru Medical College, Wardha, India; Florida International University, Miami, FL, USA (Prof R Chowdhury PhD); Department of Epidemiology (Prof R Chowdhury PhD), University of Bern, Bern, Switzerland; Health Data Research UK, London, United Kingdom (S Chung PhD); Nova Medical School (Prof J Conde PhD), Nova University of Lisbon, Lisbon, Portugal; School of Psychology (Prof S Cortese PhD), University of Southampton, Southampton, United Kingdom; Department of Family Medicine and Public Health (Prof M H Criqui MD), University of California San Diego, La Jolla, CA, USA; Department of Diagnostic and Therapeutic Technologies (Prof N Cruz-Martins PhD), Cooperativa de Ensino Superior Politécnico e Universitário (Polytechnic and University Higher Education Cooperative), Vila Nova de Famalicão, Portugal; Institute for Research and Innovation in Health (i3S) (Prof N Cruz-Martins PhD), University of Porto, Porto, Portugal; Department of Medical, Surgical, and Health Sciences (Prof M D'Oria MD), University of Trieste, Trieste, Italy; Cardio-Thoraco-Vascular Department (Prof M D'Oria MD), Azienda Sanitaria Universitaria Giuliano Isontina, Trieste, Italy; Epidemiology & Biostatistics, College of Public Health (B Dabo MSPH), Department of Medical Engineering (D H Nguyen BS), University of South Florida, Tampa, FL, USA; Department of Medical Microbiology and Parasitology (B Dabo MSPH), Bayero University, Kano, Nigeria; School of Pharmacy and Charles Perkins Centre (Z Dai PhD), Sydney Medical School (S Islam PhD), University of Sydney, Sydney, NSW, Australia; IRCCS Istituto Ortopedico Galeazzi (G Damiani MD), University of Milan, Milan, Italy; Department of Dermatology (G Damiani MD, A Grada MD), Lerner College of Medicine (L Göbölös PhD), Harrington Heart and Vascular Institute (A Guha MD), Department of Quantitative Health Science (X Liu PhD), Case Western Reserve University, Cleveland, OH, USA; Department of Public Health (S D D Darcho MPH), Haramaya University, Harar, Ethiopia; Department of Information Technology (A M Darwesh PhD), Department of Computer Science (Prof M Hosseinzadeh PhD), University of Human Development, Sulaymaniyah, Iraq; Department of Biochemistry (S Das MD), Ministry of Health and Welfare, New Delhi, India; Immunology Research Center (M Dashti MD, A Ghasemzadeh MD), Department of Radiology (M Mirza-Aghazadeh-Attari MD), Tabriz University of Medical Sciences, Tabriz, Iran; Department of Psychiatry (Prof D C Des Jarlais PhD), Clinical Research (S Khosravi MD), Institute for Critical Care Medicine (A Shaikh MD), Department of Cardiology (M Vinayak MD), Icahn School of Medicine at Mount Sinai, New York, NY, USA (A Shaikh MD); Chettinad Hospital & Research Institute (Prof V Devanbu PhD), Chettinad Academy of Research and Education, Chennai, India; Department of Pharmacy (S Dewan PhD), United International University, Dhaka, Bangladesh; Pharmacology Division (S Dewan PhD), Center for Life Sciences Research Bangladesh, Dhaka, Bangladesh; Division of Pathology (K Dhama PhD), ICAR-Indian Veterinary Research Institute, Bareilly, India; Faculty of Science (Prof D Diaz PhD), National Autonomous University of Mexico, Mexico City, Mexico; Department of Gastroenterology (L A Diaz MD), Pontifical Catholic University of Chile, Santiago, Chile; Department of Medicine (T H Do MD), Can Tho University of Medicine and Pharmacy, Can Tho, Vietnam; Department of Medicine (T C Do MD), Pham Ngoc Thach University of Medicine, Ho Chi Minh City, Vietnam; Department of Pathology (K Doan MD), Temple University Hospital, Philadelphia, PA, USA; Health Science Center (D Dongarwar MS), Department of Plastic Surgery (A M Hassan MD), University of Texas, Houston, TX, USA; Independent Consultant, South Plainfield, NJ, USA (O P Doshi MSc); Department of Cardiology (R P Doshi MD), St. Joseph's University Medical Center, Paterson, NJ, USA; School of Population Health and Environmental Sciences (A Douiri PhD), King's College London, London, United Kingdom; Office of Institutional

Analysis (J Dube MA), University of Windsor, Windsor, ON, Canada; Department of Pharmacology (S Dutta MD), All India Institute of Medical Sciences, Rajkot, India; Department of Conservative Dentistry with Endodontics (A M Dziedzic DSc), Medical University of Silesia, Katowice, Poland; Almoosa College of Health Sciences, Al Ahsa, Saudi Arabia (R A El Arab PhD); Department of Public Health and Community Medicine (Prof I F El Bayoumy DrPH), Texila American University, Tanta city, Egypt; School of Public health (Prof I F El Bayoumy DrPH), Texila American University, Guyana, Guyana; Faculty of Medicine (M Elhadi MD), University of Tripoli, Tripoli, Libya; Department of Neuropsychiatry (Prof G ELNahas MD), Department of Entomology (A M Samy PhD), Medical Ain Shams Research Institute (MASRI) (A M Samy PhD), Ain Shams University, Cairo, Egypt; Executive Committee (Prof G ELNahas MD), International Association for Women Mental Health, Potomac, MD, USA; Department of Clinical Pathology (Prof M Elshaer PhD), Mansoura University, Mansoura, Egypt; Department of Pediatrics (C Eltaha MD), Texas A&M University, Dallas, TX, USA; Department of Health Management and Policy (E F Ezenwankwo MPH), Drexel University, Philadelphia, PA, USA; Department of Oral Biology (A Fahim PhD), The University of Lahore, Lahore, Pakistan; Department of Food Hygiene and Quality Control (A Fakhri-Demeshghieh DVM), University of Tehran, Tehran, Iran; Satcher Health Leadership Institute (A O Fasanmi PhD), Morehouse School of Medicine, Atlanta, GA, USA; Laboratory of experimental Medicine (T Fazylov MD), Atchabarov Scientific-Research Institute of Fundamental and Applied Medicine (M Kulimbet MSc), National Research Institute of Fundamental and Applied Medicine named after B. Atchabarov (A Zhumagaliuly MD), Kazakh National Medical University, Almaty, Kazakhstan; National Institute for Stroke and Applied Neurosciences (Prof V L Feigin PhD), Auckland University of Technology, Auckland, New Zealand; Research Center of Neurology, Moscow (Prof V L Feigin PhD); Russia, (Prof V L Feigin PhD); Department of Public Health and Infectious Diseases (G Fekadu PhD), City University of Hong Kong, Hong Kong, China; Department of Pharmacy (G Fekadu PhD), Wollega University, Nekemte, Ethiopia; Department of Social Sciences (Prof N Ferreira PhD), University of Nicosia, Nicosia, Cyprus; Department of Psychiatry (I Filip MD), Kaiser Permanente, Mission Viejo, CA, USA; School of Health Sciences (I Filip MD), A.T. Still University, Mesa, AZ, USA; Institute of Public Health (F Fischer PhD), Charité Universitätsmedizin Berlin, Berlin, Germany; Department of Dermatology (T Fukumoto PhD), Kobe University, Kobe, Japan; Department of Community Medicine (Prof M A Gadanya MD), Bayero University Kano, Kano, Nigeria; Department of Community Medicine (Prof M A Gadanya MD), Aminu Kano Teaching Hospital, Kano, Nigeria; Department of Neurosurgery (A A Gajjar MBA), Albany Medical College, Albany, NY, USA; Department of Neurosurgery (A A Gajjar MBA), Department of Medicine (J C Lin BS), Department of Biostatistics, Epidemiology, and Informatics (J Puvvula PhD), University of Pennsylvania, Philadelphia, PA, USA; Institute of Health and Wellbeing (B Ganesan PhD), Federation University Australia, Churchill, VIC, Australia; Department of General Medicine (M Ganiyani MD), Grant Medical College & Sir J.J. Group of Hospitals, Mumbai, India; School of Public Health (X Gao PhD), Key Lab of Environment and Health (X Gao PhD), Xuzhou Medical University, Xuzhou, China; Department of Midwifery (M W Gebregergis MSc), Department of Medical Laboratory Sciences (H N Meles MSc), Adigrat University, Adigrat, Ethiopia; Department of Environmental Health (M Gebrehiwot DSc), Wollo University, Dessie, Ethiopia; Department of Reproductive and Family Health (T G Gebremeskel PhD), Axum College of Health Science, Axum, Ethiopia; College of Medicine and Public Health (T G Gebremeskel PhD), Flinders University, Adelaide, SA, Australia; Department of Radiology (A Gholamrezanezhad MD), University of Southern California, Los Angeles, CA, USA; Department of Cardiac Surgery (L Göbölös PhD), Cleveland Clinic Abu Dhabi, Abu Dhabi, United Arab Emirates; Department of Health Systems and Policy Research (M Golechha PhD), Indian Institute of Public Health, Gandhinagar, India; Department of Life Sciences, Health and Healthcare Professions (Prof D Golinelli MD), Link Campus University, Rome, Italy; Health Services Research, Evaluation and Policy Unit (Prof

D Golinelli MD), AUSL della Romagna, Ravenna, Italy; Division of Cardiovascular Medicine (A Guha MD), Ohio State University, Columbus, OH, USA; Health Directorate (S Guicciardi MD), Local Health Authority of Bologna, Bologna, Italy; Department of Internal Medicine (I Gupta MD), Independent Consultant, Bharatpur, India; NGO (I Gupta MD), Independent Consultant, Delhi, India; School of Medicine (V Gupta PhD), Institute for Mental and Physical Health and Clinical Translation (IMPACT) (W Marx PhD), Deakin University, Geelong, VIC, Australia; Faculty of Medicine Health and Human Sciences (Prof V K Gupta PhD), Macquarie University, Sydney, NSW, Australia; Department of Global Health and Population (A Haakenstad ScD), T.H. Chan School of Public Health, Boston, MA, USA; Department of Medicine (P Habibzadeh MD), University of Pittsburgh Medical Center, Pittsburgh, PA, USA; Department of Surgery (N Haep MD), Charité Medical University Berlin, Berlin, Germany; Clinician Scientist Program (N Haep MD), Berlin Institute of Health, Berlin, Germany; Department of Medicine (O I Haque MD), MedStar Health, Baltimore, MD, USA; Department of Zoology and Entomology (A I Hasaballah PhD, M G M Zeariya PhD), Al-Azhar University, Cairo, Egypt; Department of Health Research Methods, Evidence and Impact (M Hasan MPH), Department of Medicine (O P Kurmi PhD), Department of Psychiatry and Behavioural Neurosciences (A T Olagunju MD), McMaster University, Hamilton, ON, Canada; Department of Biochemistry and Molecular Biology (M Hasan MPH), Tejgaon College, Dhaka, Bangladesh; Department of Pharmacy (Prof M S Hasnain PhD), Palamau Institute of Pharmacy, Daltonganj, India; Skaane University Hospital (R J Havmoeller PhD), Skaane County Council, Malmö, Sweden; Faculty of Kinesiology (Prof J J Hebert PhD), University of New Brunswick, Fredericton, NB, Canada; School of Allied Health (Prof J J Hebert PhD), Murdoch University, Murdoch, WA, Australia; Family Health Research Institute (Z Heidary PhD), Tehran University of Medical Sciences., Tehran, Iran; Department of Medicine (M Hemmati MD), MedStar Health, Washington, DC, USA; Department of Medicine (M Hemmati MD, C J Sabet MA), Georgetown University, Washington, DC, USA; Department of Population Health (I Hidayana PhD), School of Health Professions and Human Services (I M Karaye MD), Hofstra University, Hempstead, NY, USA; Division for Health Service Promotion (Y Hiraike PhD), University of Tokyo, Tokyo, Japan; School of Dentistry (N Hoan DDS), Hanoi Medical University, Hanoi, Vietnam; Department of Pulmonology (N Horita PhD), Yokohama City University, Yokohama, Japan; National Human Genome Research Institute (NHGRI) (N Horita PhD), Center for Translation Research and Implementation Science (G A Mensah MD), National Institutes of Health, Bethesda, MD, USA; Centre for Advancing Health Outcomes, Vancouver, BC, Canada (M Hossain MSc); Department of Decision and Information Sciences (M Hossain DrPH), Pharmaceutical Health Outcomes and Policy (J D Thornton PhD), Prescription Drug Misuse Education and Research Center (J D Thornton PhD), University of Houston, Houston, TX, USA; Public Health Research Group (M Hossain DrPH), Nature Study Society of Bangladesh, Khulna, Bangladesh; Institute of Research and Development (Prof M Hosseinzadeh PhD), Faculty of Medicine (H T H Nguyen MD), Institute for Research and Training in Medicine, Biology and Pharmacy (H T H Nguyen MD), Duy Tan University, Da Nang, Vietnam; Department of Legal Medicine and Bioethics (Prof S Hostiuc PhD), Carol Davila University of Medicine and Pharmacy, Bucharest, Romania; Department of Clinical Legal Medicine (Prof S Hostiuc PhD), National Institute of Legal Medicine Mina Minovici, Bucharest, Romania; Department of Psychology (C Hu PhD), Tsinghua University, Beijing, China; Faculty of Medicine (J Huang MD), Jockey Club School of Public Health and Primary Care (C Zhong PhD), The Chinese University of Hong Kong, Hong Kong, China; International Master Program for Translational Science (H Huynh BS), Taipei Medical University, Taipei, Taiwan; Division of Infectious Diseases (K S Ikuta MD), Veterans Affairs Greater Los Angeles, Los Angeles, CA, USA; Institute for Physical Activity and Nutrition (S Islam PhD), Deakin University, Burwood, VIC, Australia; School of Pharmacy (M Islam PhD), BRAC University, Dhaka, Bangladesh; Department of Physical and Medicine (L Jacob MD), Université Paris Cité, Paris, France; Research and Development Unit (L Jacob

MD), Biomedical Research Networking Center for Mental Health Network (CiberSAM), Barcelona, Spain; Department of Health Studies (K H Jacobsen PhD), University of Richmond, Richmond, VA, USA; Department of Leukemia (A Jain MD), The University of MD Anderson Cancer Center, Houston, TX, USA; Department of Health and Safety (A A Jairoun PhD), Dubai Municipality, Dubai, United Arab Emirates; The World Academy of Sciences UNESCO, Trieste, Italy (Prof M Jakovljevic PhD); Shaanxi University of Technology, Hanzhong, China (Prof M Jakovljevic PhD); Health Informatic Lab (T Javaheri PhD), Department of Computer Science (R Rawassizadeh PhD), Boston University, Boston, MA, USA; Department of Internal Medicine (B M Jeswani MBBS), GCS Medical College, Hospital & Research Centre, Ahmedabad, India; Department of Nutrition (A Jeyakumar PhD), University of Nevada Reno, Reno, NV, USA; University of Johannesburg, Johannesburg, South Africa (A Jeyakumar PhD); Danish Center for Health Economics (E K Johnson MSc), University of Southern Denmark, Odense, Denmark; Faculty of Dentistry (K K Kanmodi MPH), University of Puthisastra, Phnom Penh, Cambodia; Office of the Executive Director (K K Kanmodi MPH), Cephas Health Research Initiative Inc, Ibadan, Nigeria; The Hansjörg Wyss Department of Plastic and Reconstructive Surgery (R S Kantar MD), NYU Langone Health, New York, NY, USA; Cleft Lip and Palate Surgery Division (R S Kantar MD), Global Smile Foundation, Norwood, MA, USA; Department of Anesthesiology (I M Karaye MD), Montefiore Medical Center, Bronx, NY, USA; Department of Medicine (A Katamreddy MD), Jacobi Medical Center, New York, NY, USA; Centre for Adolescent Health (J A Kerr PhD), Murdoch Childrens Research Institute, Parkville, VIC, Australia; Department of Psychological Medicine (J A Kerr PhD), University of Otago, Christchurch, New Zealand; Halal Research Centre of IRI, Iran Food and Drug Administration (F Khamesipour PhD), Institute of Immunology and Infectious Diseases (F Khamesipour PhD), Iranian Ministry of Health and Medical Education, Tehran, Iran; Department of Medicine (Z A Khan MD), Shadan Hospital, Hyderabad, India; Department of Physical Therapy (F Khan PhD), Department of Dental Public Health (Z S Natto DrPH), King Abdulaziz University, Jeddah, Saudi Arabia; Natural and Medical Sciences Research Center (A Khan PhD, A Ullah MS), School of Pharmacy (A K Philip PhD), University of Nizwa, Nizwa, Oman; College of Health, Wellbeing and Life Sciences (Prof K Khatab PhD), Sheffield Hallam University, Sheffield, United Kingdom; College of Arts and Sciences (Prof K Khatab PhD), Ohio University, Zanesville, OH, USA; Department of Basic Medical Sciences (M M Khatatbeh PhD), Yarmouk University, Irbid, Jordan; Department of Internal Medicine (A A Khosla MD), Corewell Health East William Beaumont University Hospital, Royal Oak, MI, USA; Department of Epidemiology and Biostatistics (S Khosravi MD), Non-Communicable Diseases Research Center (NCDRC), Tehran, Iran (F Montazeri MD); Department of Health Management and Economics (M Khosravi PhD), Qom University of Medical Sciences, Qom, Iran; Department of Public Health (J Khubchandani PhD), New Mexico State University, Las Cruces, NM, USA; Department of Pediatrics (G Kim MD), Case Western Reserve University School of Medicine, Cleveland, OH, USA; Division of Pediatric Hospital Medicine (G Kim MD), UH Rainbow Babies and Children's Hospital, Cleveland, OH, USA; Broad Institute of MIT and Harvard, Cambridge, MA, USA (M Kim MD); Millennium Prevention, Inc., Westwood, MA, USA (R W Kimokoti MD); School of Health Sciences (Prof A Kisa PhD), Kristiania University College, Oslo, Norway; Department of International Health and Sustainable Development (Prof A Kisa PhD), Tulane University, New Orleans, LA, USA; Global Healthcare Consulting, New Delhi, India (S Kochhar MD); Copernicus Institute of Sustainable Development (G Koren PhD), Utrecht University, Utrecht, Netherlands; Department of Anesthesiology (V Krishnamoorthy MD), Department of Population Health Sciences (J B Lusk MD), Duke Global Health Institute (C Wu PhD), Duke University, Durham, NC, USA; Foundation for Drug Policy Solutions, Washington, DC, USA (C M Kubeisy BA); Department of Mathematics (M Kuddus PhD), University of Rajshahi, Rajshahi, Bangladesh; Center of Medicine and Public Health (M Kulimbet MSc), Asfendiyarov Kazakh National Medical University, Almaty, Kazakhstan; Department of Medicine (V Kulkarni MS),

Digital Health and Informatics Directorate (Prof S M McPhail PhD), Queensland Health, Brisbane, QLD, Australia; GISE Hub (V Kumar PhD), Indian Institute of Technology, Mumbai, India; Centre for Studies in Economics and Planning (V Kumar PhD), Central University of Gujarat, Gandhinagar, India; Department of Cardiovascular Medicine (A Kumar MD), Cabrini Institute, Rochester, MN, USA; College of Public Health and Health Informatics (R Kumar PhD), Department of Public Health (M G M Zeariya PhD), University of Hail, Hail, Saudi Arabia; School of Medicine and Dentistry (S Kundu MPH), Griffith University, Gold Coast, QLD, Australia; Department of Nutrition and Food Science (S Kundu MPH), Patuakhali Science and Technology University, Patuakhali, Bangladesh; Faculty of Health and Life Sciences (O P Kurmi PhD), Coventry University, Coventry, United Kingdom; Department of Nursing (E F Kyei MSc), University of Massachusetts Boston, Boston, MA, USA; Department of Occupational and Environmental Health (H Lai PhD), Yangzhou University, Yangzhou, China; Department of Respiratory and Critical Care Medicine (H Lai PhD), Northern Jiangsu People's Hospital, Yangzhou, China; Division of Cancer Epidemiology and Genetics (Q Lan PhD), National Cancer Institute, Rockville, MD, USA; Chief Medical Office (Prof V C Lansingh PhD), HelpMeSee, New York, NY, USA; Mexican Institute of Ophthalmology, Queretaro, Mexico (Prof V C Lansingh PhD); Faculty of Medicine (H Le MD, N Le MD), Department of Medicine (T Nguyen MD), Department of General Medicine (V T Nguyen MD), Department of Internal Medicine (T H Tran MD), University of Medicine and Pharmacy at Ho Chi Minh City, Ho Chi Minh City, Vietnam (T D T Le MD, T T Le MD); Independent Consultant, Ho Chi Minh City, Vietnam (T D T Le MD); Department of Cardiovascular Research (H Le MD, N Le MD), Methodist Hospital, Merrillville, IN, USA; College of Optometry (J L Leasher OD), Nova Southeastern University, Fort Lauderdale, FL, USA; Department of Precision Medicine (Prof S Lee MD), Sungkyunkwan University, Suwon-si, South Korea; Department of Biomedical and Biotechnological Sciences (Prof M Libra PhD), University of Catania, Catania, Italy; Department of Neurology (Prof V Lioutas MD), Department of Psychiatry (R T Liu PhD), Harvard Medical School, Boston, MA, USA; Department of Neurology (Prof V Lioutas MD), Framingham Heart Study, Framingham, MA, USA; One Health Research Group (J López-Gil PhD), Universidad de Las Américas, Quito, Ecuador; Department of Epidemiology and Evidence-Based Medicine (P D Lopukhov PhD), I.M. Sechenov First Moscow State Medical University, Moscow, Russia; School of Medicine (Prof G Lucchetti PhD), Federal University of Juiz de Fora, Juiz de Fora, Brazil; Department of Emergency General and Trauma Surgery (Prof R Lunevicius DSc), Liverpool University Hospitals NHS Foundation Trust, Liverpool, United Kingdom; Department of Surgery (Prof R Lunevicius DSc), University of Liverpool, Liverpool, United Kingdom; Smidt Heart Institute (Y Manla MD), Cedars-Sinai Medical Center, Los Angeles, CA, USA; University Health Services (A M Marconi MD), University of Wisconsin Madison, Madison, WI, USA; Centro de Estudio e Investigación para la prevención y el tratamiento de las adicciones (Center for the Study and Investigation of Addiction Prevention and Treatment) (A M Marconi MD), Universidad de Buenos Aires (University of Buenos Aires), Buenos Aires, Argentina; Department of Food, Environmental and Nutritional Sciences (DeFENS) (M Marino PhD), University of Milan, Milano, Italy; Department of Physics and Atmospheric Science (Prof R V Martin PhD), Dalhousie University, Halifax, NS, Canada; Department of Non-communicable Diseases and Mental Health (R Martinez-Piedra BSc), Pan American Health Organization, Washington, DC, USA; Faculty of Humanities and Health Sciences (Prof R R Marzo MD), Curtin University, Sarawak, Malaysia; Jeffrey Cheah School of Medicine and Health Sciences (Prof R R Marzo MD), Monash University, Subang Jaya, Malaysia; Department of Anatomy and Developmental Biology (Y Mathangasinghe PhD), University of Colombo, Clayton, VIC, Australia; Department of Anatomy, Genetics and Biomedical Informatics (Y Mathangasinghe PhD), Monash University, Colombo, Sri Lanka; Department of Maternal-Child Nursing and Public Health (Prof F P Matozinhos PhD), Federal University of Minas Gerais, Belo Horizonte, Brazil; Australian Centre for Health Services Innovation (Prof S M McPhail PhD), Queensland University of Technology, Kelvin

Grove, QLD, Australia; Department of Public Health (T Mekene Meto MPH), Arba Minch University, Arba Minch, Ethiopia; Department of Internal Medicine (E Melese MD), School of Nursing (H B Netsere MSc), University of Gondar, Gondar, Ethiopia; Department of Medicine (G A Mensah MD), University of Cape Town, Cape Town, South Africa; Eunice Kennedy Shriver National Institute of Child Health and Human Development (L G Mensah MD), National Institute of Health, Bethesda, MD, USA; Department of Physiology (Prof S A Meo PhD), Pediatric Intensive Care Unit (Prof M Temsah MD), King Saud University, Riyadh, Saudi Arabia; University Centre Varazdin (T Mestrovic PhD), University North, Varazdin, Croatia; South African Centre for Epidemiological Modelling and Analysis (SACEMA) (L Mhlanga PhD), Stellenbosch University, Cape Town, South Africa; Stritch School of Medicine (A Mhlanga PhD), Loyola University Chicago, Chicago, IL, USA; National Cancer Registry (I Michalek PhD), Pathology Department (I Michalek PhD), Maria Sklodowska-Curie National Research Institute of Oncology, Warsaw, Poland; Pacific Institute for Research & Evaluation, Calverton, MD, USA (T R Miller PhD); School of Public Health (T R Miller PhD), Curtin University, Perth, WA, Australia; Social Determinants of Health Center (M Mirza-Aghazadeh-Attari MD), Urmia University of Medical Sciences, Urmia, Iran; Division of Cardiology (A K Mishra MD), St. Vincent College, Worcester, MA, USA; Molecular Biology Unit (N S Mohamed MSc), Bio-Statistical and Molecular Biology Department (N S Mohamed MSc), Sirius Training and Research Centre, Khartoum, Sudan; College of Applied and Natural Science (J Mohamed MSc), University of Hargeisa, Hargeisa, Somalia; Department of Pharmaceutical Sciences (S Mohammed PhD), Notre Dame of Maryland University, Baltimore, MD, USA; Department of Pharmacy (S Mohammed PhD), Mizan-Tepi University, Mizan, Ethiopia; Health Systems and Policy Research Unit (Prof S Mohammed PhD), Department of Community Medicine (A A Olorukooba MD), Ahmadu Bello University, Zaria, Nigeria; Heidelberg Institute of Global Health (HIGH) (Prof S Mohammed PhD), Technical University of Berlin, Heidelberg, Germany; Charles Sturt University (M Moni PhD), Charles Sturt University, Bathurst, NSW, Australia; The University of Queensland, Brisbane, QLD, Australia (M Moni PhD); Department of Ophthalmology and Visual Sciences (F Montazeri MD), University of California Davis, Sacramento, CA, USA; Department of Epidemiology and Biostatistics (Y Moradi PhD), Kurdistan University of Medical Sciences, Sanandaj, Iran; Computer, Electrical, and Mathematical Sciences and Engineering Division (P Moraga PhD), King Abdullah University of Science and Technology, Thuwal, Saudi Arabia; Department of Community Medicine (R Motappa MD), Department of Forensic Medicine and Toxicology (P H Shetty MD), Manipal College of Dental Sciences (Prof P K Shetty MSD), Manipal Academy of Higher Education, Mangalore, India; Clinical Epidemiology Research Unit (E Murillo-Zamora PhD), Mexican Institute of Social Security, Villa de Alvarez, Mexico; Postgraduate in Medical Sciences (E Murillo-Zamora PhD), Universidad de Colima, Colima, Mexico; Department of Computer Science (P Naghavi MS), University of Illinois Urbana-Champaign, Urbana, IL, USA; Department Health Services Research (G Naik MPH), Department of Psychology (D C Schwebel PhD), University of Alabama at Birmingham, Birmingham, AL, USA; Department of Internal Medicine (D P Nanavaty MD), The Brooklyn Hospital Center, Brooklyn, NY, USA; National Dental Research Institute Singapore (G G Nascimento PhD), Duke-NUS Medical School, Singapore, Singapore; School of Pharmacy (S O Nduaguba PhD), West Virginia University, Morgantown, WV, USA; College of Medicine and Health Sciences (H B Netsere MSc), Bahir Dar University, Bahir Dar, Ethiopia; Department of Orthopedic Surgery (A Nezameslami MD), Department of Nephrology and Hypertension (N Nikravangolsefid MD), Department of Radiology (F Nugen PhD), Department of Informatics and Radiology (S Vahdati MD), Mayo Clinic, Rochester, MN, USA; Department of Urology (T Nguyen MD), University of California Irvine, Irvine, CA, USA; Cardiovascular Research Department (H Q Nguyen MD), Methodist Hospital, Merrillville, IL, USA; Department of General Medicine (A H Nguyen MD), Thai Binh University of Medicine and Pharmacy, Thai Binh City, Vietnam; Institute for Mental Health Policy Research (Y T Nigatu PhD), Centre for

Addiction and Mental Health, Toronto, ON, Canada; Department of Statistics (S Noor MS), Shahjalal University of Science and Technology, Sylhet, Bangladesh; School of Information (F Nugen PhD), University of California Berkeley, Berkeley, CA, USA; School of Nursing (J Nutor PhD), Department of Epidemiology and Biostatistics (M Teramoto MD), Department of Bioengineering and Therapeutical Sciences (Prof M Zastrozhin PhD), University of California San Francisco, San Francisco, CA, USA; Department of Physiology (O J Nzoputam PhD), University of Benin, Edo, Nigeria; Department of Physiology (O J Nzoputam PhD), Benson Idahosa University, Benin City, Nigeria; Department of Applied Economics and Quantitative Analysis (Prof B Oancea PhD), University of Bucharest, Bucharest, Romania; PSSM Data Sciences (M Oduro PhD), Pfizer Inc., Groton, CT, USA; Independent Consultant, Sydney, NSW, Australia (S R Okeke PhD); School of Pharmacy (O C Okonji MSc), University of the Western Cape, Cape Town, South Africa; Department of Psychiatry (A T Olagunju MD), University of Lagos, Lagos, Nigeria; Slum and Rural Health Initiative Research Academy (I I Olufadewa MHS), Slum and Rural Health Initiative, Ibadan, Nigeria; Department of Biomedical Sciences (K I Onyedibe PhD), Mercer University School of Medicine, Macon, GA, USA; Center for Health Systems Research (D V Ortega-Altamirano EdD), National Institute of Public Health, Cuernavaca, Mexico; Department of Respiratory Medicine (Prof M P P A DNB), Jagadguru Sri Shivarathreeswara University, Mysore, India; Centre for Biotechnology (S K Panda PhD), Department of Analytical and Applied Economics (Prof H Rout PhD, C Swain MPhil), RUSA Centre of Excellence in Public Policy and Governance (Prof H Rout PhD), Utkal University, Bhubaneswar, India; Department of Zoology (S K Panda PhD), Katholieke Universiteit Leuven, Bhubaneswar, India; Department of Epidemiology and Community Health (R R Parikh MD), Department of Surgery (J Rickard MD), University of Minnesota, Minneapolis, MN, USA; Department of Health Policy and Management (S Park PhD), Korea University, Seoul, South Korea; Department of Medical Humanities and Social Medicine (Prof E Park PhD), Kosin University, Busan, South Korea; Department of Biomedical Data Science (S Park MD), Department of Radiology (S Ramasamy MD), Stanford University, Stanford, CA, USA; Department of Epidemiology, Human Genetics and Environmental Sciences (J R Patel PhD), The University of Texas Health Science Center at Houston, Dallas, TX, USA; Department of Epidemiology (J R Patel PhD), University of Arkansas for Medical Sciences, Little Rock, AR, USA; College of Dental Medicine (Prof S Patil PhD), Roseman University of Health Sciences, South Jordan, UT, USA; Centre of Molecular Medicine and Diagnostics (COMManD) (Prof S Patil PhD), Saveetha Dental College and Hospitals (M Tovani-Palone PhD), Saveetha University, Chennai, India; School of Population Health (Prof G Pereira PhD), Curtin University, Bentley, WA, Australia; Centre for Fertility and Health (Prof G Pereira PhD), Norwegian Institute of Public Health, Oslo, Norway; Pennsylvania Cancer and Regenerative Medicine Center (R G Pestell MD), Baruch S Blumberg Institute, Doylestown, PA, USA; Department of Medicine (R G Pestell MD), Xavier University School of Medicine, Woodbury, NY, USA; Department of Internal Medicine (H Pham MD), Weiss Memorial Hospital, Chicago, IL, USA; Shanghai Mental Health Center (Prof M R Phillips MD), Shanghai Jiao Tong University, Shanghai, China; Department of Pediatric Orthopedic Surgery (M Pigeolet MD), Boston Children's Hospital, Boston, MA, USA; University Medical Center Groningen (Prof M J Postma PhD), University of Groningen, Groningen, Netherlands; Center of Excellence in Higher Education for Pharmaceutical Care Innovation (Prof M J Postma PhD), Universitas Padjadjaran (Padjadjaran University), Bandung, Indonesia; Independent Consultant, San Diego, CA, USA (D Prabhu PhD); Department of Community Medicine (P M S Pradhan MD), Tribhuvan University, Kathmandu, Nepal; Department of Humanities and Social Sciences, NIT Rourkela (Prof J Pradhan PhD), National Institute of Technology Rourkela, Rourkela, India; Cardiovascular Research Center (M Rabiee Rad MD), Isfahan Cardiovascular Research Institute, Isfahan, Iran; College of Medicine (A Radfar MD), University of Central Florida, Orlando, FL, USA; Avicenna Medical and Clinical Research Institute, Encino, CA, USA (A Radfar MD); Institute of Health and Wellbeing (Prof M Rahman PhD), Federation

University Australia, Berwick, VIC, Australia; School of Nursing and Midwifery (Prof M Rahman PhD), La Trobe University, Melbourne, VIC, Australia; Department of Population Health (M Ramadan DrPH, M Ramadan DrPH), King Saud bin Abdulaziz University for Health Sciences, Jeddah, Saudi Arabia; School of Nursing & Health Sciences (S Ramazanu PhD), Hong Kong Metropolitan University, Hong Kong, China; Health Economics and Outcomes Research Department (A Rane MS), Agios Pharmaceuticals, Cambridge, MA, USA; Department of Pharmaceutical Economics and Policy (A Rane MS), Massachusetts College of Pharmacy and Health Sciences, Boston, MA, USA; Department of Medicine (A M Rashid MD), Jinnah Sindh Medical University, Karachi, Pakistan; Baylor University, Dallas, TX, USA (A M Rashid MD); Department of Biological Sciences (Prof E M M Redwan PhD), King Abdulaziz University, Jeddah, Egypt; Department of Protein Research (Prof E M M Redwan PhD), Research and Academic Institution, Alexandria, Egypt; Department of Public Health Sciences (T Rhee PhD), University of Connecticut, Farmington, CT, USA; Department of Surgery (J Rickard MD), University Teaching Hospital of Kigali, Kigali, Rwanda; Department of Geography and Demography (M Rodrigues PhD), University of Coimbra, Coimbra, Portugal; Department of Pharmacology and Toxicology (Departamento de Farmacologia y Toxicologia) (Prof J A B Rodriguez PhD), University of Antioquia, Medellin, Colombia; University of Warwick, Coventry, United Kingdom (Prof J A B Rodriguez PhD); Nuffield Department of Medicine (T Runghien MSc), University of Oxford, Oxford, United Kingdom; Cardiovascular Department (Prof A M A Saad MD), Zagazig University, Zagazig, Egypt; Clinical and Biomedical Research Center (Prof U Saeed PhD), Foundation University, Islamabad, Pakistan; International Center of Medical Sciences Research (ICMSR), Islamabad, Pakistan (Prof U Saeed PhD); Department of Psychosocial Science (Prof D Sagoe PhD), University of Bergen, Bergen, Norway; Department of Health and Kinesiology (M Sajib BDS), University of Illinois, Urbana-Champaign, IL, USA; Department of Global Initiatives (Prof G A Salum PhD), Child Mind Institute, New York, NY, USA; Department of Psychiatry and Legal Medicine (Prof G A Salum PhD), Federal University of Rio Grande do Sul, Porto Alegre, Brazil; Department of Anatomy (Prof V P Samuel PhD), Ras Al Khaimah Medical and Health Sciences University, Ras Al Khaimah, United Arab Emirates; Indira Gandhi Medical College and Research Institute, Puducherry, India (A Saravanan MD); Department of Orthopaedics and Trauma Surgery (B Saravi PhD), University of Freiburg, Freiburg, Germany; Department of Orthopaedics (B Saravi PhD), Loretto Hospital Freiburg, Freiburg, Germany; Department of Neurology (Prof N Scarmeas PhD), National and Kapodistrian University of Athens, Athens, Greece; Dobney Hypertension Centre (Prof M P Schlaich MD), The University of Western Australia, Perth, WA, Australia; Department of Cardiovascular Sciences (A Schuermans BSc, J Van den Eynde BSc), Katholieke Universiteit Leuven, Leuven, Belgium; National Heart, Lung, and Blood Institute (A Seylani BS), National Institute of Health, Rockville, MD, USA; Independent Consultant, Karachi, Pakistan (M A Shaikh MD); Institute of Health Policy, Management, and Evaluation (H Shakil MD), Division of Neurosurgery (H Shakil MD), University of Toronto, Toronto, ON, Canada; Department of Pathobiology (M Shamshirgaran PhD), Shahid Bahonar University of Kerman, Kerman, Iran; Department of Medicine (M Sharath MBBS), Bangalore Medical College and Research Institute, Bangalore, India; Friedman School of Nutrition Science and Policy (P Shi PhD), Tufts University, Boston, MA, USA; Department of Veterinary Public Health and Preventive Medicine (A Shittu MSc), Usmanu Danfodiyo University, Sokoto, Sokoto, Nigeria; Department of Experimental Research (V Shivarov PhD), Medical University Pleven, Sofia, Bulgaria; Department of Genetics (V Shivarov PhD), Sofia University "St. Kliment Ohridiski", Sofia, Bulgaria; The Cooper Institute, Dallas, TX, USA (K Shuval PhD); Department of Medical Microbiology and Infectious Diseases (E E Siddig MD), Erasmus University, Rotterdam, Netherlands; Division of Injury Prevention (Prof D A Sleet PhD), The Bizzell Group, Atlanta, GA, USA; Department of Systemic Pathology (R Solanki MD), Touro College of Osteopathic Medicine, Middletown, NY, USA; Department of Pathology (R Solanki MD), American University of the

Caribbean School of Medicine, Cupecoy, Saint Martin; Department of Biochemistry (S Solanki MD), American University of Integrative Sciences, Bridgetown, Barbados; Institute of Child and Adolescent Health (Y Song PhD), School of Public Health (H Zhang MS), Peking University, Beijing, China; Mental Health Research Consultant (A Sultana MD), Independent Consultant, Khulna, Bangladesh; Division of Global Mental Health (A Sultana MD), EviSyn Health, Khulna, Bangladesh; Rural Health Research Institute (Prof J Sun PhD), Charles Sturt University, Orange, NSW, Australia; Institute of Integrated Intelligence and Systems (Prof J Sun PhD), Griffith University, Brisbane, QLD, Australia; Henry JN Taub Department of Emergency Medicine (Prof L Szarpak PhD), Baylor College of Medicine, Houston, TX, USA; Department of Clinical Research and Development (Prof L Szarpak PhD), LUXMED Group, Warsaw, Poland; Department of Medical Informatics (S Tabatabaei PhD), Clinial Research Development Unit (S Tabatabaei PhD), Mashhad University of Medical Sciences, Mashhad, Iran; Department of Environmental, Agricultural and Occupational Health (J Taiba MPH), University of Nebraska Medical Center, Omaha, NE, USA; Sri Ramachandra Medical College and Research Institute, Chennai, India (J Taiba MPH); Department of Economics (N Y Tat MS), Rice University, Houston, TX, USA; Department of Research and Innovation (N Y Tat MS), Enventure Medical Innovation, Houston, TX, USA; SRM College of Pharmacy (M Tovani-Palone PhD), SRM Institute of Science and Technology (SRMIST), Chennai, India; Department of Business Analytics (T H Tran MD), University of Massachusetts Dartmouth, Dartmouth, MA, USA; School of Biomedical Engineering (N Tran MD), University of Technology Sydney, Sydney, NSW, Australia; Department of Health Sciences (S J Tromans PhD), University of Leicester, Leicester, United Kingdom; Adult Learning Disability Service (S J Tromans PhD), Leicestershire Partnership National Health Service Trust, Leicester, United Kingdom; Faculty of Medicine (T T Truyen MD), Nam Can Tho University, Can Tho, Vietnam; Faculty of Health and Life Sciences (A Udoh PhD), University of Exeter, Exeter, United Kingdom; Department of Zoology (S Ullah PhD), Division of Science and Technology (S Ullah PhD), University of Education Lahore, Lahore, Pakistan; International Center for Chemical and Biological Sciences (S Ullah MSc), University of Karachi, Karachi, Pakistan; College of Health and Sport Sciences (A G Vaithinathan MSc), University of Bahrain, Zallaq, Bahrain; Key Laboratory of Computer-Aided Drug Design (M Waqas PhD), Guangdong Medical University, Dongguan, China; Department of Biotechnology and Genetic Engineering (M Waqas PhD), Hazara University Mansehra, Mansehra, Pakistan; Department of Parasitology (Prof K G Weerakoon PhD), Rajarata University of Sri Lanka, Anuradhapura, Sri Lanka; Competence Center of Mortality-Follow-Up of the German National Cohort (R Westerman DSc), Federal Institute for Population Research, Wiesbaden, Germany; Global Health Research Center (C Wu PhD), Duke Kunshan University, Kunshan, China; Department of Food Science and Human Nutrition (Prof F Wu PhD), Michigan State University, East Lansing, MI, USA; Department of Endocrinology (Prof S Xu PhD), University of Science and Technology of China, Hefei, China; Department of Cancer Epidemiology and Prevention Research (L Yang PhD), Alberta Health Services, Calgary, AB, Canada; Department of Oncology (L Yang PhD), University of Calgary, Calgary, AB, Canada; Faculty of Medicine (Y Yano MD), Juntendo University, Tokyo, Japan; New Jersey Medical School (M Yesiltepe PhD), Rutgers University, Newark, NJ, USA; Clinical Investigation Unit (M Yesiltepe PhD), Ankara City Hospital, Ankara, Turkiye; Department of Pediatrics (Prof D Yon MD), Kyung Hee University, Seoul, South Korea; Department of Health Policy and Management (Prof M Z Younis PhD), Jackson State University, Jackson, MS, USA; School of Business & Economics (Prof M Z Younis PhD), Universiti Putra Malaysia (University of Putra Malaysia), Kuala Lumpur, Malaysia; Department of Epidemiology and Biostatistics (Prof C Yu PhD), Wuhan University, Wuhan, China; Association for Socially Applicable Research (ASAR), Pune, India (S Zadey MS); Department of Emergency Medicine (S Zadey MS), Global Emergency Medicine Innovation and Implementation (GEMINI) Research Center, Durham, NC, USA; Department of Administration (Prof M Zastrozhin PhD), PGxAI, San Francisco, CA, USA; Tianjin

Medical University General Hospital (Z Zhang MD), Tianjin Centers for Disease Control and Prevention, Tianjin, China; School of Public Health and Emergency Management (B Zhu PhD), Southern University of Science and Technology, Shenzhen, China; Institute of Public Health and Social Sciences (H Zia BDS), Khyber Medical University, Peshawar, Pakistan; Department of Biochemistry and Pharmacogenomics (M Zielińska MPharm), Medical University of Warsaw, Warsaw, Poland; Department of Clinical and Community Pharmacy (Prof S H Zyoud PhD), Clinical Research Centre (Prof S H Zyoud PhD), An-Najah National University, Nablus, Palestine.

Authors' Contributions

Managing the overall research enterprise

Farah Daoud, Erin B Hamilton, Simon I Hay, Nicholas J Kassebaum, Ali H Mokdad, Christopher J L Murray, Mohsen Naghavi, Amanda Novotney

Writing the first draft of the manuscript

Catherine Bisignano, Ali H Mokdad, Christopher J L Murray

Primary responsibility for applying analytical methods to produce estimates

Christopher J L Murray, Ali H Mokdad

Primary responsibility for seeking, cataloguing, extracting, or cleaning data; designing or coding figures and tables

Ali H Mokdad, Christopher J L Murray, Johnathan M Hsu

Providing data or critical feedback on data sources

Olugbenga Olusola Abiodun, Richard Gyan Aboagye, Ahmed Abu-Zaid, Oluwafemi Atanda Adeagbo, Victor Adekanmbi, Leticia Akua Adzigbli, Ayman Ahmed, Syed Anees Ahmed, Yazan Al-Ajlouni, Robert W Aldridge, Abid Ali, Sami Almustanyir, Omar Al Ta'ani, Mohammad Al-Wardat, Alireza Amindarolzarbi, Jalal Arabloo, Elshaimaa A Arafa, Mosab Arafat, Alok Atreya, Fekadu Belay Ayalew, Ahmed Y. Azzam, Sara Bagherieh, Abdulaziz T Bako, Kannu Bansal, Till Winfried Bärnighausen, Amadou Barrow, Mohammad-Mahdi Bastan, Sanjay Basu, Michelle L Bell, Apostolos Beloukas, Robert S Bernstein, Akshaya Srikanth Bhagavathula, Neeraj Bhala, Sonu Bhaskar, Vivek Bhat, Aadam Olalekan Bodunrin, Hamed Borhany, Dejana Braithwaite, Florentino Luciano Caetano dos Santos, Jack Cagney, Chao Cao, Joao Mauricio Castaldelli-Maia, Francieli Cembranel, Bryan Chong, Rajiv Chowdhury, Rebecca M Cogen, Joao Conde, Leslie Trumbull Cooper, Samuele Cortese, Michael H Criqui, Natalia Cruz-Martins, Zhaoli Dai, Xiaochen Dai, Giovanni Damiani, Aso Mohammad Darwesh, Saswati Das, Louisa Degenhardt, Vinoth Gnana Chellaiyan Devanbu, Michael J Diaz, Thao Huynh Phuong Do, Thanh Chi Do, Khanh Duy Doan, Rajkumar Prakashbhai Doshi, Ojas Prakashbhai Doshi, Abdel Douiri, Robert Kokou Dowou, Alireza Ebrahimi, Temitope Cyrus Ekundayo, Ibrahim Farahat El Bayoumy, Mohammed Elshaer, Chadi Eltaha, Abdel Rahman E'mar, Abidemi Omolara Fasanmi, Timur Fazylov, Luisa S Flor, Takeshi Fukumoto, Muktar A Gadanya, Avi A Gajjar, Balasankar Ganesan, William M Gardner, Elena Ghotbi, Laszlo Göbölös, Mahaveer Golechha, Ayman Grada, Avirup Guha, Ishita Gupta, Nils Haep, Demewoz Haile, Arvin Haj-Mirzaian, Aram Halimi, Abbas M Hassan, Rasmus Havmoeller, Jeffrey J Hebert, Mehdi Hemmati, Irma Hidayana, Thomas Kwadwo Hinneh, Nguyen Quoc Hoan, Nobuyuki Horita, Md Mahbub Hossain, Mehdi Hosseinzadeh, Chengxi Hu, Hong-Han Huynh, Kathryn H Jacobsen, Ammar Abdulrahman Jairoun, Mihajlo Jakovljevic, Tahereh Javaheri, Bijay Mukesh Jeswani, Emily Katherine Johnson, Rami S Kantar, Nicholas J Kassebaum, Adarsh Katamreddy, Yousef Salah Khader, Faham Khamesipour, Khaled Khatab, Atulya Aman Khosla, Majid Khosravi, Jagdish Khubchandani, Grace Kim, Adnan Kisa, Sonali Kochhar, Gerbrand Koren, Vijay Krishnamoorthy, Vijay Kumar, Om P Kurmi, Thao Thi Thu Le, Huu-Hoai Le, Nhi Huu Hanh Le, Trang Diep Thanh Le, Janet L Leasher, Massimo Libra, Stephen S Lim, Xuefeng Liu, Jay B Lusk, Agustina M Marconi, Randall V Martin, Roy Rillera Marzo, Tesfahun Mekene Meto, Endalkachew Belayneh Melese, Irmina Maria Michalek, Ted R Miller, Mouhand F H Mohamed, Ibrahim Mohammadzadeh, Salahuddin Mohammed, Shafiu Mohammed, Ali H Mokdad, Sara Momtazmanesh, Mohammad Ali Moni, Yousef Moradi, Shane Douglas Morrison, Rohith Motappa, Vincent Mougin, Christopher J L Murray, Mohsen Naghavi, Zuhair S Natto, Henok

Biresaw Netsere, Ahmadreza Nezameslami, Van Thanh Nguyen, Hien Quang Nguyen, Hau Thi Hien Nguyen, Dang H Nguyen, Anh Hoang Nguyen, Syed Toukir Ahmed Noor, Fred Nugen, Jerry John Nutor, Ogochukwu Janet Nzoputam, Bogdan Oancea, Oluwaseun Adeolu Ogundijo, Osaretin Christabel Okonji, Andrew T Olagunju, Hany A Omar, Mahesh Padukudru P A, Romil R Parikh, Sungchul Park, Maja Pasovic, Shankargouda Patil, Shrikant Pawar, Emmanuel K Peprah, Gavin Pereira, Hoang Tran Pham, Maarten J Postma, Ghazaleh Pourali, Jalandhar Pradhan, Jagadeesh Puvvula, Vafa Rahimi-Movaghar, Mohammad Rahmanian, Shakthi Kumaran Ramasamy, Sheena Ramazanu, Ahmed Mustafa Rashid, Reza Rawassizadeh, Jennifer Rickard, Monica Rodrigues, Jefferson Antonio Buendia Rodriguez, Tilleye Runghien, Aly M A Saad, Cameron John Sabet, Siamak Sabour, Umar Saeed, Mehdi Safari, Giovanni A Salum, Vijaya Paul Samuel, Abdallah M Samy, Babak Saravi, Markus P Schlaich, Art Schuermans, David C Schwebel, Allen Seylani, Masood Ali Shaikh, Ahmed Shaikh, Husain Shakil, Medha Sharath, Amin Sharifan, Peilin Shi, Aminu Shittu, Sina Shool, Sarah Brooke Sirota, Yi Song, Lauryn K Stafford, Chandan Kumar Swain, Lukasz Szarpak, Mindy D Szeto, Jabeen Taiba, Marcos Roberto Tovani-Palone, Jasmine T Tran, Sana Ullah, Jef Van den Eynde, Aaron van Donkelaar, Avina Vongpradith, Theo Vos, Kosala Gayan Weerakoon, Ronny Westerman, Felicia Wu, Suowen Xu, Yuichiro Yano, Dong Keon Yon, Mustafa Z Younis, Chuanhua Yu, Siddhesh Zadey, Michael Zastrozhin, Mohammed G M Zeariya, Magdalena Zielińska, and Sa'ed H Zyoud

Developing methods or computational machinery

Walid Adnan Al-Zyoud, Aleksandr Y Aravkin, Ahmed Y. Azzam, Giridhara Rathnaiah Babu, Sara Bagherieh, Mohammad-Mahdi Bastan, Akshaya Srikanth Bhagavathula, Sonu Bhaskar, Aadam Olalekan Bodunrin, Hamed Borhany, Michael Brauer, Dana Bryazka, Rebecca M Cogen, Garland T Culbreth, Xiaochen Dai, Aso Mohammad Darwesh, Thanh Chi Do, Khanh Duy Doan, Weijia Fu, William M Gardner, Demewoz Haile, Aram Halimi, Erin B Hamilton, Simon I Hay, Mehdi Hosseinzadeh, Hong-Han Huynh, Tahereh Javaheri, Emily Katherine Johnson, Nicholas J Kassebaum, Atulya Aman Khosla, Majid Khosravi, Adnan Kisa, Thao Thi Thu Le, Huu-Hoai Le, Nhi Huu Hanh Le, José Francisco López-Gil, Endalkachew Belayneh Melese, Madeline E Moberg, Ibrahim Mohammadzadeh, Salahuddin Mohammed, Ali H Mokdad, Mohammad Ali Moni, Yousef Moradi, Vincent Mougin, Christopher J L Murray, Mohsen Naghavi, Ahmadreza Nezameslami, Van Thanh Nguyen, Anh Hoang Nguyen, Fred Nugen, Sujogya Kumar Panda, Hoang Tran Pham, Reza Pourbabaki, Reza Rawassizadeh, Robert C Reiner Jr., Monica Rodrigues, Tilleye Runghien, Umar Saeed, Mehdi Safari, Abdallah M Samy, Austin E Schumacher, Sarah Brooke Sirota, Lauryn K Stafford, Chandan Kumar Swain, Ngoc Ha Tran, Ronny Westerman, Danting Yang, Michael Zastrozhin, Mohammed G M Zeariya, and Meixin Zhang

Providing critical feedback on methods or results

Hazim S Ababneh, Atef Abdelkader, Michael Abdelmasseh, Richard Gyan Aboagye, Hana J Abukhadijah, Ahmed Abu-Zaid, Isaac Yeboah Addo, Oluwafemi Atanda Adeagbo, Oyelola A Adegboye, Victor Adekanmbi, Temitayo Esther Adeyeoluwa, Leticia Akua Adzigbli, Aanuoluwapo Adeyimika Afolabi, Williams Agyemang-Duah, Shahzaib Ahmad, Danish Ahmad, Ayman Ahmed, Syed Anees Ahmed, Mohammed Ahmed Akkaif, Ashley E Akrami, Ema Akter, Yazan Al-Ajlouni, Ziyad Al-Aly, Manjurul Alam, Almaza Albakri, Robert W Aldridge, Syed Mahfuz Al Hasan, Abid Ali, Waad Ali, Mohammed Usman Ali, Rafat Ali, Sami Almustanyir, Ahmed Yaseen Alqutaibi, Rami Hani Al-Rifai, Mohammed Alsabri, Omar Al Ta'ani, Jaffar A Al-Tawfiq, Mohammad Al-Wardat, Hany Aly, Walid Adnan Al-Zyoud, Reza Amani, Prince M Amegbor, Alireza Amindarolzarbi, Sohrab Amiri, Francis Appiah, Jalal Arabloo, Elshaimaa A Arafa, Mosab Arafat, Demelash Areda, Sait Ashina, Alok Atreya, Fekadu Belay Ayalew, Ahmed Y. Azzam, Giridhara Rathnaiah Babu, Soroush Baghdadi, Saeed Bahramian, Razieh Bahreini, Abdulaziz T Bako, Kannu Bansal, Till Winfried Bärnighausen, Amadou

Barrow, Mohammad-Mahdi Bastan, Sanjay Basu, Kavita Batra, Ravi Batra, Mohsen Bayati, Michelle L Bell, Apostolos Beloukas, Maryam Bemanalizadeh, Habib Benzian, Azizullah Beran, Amiel Nazer C Bermudez, Robert S Bernstein, Habtamu B B Beyene, Akshaya Srikanth Bhagavathula, Neeraj Bhala, Ashish Bhargava, Sonu Bhaskar, Vivek Bhat, Aadam Olalekan Bodunrin, Sri Harsha Boppana, Hamed Borhany, Samuel Adolf Bosoka, Christopher Boxe, Dejana Braithwaite, Michael Brauer, Dana Bryazka, Yasser Bustanji, Zahid A Butt, Florentino Luciano Caetano dos Santos, Jack Cagney, Chao Cao, Angelo Capodici, Joao Mauricio Castaldelli-Maia, Francieli Cembranel, Eeshwar K Chandrasekar, An-Tian Chen, Meng Xuan Chen, Bryan Chong, Sonali Gajanan Choudhari, Rajiv Chowdhury, Sheng-Chia Chung, Joao Conde, Leslie Trumbull Cooper, Samuele Cortese, Michael H Criqui, Natalia Cruz-Martins, Garland T Culbreth, Mario D'Oria, Bashir Dabo, Zhaoli Dai, Xiaochen Dai, Giovanni Damiani, Samuel D D Darcho, Aso Mohammad Darwesh, Saswati Das, Nihar Ranjan Dash, Mohsen Dashti, Louisa Degenhardt, Don C Des Jarlais, Vinoth Gnana Chellaiyan Devanbu, Syed Masudur Rahman Dewan, Kuldeep Dhama, Daniel Diaz, Michael J Diaz, Delaney D Ding, Thao Huynh Phuong Do, Thanh Chi Do, Khanh Duy Doan, Deepa Dongarwar, E. Ray Dorsey, Rajkumar Prakashbhai Doshi, Ojas Prakashbhai Doshi, Abdel Douiri, Robert Kokou Dowou, John Dube, Siddhartha Dutta, Laura Dwyer-Lindgren, Arkadiusz Marian Dziedzic, Alireza Ebrahimi, Joshua R R Ehrlich, Temitope Cyrus Ekundayo, Rabie Adel El Arab, Ibrahim Farahat El Bayoumy, Muhammed Elhadi, Adel B Elmoselhi, Gihan ELNahas, Mohammed Elshaer, Chadi Eltaha, Mehdi Emamverdi, Francesco Esposito, Farshid Etaee, Elochukwu Fortune Ezenwankwo, Abdel Rahman E'mar, Ayesha Fahim, Aliasghar Fakhri-Demeshghieh, Abidemi Omolara Fasanmi, Valery L Feigin, Ginenus Fekadu, Abdullah Hamid Feroze, Irina Filip, Florian Fischer, Luisa S Flor, Weijia Fu, Takeshi Fukumoto, Muktar A Gadanya, Avi A Gajjar, Balasankar Ganesan, Mohammad Arfat Ganiyani, Xiang Gao, William M Gardner, Miglas Welay Gebregergis, Mesfin Gebrehiwot, Teferi Gebru Gebremeskel, Delaram J Ghadimi, Afsaneh Ghasemzadeh, Ali Gholamrezanezhad, Elena Ghotbi, Laszlo Göbölös, Mahaveer Golechha, Davide Golinelli, Ayman Grada, Avirup Guha, Stefano Guicciardi, Vivek Kumar Gupta, Ishita Gupta, Veer Bala Gupta, Annie Haakenstad, Parham Habibzadeh, Nils Haep, Demewoz Haile, Arvin Haj-Mirzaian, Aram Halimi, Obaid I Haque, Ahmed I Hasaballah, Md. Kamrul Hasan, Md Saquib Hasnain, Abbas M Hassan, Rasmus Havmoeller, Simon I Hay, Zohreh Heidary, Mehdi Hemmati, Irma Hidayana, Thomas Kwadwo Hinneh, Yuta Hiraike, Nguyen Quoc Hoan, Md. Belal Hossain, Md Mahbub Hossain, Mehdi Hosseinzadeh, Chengxi Hu, Tsegaye Gebreyes Hundie, Kiavash Hushmandi, Hong-Han Huynh, Kevin S Ikuta, Sheikh Mohammed Shariful Islam, Md. Rabiul Islam, Louis Jacob, Kathryn H Jacobsen, Akhil Jain, Ammar Abdulrahman Jairoun, Mihajlo Jakovljevic, Elham Jamshidi, Tahereh Javaheri, Bijay Mukesh Jeswani, Angeline Jeyakumar, Kehinde Kazeem Kanmodi, Rami S Kantar, Ibraheem M Karaye, Nicholas J Kassebaum, Adarsh Katamreddy, Foad Kazemi, Jessica A Kerr, Yousef Salah Khader, Faham Khamesipour, Mohammad Jobair Khan, Zeeshan Ali Khan, Fayaz Khan, Khaled Khatab, Moawiah Khatatbeh, Moein Khormali, Atulya Aman Khosla, Majid Khosravi, Jagdish Khubchandani, Min Seo Kim, Grace Kim, Ruth W Kimokoti, Adnan Kisa, Sonali Kochhar, Gerbrand Koren, Vijay Krishnamoorthy, Md Abdul Kuddus, Mukhtar Kulimbet, Vishnutheertha Kulkarni, Vijay Kumar, Ashish Kumar, Satyajit Kundu, Om P Kurmi, Evans F Kyei, Hanpeng Lai, Qing Lan, Van Charles Lansingh, Thao Thi Thu Le, Huu-Hoai Le, Nhi Huu Hanh Le, Trang Diep Thanh Le, Janet L Leasher, Wei-Chen Lee, Wei Li, Massimo Libra, Stephen S Lim, John C Lin, Jialing Lin, Vasileios-Arsenios Lioutas, Jie Liu, Xiaofeng Liu, Richard T Liu, Xuefeng Liu, José Francisco López-Gil, Giancarlo Lucchetti, Jay B Lusk, Asma Mafhoumi, Elaheh Malakan Rad, Yosef Manla, Vahid Mansouri, Emmanuel Manu, Agustina M Marconi, Mirko Marino, Ramon Martinez-Piedra, Roy Rillera Marzo, Yasith Mathangasinghe, Fernanda Penido Matozinhos, Steven M McPhail, Tesfahun Mekene Meto, Hadush Negash Meles, Endalkachew Belayneh Melese, Laverne G Mensah, George A Mensah, Sultan Ayoud Meo, Tomislav Mestrovic, Adquate Mhlanga, Laurette Mhlanga, Irmina Maria Michalek, Ted R Miller, Mohammad Mirza-

Aghazadeh-Attari, Ajay Kumar Mishra, Madeline E Moberg, Jama Mohamed, Mouhand F H Mohamed, Nouh Saad Mohamed, Ibrahim Mohammadzadeh, Salahuddin Mohammed, Shafiu Mohammed, Ali H Mokdad, Hossein Molavi Vardanjani, Sara Momtazmanesh, Mohammad Ali Moni, Fateme Montazeri, Yousef Moradi, Maziar Moradi-Lakeh, Paula Moraga, Rohith Motappa, Efren Murillo-Zamora, Christopher J L Murray, Pirouz Naghavi, Mohsen Naghavi, Gurudatta Naik, Soroush Najdaghi, Dhairya P Nanavaty, Delaram Narimani Davani, Gustavo G Nascimento, Abdulqadir J Nashwan, Zuhair S Natto, Sabina Onyinye Nduaguba, Henok Biresaw Netsere, Ahmadreza Nezameslami, Van Thanh Nguyen, Hien Quang Nguyen, Tuan Thanh Nguyen, Dang H Nguyen, Anh Hoang Nguyen, Nasrin Nikravangolsefid, Syed Toukir Ahmed Noor, Fred Nugen, Jerry John Nutor, Ogochukwu Janet Nzoputam, Bogdan Oancea, Michael Safo Oduro, Oluwaseun Adeolu Ogundijo, Osaretin Christabel Okonji, Andrew T Olagunju, Abdulhakeem Abayomi Olorukooba, Isaac Iyinoluwa Olufadewa, Hany A Omar, Abdulahi Opejin, Doris V Ortega-Altamirano, Samuel M Ostroff, Mahesh Padukudru P A, Sujogya Kumar Panda, Romil R Parikh, Sungchul Park, Seoyeon Park, Eun-Kee Park, Ava Pashaei, Maja Pasovic, Jenil R Patel, Shankargouda Patil, Shrikant Pawar, Emmanuel K Peprah, Gavin Pereira, Richard G Pestell, Hoang Tran Pham, Anil K Philip, Michael R Phillips, Manon Pigeolet, Maarten J Postma, Ghazaleh Pourali, Reza Pourbabaki, Disha Prabhu, Pranil Man Singh Pradhan, Jalandhar Pradhan, Jagadeesh Puvvula, Mehrdad Rabiee Rad, Amir Radfar, Quinn Rafferty, Vafa Rahimi-Movaghar, Muhammad Aziz Rahman, Mohammad Rahmanian, Majed Ramadan, Shakthi Kumaran Ramasamy, Amey Rane, Ahmed Mustafa Rashid, Reza Rawassizadeh, Elrashdy Moustafa Mohamed Redwan, Robert C Reiner Jr., Taeho Gregory Rhee, Jennifer Rickard, Monica Rodrigues, Jefferson Antonio Buendia Rodriguez, Himanshu Sekhar ROUT, Tilleye Runghien, Aly M A Saad, Cameron John Sabet, Siamak Sabour, Umar Saeed, Mehdi Safari, Md Refat Uz Zaman Sajib, Giovanni A Salum, Vijaya Paul Samuel, Abdallah M Samy, Babak Saravi, Nikolaos Scarmeas, Markus P Schlaich, Art Schuermans, David C Schwebel, Mahan Shafie, Nilay S Shah, Ataollah Shahbandi, Masood Ali Shaikh, Ahmed Shaikh, Husain Shakil, Muhammad Aaqib Shamim, Mohammad Ali Shamshirgaran, Medha Sharath, Amin Sharifan, Aminu Shittu, Velizar Shivarov, Sina Shool, Kerem Shuval, Emmanuel Edwar Siddig, David A Sleet, Sameh S M Soliman, Yi Song, Lauryn K Stafford, Abida Sultana, Jing Sun, Chandan Kumar Swain, Lukasz Szarpak, Mindy D Szeto, Seyyed Mohammad Tabatabaei, Celine Tabche, Jabeen Taiba, Mohamad-Hani Temsah, Masayuki Teramoto, James Douglas Thornton, Marcos Roberto Tovani-Palone, Khai Hoan Tram, Thang Huu Tran, Ngoc Ha Tran, Jasmine T Tran, Samuel Joseph Tromans, Munkhtuya Tumurkhuu, Stefanos Tyrovolas, Arit Udoh, Saeed Ullah, Sana Ullah, Atta Ullah, Sanaz Vahdati, Omid Vakili, Jef Van den Eynde, Dominique Vervoort, Manish Vinayak, Theo Vos, Muhammad Waqas, Kosala Gayan Weerakoon, Ronny Westerman, Caroline Wilkerson, Chenkai Wu, Felicia Wu, Suowen Xu, Danting Yang, Lin Yang, Metin Yesiltepe, Dong Keon Yon, Mustafa Z Younis, Chuanhua Yu, Siddhesh Zadey, Michael Zastrozhin, Mohammed G M Zeariya, Haijun Zhang, Zhiqiang Zhang, Meixin Zhang, Claire Chenwen Zhong, Bin Zhu, Abzal Zhumagaliuly, Magdalena Zielińska, and Sa'ed H Zyoud

Drafting the work or revising it critically for important intellectual content

Hazim S Ababneh, Rouzbeh Abbasgholizadeh, Atef Abdelkader, Michael Abdelmasseh, Olugbenga Olusola Abiodun, Ahmed Abu-Zaid, Isaac Yeboah Addo, Oluwafemi Atanda Adeagbo, Oyelola A Adegboye, Victor Adekanmbi, Aanuoluwapo Adeyimika Afolabi, Danish Ahmad, Ayman Ahmed, Syed Anees Ahmed, Mohammed Ahmed Akkaif, Ashley E Akrami, Ema Akter, Yazan Al-Ajlouni, Almaza Albakri, Wafa A Aldhaleei, Robert W Aldridge, Abid Ali, Waad Ali, Mohammed Usman Ali, Rafat Ali, Sami Almustanyir, Ahmad Alrawashdeh, Rami Hani Al-Rifai, Omar Al Ta'ani, Jaffar A Al-Tawfiq, Mohammad Al-Wardat, Hany Aly, Walid Adnan Al-Zyoud, Reza Amani, Alireza Amindarolzarbi, Sohrab Amiri, Abhishek Anil, Francis Appiah, Jalal Arabloo, Elshaimaa A Arafa, Ali Ardekani, Alok Atreya, Ahmed Y. Azzam, Giridhara Rathnaiah Babu, Soroush Baghdadi, Sara Bagherieh, Razieh

Bahreini, Abdulaziz T Bako, Kannu Bansal, Till Winfried Bärnighausen, Amadou Barrow, Mohammad-Mahdi Bastan, Sanjay Basu, Maryam Beiranvand, Michelle L Bell, Apostolos Beloukas, Maryam Bemanalizadeh, Habib Benzian, Habtamu B B Beyene, Kebede A Beyene, Akshaya Srikanth Bhagavathula, Neeraj Bhala, Sonu Bhaskar, Vivek Bhat, Catherine Bisignano, Hamed Borhany, Samuel Adolf Bosoka, Christopher Boxe, Edward J Boyko, Dejana Braithwaite, Raffaele Bugiardini, Yasser Bustanji, Florentino Luciano Caetano dos Santos, Angelo Capodici, Joao Mauricio Castaldelli-Maia, Francieli Cembranel, Edina Cenko, Eeshwar K Chandrasekar, Anis Ahmad Chaudhary, An-Tian Chen, Meng Xuan Chen, Gerald Chi, Bryan Chong, Rajiv Chowdhury, Joao Conde, Samuele Cortese, Michael H Criqui, Natalia Cruz-Martins, Mario D'Oria, Bashir Dabo, Zhaoli Dai, Giovanni Damiani, Samuel D D Darcho, Nihar Ranjan Dash, Mohsen Dashti, Louisa Degenhardt, Don C Des Jarlais, Syed Masudur Rahman Dewan, Luis Antonio Diaz, Daniel Diaz, Michael J Diaz, Delaney D Ding, Thanh Chi Do, Khanh Duy Doan, Deepa Dongarwar, Rajkumar Prakashbhai Doshi, Ojas Prakashbhai Doshi, Abdel Douiri, Robert Kokou Dowou, John Dube, Siddhartha Dutta, Laura Dwyer-Lindgren, Arkadiusz Marian Dziedzic, Joshua R R Ehrlich, Rabie Adel El Arab, Ibrahim Farahat El Bayoumy, Muhammed Elhadi, Adel B Elmoselhi, Gihan ELNahas, Chadi Eltaha, Mehdi Emamverdi, Francesco Esposito, Elochukwu Fortune Ezenwankwo, Abdel Rahman E'mar, Ayesha Fahim, Valery L Feigin, Nuno Ferreira, Irina Filip, Florian Fischer, Takeshi Fukumoto, Muktar A Gadanya, Avi A Gajjar, Balasankar Ganesan, Mohammad Arfat Ganiyani, Miglas Welay Gebregergis, Teferi Gebru Gebremeskel, Delaram J Ghadimi, Afsaneh Ghasemzadeh, Elena Ghotbi, Laszlo Göbölös, Mohamad Goldust, Davide Golinelli, Ayman Grada, Avirup Guha, Stefano Guicciardi, Vivek Kumar Gupta, Ishita Gupta, Veer Bala Gupta, Parham Habibzadeh, Nils Haep, Arvin Haj-Mirzaian, Aram Halimi, Obaid I Haque, Ahmed I Hasaballah, Md. Kamrul Hasan, Md Saquib Hasnain, Abbas M Hassan, Rasmus Havmoeller, Simon I Hay, Jeffrey J Hebert, Zohreh Heidary, Mehdi Hemmati, Thomas Kwadwo Hinneh, Yuta Hiraike, Nguyen Quoc Hoan, Nobuyuki Horita, Md Mahbub Hossain, Sorin Hostiuc, Johnathan M Hsu, Junjie Huang, Kiavash Hushmandi, Hong-Han Huynh, Sheikh Mohammed Shariful Islam, Md. Rabiul Islam, Louis Jacob, Kathryn H Jacobsen, Akhil Jain, Mihajlo Jakovljevic, Bijay Mukesh Jeswani, Emily Katherine Johnson, Kehinde Kazeem Kanmodi, Rami S Kantar, Shama D Karanth, Adarsh Katamreddy, Foad Kazemi, Jessica A Kerr, Yousef Salah Khader, Fayaz Khan, Ajmal Khan, Khaled Khatab, Moawiah Khatatbeh, Atulya Aman Khosla, Majid Khosravi, Sepehr Khosravi, Min Seo Kim, Grace Kim, Adnan Kisa, Sonali Kochhar, Connor M Kubeisy, Md Abdul Kuddus, Mukhtar Kulimbet, Vishnutheertha Kulkarni, Rakesh Kumar, Satyajit Kundu, Om P Kurmi, Evans F Kyei, Hanpeng Lai, Thao Thi Thu Le, Huu-Hoai Le, Nhi Huu Hanh Le, Wei Li, John C Lin, Jialing Lin, Vasileios-Arsenios Lioutas, José Francisco López-Gil, Platon D Lopukhov, Giancarlo Lucchetti, Raimundas Lunevicius, Jay B Lusk, Elaheh Malakan Rad, Vahid Mansouri, Emmanuel Manu, Mirko Marino, Randall V Martin, Ramon Martinez-Piedra, Wolfgang Marx, Roy Rillera Marzo, Yasith Mathangasinghe, Fernanda Penido Matozinhos, Steven M McPhail, Tesfahun Mekene Meto, Hadush Negash Meles, Endalkachew Belayneh Melese, George A Mensah, Sultan Ayoud Meo, Tomislav Mestrovic, Irmina Maria Michalek, Ted R Miller, Mohammad Mirza-Aghazadeh-Attari, Mouhand F H Mohamed, Nouh Saad Mohamed, Ibrahim Mohammadzadeh, Salahuddin Mohammed, Shafiu Mohammed, Ali H Mokdad, Hossein Molavi Vardanjani, Sara Momtazmanesh, Mohammad Ali Moni, Fateme Montazeri, Maziar Moradi-Lakeh, Paula Moraga, Shane Douglas Morrison, Rohith Motappa, Efren Murillo-Zamora, Christopher J L Murray, Mohsen Naghavi, Soroush Najdaghi, Dhairya P Nanavaty, Delaram Narimani Davani, Gustavo G Nascimento, Abdulqadir J Nashwan, Zuhair S Natto, Sabina Onyinye Nduaguba, Ahmadreza Nezameslami, Van Thanh Nguyen, Hien Quang Nguyen, Tuan Thanh Nguyen, Dang H Nguyen, Anh Hoang Nguyen, Yeshambel T Nigatu, Nasrin Nikravangolsefid, Fred Nugen, Jerry John Nutor, Ogochukwu Janet Nzoputam, Bogdan Oancea, Oluwaseun Adeolu Ogundijo, Sylvester Reuben Okeke, Osaretin Christabel Okonji, Andrew T Olagunju, Abdulhakeem Abayomi Olorukooba, Hany A Omar, Kenneth Ikenna Onyedibe, Doris V Ortega-

Altamirano, Samuel M Ostroff, Mahesh Padukudru P A, Sujogya Kumar Panda, Romil R Parikh, Jenil R Patel, Shankargouda Patil, Shrikant Pawar, Emmanuel K Peprah, Gavin Pereira, Richard G Pestell, Hoang Tran Pham, Anil K Philip, Michael R Phillips, Maarten J Postma, Ghazaleh Pourali, Reza Pourbabaki, Pranil Man Singh Pradhan, Jalandhar Pradhan, Amir Radfar, Vafa Rahimi-Movaghar, Mohammad Rahmanian, Majed Ramadan, Shakthi Kumaran Ramasamy, Ahmed Mustafa Rashid, Elrashdy Moustafa Mohamed Redwan, Taeho Gregory Rhee, Jennifer Rickard, Monica Rodrigues, Jefferson Antonio Buendia Rodriguez, Himanshu Sekhar ROUT, Aly M A Saad, Cameron John Sabet, Umar Saeed, Mehdi Safari, Dominic Sagoe, Md Refat Uz Zaman Sajib, Giovanni A Salum, Vijaya Paul Samuel, Abdallah M Samy, Aswini Saravanan, Babak Saravi, Nikolaos Scarmeas, Markus P Schlaich, David C Schwebel, Allen Seylani, Mahan Shafie, Nilay S Shah, Ahmed Shaikh, Husain Shakil, Muhammad Aaqib Shamim, Medha Sharath, Amin Sharifan, Manoj Sharma, Premalatha K Shetty, Pavanchand H Shetty, Aminu Shittu, Sina Shool, Emmanuel Edwar Siddig, Surjit Singh, Ranjan Solanki, Shipra Solanki, Sameh S M Soliman, Yi Song, Lauryn K Stafford, Abida Sultana, Chandan Kumar Swain, Lukasz Szarpak, Celine Tabche, Nathan Y Tat, Mohamad-Hani Temsah, Masayuki Teramoto, Marcos Roberto Tovani-Palone, Khai Hoan Tram, Thang Huu Tran, Jasmine T Tran, Samuel Joseph Tromans, Thien Tan Tri Tai Truyen, Stefanos Tyrovolas, Arit Udoh, Sanaz Vahdati, Asokan Govindaraj Vaithinathan, Omid Vakili, Jef Van den Eynde, Dominique Vervoort, Manish Vinayak, Ronny Westerman, Lin Yang, Dong Keon Yon, Michael Zastrozhin, Haijun Zhang, Zhiqiang Zhang, Claire Chenwen Zhong, Abzal Zhumagaliuly, Hafsa Zia, Makan Ziafati, and Magdalena Zielińska

Managing the estimation or publications process

Catherine Bisignano, Farah Daoud, Erin B Hamilton, Simon I Hay, Nicholas J Kassebaum, Ali H Mokdad, Christopher J L Murray, Mohsen Naghavi, Amanda Novotney, Samuel M Ostroff, and Maja Pasovic
